# Comparison of* In Vivo* Gene Expression Profiling of RPE/Choroid following Intravitreal Injection of Dexamethasone and Triamcinolone Acetonide

**DOI:** 10.1155/2016/9856736

**Published:** 2016-06-27

**Authors:** Zeljka Smit-McBride, Elad Moisseiev, Sara P. Modjtahedi, David G. Telander, Leonard M. Hjelmeland, Lawrence S. Morse

**Affiliations:** ^1^Vitreoretinal Research Laboratory, UC Davis Department of Ophthalmology, Davis, CA 95616, USA; ^2^Department of Ophthalmology and Visual Science, UC Davis Medical Center, 4860 Y Street, Suite 2400, Sacramento, CA 95817, USA

## Abstract

*Purpose*. To identify retinal pigment epithelium (RPE)/choroid genes and their relevant expression pathways affected by intravitreal injections of dexamethasone and triamcinolone acetonide in mice at clinically relevant time points for patient care.* Methods*. Differential gene expression of over 34,000 well-characterized mouse genes in the RPE/choroid of 6-week-old C57BL/6J mice was analyzed after intravitreal steroid injections at 1 week and 1 month postinjection, using Affymetrix Mouse Genome 430 2.0 microarrays. The data were analyzed using GeneSpring GX 12.5 and Ingenuity Pathway Analysis (IPA) microarray analysis software for biologically relevant changes.* Results*. Both triamcinolone and dexamethasone caused differential activation of genes involved in “Circadian Rhythm Signaling” pathway at both time points tested. Triamcinolone (TAA) uniquely induced significant changes in gene expression in “Calcium Signaling” (1 week) and “Glutamate Receptor Signaling” pathways (1 month). In contrast, dexamethasone (Dex) affected the “GABA Receptor Signaling” (1 week) and “Serotonin Receptor Signaling” (1 month) pathways. Understanding how intraocular steroids affect the gene expression of RPE/choroid is clinically relevant.* Conclusions*. This* in vivo* study has elucidated several genes and pathways that are potentially altering the circadian rhythms and several other neurotransmitter pathways in RPE/choroid during intravitreal steroid injections, which likely has consequences in the dysregulation of RPE function and neurodegeneration of the retina.

## 1. Introduction

Intravitreal injections of dexamethasone (Dex) and triamcinolone (TAA) are a mainstay in a clinical retinal practice to treat a wide range of pathologies, such as cystoid macular edema secondary to retinal vein occlusion, diabetic macular edema, choroidal neovascularization in macular degeneration, and noninfectious uveitis [1–6]. These pathologies affect the retina but also involve the RPE and choroid.

Glucocorticoids have powerful anti-inflammatory action, but the exact underlying mechanism of this effect remains poorly defined. Additionally, intravitreal administration of glucocorticoids may lead to side effects such as cataract progression, development of steroid-induced glaucoma, and central serous chorioretinopathy [7, 8].

Clinical decision making may be greatly enhanced with a better appreciation of the differential gene expression induced by intravitreal steroids. In a previous study using a mouse model, we demonstrated that intravitreal steroids affect the expression of several genetic pathways and alter the balance between neuroprotective and neurodegenerative processes in retina [9]. In this sequel study we used the same model to examine the differential gene expression effects of intravitreal injections of Dex and TAA in RPE/choroid tissue* in vivo*, since they too are affected by both disease pathogenesis and intravitreal steroidal treatment at clinically relevant time points of 1 week and 1 month postinjection. The purpose of this study was to evaluate the gene expression effects that intravitreal Dex and TAA have on the RPE/choroid and the differences between them at clinically relevant time points.

## 2. Materials and Methods

### 2.1. Animals

This study protocol was approved by the Institutional Animal Care and Use Committee at the University of California Davis before initiation. The study was conducted according to the approved protocol and in accordance with the ARVO statement for the Use of Animals in Ophthalmic and Vision Research. Six- to eight-week-old male C57BL/6J mice (Jax-West, Sacramento, CA) were used for all experiments. Mice were housed at 21°C, under a 12-hour light/12-hour dark cycle, with food and water supplied* ad libitum*. Three groups of animals with 4 animals per group were used for each experiment.

### 2.2. Mouse Anesthesia

One mL of ketamine (100 mg/mL), 0.1 mL of xylazine (100 mg/mL), and 8.9 mL of sterile water were mixed to prepare a final solution for injection. Animals were anesthetized by injecting a 0.1 mL/10 g of body weight intraperitoneally.

### 2.3. Intravitreal Injections

Intravitreal injections were performed transconjunctivally in anesthetized C57BL/6J mice with Hamilton 33 g needles delivering 1 *μ*L of solution. In group 1 (*n* = 4) animals received balanced salt solution (BSS), group 2 (*n* = 4) dexamethasone (10 *μ*g), and group 3 (*n* = 4) TAA (25 *μ*g). The solvent for Dex and TAA was BSS. All mice included in this study were injected on the same day at the same time. At weeks 1 and 4 after the injections, the eyes were harvested for ocular dissection and total RNA isolation.

### 2.4. Tissue Collection

At days 7 and 30 postinjection mice from each group were sacrificed using a CO_2_ chamber and the RPE/choroid tissue was harvested using published procedures [10]. Sample collections were done in the morning, in the period of 1 h. Briefly, mouse eyes were removed with curved forceps, the eye glued to the Petri dish with superglue, and a circumferential cut was made dorsal to limbus, removing the front of the eye. Retinal tissue was removed after severing the optic nerve and the outside of the back of the eye was cleaned of muscles and connective tissue. Dissected RPE/choroid was collected within one minute after the sacrifice of the animal, immediately placed in 300 *μ*L of RNALater, and stored at −20°C.

### 2.5. RNA Isolation

QIAGEN's RNeasy isolation kit was used to isolate total RNA. As a first step tissue was placed in Qiazol and pushed through syringe with 17 g needle several times so the pigmented cells of RPE would be released, while sclera stayed intact as a clear sheet and was removed from the tube with needle. Since the choroid tends to be firmly attached to the inner sclera, it is likely that most of it stayed attached. RNA samples were run on an Agilent BioAnalyzer microfluidics chip RNA Nano 6000 to assess quality and quantity. Out of four samples, three samples having the highest quality (RNA Integrity Number (RIN) ≥ 7 value) were labeled as probes for the Affymetrix GeneChip microarrays (Affymetrix, Santa Clara, CA).

### 2.6. Microarray Probe Labeling

RNA samples were labeled using 0.100 ug of total RNA, following the manufacturer's protocol for GeneChip Target Labeling 3′ IVT Express Kit (Affymetrix, Santa Clara, CA). After labeling, probes were hybridized to the Affymetrix Mouse Genome 430 2.0 GeneChip microarrays. This array contains 45,101 probe sets, analyzing the expression of 39,000 transcripts and variants from over 34,000 well-characterized mouse genes. Hybridization was performed in the UC Davis Genome Center Microarray Core Facility using a standard procedure (Affymetrix, Santa Clara, CA). The total data set included 18 GeneChip microarrays.

### 2.7. Microarray Data Analysis

Microarray data were analyzed using GeneSpring GX 12.5 microarray analysis software and Ingenuity Pathway Analysis (IPA) software for biologically relevant changes of expression in genes and related pathways of the RPE/choroid.

### 2.8. Statistical Analysis of Microarrays

One-way ANOVA was used in GeneSpring to identify statistically significant genes at a significance level of *p* ≤ 0.01. The Benjamini-Hochberg* post hoc* correction method was too conservative for microarray results with limited number of biological replicas such as this. No probe sets met this significance level of testing. Therefore, a different set of stringency requirements was applied to reduce the risk of false positives. To identify biologically relevant gene expression changes for each of the time point/treatment conditions, Volcano plot analysis [11] was performed, using a combination of unpaired *t*-test and asymptotic *p* value computation. The Volcano plot is a scatter plot of the fold change versus the *p* value (in −log_10_⁡*p* scale). It is commonly used to simultaneously depict the *p* value and the fold change for gene selection cutoff schemes in microarray data. It may be viewed as a summary of “statistical” significance and “biological” significance over a large number of genes. We employed the standard approach of using a *p* value (*p* ≤ 0.05) as the primary criterion followed by fold change (−1.5 ≥ FC ≥ 1.5) as the secondary criterion to select differentially expressed genes. This approach ensures control of false-positive error and preserves the desired biological significance [12].

The data discussed in this publication have been deposited in NCBI's Gene Expression Omnibus [13, 14] and are accessible through GEO Series accession number GSE49872 (http://www.ncbi.nlm.nih.gov/geo/query/acc.cgi?acc=%20GSE49872). Supplementary Material (available online at http://dx.doi.org/10.1155/2016/9856736) attached to this paper contains the complete list of the differentially expressed genes identified by ANOVA at *p* ≤ 0.01 and Volcano plot at (−1.5 ≥ FC ≥ 1.5) *p* ≤ 0.05.

### 2.9. Bioinformatics/Pathways Analysis

Pathway analysis was performed using Ingenuity Pathway Analysis (IPA) software (QIAGEN, Valencia, CA). The lists of candidate genes identified by GeneSpring analysis were uploaded to the IPA site and IPA core analysis was applied to give us the list of activated pathways and gene networks with the highest degree of significance.

### 2.10. Quantitative PCR

Quantitative PCR (qPCR) was used as a second method for validation of the results of microarray data analysis for several candidate genes (Ahrr, Arntl, Grik1, Lcn2, Per1, and Snap25). All gene expression assays (Taqman, Applied Biosystems, Foster City, CA) were conducted in the real-time PCR Research and Diagnostic Core Facility at University of California Davis. Control genes (B2M, GAPDH, and HPRT1) were also included in order to validate the results of the qPCR.

### 2.11. Statistical Analysis of qPCR

For the genes used for qPCR validation of the microarray data, threshold cycle (Ct) values were used to determine the quantity of gene copies. For each gene, the mean of three samples was calculated and used for analysis. The fold change for each gene was determined compared to the mean of all 3 control genes used.

## 3. Results

### 3.1. Genes with Significantly Altered Expression Changes

Data was analyzed using GeneSpring 12.5. Raw data was imported as  .cel files and underwent RMA normalization with using baseline values of median of all samples. Next step was filtering by expression on the raw data for the signals between 20% and 100%, to eliminate too weak signals which were on the borderline of background noise. The signals for the probes were retained only if they were present in at least 1 out of 6 conditions within the range. The number of 36,136 probe sets passed out of total of 45,101 probe sets on the GeneChip. Statistical analysis by one-way ANOVA identified 1,868 out of 36,136 probe sets differentially expressed at a *p* value of *p* ≤ 0.05 and 430/36,136 at *p* ≤ 0.01 when the entire 18-chip sample set was considered.

**Figure 1:**
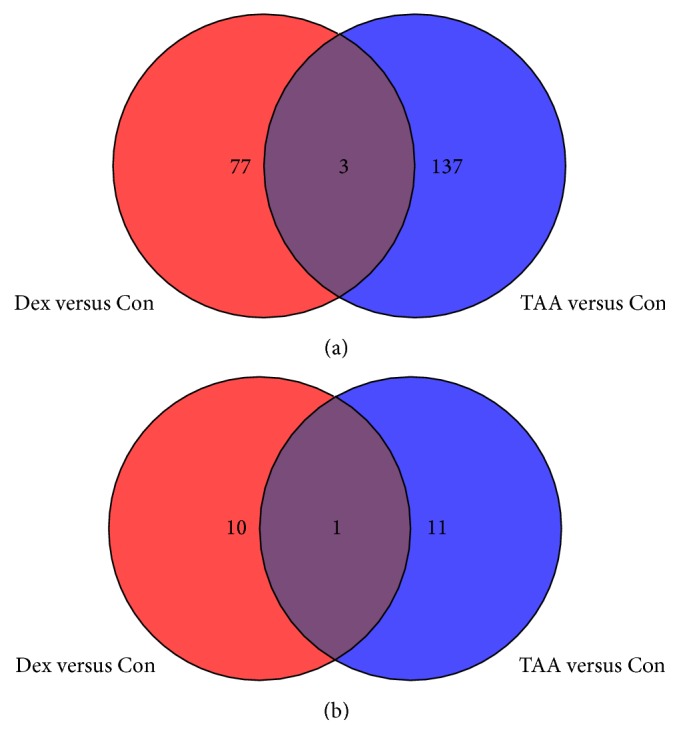
Venn diagram detailing the number of genes in each category. (a) One-week postinjection time point for fold change (FC) > 1.5, *p* < 0.05. (b) One-month postinjection time point for FC > 1.5, *p* < 0.05.

Comparison of treatment versus control for each time point using an unpaired *t*-test identified a set of common genes similarly regulated by both steroids, as well as, a set of unique genes differentially regulated by each steroid. Figures 1(a) and 1(b) represent Venn diagrams detailing the numbers of genes in each category per each time point for fold change (FC) ≥ 1.5 and *p* ≤ 0.05. A list of common genes and unique genes for each steroid/time point/regulation group is presented in Supplementary Tables S1–S6. The full lists of differentially expressed genes by ANOVA at *p* ≤ 0.01 and *p* ≤ 0.05, as well as *t*-tests −1.5 ≥ FC ≥ 1.5, *p* ≤ 0.05, can be found in the Supplementary Tables S7 and S8.

### 3.2. Biological Network and Pathway Analysis of Significantly Changed Genes

**Figure 2:**
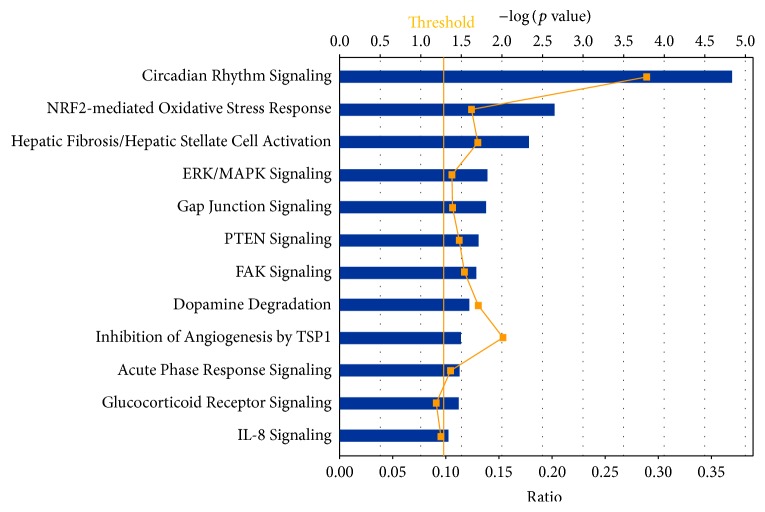
Top canonical pathways for both day 07 and day 30, ANOVA (*p* < 0.01).

**Figure 3:**
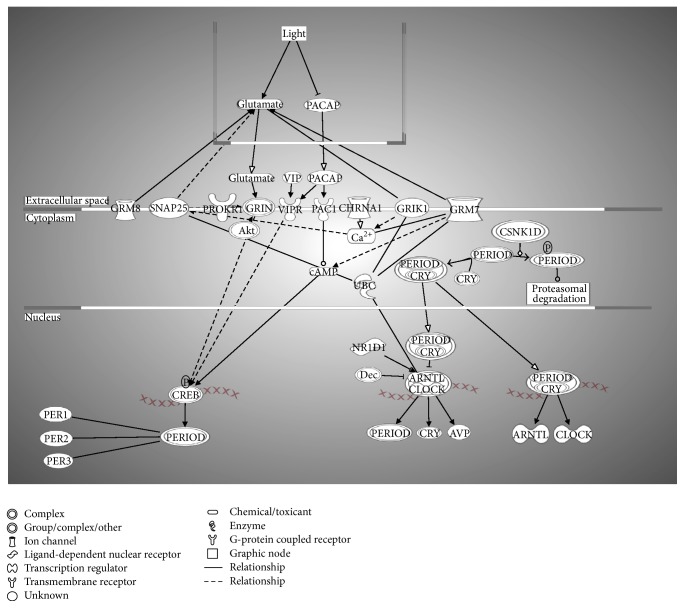
Circadian Rhythm Signaling pathway. The Entrez gene names and their symbols represented in this network: Akt; Arntl-Clock; arginine vasopressin (AVP); Aryl hydrocarbon receptor nuclear translocator-like (BMAL1); cholinergic receptor, nicotinic, alpha 1 (muscle) (CHRNA1); CK1*ε*/CK1*δ*; clock circadian regulator (CLOCK); CREB; CRY; Cry-Period; glutamate receptor, ionotropic, kainate 1 (GRIK1); GRIN; glutamate receptor, metabotropic 7 (GRM7); glutamate receptor, metabotropic 8 (GRM8); adenylate cyclase activating polypeptide 1 (pituitary) receptor type I (PAC1); adenylate cyclase activating polypeptide 1 (pituitary) (PACAP); period circadian clock 1 (PER1); period circadian clock 2 (PER2); period circadian clock 3 (PER3); period circadian clock, group (PERIOD); prokineticin receptor 1 (PROKR1); nuclear receptor subfamily 1, group D, member 1 (Rev-Erb*α*); synaptosomal-associated protein, 25 kDa (SNAP25); ubiquitin C (UBC); vasoactive intestinal peptide (VIP); vasoactive intestinal peptide receptor 2 (VIPR).

**Figure 4:**
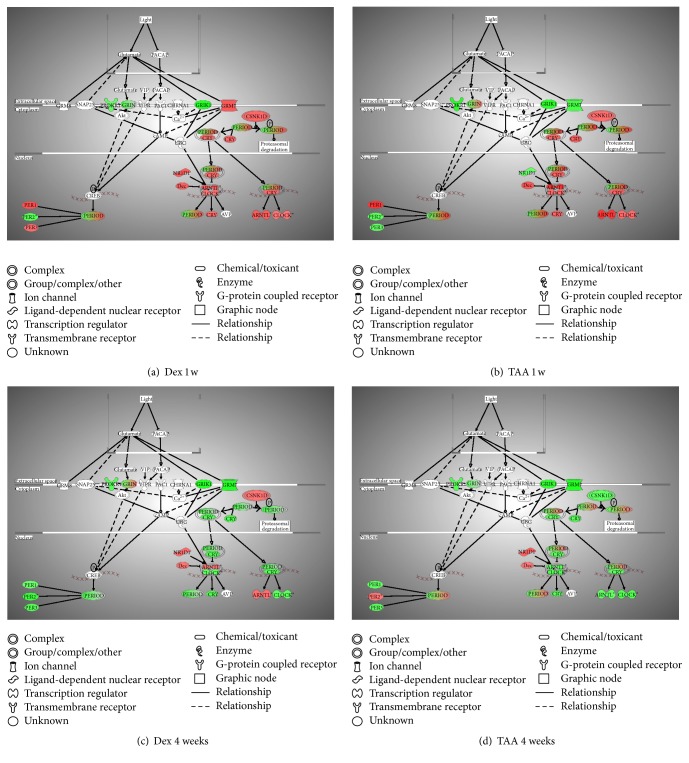
Represented is the canonical pathway “Circadian Rhythm Signaling” overlaid with the expression data from the following: (a) DEX versus Con at 1 week, (b) TAA versus Con at 1 week, (c) DEX versus Con at 1 month, and (d) TAA versus Con at 1 month postinjection. Data is represented as fold change of sample to control, *p* < 0.05 from ANOVA analysis. Red color means upregulation, while green means downregulation of the gene expression.

Biological networks and pathway analysis was performed utilizing Ingenuity Pathway Analysis (IPA) software to identify integral biological pathways that are commonly or uniquely affected by the steroids. Statistical analysis (ANOVA) of the entire 18-chip sample set identified 430 probe sets differentially expressed at *p* ≤ 0.01. From this data set the top candidate pathway affected was “Circadian Rhythm Signaling” (Figure 2). An ANOVA with more relaxed criteria of *p* ≤ 0.05 also yielded “Circadian Rhythm Signaling” as top dysregulated pathway (analysis not shown).  Figure 3 represents the canonical IPA pathway “Circadian Rhythm Signaling.” In Figure 4 the circadian rhythm signaling pathway is overlaid with expression data from each of the time point/steroid combinations.

Table 1 lists the genes that were identified by IPA analysis as key members of the pathway and their *p* values. Mammalian circadian clock generates molecular circadian rhythm through coupled transcription/translation feedback loops which involves core clock genes: Period (Per) 1 and Per 2, cryptochrome (Cry) 1 and Cry 2, Clock, and Aryl hydrocarbon receptor nuclear translocator-like (Arntl or Bmal1) genes. Recently, it has been shown that the transcription factor Npas2 (Mop4) has been able to functionally substitute for Clock in the mouse forebrain, thus adding the total number to 7 genes [15]. The circadian rhythm generation is tissue specific, and changes of the rate of transcription of mRNAs or the stability of Per and Cry proteins affect clock speed. Prokineticin receptor 1 (Prokr1), with an alternative name EG-VEGFR1, is a 7-transmembrane receptor protein from rhodopsin family, an antiapoptotic gene, involved in the signaling pathway in neovascularization [16]. The glutamate receptors (i.e., Grik1, Grm7, and Grin) are the predominant excitatory neurotransmitter receptors in the mammalian brain. Light sets off pacemakers mediated by glutamate (Glu), leading to glutamate receptor activation. Of note is that most of the clock proteins have been reported to be localized in both the nucleus and cytoplasm [17, 18]. At Dex 1 week postinjection Per1, Per3, Arntl, Clock, Cry, and Grm7 were upregulated, while Per2, Grik1, Grin, and Prokr were downregulated (Figure 4(a)). Treatment with TAA 1 week postinjection resulted in Per1, Arntl, and Clock upregulated, while Grik1, Grm7, Prokr, and partially Grin were downregulated (Figure 4(b)). At Dex 1 month postinjection most of these genes were downregulated, except Arntl, which was still upregulated (Figure 4(c)), while with TAA 1 month postinjection most of the genes were downregulated except Per2, which was still upregulated (Figure 4(d)).

**Figure 5:**
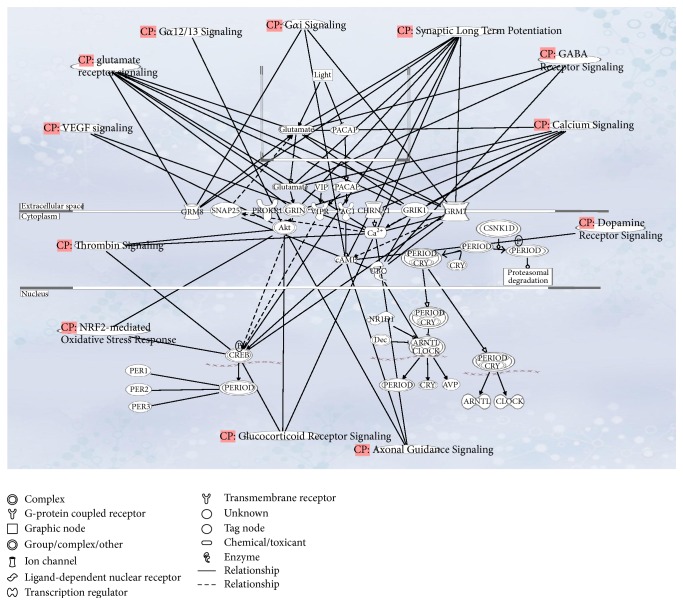
Represented is the gene network “Circadian Rhythm Signaling” showing interconnected neurotransmitter signaling pathways.

Molecular timekeeping in cells is synchronized and sustained by interneuronal neuropeptidergic signals. Genes do not work alone, but in an intricate network of interactions. Ingenuity Pathway Analysis is a hypothesis generating tool that helps interpret the data in the context of biological processes, pathways, and networks. While each gene can be interpreted as a one-dimensional representation of data, we can think of a pathway (linked list of interconnected genes) as a two-dimensional representation of data, while gene networks represent a multidimensional representation of the data.  Figure 5 is the multidimensional representation of connections between circadian rhythm pathway with glucocorticoid, dopamine, calcium, GABA, glutamate, and VEGF signaling pathways via common genes or molecules. Because of that interdependence, regulatory changes in one pathway can reflect in many others, in this case, neurotransmitter signaling pathways, causing widespread consequences, some of which are discussed in Section 4.

### 3.3. Candidate Genes and Pathways in RPE/Choroid Induced by Steroids

**Figure 6:**
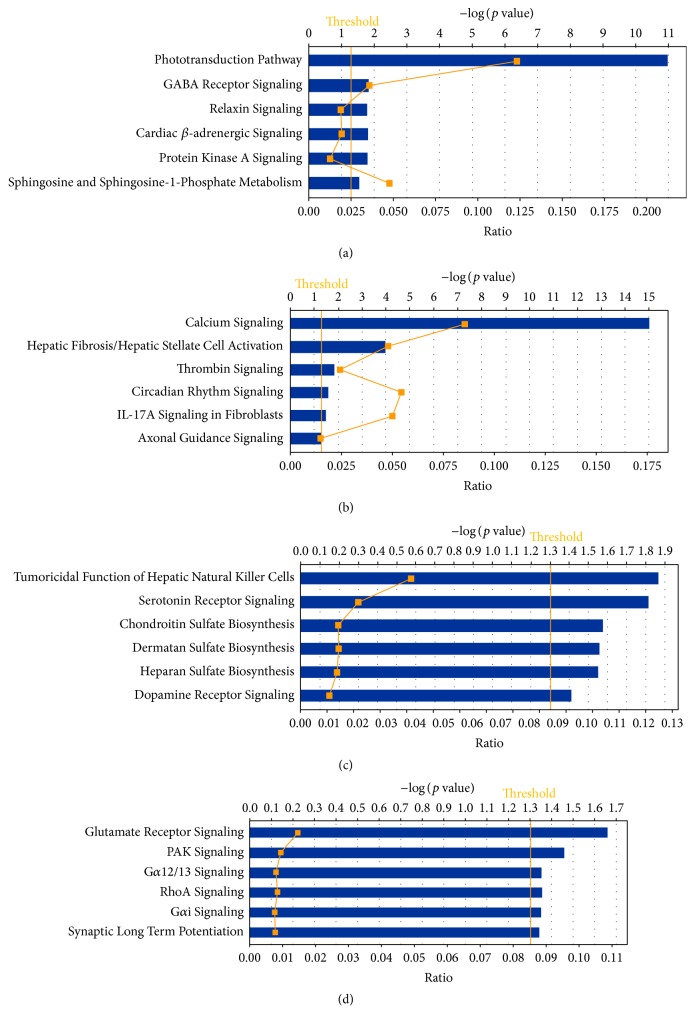
Top pathways identified by Ingenuity Pathway Analysis. (a) Top canonical pathways for DEX 1 week, *t*-test, FC 1.5, *p* < 0.05. (b) Top canonical pathways for TAA 1 week, *t*-test, FC 1.5, *p* < 0.05. (c) Top canonical pathways for DEX 30 days, *t*-test, FC 1.5, *p* < 0.05. (d) Top canonical pathways for TAA 30 days, *t*-test, FC 1.5, *p* < 0.05.

An alternative analysis by Volcano plot in GeneSpring (−1.5 ≥ FC ≥ 1.5, *p* ≤ 0.05) taking into account only genes that have the highest fold change was done for each steroid/time point to identify additional signaling pathways unique for each steroid/time point. In this section are highlighted changes in gene expression of genes of interest for ophthalmic diseases, neurodegeneration and neuroprotection, and related signal transduction pathways. The top 6 relevant canonical pathways for each time point with the significance of representation identified by IPA are presented in Figures 6(a)–6(d). Gene expression changes unique to each steroid are presented at each time point.

### 3.4. Genes Unique for Dex Week 1 Postinjection

The most affected pathway for Dex 1 week postinjection was “Phototransduction Pathway” (Figure 6(a)). The top 16 genes with altered expression in this group are all involved in visual perception and phototransduction. Most of these genes were upregulated compared to controls (Snap25, Sag, Rho, Cnga1, Gnat1, Rcvrn, Neurod1, Pde6g, Guca1a, Cacna2d4, Pde6b, Tulp1, Fscn2, Rdh12, and Olfm3), and one (Grik1) was downregulated (Table S1).

The most upregulated gene in Dex 1 week postinjection was Snap25 with a fold change of 12.73. This protein is regulated by optineurin, a pathogenic gene associated with primary open angle glaucoma (POAG) [19].

### 3.5. Genes Unique for TAA Week 1 Postinjection

The most affected pathway for TAA in week 1 postinjection was Ca^++^ signaling pathway (Figure 6(b)). Ca^++^ signaling has a role in neurotransmitter signaling, molecular transport, and vitamin and mineral metabolism. Modulation of migration and Ca^++^ signaling in retinal pigment epithelium cells has been observed as an initial step of proliferative vitreoretinopathy [20] and in angiotensin mediated Ca^++^ signaling during pathologic neovascularization and inflammation [21]. The genes involved in Ca^++^ signaling that were affected were nAchR, nicotinic acetylcholine receptor, which was downregulated, Ccl28, chemokine (FC = −1.56), Trdn, triadin (FC = −2.09), Asph, aspartate-beta-hydroxylase (FC = −1.87), Ptger3, prostaglandin E receptor 3 (FC = −2.25), SLN, and sarcolipin (FC = −2.56).

Interestingly, the most upregulated gene was Lcn2, lipocalin 2, an innate immunity protein, with a role in cellular response to oxidative stress, drug insult, IL-1 response, and TNF-alpha response (FC = 19.3). The most downregulated ones were crystallins (Cryba1, Crygs and Crybb2, Cryaa, Cryba4, Crygb, and Crygc), genes whose protein products play a role in development of the eye, in visual perception, and have a role as heat-shock proteins (Table S2). It shows that expression of signal transduction genes was influenced by TAA. The genes Arntl, Per1, Gnb3, and Arrdc2 were upregulated (FC = 1.55–2.33), while Abra, Ptger3, Dpp4, Chrna1, Ifi204, Murc, Itsn1, Prokr1, and Ddit4l were downregulated (FC = −2.42 to −1.56).

### 3.6. Common Genes for Dex and TAA at 1 Week Postinjection

Only three genes were found in common for both Dex and TAA: Myh8, Tpm3, identified as self-antigen in patients with Behcet's disease with posterior uveitis [22], and Elov1, with a role in fatty acid biosynthesis. All were downregulated (FC = −1.50 to −3.39) (Table S3).

### 3.7. Unique Genes for Dex at 1 Month Postinjection

Ten genes had significantly altered expression following Dex at 1 month postinjection (Table S4). One of the upregulated genes was Slc18a2, a solute carrier with a role in neurotransmitter transport, as well as Serotonin and Dopamine Receptor Signaling [23, 24]. The second gene was Foxs1, a member of the Notch signaling pathway, which has a role in vasculogenesis, as well as insulin receptor and VEGF receptor signaling [25]. Downregulated was Nsg2, neuron specific gene, part of the Dopamine Receptor Signaling pathway.

### 3.8. Unique Genes for TAA at 1 Month Postinjection

There were eight affected genes in this group (Table S5), the most interesting being the downregulation of glutamate receptor Grm7. This receptor has a role in neuroprotection, synaptic transmission, and regulation of Ca^++^ ion transport via voltage gated Ca^++^ channel activity.

### 3.9. Common Genes for Dex and TAA at 1 Month Postinjection

As a general observation and contrary to the data of steroid effects in the retina [9], there were far fewer genes with an altered expression at 1 month than at 1 week postinjection.

Only one gene was common for both steroids after a *t*-test with FC = 1.5, *p* < 0.05 (Table S6). That gene was Fibrillin2 (Fbn2), an extracellular matrix constituent, with Ca^++^ ion binding capabilities, mildly upregulated for both steroids (FC = 1.5–1.6).

### 3.10. Validation of the Microarray Data by qPCR

Independent qPCR validation of representative differentially expressed genes was performed using commercial gene expression assays (Taqman, Applied Biosystems). The fold changes and their direction were confirmed for the chosen genes, thus corroborating the microarray data analysis results. Data are presented in Table 2.

## 4. Discussion

Retinal pigment epithelial cells are responsible for the health and maintenance of retinal cells. RPE dysfunction plays a role in many ocular disorders including age-related macular degeneration, central serous chorioretinopathy, white-dot syndromes, and retinitis pigmentosa, which can lead to permanent visual distortion and blindness. One of the main functions of the RPE is regulation of disc shedding by photoreceptors, which is a rhythmic process essential for photoreceptor health and proper function. This process has been shown to be under circadian clock control [26–28]. The mammalian retina has its own circadian clock mechanism, originally thought to be located in the photoreceptors [29–31]. More recent studies have reported the presence of a circadian clock in RPE cells [32–34]. It has been shown that signaling is mediated by the dopamine D1 receptor and GABA [35, 36]. The mammalian retinal circadian clock mechanism is autonomous from the central master circadian clock, which in mammals resides in the hypothalamus [37].

The cyclic pattern of expression of circadian proteins is dependent on a number of external cues or “zeitgebers” including light exposure, feeding pattern, and exercise. The primary and the strongest zeitgeber is light, which starts the process, and then there are many secondary ones, conveying the information to peripheral tissue. Secondary ones start with serotonin and melatonin and then there are more downstream ones, such as insulin and glucocorticoids [38, 39]. Glucocorticoids have been demonstrated to be implicated in the entrainment of circadian rhythms [40–42]. They were shown to be capable of overcoming other entrainment factors regulating the peripheral oscillators in the kidney and lung [43] and also act as strong entraining signals for peripheral circadian oscillators and even feedback on central oscillators [44].

In our study “Circadian Rhythm Signaling” was the primary identified pathway by IPA. This finding suggests that intravitreal injection of steroids has a significant effect on the RPE/choroid, causing dysregulation of the retinal circadian clock, which is observable at 1 week postinjection and continues through 1 month postinjection. Disruption of circadian rhythm has been demonstrated to promote inflammation [45–47] and has been implicated as a contributing factor to disease pathogenesis [48, 49]. Wang et al. have shown in a diabetic mouse model that dysregulation of circadian rhythmicity resulted in increased retinal inflammation [50]. Since both inflammation and the physiological glucocorticoid metabolism are involved with circadian rhythm, it is not surprising that this is a major pathway influenced by steroid administration. Although the clinical importance of this finding is not clear, we suggest that the effect of steroids on the expression of circadian rhythm genes may be part of their therapeutic anti-inflammatory action and is not necessarily an adverse effect.

On the other hand, it has been reported that the release of glucocorticoid hormones happens in an hourly or ultradian rhythm in a pulsating manner and that levels of free glucocorticoid hormones are highly synchronized between blood, subcutaneous tissue, and the brain. By administering a bolus of steroids, this fine regulation is disrupted, and the loss of the ultradian rhythm may subsequently result in gene expression changes in downstream target tissues [51]. Our results demonstrated that the effect of both steroids on circadian rhythm gene expression was much more marked after 1 week than after 4 weeks. This may be reflecting the initial effect following the administration of a single intravitreal dose, which is subsequently waning. Accordingly, it is possible that the use of a sustained-release implant, such as Ozurdex or Iluvien, may achieve a more stable concentration and lead to prolonged effects on RPE/choroid gene expression. These implants have already proven their longer-lasting therapeutic effect in a variety of diseases [2, 4, 52–55].

### 4.1. Differences in Gene Expression Changes between Dex and TAA

The most affected pathway for Dex week 1 postinjection is Phototransduction Pathway (Figure 6(a)). Why genes that belong to the Phototransduction Pathway change in the RPE/choroid was puzzling. We do not believe that retinal tissue contamination was the cause of this, since mouse retinal tissue is very loosely attached to RPE and upon dissection separates very easily and cleanly, so there is very little, if any, chance of contamination of RPE with retinal cells.

To verify whether some of the rod and cone genes are actually expressed in RPE, we checked for the presence of several phototransduction genes listed in the tables from Newman et al. [56] (Table S7) and found several of them differentially expressed in RPE of AMD versus normal (Gngt1, GUCA, PDE6, both cone and rod specific, and RGS9). Our data suggests that their altered expression levels are a result of the Dex treatment of the eye. We hypothesize that apparent upregulation of expression of these genes may have possibly resulted from accumulation of these mRNAs due to slowed down phagocytic and autophagic processes that are dysregulated by steroid therapy.

Another one of the top five activated pathways in Dex 1 week postinjection is GABA Receptor Signaling (Figure 6(a)). GABA is the main inhibitory neurotransmitter in the mammalian CNS. The GABA transporter GAT, located in the plasma membrane of nerve terminals and glial cells, plays an important role in the termination of synaptic transmission. This neurotransmitter has also been implicated in effecting the circadian rhythm [35, 36], and therefore its activation may reflect the significant alterations demonstrated in the gene expression of this pathway. Since both Dex and TAA were shown to alter the gene expression of the circadian rhythm pathway, it is not surprising that they both altered the GABA Receptor Signaling pathway as well.

### 4.2. Connection between Circadian Rhythm Dysregulation, Glutamate Signaling, and Ca^++^ Signaling

The main signal transduction pathways that appear to be activated by steroids in the RPE are glutamate and Ca^++^ signaling. Light sets off pacemakers mediated by glutamate (Glu), leading to glutamate receptor activation [57, 58]. A recent paper by Suwanjang et al. shows how those two pathways are interconnected with glucocorticoid signaling: glucocorticoids reduce the intracellular Ca^++^ concentration in neurons and astrocytes and protect neurons against glutamate toxicity [59].

### 4.3. Neurotransmitters and Synaptic Signaling

#### 4.3.1. Glutamatergic Neurotransmission

L-glutamate is the major excitatory neurotransmitter in the central nervous system that activates both ionotropic (NMDAR) and metabotropic (GRM) glutamate receptors. Glutamanergic neurotransmission is involved in most aspects of normal neural function, such as intracellular signal transduction, axonal targeting, and synaptic clustering.

Our results show upregulation of Grm7 with Dex and downregulation with TAA at 1 week. At 1 month postinjection we see downregulation of Grm7 for both steroids. Grm7, glutamate receptor** 7**, with a role in neuroprotection, synaptic transmission, and regulation of Ca^++^ ion transport via voltage gated Ca^++^ channel activity, has been shown to confer susceptibility to many neuropathologic conditions such as age-related hearing impairment [57, 60, 61], attention-deficit/hyperactivity disorder [58, 62], and schizophrenia [63–65]. Most variants of GRM7 are expressed in brain and retina in varying abundance.

#### 4.3.2. Dopaminergic Neurotransmission

Two genes that were consistently downregulated at both time points for TAA were Rbfox1 and neuronal mRNA splicing factor Fox-1, which splices mRNAs encoding proteins important in synaptic transmission and membrane excitation [66]. The second gene is Arpp21, cAMP-regulated phosphoprotein (Table S6). The encoded protein is enriched in the caudate nucleus and cerebellar cortex. A similar protein in the mouse may be involved in regulating the effects of dopamine in the basal ganglia. At Dex 1-month postinjection time point, two members of dopaminergic neurotransmission were dysregulated, Slc18a2 and Nsg2.

Our results from differential gene expression of over 34,000 well-characterized mouse genes of the RPE/choroid of C57BL/6J mice after intravitreal steroid injections at 1 week and 1 month postinjection suggest that dysregulation of glutamatergic and dopaminergic neurotransmission signaling may represent a novel target for therapeutic approaches in the treatment of steroid-induced glaucoma.

In conclusion, in our previous study [9], we have reported that intravitreal Dex and TAA resulted in gene expression changes which potentially altered the balance between neuroprotective and neurodegenerative processes in the retina. In this study, using the same mouse model, we investigated the effects of these steroids on gene expression in the RPE/choroid and found that the major influenced pathways are circadian rhythm and several neurotransmission pathways. Comparative analysis of differential gene expression that takes place in different tissues following the administration of Dex and TAA suggests tissue-specific changes. The activation of signal transduction pathways in the RPE/choroid may mirror or complement the changes in neuroprotection and neurodegeneration processes that occur in the retina following intravitreal steroid administration. The effect on circadian rhythm pathway may be part of the anti-inflammatory action of these agents. The effect of both Dex and TAA was more significant at 1 week than at 4 weeks, which implies that a more controlled slow-release form of administration may achieve longer-lasting cellular and genetic effects. In conclusion, our findings have the potential to contribute to a more complete understanding of the mechanisms by which the steroids Dex and TAA regulate gene expression in the eye and to the development of improved therapeutics.

## Supplementary Material

Supplemental material contains the complete list of the differentially expressed genes identified by Volcano plot at -1.5≥ FC ≥1.5; p≤0.05 (Tables S1-S6), and ANOVA at p≤0.01 and p≤0.05 (Tables S7 and S8).

## Figures and Tables

**Table 1:** Circadian Rhythm Signaling genes from Ingenuity Pathway Analysis (IPA).

Symbol	Entrez gene name	Affymetrix	*p * value	Location	Type (s)
ARNTL	Aryl hydrocarbon receptor nuclear translocator-like	1425099_a_at	2.58*E* − 06	Nucleus	Transcription regulator
BHLHE40	Basic helix-loop-helix family, member e40	1418025_at	3.41*E* − 02	Nucleus	Transcription regulator
CLOCK	Clock circadian regulator	1418660_at	7.86*E* − 04	Nucleus	Transcription regulator
CRY2	Cryptochrome 2 (photolyase-like)	1426383_at	3.94*E* − 02	Nucleus	Enzyme
CSNK1D	Casein kinase 1, delta	1418889_a_at	7.26*E* − 03	Cytoplasm	Kinase
GRIN2C	Glutamate receptor, ionotropic, N-methyl D-aspartate 2C	1449245_at	2.13*E* − 02	Plasma membrane	Ion channel
GRINA	Glutamate receptor, ionotropic, N-methyl D-aspartate-associated protein 1 (glutamate binding)	1436297_a_at	4.26*E* − 02	Unknown	Ion channel
NR1D1	Nuclear receptor subfamily 1, group D, member 1	1426464_at	1.40*E* − 03	Nucleus	Ligand-dependent nuclear receptor
PER1	Period circadian clock 1	1449851_at	6.31*E* − 04	Nucleus	Other
PER2	Period circadian clock 2	1417602_at	2.63*E* − 04	Nucleus	Other
PER3	Period circadian clock 3	1421087_at	5.30*E* − 06	Nucleus	Other

**Table 2:** Comparison of microarray and qPCR results.

Fold change comparisons		
	Microarray	qPCR

Gene	Dex/1 wk (Affy)	Dex/1 wk (Taq)

Ahrr	1.71	1.93
Arntl	1.26	1.66
Grik1	−1.91	−1.35
Lcn2	4.06	11.9
Per1	1.32	1.41
Snap25	12.73	15.18

Gene	TAA/1 wk (Affy)	TAA/1 wk (Taq)

Ahrr	1.32	3.07
Arntl	1.68	1.25
Grik1	−1.45	0.43
Lcn2	19.32	39.19
Per1	1.62	1.87
Snap25	5.4	11.52

Gene	Dex/1 mo (Affy)	Dex/1 mo (Taq)

Grik1	−1.82	−1.93
Lcn2	1.64	9.3
Snap25	−5.12	−1.77

Gene	TAA/1 mo (Affy)	TAA/1 mo (Taq)

Grik1	−1.41	−1.41
Lcn2	nd	0.34
Snap25	−1.97	−0.31
