# Supplementary material for: Comparison of In Vivo Gene Expression Profiling of RPE/Choroid following Intravitreal Injection of Dexamethasone and Triamcinolone Acetonide
Source: J Ophthalmol. 2016 Jun 27;2016:9856736. doi: 10.1155/2016/9856736 (PMC4939337; doi:10.1155/2016/9856736)
Supplement: Supplementary file 1 — Supplemental material contains the complete list of the differentially expressed genes identified by Volcano plot at -1.5≥ FC ≥1.5; p≤0.05 (Tables S1-S6), and ANOVA at p≤0.01 and p≤0.05 (Tables S7 and S8). [file 9856736.f1.zip › Description.docx]

Supplemental material contains the complete list of the differentially expressed genes identified by Volcano plot at -1.5≥ FC ≥1.5; p≤0.05 (**Tables S1-S6**), and ANOVA at p≤0.01 and p≤0.05 (**Tables S7 and S8**).

Table S1 – Unique top dysregulated genes for Dex vs. Con, 1 week postinjection

Table S2 – Unique top dysregulated genes for at TAA vs. Con 1 week postinjection

Table S3 - Common dysregulated genes for Dex and TAA at 1 week postinjection

Table S4 - Unique dysregulated genes for Dex at 4 weeks postinjection

Table S5 – Unique dysregulated genes for TAA at 4 weeks postinjection

Table S6 – Common dysregulated genes for DEX and TAA at 4 weeks postinjection

Table S7 – ANOVA p<0.01

Table S8 – ANOVA p<0.05
