# Supplementary material for: Comparison of In Vivo Gene Expression Profiling of RPE/Choroid following Intravitreal Injection of Dexamethasone and Triamcinolone Acetonide
Source: J Ophthalmol. 2016 Jun 27;2016:9856736. doi: 10.1155/2016/9856736 (PMC4939337; doi:10.1155/2016/9856736)
Supplement: Supplementary file 1 — Supplemental material contains the complete list of the differentially expressed genes identified by Volcano plot at -1.5≥ FC ≥1.5; p≤0.05 (Tables S1-S6), and ANOVA at p≤0.01 and p≤0.05 (Tables S7 and S8). [file 9856736.f1.zip › Tables.pdf]

Table S1

No	Probe Set ID	Gene Symbol	Fold Change	p-value	Gene Title
1	1416828_at	Snap25	12.73	0.0444	synaptosomal-associated protein 25
2	1419025_at	Sag	7.51	0.0488	retinal S-antigen; arrestin 1
3	1425172_at	Rho*	7.48	0.0297	rhodopsin
4	1451763_at	Cnga1	7.27	0.0402	cyclic nucleotide gated channel alpha 1
5	1460212_at	Gnat1	7.01	0.0357	guanine nucleotide binding protein, alpha transducing 1
6	1450215_at	Rcvrn	6.92	0.0492	recoverin
7	1426413_at	Neurod1	6.43	0.0499	neurogenic differentiation 1
8	1450946_at	Nrl	4.94	0.0497	neural retina leucine zipper gene
9	1419740_at	Pde6b*	4.91	0.0487	phosphodiesterase 6B, cGMP, rod receptor, beta polypeptide
10	1421061_at	Guca1a	4.44	0.0425	guanylate cyclase activator 1a (retina)
11	1455436_at	Diras2	4.40	0.0375	DIRAS family, GTP-binding RAS-like 2
12	1442863_at	Cacna2d4	4.22	0.0201	calcium channel, voltage-dependent, alpha 2/delta subunit 4
13	1419085_at	Pcp2	3.54	0.0399	Purkinje cell protein 2 (L7)
14	1419225_at	Cacna2d3	3.18	0.0329	calcium channel, voltage-dependent, alpha2/delta subunit 3
15	1451582_at	Tulp1	3.07	0.0378	tubby like protein 1
16	1440605_at	Fscn2	3.04	0.0386	fascin homolog 2, actin-bundling protein, retinal (Strongylocentrotus purpuratus)
17	1424256_at	Rdh12	2.56	0.0428	retinol dehydrogenase 12
18	1429685_at	Gabrb2	2.43	0.0101	gamma-aminobutyric acid (GABA) A receptor, subunit beta 2
19	1436402_at	Dohh	2.27	0.0469	deoxyhypusine hydroxylase/monooxygenase ///
20	1441706_at	Dscaml1	2.26	0.0430	Down syndrome cell adhesion molecule-like 1
21	1425898_x_at	Olfm3	2.06	0.0400	olfactomedin 3
22	1455393_at	Cp*	1.99	0.0232	ceruloplasmin
23	1450761_s_at	Rims2*	1.84	0.0378	regulating synaptic membrane exocytosis 2
24	1420796_at	Ahrr	1.71	0.0289	aryl-hydrocarbon receptor repressor
25	1458492_x_at	Ntm*	1.69	0.0054	neurotrimin
26	1426642_at	Fn1	1.69	0.0049	fibronectin 1
27	1418724_at	Cfi	1.58	0.0427	complement component factor i
28	1436455_at	Asph	1.58	0.0344	aspartate-beta-hydroxylase
29	1455426_at	Epha3	1.54	0.0008	Eph receptor A3
31	1433966_x_at	Asns	1.52	0.0453	asparagine synthetase
32	1460081_at	Syt7	1.50	0.0160	synaptotagmin VII
1	1443820_x_at	Elovl1	-1.50	0.0030	elongation of very long chain fatty acids
2	1419453_at	Uchl5	-1.51	0.0185	ubiquitin carboxyl-terminal esterase L5
3	1449102_at	Ebf2	-1.52	0.0204	early B-cell factor 2
4	1427866_x_at	Hbb-b2	-1.53	0.0397	hemoglobin, beta adult minor chain
6	1450952_at	Pln	-1.60	0.0494	phospholamban
7	1450291_s_at	Ms4a4c	-1.78	0.0356	membrane-spanning 4-domains, subfamily A, member 4C
8	1426650_at	Myh8	-1.84	0.0097	myosin, heavy polypeptide 8, skeletal muscle, perinatal
9	1427676_a_at	Grik1	-1.91	0.0011	glutamate receptor, ionotropic, kainate 1
10	1449997_at	Tpm3	-3.39	0.0394	tropomyosin 3, gamma

* additional multiple probes

Table S2

No	Probe Set ID	Gene Symbol	Fold Change	p-value	Gene Title
1	1427747_a_at	Lcn2	19.32	0.000	lipocalin 2
2	1450826_a_at	Saa3	6.25	0.002	serum amyloid A 3
3	1419764_at	Chi3l3	4.86	0.039	chitinase 3-like 3
4	1449159_at	Gnb3	2.33	0.046	guanine nucleotide binding protein (G protein), beta 3
5	1453678_at	Mbd1	2.01	0.016	methyl-CpG binding domain protein 1
6	1424882_a_at	Nt5dc2	1.89	0.038	5'-nucleotidase domain containing 2
7	1423078_a_at	Sc4mol	1.87	0.021	sterol-C4-methyl oxidase-like
8	1431226_a_at	Fndc4	1.82	0.036	fibronectin type III domain containing 4
9	1439983_a_at	Accn3	1.77	0.001	amiloride-sensitive cation channel 3
10	1419883_s_at	Atp6v1b2	1.68	0.050	ATPase, H+ transporting, lysosomal V1 subunit B2
11	1425099_a_at	Arntl	1.68	0.050	aryl hydrocarbon receptor nuclear translocator-like
12	1426059_at	Gckr	1.68	0.041	glucokinase regulatory protein
13	1423233_at	Cebpd	1.67	0.003	CCAAT/enhancer binding protein (C/EBP), delta
14	1422253_at	Col10a1	1.65	0.030	collagen, type X, alpha 1
15	1449851_at	Per1	1.62	0.026	period homolog 1 (Drosophila)
16	1450982_at	Slc9a3r1	1.58	0.006	solute carrier family 9 (Na/H exchanger), member 3 regulator 1
17	1417672_at	Slc4a10	1.56	0.022	solute carrier family 4, Na- bicarbonate cotransporter-like, member 10
18	1455577_at	Ccl28	1.56	0.006	chemokine (C-C motif) ligand 28
19	1418706_at	Slc38a3	1.55	0.026	solute carrier family 38, member 3
20	1428352_at	Arrdc2	1.55	0.013	arrestin domain containing 2
21	1423418_at	Fdps	1.51	0.013	farnesyl diphosphate synthetase
22	1424400_a_at	Aldh1l1 /// LOC100047937	1.51	0.007	aldehyde dehydrogenase 1 family, member L1 /// 10-formyltetrahydrofolate dehydrogenase-like
23	1448130_at	Fdft1	1.50	0.019	farnesyl diphosphate farnesyl transferase 1
1	1423468_at	Steap3	-1.55	0.034	STEAP family member 3
2	1435884_at	Itsn1	-1.56	0.041	intersectin 1 (SH3 domain protein 1A)
3	1420551_at	Murc	-1.57	0.022	muscle-related coiled-coil protein
4	1448371_at	Mylpf	-1.57	0.028	myosin light chain, phosphorylatable, fast skeletal muscle
5	1459973_x_at	Dpp4	-1.58	0.030	dipeptidylpeptidase 4
6	1418852_at	Chrna1 (nAchR)	-1.63	0.007	cholinergic receptor, nicotinic, alpha polypeptide 1 (muscle)
7	1451751_at	Ddit4l	-1.64	0.037	DNA-damage-inducible transcript 4-like
8	1456543_at	Prokr1	-1.69	0.049	prokineticin receptor 1
9	1419011_at	Cryba2	-1.75	0.011	crystallin, beta A2
10	1418649_at	Egln3	-1.79	0.013	EGL nine homolog 3 (C. elegans)
11	1419603_at	Ifi204*	-1.81	0.008	interferon activated gene 204
12	1425274_at	Asph	-1.87	0.012	aspartate-beta-hydroxylase
13	1418306_at	Crybb1	-1.92	0.030	crystallin, beta B1
14	1443783_x_at	H2-Aa*	-1.97	0.016	histocompatibility 2, class II antigen A, alpha
15	1419005_at	Crybb3	-2.07	0.034	crystallin, beta B3
16	1451801_at	Trdn*	-2.09	0.014	triadin
17	1450344_a_at	Ptger3	-2.25	0.005	prostaglandin E receptor 3 (subtype EP3)
18	1458455_at	Abra	-2.42	0.000	actin-binding Rho activating protein
19	1415994_at	Cyp2e1	-2.44	0.048	cytochrome P450, family 2, subfamily e, polypeptide 1
20	1422674_s_at	Crygb /// Crygc	-2.45	0.028	crystallin, gamma B /// crystallin, gamma C
21	1420884_at	Sln	-2.56	0.041	sarcolipin
22	1420686_at	Cryba4	-2.57	0.011	crystallin, beta A4
23	1452486_a_at	Cryaa	-2.77	0.038	crystallin, alpha A
24	1418511_at	Dpt	-2.88	0.024	dermatopontin
25	1420536_at	Crybb2	-3.68	0.034	crystallin, beta B2
26	1420453_at	Crygs	-5.21	0.031	crystallin, gamma S
27	1450671_at	Cryba1	-6.10	0.041	crystallin, beta A1

* additional multiple probes

Table S3

Probe Set ID	Gene Symbol	Fold Change Dex vs control	p-value Dex vs con	Fold Change TAA vs control	p-value TAA vs control	Entrez Gene Title
**1426650_at**	Myh8	-1.84	0.010	-1.91	0.015	myosin, heavy polypeptide 8, perinatal
**1449997_at**	Tpm3	-3.39	0.039	-2.93	0.018	tropomyosin 3, gamma
**1443820_x_at**	Elovl1	-1.50	0.003	-1.54	0.002	elongation of very long chain fatty acids (FEN1/Elo2, SUR4/Elo3, yeast)-like 1

Table S4

Probe Set ID	Gene Symbol	FC ([D30-DEX] Vs [D30-Con])	p ([D30-DEX] Vs [D30-Con])	Gene Title
**1435622_at**	Hs3st3a1	1.64	0.0241	heparan sulfate (glucosamine) 3-O-sulfotransferase 3A1
**1433653_at**	Fam20a	1.51	0.0312	family with sequence similarity 20, member A
**1437079_at**	**Slc18a2**	1.71	0.0297	solute carrier family 18 (vesicular monoamine), member 2
**1437820_at**	Foxs1	1.73	0.0377	forkhead box S1
**1441779_at**	9530006C21Rik	1.73	0.0385	
**1442560_at**		1.77	0.0349	
**1446850_at**		1.73	0.0353	
**1416107_at**	**Nsg2**	-1.90	0.0432	neuron specific gene family member 2
**1429379_at**	Lyve1	-1.74	0.0220	lymphatic vessel endothelial hyaluronan receptor 1
**1453070_at**	Pcdh17	-1.70	0.0324	protocadherin 17

Table S5

Probe Set ID	Gene Symbol	FC ([D30-TAA] Vs [D30-Con])	p ([D30-TAA] Vs [D30-Con])	Gene Title
**1452670_at**	Myl9	1.83	0.0445	myosin, light polypeptide 9, regulatory
**1460741_x_at**	D17Wsu92e	1.60	0.0499	DNA segment, Chr 17, Wayne State University 92, expressed
**1439055_at**	Gm15698	2.22	0.0082	transcription elongation factor B (SIII), polypeptide 2 pseudogene
**1418314_a_at**	**Rbfox1**	-1.51	0.0347	RNA binding protein, fox-1 homolog (C. elegans) 1
**1419028_at**	**Arpp21**	-1.59	0.0093	cyclic AMP-regulated phosphoprotein, 21
**1424607_a_at**	Gm4354	-1.86	0.0117	predicted gene 4354
**1424609_a_at**	Gm4354	-1.76	0.0110	predicted gene 4354
**1427346_at**	Luzp4 /// Ott Gm10439 /// Gm15080 /// Gm15085 /// Gm15107 /// Gm15128 ///	-1.57	0.0382	leucine zipper protein 4 /// ovary testis transcribed ///predicted gene 10439 /// predicted gene 15080 /// predicted gene 15085 /// predicted gene 15107 /// predicted gene 15128
**1443119_at**	**Grm7**	-1.68	0.0421	glutamate receptor, metabotropic 7

Table S6

Probe Set ID	Gene Symbol	FC ([D30-DEX] Vs [D30-Con])	p [D30-DEX] Vs [D30-Con])	FC [D30-TAA] Vs [D30-Con])	p ([D30-TAA] Vs [D30-Con])	Gene Title
**1454830_at**	Fbn2	1.61	0.0216	1.56	0.0284	fibrillin 2

Notes : Created from Advanced Analysis operation: significance Analysis.
#Entitylist : Filtered on Expression (20.0 - 100.0)th Percentile in the Raw Data
#Interpretation : Time-Treat
#Experiment: RPE-RMA DAYS 7-30
#corrected p-value cut-off:0.01
#Post-hoc test: SNK
#Selected Test : Oneway ANOVA
#p-value computation: Asymptotic
#Multiple Testing Correction: No Correction
#
Technology : Affymetrix.GeneChip.Mouse430_2
Owner : gxuser
Created On : Sat Jan 12 21:56:26 PST 2013

Probe Set ID	p	FC ([D07-D	Log FC ([D(	FC (abs) ([I	Regulation	FC ([D07-T.	Log FC ([D(	FC (abs) ([I
1415702_a_at	0.00894	1.015185	0.021742	1.015185	up	1.093298	0.128687	1.093298
1415773_at	0.006322	-1.18211	-0.24136	1.182109	down	-1.19338	-0.25505	1.193377
1415777_at	0.008835	1.075341	0.104795	1.075341	up	1.062542	0.087519	1.062542
1415824_at	0.003315	1.155559	0.20859	1.155559	up	1.333744	0.415482	1.333744
1415976_a_at	0.008289	1.160696	0.214991	1.160696	up	1.087685	0.121261	1.087685
1415989_at	0.008556	1.174844	0.23247	1.174844	up	1.178028	0.236373	1.178028
1415991_a_at	0.006844	1.094479	0.130245	1.094479	up	-1.06092	-0.08532	1.060923
1416062_at	0.008585	-1.03411	-0.04838	1.034106	down	-1.06476	-0.09052	1.064757
1416107_at	0.007561	1.03369	0.047804	1.03369	up	1.073309	0.102065	1.073309
1416261_at	0.005074	1.079632	0.110539	1.079632	up	1.176827	0.234903	1.176827
1416344_at	0.00259	1.069641	0.097126	1.069641	up	1.219508	0.2863	1.219508
1416492_at	0.008742	-1.05048	-0.07105	1.050479	down	1.040646	0.057479	1.040646
1416629_at	0.00103	-1.00474	-0.00682	1.00474	down	1.008283	0.0119	1.008283
1416773_at	6.01E-06	-1.02918	-0.0415	1.029182	down	1.127949	0.173702	1.127949
1416774_at	1.01E-04	-1.02867	-0.04077	1.028666	down	1.188389	0.249007	1.188389
1416855_at	0.009309	-1.04573	-0.06451	1.045731	down	-1.10595	-0.14529	1.105949
1416880_at	5.29E-04	-1.004	-0.00575	1.003997	down	-1.03445	-0.04886	1.034445
1416926_at	0.00416	1.140035	0.189079	1.140035	up	1.325094	0.406094	1.325094
1416958_at	2.27E-04	1.02707	0.038534	1.02707	up	1.022347	0.031885	1.022347
1416959_at	0.009468	1.174341	0.231851	1.174341	up	1.047089	0.066384	1.047089
1417015_at	0.008286	-1.06355	-0.08888	1.063546	down	-1.00783	-0.01126	1.007833
1417199_at	0.009083	-1.09515	-0.13113	1.095148	down	1.026006	0.037039	1.026006
1417239_at	0.006737	-1.07003	-0.09766	1.070034	down	-1.00602	-0.00866	1.006024
1417262_at	2.16E-04	-1.07264	-0.10117	1.072643	down	1.235584	0.305193	1.235584
1417300_at	0.002168	1.211411	0.276689	1.211411	up	1.088731	0.122647	1.088731
1417383_at	0.001159	1.066403	0.092753	1.066403	up	1.10957	0.150001	1.10957
1417385_at	0.001447	-1.0131	-0.01877	1.013097	down	1.047976	0.067606	1.047976
1417429_at	0.001043	1.1346	0.182184	1.1346	up	1.348863	0.431744	1.348863
1417443_at	0.003675	-1.0329	-0.0467	1.032898	down	1.001754	0.002529	1.001754
1417446_at	0.006362	1.071362	0.099446	1.071362	up	1.11278	0.154168	1.11278
1417575_at	0.007742	1.074742	0.10399	1.074742	up	1.136946	0.185164	1.136946
1417592_at	0.001867	-1.00767	-0.01103	1.007675	down	-1.14036	-0.18949	1.140363
1417602_at	2.63E-04	-1.05959	-0.0835	1.059588	down	1.069647	0.097134	1.069647

1417638_at	8.05E-04	1.166295	0.221933	1.166295	up		1.027868	0.039654	1.027868
1417653_at	0.009532	1.129057	0.175118	1.129057	up		-1.79419	-0.84333	1.794189
1417696_at	0.004991	1.047684	0.067203	1.047684	up		1.182926	0.242359	1.182926
1417785_at	0.009624	1.116224	0.158626	1.116224	up		1.25655	0.329468	1.25655
1417789_at	0.001589	-1.27799	-0.35388	1.277993	down		-1.21908	-0.28579	1.219081
1417810_a_at	0.002694	1.088214	0.121963	1.088214	up		1.064324	0.089938	1.064324
1417908_s_at	0.008531	1.037777	0.053496	1.037777	up		1.131188	0.177839	1.131188
1418231_at	0.00853	-1.10507	-0.14414	1.105071	down		1.308365	0.387765	1.308365
1418286_a_at	0.006957	-1.11511	-0.15718	1.115107	down		-1.04279	-0.06045	1.04279
1418293_at	0.002929	-1.01003	-0.0144	1.010032	down		1.010485	0.015047	1.010485
1418314_a_at	0.007529	1.03422	0.048544	1.03422	up		-1.68467	-0.75247	1.684674
1418319_at	0.006802	-1.06105	-0.08549	1.061046	down		1.056891	0.079826	1.056891
1418326_at	0.004965	1.120418	0.164036	1.120418	up		1.831225	0.872809	1.831225
1418336_at	7.47E-05	1.080185	0.111279	1.080185	up		1.263629	0.337573	1.263629
1418392_a_at	0.005132	-1.31208	-0.39185	1.312076	down		-1.29972	-0.3782	1.299724
1418436_at	0.005387	-1.02819	-0.04011	1.028191	down		1.008957	0.012864	1.008957
1418441_at	0.004911	-1.03385	-0.04803	1.033852	down		1.095368	0.131415	1.095368
1418470_at	0.002038	-1.12409	-0.16875	1.124088	down		-1.20188	-0.26529	1.201878
1418592_at	0.001482	-1.0831	-0.11517	1.083104	down		-1.02408	-0.03432	1.024077
1418604_at	0.008103	-1.02505	-0.03569	1.025049	down		1.128052	0.173833	1.128052
1418659_at	8.36E-04	-1.03354	-0.04759	1.033535	down		1.053888	0.075721	1.053888
1418660_at	7.86E-04	1.022204	0.031684	1.022204	up		1.063113	0.088295	1.063113
1418697_at	1.28E-05	1.338265	0.420364	1.338265	up		1.085571	0.118453	1.085571
1418719_at	0.006554	1.041276	0.058352	1.041276	up		-1.05927	-0.08306	1.059265
1418889_a_at	0.007255	1.154195	0.206888	1.154195	up		1.106126	0.145515	1.106126
1418901_at	0.005952	-1.09758	-0.13432	1.097578	down		1.00371	0.005342	1.00371
1418932_at	6.40E-04	1.030065	0.042735	1.030065	up		1.363305	0.447109	1.363305
1419028_at	8.76E-05	-1.08216	-0.11392	1.082162	down		-1.66449	-0.73508	1.664494
1419039_at	0.009035	1.258806	0.332055	1.258806	up		1.411035	0.496754	1.411035
1419070_at	4.94E-04	1.070416	0.098171	1.070416	up		-1.05005	-0.07046	1.050049
1419115_at	0.001012	1.045781	0.064581	1.045781	up		1.268635	0.343277	1.268635
1419146_a_at	0.005978	1.004072	0.005862	1.004072	up		-1.01896	-0.02709	1.018956
1419376_at	2.15E-05	1.212851	0.278402	1.212851	up		-1.05486	-0.07705	1.054857
1419377_at	0.0076	-1.04472	-0.06311	1.044718	down		1.101436	0.139386	1.101436
1419421_at	0.00269	1.02372	0.033821	1.02372	up		-1.26157	-0.33521	1.261565
1419482_at	0.002674	-1.0436	-0.06157	1.043598	down		-1.11953	-0.1629	1.119532
1419567_at	0.002704	-1.29403	-0.37187	1.294033	down		-1.08379	-0.11608	1.083789
1419603_at	0.009697	-1.68173	-0.74995	1.68173	down		-1.81399	-0.85916	1.813988
1419739_at	0.006598	1.052779	0.074203	1.052779	up		-1.80272	-0.85018	1.802722
1419764_at	0.009317	1.23904	0.309223	1.23904	up		4.859326	2.280756	4.859326
1419888_at	0.009577	1.095991	0.132236	1.095991	up		-1.19105	-0.25223	1.191048
1420671_x_at	0.003914	-1.40329	-0.48881	1.403291	down		-1.33053	-0.412	1.330532
1420772_a_at	8.14E-04	1.289225	0.366504	1.289225	up		1.53713	0.620239	1.53713
1420946_at	0.005865	1.056379	0.079127	1.056379	up		-1.03563	-0.05051	1.035628
1420971_at	0.00963	1.016087	0.023024	1.016087	up		1.019247	0.027503	1.019247
1421037_at	1.40E-05	-1.18277	-0.24217	1.182772	down		-1.19878	-0.26157	1.198781
1421048_a_at	0.001094	-1.12439	-0.16915	1.124395	down		-1.18173	-0.2409	1.181727

1421074_at	0.004079	-1.07373	-0.10263	1.073728	down		1.173511	0.230832	1.173511
1421075_s_at	8.64E-04	-1.04184	-0.05914	1.041843	down		1.116057	0.158411	1.116057
1421087_at	5.30E-06	1.092183	0.127215	1.092183	up		-1.02533	-0.03609	1.025331
1421103_at	0.004658	1.302386	0.381157	1.302386	up		1.232478	0.301562	1.232478
1421113_at	0.008893	1.026312	0.03747	1.026312	up		1.063574	0.08892	1.063574
1421114_a_at	0.002079	-1.19739	-0.2599	1.197392	down		-1.88078	-0.91133	1.880783
1421144_at	0.009349	1.015426	0.022085	1.015426	up		1.034153	0.04845	1.034153
1421166_at	0.002422	1.053831	0.075644	1.053831	up		1.036459	0.051662	1.036459
1421198_at	0.003265	-1.18494	-0.24482	1.184942	down		1.034942	0.04955	1.034942
1421256_at	2.15E-04	1.060943	0.085347	1.060943	up		-1.07287	-0.10148	1.072872
1421486_at	0.008562	1.105902	0.145223	1.105902	up		-1.0282	-0.04012	1.028199
1421727_at	0.008232	-1.02605	-0.0371	1.026051	down		1.061258	0.085776	1.061258
1421809_at	8.66E-04	1.118424	0.161467	1.118424	up		1.131555	0.178307	1.131555
1421868_a_at	0.00399	-1.2432	-0.31405	1.243196	down		-1.16551	-0.22096	1.165508
1421939_a_at	0.008286	1.09151	0.126326	1.09151	up		-1.00422	-0.00608	1.004224
1422018_at	0.002656	1.048962	0.068962	1.048962	up		1.039198	0.055471	1.039198
1422021_at	0.007846	1.042726	0.06036	1.042726	up		1.085354	0.118166	1.085354
1422072_a_at	0.002027	1.066883	0.093402	1.066883	up		-1.05948	-0.08336	1.05948
1422155_at	0.001845	-1.13168	-0.17847	1.13168	down		1.027716	0.039442	1.027716
1422186_s_at	0.001845	1.123348	0.167805	1.123348	up		1.005475	0.007878	1.005475
1422243_at	0.003061	-1.1196	-0.16299	1.119602	down		-1.15744	-0.21094	1.157445
1422587_at	2.36E-04	1.067706	0.094515	1.067706	up		1.190741	0.251859	1.190741
1422660_at	5.85E-04	1.083312	0.115449	1.083312	up		1.097364	0.134042	1.097364
1422692_at	0.005046	1.061906	0.086656	1.061906	up		1.110146	0.150749	1.110146
1422799_at	0.00676	1.067906	0.094784	1.067906	up		1.077022	0.107048	1.077022
1422821_s_at	0.007904	1.034674	0.049177	1.034674	up		-1.02342	-0.03339	1.023417
1423074_at	0.004841	1.051055	0.071838	1.051055	up		1.115584	0.157799	1.115584
1423093_at	0.007185	1.037146	0.052619	1.037146	up		-1.05438	-0.07639	1.054377
1423110_at	0.009612	1.458377	0.544364	1.458377	up		-1.00933	-0.01339	1.009326
1423145_a_at	0.002716	1.033725	0.047853	1.033725	up		-1.52745	-0.61113	1.527455
1423184_at	0.001297	-1.02801	-0.03986	1.028012	down		-1.2163	-0.2825	1.216297
1423228_at	0.009672	-1.05426	-0.07623	1.054258	down		1.212027	0.277422	1.212027
1423244_at	0.007527	-1.28071	-0.35694	1.28071	down		-1.20501	-0.26905	1.205015
1423319_at	0.009224	1.010453	0.015003	1.010453	up		-1.05646	-0.07924	1.056459
1423407_a_at	0.004536	1.41331	0.499077	1.41331	up		1.093253	0.128628	1.093253
1423414_at	0.001939	1.225107	0.292908	1.225107	up		1.141154	0.190494	1.141154
1423468_at	3.94E-04	-1.16854	-0.22471	1.168539	down		-1.54687	-0.62935	1.546873
1423571_at	0.00726	1.098716	0.135819	1.098716	up		1.245204	0.316382	1.245204
1423738_at	0.006779	-1.03831	-0.05424	1.038309	down		1.125985	0.171187	1.125985
1423741_at	0.004796	1.11208	0.153261	1.11208	up		1.14232	0.191967	1.14232
1423777_at	0.006086	1.030167	0.042878	1.030167	up		1.178279	0.236681	1.178279
1423883_at	0.001821	-1.07555	-0.10508	1.075552	down		-1.30697	-0.38623	1.306971
1423886_at	0.002745	-1.06708	-0.09366	1.067076	down		-1.20096	-0.26418	1.200957
1424038_a_at	0.009135	1.16956	0.225966	1.16956	up		1.265873	0.340132	1.265873
1424147_at	0.006007	1.052951	0.074438	1.052951	up		1.095997	0.132244	1.095997
1424175_at	0.001917	1.077346	0.107481	1.077346	up		1.040929	0.057872	1.040929
1424196_at	0.00745	1.096749	0.133233	1.096749	up		1.224117	0.291742	1.224117

1424233_at	9.29E-05	-1.37983	-0.4645	1.379835	down	-1.71057	-0.77448	1.710568
1424234_s_at	0.002532	-1.42991	-0.51593	1.429914	down	-1.85234	-0.88935	1.852342
1424253_at	9.65E-04	-1.00251	-0.00362	1.00251	down	1.110535	0.151254	1.110535
1424287_at	0.00486	1.036929	0.052318	1.036929	up	-1.14871	-0.20001	1.14871
1424336_at	0.002738	-1.16543	-0.22087	1.165434	down	-1.08415	-0.11656	1.08415
1424598_at	1.16E-04	-1.02831	-0.04028	1.028312	down	-1.09825	-0.13521	1.098251
1424749_at	0.009488	1.028675	0.040787	1.028675	up	-1.01414	-0.02026	1.014139
1424827_a_at	0.005732	-1.00299	-0.00431	1.00299	down	1.041033	0.058016	1.041033
1424857_a_at	0.009771	-1.13408	-0.18153	1.134084	down	-1.19112	-0.25231	1.191116
1424913_at	0.003863	-1.33271	-0.41436	1.332711	down	1.292804	0.370504	1.292804
1425095_at	0.006221	-1.17527	-0.233	1.175273	down	-1.21956	-0.28637	1.219564
1425099_a_at	2.58E-06	1.265517	0.339727	1.265517	up	1.678605	0.747263	1.678605
1425187_at	0.001689	1.033036	0.04689	1.033036	up	1.153692	0.206258	1.153692
1425211_at	0.003007	-1.0268	-0.03815	1.026797	down	1.051673	0.072686	1.051673
1425234_at	0.003794	1.075076	0.104438	1.075076	up	-1.04329	-0.06115	1.043295
1425328_at	0.002548	-1.05588	-0.07845	1.05588	down	-1.02446	-0.03487	1.024462
1425451_s_at	0.009564	1.113174	0.154679	1.113174	up	2.660272	1.411574	2.660272
1425608_at	9.96E-04	1.139189	0.188007	1.139189	up	1.152059	0.204214	1.152059
1425846_a_at	0.0092	1.293703	0.371506	1.293703	up	1.113735	0.155406	1.113735
1425890_at	6.16E-04	-1.08645	-0.11962	1.086446	down	-1.14559	-0.1961	1.145593
1425937_a_at	0.006534	1.036054	0.051099	1.036054	up	1.058682	0.082269	1.058682
1426036_a_at	0.002566	1.157405	0.210893	1.157405	up	-1.04593	-0.06479	1.045929
1426080_a_at	0.009487	-1.15889	-0.21275	1.158895	down	-1.0638	-0.08923	1.0638
1426127_x_at	0.007744	1.217244	0.283618	1.217244	up	1.097922	0.134776	1.097922
1426227_s_at	0.002697	1.008892	0.012771	1.008892	up	1.108315	0.148368	1.108315
1426234_s_at	0.009701	-1.06547	-0.09149	1.065473	down	1.061342	0.08589	1.061342
1426360_at	5.70E-05	-1.09602	-0.13227	1.096019	down	-1.08145	-0.11296	1.081447
1426458_at	0.003741	1.109186	0.149501	1.109186	up	-1.05048	-0.07105	1.050478
1426462_at	0.008501	-1.07085	-0.09876	1.070851	down	-1.0594	-0.08324	1.059396
1426464_at	0.001398	1.244764	0.315872	1.244764	up	-1.03433	-0.04869	1.034328
1426849_at	8.83E-04	1.018497	0.026442	1.018497	up	1.118149	0.161112	1.118149
1426987_at	0.001138	-1.08448	-0.117	1.084477	down	1.102507	0.140788	1.102507
1427056_at	0.008824	-1.07372	-0.10262	1.073723	down	-1.34446	-0.42702	1.344457
1427168_a_at	0.009903	1.098892	0.13605	1.098892	up	-1.21426	-0.28008	1.214264
1427169_at	0.00726	-1.00368	-0.0053	1.003679	down	-1.10254	-0.14083	1.102538
1427332_at	0.003912	1.010019	0.014382	1.010019	up	1.048576	0.068431	1.048576
1427342_at	0.005208	1.044375	0.062639	1.044375	up	1.126045	0.171264	1.126045
1427450_x_at	0.001748	1.183658	0.243252	1.183658	up	1.070191	0.097868	1.070191
1427471_at	0.009824	-1.03223	-0.04577	1.032233	down	1.114634	0.15657	1.114634
1427580_a_at	5.70E-04	1.128466	0.174363	1.128466	up	-1.04983	-0.07016	1.049831
1427608_a_at	0.004167	-1.12267	-0.16693	1.12267	down	-1.08341	-0.11558	1.083412
1427958_at	0.001174	-1.0122	-0.0175	1.012204	down	1.132269	0.179216	1.132269
1428047_s_at	0.001438	1.029275	0.041628	1.029275	up	1.215106	0.281082	1.215106
1428212_x_at	0.005216	1.017299	0.024743	1.017299	up	-1.06178	-0.08649	1.061785
1428543_at	0.00272	-1.15472	-0.20754	1.154717	down	-1.13899	-0.18775	1.138985
1428577_at	0.004566	1.073619	0.102482	1.073619	up	-1.08337	-0.11552	1.083367
1428728_at	0.00127	1.011024	0.015818	1.011024	up	1.022835	0.032574	1.022835

1429003_at	0.006513	-1.01377	-0.01974	1.013773	down	-1.09292	-0.12818	1.092918
1429758_at	0.005298	1.046128	0.06506	1.046128	up	1.075117	0.104493	1.075117
1430205_a_at	0.009989	1.009859	0.014153	1.009859	up	1.158113	0.211776	1.158113
1431072_a_at	0.002404	-1.14972	-0.20129	1.149722	down	1.046577	0.065679	1.046577
1432436_a_at	0.008648	1.055651	0.078134	1.055651	up	1.269642	0.344422	1.269642
1433504_at	6.19E-04	1.17825	0.236646	1.17825	up	1.153066	0.205475	1.153066
1433569_x_at	0.008408	-1.00537	-0.00773	1.005372	down	1.09656	0.132985	1.09656
1433909_at	0.005673	-1.14722	-0.19814	1.147217	down	-1.06887	-0.09609	1.068873
1434363_x_at	0.005558	-1.10868	-0.14884	1.108679	down	-1.01473	-0.0211	1.01473
1434569_at	0.004819	-1.14573	-0.19626	1.145727	down	-1.02475	-0.03527	1.024746
1434643_at	0.008077	-1.03695	-0.05234	1.036948	down	-1.08645	-0.11962	1.086452
1434992_at	0.00233	1.093777	0.129319	1.093777	up	-1.02222	-0.03171	1.022221
1435089_at	9.92E-04	1.014991	0.021466	1.014991	up	-1.05316	-0.07472	1.053159
1435160_at	0.009729	-1.01965	-0.02808	1.019651	down	-1.13052	-0.17698	1.130518
1435802_at	0.004181	1.215417	0.281451	1.215417	up	1.248474	0.320165	1.248474
1435950_at	9.64E-04	-1.12696	-0.17243	1.126958	down	1.024975	0.035588	1.024975
1435979_a_at	0.007021	-1.02286	-0.03261	1.022864	down	1.015861	0.022703	1.015861
1436179_a_at	0.00874	1.076491	0.106336	1.076491	up	1.193671	0.255405	1.193671
1436348_at	0.002635	1.012574	0.018027	1.012574	up	-1.06713	-0.09373	1.067128
1436863_at	0.007849	1.001488	0.002145	1.001488	up	-1.13689	-0.1851	1.136892
1436935_x_at	0.005958	1.031336	0.044515	1.031336	up	-1.00458	-0.00659	1.004581
1436986_at	0.00505	-1.05645	-0.07922	1.056446	down	-1.0394	-0.05576	1.039405
1437398_a_at	0.008086	-1.05119	-0.07202	1.051189	down	1.101048	0.138878	1.101048
1437413_x_at	0.002906	1.031152	0.044257	1.031152	up	-1.03625	-0.05137	1.036247
1438033_at	0.001432	1.056304	0.079025	1.056304	up	-1.01483	-0.02124	1.014833
1438370_x_at	0.00825	1.084953	0.117633	1.084953	up	1.267359	0.341825	1.267359
1438427_at	0.004908	1.176703	0.23475	1.176703	up	1.076753	0.106687	1.076753
1439423_x_at	0.001937	-1.20495	-0.26897	1.20495	down	-1.33185	-0.41343	1.331851
1440195_at	0.001159	1.013389	0.019188	1.013389	up	-1.14826	-0.19945	1.14826
1440831_at	0.003536	-1.0252	-0.0359	1.025196	down	-1.08022	-0.11132	1.080216
1447462_at	0.001215	-1.07085	-0.09875	1.070846	down	1.019854	0.028363	1.019854
1448137_at	0.007487	1.067531	0.094278	1.067531	up	1.189375	0.250204	1.189375
1448178_a_at	0.005358	-1.00888	-0.01276	1.008884	down	1.083703	0.11597	1.083703
1448276_at	0.001805	1.227302	0.29549	1.227302	up	1.18029	0.239141	1.18029
1448293_at	0.0045	-1.05428	-0.07626	1.054285	down	1.021835	0.031163	1.021835
1448297_a_at	0.008214	1.041513	0.058681	1.041513	up	1.084845	0.117489	1.084845
1448503_at	0.001467	-1.05511	-0.07739	1.05511	down	-1.07075	-0.09862	1.070747
1448606_at	0.005572	-1.04244	-0.05996	1.042436	down	-1.12575	-0.17089	1.125752
1448916_at	0.004156	-1.02288	-0.03264	1.022881	down	-1.06294	-0.08806	1.062936
1448939_at	0.002264	-1.19709	-0.25954	1.197094	down	-1.00733	-0.01053	1.007326
1448990_a_at	0.003797	1.269927	0.344745	1.269927	up	1.103811	0.142493	1.103811
1449102_at	0.001978	-1.51509	-0.5994	1.515085	down	-1.4225	-0.50843	1.422505
1449203_at	0.009058	-1.09491	-0.13081	1.09491	down	1.208414	0.273115	1.208414
1449216_at	0.006798	-1.0142	-0.02035	1.014202	down	-1.12852	-0.17444	1.128524
1449291_a_at	1.73E-04	-1.0961	-0.13239	1.096105	down	-1.04192	-0.05924	1.041919
1449307_at	0.009306	1.044494	0.062804	1.044494	up	1.081735	0.113347	1.081735
1449329_at	0.009367	1.044772	0.063189	1.044772	up	1.07763	0.107862	1.07763

1449351_s_at	0.00822	1.059492	0.083372	1.059492	up		1.261329	0.334945	1.261329
1449408_at	0.006537	1.090381	0.124832	1.090381	up		1.036971	0.052375	1.036971
1449521_at	0.006314	1.041088	0.058092	1.041088	up		-1.02455	-0.03499	1.024551
1449536_at	0.004803	-1.18605	-0.24617	1.186051	down		-1.10578	-0.14507	1.105784
1449824_at	0.001303	-1.06272	-0.08776	1.062721	down		1.131438	0.178157	1.131438
1449845_a_at	0.001084	1.250496	0.3225	1.250496	up		1.240848	0.311326	1.240848
1449847_a_at	3.51E-04	-1.08202	-0.11373	1.082021	down		-1.19505	-0.25707	1.195051
1449851_at	6.31E-04	1.321228	0.401879	1.321228	up		1.618883	0.694999	1.618883
1449931_at	0.002002	1.060604	0.084885	1.060604	up		1.236655	0.306443	1.236655
1450506_a_at	0.002685	1.354669	0.437941	1.354669	up		1.238463	0.308551	1.238463
1450625_at	0.004088	1.250961	0.323036	1.250961	up		-1.00958	-0.01376	1.009583
1450652_at	0.008984	1.144854	0.195163	1.144854	up		-1.06376	-0.08918	1.063765
1450826_a_at	1.24E-05	1.531731	0.615163	1.531731	up		6.248886	2.643599	6.248886
1450854_at	0.001812	-1.06498	-0.09083	1.064984	down		1.128022	0.173796	1.128022
1450863_a_at	0.00996	1.236867	0.306691	1.236867	up		1.321572	0.402255	1.321572
1450982_at	0.001344	1.088727	0.122642	1.088727	up		1.577469	0.657612	1.577469
1451158_at	0.007316	-1.04626	-0.06524	1.046257	down		-1.05647	-0.07925	1.056469
1451188_at	0.008267	-1.0726	-0.10112	1.072605	down		-1.05903	-0.08274	1.059028
1451418_a_at	0.009921	-1.10194	-0.14004	1.101935	down		-1.42897	-0.51497	1.428966
1451478_at	6.47E-04	1.453623	0.539653	1.453623	up		1.409524	0.495208	1.409524
1451591_a_at	0.00555	-1.01888	-0.02698	1.018877	down		1.003043	0.004383	1.003043
1451773_s_at	5.39E-06	1.022119	0.031563	1.022119	up		1.154894	0.20776	1.154894
1452199_at	0.005712	1.019486	0.027842	1.019486	up		1.230693	0.299471	1.230693
1452228_at	0.004268	1.059502	0.083386	1.059502	up		-1.11012	-0.15072	1.110125
1452345_at	0.00334	-1.23541	-0.30499	1.235408	down		-1.74084	-0.79978	1.740835
1452387_a_at	0.003943	1.078503	0.10903	1.078503	up		1.37212	0.456407	1.37212
1452441_at	0.008984	-1.04449	-0.06279	1.044485	down		-1.00828	-0.01189	1.008279
1452470_at	9.43E-04	1.00412	0.005932	1.00412	up		-1.14126	-0.19063	1.14126
1452481_at	0.008576	-1.11149	-0.15249	1.111489	down		1.076712	0.106632	1.076712
1453169_a_at	0.006605	-1.02323	-0.03313	1.023229	down		-1.01719	-0.02459	1.01719
1453208_at	0.006942	-1.07707	-0.10711	1.07707	down		1.039154	0.05541	1.039154
1453678_at	5.21E-04	1.037013	0.052434	1.037013	up		2.01401	1.010071	2.01401
1454670_at	0.005388	1.114419	0.156292	1.114419	up		1.000154	2.22E-04	1.000154
1454675_at	0.002549	1.072539	0.10103	1.072539	up		1.035567	0.05042	1.035567
1454747_a_at	0.00129	1.074894	0.104194	1.074894	up		-1.06865	-0.09579	1.068651
1455047_at	0.001518	1.049215	0.06931	1.049215	up		1.202873	0.266484	1.202873
1455526_at	0.005298	1.101202	0.13908	1.101202	up		1.123349	0.167806	1.123349
1455981_at	0.007704	-1.14566	-0.19618	1.145664	down		-1.25115	-0.32326	1.251152
1456013_x_at	0.00188	1.165163	0.220531	1.165163	up		1.171754	0.22867	1.171754
1456120_at	0.001488	-1.05488	-0.07708	1.054878	down		-1.03072	-0.04366	1.030722
1456488_at	0.004304	1.067558	0.094314	1.067558	up		1.008805	0.012647	1.008805
1456733_x_at	0.003629	1.140429	0.189577	1.140429	up		-1.18666	-0.24691	1.186663
1459927_at	0.00787	-1.06594	-0.09213	1.065943	down		1.008232	0.011827	1.008232
1460038_at	0.00773	-1.11584	-0.15813	1.115837	down		-1.2687	-0.34335	1.268695
1460243_at	0.00934	1.031996	0.045438	1.031996	up		-1.02173	-0.03101	1.02173
1460327_at	5.75E-04	-1.05594	-0.07853	1.055942	down		-1.0991	-0.13633	1.099103
1460370_at	0.00623	1.053645	0.075388	1.053645	up		-1.08696	-0.1203	1.086962

1460420_a_at	4.59E-04	1.034901	0.049493	1.034901	up	1.034011	0.048251	1.034011
1428281_at	0.005777	1.102635	0.140956	1.102635	up	1.237669	0.307626	1.237669
1428332_at	5.37E-04	1.051781	0.072834	1.051781	up	1.179356	0.238	1.179356
1428487_s_at	0.006749	-1.06976	-0.09729	1.069765	down	-1.09667	-0.13313	1.096671
1428496_at	0.003835	1.035778	0.050714	1.035778	up	1.138186	0.186736	1.138186
1428889_at	0.008356	1.103663	0.1423	1.103663	up	1.093874	0.129447	1.093874
1428958_at	0.00629	-1.05531	-0.07766	1.055308	down	-1.10709	-0.14677	1.107091
1428987_at	0.006064	1.073227	0.101955	1.073227	up	1.160207	0.214382	1.160207
1429026_at	0.001289	1.018977	0.027121	1.018977	up	-1.09317	-0.12852	1.093173
1429093_at	0.008932	-1.08575	-0.11869	1.085752	down	-1.21053	-0.27564	1.210527
1429464_at	0.007601	-1.07235	-0.10077	1.072347	down	-1.00789	-0.01134	1.007891
1429539_at	0.008731	1.022751	0.032455	1.022751	up	1.145773	0.196321	1.145773
1429984_at	0.004592	-1.14561	-0.19611	1.145605	down	-1.11856	-0.16164	1.118557
1430435_at	0.008981	-1.0436	-0.06157	1.043604	down	-1.07425	-0.10332	1.074246
1430452_at	0.00313	1.031598	0.04488	1.031598	up	-1.08105	-0.11243	1.081047
1430515_s_at	0.008511	1.035297	0.050044	1.035297	up	1.058697	0.08229	1.058697
1430729_at	0.008817	1.187029	0.247355	1.187029	up	1.046581	0.065684	1.046581
1430786_at	0.007653	-1.18119	-0.24025	1.181195	down	-1.79531	-0.84423	1.795305
1430871_at	0.006829	1.141596	0.191052	1.141596	up	-1.06575	-0.09187	1.065754
1430997_at	0.002155	-1.10702	-0.14669	1.107025	down	1.061571	0.086201	1.061571
1431073_at	0.001331	-1.01495	-0.02142	1.014955	down	1.040933	0.057877	1.040933
1431089_at	0.003951	1.060672	0.084979	1.060672	up	1.249173	0.320974	1.249173
1431148_at	0.006829	1.172296	0.229337	1.172296	up	1.368483	0.452577	1.368483
1431273_at	0.009242	-1.03141	-0.04461	1.031407	down	1.032516	0.046164	1.032516
1431325_at	0.009143	-1.24259	-0.31335	1.242588	down	-1.13553	-0.18336	1.135525
1432141_x_at	0.006811	-1.00534	-0.00769	1.005344	down	1.0049	0.007052	1.0049
1433131_at	0.009836	-1.03614	-0.05122	1.036139	down	-1.1387	-0.18739	1.138705
1433176_at	0.002316	-1.16629	-0.22193	1.166292	down	-1.0546	-0.07669	1.054598
1433637_at	0.005784	1.098803	0.135932	1.098803	up	1.129237	0.175349	1.129237
1433692_at	5.82E-04	1.135697	0.183578	1.135697	up	1.144034	0.19413	1.144034
1433849_at	0.009818	-1.03833	-0.05427	1.038332	down	-1.01629	-0.02331	1.01629
1434058_at	0.008567	-1.02798	-0.03982	1.027984	down	-1.0321	-0.04559	1.032103
1434477_at	0.003395	-1.08147	-0.11299	1.08147	down	-1.26108	-0.33466	1.261077
1434817_s_at	0.001171	1.26141	0.335037	1.26141	up	1.216946	0.283265	1.216946
1435059_at	0.006064	1.168612	0.224796	1.168612	up	1.082665	0.114586	1.082665
1435188_at	5.32E-05	1.162406	0.217114	1.162406	up	1.021055	0.03006	1.021055
1435229_at	0.008693	1.035116	0.049792	1.035116	up	1.095595	0.131714	1.095595
1435280_at	0.005971	1.095204	0.1312	1.095204	up	-1.15613	-0.20931	1.156132
1435366_at	0.004613	1.323603	0.404471	1.323603	up	1.23463	0.304079	1.23463
1435472_at	0.009637	-1.05091	-0.07164	1.050911	down	1.023432	0.033415	1.023432
1435475_at	0.008119	-1.01078	-0.01547	1.010781	down	1.069463	0.096886	1.069463
1435576_at	0.006076	1.033949	0.048165	1.033949	up	1.061274	0.085797	1.061274
1435940_at	6.19E-04	1.068195	0.095175	1.068195	up	1.162256	0.216927	1.162256
1436013_at	0.004957	-1.23923	-0.30944	1.23923	down	-1.34669	-0.42942	1.346694
1436043_at	0.005609	1.125068	0.170012	1.125068	up	1.147757	0.198817	1.147757
1436055_at	0.003296	1.694296	0.760686	1.694296	up	1.041206	0.058256	1.041206
1436111_at	0.004498	-1.03976	-0.05626	1.039765	down	-1.07114	-0.09915	1.071144

1436438_s_at	0.009762	1.031935	0.045353	1.031935	up	1.012858	0.018431	1.012858
1436444_at	0.004742	1.102036	0.140171	1.102036	up	1.018703	0.026733	1.018703
1436476_at	0.007881	1.039976	0.05655	1.039976	up	-1.05031	-0.07082	1.05031
1436646_at	0.005697	1.264652	0.33874	1.264652	up	-1.05366	-0.07541	1.053662
1437065_at	0.009864	1.147093	0.197982	1.147093	up	-1.1668	-0.22256	1.166799
1437387_at	0.005879	-1.00416	-0.00599	1.004158	down	1.00048	6.93E-04	1.00048
1437771_at	0.002457	-1.02038	-0.02911	1.020381	down	-1.29424	-0.3721	1.294236
1437820_at	4.41E-04	-1.1108	-0.1516	1.110798	down	-1.04228	-0.05974	1.042277
1437857_at	0.007079	1.055555	0.078002	1.055555	up	1.206226	0.2705	1.206226
1437879_at	0.004123	-1.02186	-0.03119	1.021855	down	1.02639	0.037579	1.02639
1438229_at	0.008454	1.066047	0.092271	1.066047	up	-1.0661	-0.09235	1.066103
1438275_at	0.007865	-1.05859	-0.08214	1.05859	down	1.109128	0.149426	1.109128
1438412_at	0.001201	-1.02824	-0.04018	1.028239	down	-1.02294	-0.03272	1.022936
1438424_at	0.004238	1.164892	0.220197	1.164892	up	1.285701	0.362555	1.285701
1438425_at	6.61E-04	1.352303	0.435418	1.352303	up	1.063952	0.089433	1.063952
1438470_at	0.008853	-1.03989	-0.05644	1.039895	down	-1.11586	-0.15816	1.115861
1438484_at	0.00282	-1.09669	-0.13316	1.096693	down	1.124831	0.169709	1.124831
1438490_at	0.002901	1.043874	0.061947	1.043874	up	1.285679	0.36253	1.285679
1438728_at	7.08E-05	1.029525	0.041978	1.029525	up	1.248152	0.319794	1.248152
1439095_at	0.00318	1.079307	0.110105	1.079307	up	1.007166	0.010301	1.007166
1439275_s_at	0.007544	-1.02861	-0.04069	1.028606	down	-1.22797	-0.29628	1.227973
1439340_at	0.001593	1.053308	0.074928	1.053308	up	1.07588	0.105517	1.07588
1439488_at	0.003371	1.087275	0.120716	1.087275	up	-1.00539	-0.00775	1.005388
1439555_at	0.001456	-1.13174	-0.17854	1.131739	down	-1.09021	-0.1246	1.090209
1439649_at	0.008778	1.22276	0.290141	1.22276	up	1.26503	0.339172	1.26503
1439671_at	0.007035	-1.07058	-0.09839	1.070575	down	1.018945	0.027076	1.018945
1440041_at	0.004563	-1.18227	-0.24156	1.182274	down	-1.1256	-0.1707	1.125601
1440064_at	0.005639	1.137473	0.185832	1.137473	up	1.113506	0.15511	1.113506
1440200_at	0.00408	1.003623	0.005217	1.003623	up	1.153784	0.206373	1.153784
1440364_a_at	0.004444	-1.03014	-0.04285	1.030144	down	-1.06881	-0.09601	1.068814
1440390_at	0.003008	-1.04216	-0.05958	1.042159	down	-1.10167	-0.13969	1.10167
1440665_at	0.007826	1.041397	0.05852	1.041397	up	-1.1315	-0.17824	1.1315
1440715_s_at	1.35E-04	-1.08424	-0.11669	1.084242	down	1.068034	0.094958	1.068034
1440719_at	0.00898	-1.05395	-0.07581	1.053951	down	-1.07371	-0.1026	1.07371
1440943_at	0.006137	1.20319	0.266865	1.20319	up	1.026238	0.037366	1.026238
1440972_at	0.005645	-1.07499	-0.10432	1.074989	down	-1.1414	-0.1908	1.141397
1441338_at	0.002565	-1.17954	-0.23823	1.179541	down	-1.36389	-0.44773	1.363892
1441376_at	0.003968	1.038874	0.05502	1.038874	up	1.092865	0.128115	1.092865
1441585_at	0.008677	-1.0115	-0.01649	1.011498	down	-1.01879	-0.02686	1.018791
1441923_s_at	0.005809	-1.00195	-0.00281	1.001949	down	-1.16595	-0.22151	1.165954
1441924_x_at	0.007393	-1.04676	-0.06593	1.046758	down	-1.17538	-0.23313	1.175384
1441946_at	0.002168	-1.3912	-0.47633	1.391198	down	-1.7679	-0.82203	1.767896
1441952_x_at	0.005981	1.038682	0.054754	1.038682	up	-1.1028	-0.14117	1.102798
1442119_at	0.008598	1.17709	0.235224	1.17709	up	1.302737	0.381545	1.302737
1442131_at	0.00223	-1.21772	-0.28418	1.217719	down	-1.11046	-0.15115	1.110457
1442648_at	0.009873	1.040535	0.057325	1.040535	up	1.154468	0.207228	1.154468
1442686_at	0.005158	1.124532	0.169324	1.124532	up	1.117887	0.160774	1.117887

1443066_at	0.007025	-1.15569	-0.20875	1.155688	down	-1.08857	-0.12243	1.088565
1443878_at	0.006149	-1.05695	-0.07991	1.056953	down	1.006949	0.009991	1.006949
1443975_at	0.00917	-1.3486	-0.43146	1.348596	down	-1.19512	-0.25715	1.195119
1444089_at	0.001669	1.110839	0.15165	1.110839	up	1.114066	0.155834	1.114066
1444235_at	0.007557	1.339864	0.422086	1.339864	up	1.184458	0.244227	1.184458
1444255_at	8.37E-04	1.149672	0.201222	1.149672	up	1.092387	0.127484	1.092387
1444571_at	0.009935	1.148168	0.199333	1.148168	up	1.005467	0.007866	1.005467
1444766_at	0.005374	-1.18636	-0.24654	1.186356	down	-1.15986	-0.21395	1.159863
1444789_at	0.007342	-1.13053	-0.177	1.130527	down	-1.27332	-0.3486	1.273325
1445021_at	6.65E-04	-1.03934	-0.05566	1.039337	down	-1.05169	-0.0727	1.051685
1445097_at	4.40E-04	-1.16086	-0.21519	1.160858	down	-1.10622	-0.14564	1.10622
1446773_at	0.006942	-1.08488	-0.11754	1.084883	down	-1.04107	-0.05807	1.041071
1446946_at	0.002782	1.039729	0.056207	1.039729	up	-1.12018	-0.16373	1.120183
1447072_at	8.11E-04	1.017116	0.024485	1.017116	up	1.005069	0.007294	1.005069
1447171_at	0.006345	1.156314	0.209534	1.156314	up	-1.15505	-0.20796	1.155053
1447433_at	0.002786	-1.07584	-0.10546	1.075835	down	-1.03822	-0.05411	1.038215
1447500_at	0.007211	-1.18772	-0.24819	1.187716	down	-1.30016	-0.37869	1.300157
1447724_x_at	0.001546	-1.00384	-0.00554	1.003845	down	1.010831	0.015542	1.010831
1447779_x_at	0.004995	1.03808	0.053917	1.03808	up	-1.02862	-0.0407	1.028616
1452739_at	0.00288	1.081127	0.112535	1.081127	up	1.056793	0.079693	1.056793
1452853_at	0.00658	-1.08241	-0.11424	1.082405	down	1.090915	0.125538	1.090915
1452918_at	0.005711	-1.10747	-0.14726	1.107466	down	1.065703	0.091805	1.065703
1453001_at	0.003076	-1.09851	-0.13555	1.098514	down	-1.0918	-0.12671	1.0918
1453007_at	0.00651	-1.01552	-0.02222	1.015518	down	1.159033	0.212921	1.159033
1453224_at	0.006472	1.152093	0.204257	1.152093	up	1.203402	0.267119	1.203402
1453326_at	0.003666	1.04463	0.062992	1.04463	up	1.043807	0.061855	1.043807
1453402_at	0.002229	1.203831	0.267632	1.203831	up	1.103873	0.142575	1.103873
1453421_at	0.001402	-1.00607	-0.00873	1.006068	down	1.021592	0.030819	1.021592
1453502_at	0.008185	1.301001	0.379622	1.301001	up	1.052303	0.07355	1.052303
1453649_at	0.006883	1.065056	0.09093	1.065056	up	1.035032	0.049675	1.035032
1453927_at	0.008573	-1.05807	-0.08144	1.058072	down	1.137383	0.185718	1.137383
1454062_at	0.002881	-1.10587	-0.14519	1.105872	down	-1.21256	-0.27806	1.212562
1454166_at	0.002226	1.074914	0.104221	1.074914	up	1.026175	0.037277	1.026175
1454297_at	0.00368	-1.08636	-0.1195	1.086356	down	-1.02412	-0.03438	1.024116
1454320_at	0.008774	1.079038	0.109746	1.079038	up	1.03631	0.051456	1.03631
1454494_at	0.001947	-1.11449	-0.15638	1.114489	down	-1.04253	-0.06009	1.042528
1454658_at	0.006344	1.083279	0.115405	1.083279	up	1.193676	0.255411	1.193676
1454902_at	0.004367	-1.04191	-0.05922	1.041906	down	1.100497	0.138155	1.100497
1455293_at	1.12E-05	-1.07594	-0.10559	1.075937	down	1.058491	0.082009	1.058491
1455306_at	0.001412	1.097868	0.134705	1.097868	up	1.097056	0.133638	1.097056
1455311_at	0.006507	1.052355	0.073621	1.052355	up	1.173776	0.231157	1.173776
1455658_at	0.001981	-1.08015	-0.11123	1.080149	down	-1.05005	-0.07046	1.050049
1455704_at	0.008923	1.150819	0.202661	1.150819	up	1.049266	0.06938	1.049266
1455884_at	0.006257	-1.03435	-0.04872	1.03435	down	1.049757	0.070056	1.049757
1456144_at	0.006324	-1.0067	-0.00964	1.006704	down	-1.10645	-0.14594	1.106452
1456156_at	0.003299	-1.20055	-0.26369	1.200548	down	-1.36453	-0.4484	1.36453
1456210_at	6.31E-04	-1.00199	-0.00286	1.001987	down	-1.02398	-0.03419	1.023983

1456308_x_at	0.002259	1.086083	0.119135	1.086083	up	1.021811	0.031128	1.021811
1456653_a_at	2.30E-04	1.130129	0.176487	1.130129	up	1.134676	0.182281	1.134676
1456685_at	0.001906	-1.0679	-0.09477	1.067895	down	1.04503	0.063545	1.04503
1456937_at	1.33E-04	-1.11032	-0.15098	1.110324	down	-1.02131	-0.03043	1.021314
1456976_at	0.002746	1.129066	0.17513	1.129066	up	1.061103	0.085565	1.061103
1457348_at	0.008877	-1.03423	-0.04855	1.034228	down	-1.13065	-0.17716	1.130654
1457600_x_at	0.007963	1.031798	0.04516	1.031798	up	-1.01556	-0.02227	1.015558
1457625_s_at	0.005807	-1.18308	-0.24255	1.183079	down	-1.11302	-0.15448	1.113023
1457643_x_at	0.008501	1.000637	9.19E-04	1.000637	up	-1.25335	-0.32579	1.253353
1457654_at	0.007406	-1.24396	-0.31494	1.243962	down	-1.08822	-0.12197	1.088216
1457671_at	0.004172	-1.01868	-0.0267	1.018678	down	-1.20326	-0.26694	1.203255
1457740_at	0.009452	1.061019	0.08545	1.061019	up	1.165049	0.22039	1.165049
1457900_at	0.009552	-1.05442	-0.07645	1.054419	down	1.182044	0.241284	1.182044
1457927_at	1.07E-04	1.090857	0.125462	1.090857	up	1.257189	0.330201	1.257189
1458040_at	0.00381	-1.00447	-0.00644	1.004475	down	-1.06982	-0.09736	1.069816
1458343_x_at	0.002387	1.06751	0.094249	1.06751	up	1.113295	0.154835	1.113295
1458421_at	0.009179	-1.21409	-0.27988	1.214091	down	-1.06164	-0.08629	1.061638
1460446_at	0.006803	1.126452	0.171786	1.126452	up	1.159167	0.213088	1.159167
1460527_at	0.009652	-1.08837	-0.12216	1.088366	down	-1.09891	-0.13608	1.098915
1460584_at	0.004137	-1.00567	-0.00815	1.005668	down	-1.10026	-0.13785	1.100264
1460606_at	0.006237	-1.05626	-0.07897	1.056263	down	1.011075	0.01589	1.011075

Regulation	FC ([D30-D	Log FC ([D3	FC (abs) ([	Regulation	FC ([D30-T	Log FC ([D3	FC (abs) ([	Regulation	[D07-Con](
up	1.089073	0.1231	1.089073	up	1.130751	0.177281	1.130751	up	2817.307
down	-1.11653	-0.15903	1.116533	down	1.001277	0.001841	1.001277	up	729.6677
up	-1.02446	-0.03487	1.024462	down	1.078205	0.108632	1.078205	up	24.3854
up	-1.03367	-0.04778	1.033674	down	1.058069	0.081433	1.058069	up	889.3624
up	1.158179	0.211858	1.158179	up	1.046933	0.066169	1.046933	up	77.59863
up	1.26351	0.337437	1.26351	up	1.038673	0.054741	1.038673	up	266.1272
down	-1.02819	-0.04011	1.028189	down	-1.03198	-0.04542	1.031984	down	1358.737
down	1.058238	0.081665	1.058238	up	-1.07596	-0.10563	1.075962	down	128.1126
up	-1.8998	-0.92585	1.899804	down	-1.62	-0.69599	1.619999	down	370.2874
up	-1.03926	-0.05555	1.039258	down	-1.01902	-0.02718	1.019016	down	341.3287
up	1.045202	0.063781	1.045202	up	-1.0948	-0.13066	1.094796	down	4928.682
up	-1.10674	-0.14631	1.106738	down	-1.05413	-0.07606	1.054135	down	74.03568
up	1.280619	0.356841	1.280619	up	1.093114	0.128444	1.093114	up	237.7298
up	-1.02516	-0.03585	1.025162	down	1.062162	0.087003	1.062162	up	528.2935
up	-1.00431	-0.0062	1.004309	down	-1.0417	-0.05894	1.041703	down	50.36854
down	1.130001	0.176324	1.130001	up	1.160856	0.215189	1.160856	up	2973.037
down	1.095434	0.131502	1.095434	up	1.101874	0.139959	1.101874	up	1565.727
up	-1.08753	-0.12106	1.087531	down	-1.06438	-0.09002	1.064385	down	1289.281
up	-1.19651	-0.25883	1.196506	down	1.061771	0.086473	1.061771	up	5263.114
up	-1.1049	-0.14392	1.1049	down	-1.03542	-0.05022	1.035423	down	58.15938
down	1.140182	0.189264	1.140182	up	1.039053	0.05527	1.039053	up	24.61515
up	-1.09989	-0.13737	1.099895	down	-1.02995	-0.04257	1.029947	down	392.9278
down	-1.06482	-0.09061	1.064821	down	1.014026	0.020095	1.014026	up	1972.149
up	1.150194	0.201877	1.150194	up	1.193795	0.255555	1.193795	up	98.17517
up	1.2407	0.311155	1.2407	up	1.138355	0.18695	1.138355	up	256.2139
up	-1.01585	-0.02268	1.015847	down	1.053466	0.075143	1.053466	up	51.44728
up	-1.07985	-0.11083	1.079847	down	-1.09504	-0.13098	1.095039	down	1208.438
up	1.361638	0.445344	1.361638	up	1.259421	0.332761	1.259421	up	1374.053
up	1.200822	0.264022	1.200822	up	1.223319	0.290801	1.223319	up	64.03462
up	1.167015	0.222823	1.167015	up	1.080177	0.111268	1.080177	up	630.616
up	-1.02835	-0.04033	1.028349	down	1.09145	0.126246	1.09145	up	213.5799
down	-1.00853	-0.01226	1.008534	down	-1.05714	-0.08017	1.057144	down	859.729
up	-1.30755	-0.38687	1.30755	down	-1.00105	-0.00151	1.001045	down	181.822

up	1.229998	0.298656	1.229998	up	1.255022	0.327712	1.255022	up	568.2439
down	1.092074	0.127071	1.092074	up	-1.20632	-0.27061	1.206318	down	4233.515
up	1.076735	0.106663	1.076735	up	-1.02293	-0.0327	1.022927	down	859.8234
up	1.371034	0.455264	1.371034	up	1.212681	0.278201	1.212681	up	189.6584
down	-1.19839	-0.2611	1.198394	down	-1.11988	-0.16335	1.119884	down	77.50143
up	-1.01073	-0.0154	1.010733	down	1.011505	0.016504	1.011505	up	1960.509
up	1.027464	0.039088	1.027464	up	1.186373	0.246558	1.186373	up	1209.848
up	1.10993	0.150469	1.10993	up	1.105685	0.14494	1.105685	up	256.1229
down	1.290974	0.36846	1.290974	up	1.19134	0.252586	1.19134	up	252.195
up	1.050731	0.071394	1.050731	up	1.079959	0.110977	1.079959	up	697.7934
down	-1.31526	-0.39535	1.315259	down	-1.506	-0.59072	1.506003	down	217.4623
up	-1.10564	-0.14488	1.105641	down	-1.11018	-0.15079	1.110176	down	652.2568
up	1.322094	0.402825	1.322094	up	1.148829	0.200164	1.148829	up	1099.522
up	1.066653	0.093091	1.066653	up	1.046336	0.065346	1.046336	up	76.92342
down	1.092971	0.128255	1.092971	up	-1.18423	-0.24396	1.184235	down	1976.813
up	1.032775	0.046526	1.032775	up	-1.0615	-0.0861	1.0615	down	2878.758
up	-1.15478	-0.20761	1.154776	down	-1.35553	-0.43885	1.355525	down	427.9426
down	-1.05101	-0.07177	1.051007	down	1.003116	0.004488	1.003116	up	156.9716
down	-1.16791	-0.22393	1.167909	down	-1.01135	-0.01629	1.011353	down	59.9225
up	1.170879	0.227592	1.170879	up	1.022284	0.031796	1.022284	up	55.75281
up	-1.08491	-0.11757	1.084908	down	1.079434	0.110276	1.079434	up	539.1354
up	-1.09417	-0.12984	1.094173	down	-1.07261	-0.10113	1.072612	down	279.3846
up	1.457384	0.543381	1.457384	up	1.373515	0.457873	1.373515	up	1988.568
down	-1.12195	-0.16601	1.121954	down	-1.05992	-0.08396	1.059923	down	185.1911
up	1.048639	0.068518	1.048639	up	-1.08702	-0.12037	1.087015	down	163.4802
up	1.220743	0.287759	1.220743	up	-1.05424	-0.07621	1.054243	down	463.094
up	-1.01149	-0.01648	1.011491	down	-1.00559	-0.00804	1.005586	down	62.30345
down	-1.30274	-0.38154	1.302735	down	-1.58894	-0.66806	1.588938	down	72.54348
up	1.156608	0.2099	1.156608	up	-1.00089	-0.00128	1.000886	down	141.4972
down	1.168698	0.224902	1.168698	up	1.187711	0.248184	1.187711	up	1403.783
up	-1.04551	-0.06421	1.045511	down	1.034131	0.048418	1.034131	up	826.9331
down	1.031422	0.044635	1.031422	up	-1.03476	-0.0493	1.034762	down	36.33455
down	1.174216	0.231698	1.174216	up	1.080676	0.111934	1.080676	up	255.9161
up	-1.08443	-0.11693	1.084425	down	-1.00725	-0.01042	1.007249	down	186.0223
down	-1.16251	-0.21725	1.162514	down	-1.27359	-0.3489	1.273592	down	393.9243
down	1.355638	0.438972	1.355638	up	-1.13612	-0.18412	1.136123	down	455.1256
down	-1.04822	-0.06794	1.048216	down	1.016545	0.023675	1.016545	up	49.37021
down	-1.09432	-0.13003	1.094318	down	-1.0408	-0.0577	1.040802	down	165.4837
down	-1.04836	-0.06814	1.048364	down	-1.44366	-0.52973	1.443658	down	285.8078
up	1.973206	0.980542	1.973206	up	1.590612	0.669582	1.590612	up	53.6041
down	1.114878	0.156886	1.114878	up	1.038485	0.054481	1.038485	up	24.86276
down	1.07272	0.101274	1.07272	up	-1.05395	-0.0758	1.053945	down	279.1873
up	1.159509	0.213513	1.159509	up	1.02607	0.037129	1.02607	up	703.2271
down	-1.11039	-0.15106	1.110387	down	-1.20017	-0.26324	1.200169	down	353.626
up	-1.09384	-0.1294	1.093837	down	1.000774	0.001116	1.000774	up	41.23088
down	1.226683	0.294763	1.226683	up	1.059854	0.083866	1.059854	up	15.16703
down	1.031889	0.045287	1.031889	up	1.087771	0.121375	1.087771	up	99.02662

up	-1.15471	-0.20754	1.154715	down	1.120829	0.164566	1.120829	up	281.0943
up	-1.13454	-0.18211	1.134545	down	1.075929	0.105582	1.075929	up	564.6181
down	-1.14459	-0.19484	1.144594	down	-1.0894	-0.12354	1.089401	down	244.1352
up	-1.08902	-0.12303	1.08902	down	-1.23839	-0.30847	1.238393	down	143.9004
up	1.110359	0.151026	1.110359	up	1.075896	0.105539	1.075896	up	44.1348
down	-1.39372	-0.47894	1.393718	down	-1.5153	-0.5996	1.515297	down	354.5585
up	-1.01787	-0.02556	1.017873	down	-1.00544	-0.00782	1.005436	down	1428.241
up	-1.12876	-0.17473	1.128757	down	1.006745	0.009698	1.006745	up	67.48917
up	-1.3691	-0.45323	1.369099	down	-1.3322	-0.41381	1.332196	down	126.7122
down	1.254438	0.327041	1.254438	up	-1.37419	-0.45858	1.374193	down	15.03133
down	1.044017	0.062146	1.044017	up	1.092542	0.127688	1.092542	up	80.49455
up	-1.09426	-0.12996	1.094263	down	1.037105	0.052562	1.037105	up	49.39928
up	1.095764	0.131937	1.095764	up	1.115278	0.157403	1.115278	up	43.67386
down	-1.03028	-0.04303	1.030279	down	-1.06895	-0.09619	1.068945	down	19.13668
down	1.037194	0.052686	1.037194	up	1.052062	0.07322	1.052062	up	41.81533
up	1.149614	0.201149	1.149614	up	1.054749	0.0769	1.054749	up	141.8875
up	1.019337	0.027631	1.019337	up	-1.02786	-0.03965	1.027862	down	41.26659
down	1.346316	0.429017	1.346316	up	1.173396	0.23069	1.173396	up	55.5041
up	1.000653	9.41E-04	1.000653	up	-1.05651	-0.07931	1.056513	down	832.033
up	1.125935	0.171123	1.125935	up	1.116197	0.158591	1.116197	up	2868.387
down	1.086949	0.120284	1.086949	up	1.123863	0.168467	1.123863	up	53.51431
up	1.481477	0.567036	1.481477	up	1.279919	0.356053	1.279919	up	377.7947
up	-1.035	-0.04963	1.035	down	1.02916	0.041467	1.02916	up	4718.82
up	-1.09627	-0.1326	1.096267	down	1.048574	0.068428	1.048574	up	632.2778
up	1.139069	0.187854	1.139069	up	1.046661	0.065795	1.046661	up	338.667
down	1.014496	0.020764	1.014496	up	1.002188	0.003154	1.002188	up	194.2961
up	1.104604	0.143529	1.104604	up	1.147669	0.198706	1.147669	up	873.5159
down	-1.00908	-0.01304	1.009081	down	-1.03321	-0.04714	1.033212	down	43.88074
down	1.35408	0.437313	1.35408	up	1.228963	0.297441	1.228963	up	1536.954
down	-1.02906	-0.04133	1.029064	down	-1.23946	-0.30972	1.239464	down	713.5344
down	-1.36528	-0.44919	1.365275	down	-1.33581	-0.41772	1.335814	down	267.1954
up	1.161555	0.216057	1.161555	up	1.182743	0.242137	1.182743	up	112.8692
down	1.098349	0.135336	1.098349	up	1.179686	0.238403	1.179686	up	32.25351
down	1.111839	0.152948	1.111839	up	-1.00628	-0.00903	1.006277	down	175.3553
up	1.185904	0.245988	1.185904	up	1.071801	0.100037	1.071801	up	193.1928
up	1.151493	0.203506	1.151493	up	1.016694	0.023886	1.016694	up	259.0113
down	1.26498	0.339115	1.26498	up	1.174337	0.231846	1.174337	up	381.2381
up	1.190409	0.251457	1.190409	up	1.160987	0.215352	1.160987	up	204.6375
up	-1.13735	-0.18568	1.13735	down	-1.04458	-0.06292	1.044576	down	356.009
up	-1.00438	-0.0063	1.004379	down	-1.05765	-0.08086	1.057647	down	193.1355
up	-1.04824	-0.06797	1.048241	down	-1.05069	-0.07133	1.050686	down	141.8865
down	-1.05921	-0.08299	1.05921	down	1.000326	4.70E-04	1.000326	up	1882.667
down	1.247207	0.318701	1.247207	up	1.260019	0.333446	1.260019	up	202.9789
up	-1.03605	-0.05109	1.03605	down	1.026969	0.038393	1.026969	up	3119.734
up	-1.05709	-0.0801	1.057089	down	1.049857	0.070193	1.049857	up	1160.365
up	-1.08626	-0.11937	1.086264	down	1.016173	0.023147	1.016173	up	1190.967
up	1.048286	0.068032	1.048286	up	1.003049	0.004393	1.003049	up	977.2936

down	-1.12075	-0.16447	1.120754	down	-1.09179	-0.12669	1.09179	down	66.92487
down	-1.00711	-0.01022	1.007111	down	-1.14873	-0.20004	1.148734	down	166.0003
up	1.031117	0.044208	1.031117	up	-1.01166	-0.01672	1.011657	down	590.0476
down	-1.16497	-0.22029	1.164968	down	-1.00099	-0.00142	1.000988	down	367.4845
down	1.004898	0.007048	1.004898	up	-1.05643	-0.0792	1.056432	down	150.7482
down	-1.13524	-0.183	1.135244	down	1.076682	0.106592	1.076682	up	687.661
down	1.256813	0.32977	1.256813	up	1.174251	0.23174	1.174251	up	88.77799
up	-1.04098	-0.05794	1.04098	down	-1.03495	-0.04956	1.034952	down	3986.485
down	1.0776	0.107822	1.0776	up	-1.09333	-0.12872	1.093325	down	540.45
up	1.167143	0.222981	1.167143	up	1.319127	0.399583	1.319127	up	87.12655
down	-1.02029	-0.02898	1.020292	down	-1.0035	-0.00504	1.003502	down	90.75806
up	-1.03148	-0.04471	1.031479	down	-1.04881	-0.06876	1.048811	down	86.70892
up	1.030127	0.042823	1.030127	up	1.009869	0.014168	1.009869	up	309.488
up	-1.10099	-0.1388	1.100991	down	1.012117	0.017376	1.012117	up	206.0061
down	1.123459	0.167947	1.123459	up	-1.13848	-0.1871	1.138476	down	49.12167
down	1.016277	0.023293	1.016277	up	1.088205	0.12195	1.088205	up	2345.71
up	1.576831	0.657028	1.576831	up	1.27001	0.34484	1.27001	up	32.05941
up	-1.14628	-0.19696	1.14628	down	-1.21434	-0.28018	1.214343	down	83.77615
up	1.134441	0.181982	1.134441	up	1.200956	0.264183	1.200956	up	58.44863
down	-1.00062	-8.88E-04	1.000616	down	1.024029	0.034256	1.024029	up	28.34267
up	-1.00647	-0.0093	1.006469	down	1.033636	0.047728	1.033636	up	390.2743
down	-1.04194	-0.05928	1.041943	down	1.136622	0.184752	1.136622	up	51.57071
down	-1.3752	-0.45964	1.375199	down	-1.1261	-0.17134	1.126105	down	48.82709
up	1.34649	0.429204	1.34649	up	1.258947	0.332218	1.258947	up	28.49509
up	1.052357	0.073625	1.052357	up	-1.02378	-0.0339	1.023778	down	290.7628
up	1.001159	0.001671	1.001159	up	1.004291	0.006177	1.004291	up	1639.472
down	1.06314	0.088332	1.06314	up	-1.05124	-0.0721	1.051242	down	386.9888
down	1.02093	0.029884	1.02093	up	-1.10208	-0.14023	1.102084	down	80.31593
down	-1.12609	-0.17133	1.126095	down	-1.14938	-0.20086	1.149381	down	357.2954
down	1.140387	0.189523	1.140387	up	1.213865	0.279608	1.213865	up	310.5309
up	-1.11793	-0.16083	1.117929	down	-1.01326	-0.019	1.013255	down	42.37185
up	-1.11615	-0.15853	1.116153	down	-1.05785	-0.08113	1.057847	down	187.8928
down	1.079908	0.110909	1.079908	up	1.116369	0.158814	1.116369	up	70.85915
down	1.169965	0.226465	1.169965	up	1.104687	0.143637	1.104687	up	1161.407
down	1.206045	0.270283	1.206045	up	1.077357	0.107496	1.077357	up	210.9398
up	-1.11736	-0.1601	1.117364	down	-1.02893	-0.04114	1.028928	down	285.0521
up	1.128168	0.173982	1.128168	up	1.226744	0.294834	1.226744	up	125.9497
up	-1.04933	-0.06947	1.049329	down	1.050721	0.071379	1.050721	up	128.0392
up	1.070119	0.097772	1.070119	up	1.02999	0.042631	1.02999	up	82.89463
down	-1.1692	-0.22552	1.169198	down	-1.14258	-0.1923	1.142582	down	23.87768
down	-1.01435	-0.02055	1.014348	down	-1.13968	-0.18863	1.139679	down	27.34932
up	-1.16912	-0.22542	1.169118	down	-1.09543	-0.1315	1.095433	down	427.2253
up	1.004191	0.006034	1.004191	up	-1.05557	-0.07802	1.055572	down	58.09277
down	1.004288	0.006173	1.004288	up	1.048715	0.068623	1.048715	up	12169.98
down	1.02091	0.029856	1.02091	up	1.005389	0.007754	1.005389	up	185.3295
down	1.109026	0.149294	1.109026	up	1.003619	0.005211	1.003619	up	83.33617
up	1.128615	0.174554	1.128615	up	1.072404	0.100849	1.072404	up	160.868

down	-1.07464	-0.10385	1.074638	down	-1.22217	-0.28944	1.222168	down	521.7676
up	1.200896	0.264111	1.200896	up	1.215543	0.2816	1.215543	up	105.1437
up	-1.03925	-0.05554	1.039245	down	1.008263	0.011871	1.008263	up	64.64502
up	-1.00047	-6.81E-04	1.000472	down	-1.05456	-0.07665	1.054563	down	164.7921
up	1.130168	0.176537	1.130168	up	1.120183	0.163734	1.120183	up	304.6006
up	1.102106	0.140263	1.102106	up	1.19496	0.256962	1.19496	up	674.0967
up	-1.03818	-0.05406	1.038181	down	-1.02922	-0.04155	1.029222	down	5938.959
down	-1.03507	-0.04973	1.035069	down	-1.05823	-0.08165	1.058228	down	358.0365
down	1.05759	0.08078	1.05759	up	-1.04812	-0.06781	1.048123	down	268.8944
down	1.000702	0.001012	1.000702	up	1.041408	0.058535	1.041408	up	456.4422
down	-1.25543	-0.32818	1.255429	down	-1.26772	-0.34224	1.267723	down	283.8982
down	-1.05422	-0.07617	1.054218	down	1.005122	0.007371	1.005122	up	80.908
down	1.124578	0.169384	1.124578	up	1.119244	0.162524	1.119244	up	208.9646
down	-1.10028	-0.13787	1.100277	down	-1.10824	-0.14827	1.108241	down	509.4657
up	-1.0115	-0.01649	1.011496	down	1.03306	0.046924	1.03306	up	134.2431
up	1.118047	0.160981	1.118047	up	1.079045	0.109755	1.079045	up	93.91748
up	-1.1352	-0.18295	1.135203	down	-1.15326	-0.20572	1.153258	down	41.00807
up	-1.08987	-0.12416	1.089873	down	1.071183	0.099205	1.071183	up	374.3011
down	1.101801	0.139864	1.101801	up	-1.02616	-0.03726	1.026162	down	547.2561
down	1.044434	0.062721	1.044434	up	1.06783	0.094682	1.06783	up	22.60836
down	-1.08022	-0.11133	1.080224	down	1.053113	0.07466	1.053113	up	5098.132
down	1.1257	0.170823	1.1257	up	-1.08795	-0.12161	1.087947	down	32.7834
up	1.123946	0.168572	1.123946	up	1.0665	0.092884	1.0665	up	872.8716
down	1.037302	0.052836	1.037302	up	1.002095	0.003019	1.002095	up	10214.09
down	-1.05204	-0.07319	1.052039	down	-1.12936	-0.17551	1.129361	down	294.0535
up	-1.1677	-0.22367	1.167701	down	-1.05279	-0.07422	1.052793	down	327.6878
up	1.045152	0.063712	1.045152	up	1.06152	0.086131	1.06152	up	158.5132
down	1.4701	0.555914	1.4701	up	1.197854	0.260452	1.197854	up	487.3316
down	-1.03831	-0.05424	1.038313	down	1.040975	0.057935	1.040975	up	189.3863
down	1.020008	0.02858	1.020008	up	-1.08272	-0.11465	1.082716	down	1572.071
up	-1.0597	-0.08366	1.059704	down	1.109161	0.149468	1.109161	up	26.15209
up	1.125085	0.170034	1.125085	up	-1.05641	-0.07917	1.056406	down	30.75632
up	-1.05007	-0.07048	1.050069	down	1.006753	0.00971	1.006753	up	3714.364
up	1.282723	0.359209	1.282723	up	1.111542	0.152563	1.111542	up	2041.566
up	1.169444	0.225822	1.169444	up	1.104615	0.143544	1.104615	up	436.3131
up	-1.00089	-0.00128	1.000888	down	-1.00947	-0.01359	1.009467	down	950.522
down	1.157301	0.210764	1.157301	up	-1.00593	-0.00853	1.005928	down	2518.409
down	-1.11277	-0.15416	1.112774	down	-1.22612	-0.29409	1.226115	down	960.2024
down	1.095575	0.131688	1.095575	up	-1.15951	-0.21351	1.159507	down	196.9251
down	-1.00274	-0.00395	1.002743	down	-1.0043	-0.0062	1.004304	down	704.6314
up	1.062763	0.08782	1.062763	up	1.146855	0.197683	1.146855	up	57.06441
down	1.185257	0.2452	1.185257	up	1.109877	0.1504	1.109877	up	22.75139
up	-1.0384	-0.05436	1.038399	down	1.110676	0.151437	1.110676	up	449.4929
down	1.09621	0.132524	1.09621	up	-1.15125	-0.2032	1.151252	down	66.51917
down	-1.13299	-0.18013	1.132987	down	1.024625	0.035096	1.024625	up	108.2174
up	-1.4562	-0.5422	1.456196	down	-1.28932	-0.36661	1.289318	down	48.71068
up	1.00466	0.006707	1.00466	up	1.038249	0.054152	1.038249	up	172.0152

up	-1.08149	-0.11302	1.081489	down	-1.08185	-0.11351	1.081854	down	782.2923
up	1.10214	0.140308	1.10214	up	-1.00027	-3.83E-04	1.000266	down	305.7174
down	1.054941	0.077163	1.054941	up	-1.03439	-0.04878	1.034389	down	48.58709
down	-1.05588	-0.07844	1.055876	down	-1.0766	-0.10648	1.076602	down	53.07474
up	1.307951	0.387308	1.307951	up	1.043371	0.061252	1.043371	up	119.9105
up	1.103979	0.142713	1.103979	up	1.058603	0.082161	1.058603	up	133.3284
down	-1.09789	-0.13473	1.097887	down	-1.02978	-0.04234	1.029781	down	140.4946
up	-1.00058	-8.32E-04	1.000577	down	-1.06954	-0.09699	1.069536	down	301.2132
up	-1.16793	-0.22395	1.16793	down	-1.13064	-0.17714	1.130644	down	132.0961
up	-1.15408	-0.20674	1.154079	down	-1.04344	-0.06135	1.043444	down	49.52704
down	1.289431	0.366734	1.289431	up	1.125982	0.171184	1.125982	up	355.1639
down	1.241049	0.311559	1.241049	up	1.127366	0.172956	1.127366	up	1245.048
up	-1.01286	-0.01843	1.012859	down	-1.38952	-0.47458	1.389515	down	80.3855
up	1.120343	0.16394	1.120343	up	1.047419	0.066838	1.047419	up	112.0121
up	-1.31411	-0.39409	1.314113	down	-1.19253	-0.25402	1.192525	down	108.8991
up	-1.05894	-0.08262	1.058941	down	-1.05946	-0.08333	1.059458	down	1840.654
down	-1.11814	-0.16111	1.118144	down	-1.01113	-0.01596	1.011127	down	165.3395
down	1.000134	1.93E-04	1.000134	up	1.020558	0.029358	1.020558	up	1258.899
down	-1.47975	-0.56536	1.479755	down	-1.41686	-0.5027	1.416865	down	99.96481
up	1.525952	0.609709	1.525952	up	1.458428	0.544414	1.458428	up	400.668
up	1.2542	0.326767	1.2542	up	1.122056	0.166145	1.122056	up	142.357
up	-1.0915	-0.12631	1.091498	down	1.004556	0.006558	1.004556	up	17.54998
up	-1.0637	-0.08909	1.063698	down	1.013788	0.019756	1.013788	up	388.9034
down	-1.05231	-0.07356	1.052312	down	-1.02287	-0.03262	1.022869	down	56.66318
down	1.076329	0.106119	1.076329	up	-1.4478	-0.53387	1.447804	down	407.9828
up	1.092074	0.12707	1.092074	up	1.150205	0.201891	1.150205	up	767.6079
down	-1.11422	-0.15604	1.114224	down	-1.10132	-0.13923	1.101318	down	120.9566
down	-1.17849	-0.23694	1.178492	down	-1.32265	-0.40343	1.322648	down	111.9147
up	-1.14621	-0.19687	1.146213	down	-1.01461	-0.02092	1.014606	down	65.86812
down	1.062164	0.087006	1.062164	up	1.014101	0.020202	1.014101	up	459.4943
up	-1.14293	-0.19274	1.14293	down	-1.09977	-0.1372	1.099767	down	2520.412
up	1.180461	0.23935	1.180461	up	1.199486	0.262417	1.199486	up	21.45731
up	1.053128	0.074681	1.053128	up	1.099719	0.137135	1.099719	up	1283.04
up	-1.0137	-0.01963	1.013703	down	1.030901	0.043906	1.030901	up	941.9753
down	-1.03361	-0.04769	1.033608	down	-1.04251	-0.06006	1.04251	down	812.2209
up	-1.10874	-0.14893	1.108743	down	-1.04718	-0.06651	1.047179	down	569.5651
up	-1.43233	-0.51836	1.43233	down	-1.25677	-0.32972	1.256771	down	28.38785
down	1.080806	0.112107	1.080806	up	1.099698	0.137108	1.099698	up	783.6106
up	1.228305	0.296669	1.228305	up	1.134973	0.182658	1.134973	up	524.0078
down	-1.01647	-0.02356	1.016467	down	1.063477	0.088788	1.063477	up	696.669
up	-1.01784	-0.02551	1.017841	down	-1.11217	-0.15338	1.112173	down	198.5641
down	1.238846	0.308997	1.238846	up	1.012273	0.017599	1.012273	up	1648.765
up	-1.00213	-0.00307	1.00213	down	1.014725	0.021089	1.014725	up	56.23572
down	1.01398	0.020029	1.01398	up	1.024644	0.035123	1.024644	up	37.19825
down	1.089473	0.123631	1.089473	up	1.066274	0.092578	1.066274	up	575.6282
down	-1.03735	-0.0529	1.037346	down	1.092483	0.127611	1.092483	up	170.8036
down	-1.11717	-0.15985	1.117168	down	1.056778	0.079673	1.056778	up	155.4579

up	1.113482	0.155078	1.113482	up	1.095663	0.131804	1.095663	up	249.1164
up	1.012561	0.018009	1.012561	up	1.110925	0.151762	1.110925	up	72.86932
up	1.029189	0.041508	1.029189	up	-1.01963	-0.02805	1.019634	down	1076.392
down	-1.04708	-0.06637	1.047079	down	-1.07756	-0.10777	1.077563	down	400.2573
up	-1.01049	-0.01505	1.010489	down	-1.00718	-0.01032	1.007181	down	316.6069
up	1.081052	0.112436	1.081052	up	-1.02014	-0.02876	1.020137	down	508.8029
down	-1.0729	-0.10151	1.072899	down	1.050081	0.0705	1.050081	up	269.2974
up	-1.06297	-0.08811	1.062975	down	1.031482	0.044718	1.031482	up	38.47799
down	-1.02376	-0.03388	1.023762	down	-1.0038	-0.00547	1.003799	down	139.5898
down	1.04177	0.059037	1.04177	up	-1.18887	-0.24959	1.188866	down	370.5466
down	-1.18952	-0.25038	1.189521	down	-1.18008	-0.23888	1.180076	down	138.0652
up	-1.07132	-0.0994	1.071325	down	-1.15834	-0.21205	1.158335	down	283.8189
down	-1.17275	-0.22989	1.172745	down	-1.04119	-0.05823	1.041188	down	38.29248
down	1.166392	0.222052	1.166392	up	1.210432	0.275522	1.210432	up	195.1506
down	1.001022	0.001473	1.001022	up	1.019356	0.027658	1.019356	up	46.36647
up	-1.06954	-0.097	1.069544	down	-1.0254	-0.03618	1.025396	down	443.5115
up	1.074374	0.103496	1.074374	up	-1.10521	-0.14432	1.105206	down	38.83992
down	-1.02897	-0.0412	1.028966	down	-1.27233	-0.34747	1.272331	down	371.4842
down	1.042786	0.060443	1.042786	up	-1.14764	-0.19867	1.14764	down	18.60085
up	-1.50683	-0.59152	1.506832	down	-1.34537	-0.428	1.345366	down	889.319
up	-1.02331	-0.03325	1.023311	down	-1.2065	-0.27082	1.206497	down	100.963
up	-1.37921	-0.46384	1.379208	down	-1.07372	-0.10262	1.073722	down	50.06434
up	1.048188	0.067898	1.048188	up	-1.088	-0.12167	1.087997	down	276.8677
up	1.082934	0.114945	1.082934	up	1.093684	0.129196	1.093684	up	20.94887
down	-1.13839	-0.18699	1.138387	down	1.030088	0.042768	1.030088	up	55.0663
up	-1.00447	-0.00644	1.004475	down	1.110552	0.151278	1.110552	up	48.52913
down	-1.08103	-0.11241	1.081029	down	-1.03489	-0.04947	1.034888	down	32.52582
down	-1.05104	-0.07182	1.051038	down	-1.03752	-0.05313	1.037517	down	31.51534
up	1.044734	0.063136	1.044734	up	-1.04224	-0.05969	1.042243	down	219.3245
up	1.098197	0.135137	1.098197	up	1.073144	0.101843	1.073144	up	228.3895
down	1.350151	0.43312	1.350151	up	1.368685	0.45279	1.368685	up	33.90033
down	1.145489	0.195963	1.145489	up	1.151351	0.203328	1.151351	up	229.7591
down	1.000004	5.25E-06	1.000004	up	1.021395	0.030541	1.021395	up	159.6705
up	-1.0084	-0.01206	1.008395	down	1.040942	0.05789	1.040942	up	58.84943
up	-1.05246	-0.07377	1.05246	down	-1.11072	-0.15149	1.11072	down	359.726
up	-1.11737	-0.16011	1.117371	down	1.048115	0.067796	1.048115	up	175.9036
up	-1.32409	-0.405	1.32409	down	-1.27637	-0.35205	1.27637	down	298.1863
down	1.121476	0.165398	1.121476	up	1.041166	0.0582	1.041166	up	536.7015
up	-1.30667	-0.3859	1.306671	down	-1.31559	-0.39571	1.315586	down	104.881
up	1.116303	0.158729	1.116303	up	-1.00669	-0.00962	1.006689	down	345.8871
up	-1.07233	-0.10075	1.072332	down	-1.04497	-0.06346	1.044966	down	750.471
up	1.004055	0.005838	1.004055	up	1.031025	0.04408	1.031025	up	276.9576
up	-1.27237	-0.34752	1.272373	down	-1.1452	-0.1956	1.1452	down	128.327
down	-1.09505	-0.13099	1.095047	down	1.042822	0.060493	1.042822	up	79.5859
up	1.262222	0.335966	1.262222	up	1.301867	0.380582	1.301867	up	99.75948
up	1.061351	0.085901	1.061351	up	-1.04355	-0.0615	1.043554	down	50.37427
down	-1.13008	-0.17642	1.130078	down	1.10541	0.144582	1.10541	up	23.27156

up	-1.27674	-0.35247	1.276744	down	-1.20852	-0.27325	1.208525	down	257.9551
up	-1.3332	-0.41489	1.3332	down	-1.3046	-0.38361	1.304599	down	17.55845
down	-1.03184	-0.04522	1.031843	down	1.018688	0.026713	1.018688	up	107.4751
down	-1.48161	-0.56716	1.481607	down	-1.15997	-0.21408	1.159966	down	19.21606
down	-1.02191	-0.03127	1.021913	down	1.011879	0.017037	1.011879	up	247.6434
up	1.346859	0.429599	1.346859	up	1.14833	0.199538	1.14833	up	33.3701
down	1.193152	0.254778	1.193152	up	1.136299	0.184343	1.136299	up	297.3548
down	1.731015	0.791619	1.731015	up	1.361305	0.44499	1.361305	up	40.78437
up	1.055275	0.077619	1.055275	up	1.110703	0.151473	1.110703	up	135.5865
up	-1.00007	-9.93E-05	1.000069	down	1.12451	0.169296	1.12451	up	179.1692
down	1.068731	0.095898	1.068731	up	-1.00314	-0.00452	1.003136	down	374.9557
up	1.068924	0.096159	1.068924	up	-1.08876	-0.12269	1.08876	down	44.20447
down	-1.19722	-0.25969	1.197219	down	1.101173	0.139042	1.101173	up	42.29593
up	-1.12683	-0.17226	1.126826	down	-1.08773	-0.12132	1.087727	down	49.31018
up	-1.29073	-0.36819	1.290728	down	-1.15276	-0.20509	1.15276	down	97.111
down	1.175296	0.233024	1.175296	up	1.122216	0.16635	1.122216	up	23.65927
up	-1.09083	-0.12543	1.09083	down	-1.15434	-0.20707	1.154339	down	42.049
up	1.170753	0.227436	1.170753	up	1.097021	0.133591	1.097021	up	53.35535
up	-1.00301	-0.00433	1.003009	down	1.092171	0.127198	1.092171	up	194.265
up	-1.13591	-0.18385	1.135908	down	-1.24382	-0.31477	1.243815	down	119.8708
down	1.089815	0.124083	1.089815	up	1.036977	0.052385	1.036977	up	32.25834
up	1.265233	0.339403	1.265233	up	1.331611	0.413172	1.331611	up	33.14014
down	-1.00634	-0.00912	1.006344	down	1.00485	0.00698	1.00485	up	128.6187
down	-1.02682	-0.03819	1.026822	down	1.129446	0.175615	1.129446	up	45.46496
up	1.007286	0.010473	1.007286	up	1.065239	0.091177	1.065239	up	227.6625
up	-1.08768	-0.12125	1.087675	down	-1.05318	-0.07475	1.053181	down	130.3671
down	1.3358	0.417704	1.3358	up	1.016767	0.023989	1.016767	up	26.02754
up	1.036868	0.052232	1.036868	up	-1.07624	-0.10599	1.076236	down	41.98739
up	-1.19209	-0.25349	1.192086	down	-1.17747	-0.23569	1.177468	down	17.79749
down	1.024557	0.035	1.024557	up	1.024139	0.034411	1.024139	up	126.6307
down	1.028359	0.040344	1.028359	up	-1.01634	-0.02338	1.016337	down	21.50551
down	-1.09178	-0.12668	1.091776	down	-1.14727	-0.1982	1.147269	down	20.07294
up	-1.01305	-0.01871	1.013055	down	1.127071	0.172579	1.127071	up	464.4911
down	1.241337	0.311895	1.241337	up	1.039013	0.055214	1.039013	up	29.72578
up	1.228055	0.296375	1.228055	up	-1.12978	-0.17604	1.129777	down	31.99305
down	-1.09862	-0.13569	1.098617	down	1.005688	0.008183	1.005688	up	254.1956
down	-1.0554	-0.07779	1.055399	down	-1.01711	-0.02448	1.017115	down	132.7015
up	-1.3351	-0.41695	1.335101	down	-1.1259	-0.17107	1.125897	down	129.4631
down	1.16079	0.215107	1.16079	up	1.082793	0.114758	1.082793	up	30.57228
down	1.649329	0.721879	1.649329	up	1.55789	0.639594	1.55789	up	2488.843
down	1.399177	0.484578	1.399177	up	1.513462	0.597852	1.513462	up	1768.289
down	1.071613	0.099784	1.071613	up	1.011837	0.016977	1.011837	up	457.49
down	-1.03373	-0.04786	1.033727	down	1.048242	0.067972	1.048242	up	1015.336
up	-1.19233	-0.25378	1.192326	down	-1.1189	-0.16208	1.118896	down	90.05883
down	1.046035	0.064931	1.046035	up	1.146687	0.197471	1.146687	up	28.38289
up	-1.05874	-0.08235	1.058742	down	1.02553	0.03637	1.02553	up	134.4868
up	-1.30296	-0.38179	1.302957	down	-1.26433	-0.33838	1.264335	down	31.59609

down	-1.34175	-0.42411	1.341747	down	-1.09579	-0.13197	1.095792	down	47.46273
up	1.017091	0.024448	1.017091	up	1.066895	0.093419	1.066895	up	109.4242
down	-1.12819	-0.17401	1.128189	down	1.03137	0.044562	1.03137	up	92.35952
up	1.271839	0.346917	1.271839	up	1.180828	0.239799	1.180828	up	1781.009
up	1.006745	0.009698	1.006745	up	1.011624	0.016673	1.011624	up	104.849
up	1.042914	0.06062	1.042914	up	-1.048	-0.06764	1.048005	down	54.41101
up	-1.06822	-0.09521	1.068219	down	-1.26084	-0.33438	1.260836	down	16.22008
down	-1.15203	-0.20417	1.152027	down	-1.13244	-0.17944	1.132443	down	42.52525
down	1.035624	0.050501	1.035624	up	-1.16512	-0.22048	1.16512	down	111.1903
down	-1.02726	-0.0388	1.027259	down	-1.23559	-0.3052	1.235589	down	25.48068
down	1.0016	0.002306	1.0016	up	-1.04178	-0.05905	1.041777	down	32.99616
down	1.018025	0.025773	1.018025	up	1.111921	0.153054	1.111921	up	27.86633
down	1.039326	0.055648	1.039326	up	-1.20387	-0.26768	1.203867	down	36.53431
up	-1.24213	-0.31281	1.242127	down	-1.26106	-0.33464	1.261064	down	16.14294
down	1.106903	0.146528	1.106903	up	1.075652	0.105211	1.075652	up	22.70597
down	-1.05743	-0.08056	1.057432	down	1.01677	0.023993	1.01677	up	33.35917
down	-1.30147	-0.38015	1.301475	down	-1.22685	-0.29495	1.226846	down	144.9843
up	-1.07658	-0.10646	1.076585	down	-1.0608	-0.08515	1.060799	down	609.5981
down	1.156334	0.209558	1.156334	up	1.051536	0.072498	1.051536	up	85.55007
up	1.160171	0.214338	1.160171	up	1.02948	0.041915	1.02948	up	502.0288
up	1.132377	0.179354	1.132377	up	1.060114	0.084219	1.060114	up	41.24048
up	-1.03965	-0.05609	1.039646	down	-1.04489	-0.06336	1.044894	down	1892.072
down	-1.02718	-0.03869	1.027184	down	-1.00034	-4.96E-04	1.000344	down	544.6856
up	1.171453	0.228299	1.171453	up	1.197117	0.259565	1.197117	up	336.842
up	1.000474	6.84E-04	1.000474	up	-1.1344	-0.18193	1.134399	down	16.19888
up	1.41199	0.49773	1.41199	up	1.211577	0.276886	1.211577	up	190.0934
up	-1.25045	-0.32244	1.250446	down	-1.09412	-0.12977	1.094121	down	22.71322
up	-1.16797	-0.224	1.167966	down	-1.04243	-0.05995	1.042433	down	157.6278
up	-1.08305	-0.11509	1.083046	down	-1.03449	-0.04893	1.034495	down	16.35503
up	-1.23161	-0.30054	1.231605	down	-1.12525	-0.17025	1.125255	down	76.98753
up	-1.15051	-0.20227	1.15051	down	-1.09988	-0.13735	1.099881	down	19.36971
down	1.025128	0.035804	1.025128	up	1.111803	0.152901	1.111803	up	22.07111
up	-1.11261	-0.15395	1.112609	down	-1.06646	-0.09283	1.066457	down	22.75812
down	1.185814	0.245877	1.185814	up	1.009844	0.014132	1.009844	up	23.617
up	-1.04349	-0.06142	1.043493	down	-1.202	-0.26544	1.202	down	35.52529
down	-1.13355	-0.18084	1.133547	down	-1.11848	-0.16154	1.118479	down	31.5927
up	-1.00218	-0.00314	1.002179	down	-1.03519	-0.0499	1.035192	down	643.2064
up	-1.06961	-0.09708	1.069608	down	-1.0978	-0.13462	1.097803	down	103.408
up	-1.08088	-0.11221	1.080884	down	-1.07879	-0.10942	1.078792	down	191.4223
up	1.061172	0.085658	1.061172	up	-1.01158	-0.01661	1.011577	down	254.452
up	-1.019	-0.02715	1.018997	down	-1.05207	-0.07323	1.05207	down	362.1983
down	-1.13654	-0.18465	1.136543	down	-1.07351	-0.10233	1.073507	down	446.6021
up	1.220506	0.287479	1.220506	up	1.155494	0.208509	1.155494	up	165.293
up	1.025352	0.03612	1.025352	up	-1.06993	-0.09752	1.069935	down	229.1301
down	1.134389	0.181915	1.134389	up	1.236022	0.305704	1.236022	up	118.5087
down	-1.02098	-0.02995	1.020977	down	-1.10113	-0.13899	1.101132	down	191.9665
down	1.147311	0.198257	1.147311	up	1.111842	0.152952	1.111842	up	122.2997

up	1.0277	0.03942	1.0277	up	1.122846	0.16716	1.122846	up	1186.225
up	1.056665	0.079518	1.056665	up	1.053728	0.075503	1.053728	up	235.9023
up	-1.73761	-0.7971	1.737606	down	-1.45373	-0.53976	1.45373	down	13.7676
down	1.168904	0.225156	1.168904	up	1.154777	0.207614	1.154777	up	61.5473
up	1.188119	0.248679	1.188119	up	-1.01208	-0.01732	1.012076	down	32.35303
down	1.157378	0.21086	1.157378	up	1.067051	0.093629	1.067051	up	83.65557
down	1.254091	0.326642	1.254091	up	1.188065	0.248613	1.188065	up	91.98676
down	-1.19118	-0.25239	1.191179	down	-1.32002	-0.40056	1.320018	down	189.4129
down	1.018343	0.026224	1.018343	up	-1.00329	-0.00473	1.003286	down	338.4432
down	-1.08477	-0.11739	1.084774	down	-1.0047	-0.00676	1.0047	down	24.49687
down	-1.24357	-0.31449	1.24357	down	-1.30112	-0.37976	1.301124	down	134.0288
up	1.091545	0.126371	1.091545	up	-1.06818	-0.09516	1.068183	down	24.21968
up	-1.2024	-0.26592	1.202405	down	-1.14378	-0.19381	1.143782	down	85.37199
up	-1.11863	-0.16173	1.118631	down	1.094397	0.130136	1.094397	up	152.234
down	-1.04355	-0.0615	1.043549	down	1.082831	0.114808	1.082831	up	86.89791
up	-1.09165	-0.12651	1.091649	down	-1.01549	-0.02218	1.01549	down	188.522
down	-1.04358	-0.06154	1.043579	down	1.081176	0.112602	1.081176	up	28.78334
up	-1.11353	-0.15515	1.113535	down	-1.13794	-0.18643	1.137944	down	532.7609
down	-1.11132	-0.15227	1.11132	down	-1.0934	-0.12882	1.093396	down	22.71561
down	-1.14027	-0.18938	1.140272	down	-1.15389	-0.2065	1.153885	down	483.9817
up	-1.09547	-0.13155	1.095471	down	-1.00675	-0.00971	1.006751	down	21.99899

[D07-DEX](	[D07-TAA](	[D30-Con](	[D30-DEX](	[D30-TAA](	[D07-Con](	[D07-DEX](	[D07-TAA](	[D30-Con](	[D30-DEX](
2859.596	3075.528	2999.391	3272.827	3392.664	-0.1192	-0.09746	0.009485	-0.02687	0.096227
614.575	602.7229	436.4609	391.0393	430.1369	0.388609	0.147245	0.133559	-0.35746	-0.51649
26.20618	25.94585	21.89092	21.31137	23.54136	0.038448	0.143243	0.125967	-0.12088	-0.15575
1028.385	1185.066	759.1442	729.97	799.3911	-0.01011	0.198485	0.405376	-0.25381	-0.30159
89.9768	84.31054	71.62395	83.25401	75.00314	-0.0279	0.187091	0.093362	-0.14084	0.071016
312.78	314.2325	215.0126	266.9626	219.2294	0.002208	0.234678	0.238581	-0.33693	5.08E-04
1486.302	1282.158	1521.033	1480.674	1475.725	-0.10283	0.027414	-0.18815	0.06064	0.020534
124.1148	120.3275	101.7806	106.589	93.81995	0.166876	0.118493	0.076353	-0.18095	-0.09929
386.576	400.1713	835.3039	434.3307	510.2143	-0.17597	-0.12817	-0.07391	0.961905	0.036054
368.6522	401.4504	332.9652	320.2955	328.2408	0.001064	0.111603	0.235966	-0.03397	-0.08953
5274.445	6003.127	4228.539	4420.594	3888.109	0.097652	0.194778	0.383952	-0.1194	-0.05562
70.44769	77.54549	64.99497	58.7282	61.70631	0.133181	0.062133	0.19066	-0.05355	-0.19986
236.8358	239.527	171.3068	218.0072	185.946	0.087379	0.080556	0.099279	-0.39544	-0.0386
513.0443	595.5978	346.9877	340.8624	367.8252	0.233217	0.191719	0.406919	-0.37529	-0.41114
48.98711	59.78547	41.56685	41.58479	39.91618	0.164567	0.123793	0.413574	-0.11168	-0.11788
2815.275	2677.312	3694.569	4122.43	4252.376	-0.17902	-0.24353	-0.3243	0.13424	0.310564
1560.071	1514.555	1294.413	1416.676	1425.416	0.089066	0.083311	0.040209	-0.18679	-0.05529
1473.832	1717.598	1235.3	1140.593	1160.582	-0.01455	0.174529	0.391544	-0.07964	-0.2007
5374.273	5360.663	3593.374	3015.404	3831.693	0.260761	0.299295	0.292646	-0.28571	-0.54453
69.93084	60.61929	42.46956	38.32498	40.68721	0.163899	0.39575	0.230284	-0.30291	-0.44683
23.12992	24.37966	20.79853	23.72509	21.62123	0.088662	-2.20E-04	0.077406	-0.1515	0.03776
358.9058	403.3901	448.5873	408.2067	435.1004	-0.0628	-0.19393	-0.02576	0.125489	-0.01188
1841.899	1954.731	1655.267	1559.28	1679.586	0.173325	0.075668	0.16466	-0.07508	-0.16569
90.5044	122.2505	41.58098	46.75821	50.65198	0.342401	0.241231	0.647594	-0.91392	-0.71205
311.0666	279.0681	155.6952	189.1763	172.3843	0.197568	0.474256	0.320215	-0.5488	-0.23764
54.78024	57.00463	61.65402	60.61317	64.91377	-0.17668	-0.08392	-0.02668	0.084793	0.062109
1192.506	1265.014	1646.655	1525.112	1508.59	-0.15817	-0.17695	-0.09057	0.287432	0.176606
1549.119	1877.25	810.9565	1071.147	994.3539	0.250717	0.432901	0.682461	-0.54467	-0.09933
61.99804	64.17873	50.60786	60.53965	61.64171	0.058243	0.011546	0.060772	-0.28679	-0.02277
675.7781	701.7582	516.1765	597.238	553.046	0.038821	0.138267	0.192989	-0.26313	-0.04031
228.9577	241.8674	271.7793	265.7643	296.4121	-0.23771	-0.13372	-0.05255	0.115954	0.075623
850.0234	751.1546	1089.216	1078.243	1026.481	-0.10188	-0.11291	-0.29137	0.235617	0.223357
170.9813	192.5283	86.77016	66.29911	84.19886	0.443945	0.360442	0.541079	-0.66907	-1.05594

661.4872	592.3865	323.1535	392.8744	398.2261	0.254901	0.476834	0.294556	-0.58684	-0.28818
4751.456	2336.824	4272.665	4708.485	3539.084	0.028578	0.203697	-0.81475	0.035471	0.162542
903.7491	1030.912	705.3524	757.2076	689.358	0.102337	0.16954	0.344697	-0.18727	-0.08061
211.569	237.452	155.4739	210.9437	187.0837	-0.09118	0.067446	0.238288	-0.39002	0.065245
59.42557	64.50854	37.11855	30.54248	32.86828	0.805808	0.451928	0.520014	-0.23973	-0.50083
2133.534	2088.129	2448.634	2419.192	2469.265	-0.17613	-0.05417	-0.08619	0.140864	0.125462
1256.752	1368.924	1315.612	1352.143	1559.707	-0.13799	-0.08449	0.039851	-0.01972	0.019372
230.1853	339.9388	178.817	195.2852	193.8252	0.272287	0.128147	0.660052	-0.26144	-0.11098
227.0655	243.0468	274.2816	349.7174	323.1021	-0.06631	-0.22349	-0.12676	0.035301	0.403761
691.959	705.7476	501.6021	521.7559	535.9091	0.203734	0.189333	0.218781	-0.28988	-0.21849
226.0027	128.7089	295.4699	229.8143	198.5588	-0.02865	0.019889	-0.78112	0.429323	0.033976
613.9896	688.8581	664.962	601.4282	598.7633	0.055521	-0.02997	0.135348	0.08464	-0.06024
1228.886	2178.279	772.9816	1009.357	873.8191	0.066705	0.230741	0.939514	-0.4613	-0.05848
83.02538	97.08427	94.11201	100.3206	98.53421	-0.2857	-0.17443	0.051869	0.006015	0.099106
1494.821	1516.335	1369.701	1499.356	1155.719	0.420241	0.02839	0.042036	-0.10194	0.026317
2801.274	2905.84	2549.323	2630.751	2399.343	0.045676	0.005567	0.05854	-0.13276	-0.08623
415.2368	483.6248	320.1946	277.0023	238.8585	0.277332	0.229303	0.408747	-0.14431	-0.35192
139.3306	130.5296	113.2921	107.9689	113.841	0.373495	0.204741	0.108205	-0.09104	-0.16281
55.3304	58.58614	50.63102	43.29033	50.34723	0.137787	0.022615	0.103462	-0.10733	-0.33125
53.78832	61.80632	90.0008	109.2846	91.93211	-0.51238	-0.54807	-0.33854	0.207182	0.434773
522.5707	569.1106	739.5037	682.7647	797.0376	-0.14278	-0.19037	-0.06706	0.305663	0.18809
287.349	297.143	405.4097	371.2336	379.704	-0.2166	-0.18491	-0.1283	0.319306	0.189465
2676.874	2187.299	861.2708	1217.041	1147.836	0.357022	0.777386	0.475476	-0.89379	-0.35041
192.2605	174.1485	237.4824	210.9976	224.3256	-0.1253	-0.06695	-0.20836	0.234995	0.068981
189.1753	180.8366	154.6342	163.0615	142.0894	0.002708	0.209596	0.148224	-0.07903	-0.01051
420.3439	464.4034	339.7734	411.5694	319.5732	0.158942	0.024618	0.164284	-0.29635	-0.00859
64.87732	85.07199	123.5884	126.2796	123.2693	-0.58921	-0.54647	-0.1421	0.391981	0.375498
66.75407	43.37175	76.4092	58.71986	48.13363	0.164201	0.050285	-0.57088	0.239935	-0.14161
176.9098	200.3554	135.2878	156.0024	134.586	-0.13857	0.193489	0.358188	-0.2027	0.0072
1505.99	1347.292	937.8074	1096.357	1117.999	0.202162	0.300333	0.131706	-0.37966	-0.15475
864.0566	1049.724	712.7071	683.5342	735.4829	0.070468	0.135049	0.413745	-0.14655	-0.21075
36.47232	35.63047	30.8649	31.68812	29.7545	0.07005	0.075912	0.042958	-0.17072	-0.12609
305.988	239.1457	132.069	154.5836	141.9533	0.43064	0.709043	0.353593	-0.50504	-0.27334
177.4539	204.1825	215.953	199.5331	214.6389	-0.14016	-0.20327	-7.72E-04	0.080178	-0.03675
401.3437	310.34	504.8877	430.9928	392.9975	-0.01514	0.018686	-0.35035	0.339824	0.122576
437.9681	406.9132	272.9236	360.1603	234.5277	0.255414	0.193848	0.092518	-0.53363	-0.09466
38.15626	45.52661	44.60457	42.63582	45.39927	0.144664	-0.22721	0.028581	-5.40E-04	-0.06848
99.69553	90.09943	87.08718	79.99195	84.45586	0.847168	0.097222	-0.012	-0.07299	-0.20302
302.1034	158.2084	268.7715	251.5938	187.3624	0.17391	0.248113	-0.67627	0.060554	-0.00759
69.93444	307.2491	35.42575	75.40517	54.38148	-0.21841	0.090812	2.062346	-0.81941	0.161132
27.26502	20.86982	23.39307	26.01633	24.19992	0.022189	0.154425	-0.23004	-0.07119	0.085692
196.0427	208.056	165.6262	178.0106	157.0345	0.545173	0.056359	0.13317	-0.19289	-0.09162
906.4453	1149.338	420.9677	486.1644	441.7392	0.189495	0.555999	0.809734	-0.55944	-0.34593
371.9162	340.3796	303.1434	270.2611	249.2069	0.106628	0.185755	0.056122	-0.13024	-0.2813
41.86191	41.99077	36.67331	33.56285	36.68575	0.107953	0.130977	0.135456	-0.05762	-0.18701
12.75405	12.76377	39.2201	47.29601	40.52587	-0.49272	-0.7349	-0.75429	0.831724	1.126487
87.98801	83.95109	74.46883	76.95947	81.02828	0.257778	0.088629	0.016881	-0.15214	-0.10685

258.9049	328.2459	210.195	181.8043	237.8532	0.15691	0.054281	0.387742	-0.24932	-0.45686
540.8054	632.5998	428.6805	382.4287	460.9956	0.18831	0.129172	0.346721	-0.20722	-0.38933
266.7094	238.9932	155.2055	136.6163	142.78	0.342436	0.469651	0.306346	-0.31349	-0.50832
186.3931	178.4321	139.9473	127.1392	111.6414	0.049112	0.430269	0.350674	-7.67E-04	-0.1238
45.33089	47.00493	38.35488	42.54805	41.29981	0.024314	0.061784	0.113234	-0.17976	-0.02873
286.8054	184.0816	211.5347	148.3528	139.017	0.887058	0.627163	-0.02427	0.16214	-0.3168
1454.602	1478.374	1811.892	1780.863	1812.502	-0.16076	-0.13868	-0.11231	0.181828	0.15627
71.18911	69.99146	64.5967	57.13971	65.04856	0.002312	0.077956	0.053975	-0.06322	-0.23796
106.281	133.8588	106.704	77.84264	80.19752	0.267345	0.022529	0.316895	0.023614	-0.42961
15.92566	14.00089	17.12687	21.38352	12.37873	-0.05051	0.034841	-0.15198	0.12762	0.454661
89.09295	78.26696	74.61386	77.86429	81.59329	0.033784	0.179008	-0.00634	-0.07623	-0.01408
48.10506	52.39322	38.28147	35.12312	39.53478	0.106435	0.069332	0.192211	-0.275	-0.40496
48.82327	49.39727	49.6351	54.3954	55.49392	-0.20287	-0.04141	-0.02457	-0.01715	0.114787
15.41075	16.40724	15.62992	15.2197	14.62717	0.260662	-0.05339	0.039704	-0.03022	-0.07326
45.75109	41.68998	34.89807	36.06163	36.57787	0.101249	0.227575	0.095167	-0.16536	-0.11268
147.7012	146.2648	92.60712	106.2278	97.43121	0.331999	0.400961	0.38747	-0.26837	-0.06722
42.97685	44.78259	34.73589	35.65155	33.91036	0.070825	0.131185	0.188991	-0.17706	-0.14943
59.12629	52.6514	40.12722	54.01702	47.22939	0.056138	0.14954	-0.02722	-0.40917	0.019845
732.2944	855.1359	1216.279	1227.516	1162.428	-0.25548	-0.43395	-0.21604	0.293888	0.29483
3220.542	2891.578	2385.337	2682.806	2660.664	0.077282	0.245087	0.08516	-0.18857	-0.01744
47.77181	46.30237	39.73605	43.15018	44.78235	0.24569	0.082704	0.034747	-0.18377	-0.06348
410.5365	450.0295	560.8821	820.6605	705.5991	-0.39185	-0.29734	-0.13999	0.150807	0.717844
5132.692	5186.521	3636.701	3524.791	3753.992	0.09819	0.213639	0.232232	-0.28046	-0.33009
669.7321	701.1917	788.7311	717.4898	826.668	-0.18358	-0.09692	-0.03283	0.135379	0.00278
361.7177	364.505	372.9099	425.139	389.6785	-0.1596	-0.06481	-0.05255	-0.02182	0.166034
200.6845	189.6161	165.5522	167.8316	165.8375	0.124804	0.173981	0.09141	-0.10542	-0.08465
918.6597	976.9707	960.9979	1060.115	1101.672	-0.17331	-0.10147	-0.01551	-0.03773	0.1058
45.49054	41.58743	37.76367	37.42	36.62012	0.126196	0.178815	0.049805	-0.08946	-0.10251
2268.415	1564.343	996.114	1302.944	1192.211	0.170714	0.715078	0.157322	-0.50687	-0.06956
745.1348	472.7214	401.4846	381.8701	329.6052	0.722345	0.770198	0.111215	-0.14242	-0.18375
259.9159	217.9877	221.0936	161.864	164.7097	0.335464	0.295606	0.052968	0.069099	-0.38009
107.1777	137.7444	79.09457	92.75195	93.61481	0.097069	0.020841	0.374491	-0.4206	-0.20454
25.19157	26.73223	25.13944	27.56134	29.58366	0.229958	-0.12699	-0.03909	-0.13236	0.002973
176.7515	166.4025	124.684	137.1409	122.744	0.213441	0.228444	0.134205	-0.28945	-0.1365
270.6182	208.6415	147.0694	172.0047	156.3993	0.06274	0.561818	0.191368	-0.33458	-0.08859
317.0398	297.6438	218.1438	250.2503	221.2197	0.039021	0.331929	0.229515	-0.21494	-0.01143
326.3797	250.5594	176.9702	221.7184	207.2016	0.675891	0.451185	0.046537	-0.44919	-0.11008
224.0759	253.7794	171.3364	202.8623	198.0383	-0.03477	0.101052	0.281615	-0.29258	-0.04112
341.7872	399.31	430.9859	378.4682	412.5351	-0.12895	-0.18318	0.04224	0.150564	-0.03511
213.8692	219.8307	250.8142	249.6965	237.9101	-0.21379	-0.06053	-0.02182	0.168863	0.16256
146.0571	167.0084	167.6472	159.6523	159.3076	-0.13933	-0.09645	0.097352	0.099678	0.031708
1752.729	1441.072	2107.738	2006.366	2116.143	0.0067	-0.09838	-0.37953	0.168796	0.085807
190.5442	168.6069	114.9351	141.2957	142.6367	0.370694	0.277031	0.10651	-0.46968	-0.15098
3665.733	3949.545	3376.239	3259.708	3464.386	-0.12559	0.100379	0.214545	-0.01266	-0.06376
1222.164	1272.607	1392.171	1324.292	1462.573	-0.14286	-0.06842	-0.01061	0.120057	0.03996
1281.891	1238.222	937.3901	859.5092	948.2295	0.127817	0.235298	0.185689	-0.2282	-0.34758
1072.605	1194.454	923.3393	966.2233	927.667	-0.00527	0.127961	0.28647	-0.08783	-0.0198

48.16093	39.37952	34.19608	30.48493	31.2764	0.866051	0.401556	0.091576	-0.0978	-0.26227
115.8489	91.59055	76.22579	76.40051	67.98617	0.875336	0.359408	-0.01401	-0.25719	-0.26741
588.9648	655.5225	687.7813	709.4785	680.1555	-0.14393	-0.14754	0.007327	0.076868	0.121076
380.5731	318.6004	207.1801	175.2882	199.1909	0.258072	0.31039	0.058058	-0.64412	-0.86441
129.9633	139.0271	176.2096	176.7739	166.3228	-0.1124	-0.33326	-0.22896	0.111282	0.11833
672.1829	625.9128	494.4642	435.4903	532.7681	0.238272	0.197993	0.103064	-0.23705	-0.42005
91.40839	87.54696	64.24113	80.52006	75.55252	0.079172	0.119959	0.058915	-0.39217	-0.0624
3975.978	4151.232	3831.836	3686.1	3702.892	0.033052	0.028745	0.091068	-0.02401	-0.08195
477.4607	453.7549	445.0886	480.0937	406.6573	0.248088	0.066561	-0.00423	-0.0358	0.072021
63.62001	109.6176	54.29356	62.96979	71.16805	0.33669	-0.07767	0.707193	-0.31132	-0.08834
77.25839	74.35611	93.75091	91.93707	93.45519	0.073237	-0.15976	-0.21313	0.117801	0.088818
109.9723	145.8284	414.3097	401.8742	388.5541	-1.36621	-1.02649	-0.61895	0.880511	0.835797
320.0652	359.3284	263.2755	270.4596	265.2145	0.071649	0.118539	0.277906	-0.1661	-0.12327
200.4754	216.6003	242.0748	220.4118	245.0457	-0.08387	-0.12202	-0.01118	0.15058	0.011777
52.63714	46.95308	49.35729	55.48539	43.35129	-0.01895	0.085489	-0.0801	-0.00761	0.160341
2220.956	2290.112	1728.126	1763.584	1880.293	0.153777	0.075331	0.11891	-0.29162	-0.26832
35.65073	95.8157	26.47511	43.70039	33.84781	-0.08891	0.065771	1.322666	-0.36979	0.287234
94.85728	96.16714	82.37635	71.75629	67.62415	-0.01774	0.17027	0.186478	-0.03779	-0.23475
75.13294	64.70808	89.15804	102.4048	107.647	-0.54166	-0.17015	-0.38625	0.088555	0.270537
26.07194	24.72567	23.19869	23.17222	23.74916	0.204441	0.084825	0.008346	-0.0849	-0.08579
403.7147	412.5717	436.3047	433.6463	451.0602	-0.11947	-0.06837	-0.0372	0.043964	0.034661
59.37772	49.07992	37.98288	36.89558	43.44264	0.144253	0.355147	0.079468	-0.29121	-0.35049
42.13306	45.96093	55.17442	40.51579	49.1689	0.026703	-0.18605	-0.06252	0.203319	-0.25632
34.65787	31.25956	26.65613	36.20598	33.48797	-0.18233	0.101289	-0.04755	-0.27977	0.149433
293.5923	322.3026	345.2842	364.5463	337.3318	-0.18208	-0.16931	-0.03371	0.066043	0.139667
1539.284	1739.026	1757.748	1760.499	1766.841	-0.06514	-0.15664	0.020749	0.036108	0.037779
353.4062	357.8788	303.9237	323.6696	289.2738	0.199349	0.067076	0.086386	-0.14887	-0.06054
89.95157	76.32134	52.76593	53.00168	46.94267	0.161458	0.310959	0.090412	-0.4832	-0.45332
333.5735	337.3102	375.9739	333.8548	327.0123	0.059319	-0.03944	-0.02392	0.132671	-0.03866
382.9302	294.4319	194.1292	219.6033	234.832	0.242399	0.55827	0.193704	-0.41865	-0.22913
43.12556	47.33477	38.30772	34.32807	37.74175	0.065966	0.092408	0.227078	-0.08097	-0.2418
173.2313	207.0293	232.6172	207.9207	219.2279	-0.11905	-0.23605	0.021735	0.185405	0.026871
66.05924	53.17458	76.83219	83.06432	86.23762	-0.05832	-0.16094	-0.48534	0.06142	0.172329
1253.555	960.7573	651.5534	756.666	714.0217	0.420265	0.556314	0.140183	-0.39895	-0.17248
210.094	191.0231	155.776	186.827	167.3435	0.165531	0.160233	0.024703	-0.27736	-0.00708
287.3868	298.2684	341.5864	305.6471	332.3282	-0.09506	-0.08068	-0.02663	0.169017	0.008918
131.1994	141.2782	138.3989	155.9532	169.6606	-0.19175	-0.12911	-0.02048	-0.05233	0.121654
151.6133	136.9493	117.6379	111.8027	123.2723	0.017636	0.260888	0.115503	-0.10591	-0.17538
80.74707	92.47794	61.82489	65.07882	62.79736	0.176063	0.130294	0.332633	-0.27426	-0.17648
26.94914	22.72779	29.54686	25.2597	25.85043	-0.09683	0.077535	-0.16699	0.21009	-0.01543
24.38113	25.22075	24.08039	23.69813	21.09966	0.168282	0.001349	0.052701	-0.01717	-0.03772
421.6016	482.9196	522.8209	447.809	477.476	-0.12354	-0.14104	0.055678	0.170059	-0.05536
59.80419	70.53547	55.3607	55.67744	52.53836	0.036395	0.078023	0.317476	-0.03123	-0.02519
12378.36	11460.15	11971.59	12026.8	12555.98	-0.00266	0.02208	-0.08915	-0.02623	-0.02006
161.1169	162.6066	120.907	123.7146	120.9064	0.344975	0.137435	0.157226	-0.27893	-0.24908
89.74817	77.05795	96.21664	106.5338	96.40481	-0.17901	-0.07653	-0.29453	0.029001	0.178295
162.6275	164.3738	173.2013	195.7533	185.7628	-0.07811	-0.0623	-0.04554	0.029555	0.204109

517.6549	480.9991	394.6224	370.8215	324.1559	0.218722	0.198987	0.090537	-0.18759	-0.29145
110.1391	113.0396	82.70029	99.37717	100.5978	0.006894	0.071954	0.111387	-0.34427	-0.08016
65.1254	74.62576	55.20919	53.31118	55.61072	0.118128	0.132281	0.329904	-0.10269	-0.15823
142.9963	172.1406	127.5633	127.8618	120.99	0.250387	0.049102	0.316066	-0.1185	-0.11918
318.4127	383.0773	203.4192	233.3252	230.4734	0.105311	0.183444	0.449732	-0.47354	-0.297
795.1826	777.576	764.234	842.8767	912.7571	-0.23462	0.002028	-0.02914	-0.05558	0.084685
5907.986	6520.682	6635.843	6393.202	6444.322	-0.11131	-0.11904	0.021672	0.048078	-0.00598
315.3845	335.3902	505.8977	484.1011	469.4403	-0.0683	-0.26644	-0.16439	0.403109	0.353381
242.9031	264.9869	245.2461	259.3957	234.0161	0.069912	-0.07893	0.048816	-0.06317	0.017609
398.7777	445.4487	397.7536	398.0525	414.2146	0.153735	-0.04253	0.118468	-0.04451	-0.0435
272.9417	259.9881	245.1475	192.8319	189.907	0.17955	0.127206	0.059925	-0.05041	-0.37859
88.51679	79.18969	93.62449	88.76138	94.15287	-0.14193	-0.01261	-0.17364	0.068603	-0.00757
212.0732	198.3309	170.8765	192.0032	191.0008	0.072708	0.094175	-0.00201	-0.21962	-0.05023
497.5283	448.7982	610.3148	555.8435	551.6675	-0.06457	-0.09265	-0.24155	0.202967	0.0651
162.7629	167.1825	180.5698	178.5869	186.1434	-0.33684	-0.05539	-0.01668	0.09041	0.073919
83.612	96.34899	102.5121	114.5991	110.9429	-0.09346	-0.2659	-0.05787	0.033386	0.194367
40.09757	41.66982	44.42482	39.19889	38.51735	0.004705	-0.02791	0.027407	0.119878	-0.06307
402.3807	446.193	547.0026	498.0032	576.691	-0.21224	-0.1059	0.043167	0.316134	0.191974
554.143	512.8164	492.6834	542.5334	480.6481	0.053232	0.071259	-0.0405	-0.09914	0.040722
22.65931	19.89165	17.77317	18.61204	18.90259	0.194713	0.196859	0.009618	-0.15817	-0.09545
5255.51	5073.298	5487.032	5079.807	5780.428	-0.04368	8.37E-04	-0.05027	0.06273	-0.0486
31.03048	31.57336	24.42019	27.25495	22.35793	0.175345	0.096126	0.119588	-0.26326	-0.09244
828.289	961.093	690.3273	776.8244	738.1463	0.118827	0.046805	0.257704	-0.21654	-0.04797
10529.5	9855.773	10783.91	11182.7	10802.17	-0.04459	-3.36E-04	-0.09596	0.033215	0.086051
316.1031	294.3855	192.837	182.3196	170.2262	0.341173	0.420198	0.31993	-0.28075	-0.35394
354.0886	417.6995	525.3867	445.7394	493.7301	-0.28684	-0.16921	0.054985	0.380191	0.156521
186.5381	170.7086	137.7566	143.8582	145.9038	0.039995	0.274745	0.146682	-0.16557	-0.10186
395.2228	381.146	151.7284	209.8335	173.8881	1.060139	0.791166	0.646706	-0.67952	-0.1236
191.8876	164.7848	232.2781	223.9198	242.1525	-0.14792	-0.12873	-0.34737	0.14979	0.095549
1538.317	1459.466	1229.862	1259.788	1133.309	0.189179	0.153278	0.077859	-0.16822	-0.13964
24.42839	26.85925	16.12455	15.08048	17.61851	0.342696	0.243945	0.371059	-0.3675	-0.45116
32.80473	36.55401	27.17468	30.30274	25.63914	0.008324	0.102602	0.258528	-0.18098	-0.01094
3684.77	4027.702	4401.655	4199.51	4437.423	-0.14958	-0.16234	-0.03361	0.094539	0.024055
2492.455	2402.384	1338.395	1749.558	1486.93	0.045041	0.340532	0.284183	-0.55461	-0.1954
416.7502	444.4787	286.038	333.1381	312.6114	0.257216	0.180952	0.288379	-0.36177	-0.13595
990.1711	1030.037	1280.406	1289.767	1268.952	-0.20959	-0.15091	-0.0921	0.217983	0.216702
2388.323	2356.452	1796.953	2074.904	1785.388	0.191134	0.11374	0.092516	-0.30246	-0.09169
908.4549	850.0422	630.9598	572.4523	511.6006	0.340656	0.280697	0.169768	-0.25907	-0.41323
192.4798	185.2593	193.8359	212.4698	167.2152	0.005631	-0.02701	-0.08242	-0.01644	0.115244
588.4762	697.6726	520.7329	521.2011	522.6201	0.27746	0.017924	0.266929	-0.15541	-0.15936
71.80929	63.21703	42.5683	44.91552	48.39392	0.196859	0.541604	0.339352	-0.22313	-0.13531
14.83877	15.84686	12.28147	14.32911	13.39571	0.618979	0.01958	0.110546	-0.27875	-0.03355
408.6203	543.4156	278.4422	268.0562	315.9054	0.191249	0.060436	0.464363	-0.52053	-0.57489
65.52411	59.07643	73.28074	80.60914	63.60325	-0.02249	-0.04283	-0.19692	0.118536	0.25106
98.61822	103.8614	91.35278	80.56406	93.54873	0.163367	0.030982	0.104124	-0.08094	-0.26107
50.83962	52.95429	72.10171	49.02081	55.31801	-0.11716	-0.05435	-0.00381	0.434231	-0.10797
179.7199	185.0487	194.7853	195.6725	202.2169	-0.14437	-0.08119	-0.03651	0.037097	0.043804

828.2558	983.1663	708.277	656.637	654.1279	0.089616	0.172989	0.424562	-0.05391	-0.16693
333.3611	318.3361	273.9617	302.1133	273.7848	0.041699	0.166532	0.094075	-0.11708	0.023233
51.11187	47.42811	37.17847	39.00454	35.83858	0.176848	0.234939	0.141856	-0.21843	-0.14126
44.78488	47.95372	57.74296	54.68996	53.56711	0.031987	-0.21418	-0.11308	0.15155	0.073111
110.7821	140.6023	44.19707	56.83509	43.92012	0.479768	0.392005	0.657925	-0.98675	-0.59944
166.7121	165.3349	135.6789	149.7583	143.4394	-0.14281	0.179689	0.168515	-0.11837	0.02434
129.8511	117.6305	77.14825	69.19354	73.327	0.401949	0.288221	0.144877	-0.5033	-0.63803
397.446	494.8434	255.2255	256.6215	238.514	-0.02252	0.379358	0.672478	-0.27111	-0.27194
139.7499	163.0454	91.42762	79.51706	79.81918	0.150559	0.235445	0.457003	-0.41015	-0.63411
67.35392	61.31505	62.81018	54.49663	60.28811	-0.2892	0.148738	0.019348	0.053047	-0.15369
448.8293	353.0642	227.7918	287.6378	249.4112	0.183105	0.506141	0.169346	-0.49585	-0.12911
1404.76	1189.644	726.9803	885.6643	802.9179	0.417623	0.612786	0.328444	-0.36544	-0.05388
125.4029	513.5582	91.14691	86.4718	62.92863	-0.14771	0.467449	2.495885	-0.01316	-0.03159
105.1324	126.5778	127.364	142.7751	133.6845	-0.14259	-0.23342	0.03121	0.042947	0.206888
135.4755	143.8903	229.96	177.7489	188.317	-0.47325	-0.16656	-0.071	0.573568	0.179479
2018.584	2924.317	1617.19	1493.704	1495.933	0.045931	0.168573	0.703543	-0.17605	-0.25867
158.0778	157.2098	134.7359	120.9026	133.941	0.219552	0.154315	0.140302	-0.07443	-0.23553
1173.567	1189.366	1053.345	1054.328	1077.903	0.141793	0.040675	0.059052	-0.11521	-0.11501
89.93357	69.63291	134.0309	90.66831	94.00443	0.078838	-0.0612	-0.43613	0.499701	-0.06566
563.4569	552.0186	168.3277	245.0592	229.7169	0.403994	0.943648	0.899203	-0.90144	-0.29173
139.6803	142.6145	145.0582	181.1619	162.2627	-0.06657	-0.09355	-0.06219	-0.04419	0.282579
17.93013	20.27408	16.32214	14.95276	16.39843	0.056262	0.087825	0.264022	-0.04804	-0.17435
393.9873	475.6706	360.7787	339.2438	366.5881	0.035414	0.063256	0.334885	-0.0641	-0.15319
60.1418	51.18448	49.74678	47.19682	48.58934	0.137303	0.220689	-0.01342	-0.05272	-0.12629
337.4894	241.5779	181.7105	190.5784	130.78	0.885232	0.580245	0.085452	-0.32861	-0.22249
828.2901	1050.788	786.0015	860.292	903.4914	-0.15414	-0.04511	0.302269	-0.11901	0.008062
115.8543	120.0878	96.85333	87.2806	88.68459	0.139383	0.076591	0.127488	-0.18695	-0.34299
112.5262	98.01461	95.42687	80.55037	71.71571	0.2504	0.256331	0.059772	0.012659	-0.22428
59.27168	70.8246	69.31259	60.58619	68.30631	-0.03055	-0.18304	0.076083	0.044988	-0.15189
451.8897	453.0745	346.7833	367.8375	350.7047	0.208408	0.175279	0.183819	-0.20264	-0.11563
2336.739	2618.316	2382.534	2089.912	2164.792	0.103045	-0.00407	0.158455	0.022197	-0.17054
22.33481	44.05693	18.13428	21.57283	21.77529	-0.02382	0.028616	0.986253	-0.26268	-0.02333
1429.671	1283.237	1153.071	1210.966	1263.459	0.010832	0.167124	0.011054	-0.14837	-0.07369
1011.212	979.1074	1215.829	1200.046	1257.879	-0.2384	-0.13737	-0.18798	0.130817	0.111182
872.2563	760.3991	915.2285	885.5208	878.7025	-0.1091	-0.00491	-0.20489	0.064523	0.016834
598.8027	686.7358	500.682	452.6138	482.1167	0.044722	0.114031	0.311206	-0.14213	-0.29105
31.34056	31.89851	45.60965	31.60305	36.45841	-0.19516	-0.05608	-0.02736	0.476168	-0.0422
687.8754	625.8306	579.5565	626.3067	637.0739	0.305138	0.108954	-0.01812	-0.1301	-0.01799
611.8385	613.9063	565.1709	692.5718	640.2703	-0.24213	-0.0216	-0.01346	-0.13654	0.160126
661.6788	674.6711	481.1268	474.9218	513.4907	0.234882	0.157806	0.191227	-0.30136	-0.32492
212.1808	200.6952	174.5915	171.8355	157.5708	0.092382	0.186696	0.105029	-0.09307	-0.11858
1881.511	1395.563	1348.376	1665.303	1356.219	0.099824	0.289401	-0.14709	-0.19893	0.11007
52.73323	56.6207	48.59682	48.36489	49.16265	0.135031	0.042901	0.146859	-0.07809	-0.08116
33.11263	29.16496	23.47607	23.75343	23.92258	0.462726	0.3046	0.119381	-0.19585	-0.17582
593.593	566.3024	440.8138	477.6722	466.3091	0.13516	0.180598	0.104147	-0.25983	-0.1362
161.7532	155.6249	142.3097	137.2703	155.4055	0.13642	0.057889	9.38E-05	-0.12705	-0.17995
163.9333	143.0134	188.1697	169.3458	198.8514	-0.08259	-0.0072	-0.20289	0.191161	0.031316

257.892	257.5021	209.2621	232.9405	229.2046	0.056471	0.105964	0.104722	-0.19593	-0.04085
80.17781	89.97189	86.79879	87.80335	96.56388	-0.23784	-0.09688	0.069786	0.01532	0.033329
1131.762	1268.021	934.6033	962.741	915.0659	0.038719	0.111553	0.276718	-0.16626	-0.12475
370.8987	363.3345	292.0729	279.9616	270.9813	0.315356	0.218062	0.182225	-0.12759	-0.19396
327.5434	359.9596	420.6106	419.0494	416.3782	-0.21857	-0.16785	-0.03183	0.188145	0.173091
561.9879	556.5835	510.4552	552.3117	500.6065	-0.04713	0.095166	0.082314	-0.04257	0.06987
255.8909	244.3154	193.989	182.5532	203.6599	0.299496	0.221832	0.152722	-0.17028	-0.2718
41.58954	44.64548	31.99954	30.29974	33.01486	0.073242	0.175197	0.287624	-0.19614	-0.28424
142.251	127.9471	165.3226	161.7208	165.2539	-0.06252	-0.0354	-0.19104	0.181223	0.147343
340.1651	305.6325	348.6155	362.4732	292.5869	0.139963	0.021268	-0.13567	0.053271	0.112308
128.7144	137.0572	129.7371	109.4194	110.1724	0.114301	0.013529	0.102961	0.026449	-0.22393
290.0024	325.056	354.7304	331.4659	307.2683	-0.15125	-0.1188	0.045068	0.171584	0.072189
33.40339	34.17574	31.00305	26.54173	29.89414	0.232938	0.036827	0.071299	-0.06874	-0.29863
186.8654	181.1611	145.9834	170.5596	176.7087	0.135569	0.073994	0.032244	-0.28105	-0.059
47.74509	42.96072	35.95063	35.97986	36.64723	0.198338	0.243219	0.085909	-0.17011	-0.16864
459.4261	469.9149	406.6527	380.0152	396.6064	0.066746	0.11679	0.149035	-0.05945	-0.15644
46.08945	40.65979	41.64656	44.71832	37.58163	-0.0908	0.156558	-0.02511	0.006205	0.109701
316.0393	208.4852	355.8457	339.1158	274.6244	0.289982	0.049736	-0.55425	0.196299	0.155103
21.30649	17.48006	18.46073	19.26799	16.08683	0.015926	0.206978	-0.07595	0.004098	0.064541
803.9905	942.6249	610.0236	382.6654	415.2687	0.217436	0.070749	0.303637	-0.45614	-1.04766
99.33887	104.9779	112.5342	109.9195	93.3308	-0.03556	-0.05698	0.022312	0.122197	0.088952
52.89652	62.23262	82.58349	59.79451	75.74098	-0.31312	-0.22815	0.007849	0.393674	-0.07017
318.3108	383.4016	193.0193	202.4163	176.5867	0.212099	0.441437	0.664677	-0.28849	-0.22059
20.35734	21.62273	23.29845	25.22135	25.59783	-0.08368	-0.12829	-0.03752	0.06983	0.184776
43.3795	47.42339	31.29955	27.44499	31.9868	0.485679	0.172331	0.30232	-0.31836	-0.50535
48.24962	48.74858	48.74397	48.53479	54.12661	-0.01436	-0.02205	-0.00731	-0.0077	-0.01415
31.36384	28.55667	28.01969	25.93183	27.01218	0.182033	0.130815	-0.00536	-0.03618	-0.14858
26.96406	29.83183	26.34377	25.08233	25.40884	0.238607	0.016678	0.161914	-0.01588	-0.08769
240.3936	247.3641	280.6577	294.0092	268.9999	-0.24751	-0.11158	-0.07216	0.110174	0.17331
259.2871	261.3387	265.9492	292.1665	286.0547	-0.1995	-0.01592	-0.00537	0.020617	0.155755
32.76025	33.59135	23.25451	31.3508	31.8984	0.129237	0.074969	0.105925	-0.41678	0.016335
223.5943	222.8296	174.3135	200.5379	201.302	0.080273	0.040454	0.034685	-0.31875	-0.12279
147.8003	127.0849	115.0026	114.5761	116.9964	0.365366	0.252372	0.03071	-0.11597	-0.11597
74.48452	71.92954	44.0626	43.41723	46.61509	0.033191	0.368228	0.316455	-0.39361	-0.40567
418.704	388.6539	336.6917	319.7404	303.4735	0.027598	0.252394	0.142184	-0.06408	-0.13784
204.321	181.124	119.7474	106.4596	124.8785	0.235525	0.452639	0.265585	-0.33085	-0.49095
310.203	327.2927	468.1191	353.5728	364.2081	-0.20936	-0.15957	-0.07765	0.430427	0.025425
589.8137	465.1496	574.316	643.6719	600.503	-0.09285	0.038348	-0.30216	0.004513	0.169911
137.611	129.5855	274.7477	210.8241	201.026	-0.59501	-0.19054	-0.29093	0.746647	0.360752
328.8204	353.6653	358.2909	400.2581	355.4342	-0.04419	-0.11583	-0.01077	0.006196	0.164924
742.4663	802.3725	876.2581	817.6881	838.9949	-0.10815	-0.12362	-0.01127	0.116638	0.015886
286.7205	293.5984	220.3829	220.6427	226.3586	0.105407	0.153573	0.191205	-0.23378	-0.22795
136.8723	149.1768	218.6775	172.2973	189.8821	-0.2993	-0.20413	-0.08237	0.467412	0.119889
63.19375	58.22416	96.63561	87.49176	100.536	-0.0825	-0.39194	-0.51192	0.213948	0.082955
111.2907	113.2231	78.3279	98.84779	102.0848	-0.0501	0.119908	0.148713	-0.38323	-0.04727
85.02749	51.83242	42.03977	44.70699	40.30027	0.125377	0.886063	0.183633	-0.1026	-0.0167
22.37963	21.72809	22.95169	20.28052	25.36902	0.054105	-0.00215	-0.04505	0.032783	-0.14364

266.205	261.8244	360.5113	281.8757	299.1164	-0.06933	-0.02398	-0.0509	0.407262	0.054793
19.43051	17.9146	23.38779	17.54391	17.90334	-0.04351	0.096658	-0.01678	0.367373	-0.04752
111.5437	102.743	127.2689	123.1545	129.5402	-0.13461	-0.07806	-0.20543	0.110092	0.064868
24.33747	18.32591	29.50062	19.79065	25.63258	-0.15737	0.181372	-0.23278	0.450023	-0.11714
284.5188	211.9939	217.0315	212.483	220.8767	0.129118	0.3271	-0.09344	-0.06191	-0.09318
33.1084	33.41941	25.03625	33.89663	28.75624	0.082684	0.076697	0.083376	-0.3268	0.1028
288.6677	227.6832	164.8431	195.2166	184.4644	0.422919	0.393811	0.050818	-0.44128	-0.1865
36.71285	39.57206	45.26465	78.51492	60.71954	-0.14673	-0.29833	-0.20647	-0.01548	0.776137
142.6942	163.4068	162.2492	170.6077	179.5648	-0.21967	-0.14167	0.050828	0.037512	0.115132
175.5788	184.0046	153.2261	153.2324	172.474	0.076734	0.045543	0.114313	-0.1476	-0.1477
399.8541	351.511	386.126	412.3811	384.83	-0.04904	0.043227	-0.14139	-0.0065	0.0894
41.86419	49.05986	48.57146	52.07673	44.62871	-0.07914	-0.16128	0.070289	0.056887	0.153046
41.15196	41.38044	48.34883	40.38948	53.44191	-0.02091	-0.06109	-0.05363	0.170721	-0.08897
57.32234	63.24261	67.0758	59.56207	61.69791	-0.25772	-0.03753	0.104831	0.186942	0.014678
132.0248	103.2041	93.63512	72.25791	80.33688	0.028205	0.463623	0.117638	-0.0384	-0.40658
22.8219	21.1857	18.18099	21.35028	20.40261	0.164606	0.108169	0.006449	-0.21479	0.018239
38.33953	47.26324	47.95893	44.04042	41.50995	-0.03021	-0.16337	0.1395	0.158751	0.033325
55.65149	68.81242	48.43098	56.65633	53.33906	-0.06105	8.93E-04	0.301476	-0.20046	0.026975
199.1804	241.5062	244.1498	243.4184	266.8361	-0.32822	-0.28624	-0.00843	0.008022	0.003688
129.5353	119.467	101.6163	89.25832	81.67867	0.185914	0.296019	0.196216	-0.04014	-0.22398
31.42835	26.29448	27.44906	29.92225	28.46534	0.162971	0.122281	-0.13331	-0.07133	0.052748
35.15675	35.81563	23.14632	29.15553	30.72097	0.115047	0.189975	0.220564	-0.4085	-0.06909
139.5754	127.6433	161.1963	160.3066	162.1731	-0.17822	-0.05751	-0.18598	0.148344	0.13922
40.12693	41.71968	29.61748	28.72357	33.1681	0.324613	0.146071	0.200009	-0.30091	-0.3391
279.9237	288.3811	195.6347	195.846	208.3521	0.024937	0.315078	0.364109	-0.20566	-0.19519
121.5433	132.4684	154.1675	141.9373	146.4252	-0.0944	-0.19278	-0.06732	0.151614	0.030366
21.96722	23.09271	20.54133	27.49413	20.95548	0.187715	-0.05385	0.017019	-0.15096	0.266739
47.74258	46.72337	49.5129	51.45136	46.00824	-0.15605	0.029786	-9.36E-04	0.083365	0.135598
17.83479	20.65715	25.84633	21.69088	21.96179	-0.2424	-0.23718	-0.03602	0.301297	0.047808
123.0707	118.7994	143.0909	146.3409	146.5462	-0.07528	-0.11813	-0.17129	0.098747	0.133747
20.6761	19.51174	17.92389	18.42898	17.66906	0.176724	0.117148	0.037032	-0.0855	-0.04516
20.92915	17.76676	20.27661	18.55989	17.65709	0.06611	0.12463	-0.11213	0.078705	-0.04797
429.0896	496.2901	536.178	529.4704	605.1514	-0.15355	-0.27024	-0.05859	0.054109	0.035397
28.15422	27.65589	26.16499	32.54804	27.20182	0.100492	0.024684	-0.00211	-0.08088	0.231015
38.54114	32.73574	31.63234	38.87584	27.94701	-0.07	0.196864	-0.03263	-0.08557	0.210801
234.6368	221.2697	171.6773	158.1006	171.4457	0.28208	0.177758	0.09128	-0.28478	-0.42047
112.2947	97.45936	129.7142	123.0863	127.9885	0.11505	-0.12318	-0.33268	0.084935	0.007146
134.841	141.4933	167.5801	125.4695	148.3352	-0.12757	-0.07255	5.41E-04	0.239559	-0.17739
30.23696	29.97893	24.59123	28.48952	26.53712	0.093146	0.076653	0.066288	-0.22464	-0.00953
2508.282	2110.599	1039.98	1696.099	1571.553	0.375793	0.372984	0.154282	-0.91411	-0.19223
1720.29	1508.667	808.4302	1126.518	1210.434	0.331346	0.265418	0.098214	-0.83172	-0.34714
324.2353	259.1617	233.3815	251.8985	235.8876	0.817707	0.341379	-0.00433	-0.12937	-0.02959
1056.383	920.9312	1199.665	1160.726	1267.165	-0.13071	-0.07595	-0.27187	0.110968	0.063112
104.8481	115.9922	134.1558	112.4512	120.0922	-0.34524	-0.11002	0.036306	0.248381	-0.0054
23.26022	25.57307	21.71996	22.68585	24.87898	0.25821	-0.02597	0.107057	-0.12667	-0.06174
137.3472	152.2276	181.9341	171.8682	186.4787	-0.29831	-0.24099	-0.09108	0.166556	0.084206
35.49907	35.34109	46.26364	35.28405	36.53035	-0.19185	-0.02252	-0.03107	0.350527	-0.03126

40.96655	43.63644	69.24867	51.00374	62.24831	-0.05755	-0.2663	-0.17998	0.470392	0.04628
102.7278	109.1909	78.66692	79.44292	83.32965	0.183112	0.103201	0.193104	-0.29484	-0.27039
68.2365	75.45026	53.52475	47.58475	55.30818	0.611396	0.179938	0.354242	-0.14904	-0.32305
1978.448	1977.66	1249.816	1564.278	1454.615	0.116815	0.268465	0.272649	-0.41255	-0.06564
140.458	123.9511	149.8216	149.25	149.7597	-0.35753	0.064555	-0.1133	0.143141	0.152839
62.58307	59.44338	64.21848	66.93613	61.31025	-0.18232	0.018906	-0.05483	0.056149	0.11677
18.59825	16.30689	17.70649	16.53909	13.98123	-0.04456	0.154776	-0.03669	0.076691	-0.01852
35.85239	36.67756	38.82365	33.71664	34.28621	0.222916	-0.02362	0.008961	0.090269	-0.11391
98.00238	87.27502	97.95398	101.3353	84.10033	0.189106	0.01211	-0.15949	0.009024	0.059525
24.73151	24.56399	17.28313	16.93153	14.03564	0.367882	0.312219	0.29518	-0.19033	-0.22913
28.41743	29.82664	28.76752	28.8002	27.60984	0.194516	-0.02067	0.048879	-0.00406	-0.00175
25.67148	26.7497	23.21487	23.65614	25.81397	0.096641	-0.0209	0.038572	-0.16745	-0.14168
38.02072	32.60765	34.30688	35.73925	28.56942	0.087539	0.143746	-0.0762	-0.00182	0.053826
16.42919	16.27132	20.62532	16.59887	16.35104	-0.03545	-0.01097	-0.02816	0.318386	0.005574
26.38608	19.66747	18.76681	20.8028	20.16001	0.132927	0.34246	-0.07503	-0.14197	0.004556
31.03043	32.12777	29.0144	27.41271	29.46495	0.149727	0.04427	0.095622	-0.05247	-0.13303
121.2615	111.2309	187.6481	144.2131	152.0187	0.044003	-0.20419	-0.33468	0.415879	0.035732
608.1252	615.9653	737.428	685.427	695.5168	-0.11385	-0.11938	-0.09831	0.161301	0.054839
88.7127	83.08041	80.81336	93.40852	84.96861	-0.0041	0.049822	-0.0448	-0.08512	0.12444
542.7717	530.2108	421.1342	487.8359	434.4289	0.024268	0.136804	0.103961	-0.23128	-0.01694
38.04787	44.88048	34.60807	39.15841	36.63391	0.090911	-0.02333	0.216449	-0.1602	0.019154
1708.23	2016.105	1558.511	1502.004	1503.121	0.16434	0.017078	0.256145	-0.11683	-0.17292
495.0284	499.7406	396.234	384.2441	395.2495	0.285809	0.150256	0.1591	-0.17589	-0.21459
331.7267	390.2313	283.471	329.6558	337.3333	0.018212	-0.004	0.231133	-0.24056	-0.01226
18.58524	19.41715	19.85397	19.8541	17.49781	-0.21946	-0.0152	0.047659	0.078963	0.079647
200.0076	198.8076	212.628	301.6399	259.6428	-0.18232	-0.11932	-0.12046	-0.02334	0.474394
27.41569	25.11302	29.9715	23.97116	27.38534	-0.1835	0.084137	-0.04092	0.216812	-0.10563
155.8621	160.6097	225.4014	193.0352	216.6965	-0.17266	-0.18139	-0.14184	0.349928	0.125929
21.33554	17.25374	17.57849	16.25704	17.02098	-0.08326	0.296364	-0.00971	0.020572	-0.09452
82.03473	79.63513	100.5211	81.90116	89.58447	-0.1236	-0.03267	-0.07393	0.262725	-0.03781
18.32009	22.05975	22.72863	19.72478	20.63284	-0.06031	-0.14175	0.125409	0.169954	-0.03232
19.97975	18.1793	17.25112	17.6719	19.1506	0.239656	0.094471	-0.0384	-0.11621	-0.08041
24.46898	23.31795	22.24907	19.99653	20.86074	0.044052	0.148273	0.081329	0.013049	-0.1409
21.75744	23.11862	24.24186	28.72846	24.47296	-0.00688	-0.12638	-0.04126	0.028387	0.274264
38.31809	36.79541	39.45039	37.71965	32.73841	-0.06507	0.044672	-0.01362	0.083446	0.022025
28.33299	30.31355	28.91999	25.54224	25.89713	0.156647	2.64E-04	0.096561	0.029872	-0.15097
697.1346	768.1582	646.1328	644.9937	626.5327	-0.0321	0.083301	0.223307	-0.02535	-0.02849
99.67529	114.0728	136.8173	127.7657	124.942	-0.19908	-0.25831	-0.06093	0.202644	0.105562
177.3669	202.2225	268.0946	248.265	248.8092	-0.22124	-0.32684	-0.13923	0.268687	0.156475
279.2962	279.1553	317.2304	336.6558	314.6794	-0.1661	-0.03139	-0.03246	0.150605	0.236264
380.5824	424.625	431.2078	422.8972	409.9388	-0.17163	-0.09801	0.059529	0.081773	0.054624
413.922	426.6154	314.1748	274.6535	289.3029	0.183421	0.07219	0.112964	-0.34798	-0.53263
189.7356	173.5732	137.3933	168.011	158.9922	-0.02379	0.178868	0.045588	-0.2869	5.80E-04
221.5697	240.5648	264.1603	269.7116	245.7709	-0.09141	-0.14014	-0.02136	0.10761	0.14373
117.8558	107.1222	89.03717	100.8342	109.6311	0.112271	0.102631	-0.03367	-0.30404	-0.12213
158.534	139.3109	108.735	107.0457	98.74706	0.513283	0.24959	0.064879	-0.3022	-0.33215
122.3181	120.8088	170.6314	197.5361	190.2524	-0.3362	-0.33907	-0.3704	0.144255	0.342512

1291.03	1212.536	1420.435	1458.965	1594.772	-0.18214	-0.063	-0.15101	0.074589	0.114009
266.0038	268.0326	137.8267	143.1619	142.7428	0.348443	0.52493	0.530724	-0.44587	-0.36635
12.84149	14.30381	33.5439	19.24043	22.17423	-0.20314	-0.29791	-0.1396	1.03236	0.235259
55.09921	60.02828	72.64866	84.80708	83.7579	-0.16471	-0.31569	-0.19513	0.080352	0.305508
36.63035	34.32294	28.8051	34.24881	28.48248	0.001818	0.176948	0.087383	-0.16543	0.083248
80.8764	73.79544	65.55544	75.59991	69.72137	0.16173	0.113176	-0.01543	-0.19147	0.019394
94.89071	90.57267	83.72885	105.4624	99.4068	-0.01463	0.03053	-0.0369	-0.15088	0.175762
159.1607	169.1514	137.4877	116.9253	106.4992	0.358796	0.116249	0.204312	-0.09639	-0.34878
336.9497	268.9241	275.0382	280.2504	273.557	0.228113	0.229033	-0.09768	-0.06765	-0.04143
19.74841	22.52903	26.55914	24.43249	26.4044	0.033922	-0.28102	-0.08804	0.145338	0.027943
131.623	111.1854	180.6788	145.4154	140.3968	-0.02505	-0.05175	-0.29199	0.405662	0.091173
25.52841	28.04187	29.73418	32.34796	27.72952	-0.21189	-0.12644	0.008497	0.088069	0.214441
81.201	101.31	75.18733	63.27568	65.33317	0.086269	0.009821	0.327552	-0.10676	-0.37268
165.1028	190.006	103.7895	93.89803	114.2793	0.226377	0.351839	0.556579	-0.31254	-0.47427
86.09415	81.10464	58.20684	55.61654	62.50813	0.253739	0.247298	0.156376	-0.33767	-0.39916
201.2238	209.8519	242.5978	221.3536	237.7966	-0.18703	-0.09278	-0.0322	0.170671	0.044162
23.68738	27.07048	23.88877	22.86346	25.94383	0.226489	-0.05339	0.140198	-0.04197	-0.10351
600.0703	617.8785	790.3814	713.4204	692.0192	-0.26008	-0.08829	-0.04699	0.303557	0.148411
20.87128	20.66587	20.88031	18.78807	19.10901	0.147286	0.025123	0.011207	0.026557	-0.12572
481.0835	440.1953	665.5685	583.6762	572.2447	-0.09201	-0.10017	-0.22986	0.358337	0.168959
20.9332	22.2859	13.27322	11.89424	13.35687	0.295423	0.216454	0.311313	-0.45032	-0.58187

[D30-TAA](	Gene Symb	Gene Title	Pathway	Gene Onto	Gene Onto	Gene Ontology Molecular Function
0.150408	Ctbp1	C-terminal	binding pro	0000122 /,	0005634 /,	0003700 // sequence-specific DNA binding tr
-0.35562	Ncl	nucleolin	mRNA_pro	0001525 /,	0005634 /,	0000166 // nucleotide binding // inferred fro
-0.01225	Pnliprp1	pancreatic	lipase relat	0006629 /,	0005576 /,	0003824 // catalytic activity // inferred from
-0.17238	Scd2	stearoyl-Co	Fatty_Acid	0006629 /,	0005783 /,	0004768 // stearoyl-CoA 9-desaturase activit
-0.07467	Carhsp1	calcium regulated hea		0006355 /,	0005737 /,	0003676 // nucleic acid binding // inferred fr
-0.28219	Vcam1	vascular cell adhesion		0001666 /,	0005615 /,	0005178 // integrin binding // not recorded
0.01522	Klhdc3	kelch domain containi		0007126 /,	0000790 /,	0003682 // chromatin binding // inferred fro
-0.28658	Tbc1d15	TBC1 domain family, r		0032313 /,	0005576 /,	0005096 // GTPase activator activity // infer
0.265912	Nsg2	neuron specific gene f		0007212 /,	0005768 /,	0050780 // dopamine receptor binding // inf
-0.06115	Tmem19	transmembrane protein 19			0016020 // membrane // inferred from electronic anno	
-0.25006	Lamp2	lysosomal-associated membrane			0005624 // membrane fraction // not recorded /// 0005	
-0.12961	Ccne1	cyclin E1	Cell_Cycle_	0001547 /,	0000307 /,	0005515 // protein binding // inferred from
-0.267	Slc1a5	solute carrier family 1		0003333 /,	0016020 /,	0005515 // protein binding // inferred from
-0.28829	Wee1	WEE 1 hon	Cell_Cycle_	0006468 /,	0005634 /,	0000166 // nucleotide binding // inferred fro
-0.17063	Wee1	WEE 1 hon	Cell_Cycle_	0006468 /,	0005634 /,	0000166 // nucleotide binding // inferred fro
0.349429	Gas1	growth arrest specific		0002053 /,	0005886 /,	0005515 // protein binding // inferred from
-0.04684	Mcl1	myeloid ce	Apoptosis_	0006915 /,	0005634 /,	0005515 // protein binding // inferred from
-0.16966	Trp53inp1	transformation relate		0006915 /,	0005634 // nucleus // inferred from direct assay /// 000	
-0.19923	Nr1d2	nuclear rec	Circadian_	0006350 /,	0005634 /,	0003677 // DNA binding // inferred from dir
-0.35313	Nr1d2	nuclear rec	Circadian_	0006350 /,	0005634 /,	0003677 // DNA binding // inferred from dir
-0.09623	Rassf3	Ras association (RalGI		0007165 /,	0005737 /,	0005515 // protein binding // inferred from
0.082919	Tmem183a	transmembrane protein 183A			0016020 // membrane // inferred from electronic anno	
-0.05499	Cetn3	centrin 3		0007049 /,	0005737 /,	0005509 // calcium ion binding // inferred fr
-0.65837	Ptgs2	prostaglan	Eicosanoid	0001516 /,	0005634 /,	0004601 // peroxidase activity // inferred fro
-0.36185	Smpdl3b	sphingomyelin phospl		0006685 /,	0005576 /,	0004767 // sphingomyelin phosphodiesteras
0.159936	Entpd5	ectonucleoside tripho		0008152 /,	0005783 /,	0016787 // hydrolase activity // inferred fron
0.15645	Npepps	aminopeptidase puro		0006508 /,	0005634 /,	0004177 // aminopeptidase activity // not re
-0.21191	Fmo1	flavin containing mon		0006082 /,	0005783 /,	0004497 // monooxygenase activity // not re
0.004009	Fam151a	family with sequence simliarity 1			0016020 // membrane // inferred from electronic anno	
-0.15186	Slc12a4	solute carrier family 1		0006810 /,	0016020 /,	0005215 // transporter activity // inferred fr
0.242199	Otub2	OTU domain, ubiquitii		0006519 // cellular an		0004843 // ubiquitin-specific protease activi
0.155445	Mtor	mechanistic target of		0001938 /,	0000139 /,	0000166 // nucleotide binding // inferred fro
-0.67058	Per2	period hon	Circadian_	0006350 /,	0005634 /,	0004871 // signal transducer activity // infer

-0.25912	Lefty1	left right determinatic	0007275 /,	0005576 /,	0005125 // cytokine activity // inferred from	
-0.23514	Pvalb	parvalbumin		0005737 /,	0005509 // calcium ion binding // inferred fr	
-0.21998	Soat1	sterol O-ac Statin_Patl	0006629 /,	0005783 /,	0000062 // acyl-CoA binding // inferred from	
-0.11182	Pla1a	phospholipase A1 mei	0006629 /,	0005576 /,	0003824 // catalytic activity // inferred from	
-0.40308	Ccl11	chemokine (C-C motif	0001938 /,	0005576 /,	0005125 // cytokine activity // inferred from	
0.157368	Kcnb1 /// I	potassium Calcium_re	0006810 /,	0005737 /,	0005216 // ion channel activity // inferred fr	
0.226842	Ube2l3	ubiquitin-conjugating	0000209 /,	0005634 /,	0000166 // nucleotide binding // inferred fro	
-0.1165	Lims1	LIM and senescent cel	0007160 /,	0005886 /,	0005515 // protein binding // inferred from	
0.287887	Efnb1	ephrin B1	0001755 /,	0016020 /,	0005515 // protein binding // inferred from	
-0.17891	Ifit2	interferon-induced pr	0035457 // cellular re		0005488 // binding // inferred from electron	
-0.1614	Rbfox1	RNA bindir mRNA_prc	0006397 /,	0005634 /,	0000166 // nucleotide binding // inferred fro	
-0.06615	Ufsp2	UFM1-specific peptida	0008152 // metabolic		0008233 // peptidase activity // inferred fron	
-0.26114	Slc7a5	solute carrier family 7	0003333 /,	0005737 /,	0015171 // amino acid transmembrane tran	
0.071361	Afg3l1	AFG3(ATPa mRNA_prc	0006508 /,	0005739 /,	0000166 // nucleotide binding // inferred fro	
-0.34589	Gbp3	guanylate binding protein 3		0005737 /,	0000166 // nucleotide binding // inferred fro	
-0.21886	Stx7	syntaxin 7	0006886 /,	0005768 /,	0005484 // SNAP receptor activity // inferre	
-0.58316	Col8a1	collagen, type VIII, alp	0001525 /,	0005576 // extracellular region // inferred from electro		
-0.08655	Yes1	Yamaguchi sarcoma v	0006468 /,	0005624 /,	0000166 // nucleotide binding // inferred fro	
-0.12361	Dnaja4	DnaJ (Hsp40) homolog	0006457 /,	0016020 /,	0005524 // ATP binding // inferred from elec	
0.238978	Avpr1a	arginine va GPCRDB_C	0001992 /,	0005886 /,	0004871 // signal transducer activity // infer	
0.415939	Clock	circadian lc Circadian_	0006350 /,	0005634 /,	0003677 // DNA binding // inferred from ele	
0.218178	Clock	circadian lc Circadian_	0006350 /,	0005634 /,	0003677 // DNA binding // inferred from ele	
-0.43592	Inmt	indolethylamine N-me	0008152 /,	0005737 /,	0008168 // methyltransferase activity // infe	
0.151035	Haus8	4HAUS augmin-like cc	0007049 /,	0000922 // spindle pole // inferred from electronic ann		
-0.1994	Csnk1d	casein kinase 1, delta	0006468 /,	0005625 /,	0000166 // nucleotide binding // inferred fro	
-0.37256	Cebpb	CCAAT/enl Circadian_	0001892 /,	0005634 /,	0003677 // DNA binding // inferred from dir	
0.383945	Nfil3	nuclear factor, interle	0006350 /,	0005634 /,	0003677 // DNA binding // inferred from ele	
-0.42813	Arpp21	cyclic AMP-regulated	0034605 /,	0005737 /,	0003676 // nucleic acid binding // inferred fi	
-0.20398	Cyp2d22	cytochrome P450, fan	0010033 /,	0005737 /,	0004497 // monooxygenase activity // not re	
-0.13147	Cys1	cystin 1	0006810 /,	0005737 /,	0005215 // transporter activity // inferred fr	
-0.09813	Alg14	asparagine-linked glycosylation 1		0005783 // endoplasmic reticulum // inferred from elec		
-0.22002	Gck	glucokinas Glycolysis_	0001678 /,	0005625 /,	0000166 // nucleotide binding // inferred fro	
-0.3931	Fibin	fin bud initiation factor homolog		0005576 // extracellular region // inferred from electro		
0.069757	Med9	mediator of RNA poly	0006350 /,	0005634 /,	0005515 // protein binding // inferred from	
-0.00908	Ank1	ankyrin 1, erythroid	0006779 /,	0005634 /,	0005515 // protein binding // inferred from	
-0.71775	C3ar1	compleme GPCRDB_C	0002430 /,	0005886 /,	0001850 // complement component C3a bin	
0.023135	Fank1	fibronectin type 3 and ankyrin re		0005634 // nucleus // inferred from electronic annotati		
-0.13068	Ifi204	interferon activated g	0006350 /,	0005634 /,	0003712 // transcription cofactor activity //	
-0.46917	Tpm2	tropomyos Striated_m	0006936 /,	0005737 /,	0003779 // actin binding // inferred from ele	
-0.14983	Chi3l3	chitinase 3-like 3	0005975 /,	0005576 /,	0003824 // catalytic activity // inferred from	
-0.01671						
-0.26869	Ms4a4c	membrane-spanning 4-domains,		0016021 // integral to membrane // inferred from elect		
-0.52231	Tsc22d3	TSC22 domain family,	0006355 // regulation		0003700 // sequence-specific DNA binding ti	
-0.39348	Atrx	alpha thalassemia/me	0006281 /,	0000228 /,	0000166 // nucleotide binding // inferred fro	
-0.0565	Ubr1	ubiquitin protein ligas	0006511 /,	0000151 /,	0004842 // ubiquitin-protein ligase activity /	
0.91559	Npas2	neuronal PAS domain	0006350 /,	0005634 /,	0003677 // DNA binding // not recorded ///	
-0.03076	Ypel1	yippee-like 1 (Drosophila)		0005634 // nucleus // inferred from electronic annotati		

-0.08476	Cyp7b1	cytochrome P450, fan		0006629 /,	0005783 /,	0004497 // monooxygenase activity // inferr
-0.10163	Cyp7b1	cytochrome P450, fan		0006629 /,	0005783 /,	0004497 // monooxygenase activity // inferr
-0.43702	Per3	period homolog 3 (Dr		0006350 /,	0005634 /,	0004871 // signal transducer activity // infer
-0.30924	Bmp2k	BMP2 inducible kinas		0006468 /,	0005634 /,	0000166 // nucleotide binding // inferred fro
-0.07422	Pga5	pepsinogen 5, group I		0006508 /,	0005625 /,	0004190 // aspartic-type endopeptidase acti
-0.43746	Epyc	epiphycan			0005576 /,	0005515 // protein binding // inferred from
0.174006	Rpgrip1	retinitis pigmentosa G		0006260 /,	0005634 /,	0005515 // protein binding // inferred from
-0.05353	Atrn	attractin		0006954 /,	0005737 /,	0004872 // receptor activity // inferred from
-0.39019	Itgav	integrin al	Integrin-m	0001525 /,	0005886 /,	0004872 // receptor activity // inferred from
-0.33096	Gzmc	granzyme C		0006508 // proteolysi		0003824 // catalytic activity // inferred from
0.051459	Egr3	early growth respons		0006350 /,	0005622 /,	0003676 // nucleic acid binding // inferred fi
-0.22244	Eya1	eyes absent 1 homolo		0001656 /,	0005634 /,	0003824 // catalytic activity // inferred from
0.140253	Dgcr2	DiGeorge syndrome c		0007155 /,	0005792 /,	0004872 // receptor activity // inferred from
-0.12641	Pnlip	pancreatic lipase		0006629 /,	0005615 /,	0003824 // catalytic activity // inferred from
-0.09214	Stag1	stromal antigen 1		0007049 /,	0000785 /,	0005488 // binding // inferred from electror
-0.19147	Hivep2	human immunodefici		0006350 /,	0005622 /,	0003676 // nucleic acid binding // inferred fi
-0.21671	Spry4	sprouty homolog 4 (D		0007275 /,	0005737 /,	0005515 // protein binding // inferred from
-0.17848	Gstm6	glutathione S-transfer		0008152 /,	0005737 /,	0004364 // glutathione transferase activity /
0.214577	Hist2h3c2-	histone cluster 2, H3c		0006334 /,	0000786 /,	0003677 // DNA binding // inferred from ele
-0.02998	Cyb5r3	cytochrome b5 reduct		0006694 /,	0005625 /,	0004128 // cytochrome-b5 reductase activit
-0.0153	Fgf7	fibroblast growth fact		0001541 /,	0005576 /,	0005111 // type 2 fibroblast growth factor re
0.50686	Tmem45a	transmembrane protein 45a			0005887 // integral to plasma membrane // inferred fro	
-0.239	Rbm3	RNA bindir	mRNA_prc	0006412 /,	0005634 /,	0000166 // nucleotide binding // inferred fro
0.203807	Sub1	SUB1 homolog (S. cer		0006350 /,	0005634 /,	0003677 // DNA binding // inferred from ele
0.043975	Bat2	HLA-B associated transcript 2			0005634 /,	0005515 // protein binding // inferred from
-0.10226	Stard5	StAR-related lipid trar		0006694 // steroid bi		0008289 // lipid binding // inferred from ele
0.160977	Lman2	lectin, mannose-bindi		0006810 /,	0000139 /,	0005529 // sugar binding // inferred from el
-0.1366	Incenp	inner centromere pro		0000910 /,	0000775 /,	0005515 // protein binding // inferred from
-0.20943	Col1a2	collagen, t	Inflammat	0001501 /,	0005576 /,	0005201 // extracellular matrix structural co
-0.45214	Tcap	titin-cap	Striated_m	0001756 /,	0005737 /,	0005515 // protein binding // inferred from
-0.34862	Itsn2	intersectin 2		0006897 /,	0005622 /,	0005089 // Rho guanyl-nucleotide exchange
-0.17846	B4galt6	UDP-Gal:betaGlcNAc I		0005975 /,	0005794 /,	0008378 // galactosyltransferase activity // r
0.10604	Cyp2c68	cytochrome P450, fan		0055114 /,	0005783 /,	0004497 // monooxygenase activity // inferr
-0.29847	Hhex	hematopoietically exp		0000122 /,	0005634 /,	0003677 // DNA binding // inferred from ele
-0.23454	Fbln2	fibulin 2		0010811 /,	0005576 /,	0005509 // calcium ion binding // inferred fr
-0.19105	Ptgs1	prostaglan	Eicosanoid	0001516 /,	0005634 /,	0004601 // peroxidase activity // inferred fro
-0.21735	Steap3	STEAP family member		0006810 /,	0005768 /,	0000293 // ferric-chelate reductase activity ,
-0.07723	S1pr1	sphingosin	GPCRDB_C	0001525 /,	0005886 /,	0001619 // lysosphingolipid and lysophosph
0.087646	Oxa1l	oxidase assembly 1-lik		0009060 /,	0005739 // mitochondrion // inferred from direct assay	
0.088005	Rbm10	RNA bindir	mRNA_processing_bi		0005622 /,	0000166 // nucleotide binding // inferred fro
0.028345	Usp20	ubiquitin specific pep		0006511 /,	0005737 /,	0001664 // G-protein-coupled receptor bind
0.169265	Acsl1	acyl-CoA s	Fatty_Acid	0006629 /,	0005625 /,	0000166 // nucleotide binding // inferred fro
-0.13623	Lamc1	laminin, ga	Inflammat	0006461 /,	0005576 /,	0005201 // extracellular matrix structural co
0.025729	2310044H:	RIKEN cDNA 2310044H10 gene			0005576 // extracellular region // inferred from electro	
0.190249	Ahsa1	AHA1, activator of he		0006457 /,	0005737 /,	0001671 // ATPase activator activity // not r
-0.20506	Tef	thyrotroph embryonic		0006350 /,	0005634 /,	0003677 // DNA binding // inferred from ele
-0.08344	Yipf1	Yip1 domain family, member 1			0016020 // membrane // inferred from electronic anno	

-0.2245	Meox2	mesenchyme homeob	0001525 /,	0005634 /,	0003677 // DNA binding // inferred from ele
-0.45723	Meox2	mesenchyme homeob	0001525 /,	0005634 /,	0003677 // DNA binding // inferred from ele
0.060147	Fam114a2	family with sequence similarity 114, member A2			
-0.64555	Prkx	protein kinase, X-linke	0006468 // protein pl		0000166 // nucleotide binding // inferred fro
0.032082	Ppcdc	phosphopantothenoy	0015937 // coenzyme		0003824 // catalytic activity // inferred from
-0.13046	Ddx6	DEAD (Asp mRNA_prc	0006200 /,	0000932 /,	0000166 // nucleotide binding // inferred fro
-0.16043	Wdfy1	WD repeat and FYVE domain con		0005634 /,	0005545 // phosphatidylinositol binding // ir
-0.07358	Csnk1a1	casein kinase 1, alpha	0000902 /,	0000775 /,	0000166 // nucleotide binding // inferred fro
-0.16452	Trim34	tripartite motif-containing 34		0005622 /,	0005515 // protein binding // inferred from
0.088264	2310044G:	RIKEN cDNA 2310044G17 gene			
0.112757	BC002059	cDNA sequence BC002059		0005622 /,	0003676 // nucleic acid binding // inferred fı
0.811756	Arntl	aryl hydrocarbon rece	0000060 /,	0005634 /,	0003677 // DNA binding // inferred from phy
-0.15193	Sel1l	sel-1 suppressor of lin	0007219 /,	0005783 /,	0005488 // binding // inferred from electror
0.167956	Gatc	glutamyl-tl mRNA_prc	0006450 /,	0005739 // mitochondrion // inferred from direct assay	
-0.19471	Col20a1	collagen, type XX, alpl	0007155 /,	0005576 /,	0005198 // structural molecule activity // inf
-0.16967	Fam76a	family with sequence similarity 76, member A			
-0.02495	Chi3l3 /// (	chitinase 3-like 3 /// c	0005975 /,	0005576 /,	0003824 // catalytic activity // inferred from
-0.31796	Dusp3	dual specificity phosp	0000188 /,	0001772 /,	0004721 // phosphoprotein phosphatase ac
0.352739	Caln1	calneuron 1		0005737 /,	0005509 // calcium ion binding // not record
-0.05065	Ly6i	lymphocyte antigen 6 complex, lo		0005886 // plasma membrane // traceable author state	
0.091691	Hexim1	hexamethylene bis-ac	0000122 /,	0005634 /,	0004861 // cyclin-dependent protein kinase
-0.10646	Pde1c	phosphodi G_Protein_	0006198 /,	0043025 /,	0003824 // catalytic activity // inferred from
0.031978	Kcnq2	potassium voltage-gat	0006810 /,	0008076 /,	0005216 // ion channel activity // inferred fr
0.052447	Klra18	killer cell lectin-like re	0007155 /,	0005886 /,	0004872 // receptor activity // inferred from
0.03214	Vps37c	vacuolar protein sorti	0006810 /,	0005768 // endosome // inferred from electronic annot	
0.042286	Ap1ar	adaptor-related protein complex	0030133 // transport vesicle // inferred from electronic		
-0.22096	Zc3h11a	zinc finger CCCH type containing 11A			0003676 // nucleic acid binding // inferred fı
-0.62343	Slmap	sarcolemma associated protein		0005737 // cytoplasm // inferred from electronic annot	
-0.06819	Gphn	gephyrin	0006777 /,	0005622 /,	0000166 // nucleotide binding // inferred fro
-0.13904	Nr1d1	nuclear receptor subfa	0006350 /,	0005634 /,	0003677 // DNA binding // inferred from dir
-0.09997	Sec24b	Sec24 related gene fa	0006810 /,	0030127 /,	0005515 // protein binding // inferred from
0.104274	5430417L2	RIKEN cDNA 5430417L22 gene			
0.220234	Adamts15	a disintegrin-like and ı	0006508 /,	0005576 /,	0004222 // metalloendopeptidase activity //
-0.25531	Col14a1	collagen, type XIV, alp	0007155 /,	0005576 /,	0005198 // structural molecule activity // inf
-0.16986	Pard3b	par-3 partitioning defe	0007049 /,	0005923 /,	0005515 // protein binding // inferred from
0.127875	Med19	mediator of RNA poly	0006350 /,	0005634 /,	0005515 // protein binding // inferred from
0.242506	Fastkd1	FAST kinase domains :	0006915 // apoptosis		0004672 // protein kinase activity // inferrec
-0.03453	Myo1b	myosin IB	0008152 /,	0005903 /,	0000166 // nucleotide binding // inferred fro
-0.23163	Fbxl3	F-box and leucine-rich	0031146 /,	0005634 /,	0004842 // ubiquitin-protein ligase activity /
0.017793	Rian	RNA imprinted and accumulated		0005634 // nucleus // inferred from direct assay	
-0.2058	Tcrg-V1	T-cell receptor gamma, variable 1			0004872 // receptor activity // inferred from
0.038558	Abhd10	abhydrolase domain c	0006508 /,	0005739 /,	0008233 // peptidase activity // inferred fron
-0.10925	Zfa /// Zfx	zinc finger protein, au	0001541 /,	0005622 /,	0003676 // nucleic acid binding // inferred fı
0.042394	Gm16382 ,	ribosomal Ribosomal	0006412 /,	0005622 /,	0003735 // structural constituent of ribosom
-0.27118	Ppat	phosphoribosyl pyrop	0006164 /,	0005625 /,	0004044 // amidophosphoribosyltransferase
0.034212	Ppfia4	protein tyrosine phosphatase, receptor type			0005515 // protein binding // inferred from
0.130404	Ddx51	DEAD (Asp-Glu-Ala-As	0006200 /,	0005634 /,	0000166 // nucleotide binding // inferred fro

-0.47704	Snw1	SNW domain containi	0000398 /,	0005634 /,	0003713 // transcription coactivator activity
-0.06267	1700017B(	RIKEN cDNA 1700017B05 gene			
-0.09082	Cdc37l1	cell division cycle 37 homolog (S.		0005737 // cytoplasm // inferred from direct assay /// (	
-0.19514	Ccdc50	coiled-coil domain containing 50		0005737 // cytoplasm // inferred from electronic annot	
-0.30981	Ak3	adenylate kinase 3	0006139 /,	0005737 /,	0000166 // nucleotide binding // inferred fro
0.201384	Pygb	brain glycogen phospl	0005975 /,	0005625 /,	0004645 // phosphorylase activity // inferre
0.006524	Ran	RAN, member RAS on	0006184 /,	0005634 /,	0000166 // nucleotide binding // inferred fro
0.321458	Syt17	synaptotagmin XVII	0006810 /,	0005802 /,	0005215 // transporter activity // inferred fr
-0.13098	Paxip1	PAX interacting (with	0006974 /,	0005622 /,	0005515 // protein binding // inferred from
0.014021	Tada2b	transcriptional adapto	0045449 /,	0005634 /,	0003677 // DNA binding // inferred from ele
-0.39265	Tbl1x	transducin (beta)-like	0006350 /,	0005634 /,	0003677 // DNA binding // inferred from dir
0.075974	Zfr2	zinc finger RNA bindin	0006955 /,	0005622 /,	0003723 // RNA binding // inferred from ele
-0.05709	2010111I0	RIKEN cDNA 2010111I	0006508 /,	0005737 /,	0004177 // aminopeptidase activity // inferr
0.054696	Ahsa2	AHA1, activator of hea	0006950 /,	0005737 /,	0001671 // ATPase activator activity // infer
0.137333	Zbtb45	zinc finger and BTB domain conta		0005622 /,	0003676 // nucleic acid binding // inferred fr
0.143141	Hr	hairless	0006350 /,	0000118 /,	0003677 // DNA binding // inferred from ele
-0.08584	Myo15b	myosin XVB		0005856 /,	0005488 // binding // inferred from electror
0.41534	Dnajc5	DnaJ (Hsp40) homolog	0006457 /,	0016020 /,	0031072 // heat shock protein binding // inf
-0.1364	Gm505	Predicted gene 505		0016607 // nuclear speck // inferred from electronic an	
-0.06349	1700010I1	RIKEN cDNA 1700010I14 gene			
0.13739	Clns1a	chloride channel, nucl	0006821 /,	0005634 /,	0005515 // protein binding // inferred from
-0.38487	Sntb2	syntrophin, basic 2		0005737 /,	0003779 // actin binding // inferred from ele
-0.12365	Aldh9a1	aldehyde dehydrogen	0001822 /,	0005634 /,	0004029 // aldehyde dehydrogenase (NAD)
0.036234	Rps29	ribosomal protein S29	0006412 /,	0005622 /,	0003735 // structural constituent of ribosom
-0.45626	Tef	thyrotroph embryonic	0006350 /,	0005634 /,	0003677 // DNA binding // inferred from ele
0.30597	Dos	downstream of Stk11		0016020 // membrane // inferred from electronic anno	
-0.07944	Fam120b	family with sequence	0006350 /,	0005634 /,	0005515 // protein binding // inferred from
-0.41906	U46068	cDNA sequence U46068		0005576 /,	0008289 // lipid binding // inferred from ele
0.207725	Serbp1	serpine1 mRNA bindir	0045767 /,	0005634 /,	0003723 // RNA binding // inferred from ele
-0.28287	Bach1	BTB and CNC homolog	0006350 /,	0005634 /,	0003677 // DNA binding // inferred from dir
-0.21803					
-0.26014	Aldh7a1	aldehyde dehydrogen	0008152 /,	0005634 /,	0004029 // aldehyde dehydrogenase (NAD)
0.104249	Cct3	chaperonin containing	0006457 /,	0005737 /,	0000166 // nucleotide binding // inferred fro
-0.40204	Tspan4	tetraspanin 4		0016020 /,	0005515 // protein binding // inferred from
-0.21822	Ebf1	early B-cell factor 1	0006350 /,	0005634 /,	0003677 // DNA binding // inferred from dir
0.204388	Tnk2	tyrosine ki G13_Signa	0006468 /,	0005886 /,	0000166 // nucleotide binding // inferred fro
-0.31098	Mcl1	myeloid ce Apoptosis_	0006915 /,	0005634 /,	0005515 // protein binding // inferred from
-0.55316	Lpar1	lysophosphatidic acid	0000187 /,	0005737 /,	0001619 // lysosphingolipid and lysophosph
-0.22996	Mafg	v-maf musculoaponeu	0001701 /,	0005634 /,	0003677 // DNA binding // inferred from dir
-0.16161	Usp25	ubiquitin specific pept	0006511 /,	0000502 /,	0004221 // ubiquitin thiolesterase activity //
-0.02544	Myo1b	myosin IB	0008152 /,	0005903 /,	0000166 // nucleotide binding // inferred fro
-0.12835	Ebf2	early B-cell factor 2	0006350 /,	0005634 /,	0003677 // DNA binding // inferred from dir
-0.3691	Slco1a5	solute carrier organic	0006810 /,	0005624 /,	0005215 // transporter activity // inferred fr
-0.08467	Itgae	integrin alp Integrin-m	0007155 /,	0008305 /,	0004872 // receptor activity // inferred from
-0.04584	Dcbld1	discoidin, CUB and LCCL domain		0016020 // membrane // inferred from electronic anno	
0.067623	Dbndd1	dysbindin (dystrobrevin binding		0005737 /,	0005515 // protein binding // inferred from
0.091249	Zfp235	zinc finger protein 235	0006355 /,	0005622 /,	0003676 // nucleic acid binding // inferred fr

-0.16742	Pdgfc	platelet-derived grow	0007171 /,	0005576 /,	0005161 // platelet-derived growth factor re	
-0.11746	Jam2	junction adhesion molecule 2		0005886 // plasma membrane // inferred from electron		
-0.26721	Cd93	CD93 antigen	0007155 /,	0005886 /,	0001849 // complement component C1q bir	
0.045066	Kcnn1	potassium intermedia	0006810 /,	0016020 /,	0005216 // ion channel activity // inferred fr	
-0.92549	Prg4	proteoglycan 4 (mega	0006955 /,	0005576 /,	0005044 // scavenger receptor activity // inf	
-0.03621	Ephb4	Eph receptor B4	0006468 /,	0005887 /,	0000166 // nucleotide binding // inferred fro	
-0.54564	Col4a3bp	collagen, type IV, alph	0000902 /,	0005737 /,	0005515 // protein binding // inferred from	
-0.3681	Per1	period hon	Circadian_	0006350 /,	0005634 /,	0004871 // signal transducer activity // infer
-0.5873	Cpeb4	cytoplasmi	mRNA_processing_binding_Reac		0000166 // nucleotide binding // inferred fro	
-0.00831	Aen	apoptosis enhancing r	0006915 /,	0005622 /,	0003676 // nucleic acid binding // inferred fi	
-0.32466	Col5a2	collagen, type V, alpha	0001501 /,	0005576 /,	0005201 // extracellular matrix structural co	
-0.19248	Ctsk	cathepsin K	0001957 /,	0005615 /,	0004197 // cysteine-type endopeptidase act	
-0.48774	Saa3	serum amyloid A 3	0006953 /,	0005576 // extracellular region // inferred from electro		
0.109786	Pa2g4	proliferation-associate	0006350 /,	0005634 /,	0003677 // DNA binding // not recorded ///	
0.319548	Dclk1	doublecortin-like kina	0001764 // neuron mi		0000166 // nucleotide binding // inferred fro	
-0.25937	Slc9a3r1	solute carrier family 9	0016055 /,	0001726 /,	0005515 // protein binding // inferred from	
-0.09039	Trip12	thyroid hormone rece	0006464 /,	0005622 /,	0005488 // binding // inferred from electron	
-0.08585	Wdr26	WD repeat domain 26		0005737 // cytoplasm // inferred from electronic annot		
-0.003	Spsb4	splA/ryanodine recep	0023034 /,	0005737 /,	0005515 // protein binding // inferred from	
-0.35703	Angptl7	angiopoietin-like 7	0007165 /,	0005576 /,	0005102 // receptor binding // inferred from	
0.121956	Efnb1	ephrin B1	0001755 /,	0016020 /,	0005515 // protein binding // inferred from	
-0.04148	Polr3f	polymerase (RNA) III (	0006350 /,	0005634 /,	0003677 // DNA binding // inferred from ele	
-0.04434	Tmem209	transmembrane protein 209		0016020 // membrane // inferred from electronic anno		
-0.08534	Tbc1d23	TBC1 domain family, r	0032313 /,	0005622 /,	0005097 // Rab GTPase activator activity // i	
-0.86248	Lmod2	leiomodin 2 (cardiac)		0005856 /,	0003779 // actin binding // inferred from ele	
0.082884	Amotl2	angiomotin-like 2		0005923 /,	0005515 // protein binding // inferred from	
-0.32618	Phf3	PHD finger protein 3	0006350 /,	0005634 /,	0005515 // protein binding // inferred from	
-0.39077	Cep350	centrosomal protein 350				
0.024068	Plcb2	phospholipase C, beta	0001580 /,	0005794 /,	0004435 // phosphoinositide phospholipase	
-0.18244	Gtf2h1	general tra	RNA_trans	0006281 /,	0005634 /,	0004672 // protein kinase activity // inferred
-0.115	2700089E2	RIKEN cDNA 2700089E24 gene				
-2.66E-04	Mbd1	methyl-CpG binding d	0006306 /,	0000785 /,	0003677 // DNA binding // inferred from dir	
-0.01123	Rere	arginine glutamic acid	0006338 /,	0000118 /,	0003677 // DNA binding // inferred from ele	
0.174723	Thra	thyroid ho	Nuclear_R	0001502 /,	0005634 /,	0002153 // steroid receptor RNA activator R
0.004461	Klhdc3	kelch domain contain	0007126 /,	0000790 /,	0003682 // chromatin binding // inferred fro	
-0.20863	Fbxo3	F-box protein 3		0005634 // nucleus // inferred from electronic annotati		
0.146447	Diras1	DIRAS family, GTP-bin	0006184 /,	0005622 /,	0000166 // nucleotide binding // inferred fro	
0.00701	Gm13654 ,	predicted g	mRNA_pro	0000028 /,	0005622 /,	0003735 // structural constituent of ribosom
0.046115	Slc35a4	solute carrier family 3	0006810 /,	0000139 /,	0005351 // sugar:hydrogen symporter activi	
-0.21257	Secisbp2l	SECIS binding protein 2-like				
-0.24645	Wdr33	WD repeat domain 33		0005634 /,	0005515 // protein binding // inferred from	
-0.18133	Serpinh1	serine (or cysteine) pe	0006950 /,	0005737 /,	0004867 // serine-type endopeptidase inhib	
-0.057	Mir425	microRNA 425				
-0.16073	Pou3f1	POU domain, class 3,	0006350 /,	0005634 /,	0003677 // DNA binding // inferred from dir	
-0.16725	Sptlc2	serine palmitoyltransf	0006686 /,	0005739 /,	0003824 // catalytic activity // inferred from	
5.56E-04	Gpr88	G-protein (	GPCRDB_C	0007165 /,	0005886 /,	0004871 // signal transducer activity // infer
0.270834	Top1mt	DNA topoisomerase 1	0006265 /,	0005694 /,	0000166 // nucleotide binding // inferred fro	

-0.06413	Egfr	epidermal growth fact	0000186 /,	0005622 /,	0000166 // nucleotide binding // inferred fro
0.167082	Trub1	TruB pseudouridine (p	0001522 // pseudouri		0003723 // RNA binding // inferred from ele
-0.19431	Pik3ip1	phosphoinositide-3-kinase intera		0016020 // membrane // inferred from electronic anno	
-0.23537	Coq10b	coenzyme Q10 homolog B (S. cer		0005739 // mitochondrion // inferred from direct assay	
0.177823	Secisbp2	SECIS binding protein 2		0005739 /,	0003723 // RNA binding // not recorded ///
-0.07133	Alkbh3	alkB, alkylation repair	0006281 /,	0005634 /,	0004519 // endonuclease activity // inferred
-0.09978	Paqr8	progestin and adipoQ	0007165 /,	0005886 /,	0004872 // receptor activity // inferred from
-0.15142	Dynlrb2	dynein light chain roa	0006810 /,	0005737 /,	0003774 // motor activity // inferred from e
0.175753	Hexim2	hexamthylene bis-ace	0000122 /,	0005634 /,	0004861 // cyclin-dependent protein kinase
-0.19632	Ddi2	DNA-damage inducibl	0006508 // proteolysi		0004190 // aspartic-type endopeptidase acti
-0.21243	Prkaa2	protein kinase, AMP-a	0006468 /,	0005634 /,	0000166 // nucleotide binding // inferred fro
-0.04047	Bcl2l13	BCL2-like 13 (apoptos	0006915 /,	0005739 // mitochondrion // inferred from direct assay	
-0.12697	5730455O:	RIKEN cDNA 5730455O13 gene		0005634 /,	0005515 // protein binding // inferred from
-0.00553	Aff3	AF4/FMR2 family, me	0006350 /,	0005634 /,	0003677 // DNA binding // inferred from ele
-0.14246	Cyp20a1	cytochrome P450, fan	0055114 /,	0016020 /,	0004497 // monooxygenase activity // inferr
-0.09563	Aasdhppt	aminoadipate-semialc	0009059 /,	0005737 /,	0000287 // magnesium ion binding // inferre
-0.13811	Myg1	melanocyte proliferating gene 1		0005634 // nucleus // inferred from electronic annotati	
-0.15118	1110002E2	RIKEN cDNA 1110002E22 gene			
-0.19457	Plcb4	phospholipase C, beta	0006629 /,	0005626 /,	0004435 // phosphoinositide phospholipase
-0.88414	Cd47	CD47 antigen (Rh-rela	0007155 /,	0005886 /,	0005515 // protein binding // inferred from
-0.14863	Ptar1	protein prenyltransferase alpha subunit repeat containing 1			
0.291054	Cpsf2	cleavage a mRNA_prc	0006397 /,	0005634 /,	0003723 // RNA binding // inferred from ele
-0.41016	1700018L0	RIKEN cDNA 1700018L02 gene			
0.199026	2810417H:	RIKEN cDNA 2810417H13 gene		0005634 // nucleus // inferred from electronic annotati	
-0.27559	Cml3	camello-like 3	0001702 /,	0005615 /,	0008080 // N-acetyltransferase activity // in
0.143573	Gsdma2	gasdermin A2			
-0.08565	5033425B0	RIKEN cDNA 5033425B01 gene			
-0.06901	4930563F1	RIKEN cDNA 4930563F15 gene			
0.050483	Adck2	aarF domain containir	0008152 /,	0005739 /,	0000166 // nucleotide binding // inferred fro
0.122461	Nat10	N-acetyltransferase 1(	0008152 /,	0005634 /,	0000166 // nucleotide binding // inferred fro
0.036006	Cdc27	cell division cycle 27 h	0007091 /,	0005634 /,	0005488 // binding // inferred from electror
-0.11542	Mtmr12	myotubularin related	0016311 /,	0005737 /,	0016791 // phosphatase activity // inferred
-0.08543	Heca	headcase homolog (Drosophila)			
-0.33572	Rprd2	regulation of nuclear pre-mRNA domain con			0005515 // protein binding // inferred from
-0.21557					
-0.26305	Gm129	predicted gene 129			
0.078381	Gramd1b	GRAM domain containing 1B		0016020 // membrane // inferred from electronic anno	
0.062713	AI452195	expressed sequence AI452195			
0.350942	D430042O	RIKEN cDNA D430042O09 gene			
-0.00342	Kremen1	kringle containing trai	0016055 /,	0016020 // membrane // inferred from sequence or str	
0.053182	Lman2l	lectin, mannose-bindi	0015031 /,	0000139 /,	0005529 // sugar binding // inferred from el
-0.1897	AW41377	expressed sequence AW413774			
0.271812	Dclk1	doublecortin-like kina	0001764 // neuron mi		0000166 // nucleotide binding // inferred fro
0.274441	Gsg1l	GSG1-like		0016021 // integral to membrane // inferred from elect	
-0.00265	Scn7a	sodium channel, volta	0006810 /,	0001518 /,	0005216 // ion channel activity // inferred fr
-0.1641	Lrrc15	leucine rich repeat containing 15		0005622 /,	0005515 // protein binding // inferred from
0.177365	Tmem212	transmembrane protein 212		0016020 // membrane // inferred from electronic anno	

0.134015	Dcaf5	DDB1 and CUL4 associated factor		0005739 // mitochondrion // inferred from direct assay	
-0.01623	6030405A1	RIKEN cDNA 6030405A18 gene		0016020 // membrane // inferred from electronic anno	
0.136804	Dand5	DAN domain family, m	0007368 /,	0005576 // extracellular region // inferred from electro	
0.23594					
-0.04487	Zbtb20	zinc finger and BTB dc	0006350 /,	0005622 /,	0003676 // nucleic acid binding // inferred fi
-0.12726	Susd5	sushi domain containing 5			
-0.25694	Lrrn4cl	LRRN4 C-terminal like		0016020 // membrane // inferred from electronic anno	
0.429508	Foxs1	forkhead box S1	0001503 /,	0005634 /,	0003677 // DNA binding // inferred from dir
0.188986	Dpy19l3	dpy-19-like 3 (C. elegans)		0016020 // membrane // inferred from electronic anno	
0.021691	Alg10b	asparagine-linked glyc	0008152 /,	0005886 /,	0015459 // potassium channel regulator acti
-0.01101	Pggt1b	protein geranylgerany	0008152 /,	0005953 /,	0003824 // catalytic activity // inferred from
-0.0658	C130046K2	RIKEN cDNA C130046K22 gene			
0.309763					
0.065625	Tbc1d24	TBC1 domain family, r	0032313 /,	0005622 /,	0005096 // GTPase activator activity // infer
-0.24349	Gtf3c1	general transcription	0006350 /,	0000127 /,	0003677 // DNA binding // inferred from ele
-0.04843	Socs2	suppressor of cytokin	0007595 // lactation /	0005131 // growth hormone receptor bindir	
-0.04832	Ado	2-aminoethanethiol (c	0055114 /,	0005739 /,	0016491 // oxidoreductase activity // inferre
-0.06687	Slc39a14	solute carrier family 3	0006810 /,	0005886 /,	0005385 // zinc ion transmembrane transpo
0.13522	Nif3l1	Ngg1 interacting facto	0045893 /,	0005737 /,	0005515 // protein binding // inferred from
-0.35491	Srsf11	serine/argi mRNA_processing_binding_Reac		0000166 // nucleotide binding // inferred fro	
-0.01895	9530010C2	RIKEN cDNA 9530010C24 gene			
0.004677	D630036G	RIKEN cDNA D630036G22 gene			
0.155324	Dot1l	DOT1-like, histone H3	0008152 // metabolic	0003677 // DNA binding // inferred from ele	
-0.1253	Rlf	rearranged L-myc fusion sequenc		0005622 /,	0003676 // nucleic acid binding // inferred fi
-0.11448	Adc	arginine decarboxylas	0006591 /,	0005739 /,	0003824 // catalytic activity // inferred from
0.07686	4930466K1	RIKEN cDNA 4930466K18 gene			
-0.12698					
-0.02263	Etl4	enhancer trap locus 4	0007275 /,	0005737 // cytoplasm // inferred from direct assay /// (	
0.065609	Fam184b	family with sequence similarity 184, member B			
0.133158	Aasdh	aminoadipate-semialc	0006629 // lipid meta	0000036 // acyl carrier activity // inferred fro	
-0.10888	Fam171a2	family with sequence similarity 1		0016020 // membrane // inferred from electronic anno	
-0.1195					
0.226688	Cdkn2aipn	CDKN2A interacting protein N-terminal like			
-0.02567	Gle1	GLE1 RNA export mec	0006810 /,	0005634 /,	0005515 // protein binding // inferred from
-0.26161	B230208H1	RIKEN cDNA B230208	0007264 /,	0005622 /,	0000166 // nucleotide binding // inferred fro
-0.27659	Nsd1	nuclear receptor-bind	0000122 /,	0005634 /,	0003682 // chromatin binding // inferred fro
0.060452	5930412G1	RIKEN cDNA 5930412G12 gene			
0.068484	Gabarapl2	gamma-aminobutyric	0006810 /,	0000139 /,	0005515 // protein binding // inferred from
-0.10988	Spg7	Spastic paraplegia 7 h	0006508 /,	0005739 /,	0000166 // nucleotide binding // inferred fro
-0.27451	Edn3	endothelin 3	0001755 /,	0005576 /,	0005179 // hormone activity // inferred fror
-0.23387	Edn3	endothelin 3	0001755 /,	0005576 /,	0005179 // hormone activity // inferred fror
-0.11239	Itih5	inter-alpha (globulin)	0010466 /,	0005576 /,	0004867 // serine-type endopeptidase inhib
0.17894	Lynx1	Ly6/neurotoxin 1	0007271 /,	0005624 /,	0005515 // protein binding // inferred from
0.086305	AI449212	expressed sequence AI449212			
0.070802	LOC10050	hypothetical LOC100503166			
0.202926	Zfp563	zinc finger protein 56	0006355 /,	0005622 /,	0003676 // nucleic acid binding // inferred fi
0.012148					

0.338418						
-0.20142	Rapgef6	Rap guanine nucleotic	0007165 /,	0005622 /,	0005085 // guanyl-nucleotide exchange fact	
-0.10448	Trip11	thyroid hormone rece	0007165 /,	0005634 /,	0004872 // receptor activity // inferred from	
-0.17275	Spnb2	spectrin beta 2	0007182 /,	0005634 /,	0003779 // actin binding // inferred from ele	
0.159814	1700025G(	RIKEN cDNA 1700025G04 gene				
-0.0115	D630045J1	RIKEN cDNA D630045J12 gene		0016020 // membrane // inferred from electronic anno		
-0.25769						
-0.08917	Atxn7l1	ataxin 7-like 1				
-0.21145						
-0.49553	Spg11	spastic paraplegia 11		0005634 // nucleus // inferred from electronic annotati		
-0.06311						
-0.0144	E230025N:	Riken cDNA E230025N	0007018 // microtubu		0003777 // microtubule motor activity // inf	
-0.2695						
-0.01625						
-0.03676						
-0.02848	Wdfy3	WD repeat and FYVE (	0008152 /,	0005635 /,	0003831 // beta-N-acetylglucosaminylglycop	
0.120925	Cux2	cut-like homeobox 2	0006350 /,	0005634 /,	0003677 // DNA binding // inferred from dir	
0.07615	Opa3	optic atrophy 3 (human)		0005739 // mitochondrion // inferred from direct assay		
-0.01262	Bat1a	HLA-B-ass(	mRNA_pro	0006200 /,	0005634 /,	0000166 // nucleotide binding // inferred fro
-0.18936	Fbxo7	F-box protein 7	0031647 /,	0043234 /,	0005515 // protein binding // inferred from	
-0.07598	Shpk	sedoheptulokinase	0005975 /,	0005737 /,	0000166 // nucleotide binding // inferred fro	
-0.18019	D19Ertd73	DNA segment, Chr 19, ERATO Doi 737, expressed				
-0.17639	Rnaseh2b	ribonuclease H2, subunit B		0005634 // nucleus // inferred from electronic annotati		
0.019008	3110082I1	RIKEN cDNA 3110082I17 gene			0005515 // protein binding // inferred from	
-0.10296	Zfand5	zinc finger, AN1-type (	0001701 // in utero e		0003677 // DNA binding // inferred from ele	
0.25355	C2cd4b	C2 calcium-dependent domain containing 4B				
0.087039	6430500C:	RIKEN cDNA 6430500C12 gene				
0.289974	Srr	serine racemase	0006520 /,	0005625 /,	0003824 // catalytic activity // inferred from	
-0.02835	2210408I2	RIKEN cDNA 2210408I21 gene				
0.092473	C030039L(	RIKEN cDNA C030039	0006355 /,	0005622 /,	0003676 // nucleic acid binding // inferred fr	
0.032607	Mrpl47	mitochondrial ribosor	0006412 /,	0005739 /,	0003735 // structural constituent of ribosom	
0.036687	4933402D:	RIKEN cDNA 4933402D24 gene				
-0.07978	1700040N(	RIKEN cDNA 1700040N02 gene				
0.042519	4631402F2	RIKEN cDNA 4631402F24 gene				
-0.18199	5830420C(	RIKEN cDNA 5830420C07 gene				
-0.13167						
-0.07525	Ilvbl	ilvB (bacterial acetola	0008152 /,	0016020 /,	0000287 // magnesium ion binding // inferre	
0.068025	Prkcz	protein kin	Calcium_re	0000226 /,	0005625 /,	0000166 // nucleotide binding // inferred fro
0.159271	Leo1	Leo1, Paf1/RNA polyn	0006350 /,	0005634 /,	0005515 // protein binding // inferred from	
0.134	Fam193a	family with sequence similarity 193, member A				
0.008543	Dgcr8	DiGeorge syndrome c	0007051 /,	0005622 /,	0003723 // RNA binding // inferred from ele	
-0.45031	Cggbp1	CGG triplet repeat bin	0006350 /,	0005634 /,	0003677 // DNA binding // inferred from ele	
-0.07839	Xkr8	X Kell blood group precursor related family member 8 homolog				
0.010088	Dpp9	dipeptidylpeptidase 9	0006508 /,	0005737 /,	0004177 // aminopeptidase activity // inferr	
0.001663	Nav3	neuron navigator 3	0008152 /,	0005634 /,	0000166 // nucleotide binding // inferred fro	
-0.44119	Lepr	leptin receptor	0001525 /,	0005576 /,	0004872 // receptor activity // inferred from	
0.297207	5430407P1	RIKEN cDNA 5430407P10 gene				

0.24175	Trim28	tripartite motif-contai	0000122 /,	0000785 /,	0003700 // sequence-specific DNA binding tr	
-0.37037	Mthfd1l	methylenetetrahydro	0006730 /,	0005739 /,	0000166 // nucleotide binding // inferred fro	
0.4926	Nsg2	neuron specific gene f	0007212 /,	0005768 /,	0050780 // dopamine receptor binding // inf	
0.287966	Cdh26	cadherin-like 26	0007155 /,	0005886 /,	0005509 // calcium ion binding // inferred fr	
-0.18275	Wnt5a	wingless-re	Wnt_Signa	0001667 /,	0005576 /,	0004871 // signal transducer activity // infer
-0.09784						
0.097733	Nkd2	naked cuticle 2 homol	0006810 /,	0005737 /,	0005509 // calcium ion binding // inferred fr	
-0.49694	Cdkl2	cyclin-dependent kina	0006468 /,	0005634 /,	0000166 // nucleotide binding // inferred fro	
-0.07238	2610316D(	RIKEN cDNA 2610316D01 gene				
0.138573	Osgep	O-sialoglycoprotein er	0006508 // proteolysi		0004175 // endopeptidase activity // inferre	
0.025903	Homer2	homer homolog 2 (Dr	0007216 /,	0005737 /,	0003779 // actin binding // inferred from dir	
-0.00709	Arntl	Aryl hydrocarbon rece	0000060 /,	0005634 /,	0003677 // DNA binding // inferred from phy	
-0.30057	Ascc3	activating signal coint	0008152 /,	0005622 /,	0003676 // nucleic acid binding // inferred fr	
-0.1824	Trpm3	transient receptor pot	0006810 /,	0016020 /,	0004872 // receptor activity // inferred from	
-0.22286	D7Wsu130	DNA segment, Chr 7, Wayne State University 130, expressed				
0.148494	Med6	mediator of RNA poly	0006350 /,	0005634 /,	0003677 // DNA binding // inferred from dir	
0.070636	Kcnq3	potassium voltage-gat	0006810 /,	0008076 /,	0005216 // ion channel activity // inferred fr	
0.117128	Wrb	tryptophan rich basic protein		0005634 // nucleus // inferred from electronic annotati		
-0.10226	4933407C(	RIKEN cDNA 4933407C09 gene				
0.151837	Ppip5k1	diphosphoinositol per	0006020 /,	0005737 /,	0000166 // nucleotide binding // inferred fro	
-0.46003	Hsd17b13	hydroxysteroid (17-be	0008152 /,	0005576 /,	0003824 // catalytic activity // inferred from	

ranscription factor activity // inferred from direct assay /// 0003824 // catalytic activity // inferred from electron
om electronic annotation /// 0003676 // nucleic acid binding // inferred from electronic annotation /// 0003677
electronic annotation /// 0004806 // triglyceride lipase activity // inferred from electronic annotation /// 0005
ty // not recorded /// 0004768 // stearoyl-CoA 9-desaturase activity // inferred from electronic annotation /// 0
rom electronic annotation /// 0003677 // DNA binding // inferred from electronic annotation /// 0005515 // pro
/// 0005178 // integrin binding // inferred from electronic annotation /// 0050839 // cell adhesion molecule bin
om sequence or structural similarity /// 0005515 // protein binding // inferred from electronic annotation
red from electronic annotation /// 0005097 // Rab GTPase activator activity // inferred from electronic annotati
ferred from electronic annotation
tation /// 0016021 // integral to membrane // inferred from electronic annotation
5764 // lysosome // inferred from direct assay /// 0005764 // lysosome // not recorded /// 0005764 // lysosome
physical interaction /// 0005515 // protein binding // inferred from electronic annotation /// 0016301 // kinase
electronic annotation /// 0015171 // amino acid transmembrane transporter activity // not recorded /// 00151
om electronic annotation /// 0000287 // magnesium ion binding // inferred from electronic annotation /// 0004
om electronic annotation /// 0000287 // magnesium ion binding // inferred from electronic annotation /// 0004
physical interaction
physical interaction /// 0005515 // protein binding // inferred from electronic annotation /// 0046982 // protei
05634 // nucleus // inferred from electronic annotation /// 0005730 // nucleolus // inferred from direct assay //
ect assay /// 0003677 // DNA binding // inferred from electronic annotation /// 0003700 // sequence-specific D
ect assay /// 0003677 // DNA binding // inferred from electronic annotation /// 0003700 // sequence-specific D
electronic annotation
tation /// 0016021 // integral to membrane // inferred from electronic annotation
rom electronic annotation /// 0031683 // G-protein beta/gamma-subunit binding // inferred from direct assay
om electronic annotation /// 0004666 // prostaglandin-endoperoxide synthase activity // inferred from electron
se activity // inferred from electronic annotation /// 0016787 // hydrolase activity // inferred from electronic an
m electronic annotation /// 0017110 // nucleoside-diphosphatase activity // inferred from electronic annotation
ecorded /// 0004177 // aminopeptidase activity // inferred from electronic annotation /// 0005515 // protein bi
ecorded /// 0004497 // monooxygenase activity // inferred from electronic annotation /// 0004499 // flavin-con
tation /// 0016021 // integral to membrane // inferred from electronic annotation
rom electronic annotation /// 0015293 // symporter activity // inferred from electronic annotation /// 0015377
ty // inferred from electronic annotation /// 0004843 // ubiquitin-specific protease activity // inferred from seq
om electronic annotation /// 0004674 // protein serine/threonine kinase activity // inferred from direct assay //
red from electronic annotation /// 0004872 // receptor activity // inferred from electronic annotation /// 0005

ı electronic annotation /// 0005160 // transforming growth factor beta receptor binding // inferred from electrc
rom electronic annotation /// 0046872 // metal ion binding // inferred from electronic annotation
ı electronic annotation /// 0004607 // phosphatidylcholine-sterol O-acyltransferase activity // inferred from ele
ı electronic annotation /// 0016787 // hydrolase activity // inferred from electronic annotation
ı electronic annotation /// 0008009 // chemokine activity // inferred from electronic annotation
rom electronic annotation /// 0005244 // voltage-gated ion channel activity // inferred from electronic annotati
om electronic annotation /// 0003713 // transcription coactivator activity // inferred from sequence or structura
physical interaction /// 0008270 // zinc ion binding // inferred from electronic annotation /// 0046872 // metal
physical interaction /// 0005515 // protein binding // inferred from electronic annotation /// 0046875 // ephrin
nic annotation
om electronic annotation /// 0003676 // nucleic acid binding // inferred from electronic annotation /// 0003723
m electronic annotation /// 0008234 // cysteine-type peptidase activity // inferred from electronic annotation /
sporter activity // inferred from electronic annotation /// 0015179 // L-amino acid transmembrane transporter
om electronic annotation /// 0004222 // metalloendopeptidase activity // not recorded /// 0004222 // metalloe
om electronic annotation /// 0003924 // GTPase activity // inferred from sequence or structural similarity /// 00
d from electronic annotation /// 0005515 // protein binding // inferred from physical interaction /// 0005515 //
nic annotation /// 0005578 // proteinaceous extracellular matrix // inferred from electronic annotation /// 000
om electronic annotation /// 0004672 // protein kinase activity // inferred from electronic annotation /// 00047
ctronic annotation /// 0031072 // heat shock protein binding // inferred from electronic annotation /// 004687
red from electronic annotation /// 0004872 // receptor activity // inferred from electronic annotation /// 0004
ctronic annotation /// 0003700 // sequence-specific DNA binding transcription factor activity // inferred from d
ctronic annotation /// 0003700 // sequence-specific DNA binding transcription factor activity // inferred from d
erred from electronic annotation /// 0016740 // transferase activity // inferred from electronic annotation /// 0
otation /// 0005737 // cytoplasm // inferred from electronic annotation /// 0005815 // microtubule organizing
om electronic annotation /// 0001948 // glycoprotein binding // not recorded /// 0004672 // protein kinase acti
ect assay /// 0003677 // DNA binding // not recorded /// 0003677 // DNA binding // inferred from electronic an
ctronic annotation /// 0003700 // sequence-specific DNA binding transcription factor activity // not recorded //
rom electronic annotation
ecorded /// 0004497 // monooxygenase activity // inferred from electronic annotation /// 0004509 // steroid 2
rom electronic annotation
ctronic annotation /// 0005789 // endoplasmic reticulum membrane // inferred from electronic annotation /// (
om electronic annotation /// 0000287 // magnesium ion binding // not recorded /// 0000287 // magnesium ion
nic annotation /// 0005794 // Golgi apparatus // inferred from electronic annotation
physical interaction /// 0005515 // protein binding // inferred from electronic annotation /// 0016455 // RNA p
electronic annotation
ding // not recorded /// 0001850 // complement component C3a binding // inferred from electronic annotatio
on /// 0005737 // cytoplasm // inferred from electronic annotation
inferred from genetic interaction /// 0005515 // protein binding // inferred from physical interaction /// 00055
ctronic annotation /// 0005200 // structural constituent of cytoskeleton // traceable author statement /// 000
ı electronic annotation /// 0004553 // hydrolase activity, hydrolyzing O-glycosyl compounds // inferred from ele

ronic annotation
ranscription factor activity // inferred from electronic annotation
om electronic annotation /// 0003676 // nucleic acid binding // inferred from electronic annotation /// 0003677
/ inferred from direct assay /// 0004842 // ubiquitin-protein ligase activity // inferred from genetic interaction /
0003677 // DNA binding // inferred from electronic annotation /// 0003700 // sequence-specific DNA binding t
on

red from electronic annotation /// 0005506 // iron ion binding // inferred from electronic annotation /// 00083
red from electronic annotation /// 0005506 // iron ion binding // inferred from electronic annotation /// 00083
red from electronic annotation /// 0005515 // protein binding // inferred from physical interaction
om electronic annotation /// 0004672 // protein kinase activity // inferred from direct assay /// 0004672 // prot
ivity // inferred from direct assay /// 0004190 // aspartic-type endopeptidase activity // inferred from electroni
electronic annotation
physical interaction /// 0005525 // GTP binding // inferred from electronic annotation
physical interaction /// 0004872 // receptor activity // inferred from electronic annotation /// 0005488 // bind
electronic annotation /// 0005515 // protein binding // inferred from electronic annotation /// 0042277 // pep
electronic annotation /// 0004252 // serine-type endopeptidase activity // inferred from electronic annotation
rom electronic annotation /// 0003677 // DNA binding // inferred from electronic annotation /// 0008270 // zin
electronic annotation /// 0004721 // phosphoprotein phosphatase activity // inferred from electronic annotati
electronic annotation /// 0005488 // binding // inferred from electronic annotation /// 0005529 // sugar bindi
electronic annotation /// 0004806 // triglyceride lipase activity // not recorded /// 0004806 // triglyceride lipas
ic annotation /// 0005515 // protein binding // inferred from electronic annotation
rom electronic annotation /// 0003677 // DNA binding // inferred from electronic annotation /// 0003700 // sec
physical interaction /// 0005515 // protein binding // inferred from electronic annotation
/ inferred from electronic annotation /// 0016740 // transferase activity // inferred from electronic annotation
ctronic annotation /// 0005515 // protein binding // inferred from electronic annotation
y // not recorded /// 0004128 // cytochrome-b5 reductase activity // inferred from electronic annotation /// 00
eceptor binding // not recorded /// 0005111 // type 2 fibroblast growth factor receptor binding // inferred from
om sequence or structural similarity /// 0016020 // membrane // inferred from electronic annotation /// 00160
om electronic annotation /// 0003676 // nucleic acid binding // inferred from electronic annotation /// 0003723
ctronic annotation /// 0003697 // single-stranded DNA binding // inferred from electronic annotation /// 0003
electronic annotation
ctronic annotation /// 0015485 // cholesterol binding // not recorded /// 0015485 // cholesterol binding // infe
ectronic annotation
electronic annotation
nstituent // inferred from electronic annotation /// 0005515 // protein binding // inferred from physical interac
electronic annotation /// 0031432 // titin binding // inferred from electronic annotation /// 0070080 // titin Z d
factor activity // inferred from electronic annotation /// 0005509 // calcium ion binding // inferred from electr
not recorded /// 0008489 // UDP-galactose:glucosylceramide beta-1,4-galactosyltransferase activity // not reco
red from electronic annotation /// 0005506 // iron ion binding // inferred from electronic annotation /// 00090
ctronic annotation /// 0003682 // chromatin binding // inferred from direct assay /// 0003700 // sequence-spe
om electronic annotation /// 0005515 // protein binding // inferred from physical interaction /// 0050840 // ex
om electronic annotation /// 0004666 // prostaglandin-endoperoxide synthase activity // inferred from electror
// inferred from direct assay /// 0000293 // ferric-chelate reductase activity // inferred from mutant phenotype
atidic acid receptor activity // inferred from electronic annotation /// 0004871 // signal transducer activity // in
/// 0005739 // mitochondrion // inferred from electronic annotation /// 0005743 // mitochondrial inner memb
om electronic annotation /// 0003676 // nucleic acid binding // inferred from electronic annotation /// 0003677
ing // inferred from electronic annotation /// 0004197 // cysteine-type endopeptidase activity // inferred from
om electronic annotation /// 0003824 // catalytic activity // inferred from electronic annotation /// 0004467 // l
nstituent // inferred from electronic annotation /// 0005201 // extracellular matrix structural constituent // inf
nic annotation /// 0016020 // membrane // inferred from electronic annotation /// 0016021 // integral to mem
ecorded /// 0001671 // ATPase activator activity // inferred from electronic annotation /// 0001671 // ATPase a
ctronic annotation /// 0003690 // double-stranded DNA binding // inferred from direct assay /// 0003700 // se
tation /// 0016021 // integral to membrane // inferred from electronic annotation /// 0030133 // transport ves

ctronic annotation /// 0003700 // sequence-specific DNA binding transcription factor activity // inferred from e
ctronic annotation /// 0003700 // sequence-specific DNA binding transcription factor activity // inferred from e

om electronic annotation /// 0004672 // protein kinase activity // inferred from electronic annotation /// 00046
electronic annotation /// 0004633 // phosphopantothenoylcysteine decarboxylase activity // inferred from ele
om electronic annotation /// 0003676 // nucleic acid binding // inferred from electronic annotation /// 0003723
nferred from electronic annotation /// 0008270 // zinc ion binding // inferred from electronic annotation /// 00
om electronic annotation /// 0000287 // magnesium ion binding // not recorded /// 0001948 // glycoprotein bir
electronic annotation /// 0008270 // zinc ion binding // inferred from electronic annotation /// 0046872 // met

rom electronic annotation /// 0008270 // zinc ion binding // inferred from electronic annotation
ysical interaction /// 0003677 // DNA binding // not recorded /// 0003677 // DNA binding // inferred from elect
ic annotation /// 0005515 // protein binding // inferred from electronic annotation

ferred from electronic annotation

electronic annotation /// 0004553 // hydrolase activity, hydrolyzing O-glycosyl compounds // inferred from ele
tivity // inferred from direct assay /// 0004721 // phosphoprotein phosphatase activity // inferred from electror
ded /// 0005509 // calcium ion binding // inferred from electronic annotation
ement /// 0005886 // plasma membrane // inferred from electronic annotation /// 0016020 // membrane // inf
inhibitor activity // inferred from electronic annotation /// 0004861 // cyclin-dependent protein kinase inhibito
electronic annotation /// 0004114 // 3',5'-cyclic-nucleotide phosphodiesterase activity // inferred from electro
rom electronic annotation /// 0005244 // voltage-gated ion channel activity // inferred from electronic annotati
electronic annotation /// 0005488 // binding // inferred from electronic annotation /// 0005529 // sugar bindi
tation /// 0016020 // membrane // inferred from electronic annotation /// 0031902 // late endosome membrar
annotation
rom electronic annotation /// 0005515 // protein binding // inferred from electronic annotation /// 0008270 //
ation /// 0005783 // endoplasmic reticulum // inferred from electronic annotation /// 0005815 // microtubule
om electronic annotation /// 0003824 // catalytic activity // inferred from electronic annotation /// 0005102 //
ect assay /// 0003677 // DNA binding // inferred from electronic annotation /// 0003700 // sequence-specific D
electronic annotation /// 0008270 // zinc ion binding // inferred from electronic annotation

inferred from electronic annotation /// 0008233 // peptidase activity // inferred from electronic annotation //
ferred from electronic annotation
electronic annotation
physical interaction /// 0016455 // RNA polymerase II transcription mediator activity // inferred from electronic
d from electronic annotation /// 0005524 // ATP binding // inferred from electronic annotation
om electronic annotation /// 0003774 // motor activity // inferred from electronic annotation /// 0003779 // ac
/ inferred from direct assay /// 0005515 // protein binding // inferred from physical interaction

electronic annotation
m electronic annotation /// 0008236 // serine-type peptidase activity // inferred from electronic annotation ///
rom electronic annotation /// 0003677 // DNA binding // inferred from electronic annotation /// 0008270 // zin
e // inferred from electronic annotation
activity // not recorded /// 0004044 // amidophosphoribosyltransferase activity // inferred from electronic an
electronic annotation
om electronic annotation /// 0003676 // nucleic acid binding // inferred from electronic annotation /// 0003723

// not recorded /// 0005112 // Notch binding // inferred from electronic annotation /// 0005515 // protein bin

)005737 // cytoplasm // inferred from electronic annotation
:ation
om electronic annotation /// 0004017 // adenylate kinase activity // not recorded /// 0004017 // adenylate kina
d from electronic annotation /// 0005529 // sugar binding // not recorded /// 0005529 // sugar binding // inferr
om electronic annotation /// 0003924 // GTPase activity // inferred from electronic annotation /// 0005515 // p
rom electronic annotation
physical interaction
:ctronic annotation /// 0005515 // protein binding // inferred from physical interaction /// 0008270 // zinc ion b
ect assay /// 0003682 // chromatin binding // inferred from direct assay /// 0003714 // transcription corepresso
ctronic annotation /// 0005524 // ATP binding // inferred from electronic annotation /// 0008270 // zinc ion bir
red from sequence or structural similarity /// 0004177 // aminopeptidase activity // inferred from electronic anr
red from electronic annotation /// 0051087 // chaperone binding // inferred from electronic annotation
rom electronic annotation /// 0005515 // protein binding // inferred from electronic annotation /// 0008270 //
:ctronic annotation /// 0003714 // transcription corepressor activity // inferred from direct assay /// 0003714 //
iic annotation
erred from electronic annotation /// 0051082 // unfolded protein binding // inferred from electronic annotatior
notation

electronic annotation
:ctronic annotation /// 0005515 // protein binding // inferred from electronic annotation /// 0005515 // proteir
activity // not recorded /// 0004029 // aldehyde dehydrogenase (NAD) activity // inferred from electronic anno
ie // not recorded /// 0003735 // structural constituent of ribosome // inferred from electronic annotation /// (
:ctronic annotation /// 0003690 // double-stranded DNA binding // inferred from direct assay /// 0003700 // se
tation /// 0016021 // integral to membrane // inferred from electronic annotation
electronic annotation
ctronic annotation
ctronic annotation /// 0003730 // mRNA 3'-UTR binding // inferred from electronic annotation /// 0003730 // m
ect assay /// 0003677 // DNA binding // inferred from electronic annotation /// 0003700 // sequence-specific D

activity // inferred from electronic annotation /// 0004043 // L-aminoadipate-semialdehyde dehydrogenase act
om electronic annotation /// 0005515 // protein binding // inferred from electronic annotation /// 0005524 // A
physical interaction
ect assay /// 0003677 // DNA binding // inferred from electronic annotation /// 0003700 // sequence-specific D
om electronic annotation /// 0000287 // magnesium ion binding // inferred from electronic annotation /// 0004
physical interaction /// 0005515 // protein binding // inferred from electronic annotation /// 0046982 // protei
atidic acid receptor activity // inferred from direct assay /// 0001619 // lysosphingolipid and lysophosphatidic a
ect assay /// 0003677 // DNA binding // inferred from physical interaction /// 0003677 // DNA binding // inferre
/ inferred from electronic annotation /// 0004843 // ubiquitin-specific protease activity // inferred from sequen
om electronic annotation /// 0003774 // motor activity // inferred from electronic annotation /// 0003779 // ac
ect assay /// 0003677 // DNA binding // inferred from electronic annotation /// 0005515 // protein binding // ir
rom electronic annotation /// 0008028 // monocarboxylic acid transmembrane transporter activity // not recorc
i electronic annotation
tation /// 0016021 // integral to membrane // inferred from electronic annotation
electronic annotation
rom electronic annotation /// 0008270 // zinc ion binding // inferred from electronic annotation /// 0046872 //

:eceptor binding // inferred from direct assay /// 0005161 // platelet-derived growth factor receptor binding // ir
nic annotation /// 0005923 // tight junction // inferred from electronic annotation /// 0016020 // membrane //
nding // inferred from electronic annotation /// 0004872 // receptor activity // inferred from electronic annotati
rom electronic annotation /// 0005515 // protein binding // inferred from electronic annotation /// 0005516 //
ferred from electronic annotation /// 0030247 // polysaccharide binding // inferred from electronic annotation
om electronic annotation /// 0004672 // protein kinase activity // inferred from electronic annotation /// 00047
electronic annotation /// 0016301 // kinase activity // inferred from direct assay
rred from electronic annotation /// 0005515 // protein binding // inferred from physical interaction
om electronic annotation /// 0003676 // nucleic acid binding // inferred from electronic annotation /// 0003723
rom electronic annotation /// 0004518 // nuclease activity // inferred from electronic annotation /// 0004527 /
nstituent // inferred from electronic annotation /// 0046332 // SMAD binding // inferred from physical interact
tivity // inferred from electronic annotation /// 0005515 // protein binding // inferred from electronic annotatio
nic annotation /// 0034364 // high-density lipoprotein particle // inferred from electronic annotation
0003700 // sequence-specific DNA binding transcription factor activity // not recorded /// 0003723 // RNA bind
om electronic annotation /// 0004672 // protein kinase activity // not recorded /// 0004672 // protein kinase ac
electronic annotation /// 0030165 // PDZ domain binding // inferred from electronic annotation /// 0031698 //
nic annotation /// 0016881 // acid-amino acid ligase activity // inferred from electronic annotation
tation
electronic annotation
electronic annotation
physical interaction /// 0005515 // protein binding // inferred from electronic annotation /// 0046875 // ephrin
ctronic annotation /// 0003899 // DNA-directed RNA polymerase activity // inferred from electronic annotation
tation /// 0016021 // integral to membrane // inferred from electronic annotation
nferred from electronic annotation
ctronic annotation /// 0005523 // tropomyosin binding // inferred from electronic annotation
physical interaction /// 0042802 // identical protein binding // inferred from direct assay
electronic annotation /// 0008270 // zinc ion binding // inferred from electronic annotation /// 0046872 // met

C activity // inferred from electronic annotation /// 0004629 // phospholipase C activity // not recorded /// 000
d from electronic annotation /// 0004672 // protein kinase activity // inferred from sequence or structural simila

ect assay /// 0003677 // DNA binding // inferred from electronic annotation /// 0008270 // zinc ion binding // ir
ctronic annotation /// 0003700 // sequence-specific DNA binding transcription factor activity // inferred from e
NA binding // inferred from direct assay /// 0003677 // DNA binding // inferred from direct assay /// 0003677 /
om sequence or structural similarity /// 0005515 // protein binding // inferred from electronic annotation
on
om electronic annotation /// 0003924 // GTPase activity // inferred from electronic annotation /// 0005525 // G
ne // not recorded /// 0003735 // structural constituent of ribosome // inferred from electronic annotation
ty // inferred from electronic annotation

electronic annotation
itor activity // inferred from electronic annotation /// 0005515 // protein binding // inferred from physical inter

ect assay /// 0003677 // DNA binding // inferred from electronic annotation /// 0003700 // sequence-specific D
electronic annotation /// 0004758 // serine C-palmitoyltransferase activity // inferred from direct assay /// 000
rred from electronic annotation /// 0004872 // receptor activity // inferred from electronic annotation /// 0004
om electronic annotation /// 0003677 // DNA binding // inferred from electronic annotation /// 0003916 // DNA

ɔm electronic annotation /// 0004672 // protein kinase activity // inferred from electronic annotation /// 00047
ctronic annotation /// 0009982 // pseudouridine synthase activity // inferred from electronic annotation /// 00
tation /// 0016021 // integral to membrane // inferred from electronic annotation
/// 0005739 // mitochondrion // inferred from electronic annotation /// 0005743 // mitochondrial inner memb
0003723 // RNA binding // inferred from electronic annotation /// 0003730 // mRNA 3'-UTR binding // not reco
l from direct assay /// 0004527 // exonuclease activity // inferred from direct assay /// 0008198 // ferrous iron l
ı electronic annotation /// 0005496 // steroid binding // inferred from electronic annotation /// 0008289 // lipic
lectronic annotation /// 0005515 // protein binding // inferred from physical interaction
inhibitor activity // inferred from electronic annotation /// 0005515 // protein binding // inferred from electror
ivity // inferred from electronic annotation
ɔm electronic annotation /// 0004672 // protein kinase activity // not recorded /// 0004672 // protein kinase ac
/// 0005739 // mitochondrion // inferred from electronic annotation /// 0016020 // membrane // inferred fror
electronic annotation
ctronic annotation /// 0003700 // sequence-specific DNA binding transcription factor activity // traceable autho
red from electronic annotation /// 0005506 // iron ion binding // inferred from electronic annotation /// 00090
ed from electronic annotation /// 0000287 // magnesium ion binding // inferred from sequence or structural sin
on /// 0005739 // mitochondrion // inferred from electronic annotation

C activity // inferred from electronic annotation /// 0004629 // phospholipase C activity // inferred from electr
physical interaction /// 0005515 // protein binding // inferred from electronic annotation /// 0070053 // throm

ctronic annotation /// 0005515 // protein binding // inferred from electronic annotation /// 0016787 // hydrola

on /// 0005739 // mitochondrion // inferred from electronic annotation
ferred from electronic annotation /// 0008080 // N-acetyltransferase activity // non-traceable author statemen

ɔm electronic annotation /// 0004674 // protein serine/threonine kinase activity // inferred from electronic ann
ɔm electronic annotation /// 0005515 // protein binding // inferred from electronic annotation /// 0005524 // A
ic annotation
from electronic annotation

electronic annotation

tation /// 0016021 // integral to membrane // inferred from electronic annotation

uctural similarity /// 0016020 // membrane // inferred from electronic annotation /// 0016021 // integral to me
ectronic annotation

ɔm electronic annotation /// 0004672 // protein kinase activity // not recorded /// 0004672 // protein kinase ac
ronic annotation
rom electronic annotation /// 0005244 // voltage-gated ion channel activity // inferred from electronic annotati
electronic annotation
tation /// 0016021 // integral to membrane // inferred from electronic annotation

/// 0080008 // CUL4 RING ubiquitin ligase complex // inferred from electronic annotation /// 0080008 // CUL4
tation /// 0016021 // integral to membrane // inferred from electronic annotation
nic annotation

rom electronic annotation /// 0003677 // DNA binding // inferred from direct assay /// 0003677 // DNA binding

tation /// 0016021 // integral to membrane // inferred from electronic annotation
ect assay /// 0003677 // DNA binding // inferred from electronic annotation /// 0003690 // double-stranded DN
tation /// 0016021 // integral to membrane // inferred from electronic annotation
ivity // not recorded /// 0015459 // potassium channel regulator activity // inferred from electronic annotation
electronic annotation /// 0004659 // prenyltransferase activity // inferred from electronic annotation /// 0004

red from electronic annotation /// 0005097 // Rab GTPase activator activity // inferred from electronic annotati
ctronic annotation /// 0003709 // RNA polymerase III transcription factor activity // not recorded /// 0003709 /
ng // inferred from physical interaction /// 0005159 // insulin-like growth factor receptor binding // inferred fron
ed from electronic annotation /// 0016702 // oxidoreductase activity, acting on single donors with incorporation
rter activity // inferred from direct assay /// 0015093 // ferrous iron transmembrane transporter activity // infe
electronic annotation /// 0008134 // transcription factor binding // inferred from electronic annotation
om electronic annotation /// 0003676 // nucleic acid binding // inferred from electronic annotation

ctronic annotation /// 0005515 // protein binding // inferred from electronic annotation /// 0008168 // methyl
rom electronic annotation /// 0005515 // protein binding // inferred from electronic annotation /// 0008270 //
electronic annotation /// 0004586 // ornithine decarboxylase activity // inferred from direct assay /// 0005515

0005737 // cytoplasm // inferred from electronic annotation

om electronic annotation /// 0000166 // nucleotide binding // inferred from electronic annotation /// 0003824
tation /// 0016021 // integral to membrane // inferred from electronic annotation

electronic annotation
om electronic annotation /// 0005525 // GTP binding // inferred from electronic annotation
om direct assay /// 0003712 // transcription cofactor activity // inferred from direct assay /// 0003712 // transcr

physical interaction /// 0005515 // protein binding // inferred from electronic annotation
om electronic annotation /// 0004222 // metalloendopeptidase activity // inferred from electronic annotation /
n electronic annotation /// 0031708 // endothelin B receptor binding // inferred from electronic annotation
n electronic annotation /// 0031708 // endothelin B receptor binding // inferred from electronic annotation
itor activity // inferred from electronic annotation /// 0030414 // peptidase inhibitor activity // inferred from el
physical interaction /// 0008200 // ion channel inhibitor activity // inferred from direct assay /// 0030550 // ace

rom electronic annotation /// 0008270 // zinc ion binding // inferred from electronic annotation /// 0046872 //

or activity // inferred from electronic annotation /// 0005515 // protein binding // inferred from electronic ann
electronic annotation /// 0005515 // protein binding // inferred from electronic annotation
ctronic annotation /// 0005200 // structural constituent of cytoskeleton // inferred from electronic annotation

tation /// 0016021 // integral to membrane // inferred from electronic annotation

on /// 0005737 // cytoplasm // inferred from electronic annotation /// 0005829 // cytosol // inferred from elec

erred from electronic annotation /// 0005524 // ATP binding // inferred from electronic annotation

peptide beta-1,4-galactosyltransferase activity // inferred from direct assay /// 0005488 // binding // inferred fr
ect assay /// 0003677 // DNA binding // inferred from electronic annotation /// 0003700 // sequence-specific D
/// 0005739 // mitochondrion // inferred from electronic annotation
om electronic annotation /// 0003676 // nucleic acid binding // inferred from electronic annotation /// 0003723
electronic annotation
om electronic annotation /// 0005524 // ATP binding // inferred from electronic annotation /// 0016301 // kina

on
electronic annotation
ctronic annotation /// 0008270 // zinc ion binding // inferred from electronic annotation /// 0046872 // metal i

electronic annotation /// 0003941 // L-serine ammonia-lyase activity // inferred from electronic annotation ///

rom electronic annotation /// 0008270 // zinc ion binding // inferred from electronic annotation /// 0046872 //
ne // inferred from electronic annotation

ed from electronic annotation /// 0003824 // catalytic activity // inferred from electronic annotation /// 001674
om electronic annotation /// 0004672 // protein kinase activity // inferred from direct assay /// 0004672 // prot
electronic annotation

ctronic annotation /// 0003725 // double-stranded RNA binding // inferred from electronic annotation /// 0005
ctronic annotation /// 0003690 // double-stranded DNA binding // not recorded

ed from electronic annotation /// 0008233 // peptidase activity // inferred from electronic annotation /// 0008
om electronic annotation /// 0005524 // ATP binding // inferred from electronic annotation /// 0017111 // nucl
electronic annotation /// 0004888 // transmembrane receptor activity // traceable author statement /// 0004

ranscription factor activity // inferred from direct assay /// 0003713 // transcription coactivator activity // inferr
om electronic annotation /// 0003824 // catalytic activity // inferred from electronic annotation /// 0004329 // f
ferred from electronic annotation
rom electronic annotation
rred from electronic annotation /// 0005102 // receptor binding // inferred from physical interaction /// 000510

rom electronic annotation /// 0005515 // protein binding // inferred from electronic annotation
om electronic annotation /// 0004672 // protein kinase activity // inferred from electronic annotation /// 00046

d from electronic annotation /// 0004222 // metalloendopeptidase activity // inferred from electronic annotati
rect assay /// 0003824 // catalytic activity // inferred from electronic annotation /// 0004601 // peroxidase activ
ysical interaction /// 0003677 // DNA binding // not recorded /// 0003677 // DNA binding // inferred from elect
rom electronic annotation /// 0004386 // helicase activity // inferred from electronic annotation /// 0005524 //
n electronic annotation /// 0005216 // ion channel activity // inferred from electronic annotation /// 0005261 //

ect assay /// 0003713 // transcription coactivator activity // not recorded /// 0005515 // protein binding // infe
rom electronic annotation /// 0005244 // voltage-gated ion channel activity // inferred from electronic annotati
on /// 0005730 // nucleolus // inferred from electronic annotation /// 0016020 // membrane // inferred from e

om electronic annotation /// 0000827 // inositol 1,3,4,5,6-pentakisphosphate kinase activity // inferred from ele
n electronic annotation /// 0005488 // binding // inferred from electronic annotation /// 0016491 // oxidoreduct

nic annotation /// 0005488 // binding // inferred from electronic annotation /// 0005515 // protein binding // ir
' // DNA binding // inferred from electronic annotation /// 0003723 // RNA binding // inferred from direct assay
509 // calcium ion binding // inferred from sequence or structural similarity /// 0005515 // protein binding // in
0005506 // iron ion binding // inferred from electronic annotation /// 0016491 // oxidoreductase activity // infer
otein binding // inferred from physical interaction
nding // inferred from electronic annotation

ion /// 0005097 // Rab GTPase activator activity // inferred from direct assay

e // inferred from electronic annotation /// 0005765 // lysosomal membrane // not recorded /// 0005765 // lys
activity // inferred from direct assay /// 0016538 // cyclin-dependent protein kinase regulator activity // inferre
75 // neutral amino acid transmembrane transporter activity // inferred from direct assay /// 0015194 // L-serir
672 // protein kinase activity // inferred from electronic annotation /// 0004674 // protein serine/threonine kir
672 // protein kinase activity // inferred from electronic annotation /// 0004674 // protein serine/threonine kir

n heterodimerization activity // not recorded /// 0046982 // protein heterodimerization activity // inferred from
'/ 0005737 // cytoplasm // inferred from electronic annotation
NA binding transcription factor activity // inferred from direct assay /// 0003700 // sequence-specific DNA bind
NA binding transcription factor activity // inferred from direct assay /// 0003700 // sequence-specific DNA bind

nic annotation /// 0005515 // protein binding // inferred from electronic annotation /// 0008289 // lipid binding
notation /// 0016798 // hydrolase activity, acting on glycosyl bonds // inferred from electronic annotation
n
nding // inferred from electronic annotation /// 0008233 // peptidase activity // inferred from electronic annot
ntaining monooxygenase activity // not recorded /// 0004499 // flavin-containing monooxygenase activity // inf

// cation:chloride symporter activity // inferred from electronic annotation
uence or structural similarity /// 0005515 // protein binding // inferred from electronic annotation /// 0008233
'/ 0004674 // protein serine/threonine kinase activity // not recorded /// 0004674 // protein serine/threonine k
515 // protein binding // inferred from physical interaction /// 0005515 // protein binding // inferred from elect

onic annotation /// 0008083 // growth factor activity // inferred from electronic annotation

ctronic annotation /// 0004772 // sterol O-acyltransferase activity // inferred from direct assay /// 0004772 // s

on /// 0005249 // voltage-gated potassium channel activity // not recorded /// 0005249 // voltage-gated potass
al similarity /// 0004842 // ubiquitin-protein ligase activity // inferred from electronic annotation /// 0004842 //
ion binding // inferred from electronic annotation
receptor binding // inferred from physical interaction

// RNA binding // inferred from electronic annotation
// 0016787 // hydrolase activity // inferred from electronic annotation /// 0016790 // thiolester hydrolase activ
activity // inferred from direct assay
endopeptidase activity // inferred from electronic annotation /// 0005524 // ATP binding // inferred from electr
03924 // GTPase activity // inferred from electronic annotation /// 0005525 // GTP binding // inferred from ele
protein binding // inferred from electronic annotation /// 0032403 // protein complex binding // not recorded
5604 // basement membrane // inferred from electronic annotation
13 // protein tyrosine kinase activity // not recorded /// 0004713 // protein tyrosine kinase activity // inferred f
2 // metal ion binding // inferred from electronic annotation /// 0051082 // unfolded protein binding // inferred
930 // G-protein coupled receptor activity // inferred from electronic annotation /// 0005000 // vasopressin rec
irect assay /// 0003700 // sequence-specific DNA binding transcription factor activity // not recorded /// 00037
irect assay /// 0003700 // sequence-specific DNA binding transcription factor activity // not recorded /// 00037
030748 // amine N-methyltransferase activity // inferred from electronic annotation
center // inferred from electronic annotation /// 0005819 // spindle // inferred from electronic annotation /// 0
vity // inferred from direct assay /// 0004672 // protein kinase activity // inferred from electronic annotation //
notation /// 0003700 // sequence-specific DNA binding transcription factor activity // inferred from electronic a
/ 0003700 // sequence-specific DNA binding transcription factor activity // inferred from electronic annotation

1-monooxygenase activity // not recorded /// 0004509 // steroid 21-monooxygenase activity // inferred from el

0016020 // membrane // inferred from electronic annotation /// 0016021 // integral to membrane // inferred fr
binding // inferred from electronic annotation /// 0004340 // glucokinase activity // inferred from direct assay ,

olymerase II transcription mediator activity // inferred from electronic annotation

n /// 0004871 // signal transducer activity // inferred from electronic annotation /// 0004872 // receptor activit

15 // protein binding // inferred from electronic annotation
5515 // protein binding // inferred from physical interaction
ctronic annotation /// 0004563 // beta-N-acetylhexosaminidase activity // inferred from direct assay /// 00045

// DNA binding // inferred from electronic annotation /// 0003682 // chromatin binding // inferred from direct
/// 0004842 // ubiquitin-protein ligase activity // inferred from electronic annotation /// 0005515 // protein bin
ranscription factor activity // inferred from electronic annotation /// 0004871 // signal transducer activity // inf

96 // oxysterol 7-alpha-hydroxylase activity // inferred from direct assay /// 0009055 // electron carrier activity /
96 // oxysterol 7-alpha-hydroxylase activity // inferred from direct assay /// 0009055 // electron carrier activity /

tein kinase activity // inferred from electronic annotation /// 0004674 // protein serine/threonine kinase activity
c annotation /// 0008233 // peptidase activity // inferred from electronic annotation /// 0016787 // hydrolase a

ling // inferred from electronic annotation /// 0005515 // protein binding // inferred from physical interaction /
otide binding // not recorded
/// 0008233 // peptidase activity // inferred from electronic annotation /// 0008236 // serine-type peptidase a
c ion binding // inferred from electronic annotation /// 0046872 // metal ion binding // inferred from electronic
on /// 0004725 // protein tyrosine phosphatase activity // inferred from direct assay /// 0004725 // protein tyr
ng // inferred from electronic annotation
se activity // inferred from electronic annotation /// 0016298 // lipase activity // not recorded /// 0016298 // lip

quence-specific DNA binding transcription factor activity // traceable author statement /// 0008270 // zinc ion b

016208 // AMP binding // not recorded /// 0016491 // oxidoreductase activity // inferred from electronic annota
electronic annotation /// 0005515 // protein binding // inferred from physical interaction /// 0008083 // growt
21 // integral to membrane // inferred from electronic annotation
// RNA binding // inferred from electronic annotation /// 0005515 // protein binding // inferred from electroni
713 // transcription coactivator activity // inferred from electronic annotation /// 0005488 // binding // inferred

rred from electronic annotation /// 0017127 // cholesterol transporter activity // inferred from electronic anno

ction /// 0005515 // protein binding // inferred from electronic annotation /// 0030674 // protein binding, bridg
omain binding // inferred from electronic annotation
onic annotation /// 0005515 // protein binding // inferred from physical interaction /// 0005515 // protein bind
rded /// 0008489 // UDP-galactose:glucosylceramide beta-1,4-galactosyltransferase activity // inferred from ele
55 // electron carrier activity // inferred from electronic annotation /// 0016491 // oxidoreductase activity // inf
cific DNA binding transcription factor activity // inferred from direct assay /// 0003700 // sequence-specific DN
tracellular matrix binding // inferred from direct assay
ic annotation /// 0008289 // lipid binding // not recorded /// 0008289 // lipid binding // inferred from electron
/// 0003824 // catalytic activity // inferred from electronic annotation /// 0005488 // binding // inferred from e
ferred from electronic annotation /// 0004872 // receptor activity // inferred from electronic annotation /// 000
brane // inferred from direct assay /// 0005743 // mitochondrial inner membrane // inferred from electronic an
// DNA binding // inferred from electronic annotation /// 0003697 // single-stranded DNA binding // inferred f
electronic annotation /// 0004197 // cysteine-type endopeptidase activity // inferred from sequence or structu
long-chain fatty acid-CoA ligase activity // not recorded /// 0004467 // long-chain fatty acid-CoA ligase activity /
erred from sequence or structural similarity /// 0005515 // protein binding // inferred from physical interaction
brane // inferred from electronic annotation
ctivator activity // inferred from sequence or structural similarity /// 0005515 // protein binding // inferred fro
quence-specific DNA binding transcription factor activity // inferred from electronic annotation /// 0005515 // p
icle // inferred from electronic annotation

lectronic annotation /// 0005515 // protein binding // inferred from electronic annotation /// 0030528 // transc
lectronic annotation /// 0005515 // protein binding // inferred from electronic annotation /// 0030528 // transc

674 // protein serine/threonine kinase activity // inferred from sequence or structural similarity /// 0004674 // p
ctronic annotation /// 0016829 // lyase activity // inferred from electronic annotation /// 0016831 // carboxy-ly
3 // RNA binding // inferred from electronic annotation /// 0004386 // helicase activity // inferred from electron
46872 // metal ion binding // inferred from electronic annotation
nding // not recorded /// 0004672 // protein kinase activity // inferred from electronic annotation /// 0004674 /
al ion binding // inferred from electronic annotation

ronic annotation /// 0003700 // sequence-specific DNA binding transcription factor activity // inferred from dire

ctronic annotation /// 0004563 // beta-N-acetylhexosaminidase activity // inferred from direct assay /// 00045
nic annotation /// 0004725 // protein tyrosine phosphatase activity // inferred from electronic annotation /// 00

erred from electronic annotation /// 0031225 // anchored to membrane // inferred from electronic annotation
r activity // inferred from sequence or structural similarity /// 0005515 // protein binding // inferred from elect
nic annotation /// 0004115 // 3',5'-cyclic-AMP phosphodiesterase activity // not recorded /// 0004115 // 3',5'-c
on /// 0005249 // voltage-gated potassium channel activity // inferred from electronic annotation /// 0005267 /
ng // inferred from electronic annotation
ne // inferred from electronic annotation

zinc ion binding // inferred from electronic annotation /// 0046872 // metal ion binding // inferred from electro
organizing center // inferred from electronic annotation /// 0005856 // cytoskeleton // inferred from electronic
receptor binding // not recorded /// 0005524 // ATP binding // inferred from electronic annotation /// 0008092
NA binding transcription factor activity // inferred from direct assay /// 0003700 // sequence-specific DNA bind

/ 0008237 // metallopeptidase activity // inferred from electronic annotation /// 0008270 // zinc ion binding //

c annotation

tin binding // inferred from electronic annotation /// 0005515 // protein binding // inferred from electronic ann

0016787 // hydrolase activity // inferred from electronic annotation
c ion binding // inferred from electronic annotation /// 0030528 // transcription regulator activity // inferred fro

notation /// 0016740 // transferase activity // inferred from electronic annotation /// 0016757 // transferase ac

3 // RNA binding // inferred from electronic annotation /// 0004386 // helicase activity // inferred from electron

ding // inferred from physical interaction /// 0005515 // protein binding // inferred from electronic annotation

se activity // traceable author statement /// 0004017 // adenylate kinase activity // inferred from electronic an
red from electronic annotation /// 0008144 // drug binding // not recorded /// 0008144 // drug binding // inferi
rotein binding // inferred from physical interaction /// 0005515 // protein binding // inferred from electronic ar

inding // inferred from electronic annotation
r activity // inferred from mutant phenotype /// 0005515 // protein binding // inferred from physical interactic
ding // inferred from electronic annotation /// 0016740 // transferase activity // inferred from electronic annot
otation /// 0005488 // binding // inferred from electronic annotation /// 0008233 // peptidase activity // inferr

zinc ion binding // inferred from electronic annotation /// 0046872 // metal ion binding // inferred from electro
transcription corepressor activity // not recorded /// 0003714 // transcription corepressor activity // inferred f

n

binding // inferred from physical interaction /// 0005516 // calmodulin binding // inferred from electronic ann
tation /// 0016491 // oxidoreductase activity // inferred from electronic annotation /// 0016620 // oxidoreduct
008270 // zinc ion binding // not recorded /// 0046872 // metal ion binding // inferred from electronic annotat
quence-specific DNA binding transcription factor activity // inferred from electronic annotation /// 0005515 // p

nRNA 3'-UTR binding // inferred from sequence or structural similarity /// 0005515 // protein binding // inferred
NA binding transcription factor activity // inferred from direct assay /// 0003700 // sequence-specific DNA bind

ivity // inferred from electronic annotation /// 0008802 // betaine-aldehyde dehydrogenase activity // inferred
TP binding // inferred from electronic annotation /// 0051082 // unfolded protein binding // inferred from elec

NA binding transcription factor activity // inferred from direct assay /// 0005515 // protein binding // inferred f
672 // protein kinase activity // inferred from electronic annotation /// 0004713 // protein tyrosine kinase activ
n heterodimerization activity // not recorded /// 0046982 // protein heterodimerization activity // inferred from
cid receptor activity // not recorded /// 0001619 // lysosphingolipid and lysophosphatidic acid receptor activity
d from electronic annotation /// 0003700 // sequence-specific DNA binding transcription factor activity // infer
ce or structural similarity /// 0008233 // peptidase activity // inferred from electronic annotation /// 0008234 /
tin binding // inferred from electronic annotation /// 0005515 // protein binding // inferred from electronic ann
nferred from physical interaction /// 0030528 // transcription regulator activity // inferred from electronic anno
led /// 0008028 // monocarboxylic acid transmembrane transporter activity // inferred from electronic annotat

metal ion binding // inferred from electronic annotation

ıferred from electronic annotation /// 0008083 // growth factor activity // inferred from electronic annotation /
inferred from electronic annotation /// 0016021 // integral to membrane // inferred from electronic annotatior
ion /// 0005488 // binding // inferred from electronic annotation /// 0005509 // calcium ion binding // inferred
calmodulin binding // inferred from direct assay /// 0005516 // calmodulin binding // inferred from electronic a

'13 // protein tyrosine kinase activity // inferred from electronic annotation /// 0004714 // transmembrane rece

; // RNA binding // inferred from electronic annotation
/ exonuclease activity // inferred from electronic annotation /// 0004527 // exonuclease activity // inferred fror
ion
n /// 0008233 // peptidase activity // inferred from electronic annotation /// 0008234 // cysteine-type peptidas

ling // inferred from electronic annotation
:tivity // inferred from electronic annotation /// 0004674 // protein serine/threonine kinase activity // inferred f
beta-2 adrenergic receptor binding // inferred from electronic annotation

ı receptor binding // inferred from physical interaction
ı /// 0005515 // protein binding // inferred from electronic annotation

al ion binding // inferred from electronic annotation

)4629 // phospholipase C activity // inferred from electronic annotation /// 0004871 // signal transducer activity
arity /// 0005515 // protein binding // inferred from electronic annotation /// 0008094 // DNA-dependent ATPa

ıferred from electronic annotation /// 0046872 // metal ion binding // inferred from electronic annotation
lectronic annotation /// 0005515 // protein binding // inferred from physical interaction /// 0008270 // zinc ion
/ DNA binding // inferred from electronic annotation /// 0003700 // sequence-specific DNA binding transcriptio

iTP binding // inferred from electronic annotation

'action /// 0051082 // unfolded protein binding // inferred from direct assay

NA binding transcription factor activity // not recorded /// 0003700 // sequence-specific DNA binding transcript
)4758 // serine C-palmitoyltransferase activity // inferred from electronic annotation /// 0008415 // acyltransfe
388 // transmembrane receptor activity // inferred from sequence or structural similarity /// 0004930 // G-prot
\ topoisomerase activity // inferred from electronic annotation /// 0003917 // DNA topoisomerase type I activit

'13 // protein tyrosine kinase activity // inferred from electronic annotation /// 0004713 // protein tyrosine kina
16853 // isomerase activity // inferred from electronic annotation

orane // inferred from electronic annotation /// 0016020 // membrane // inferred from electronic annotation
orded /// 0003730 // mRNA 3'-UTR binding // inferred from electronic annotation
binding // inferred from electronic annotation /// 0008198 // ferrous iron binding // inferred from sequence or
d binding // inferred from electronic annotation

nic annotation /// 0016564 // transcription repressor activity // inferred from electronic annotation /// 0017069

tivity // inferred from electronic annotation /// 0004674 // protein serine/threonine kinase activity // not recor
n electronic annotation /// 0016021 // integral to membrane // inferred from sequence or structural similarity

or statement
55 // electron carrier activity // inferred from electronic annotation /// 0016491 // oxidoreductase activity // inf
nilarity /// 0005515 // protein binding // inferred from electronic annotation /// 0008897 // holo-[acyl-carrier-p

onic annotation /// 0004871 // signal transducer activity // inferred from electronic annotation /// 0005509 // c
bospondin receptor activity // inferred from electronic annotation

ise activity // inferred from electronic annotation

t /// 0008415 // acyltransferase activity // inferred from electronic annotation /// 0016740 // transferase activit

otation /// 0005524 // ATP binding // inferred from electronic annotation /// 0016301 // kinase activity // infer
ATP binding // inferred from electronic annotation /// 0008080 // N-acetyltransferase activity // inferred from el

embrane // non-traceable author statement /// 0016021 // integral to membrane // inferred from electronic an

tivity // inferred from electronic annotation /// 0004674 // protein serine/threonine kinase activity // inferred f

on /// 0005248 // voltage-gated sodium channel activity // inferred from electronic annotation /// 0005272 // s

RING ubiquitin ligase complex // inferred from sequence or structural similarity

// inferred from electronic annotation /// 0005515 // protein binding // inferred from electronic annotation ///

IA binding // inferred from sequence or structural similarity /// 0003700 // sequence-specific DNA binding trans

/// 0016740 // transferase activity // inferred from electronic annotation /// 0016757 // transferase activity, tra
662 // CAAX-protein geranylgeranyltransferase activity // not recorded /// 0004662 // CAAX-protein geranylgera

ion
/ RNA polymerase III transcription factor activity // inferred from electronic annotation /// 0005515 // protein b
n sequence or structural similarity /// 0005515 // protein binding // inferred from electronic annotation /// 000
of molecular oxygen, incorporation of two atoms of oxygen // inferred from electronic annotation /// 0046872
rred from direct assay /// 0046873 // metal ion transmembrane transporter activity // inferred from electronic

transferase activity // inferred from electronic annotation /// 0016740 // transferase activity // inferred from el
zinc ion binding // inferred from electronic annotation
// protein binding // inferred from physical interaction /// 0008792 // arginine decarboxylase activity // inferre

// catalytic activity // inferred from electronic annotation /// 0004043 // L-aminoadipate-semialdehyde dehydro

iption cofactor activity // inferred from sequence or structural similarity /// 0003712 // transcription cofactor a

// 0005524 // ATP binding // inferred from electronic annotation /// 0008233 // peptidase activity // inferred fr

ectronic annotation
etylcholine receptor inhibitor activity // inferred from direct assay

metal ion binding // inferred from electronic annotation

otation

/// 0005515 // protein binding // inferred from physical interaction /// 0005515 // protein binding // inferred fr

tronic annotation /// 0016020 // membrane // inferred from electronic annotation /// 0016021 // integral to m

om electronic annotation /// 0005545 // phosphatidylinositol binding // not recorded /// 0008270 // zinc ion bi
NA binding transcription factor activity // inferred from electronic annotation /// 0016564 // transcription repre

3 // RNA binding // inferred from electronic annotation /// 0004386 // helicase activity // inferred from electron

se activity // inferred from electronic annotation /// 0016740 // transferase activity // inferred from electronic a

on binding // inferred from electronic annotation

/ 0005509 // calcium ion binding // inferred from direct assay /// 0005515 // protein binding // inferred from ph

metal ion binding // inferred from electronic annotation

0 // transferase activity // inferred from electronic annotation /// 0030976 // thiamin pyrophosphate binding //
tein kinase activity // inferred from electronic annotation /// 0004674 // protein serine/threonine kinase activity

096 // GTPase activator activity // inferred from electronic annotation /// 0005515 // protein binding // inferre

236 // serine-type peptidase activity // inferred from electronic annotation /// 0016787 // hydrolase activity // i
eoside-triphosphatase activity // inferred from electronic annotation
896 // cytokine receptor activity // inferred from electronic annotation /// 0005515 // protein binding // inferre

red from direct assay /// 0004672 // protein kinase activity // inferred from direct assay /// 0005515 // protein l
formate-tetrahydrofolate ligase activity // not recorded /// 0004329 // formate-tetrahydrofolate ligase activity ,

02 // receptor binding // traceable author statement /// 0005109 // frizzled binding // inferred from physical int

74 // protein serine/threonine kinase activity // inferred from electronic annotation /// 0004693 // cyclin-deper

on /// 0005515 // protein binding // inferred from electronic annotation /// 0008233 // peptidase activity // inf
vity // inferred from electronic annotation /// 0005515 // protein binding // inferred from physical interaction //
ronic annotation /// 0003700 // sequence-specific DNA binding transcription factor activity // inferred from dire
ATP binding // inferred from electronic annotation /// 0008026 // ATP-dependent helicase activity // inferred f
' cation channel activity // not recorded /// 0005261 // cation channel activity // inferred from electronic annota

rred from physical interaction /// 0016455 // RNA polymerase II transcription mediator activity // inferred from
on /// 0005249 // voltage-gated potassium channel activity // inferred from electronic annotation /// 0005267 ,
electronic annotation /// 0016021 // integral to membrane // inferred from electronic annotation

ctronic annotation /// 0000827 // inositol 1,3,4,5,6-pentakisphosphate kinase activity // inferred from sequenc
tase activity // inferred from electronic annotation

nferred from physical interaction /// 0005515 // protein binding // inferred from electronic annotation /// 0008
' /// 0003723 // RNA binding // inferred from electronic annotation /// 0003723 // RNA binding // inferred from
ferred from electronic annotation /// 0016787 // hydrolase activity // inferred from electronic annotation /// 0(
rred from electronic annotation /// 0016717 // oxidoreductase activity, acting on paired donors, with oxidation

osomal membrane // inferred from electronic annotation /// 0005768 // endosome // not recorded /// 00057
ed from direct assay /// 0032403 // protein complex binding // not recorded /// 0032403 // protein complex bir
ne transmembrane transporter activity // not recorded /// 0015293 // symporter activity // inferred from electr
nase activity // inferred from electronic annotation /// 0004713 // protein tyrosine kinase activity // inferred fro
nase activity // inferred from electronic annotation /// 0004713 // protein tyrosine kinase activity // inferred fro

n electronic annotation /// 0046983 // protein dimerization activity // not recorded /// 0051400 // BH domain b

ing transcription factor activity // inferred from electronic annotation /// 0003707 // steroid hormone receptor
ing transcription factor activity // inferred from electronic annotation /// 0003707 // steroid hormone receptor

: // not recorded /// 0008289 // lipid binding // inferred from electronic annotation /// 0016491 // oxidoreducta

ation /// 0008237 // metallopeptidase activity // inferred from electronic annotation /// 0008270 // zinc ion bir
erred from electronic annotation /// 0016491 // oxidoreductase activity // inferred from electronic annotation ,

// peptidase activity // inferred from electronic annotation /// 0008234 // cysteine-type peptidase activity // in
inase activity // inferred from electronic annotation /// 0005488 // binding // inferred from electronic annotatic
ronic annotation

sterol O-acyltransferase activity // not recorded /// 0004772 // sterol O-acyltransferase activity // inferred from

sium channel activity // inferred from electronic annotation /// 0005251 // delayed rectifier potassium channel a
/ ubiquitin-protein ligase activity // inferred from sequence or structural similarity /// 0005524 // ATP binding //

ity // inferred from direct assay /// 0019783 // small conjugating protein-specific protease activity // inferred fr

onic annotation /// 0008233 // peptidase activity // inferred from electronic annotation /// 0008237 // metallop
ctronic annotation

rom electronic annotation /// 0004715 // non-membrane spanning protein tyrosine kinase activity // inferred fr
l from electronic annotation
eptor activity // not recorded /// 0005000 // vasopressin receptor activity // inferred from electronic annotation
00 // sequence-specific DNA binding transcription factor activity // inferred from electronic annotation /// 0004
00 // sequence-specific DNA binding transcription factor activity // inferred from electronic annotation /// 0004

0005856 // cytoskeleton // inferred from electronic annotation /// 0005874 // microtubule // inferred from elec
/ 0004672 // protein kinase activity // inferred from sequence or structural similarity /// 0004674 // protein ser
annotation /// 0003705 // sequence-specific enhancer binding RNA polymerase II transcription factor activity //
/// 0030528 // transcription regulator activity // inferred from electronic annotation /// 0043565 // sequence-s

ectronic annotation /// 0005506 // iron ion binding // inferred from electronic annotation /// 0008391 // arach

rom electronic annotation
/// 0004340 // glucokinase activity // inferred from mutant phenotype /// 0004340 // glucokinase activity // no

y // inferred from electronic annotation /// 0004930 // G-protein coupled receptor activity // inferred from elec

68 // chitinase activity // inferred from direct assay /// 0004568 // chitinase activity // inferred from electronic a

assay /// 0004386 // helicase activity // inferred from electronic annotation /// 0005515 // protein binding // in
ding // inferred from physical interaction /// 0008270 // zinc ion binding // inferred from electronic annotation
erred from electronic annotation /// 0030528 // transcription regulator activity // inferred from electronic anno

// inferred from electronic annotation /// 0016491 // oxidoreductase activity // inferred from electronic annota
// inferred from electronic annotation /// 0016491 // oxidoreductase activity // inferred from electronic annota

/ // inferred from electronic annotation /// 0005524 // ATP binding // inferred from electronic annotation /// 0C
ictivity // inferred from electronic annotation

// 0005529 // sugar binding // inferred from electronic annotation

ctivity // inferred from electronic annotation /// 0016787 // hydrolase activity // inferred from electronic annot
: annotation
osine phosphatase activity // inferred from electronic annotation /// 0005515 // protein binding // inferred fron

ase activity // inferred from electronic annotation /// 0016787 // hydrolase activity // inferred from electronic a

inding // inferred from electronic annotation /// 0046872 // metal ion binding // traceable author statement //

ation /// 0043531 // ADP binding // not recorded /// 0050660 // FAD or FADH2 binding // not recorded /// 0051
th factor activity // not recorded /// 0008083 // growth factor activity // inferred from electronic annotation ///

c annotation /// 0043023 // ribosomal large subunit binding // inferred from physical interaction
from electronic annotation /// 0005515 // protein binding // inferred from electronic annotation

tation

ing // inferred from electronic annotation /// 0042802 // identical protein binding // inferred from electronic ar

ing // inferred from electronic annotation
ctronic annotation /// 0016740 // transferase activity // inferred from electronic annotation /// 0016757 // trar
ferred from electronic annotation /// 0020037 // heme binding // inferred from electronic annotation /// 00468
\ binding transcription factor activity // inferred from electronic annotation /// 0005515 // protein binding // in

ic annotation /// 0016491 // oxidoreductase activity // inferred from electronic annotation /// 0016702 // oxidc
electronic annotation /// 0005506 // iron ion binding // inferred from electronic annotation /// 0009055 // elect
04930 // G-protein coupled receptor activity // not recorded /// 0004930 // G-protein coupled receptor activity
notation /// 0016020 // membrane // inferred from electronic annotation /// 0016021 // integral to membrane
rom electronic annotation /// 0003723 // RNA binding // inferred from electronic annotation /// 0008270 // zin
ral similarity /// 0004221 // ubiquitin thiolesterase activity // inferred from electronic annotation /// 0004221 /
/ inferred from electronic annotation /// 0005524 // ATP binding // inferred from electronic annotation /// 001
/// 0043208 // glycosphingolipid binding // inferred from direct assay

n electronic annotation /// 0051087 // chaperone binding // not recorded /// 0051087 // chaperone binding //
rotein binding // inferred from physical interaction /// 0016563 // transcription activator activity // inferred fro

cription regulator activity // inferred from electronic annotation /// 0043565 // sequence-specific DNA binding /
cription regulator activity // inferred from electronic annotation /// 0043565 // sequence-specific DNA binding /

protein serine/threonine kinase activity // inferred from electronic annotation /// 0005524 // ATP binding // infe
ase activity // inferred from electronic annotation
ic annotation /// 0005515 // protein binding // inferred from electronic annotation /// 0005524 // ATP binding /

/ protein serine/threonine kinase activity // not recorded /// 0004674 // protein serine/threonine kinase activit

ect assay /// 0003700 // sequence-specific DNA binding transcription factor activity // not recorded /// 000370C

68 // chitinase activity // inferred from direct assay /// 0004568 // chitinase activity // inferred from electronic a
008138 // protein tyrosine/serine/threonine phosphatase activity // inferred from direct assay /// 0008138 // pr

ronic annotation /// 0016564 // transcription repressor activity // inferred from electronic annotation /// 00165
yclic-AMP phosphodiesterase activity // inferred from electronic annotation /// 0004117 // calmodulin-depend
/ potassium channel activity // inferred from electronic annotation /// 0047485 // protein N-terminus binding /

nic annotation
annotation /// 0005886 // plasma membrane // inferred from electronic annotation /// 0016020 // membrane
// cytoskeletal protein binding // traceable author statement /// 0016740 // transferase activity // inferred from
ing transcription factor activity // inferred from electronic annotation /// 0003707 // steroid hormone receptor

inferred from electronic annotation /// 0016787 // hydrolase activity // inferred from electronic annotation ///

notation /// 0005516 // calmodulin binding // inferred from electronic annotation /// 0005524 // ATP binding //

om electronic annotation /// 0046872 // metal ion binding // inferred from electronic annotation

tivity, transferring glycosyl groups // inferred from electronic annotation /// 0046872 // metal ion binding // inf

ic annotation /// 0005524 // ATP binding // inferred from electronic annotation /// 0008026 // ATP-dependent

/// 0016564 // transcription repressor activity // not recorded /// 0016564 // transcription repressor activity //

notation /// 0005524 // ATP binding // inferred from electronic annotation /// 0005525 // GTP binding // inferr
red from electronic annotation /// 0008184 // glycogen phosphorylase activity // not recorded /// 0016740 // tr
notation /// 0005525 // GTP binding // inferred from electronic annotation

on /// 0010843 // promoter binding // inferred from direct assay
tation
red from electronic annotation /// 0008237 // metallopeptidase activity // inferred from sequence or structural

onic annotation
rom electronic annotation /// 0005515 // protein binding // inferred from physical interaction /// 0042809 // vi

otation
ase activity, acting on the aldehyde or oxo group of donors, NAD or NADP as acceptor // inferred from direct as
tion
protein binding // inferred from physical interaction /// 0016563 // transcription activator activity // inferred fro

d from electronic annotation
ing transcription factor activity // inferred from electronic annotation /// 0005515 // protein binding // inferred

from electronic annotation /// 0016491 // oxidoreductase activity // inferred from electronic annotation
tronic annotation

rom physical interaction /// 0030528 // transcription regulator activity // inferred from electronic annotation //
vity // not recorded /// 0004713 // protein tyrosine kinase activity // inferred from electronic annotation /// 000
n electronic annotation /// 0046983 // protein dimerization activity // not recorded /// 0051400 // BH domain b
// inferred from electronic annotation /// 0001965 // G-protein alpha-subunit binding // not recorded /// 0001
red from electronic annotation /// 0043565 // sequence-specific DNA binding // inferred from electronic annot
/ cysteine-type peptidase activity // inferred from electronic annotation /// 0016787 // hydrolase activity // infe
notation /// 0005516 // calmodulin binding // inferred from electronic annotation /// 0005524 // ATP binding //
tation /// 0046872 // metal ion binding // inferred from electronic annotation
ion /// 0008514 // organic anion transmembrane transporter activity // not recorded /// 0015125 // bile acid tr

/// 0042803 // protein homodimerization activity // inferred from electronic annotation /// 0043498 // cell surf
n /// 0030054 // cell junction // inferred from electronic annotation
from electronic annotation /// 0005515 // protein binding // inferred from electronic annotation /// 0005529 /
nnotation /// 0015269 // calcium-activated potassium channel activity // inferred from electronic annotation //

eptor protein tyrosine kinase activity // inferred from electronic annotation /// 0004872 // receptor activity // ir

n sequence or structural similarity /// 0016787 // hydrolase activity // inferred from electronic annotation

se activity // inferred from electronic annotation /// 0016787 // hydrolase activity // inferred from electronic an

rom electronic annotation /// 0005524 // ATP binding // inferred from electronic annotation /// 0016301 // kin

y // not recorded /// 0004871 // signal transducer activity // inferred from electronic annotation /// 0005509 //
se activity // inferred from electronic annotation /// 0008094 // DNA-dependent ATPase activity // inferred fro

binding // inferred from electronic annotation /// 0043565 // sequence-specific DNA binding // inferred from e
n factor activity // inferred from electronic annotation /// 0003707 // steroid hormone receptor activity // infer

tion factor activity // inferred from electronic annotation /// 0003705 // sequence-specific enhancer binding RN
rase activity // inferred from electronic annotation /// 0016740 // transferase activity // inferred from electroni
ein coupled receptor activity // inferred from sequence or structural similarity /// 0004930 // G-protein couplec
y // inferred from electronic annotation /// 0003918 // DNA topoisomerase (ATP-hydrolyzing) activity // inferre

ıse activity // inferred from sequence or structural similarity /// 0004714 // transmembrane receptor protein tyı

structural similarity /// 0010302 // 2-oxoglutarate-dependent dioxygenase activity // inferred from direct assay

ı // snRNA binding // inferred from electronic annotation

ded /// 0004674 // protein serine/threonine kinase activity // inferred from electronic annotation /// 0004679 ,
/// 0016021 // integral to membrane // inferred from electronic annotation /// 0031966 // mitochondrial meml

ferred from electronic annotation /// 0020037 // heme binding // inferred from electronic annotation /// 00468
rotein] synthase activity // inferred from electronic annotation /// 0008897 // holo-[acyl-carrier-protein] syntha

calcium ion binding // inferred from electronic annotation /// 0005515 // protein binding // inferred from physic

ty // inferred from electronic annotation

red from electronic annotation /// 0016740 // transferase activity // inferred from electronic annotation
lectronic annotation /// 0008415 // acyltransferase activity // inferred from electronic annotation /// 0016740 /

notation

from electronic annotation /// 0005524 // ATP binding // inferred from electronic annotation /// 0016301 // kin

odium channel activity // inferred from electronic annotation

/ 0008270 // zinc ion binding // inferred from electronic annotation /// 0046872 // metal ion binding // inferred

scription factor activity // inferred from sequence or structural similarity /// 0003700 // sequence-specific DNA b

ansferring glycosyl groups // inferred from electronic annotation /// 0016758 // transferase activity, transferring
anyltransferase activity // inferred from electronic annotation /// 0005515 // protein binding // inferred from e

binding // inferred from electronic annotation
)5515 // protein binding // inferred from physical interaction
2 // metal ion binding // inferred from electronic annotation /// 0047800 // cysteamine dioxygenase activity // in
annotation

ectronic annotation /// 0018024 // histone-lysine N-methyltransferase activity // inferred from electronic anno

ed from direct assay /// 0042978 // ornithine decarboxylase activator activity // inferred from genetic interaction

ogenase activity // inferred from electronic annotation /// 0005524 // ATP binding // inferred from electronic an

ctivity // inferred from electronic annotation /// 0003714 // transcription corepressor activity // inferred from c

om electronic annotation /// 0008237 // metallopeptidase activity // inferred from electronic annotation /// 00

rom electronic annotation /// 0005516 // calmodulin binding // inferred from electronic annotation /// 003240[illegible]

embrane // inferred from electronic annotation

nding // inferred from electronic annotation /// 0016740 // transferase activity // inferred from electronic anno
essor activity // inferred from direct assay /// 0030528 // transcription regulator activity // inferred from electro

ic annotation /// 0005524 // ATP binding // inferred from electronic annotation /// 0008026 // ATP-dependent

annotation /// 0016773 // phosphotransferase activity, alcohol group as acceptor // inferred from electronic an

ysical interaction /// 0005524 // ATP binding // inferred from direct assay /// 0016594 // glycine binding // infe

/ inferred from electronic annotation /// 0046872 // metal ion binding // inferred from electronic annotation
/ // not recorded /// 0004674 // protein serine/threonine kinase activity // inferred from electronic annotation

d from electronic annotation /// 0046872 // metal ion binding // inferred from electronic annotation

inferred from electronic annotation

d from physical interaction /// 0005515 // protein binding // inferred from electronic annotation /// 0016500 //

binding // inferred from physical interaction /// 0005515 // protein binding // inferred from electronic annotatic
// inferred from electronic annotation /// 0005488 // binding // inferred from electronic annotation /// 000551

eraction /// 0005109 // frizzled binding // inferred from electronic annotation /// 0005110 // frizzled-2 binding ,

ndent protein kinase activity // inferred from electronic annotation /// 0005524 // ATP binding // inferred from

erred from electronic annotation /// 0008237 // metallopeptidase activity // inferred from electronic annotatio
/ 0005515 // protein binding // inferred from electronic annotation /// 0016209 // antioxidant activity // inferr
ect assay /// 0003700 // sequence-specific DNA binding transcription factor activity // not recorded /// 000370C
rom electronic annotation /// 0016787 // hydrolase activity // inferred from electronic annotation
ation

electronic annotation
// potassium channel activity // inferred from electronic annotation

e or structural similarity /// 0000832 // inositol hexakisphosphate 5-kinase activity // inferred from electronic a

134 // transcription factor binding // inferred from physical interaction /// 0008134 // transcription factor bindi
sequence or structural similarity /// 0005515 // protein binding // inferred from physical interaction /// 00055:
)46872 // metal ion binding // inferred from electronic annotation
of a pair of donors resulting in the reduction of molecular oxygen to two molecules of water // inferred from ele

8 // endosome // inferred from electronic annotation /// 0005770 // late endosome // inferred from direct assa
nding // inferred from electronic annotation
onic annotation /// 0017153 // sodium:dicarboxylate symporter activity // inferred from electronic annotation
m electronic annotation /// 0004715 // non-membrane spanning protein tyrosine kinase activity // inferred fron
m electronic annotation /// 0004715 // non-membrane spanning protein tyrosine kinase activity // inferred fron

binding // not recorded

activity // inferred from electronic annotation /// 0004872 // receptor activity // inferred from electronic annot
activity // inferred from electronic annotation /// 0004872 // receptor activity // inferred from electronic annot

ase activity // inferred from electronic annotation /// 0016702 // oxidoreductase activity, acting on single donor

nding // inferred from electronic annotation /// 0016787 // hydrolase activity // inferred from electronic annota
/// 0050660 // FAD or FADH2 binding // inferred from electronic annotation /// 0050661 // NADP or NADPH bin

ferred from electronic annotation /// 0008242 // omega peptidase activity // inferred from electronic annotatio
on /// 0005515 // protein binding // inferred from physical interaction /// 0005515 // protein binding // inferrec

electronic annotation /// 0008415 // acyltransferase activity // inferred from electronic annotation /// 0015485

activity // not recorded /// 0005267 // potassium channel activity // inferred from electronic annotation /// 000
' inferred from electronic annotation /// 0016874 // ligase activity // inferred from electronic annotation /// 001

om direct assay

peptidase activity // inferred from electronic annotation /// 0008270 // zinc ion binding // inferred from electro

rom electronic annotation /// 0005515 // protein binding // inferred from physical interaction /// 0005515 // pr

n /// 0017046 // peptide hormone binding // not recorded /// 0017046 // peptide hormone binding // inferred
402 // histone acetyltransferase activity // inferred from electronic annotation /// 0004871 // signal transducer
402 // histone acetyltransferase activity // inferred from electronic annotation /// 0004871 // signal transducer

tronic annotation /// 0070652 // HAUS complex // inferred from sequence or structural similarity
ine/threonine kinase activity // not recorded /// 0004674 // protein serine/threonine kinase activity // inferred
inferred from direct assay /// 0005515 // protein binding // inferred from physical interaction /// 0005515 // pr
pecific DNA binding // inferred from electronic annotation /// 0046983 // protein dimerization activity // inferre

idonic acid monooxygenase activity // not recorded /// 0008391 // arachidonic acid monooxygenase activity //

t recorded /// 0004340 // glucokinase activity // inferred from electronic annotation /// 0004340 // glucokinase

tronic annotation /// 0004942 // anaphylatoxin receptor activity // inferred from electronic annotation /// 000

annotation /// 0005529 // sugar binding // traceable author statement /// 0005529 // sugar binding // inferred

nferred from physical interaction /// 0005515 // protein binding // inferred from electronic annotation /// 0005
/// 0016874 // ligase activity // inferred from electronic annotation /// 0046872 // metal ion binding // inferred
tation /// 0051879 // Hsp90 protein binding // inferred from electronic annotation

tion /// 0020037 // heme binding // inferred from electronic annotation /// 0033783 // 25-hydroxycholesterol [illegible]
tion /// 0020037 // heme binding // inferred from electronic annotation /// 0033783 // 25-hydroxycholesterol [illegible]

)16301 // kinase activity // inferred from electronic annotation /// 0016740 // transferase activity // inferred fro

ation

n physical interaction /// 0016787 // hydrolase activity // inferred from electronic annotation /// 0046872 // me

annotation

/ 0046872 // metal ion binding // inferred from electronic annotation

.287 // NAD or NADH binding // not recorded
' 0008201 // heparin binding // not recorded /// 0008201 // heparin binding // inferred from electronic annotat

nnotation /// 0046332 // SMAD binding // inferred from physical interaction /// 0048407 // platelet-derived gro

nsferase activity, transferring glycosyl groups // inferred from electronic annotation /// 0046872 // metal ion bir
72 // metal ion binding // inferred from electronic annotation /// 0070330 // aromatase activity // inferred fron
ferred from electronic annotation /// 0008134 // transcription factor binding // inferred from electronic annota

oreductase activity, acting on single donors with incorporation of molecular oxygen, incorporation of two atoms
tron carrier activity // inferred from electronic annotation /// 0016491 // oxidoreductase activity // inferred fron
// inferred from electronic annotation /// 0046625 // sphingolipid binding // not recorded /// 0046625 // sphin
: // inferred from electronic annotation /// 0032592 // integral to mitochondrial membrane // inferred from ele
c ion binding // inferred from electronic annotation /// 0030528 // transcription regulator activity // inferred fro
/ ubiquitin thiolesterase activity // inferred from sequence or structural similarity /// 0008233 // peptidase activ
6874 // ligase activity // inferred from electronic annotation /// 0043758 // acetate-CoA ligase (ADP-forming) ac

inferred from electronic annotation
m direct assay /// 0043565 // sequence-specific DNA binding // inferred from electronic annotation /// 004698

// not recorded /// 0043565 // sequence-specific DNA binding // inferred from electronic annotation
// not recorded /// 0043565 // sequence-specific DNA binding // inferred from electronic annotation

erred from electronic annotation /// 0016301 // kinase activity // inferred from electronic annotation /// 00167

// inferred from electronic annotation /// 0008026 // ATP-dependent helicase activity // inferred from electroni

ty // inferred from electronic annotation /// 0005524 // ATP binding // not recorded /// 0005524 // ATP binding

) // sequence-specific DNA binding transcription factor activity // inferred from electronic annotation /// 00048

annotation /// 0005529 // sugar binding // traceable author statement /// 0005529 // sugar binding // inferred

rotein tyrosine/serine/threonine phosphatase activity // inferred from electronic annotation /// 0016787 // hyd

564 // transcription repressor activity // inferred from sequence or structural similarity /// 0016566 // specific tr

ent cyclic-nucleotide phosphodiesterase activity // not recorded /// 0004117 // calmodulin-dependent cyclic-nu

// not recorded

// inferred from electronic annotation /// 0016021 // integral to membrane // inferred from direct assay /// 00

m electronic annotation /// 0016779 // nucleotidyltransferase activity // inferred from electronic annotation ///

activity // inferred from electronic annotation /// 0004872 // receptor activity // inferred from electronic annot

0046872 // metal ion binding // inferred from electronic annotation

inferred from electronic annotation

ferred from electronic annotation

helicase activity // inferred from electronic annotation /// 0016787 // hydrolase activity // inferred from electro

inferred from electronic annotation /// 0042809 // vitamin D receptor binding // not recorded

ed from electronic annotation /// 0016301 // kinase activity // inferred from electronic annotation /// 0016740
ansferase activity // inferred from electronic annotation /// 0016757 // transferase activity, transferring glycos

similarity /// 0008237 // metallopeptidase activity // inferred from electronic annotation /// 0008270 // zinc ior

tamin D receptor binding // not recorded /// 0042809 // vitamin D receptor binding // inferred from electronic

say /// 0019145 // aminobutyraldehyde dehydrogenase activity // not recorded /// 0019145 // aminobutyraldel

m direct assay /// 0043565 // sequence-specific DNA binding // inferred from electronic annotation /// 004698

from physical interaction /// 0005515 // protein binding // inferred from electronic annotation /// 0043565 //

/ 0046872 // metal ion binding // inferred from electronic annotation /// 0070742 // C2H2 zinc finger domain b
04715 // non-membrane spanning protein tyrosine kinase activity // inferred from electronic annotation /// 000
inding // not recorded
965 // G-protein alpha-subunit binding // inferred from electronic annotation /// 0004871 // signal transducer a
ation
rred from electronic annotation
inferred from electronic annotation

ansmembrane transporter activity // not recorded /// 0015125 // bile acid transmembrane transporter activity

ace binding // inferred from electronic annotation

/ sugar binding // inferred from electronic annotation
/ 0016286 // small conductance calcium-activated potassium channel activity // not recorded /// 0016286 // sm

nferred from electronic annotation /// 0005003 // ephrin receptor activity // inferred from electronic annotation

notation

ase activity // inferred from electronic annotation /// 0016740 // transferase activity // inferred from electronic

calcium ion binding // inferred from electronic annotation /// 0005515 // protein binding // inferred from elect
n sequence or structural similarity /// 0008353 // RNA polymerase II carboxy-terminal domain kinase activity //

electronic annotation /// 0046872 // metal ion binding // inferred from electronic annotation
red from electronic annotation /// 0003727 // single-stranded RNA binding // inferred from direct assay /// 000

A polymerase II transcription factor activity // not recorded /// 0016563 // transcription activator activity // infe
c annotation /// 0016769 // transferase activity, transferring nitrogenous groups // inferred from electronic anr
l receptor activity // inferred from electronic annotation
d from electronic annotation /// 0005524 // ATP binding // inferred from electronic annotation /// 0016853 // i

rosine kinase activity // inferred from electronic annotation /// 0004716 // receptor signaling protein tyrosine k

/// 0016491 // oxidoreductase activity // inferred from electronic annotation /// 0016702 // oxidoreductase ac

// AMP-activated protein kinase activity // not recorded /// 0004679 // AMP-activated protein kinase activity //
brane // inferred from electronic annotation

72 // metal ion binding // inferred from electronic annotation
ase activity // inferred from sequence or structural similarity /// 0016740 // transferase activity // inferred from

cal interaction /// 0008081 // phosphoric diester hydrolase activity // inferred from electronic annotation /// 00

// transferase activity // inferred from electronic annotation

ase activity // inferred from electronic annotation /// 0016740 // transferase activity // inferred from electronic

from electronic annotation

binding transcription factor activity // inferred from electronic annotation /// 0003704 // specific RNA polymera

g hexosyl groups // inferred from electronic annotation
lectronic annotation /// 0008144 // drug binding // not recorded /// 0008144 // drug binding // inferred from e

nferred from electronic annotation

tation /// 0042054 // histone methyltransferase activity // inferred from direct assay

n

nnotation /// 0016491 // oxidoreductase activity // inferred from electronic annotation /// 0016874 // ligase act

direct assay /// 0004872 // receptor activity // inferred from electronic annotation /// 0005515 // protein bindir

08270 // zinc ion binding // inferred from electronic annotation /// 0016787 // hydrolase activity // inferred fro

3 // protein complex binding // not recorded /// 0032403 // protein complex binding // inferred from electronic

tation /// 0016757 // transferase activity, transferring glycosyl groups // inferred from electronic annotation ///
onic annotation /// 0043565 // sequence-specific DNA binding // inferred from electronic annotation

helicase activity // inferred from electronic annotation /// 0016787 // hydrolase activity // inferred from electro

notation /// 0050277 // sedoheptulokinase activity // inferred from electronic annotation

rred from direct assay /// 0016853 // isomerase activity // inferred from electronic annotation /// 0018114 // tl

/// 0004697 // protein kinase C activity // inferred from electronic annotation /// 0005515 // protein binding //

/ protein-hormone receptor activity // not recorded /// 0016500 // protein-hormone receptor activity // inferre

on /// 0008270 // zinc ion binding // inferred from electronic annotation /// 0016564 // transcription repressor
5 // protein binding // inferred from electronic annotation /// 0005524 // ATP binding // inferred from electron

// inferred from direct assay /// 0005115 // receptor tyrosine kinase-like orphan receptor binding // inferred fro

electronic annotation /// 0016301 // kinase activity // inferred from electronic annotation /// 0016740 // trans

n /// 0008270 // zinc ion binding // inferred from electronic annotation /// 0016787 // hydrolase activity // infe
ed from electronic annotation /// 0016491 // oxidoreductase activity // inferred from electronic annotation ///
) // sequence-specific DNA binding transcription factor activity // inferred from electronic annotation /// 00048

nnotation /// 0000832 // inositol hexakisphosphate 5-kinase activity // inferred from sequence or structural sim

ng // inferred from electronic annotation /// 0016491 // oxidoreductase activity // inferred from electronic ann
15 // protein binding // inferred from electronic annotation /// 0008022 // protein C-terminus binding // inferre

ectronic annotation

ay /// 0005770 // late endosome // not recorded /// 0005886 // plasma membrane // inferred from electronic a

m electronic annotation /// 0005515 // protein binding // inferred from electronic annotation /// 0005524 // AT
m electronic annotation /// 0005515 // protein binding // inferred from electronic annotation /// 0005524 // AT

tation /// 0004879 // ligand-dependent nuclear receptor activity // inferred from electronic annotation /// 0004
tation /// 0004879 // ligand-dependent nuclear receptor activity // inferred from electronic annotation /// 0004

s with incorporation of molecular oxygen, incorporation of two atoms of oxygen // not recorded /// 0016702 //

tion /// 0046872 // metal ion binding // inferred from electronic annotation
nding // inferred from electronic annotation

on /// 0016787 // hydrolase activity // inferred from electronic annotation
d from electronic annotation /// 0005524 // ATP binding // inferred from electronic annotation /// 0008144 // d

; // cholesterol binding // inferred from electronic annotation /// 0016740 // transferase activity // inferred fror

)5515 // protein binding // inferred from electronic annotation /// 0005515 // protein binding // inferred from p
19787 // small conjugating protein ligase activity // inferred from electronic annotation

nic annotation /// 0016787 // hydrolase activity // inferred from electronic annotation /// 0017111 // nucleosid

rotein binding // inferred from electronic annotation /// 0005524 // ATP binding // inferred from electronic ann

from electronic annotation /// 0031894 // V1A vasopressin receptor binding // not recorded /// 0031894 // V1A
activity // inferred from electronic annotation /// 0005515 // protein binding // inferred from physical interact
activity // inferred from electronic annotation /// 0005515 // protein binding // inferred from physical interact

from electronic annotation /// 0005515 // protein binding // inferred from physical interaction /// 0005515 // p
rotein binding // inferred from electronic annotation /// 0008134 // transcription factor binding // not recorded
ed from electronic annotation

inferred from electronic annotation /// 0009055 // electron carrier activity // inferred from electronic annotatic

activity // inferred from sequence or structural similarity /// 0004396 // hexokinase activity // inferred from ele

4943 // C3a anaphylatoxin receptor activity // not recorded /// 0004943 // C3a anaphylatoxin receptor activity /

from electronic annotation /// 0008061 // chitin binding // inferred from electronic annotation /// 0043169 // c

524 // ATP binding // inferred from electronic annotation /// 0008270 // zinc ion binding // inferred from electr
l from electronic annotation

7alpha-hydroxylase activity // inferred from electronic annotation /// 0046872 // metal ion binding // inferred f
7alpha-hydroxylase activity // inferred from electronic annotation /// 0046872 // metal ion binding // inferred f

om electronic annotation /// 0019208 // phosphatase regulator activity // inferred from direct assay

etal ion binding // inferred from electronic annotation

ion /// 0042056 // chemoattractant activity // inferred from electronic annotation

owth factor binding // not recorded /// 0048407 // platelet-derived growth factor binding // inferred from electr

nding // inferred from electronic annotation
n electronic annotation
tion /// 0008190 // eukaryotic initiation factor 4E binding // inferred from electronic annotation /// 0008301 //

of oxygen // inferred from electronic annotation /// 0020037 // heme binding // inferred from electronic anno
m electronic annotation /// 0046872 // metal ion binding // inferred from electronic annotation /// 0050660 //
golipid binding // inferred from electronic annotation
ctronic annotation /// 0043234 // protein complex // inferred from electronic annotation
om electronic annotation /// 0046872 // metal ion binding // inferred from electronic annotation
vity // inferred from electronic annotation /// 0008234 // cysteine-type peptidase activity // inferred from electr
ctivity // inferred from electronic annotation

3 // protein dimerization activity // inferred from electronic annotation

40 // transferase activity // inferred from electronic annotation

ic annotation /// 0016787 // hydrolase activity // inferred from electronic annotation

; // inferred from electronic annotation /// 0016301 // kinase activity // inferred from electronic annotation ///

71 // signal transducer activity // inferred from electronic annotation /// 0004872 // receptor activity // inferrec

from electronic annotation /// 0008061 // chitin binding // inferred from electronic annotation /// 0043169 // c
lrolase activity // inferred from electronic annotation /// 0016791 // phosphatase activity // inferred from electr

ranscriptional repressor activity // inferred from direct assay /// 0017069 // snRNA binding // inferred from elec
ıcleotide phosphodiesterase activity // inferred from electronic annotation /// 0005516 // calmodulin binding //

)16021 // integral to membrane // inferred from electronic annotation /// 0030018 // Z disc // inferred from ele
/ 0032947 // protein complex scaffold // not recorded /// 0042803 // protein homodimerization activity // not r
tation /// 0004879 // ligand-dependent nuclear receptor activity // inferred from electronic annotation /// 0008

onic annotation

// transferase activity // inferred from electronic annotation /// 0016776 // phosphotransferase activity, phosp
yl groups // inferred from electronic annotation /// 0030170 // pyridoxal phosphate binding // inferred from ele

n binding // inferred from electronic annotation /// 0016787 // hydrolase activity // inferred from electronic an

annotation /// 0042826 // histone deacetylase binding // not recorded /// 0042826 // histone deacetylase bind

hyde dehydrogenase activity // inferred from electronic annotation /// 0042803 // protein homodimerization ac

3 // protein dimerization activity // inferred from electronic annotation

sequence-specific DNA binding // inferred from electronic annotation /// 0046983 // protein dimerization activi

inding // not recorded
4872 // receptor activity // inferred from electronic annotation /// 0005515 // protein binding // inferred from

activity // inferred from electronic annotation /// 0004872 // receptor activity // inferred from electronic annota

// inferred from electronic annotation /// 0015349 // thyroid hormone transmembrane transporter activity // r

nall conductance calcium-activated potassium channel activity // inferred from electronic annotation

n /// 0005515 // protein binding // inferred from physical interaction /// 0005515 // protein binding // inferred

: annotation

:ronic annotation /// 0005543 // phospholipid binding // not recorded /// 0005543 // phospholipid binding // in
' inferred from electronic annotation /// 0008353 // RNA polymerase II carboxy-terminal domain kinase activity

)3727 // single-stranded RNA binding // not recorded /// 0003727 // single-stranded RNA binding // inferred frc

erred from direct assay /// 0030528 // transcription regulator activity // inferred from electronic annotation ///
notation /// 0030170 // pyridoxal phosphate binding // inferred from electronic annotation

somerase activity // inferred from electronic annotation

inase activity // inferred from electronic annotation /// 0004871 // signal transducer activity // inferred from di

tivity, acting on single donors with incorporation of molecular oxygen, incorporation of two atoms of oxygen //

traceable author statement /// 0004679 // AMP-activated protein kinase activity // inferred from electronic an

electronic annotation /// 0046872 // metal ion binding // inferred from electronic annotation

51019 // mitogen-activated protein kinase binding // not recorded

: annotation

ıse II transcription factor activity // inferred from sequence or structural similarity /// 0003705 // sequence-spec

lectronic annotation /// 0008270 // zinc ion binding // not recorded /// 0008270 // zinc ion binding // inferred f

tivity // inferred from electronic annotation /// 0016878 // acid-thiol ligase activity // inferred from sequence or

ıg // inferred from physical interaction /// 0005515 // protein binding // inferred from electronic annotation ///

m electronic annotation /// 0017111 // nucleoside-triphosphatase activity // inferred from electronic annotatio

: annotation

/ 0046872 // metal ion binding // inferred from electronic annotation

onic annotation

hreonine racemase activity // inferred from direct assay /// 0030165 // PDZ domain binding // inferred from ele

inferred from physical interaction /// 0005515 // protein binding // inferred from electronic annotation /// 000

d from electronic annotation /// 0017046 // peptide hormone binding // not recorded /// 0017046 // peptide h

activity // inferred from direct assay /// 0043565 // sequence-specific DNA binding // inferred from direct assay
ic annotation /// 0016874 // ligase activity // inferred from electronic annotation /// 0042803 // protein homod

m electronic annotation /// 0005125 // cytokine activity // inferred from direct assay /// 0005515 // protein bi

ferase activity // inferred from electronic annotation

rred from electronic annotation /// 0046872 // metal ion binding // inferred from electronic annotation
0016787 // hydrolase activity // inferred from electronic annotation /// 0030160 // GKAP/Homer scaffold activi
71 // signal transducer activity // inferred from electronic annotation /// 0004872 // receptor activity // inferred

nilarity /// 0003993 // acid phosphatase activity // inferred from electronic annotation /// 0005524 // ATP bindi

otation /// 0016564 // transcription repressor activity // inferred from direct assay /// 0016616 // oxidoreducta
ed from electronic annotation /// 0042162 // telomeric DNA binding // inferred from electronic annotation /// 0

nnotation /// 0010008 // endosome membrane // inferred from electronic annotation /// 0016020 // membrar

P binding // inferred from electronic annotation /// 0016301 // kinase activity // inferred from electronic annot
P binding // inferred from electronic annotation /// 0016301 // kinase activity // inferred from electronic annot

887 // thyroid hormone receptor activity // inferred from electronic annotation /// 0008270 // zinc ion binding
887 // thyroid hormone receptor activity // inferred from electronic annotation /// 0008270 // zinc ion binding

oxidoreductase activity, acting on single donors with incorporation of molecular oxygen, incorporation of two a

lrug binding // inferred from electronic annotation /// 0016301 // kinase activity // not recorded /// 0016301 //

n electronic annotation /// 0034736 // cholesterol O-acyltransferase activity // inferred from electronic annotat

physical interaction /// 0008092 // cytoskeletal protein binding // inferred from direct assay /// 0008270 // zinc

le-triphosphatase activity // inferred from electronic annotation /// 0046872 // metal ion binding // inferred fro

otation /// 0016301 // kinase activity // inferred from electronic annotation /// 0016740 // transferase activity /

A vasopressin receptor binding // inferred from electronic annotation
ion /// 0008134 // transcription factor binding // not recorded /// 0008415 // acyltransferase activity // inferred
ion /// 0008134 // transcription factor binding // not recorded /// 0008415 // acyltransferase activity // inferred

protein binding // inferred from electronic annotation /// 0005524 // ATP binding // not recorded /// 0005524 /
l /// 0008134 // transcription factor binding // inferred from electronic annotation /// 0016563 // transcription

on /// 0016491 // oxidoreductase activity // inferred from electronic annotation /// 0016712 // oxidoreductase a

ectronic annotation /// 0005515 // protein binding // inferred from physical interaction /// 0005515 // protein b

// inferred from electronic annotation

cation binding // inferred from electronic annotation

ronic annotation /// 0016787 // hydrolase activity // inferred from electronic annotation /// 0046872 // metal io

rom electronic annotation
rom electronic annotation

ronic annotation

DNA bending activity // inferred from electronic annotation /// 0016564 // transcription repressor activity // in

tation /// 0046872 // metal ion binding // inferred from electronic annotation
FAD or FADH2 binding // inferred from electronic annotation

ronic annotation /// 0008270 // zinc ion binding // inferred from electronic annotation /// 0016787 // hydrolase

0016740 // transferase activity // inferred from electronic annotation /// 0042277 // peptide binding // not rec[illegible]

[illegible] from electronic annotation /// 0005515 // protein binding // inferred from physical interaction /// 0008134 //

[illegible]cation binding // inferred from electronic annotation
[illegible]ronic annotation /// 0033549 // MAP kinase phosphatase activity // inferred from electronic annotation

[illegible]tronic annotation /// 0017069 // snRNA binding // inferred from sequence or structural similarity
[illegible] inferred from electronic annotation /// 0016787 // hydrolase activity // inferred from electronic annotation //[illegible]

[illegible]ctronic annotation /// 0031430 // M band // inferred from electronic annotation /// 0042383 // sarcolemma //
[illegible]ecorded /// 0046872 // metal ion binding // inferred from electronic annotation
[illegible]270 // zinc ion binding // inferred from electronic annotation /// 0043565 // sequence-specific DNA binding // [illegible]

hate group as acceptor // inferred from electronic annotation /// 0019201 // nucleotide kinase activity // inferr
ctronic annotation /// 0042803 // protein homodimerization activity // not recorded /// 0042803 // protein hor

notation /// 0046872 // metal ion binding // inferred from electronic annotation

ing // inferred from electronic annotation /// 0046872 // metal ion binding // inferred from electronic annotatio

ctivity // not recorded /// 0042803 // protein homodimerization activity // inferred from electronic annotation /

ity // inferred from electronic annotation

physical interaction /// 0005515 // protein binding // inferred from electronic annotation /// 0005524 // ATP bi

ation /// 0004930 // G-protein coupled receptor activity // inferred from electronic annotation /// 0005543 // p

not recorded /// 0015349 // thyroid hormone transmembrane transporter activity // inferred from electronic an

from electronic annotation /// 0005524 // ATP binding // inferred from electronic annotation /// 0016301 // kir

nferred from electronic annotation /// 0008081 // phosphoric diester hydrolase activity // inferred from electror
// inferred from sequence or structural similarity

om electronic annotation /// 0004872 // receptor activity // inferred from electronic annotation /// 0004879 // l

0043565 // sequence-specific DNA binding // not recorded /// 0043565 // sequence-specific DNA binding // inf

rect assay /// 0004872 // receptor activity // inferred from electronic annotation /// 0004888 // transmembran[illegible]

inferred from electronic annotation /// 0043734 // DNA-N1-methyladenine dioxygenase activity // inferred fro[illegible]

notation /// 0005515 // protein binding // inferred from electronic annotation /// 0005524 // ATP binding // no[illegible]

cific enhancer binding RNA polymerase II transcription factor activity // inferred from sequence or structural sim

from electronic annotation /// 0016740 // transferase activity // inferred from electronic annotation /// 001984

r structural similarity /// 0031177 // phosphopantetheine binding // inferred from electronic annotation /// 004

0008168 // methyltransferase activity // inferred from electronic annotation /// 0008270 // zinc ion binding //

on /// 0046872 // metal ion binding // inferred from electronic annotation

ctronic annotation /// 0030170 // pyridoxal phosphate binding // inferred from mutant phenotype /// 0030170

5524 // ATP binding // inferred from electronic annotation /// 0008270 // zinc ion binding // inferred from elec

hormone binding // inferred from electronic annotation

/// 0046872 // metal ion binding // inferred from electronic annotation /// 0070087 // chromo shadow domai
limerization activity // inferred from electronic annotation

nding // inferred from physical interaction

ity // inferred from direct assay /// 0035256 // metabotropic glutamate receptor binding // not recorded /// 00
from electronic annotation /// 0005515 // protein binding // inferred from physical interaction /// 0008134 //

ng // inferred from electronic annotation /// 0016301 // kinase activity // inferred from electronic annotation //

ıse activity, acting on the CH-OH group of donors, NAD or NADP as acceptor // inferred from electronic annotati
)042162 // telomeric DNA binding // inferred from sequence or structural similarity

ıe // inferred from electronic annotation /// 0016021 // integral to membrane // inferred from electronic annot

tation /// 0016740 // transferase activity // inferred from electronic annotation /// 0046872 // metal ion binding
tation /// 0016740 // transferase activity // inferred from electronic annotation /// 0046872 // metal ion binding

// inferred from electronic annotation /// 0043565 // sequence-specific DNA binding // inferred from electronic
// inferred from electronic annotation /// 0043565 // sequence-specific DNA binding // inferred from electronic

ıtoms of oxygen // inferred from electronic annotation /// 0020037 // heme binding // inferred from electronic

' kinase activity // inferred from electronic annotation /// 0016740 // transferase activity // inferred from electr

tion /// 0034737 // ergosterol O-acyltransferase activity // inferred from electronic annotation /// 0034738 // la

ion binding // inferred from electronic annotation /// 0015271 // outward rectifier potassium channel activity /

om electronic annotation

// inferred from electronic annotation

d from electronic annotation /// 0016740 // transferase activity // inferred from electronic annotation /// 0030
d from electronic annotation /// 0016740 // transferase activity // inferred from electronic annotation /// 0030

/ ATP binding // inferred from electronic annotation /// 0016301 // kinase activity // inferred from electronic an
activator activity // inferred from direct assay /// 0042803 // protein homodimerization activity // inferred from

activity, acting on paired donors, with incorporation or reduction of molecular oxygen, reduced flavin or flavopr

binding // inferred from electronic annotation /// 0005524 // ATP binding // not recorded /// 0005524 // ATP bi

on binding // inferred from electronic annotation /// 0070087 // chromo shadow domain binding // inferred fro

ferred from direct assay /// 0016564 // transcription repressor activity // inferred from electronic annotation //

e activity // inferred from electronic annotation /// 0046872 // metal ion binding // inferred from electronic ann

orded /// 0051219 // phosphoprotein binding // not recorded

transcription factor binding // not recorded /// 0017162 // aryl hydrocarbon receptor binding // inferred from e

/ 0030552 // cAMP binding // not recorded /// 0030552 // cAMP binding // inferred from electronic annotation

inferred from electronic annotation

inferred from electronic annotation /// 0046872 // metal ion binding // inferred from electronic annotation

ed from electronic annotation /// 0019205 // nucleobase, nucleoside, nucleotide kinase activity // inferred from
modimerization activity // inferred from electronic annotation

on /// 0046966 // thyroid hormone receptor binding // not recorded /// 0046966 // thyroid hormone receptor b

/// 0043176 // amine binding // not recorded /// 0043176 // amine binding // inferred from electronic annotati

nding // inferred from electronic annotation /// 0016301 // kinase activity // inferred from electronic annotatio

hospholipid binding // not recorded /// 0005543 // phospholipid binding // inferred from electronic annotation

notation

ase activity // inferred from electronic annotation /// 0016740 // transferase activity // inferred from electroni

ic annotation /// 0016787 // hydrolase activity // inferred from electronic annotation /// 0046872 // metal ion

igand-dependent nuclear receptor activity // inferred from direct assay /// 0004879 // ligand-dependent nucle

erred from electronic annotation

e receptor activity // not recorded /// 0004888 // transmembrane receptor activity // inferred from electronic a

n electronic annotation /// 0043734 // DNA-N1-methyladenine dioxygenase activity // inferred from sequence [illegible]

t recorded /// 0005524 // ATP binding // inferred from electronic annotation /// 0016301 // kinase activity // ir

ilarity /// 0008134 // transcription factor binding // inferred from sequence or structural similarity /// 0008301

0 // isoprenoid binding // not recorded /// 0019840 // isoprenoid binding // inferred from electronic annotatior

8037 // cofactor binding // inferred from electronic annotation

inferred from electronic annotation /// 0016740 // transferase activity // inferred from electronic annotation //

) // pyridoxal phosphate binding // not recorded /// 0030170 // pyridoxal phosphate binding // inferred from ele

tronic annotation /// 0015459 // potassium channel regulator activity // not recorded /// 0015459 // potassium

ı binding // inferred from electronic annotation

35256 // metabotropic glutamate receptor binding // inferred from electronic annotation /// 0051920 // peroxi
transcription factor binding // not recorded /// 0017162 // aryl hydrocarbon receptor binding // inferred from e

// 0016740 // transferase activity // inferred from electronic annotation /// 0033857 // diphosphoinositol-penta

on /// 0019904 // protein domain specific binding // inferred from electronic annotation /// 0042803 // protein

tation /// 0030670 // phagocytic vesicle membrane // inferred from mutant phenotype /// 0031088 // platelet d

g // inferred from electronic annotation
g // inferred from electronic annotation

c annotation /// 0046872 // metal ion binding // inferred from electronic annotation
c annotation /// 0046872 // metal ion binding // inferred from electronic annotation

annotation /// 0046872 // metal ion binding // inferred from electronic annotation

onic annotation /// 0016772 // transferase activity, transferring phosphorus-containing groups // inferred from

anosterol O-acyltransferase activity // inferred from electronic annotation

/ not recorded /// 0016301 // kinase activity // inferred from electronic annotation /// 0046872 // metal ion bi

528 // transcription regulator activity // inferred from electronic annotation
528 // transcription regulator activity // inferred from electronic annotation

nnotation /// 0016740 // transferase activity // inferred from electronic annotation /// 0042277 // peptide bind
direct assay /// 0042803 // protein homodimerization activity // not recorded /// 0042803 // protein homodin

otein as one donor, and incorporation of one atom of oxygen // inferred from electronic annotation /// 002003

nding // inferred from electronic annotation /// 0005524 // ATP binding // inferred from sequence or structural

m electronic annotation

'/ 0016565 // general transcriptional repressor activity // not recorded /// 0016565 // general transcriptional re

notation

electronic annotation /// 0030528 // transcription regulator activity // inferred from electronic annotation /// 00

/// 0046872 // metal ion binding // inferred from electronic annotation /// 0048101 // calcium- and calmodulin

ı electronic annotation /// 0042802 // identical protein binding // not recorded /// 0042802 // identical protein

ɔinding // inferred from electronic annotation

on /// 0047105 // 4-trimethylammoniobutyraldehyde dehydrogenase activity // not recorded /// 0047105 // 4-[illegible]

ı /// 0016740 // transferase activity // inferred from electronic annotation /// 0046872 // metal ion binding // [illegible]

/// 0030165 // PDZ domain binding // inferred from physical interaction

c annotation

binding // inferred from electronic annotation

ar receptor activity // inferred from electronic annotation /// 0004887 // thyroid hormone receptor activity // n

ınnotation /// 0005006 // epidermal growth factor receptor activity // inferred from direct assay /// 0005006 //

or structural similarity /// 0046872 // metal ion binding // inferred from electronic annotation /// 0051747 // cy

ıferred from electronic annotation /// 0016740 // transferase activity // inferred from electronic annotation ///

// DNA bending activity // inferred from sequence or structural similarity /// 0010843 // promoter binding // in

n /// 0042277 // peptide binding // not recorded /// 0042277 // peptide binding // inferred from electronic ann

'/ 0016922 // ligand-dependent nuclear receptor binding // inferred from physical interaction /// 0018024 // his

ectronic annotation /// 0030378 // serine racemase activity // inferred from direct assay /// 0030378 // serine r

channel regulator activity // inferred from electronic annotation /// 0016301 // kinase activity // inferred from

redoxin activity // inferred from electronic annotation
electronic annotation /// 0030528 // transcription regulator activity // inferred from electronic annotation /// 0[illegible]

akisphosphate kinase activity // inferred from electronic annotation /// 0033857 // diphosphoinositol-pentakisp

ı homodimerization activity // not recorded /// 0048037 // cofactor binding // in

ɔdense granule membrane // not recorded

electronic annotation /// 0016773 // phosphotransferase activity, alcohol group

nding // inferred from electronic annotation /// 0047485 // protein N-terminus bind

ing // not recorded /// 0051219 // phosphoprotein binding // not recorded
nerization activity // inferred from electronic annotation /// 0043565 // sequence-spec

7 // heme binding // inferred from electronic annotation /// 0046872 // metal ion binding // infer

l similarity /// 0005536 // glucose binding // not recorded /// 0005536 // glucose bindin

pressor activity // inferred from electronic annotation /// 0030528 // transcr

051879 // Hsp90 protein binding // inferred from electronic annotation

-regulated 3',5'-cyclic-GMP phosphodiesterase activity // not recorded /// 0048101 // calcium

binding // inferred from electronic annotation /// 0046899 // nucleoside triphospha

trimethylammoniobutyraldehyde dehydrogenase activity // inferred from electronic annotation /// 0051287 //

inferred from electronic annotation

ot recorded /// 0004887 // thyroid hormone receptor activity // traceable author statement

' epidermal growth factor receptor activity // not recorded /// 0005006 // epiderm

ytosine C-5 DNA demethylase activity // inferred from direct assay

0030674 // protein binding, bridging // not recorded /// 0030674 // protein binding

ıferred from sequence or structural similarity /// 0016563 // transcription activ

otation /// 0046872 // metal ion binding // inferred from electronic annotation

ꜱtone-lysine N-methyltransferase activity // inferred from electronic anno

acemase activity // not recorded /// 0030378 // serine racemase activity // inferred fr

electronic annotation /// 0016740 // transferase activity // inferred from ele

051879 // Hsp90 protein binding // inferred from electronic annotation

hosphate kinase activity // inferred from sequence or structural similarity

NAD

Notes : Created from Advanced Analysis operation: significance Analysis.
#Entitylist : Filtered on Expression (20.0 - 100.0)th Percentile in the Raw Data
#Interpretation : Time-Treat
#Experiment: RPE-RMA DAYS 7-30
#corrected p-value cut-off:0.05
#Post-hoc test: SNK
#Selected Test : Oneway ANOVA
#p-value computation: Asymptotic
#Multiple Testing Correction: No Correction
#
Technology : Affymetrix.GeneChip.Mouse430_2
Owner : gxuser
Created On : Sat Jan 12 21:55:15 PST 2013

Probe Set ID	p	FC ([D07-D	Log FC ([D(	FC (abs) ([I	Regulation	FC ([D07-T.	Log FC ([D(	FC (abs) ([I
1415686_at	0.028478	1.004048	0.005829	1.004048	up	1.12525	0.170246	1.12525
1415702_a_at	0.00894	1.015185	0.021742	1.015185	up	1.093298	0.128687	1.093298
1415709_s_at	0.021965	-1.00916	-0.01316	1.009165	down	1.044821	0.063255	1.044821
1415712_at	0.033531	1.078887	0.109543	1.078887	up	1.019376	0.027687	1.019376
1415755_a_at	0.023762	1.197487	0.260009	1.197487	up	1.244129	0.315136	1.244129
1415773_at	0.006322	-1.18211	-0.24136	1.182109	down	-1.19338	-0.25505	1.193377
1415775_at	0.034024	1.032355	0.045939	1.032355	up	1.100697	0.138417	1.100697
1415777_at	0.008835	1.075341	0.104795	1.075341	up	1.062542	0.087519	1.062542
1415814_at	0.014919	1.144022	0.194115	1.144022	up	1.352191	0.435299	1.352191
1415824_at	0.003315	1.155559	0.20859	1.155559	up	1.333744	0.415482	1.333744
1415854_at	0.013487	-1.15159	-0.20363	1.151594	down	1.103008	0.141444	1.103008
1415855_at	0.01783	1.097497	0.134216	1.097497	up	1.157213	0.210654	1.157213
1415882_at	0.027727	1.087519	0.12104	1.087519	up	1.197439	0.259952	1.197439
1415916_a_at	0.046218	1.064919	0.090744	1.064919	up	1.155312	0.208283	1.155312
1415920_at	0.019361	1.056473	0.079256	1.056473	up	1.067067	0.093651	1.067067
1415925_a_at	0.012924	-1.05263	-0.074	1.052633	down	1.03082	0.043793	1.03082
1415931_at	0.015963	-1.18628	-0.24644	1.186278	down	-1.12302	-0.16738	1.12302
1415935_at	0.034353	-1.08614	-0.1192	1.086136	down	-1.50536	-0.59011	1.505356
1415970_at	0.020744	1.112365	0.15363	1.112365	up	-1.00608	-0.00875	1.006084
1415976_a_at	0.008289	1.160696	0.214991	1.160696	up	1.087685	0.121261	1.087685
1415989_at	0.008556	1.174844	0.23247	1.174844	up	1.178028	0.236373	1.178028
1415991_a_at	0.006844	1.094479	0.130245	1.094479	up	-1.06092	-0.08532	1.060923
1416005_at	0.048665	1.100028	0.137541	1.100028	up	1.17586	0.233716	1.17586
1416008_at	0.028908	1.00248	0.003574	1.00248	up	-1.25507	-0.32777	1.255074
1416024_x_at	0.022875	1.005688	0.008182	1.005688	up	1.067135	0.093743	1.067135
1416062_at	0.008585	-1.03411	-0.04838	1.034106	down	-1.06476	-0.09052	1.064757
1416073_a_at	0.030268	1.099027	0.136227	1.099027	up	1.132286	0.179238	1.132286
1416077_at	0.011341	1.008609	0.012367	1.008609	up	-1.08631	-0.11943	1.086307
1416084_at	0.02704	-1.04863	-0.06851	1.048635	down	-1.05801	-0.08135	1.05801
1416107_at	0.007561	1.03369	0.047804	1.03369	up	1.073309	0.102065	1.073309
1416121_at	0.049952	1.256414	0.329312	1.256414	up	1.307455	0.386761	1.307455
1416132_at	0.018399	1.110023	0.150589	1.110023	up	1.087802	0.121415	1.087802
1416160_at	0.034178	-1.02037	-0.0291	1.020374	down	-1.08877	-0.1227	1.088769

1416177_at	0.029051	-1.06051	-0.08476	1.060509	down	-1.0312	-0.04432	1.031197
1416261_at	0.005074	1.079632	0.110539	1.079632	up	1.176827	0.234903	1.176827
1416283_at	0.042129	1.166262	0.221891	1.166262	up	1.17705	0.235176	1.17705
1416307_at	0.024045	1.130828	0.17738	1.130828	up	1.195517	0.257634	1.195517
1416308_at	0.026026	-1.01511	-0.02164	1.015111	down	1.093306	0.128697	1.093306
1416315_at	0.033543	1.114173	0.155973	1.114173	up	1.088357	0.122152	1.088357
1416343_a_at	0.024122	1.057854	0.081141	1.057854	up	1.228062	0.296383	1.228062
1416344_at	0.00259	1.069641	0.097126	1.069641	up	1.219508	0.2863	1.219508
1416357_a_at	0.013238	1.256822	0.329781	1.256822	up	-1.00588	-0.00846	1.00588
1416402_at	0.021305	-1.11158	-0.15261	1.111578	down	-1.05215	-0.07334	1.052147
1416447_at	0.022343	-1.12653	-0.17188	1.126527	down	1.074867	0.104159	1.074867
1416470_a_at	0.041147	1.038647	0.054705	1.038647	up	1.031466	0.044696	1.031466
1416488_at	0.019167	-1.05682	-0.07973	1.056822	down	-1.01847	-0.0264	1.018467
1416492_at	0.008742	-1.05048	-0.07105	1.050479	down	1.040646	0.057479	1.040646
1416498_at	0.023277	1.074747	0.103997	1.074747	up	-1.22968	-0.29829	1.229683
1416501_at	0.014609	1.022794	0.032515	1.022794	up	1.040833	0.057739	1.040833
1416517_at	0.022784	1.146008	0.196617	1.146008	up	1.132989	0.180133	1.132989
1416548_at	0.041162	1.125563	0.170646	1.125563	up	1.236428	0.306178	1.236428
1416549_at	0.038794	1.079725	0.110664	1.079725	up	1.102261	0.140466	1.102261
1416568_a_at	0.041244	1.120847	0.164589	1.120847	up	1.015577	0.0223	1.015577
1416629_at	0.00103	-1.00474	-0.00682	1.00474	down	1.008283	0.0119	1.008283
1416692_at	0.01533	1.094048	0.129675	1.094048	up	1.208467	0.273178	1.208467
1416725_at	0.018688	-1.0035	-0.00504	1.0035	down	1.067421	0.094129	1.067421
1416740_at	0.033329	1.175964	0.233843	1.175964	up	-1.27736	-0.35316	1.277357
1416751_a_at	0.048723	-1.14389	-0.19394	1.143887	down	-1.05374	-0.07552	1.05374
1416773_at	6.01E-06	-1.02918	-0.0415	1.029182	down	1.127949	0.173702	1.127949
1416774_at	1.01E-04	-1.02867	-0.04077	1.028666	down	1.188389	0.249007	1.188389
1416791_a_at	0.037811	1.067647	0.094434	1.067647	up	1.086335	0.119469	1.086335
1416801_at	0.02289	1.098553	0.135604	1.098553	up	1.004742	0.006825	1.004742
1416808_at	0.01187	-1.08235	-0.11417	1.082352	down	-1.3185	-0.3989	1.318499
1416835_s_at	0.049144	1.039874	0.056408	1.039874	up	1.205471	0.269596	1.205471
1416855_at	0.009309	-1.04573	-0.06451	1.045731	down	-1.10595	-0.14529	1.105949
1416871_at	0.010676	1.02062	0.029445	1.02062	up	1.442476	0.528548	1.442476
1416873_a_at	0.0326	1.036673	0.051961	1.036673	up	1.222413	0.289732	1.222413
1416880_at	5.29E-04	-1.004	-0.00575	1.003997	down	-1.03445	-0.04886	1.034445
1416881_at	0.033237	-1.09631	-0.13266	1.09631	down	-1.14495	-0.19528	1.144947
1416901_at	0.027301	1.02194	0.03131	1.02194	up	1.100447	0.13809	1.100447
1416925_at	0.041369	-1.055	-0.07724	1.054998	down	-1.0214	-0.03055	1.021405
1416926_at	0.00416	1.140035	0.189079	1.140035	up	1.325094	0.406094	1.325094
1416928_at	0.04906	-1.04349	-0.06142	1.043494	down	-1.00892	-0.01281	1.008921
1416958_at	2.27E-04	1.02707	0.038534	1.02707	up	1.022347	0.031885	1.022347
1416959_at	0.009468	1.174341	0.231851	1.174341	up	1.047089	0.066384	1.047089
1416992_at	0.020328	1.223409	0.290907	1.223409	up	-1.00026	-3.71E-04	1.000257
1416993_at	0.023031	-1.01973	-0.02818	1.019728	down	1.063751	0.089161	1.063751
1417007_a_at	0.037889	-1.1123	-0.15355	1.1123	down	-1.04038	-0.05711	1.04038
1417015_at	0.008286	-1.06355	-0.08888	1.063546	down	-1.00783	-0.01126	1.007833
1417022_at	0.012784	1.134498	0.182054	1.134498	up	1.095366	0.131413	1.095366

1417060_at	0.032844	1.016526	0.023647	1.016526	up		1.078227	0.108661	1.078227
1417062_at	0.017494	1.056559	0.079373	1.056559	up		1.141689	0.19117	1.141689
1417086_at	0.01235	-1.04458	-0.06292	1.044578	down		1.056105	0.078754	1.056105
1417109_at	0.03712	1.129156	0.175245	1.129156	up		1.039001	0.055197	1.039001
1417130_s_at	0.01423	1.050264	0.070752	1.050264	up		-1.01176	-0.01686	1.011757
1417143_at	0.030346	-1.06216	-0.087	1.062162	down		-1.19574	-0.2579	1.195736
1417148_at	0.02734	1.219996	0.286876	1.219996	up		1.138378	0.18698	1.138378
1417155_at	0.030214	1.000088	1.26E-04	1.000088	up		-1.19555	-0.25767	1.195546
1417164_at	0.04273	-1.24195	-0.3126	1.241947	down		-1.28647	-0.36342	1.28647
1417180_at	0.02394	1.048981	0.068989	1.048981	up		1.03599	0.051009	1.03599
1417193_at	0.047347	1.011748	0.01685	1.011748	up		1.004406	0.006342	1.004406
1417199_at	0.009083	-1.09515	-0.13113	1.095148	down		1.026006	0.037039	1.026006
1417200_at	0.017258	1.020397	0.029131	1.020397	up		1.184659	0.244472	1.184659
1417239_at	0.006737	-1.07003	-0.09766	1.070034	down		-1.00602	-0.00866	1.006024
1417250_at	0.018074	1.0917	0.126576	1.0917	up		1.176039	0.233936	1.176039
1417257_at	0.016061	-1.2131	-0.2787	1.213101	down		-1.0987	-0.13579	1.098697
1417262_at	2.16E-04	-1.07264	-0.10117	1.072643	down		1.235584	0.305193	1.235584
1417283_at	0.031776	1.21694	0.283258	1.21694	up		1.054109	0.076024	1.054109
1417300_at	0.002168	1.211411	0.276689	1.211411	up		1.088731	0.122647	1.088731
1417324_at	0.02883	1.137533	0.185908	1.137533	up		1.121676	0.165656	1.121676
1417341_a_at	0.02153	-1.00951	-0.01365	1.009506	down		-1.01285	-0.01843	1.012854
1417383_at	0.001159	1.066403	0.092753	1.066403	up		1.10957	0.150001	1.10957
1417385_at	0.001447	-1.0131	-0.01877	1.013097	down		1.047976	0.067606	1.047976
1417386_at	0.010694	1.094532	0.130314	1.094532	up		1.094918	0.130823	1.094918
1417429_at	0.001043	1.1346	0.182184	1.1346	up		1.348863	0.431744	1.348863
1417443_at	0.003675	-1.0329	-0.0467	1.032898	down		1.001754	0.002529	1.001754
1417446_at	0.006362	1.071362	0.099446	1.071362	up		1.11278	0.154168	1.11278
1417453_at	0.014704	-1.1007	-0.13843	1.100705	down		1.062337	0.087242	1.062337
1417455_at	0.012734	1.093007	0.128303	1.093007	up		-1.02644	-0.03764	1.026436
1417461_at	0.02473	1.329889	0.411305	1.329889	up		1.112298	0.153543	1.112298
1417463_a_at	0.014869	-1.0988	-0.13593	1.0988	down		-1.00464	-0.00668	1.004642
1417464_at	0.018947	-1.01928	-0.02755	1.019281	down		-1.58888	-0.66801	1.588884
1417472_at	0.04292	1.090496	0.124985	1.090496	up		-1.04131	-0.0584	1.041308
1417488_at	0.014418	-1.06868	-0.09584	1.068685	down		1.065051	0.090923	1.065051
1417528_at	0.049699	1.068017	0.094934	1.068017	up		1.00016	2.31E-04	1.00016
1417555_at	0.030478	1.017647	0.025237	1.017647	up		1.134833	0.18248	1.134833
1417558_at	0.030332	1.119353	0.162665	1.119353	up		-1.07099	-0.09894	1.070987
1417568_at	0.02607	1.084178	0.116602	1.084178	up		1.143899	0.19396	1.143899
1417575_at	0.007742	1.074742	0.10399	1.074742	up		1.136946	0.185164	1.136946
1417585_at	0.028229	-1.03115	-0.04425	1.031147	down		-1.27705	-0.35282	1.27705
1417592_at	0.001867	-1.00767	-0.01103	1.007675	down		-1.14036	-0.18949	1.140363
1417601_at	0.03334	-1.07143	-0.09953	1.071425	down		1.709805	0.773832	1.709805
1417602_at	2.63E-04	-1.05959	-0.0835	1.059588	down		1.069647	0.097134	1.069647
1417618_at	0.032946	1.141744	0.191239	1.141744	up		1.070086	0.097727	1.070086
1417623_at	0.032588	-1.17544	-0.2332	1.175443	down		-1.12131	-0.16518	1.121309
1417638_at	8.05E-04	1.166295	0.221933	1.166295	up		1.027868	0.039654	1.027868
1417644_at	0.020013	1.117682	0.16051	1.117682	up		1.072122	0.100469	1.072122

1417649_at	0.049805	1.009557	0.013723	1.009557	up	1.040557	0.057356	1.040557
1417653_at	0.009532	1.129057	0.175118	1.129057	up	-1.79419	-0.84333	1.794189
1417655_a_at	0.011796	1.151539	0.203563	1.151539	up	1.208059	0.272691	1.208059
1417667_a_at	0.030353	-1.02338	-0.03334	1.023379	down	1.117034	0.159673	1.117034
1417668_at	0.034314	1.129766	0.176023	1.129766	up	1.233976	0.303314	1.233976
1417683_at	0.026513	1.071325	0.099396	1.071325	up	1.141936	0.191481	1.141936
1417692_at	0.043559	1.201346	0.264652	1.201346	up	1.113473	0.155066	1.113473
1417696_at	0.004991	1.047684	0.067203	1.047684	up	1.182926	0.242359	1.182926
1417697_at	0.01727	1.211637	0.276957	1.211637	up	1.173235	0.230491	1.173235
1417721_s_at	0.031098	1.05939	0.083234	1.05939	up	-1.06005	-0.08414	1.060055
1417730_at	0.029511	1.008039	0.011551	1.008039	up	1.155715	0.208785	1.155715
1417757_at	0.024878	1.183638	0.243228	1.183638	up	1.12181	0.165828	1.12181
1417766_at	0.021109	1.098192	0.13513	1.098192	up	1.08132	0.112794	1.08132
1417781_at	0.042899	1.024519	0.034947	1.024519	up	1.093678	0.129188	1.093678
1417785_at	0.009624	1.116224	0.158626	1.116224	up	1.25655	0.329468	1.25655
1417787_at	0.044795	-1.00277	-0.00399	1.00277	down	-1.08703	-0.12039	1.087028
1417789_at	0.001589	-1.27799	-0.35388	1.277993	down	-1.21908	-0.28579	1.219081
1417810_a_at	0.002694	1.088214	0.121963	1.088214	up	1.064324	0.089938	1.064324
1417832_at	0.029232	-1.04242	-0.05993	1.042417	down	1.00643	0.009247	1.00643
1417860_a_at	0.016042	-1.1657	-0.2212	1.1657	down	-1.0925	-0.12763	1.092496
1417869_s_at	0.026777	-1.02517	-0.03586	1.025167	down	-1.11662	-0.15913	1.116616
1417871_at	0.011942	1.017355	0.024823	1.017355	up	1.386353	0.471295	1.386353
1417907_at	0.020149	1.000464	6.69E-04	1.000464	up	1.081399	0.112899	1.081399
1417908_s_at	0.008531	1.037777	0.053496	1.037777	up	1.131188	0.177839	1.131188
1417925_at	0.044661	-1.09691	-0.13344	1.096906	down	-1.31797	-0.39832	1.317971
1417960_at	0.048807	1.182323	0.241624	1.182323	up	1.108902	0.149131	1.108902
1417964_at	0.039429	1.006164	0.008866	1.006164	up	1.081641	0.113221	1.081641
1418003_at	0.031669	1.250358	0.322341	1.250358	up	1.301237	0.379884	1.301237
1418025_at	0.034141	1.22074	0.287756	1.22074	up	1.287443	0.364509	1.287443
1418118_at	0.04221	1.156197	0.209388	1.156197	up	1.062419	0.087353	1.062419
1418120_at	0.02843	-1.07563	-0.10518	1.075629	down	-1.05999	-0.08405	1.05999
1418174_at	0.010533	1.171866	0.228807	1.171866	up	-1.032	-0.04544	1.032001
1418178_at	0.047148	1.377855	0.462424	1.377855	up	1.207352	0.271846	1.207352
1418201_at	0.02523	1.102569	0.140869	1.102569	up	1.151583	0.203618	1.151583
1418230_a_at	0.047612	-1.03972	-0.05619	1.039717	down	1.074642	0.103856	1.074642
1418231_at	0.00853	-1.10507	-0.14414	1.105071	down	1.308365	0.387765	1.308365
1418275_a_at	0.013771	1.159389	0.213365	1.159389	up	1.059146	0.082901	1.059146
1418280_at	0.04892	1.056767	0.079657	1.056767	up	1.003802	0.005475	1.003802
1418285_at	0.034705	1.042162	0.05958	1.042162	up	-1.03529	-0.05004	1.035294
1418286_a_at	0.006957	-1.11511	-0.15718	1.115107	down	-1.04279	-0.06045	1.04279
1418293_at	0.002929	-1.01003	-0.0144	1.010032	down	1.010485	0.015047	1.010485
1418314_a_at	0.007529	1.03422	0.048544	1.03422	up	-1.68467	-0.75247	1.684674
1418319_at	0.006802	-1.06105	-0.08549	1.061046	down	1.056891	0.079826	1.056891
1418326_at	0.004965	1.120418	0.164036	1.120418	up	1.831225	0.872809	1.831225
1418336_at	7.47E-05	1.080185	0.111279	1.080185	up	1.263629	0.337573	1.263629
1418392_a_at	0.005132	-1.31208	-0.39185	1.312076	down	-1.29972	-0.3782	1.299724
1418409_at	0.025058	-1.03191	-0.04532	1.031913	down	1.081778	0.113405	1.081778

1418416_x_at	0.046404	-1.057	-0.07998	1.057001	down	1.162442	0.217159	1.162442
1418421_at	0.033747	1.075654	0.105213	1.075654	up	-1.09037	-0.12481	1.090366
1418436_at	0.005387	-1.02819	-0.04011	1.028191	down	1.008957	0.012864	1.008957
1418441_at	0.004911	-1.03385	-0.04803	1.033852	down	1.095368	0.131415	1.095368
1418442_at	0.025082	1.035824	0.050778	1.035824	up	1.118587	0.161678	1.118587
1418453_a_at	0.045523	1.082255	0.114041	1.082255	up	1.393791	0.479014	1.393791
1418470_at	0.002038	-1.12409	-0.16875	1.124088	down	-1.20188	-0.26529	1.201878
1418479_at	0.026511	-1.02099	-0.02996	1.020987	down	-1.02779	-0.03955	1.027791
1418493_a_at	0.034637	1.133043	0.180203	1.133043	up	1.119409	0.162737	1.119409
1418511_at	0.010883	-1.21195	-0.27733	1.211948	down	-2.88442	-1.52828	2.884421
1418566_s_at	0.046855	-1.23477	-0.30425	1.234773	down	-1.01757	-0.02513	1.017569
1418592_at	0.001482	-1.0831	-0.11517	1.083104	down	-1.02408	-0.03432	1.024077
1418603_at	0.041489	-1.06813	-0.09509	1.068134	down	-1.16775	-0.22374	1.167754
1418604_at	0.008103	-1.02505	-0.03569	1.025049	down	1.128052	0.173833	1.128052
1418651_at	0.023935	-1.03318	-0.04709	1.033179	down	1.143058	0.192899	1.143058
1418659_at	8.36E-04	-1.03354	-0.04759	1.033535	down	1.053888	0.075721	1.053888
1418660_at	7.86E-04	1.022204	0.031684	1.022204	up	1.063113	0.088295	1.063113
1418670_s_at	0.031952	1.002863	0.004124	1.002863	up	-1.20924	-0.27411	1.209244
1418697_at	1.28E-05	1.338265	0.420364	1.338265	up	1.085571	0.118453	1.085571
1418712_at	0.031027	1.124559	0.169359	1.124559	up	-1.16093	-0.21528	1.16093
1418719_at	0.006554	1.041276	0.058352	1.041276	up	-1.05927	-0.08306	1.059265
1418733_at	0.04727	-1.18968	-0.25057	1.189675	down	-1.25686	-0.32983	1.256862
1418742_at	0.020035	-1.26487	-0.33899	1.264867	down	-1.11544	-0.15761	1.115439
1418758_a_at	0.012446	1.067928	0.094815	1.067928	up	1.242173	0.312866	1.242173
1418812_a_at	0.047048	-1.16308	-0.21795	1.163078	down	-1.25657	-0.3295	1.256574
1418865_at	0.039758	1.022072	0.031497	1.022072	up	1.048531	0.068369	1.048531
1418873_at	0.029142	1.091746	0.126637	1.091746	up	1.16085	0.215182	1.16085
1418889_a_at	0.007255	1.154195	0.206888	1.154195	up	1.106126	0.145515	1.106126
1418901_at	0.005952	-1.09758	-0.13432	1.097578	down	1.00371	0.005342	1.00371
1418932_at	6.40E-04	1.030065	0.042735	1.030065	up	1.363305	0.447109	1.363305
1418937_at	0.027567	-1.07957	-0.11046	1.07957	down	-1.42806	-0.51405	1.428058
1418942_at	0.013112	-1.06494	-0.09077	1.06494	down	1.099043	0.136247	1.099043
1418954_at	0.049995	1.036386	0.051562	1.036386	up	-1.07882	-0.10946	1.078822
1418990_at	0.042143	-1.39654	-0.48185	1.396536	down	-1.1865	-0.24671	1.186498
1419028_at	8.76E-05	-1.08216	-0.11392	1.082162	down	-1.66449	-0.73508	1.664494
1419039_at	0.009035	1.258806	0.332055	1.258806	up	1.411035	0.496754	1.411035
1419040_at	0.043737	1.232224	0.301265	1.232224	up	1.375056	0.45949	1.375056
1419053_at	0.049082	1.093091	0.128413	1.093091	up	1.097639	0.134404	1.097639
1419054_a_at	0.023618	1.053221	0.074808	1.053221	up	1.099199	0.136453	1.099199
1419070_at	4.94E-04	1.070416	0.098171	1.070416	up	-1.05005	-0.07046	1.050049
1419078_at	0.037016	-1.05953	-0.08342	1.059527	down	-1.03304	-0.0469	1.033041
1419079_at	0.038138	-1.02237	-0.03192	1.022372	down	-1.09182	-0.12673	1.091816
1419115_at	0.001012	1.045781	0.064581	1.045781	up	1.268635	0.343277	1.268635
1419123_a_at	0.014423	-1.00507	-0.0073	1.005074	down	1.239423	0.309669	1.239423
1419124_at	0.021083	-1.15576	-0.20884	1.155761	down	-1.30459	-0.3836	1.304592
1419144_at	0.039768	-1.29806	-0.37635	1.298057	down	1.134756	0.182381	1.134756
1419146_a_at	0.005978	1.004072	0.005862	1.004072	up	-1.01896	-0.02709	1.018956

1419147_at	0.015323	-1.17642	-0.23441	1.176423	down	1.027131	0.03862	1.027131
1419168_at	0.028921	-1.10102	-0.13884	1.101018	down	-1.08909	-0.12312	1.089088
1419184_a_at	0.040197	1.034604	0.049079	1.034604	up	1.071157	0.099169	1.071157
1419188_s_at	0.026812	1.102694	0.141032	1.102694	up	1.024891	0.03547	1.024891
1419255_at	0.0162	1.266748	0.341129	1.266748	up	1.038037	0.053857	1.038037
1419262_at	0.047515	1.048554	0.068401	1.048554	up	1.234113	0.303474	1.234113
1419353_at	0.032456	1.001688	0.002433	1.001688	up	1.090117	0.124483	1.090117
1419358_at	0.025571	1.277636	0.353477	1.277636	up	1.259338	0.332665	1.259338
1419376_at	2.15E-05	1.212851	0.278402	1.212851	up	-1.05486	-0.07705	1.054857
1419377_at	0.0076	-1.04472	-0.06311	1.044718	down	1.101436	0.139386	1.101436
1419421_at	0.00269	1.02372	0.033821	1.02372	up	-1.26157	-0.33521	1.261565
1419448_at	0.036757	-1.20854	-0.27326	1.208535	down	-1.04373	-0.06175	1.043734
1419467_at	0.023342	1.264634	0.33872	1.264634	up	-1.05997	-0.08402	1.059969
1419482_at	0.002674	-1.0436	-0.06157	1.043598	down	-1.11953	-0.1629	1.119532
1419508_at	0.045168	-1.01572	-0.0225	1.015717	down	-1.05588	-0.07845	1.055882
1419530_at	0.030554	-1.36141	-0.44511	1.361413	down	1.004196	0.006041	1.004196
1419545_a_at	0.036249	-1.08055	-0.11176	1.080548	down	1.080344	0.111491	1.080344
1419558_at	0.039451	1.144618	0.194867	1.144618	up	1.003211	0.004625	1.003211
1419567_at	0.002704	-1.29403	-0.37187	1.294033	down	-1.08379	-0.11608	1.083789
1419572_a_at	0.033933	1.018693	0.026719	1.018693	up	-1.01074	-0.01541	1.010741
1419574_at	0.043533	1.225342	0.293185	1.225342	up	1.347019	0.429771	1.347019
1419594_at	0.045681	-1.35053	-0.43352	1.350529	down	-1.20535	-0.26945	1.205352
1419603_at	0.009697	-1.68173	-0.74995	1.68173	down	-1.81399	-0.85916	1.813988
1419621_at	0.029156	1.012035	0.017259	1.012035	up	-1.12585	-0.17101	1.12585
1419648_at	0.044226	1.040081	0.056695	1.040081	up	-1.05498	-0.07721	1.054976
1419702_at	0.021775	-1.02505	-0.03569	1.025047	down	-1.04683	-0.06603	1.046835
1419739_at	0.006598	1.052779	0.074203	1.052779	up	-1.80272	-0.85018	1.802722
1419764_at	0.009317	1.23904	0.309223	1.23904	up	4.859326	2.280756	4.859326
1419879_s_at	0.024445	1.003085	0.004444	1.003085	up	1.028735	0.040872	1.028735
1419888_at	0.009577	1.095991	0.132236	1.095991	up	-1.19105	-0.25223	1.191048
1420054_s_at	0.016743	-1.03959	-0.05601	1.03959	down	-1.05308	-0.07462	1.053082
1420123_at	0.041831	1.059472	0.083345	1.059472	up	1.22623	0.29423	1.22623
1420389_at	0.020149	-1.28367	-0.36028	1.283675	down	-1.1903	-0.25132	1.190299
1420397_a_at	0.035725	1.174948	0.232597	1.174948	up	1.045036	0.063552	1.045036
1420401_a_at	0.027832	1.317665	0.397983	1.317665	up	1.040435	0.057187	1.040435
1420465_s_at	0.034193	-1.31706	-0.39732	1.317062	down	-4.47883	-2.16312	4.478828
1420477_at	0.025999	-1.03716	-0.05263	1.037157	down	1.077045	0.107079	1.077045
1420491_at	0.039531	-1.0978	-0.13462	1.097802	down	-1.17686	-0.23494	1.176858
1420512_at	0.041401	1.091967	0.126929	1.091967	up	-1.09257	-0.12773	1.092574
1420522_at	0.012965	-1.13806	-0.18658	1.138061	down	-1.09436	-0.13009	1.094364
1420523_at	0.03872	-1.08335	-0.11549	1.083345	down	-1.09597	-0.13221	1.095974
1420526_at	0.012126	-1.0633	-0.08854	1.063297	down	1.171599	0.228479	1.171599
1420543_at	0.031525	1.127941	0.173691	1.127941	up	-1.07433	-0.10344	1.074333
1420584_at	0.039035	-1.22191	-0.28914	1.221911	down	-1.09324	-0.1286	1.093235
1420620_a_at	0.024465	1.043384	0.061271	1.043384	up	1.273472	0.348768	1.273472
1420631_a_at	0.041732	1.050613	0.071231	1.050613	up	1.001554	0.00224	1.001554
1420671_x_at	0.003914	-1.40329	-0.48881	1.403291	down	-1.33053	-0.412	1.330532

1420696_at	0.012222	1.049787	0.070096	1.049787	up	1.247778	0.319361	1.247778
1420719_at	0.017704	1.005827	0.008382	1.005827	up	1.008817	0.012664	1.008817
1420763_at	0.041665	-1.05522	-0.07754	1.055219	down	-1.01284	-0.0184	1.012837
1420772_a_at	8.14E-04	1.289225	0.366504	1.289225	up	1.53713	0.620239	1.53713
1420778_at	0.032539	1.06211	0.086933	1.06211	up	1.160642	0.214923	1.160642
1420797_at	0.010233	-1.43913	-0.52519	1.439126	down	-1.14881	-0.20015	1.148814
1420896_at	0.012732	1.064418	0.090065	1.064418	up	1.066775	0.093255	1.066775
1420907_at	0.037182	-1.006	-0.00864	1.006004	down	1.05577	0.078296	1.05577
1420913_at	0.024938	1.203548	0.267294	1.203548	up	1.140752	0.189986	1.140752
1420936_s_at	0.03387	-1.03437	-0.04876	1.034373	down	1.061085	0.08554	1.061085
1420946_at	0.005865	1.056379	0.079127	1.056379	up	-1.03563	-0.05051	1.035628
1420960_at	0.030769	-1.04042	-0.05716	1.040416	down	1.000776	0.001119	1.000776
1420971_at	0.00963	1.016087	0.023024	1.016087	up	1.019247	0.027503	1.019247
1421027_a_at	0.019667	-1.1975	-0.26002	1.197497	down	-1.42624	-0.51222	1.426239
1421037_at	1.40E-05	-1.18277	-0.24217	1.182772	down	-1.19878	-0.26157	1.198781
1421045_at	0.048958	1.109602	0.150042	1.109602	up	-1.19172	-0.25305	1.191721
1421048_a_at	0.001094	-1.12439	-0.16915	1.124395	down	-1.18173	-0.2409	1.181727
1421064_at	0.041861	-1.03522	-0.04993	1.035217	down	1.180261	0.239106	1.180261
1421074_at	0.004079	-1.07373	-0.10263	1.073728	down	1.173511	0.230832	1.173511
1421075_s_at	8.64E-04	-1.04184	-0.05914	1.041843	down	1.116057	0.158411	1.116057
1421087_at	5.30E-06	1.092183	0.127215	1.092183	up	-1.02533	-0.03609	1.025331
1421103_at	0.004658	1.302386	0.381157	1.302386	up	1.232478	0.301562	1.232478
1421113_at	0.008893	1.026312	0.03747	1.026312	up	1.063574	0.08892	1.063574
1421114_a_at	0.002079	-1.19739	-0.2599	1.197392	down	-1.88078	-0.91133	1.880783
1421127_at	0.029701	-1.05019	-0.07065	1.050192	down	-1.0398	-0.05631	1.039803
1421136_at	0.025499	1.076236	0.105994	1.076236	up	-1.0732	-0.10192	1.073203
1421144_at	0.009349	1.015426	0.022085	1.015426	up	1.034153	0.04845	1.034153
1421163_a_at	0.021958	-1.18086	-0.23984	1.180858	down	-1.18523	-0.24517	1.185232
1421166_at	0.002422	1.053831	0.075644	1.053831	up	1.036459	0.051662	1.036459
1421189_at	0.019465	-1.19903	-0.26187	1.19903	down	1.01859	0.026574	1.01859
1421198_at	0.003265	-1.18494	-0.24482	1.184942	down	1.034942	0.04955	1.034942
1421204_a_at	0.029152	1.006137	0.008827	1.006137	up	1.068048	0.094977	1.068048
1421211_a_at	0.037453	-1.04033	-0.05704	1.040326	down	-1.0837	-0.11597	1.083704
1421239_at	0.043516	-1.17804	-0.23638	1.178036	down	-1.06318	-0.08838	1.063179
1421256_at	2.15E-04	1.060943	0.085347	1.060943	up	-1.07287	-0.10148	1.072872
1421265_a_at	0.032441	1.028032	0.039886	1.028032	up	-1.16357	-0.21856	1.163575
1421303_at	0.035156	-1.19546	-0.25757	1.195464	down	-1.10953	-0.14994	1.109527
1421335_a_at	0.046737	1.108373	0.148443	1.108373	up	1.077006	0.107026	1.077006
1421358_at	0.019793	1.078811	0.109442	1.078811	up	-1.0836	-0.11583	1.083596
1421378_s_at	0.01599	1.02081	0.029715	1.02081	up	1.216964	0.283287	1.216964
1421486_at	0.008562	1.105902	0.145223	1.105902	up	-1.0282	-0.04012	1.028199
1421535_a_at	0.014005	-1.3461	-0.42878	1.346099	down	-1.08539	-0.11821	1.085389
1421560_at	0.032646	-1.22104	-0.28811	1.221042	down	-1.02966	-0.04217	1.02966
1421727_at	0.008232	-1.02605	-0.0371	1.026051	down	1.061258	0.085776	1.061258
1421735_a_at	0.015199	1.107446	0.147236	1.107446	up	-1.09821	-0.13516	1.098212
1421739_a_at	0.018817	1.084225	0.116664	1.084225	up	-1.19253	-0.25402	1.19253
1421809_at	8.66E-04	1.118424	0.161467	1.118424	up	1.131555	0.178307	1.131555

1421814_at	0.040296	-1.03523	-0.04995	1.035231	down	1.066128	0.092381	1.066128
1421831_at	0.044627	-1.06296	-0.08809	1.062962	down	-1.13284	-0.17994	1.132837
1421848_at	0.019244	1.170113	0.226647	1.170113	up	1.204205	0.268082	1.204205
1421868_a_at	0.00399	-1.2432	-0.31405	1.243196	down	-1.16551	-0.22096	1.165508
1421886_at	0.027207	-1.11286	-0.15428	1.112864	down	-1.2183	-0.28487	1.218301
1421908_a_at	0.022417	1.046551	0.065643	1.046551	up	1.096254	0.132581	1.096254
1421939_a_at	0.008286	1.09151	0.126326	1.09151	up	-1.00422	-0.00608	1.004224
1421943_at	0.044179	-1.04393	-0.06202	1.043925	down	1.022347	0.031885	1.022347
1422017_s_at	0.02165	-1.00547	-0.00787	1.00547	down	-1.1336	-0.18091	1.133601
1422018_at	0.002656	1.048962	0.068962	1.048962	up	1.039198	0.055471	1.039198
1422021_at	0.007846	1.042726	0.06036	1.042726	up	1.085354	0.118166	1.085354
1422026_at	0.025401	-1.33451	-0.41631	1.334513	down	-1.99296	-0.99491	1.992961
1422060_at	0.02814	-1.08853	-0.12239	1.088533	down	-1.03967	-0.05612	1.039667
1422069_at	0.045866	-1.16383	-0.21888	1.163833	down	-1.05922	-0.08301	1.059224
1422072_a_at	0.002027	1.066883	0.093402	1.066883	up	-1.05948	-0.08336	1.05948
1422095_a_at	0.013383	-1.08251	-0.11438	1.082507	down	-1.15351	-0.20603	1.15351
1422136_at	0.011589	-1.0671	-0.09369	1.067096	down	-1.05072	-0.07138	1.050723
1422155_at	0.001845	-1.13168	-0.17847	1.13168	down	1.027716	0.039442	1.027716
1422161_at	0.041021	-1.17182	-0.22875	1.17182	down	-1.36919	-0.45332	1.369185
1422168_a_at	0.016729	-1.34238	-0.42479	1.342377	down	-1.12022	-0.16378	1.12022
1422177_at	0.049146	-1.12129	-0.16516	1.121288	down	1.320609	0.401203	1.320609
1422186_s_at	0.001845	1.123348	0.167805	1.123348	up	1.005475	0.007878	1.005475
1422195_s_at	0.022754	1.094536	0.13032	1.094536	up	-1.28788	-0.365	1.287883
1422234_at	0.048173	1.044546	0.062876	1.044546	up	-1.02683	-0.03819	1.026827
1422243_at	0.003061	-1.1196	-0.16299	1.119602	down	-1.15744	-0.21094	1.157445
1422253_at	0.033369	1.676578	0.74552	1.676578	up	1.645291	0.718343	1.645291
1422266_at	0.010603	1.132044	0.17893	1.132044	up	-1.01991	-0.02844	1.019911
1422285_at	0.015888	-1.00956	-0.01372	1.009559	down	-1.10581	-0.14511	1.105813
1422401_at	0.019231	1.070338	0.098066	1.070338	up	1.010629	0.015253	1.010629
1422437_at	0.014805	1.336277	0.418219	1.336277	up	-1.04166	-0.05889	1.041663
1422438_at	0.04752	1.231832	0.300805	1.231832	up	1.247752	0.319331	1.247752
1422480_at	0.027442	1.088109	0.121823	1.088109	up	1.060062	0.084149	1.060062
1422489_at	0.011797	-1.01025	-0.01471	1.010251	down	-1.109	-0.14926	1.108998
1422497_at	0.019196	-1.09868	-0.13577	1.098682	down	1.078816	0.109449	1.078816
1422532_at	0.027327	1.087108	0.120495	1.087108	up	1.028856	0.04104	1.028856
1422571_at	0.032072	1.248175	0.31982	1.248175	up	-1.02685	-0.03822	1.026846
1422587_at	2.36E-04	1.067706	0.094515	1.067706	up	1.190741	0.251859	1.190741
1422598_at	0.043432	1.134584	0.182163	1.134584	up	-1.43521	-0.52126	1.435212
1422606_at	0.017217	2.49336	1.318091	2.49336	up	-1.01295	-0.01856	1.012947
1422607_at	0.035673	1.029404	0.04181	1.029404	up	-1.04395	-0.06205	1.043951
1422632_at	0.037	1.443076	0.529147	1.443076	up	1.215324	0.281341	1.215324
1422660_at	5.85E-04	1.083312	0.115449	1.083312	up	1.097364	0.134042	1.097364
1422665_a_at	0.049506	-1.01613	-0.02309	1.016133	down	1.073953	0.102931	1.073953
1422680_at	0.023781	-1.10673	-0.1463	1.106725	down	-1.11364	-0.15528	1.113637
1422692_at	0.005046	1.061906	0.086656	1.061906	up	1.110146	0.150749	1.110146
1422741_a_at	0.041984	1.132191	0.179117	1.132191	up	-1.16756	-0.2235	1.167562
1422799_at	0.00676	1.067906	0.094784	1.067906	up	1.077022	0.107048	1.077022

1422815_at	0.049098	-1.13843	-0.18704	1.138427	down	-1.13924	-0.18807	1.139241
1422821_s_at	0.007904	1.034674	0.049177	1.034674	up	-1.02342	-0.03339	1.023417
1422827_x_at	0.037849	1.087403	0.120887	1.087403	up	1.214208	0.280016	1.214208
1422857_at	0.048593	-1.02679	-0.03814	1.02679	down	1.083802	0.116101	1.083802
1422882_at	0.034431	1.086977	0.120321	1.086977	up	1.048392	0.068178	1.048392
1422896_at	0.018407	1.070673	0.098518	1.070673	up	-1.00392	-0.00564	1.003916
1422905_s_at	0.012862	1.010561	0.015157	1.010561	up	1.106555	0.146075	1.106555
1422965_at	0.03368	1.105517	0.144721	1.105517	up	1.163814	0.21886	1.163814
1422971_at	0.021781	-1.11515	-0.15723	1.115146	down	-1.07894	-0.10961	1.07894
1423057_at	0.038649	1.022044	0.031457	1.022044	up	1.031875	0.045268	1.031875
1423059_at	0.032471	1.021807	0.031123	1.021807	up	1.164866	0.220164	1.164866
1423072_at	0.048803	1.028498	0.040538	1.028498	up	-1.14112	-0.19045	1.14112
1423074_at	0.004841	1.051055	0.071838	1.051055	up	1.115584	0.157799	1.115584
1423076_at	0.012454	-1.02072	-0.02959	1.020721	down	1.128145	0.173952	1.128145
1423078_a_at	0.038307	1.206752	0.27113	1.206752	up	1.866731	0.900514	1.866731
1423088_at	0.010155	1.068412	0.095469	1.068412	up	1.032349	0.045931	1.032349
1423093_at	0.007185	1.037146	0.052619	1.037146	up	-1.05438	-0.07639	1.054377
1423094_at	0.022325	1.080228	0.111335	1.080228	up	1.205329	0.269427	1.205329
1423095_s_at	0.016209	-1.04092	-0.05786	1.040921	down	1.125753	0.17089	1.125753
1423110_at	0.009612	1.458377	0.544364	1.458377	up	-1.00933	-0.01339	1.009326
1423145_a_at	0.002716	1.033725	0.047853	1.033725	up	-1.52745	-0.61113	1.527455
1423166_at	0.045416	1.007571	0.010882	1.007571	up	-1.0544	-0.07642	1.054398
1423184_at	0.001297	-1.02801	-0.03986	1.028012	down	-1.2163	-0.2825	1.216297
1423204_at	0.042581	1.041297	0.058382	1.041297	up	1.191567	0.25286	1.191567
1423207_at	0.038424	-1.04273	-0.06037	1.042731	down	1.034528	0.048973	1.034528
1423228_at	0.009672	-1.05426	-0.07623	1.054258	down	1.212027	0.277422	1.212027
1423238_at	0.025474	-1.06274	-0.08779	1.062743	down	-1.75418	-0.8108	1.754184
1423244_at	0.007527	-1.28071	-0.35694	1.28071	down	-1.20501	-0.26905	1.205015
1423312_at	0.03671	-1.01925	-0.02751	1.019249	down	-1.20948	-0.27439	1.20948
1423319_at	0.009224	1.010453	0.015003	1.010453	up	-1.05646	-0.07924	1.056459
1423320_at	0.02599	1.068705	0.095863	1.068705	up	1.053845	0.075662	1.053845
1423321_at	0.010472	1.074178	0.103232	1.074178	up	-1.03898	-0.05517	1.038984
1423343_at	0.013805	1.098678	0.135768	1.098678	up	1.394781	0.480038	1.394781
1423365_at	0.040543	1.088617	0.122497	1.088617	up	-1.05698	-0.07994	1.056976
1423407_a_at	0.004536	1.41331	0.499077	1.41331	up	1.093253	0.128628	1.093253
1423414_at	0.001939	1.225107	0.292908	1.225107	up	1.141154	0.190494	1.141154
1423434_at	0.021618	-1.01896	-0.02709	1.018955	down	1.118668	0.161782	1.118668
1423456_at	0.015265	1.099592	0.136969	1.099592	up	-1.11692	-0.15953	1.116923
1423460_at	0.042348	1.0875	0.121016	1.0875	up	1.080194	0.11129	1.080194
1423468_at	3.94E-04	-1.16854	-0.22471	1.168539	down	-1.54687	-0.62935	1.546873
1423503_at	0.018534	1.038497	0.054496	1.038497	up	1.240145	0.310509	1.240145
1423543_at	0.043779	1.010734	0.015404	1.010734	up	1.008096	0.011633	1.008096
1423563_at	0.026869	1.122485	0.166696	1.122485	up	-1.0315	-0.04475	1.031502
1423571_at	0.00726	1.098716	0.135819	1.098716	up	1.245204	0.316382	1.245204
1423594_a_at	0.041342	1.121161	0.164994	1.121161	up	1.070311	0.09803	1.070311
1423596_at	0.047743	1.070651	0.098488	1.070651	up	1.317813	0.398146	1.317813
1423606_at	0.044076	1.375094	0.459531	1.375094	up	-1.58711	-0.6664	1.587111

1423611_at	0.025521	1.207902	0.272504	1.207902	up	-1.00104	-0.0015	1.001039
1423628_s_at	0.020272	1.131976	0.178843	1.131976	up	1.057269	0.080342	1.057269
1423650_at	0.021494	1.129472	0.175649	1.129472	up	1.199608	0.262563	1.199608
1423669_at	0.020437	1.491997	0.577244	1.491997	up	-1.31023	-0.38982	1.310231
1423680_at	0.024498	1.130576	0.177058	1.130576	up	1.304382	0.383366	1.304382
1423694_at	0.018837	1.345818	0.428483	1.345818	up	1.308131	0.387507	1.308131
1423707_at	0.012224	1.05758	0.080767	1.05758	up	1.054896	0.077101	1.054896
1423719_at	0.010188	-1.1335	-0.18079	1.133505	down	-1.15858	-0.21235	1.158576
1423738_at	0.006779	-1.03831	-0.05424	1.038309	down	1.125985	0.171187	1.125985
1423741_at	0.004796	1.11208	0.153261	1.11208	up	1.14232	0.191967	1.14232
1423750_a_at	0.041898	1.110357	0.151024	1.110357	up	1.059194	0.082967	1.059194
1423765_at	0.026882	1.028011	0.039855	1.028011	up	1.003443	0.004959	1.003443
1423769_at	0.015821	1.037206	0.052702	1.037206	up	1.06255	0.087531	1.06255
1423777_at	0.006086	1.030167	0.042878	1.030167	up	1.178279	0.236681	1.178279
1423778_at	0.042234	-1.16811	-0.22417	1.168107	down	-1.04369	-0.0617	1.043695
1423805_at	0.01357	1.190118	0.251105	1.190118	up	1.037312	0.05285	1.037312
1423810_at	0.027333	1.079306	0.110103	1.079306	up	1.105645	0.144888	1.105645
1423824_at	0.035855	-1.05113	-0.07195	1.051134	down	1.197964	0.260585	1.197964
1423825_at	0.035758	-1.04218	-0.0596	1.04218	down	1.202384	0.265897	1.202384
1423838_s_at	0.027489	1.109873	0.150395	1.109873	up	1.103527	0.142122	1.103527
1423883_at	0.001821	-1.07555	-0.10508	1.075552	down	-1.30697	-0.38623	1.306971
1423886_at	0.002745	-1.06708	-0.09366	1.067076	down	-1.20096	-0.26418	1.200957
1423943_at	0.025382	1.011495	0.016489	1.011495	up	1.040267	0.056953	1.040267
1424033_at	0.013438	1.027204	0.038722	1.027204	up	1.025477	0.036295	1.025477
1424038_a_at	0.009135	1.16956	0.225966	1.16956	up	1.265873	0.340132	1.265873
1424044_at	0.020806	1.193343	0.255008	1.193343	up	1.042371	0.059869	1.042371
1424050_s_at	0.019581	1.012638	0.018118	1.012638	up	-1.0024	-0.00345	1.002397
1424056_at	0.044204	1.127787	0.173495	1.127787	up	1.094326	0.130042	1.094326
1424076_at	0.025371	1.165716	0.221217	1.165716	up	1.330542	0.412014	1.330542
1424095_at	0.033725	1.059229	0.083014	1.059229	up	1.171974	0.22894	1.171974
1424131_at	0.011971	1.30511	0.384172	1.30511	up	-1.14879	-0.20011	1.148789
1424147_at	0.006007	1.052951	0.074438	1.052951	up	1.095997	0.132244	1.095997
1424175_at	0.001917	1.077346	0.107481	1.077346	up	1.040929	0.057872	1.040929
1424185_a_at	0.010113	1.139337	0.188195	1.139337	up	1.302434	0.381211	1.302434
1424196_at	0.00745	1.096749	0.133233	1.096749	up	1.224117	0.291742	1.224117
1424217_at	0.021813	1.09189	0.126828	1.09189	up	1.035977	0.050992	1.035977
1424223_at	0.018025	1.024775	0.035307	1.024775	up	-1.00416	-0.00599	1.004161
1424228_at	0.01394	-1.06197	-0.08674	1.061967	down	-1.03128	-0.04443	1.031276
1424229_at	0.024004	1.054818	0.076994	1.054818	up	1.077997	0.108353	1.077997
1424233_at	9.29E-05	-1.37983	-0.4645	1.379835	down	-1.71057	-0.77448	1.710568
1424234_s_at	0.002532	-1.42991	-0.51593	1.429914	down	-1.85234	-0.88935	1.852342
1424253_at	9.65E-04	-1.00251	-0.00362	1.00251	down	1.110535	0.151254	1.110535
1424267_at	0.016838	1.003296	0.004747	1.003296	up	1.145369	0.195812	1.145369
1424284_at	0.047555	1.028796	0.040956	1.028796	up	1.024612	0.035078	1.024612
1424287_at	0.00486	1.036929	0.052318	1.036929	up	-1.14871	-0.20001	1.14871
1424336_at	0.002738	-1.16543	-0.22087	1.165434	down	-1.08415	-0.11656	1.08415
1424373_at	0.028263	1.064584	0.09029	1.064584	up	1.154473	0.207234	1.154473

1424395_at	0.019096	-1.06562	-0.0917	1.065624	down	-1.04335	-0.06122	1.043345
1424400_a_at	0.029881	1.08623	0.11933	1.08623	up	1.508485	0.5931	1.508485
1424438_a_at	0.0407	1.069746	0.097268	1.069746	up	-1.01001	-0.01437	1.010007
1424455_at	0.041654	1.147048	0.197926	1.147048	up	1.054399	0.076421	1.054399
1424488_a_at	0.046868	1.031426	0.04464	1.031426	up	1.025695	0.036602	1.025695
1424490_at	0.035039	-1.07047	-0.09824	1.07047	down	-1.15163	-0.20368	1.151634
1424535_at	0.01497	1.044046	0.062185	1.044046	up	1.049618	0.069865	1.049618
1424588_at	0.017544	1.111957	0.1531	1.111957	up	1.17042	0.227026	1.17042
1424598_at	1.16E-04	-1.02831	-0.04028	1.028312	down	-1.09825	-0.13521	1.098251
1424603_at	0.041634	-1.05213	-0.07331	1.052128	down	1.024607	0.035071	1.024607
1424642_at	0.044078	-1.0505	-0.07108	1.050502	down	-1.04846	-0.06827	1.048456
1424695_at	0.036898	-1.19501	-0.25703	1.195014	down	-1.03329	-0.04725	1.033294
1424705_at	0.03743	1.057454	0.080594	1.057454	up	1.182984	0.242431	1.182984
1424719_a_at	0.012197	1.076818	0.106774	1.076818	up	-1.12783	-0.17355	1.127833
1424733_at	0.022542	1.105913	0.145238	1.105913	up	1.084113	0.116515	1.084113
1424749_at	0.009488	1.028675	0.040787	1.028675	up	-1.01414	-0.02026	1.014139
1424804_at	0.037312	-1.07756	-0.10777	1.077564	down	1.069906	0.097485	1.069906
1424805_a_at	0.035326	1.152657	0.204963	1.152657	up	1.289189	0.366463	1.289189
1424827_a_at	0.005732	-1.00299	-0.00431	1.00299	down	1.041033	0.058016	1.041033
1424857_a_at	0.009771	-1.13408	-0.18153	1.134084	down	-1.19112	-0.25231	1.191116
1424875_at	0.026657	-1.36012	-0.44374	1.360122	down	-1.20777	-0.27235	1.207773
1424876_s_at	0.031761	-1.1727	-0.22983	1.172696	down	-1.09921	-0.13647	1.099209
1424913_at	0.003863	-1.33271	-0.41436	1.332711	down	1.292804	0.370504	1.292804
1424926_at	0.044593	1.007882	0.011327	1.007882	up	1.137416	0.18576	1.137416
1424928_at	0.035526	1.10577	0.145051	1.10577	up	-1.04426	-0.06249	1.044264
1424932_at	0.016642	1.205755	0.269937	1.205755	up	1.113307	0.154852	1.113307
1424940_s_at	0.024994	1.089907	0.124205	1.089907	up	1.371046	0.455277	1.371046
1424986_s_at	0.026262	-1.00259	-0.00373	1.002592	down	-1.17521	-0.23291	1.175206
1425006_a_at	0.016435	-1.01414	-0.02025	1.014136	down	-1.05719	-0.08024	1.057195
1425011_x_at	0.014281	-1.02628	-0.03742	1.026278	down	1.064375	0.090006	1.064375
1425015_at	0.045285	-1.05236	-0.07363	1.05236	down	-1.1666	-0.22231	1.166596
1425048_a_at	0.041037	-1.14234	-0.192	1.142345	down	-1.13318	-0.18037	1.133177
1425077_at	0.022522	1.052871	0.074329	1.052871	up	1.087703	0.121284	1.087703
1425091_at	0.011901	-1.0552	-0.07752	1.055205	down	-1.015	-0.02148	1.015
1425095_at	0.006221	-1.17527	-0.233	1.175273	down	-1.21956	-0.28637	1.219564
1425099_a_at	2.58E-06	1.265517	0.339727	1.265517	up	1.678605	0.747263	1.678605
1425109_at	0.039499	1.030907	0.043915	1.030907	up	1.109848	0.150362	1.109848
1425147_at	0.015691	1.02268	0.032355	1.02268	up	1.012898	0.018488	1.012898
1425153_at	0.013139	-1.51206	-0.59652	1.512062	down	-2.2745	-1.18555	2.274502
1425162_at	0.027392	-1.00232	-0.00335	1.002324	down	1.134896	0.182559	1.134896
1425187_at	0.001689	1.033036	0.04689	1.033036	up	1.153692	0.206258	1.153692
1425211_at	0.003007	-1.0268	-0.03815	1.026797	down	1.051673	0.072686	1.051673
1425227_a_at	0.023891	-1.09663	-0.13307	1.096626	down	1.05839	0.081871	1.05839
1425234_at	0.003794	1.075076	0.104438	1.075076	up	-1.04329	-0.06115	1.043295
1425274_at	0.035338	-1.05589	-0.07845	1.055885	down	-1.86754	-0.90114	1.867543
1425281_a_at	0.016247	1.129812	0.176083	1.129812	up	1.473424	0.559172	1.473424
1425328_at	0.002548	-1.05588	-0.07845	1.05588	down	-1.02446	-0.03487	1.024462

1425333_at	0.048134	-1.03846	-0.05444	1.038458	down	-1.15604	-0.20919	1.156036
1425347_a_at	0.024734	1.282449	0.358902	1.282449	up	1.026602	0.037877	1.026602
1425394_at	0.029345	1.004154	0.005981	1.004154	up	-1.10781	-0.14772	1.107814
1425401_at	0.020568	-1.01327	-0.01902	1.01327	down	1.009404	0.013504	1.009404
1425420_s_at	0.022234	1.078289	0.108743	1.078289	up	1.145534	0.19602	1.145534
1425434_a_at	0.025156	-1.21391	-0.27966	1.213906	down	-1.03845	-0.05444	1.038454
1425444_a_at	0.046286	-1.02039	-0.02911	1.020386	down	1.043235	0.061063	1.043235
1425451_s_at	0.009564	1.113174	0.154679	1.113174	up	2.660272	1.411574	2.660272
1425457_a_at	0.04735	1.074601	0.103801	1.074601	up	-1.27998	-0.35612	1.279977
1425460_at	0.040864	-1.02145	-0.03062	1.021453	down	1.088782	0.122716	1.088782
1425507_at	0.043733	1.04368	0.06168	1.04368	up	1.157926	0.211543	1.157926
1425553_s_at	0.027465	1.071352	0.099433	1.071352	up	1.08039	0.111553	1.08039
1425557_x_at	0.024387	1.025911	0.036905	1.025911	up	1.054431	0.076465	1.054431
1425567_a_at	0.028552	1.085297	0.11809	1.085297	up	1.048275	0.068017	1.048275
1425608_at	9.96E-04	1.139189	0.188007	1.139189	up	1.152059	0.204214	1.152059
1425625_at	0.041024	-1.24964	-0.32151	1.249641	down	-1.15771	-0.21127	1.15771
1425654_a_at	0.018751	1.033956	0.048175	1.033956	up	-1.15384	-0.20644	1.153837
1425693_at	0.034668	-1.20577	-0.26996	1.205771	down	-1.10034	-0.13795	1.100338
1425734_a_at	0.035687	-1.09592	-0.13214	1.095921	down	-1.1196	-0.16298	1.119598
1425739_at	0.036557	-1.05122	-0.07207	1.051222	down	1.064434	0.090086	1.064434
1425767_a_at	0.035021	1.045955	0.06482	1.045955	up	1.193335	0.254999	1.193335
1425780_a_at	0.02732	1.123951	0.168579	1.123951	up	1.086267	0.119378	1.086267
1425803_a_at	0.048219	1.069866	0.09743	1.069866	up	1.019892	0.028417	1.019892
1425807_at	0.036696	-1.07857	-0.10912	1.078569	down	-1.04478	-0.06319	1.044776
1425824_a_at	0.029178	-1.01641	-0.02348	1.016405	down	-1.09914	-0.13638	1.099141
1425844_a_at	0.016504	1.100766	0.138508	1.100766	up	1.248204	0.319853	1.248204
1425846_a_at	0.0092	1.293703	0.371506	1.293703	up	1.113735	0.155406	1.113735
1425877_at	0.028534	-1.11216	-0.15337	1.112165	down	-1.0239	-0.03407	1.0239
1425890_at	6.16E-04	-1.08645	-0.11962	1.086446	down	-1.14559	-0.1961	1.145593
1425924_at	0.012186	-1.11908	-0.16232	1.119083	down	-1.1393	-0.18814	1.139296
1425937_a_at	0.006534	1.036054	0.051099	1.036054	up	1.058682	0.082269	1.058682
1425956_a_at	0.032018	-1.0432	-0.06101	1.043197	down	1.09282	0.128055	1.09282
1425974_a_at	0.011251	-1.06712	-0.09373	1.067124	down	1.014691	0.021041	1.014691
1426004_a_at	0.023297	-1.09453	-0.13031	1.094529	down	1.054327	0.076322	1.054327
1426033_at	0.030006	1.111117	0.15201	1.111117	up	1.242879	0.313686	1.242879
1426036_a_at	0.002566	1.157405	0.210893	1.157405	up	-1.04593	-0.06479	1.045929
1426080_a_at	0.009487	-1.15889	-0.21275	1.158895	down	-1.0638	-0.08923	1.0638
1426120_a_at	0.046102	1.090486	0.124971	1.090486	up	1.004618	0.006647	1.004618
1426127_x_at	0.007744	1.217244	0.283618	1.217244	up	1.097922	0.134776	1.097922
1426152_a_at	0.026885	-1.04926	-0.06938	1.049263	down	-1.00747	-0.01073	1.007467
1426219_at	0.01673	-1.00157	-0.00226	1.00157	down	1.109173	0.149484	1.109173
1426227_s_at	0.002697	1.008892	0.012771	1.008892	up	1.108315	0.148368	1.108315
1426231_at	0.016001	-1.00027	-3.87E-04	1.000268	down	1.147474	0.198462	1.147474
1426234_s_at	0.009701	-1.06547	-0.09149	1.065473	down	1.061342	0.08589	1.061342
1426285_at	0.033939	1.240712	0.311168	1.240712	up	1.046556	0.065649	1.046556
1426311_s_at	0.022824	-1.07502	-0.10436	1.075019	down	1.064985	0.090833	1.064985
1426343_at	0.02934	-1.03075	-0.04369	1.030747	down	1.07767	0.107915	1.07767

1426357_at	0.031743	1.110844	0.151656	1.110844	up	-1.0441	-0.06226	1.044097
1426360_at	5.70E-05	-1.09602	-0.13227	1.096019	down	-1.08145	-0.11296	1.081447
1426361_at	0.046863	-1.00789	-0.01134	1.007892	down	-1.2459	-0.31719	1.245901
1426368_at	0.019902	-1.03328	-0.04724	1.033283	down	-1.02132	-0.03043	1.021317
1426383_at	0.039444	1.123089	0.167473	1.123089	up	1.178584	0.237054	1.178584
1426448_at	0.022499	-1.06056	-0.08483	1.060563	down	1.11216	0.153364	1.11216
1426450_at	0.041854	-1.01464	-0.02097	1.014639	down	-1.30522	-0.3843	1.305222
1426453_at	0.042274	1.129215	0.17532	1.129215	up	1.356826	0.440236	1.356826
1426456_a_at	0.011508	1.047885	0.067481	1.047885	up	1.166403	0.222066	1.166403
1426458_at	0.003741	1.109186	0.149501	1.109186	up	-1.05048	-0.07105	1.050478
1426462_at	0.008501	-1.07085	-0.09876	1.070851	down	-1.0594	-0.08324	1.059396
1426464_at	0.001398	1.244764	0.315872	1.244764	up	-1.03433	-0.04869	1.034328
1426498_at	0.042901	1.180105	0.238916	1.180105	up	1.251273	0.323396	1.251273
1426504_a_at	0.03946	-1.0156	-0.02233	1.015599	down	1.013171	0.018878	1.013171
1426505_at	0.022122	1.003388	0.00488	1.003388	up	-1.14644	-0.19715	1.146435
1426529_a_at	0.014859	1.092361	0.127449	1.092361	up	1.019782	0.02826	1.019782
1426548_a_at	0.015954	1.017193	0.024594	1.017193	up	1.112767	0.154152	1.112767
1426574_a_at	0.023178	1.216707	0.282982	1.216707	up	1.051148	0.071966	1.051148
1426584_a_at	0.04538	1.115944	0.158265	1.115944	up	1.127589	0.173241	1.127589
1426614_at	0.0324	1.047576	0.067054	1.047576	up	1.025952	0.036964	1.025952
1426631_at	0.013033	1.021349	0.030475	1.021349	up	1.052918	0.074393	1.052918
1426642_at	0.034782	1.691823	0.758579	1.691823	up	-1.05284	-0.07428	1.052837
1426650_at	0.048049	-1.84134	-0.88075	1.841336	down	-1.91176	-0.9349	1.911759
1426653_at	0.045074	1.088581	0.122448	1.088581	up	-1.01733	-0.02479	1.017333
1426683_at	0.045724	1.152074	0.204233	1.152074	up	1.174926	0.232569	1.174926
1426698_a_at	0.036157	1.023624	0.033686	1.023624	up	1.047707	0.067236	1.047707
1426732_at	0.016626	-1.0453	-0.06392	1.045299	down	-1.00659	-0.00947	1.006587
1426746_at	0.020136	-1.08803	-0.12171	1.088026	down	1.041424	0.058558	1.041424
1426777_a_at	0.013022	-1.14396	-0.19403	1.143957	down	-1.19966	-0.26262	1.199656
1426796_at	0.047427	1.042351	0.059841	1.042351	up	1.011199	0.016067	1.011199
1426831_at	0.048704	-1.09383	-0.12939	1.093835	down	1.361856	0.445574	1.361856
1426849_at	8.83E-04	1.018497	0.026442	1.018497	up	1.118149	0.161112	1.118149
1426851_a_at	0.035114	-1.30963	-0.38916	1.309629	down	-1.29143	-0.36897	1.291429
1426853_at	0.013725	1.012273	0.017598	1.012273	up	1.014359	0.020568	1.014359
1426866_at	0.031426	1.012679	0.018176	1.012679	up	-1.01059	-0.0152	1.010589
1426915_at	0.019588	1.048674	0.068566	1.048674	up	1.119338	0.162646	1.119338
1426937_at	0.032879	-1.23643	-0.30618	1.236427	down	-1.34084	-0.42314	1.340844
1426944_at	0.011408	1.084836	0.117476	1.084836	up	1.149879	0.201482	1.149879
1426947_x_at	0.035783	1.08922	0.123295	1.08922	up	-1.27744	-0.35326	1.27744
1426957_at	0.028322	1.082919	0.114925	1.082919	up	-1.05123	-0.07208	1.051233
1426958_at	0.034814	1.019889	0.028413	1.019889	up	-1.02978	-0.04234	1.02978
1426969_at	0.046464	1.015119	0.021649	1.015119	up	1.134927	0.1826	1.134927
1426978_at	0.027963	-1.20849	-0.2732	1.208486	down	-1.01169	-0.01677	1.011689
1426982_at	0.033601	1.152488	0.204752	1.152488	up	1.179545	0.238231	1.179545
1426987_at	0.001138	-1.08448	-0.117	1.084477	down	1.102507	0.140788	1.102507
1426992_at	0.01592	1.027888	0.039683	1.027888	up	1.161225	0.215647	1.161225
1427012_at	0.04587	-1.0616	-0.08624	1.061601	down	1.058243	0.081671	1.058243

1427041_at	0.018498	-1.23696	-0.3068	1.23696	down	-1.40269	-0.4882	1.402691
1427056_at	0.008824	-1.07372	-0.10262	1.073723	down	-1.34446	-0.42702	1.344457
1427079_at	0.048798	1.157495	0.211006	1.157495	up	1.04795	0.06757	1.04795
1427139_at	0.041453	1.085896	0.118886	1.085896	up	-1.00008	-1.22E-04	1.000085
1427155_at	0.049387	-1.09002	-0.12436	1.090025	down	-1.09786	-0.1347	1.097863
1427168_a_at	0.009903	1.098892	0.13605	1.098892	up	-1.21426	-0.28008	1.214264
1427169_at	0.00726	-1.00368	-0.0053	1.003679	down	-1.10254	-0.14083	1.102538
1427191_at	0.038796	1.135996	0.183957	1.135996	up	1.012546	0.017987	1.012546
1427200_at	0.017787	-1.11011	-0.15071	1.110114	down	-1.01835	-0.02623	1.018351
1427316_s_at	0.047282	1.078664	0.109246	1.078664	up	1.150642	0.202439	1.150642
1427319_at	0.040982	-1.10496	-0.144	1.104963	down	-1.02311	-0.03296	1.023107
1427331_at	0.02733	-1.01162	-0.01667	1.011624	down	-1.11507	-0.15714	1.115074
1427332_at	0.003912	1.010019	0.014382	1.010019	up	1.048576	0.068431	1.048576
1427342_at	0.005208	1.044375	0.062639	1.044375	up	1.126045	0.171264	1.126045
1427344_s_at	0.010252	1.1011	0.138946	1.1011	up	-1.09624	-0.13257	1.096244
1427450_x_at	0.001748	1.183658	0.243252	1.183658	up	1.070191	0.097868	1.070191
1427456_at	0.045588	-1.03057	-0.04345	1.030574	down	-1.02311	-0.03297	1.023114
1427471_at	0.009824	-1.03223	-0.04577	1.032233	down	1.114634	0.15657	1.114634
1427511_at	0.028193	1.059091	0.082827	1.059091	up	-1.09822	-0.13517	1.09822
1427522_at	0.031872	1.066404	0.092755	1.066404	up	1.40678	0.492397	1.40678
1427544_a_at	0.013294	-1.12154	-0.16548	1.121542	down	-1.0247	-0.0352	1.024701
1427580_a_at	5.70E-04	1.128466	0.174363	1.128466	up	-1.04983	-0.07016	1.049831
1427586_at	0.045716	-1.09837	-0.13536	1.098367	down	1.144358	0.194538	1.144358
1427608_a_at	0.004167	-1.12267	-0.16693	1.12267	down	-1.08341	-0.11558	1.083412
1427639_a_at	0.036356	-1.00521	-0.00749	1.005208	down	1.149041	0.20043	1.149041
1427649_at	0.045921	-1.12041	-0.16402	1.120408	down	-1.18209	-0.24134	1.182093
1427656_at	0.034294	-1.25281	-0.32517	1.252809	down	-1.09536	-0.1314	1.095359
1427670_a_at	0.034924	1.04505	0.063572	1.04505	up	1.043888	0.061967	1.043888
1427676_a_at	0.035496	-1.91255	-0.93549	1.912545	down	-1.44895	-0.53501	1.448949
1427679_at	0.016644	1.042968	0.060695	1.042968	up	1.022747	0.032449	1.022747
1427785_x_at	0.028975	1.021002	0.029985	1.021002	up	-1.13394	-0.18134	1.133937
1427849_a_at	0.022408	-1.06687	-0.09339	1.066871	down	-1.10429	-0.14312	1.104293
1427852_x_at	0.037926	-1.06758	-0.09435	1.067582	down	-1.10694	-0.14658	1.10694
1427876_at	0.022672	1.046703	0.065852	1.046703	up	1.185727	0.245772	1.185727
1427883_a_at	0.026322	1.136671	0.184815	1.136671	up	-1.28879	-0.36602	1.288789
1427887_at	0.046658	-1.0329	-0.0467	1.032901	down	1.072254	0.100647	1.072254
1427897_s_at	0.019839	1.023537	0.033564	1.023537	up	1.043822	0.061876	1.043822
1427906_at	0.047418	1.160424	0.214651	1.160424	up	1.08381	0.116112	1.08381
1427911_at	0.017493	1.074188	0.103247	1.074188	up	-1.12227	-0.16642	1.122267
1427914_a_at	0.030599	-1.05506	-0.07732	1.055058	down	-1.03583	-0.05079	1.035831
1427932_s_at	0.023362	1.231784	0.300749	1.231784	up	1.288459	0.365647	1.288459
1427934_at	0.025963	-1.16538	-0.2208	1.16538	down	1.085752	0.118694	1.085752
1427943_at	0.032424	-1.13568	-0.18356	1.135681	down	-1.15738	-0.21087	1.157383
1427958_at	0.001174	-1.0122	-0.0175	1.012204	down	1.132269	0.179216	1.132269
1428015_at	0.033849	1.063054	0.088215	1.063054	up	1.12003	0.163538	1.12003
1428018_a_at	0.024074	1.008184	0.011758	1.008184	up	-1.11032	-0.15097	1.110317
1428028_at	0.031608	-1.07385	-0.1028	1.073853	down	-1.17313	-0.23036	1.173126

1428047_s_at	0.001438	1.029275	0.041628	1.029275	up	1.215106	0.281082	1.215106
1428058_at	0.048538	-1.26593	-0.34019	1.265926	down	-1.26735	-0.34182	1.267353
1428065_at	0.047835	1.090353	0.124796	1.090353	up	-1.09051	-0.125	1.090511
1428067_at	0.020852	1.072965	0.101603	1.072965	up	1.062398	0.087324	1.062398
1428090_at	0.026451	1.016795	0.024029	1.016795	up	1.026251	0.037383	1.026251
1428107_at	0.044757	-1.04673	-0.06589	1.046731	down	1.072478	0.100948	1.072478
1428117_x_at	0.039837	1.024814	0.035362	1.024814	up	-1.12689	-0.17234	1.126886
1428125_at	0.038536	1.04028	0.056972	1.04028	up	1.053326	0.074952	1.053326
1428197_at	0.046595	1.154028	0.206678	1.154028	up	1.120677	0.16437	1.120677
1428212_x_at	0.005216	1.017299	0.024743	1.017299	up	-1.06178	-0.08649	1.061785
1428242_at	0.018048	-1.07599	-0.10566	1.075988	down	-1.20803	-0.27266	1.208032
1428309_s_at	0.026627	1.109233	0.149563	1.109233	up	1.043236	0.061066	1.043236
1428337_at	0.047728	1.020918	0.029867	1.020918	up	1.037574	0.053214	1.037574
1428369_s_at	0.026367	-1.01439	-0.02062	1.014395	down	1.02375	0.033864	1.02375
1428543_at	0.00272	-1.15472	-0.20754	1.154717	down	-1.13899	-0.18775	1.138985
1428577_at	0.004566	1.073619	0.102482	1.073619	up	-1.08337	-0.11552	1.083367
1428578_s_at	0.033107	1.029053	0.041317	1.029053	up	-1.0855	-0.11837	1.085504
1428580_at	0.010283	1.017041	0.024378	1.017041	up	1.010458	0.01501	1.010458
1428728_at	0.00127	1.011024	0.015818	1.011024	up	1.022835	0.032574	1.022835
1428745_a_at	0.029063	1.21436	0.280196	1.21436	up	1.064363	0.08999	1.064363
1428810_at	0.018003	-1.01735	-0.02482	1.017354	down	1.004165	0.005996	1.004165
1428870_at	0.010877	1.05491	0.077119	1.05491	up	1.130629	0.177126	1.130629
1428871_at	0.014226	-1.00739	-0.01062	1.007388	down	-1.13144	-0.17816	1.131437
1429003_at	0.006513	-1.01377	-0.01974	1.013773	down	-1.09292	-0.12818	1.092918
1429159_at	0.017533	-1.14372	-0.19373	1.143719	down	-1.74835	-0.806	1.748352
1429222_at	0.040134	-1.10526	-0.14439	1.105264	down	1.136585	0.184705	1.136585
1429247_at	0.019493	1.065552	0.091601	1.065552	up	1.020718	0.029585	1.020718
1429280_at	0.027423	-1.23401	-0.30335	1.234006	down	-1.1649	-0.2202	1.164897
1429301_at	0.016854	-1.11903	-0.16224	1.119026	down	1.014674	0.021017	1.014674
1429318_a_at	0.016524	-1.06644	-0.0928	1.066438	down	-1.07119	-0.09922	1.071195
1429339_a_at	0.01576	1.110837	0.151648	1.110837	up	1.358715	0.442242	1.358715
1429379_at	0.022392	-1.39057	-0.47568	1.390572	down	-1.75685	-0.81299	1.756851
1429711_at	0.027945	1.060873	0.085252	1.060873	up	-1.04761	-0.0671	1.047612
1429758_at	0.005298	1.046128	0.06506	1.046128	up	1.075117	0.104493	1.075117
1429832_at	0.026081	-1.00323	-0.00465	1.00323	down	1.062969	0.088099	1.062969
1430205_a_at	0.009989	1.009859	0.014153	1.009859	up	1.158113	0.211776	1.158113
1430295_at	0.041018	-1.00649	-0.00933	1.006487	down	1.052585	0.073937	1.052585
1430355_a_at	0.013707	1.158471	0.212221	1.158471	up	-1.12277	-0.16706	1.122769
1430671_a_at	0.026408	-1.04099	-0.05796	1.040991	down	1.031521	0.044773	1.031521
1430838_x_at	0.023625	1.035832	0.05079	1.035832	up	1.262163	0.335898	1.262163
1431072_a_at	0.002404	-1.14972	-0.20129	1.149722	down	1.046577	0.065679	1.046577
1431125_a_at	0.022337	1.023198	0.033085	1.023198	up	1.03373	0.04786	1.03373
1431464_a_at	0.049477	-1.3795	-0.46415	1.379502	down	-1.30867	-0.3881	1.308673
1431549_at	0.048284	1.017041	0.024378	1.017041	up	1.170629	0.227284	1.170629
1431646_a_at	0.023273	-1.14618	-0.19683	1.146179	down	1.098564	0.135619	1.098564
1431829_a_at	0.0206	1.21925	0.285994	1.21925	up	1.115078	0.157144	1.115078
1431830_at	0.045078	1.080027	0.111067	1.080027	up	1.04576	0.064552	1.04576

1431920_a_at	0.031031	1.242215	0.312915	1.242215	up	1.250442	0.322438	1.250442
1431939_a_at	0.020173	-1.05855	-0.08208	1.058546	down	-1.0258	-0.03674	1.025796
1432312_a_at	0.042277	-1.0292	-0.04153	1.029203	down	1.10437	0.143224	1.10437
1432331_a_at	0.036335	-1.01006	-0.01444	1.010062	down	-1.028	-0.03984	1.027998
1432436_a_at	0.008648	1.055651	0.078134	1.055651	up	1.269642	0.344422	1.269642
1433430_s_at	0.030574	1.018308	0.026174	1.018308	up	1.036226	0.051339	1.036226
1433431_at	0.044437	-1.17877	-0.23728	1.178769	down	1.012641	0.018123	1.012641
1433494_at	0.027519	1.243659	0.314591	1.243659	up	1.330155	0.411595	1.330155
1433504_at	6.19E-04	1.17825	0.236646	1.17825	up	1.153066	0.205475	1.153066
1433538_at	0.03738	1.240105	0.310462	1.240105	up	1.108942	0.149183	1.108942
1433569_x_at	0.008408	-1.00537	-0.00773	1.005372	down	1.09656	0.132985	1.09656
1433661_at	0.04999	1.100853	0.138622	1.100853	up	1.000095	1.37E-04	1.000095
1433691_at	0.018112	1.043103	0.060882	1.043103	up	1.201983	0.265416	1.201983
1433706_a_at	0.024656	1.029527	0.041981	1.029527	up	1.059588	0.083503	1.059588
1433724_at	0.02017	1.136616	0.184745	1.136616	up	1.218817	0.285481	1.218817
1433756_at	0.034448	-1.11466	-0.15661	1.114663	down	1.022584	0.032219	1.022584
1433774_x_at	0.013549	1.05586	0.078419	1.05586	up	1.148757	0.200074	1.148757
1433842_at	0.018295	-1.15378	-0.20637	1.153778	down	-1.08317	-0.11526	1.083171
1433881_at	0.010759	1.162025	0.216641	1.162025	up	1.232911	0.302068	1.232911
1433909_at	0.005673	-1.14722	-0.19814	1.147217	down	-1.06887	-0.09609	1.068873
1433916_at	0.028429	-1.01407	-0.02015	1.014066	down	1.050436	0.070988	1.050436
1434045_at	0.037208	-1.13327	-0.18049	1.133272	down	-1.08235	-0.11417	1.082353
1434121_at	0.022064	-1.03234	-0.04592	1.032342	down	1.269455	0.344209	1.269455
1434134_at	0.01779	1.070376	0.098118	1.070376	up	-1.00645	-0.00927	1.006448
1434155_a_at	0.014443	1.02415	0.034427	1.02415	up	1.044511	0.062828	1.044511
1434230_at	0.024221	1.056735	0.079613	1.056735	up	1.153351	0.205832	1.153351
1434237_at	0.040798	1.657553	0.729055	1.657553	up	1.450979	0.537027	1.450979
1434363_x_at	0.005558	-1.10868	-0.14884	1.108679	down	-1.01473	-0.0211	1.01473
1434416_a_at	0.017176	-1.00528	-0.0076	1.005284	down	-1.1382	-0.18675	1.1382
1434417_at	0.03891	1.120838	0.164578	1.120838	up	1.061378	0.085938	1.061378
1434438_at	0.03809	-1.12575	-0.17089	1.125752	down	-1.13845	-0.18707	1.138452
1434479_at	0.010349	1.209409	0.274302	1.209409	up	-1.18488	-0.24474	1.184879
1434555_at	0.031847	1.151322	0.203291	1.151322	up	1.167885	0.223898	1.167885
1434569_at	0.004819	-1.14573	-0.19626	1.145727	down	-1.02475	-0.03527	1.024746
1434599_a_at	0.045442	1.033875	0.048062	1.033875	up	1.125713	0.170839	1.125713
1434600_at	0.044118	1.109525	0.149942	1.109525	up	1.156121	0.209292	1.156121
1434609_at	0.032911	1.030747	0.043691	1.030747	up	1.047841	0.06742	1.047841
1434611_at	0.037073	1.103699	0.142346	1.103699	up	1.038923	0.055089	1.038923
1434643_at	0.008077	-1.03695	-0.05234	1.036948	down	-1.08645	-0.11962	1.086452
1434658_at	0.018841	1.143548	0.193517	1.143548	up	1.077387	0.107536	1.077387
1434679_at	0.037314	-1.1289	-0.17491	1.128897	down	1.013408	0.019216	1.013408
1434688_x_at	0.022486	1.083891	0.11622	1.083891	up	1.133756	0.18111	1.133756
1434881_s_at	0.012787	1.14538	0.195827	1.14538	up	1.059273	0.083075	1.059273
1434886_at	0.019259	-1.02684	-0.03821	1.026837	down	1.117673	0.160498	1.117673
1434904_at	0.028986	1.062024	0.086816	1.062024	up	-1.08225	-0.11403	1.08225
1434924_at	0.044955	1.017597	0.025166	1.017597	up	1.024085	0.034336	1.024085
1434946_at	0.048544	1.072136	0.100488	1.072136	up	1.002981	0.004294	1.002981

1434964_at	0.023347	1.060136	0.08425	1.060136	up	1.046114	0.06504	1.046114
1434992_at	0.00233	1.093777	0.129319	1.093777	up	-1.02222	-0.03171	1.022221
1435089_at	9.92E-04	1.014991	0.021466	1.014991	up	-1.05316	-0.07472	1.053159
1435160_at	0.009729	-1.01965	-0.02808	1.019651	down	-1.13052	-0.17698	1.130518
1435170_at	0.031866	1.020483	0.029252	1.020483	up	1.06876	0.095937	1.06876
1435216_a_at	0.018585	1.120114	0.163645	1.120114	up	1.19695	0.259362	1.19695
1435414_s_at	0.036172	1.053734	0.075511	1.053734	up	-1.02732	-0.03889	1.02732
1435634_at	0.040226	-1.1326	-0.17964	1.132603	down	1.092255	0.12731	1.092255
1435684_at	0.039073	-1.13727	-0.18558	1.137275	down	-1.07792	-0.10825	1.077916
1435788_at	0.044741	1.045612	0.064348	1.045612	up	1.107359	0.147123	1.107359
1435802_at	0.004181	1.215417	0.281451	1.215417	up	1.248474	0.320165	1.248474
1435896_at	0.039027	-1.06121	-0.08571	1.061212	down	1.122351	0.166524	1.122351
1435903_at	0.03612	-1.14016	-0.18923	1.140156	down	-1.37828	-0.46287	1.37828
1435919_at	0.010568	-1.09613	-0.13242	1.096134	down	1.103719	0.142372	1.103719
1435950_at	9.64E-04	-1.12696	-0.17243	1.126958	down	1.024975	0.035588	1.024975
1435965_at	0.037794	1.036994	0.052408	1.036994	up	-1.06599	-0.09219	1.065986
1435979_a_at	0.007021	-1.02286	-0.03261	1.022864	down	1.015861	0.022703	1.015861
1436042_at	0.03038	1.149966	0.201591	1.149966	up	-1.11167	-0.15273	1.111674
1436179_a_at	0.00874	1.076491	0.106336	1.076491	up	1.193671	0.255405	1.193671
1436226_at	0.023209	-1.13517	-0.1829	1.135165	down	-1.04381	-0.06185	1.043807
1436297_a_at	0.042568	-1.12675	-0.17217	1.12675	down	1.042643	0.060245	1.042643
1436348_at	0.002635	1.012574	0.018027	1.012574	up	-1.06713	-0.09373	1.067128
1436364_x_at	0.022269	1.065163	0.091074	1.065163	up	-1.29178	-0.36936	1.291776
1436442_at	0.036044	-1.01773	-0.02535	1.017726	down	1.067408	0.094111	1.067408
1436448_a_at	0.031078	1.203525	0.267266	1.203525	up	1.178829	0.237355	1.178829
1436451_a_at	0.014454	1.0163	0.023326	1.0163	up	1.053121	0.07467	1.053121
1436664_a_at	0.029517	1.033674	0.047781	1.033674	up	1.172777	0.229928	1.172777
1436669_at	0.029842	-1.00305	-0.00439	1.003045	down	1.135987	0.183947	1.135987
1436677_at	0.031159	1.008131	0.011684	1.008131	up	1.215476	0.281521	1.215476
1436853_a_at	0.049923	-1.23014	-0.29882	1.230137	down	-1.02671	-0.03804	1.026715
1436856_x_at	0.039258	1.026775	0.03812	1.026775	up	1.017828	0.025494	1.017828
1436863_at	0.007849	1.001488	0.002145	1.001488	up	-1.13689	-0.1851	1.136892
1436872_at	0.027645	-1.22787	-0.29616	1.227871	down	-1.07427	-0.10335	1.074265
1436924_x_at	0.02595	1.004287	0.006171	1.004287	up	-1.07169	-0.09989	1.071688
1436935_x_at	0.005958	1.031336	0.044515	1.031336	up	-1.00458	-0.00659	1.004581
1436979_x_at	0.043806	-1.01317	-0.01887	1.013168	down	1.009733	0.013974	1.009733
1436986_at	0.00505	-1.05645	-0.07922	1.056446	down	-1.0394	-0.05576	1.039405
1437032_x_at	0.026292	-1.057	-0.07998	1.057005	down	1.037587	0.053232	1.037587
1437102_at	0.019653	-1.11536	-0.15751	1.115361	down	-1.06739	-0.09409	1.067391
1437120_at	0.025753	-1.00277	-0.00399	1.002772	down	1.093098	0.128423	1.093098
1437223_s_at	0.046305	1.07747	0.107647	1.07747	up	1.197344	0.259837	1.197344
1437256_at	0.021496	-1.08637	-0.11952	1.08637	down	-1.05334	-0.07496	1.053335
1437289_at	0.044565	1.00527	0.007583	1.00527	up	1.036411	0.051596	1.036411
1437367_at	0.026882	-1.16217	-0.21682	1.162171	down	-1.10035	-0.13796	1.100348
1437398_a_at	0.008086	-1.05119	-0.07202	1.051189	down	1.101048	0.138878	1.101048
1437413_x_at	0.002906	1.031152	0.044257	1.031152	up	-1.03625	-0.05137	1.036247
1437434_a_at	0.038392	-1.02326	-0.03317	1.023259	down	1.25467	0.327308	1.25467

1437454_a_at	0.027206	1.14267	0.192408	1.14267	up	1.189315	0.250131	1.189315
1437527_x_at	0.021449	-1.00077	-0.00111	1.000769	down	-1.0481	-0.06777	1.048098
1437628_s_at	0.03408	1.05394	0.075792	1.05394	up	-1.0009	-0.00129	1.000897
1437810_a_at	0.037999	-1.07923	-0.11	1.079228	down	-1.0465	-0.06558	1.046502
1438033_at	0.001432	1.056304	0.079025	1.056304	up	-1.01483	-0.02124	1.014833
1438070_at	0.029178	1.059112	0.082856	1.059112	up	1.040125	0.056757	1.040125
1438083_at	0.041928	1.010816	0.01552	1.010816	up	1.051321	0.072203	1.051321
1438096_a_at	0.011618	1.004008	0.005771	1.004008	up	1.019919	0.028454	1.019919
1438115_a_at	0.031082	-1.05991	-0.08394	1.059905	down	1.159357	0.213325	1.159357
1438116_x_at	0.048417	-1.06241	-0.08734	1.062406	down	1.170083	0.226611	1.170083
1438211_s_at	0.020951	-1.03276	-0.0465	1.032759	down	-1.1019	-0.13999	1.101896
1438289_a_at	0.016027	-1.10458	-0.1435	1.104579	down	-1.0913	-0.12604	1.091296
1438370_x_at	0.00825	1.084953	0.117633	1.084953	up	1.267359	0.341825	1.267359
1438427_at	0.004908	1.176703	0.23475	1.176703	up	1.076753	0.106687	1.076753
1438556_a_at	0.04812	-1.09015	-0.12453	1.09015	down	-1.2147	-0.2806	1.2147
1438609_x_at	0.038536	-1.00178	-0.00257	1.001784	down	-1.47383	-0.55957	1.473825
1438721_a_at	0.02198	-1.01497	-0.02143	1.014968	down	-1.19341	-0.25509	1.193407
1438769_a_at	0.021876	1.004795	0.006901	1.004795	up	-1.05602	-0.07864	1.056019
1438843_x_at	0.02391	1.0015	0.002163	1.0015	up	1.090858	0.125463	1.090858
1438844_x_at	0.045105	-1.16357	-0.21855	1.163566	down	-1.08404	-0.11642	1.084043
1438923_at	0.02077	-1.05724	-0.0803	1.057239	down	-1.03014	-0.04284	1.030138
1438943_x_at	0.028718	1.090022	0.124358	1.090022	up	1.050421	0.070968	1.050421
1438951_x_at	0.046927	-1.0135	-0.01934	1.013499	down	-1.04989	-0.07024	1.049892
1438963_s_at	0.0194	1.058529	0.082061	1.058529	up	-1.15766	-0.21122	1.157664
1439028_at	0.042227	-1.27996	-0.3561	1.279961	down	-1.19967	-0.26264	1.19967
1439032_at	0.039484	1.069828	0.097379	1.069828	up	1.246421	0.317792	1.246421
1439257_x_at	0.014181	1.058123	0.081507	1.058123	up	1.066711	0.093169	1.066711
1439423_x_at	0.001937	-1.20495	-0.26897	1.20495	down	-1.33185	-0.41343	1.331851
1439959_at	0.014544	1.165326	0.220734	1.165326	up	-1.10161	-0.13961	1.101609
1440195_at	0.001159	1.013389	0.019188	1.013389	up	-1.14826	-0.19945	1.14826
1440831_at	0.003536	-1.0252	-0.0359	1.025196	down	-1.08022	-0.11132	1.080216
1440963_at	0.021121	-1.01549	-0.02217	1.015486	down	-1.09483	-0.13071	1.094833
1441344_at	0.014226	-1.19792	-0.26054	1.197924	down	-1.18284	-0.24226	1.182842
1442006_at	0.033862	1.125013	0.169941	1.125013	up	1.109364	0.149733	1.109364
1446086_s_at	0.013152	1.037537	0.053163	1.037537	up	1.16034	0.214547	1.16034
1447462_at	0.001215	-1.07085	-0.09875	1.070846	down	1.019854	0.028363	1.019854
1447632_at	0.036316	-1.03258	-0.04626	1.032583	down	-1.05432	-0.07631	1.054318
1447653_x_at	0.043893	-1.11086	-0.15168	1.110863	down	-1.2336	-0.30287	1.233596
1447924_at	0.010715	-1.01568	-0.02244	1.015679	down	-1.04229	-0.05976	1.04229
1447984_at	0.040645	1.092385	0.127481	1.092385	up	-1.13476	-0.18239	1.134759
1448056_at	0.035382	-1.05516	-0.07746	1.055156	down	-1.08478	-0.11741	1.084783
1448130_at	0.034876	1.173526	0.23085	1.173526	up	1.503216	0.588052	1.503216
1448137_at	0.007487	1.067531	0.094278	1.067531	up	1.189375	0.250204	1.189375
1448148_at	0.037738	1.028974	0.041206	1.028974	up	-1.0586	-0.08216	1.058603
1448173_a_at	0.043995	1.08433	0.116804	1.08433	up	1.066651	0.093088	1.066651
1448178_a_at	0.005358	-1.00888	-0.01276	1.008884	down	1.083703	0.11597	1.083703
1448194_a_at	0.034615	1.185996	0.246099	1.185996	up	-1.20891	-0.27371	1.208911

1448195_at	0.048097	-1.1017	-0.13973	1.101697	down	-1.07397	-0.10296	1.073974
1448230_at	0.024809	1.010136	0.014549	1.010136	up	1.061745	0.086438	1.061745
1448276_at	0.001805	1.227302	0.29549	1.227302	up	1.18029	0.239141	1.18029
1448293_at	0.0045	-1.05428	-0.07626	1.054285	down	1.021835	0.031163	1.021835
1448297_a_at	0.008214	1.041513	0.058681	1.041513	up	1.084845	0.117489	1.084845
1448337_at	0.046744	1.107712	0.147583	1.107712	up	1.094553	0.130341	1.094553
1448345_at	0.018439	1.028125	0.040016	1.028125	up	1.043395	0.061286	1.043395
1448356_at	0.038665	-1.01904	-0.02721	1.019037	down	1.000345	4.97E-04	1.000345
1448357_at	0.015684	1.013777	0.01974	1.013777	up	-1.17679	-0.23485	1.176785
1448415_a_at	0.02524	1.23938	0.309618	1.23938	up	1.266311	0.340632	1.266311
1448449_at	0.02978	1.20808	0.272716	1.20808	up	1.245394	0.316602	1.245394
1448458_at	0.015281	1.013715	0.019652	1.013715	up	-1.19192	-0.25328	1.191917
1448484_at	0.036272	-1.00654	-0.00941	1.006545	down	1.085643	0.11855	1.085643
1448494_at	0.043994	-1.08112	-0.11252	1.081119	down	-1.14471	-0.19499	1.144713
1448503_at	0.001467	-1.05511	-0.07739	1.05511	down	-1.07075	-0.09862	1.070747
1448549_a_at	0.037504	1.144156	0.194284	1.144156	up	1.273388	0.348672	1.273388
1448558_a_at	0.017953	-1.00567	-0.00815	1.005667	down	-1.05724	-0.08031	1.057243
1448571_a_at	0.019867	-1.13922	-0.18804	1.139215	down	-1.00882	-0.01267	1.008824
1448590_at	0.03247	1.207084	0.271526	1.207084	up	-1.14684	-0.19767	1.146845
1448593_at	0.035944	1.017162	0.024549	1.017162	up	-1.24458	-0.31566	1.244583
1448598_at	0.018884	1.077238	0.107338	1.077238	up	1.230479	0.29922	1.230479
1448606_at	0.005572	-1.04244	-0.05996	1.042436	down	-1.12575	-0.17089	1.125752
1448633_at	0.024637	1.037832	0.053573	1.037832	up	1.150949	0.202823	1.150949
1448647_at	0.043516	-1.00085	-0.00122	1.000846	down	1.016838	0.02409	1.016838
1448652_at	0.03174	1.021926	0.031291	1.021926	up	1.015182	0.021739	1.015182
1448657_a_at	0.010979	1.005561	0.008001	1.005561	up	1.08533	0.118134	1.08533
1448670_at	0.041716	1.069256	0.096608	1.069256	up	1.138204	0.186759	1.138204
1448691_at	0.03393	-1.00987	-0.01418	1.009874	down	-1.04346	-0.06137	1.043457
1448710_at	0.044015	-1.20974	-0.2747	1.209742	down	-1.15834	-0.21206	1.15834
1448757_at	0.034731	1.136506	0.184605	1.136506	up	1.101057	0.138889	1.101057
1448831_at	0.046116	1.038449	0.05443	1.038449	up	1.001664	0.002399	1.001664
1448896_at	0.044638	1.026104	0.037177	1.026104	up	1.045255	0.063855	1.045255
1448916_at	0.004156	-1.02288	-0.03264	1.022881	down	-1.06294	-0.08806	1.062936
1448923_at	0.031712	1.006416	0.009227	1.006416	up	-1.03323	-0.04717	1.033234
1448929_at	0.028067	-1.33904	-0.4212	1.339037	down	-1.22924	-0.29777	1.229241
1448939_at	0.002264	-1.19709	-0.25954	1.197094	down	-1.00733	-0.01053	1.007326
1448965_at	0.049567	-1.10222	-0.14041	1.102218	down	-1.0784	-0.10889	1.078398
1448990_a_at	0.003797	1.269927	0.344745	1.269927	up	1.103811	0.142493	1.103811
1448995_at	0.01178	-1.19852	-0.26125	1.198518	down	-1.43988	-0.52595	1.439878
1449082_at	0.042393	1.097645	0.134412	1.097645	up	-1.18441	-0.24417	1.184408
1449089_at	0.037513	1.065963	0.092157	1.065963	up	1.052448	0.07375	1.052448
1449102_at	0.001978	-1.51509	-0.5994	1.515085	down	-1.4225	-0.50843	1.422505
1449113_at	0.036671	1.054178	0.076118	1.054178	up	1.012629	0.018106	1.012629
1449143_at	0.022892	-1.17818	-0.23656	1.178181	down	-1.12901	-0.17506	1.129015
1449145_a_at	0.023706	1.09035	0.124791	1.09035	up	1.053448	0.075119	1.053448
1449149_at	0.02958	1.017281	0.024717	1.017281	up	-1.08511	-0.11784	1.085109
1449202_at	0.046147	-1.04802	-0.06767	1.048021	down	1.047902	0.067503	1.047902

1449203_at	0.009058	-1.09491	-0.13081	1.09491	down	1.208414	0.273115	1.208414
1449216_at	0.006798	-1.0142	-0.02035	1.014202	down	-1.12852	-0.17444	1.128524
1449245_at	0.02126	-1.28868	-0.3659	1.288682	down	-1.15243	-0.20467	1.152426
1449276_at	0.046855	-1.02995	-0.04257	1.029948	down	-1.06026	-0.08441	1.060256
1449291_a_at	1.73E-04	-1.0961	-0.13239	1.096105	down	-1.04192	-0.05924	1.041919
1449301_at	0.013199	-1.0396	-0.05603	1.039604	down	-1.11067	-0.15144	1.110675
1449307_at	0.009306	1.044494	0.062804	1.044494	up	1.081735	0.113347	1.081735
1449329_at	0.009367	1.044772	0.063189	1.044772	up	1.07763	0.107862	1.07763
1449346_s_at	0.018569	1.128066	0.173851	1.128066	up	1.058579	0.082129	1.058579
1449351_s_at	0.00822	1.059492	0.083372	1.059492	up	1.261329	0.334945	1.261329
1449408_at	0.006537	1.090381	0.124832	1.090381	up	1.036971	0.052375	1.036971
1449411_at	0.013174	1.081141	0.112555	1.081141	up	1.19672	0.259085	1.19672
1449514_at	0.030557	1.18925	0.250052	1.18925	up	1.148721	0.200028	1.148721
1449521_at	0.006314	1.041088	0.058092	1.041088	up	-1.02455	-0.03499	1.024551
1449536_at	0.004803	-1.18605	-0.24617	1.186051	down	-1.10578	-0.14507	1.105784
1449538_a_at	0.010905	-1.08805	-0.12175	1.088051	down	-1.05165	-0.07265	1.051649
1449550_at	0.040783	-1.12289	-0.16721	1.122886	down	-1.17873	-0.23723	1.178726
1449632_s_at	0.036911	1.186099	0.246225	1.186099	up	1.024301	0.03464	1.024301
1449714_at	0.03729	1.049831	0.070157	1.049831	up	1.111208	0.152129	1.111208
1449799_s_at	0.037107	1.045236	0.063828	1.045236	up	1.246536	0.317924	1.246536
1449824_at	0.001303	-1.06272	-0.08776	1.062721	down	1.131438	0.178157	1.131438
1449825_at	0.035994	-1.06293	-0.08805	1.062933	down	1.023894	0.034066	1.023894
1449845_a_at	0.001084	1.250496	0.3225	1.250496	up	1.240848	0.311326	1.240848
1449847_a_at	3.51E-04	-1.08202	-0.11373	1.082021	down	-1.19505	-0.25707	1.195051
1449851_at	6.31E-04	1.321228	0.401879	1.321228	up	1.618883	0.694999	1.618883
1449872_at	0.048145	1.068753	0.095929	1.068753	up	-1.46677	-0.55265	1.466773
1449928_at	0.029547	1.006125	0.008809	1.006125	up	1.184276	0.244005	1.184276
1449931_at	0.002002	1.060604	0.084885	1.060604	up	1.236655	0.306443	1.236655
1449954_at	0.03622	1.078415	0.108912	1.078415	up	1.23101	0.299842	1.23101
1449956_at	0.043162	1.084413	0.116914	1.084413	up	-1.05601	-0.07862	1.056011
1449981_a_at	0.020438	1.032928	0.04674	1.032928	up	1.086404	0.119561	1.086404
1449996_a_at	0.047971	-4.58006	-2.19537	4.580064	down	-3.08287	-1.62428	3.082874
1449997_at	0.021428	-3.39345	-1.76275	3.393453	down	-2.92697	-1.54941	2.926967
1450026_a_at	0.033853	1.007478	0.010748	1.007478	up	1.229959	0.29861	1.229959
1450054_at	0.039388	1.087529	0.121054	1.087529	up	1.123725	0.168289	1.123725
1450063_at	0.032421	1.127378	0.172971	1.127378	up	-1.03231	-0.04588	1.032311
1450100_a_at	0.031397	-1.02814	-0.04003	1.028137	down	-1.05755	-0.08072	1.057547
1450104_at	0.026729	-1.0671	-0.0937	1.067102	down	1.018616	0.026611	1.018616
1450135_at	0.032211	-1.1756	-0.2334	1.175602	down	-1.00835	-0.01199	1.008347
1450148_at	0.042485	-1.1724	-0.22947	1.172401	down	1.005204	0.007488	1.005204
1450149_a_at	0.023019	-1.00585	-0.00842	1.005852	down	1.05473	0.076874	1.05473
1450165_at	0.018011	-1.02602	-0.03705	1.026017	down	-1.27748	-0.3533	1.277479
1450173_at	0.041099	1.053321	0.074945	1.053321	up	-1.09458	-0.13038	1.094582
1450177_at	0.02063	-1.01938	-0.02769	1.019378	down	-1.06103	-0.08547	1.06103
1450190_at	0.02584	-1.23468	-0.30414	1.234682	down	-1.30539	-0.38449	1.305395
1450278_at	0.025601	-1.011	-0.01578	1.011	down	-1.21689	-0.2832	1.216893
1450291_s_at	0.025938	-1.7803	-0.83212	1.780301	down	-1.53383	-0.61714	1.533831

1450303_at	0.025343	1.059453	0.083319	1.059453	up	1.088004	0.121684	1.088004
1450313_at	0.049089	-1.16324	-0.21815	1.163241	down	-1.15656	-0.20984	1.15656
1450321_at	0.024264	-1.15974	-0.2138	1.159735	down	-1.12103	-0.16482	1.12103
1450327_at	0.048703	-1.20283	-0.26643	1.202828	down	-1.17896	-0.23751	1.178958
1450365_at	0.042614	1.149741	0.201309	1.149741	up	-1.01189	-0.01705	1.011888
1450429_at	0.047604	1.100704	0.138426	1.100704	up	1.106819	0.146419	1.106819
1450439_at	0.033817	1.043837	0.061896	1.043837	up	1.015819	0.022643	1.015819
1450472_s_at	0.038773	-1.00992	-0.01424	1.009916	down	-1.0896	-0.1238	1.089605
1450500_at	0.014643	-1.18255	-0.2419	1.182546	down	-1.26646	-0.34081	1.266464
1450501_at	0.016089	-1.12975	-0.176	1.129749	down	-1.22281	-0.2902	1.222809
1450506_a_at	0.002685	1.354669	0.437941	1.354669	up	1.238463	0.308551	1.238463
1450511_at	0.012197	-1.08026	-0.11138	1.080262	down	-1.0605	-0.08474	1.060496
1450534_x_at	0.042072	1.128793	0.174781	1.128793	up	1.018748	0.026797	1.018748
1450537_at	0.029398	-1.09724	-0.13388	1.097239	down	-1.07865	-0.10923	1.078649
1450625_at	0.004088	1.250961	0.323036	1.250961	up	-1.00958	-0.01376	1.009583
1450629_at	0.031718	-1.04248	-0.06002	1.042477	down	-1.1745	-0.23205	1.174499
1450634_at	0.018435	1.098899	0.136059	1.098899	up	1.167024	0.222834	1.167024
1450652_at	0.008984	1.144854	0.195163	1.144854	up	-1.06376	-0.08918	1.063765
1450671_at	0.040022	-1.21907	-0.28578	1.219068	down	-6.10198	-2.60928	6.101975
1450722_at	0.026612	-1.00305	-0.0044	1.003053	down	-1.02325	-0.03316	1.023254
1450778_a_at	0.040099	-1.13986	-0.18886	1.139862	down	-1.13654	-0.18465	1.136543
1450826_a_at	1.24E-05	1.531731	0.615163	1.531731	up	6.248886	2.643599	6.248886
1450836_at	0.049029	1.056592	0.079418	1.056592	up	-1.01343	-0.01924	1.013429
1450843_a_at	0.013364	1.064111	0.089649	1.064111	up	-1.14926	-0.20071	1.149262
1450854_at	0.001812	-1.06498	-0.09083	1.064984	down	1.128022	0.173796	1.128022
1450858_a_at	0.020374	-1.04616	-0.0651	1.046159	down	1.104103	0.142875	1.104103
1450863_a_at	0.00996	1.236867	0.306691	1.236867	up	1.321572	0.402255	1.321572
1450868_at	0.02721	1.162934	0.21777	1.162934	up	1.351117	0.434152	1.351117
1450890_a_at	0.016024	-1.12071	-0.16441	1.12071	down	-1.01308	-0.01875	1.013083
1450894_a_at	0.027421	1.035875	0.05085	1.035875	up	1.040616	0.057437	1.040616
1450900_at	0.017759	-1.07907	-0.10979	1.079069	down	-1.00269	-0.00388	1.002693
1450915_at	0.012483	-1.03321	-0.04714	1.033214	down	1.213706	0.279419	1.213706
1450982_at	0.001344	1.088727	0.122642	1.088727	up	1.577469	0.657612	1.577469
1451007_at	0.02812	1.006025	0.008666	1.006025	up	1.039566	0.055981	1.039566
1451031_at	0.02491	-1.60178	-0.67968	1.601785	down	-2.34586	-1.23012	2.345862
1451061_at	0.040116	1.049047	0.069079	1.049047	up	-1.02728	-0.03883	1.02728
1451073_at	0.046522	-1.06208	-0.0869	1.062082	down	1.011171	0.016027	1.011171
1451078_at	0.047034	-1.03189	-0.04528	1.031885	down	-1.08354	-0.11576	1.083544
1451102_at	0.034867	1.048152	0.067848	1.048152	up	1.019684	0.028122	1.019684
1451105_at	0.025618	1.478975	0.564598	1.478975	up	1.142886	0.192682	1.142886
1451132_at	0.048648	1.192803	0.254356	1.192803	up	1.150464	0.202215	1.150464
1451158_at	0.007316	-1.04626	-0.06524	1.046257	down	-1.05647	-0.07925	1.056469
1451181_at	0.026126	-1.04948	-0.06967	1.049477	down	-1.27972	-0.35583	1.279724
1451188_at	0.008267	-1.0726	-0.10112	1.072605	down	-1.05903	-0.08274	1.059028
1451220_at	0.012847	-1.10101	-0.13883	1.101014	down	1.057733	0.080975	1.057733
1451314_a_at	0.035784	1.06903	0.096302	1.06903	up	1.095315	0.131345	1.095315
1451345_at	0.017189	1.036394	0.051573	1.036394	up	1.088437	0.122258	1.088437

1451346_at	0.028074	-1.13792	-0.1864	1.137917	down	1.013388	0.019187	1.013388
1451355_at	0.013853	1.071831	0.100078	1.071831	up	-1.15565	-0.2087	1.155646
1451359_at	0.01366	1.001019	0.001469	1.001019	up	1.021086	0.030104	1.021086
1451361_a_at	0.04442	1.155682	0.208744	1.155682	up	1.116822	0.1594	1.116822
1451379_at	0.031066	1.022368	0.031915	1.022368	up	-1.07712	-0.10718	1.077122
1451418_a_at	0.009921	-1.10194	-0.14004	1.101935	down	-1.42897	-0.51497	1.428966
1451437_at	0.018987	-1.19731	-0.2598	1.19731	down	-1.01498	-0.02145	1.014979
1451452_a_at	0.015559	-1.22443	-0.29211	1.224427	down	-1.07067	-0.09851	1.07067
1451478_at	6.47E-04	1.453623	0.539653	1.453623	up	1.409524	0.495208	1.409524
1451490_at	0.020833	1.14286	0.192648	1.14286	up	1.417131	0.502974	1.417131
1451506_at	0.03603	-1.12523	-0.17022	1.125233	down	-1.28309	-0.35962	1.283091
1451568_at	0.046299	1.009946	0.014277	1.009946	up	1.130721	0.177243	1.130721
1451585_x_at	0.016678	-1.05235	-0.07361	1.05235	down	-1.11179	-0.15288	1.11179
1451591_a_at	0.00555	-1.01888	-0.02698	1.018877	down	1.003043	0.004383	1.003043
1451715_at	0.040331	-1.02207	-0.0315	1.022073	down	-1.14591	-0.1965	1.145912
1451727_at	0.027304	-1.16363	-0.21864	1.163633	down	-1.08209	-0.11381	1.082085
1451738_at	0.0468	1.050195	0.070658	1.050195	up	-1.01109	-0.01592	1.011093
1451766_at	0.014794	-1.09218	-0.12722	1.092184	down	1.149515	0.201026	1.149515
1451769_s_at	0.045629	1.289489	0.3668	1.289489	up	1.243012	0.313841	1.243012
1451773_s_at	5.39E-06	1.022119	0.031563	1.022119	up	1.154894	0.20776	1.154894
1451793_at	0.018685	1.193016	0.254614	1.193016	up	1.081211	0.112648	1.081211
1451797_at	0.027736	1.011686	0.016762	1.011686	up	1.138923	0.18767	1.138923
1451838_a_at	0.044664	-1.45497	-0.54099	1.454971	down	1.386293	0.471232	1.386293
1451848_a_at	0.020854	1.062802	0.087872	1.062802	up	1.154334	0.207061	1.154334
1451925_at	0.017221	-1.12949	-0.17567	1.129486	down	-1.27832	-0.35424	1.278316
1451978_at	0.037479	1.105468	0.144657	1.105468	up	1.014574	0.020873	1.014574
1451994_s_at	0.02067	1.031747	0.045089	1.031747	up	-1.02741	-0.03901	1.027412
1452037_at	0.022605	1.029288	0.041646	1.029288	up	-1.0201	-0.02871	1.020102
1452084_at	0.02466	1.088201	0.121944	1.088201	up	1.097729	0.134522	1.097729
1452087_at	0.032241	-1.03708	-0.05253	1.03708	down	-1.16551	-0.22097	1.165514
1452155_a_at	0.037558	1.184442	0.244207	1.184442	up	1.122482	0.166692	1.122482
1452159_at	0.036176	1.014525	0.020804	1.014525	up	1.088746	0.122668	1.088746
1452163_at	0.029169	-1.09336	-0.12876	1.093356	down	-1.11972	-0.16313	1.119716
1452188_at	0.045074	1.010291	0.014771	1.010291	up	1.02521	0.035919	1.02521
1452199_at	0.005712	1.019486	0.027842	1.019486	up	1.230693	0.299471	1.230693
1452226_at	0.018986	-1.02558	-0.03644	1.025578	down	1.114967	0.157001	1.114967
1452228_at	0.004268	1.059502	0.083386	1.059502	up	-1.11012	-0.15072	1.110125
1452244_at	0.012462	-1.07868	-0.10926	1.078677	down	-1.3293	-0.41066	1.329298
1452266_at	0.018439	1.050176	0.070631	1.050176	up	1.20807	0.272704	1.20807
1452287_at	0.019673	-1.01033	-0.01483	1.01033	down	1.291456	0.368998	1.291456
1452327_at	0.031346	1.041862	0.059164	1.041862	up	1.180682	0.23962	1.180682
1452335_at	0.011364	-1.06671	-0.09316	1.066707	down	1.155791	0.20888	1.155791
1452339_at	0.047411	1.019533	0.027908	1.019533	up	1.119056	0.162282	1.119056
1452345_at	0.00334	-1.23541	-0.30499	1.235408	down	-1.74084	-0.79978	1.740835
1452349_x_at	0.031569	-1.40518	-0.49075	1.405179	down	-1.50088	-0.58581	1.500879
1452351_at	0.02834	1.041896	0.059211	1.041896	up	-1.06277	-0.08783	1.062773
1452353_at	0.019049	1.001953	0.002815	1.001953	up	-1.09308	-0.1284	1.093078

1452357_at	0.039475	1.200419	0.263538	1.200419	up	-1.10325	-0.14176	1.103249
1452359_at	0.014297	-1.15541	-0.20841	1.155414	down	-1.13402	-0.18145	1.134024
1452360_a_at	0.030424	1.058038	0.081392	1.058038	up	-1.02972	-0.04225	1.029718
1452380_at	0.044651	1.095847	0.132046	1.095847	up	1.008663	0.012444	1.008663
1452387_a_at	0.003943	1.078503	0.10903	1.078503	up	1.37212	0.456407	1.37212
1452441_at	0.008984	-1.04449	-0.06279	1.044485	down	-1.00828	-0.01189	1.008279
1452450_at	0.014186	1.021372	0.030508	1.021372	up	-1.08931	-0.12342	1.08931
1452470_at	9.43E-04	1.00412	0.005932	1.00412	up	-1.14126	-0.19063	1.14126
1452481_at	0.008576	-1.11149	-0.15249	1.111489	down	1.076712	0.106632	1.076712
1452514_a_at	0.018273	1.060742	0.085074	1.060742	up	-1.09309	-0.12841	1.093088
1452595_at	0.035148	1.022915	0.032686	1.022915	up	1.079282	0.110072	1.079282
1452596_at	0.010638	-1.04752	-0.06697	1.047515	down	-1.10233	-0.14056	1.102332
1452635_x_at	0.021833	-1.06675	-0.09322	1.066749	down	-1.0953	-0.13133	1.095305
1452636_x_at	0.026996	-1.04581	-0.06462	1.045813	down	-1.02417	-0.03446	1.024172
1452768_at	0.012719	1.08198	0.113674	1.08198	up	1.229636	0.298231	1.229636
1452772_at	0.03716	1.009462	0.013586	1.009462	up	-1.08486	-0.11751	1.084863
1452830_s_at	0.028646	1.201774	0.265165	1.201774	up	-1.02512	-0.03579	1.025116
1452831_s_at	0.049814	1.100599	0.138289	1.100599	up	1.062571	0.08756	1.062571
1452864_at	0.02635	1.081857	0.11351	1.081857	up	-1.0049	-0.00705	1.0049
1453169_a_at	0.006605	-1.02323	-0.03313	1.023229	down	-1.01719	-0.02459	1.01719
1453181_x_at	0.014211	1.067462	0.094185	1.067462	up	1.144024	0.194118	1.144024
1453208_at	0.006942	-1.07707	-0.10711	1.07707	down	1.039154	0.05541	1.039154
1453281_at	0.035921	-1.01489	-0.02132	1.01489	down	-1.06173	-0.08642	1.061734
1453412_a_at	0.022257	1.104007	0.142749	1.104007	up	1.086392	0.119545	1.086392
1453461_at	0.04878	-1.03743	-0.05301	1.037426	down	1.076025	0.105711	1.076025
1453678_at	5.21E-04	1.037013	0.052434	1.037013	up	2.01401	1.010071	2.01401
1454136_a_at	0.029644	-1.13001	-0.17633	1.130007	down	1.005711	0.008216	1.005711
1454605_a_at	0.033749	-1.00446	-0.00642	1.004463	down	1.061058	0.085504	1.061058
1454643_at	0.019028	1.003052	0.004396	1.003052	up	-1.02181	-0.03112	1.021808
1454670_at	0.005388	1.114419	0.156292	1.114419	up	1.000154	2.22E-04	1.000154
1454675_at	0.002549	1.072539	0.10103	1.072539	up	1.035567	0.05042	1.035567
1454722_at	0.033496	1.078307	0.108768	1.078307	up	1.098922	0.136089	1.098922
1454747_a_at	0.00129	1.074894	0.104194	1.074894	up	-1.06865	-0.09579	1.068651
1454860_x_at	0.010571	1.210195	0.27524	1.210195	up	1.176204	0.234138	1.176204
1454881_s_at	0.048551	1.502	0.586885	1.502	up	1.320977	0.401605	1.320977
1455026_at	0.040457	1.025333	0.036092	1.025333	up	-1.03573	-0.05064	1.035725
1455030_at	0.043106	-1.10408	-0.14284	1.104077	down	-1.11121	-0.15213	1.111207
1455047_at	0.001518	1.049215	0.06931	1.049215	up	1.202873	0.266484	1.202873
1455205_a_at	0.013086	1.055335	0.077702	1.055335	up	-1.03266	-0.04637	1.032663
1455286_at	0.045105	-1.0087	-0.0125	1.008699	down	1.011985	0.017188	1.011985
1455479_a_at	0.032353	1.062741	0.08779	1.062741	up	1.129933	0.176237	1.129933
1455491_at	0.042643	1.034238	0.048569	1.034238	up	1.020438	0.029188	1.020438
1455526_at	0.005298	1.101202	0.13908	1.101202	up	1.123349	0.167806	1.123349
1455534_s_at	0.019539	-1.04032	-0.05703	1.040323	down	1.017402	0.024889	1.017402
1455556_at	0.01393	-1.06171	-0.08639	1.061712	down	-1.1369	-0.18511	1.136903
1455631_at	0.040713	1.015138	0.021676	1.015138	up	-1.04726	-0.06661	1.047256
1455677_s_at	0.0125	-1.08023	-0.11134	1.080231	down	-1.02762	-0.0393	1.027617

1455733_at	0.043871	-1.11252	-0.15383	1.11252	down	-1.18182	-0.24101	1.181823
1455758_at	0.01443	-1.12197	-0.16604	1.121975	down	-1.09854	-0.13558	1.098538
1455829_at	0.030822	1.019941	0.028486	1.019941	up	1.02868	0.040795	1.02868
1455955_s_at	0.014496	1.057602	0.080796	1.057602	up	1.057531	0.0807	1.057531
1455965_at	0.018008	-1.048	-0.06764	1.048001	down	-1.13218	-0.1791	1.132177
1455981_at	0.007704	-1.14566	-0.19618	1.145664	down	-1.25115	-0.32326	1.251152
1456013_x_at	0.00188	1.165163	0.220531	1.165163	up	1.171754	0.22867	1.171754
1456120_at	0.001488	-1.05488	-0.07708	1.054878	down	-1.03072	-0.04366	1.030722
1456170_x_at	0.049234	1.084521	0.117058	1.084521	up	1.023923	0.034108	1.023923
1456349_x_at	0.016948	-1.07031	-0.09803	1.070313	down	-1.03375	-0.04789	1.033749
1456438_x_at	0.014986	1.049404	0.06957	1.049404	up	1.040206	0.05687	1.040206
1456488_at	0.004304	1.067558	0.094314	1.067558	up	1.008805	0.012647	1.008805
1456543_at	0.01391	-1.08611	-0.11917	1.086108	down	-1.69021	-0.75721	1.690213
1456603_at	0.022796	-1.08712	-0.12051	1.08712	down	-1.13367	-0.181	1.133673
1456606_a_at	0.040269	1.132971	0.180111	1.132971	up	1.027933	0.039746	1.027933
1456702_x_at	0.042207	-1.22432	-0.29198	1.224319	down	-1.08419	-0.11661	1.084187
1456733_x_at	0.003629	1.140429	0.189577	1.140429	up	-1.18666	-0.24691	1.186663
1457695_at	0.028647	-1.02869	-0.04081	1.028693	down	1.032908	0.046712	1.032908
1459546_s_at	0.037268	1.211078	0.276292	1.211078	up	1.148431	0.199665	1.148431
1459927_at	0.00787	-1.06594	-0.09213	1.065943	down	1.008232	0.011827	1.008232
1460008_x_at	0.014199	1.003408	0.004908	1.003408	up	-1.06876	-0.09594	1.068764
1460038_at	0.00773	-1.11584	-0.15813	1.115837	down	-1.2687	-0.34335	1.268695
1460194_at	0.03913	-1.01591	-0.02278	1.015915	down	1.043357	0.061233	1.043357
1460204_at	0.010454	1.01363	0.019531	1.01363	up	-1.10286	-0.14124	1.102855
1460206_at	0.024649	-1.02595	-0.03696	1.025953	down	1.038539	0.054556	1.038539
1460210_at	0.023122	1.074376	0.103499	1.074376	up	-1.04223	-0.05967	1.042227
1460224_at	0.042008	1.005099	0.007338	1.005099	up	-1.00805	-0.01157	1.008051
1460225_at	0.010556	1.031458	0.044685	1.031458	up	1.239663	0.309948	1.239663
1460238_at	0.027821	-1.03128	-0.04444	1.03128	down	1.017036	0.024371	1.017036
1460243_at	0.00934	1.031996	0.045438	1.031996	up	-1.02173	-0.03101	1.02173
1460316_at	0.046743	-1.15362	-0.20617	1.153622	down	-1.21703	-0.28337	1.217035
1460327_at	5.75E-04	-1.05594	-0.07853	1.055942	down	-1.0991	-0.13633	1.099103
1460357_at	0.043513	1.016331	0.02337	1.016331	up	1.004569	0.006576	1.004569
1460370_at	0.00623	1.053645	0.075388	1.053645	up	-1.08696	-0.1203	1.086962
1460390_at	0.048296	1.034252	0.048587	1.034252	up	-1.03234	-0.04592	1.032342
1460420_a_at	4.59E-04	1.034901	0.049493	1.034901	up	1.034011	0.048251	1.034011
1460655_a_at	0.021295	1.020656	0.029496	1.020656	up	1.06531	0.091273	1.06531
1460674_at	0.034411	1.122798	0.167098	1.122798	up	1.058387	0.081868	1.058387
1460684_at	0.031999	1.102048	0.140187	1.102048	up	1.367747	0.451801	1.367747
1460688_s_at	0.011538	-1.02512	-0.03579	1.025117	down	1.090862	0.125468	1.090862
1460695_a_at	0.010328	-1.02254	-0.03216	1.022542	down	-1.0489	-0.06888	1.048903
1460696_at	0.022841	1.19229	0.253736	1.19229	up	1.224582	0.292289	1.224582
1460712_s_at	0.026968	1.032708	0.046432	1.032708	up	1.141275	0.190646	1.141275
1460733_at	0.042706	-1.03814	-0.054	1.038136	down	1.093828	0.129385	1.093828
1460740_at	0.016056	1.004411	0.00635	1.004411	up	1.328744	0.410063	1.328744
1419853_a_at	0.013833	1.067004	0.093566	1.067004	up	-1.23779	-0.30776	1.237788
1419996_s_at	0.015861	-1.02518	-0.03588	1.025181	down	1.006653	0.009567	1.006653

1420181_at	0.047142	-1.00575	-0.00827	1.005746	down	-1.17152	-0.22838	1.171516
1420238_at	0.035121	1.096336	0.13269	1.096336	up	1.269813	0.344617	1.269813
1428176_at	0.04604	-1.01194	-0.01712	1.011937	down	-1.01081	-0.01551	1.01081
1428190_at	0.01737	1.060383	0.084586	1.060383	up	1.166422	0.222089	1.166422
1428198_at	0.046374	1.195518	0.257636	1.195518	up	1.218662	0.285299	1.218662
1428220_at	0.015274	1.010068	0.014453	1.010068	up	-1.01992	-0.02845	1.019917
1428232_at	0.035924	-1.05664	-0.07948	1.056636	down	1.032293	0.045852	1.032293
1428261_at	0.019628	1.082639	0.114552	1.082639	up	1.036847	0.052203	1.036847
1428281_at	0.005777	1.102635	0.140956	1.102635	up	1.237669	0.307626	1.237669
1428285_at	0.035175	1.06828	0.09529	1.06828	up	1.089796	0.124057	1.089796
1428313_at	0.020889	-1.06844	-0.09551	1.068444	down	-1.01443	-0.02067	1.014433
1428332_at	5.37E-04	1.051781	0.072834	1.051781	up	1.179356	0.238	1.179356
1428336_at	0.021842	1.012426	0.017816	1.012426	up	-1.18851	-0.24915	1.188506
1428352_at	0.040036	1.366175	0.450142	1.366175	up	1.548783	0.631135	1.548783
1428387_at	0.019726	1.030712	0.043641	1.030712	up	1.154685	0.2075	1.154685
1428393_at	0.043404	1.210673	0.275809	1.210673	up	1.099506	0.136856	1.099506
1428403_at	0.013722	-1.02226	-0.03177	1.022263	down	1.045977	0.064851	1.045977
1428444_at	0.04409	-1.05444	-0.07648	1.054444	down	-1.22775	-0.29601	1.227748
1428487_s_at	0.006749	-1.06976	-0.09729	1.069765	down	-1.09667	-0.13313	1.096671
1428493_at	0.017058	-1.06248	-0.08743	1.062477	down	1.077884	0.108201	1.077884
1428496_at	0.003835	1.035778	0.050714	1.035778	up	1.138186	0.186736	1.138186
1428499_at	0.01968	1.12405	0.168707	1.12405	up	1.093564	0.129037	1.093564
1428562_at	0.010462	-1.04313	-0.06092	1.043128	down	1.093684	0.129196	1.093684
1428579_at	0.02471	-1.0003	-4.32E-04	1.0003	down	1.184238	0.243958	1.184238
1428647_at	0.042713	1.073974	0.102959	1.073974	up	1.059066	0.082793	1.059066
1428683_at	0.015212	1.090083	0.124438	1.090083	up	1.188993	0.24974	1.188993
1428808_at	0.016647	1.092602	0.127768	1.092602	up	-1.16524	-0.22063	1.165242
1428811_at	0.013408	1.001812	0.002612	1.001812	up	1.147472	0.198459	1.147472
1428883_at	0.027076	1.091861	0.126789	1.091861	up	1.031827	0.045201	1.031827
1428886_at	0.022282	1.104456	0.143335	1.104456	up	1.115594	0.157812	1.115594
1428889_at	0.008356	1.103663	0.1423	1.103663	up	1.093874	0.129447	1.093874
1428958_at	0.00629	-1.05531	-0.07766	1.055308	down	-1.10709	-0.14677	1.107091
1428960_at	0.011861	1.000607	8.76E-04	1.000607	up	-1.02961	-0.04209	1.029606
1428979_at	0.014831	-1.03782	-0.05355	1.037816	down	-1.04815	-0.06785	1.048153
1428987_at	0.006064	1.073227	0.101955	1.073227	up	1.160207	0.214382	1.160207
1428996_at	0.0131	1.088278	0.122048	1.088278	up	1.159917	0.214022	1.159917
1429026_at	0.001289	1.018977	0.027121	1.018977	up	-1.09317	-0.12852	1.093173
1429091_at	0.028033	1.0264	0.037593	1.0264	up	-1.05233	-0.07359	1.052333
1429093_at	0.008932	-1.08575	-0.11869	1.085752	down	-1.21053	-0.27564	1.210527
1429099_at	0.030271	1.0503	0.070801	1.0503	up	1.061692	0.086365	1.061692
1429100_at	0.027774	-1.11107	-0.15195	1.111069	down	-1.14623	-0.19689	1.146228
1429184_at	0.026743	-1.06305	-0.08821	1.063047	down	-1.16922	-0.22555	1.169222
1429230_at	0.025298	-1.11371	-0.15538	1.113714	down	-1.01987	-0.02839	1.019873
1429312_s_at	0.027053	-1.01159	-0.01663	1.011593	down	1.102389	0.140633	1.102389
1429313_at	0.047918	1.126301	0.171592	1.126301	up	1.255035	0.327728	1.255035
1429315_at	0.042269	1.071228	0.099265	1.071228	up	1.110602	0.151341	1.110602
1429329_at	0.027943	1.136817	0.185	1.136817	up	1.026762	0.038102	1.026762

1429336_at	0.013714	-1.14523	-0.19564	1.145235	down	1.134247	0.181735	1.134247
1429348_at	0.019242	1.137352	0.185678	1.137352	up	1.28579	0.362655	1.28579
1429351_at	0.01145	-1.00441	-0.00635	1.004411	down	1.182114	0.24137	1.182114
1429392_at	0.03948	-1.00469	-0.00675	1.00469	down	1.025507	0.036337	1.025507
1429435_x_at	0.037277	-1.04907	-0.06912	1.049073	down	-1.10044	-0.13808	1.100443
1429444_at	0.047243	1.162001	0.216611	1.162001	up	1.221836	0.289051	1.221836
1429454_at	0.026842	1.070896	0.098818	1.070896	up	1.081809	0.113446	1.081809
1429464_at	0.007601	-1.07235	-0.10077	1.072347	down	-1.00789	-0.01134	1.007891
1429537_at	0.032138	1.208882	0.273673	1.208882	up	-1.07727	-0.10738	1.077267
1429539_at	0.008731	1.022751	0.032455	1.022751	up	1.145773	0.196321	1.145773
1429550_at	0.039314	-1.12586	-0.17102	1.125857	down	-1.10403	-0.14278	1.104029
1429576_at	0.031712	-1.00822	-0.01181	1.00822	down	-1.03629	-0.05142	1.036286
1429648_at	0.029875	-1.06018	-0.08431	1.060184	down	-1.01503	-0.02152	1.015031
1429679_at	0.022883	1.193931	0.255719	1.193931	up	-1.23806	-0.30808	1.238063
1429821_at	0.034583	-1.03542	-0.05022	1.035421	down	-1.03729	-0.05281	1.037285
1429926_at	0.031075	1.072892	0.101505	1.072892	up	-1.12879	-0.17478	1.128793
1429931_at	0.015769	1.147109	0.198003	1.147109	up	1.182862	0.242282	1.182862
1429972_s_at	0.024931	-1.0188	-0.02687	1.018803	down	-1.0556	-0.07806	1.055598
1429984_at	0.004592	-1.14561	-0.19611	1.145605	down	-1.11856	-0.16164	1.118557
1429990_at	0.013506	-1.11269	-0.15405	1.112688	down	-1.15421	-0.20691	1.15421
1430004_s_at	0.02374	-1.13812	-0.18665	1.138118	down	-1.02563	-0.03651	1.025629
1430034_at	0.048264	-1.00433	-0.00623	1.00433	down	1.025136	0.035815	1.025136
1430036_at	0.02208	-1.02418	-0.03446	1.024175	down	-1.57126	-0.65192	1.571256
1430064_at	0.022549	1.035601	0.050468	1.035601	up	1.142819	0.192596	1.142819
1430081_at	0.048499	1.169515	0.225911	1.169515	up	1.287308	0.364357	1.287308
1430129_a_at	0.037008	1.037113	0.052573	1.037113	up	1.195874	0.258065	1.195874
1430168_at	0.018621	1.023517	0.033536	1.023517	up	-1.03328	-0.04722	1.033275
1430208_at	0.011157	-1.06362	-0.08899	1.063624	down	-1.1495	-0.201	1.149497
1430229_at	0.046521	1.14814	0.199299	1.14814	up	1.160748	0.215055	1.160748
1430255_at	0.04858	1.070607	0.098428	1.070607	up	-1.02971	-0.04224	1.029714
1430268_at	0.033644	1.111531	0.152549	1.111531	up	1.195945	0.258152	1.195945
1430410_at	0.026407	1.048151	0.067846	1.048151	up	-1.1354	-0.1832	1.1354
1430413_at	0.021116	-1.02513	-0.0358	1.025127	down	1.074165	0.103215	1.074165
1430416_at	0.037149	-1.02767	-0.03937	1.027669	down	-1.16452	-0.21974	1.164521
1430422_at	0.032575	1.188453	0.249085	1.188453	up	1.056514	0.079312	1.056514
1430435_at	0.008981	-1.0436	-0.06157	1.043604	down	-1.07425	-0.10332	1.074246
1430442_at	0.025982	1.186984	0.247301	1.186984	up	1.119362	0.162676	1.119362
1430452_at	0.00313	1.031598	0.04488	1.031598	up	-1.08105	-0.11243	1.081047
1430515_s_at	0.008511	1.035297	0.050044	1.035297	up	1.058697	0.08229	1.058697
1430534_at	0.013215	-1.31097	-0.39064	1.310971	down	-1.81921	-0.86331	1.819208
1430577_at	0.021966	-1.20992	-0.27491	1.209918	down	1.020785	0.029679	1.020785
1430639_at	0.046949	-1.08946	-0.12361	1.089456	down	-1.0668	-0.09329	1.066797
1430641_at	0.035689	-1.12827	-0.17411	1.128266	down	-1.07228	-0.10069	1.072283
1430648_at	0.04646	1.003817	0.005496	1.003817	up	-1.04274	-0.06037	1.042736
1430728_at	0.041786	1.002906	0.004186	1.002906	up	-1.03511	-0.04979	1.035113
1430729_at	0.008817	1.187029	0.247355	1.187029	up	1.046581	0.065684	1.046581
1430748_at	0.017824	-1.23737	-0.30727	1.237368	down	-1.04142	-0.05855	1.041417

1430756_at	0.013116	1.075312	0.104755	1.075312	up	1.304502	0.3835	1.304502
1430757_at	0.049212	-1.01385	-0.01984	1.013848	down	-1.17554	-0.23332	1.175537
1430786_at	0.007653	-1.18119	-0.24025	1.181195	down	-1.79531	-0.84423	1.795305
1430807_at	0.015424	1.133156	0.180346	1.133156	up	-1.10651	-0.14601	1.106507
1430857_s_at	0.031449	-1.15019	-0.20187	1.150186	down	1.038047	0.053871	1.038047
1430871_at	0.006829	1.141596	0.191052	1.141596	up	-1.06575	-0.09187	1.065754
1430930_at	0.041041	-1.07687	-0.10685	1.076874	down	-1.0044	-0.00633	1.004396
1430997_at	0.002155	-1.10702	-0.14669	1.107025	down	1.061571	0.086201	1.061571
1431047_at	0.030986	-1.09842	-0.13543	1.09842	down	-1.15498	-0.20787	1.154983
1431073_at	0.001331	-1.01495	-0.02142	1.014955	down	1.040933	0.057877	1.040933
1431089_at	0.003951	1.060672	0.084979	1.060672	up	1.249173	0.320974	1.249173
1431148_at	0.006829	1.172296	0.229337	1.172296	up	1.368483	0.452577	1.368483
1431158_at	0.010189	1.116607	0.159121	1.116607	up	1.037893	0.053658	1.037893
1431258_at	0.022704	-1.14964	-0.20118	1.149637	down	-1.03925	-0.05555	1.039255
1431273_at	0.009242	-1.03141	-0.04461	1.031407	down	1.032516	0.046164	1.032516
1431297_a_at	0.045191	1.091394	0.126172	1.091394	up	-1.09728	-0.13394	1.097283
1431323_at	0.012883	1.024515	0.034941	1.024515	up	1.091977	0.126943	1.091977
1431325_at	0.009143	-1.24259	-0.31335	1.242588	down	-1.13553	-0.18336	1.135525
1431381_at	0.019769	1.060851	0.085222	1.060851	up	1.2191	0.285816	1.2191
1431465_s_at	0.029192	-1.04164	-0.05886	1.041643	down	-1.16063	-0.21491	1.160635
1431599_at	0.039245	1.001739	0.002506	1.001739	up	-1.10142	-0.13936	1.101416
1431637_at	0.018611	-1.15085	-0.2027	1.150853	down	-1.06545	-0.09146	1.065448
1431780_at	0.032001	-1.03762	-0.05328	1.037621	down	-1.23788	-0.30787	1.237882
1431842_at	0.047217	-1.14815	-0.1993	1.148145	down	-1.22223	-0.28952	1.22223
1431924_at	0.018461	1.025991	0.037018	1.025991	up	1.016377	0.023436	1.016377
1431956_at	0.012132	-1.00706	-0.01015	1.00706	down	-1.06798	-0.09488	1.067976
1431977_at	0.029576	1.07578	0.105383	1.07578	up	-1.01749	-0.02501	1.017486
1432008_at	0.025785	-1.05275	-0.07416	1.052745	down	-1.05892	-0.0826	1.058922
1432059_x_at	0.021026	1.278394	0.354332	1.278394	up	1.809228	0.855374	1.809228
1432141_x_at	0.006811	-1.00534	-0.00769	1.005344	down	1.0049	0.007052	1.0049
1432186_at	0.034744	-1.01181	-0.01693	1.011807	down	1.075808	0.10542	1.075808
1432267_at	0.044837	1.070606	0.098428	1.070606	up	1.020034	0.028618	1.020034
1432447_a_at	0.010696	1.062192	0.087044	1.062192	up	1.085457	0.118302	1.085457
1432449_at	0.02627	-1.26362	-0.33756	1.263617	down	-1.17124	-0.22804	1.171243
1432566_at	0.026082	-1.32445	-0.4054	1.324452	down	-1.08325	-0.11536	1.083246
1432601_at	0.036973	-1.12532	-0.17034	1.125325	down	-1.153	-0.20539	1.153
1432730_at	0.040193	-1.03644	-0.05163	1.036438	down	-1.1099	-0.15042	1.109895
1432787_at	0.025602	-1.1695	-0.22589	1.1695	down	-1.16325	-0.21816	1.163251
1432840_at	0.044395	1.138429	0.187044	1.138429	up	-1.01769	-0.0253	1.017694
1432853_at	0.016325	-1.02863	-0.04073	1.028631	down	-1.09802	-0.13491	1.098024
1432869_at	0.029492	-1.10895	-0.14919	1.108949	down	-1.13088	-0.17744	1.130878
1432905_at	0.048681	-1.07512	-0.1045	1.075125	down	-1.19366	-0.25539	1.193659
1433048_at	0.039575	-1.04397	-0.06208	1.043968	down	-1.25274	-0.32509	1.252743
1433091_at	0.023636	-1.066	-0.09221	1.066005	down	-1.00982	-0.0141	1.00982
1433131_at	0.009836	-1.03614	-0.05122	1.036139	down	-1.1387	-0.18739	1.138705
1433176_at	0.002316	-1.16629	-0.22193	1.166292	down	-1.0546	-0.07669	1.054598
1433218_at	0.036187	-1.06523	-0.09116	1.065228	down	-1.05289	-0.07436	1.052892

1433280_at	0.014742	-1.00016	-2.24E-04	1.000156	down	-1.01695	-0.02425	1.01695
1433370_at	0.022487	-1.18538	-0.24534	1.185375	down	1.014766	0.021147	1.014766
1433396_at	0.043025	-1.10412	-0.1429	1.104123	down	-1.01876	-0.02681	1.018758
1433523_at	0.045622	1.091374	0.126145	1.091374	up	-1.01712	-0.02448	1.017115
1433524_at	0.034607	1.174546	0.232104	1.174546	up	1.093947	0.129543	1.093947
1433555_at	0.046342	-1.0676	-0.09437	1.067598	down	1.096454	0.132845	1.096454
1433561_at	0.026209	-1.05077	-0.07144	1.050768	down	-1.00571	-0.00821	1.005706
1433637_at	0.005784	1.098803	0.135932	1.098803	up	1.129237	0.175349	1.129237
1433653_at	0.024346	-1.04875	-0.06868	1.048755	down	-1.12807	-0.17386	1.128072
1433657_at	0.021457	1.035367	0.050142	1.035367	up	-1.16203	-0.21665	1.162033
1433692_at	5.82E-04	1.135697	0.183578	1.135697	up	1.144034	0.19413	1.144034
1433763_at	0.013681	1.061514	0.086123	1.061514	up	1.033072	0.04694	1.033072
1433817_at	0.039826	1.064149	0.089701	1.064149	up	1.155264	0.208223	1.155264
1433819_s_at	0.022915	-1.03443	-0.04884	1.034431	down	1.16742	0.223323	1.16742
1433849_at	0.009818	-1.03833	-0.05427	1.038332	down	-1.01629	-0.02331	1.01629
1433861_at	0.01553	-1.09997	-0.13747	1.099974	down	-1.0697	-0.0972	1.069695
1433868_at	0.02965	1.141756	0.191255	1.141756	up	1.071094	0.099085	1.071094
1433905_at	0.012239	1.011759	0.016865	1.011759	up	1.094794	0.130659	1.094794
1433927_at	0.029423	1.021514	0.030709	1.021514	up	1.207505	0.272029	1.207505
1433933_s_at	0.025344	1.068217	0.095204	1.068217	up	1.015574	0.022295	1.015574
1433943_at	0.028216	1.153938	0.206566	1.153938	up	1.300722	0.379313	1.300722
1433974_at	0.042823	1.062966	0.088096	1.062966	up	1.158969	0.212842	1.158969
1434002_at	0.043144	1.006103	0.008778	1.006103	up	-1.0602	-0.08434	1.060204
1434058_at	0.008567	-1.02798	-0.03982	1.027984	down	-1.0321	-0.04559	1.032103
1434077_at	0.046595	1.089481	0.123641	1.089481	up	1.122079	0.166175	1.122079
1434154_at	0.03817	1.160584	0.214851	1.160584	up	1.120821	0.164556	1.120821
1434156_at	0.022201	1.120439	0.164065	1.120439	up	1.377588	0.462144	1.377588
1434161_at	0.044333	-1.07202	-0.10033	1.072016	down	1.205666	0.269831	1.205666
1434187_at	0.026686	1.07125	0.099295	1.07125	up	1.16418	0.219314	1.16418
1434221_at	0.01382	1.00252	0.003632	1.00252	up	1.017022	0.024351	1.017022
1434225_at	0.020066	1.045991	0.06487	1.045991	up	1.06883	0.096033	1.06883
1434285_at	0.026423	1.041228	0.058286	1.041228	up	1.148189	0.19936	1.148189
1434304_s_at	0.045015	-1.10313	-0.1416	1.10313	down	1.057148	0.080177	1.057148
1434344_at	0.046647	1.035467	0.050281	1.035467	up	1.207861	0.272454	1.207861
1434354_at	0.040741	1.042305	0.059778	1.042305	up	1.191562	0.252854	1.191562
1434368_at	0.038628	-1.11744	-0.1602	1.117441	down	-1.09026	-0.12467	1.090258
1434477_at	0.003395	-1.08147	-0.11299	1.08147	down	-1.26108	-0.33466	1.261077
1434480_at	0.023013	-1.04841	-0.0682	1.048411	down	-1.17655	-0.23456	1.176551
1434505_a_at	0.033308	1.053051	0.074575	1.053051	up	1.200119	0.263177	1.200119
1434593_at	0.010653	-1.02383	-0.03398	1.023834	down	1.126724	0.172134	1.126724
1434621_at	0.01865	1.310215	0.389803	1.310215	up	1.143895	0.193954	1.143895
1434630_at	0.01123	-1.03809	-0.05394	1.038093	down	1.076065	0.105765	1.076065
1434656_at	0.035215	1.074153	0.1032	1.074153	up	1.082186	0.113948	1.082186
1434708_at	0.031108	-1.02736	-0.03893	1.027355	down	1.07332	0.102081	1.07332
1434788_at	0.04199	-1.03609	-0.05116	1.036094	down	-1.05213	-0.07332	1.052134
1434817_s_at	0.001171	1.26141	0.335037	1.26141	up	1.216946	0.283265	1.216946
1434869_at	0.044546	-1.0656	-0.09167	1.065599	down	1.049579	0.06981	1.049579

1434880_at	0.045044	1.146661	0.197439	1.146661	up		1.060503	0.084749	1.060503
1434887_at	0.021828	-1.00732	-0.01052	1.007321	down		1.073203	0.101923	1.073203
1434894_at	0.047744	1.148136	0.199293	1.148136	up		-1.02977	-0.04232	1.02977
1434956_at	0.045841	1.00882	0.012669	1.00882	up		-1.09141	-0.1262	1.091412
1434982_at	0.015718	-1.33428	-0.41606	1.334276	down		-1.15018	-0.20186	1.15018
1435010_at	0.030345	-1.07209	-0.10043	1.072095	down		-1.04014	-0.05678	1.040145
1435017_at	0.02754	1.049703	0.069981	1.049703	up		1.007666	0.011017	1.007666
1435024_at	0.045613	1.054125	0.076046	1.054125	up		-1.13551	-0.18334	1.13551
1435059_at	0.006064	1.168612	0.224796	1.168612	up		1.082665	0.114586	1.082665
1435088_at	0.01102	1.06244	0.087381	1.06244	up		-1.06863	-0.09577	1.068632
1435109_at	0.039582	1.037193	0.052685	1.037193	up		-1.04646	-0.06552	1.04646
1435142_at	0.030026	1.042316	0.059793	1.042316	up		1.094567	0.13036	1.094567
1435188_at	5.32E-05	1.162406	0.217114	1.162406	up		1.021055	0.03006	1.021055
1435229_at	0.008693	1.035116	0.049792	1.035116	up		1.095595	0.131714	1.095595
1435234_at	0.049196	1.097619	0.134378	1.097619	up		1.157894	0.211503	1.157894
1435253_at	0.014903	1.002429	0.003501	1.002429	up		1.00689	0.009906	1.00689
1435264_at	0.040872	-1.17012	-0.22666	1.170119	down		-1.64068	-0.71429	1.64068
1435280_at	0.005971	1.095204	0.1312	1.095204	up		-1.15613	-0.20931	1.156132
1435319_at	0.018579	-1.01561	-0.02235	1.015614	down		1.103442	0.142011	1.103442
1435339_at	0.035201	-1.0249	-0.03548	1.024901	down		1.052758	0.074173	1.052758
1435343_at	0.02157	-1.13396	-0.18137	1.133962	down		-1.01892	-0.02704	1.018922
1435366_at	0.004613	1.323603	0.404471	1.323603	up		1.23463	0.304079	1.23463
1435384_at	0.023395	-1.14405	-0.19415	1.14405	down		-1.00939	-0.01348	1.009385
1435425_at	0.040522	-1.11775	-0.16059	1.117748	down		1.072416	0.100865	1.072416
1435459_at	0.03681	1.283353	0.359919	1.283353	up		1.589332	0.66842	1.589332
1435472_at	0.009637	-1.05091	-0.07164	1.050911	down		1.023432	0.033415	1.023432
1435475_at	0.008119	-1.01078	-0.01547	1.010781	down		1.069463	0.096886	1.069463
1435484_at	0.013037	-1.08376	-0.11605	1.083763	down		-1.13179	-0.17861	1.131789
1435489_at	0.011887	1.069547	0.097	1.069547	up		-1.01309	-0.01876	1.013091
1435490_at	0.028659	-1.088	-0.12168	1.088	down		1.002888	0.004161	1.002888
1435499_at	0.044891	1.27829	0.354215	1.27829	up		1.165188	0.220563	1.165188
1435512_at	0.049234	-1.094	-0.12962	1.094004	down		1.053848	0.075667	1.053848
1435536_at	0.032761	1.117116	0.159779	1.117116	up		-1.0163	-0.02332	1.016297
1435546_a_at	0.012307	-1.13221	-0.17915	1.132214	down		-1.06257	-0.08756	1.062569
1435576_at	0.006076	1.033949	0.048165	1.033949	up		1.061274	0.085797	1.061274
1435589_at	0.01742	1.045014	0.063523	1.045014	up		-1.21149	-0.27678	1.211487
1435653_at	0.019712	1.011142	0.015986	1.011142	up		-1.03848	-0.05447	1.038481
1435663_at	0.019287	-1.38524	-0.47013	1.385238	down		-1.3899	-0.47498	1.389903
1435775_at	0.043722	1.052291	0.073533	1.052291	up		1.134753	0.182378	1.134753
1435888_at	0.012092	1.286215	0.363131	1.286215	up		1.103102	0.141566	1.103102
1435892_at	0.01128	1.088925	0.122905	1.088925	up		-1.0177	-0.02531	1.017697
1435893_at	0.012385	-1.03357	-0.04763	1.033568	down		1.051354	0.072249	1.051354
1435928_at	0.036767	1.150413	0.202152	1.150413	up		1.152259	0.204464	1.152259
1435940_at	6.19E-04	1.068195	0.095175	1.068195	up		1.162256	0.216927	1.162256
1435961_at	0.046978	1.066665	0.093107	1.066665	up		1.078602	0.109162	1.078602
1435990_at	0.03122	-1.07086	-0.09877	1.070858	down		-1.50796	-0.5926	1.50796
1435993_at	0.039362	1.166586	0.222293	1.166586	up		1.168577	0.224752	1.168577

1436008_at	0.015378	-1.22363	-0.29117	1.22363	down	-1.10901	-0.14927	1.109009
1436010_at	0.032657	-1.04056	-0.05736	1.04056	down	1.056917	0.079862	1.056917
1436013_at	0.004957	-1.23923	-0.30944	1.23923	down	-1.34669	-0.42942	1.346694
1436043_at	0.005609	1.125068	0.170012	1.125068	up	1.147757	0.198817	1.147757
1436044_at	0.033581	1.1139	0.155619	1.1139	up	1.145324	0.195755	1.145324
1436055_at	0.003296	1.694296	0.760686	1.694296	up	1.041206	0.058256	1.041206
1436100_at	0.034586	1.247587	0.319141	1.247587	up	1.124973	0.16989	1.124973
1436111_at	0.004498	-1.03976	-0.05626	1.039765	down	-1.07114	-0.09915	1.071144
1436118_at	0.029077	-1.064	-0.0895	1.064002	down	-1.13428	-0.18178	1.134281
1436171_at	0.018503	-1.06671	-0.09317	1.066711	down	-1.19646	-0.25877	1.19646
1436191_at	0.039479	-1.10723	-0.14696	1.107231	down	-1.05066	-0.0713	1.050662
1436210_at	0.048996	-1.0619	-0.08665	1.0619	down	1.135472	0.183292	1.135472
1436256_at	0.015488	-1.16032	-0.21452	1.160319	down	-1.06354	-0.08887	1.063538
1436265_at	0.029866	-1.11031	-0.15097	1.110312	down	-1.10111	-0.13896	1.101109
1436275_at	0.020265	1.039923	0.056476	1.039923	up	-1.04038	-0.0571	1.040375
1436293_x_at	0.044289	-1.06921	-0.09655	1.069215	down	1.055312	0.07767	1.055312
1436314_at	0.049276	-1.15181	-0.2039	1.15181	down	-1.00122	-0.00175	1.001217
1436354_at	0.025305	1.120663	0.164352	1.120663	up	1.014864	0.021286	1.014864
1436373_at	0.029337	-1.0139	-0.01992	1.013902	down	-1.10061	-0.13831	1.100612
1436377_at	0.013231	1.106077	0.145452	1.106077	up	1.188533	0.249181	1.188533
1436401_at	0.047424	-1.00498	-0.00717	1.004983	down	1.011021	0.015813	1.011021
1436410_at	0.042837	-1.14643	-0.19715	1.146431	down	1.00505	0.007268	1.00505
1436437_at	0.045565	-1.13229	-0.17924	1.132288	down	-1.13241	-0.1794	1.132415
1436438_s_at	0.009762	1.031935	0.045353	1.031935	up	1.012858	0.018431	1.012858
1436444_at	0.004742	1.102036	0.140171	1.102036	up	1.018703	0.026733	1.018703
1436476_at	0.007881	1.039976	0.05655	1.039976	up	-1.05031	-0.07082	1.05031
1436478_at	0.012072	1.062403	0.087331	1.062403	up	-1.17923	-0.23785	1.179231
1436513_at	0.040549	1.181504	0.240624	1.181504	up	-1.12083	-0.16457	1.120833
1436536_at	0.019073	1.17053	0.227162	1.17053	up	1.238206	0.308251	1.238206
1436560_at	0.033357	1.195906	0.258104	1.195906	up	1.145862	0.196433	1.145862
1436625_at	0.042787	-1.04499	-0.06348	1.044986	down	-1.06225	-0.08712	1.062247
1436646_at	0.005697	1.264652	0.33874	1.264652	up	-1.05366	-0.07541	1.053662
1436672_at	0.02976	-1.01315	-0.01885	1.013151	down	1.055252	0.077587	1.055252
1436711_at	0.038259	1.039953	0.056519	1.039953	up	1.056867	0.079794	1.056867
1436712_at	0.019674	-1.10701	-0.14667	1.107008	down	-1.28187	-0.35825	1.28187
1436755_at	0.02024	-1.22051	-0.28748	1.220507	down	-1.67742	-0.74625	1.677422
1436778_at	0.047217	1.012302	0.017639	1.012302	up	-1.057	-0.07997	1.056995
1436920_at	0.035254	1.827839	0.870139	1.827839	up	1.255578	0.328352	1.255578
1437022_at	0.049851	1.083387	0.115549	1.083387	up	-1.13898	-0.18774	1.138982
1437049_at	0.030701	1.152917	0.205289	1.152917	up	1.263735	0.337694	1.263735
1437065_at	0.009864	1.147093	0.197982	1.147093	up	-1.1668	-0.22256	1.166799
1437127_at	0.019794	-1.03629	-0.05143	1.036295	down	1.06593	0.092112	1.06593
1437174_at	0.010445	1.010289	0.014767	1.010289	up	1.227038	0.29518	1.227038
1437221_at	0.019577	-1.28743	-0.36449	1.28743	down	-1.02505	-0.0357	1.025054
1437222_x_at	0.043235	-1.18822	-0.24881	1.188224	down	1.006835	0.009827	1.006835
1437244_at	0.037369	1.123802	0.168388	1.123802	up	1.348224	0.431061	1.348224
1437387_at	0.005879	-1.00416	-0.00599	1.004158	down	1.00048	6.93E-04	1.00048

1437410_at	0.045764	1.142533	0.192235	1.142533	up	-1.00375	-0.00539	1.003745
1437424_at	0.041222	-1.06511	-0.09101	1.065113	down	1.12273	0.167011	1.12273
1437462_x_at	0.013069	-1.08822	-0.12197	1.08822	down	-1.16211	-0.21674	1.162108
1437478_s_at	0.014349	-1.00163	-0.00234	1.001626	down	1.124813	0.169685	1.124813
1437492_at	0.023247	1.15714	0.210564	1.15714	up	-1.08405	-0.11643	1.084052
1437506_at	0.044827	1.137901	0.186374	1.137901	up	1.056234	0.078929	1.056234
1437598_at	0.024257	1.008974	0.012889	1.008974	up	-1.11929	-0.16259	1.119293
1437617_x_at	0.039309	1.057739	0.080984	1.057739	up	-1.10005	-0.13757	1.100053
1437623_x_at	0.042499	-1.21582	-0.28193	1.215824	down	-1.12943	-0.17559	1.129427
1437702_at	0.028252	-1.05799	-0.08132	1.057985	down	-1.1299	-0.1762	1.129905
1437745_at	0.045546	1.322908	0.403713	1.322908	up	1.203285	0.266978	1.203285
1437771_at	0.002457	-1.02038	-0.02911	1.020381	down	-1.29424	-0.3721	1.294236
1437788_at	0.048748	1.047642	0.067146	1.047642	up	1.139002	0.18777	1.139002
1437820_at	4.41E-04	-1.1108	-0.1516	1.110798	down	-1.04228	-0.05974	1.042277
1437857_at	0.007079	1.055555	0.078002	1.055555	up	1.206226	0.2705	1.206226
1437879_at	0.004123	-1.02186	-0.03119	1.021855	down	1.02639	0.037579	1.02639
1437921_x_at	0.037849	1.021666	0.030923	1.021666	up	-1.01045	-0.01499	1.010447
1437933_at	0.035201	1.183733	0.243344	1.183733	up	1.20176	0.265149	1.20176
1438032_at	0.021581	-1.00028	-4.08E-04	1.000283	down	-1.01233	-0.01768	1.012327
1438060_at	0.017319	-1.18294	-0.24238	1.182942	down	-1.0114	-0.01635	1.011397
1438072_at	0.024765	1.164391	0.219576	1.164391	up	-1.25711	-0.33012	1.257114
1438102_at	0.028338	-1.11759	-0.16039	1.117591	down	-1.03625	-0.05137	1.036245
1438123_at	0.045752	1.084655	0.117236	1.084655	up	1.264733	0.338833	1.264733
1438229_at	0.008454	1.066047	0.092271	1.066047	up	-1.0661	-0.09235	1.066103
1438270_at	0.036862	-1.05039	-0.07093	1.050392	down	-1.0977	-0.13448	1.097701
1438275_at	0.007865	-1.05859	-0.08214	1.05859	down	1.109128	0.149426	1.109128
1438334_at	0.047472	1.077442	0.10761	1.077442	up	-1.08281	-0.11478	1.082808
1438412_at	0.001201	-1.02824	-0.04018	1.028239	down	-1.02294	-0.03272	1.022936
1438424_at	0.004238	1.164892	0.220197	1.164892	up	1.285701	0.362555	1.285701
1438425_at	6.61E-04	1.352303	0.435418	1.352303	up	1.063952	0.089433	1.063952
1438432_at	0.045284	1.192166	0.253586	1.192166	up	1.019848	0.028354	1.019848
1438470_at	0.008853	-1.03989	-0.05644	1.039895	down	-1.11586	-0.15816	1.115861
1438484_at	0.00282	-1.09669	-0.13316	1.096693	down	1.124831	0.169709	1.124831
1438488_at	0.012844	-1.07978	-0.11074	1.079783	down	1.054195	0.076142	1.054195
1438490_at	0.002901	1.043874	0.061947	1.043874	up	1.285679	0.36253	1.285679
1438491_x_at	0.047262	-1.00872	-0.01252	1.008716	down	1.103927	0.142645	1.103927
1438648_x_at	0.04923	1.368948	0.453067	1.368948	up	1.345004	0.42761	1.345004
1438660_at	0.043528	-1.07793	-0.10827	1.077935	down	-1.12232	-0.16649	1.122323
1438662_at	0.025486	-1.04443	-0.06271	1.044429	down	1.248164	0.319807	1.248164
1438684_at	0.032502	-1.01518	-0.02173	1.015178	down	-1.24797	-0.31958	1.247971
1438713_at	0.023671	-1.03464	-0.04913	1.03464	down	-1.0552	-0.07752	1.055201
1438725_at	0.015245	-1.08797	-0.12164	1.08797	down	-1.17486	-0.23249	1.174857
1438728_at	7.08E-05	1.029525	0.041978	1.029525	up	1.248152	0.319794	1.248152
1438738_at	0.034889	-1.09833	-0.13531	1.09833	down	-1.18471	-0.24453	1.184707
1438778_at	0.020047	1.029786	0.042345	1.029786	up	-1.06432	-0.08993	1.064318
1438830_at	0.015199	-1.14807	-0.19922	1.148075	down	-1.04069	-0.05754	1.040691
1438858_x_at	0.027407	-1.08111	-0.11251	1.081111	down	-1.66527	-0.73575	1.665266

1438862_at	0.042272	1.114535	0.156441	1.114535	up	1.125745	0.17088	1.125745
1438897_at	0.037939	-1.1093	-0.14965	1.109299	down	-1.09749	-0.1342	1.097488
1439000_at	0.044391	-1.19954	-0.26248	1.199542	down	-1.0336	-0.04768	1.033605
1439050_at	0.034062	-1.02705	-0.0385	1.027047	down	1.379496	0.464141	1.379496
1439087_a_at	0.017716	1.029855	0.042441	1.029855	up	1.118262	0.161258	1.118262
1439095_at	0.00318	1.079307	0.110105	1.079307	up	1.007166	0.010301	1.007166
1439104_at	0.023226	1.074001	0.102996	1.074001	up	-1.07396	-0.10294	1.073962
1439108_at	0.014969	1.099538	0.136897	1.099538	up	-1.21721	-0.28357	1.217205
1439179_a_at	0.01679	-1.191	-0.25217	1.190998	down	-1.10815	-0.14815	1.108149
1439275_s_at	0.007544	-1.02861	-0.04069	1.028606	down	-1.22797	-0.29628	1.227973
1439307_at	0.038814	-1.17159	-0.22847	1.171588	down	-1.11264	-0.15399	1.112642
1439340_at	0.001593	1.053308	0.074928	1.053308	up	1.07588	0.105517	1.07588
1439488_at	0.003371	1.087275	0.120716	1.087275	up	-1.00539	-0.00775	1.005388
1439497_at	0.045425	1.008052	0.01157	1.008052	up	-1.11697	-0.15959	1.116973
1439555_at	0.001456	-1.13174	-0.17854	1.131739	down	-1.09021	-0.1246	1.090209
1439576_at	0.046704	1.09543	0.131498	1.09543	up	1.134823	0.182467	1.134823
1439592_at	0.012315	1.082403	0.114237	1.082403	up	1.100295	0.13789	1.100295
1439616_at	0.023747	1.073722	0.10262	1.073722	up	-1.15295	-0.20533	1.152949
1439649_at	0.008778	1.22276	0.290141	1.22276	up	1.26503	0.339172	1.26503
1439667_at	0.041754	-1.00268	-0.00385	1.002676	down	-1.07206	-0.10039	1.072064
1439671_at	0.007035	-1.07058	-0.09839	1.070575	down	1.018945	0.027076	1.018945
1439789_at	0.015791	1.135002	0.182695	1.135002	up	1.023551	0.033583	1.023551
1439827_at	0.020134	1.045023	0.063535	1.045023	up	-1.35943	-0.443	1.359429
1439851_at	0.020546	1.017327	0.024783	1.017327	up	-1.00342	-0.00492	1.003418
1439859_at	0.017915	-1.07122	-0.09925	1.071218	down	-1.05811	-0.08149	1.05811
1439906_at	0.021521	-1.08775	-0.12134	1.087747	down	-1.23416	-0.30353	1.234162
1439979_at	0.029158	1.021542	0.030749	1.021542	up	1.051245	0.072098	1.051245
1439992_at	0.0452	-1.05451	-0.07657	1.054511	down	-1.01822	-0.02605	1.018219
1440000_at	0.022304	-1.10529	-0.14443	1.105293	down	-1.29154	-0.36909	1.291541
1440041_at	0.004563	-1.18227	-0.24156	1.182274	down	-1.1256	-0.1707	1.125601
1440046_at	0.049249	-1.1199	-0.16337	1.119903	down	-1.2135	-0.27917	1.213495
1440064_at	0.005639	1.137473	0.185832	1.137473	up	1.113506	0.15511	1.113506
1440085_at	0.02147	1.188656	0.249331	1.188656	up	-1.00296	-0.00426	1.002957
1440086_at	0.021576	-1.00481	-0.00693	1.004813	down	1.031047	0.04411	1.031047
1440119_at	0.038056	-1.14298	-0.1928	1.142978	down	-1.30824	-0.38763	1.308238
1440161_at	0.022555	-1.00379	-0.00546	1.003792	down	-1.29181	-0.3694	1.291815
1440200_at	0.00408	1.003623	0.005217	1.003623	up	1.153784	0.206373	1.153784
1440223_at	0.034781	1.173232	0.230489	1.173232	up	-1.01657	-0.0237	1.016565
1440232_at	0.03866	-1.14032	-0.18943	1.140316	down	-1.14076	-0.18999	1.140756
1440269_at	0.032627	-1.12694	-0.17241	1.126938	down	-1.00853	-0.01226	1.008532
1440282_at	0.011765	1.072814	0.1014	1.072814	up	1.054365	0.076374	1.054365
1440314_at	0.028525	1.299545	0.378007	1.299545	up	-1.21446	-0.28032	1.214462
1440318_at	0.044185	1.153624	0.206174	1.153624	up	1.290453	0.367877	1.290453
1440364_a_at	0.004444	-1.03014	-0.04285	1.030144	down	-1.06881	-0.09601	1.068814
1440369_at	0.020241	-1.03149	-0.04473	1.031489	down	1.007424	0.010671	1.007424
1440378_at	0.026713	-1.06269	-0.08772	1.062693	down	1.072403	0.100847	1.072403
1440390_at	0.003008	-1.04216	-0.05958	1.042159	down	-1.10167	-0.13969	1.10167

1440527_at	0.030729	-1.27504	-0.35054	1.275037	down	-1.85097	-0.88828	1.85097
1440573_at	0.049529	1.173173	0.230416	1.173173	up	-1.21534	-0.28136	1.215341
1440583_at	0.034392	-1.30851	-0.38793	1.308513	down	-1.04018	-0.05683	1.04018
1440665_at	0.007826	1.041397	0.05852	1.041397	up	-1.1315	-0.17824	1.1315
1440715_s_at	1.35E-04	-1.08424	-0.11669	1.084242	down	1.068034	0.094958	1.068034
1440718_at	0.045643	-1.11141	-0.15239	1.111409	down	-1.11334	-0.1549	1.113342
1440719_at	0.00898	-1.05395	-0.07581	1.053951	down	-1.07371	-0.1026	1.07371
1440755_at	0.024037	-1.03922	-0.0555	1.03922	down	1.116762	0.159322	1.116762
1440758_at	0.025039	1.091475	0.126279	1.091475	up	1.038469	0.054458	1.038469
1440925_at	0.035593	1.034783	0.049329	1.034783	up	-1.04577	-0.06456	1.045769
1440943_at	0.006137	1.20319	0.266865	1.20319	up	1.026238	0.037366	1.026238
1440969_at	0.039666	1.140532	0.189706	1.140532	up	-1.05017	-0.07062	1.050167
1440972_at	0.005645	-1.07499	-0.10432	1.074989	down	-1.1414	-0.1908	1.141397
1440980_at	0.02012	-1.16584	-0.22138	1.165844	down	-1.17135	-0.22818	1.171354
1441010_at	0.046944	-1.00506	-0.00728	1.005061	down	1.040964	0.057921	1.040964
1441055_at	0.047294	-1.31587	-0.39602	1.315871	down	-1.15149	-0.2035	1.151487
1441143_at	0.046736	-1.02083	-0.02974	1.020831	down	-1.04444	-0.06273	1.04444
1441175_at	0.031315	-1.15857	-0.21234	1.158565	down	-1.08297	-0.115	1.082972
1441226_at	0.017094	1.138725	0.18742	1.138725	up	1.368341	0.452428	1.368341
1441327_a_at	0.025834	1.198602	0.261353	1.198602	up	1.288384	0.365562	1.288384
1441338_at	0.002565	-1.17954	-0.23823	1.179541	down	-1.36389	-0.44773	1.363892
1441339_at	0.048463	-1.0083	-0.01193	1.008303	down	-1.21578	-0.28189	1.215785
1441376_at	0.003968	1.038874	0.05502	1.038874	up	1.092865	0.128115	1.092865
1441377_at	0.025665	1.097029	0.133601	1.097029	up	1.125517	0.170588	1.125517
1441438_at	0.035013	1.026482	0.037708	1.026482	up	-1.1028	-0.14117	1.102798
1441453_at	0.035483	-1.06336	-0.08862	1.063356	down	-1.18308	-0.24255	1.183081
1441456_at	0.028552	-1.13035	-0.17677	1.130353	down	-1.22513	-0.29294	1.225131
1441461_at	0.027884	-1.01505	-0.02155	1.015047	down	1.019843	0.028347	1.019843
1441572_at	0.04042	1.270739	0.345668	1.270739	up	1.050884	0.071603	1.050884
1441585_at	0.008677	-1.0115	-0.01649	1.011498	down	-1.01879	-0.02686	1.018791
1441591_at	0.017216	1.119012	0.162225	1.119012	up	1.16496	0.22028	1.16496
1441607_at	0.019884	1.292834	0.370537	1.292834	up	1.099764	0.137194	1.099764
1441615_at	0.024305	1.171171	0.227952	1.171171	up	1.07196	0.100251	1.07196
1441639_at	0.047264	1.005796	0.008337	1.005796	up	1.078395	0.108885	1.078395
1441697_at	0.015804	-1.19123	-0.25245	1.191227	down	-1.06879	-0.09598	1.068788
1441728_at	0.020978	1.014764	0.021144	1.014764	up	1.063047	0.088205	1.063047
1441753_at	0.047999	1.029202	0.041526	1.029202	up	1.143433	0.193372	1.143433
1441807_s_at	0.015814	-1.15848	-0.21223	1.15848	down	-1.45956	-0.54554	1.459565
1441819_x_at	0.025933	-1.33836	-0.42046	1.338356	down	-1.09033	-0.12476	1.090326
1441905_x_at	0.048907	1.232689	0.301809	1.232689	up	1.323743	0.404623	1.323743
1441923_s_at	0.005809	-1.00195	-0.00281	1.001949	down	-1.16595	-0.22151	1.165954
1441924_x_at	0.007393	-1.04676	-0.06593	1.046758	down	-1.17538	-0.23313	1.175384
1441946_at	0.002168	-1.3912	-0.47633	1.391198	down	-1.7679	-0.82203	1.767896
1441952_x_at	0.005981	1.038682	0.054754	1.038682	up	-1.1028	-0.14117	1.102798
1441996_at	0.031025	-1.0053	-0.00762	1.005297	down	-1.05301	-0.07452	1.053008
1442051_at	0.011991	1.056786	0.079684	1.056786	up	1.209927	0.27492	1.209927
1442119_at	0.008598	1.17709	0.235224	1.17709	up	1.302737	0.381545	1.302737

1442131_at	0.00223	-1.21772	-0.28418	1.217719	down	-1.11046	-0.15115	1.110457
1442139_at	0.022027	1.014525	0.020804	1.014525	up	-1.06391	-0.08937	1.063907
1442141_at	0.041071	-1.01093	-0.01568	1.010929	down	-1.06806	-0.09499	1.068059
1442204_at	0.028295	-1.1657	-0.22119	1.165696	down	-1.36064	-0.44429	1.36064
1442357_at	0.044502	1.134421	0.181956	1.134421	up	1.008877	0.01275	1.008877
1442460_at	0.018623	1.122163	0.166282	1.122163	up	-1.05417	-0.07611	1.054171
1442471_at	0.029477	-1.22595	-0.2939	1.225951	down	-1.11317	-0.15467	1.11317
1442560_at	0.032901	1.007002	0.010067	1.007002	up	1.048124	0.06781	1.048124
1442567_at	0.042329	-1.19286	-0.25443	1.192862	down	1.026995	0.038429	1.026995
1442648_at	0.009873	1.040535	0.057325	1.040535	up	1.154468	0.207228	1.154468
1442662_at	0.011478	1.167631	0.223584	1.167631	up	-1.05685	-0.07977	1.056847
1442676_at	0.044903	-1.00054	-7.75E-04	1.000538	down	1.250253	0.32222	1.250253
1442686_at	0.005158	1.124532	0.169324	1.124532	up	1.117887	0.160774	1.117887
1442785_at	0.045888	1.153254	0.20571	1.153254	up	-1.04556	-0.06428	1.045564
1442917_at	0.035565	-1.06695	-0.0935	1.066953	down	-1.10233	-0.14056	1.102329
1442977_at	0.020431	1.094672	0.130499	1.094672	up	-1.10333	-0.14187	1.103335
1442982_at	0.034353	1.107191	0.146904	1.107191	up	1.100505	0.138166	1.100505
1442985_at	0.042601	-1.128	-0.17377	1.128	down	-1.12762	-0.17328	1.127616
1443066_at	0.007025	-1.15569	-0.20875	1.155688	down	-1.08857	-0.12243	1.088565
1443119_at	0.012582	1.266891	0.341293	1.266891	up	-1.26105	-0.33463	1.261052
1443183_at	0.048454	-1.04319	-0.06101	1.043194	down	1.080418	0.111589	1.080418
1443198_at	0.01273	1.001599	0.002305	1.001599	up	1.074701	0.103935	1.074701
1443232_at	0.040546	1.06598	0.092181	1.06598	up	1.012273	0.017599	1.012273
1443320_at	0.03174	1.162079	0.216708	1.162079	up	1.099684	0.137089	1.099684
1443524_x_at	0.040925	-1.2114	-0.27667	1.211395	down	-1.07135	-0.09944	1.071354
1443640_at	0.026184	-1.00125	-0.0018	1.001247	down	-1.02945	-0.04187	1.029448
1443878_at	0.006149	-1.05695	-0.07991	1.056953	down	1.006949	0.009991	1.006949
1443900_at	0.025883	-1.0515	-0.07246	1.051505	down	1.002882	0.004151	1.002882
1443901_at	0.02913	-1.03607	-0.05113	1.036075	down	-1.13376	-0.18111	1.133757
1443906_at	0.04495	-1.01722	-0.02464	1.017225	down	-1.59654	-0.67495	1.596541
1443910_at	0.036618	-1.16735	-0.22323	1.167345	down	-1.01975	-0.02821	1.019746
1443923_at	0.015266	1.071802	0.100038	1.071802	up	-1.00736	-0.01058	1.007359
1443931_at	0.018677	-1.08329	-0.11541	1.083285	down	-1.13156	-0.17831	1.13156
1443975_at	0.00917	-1.3486	-0.43146	1.348596	down	-1.19512	-0.25715	1.195119
1444082_at	0.046473	-1.01015	-0.01457	1.010148	down	-1.05782	-0.08109	1.057818
1444089_at	0.001669	1.110839	0.15165	1.110839	up	1.114066	0.155834	1.114066
1444124_a_at	0.038905	-1.11277	-0.15415	1.112769	down	-1.2049	-0.26891	1.204898
1444143_at	0.046372	1.284469	0.361172	1.284469	up	1.069738	0.097257	1.069738
1444157_a_at	0.019257	1.095596	0.131716	1.095596	up	1.021405	0.030555	1.021405
1444161_at	0.02283	1.017499	0.025027	1.017499	up	1.060937	0.085339	1.060937
1444170_at	0.028854	-1.06644	-0.09281	1.066443	down	1.13226	0.179206	1.13226
1444178_at	0.046829	1.123263	0.167696	1.123263	up	1.048798	0.068738	1.048798
1444199_at	0.040586	1.129234	0.175345	1.129234	up	1.042623	0.060218	1.042623
1444235_at	0.007557	1.339864	0.422086	1.339864	up	1.184458	0.244227	1.184458
1444255_at	8.37E-04	1.149672	0.201222	1.149672	up	1.092387	0.127484	1.092387
1444260_at	0.017944	1.147396	0.198363	1.147396	up	1.043464	0.061381	1.043464
1444439_at	0.045706	-1.22208	-0.28934	1.22208	down	1.09352	0.12898	1.09352

1444464_at	0.04932	-1.11525	-0.15737	1.115252	down	-1.01198	-0.01718	1.011982
1444488_at	0.019194	-1.07851	-0.10904	1.078507	down	-1.16624	-0.22186	1.16624
1444571_at	0.009935	1.148168	0.199333	1.148168	up	1.005467	0.007866	1.005467
1444628_at	0.030842	-1.17491	-0.23255	1.174906	down	-1.12741	-0.17302	1.127413
1444633_at	0.029457	-1.0525	-0.07382	1.052497	down	-1.09003	-0.12437	1.090029
1444637_at	0.012658	-1.07793	-0.10826	1.077926	down	1.00943	0.01354	1.00943
1444640_at	0.03243	1.237087	0.306947	1.237087	up	1.186781	0.247054	1.186781
1444682_at	0.047994	1.057774	0.081031	1.057774	up	1.079978	0.111002	1.079978
1444735_at	0.01353	-1.00664	-0.00955	1.00664	down	-1.00247	-0.00356	1.002472
1444766_at	0.005374	-1.18636	-0.24654	1.186356	down	-1.15986	-0.21395	1.159863
1444769_at	0.030399	-1.1853	-0.24525	1.185302	down	1.039894	0.056437	1.039894
1444789_at	0.007342	-1.13053	-0.177	1.130527	down	-1.27332	-0.3486	1.273325
1444855_at	0.029971	1.11603	0.158376	1.11603	up	1.090825	0.12542	1.090825
1444995_at	0.020652	-1.02152	-0.03072	1.021524	down	1.000879	0.001268	1.000879
1445021_at	6.65E-04	-1.03934	-0.05566	1.039337	down	-1.05169	-0.0727	1.051685
1445097_at	4.40E-04	-1.16086	-0.21519	1.160858	down	-1.10622	-0.14564	1.10622
1445199_at	0.018853	1.191984	0.253364	1.191984	up	1.154659	0.207467	1.154659
1445255_at	0.011492	1.066286	0.092595	1.066286	up	-1.06094	-0.08535	1.060945
1445266_at	0.035703	-1.02056	-0.02935	1.020556	down	1.015027	0.021518	1.015027
1445309_at	0.042572	1.095118	0.131086	1.095118	up	-1.07127	-0.09932	1.07127
1445313_at	0.027835	1.26046	0.333951	1.26046	up	1.092957	0.128236	1.092957
1445320_at	0.022115	-1.2335	-0.30275	1.233497	down	-1.06465	-0.09038	1.064653
1445373_at	0.0497	-1.13213	-0.17903	1.132126	down	-1.08728	-0.12073	1.087284
1445385_at	0.028266	1.066622	0.093049	1.066622	up	1.171731	0.228642	1.171731
1445555_at	0.026506	1.518842	0.602972	1.518842	up	1.059232	0.083018	1.059232
1445632_at	0.028227	-1.03937	-0.05571	1.039369	down	1.040533	0.057322	1.040533
1445679_at	0.013652	1.280678	0.356907	1.280678	up	1.010117	0.014522	1.010117
1445692_x_at	0.04742	-1.28381	-0.36043	1.283808	down	1.044134	0.062306	1.044134
1445838_at	0.011356	1.095392	0.131448	1.095392	up	-1.1215	-0.16543	1.1215
1446021_at	0.012689	1.194851	0.25683	1.194851	up	-1.02263	-0.03228	1.022626
1446056_at	0.043631	1.166676	0.222404	1.166676	up	1.074782	0.104044	1.074782
1446230_at	0.020798	-1.02741	-0.03901	1.027411	down	1.026642	0.037933	1.026642
1446237_at	0.019509	1.032031	0.045486	1.032031	up	-1.18766	-0.24812	1.187662
1446258_at	0.039994	-1.03134	-0.04452	1.031341	down	-1.12298	-0.16733	1.122982
1446294_at	0.016833	-1.15832	-0.21203	1.158317	down	-1.18171	-0.24088	1.181712
1446331_at	0.02313	-1.19258	-0.25409	1.192585	down	-1.37859	-0.46319	1.378586
1446618_at	0.037602	1.08253	0.114407	1.08253	up	1.034641	0.04913	1.034641
1446720_at	0.020115	-1.0411	-0.05811	1.041099	down	-1.07842	-0.10892	1.078418
1446744_at	0.048588	-1.10985	-0.15037	1.109853	down	-1.05814	-0.08153	1.058137
1446771_at	0.013044	1.067205	0.093838	1.067205	up	-1.11353	-0.15514	1.11353
1446773_at	0.006942	-1.08488	-0.11754	1.084883	down	-1.04107	-0.05807	1.041071
1446890_at	0.021766	1.177517	0.235748	1.177517	up	-1.04623	-0.0652	1.046232
1446923_at	0.039345	-1.1338	-0.18117	1.133801	down	1.021656	0.030909	1.021656
1446928_at	0.042239	-1.10707	-0.14675	1.107072	down	-1.46109	-0.54704	1.461089
1446946_at	0.002782	1.039729	0.056207	1.039729	up	-1.12018	-0.16373	1.120183
1447072_at	8.11E-04	1.017116	0.024485	1.017116	up	1.005069	0.007294	1.005069
1447162_at	0.011275	-1.0107	-0.01535	1.010699	down	1.217248	0.283623	1.217248

1447171_at	0.006345	1.156314	0.209534	1.156314	up	-1.15505	-0.20796	1.155053
1447223_at	0.032596	1.112948	0.154386	1.112948	up	1.006043	0.008691	1.006043
1447345_at	0.02602	-1.11069	-0.15146	1.110691	down	-1.13937	-0.18824	1.139373
1447364_x_at	0.038201	1.109792	0.150289	1.109792	up	1.076852	0.106819	1.076852
1447382_at	0.031616	-1.07425	-0.10333	1.074248	down	1.178339	0.236755	1.178339
1447411_at	0.042158	1.060466	0.084699	1.060466	up	-1.03887	-0.05501	1.038867
1447433_at	0.002786	-1.07584	-0.10546	1.075835	down	-1.03822	-0.05411	1.038215
1447483_s_at	0.014495	-1.06366	-0.08904	1.063659	down	1.141841	0.191361	1.141841
1447500_at	0.007211	-1.18772	-0.24819	1.187716	down	-1.30016	-0.37869	1.300157
1447541_s_at	0.030809	-1.1636	-0.2186	1.163602	down	-1.3476	-0.43039	1.347601
1447570_s_at	0.049597	-1.52404	-0.6079	1.524044	down	-1.18074	-0.23969	1.180739
1447609_at	0.015529	1.01917	0.027395	1.01917	up	-1.12142	-0.16532	1.121417
1447672_x_at	0.012449	1.050732	0.071395	1.050732	up	-1.04112	-0.05814	1.041123
1447724_x_at	0.001546	-1.00384	-0.00554	1.003845	down	1.010831	0.015542	1.010831
1447752_x_at	0.037537	1.072284	0.100687	1.072284	up	1.11544	0.157613	1.11544
1447771_at	0.033023	1.09927	0.136545	1.09927	up	-1.17444	-0.23197	1.174435
1447779_x_at	0.004995	1.03808	0.053917	1.03808	up	-1.02862	-0.0407	1.028616
1447818_x_at	0.044432	1.052086	0.073253	1.052086	up	1.138215	0.186773	1.138215
1447823_x_at	0.034699	1.229078	0.297577	1.229078	up	-1.0886	-0.12248	1.088602
1447870_x_at	0.025764	-1.28489	-0.36164	1.284887	down	-1.92645	-0.94594	1.926447
1447931_at	0.028249	-1.03094	-0.04396	1.030942	down	-1.00801	-0.0115	1.008006
1447948_at	0.048288	-1.01452	-0.0208	1.014519	down	-1.13953	-0.18843	1.139527
1452719_at	0.044386	1.06979	0.097328	1.06979	up	1.208223	0.272887	1.208223
1452727_at	0.026034	1.017091	0.024448	1.017091	up	-1.13056	-0.17704	1.130562
1452739_at	0.00288	1.081127	0.112535	1.081127	up	1.056793	0.079693	1.056793
1452761_a_at	0.026732	1.037602	0.053253	1.037602	up	-1.01447	-0.02073	1.014474
1452805_at	0.046762	1.128631	0.174574	1.128631	up	1.089489	0.123652	1.089489
1452833_at	0.031865	1.223873	0.291454	1.223873	up	1.256875	0.329841	1.256875
1452850_s_at	0.013879	1.098517	0.135557	1.098517	up	1.150422	0.202163	1.150422
1452853_at	0.00658	-1.08241	-0.11424	1.082405	down	1.090915	0.125538	1.090915
1452859_at	0.0382	1.050876	0.071592	1.050876	up	1.058702	0.082297	1.058702
1452918_at	0.005711	-1.10747	-0.14726	1.107466	down	1.065703	0.091805	1.065703
1453001_at	0.003076	-1.09851	-0.13555	1.098514	down	-1.0918	-0.12671	1.0918
1453007_at	0.00651	-1.01552	-0.02222	1.015518	down	1.159033	0.212921	1.159033
1453024_at	0.049286	-1.13891	-0.18766	1.138913	down	1.182275	0.241565	1.182275
1453070_at	0.048588	1.227099	0.295252	1.227099	up	-1.01755	-0.0251	1.017548
1453093_at	0.032864	-1.08357	-0.11579	1.083565	down	1.070846	0.098751	1.070846
1453114_at	0.030306	-1.16029	-0.21448	1.160286	down	-1.04258	-0.06016	1.042582
1453116_at	0.019827	-1.07462	-0.10382	1.074618	down	1.097583	0.13433	1.097583
1453123_at	0.017263	1.093845	0.129408	1.093845	up	1.007787	0.011191	1.007787
1453127_at	0.01169	1.145276	0.195696	1.145276	up	-1.05052	-0.0711	1.050516
1453135_at	0.043119	1.10772	0.147594	1.10772	up	-1.01359	-0.01948	1.013595
1453146_at	0.027442	1.152187	0.204375	1.152187	up	-1.04414	-0.06231	1.044139
1453150_at	0.029864	-1.02341	-0.03338	1.023407	down	1.18216	0.241425	1.18216
1453189_at	0.03018	1.100771	0.138514	1.100771	up	1.064908	0.090729	1.064908
1453224_at	0.006472	1.152093	0.204257	1.152093	up	1.203402	0.267119	1.203402
1453286_at	0.038022	1.233029	0.302206	1.233029	up	1.049629	0.069879	1.049629

1453306_at	0.018399	-1.07124	-0.09929	1.071244	down	-1.09539	-0.13144	1.095389
1453310_at	0.040847	-1.18525	-0.2452	1.185255	down	1.099761	0.13719	1.099761
1453326_at	0.003666	1.04463	0.062992	1.04463	up	1.043807	0.061855	1.043807
1453328_at	0.04574	1.059231	0.083017	1.059231	up	1.167874	0.223885	1.167874
1453337_at	0.022366	-1.03831	-0.05423	1.038307	down	-1.01601	-0.02292	1.016015
1453402_at	0.002229	1.203831	0.267632	1.203831	up	1.103873	0.142575	1.103873
1453421_at	0.001402	-1.00607	-0.00873	1.006068	down	1.021592	0.030819	1.021592
1453502_at	0.008185	1.301001	0.379622	1.301001	up	1.052303	0.07355	1.052303
1453513_at	0.032037	-1.02753	-0.03917	1.027525	down	1.041398	0.058522	1.041398
1453520_at	0.033954	-1.09746	-0.13416	1.097456	down	1.057884	0.081181	1.057884
1453539_at	0.029228	-1.07567	-0.10524	1.075674	down	-1.0217	-0.03098	1.021704
1453544_at	0.047344	-1.24537	-0.31657	1.245368	down	-1.04551	-0.06421	1.045514
1453649_at	0.006883	1.065056	0.09093	1.065056	up	1.035032	0.049675	1.035032
1453657_at	0.044964	-1.02277	-0.03249	1.022773	down	-1.59423	-0.67286	1.594232
1453860_s_at	0.019869	1.173306	0.230579	1.173306	up	-1.14039	-0.18953	1.140389
1453927_at	0.008573	-1.05807	-0.08144	1.058072	down	1.137383	0.185718	1.137383
1453931_at	0.013423	-1.10594	-0.14527	1.105936	down	-1.17226	-0.22929	1.172256
1454033_at	0.027807	-1.06264	-0.08765	1.062639	down	1.184783	0.244622	1.184783
1454062_at	0.002881	-1.10587	-0.14519	1.105872	down	-1.21256	-0.27806	1.212562
1454166_at	0.002226	1.074914	0.104221	1.074914	up	1.026175	0.037277	1.026175
1454171_x_at	0.041478	-1.03787	-0.05362	1.037868	down	-1.16947	-0.22585	1.169467
1454224_at	0.017349	-1.0151	-0.02162	1.015101	down	-1.16463	-0.21987	1.164625
1454235_a_at	0.030168	-1.00169	-0.00243	1.001688	down	-1.06057	-0.08483	1.060566
1454250_at	0.038294	-1.16603	-0.22161	1.16603	down	1.039295	0.055606	1.039295
1454266_at	0.037481	-1.18691	-0.24722	1.186915	down	-1.15372	-0.20629	1.153715
1454297_at	0.00368	-1.08636	-0.1195	1.086356	down	-1.02412	-0.03438	1.024116
1454320_at	0.008774	1.079038	0.109746	1.079038	up	1.03631	0.051456	1.03631
1454494_at	0.001947	-1.11449	-0.15638	1.114489	down	-1.04253	-0.06009	1.042528
1454540_at	0.030442	1.131215	0.177873	1.131215	up	1.169565	0.225972	1.169565
1454637_at	0.032358	1.067431	0.094143	1.067431	up	1.056299	0.079018	1.056299
1454655_at	0.044929	1.18006	0.23886	1.18006	up	1.003292	0.004741	1.003292
1454658_at	0.006344	1.083279	0.115405	1.083279	up	1.193676	0.255411	1.193676
1454740_at	0.038134	1.017861	0.02554	1.017861	up	-1.07113	-0.09913	1.071129
1454777_at	0.023615	1.151974	0.204108	1.151974	up	1.078207	0.108635	1.078207
1454830_at	0.043863	1.116176	0.158565	1.116176	up	1.272955	0.348182	1.272955
1454834_at	0.026426	1.054595	0.076689	1.054595	up	-1.12918	-0.17528	1.129183
1454852_at	0.01073	1.151959	0.20409	1.151959	up	1.067221	0.093859	1.067221
1454902_at	0.004367	-1.04191	-0.05922	1.041906	down	1.100497	0.138155	1.100497
1454934_at	0.042803	1.177591	0.235839	1.177591	up	1.079254	0.110035	1.079254
1455028_at	0.02787	1.183026	0.242482	1.183026	up	-1.04709	-0.06638	1.047086
1455096_at	0.038821	-1.112	-0.15316	1.112001	down	-1.16995	-0.22645	1.16995
1455103_at	0.044474	1.21475	0.280659	1.21475	up	1.148851	0.200191	1.148851
1455107_at	0.022371	-1.01129	-0.0162	1.011293	down	1.026518	0.037759	1.026518
1455162_at	0.018343	-1.0405	-0.05728	1.040505	down	1.010888	0.015624	1.010888
1455224_at	0.049489	1.010264	0.014733	1.010264	up	-1.59619	-0.67464	1.596194
1455226_at	0.011396	1.030345	0.043128	1.030345	up	-1.1628	-0.2176	1.162796
1455228_at	0.029541	1.006048	0.0087	1.006048	up	1.036142	0.051222	1.036142

1455248_at	0.047771	1.01294	0.018549	1.01294	up	-1.01517	-0.02173	1.015174
1455250_at	0.022835	1.112332	0.153588	1.112332	up	1.183096	0.242568	1.183096
1455261_at	0.010793	1.0813	0.112767	1.0813	up	1.130938	0.17752	1.130938
1455277_at	0.037494	1.138621	0.187288	1.138621	up	1.082497	0.114363	1.082497
1455293_at	1.12E-05	-1.07594	-0.10559	1.075937	down	1.058491	0.082009	1.058491
1455306_at	0.001412	1.097868	0.134705	1.097868	up	1.097056	0.133638	1.097056
1455311_at	0.006507	1.052355	0.073621	1.052355	up	1.173776	0.231157	1.173776
1455358_at	0.011012	1.082234	0.114013	1.082234	up	-1.39054	-0.47565	1.390544
1455361_at	0.032743	-1.03152	-0.04477	1.031517	down	-1.05594	-0.07852	1.055937
1455378_at	0.047705	1.016001	0.022902	1.016001	up	1.017954	0.025673	1.017954
1455391_at	0.024053	-1.02455	-0.03499	1.024549	down	1.054229	0.076188	1.054229
1455411_at	0.010008	-1.06287	-0.08797	1.062872	down	1.076981	0.106993	1.076981
1455412_at	0.02154	-1.11815	-0.16112	1.118154	down	1.011459	0.016437	1.011459
1455542_at	0.016202	1.136379	0.184444	1.136379	up	-1.15034	-0.20207	1.150344
1455565_at	0.033689	1.159749	0.213813	1.159749	up	1.156787	0.210124	1.156787
1455586_at	0.021951	-1.10916	-0.14947	1.109163	down	-1.0857	-0.11863	1.085705
1455614_at	0.01071	-1.04649	-0.06556	1.046494	down	-1.03692	-0.05231	1.036922
1455658_at	0.001981	-1.08015	-0.11123	1.080149	down	-1.05005	-0.07046	1.050049
1455659_at	0.045023	-1.0018	-0.00259	1.001799	down	-1.08527	-0.11805	1.085268
1455680_at	0.015398	1.167464	0.223379	1.167464	up	1.06058	0.084853	1.06058
1455681_at	0.012814	1.133416	0.180677	1.133416	up	1.068216	0.095203	1.068216
1455704_at	0.008923	1.150819	0.202661	1.150819	up	1.049266	0.06938	1.049266
1455755_at	0.029291	-1.06817	-0.09514	1.068171	down	1.071231	0.09927	1.071231
1455756_at	0.048906	1.059498	0.083381	1.059498	up	-1.01903	-0.02719	1.019026
1455778_at	0.047229	1.140819	0.19007	1.140819	up	1.081286	0.112748	1.081286
1455833_at	0.041207	-1.04644	-0.06549	1.046442	down	1.085648	0.118556	1.085648
1455884_at	0.006257	-1.03435	-0.04872	1.03435	down	1.049757	0.070056	1.049757
1455903_at	0.026416	1.011321	0.016241	1.011321	up	-1.18826	-0.24885	1.188257
1455998_at	0.042247	-1.042	-0.05935	1.041996	down	-1.17501	-0.23267	1.175007
1456018_at	0.038525	1.073072	0.101746	1.073072	up	1.143817	0.193857	1.143817
1456060_at	0.01424	-1.28417	-0.36084	1.284175	down	-1.07718	-0.10725	1.077176
1456121_at	0.043714	1.297714	0.375972	1.297714	up	1.283853	0.36048	1.283853
1456143_at	0.049301	-1.00802	-0.01152	1.00802	down	1.028134	0.040028	1.028134
1456144_at	0.006324	-1.0067	-0.00964	1.006704	down	-1.10645	-0.14594	1.106452
1456156_at	0.003299	-1.20055	-0.26369	1.200548	down	-1.36453	-0.4484	1.36453
1456210_at	6.31E-04	-1.00199	-0.00286	1.001987	down	-1.02398	-0.03419	1.023983
1456217_at	0.022802	1.135449	0.183263	1.135449	up	1.018425	0.02634	1.018425
1456308_x_at	0.002259	1.086083	0.119135	1.086083	up	1.021811	0.031128	1.021811
1456320_at	0.023262	-1.12671	-0.17212	1.12671	down	1.104545	0.143452	1.104545
1456356_at	0.04309	-1.05651	-0.0793	1.056507	down	1.090277	0.124695	1.090277
1456389_at	0.041025	1.118842	0.162006	1.118842	up	1.194016	0.255822	1.194016
1456450_at	0.020064	-1.08076	-0.11205	1.080764	down	1.079958	0.110975	1.079958
1456468_x_at	0.032855	1.11671	0.159254	1.11671	up	-1.4389	-0.52496	1.438896
1456487_at	0.041523	1.545977	0.628519	1.545977	up	1.483336	0.568846	1.483336
1456499_at	0.033873	1.080685	0.111946	1.080685	up	-1.02345	-0.03345	1.023454
1456518_at	0.027061	1.124842	0.169722	1.124842	up	1.132135	0.179046	1.132135
1456602_at	0.040301	-1.03686	-0.05222	1.036857	down	-1.05715	-0.08018	1.057153

1456633_at	0.04575	-1.12403	-0.16868	1.124032	down	1.062151	0.086988	1.062151
1456639_at	0.024414	1.126918	0.172383	1.126918	up	-1.02731	-0.03887	1.027309
1456653_a_at	2.30E-04	1.130129	0.176487	1.130129	up	1.134676	0.182281	1.134676
1456660_a_at	0.014712	1.022662	0.032329	1.022662	up	-1.00101	-0.00146	1.001012
1456685_at	0.001906	-1.0679	-0.09477	1.067895	down	1.04503	0.063545	1.04503
1456763_at	0.010958	1.100383	0.138006	1.100383	up	1.032477	0.04611	1.032477
1456769_at	0.036837	-1.01659	-0.02374	1.016594	down	1.03751	0.053125	1.03751
1456772_at	0.047231	-1.22534	-0.29319	1.225343	down	-1.23146	-0.30037	1.231459
1456775_at	0.031831	1.07832	0.108785	1.07832	up	1.044733	0.063134	1.044733
1456812_at	0.024932	1.056456	0.079233	1.056456	up	1.056502	0.079295	1.056502
1456874_at	0.010041	1.029892	0.042494	1.029892	up	1.086958	0.120296	1.086958
1456892_at	0.02073	-1.01451	-0.02078	1.014507	down	-1.04322	-0.06105	1.043224
1456900_at	0.036871	1.210852	0.276023	1.210852	up	1.121	0.164786	1.121
1456926_at	0.035845	-1.01661	-0.02377	1.016614	down	-1.02754	-0.03919	1.027537
1456937_at	1.33E-04	-1.11032	-0.15098	1.110324	down	-1.02131	-0.03043	1.021314
1456976_at	0.002746	1.129066	0.17513	1.129066	up	1.061103	0.085565	1.061103
1457009_at	0.048445	-1.18606	-0.24617	1.186055	down	-1.01548	-0.02215	1.015475
1457029_at	0.027005	1.175224	0.232936	1.175224	up	-1.09026	-0.12467	1.090262
1457124_at	0.023108	-1.25168	-0.32386	1.251677	down	1.053388	0.075036	1.053388
1457164_at	0.033476	-1.18596	-0.24605	1.185959	down	-1.01193	-0.01711	1.01193
1457196_at	0.043923	-1.18344	-0.24299	1.18344	down	-1.17394	-0.23136	1.173938
1457295_at	0.033204	-1.30312	-0.38197	1.303117	down	-1.16031	-0.21451	1.160312
1457321_at	0.014947	-1.0322	-0.04572	1.032201	down	-1.14912	-0.20053	1.149117
1457329_at	0.01591	-1.25885	-0.33211	1.258854	down	-1.11273	-0.1541	1.112727
1457348_at	0.008877	-1.03423	-0.04855	1.034228	down	-1.13065	-0.17716	1.130654
1457350_at	0.013391	-1.14857	-0.19984	1.148569	down	-1.10926	-0.1496	1.109259
1457373_at	0.01979	-1.07914	-0.10988	1.079138	down	1.341862	0.424236	1.341862
1457397_at	0.046574	-1.05792	-0.08124	1.057925	down	-1.03121	-0.04434	1.031208
1457404_at	0.032463	-1.05511	-0.0774	1.055112	down	-1.11524	-0.15736	1.115242
1457413_at	0.021021	-1.01743	-0.02493	1.017432	down	-1.14954	-0.20106	1.14954
1457511_at	0.045125	-1.20338	-0.26709	1.203379	down	-1.19881	-0.2616	1.198808
1457555_at	0.025613	1.038024	0.05384	1.038024	up	1.201665	0.265034	1.201665
1457600_x_at	0.007963	1.031798	0.04516	1.031798	up	-1.01556	-0.02227	1.015558
1457625_s_at	0.005807	-1.18308	-0.24255	1.183079	down	-1.11302	-0.15448	1.113023
1457643_x_at	0.008501	1.000637	9.19E-04	1.000637	up	-1.25335	-0.32579	1.253353
1457654_at	0.007406	-1.24396	-0.31494	1.243962	down	-1.08822	-0.12197	1.088216
1457671_at	0.004172	-1.01868	-0.0267	1.018678	down	-1.20326	-0.26694	1.203255
1457721_at	0.033125	-1.01041	-0.01495	1.010414	down	1.168916	0.225172	1.168916
1457726_at	0.049319	1.140958	0.190245	1.140958	up	1.103341	0.141879	1.103341
1457740_at	0.009452	1.061019	0.08545	1.061019	up	1.165049	0.22039	1.165049
1457741_at	0.044823	1.004985	0.007174	1.004985	up	-1.07848	-0.109	1.078483
1457836_at	0.016524	1.043392	0.061281	1.043392	up	1.170187	0.226738	1.170187
1457882_at	0.016558	1.049985	0.070369	1.049985	up	1.001108	0.001598	1.001108
1457900_at	0.009552	-1.05442	-0.07645	1.054419	down	1.182044	0.241284	1.182044
1457915_at	0.044068	-1.02345	-0.03344	1.023448	down	1.130888	0.177455	1.130888
1457927_at	1.07E-04	1.090857	0.125462	1.090857	up	1.257189	0.330201	1.257189
1457956_at	0.013362	-1.09253	-0.12768	1.092534	down	-1.04394	-0.06204	1.043942

1457969_at	0.011808	1.024954	0.035559	1.024954	up	1.248874	0.320628	1.248874
1458040_at	0.00381	-1.00447	-0.00644	1.004475	down	-1.06982	-0.09736	1.069816
1458066_at	0.016817	-1.13671	-0.18486	1.136706	down	1.047123	0.066431	1.047123
1458141_at	0.04029	1.078195	0.108618	1.078195	up	-1.02153	-0.03074	1.021534
1458165_at	0.035558	-1.07424	-0.10331	1.074235	down	-1.13393	-0.18133	1.133929
1458170_at	0.022674	1.045013	0.063521	1.045013	up	1.091482	0.126289	1.091482
1458172_at	0.02203	1.010265	0.014733	1.010265	up	-1.01169	-0.01676	1.011688
1458226_at	0.030033	-1.18291	-0.24234	1.182908	down	-1.05526	-0.0776	1.055265
1458264_at	0.01694	1.04733	0.066716	1.04733	up	1.03135	0.044533	1.03135
1458280_at	0.024298	1.064781	0.090557	1.064781	up	1.088798	0.122737	1.088798
1458332_x_at	0.020211	-1.01815	-0.02595	1.018152	down	-1.04052	-0.05731	1.040522
1458343_x_at	0.002387	1.06751	0.094249	1.06751	up	1.113295	0.154835	1.113295
1458371_at	0.026149	1.024997	0.03562	1.024997	up	1.098936	0.136108	1.098936
1458381_at	0.010807	1.029571	0.042043	1.029571	up	-1.09297	-0.12825	1.092967
1458398_at	0.028007	-1.09222	-0.12726	1.092216	down	-1.12301	-0.16738	1.123014
1458421_at	0.009179	-1.21409	-0.27988	1.214091	down	-1.06164	-0.08629	1.061638
1458449_at	0.025504	-1.0836	-0.11583	1.0836	down	-1.20704	-0.27147	1.207036
1458455_at	0.031733	-1.32349	-0.40435	1.323491	down	-2.41699	-1.27321	2.416991
1458492_x_at	0.034243	1.693136	0.759698	1.693136	up	1.26221	0.335952	1.26221
1458499_at	0.020731	-1.0519	-0.073	1.051898	down	1.00477	0.006866	1.00477
1458521_at	0.039479	-1.11231	-0.15356	1.112313	down	-1.0535	-0.07519	1.053502
1458529_at	0.013226	-1.04854	-0.06838	1.048538	down	-1.07509	-0.10446	1.075092
1458601_at	0.02638	1.199123	0.261979	1.199123	up	-1.10425	-0.14307	1.10425
1458809_at	0.022919	1.156024	0.209171	1.156024	up	1.004815	0.00693	1.004815
1458843_at	0.030258	1.121834	0.165859	1.121834	up	1.074387	0.103514	1.074387
1459097_at	0.028537	1.072329	0.100748	1.072329	up	1.005182	0.007456	1.005182
1459271_at	0.049951	1.039657	0.056108	1.039657	up	-1.0036	-0.00518	1.003597
1459512_at	0.032824	1.035393	0.050178	1.035393	up	-1.1471	-0.198	1.147105
1459590_at	0.013473	-1.10225	-0.14045	1.102247	down	-1.01124	-0.01613	1.011245
1459679_s_at	0.038073	1.216609	0.282866	1.216609	up	1.021481	0.030662	1.021481
1459710_at	0.017685	1.094145	0.129804	1.094145	up	-1.10185	-0.13993	1.101852
1459746_at	0.032476	1.04583	0.064648	1.04583	up	-1.00891	-0.0128	1.008914
1459788_at	0.01723	-1.29754	-0.37578	1.297544	down	-1.25045	-0.32245	1.250448
1459832_s_at	0.033603	1.088846	0.1228	1.088846	up	1.178433	0.236869	1.178433
1459984_at	0.049016	-1.12048	-0.16412	1.120481	down	-1.28706	-0.36408	1.287059
1460035_at	0.041615	1.100563	0.138242	1.100563	up	1.089635	0.123845	1.089635
1460073_at	0.011693	1.139272	0.188112	1.139272	up	1.149112	0.200519	1.149112
1460077_at	0.026163	-1.02011	-0.02872	1.020109	down	-1.3404	-0.42266	1.340398
1460081_at	0.024477	1.504925	0.589692	1.504925	up	1.224081	0.291699	1.224081
1460106_at	0.046988	-1.03258	-0.04625	1.032576	down	-1.12727	-0.17284	1.127273
1460446_at	0.006803	1.126452	0.171786	1.126452	up	1.159167	0.213088	1.159167
1460527_at	0.009652	-1.08837	-0.12216	1.088366	down	-1.09891	-0.13608	1.098915
1460559_at	0.023308	1.1004	0.138028	1.1004	up	-1.0051	-0.00734	1.0051
1460563_at	0.030799	-1.05227	-0.0735	1.05227	down	1.115246	0.157361	1.115246
1460567_at	0.019325	1.17269	0.229822	1.17269	up	-1.01492	-0.02137	1.014923
1460584_at	0.004137	-1.00567	-0.00815	1.005668	down	-1.10026	-0.13785	1.100264
1460591_at	0.016119	-1.07082	-0.09872	1.070822	down	-1.27421	-0.34961	1.274213

1460606_at	0.006237	-1.05626	-0.07897	1.056263	down	1.011075	0.01589	1.011075

Regulation	FC ([D30-D	Log FC ([D3	FC (abs) ([D	Regulation	FC ([D30-T	Log FC ([D3	FC (abs) ([D	Regulation	[D07-Con](
up	-1.08245	-0.1143	1.082453	down	1.035726	0.050642	1.035726	up	870.6018
up	1.089073	0.1231	1.089073	up	1.130751	0.177281	1.130751	up	2817.307
up	1.046791	0.065974	1.046791	up	-1.07155	-0.0997	1.071554	down	344.7831
up	-1.12601	-0.17122	1.126014	down	-1.09132	-0.12607	1.091316	down	211.5262
up	1.041449	0.058592	1.041449	up	1.09758	0.134327	1.09758	up	1058.851
down	-1.11653	-0.15903	1.116533	down	1.001277	0.001841	1.001277	up	729.6677
up	-1.03578	-0.05072	1.035783	down	-1.00186	-0.00268	1.00186	down	5682.99
up	-1.02446	-0.03487	1.024462	down	1.078205	0.108632	1.078205	up	24.3854
up	-1.06048	-0.08472	1.060482	down	-1.02011	-0.02872	1.020108	down	1666.284
up	-1.03367	-0.04778	1.033674	down	1.058069	0.081433	1.058069	up	889.3624
up	1.383385	0.468202	1.383385	up	1.207003	0.271429	1.207003	up	158.6388
up	1.170382	0.226979	1.170382	up	1.115309	0.157443	1.115309	up	2971.213
up	-1.02488	-0.03545	1.024878	down	-1.13451	-0.18207	1.134511	down	2087.52
up	1.060965	0.085378	1.060965	up	1.078245	0.108685	1.078245	up	956.0788
up	-1.10353	-0.14213	1.10353	down	1.043909	0.061995	1.043909	up	1814.289
up	1.169936	0.226429	1.169936	up	1.06192	0.086675	1.06192	up	682.5323
down	-1.20782	-0.27241	1.207823	down	1.039543	0.05595	1.039543	up	30.514
down	1.031655	0.044961	1.031655	up	1.034367	0.048749	1.034367	up	211.408
down	-1.08927	-0.12336	1.08927	down	-1.12833	-0.17419	1.128331	down	4583.945
up	1.158179	0.211858	1.158179	up	1.046933	0.066169	1.046933	up	77.59863
up	1.26351	0.337437	1.26351	up	1.038673	0.054741	1.038673	up	266.1272
down	-1.02819	-0.04011	1.028189	down	-1.03198	-0.04542	1.031984	down	1358.737
up	-1.11777	-0.16062	1.117767	down	-1.12322	-0.16764	1.123219	down	1573.589
down	-1.13134	-0.17803	1.131341	down	-1.41157	-0.4973	1.411567	down	345.2832
up	-1.02566	-0.03655	1.02566	down	1.026346	0.037517	1.026346	up	2649.963
down	1.058238	0.081665	1.058238	up	-1.07596	-0.10563	1.075962	down	128.1126
up	1.054845	0.077031	1.054845	up	1.09373	0.129256	1.09373	up	455.4964
down	1.552494	0.634588	1.552494	up	1.163009	0.217862	1.163009	up	308.1458
down	1.097087	0.133678	1.097087	up	-1.18111	-0.24015	1.181112	down	260.3373
up	-1.8998	-0.92585	1.899804	down	-1.62	-0.69599	1.619999	down	370.2874
up	1.309442	0.388952	1.309442	up	1.085059	0.117774	1.085059	up	604.5383
up	-1.35521	-0.43852	1.35521	down	-1.21572	-0.28181	1.215717	down	181.5671
down	1.248022	0.319643	1.248022	up	1.275042	0.350545	1.275042	up	87.09032

down	-1.1192	-0.16247	1.119204	down	-1.00619	-0.0089	1.006191	down	1259.959
up	-1.03926	-0.05555	1.039258	down	-1.01902	-0.02718	1.019016	down	341.3287
up	1.013449	0.019274	1.013449	up	1.055638	0.078115	1.055638	up	513.9077
up	1.129282	0.175405	1.129282	up	1.043295	0.061147	1.043295	up	636.6631
up	1.079887	0.11088	1.079887	up	-1.02665	-0.03795	1.026651	down	1939.502
up	1.171312	0.228126	1.171312	up	1.15642	0.209665	1.15642	up	891.8311
up	1.100994	0.138807	1.100994	up	-1.01651	-0.02362	1.016505	down	825.3887
up	1.045202	0.063781	1.045202	up	-1.0948	-0.13066	1.094796	down	4928.682
down	1.486056	0.571488	1.486056	up	1.271648	0.3467	1.271648	up	192.5206
down	1.010776	0.015463	1.010776	up	1.128832	0.174831	1.128832	up	183.6964
up	-1.16252	-0.21726	1.162525	down	-1.22526	-0.29308	1.225256	down	1727.999
up	1.041789	0.059063	1.041789	up	1.109926	0.150463	1.109926	up	2800.122
down	-1.08978	-0.12403	1.089778	down	-1.12159	-0.16554	1.121589	down	1016.077
up	-1.10674	-0.14631	1.106738	down	-1.05413	-0.07606	1.054135	down	74.03568
down	1.154096	0.206763	1.154096	up	1.188799	0.249505	1.188799	up	1327.354
up	-1.09168	-0.12655	1.091683	down	-1.16415	-0.21927	1.164146	down	382.6178
up	-1.00019	-2.76E-04	1.000191	down	1.040397	0.057134	1.040397	up	258.0052
up	1.041394	0.058516	1.041394	up	1.155363	0.208346	1.155363	up	177.7415
up	1.00792	0.011381	1.00792	up	1.013002	0.018637	1.013002	up	163.1945
up	1.095935	0.132162	1.095935	up	-1.105	-0.14405	1.105001	down	163.3462
up	1.280619	0.356841	1.280619	up	1.093114	0.128444	1.093114	up	237.7298
up	-1.07573	-0.10531	1.075728	down	-1.02247	-0.03206	1.02247	down	141.4768
up	1.052273	0.07351	1.052273	up	1.216841	0.283141	1.216841	up	39.05167
down	1.380555	0.465248	1.380555	up	1.169354	0.225712	1.169354	up	714.7083
down	-1.02935	-0.04173	1.029347	down	-1.0588	-0.08243	1.058803	down	189.1891
up	-1.02516	-0.03585	1.025162	down	1.062162	0.087003	1.062162	up	528.2935
up	-1.00431	-0.0062	1.004309	down	-1.0417	-0.05894	1.041703	down	50.36854
up	1.110902	0.151731	1.110902	up	-1.02275	-0.03245	1.02275	down	551.0407
up	-1.03133	-0.0445	1.031325	down	-1.0661	-0.09235	1.066103	down	155.4379
down	1.121599	0.165556	1.121599	up	1.005836	0.008396	1.005836	up	1297.714
up	-1.40066	-0.48611	1.400661	down	-1.31043	-0.39004	1.310431	down	667.6527
down	1.130001	0.176324	1.130001	up	1.160856	0.215189	1.160856	up	2973.037
up	1.131833	0.17866	1.131833	up	-1.06339	-0.08867	1.063393	down	103.7795
up	1.292349	0.369995	1.292349	up	-1.09656	-0.13299	1.096563	down	645.6057
down	1.095434	0.131502	1.095434	up	1.101874	0.139959	1.101874	up	1565.727
down	1.105439	0.144619	1.105439	up	-1.10623	-0.14565	1.10623	down	807.9775
up	1.084462	0.11698	1.084462	up	1.033737	0.04787	1.033737	up	7729.965
down	-1.01218	-0.01746	1.012178	down	1.077487	0.107671	1.077487	up	385.9098
up	-1.08753	-0.12106	1.087531	down	-1.06438	-0.09002	1.064385	down	1289.281
down	-1.07853	-0.10907	1.078534	down	-1.0009	-0.0013	1.000899	down	582.1258
up	-1.19651	-0.25883	1.196506	down	1.061771	0.086473	1.061771	up	5263.114
up	-1.1049	-0.14392	1.1049	down	-1.03542	-0.05022	1.035423	down	58.15938
down	1.198549	0.261289	1.198549	up	1.109014	0.149278	1.109014	up	230.3597
up	1.127442	0.173053	1.127442	up	1.190216	0.251224	1.190216	up	433.0242
down	-1.00232	-0.00335	1.002322	down	1.006213	0.008936	1.006213	up	4433.017
down	1.140182	0.189264	1.140182	up	1.039053	0.05527	1.039053	up	24.61515
up	-1.14509	-0.19547	1.145094	down	-1.1006	-0.13829	1.100601	down	39.87194

up	1.006737	0.009687	1.006737	up	1.073333	0.102098	1.073333	up	222.9345
up	1.031438	0.044657	1.031438	up	1.1137	0.15536	1.1137	up	297.6074
up	-1.04335	-0.06122	1.043348	down	1.049167	0.069244	1.049167	up	505.9554
up	1.528305	0.611932	1.528305	up	1.235481	0.305073	1.235481	up	529.5925
down	1.456254	0.542262	1.456254	up	1.481026	0.566597	1.481026	up	395.2318
down	-1.02121	-0.03028	1.021207	down	1.125088	0.170038	1.125088	up	492.0723
up	1.382576	0.467359	1.382576	up	1.221792	0.288998	1.221792	up	358.0007
down	-1.28162	-0.35797	1.281617	down	-1.32257	-0.40334	1.322567	down	64.69349
down	-1.01232	-0.01767	1.012324	down	-1.12967	-0.1759	1.12967	down	147.8832
up	1.09366	0.129164	1.09366	up	1.083493	0.11569	1.083493	up	183.0182
up	1.000904	0.001303	1.000904	up	1.03226	0.045807	1.03226	up	2643.454
up	-1.09989	-0.13737	1.099895	down	-1.02995	-0.04257	1.029947	down	392.9278
up	1.003311	0.004769	1.003311	up	1.055992	0.078599	1.055992	up	288.8524
down	-1.06482	-0.09061	1.064821	down	1.014026	0.020095	1.014026	up	1972.149
up	1.033048	0.046907	1.033048	up	1.088443	0.122266	1.088443	up	622.7853
down	1.041586	0.058782	1.041586	up	-1.04048	-0.05726	1.040485	down	34.18471
up	1.150194	0.201877	1.150194	up	1.193795	0.255555	1.193795	up	98.17517
up	1.067834	0.094687	1.067834	up	1.028075	0.039945	1.028075	up	566.7329
up	1.2407	0.311155	1.2407	up	1.138355	0.18695	1.138355	up	256.2139
up	-1.02227	-0.03177	1.022265	down	1.026269	0.037408	1.026269	up	387.8341
down	1.122091	0.16619	1.122091	up	1.161285	0.215722	1.161285	up	184.6145
up	-1.01585	-0.02268	1.015847	down	1.053466	0.075143	1.053466	up	51.44728
up	-1.07985	-0.11083	1.079847	down	-1.09504	-0.13098	1.095039	down	1208.438
up	-1.01229	-0.01762	1.01229	down	-1.09014	-0.12452	1.090143	down	467.1503
up	1.361638	0.445344	1.361638	up	1.259421	0.332761	1.259421	up	1374.053
up	1.200822	0.264022	1.200822	up	1.223319	0.290801	1.223319	up	64.03462
up	1.167015	0.222823	1.167015	up	1.080177	0.111268	1.080177	up	630.616
up	-1.06941	-0.09682	1.069413	down	-1.00172	-0.00247	1.001717	down	126.0451
down	1.421	0.506907	1.421	up	1.320396	0.400971	1.320396	up	2087.855
up	-1.16867	-0.22487	1.16867	down	-1.19618	-0.25844	1.196183	down	93.45768
down	1.135896	0.18383	1.135896	up	-1.01378	-0.01974	1.013778	down	5544.617
down	-1.01594	-0.02282	1.015942	down	-1.16414	-0.21927	1.164142	down	10659.11
down	1.129283	0.175407	1.129283	up	1.110104	0.150695	1.110104	up	698.6282
up	-1.18464	-0.24445	1.184643	down	-1.28893	-0.36617	1.288927	down	24.00804
up	-1.05786	-0.08115	1.057861	down	-1.0493	-0.06943	1.049302	down	26.22423
up	-1.24512	-0.31629	1.24512	down	-1.05917	-0.08293	1.059167	down	140.5656
down	-1.07116	-0.09918	1.071162	down	-1.11426	-0.15609	1.114262	down	318.5464
up	-1.04103	-0.05801	1.041027	down	1.036608	0.051871	1.036608	up	318.3474
up	-1.02835	-0.04033	1.028349	down	1.09145	0.126246	1.09145	up	213.5799
down	-1.02408	-0.03433	1.024082	down	-1.02829	-0.04024	1.028286	down	140.6638
down	-1.00853	-0.01226	1.008534	down	-1.05714	-0.08017	1.057144	down	859.729
up	1.232074	0.301089	1.232074	up	1.169696	0.226133	1.169696	up	104.9949
up	-1.30755	-0.38687	1.30755	down	-1.00105	-0.00151	1.001045	down	181.822
up	1.204316	0.268214	1.204316	up	1.265713	0.33995	1.265713	up	94.26022
down	1.078222	0.108654	1.078222	up	1.008641	0.012413	1.008641	up	1556.85
up	1.229998	0.298656	1.229998	up	1.255022	0.327712	1.255022	up	568.2439
up	1.196745	0.259115	1.196745	up	1.091325	0.126081	1.091325	up	1660.444

up	1.152033	0.204182	1.152033	up	1.180661	0.239594	1.180661	up	1638.777
down	1.092074	0.127071	1.092074	up	-1.20632	-0.27061	1.206318	down	4233.515
up	1.021988	0.031379	1.021988	up	-1.04569	-0.06445	1.045688	down	330.9774
up	-1.01777	-0.02542	1.017773	down	-1.01506	-0.02157	1.015061	down	236.1729
up	-1.0166	-0.02375	1.016597	down	1.119434	0.162769	1.119434	up	61.53463
up	1.024268	0.034594	1.024268	up	1.033895	0.04809	1.033895	up	481.9718
up	1.115539	0.157742	1.115539	up	1.035267	0.050002	1.035267	up	254.315
up	1.076735	0.106663	1.076735	up	-1.02293	-0.0327	1.022927	down	859.8234
up	1.147504	0.198499	1.147504	up	-1.01142	-0.01639	1.011422	down	284.897
down	1.185408	0.245383	1.185408	up	-1.18595	-0.24604	1.185948	down	317.2827
up	1.149016	0.200398	1.149016	up	1.214609	0.280492	1.214609	up	384.3555
up	-1.25957	-0.33293	1.259565	down	-1.11807	-0.16101	1.118069	down	442.433
up	-1.01279	-0.01834	1.012793	down	1.093025	0.128326	1.093025	up	1541.943
up	-1.03955	-0.05595	1.039546	down	1.037684	0.053367	1.037684	up	297.7503
up	1.371034	0.455264	1.371034	up	1.212681	0.278201	1.212681	up	189.6584
down	1.147413	0.198385	1.147413	up	1.003125	0.004501	1.003125	up	30.17147
down	-1.19839	-0.2611	1.198394	down	-1.11988	-0.16335	1.119884	down	77.50143
up	-1.01073	-0.0154	1.010733	down	1.011505	0.016504	1.011505	up	1960.509
up	-1.13262	-0.17966	1.13262	down	-1.08528	-0.11807	1.085283	down	324.4019
down	1.174625	0.2322	1.174625	up	1.074576	0.103768	1.074576	up	75.81403
down	1.210576	0.275693	1.210576	up	-1.00922	-0.01325	1.009225	down	1348.298
up	1.067948	0.094841	1.067948	up	1.104359	0.14321	1.104359	up	105.4124
up	-1.01725	-0.02467	1.01725	down	1.104926	0.14395	1.104926	up	1543.642
up	1.027464	0.039088	1.027464	up	1.186373	0.246558	1.186373	up	1209.848
down	-1.05287	-0.07433	1.052874	down	-1.07402	-0.10302	1.074016	down	47.41066
up	1.198196	0.260864	1.198196	up	-1.09802	-0.1349	1.098016	down	131.608
up	1.062319	0.087217	1.062319	up	1.008633	0.012402	1.008633	up	3392.859
up	1.505724	0.590457	1.505724	up	1.451922	0.537964	1.451922	up	242.6276
up	1.046491	0.06556	1.046491	up	1.00781	0.011223	1.00781	up	1781.989
up	1.198751	0.261532	1.198751	up	1.016602	0.023755	1.016602	up	43.49676
down	-1.05984	-0.08385	1.059844	down	-1.12537	-0.1704	1.125374	down	133.3788
down	-1.12185	-0.16587	1.121846	down	1.023779	0.033904	1.023779	up	1757.898
up	-1.74435	-0.80269	1.744346	down	-1.30667	-0.3859	1.306675	down	42.53797
up	1.461978	0.547921	1.461978	up	1.241265	0.311811	1.241265	up	414.5515
up	1.09825	0.135206	1.09825	up	1.141404	0.190809	1.141404	up	971.0216
up	1.10993	0.150469	1.10993	up	1.105685	0.14494	1.105685	up	256.1229
up	1.005284	0.007603	1.005284	up	-1.17393	-0.23135	1.173934	down	344.3434
up	1.063196	0.088408	1.063196	up	1.040119	0.056748	1.040119	up	184.5243
down	1.293513	0.371295	1.293513	up	1.109679	0.150142	1.109679	up	138.1755
down	1.290974	0.36846	1.290974	up	1.19134	0.252586	1.19134	up	252.195
up	1.050731	0.071394	1.050731	up	1.079959	0.110977	1.079959	up	697.7934
down	-1.31526	-0.39535	1.315259	down	-1.506	-0.59072	1.506003	down	217.4623
up	-1.10564	-0.14488	1.105641	down	-1.11018	-0.15079	1.110176	down	652.2568
up	1.322094	0.402825	1.322094	up	1.148829	0.200164	1.148829	up	1099.522
up	1.066653	0.093091	1.066653	up	1.046336	0.065346	1.046336	up	76.92342
down	1.092971	0.128255	1.092971	up	-1.18423	-0.24396	1.184235	down	1976.813
up	1.056275	0.078985	1.056275	up	1.06759	0.094358	1.06759	up	115.3989

up	-1.1896	-0.25047	1.189595	down	-1.17388	-0.23128	1.173878	down	86.79374
down	1.086297	0.119418	1.086297	up	1.304742	0.383764	1.304742	up	130.6969
up	1.032775	0.046526	1.032775	up	-1.0615	-0.0861	1.0615	down	2878.758
up	-1.15478	-0.20761	1.154776	down	-1.35553	-0.43885	1.355525	down	427.9426
up	-1.07178	-0.10001	1.071783	down	-1.05514	-0.07744	1.055143	down	1272.601
up	-1.02024	-0.02891	1.020239	down	-1.09858	-0.13564	1.098578	down	2030.036
down	-1.05101	-0.07177	1.051007	down	1.003116	0.004488	1.003116	up	156.9716
down	-1.07537	-0.10483	1.075365	down	-1.06479	-0.09057	1.064788	down	789.3572
up	1.171283	0.228089	1.171283	up	1.090652	0.125191	1.090652	up	543.8123
down	-1.26823	-0.34281	1.268228	down	-1.23248	-0.30156	1.232478	down	1813.684
down	1.005803	0.008348	1.005803	up	1.003699	0.005327	1.003699	up	853.6102
down	-1.16791	-0.22393	1.167909	down	-1.01135	-0.01629	1.011353	down	59.9225
down	1.166103	0.221695	1.166103	up	1.046048	0.064949	1.046048	up	67.65712
up	1.170879	0.227592	1.170879	up	1.022284	0.031796	1.022284	up	55.75281
up	1.109425	0.149812	1.109425	up	1.187562	0.248002	1.187562	up	276.2896
up	-1.08491	-0.11757	1.084908	down	1.079434	0.110276	1.079434	up	539.1354
up	-1.09417	-0.12984	1.094173	down	-1.07261	-0.10113	1.072612	down	279.3846
down	1.209387	0.274276	1.209387	up	1.234577	0.304017	1.234577	up	129.3548
up	1.457384	0.543381	1.457384	up	1.373515	0.457873	1.373515	up	1988.568
down	1.299509	0.377967	1.299509	up	1.177937	0.236263	1.177937	up	230.5336
down	-1.12195	-0.16601	1.121954	down	-1.05992	-0.08396	1.059923	down	185.1911
down	1.555792	0.63765	1.555792	up	1.318213	0.398583	1.318213	up	168.6008
down	-1.09404	-0.12966	1.094036	down	1.004131	0.005947	1.004131	up	24.76485
up	1.150734	0.202555	1.150734	up	1.091777	0.126678	1.091777	up	2331.855
down	1.100583	0.138268	1.100583	up	1.069325	0.0967	1.069325	up	45.89514
up	-1.31007	-0.38964	1.310065	down	-1.35411	-0.43734	1.354108	down	208.8669
up	1.070251	0.097949	1.070251	up	1.053362	0.075001	1.053362	up	225.9776
up	1.048639	0.068518	1.048639	up	-1.08702	-0.12037	1.087015	down	163.4802
up	1.220743	0.287759	1.220743	up	-1.05424	-0.07621	1.054243	down	463.094
up	-1.01149	-0.01648	1.011491	down	-1.00559	-0.00804	1.005586	down	62.30345
down	-1.03708	-0.05253	1.03708	down	1.148464	0.199706	1.148464	up	218.3793
up	-1.14694	-0.19779	1.146938	down	-1.00542	-0.0078	1.005418	down	298.4917
down	-1.32343	-0.40428	1.323426	down	-1.21226	-0.2777	1.212261	down	152.1418
down	1.12802	0.173793	1.12802	up	1.112113	0.153304	1.112113	up	476.0079
down	-1.30274	-0.38154	1.302735	down	-1.58894	-0.66806	1.588938	down	72.54348
up	1.156608	0.2099	1.156608	up	-1.00089	-0.00128	1.000886	down	141.4972
up	1.166656	0.222379	1.166656	up	1.063097	0.088274	1.063097	up	384.953
up	1.075988	0.105662	1.075988	up	1.067776	0.09461	1.067776	up	466.8489
up	-1.03942	-0.05578	1.039421	down	-1.0939	-0.12949	1.093904	down	821.7075
down	1.168698	0.224902	1.168698	up	1.187711	0.248184	1.187711	up	1403.783
down	-1.02981	-0.04237	1.029807	down	1.016314	0.023347	1.016314	up	78.79592
down	-1.1267	-0.1721	1.126699	down	1.131806	0.178626	1.131806	up	25.91967
up	-1.04551	-0.06421	1.045511	down	1.034131	0.048418	1.034131	up	826.9331
up	1.000678	9.77E-04	1.000678	up	-1.01773	-0.02535	1.017729	down	1357.703
down	-1.22226	-0.28955	1.222259	down	-1.20546	-0.26958	1.205457	down	45.03019
up	-1.30251	-0.38129	1.30251	down	-1.28126	-0.35756	1.281258	down	188.6431
down	1.031422	0.044635	1.031422	up	-1.03476	-0.0493	1.034762	down	36.33455

up	-1.09004	-0.12438	1.090037	down	1.034161	0.04846	1.034161	up	93.57954
down	-1.03716	-0.05264	1.037162	down	-1.01957	-0.02796	1.019568	down	131.6435
up	1.375342	0.45979	1.375342	up	1.224509	0.292203	1.224509	up	343.8823
up	1.048431	0.068232	1.048431	up	1.023294	0.03322	1.023294	up	280.6717
up	1.327311	0.408506	1.327311	up	1.099006	0.1362	1.099006	up	457.7243
up	1.02286	0.032609	1.02286	up	1.013248	0.018987	1.013248	up	212.1901
up	-1.17171	-0.22862	1.171713	down	-1.05504	-0.0773	1.055039	down	147.0982
up	1.119922	0.163398	1.119922	up	-1.04273	-0.06037	1.042731	down	267.451
down	1.174216	0.231698	1.174216	up	1.080676	0.111934	1.080676	up	255.9161
up	-1.08443	-0.11693	1.084425	down	-1.00725	-0.01042	1.007249	down	186.0223
down	-1.16251	-0.21725	1.162514	down	-1.27359	-0.3489	1.273592	down	393.9243
down	1.008794	0.012632	1.008794	up	1.231601	0.300535	1.231601	up	87.37173
down	1.421938	0.507859	1.421938	up	1.35103	0.43406	1.35103	up	617.7143
down	1.355638	0.438972	1.355638	up	-1.13612	-0.18412	1.136123	down	455.1256
down	1.016395	0.023461	1.016395	up	-1.01388	-0.01988	1.013876	down	69.81247
up	1.024021	0.034245	1.024021	up	1.085087	0.117811	1.085087	up	37.7931
up	-1.08544	-0.11829	1.085445	down	-1.08647	-0.11965	1.086474	down	2606.102
up	-1.01013	-0.01454	1.010128	down	1.00601	0.008645	1.00601	up	23.70878
down	-1.04822	-0.06794	1.048216	down	1.016545	0.023675	1.016545	up	49.37021
down	1.264839	0.338953	1.264839	up	1.213912	0.279664	1.213912	up	125.0236
up	-1.08996	-0.12428	1.089965	down	1.031779	0.045134	1.031779	up	130.639
down	1.10662	0.14616	1.10662	up	1.057301	0.080386	1.057301	up	30.36975
down	-1.09432	-0.13003	1.094318	down	-1.0408	-0.0577	1.040802	down	165.4837
down	1.203375	0.267086	1.203375	up	-1.02722	-0.03875	1.027221	down	88.20757
down	1.338122	0.42021	1.338122	up	1.384332	0.46919	1.384332	up	295.6296
down	-1.02344	-0.03342	1.023436	down	-1.03477	-0.0493	1.034766	down	227.7
down	-1.04836	-0.06814	1.048364	down	-1.44366	-0.52973	1.443658	down	285.8078
up	1.973206	0.980542	1.973206	up	1.590612	0.669582	1.590612	up	53.6041
up	1.052169	0.073367	1.052169	up	1.068289	0.095302	1.068289	up	2614.28
down	1.114878	0.156886	1.114878	up	1.038485	0.054481	1.038485	up	24.86276
down	1.083003	0.115037	1.083003	up	1.094559	0.13035	1.094559	up	97.94035
up	1.114204	0.156013	1.114204	up	1.080236	0.111346	1.080236	up	2803.103
down	1.502718	0.587574	1.502718	up	1.146174	0.196826	1.146174	up	413.7824
up	1.082977	0.115003	1.082977	up	1.000761	0.001097	1.000761	up	120.7932
up	1.19157	0.252864	1.19157	up	1.09332	0.128715	1.09332	up	343.2531
down	1.102675	0.141008	1.102675	up	-1.14242	-0.19209	1.142416	down	186.9239
up	-1.19973	-0.26271	1.199732	down	-1.09412	-0.12977	1.094117	down	1047.237
down	-1.1568	-0.21014	1.156802	down	-1.00563	-0.0081	1.005631	down	20.94985
down	1.6072	0.684549	1.6072	up	1.567596	0.648554	1.567596	up	763.7213
down	1.004123	0.005937	1.004123	up	-1.04805	-0.06771	1.048053	down	231.1009
down	1.082772	0.11473	1.082772	up	1.044611	0.062966	1.044611	up	22.12831
up	-1.13885	-0.18757	1.138847	down	-1.13775	-0.18619	1.137753	down	101.8356
down	-1.20137	-0.26468	1.201365	down	-1.17729	-0.23547	1.177293	down	117.9948
down	1.044895	0.063357	1.044895	up	1.053073	0.074605	1.053073	up	146.7801
up	-1.09642	-0.13281	1.096424	down	-1.07062	-0.09845	1.070622	down	466.0072
up	-1.01666	-0.02383	1.016658	down	-1.03765	-0.05332	1.037648	down	871.7835
down	1.07272	0.101274	1.07272	up	-1.05395	-0.0758	1.053945	down	279.1873

up	1.034306	0.048663	1.034306	up	1.067994	0.094904	1.067994	up	285.2304
up	1.162762	0.217556	1.162762	up	1.220102	0.287002	1.220102	up	92.82024
down	1.104467	0.143351	1.104467	up	1.029422	0.041834	1.029422	up	19.17744
up	1.159509	0.213513	1.159509	up	1.02607	0.037129	1.02607	up	703.2271
up	1.207777	0.272354	1.207777	up	1.096229	0.132549	1.096229	up	87.86771
down	-1.00303	-0.00437	1.00303	down	1.008657	0.012436	1.008657	up	53.54171
up	1.016783	0.024012	1.016783	up	1.023047	0.032873	1.023047	up	119.7443
up	1.057372	0.080482	1.057372	up	1.100985	0.138795	1.100985	up	1268.605
up	1.125221	0.170209	1.125221	up	1.113177	0.154682	1.113177	up	399.8713
up	-1.11123	-0.15216	1.111231	down	1.032047	0.045508	1.032047	up	98.88342
down	-1.11039	-0.15106	1.110387	down	-1.20017	-0.26324	1.200169	down	353.626
up	1.155447	0.208451	1.155447	up	-1.10053	-0.1382	1.10053	down	41.49437
up	-1.09384	-0.1294	1.093837	down	1.000774	0.001116	1.000774	up	41.23088
down	-1.11796	-0.16087	1.117961	down	-1.32043	-0.40101	1.320432	down	938.4929
down	1.226683	0.294763	1.226683	up	1.059854	0.083866	1.059854	up	15.16703
down	1.594105	0.672747	1.594105	up	1.274615	0.350061	1.274615	up	551.4731
down	1.031889	0.045287	1.031889	up	1.087771	0.121375	1.087771	up	99.02662
up	-1.24258	-0.31334	1.242577	down	-1.04661	-0.06572	1.046611	down	210.1065
up	-1.15471	-0.20754	1.154715	down	1.120829	0.164566	1.120829	up	281.0943
up	-1.13454	-0.18211	1.134545	down	1.075929	0.105582	1.075929	up	564.6181
down	-1.14459	-0.19484	1.144594	down	-1.0894	-0.12354	1.089401	down	244.1352
up	-1.08902	-0.12303	1.08902	down	-1.23839	-0.30847	1.238393	down	143.9004
up	1.110359	0.151026	1.110359	up	1.075896	0.105539	1.075896	up	44.1348
down	-1.39372	-0.47894	1.393718	down	-1.5153	-0.5996	1.515297	down	354.5585
down	1.029661	0.04217	1.029661	up	-1.01982	-0.02831	1.019816	down	303.6723
down	1.565077	0.646234	1.565077	up	1.654864	0.726713	1.654864	up	434.4763
up	-1.01787	-0.02556	1.017873	down	-1.00544	-0.00782	1.005436	down	1428.241
down	-1.00334	-0.0048	1.003335	down	1.082623	0.114531	1.082623	up	233.1659
up	-1.12876	-0.17473	1.128757	down	1.006745	0.009698	1.006745	up	67.48917
up	-1.56939	-0.65021	1.569392	down	-1.31791	-0.39826	1.317914	down	131.3814
up	-1.3691	-0.45323	1.369099	down	-1.3322	-0.41381	1.332196	down	126.7122
up	1.174054	0.231499	1.174054	up	1.144113	0.19423	1.144113	up	129.7859
down	-1.03183	-0.0452	1.031828	down	-1.25805	-0.33118	1.258045	down	28.1903
down	1.016228	0.023224	1.016228	up	1.097402	0.134092	1.097402	up	185.8026
down	1.254438	0.327041	1.254438	up	-1.37419	-0.45858	1.374193	down	15.03133
down	1.234398	0.303808	1.234398	up	-1.06479	-0.09057	1.064788	down	64.41387
down	-1.02716	-0.03866	1.027161	down	1.071076	0.09906	1.071076	up	74.58476
up	1.397891	0.483252	1.397891	up	1.171847	0.228784	1.171847	up	162.6969
down	1.167026	0.222837	1.167026	up	-1.05902	-0.08273	1.059023	down	688.28
up	-1.06009	-0.08418	1.060088	down	1.050833	0.071534	1.050833	up	160.9119
down	1.044017	0.062146	1.044017	up	1.092542	0.127688	1.092542	up	80.49455
down	-1.08258	-0.11447	1.082579	down	-1.08142	-0.11293	1.081419	down	67.15221
down	1.117114	0.159776	1.117114	up	-1.06201	-0.0868	1.06201	down	40.14721
up	-1.09426	-0.12996	1.094263	down	1.037105	0.052562	1.037105	up	49.39928
down	-1.07211	-0.10046	1.072112	down	-1.17702	-0.23514	1.177019	down	105.8219
down	-1.1687	-0.2249	1.1687	down	-1.25405	-0.32659	1.254046	down	38.99765
up	1.095764	0.131937	1.095764	up	1.115278	0.157403	1.115278	up	43.67386

up	1.1364	0.184471	1.1364	up	-1.0421	-0.0595	1.042101	down	3405.635
down	1.113785	0.155471	1.113785	up	1.021408	0.030559	1.021408	up	72.43608
up	1.046873	0.066087	1.046873	up	1.109118	0.149413	1.109118	up	26.82519
down	-1.03028	-0.04303	1.030279	down	-1.06895	-0.09619	1.068945	down	19.13668
down	1.049544	0.069763	1.049544	up	1.330514	0.411984	1.330514	up	23.2584
up	1.123919	0.168538	1.123919	up	1.04456	0.062895	1.04456	up	181.9258
down	1.037194	0.052686	1.037194	up	1.052062	0.07322	1.052062	up	41.81533
up	-1.11941	-0.16274	1.119409	down	1.068047	0.094975	1.068047	up	319.3959
down	-1.05185	-0.07293	1.051848	down	-1.10734	-0.1471	1.10734	down	92.09772
up	1.149614	0.201149	1.149614	up	1.054749	0.0769	1.054749	up	141.8875
up	1.019337	0.027631	1.019337	up	-1.02786	-0.03965	1.027862	down	41.26659
down	-1.02417	-0.03446	1.024172	down	-1.097	-0.13356	1.096998	down	95.49891
down	1.010392	0.014916	1.010392	up	1.10641	0.145886	1.10641	up	36.15228
down	1.105385	0.144549	1.105385	up	1.164952	0.220271	1.164952	up	32.98294
down	1.346316	0.429017	1.346316	up	1.173396	0.23069	1.173396	up	55.5041
down	1.034635	0.049122	1.034635	up	-1.00116	-0.00168	1.001165	down	43.8139
down	-1.14792	-0.19902	1.147921	down	-1.1325	-0.17951	1.132497	down	45.91834
up	1.000653	9.41E-04	1.000653	up	-1.05651	-0.07931	1.056513	down	832.033
down	1.057471	0.080619	1.057471	up	1.090108	0.124472	1.090108	up	51.18851
down	-1.16895	-0.22521	1.168947	down	-1.03157	-0.04484	1.03157	down	89.64856
up	1.186567	0.246794	1.186567	up	1.285563	0.362401	1.285563	up	90.3327
up	1.125935	0.171123	1.125935	up	1.116197	0.158591	1.116197	up	2868.387
down	-1.05174	-0.07278	1.051739	down	-1.23965	-0.30994	1.239652	down	38.9656
down	-1.08836	-0.12215	1.088357	down	1.047332	0.066718	1.047332	up	83.8522
down	1.086949	0.120284	1.086949	up	1.123863	0.168467	1.123863	up	53.51431
up	1.212749	0.27828	1.212749	up	1.230293	0.299002	1.230293	up	45.79455
down	-1.1576	-0.21113	1.157597	down	-1.10184	-0.13992	1.101845	down	20.89571
down	1.172606	0.229718	1.172606	up	1.042583	0.060162	1.042583	up	37.90318
up	1.155913	0.209032	1.155913	up	1.069357	0.096744	1.069357	up	54.91591
down	1.364816	0.448707	1.364816	up	1.117354	0.160087	1.117354	up	1727.989
up	1.114208	0.156019	1.114208	up	1.09919	0.13644	1.09919	up	3905.434
up	-1.01759	-0.02516	1.01759	down	1.021014	0.030002	1.021014	up	3540.745
down	1.017206	0.024612	1.017206	up	-1.10152	-0.1395	1.10152	down	270.0085
up	-1.06046	-0.08469	1.060463	down	-1.02443	-0.03483	1.024434	down	1966.912
up	1.088775	0.122706	1.088775	up	1.000778	0.001122	1.000778	up	492.7524
down	1.32192	0.402635	1.32192	up	1.342605	0.425035	1.342605	up	705.7014
up	1.481477	0.567036	1.481477	up	1.279919	0.356053	1.279919	up	377.7947
down	1.078457	0.108968	1.078457	up	-1.21463	-0.28052	1.214634	down	1307.857
down	-1.01787	-0.02555	1.01787	down	1.129526	0.175717	1.129526	up	17.98023
down	1.062352	0.087262	1.062352	up	1.073591	0.102444	1.073591	up	208.7855
up	1.052633	0.074003	1.052633	up	1.003405	0.004904	1.003405	up	85.60644
up	-1.035	-0.04963	1.035	down	1.02916	0.041467	1.02916	up	4718.82
up	-1.21045	-0.27554	1.210447	down	-1.23168	-0.30063	1.231684	down	736.3125
down	-1.16047	-0.21471	1.160469	down	-1.03594	-0.05094	1.035942	down	272.6245
up	-1.09627	-0.1326	1.096267	down	1.048574	0.068428	1.048574	up	632.2778
down	-1.0295	-0.04194	1.0295	down	1.143854	0.193902	1.143854	up	44.27764
up	1.139069	0.187854	1.139069	up	1.046661	0.065795	1.046661	up	338.667

down	1.039544	0.055951	1.039544	up	1.091851	0.126776	1.091851	up	41.33183
down	1.014496	0.020764	1.014496	up	1.002188	0.003154	1.002188	up	194.2961
up	1.114674	0.156622	1.114674	up	-1.01202	-0.01724	1.012023	down	164.1926
up	-1.05022	-0.07069	1.050218	down	-1.10303	-0.14147	1.103027	down	187.8337
up	1.013258	0.019001	1.013258	up	-1.12695	-0.17242	1.126951	down	38.18986
down	1.034104	0.048381	1.034104	up	1.102453	0.140717	1.102453	up	41.5912
up	-1.10552	-0.14472	1.105519	down	1.11714	0.15981	1.11714	up	392.3423
up	1.027926	0.039737	1.027926	up	-1.01469	-0.02105	1.014695	down	322.3272
down	1.006145	0.008838	1.006145	up	-1.05551	-0.07794	1.055512	down	119.6111
up	-1.04441	-0.06269	1.044411	down	-1.04897	-0.06898	1.048971	down	2807.38
up	1.061144	0.08562	1.061144	up	-1.00627	-0.00901	1.006265	down	903.177
down	-1.16673	-0.22247	1.166727	down	-1.31452	-0.39454	1.314519	down	234.252
up	1.104604	0.143529	1.104604	up	1.147669	0.198706	1.147669	up	873.5159
up	1.172244	0.229272	1.172244	up	1.130519	0.176985	1.130519	up	654.5358
up	-1.02663	-0.03791	1.026628	down	1.084122	0.116527	1.084122	up	1210.467
up	1.114818	0.156808	1.114818	up	1.096938	0.133482	1.096938	up	846.8662
down	-1.00908	-0.01304	1.009081	down	-1.03321	-0.04714	1.033212	down	43.88074
up	-1.05194	-0.07305	1.051938	down	-1.17589	-0.23376	1.175893	down	370.2143
up	-1.05007	-0.07048	1.050067	down	-1.07591	-0.10556	1.07591	down	1311.83
down	1.35408	0.437313	1.35408	up	1.228963	0.297441	1.228963	up	1536.954
down	-1.02906	-0.04133	1.029064	down	-1.23946	-0.30972	1.239464	down	713.5344
down	1.28734	0.364394	1.28734	up	-1.16156	-0.21606	1.161556	down	546.3445
down	-1.36528	-0.44919	1.365275	down	-1.33581	-0.41772	1.335814	down	267.1954
up	1.01332	0.019089	1.01332	up	-1.04923	-0.06934	1.049235	down	159.954
up	-1.0606	-0.08489	1.060603	down	-1.0573	-0.08039	1.057302	down	396.4107
up	1.161555	0.216057	1.161555	up	1.182743	0.242137	1.182743	up	112.8692
down	-1.02373	-0.03384	1.023733	down	-1.55015	-0.63241	1.550152	down	264.2564
down	1.098349	0.135336	1.098349	up	1.179686	0.238403	1.179686	up	32.25351
down	1.094313	0.130025	1.094313	up	1.069184	0.096511	1.069184	up	58.60394
down	1.111839	0.152948	1.111839	up	-1.00628	-0.00903	1.006277	down	175.3553
up	-1.07144	-0.09956	1.071443	down	-1.2485	-0.32019	1.248496	down	28.66966
down	1.29246	0.37012	1.29246	up	1.170511	0.227138	1.170511	up	1033.148
up	-1.03832	-0.05424	1.038316	down	-1.10134	-0.13926	1.101341	down	2195.32
down	1.2033	0.266996	1.2033	up	1.144861	0.195173	1.144861	up	129.5442
up	1.185904	0.245988	1.185904	up	1.071801	0.100037	1.071801	up	193.1928
up	1.151493	0.203506	1.151493	up	1.016694	0.023886	1.016694	up	259.0113
up	1.223605	0.291138	1.223605	up	-1.01968	-0.02811	1.019677	down	47.09863
down	-1.03661	-0.05188	1.036614	down	-1.08408	-0.11647	1.084077	down	168.4546
up	1.139819	0.188805	1.139819	up	-1.0443	-0.06253	1.044298	down	261.5536
down	1.26498	0.339115	1.26498	up	1.174337	0.231846	1.174337	up	381.2381
up	-1.10013	-0.13768	1.100132	down	1.052439	0.073736	1.052439	up	678.9189
up	1.121919	0.165969	1.121919	up	1.038528	0.05454	1.038528	up	357.912
down	1.088493	0.122332	1.088493	up	-1.01818	-0.026	1.018183	down	65.85014
up	1.190409	0.251457	1.190409	up	1.160987	0.215352	1.160987	up	204.6375
up	1.357113	0.440541	1.357113	up	1.018645	0.026651	1.018645	up	1153.384
up	1.037266	0.052786	1.037266	up	1.004921	0.007081	1.004921	up	657.9764
down	1.164073	0.219181	1.164073	up	1.088231	0.121984	1.088231	up	1043.833

down	1.079222	0.109991	1.079222	up	1.037693	0.053379	1.037693	up	153.9099
up	-1.01001	-0.01437	1.010013	down	1.04243	0.05995	1.04243	up	422.3218
up	1.152864	0.205222	1.152864	up	1.029897	0.0425	1.029897	up	311.1758
down	1.512206	0.596655	1.512206	up	1.376233	0.460725	1.376233	up	223.69
up	-1.08843	-0.12225	1.088433	down	-1.02169	-0.03096	1.021693	down	1471.51
up	1.271718	0.346778	1.271718	up	1.109588	0.150024	1.109588	up	625.5736
up	-1.05691	-0.07985	1.056906	down	1.02253	0.032143	1.02253	up	1416.877
down	1.936605	0.953529	1.936605	up	1.48159	0.567146	1.48159	up	454.9476
up	-1.13735	-0.18568	1.13735	down	-1.04458	-0.06292	1.044576	down	356.009
up	-1.00438	-0.0063	1.004379	down	-1.05765	-0.08086	1.057647	down	193.1355
up	-1.01056	-0.01515	1.010556	down	-1.02844	-0.04046	1.028444	down	603.8591
up	1.137373	0.185706	1.137373	up	1.17228	0.229317	1.17228	up	391.3654
up	1.131904	0.178752	1.131904	up	1.080878	0.112204	1.080878	up	288.4287
up	-1.04824	-0.06797	1.048241	down	-1.05069	-0.07133	1.050686	down	141.8865
down	-1.07784	-0.10814	1.077839	down	-1.05543	-0.07784	1.055434	down	28.80581
up	1.269244	0.34397	1.269244	up	1.07043	0.09819	1.07043	up	389.2567
up	-1.04506	-0.06358	1.045056	down	1.081168	0.11259	1.081168	up	210.5323
up	1.043524	0.061464	1.043524	up	1.110076	0.150659	1.110076	up	2231.898
up	-1.0234	-0.03336	1.023395	down	1.0559	0.078473	1.0559	up	1258.94
up	-1.01668	-0.02387	1.016682	down	-1.02365	-0.03373	1.023653	down	1292.273
down	-1.05921	-0.08299	1.05921	down	1.000326	4.70E-04	1.000326	up	1882.667
down	1.247207	0.318701	1.247207	up	1.260019	0.333446	1.260019	up	202.9789
up	1.100803	0.138556	1.100803	up	1.132752	0.179832	1.132752	up	404.4835
up	-1.06104	-0.08547	1.061036	down	-1.00046	-6.64E-04	1.00046	down	967.8926
up	-1.03605	-0.05109	1.03605	down	1.026969	0.038393	1.026969	up	3119.734
up	-1.00778	-0.01119	1.007784	down	-1.03046	-0.04329	1.03046	down	162.304
down	1.188097	0.248653	1.188097	up	1.122572	0.166808	1.122572	up	615.6129
up	-1.0698	-0.09733	1.069795	down	-1.03682	-0.05217	1.03682	down	471.6424
up	-1.21337	-0.27902	1.21337	down	-1.06417	-0.08972	1.064166	down	222.6478
up	-1.07985	-0.11083	1.079851	down	-1.0098	-0.01407	1.009799	down	1168.621
down	1.435057	0.521108	1.435057	up	1.375743	0.460211	1.375743	up	864.3143
up	-1.05709	-0.0801	1.057089	down	1.049857	0.070193	1.049857	up	1160.365
up	-1.08626	-0.11937	1.086264	down	1.016173	0.023147	1.016173	up	1190.967
up	1.03348	0.047511	1.03348	up	-1.07018	-0.09786	1.070181	down	2318.053
up	1.048286	0.068032	1.048286	up	1.003049	0.004393	1.003049	up	977.2936
up	1.003438	0.004952	1.003438	up	-1.15642	-0.20966	1.156419	down	66.95858
down	-1.15922	-0.21315	1.159217	down	-1.15115	-0.20307	1.151147	down	332.678
down	-1.06781	-0.09465	1.067806	down	1.033885	0.048076	1.033885	up	110.7552
up	-1.11342	-0.15499	1.113415	down	1.118233	0.161221	1.118233	up	36.24921
down	-1.12075	-0.16447	1.120754	down	-1.09179	-0.12669	1.09179	down	66.92487
down	-1.00711	-0.01022	1.007111	down	-1.14873	-0.20004	1.148734	down	166.0003
up	1.031117	0.044208	1.031117	up	-1.01166	-0.01672	1.011657	down	590.0476
up	1.048526	0.068362	1.048526	up	1.030373	0.043167	1.030373	up	131.2404
up	1.116578	0.159083	1.116578	up	1.175636	0.233442	1.175636	up	134.69
down	-1.16497	-0.22029	1.164968	down	-1.00099	-0.00142	1.000988	down	367.4845
down	1.004898	0.007048	1.004898	up	-1.05643	-0.0792	1.056432	down	150.7482
up	-1.00598	-0.0086	1.005976	down	-1.06062	-0.08491	1.060624	down	2013.273

down	-1.15587	-0.20898	1.155869	down	-1.05568	-0.07817	1.055679	down	251.9339
up	-1.04082	-0.05773	1.040824	down	1.049498	0.069699	1.049498	up	344.7418
down	1.130176	0.176547	1.130176	up	1.082302	0.114103	1.082302	up	1442.096
up	-1.23113	-0.29998	1.231129	down	-1.06356	-0.0889	1.06356	down	1073.255
up	-1.11118	-0.1521	1.111184	down	-1.08252	-0.11439	1.08252	down	470.8868
down	-1.01274	-0.01826	1.012738	down	-1.02206	-0.03148	1.022062	down	163.557
up	1.083782	0.116074	1.083782	up	1.017045	0.024383	1.017045	up	171.9025
up	-1.15478	-0.20762	1.154783	down	-1.04241	-0.05993	1.042412	down	402.2732
down	-1.13524	-0.183	1.135244	down	1.076682	0.106592	1.076682	up	687.661
up	1.010529	0.015111	1.010529	up	1.03428	0.048627	1.03428	up	758.451
down	1.04223	0.059674	1.04223	up	1.19184	0.25319	1.19184	up	407.2918
down	1.309302	0.388798	1.309302	up	1.183472	0.243025	1.183472	up	69.51971
up	1.067296	0.09396	1.067296	up	-1.07704	-0.10707	1.077038	down	143.9662
down	-1.43637	-0.52242	1.436368	down	-1.16888	-0.22513	1.168882	down	110.9774
up	1.5429	0.625644	1.5429	up	1.265656	0.339885	1.265656	up	354.8297
down	1.256813	0.32977	1.256813	up	1.174251	0.23174	1.174251	up	88.77799
up	-1.03722	-0.05273	1.037223	down	1.022011	0.031411	1.022011	up	224.5979
up	1.10782	0.147724	1.10782	up	1.119896	0.163365	1.119896	up	182.886
up	-1.04098	-0.05794	1.04098	down	-1.03495	-0.04956	1.034952	down	3986.485
down	1.0776	0.107822	1.0776	up	-1.09333	-0.12872	1.093325	down	540.45
down	1.119963	0.163451	1.119963	up	1.242793	0.313586	1.242793	up	88.4409
down	1.066158	0.092422	1.066158	up	1.01874	0.026785	1.01874	up	691.0805
up	1.167143	0.222981	1.167143	up	1.319127	0.399583	1.319127	up	87.12655
up	-1.07597	-0.10564	1.075973	down	-1.08169	-0.11328	1.081688	down	180.5207
down	-1.14255	-0.19226	1.14255	down	-1.02745	-0.03906	1.027447	down	115.0166
up	1.585393	0.66484	1.585393	up	1.593196	0.671924	1.593196	up	464.6429
up	-1.03468	-0.04919	1.034681	down	-1.07459	-0.10379	1.074595	down	282.5972
down	-1.31175	-0.39149	1.311746	down	-1.3725	-0.4568	1.372496	down	135.4964
down	1.010338	0.014839	1.010338	up	1.066205	0.092485	1.066205	up	175.7503
up	-1.1455	-0.19598	1.145501	down	1.002623	0.003779	1.002623	up	599.6914
down	-1.0762	-0.10594	1.076196	down	1.161865	0.216442	1.161865	up	117.8756
down	-1.09356	-0.12903	1.093559	down	-1.06446	-0.09012	1.064456	down	3056.961
up	-1.13069	-0.17721	1.130693	down	-1.13841	-0.18702	1.138414	down	96.75254
down	1.277809	0.353672	1.277809	up	1.141889	0.191423	1.141889	up	33.32467
down	-1.02029	-0.02898	1.020292	down	-1.0035	-0.00504	1.003502	down	90.75806
up	-1.03148	-0.04471	1.031479	down	-1.04881	-0.06876	1.048811	down	86.70892
up	1.13087	0.177433	1.13087	up	1.158964	0.212836	1.158964	up	39.47292
up	-1.07124	-0.09928	1.071237	down	-1.17329	-0.23055	1.173286	down	137.936
down	-1.04044	-0.05719	1.04044	down	-1.92809	-0.94717	1.92809	down	2792.029
up	-1.7395	-0.79868	1.739504	down	-1.48759	-0.57298	1.487593	down	153.752
up	1.030127	0.042823	1.030127	up	1.009869	0.014168	1.009869	up	309.488
up	-1.10099	-0.1388	1.100991	down	1.012117	0.017376	1.012117	up	206.0061
up	-1.10455	-0.14346	1.104553	down	-1.04324	-0.06108	1.043243	down	102.1237
down	1.123459	0.167947	1.123459	up	-1.13848	-0.1871	1.138476	down	49.12167
down	1.061424	0.086001	1.061424	up	-1.10299	-0.14142	1.102989	down	133.5355
up	1.190416	0.251465	1.190416	up	1.151099	0.203012	1.151099	up	181.0008
down	1.016277	0.023293	1.016277	up	1.088205	0.12195	1.088205	up	2345.71

down	1.142494	0.192186	1.142494	up	1.186418	0.246613	1.186418	up	699.6418
up	-1.07128	-0.09934	1.071281	down	-1.21191	-0.27728	1.211908	down	78.44198
down	1.010746	0.01542	1.010746	up	1.082393	0.114224	1.082393	up	25.05905
up	-1.1299	-0.1762	1.129902	down	-1.11753	-0.16032	1.117533	down	43.05139
up	1.252641	0.324973	1.252641	up	1.199386	0.262296	1.199386	up	75.72656
down	1.019508	0.027873	1.019508	up	1.06429	0.089891	1.06429	up	82.38564
up	1.111287	0.152231	1.111287	up	1.224995	0.292776	1.224995	up	89.14966
up	1.576831	0.657028	1.576831	up	1.27001	0.34484	1.27001	up	32.05941
down	1.004353	0.006267	1.004353	up	-1.11145	-0.15245	1.111454	down	34.34367
up	1.072116	0.10046	1.072116	up	-1.03434	-0.04871	1.034338	down	395.4791
up	-1.01744	-0.02495	1.017442	down	1.042213	0.05965	1.042213	up	737.9222
up	1.137361	0.18569	1.137361	up	1.090017	0.124351	1.090017	up	753.8361
up	1.061252	0.085767	1.061252	up	1.159121	0.213031	1.159121	up	140.1125
up	1.222724	0.290099	1.222724	up	1.086919	0.120244	1.086919	up	4811.838
up	-1.14628	-0.19696	1.14628	down	-1.21434	-0.28018	1.214343	down	83.77615
down	1.129246	0.17536	1.129246	up	1.092344	0.127427	1.092344	up	23.56731
down	1.216311	0.282512	1.216311	up	1.277037	0.3528	1.277037	up	236.5047
down	1.049553	0.069775	1.049553	up	1.118495	0.161559	1.118495	up	95.83975
down	1.01607	0.023	1.01607	up	1.053523	0.075222	1.053523	up	51.84906
up	1.267243	0.341694	1.267243	up	1.068463	0.095538	1.068463	up	20.61243
up	1.073832	0.102769	1.073832	up	1.036946	0.052341	1.036946	up	35.72963
up	-1.00507	-0.0073	1.005074	down	-1.01304	-0.01869	1.013042	down	1221.831
up	-1.09482	-0.13069	1.094821	down	-1.00407	-0.00586	1.004073	down	163.3581
down	1.180511	0.239411	1.180511	up	1.125173	0.170146	1.125173	up	63.28035
down	-1.07319	-0.10191	1.073193	down	-1.06661	-0.09303	1.066608	down	96.49412
up	1.027784	0.039538	1.027784	up	-1.06592	-0.0921	1.065918	down	79.08085
up	1.134441	0.181982	1.134441	up	1.200956	0.264183	1.200956	up	58.44863
down	1.040074	0.056686	1.040074	up	1.123069	0.167446	1.123069	up	56.57647
down	-1.00062	-8.88E-04	1.000616	down	1.024029	0.034256	1.024029	up	28.34267
down	-1.04096	-0.05791	1.040956	down	-1.09438	-0.13011	1.094379	down	26.52183
up	-1.00647	-0.0093	1.006469	down	1.033636	0.047728	1.033636	up	390.2743
up	-1.05855	-0.08208	1.058546	down	1.028655	0.040759	1.028655	up	317.2325
up	1.036532	0.051764	1.036532	up	1.168073	0.22413	1.168073	up	1489.521
up	1.340899	0.423201	1.340899	up	1.165692	0.221187	1.165692	up	91.54041
up	-1.12324	-0.16767	1.123241	down	-1.05476	-0.07692	1.054765	down	21.83267
down	-1.04194	-0.05928	1.041943	down	1.136622	0.184752	1.136622	up	51.57071
down	-1.3752	-0.45964	1.375199	down	-1.1261	-0.17134	1.126105	down	48.82709
up	1.034887	0.049473	1.034887	up	1.012175	0.017458	1.012175	up	27.59736
up	1.34649	0.429204	1.34649	up	1.258947	0.332218	1.258947	up	28.49509
down	1.123612	0.168144	1.123612	up	1.084109	0.116509	1.084109	up	209.1858
up	-1.01146	-0.01644	1.011461	down	1.057639	0.080847	1.057639	up	4026.306
up	1.052357	0.073625	1.052357	up	-1.02378	-0.0339	1.023778	down	290.7628
up	-1.03138	-0.04457	1.031377	down	-1.20475	-0.26873	1.204746	down	1665.817
up	1.001159	0.001671	1.001159	up	1.004291	0.006177	1.004291	up	1639.472
up	1.459758	0.545729	1.459758	up	1.359294	0.442857	1.359294	up	697.0718
up	-1.07972	-0.11066	1.079725	down	-1.13573	-0.18361	1.135726	down	707.5933
up	-1.06407	-0.0896	1.064075	down	1.006778	0.009745	1.006778	up	259.3086

down	-1.08669	-0.11994	1.086692	down	-1.01074	-0.01541	1.010737	down	329.0323
down	1.06314	0.088332	1.06314	up	-1.05124	-0.0721	1.051242	down	386.9888
down	1.011623	0.016672	1.011623	up	-1.01064	-0.01527	1.01064	down	522.2763
down	1.014882	0.021312	1.014882	up	1.242191	0.312887	1.242191	up	399.3511
up	-1.16631	-0.22195	1.166305	down	-1.09224	-0.1273	1.092245	down	302.1949
up	-1.12098	-0.16476	1.120983	down	1.060328	0.084511	1.060328	up	461.5002
down	-1.07893	-0.10959	1.078925	down	-1.09367	-0.12918	1.093673	down	220.2658
up	1.055985	0.078589	1.055985	up	1.032565	0.046233	1.032565	up	769.4852
up	-1.07879	-0.10942	1.078791	down	1.039095	0.055328	1.039095	up	362.3502
down	1.02093	0.029884	1.02093	up	-1.10208	-0.14023	1.102084	down	80.31593
down	-1.12609	-0.17133	1.126095	down	-1.14938	-0.20086	1.149381	down	357.2954
down	1.140387	0.189523	1.140387	up	1.213865	0.279608	1.213865	up	310.5309
up	-1.09316	-0.12851	1.093164	down	-1.47363	-0.55938	1.473634	down	69.7207
up	-1.04938	-0.06954	1.049379	down	1.061398	0.085966	1.061398	up	639.2482
down	1.175582	0.233375	1.175582	up	-1.22997	-0.29862	1.229968	down	112.09
up	1.183968	0.243629	1.183968	up	1.214632	0.28052	1.214632	up	683.7303
up	1.055425	0.077825	1.055425	up	1.091941	0.126894	1.091941	up	235.7234
up	1.041334	0.058433	1.041334	up	-1.00243	-0.0035	1.002432	down	1002.254
up	1.037738	0.053442	1.037738	up	1.047422	0.066842	1.047422	up	720.4492
up	-1.15816	-0.21184	1.158164	down	-1.15728	-0.21074	1.157284	down	1503.955
up	1.175119	0.232806	1.175119	up	1.203094	0.26675	1.203094	up	195.5601
down	1.498554	0.583571	1.498554	up	1.256618	0.329546	1.256618	up	1414.94
down	-1.04328	-0.06113	1.043282	down	-1.24459	-0.31567	1.244588	down	66.96719
down	1.027861	0.039645	1.027861	up	1.015454	0.022125	1.015454	up	74.99274
up	-1.24551	-0.31673	1.245506	down	-1.19825	-0.26093	1.198254	down	571.0329
up	1.024656	0.035139	1.024656	up	1.019314	0.027599	1.019314	up	1200.88
down	-1.19882	-0.26161	1.19882	down	-1.34333	-0.42581	1.343328	down	29.59329
up	-1.06759	-0.09436	1.067591	down	-1.07629	-0.10606	1.076288	down	1560.426
down	-1.02015	-0.02878	1.020148	down	-1.0033	-0.00475	1.003297	down	244.9494
up	1.014722	0.021085	1.014722	up	-1.11257	-0.15389	1.112568	down	552.873
up	-1.01378	-0.01975	1.013782	down	1.008503	0.012216	1.008503	up	600.3167
up	-1.11793	-0.16083	1.117929	down	-1.01326	-0.019	1.013255	down	42.37185
down	1.657868	0.729329	1.657868	up	1.443442	0.529513	1.443442	up	762.907
up	-1.03891	-0.05507	1.038911	down	-1.13983	-0.18882	1.139831	down	899.6521
down	1.23496	0.304465	1.23496	up	1.123635	0.168173	1.123635	up	604.9068
up	1.262242	0.335988	1.262242	up	1.126002	0.17121	1.126002	up	340.9518
down	1.097048	0.133626	1.097048	up	1.238232	0.308281	1.238232	up	251.3741
up	1.166359	0.222012	1.166359	up	1.124579	0.169385	1.124579	up	518.3522
down	1.250926	0.322996	1.250926	up	1.113192	0.154703	1.113192	up	499.9845
down	1.067659	0.094451	1.067659	up	1.050525	0.07111	1.050525	up	207.9827
down	1.261698	0.335366	1.261698	up	1.141724	0.191213	1.141724	up	31.17028
up	-1.20946	-0.27436	1.20946	down	-1.15191	-0.20403	1.151913	down	152.3499
down	-1.0868	-0.12009	1.086803	down	-1.11437	-0.15622	1.114367	down	438.8709
up	-1.05978	-0.08377	1.059784	down	-1.02176	-0.03105	1.021757	down	507.597
up	-1.11615	-0.15853	1.116153	down	-1.05785	-0.08113	1.057847	down	187.8928
up	-1.09344	-0.12888	1.093443	down	1.051168	0.071994	1.051168	up	710.416
up	-1.09632	-0.13267	1.09632	down	1.006463	0.009294	1.006463	up	88.16366

down	1.403408	0.488934	1.403408	up	-1.08943	-0.12358	1.089433	down	240.4795
down	1.079908	0.110909	1.079908	up	1.116369	0.158814	1.116369	up	70.85915
up	-1.14679	-0.1976	1.146786	down	-1.15613	-0.2093	1.156127	down	103.4493
down	1.602955	0.680734	1.602955	up	1.209313	0.274187	1.209313	up	265.36
down	1.232042	0.301051	1.232042	up	1.153318	0.205791	1.153318	up	91.66077
down	1.169965	0.226465	1.169965	up	1.104687	0.143637	1.104687	up	1161.407
down	1.206045	0.270283	1.206045	up	1.077357	0.107496	1.077357	up	210.9398
up	1.096179	0.132483	1.096179	up	1.073736	0.10264	1.073736	up	1336.23
down	-1.22022	-0.28714	1.220221	down	-1.18037	-0.23924	1.18037	down	500.3871
up	1.063296	0.088544	1.063296	up	-1.09315	-0.12849	1.093152	down	140.6522
down	-1.00797	-0.01146	1.007975	down	-1.19148	-0.25275	1.191476	down	246.3908
down	1.17793	0.236254	1.17793	up	1.092063	0.127055	1.092063	up	88.79981
up	-1.11736	-0.1601	1.117364	down	-1.02893	-0.04114	1.028928	down	285.0521
up	1.128168	0.173982	1.128168	up	1.226744	0.294834	1.226744	up	125.9497
down	-1.35452	-0.43778	1.354521	down	-1.07224	-0.10063	1.072243	down	122.5447
up	-1.04933	-0.06947	1.049329	down	1.050721	0.071379	1.050721	up	128.0392
down	-1.07783	-0.10813	1.077827	down	-1.04075	-0.05762	1.04075	down	445.0204
up	1.070119	0.097772	1.070119	up	1.02999	0.042631	1.02999	up	82.89463
down	1.262685	0.336495	1.262685	up	-1.18088	-0.23986	1.180879	down	32.34388
up	1.120043	0.163554	1.120043	up	1.084897	0.117558	1.084897	up	66.72354
down	-1.07951	-0.11038	1.079513	down	-1.00939	-0.01349	1.009394	down	276.301
down	-1.1692	-0.22552	1.169198	down	-1.14258	-0.1923	1.142582	down	23.87768
up	-1.02971	-0.04223	1.029706	down	1.002089	0.00301	1.002089	up	34.63279
down	-1.01435	-0.02055	1.014348	down	-1.13968	-0.18863	1.139679	down	27.34932
up	-1.17321	-0.23046	1.173208	down	-1.08635	-0.11949	1.086351	down	46.78146
down	1.100549	0.138224	1.100549	up	1.073958	0.102937	1.073958	up	24.04349
down	-1.04033	-0.05704	1.040332	down	-1.18567	-0.24571	1.185674	down	43.40157
up	-1.02488	-0.03546	1.024882	down	1.027745	0.039482	1.027745	up	2977.148
down	-1.82496	-0.86787	1.824963	down	-1.40951	-0.49519	1.409511	down	360.5134
up	-1.02719	-0.0387	1.027188	down	-1.05112	-0.07193	1.051122	down	17.74441
down	-1.01593	-0.0228	1.015927	down	-1.04805	-0.06771	1.048049	down	520.0554
down	-1.04078	-0.05766	1.040775	down	-1.01184	-0.01698	1.011836	down	66.9085
down	-1.01525	-0.02184	1.015254	down	1.108969	0.14922	1.108969	up	53.31636
up	-1.19929	-0.26218	1.199287	down	-1.11239	-0.15366	1.112389	down	622.1436
down	1.202187	0.265661	1.202187	up	1.024587	0.035042	1.024587	up	7515.933
up	-1.07973	-0.11067	1.079728	down	-1.07644	-0.10627	1.076442	down	771.6064
up	1.045942	0.064803	1.045942	up	1.075612	0.105158	1.075612	up	440.0999
up	-1.015	-0.02147	1.014995	down	-1.01517	-0.02172	1.015172	down	266.1711
down	1.250879	0.322942	1.250879	up	1.029953	0.042579	1.029953	up	140.1382
down	-1.08092	-0.11226	1.080917	down	-1.14771	-0.19876	1.14771	down	2616.44
up	1.239758	0.310059	1.239758	up	1.183511	0.243073	1.183511	up	1563.575
up	-1.01594	-0.02282	1.015942	down	1.074547	0.103728	1.074547	up	1787.568
down	-1.10514	-0.14423	1.105142	down	-1.17084	-0.22755	1.170845	down	1942.679
up	-1.16912	-0.22542	1.169118	down	-1.09543	-0.1315	1.095433	down	427.2253
up	-1.154	-0.20664	1.153999	down	-1.10245	-0.14071	1.102449	down	63.05581
down	1.358215	0.441711	1.358215	up	-1.07012	-0.09777	1.070117	down	490.4417
down	1.046737	0.065899	1.046737	up	-1.11829	-0.16129	1.118286	down	54.13587

up	1.004191	0.006034	1.004191	up	-1.05557	-0.07802	1.055572	down	58.09277
down	-1.05446	-0.07651	1.054465	down	-1.0267	-0.03801	1.026699	down	64.91464
down	1.187987	0.248519	1.187987	up	1.054842	0.077026	1.054842	up	655.1697
up	1.525434	0.60922	1.525434	up	1.346175	0.428866	1.346175	up	140.4088
up	1.012051	0.017282	1.012051	up	1.027877	0.039668	1.027877	up	307.3457
up	1.044125	0.062295	1.044125	up	1.083114	0.115185	1.083114	up	1789.043
down	1.111818	0.152921	1.111818	up	1.012382	0.017753	1.012382	up	118.1801
up	-1.02552	-0.03636	1.025521	down	1.100873	0.138648	1.100873	up	1033.881
up	1.101801	0.139863	1.101801	up	1.087881	0.121521	1.087881	up	1398.227
down	1.004288	0.006173	1.004288	up	1.048715	0.068623	1.048715	up	12169.98
down	1.234957	0.304461	1.234957	up	1.065609	0.091678	1.065609	up	91.96527
up	1.008119	0.011666	1.008119	up	1.029534	0.041991	1.029534	up	1218.914
up	-1.06739	-0.09409	1.067389	down	1.071245	0.099288	1.071245	up	479.6332
up	-1.08645	-0.11962	1.086446	down	-1.17827	-0.23667	1.178272	down	1510.53
down	1.02091	0.029856	1.02091	up	1.005389	0.007754	1.005389	up	185.3295
down	1.109026	0.149294	1.109026	up	1.003619	0.005211	1.003619	up	83.33617
down	1.12506	0.170002	1.12506	up	-1.09295	-0.12823	1.09295	down	126.6683
up	1.157403	0.210891	1.157403	up	-1.05379	-0.07559	1.053795	down	984.647
up	1.128615	0.174554	1.128615	up	1.072404	0.100849	1.072404	up	160.868
up	-1.13996	-0.18898	1.139956	down	-1.08177	-0.11339	1.08177	down	166.0437
up	1.016428	0.023508	1.016428	up	-1.01647	-0.02357	1.016475	down	255.3029
up	-1.08124	-0.11269	1.081245	down	1.141722	0.191211	1.141722	up	126.0623
down	-1.01466	-0.021	1.014662	down	-1.04749	-0.06694	1.047493	down	551.9162
down	-1.07464	-0.10385	1.074638	down	-1.22217	-0.28944	1.222168	down	521.7676
down	-1.16072	-0.21501	1.160715	down	-1.16503	-0.22037	1.165029	down	1640.107
up	1.054062	0.075959	1.054062	up	1.009248	0.01328	1.009248	up	24.4315
up	1.346818	0.429555	1.346818	up	1.025644	0.036529	1.025644	up	30.69414
down	1.046157	0.065099	1.046157	up	1.079944	0.110956	1.079944	up	37.01614
up	1.141889	0.191422	1.141889	up	1.03249	0.046128	1.03249	up	122.6645
down	1.106691	0.146252	1.106691	up	-1.0108	-0.01549	1.010795	down	786.499
up	1.101321	0.139234	1.101321	up	-1.08586	-0.11884	1.085858	down	310.9217
down	-1.73537	-0.79524	1.735366	down	-1.55847	-0.64013	1.55847	down	154.5456
down	-1.30702	-0.38628	1.307016	down	-1.30992	-0.38948	1.309924	down	594.8845
up	1.200896	0.264111	1.200896	up	1.215543	0.2816	1.215543	up	105.1437
up	1.143053	0.192892	1.143053	up	1.190444	0.2515	1.190444	up	67.39072
up	-1.03925	-0.05554	1.039245	down	1.008263	0.011871	1.008263	up	64.64502
up	1.095449	0.131523	1.095449	up	1.053591	0.075314	1.053591	up	301.3032
down	1.11358	0.155205	1.11358	up	1.172958	0.230151	1.172958	up	24.83102
up	1.031841	0.04522	1.031841	up	-1.03043	-0.04325	1.030431	down	68.87515
up	-1.00196	-0.00283	1.001961	down	1.0702	0.097881	1.0702	up	54.23431
up	-1.00047	-6.81E-04	1.000472	down	-1.05456	-0.07665	1.054563	down	164.7921
up	1.012437	0.017831	1.012437	up	1.024469	0.034876	1.024469	up	262.5478
down	1.115181	0.157278	1.115181	up	1.097166	0.133782	1.097166	up	27.19392
up	-1.13827	-0.18685	1.138272	down	-1.05089	-0.07161	1.050887	down	22.39903
up	-1.23306	-0.30224	1.233061	down	1.086036	0.119072	1.086036	up	34.13014
up	1.224077	0.291695	1.224077	up	1.068404	0.095457	1.068404	up	63.08001
up	-1.16191	-0.2165	1.161912	down	-1.1568	-0.21014	1.156803	down	227.7608

up	1.201238	0.264523	1.201238	up	1.262008	0.335721	1.262008	up	57.7146
down	1.044797	0.063223	1.044797	up	1.214856	0.280785	1.214856	up	105.7584
up	1.037503	0.053115	1.037503	up	1.21658	0.282832	1.21658	up	34.23323
down	1.427808	0.513802	1.427808	up	1.268624	0.343264	1.268624	up	239.0392
up	1.130168	0.176537	1.130168	up	1.120183	0.163734	1.120183	up	304.6006
up	1.063646	0.089018	1.063646	up	1.068921	0.096155	1.068921	up	443.2549
up	1.073526	0.102357	1.073526	up	1.147006	0.197873	1.147006	up	22.6952
up	-1.08809	-0.1218	1.088089	down	-1.10867	-0.14884	1.108674	down	124.2544
up	1.102106	0.140263	1.102106	up	1.19496	0.256962	1.19496	up	674.0967
up	1.342764	0.425206	1.342764	up	1.132487	0.179495	1.132487	up	313.8546
up	-1.03818	-0.05406	1.038181	down	-1.02922	-0.04155	1.029222	down	5938.959
up	1.141559	0.191005	1.141559	up	1.278917	0.354923	1.278917	up	196.1242
up	1.064592	0.0903	1.064592	up	-1.19322	-0.25486	1.193224	down	399.5985
up	-1.20943	-0.27433	1.209431	down	-1.01179	-0.01691	1.011792	down	4374.284
up	1.213313	0.278952	1.213313	up	1.129897	0.176191	1.129897	up	140.3924
up	1.038966	0.055148	1.038966	up	1.143647	0.193642	1.143647	up	445.0436
up	-1.05907	-0.0828	1.059073	down	-1.06367	-0.08905	1.06367	down	136.511
down	1.140283	0.189391	1.140283	up	1.018066	0.025831	1.018066	up	2834.275
up	1.020991	0.029971	1.020991	up	1.012755	0.018285	1.012755	up	173.5931
down	-1.03507	-0.04973	1.035069	down	-1.05823	-0.08165	1.058228	down	358.0365
up	-1.03418	-0.04849	1.034181	down	1.021954	0.031329	1.021954	up	1348.005
down	1.01021	0.014655	1.01021	up	-1.15767	-0.21122	1.15767	down	1097.901
up	1.191978	0.253358	1.191978	up	-1.10458	-0.1435	1.104582	down	635.9064
down	1.153985	0.206625	1.153985	up	1.075979	0.105649	1.075979	up	627.457
up	-1.01289	-0.01848	1.012894	down	-1.00301	-0.00433	1.003006	down	706.6602
up	-1.05219	-0.07339	1.052189	down	-1.06095	-0.08535	1.060946	down	922.3208
up	-1.13628	-0.18432	1.136285	down	-1.31292	-0.39278	1.312919	down	470.1045
down	1.05759	0.08078	1.05759	up	-1.04812	-0.06781	1.048123	down	268.8944
down	-1.02564	-0.03653	1.025644	down	-1.11658	-0.15909	1.116582	down	396.273
up	1.018612	0.026604	1.018612	up	-1.04725	-0.06661	1.047252	down	185.0621
down	1.246738	0.318158	1.246738	up	1.06349	0.088806	1.06349	up	411.3726
down	1.215611	0.281682	1.215611	up	1.149903	0.201512	1.149903	up	321.489
up	-1.10449	-0.14338	1.10449	down	-1.07763	-0.10786	1.077631	down	2306.238
down	1.000702	0.001012	1.000702	up	1.041408	0.058535	1.041408	up	456.4422
up	-1.10529	-0.14443	1.105294	down	-1.06714	-0.09374	1.067136	down	856.3239
up	-1.09549	-0.13158	1.09549	down	-1.09105	-0.12571	1.091046	down	1050.05
up	-1.04023	-0.0569	1.040229	down	-1.1075	-0.14731	1.107499	down	424.8884
up	-1.26337	-0.33728	1.26337	down	-1.22394	-0.29153	1.223939	down	273.9972
down	-1.25543	-0.32818	1.255429	down	-1.26772	-0.34224	1.267723	down	283.8982
up	1.057345	0.080446	1.057345	up	1.020971	0.029942	1.020971	up	469.4689
up	-1.10323	-0.14174	1.103232	down	-1.09892	-0.13609	1.098922	down	23.38173
up	1.050625	0.071248	1.050625	up	1.127873	0.173605	1.127873	up	890.2591
up	1.022633	0.032289	1.022633	up	1.158858	0.212703	1.158858	up	948.301
up	-1.0394	-0.05574	1.039396	down	-1.02768	-0.03939	1.027678	down	239.698
down	-1.08215	-0.1139	1.082147	down	-1.00334	-0.00481	1.003341	down	570.627
up	1.030802	0.043767	1.030802	up	1.014733	0.021099	1.014733	up	464.5131
up	-1.25879	-0.33204	1.258792	down	-1.23767	-0.30763	1.237673	down	59.01073

up	1.005819	0.008371	1.005819	up	-1.18164	-0.24079	1.181641	down	170.2858
down	-1.05422	-0.07617	1.054218	down	1.005122	0.007371	1.005122	up	80.908
down	1.124578	0.169384	1.124578	up	1.119244	0.162524	1.119244	up	208.9646
down	-1.10028	-0.13787	1.100277	down	-1.10824	-0.14827	1.108241	down	509.4657
up	-1.25074	-0.32278	1.250736	down	-1.0877	-0.12128	1.087696	down	516.4639
up	-1.03276	-0.0465	1.032757	down	-1.07668	-0.10659	1.076677	down	31.3628
down	-1.08306	-0.11511	1.083055	down	-1.03752	-0.05314	1.037521	down	1356.53
up	-1.14464	-0.1949	1.144642	down	1.058664	0.082245	1.058664	up	597.773
down	-1.00029	-4.16E-04	1.000289	down	1.013849	0.019842	1.013849	up	673.3262
up	-1.29429	-0.37216	1.294286	down	-1.08658	-0.1198	1.086582	down	454.4688
up	-1.0115	-0.01649	1.011496	down	1.03306	0.046924	1.03306	up	134.2431
up	1.137667	0.186078	1.137667	up	1.105996	0.145346	1.105996	up	247.3828
down	1.393638	0.478856	1.393638	up	-1.14444	-0.19464	1.144437	down	443.2011
up	-1.15494	-0.20781	1.154937	down	-1.23121	-0.30008	1.231213	down	24.76325
up	1.118047	0.160981	1.118047	up	1.079045	0.109755	1.079045	up	93.91748
down	1.176601	0.234625	1.176601	up	1.079561	0.110445	1.079561	up	239.5938
up	-1.1352	-0.18295	1.135203	down	-1.15326	-0.20572	1.153258	down	41.00807
down	-1.40795	-0.4936	1.407952	down	-1.30947	-0.38899	1.309473	down	79.08599
up	-1.08987	-0.12416	1.089873	down	1.071183	0.099205	1.071183	up	374.3011
down	-1.06475	-0.09051	1.064746	down	-1.02267	-0.03234	1.022667	down	1025.264
up	1.044008	0.062133	1.044008	up	-1.00164	-0.00237	1.001643	down	2311.534
down	1.101801	0.139864	1.101801	up	-1.02616	-0.03726	1.026162	down	547.2561
down	1.230139	0.298821	1.230139	up	1.150846	0.202694	1.150846	up	1445.206
up	1.330158	0.411597	1.330158	up	1.179573	0.238265	1.179573	up	43.73544
up	1.123329	0.16778	1.123329	up	1.064167	0.089725	1.064167	up	273.3102
up	1.054154	0.076086	1.054154	up	-1.009	-0.01293	1.009002	down	7278.283
up	1.182827	0.242239	1.182827	up	1.060345	0.084534	1.060345	up	52.36746
up	-1.06919	-0.09652	1.069195	down	1.219834	0.286685	1.219834	up	298.8684
up	1.075254	0.104678	1.075254	up	1.037946	0.053732	1.037946	up	28.50715
down	1.115844	0.158135	1.115844	up	1.11409	0.155866	1.11409	up	1305.625
up	1.072435	0.10089	1.072435	up	1.042836	0.060513	1.042836	up	169.3717
down	1.044434	0.062721	1.044434	up	1.06783	0.094682	1.06783	up	22.60836
down	-1.0028	-0.00403	1.002797	down	-1.03629	-0.05142	1.036286	down	34.8167
down	1.013191	0.018906	1.013191	up	1.055397	0.077786	1.055397	up	12764.18
down	-1.08022	-0.11133	1.080224	down	1.053113	0.07466	1.053113	up	5098.132
up	-1.00116	-0.00167	1.00116	down	-1.00571	-0.00821	1.00571	down	1602.66
down	1.1257	0.170823	1.1257	up	-1.08795	-0.12161	1.087947	down	32.7834
up	1.017969	0.025694	1.017969	up	-1.03657	-0.05181	1.036567	down	418.3971
down	1.059676	0.083623	1.059676	up	-1.02738	-0.03897	1.027379	down	220.4177
up	-1.01055	-0.01514	1.010552	down	1.009871	0.014171	1.009871	up	140.2282
up	-1.04025	-0.05693	1.040253	down	-1.01682	-0.02406	1.016816	down	2868.158
down	1.161429	0.215901	1.161429	up	1.077197	0.107282	1.077197	up	22.3151
up	-1.12518	-0.17016	1.125181	down	-1.13491	-0.18257	1.134907	down	783.6274
down	1.443726	0.529797	1.443726	up	1.281451	0.357778	1.281451	up	37.87684
up	1.123946	0.168572	1.123946	up	1.0665	0.092884	1.0665	up	872.8716
down	1.037302	0.052836	1.037302	up	1.002095	0.003019	1.002095	up	10214.09
up	1.086717	0.119976	1.086717	up	1.10284	0.141223	1.10284	up	890.8838

up	-1.1075	-0.1473	1.107497	down	-1.05643	-0.0792	1.056431	down	3811.544
down	-1.03878	-0.05489	1.038782	down	1.027237	0.038768	1.027237	up	5790.277
down	1.010464	0.015018	1.010464	up	1.043247	0.061081	1.043247	up	2081.503
down	-1.12718	-0.17272	1.127184	down	-1.0387	-0.05478	1.038704	down	26.48612
down	-1.05204	-0.07319	1.052039	down	-1.12936	-0.17551	1.129361	down	294.0535
up	-1.07411	-0.10315	1.074115	down	-1.05733	-0.08043	1.057332	down	64.54367
up	1.333789	0.415531	1.333789	up	1.121334	0.165216	1.121334	up	45.90912
up	1.075457	0.104949	1.075457	up	1.172959	0.230152	1.172959	up	938.8278
up	-1.0607	-0.08501	1.060699	down	1.029063	0.041331	1.029063	up	8062.453
up	-1.00635	-0.00913	1.006349	down	1.04912	0.069179	1.04912	up	6742.664
down	-1.07185	-0.1001	1.071845	down	-1.00738	-0.0106	1.007376	down	8764.698
down	-1.16433	-0.2195	1.164329	down	-1.02353	-0.03355	1.023527	down	4887.248
up	-1.1677	-0.22367	1.167701	down	-1.05279	-0.07422	1.052793	down	327.6878
up	1.045152	0.063712	1.045152	up	1.06152	0.086131	1.06152	up	158.5132
down	1.197803	0.260391	1.197803	up	1.039017	0.055219	1.039017	up	775.6897
down	-1.0427	-0.06032	1.042696	down	-1.18729	-0.24768	1.187294	down	2040.504
down	1.333399	0.415108	1.333399	up	-1.02296	-0.03275	1.022959	down	39.39784
down	1.04061	0.057429	1.04061	up	-1.04737	-0.06676	1.047366	down	162.2922
up	-1.03058	-0.04346	1.030583	down	-1.02592	-0.03691	1.025916	down	992.9641
down	-1.02116	-0.03021	1.021161	down	-1.00442	-0.00637	1.004424	down	135.6076
down	-1.24273	-0.31352	1.242734	down	-1.25348	-0.32594	1.25348	down	41.06103
up	-1.04751	-0.06696	1.047507	down	1.035464	0.050278	1.035464	up	3508.21
down	-1.01478	-0.02117	1.014779	down	-1.05891	-0.08258	1.058912	down	379.4919
down	1.052444	0.073744	1.052444	up	-1.11713	-0.15979	1.117125	down	243.9386
down	-1.0904	-0.12485	1.090397	down	-1.16485	-0.22015	1.164852	down	150.6235
up	1.006647	0.009558	1.006647	up	1.077449	0.10762	1.077449	up	109.9936
up	-1.05428	-0.07626	1.05428	down	1.065688	0.091784	1.065688	up	4363.721
down	1.4701	0.555914	1.4701	up	1.197854	0.260452	1.197854	up	487.3316
down	-1.48808	-0.57345	1.488076	down	-1.42366	-0.5096	1.423657	down	46.30449
down	-1.03831	-0.05424	1.038313	down	1.040975	0.057935	1.040975	up	189.3863
down	1.020008	0.02858	1.020008	up	-1.08272	-0.11465	1.082716	down	1572.071
down	1.145683	0.196208	1.145683	up	1.167598	0.223543	1.167598	up	82.83631
down	-1.13691	-0.18512	1.136915	down	-1.23281	-0.30195	1.232806	down	36.38732
up	-1.0313	-0.04446	1.031298	down	-1.09833	-0.13531	1.098328	down	376.4413
up	1.29798	0.376268	1.29798	up	1.30548	0.38458	1.30548	up	155.0963
up	-1.0597	-0.08366	1.059704	down	1.109161	0.149468	1.109161	up	26.15209
down	1.080217	0.111321	1.080217	up	1.039627	0.056066	1.039627	up	154.7621
down	-1.02346	-0.03345	1.023459	down	1.050487	0.071058	1.050487	up	139.6071
down	-1.03315	-0.04704	1.033146	down	-1.02908	-0.04135	1.029076	down	103.8645
down	-1.09429	-0.12999	1.094288	down	-1.0861	-0.11916	1.086103	down	68.93671
down	1.165677	0.221168	1.165677	up	-1.06026	-0.08442	1.060262	down	19.02157
up	1.01066	0.015298	1.01066	up	1.000047	6.83E-05	1.000047	up	494.1859
up	1.125085	0.170034	1.125085	up	-1.05641	-0.07917	1.056406	down	30.75632
down	1.389041	0.474089	1.389041	up	1.118642	0.161749	1.118642	up	1689.438
up	1.22251	0.289847	1.22251	up	1.109266	0.149605	1.109266	up	133.2998
up	-1.05007	-0.07048	1.050069	down	1.006753	0.00971	1.006753	up	3714.364
down	-1.32106	-0.4017	1.321064	down	-1.26801	-0.34257	1.268013	down	2094.297

down	-1.1244	-0.16915	1.124396	down	1.029427	0.041841	1.029427	up	328.6393
up	-1.06554	-0.09159	1.065541	down	-1.12302	-0.16738	1.12302	down	724.1213
up	1.282723	0.359209	1.282723	up	1.111542	0.152563	1.111542	up	2041.566
up	1.169444	0.225822	1.169444	up	1.104615	0.143544	1.104615	up	436.3131
up	-1.00089	-0.00128	1.000888	down	-1.00947	-0.01359	1.009467	down	950.522
up	-1.03193	-0.04534	1.031928	down	-1.07856	-0.10911	1.078563	down	356.5495
up	1.16647	0.22215	1.16647	up	1.160664	0.21495	1.160664	up	658.4648
up	-1.02242	-0.03198	1.022417	down	-1.00238	-0.00343	1.002384	down	2569.383
down	1.17441	0.231936	1.17441	up	1.202205	0.265683	1.202205	up	193.2632
up	1.099286	0.136567	1.099286	up	1.072798	0.101379	1.072798	up	1327.55
up	1.396277	0.481585	1.396277	up	1.228982	0.297464	1.228982	up	86.04203
down	-1.01468	-0.02102	1.01468	down	-1.22597	-0.29392	1.225969	down	68.7001
up	-1.01019	-0.01463	1.01019	down	-1.01355	-0.01942	1.01355	down	1831.733
down	1.242424	0.313157	1.242424	up	1.19296	0.254545	1.19296	up	506.5639
down	1.157301	0.210764	1.157301	up	-1.00593	-0.00853	1.005928	down	2518.409
up	1.09853	0.135574	1.09853	up	1.075406	0.104882	1.075406	up	458.3399
down	1.153086	0.2055	1.153086	up	1.137694	0.186113	1.137694	up	839.7827
down	1.086196	0.119284	1.086196	up	1.023363	0.033318	1.023363	up	171.4697
down	1.383869	0.468707	1.383869	up	1.203605	0.267361	1.203605	up	1115.892
down	1.163757	0.218789	1.163757	up	1.045211	0.063794	1.045211	up	346.2677
up	-1.06322	-0.08844	1.063221	down	-1.07712	-0.10718	1.077122	down	79.77299
down	-1.11277	-0.15416	1.112774	down	-1.22612	-0.29409	1.226115	down	960.2024
up	-1.07602	-0.1057	1.076018	down	-1.0606	-0.08489	1.060604	down	601.7314
up	-1.01706	-0.0244	1.017056	down	-1.03295	-0.04678	1.032955	down	810.7706
up	-1.02121	-0.03029	1.021215	down	1.00749	0.010766	1.00749	up	123.2674
up	-1.11148	-0.15248	1.111479	down	-1.02537	-0.03614	1.025365	down	1380.93
up	-1.12455	-0.16935	1.124551	down	-1.05453	-0.0766	1.054527	down	3203.726
down	1.074484	0.103644	1.074484	up	1.049278	0.069397	1.049278	up	411.0553
down	1.125701	0.170824	1.125701	up	-1.0289	-0.0411	1.0289	down	80.50425
up	1.194172	0.256011	1.194172	up	1.148512	0.199765	1.148512	up	160.5223
up	1.299979	0.378488	1.299979	up	1.334344	0.41613	1.334344	up	146.2747
up	-1.08847	-0.1223	1.088468	down	-1.05196	-0.07308	1.051958	down	551.9991
down	1.095575	0.131688	1.095575	up	-1.15951	-0.21351	1.159507	down	196.9251
down	1.065923	0.092103	1.065923	up	-1.02225	-0.03174	1.022247	down	465.6607
down	-1.26212	-0.33585	1.262124	down	-1.25376	-0.32626	1.253756	down	286.4747
down	-1.00274	-0.00395	1.002743	down	-1.0043	-0.0062	1.004304	down	704.6314
down	1.008734	0.012546	1.008734	up	1.05151	0.072462	1.05151	up	59.30023
up	1.062763	0.08782	1.062763	up	1.146855	0.197683	1.146855	up	57.06441
down	1.021578	0.0308	1.021578	up	-1.07394	-0.10292	1.073943	down	1164.004
down	1.351007	0.434035	1.351007	up	1.187342	0.247736	1.187342	up	473.2646
up	1.090942	0.125574	1.090942	up	1.03655	0.051789	1.03655	up	421.7855
down	1.185257	0.2452	1.185257	up	1.109877	0.1504	1.109877	up	22.75139
up	1.042483	0.060024	1.042483	up	1.000567	8.17E-04	1.000567	up	206.4793
down	1.027541	0.039196	1.027541	up	-1.02182	-0.03114	1.02182	down	35.98347
up	1.123834	0.168429	1.123834	up	1.084378	0.116867	1.084378	up	3128.763
down	1.127358	0.172945	1.127358	up	1.017384	0.024865	1.017384	up	67.09824
up	-1.0268	-0.03816	1.0268	down	-1.17978	-0.23852	1.179782	down	125.7081

up	-1.0384	-0.05436	1.038399	down	1.110676	0.151437	1.110676	up	449.4929
down	1.09621	0.132524	1.09621	up	-1.15125	-0.2032	1.151252	down	66.51917
down	-1.0532	-0.07477	1.053196	down	-1.04154	-0.05871	1.041538	down	31.71535
down	1.231123	0.299975	1.231123	up	1.029398	0.041801	1.029398	up	20.90869
down	-1.13299	-0.18013	1.132987	down	1.024625	0.035096	1.024625	up	108.2174
down	1.11622	0.158621	1.11622	up	1.093201	0.128559	1.093201	up	37.68827
up	-1.4562	-0.5422	1.456196	down	-1.28932	-0.36661	1.289318	down	48.71068
up	1.00466	0.006707	1.00466	up	1.038249	0.054152	1.038249	up	172.0152
up	1.13244	0.179435	1.13244	up	1.09518	0.131168	1.09518	up	166.5812
up	-1.08149	-0.11302	1.081489	down	-1.08185	-0.11351	1.081854	down	782.2923
up	1.10214	0.140308	1.10214	up	-1.00027	-3.83E-04	1.000266	down	305.7174
up	-1.14183	-0.19134	1.141826	down	-1.13751	-0.18588	1.137509	down	20.0732
up	1.340758	0.423049	1.340758	up	1.219876	0.286734	1.219876	up	167.0847
down	1.054941	0.077163	1.054941	up	-1.03439	-0.04878	1.034389	down	48.58709
down	-1.05588	-0.07844	1.055876	down	-1.0766	-0.10648	1.076602	down	53.07474
down	-1.06855	-0.09565	1.068546	down	-1.01561	-0.02234	1.015605	down	21.59452
down	1.229474	0.298041	1.229474	up	1.255309	0.328043	1.255309	up	208.5062
up	1.436746	0.522805	1.436746	up	1.275035	0.350537	1.275035	up	624.5017
up	1.104959	0.143993	1.104959	up	1.089781	0.124038	1.089781	up	978.4199
up	-1.12233	-0.1665	1.122333	down	1.095797	0.13198	1.095797	up	414.2828
up	1.307951	0.387308	1.307951	up	1.043371	0.061252	1.043371	up	119.9105
up	1.210578	0.275697	1.210578	up	1.157873	0.211477	1.157873	up	29.76056
up	1.103979	0.142713	1.103979	up	1.058603	0.082161	1.058603	up	133.3284
down	-1.09789	-0.13473	1.097887	down	-1.02978	-0.04234	1.029781	down	140.4946
up	-1.00058	-8.32E-04	1.000577	down	-1.06954	-0.09699	1.069536	down	301.2132
down	-1.13077	-0.1773	1.130766	down	-1.40709	-0.49272	1.407094	down	115.3266
up	-1.00778	-0.01118	1.007781	down	1.039722	0.056197	1.039722	up	294.3846
up	-1.16793	-0.22395	1.16793	down	-1.13064	-0.17714	1.130644	down	132.0961
up	1.271841	0.346918	1.271841	up	1.194434	0.256327	1.194434	up	139.1717
down	-1.0746	-0.1038	1.074602	down	-1.0945	-0.13027	1.0945	down	36.3804
up	1.153622	0.20617	1.153622	up	1.111996	0.153152	1.111996	up	144.9041
down	-1.11459	-0.15651	1.114586	down	-2.04798	-1.0342	2.047982	down	625.4124
down	-1.18689	-0.24719	1.186893	down	-1.75664	-0.81282	1.756641	down	613.0159
up	1.04382	0.061873	1.04382	up	1.010698	0.015352	1.010698	up	1193.97
up	1.014857	0.021276	1.014857	up	1.035653	0.05054	1.035653	up	2056.414
down	-1.34118	-0.4235	1.341177	down	-1.06903	-0.0963	1.069026	down	45.53191
down	-1.23696	-0.3068	1.236958	down	-1.12209	-0.16619	1.122091	down	74.70206
up	1.161834	0.216404	1.161834	up	-1.03743	-0.05301	1.037429	down	68.56607
down	-1.04785	-0.06743	1.047852	down	-1.02898	-0.04122	1.028983	down	115.6807
up	1.407065	0.492689	1.407065	up	1.064472	0.090138	1.064472	up	192.7798
up	-1.07173	-0.09995	1.071734	down	-1.05034	-0.07085	1.050336	down	8598.077
down	1.161788	0.216347	1.161788	up	-1.02652	-0.03776	1.026522	down	405.885
down	1.081692	0.113289	1.081692	up	1.061037	0.085475	1.061037	up	74.59858
down	1.106397	0.145869	1.106397	up	-1.13317	-0.18036	1.133166	down	27.98707
down	-1.03537	-0.05014	1.035368	down	1.018301	0.026164	1.018301	up	38.25878
down	1.033661	0.047763	1.033661	up	1.046869	0.066081	1.046869	up	24.95929
down	1.17153	0.228394	1.17153	up	-1.08671	-0.11997	1.086715	down	140.0732

up	-2.27096	-1.1833	2.270957	down	-1.84534	-0.88388	1.845335	down	123.1854
down	1.072182	0.100549	1.072182	up	-1.08333	-0.11547	1.083327	down	78.76028
down	-1.07369	-0.10258	1.073691	down	1.042908	0.060612	1.042908	up	30.53207
down	1.144046	0.194145	1.144046	up	1.06854	0.09564	1.06854	up	54.5558
down	-1.09252	-0.12766	1.092519	down	1.032045	0.045506	1.032045	up	59.9148
up	1.030823	0.043797	1.030823	up	1.035723	0.050638	1.035723	up	1115.456
up	-1.04576	-0.06456	1.045763	down	1.066	0.092207	1.066	up	55.48357
down	1.083947	0.116294	1.083947	up	1.043661	0.061653	1.043661	up	40.26323
down	-1.09355	-0.12902	1.093554	down	-1.06431	-0.08991	1.064307	down	69.14326
down	-1.03819	-0.05407	1.038191	down	1.025991	0.037018	1.025991	up	65.54825
up	-1.15408	-0.20674	1.154079	down	-1.04344	-0.06135	1.043444	down	49.52704
down	1.009014	0.012946	1.009014	up	-1.03239	-0.04599	1.032389	down	57.16046
up	1.043926	0.062019	1.043926	up	-1.21845	-0.28505	1.218454	down	83.18156
down	1.060066	0.084154	1.060066	up	1.193468	0.25516	1.193468	up	44.22477
down	1.289431	0.366734	1.289431	up	1.125982	0.171184	1.125982	up	355.1639
down	1.293526	0.371309	1.293526	up	1.297048	0.375232	1.297048	up	921.9683
up	-1.05543	-0.07783	1.055428	down	-1.10798	-0.14793	1.107978	down	1268.908
down	1.241049	0.311559	1.241049	up	1.127366	0.172956	1.127366	up	1245.048
down	-3.3974	-1.76443	3.397399	down	-3.32815	-1.73472	3.328148	down	491.8449
down	-1.02884	-0.04102	1.028841	down	-1.01597	-0.02286	1.01597	down	177.7383
down	-1.13866	-0.18733	1.138658	down	1.036085	0.051142	1.036085	up	819.5064
up	-1.01286	-0.01843	1.012859	down	-1.38952	-0.47458	1.389515	down	80.3855
down	1.031246	0.044388	1.031246	up	1.15063	0.202424	1.15063	up	35.99077
down	1.30502	0.384072	1.30502	up	-1.01177	-0.01688	1.01177	down	807.1899
up	1.120343	0.16394	1.120343	up	1.047419	0.066838	1.047419	up	112.0121
up	-1.08697	-0.12031	1.086967	down	-1.05056	-0.07117	1.050565	down	4299.064
up	-1.31411	-0.39409	1.314113	down	-1.19253	-0.25402	1.192525	down	108.8991
up	1.091716	0.126597	1.091716	up	1.058546	0.082083	1.058546	up	1202.745
down	-1.09591	-0.13213	1.095911	down	-1.05102	-0.07179	1.051017	down	1179.55
up	-1.06241	-0.08734	1.062407	down	-1.05169	-0.07271	1.051694	down	1855.212
down	-1.15617	-0.20936	1.156175	down	-1.16776	-0.22375	1.167761	down	434.022
up	1.170505	0.227131	1.170505	up	1.076925	0.106918	1.076925	up	470.8477
up	-1.05894	-0.08262	1.058941	down	-1.05946	-0.08333	1.059458	down	1840.654
up	-1.02883	-0.041	1.028829	down	-1.10437	-0.14322	1.104367	down	52.6718
down	-1.0139	-0.01992	1.013905	down	-1.10975	-0.15023	1.109745	down	651.1466
down	1.036423	0.051613	1.036423	up	1.027693	0.039409	1.027693	up	22.79565
up	1.066838	0.093342	1.066838	up	1.146271	0.196948	1.146271	up	774.4193
down	1.125671	0.170785	1.125671	up	1.002281	0.003287	1.002281	up	237.0358
up	-1.00879	-0.01262	1.008788	down	1.108489	0.148595	1.108489	up	582.0757
up	-1.17461	-0.23218	1.174609	down	1.098897	0.136055	1.098897	up	27.91547
up	1.121692	0.165676	1.121692	up	1.033364	0.047348	1.033364	up	1592.679
down	-1.11814	-0.16111	1.118144	down	-1.01113	-0.01596	1.011127	down	165.3395
down	-1.03082	-0.04379	1.03082	down	1.002328	0.003355	1.002328	up	28.94803
down	1.000134	1.93E-04	1.000134	up	1.020558	0.029358	1.020558	up	1258.899
up	-1.27315	-0.3484	1.27315	down	-1.1731	-0.23032	1.173097	down	799.6895
up	1.16358	0.21857	1.16358	up	1.197788	0.260373	1.197788	up	189.1081
up	-1.10562	-0.14486	1.105623	down	-1.033	-0.04684	1.033002	down	116.076

up	-1.08523	-0.118	1.085232	down	-1.02548	-0.03629	1.025475	down	304.9149
down	1.193465	0.255156	1.193465	up	-1.01058	-0.01519	1.010583	down	522.5092
up	-1.14115	-0.19049	1.141152	down	-1.03843	-0.05441	1.038432	down	517.2759
up	1.221148	0.288238	1.221148	up	-1.02276	-0.03247	1.022759	down	222.8727
down	1.012999	0.018633	1.012999	up	-1.03292	-0.04672	1.032916	down	450.4676
down	-1.47975	-0.56536	1.479755	down	-1.41686	-0.5027	1.416865	down	99.96481
down	-1.00156	-0.00224	1.001556	down	1.072564	0.101064	1.072564	up	365.4865
down	-1.07817	-0.10858	1.078166	down	-1.0022	-0.00318	1.002204	down	60.3566
up	1.525952	0.609709	1.525952	up	1.458428	0.544414	1.458428	up	400.668
up	1.059688	0.08364	1.059688	up	1.038394	0.054354	1.038394	up	664.7329
down	1.043324	0.061187	1.043324	up	-1.30749	-0.3868	1.307491	down	258.7352
up	-1.09029	-0.12471	1.090287	down	-1.05481	-0.07698	1.054806	down	77.42541
down	1.017798	0.025452	1.017798	up	1.007801	0.01121	1.007801	up	155.6302
up	1.2542	0.326767	1.2542	up	1.122056	0.166145	1.122056	up	142.357
down	1.209024	0.273842	1.209024	up	-1.19034	-0.25138	1.190344	down	136.5206
down	1.015648	0.022401	1.015648	up	-1.00927	-0.01331	1.00927	down	786.7225
down	1.223193	0.290652	1.223193	up	-1.06351	-0.08884	1.063514	down	135.1536
up	1.209801	0.274769	1.209801	up	-1.16719	-0.22303	1.167185	down	43.04071
up	-1.33221	-0.41382	1.332206	down	-1.14839	-0.19961	1.148391	down	37.33745
up	-1.0915	-0.12631	1.091498	down	1.004556	0.006558	1.004556	up	17.54998
up	1.060075	0.084166	1.060075	up	-1.06394	-0.08942	1.063942	down	248.4294
up	1.03828	0.054195	1.03828	up	-1.22911	-0.29762	1.229114	down	29.19389
up	-1.04339	-0.06127	1.043387	down	1.123885	0.168494	1.123885	up	131.9041
up	-1.06738	-0.09408	1.067384	down	1.062994	0.088133	1.062994	up	408.6495
down	-1.14557	-0.19606	1.145567	down	-1.06578	-0.09191	1.065784	down	26.54825
up	1.362736	0.446506	1.362736	up	1.195948	0.258155	1.195948	up	2522.56
down	-1.08041	-0.11158	1.080412	down	-1.06307	-0.08824	1.063073	down	974.1723
down	1.029823	0.042396	1.029823	up	1.112128	0.153323	1.112128	up	581.2336
up	-1.05935	-0.08318	1.059349	down	1.083205	0.115307	1.083205	up	547.616
down	1.194387	0.256271	1.194387	up	-1.2551	-0.32781	1.255102	down	84.66191
up	1.015119	0.021649	1.015119	up	-1.02596	-0.03697	1.025958	down	2704.451
up	-1.10481	-0.14379	1.104807	down	1.007317	0.010518	1.007317	up	1391.519
down	1.404748	0.490311	1.404748	up	1.138951	0.187706	1.138951	up	834.6686
up	1.095381	0.131433	1.095381	up	1.002105	0.003034	1.002105	up	161.4538
up	-1.0637	-0.08909	1.063698	down	1.013788	0.019756	1.013788	up	388.9034
up	1.061466	0.086058	1.061466	up	1.029992	0.042633	1.029992	up	181.9437
down	-1.05231	-0.07356	1.052312	down	-1.02287	-0.03262	1.022869	down	56.66318
down	1.10374	0.1424	1.10374	up	1.156774	0.210107	1.156774	up	192.9773
up	-1.11937	-0.16269	1.119371	down	-1.04477	-0.06319	1.044774	down	673.4384
up	-1.35044	-0.43343	1.350443	down	-1.22898	-0.29746	1.22898	down	73.61059
up	1.036533	0.051766	1.036533	up	1.016596	0.023747	1.016596	up	1012.71
up	-1.06734	-0.09401	1.067336	down	-1.03457	-0.04902	1.034565	down	229.9146
up	1.129626	0.175845	1.129626	up	1.054325	0.076319	1.054325	up	37.06855
down	1.076329	0.106119	1.076329	up	-1.4478	-0.53387	1.447804	down	407.9828
down	-1.02062	-0.02945	1.020624	down	-1.06902	-0.0963	1.069025	down	1178.708
down	1.102856	0.141244	1.102856	up	1.049312	0.069444	1.049312	up	145.3383
down	-1.17413	-0.23159	1.174127	down	-1.09492	-0.13082	1.094918	down	501.3591

down	-1.02967	-0.04218	1.029666	down	-1.00718	-0.01032	1.007177	down	141.1054
down	1.151998	0.204138	1.151998	up	1.069312	0.096682	1.069312	up	1789.252
down	-1.02243	-0.032	1.022427	down	-1.06777	-0.0946	1.067772	down	215.689
up	-1.78603	-0.83676	1.786029	down	-1.4117	-0.49743	1.411699	down	34.61642
up	1.092074	0.12707	1.092074	up	1.150205	0.201891	1.150205	up	767.6079
down	-1.11422	-0.15604	1.114224	down	-1.10132	-0.13923	1.101318	down	120.9566
down	1.277104	0.352876	1.277104	up	1.223314	0.290795	1.223314	up	90.71233
down	-1.17849	-0.23694	1.178492	down	-1.32265	-0.40343	1.322648	down	111.9147
up	-1.14621	-0.19687	1.146213	down	-1.01461	-0.02092	1.014606	down	65.86812
down	1.275389	0.350937	1.275389	up	1.135235	0.182991	1.135235	up	312.8265
up	1.11387	0.155581	1.11387	up	1.168454	0.2246	1.168454	up	51.37463
down	-1.16822	-0.22431	1.168216	down	-1.11456	-0.15648	1.114561	down	1051.308
down	1.007874	0.011315	1.007874	up	1.162823	0.217631	1.162823	up	74.74431
down	1.061923	0.086679	1.061923	up	1.02507	0.035722	1.02507	up	161.0998
up	1.081124	0.112532	1.081124	up	1.15742	0.210912	1.15742	up	448.0855
down	1.033011	0.046856	1.033011	up	-1.11484	-0.15683	1.114837	down	139.2734
down	1.226248	0.29425	1.226248	up	1.204101	0.267956	1.204101	up	123.9652
up	-1.03739	-0.05295	1.037386	down	-1.13609	-0.18408	1.136092	down	168.0326
down	-1.20763	-0.27218	1.207634	down	-1.01057	-0.01516	1.010565	down	161.3537
down	1.062164	0.087006	1.062164	up	1.014101	0.020202	1.014101	up	459.4943
up	1.221987	0.289229	1.221987	up	1.137312	0.185629	1.137312	up	476.7878
up	-1.14293	-0.19274	1.14293	down	-1.09977	-0.1372	1.099767	down	2520.412
down	1.114457	0.156341	1.114457	up	1.054399	0.076421	1.054399	up	50.18469
up	-1.10112	-0.13897	1.101119	down	-1.04459	-0.06294	1.044592	down	733.752
up	-1.03092	-0.04394	1.030922	down	-1.0126	-0.01807	1.012602	down	80.2909
up	1.180461	0.23935	1.180461	up	1.199486	0.262417	1.199486	up	21.45731
up	-1.12326	-0.16769	1.123255	down	-1.07353	-0.10236	1.07353	down	678.161
up	1.013364	0.019152	1.013364	up	1.037578	0.05322	1.037578	up	523.2943
down	-1.0161	-0.02304	1.0161	down	-1.0515	-0.07245	1.051504	down	851.8809
up	1.053128	0.074681	1.053128	up	1.099719	0.137135	1.099719	up	1283.04
up	-1.0137	-0.01963	1.013703	down	1.030901	0.043906	1.030901	up	941.9753
up	-1.05662	-0.07945	1.056618	down	1.000299	4.31E-04	1.000299	up	1968.894
down	-1.03361	-0.04769	1.033608	down	-1.04251	-0.06006	1.04251	down	812.2209
up	-1.0063	-0.00905	1.006296	down	1.014308	0.020496	1.014308	up	2038.248
up	-1.02985	-0.04243	1.029847	down	-1.28985	-0.3672	1.289848	down	1343.533
down	-1.11884	-0.162	1.118841	down	-1.31556	-0.39568	1.315562	down	48.51429
down	1.032574	0.046246	1.032574	up	-1.04409	-0.06225	1.04409	down	182.8669
up	-1.10874	-0.14893	1.108743	down	-1.04718	-0.06651	1.047179	down	569.5651
down	1.065773	0.091901	1.065773	up	-1.03182	-0.04518	1.031815	down	590.1397
up	-1.10997	-0.15052	1.10997	down	-1.07256	-0.10105	1.072556	down	5149.952
up	1.028178	0.040091	1.028178	up	-1.0907	-0.12526	1.090704	down	1650.07
up	-1.20042	-0.26354	1.200421	down	-1.17211	-0.22911	1.172115	down	812.0522
up	-1.43233	-0.51836	1.43233	down	-1.25677	-0.32972	1.256771	down	28.38785
up	1.03929	0.055598	1.03929	up	-1.11074	-0.15153	1.110744	down	1070.484
down	1.022563	0.032189	1.022563	up	-1.02953	-0.04198	1.029525	down	1881.59
down	-1.00317	-0.00457	1.003172	down	-1.04339	-0.06128	1.043391	down	102.8049
down	1.085858	0.118835	1.085858	up	1.174026	0.231465	1.174026	up	124.3873

down	-1.16709	-0.22291	1.167086	down	-1.01851	-0.02646	1.01851	down	234.2942
down	-1.11759	-0.16039	1.117588	down	-1.14536	-0.1958	1.145362	down	43.31476
up	-1.08945	-0.1236	1.089449	down	1.004291	0.006178	1.004291	up	283.2751
up	1.013828	0.019813	1.013828	up	1.093544	0.129011	1.093544	up	5215.351
down	1.238792	0.308934	1.238792	up	1.066999	0.093559	1.066999	up	55.22139
down	1.080806	0.112107	1.080806	up	1.099698	0.137108	1.099698	up	783.6106
up	1.228305	0.296669	1.228305	up	1.134973	0.182658	1.134973	up	524.0078
down	-1.01647	-0.02356	1.016467	down	1.063477	0.088788	1.063477	up	696.669
up	-1.0172	-0.0246	1.017197	down	1.043486	0.061411	1.043486	up	2218.823
down	-1.18216	-0.24143	1.182162	down	-1.0456	-0.06433	1.045599	down	3211.692
up	-1.05772	-0.08095	1.057718	down	1.048342	0.06811	1.048342	up	4413.081
up	-1.01784	-0.02551	1.017841	down	-1.11217	-0.15338	1.112173	down	198.5641
down	-1.16381	-0.21885	1.163805	down	-1.18744	-0.24785	1.187437	down	26.00555
down	1.184431	0.244195	1.184431	up	1.24369	0.314627	1.24369	up	1105.524
up	-1.1985	-0.26123	1.198498	down	-1.22937	-0.29792	1.229369	down	86.75666
down	-1.07884	-0.10948	1.078836	down	1.006862	0.009865	1.006862	up	1832.881
down	1.238846	0.308997	1.238846	up	1.012273	0.017599	1.012273	up	1648.765
up	-1.07553	-0.10504	1.075527	down	-1.05285	-0.07431	1.052854	down	195.0322
up	1.214905	0.280843	1.214905	up	1.015757	0.022555	1.015757	up	146.717
up	-1.00213	-0.00307	1.00213	down	1.014725	0.021089	1.014725	up	56.23572
down	1.014411	0.020642	1.014411	up	1.049351	0.069498	1.049351	up	12956.04
down	1.01398	0.020029	1.01398	up	1.024644	0.035123	1.024644	up	37.19825
up	-1.01413	-0.02025	1.014135	down	-1.0303	-0.04307	1.030305	down	1622.926
down	1.175535	0.233318	1.175535	up	-1.02836	-0.04035	1.028361	down	454.9703
up	1.102502	0.140782	1.102502	up	1.128036	0.173814	1.128036	up	277.9025
down	1.273648	0.348966	1.273648	up	1.150301	0.202011	1.150301	up	379.7865
down	-1.04196	-0.05929	1.041956	down	1.018577	0.026555	1.018577	up	986.9075
up	1.025558	0.036409	1.025558	up	-1.03731	-0.05285	1.037312	down	83.67361
up	-1.096	-0.13225	1.096001	down	1.033686	0.047798	1.033686	up	21.33985
down	1.089473	0.123631	1.089473	up	1.066274	0.092578	1.066274	up	575.6282
down	1.123199	0.167613	1.123199	up	1.183643	0.243234	1.183643	up	76.82902
down	-1.03735	-0.0529	1.037346	down	1.092483	0.127611	1.092483	up	170.8036
up	-1.00594	-0.00855	1.005942	down	-1.13494	-0.18262	1.134944	down	693.6226
down	-1.11717	-0.15985	1.117168	down	1.056778	0.079673	1.056778	up	155.4579
down	-1.12588	-0.17106	1.125885	down	-1.00805	-0.01157	1.008052	down	195.6152
up	1.113482	0.155078	1.113482	up	1.095663	0.131804	1.095663	up	249.1164
up	-1.11409	-0.15586	1.114087	down	-1.0444	-0.06268	1.044401	down	214.3687
up	1.171997	0.228969	1.171997	up	1.10915	0.149454	1.10915	up	428.7306
up	-1.00031	-4.48E-04	1.000311	down	-1.07034	-0.09807	1.07034	down	361.0144
up	-1.00657	-0.00944	1.006566	down	1.054508	0.07657	1.054508	up	783.0325
down	1.250803	0.322854	1.250803	up	1.142386	0.19205	1.142386	up	627.7996
up	1.090726	0.125288	1.090726	up	1.037841	0.053586	1.037841	up	309.7795
up	-1.08994	-0.12424	1.089936	down	-1.01028	-0.01476	1.010284	down	578.7874
up	1.061877	0.086617	1.061877	up	1.092838	0.128079	1.092838	up	720.6613
up	1.017621	0.0252	1.017621	up	-1.00218	-0.00315	1.002183	down	1003.824
down	1.364703	0.448587	1.364703	up	1.23727	0.30716	1.23727	up	21.73682
up	1.082286	0.114082	1.082286	up	1.111697	0.152764	1.111697	up	179.2689

down	-1.00162	-0.00234	1.001623	down	1.053962	0.075823	1.053962	up	34.23631
up	-1.02597	-0.037	1.025975	down	-1.09559	-0.13171	1.095588	down	33.05372
down	1.341304	0.423637	1.341304	up	1.021991	0.031383	1.021991	up	260.3009
up	1.074691	0.103923	1.074691	up	1.195792	0.257966	1.195792	up	684.8144
up	-1.27901	-0.35502	1.279007	down	-1.14187	-0.1914	1.141875	down	305.636
down	-1.11015	-0.15075	1.110149	down	-1.02731	-0.03887	1.027306	down	177.8937
up	-1.07398	-0.10297	1.07398	down	-1.10546	-0.14464	1.105455	down	475.7095
up	-1.06002	-0.08409	1.06002	down	1.045829	0.064647	1.045829	up	131.2051
up	1.012561	0.018009	1.012561	up	1.110925	0.151762	1.110925	up	72.86932
up	1.019172	0.027398	1.019172	up	1.043868	0.061939	1.043868	up	83.74654
down	-1.03345	-0.04747	1.033451	down	-1.04724	-0.0666	1.047243	down	105.5622
up	1.029189	0.041508	1.029189	up	-1.01963	-0.02805	1.019634	down	1076.392
down	1.291325	0.368852	1.291325	up	1.400653	0.4861	1.400653	up	140.1635
up	1.136193	0.184208	1.136193	up	1.306428	0.385628	1.306428	up	44.54258
up	-1.4776	-0.56326	1.477603	down	-1.08761	-0.12116	1.087612	down	192.5642
up	-1.56991	-0.65068	1.56991	down	-1.19861	-0.26136	1.19861	down	128.6946
up	-1.09579	-0.13197	1.095788	down	1.077478	0.107658	1.077478	up	68.15972
down	1.287227	0.364267	1.287227	up	-1.11753	-0.16032	1.117531	down	276.1471
down	-1.04708	-0.06637	1.047079	down	-1.07756	-0.10777	1.077563	down	400.2573
up	-1.03818	-0.05406	1.038185	down	-1.0605	-0.08474	1.060498	down	570.1559
up	-1.01049	-0.01505	1.010489	down	-1.00718	-0.01032	1.007181	down	316.6069
up	-1.09751	-0.13423	1.097509	down	1.096737	0.133218	1.096737	up	125.9895
up	1.091368	0.126138	1.091368	up	1.045991	0.064871	1.045991	up	935.0026
up	1.069153	0.096468	1.069153	up	1.023111	0.032962	1.023111	up	2857.873
up	-1.07402	-0.10303	1.074025	down	1.056794	0.079694	1.056794	up	756.9484
up	1.172131	0.229134	1.172131	up	1.119425	0.162758	1.119425	up	282.0061
down	-1.00464	-0.00667	1.004636	down	1.170779	0.227469	1.170779	up	428.2622
up	1.043824	0.061879	1.043824	up	1.03828	0.054196	1.03828	up	1065.457
up	-1.13017	-0.17654	1.130171	down	-1.1317	-0.17849	1.1317	down	834.8826
up	-1.1559	-0.20901	1.155895	down	-1.12195	-0.166	1.121947	down	383.1625
up	1.081052	0.112436	1.081052	up	-1.02014	-0.02876	1.020137	down	508.8029
down	-1.0729	-0.10151	1.072899	down	1.050081	0.0705	1.050081	up	269.2974
down	-1.06358	-0.08893	1.063584	down	1.169577	0.225987	1.169577	up	39.71676
down	-1.23305	-0.30223	1.233052	down	-1.18859	-0.24925	1.188586	down	267.0659
up	-1.06297	-0.08811	1.062975	down	1.031482	0.044718	1.031482	up	38.47799
up	-1.08979	-0.12405	1.089788	down	1.021321	0.030437	1.021321	up	105.075
down	-1.02376	-0.03388	1.023762	down	-1.0038	-0.00547	1.003799	down	139.5898
down	1.207762	0.272337	1.207762	up	1.046991	0.06625	1.046991	up	140.732
down	1.04177	0.059037	1.04177	up	-1.18887	-0.24959	1.188866	down	370.5466
up	1.175306	0.233037	1.175306	up	1.059979	0.084035	1.059979	up	87.22134
down	1.208268	0.272941	1.208268	up	1.172594	0.229704	1.172594	up	238.8093
down	1.355122	0.438422	1.355122	up	-1.3594	-0.44297	1.359404	down	1181.914
down	1.093024	0.128325	1.093024	up	1.104761	0.143734	1.104761	up	21.79768
up	1.377357	0.461903	1.377357	up	1.326306	0.407413	1.326306	up	38.45139
up	1.298209	0.376522	1.298209	up	1.24362	0.314546	1.24362	up	330.271
up	-1.13826	-0.18684	1.138265	down	-1.01741	-0.0249	1.017409	down	121.6141
up	1.162613	0.217371	1.162613	up	1.079048	0.109759	1.079048	up	64.55196

up	1.048463	0.068276	1.048463	up	-1.01609	-0.02303	1.016092	down	227.8386
up	-1.17091	-0.22764	1.170915	down	-1.06642	-0.09277	1.066417	down	3892.824
up	-1.03028	-0.04303	1.030275	down	1.082562	0.114449	1.082562	up	541.8465
up	-1.0182	-0.02602	1.018201	down	-1.00706	-0.01014	1.007055	down	81.89742
down	-1.1542	-0.20689	1.154197	down	-1.12133	-0.16521	1.121329	down	413.4538
up	1.161863	0.216441	1.161863	up	1.067799	0.094641	1.067799	up	53.34436
up	-1.00359	-0.00518	1.003594	down	-1.01581	-0.02263	1.015807	down	1342.289
down	-1.18952	-0.25038	1.189521	down	-1.18008	-0.23888	1.180076	down	138.0652
down	-1.07435	-0.10346	1.074349	down	-1.1477	-0.19874	1.147697	down	203.0011
up	-1.07132	-0.0994	1.071325	down	-1.15834	-0.21205	1.158335	down	283.8189
down	-1.05385	-0.07566	1.053845	down	-1.02502	-0.03565	1.025016	down	32.42041
down	-1.26023	-0.33368	1.260225	down	-1.24644	-0.31782	1.246442	down	23.70123
down	-1.08697	-0.12031	1.08697	down	-1.11236	-0.15362	1.112358	down	1069.019
down	1.008377	0.012035	1.008377	up	-1.00004	-5.33E-05	1.000037	down	33.37072
down	1.210924	0.276108	1.210924	up	1.223218	0.290681	1.223218	up	19.38634
down	1.224177	0.291812	1.224177	up	1.164905	0.220213	1.164905	up	21.43288
up	-1.07596	-0.10562	1.075957	down	1.098838	0.135979	1.098838	up	27.87509
down	1.056763	0.079652	1.056763	up	-1.00606	-0.00872	1.006059	down	63.43875
down	-1.17275	-0.22989	1.172745	down	-1.04119	-0.05823	1.041188	down	38.29248
down	-1.01084	-0.01555	1.010838	down	1.090433	0.124901	1.090433	up	23.15253
down	1.0909	0.125519	1.0909	up	1.060829	0.085192	1.060829	up	19.59288
up	-1.31586	-0.396	1.315858	down	-1.21468	-0.28058	1.214681	down	115.121
down	1.187888	0.248398	1.187888	up	-1.06486	-0.09066	1.064857	down	120.8417
up	-1.18583	-0.2459	1.185829	down	-1.21994	-0.28681	1.219938	down	135.5918
up	-1.10467	-0.14361	1.104667	down	-1.07348	-0.1023	1.073483	down	54.02037
up	1.036906	0.052285	1.036906	up	1.038236	0.054135	1.038236	up	615.623
down	-1.1937	-0.25544	1.193699	down	-1.08481	-0.11744	1.084807	down	88.01992
down	1.108709	0.14888	1.108709	up	1.098015	0.134898	1.098015	up	42.28245
up	-1.23364	-0.30292	1.233643	down	-1.15077	-0.2026	1.150771	down	24.68682
down	1.161294	0.215733	1.161294	up	1.178555	0.237019	1.178555	up	17.50441
up	1.075217	0.104628	1.075217	up	1.034098	0.048373	1.034098	up	17.43168
down	1.061241	0.085753	1.061241	up	1.042249	0.0597	1.042249	up	21.75233
up	-1.10585	-0.14516	1.105855	down	1.075792	0.105399	1.075792	up	51.53552
down	-1.09157	-0.1264	1.091569	down	-1.0536	-0.07532	1.053596	down	24.07215
up	-1.01314	-0.01883	1.013135	down	-1.09139	-0.12616	1.091387	down	26.82999
down	1.166392	0.222052	1.166392	up	1.210432	0.275522	1.210432	up	195.1506
up	1.065084	0.090967	1.065084	up	1.032994	0.046832	1.032994	up	40.56531
down	1.001022	0.001473	1.001022	up	1.019356	0.027658	1.019356	up	46.36647
up	-1.06954	-0.097	1.069544	down	-1.0254	-0.03618	1.025396	down	443.5115
down	1.193481	0.255176	1.193481	up	-1.13713	-0.1854	1.137131	down	106.7554
up	1.111086	0.15197	1.111086	up	1.144698	0.194966	1.144698	up	90.34888
down	1.150595	0.20238	1.150595	up	1.131564	0.178318	1.131564	up	25.77795
down	-1.14471	-0.19498	1.144712	down	-1.12402	-0.16866	1.124017	down	59.69233
down	-1.2598	-0.33319	1.259799	down	-1.31613	-0.3963	1.31613	down	163.6126
down	1.052159	0.073352	1.052159	up	-1.15858	-0.21236	1.158583	down	26.34048
up	1.074374	0.103496	1.074374	up	-1.10521	-0.14432	1.105206	down	38.83992
down	1.020636	0.029468	1.020636	up	-1.02638	-0.03757	1.026385	down	24.47535

up	1.22688	0.294994	1.22688	up	1.06195	0.086716	1.06195	up	58.82492
down	-1.04228	-0.05975	1.042282	down	1.000254	3.66E-04	1.000254	up	73.93615
down	-1.02897	-0.0412	1.028966	down	-1.27233	-0.34747	1.272331	down	371.4842
down	-1.00318	-0.00458	1.003179	down	-1.01773	-0.02536	1.017731	down	28.27593
up	-1.01687	-0.02414	1.016874	down	-1.01737	-0.02484	1.017367	down	82.2309
down	1.042786	0.060443	1.042786	up	-1.14764	-0.19867	1.14764	down	18.60085
down	-1.06763	-0.09441	1.067629	down	-1.13416	-0.18162	1.13416	down	48.45247
up	-1.50683	-0.59152	1.506832	down	-1.34537	-0.428	1.345366	down	889.319
down	1.108942	0.149184	1.108942	up	1.136726	0.184884	1.136726	up	83.62369
up	-1.02331	-0.03325	1.023311	down	-1.2065	-0.27082	1.206497	down	100.963
up	-1.37921	-0.46384	1.379208	down	-1.07372	-0.10262	1.073722	down	50.06434
up	1.048188	0.067898	1.048188	up	-1.088	-0.12167	1.087997	down	276.8677
up	-1.2897	-0.36703	1.289697	down	-1.34807	-0.43089	1.348068	down	26.01752
down	-1.041	-0.05798	1.041004	down	1.057302	0.080388	1.057302	up	32.10883
up	1.082934	0.114945	1.082934	up	1.093684	0.129196	1.093684	up	20.94887
down	1.421408	0.507321	1.421408	up	1.172503	0.229592	1.172503	up	102.6073
up	-1.09771	-0.1345	1.097712	down	-1.0803	-0.11144	1.080303	down	30.0931
down	-1.13839	-0.18699	1.138387	down	1.030088	0.042768	1.030088	up	55.0663
up	1.161387	0.215849	1.161387	up	-1.10685	-0.14646	1.106853	down	79.43367
down	-1.0003	-4.26E-04	1.000295	down	1.082454	0.114306	1.082454	up	42.75005
down	-1.02278	-0.0325	1.022782	down	-1.12293	-0.16726	1.122927	down	48.21715
down	-1.13125	-0.17792	1.131251	down	-1.1385	-0.18713	1.138499	down	29.6373
down	1.197344	0.259837	1.197344	up	1.097334	0.134003	1.097334	up	27.80984
down	1.052917	0.074392	1.052917	up	1.093637	0.129134	1.093637	up	20.31389
up	-1.20837	-0.27306	1.20837	down	-1.14491	-0.19524	1.144914	down	17.99857
down	-1.19456	-0.25648	1.194557	down	-1.18002	-0.23881	1.180019	down	19.00935
down	1.21083	0.275996	1.21083	up	-1.00067	-9.69E-04	1.000672	down	36.74838
down	-1.07742	-0.10758	1.077416	down	1.075913	0.105561	1.075913	up	19.84314
up	1.029566	0.042037	1.029566	up	1.231828	0.300801	1.231828	up	36.70393
up	-1.00447	-0.00644	1.004475	down	1.110552	0.151278	1.110552	up	48.52913
up	-1.09976	-0.13719	1.099759	down	-1.10113	-0.13898	1.101127	down	29.07417
up	1.064297	0.0899	1.064297	up	-1.00425	-0.00611	1.004245	down	26.06588
up	-1.05432	-0.07632	1.054323	down	-1.06667	-0.09312	1.066674	down	109.5562
down	-1.00967	-0.01389	1.009674	down	1.045808	0.064619	1.045808	up	30.39124
down	1.177725	0.236003	1.177725	up	1.250432	0.322426	1.250432	up	31.57143
down	1.048966	0.068967	1.048966	up	-1.17002	-0.22653	1.170016	down	24.88104
down	1.190297	0.251321	1.190297	up	1.025377	0.036155	1.025377	up	37.27019
down	-1.1066	-0.14614	1.106603	down	-1.003	-0.00431	1.002995	down	41.56013
down	1.064508	0.090187	1.064508	up	1.05388	0.075711	1.05388	up	19.195
down	1.12402	0.168667	1.12402	up	1.128186	0.174005	1.128186	up	43.73709
down	-1.05847	-0.08198	1.058471	down	1.000688	9.93E-04	1.000688	up	24.26139
down	1.136794	0.184971	1.136794	up	1.031194	0.044316	1.031194	up	24.38004
down	1.054023	0.075906	1.054023	up	1.119608	0.162994	1.119608	up	30.98245
down	-1.17725	-0.23543	1.177255	down	-1.15959	-0.21362	1.159595	down	18.81603
down	-1.08103	-0.11241	1.081029	down	-1.03489	-0.04947	1.034888	down	32.52582
down	-1.05104	-0.07182	1.051038	down	-1.03752	-0.05313	1.037517	down	31.51534
down	-1.11655	-0.15905	1.116551	down	-1.00385	-0.00554	1.003846	down	39.6883

down	1.076178	0.105917	1.076178	up	-1.03801	-0.05383	1.038013	down	19.69562
up	-1.19347	-0.25516	1.193466	down	-1.33561	-0.4175	1.335608	down	32.82955
down	1.037569	0.053208	1.037569	up	-1.11339	-0.15496	1.113392	down	29.85723
down	-1.00182	-0.00262	1.001816	down	-1.06114	-0.08562	1.061144	down	41.16457
up	-1.12292	-0.16725	1.122919	down	-1.07187	-0.10013	1.071868	down	218.2443
up	1.009534	0.013689	1.009534	up	-1.00528	-0.0076	1.00528	down	1205.821
down	1.080745	0.112026	1.080745	up	1.132633	0.17968	1.132633	up	1680.138
up	1.044734	0.063136	1.044734	up	-1.04224	-0.05969	1.042243	down	219.3245
down	1.513872	0.598243	1.513872	up	1.346234	0.428929	1.346234	up	126.7432
down	-1.00357	-0.00513	1.003565	down	-1.13653	-0.18464	1.136532	down	165.021
up	1.098197	0.135137	1.098197	up	1.073144	0.101843	1.073144	up	228.3895
up	1.059893	0.083918	1.059893	up	-1.00054	-7.77E-04	1.000539	down	189.9458
up	-1.14291	-0.19271	1.142907	down	-1.28645	-0.36339	1.286448	down	382.7113
up	-1.32397	-0.40487	1.323966	down	-1.15225	-0.20445	1.152247	down	787.2087
down	1.350151	0.43312	1.350151	up	1.368685	0.45279	1.368685	up	33.90033
down	1.155558	0.208589	1.155558	up	1.13526	0.183022	1.13526	up	162.578
up	-1.00222	-0.00321	1.002224	down	-1.16023	-0.21441	1.160227	down	203.4741
up	1.016799	0.024034	1.016799	up	1.038503	0.054506	1.038503	up	491.7886
up	-1.16334	-0.21827	1.163336	down	-1.05449	-0.07654	1.054489	down	770.0749
up	1.343844	0.426366	1.343844	up	1.215018	0.280977	1.215018	up	762.7065
up	1.44183	0.527901	1.44183	up	1.289986	0.367355	1.289986	up	86.47382
up	-1.06995	-0.09754	1.06995	down	1.004736	0.006816	1.004736	up	144.5381
down	1.0015	0.002163	1.0015	up	1.086746	0.120014	1.086746	up	1101.328
down	1.145489	0.195963	1.145489	up	1.151351	0.203328	1.151351	up	229.7591
up	-1.08195	-0.11364	1.081951	down	-1.11995	-0.16343	1.119948	down	81.24844
up	-1.27684	-0.35258	1.276841	down	-1.05742	-0.08055	1.057422	down	94.37268
up	-1.0831	-0.11516	1.083096	down	1.039822	0.056336	1.039822	up	275.2184
up	-1.11521	-0.15732	1.115212	down	-1.08657	-0.11978	1.086566	down	960.9487
up	-1.30978	-0.38932	1.309778	down	-1.01331	-0.01907	1.013309	down	419.5172
up	1.297463	0.375694	1.297463	up	1.019334	0.027626	1.019334	up	237.562
up	1.171427	0.228267	1.171427	up	-1.06078	-0.08512	1.060778	down	993.9943
up	1.110697	0.151466	1.110697	up	1.037822	0.053559	1.037822	up	808.0189
up	-1.01036	-0.01487	1.01036	down	-1.07527	-0.10469	1.075266	down	386.9226
up	-1.09128	-0.12602	1.091283	down	-1.0854	-0.11823	1.0854	down	765.625
up	1.056264	0.07897	1.056264	up	1.230691	0.299468	1.230691	up	481.4241
down	1.180882	0.239865	1.180882	up	-1.03712	-0.05259	1.037125	down	45.48573
down	1.000004	5.25E-06	1.000004	up	1.021395	0.030541	1.021395	up	159.6705
down	-1.05343	-0.0751	1.053431	down	-1.08348	-0.11568	1.083485	down	278.7245
up	-1.19512	-0.25716	1.195122	down	-1.17416	-0.23163	1.174159	down	37.34211
up	-1.10739	-0.14716	1.107385	down	-1.11402	-0.15577	1.114015	down	172.5638
up	1.303966	0.382907	1.303966	up	1.118427	0.161471	1.118427	up	905.6787
up	1.073084	0.101763	1.073084	up	1.083054	0.115105	1.083054	up	269.072
up	-1.04878	-0.06872	1.048784	down	-1.01326	-0.019	1.01326	down	476.7033
up	1.01308	0.018748	1.01308	up	1.085667	0.118581	1.085667	up	528.0057
down	-1.25749	-0.33055	1.257492	down	1.028019	0.039866	1.028019	up	217.5303
up	-1.0084	-0.01206	1.008395	down	1.040942	0.05789	1.040942	up	58.84943
up	1.025862	0.036836	1.025862	up	1.084997	0.11769	1.084997	up	234.583

up	1.114519	0.156422	1.114519	up	1.132177	0.1791	1.132177	up	189.3245
up	-1.13102	-0.17762	1.131017	down	1.014687	0.021035	1.014687	up	132.457
down	1.039677	0.056136	1.039677	up	1.054516	0.076581	1.054516	up	160.445
down	-1.07085	-0.09876	1.070851	down	-1.11758	-0.16038	1.117583	down	291.5378
down	1.009345	0.01342	1.009345	up	-1.16068	-0.21497	1.160681	down	32.14162
down	-1.03236	-0.04595	1.032362	down	-1.042	-0.05935	1.041996	down	441.8257
up	1.026977	0.038404	1.026977	up	-1.04404	-0.06218	1.044042	down	1112.742
down	-1.04246	-0.05999	1.04246	down	-1.05597	-0.07858	1.055975	down	43.09892
up	-1.05246	-0.07377	1.05246	down	-1.11072	-0.15149	1.11072	down	359.726
down	-1.10754	-0.14735	1.107537	down	-1.08712	-0.12051	1.087116	down	582.6735
down	1.031184	0.044301	1.031184	up	1.012437	0.017832	1.012437	up	118.422
up	1.196838	0.259228	1.196838	up	-1.02705	-0.0385	1.027047	down	341.4485
up	-1.11737	-0.16011	1.117371	down	1.048115	0.067796	1.048115	up	175.9036
up	-1.32409	-0.405	1.32409	down	-1.27637	-0.35205	1.27637	down	298.1863
up	-1.08197	-0.11366	1.081971	down	-1.19937	-0.26227	1.199368	down	247.6711
up	1.05982	0.08382	1.05982	up	1.0926	0.127765	1.0926	up	600.3366
down	-1.01953	-0.0279	1.01953	down	-1.07291	-0.10153	1.072913	down	211.4127
down	1.121476	0.165398	1.121476	up	1.041166	0.0582	1.041166	up	536.7015
up	-1.15282	-0.20517	1.152825	down	-1.11295	-0.15439	1.112952	down	144.8326
up	-1.06579	-0.09192	1.065787	down	-1.04574	-0.06453	1.045744	down	599.4239
down	1.162489	0.217217	1.162489	up	-1.04971	-0.06999	1.049708	down	515.7899
up	-1.30667	-0.3859	1.306671	down	-1.31559	-0.39571	1.315586	down	104.881
down	-1.23994	-0.31027	1.239936	down	-1.08197	-0.11366	1.081969	down	397.3594
up	1.196156	0.258405	1.196156	up	1.025736	0.036659	1.025736	up	84.21326
up	1.004029	0.005801	1.004029	up	1.144982	0.195325	1.144982	up	281.4276
up	1.116303	0.158729	1.116303	up	-1.00669	-0.00962	1.006689	down	345.8871
up	-1.07233	-0.10075	1.072332	down	-1.04497	-0.06346	1.044966	down	750.471
down	1.096663	0.13312	1.096663	up	1.102153	0.140325	1.102153	up	2039.972
down	-1.21147	-0.27675	1.211467	down	-1.048	-0.06764	1.048	down	148.7607
up	1.06544	0.09145	1.06544	up	-1.10309	-0.14155	1.103092	down	268.8122
up	-1.19637	-0.25866	1.196367	down	-1.08043	-0.1116	1.080428	down	55.6218
up	-1.2014	-0.26472	1.201403	down	-1.06919	-0.09652	1.069189	down	134.1679
down	-1.00347	-0.005	1.003471	down	-1.05294	-0.07442	1.05294	down	584.7347
down	-1.03226	-0.04581	1.032261	down	1.0187	0.026729	1.0187	up	1177.312
up	1.004055	0.005838	1.004055	up	1.031025	0.04408	1.031025	up	276.9576
down	1.039123	0.055367	1.039123	up	1.18697	0.247283	1.18697	up	647.1476
down	-1.07315	-0.10185	1.073146	down	-1.05166	-0.07267	1.051659	down	1462.301
down	-1.03277	-0.04652	1.032774	down	-1.07601	-0.1057	1.076013	down	55.54233
up	-1.09632	-0.13266	1.096315	down	-1.17113	-0.2279	1.17113	down	401.3273
up	1.418958	0.504832	1.418958	up	1.539874	0.622812	1.539874	up	262.97
down	-1.17413	-0.23159	1.17413	down	-1.12731	-0.17288	1.127306	down	35.25973
up	-1.38705	-0.47202	1.387047	down	-1.2027	-0.26628	1.202703	down	702.3489
up	-1.37539	-0.45984	1.375392	down	-1.23574	-0.30537	1.235738	down	263.6232
up	-1.27237	-0.34752	1.272373	down	-1.1452	-0.1956	1.1452	down	128.327
up	-1.23058	-0.29934	1.230581	down	-1.01558	-0.0223	1.015576	down	159.3318
down	1.336438	0.418393	1.336438	up	1.242211	0.31291	1.242211	up	243.037
up	1.077072	0.107115	1.077072	up	1.086692	0.119943	1.086692	up	141.4312

down	-1.01276	-0.01829	1.012761	down	1.035752	0.050679	1.035752	up	123.229
up	1.023375	0.033334	1.023375	up	-1.02216	-0.03162	1.022163	down	71.02048
down	-1.09505	-0.13099	1.095047	down	1.042822	0.060493	1.042822	up	79.5859
up	1.262222	0.335966	1.262222	up	1.301867	0.380582	1.301867	up	99.75948
up	1.25615	0.329009	1.25615	up	1.339834	0.422054	1.339834	up	308.9968
up	1.061351	0.085901	1.061351	up	-1.04355	-0.0615	1.043554	down	50.37427
up	-1.55485	-0.63677	1.554847	down	-1.42166	-0.50758	1.421662	down	42.90646
down	-1.13008	-0.17642	1.130078	down	1.10541	0.144582	1.10541	up	23.27156
down	1.079008	0.109705	1.079008	up	-1.01231	-0.01765	1.012312	down	244.5138
down	1.183608	0.243191	1.183608	up	-1.17869	-0.23718	1.178688	down	256.1606
down	-1.06053	-0.08478	1.060528	down	-1.14273	-0.19249	1.142733	down	279.5878
up	1.158337	0.212055	1.158337	up	1.165171	0.220542	1.165171	up	203.5734
down	-1.08247	-0.11433	1.082474	down	-1.07389	-0.10284	1.073887	down	107.8136
down	-1.21982	-0.28667	1.219823	down	-1.08031	-0.11144	1.080307	down	135.3084
down	-1.29378	-0.37159	1.293779	down	-1.23919	-0.3094	1.239188	down	42.40541
up	-1.17484	-0.23247	1.174843	down	-1.05297	-0.07447	1.052972	down	3444.542
down	-1.03757	-0.05321	1.037572	down	-1.13487	-0.18253	1.134874	down	162.1559
up	1.133298	0.180527	1.133298	up	1.027476	0.039105	1.027476	up	104.4497
down	1.166885	0.222663	1.166885	up	1.099646	0.137039	1.099646	up	128.3457
up	1.120824	0.16456	1.120824	up	1.096587	0.13302	1.096587	up	234.8748
up	-1.29512	-0.37309	1.295119	down	-1.27529	-0.35083	1.275292	down	164.1952
up	-1.20862	-0.27336	1.20862	down	-1.12061	-0.16428	1.12061	down	20.08505
down	1.213438	0.2791	1.213438	up	1.125567	0.170652	1.125567	up	53.14179
up	-1.27674	-0.35247	1.276744	down	-1.20852	-0.27325	1.208525	down	257.9551
up	-1.3332	-0.41489	1.3332	down	-1.3046	-0.38361	1.304599	down	17.55845
down	-1.03184	-0.04522	1.031843	down	1.018688	0.026713	1.018688	up	107.4751
down	-1.09737	-0.13405	1.097372	down	-1.14149	-0.19092	1.141492	down	122.3759
down	-1.15806	-0.2117	1.158055	down	-1.06555	-0.09159	1.065547	down	133.1645
up	1.171895	0.228844	1.171895	up	-1.07243	-0.10089	1.072433	down	39.96498
up	-1.26407	-0.33808	1.264069	down	-1.22775	-0.29602	1.227755	down	178.5828
down	1.195573	0.257702	1.195573	up	-1.14746	-0.19844	1.147461	down	120.0982
down	-1.48161	-0.56716	1.481607	down	-1.15997	-0.21408	1.159966	down	19.21606
up	1.065275	0.091226	1.065275	up	1.114999	0.157043	1.114999	up	127.0771
up	1.093396	0.128816	1.093396	up	1.047007	0.066271	1.047007	up	180.6053
down	-1.04187	-0.05917	1.04187	down	1.027033	0.038482	1.027033	up	32.36129
down	1.122252	0.166397	1.122252	up	-1.00617	-0.00888	1.006173	down	51.40341
down	1.209352	0.274234	1.209352	up	-1.26763	-0.34213	1.267626	down	666.885
up	-1.65186	-0.72409	1.651863	down	-1.21262	-0.27813	1.212624	down	38.48028
down	-1.13564	-0.1835	1.135638	down	-1.09949	-0.13683	1.099488	down	237.7019
up	-1.15937	-0.21335	1.159375	down	-1.10047	-0.13812	1.100473	down	88.1712
down	-1.02191	-0.03127	1.021913	down	1.011879	0.017037	1.011879	up	247.6434
up	1.119358	0.162672	1.119358	up	-1.08456	-0.11711	1.084563	down	140.9633
up	1.0587	0.082294	1.0587	up	1.110918	0.151752	1.110918	up	314.5498
down	-1.01053	-0.01511	1.01053	down	1.123671	0.16822	1.123671	up	85.24683
up	-1.00849	-0.0122	1.00849	down	1.107344	0.147103	1.107344	up	86.00067
up	1.317903	0.398245	1.317903	up	1.256405	0.329302	1.256405	up	123.6991
up	1.346859	0.429599	1.346859	up	1.14833	0.199538	1.14833	up	33.3701

down	1.34091	0.423213	1.34091	up	-1.09754	-0.13428	1.097543	down	77.52999
up	1.033537	0.04759	1.033537	up	1.08512	0.117854	1.08512	up	321.46
down	1.07267	0.101206	1.07267	up	1.194068	0.255885	1.194068	up	118.275
up	1.097406	0.134097	1.097406	up	-1.0288	-0.04096	1.028799	down	5692.655
down	1.400156	0.485588	1.400156	up	1.338429	0.420541	1.338429	up	317.7454
up	1.302451	0.381229	1.302451	up	1.362225	0.445965	1.362225	up	46.81404
down	1.009482	0.013615	1.009482	up	-1.00965	-0.01386	1.009654	down	1569.695
down	1.056966	0.079928	1.056966	up	-1.01048	-0.01504	1.010476	down	306.5859
down	1.027674	0.039382	1.027674	up	1.09542	0.131484	1.09542	up	57.09835
down	-1.08587	-0.11885	1.085872	down	-1.12821	-0.17404	1.128212	down	64.56933
up	-1.20482	-0.26882	1.204819	down	-1.2875	-0.36457	1.287496	down	63.73836
down	1.193152	0.254778	1.193152	up	1.136299	0.184343	1.136299	up	297.3548
up	-1.19894	-0.26176	1.198942	down	-1.12768	-0.17336	1.127678	down	84.49345
down	1.731015	0.791619	1.731015	up	1.361305	0.44499	1.361305	up	40.78437
up	1.055275	0.077619	1.055275	up	1.110703	0.151473	1.110703	up	135.5865
up	-1.00007	-9.93E-05	1.000069	down	1.12451	0.169296	1.12451	up	179.1692
down	-1.11717	-0.15985	1.117169	down	-1.11583	-0.15812	1.115832	down	273.2089
up	1.17487	0.232501	1.17487	up	1.251014	0.323098	1.251014	up	71.32055
down	1.049677	0.069946	1.049677	up	-1.0729	-0.10151	1.072897	down	408.1786
down	1.020087	0.028692	1.020087	up	1.127196	0.172739	1.127196	up	87.65987
down	1.105795	0.145084	1.105795	up	-1.02954	-0.04201	1.029544	down	48.77538
down	-1.16406	-0.21917	1.164065	down	-1.10962	-0.15007	1.109622	down	38.88614
up	-1.02555	-0.03639	1.025547	down	-1.04838	-0.06816	1.048376	down	62.96196
down	1.068731	0.095898	1.068731	up	-1.00314	-0.00452	1.003136	down	374.9557
down	1.107871	0.147789	1.107871	up	1.025279	0.036016	1.025279	up	71.7678
up	1.068924	0.096159	1.068924	up	-1.08876	-0.12269	1.08876	down	44.20447
down	-1.00389	-0.0056	1.003892	down	1.074092	0.103118	1.074092	up	44.06398
down	-1.19722	-0.25969	1.197219	down	1.101173	0.139042	1.101173	up	42.29593
up	-1.12683	-0.17226	1.126826	down	-1.08773	-0.12132	1.087727	down	49.31018
up	-1.29073	-0.36819	1.290728	down	-1.15276	-0.20509	1.15276	down	97.111
up	1.211396	0.276671	1.211396	up	1.019509	0.027874	1.019509	up	542.0873
down	1.175296	0.233024	1.175296	up	1.122216	0.16635	1.122216	up	23.65927
up	-1.09083	-0.12543	1.09083	down	-1.15434	-0.20707	1.154339	down	42.049
up	-1.06448	-0.09015	1.064482	down	-1.13268	-0.17974	1.132684	down	28.8716
up	1.170753	0.227436	1.170753	up	1.097021	0.133591	1.097021	up	53.35535
up	1.016527	0.023649	1.016527	up	1.018046	0.025802	1.018046	up	58.02479
up	-1.05023	-0.07071	1.050233	down	1.025482	0.036302	1.025482	up	262.2819
down	1.045056	0.063581	1.045056	up	1.051054	0.071837	1.051054	up	68.23973
up	-1.04188	-0.05918	1.041876	down	1.062793	0.087861	1.062793	up	20.75973
down	1.223315	0.290796	1.223315	up	1.060687	0.084998	1.060687	up	324.4004
down	1.089804	0.124069	1.089804	up	1.055967	0.078565	1.055967	up	79.24807
down	-1.14246	-0.19214	1.142457	down	-1.00557	-0.00802	1.005574	down	112.6098
up	-1.00301	-0.00433	1.003009	down	1.092171	0.127198	1.092171	up	194.265
down	1.111219	0.152142	1.111219	up	1.000497	7.17E-04	1.000497	up	19.31617
down	1.016892	0.024166	1.016892	up	-1.09067	-0.12522	1.090671	down	93.41312
down	-1.05907	-0.0828	1.059075	down	-1.19349	-0.25519	1.193491	down	43.52124
down	1.05893	0.082607	1.05893	up	-1.33245	-0.41408	1.332446	down	3654.476

up	-1.04303	-0.06078	1.043032	down	-1.2905	-0.36793	1.290501	down	24.2597
down	1.166885	0.222662	1.166885	up	1.27734	0.353142	1.27734	up	38.02074
down	-1.03826	-0.05417	1.038264	down	1.070153	0.097817	1.070153	up	23.62235
up	-1.05982	-0.08382	1.059818	down	1.056649	0.079496	1.056649	up	439.3612
up	1.043931	0.062027	1.043931	up	1.004179	0.006016	1.004179	up	145.5921
up	-1.13591	-0.18385	1.135908	down	-1.24382	-0.31477	1.243815	down	119.8708
down	1.131101	0.177727	1.131101	up	1.188427	0.249053	1.188427	up	156.5924
down	-1.06043	-0.08465	1.060433	down	-1.10493	-0.14396	1.104932	down	404.2665
down	1.03564	0.050523	1.03564	up	1.124834	0.169712	1.124834	up	31.98113
down	1.089815	0.124083	1.089815	up	1.036977	0.052385	1.036977	up	32.25834
down	-1.15156	-0.20359	1.151564	down	-1.01027	-0.01474	1.010269	down	46.43497
up	1.265233	0.339403	1.265233	up	1.331611	0.413172	1.331611	up	33.14014
down	-1.00634	-0.00912	1.006344	down	1.00485	0.00698	1.00485	up	128.6187
down	1.408803	0.49447	1.408803	up	1.339341	0.421523	1.339341	up	41.59031
down	-1.02682	-0.03819	1.026822	down	1.129446	0.175615	1.129446	up	45.46496
up	1.060083	0.084178	1.060083	up	1.090957	0.125594	1.090957	up	33.27142
up	-1.10616	-0.14556	1.106159	down	-1.04407	-0.06221	1.044066	down	18.53069
down	1.164518	0.219733	1.164518	up	1.371712	0.455977	1.371712	up	411.0155
up	1.007286	0.010473	1.007286	up	1.065239	0.091177	1.065239	up	227.6625
down	1.036416	0.051604	1.036416	up	-1.01566	-0.02242	1.015662	down	71.35703
up	-1.08768	-0.12125	1.087675	down	-1.05318	-0.07475	1.053181	down	130.3671
up	1.101576	0.139569	1.101576	up	1.001952	0.002814	1.001952	up	189.0614
down	-1.06591	-0.09209	1.065911	down	-1.083	-0.11503	1.082999	down	138.0654
down	-1.02813	-0.04002	1.028127	down	1.032468	0.046097	1.032468	up	144.6973
down	-1.2481	-0.31973	1.248098	down	-1.19037	-0.25141	1.190374	down	191.928
down	-1.07016	-0.09783	1.070163	down	1.021053	0.030058	1.021053	up	221.0963
up	-1.02741	-0.03902	1.027413	down	-1.00873	-0.01254	1.008731	down	168.3227
down	1.128504	0.174412	1.128504	up	-1.08364	-0.11589	1.083641	down	27.09291
down	-1.04162	-0.05883	1.041619	down	1.100019	0.137529	1.100019	up	29.56595
down	1.3358	0.417704	1.3358	up	1.016767	0.023989	1.016767	up	26.02754
down	-1.17629	-0.23425	1.176292	down	1.079517	0.110386	1.079517	up	67.28014
up	1.036868	0.052232	1.036868	up	-1.07624	-0.10599	1.076236	down	41.98739
down	-1.03476	-0.0493	1.034764	down	1.013958	0.019997	1.013958	up	78.37821
up	-1.01095	-0.01571	1.010948	down	1.004224	0.00608	1.004224	up	75.24905
down	1.109408	0.14979	1.109408	up	1.053309	0.074929	1.053309	up	47.0674
down	-1.04767	-0.06719	1.047673	down	-1.02124	-0.03033	1.021243	down	70.80136
up	-1.19209	-0.25349	1.192086	down	-1.17747	-0.23569	1.177468	down	17.79749
down	-1.03774	-0.05345	1.03774	down	-1.27824	-0.35416	1.278242	down	70.07725
down	1.188011	0.248548	1.188011	up	1.092649	0.12783	1.092649	up	46.39079
down	-1.04597	-0.06485	1.045975	down	-1.04353	-0.06148	1.043533	down	44.42034
up	-1.5854	-0.66485	1.585403	down	-1.19791	-0.26051	1.197906	down	67.3726
down	1.110622	0.151367	1.110622	up	1.080094	0.111156	1.080094	up	27.91868
up	-1.02912	-0.04142	1.029123	down	-1.27574	-0.35134	1.275743	down	171.6667
down	1.024557	0.035	1.024557	up	1.024139	0.034411	1.024139	up	126.6307
up	-1.14541	-0.19587	1.145412	down	1.056534	0.07934	1.056534	up	37.10574
up	-1.03563	-0.05051	1.035632	down	-1.20681	-0.2712	1.206808	down	21.77942
down	1.028359	0.040344	1.028359	up	-1.01634	-0.02338	1.016337	down	21.50551

down	-1.0001	-1.44E-04	1.0001	down	1.024419	0.034806	1.024419	up	112.7417
down	-1.10565	-0.14489	1.105648	down	-1.0834	-0.11556	1.083397	down	33.2011
down	-1.10785	-0.14776	1.107851	down	-1.07057	-0.09838	1.070567	down	25.53593
down	-1.09178	-0.12668	1.091776	down	-1.14727	-0.1982	1.147269	down	20.07294
up	-1.01305	-0.01871	1.013055	down	1.127071	0.172579	1.127071	up	464.4911
down	-1.00074	-0.00107	1.000741	down	-1.05659	-0.07942	1.056595	down	19.32418
down	1.241337	0.311895	1.241337	up	1.039013	0.055214	1.039013	up	29.72578
up	1.119364	0.16268	1.119364	up	-1.09162	-0.12647	1.091621	down	16.27263
up	1.037074	0.052519	1.037074	up	-1.02734	-0.03892	1.027341	down	19.50327
down	1.366269	0.450241	1.366269	up	-1.0294	-0.04181	1.029402	down	47.00266
up	1.228055	0.296375	1.228055	up	-1.12978	-0.17604	1.129777	down	31.99305
down	-1.09228	-0.12735	1.092282	down	-1.0977	-0.13448	1.097698	down	154.201
down	-1.09862	-0.13569	1.098617	down	1.005688	0.008183	1.005688	up	254.1956
down	1.060831	0.085195	1.060831	up	1.162898	0.217724	1.162898	up	22.14784
up	1.095611	0.131735	1.095611	up	1.073076	0.101752	1.073076	up	60.47384
down	-1.0601	-0.0842	1.060102	down	1.000996	0.001436	1.000996	up	29.40772
down	-1.07914	-0.10988	1.079141	down	-1.04772	-0.06725	1.047716	down	52.30086
down	1.109137	0.149437	1.109137	up	1.036016	0.051046	1.036016	up	122.129
up	1.103543	0.142143	1.103543	up	1.093871	0.129443	1.093871	up	315.7726
up	-1.16125	-0.21568	1.161255	down	1.043246	0.06108	1.043246	up	150.7056
down	-1.0554	-0.07779	1.055399	down	-1.01711	-0.02448	1.017115	down	132.7015
down	1.119633	0.163026	1.119633	up	1.130011	0.176337	1.130011	up	44.49147
up	-1.3351	-0.41695	1.335101	down	-1.1259	-0.17107	1.125897	down	129.4631
up	1.034758	0.049293	1.034758	up	-1.05841	-0.0819	1.058414	down	18.33695
down	1.307037	0.3863	1.307037	up	1.206637	0.270991	1.206637	up	59.11035
down	1.140796	0.19004	1.140796	up	1.061892	0.086637	1.061892	up	35.04626
down	1.078269	0.108717	1.078269	up	1.001884	0.002715	1.001884	up	20.88578
up	1.352622	0.435759	1.352622	up	1.075654	0.105215	1.075654	up	28.30026
up	-1.47112	-0.55691	1.471118	down	-1.21926	-0.286	1.219256	down	32.54312
down	1.16079	0.215107	1.16079	up	1.082793	0.114758	1.082793	up	30.57228
up	-1.14038	-0.18952	1.140382	down	1.011297	0.016207	1.011297	up	37.91983
up	-1.26545	-0.33965	1.265448	down	-1.24127	-0.31182	1.241271	down	82.2344
up	-1.07029	-0.098	1.07029	down	-1.04309	-0.06087	1.043094	down	19.82699
up	-1.04143	-0.05856	1.041426	down	-1.15223	-0.20443	1.152234	down	27.14655
down	-1.01587	-0.02271	1.015869	down	-1.01115	-0.016	1.01115	down	44.43637
up	-1.21505	-0.28101	1.215046	down	-1.29977	-0.37826	1.299772	down	16.4169
up	1.037798	0.053525	1.037798	up	-1.11572	-0.15797	1.115719	down	23.99525
down	-1.17811	-0.23648	1.178112	down	-1.05045	-0.07101	1.050452	down	34.49675
down	1.050982	0.071737	1.050982	up	-1.09214	-0.12716	1.092139	down	31.95937
up	-1.85945	-0.89488	1.85945	down	-1.22273	-0.29011	1.222732	down	70.27911
down	1.649329	0.721879	1.649329	up	1.55789	0.639594	1.55789	up	2488.843
down	1.399177	0.484578	1.399177	up	1.513462	0.597852	1.513462	up	1768.289
down	1.071613	0.099784	1.071613	up	1.011837	0.016977	1.011837	up	457.49
down	-1.03373	-0.04786	1.033727	down	1.048242	0.067972	1.048242	up	1015.336
down	1.094815	0.130687	1.094815	up	-1.05453	-0.0766	1.05453	down	180.8269
up	-1.23843	-0.30851	1.238429	down	-1.34235	-0.42476	1.342353	down	391.5656
up	-1.19233	-0.25378	1.192326	down	-1.1189	-0.16208	1.118896	down	90.05883

down	1.046035	0.064931	1.046035	up	1.146687	0.197471	1.146687	up	28.38289
down	-1.13888	-0.18761	1.138879	down	1.008535	0.012261	1.008535	up	50.24976
down	1.170443	0.227055	1.170443	up	-1.03615	-0.05123	1.036151	down	68.59194
down	-1.07627	-0.10604	1.076269	down	-1.01509	-0.02161	1.015092	down	35.54452
up	1.030798	0.043762	1.030798	up	1.049552	0.069774	1.049552	up	24.23666
down	-1.08887	-0.12284	1.088874	down	-1.07445	-0.10359	1.074447	down	18.18325
down	1.090445	0.124917	1.090445	up	1.068349	0.095383	1.068349	up	101.1761
up	1.773794	0.826838	1.773794	up	1.025884	0.036867	1.025884	up	105.6214
up	-1.1636	-0.2186	1.163602	down	-1.06585	-0.09201	1.065853	down	25.66692
up	-1.05874	-0.08235	1.058742	down	1.02553	0.03637	1.02553	up	134.4868
down	-1.10773	-0.1476	1.107727	down	-1.08439	-0.11689	1.084393	down	33.46239
up	1.045707	0.064479	1.045707	up	1.129281	0.175405	1.129281	up	41.39631
up	-1.30296	-0.38179	1.302957	down	-1.26433	-0.33838	1.264335	down	31.59609
down	1.028879	0.041074	1.028879	up	-1.01329	-0.01904	1.013288	down	16.89761
down	1.450413	0.536464	1.450413	up	1.147479	0.198467	1.147479	up	41.85157
down	1.476328	0.562013	1.476328	up	1.302803	0.381619	1.302803	up	58.64343
up	-1.03866	-0.05473	1.038661	down	1.063733	0.089136	1.063733	up	301.2479
down	-1.111	-0.15186	1.110998	down	-1.07009	-0.09774	1.070093	down	25.5601
down	-1.34175	-0.42411	1.341747	down	-1.09579	-0.13197	1.095792	down	47.46273
down	-1.9541	-0.9665	1.954095	down	-1.68063	-0.749	1.680632	down	118.4618
up	-1.01546	-0.02213	1.015456	down	-1.04445	-0.06274	1.044445	down	35.09649
up	1.226488	0.294533	1.226488	up	1.148375	0.199594	1.148375	up	32.09534
up	-2.86599	-1.51903	2.86599	down	-2.07457	-1.05281	2.074566	down	59.55392
up	-1.08753	-0.12106	1.08753	down	-1.11835	-0.16137	1.118348	down	19.94966
down	1.030683	0.0436	1.030683	up	1.049659	0.069921	1.049659	up	616.807
down	1.070399	0.098148	1.070399	up	-1.11127	-0.15221	1.111268	down	160.6287
up	1.017091	0.024448	1.017091	up	1.066895	0.093419	1.066895	up	109.4242
up	1.064691	0.090434	1.064691	up	1.052388	0.073666	1.052388	up	100.6954
down	-1.01512	-0.02164	1.015116	down	-1.02279	-0.03251	1.022791	down	114.1136
down	-1.00131	-0.00189	1.001309	down	-1.25547	-0.32823	1.255469	down	85.89108
down	1.008794	0.012631	1.008794	up	-1.02595	-0.03696	1.025952	down	26.01377
down	-1.07198	-0.10028	1.071982	down	-1.06366	-0.08904	1.063661	down	333.8157
down	1.047023	0.066292	1.047023	up	-1.01726	-0.02469	1.017265	down	93.84357
down	-1.12819	-0.17401	1.128189	down	1.03137	0.044562	1.03137	up	92.35952
down	-1.0434	-0.06129	1.043397	down	1.005132	0.007385	1.005132	up	24.07664
up	1.271839	0.346917	1.271839	up	1.180828	0.239799	1.180828	up	1781.009
down	1.057505	0.080664	1.057505	up	1.174726	0.232325	1.174726	up	47.14351
up	-1.05182	-0.07289	1.051821	down	-1.12387	-0.16848	1.123871	down	32.18617
up	-1.22011	-0.28701	1.22011	down	-1.20766	-0.27221	1.207658	down	614.9384
up	-1.01015	-0.01457	1.01015	down	1.05913	0.08288	1.05913	up	20.15488
up	-1.0123	-0.01764	1.0123	down	1.067457	0.094178	1.067457	up	31.89447
up	-1.13676	-0.18493	1.13676	down	-1.24013	-0.31049	1.240133	down	103.3622
up	1.264425	0.338482	1.264425	up	1.000892	0.001287	1.000892	up	113.3672
up	1.006745	0.009698	1.006745	up	1.011624	0.016673	1.011624	up	104.849
up	1.042914	0.06062	1.042914	up	-1.048	-0.06764	1.048005	down	54.41101
up	1.123132	0.167528	1.123132	up	1.135219	0.182971	1.135219	up	15.71005
up	-1.05795	-0.08128	1.057955	down	-1.01434	-0.02054	1.014337	down	56.15828

down	-1.15681	-0.21016	1.156813	down	-1.08495	-0.11763	1.084948	down	46.75801
down	-1.19339	-0.25507	1.193394	down	-1.0782	-0.10863	1.078203	down	36.07698
up	-1.06822	-0.09521	1.068219	down	-1.26084	-0.33438	1.260836	down	16.22008
down	1.173419	0.230718	1.173419	up	1.006261	0.009004	1.006261	up	52.21478
down	-1.18105	-0.24007	1.18105	down	-1.00227	-0.00328	1.002275	down	24.5082
up	-1.00313	-0.00451	1.003132	down	1.03695	0.052346	1.03695	up	47.71677
up	-1.09773	-0.13452	1.097729	down	-1.18318	-0.24267	1.183178	down	101.1782
up	1.029916	0.042527	1.029916	up	1.009115	0.01309	1.009115	up	40.1279
down	-1.06842	-0.09548	1.068419	down	1.166426	0.222095	1.166426	up	39.96763
down	-1.15203	-0.20417	1.152027	down	-1.13244	-0.17944	1.132443	down	42.52525
up	-1.27526	-0.35079	1.275258	down	-1.28326	-0.35981	1.283259	down	21.33573
down	1.035624	0.050501	1.035624	up	-1.16512	-0.22048	1.16512	down	111.1903
up	1.161123	0.215521	1.161123	up	-1.03112	-0.04421	1.031117	down	39.06845
up	-1.3017	-0.3804	1.301701	down	-1.23592	-0.30559	1.235924	down	23.5362
down	-1.02726	-0.0388	1.027259	down	-1.23559	-0.3052	1.235589	down	25.48068
down	1.0016	0.002306	1.0016	up	-1.04178	-0.05905	1.041777	down	32.99616
up	-1.13569	-0.18356	1.135685	down	1.006779	0.009748	1.006779	up	64.30349
down	-1.22858	-0.297	1.228584	down	-1.12682	-0.17226	1.126819	down	22.65042
up	-1.07729	-0.10741	1.07729	down	1.033172	0.047081	1.033172	up	29.37737
down	1.020001	0.028571	1.020001	up	-1.09679	-0.13329	1.096792	down	19.66542
up	1.03016	0.042868	1.03016	up	1.055579	0.078035	1.055579	up	18.05671
down	1.107197	0.146912	1.107197	up	1.044966	0.063455	1.044966	up	29.29286
down	-1.21101	-0.27621	1.211009	down	-1.11647	-0.15894	1.116465	down	25.11596
up	-1.324	-0.40491	1.324004	down	-1.20626	-0.27054	1.20626	down	32.59031
up	1.091953	0.12691	1.091953	up	-1.01728	-0.02472	1.017283	down	46.19644
up	1.012846	0.018415	1.012846	up	-1.04201	-0.05937	1.042013	down	94.47483
up	-1.67531	-0.74442	1.675306	down	-1.41554	-0.50135	1.415536	down	62.47501
up	-1.03971	-0.05618	1.039712	down	-1.20012	-0.26318	1.200121	down	33.17794
down	1.054455	0.076497	1.054455	up	1.063857	0.089304	1.063857	up	40.01978
down	-1.02238	-0.03194	1.022383	down	-1.02192	-0.03128	1.021916	down	43.15964
up	-1.03647	-0.05168	1.036473	down	-1.17012	-0.22666	1.170123	down	24.88589
up	-1.04919	-0.06928	1.049191	down	-1.2049	-0.26891	1.204896	down	15.98849
down	-1.1957	-0.25786	1.195702	down	-1.07158	-0.09973	1.071575	down	74.08019
down	-1.06838	-0.09542	1.06838	down	1.024712	0.035219	1.024712	up	49.18923
down	1.109237	0.149567	1.109237	up	1.194497	0.256403	1.194497	up	171.328
down	1.19017	0.251167	1.19017	up	1.18009	0.238896	1.18009	up	59.4144
up	1.335923	0.417837	1.335923	up	1.006505	0.009354	1.006505	up	97.09263
down	1.174541	0.232097	1.174541	up	1.031492	0.044733	1.031492	up	24.40281
down	1.02404	0.034272	1.02404	up	1.102574	0.140876	1.102574	up	36.88969
down	-1.09768	-0.13446	1.097682	down	-1.22462	-0.29234	1.224623	down	78.14895
down	1.018025	0.025773	1.018025	up	1.111921	0.153054	1.111921	up	27.86633
down	1.136292	0.184334	1.136292	up	1.061423	0.086	1.061423	up	46.26381
up	1.236889	0.306716	1.236889	up	1.14169	0.191171	1.14169	up	68.5379
down	-1.01823	-0.02606	1.01823	down	1.130022	0.176351	1.130022	up	543.7831
down	1.039326	0.055648	1.039326	up	-1.20387	-0.26768	1.203867	down	36.53431
up	-1.24213	-0.31281	1.242127	down	-1.26106	-0.33464	1.261064	down	16.14294
up	1.085413	0.118244	1.085413	up	1.066834	0.093336	1.066834	up	28.80615

down	1.106903	0.146528	1.106903	up	1.075652	0.105211	1.075652	up	22.70597
up	1.000744	0.001072	1.000744	up	1.071279	0.099334	1.071279	up	458.0061
down	1.375844	0.460317	1.375844	up	1.142721	0.192474	1.142721	up	34.45505
up	-1.0345	-0.04893	1.034496	down	1.223954	0.29155	1.223954	up	110.1267
up	1.074787	0.104051	1.074787	up	1.113416	0.154992	1.113416	up	36.05436
down	1.169629	0.226051	1.169629	up	-1.01214	-0.0174	1.012136	down	21.52555
down	-1.05743	-0.08056	1.057432	down	1.01677	0.023993	1.01677	up	33.35917
up	1.219459	0.286242	1.219459	up	-1.00035	-5.09E-04	1.000353	down	17.64571
down	-1.30147	-0.38015	1.301475	down	-1.22685	-0.29495	1.226846	down	144.9843
down	1.061984	0.086761	1.061984	up	-1.15519	-0.20813	1.155193	down	43.93784
down	1.06642	0.092775	1.06642	up	1.171462	0.228311	1.171462	up	464.9346
down	-1.31291	-0.39277	1.312911	down	-1.11992	-0.1634	1.11992	down	23.62096
down	-1.01379	-0.01976	1.013793	down	1.000837	0.001207	1.000837	up	518.0082
up	-1.07658	-0.10646	1.076585	down	-1.0608	-0.08515	1.060799	down	609.5981
up	1.022525	0.032136	1.022525	up	1.206964	0.271383	1.206964	up	126.4563
down	-1.20936	-0.27425	1.209364	down	-1.00399	-0.00574	1.003985	down	16.16084
down	1.156334	0.209558	1.156334	up	1.051536	0.072498	1.051536	up	85.55007
up	1.080782	0.112075	1.080782	up	1.034494	0.048925	1.034494	up	197.1958
down	1.181917	0.241128	1.181917	up	-1.04887	-0.06884	1.048871	down	63.73785
down	-1.04352	-0.06146	1.043524	down	-1.32037	-0.40095	1.320374	down	1663.261
down	1.009971	0.014314	1.009971	up	-1.15696	-0.21034	1.156965	down	481.7015
down	1.149127	0.200538	1.149127	up	-1.01691	-0.02419	1.01691	down	69.99374
up	1.006868	0.009874	1.006868	up	-1.08183	-0.11347	1.081829	down	449.1811
down	-1.0672	-0.09383	1.0672	down	-1.11102	-0.15189	1.111021	down	345.5026
up	1.160171	0.214338	1.160171	up	1.02948	0.041915	1.02948	up	502.0288
down	1.08218	0.11394	1.08218	up	1.09144	0.126232	1.09144	up	1619.245
up	1.007905	0.011359	1.007905	up	1.05607	0.078706	1.05607	up	155.9437
up	-1.02086	-0.02979	1.020863	down	-1.08246	-0.11431	1.082459	down	2191.544
up	-1.05637	-0.07912	1.056372	down	-1.09047	-0.12495	1.090468	down	454.9776
up	1.132377	0.179354	1.132377	up	1.060114	0.084219	1.060114	up	41.24048
up	-1.09318	-0.12853	1.093181	down	-1.13829	-0.18687	1.138294	down	113.1554
up	-1.03965	-0.05609	1.039646	down	-1.04489	-0.06336	1.044894	down	1892.072
down	-1.02718	-0.03869	1.027184	down	-1.00034	-4.96E-04	1.000344	down	544.6856
up	1.171453	0.228299	1.171453	up	1.197117	0.259565	1.197117	up	336.842
up	-1.08411	-0.11651	1.084112	down	-1.1149	-0.15691	1.114898	down	104.3153
down	-1.69907	-0.76475	1.699074	down	-1.30836	-0.38776	1.308363	down	18.73614
up	1.067782	0.094617	1.067782	up	1.092695	0.127891	1.092695	up	107.139
down	-1.04408	-0.06224	1.044084	down	-1.0571	-0.08011	1.0571	down	50.54391
up	1.108376	0.148447	1.108376	up	1.084611	0.117177	1.084611	up	90.27885
up	-1.04727	-0.06663	1.04727	down	1.154396	0.207139	1.154396	up	20.69777
down	-1.34584	-0.4285	1.345835	down	-1.31526	-0.39535	1.315262	down	77.0098
down	-1.02224	-0.03174	1.022242	down	-1.08408	-0.11647	1.084078	down	56.93355
down	-1.06403	-0.08953	1.064026	down	-1.1606	-0.21487	1.160597	down	373.1819
up	1.196815	0.2592	1.196815	up	1.026956	0.038375	1.026956	up	18.38181
up	1.086875	0.120186	1.086875	up	-1.12577	-0.17092	1.125774	down	41.5471
up	1.000474	6.84E-04	1.000474	up	-1.1344	-0.18193	1.134399	down	16.19888
up	1.084544	0.117088	1.084544	up	-1.0275	-0.03914	1.027504	down	277.3151

down	-1.1304	-0.17683	1.1304	down	-1.11069	-0.15146	1.11069	down	184.998
up	-1.01147	-0.01646	1.011472	down	-1.12532	-0.17033	1.125315	down	82.40397
up	1.41199	0.49773	1.41199	up	1.211577	0.276886	1.211577	up	190.0934
up	-1.14318	-0.19305	1.143178	down	1.014687	0.021035	1.014687	up	62.78101
down	1.091886	0.126822	1.091886	up	1.224167	0.291801	1.224167	up	22.75359
up	-1.25045	-0.32244	1.250446	down	-1.09412	-0.12977	1.094121	down	22.71322
up	-1.16797	-0.224	1.167966	down	-1.04243	-0.05995	1.042433	down	157.6278
up	-1.08305	-0.11509	1.083046	down	-1.03449	-0.04893	1.034495	down	16.35503
up	-1.07163	-0.0998	1.071627	down	-1.06995	-0.09755	1.069953	down	27.21011
up	-1.07038	-0.09812	1.070377	down	-1.00821	-0.01179	1.008207	down	40.53215
down	1.056742	0.079623	1.056742	up	-1.0895	-0.12367	1.0895	down	21.57484
down	-1.02185	-0.03119	1.021854	down	1.031438	0.044657	1.031438	up	24.74056
up	-1.23161	-0.30054	1.231605	down	-1.12525	-0.17025	1.125255	down	76.98753
down	1.077312	0.107436	1.077312	up	-1.30194	-0.38066	1.301939	down	146.3982
down	1.350462	0.433453	1.350462	up	1.27483	0.350305	1.27483	up	78.87193
up	-1.15051	-0.20227	1.15051	down	-1.09988	-0.13735	1.099881	down	19.36971
down	1.196794	0.259175	1.196794	up	1.224314	0.291974	1.224314	up	32.47603
up	-1.00577	-0.00829	1.005765	down	1.028384	0.040379	1.028384	up	19.34133
down	1.025128	0.035804	1.025128	up	1.111803	0.152901	1.111803	up	22.07111
up	-1.11261	-0.15395	1.112609	down	-1.06646	-0.09283	1.066457	down	22.75812
down	-1.0695	-0.09693	1.069496	down	-1.20869	-0.27344	1.20869	down	160.6022
down	-1.06496	-0.0908	1.064959	down	-1.04944	-0.06961	1.049437	down	19.86652
down	1.153276	0.205737	1.153276	up	1.083853	0.116169	1.083853	up	66.79803
up	-1.00643	-0.00924	1.006427	down	1.007438	0.010691	1.007438	up	22.04617
down	-1.13073	-0.17726	1.130732	down	-1.11793	-0.16083	1.117927	down	21.15584
down	1.185814	0.245877	1.185814	up	1.009844	0.014132	1.009844	up	23.617
up	-1.04349	-0.06142	1.043493	down	-1.202	-0.26544	1.202	down	35.52529
down	-1.13355	-0.18084	1.133547	down	-1.11848	-0.16154	1.118479	down	31.5927
up	-1.14402	-0.19411	1.144018	down	1.024391	0.034767	1.024391	up	43.98906
up	-1.06471	-0.09046	1.064707	down	-1.00173	-0.00249	1.001726	down	298.7967
up	-1.16273	-0.21752	1.162735	down	-1.13282	-0.17992	1.132823	down	32.0952
up	-1.00218	-0.00314	1.002179	down	-1.03519	-0.0499	1.035192	down	643.2064
down	-1.09168	-0.12655	1.091681	down	-1.08578	-0.11873	1.08578	down	1201.591
up	1.20807	0.272704	1.20807	up	1.088036	0.121727	1.088036	up	469.8077
up	1.61141	0.688324	1.61141	up	1.556709	0.638499	1.556709	up	284.6016
down	-1.07362	-0.10249	1.073621	down	1.111553	0.152576	1.111553	up	1568.862
up	1.141696	0.191178	1.141696	up	1.266772	0.341157	1.266772	up	1069.542
up	-1.06961	-0.09708	1.069608	down	-1.0978	-0.13462	1.097803	down	103.408
up	1.213918	0.279671	1.213918	up	1.121471	0.165393	1.121471	up	566.4774
down	-1.59291	-0.67167	1.592912	down	-1.43515	-0.5212	1.435149	down	68.2685
down	-1.19569	-0.25784	1.195691	down	1.018635	0.026638	1.018635	up	1689.195
up	-1.02839	-0.04039	1.028395	down	-1.07889	-0.10955	1.078889	down	560.3748
up	-1.03644	-0.05164	1.036439	down	1.042462	0.059995	1.042462	up	344.6686
up	1.087119	0.12051	1.087119	up	1.363439	0.447251	1.363439	up	73.53547
down	-1.05297	-0.07446	1.052967	down	-1.12206	-0.16615	1.122061	down	268.6446
down	-1.16163	-0.21615	1.16163	down	-1.12025	-0.16382	1.120252	down	132.7339
up	-1.21516	-0.28115	1.215162	down	-1.01148	-0.01646	1.011476	down	143.9322

down	-1.01567	-0.02243	1.015666	down	1.169418	0.225791	1.169418	up	281.2833
up	1.161189	0.215603	1.161189	up	1.314986	0.395047	1.314986	up	143.4774
up	-1.23558	-0.30519	1.23558	down	-1.2251	-0.2929	1.225101	down	360.3538
up	1.559238	0.640841	1.559238	up	1.632877	0.707416	1.632877	up	105.4294
up	-1.08088	-0.11221	1.080884	down	-1.07879	-0.10942	1.078792	down	191.4223
up	1.061172	0.085658	1.061172	up	-1.01158	-0.01661	1.011577	down	254.452
up	-1.019	-0.02715	1.018997	down	-1.05207	-0.07323	1.05207	down	362.1983
down	-1.42655	-0.51253	1.426551	down	-1.48765	-0.57304	1.487652	down	310.5948
down	-1.21537	-0.28139	1.215365	down	-1.06489	-0.09071	1.064891	down	97.94598
up	-1.52708	-0.61077	1.527076	down	-1.2424	-0.31313	1.2424	down	89.1723
up	1.010157	0.01458	1.010157	up	1.035173	0.049872	1.035173	up	396.377
up	-1.08818	-0.12191	1.088178	down	1.011851	0.016997	1.011851	up	107.5759
up	-1.14237	-0.19203	1.142371	down	-1.30674	-0.38597	1.306736	down	70.74984
down	-1.02299	-0.0328	1.022994	down	1.080648	0.111896	1.080648	up	39.62804
up	1.195772	0.257942	1.195772	up	-1.04661	-0.06572	1.046608	down	41.78662
down	-1.0935	-0.12895	1.0935	down	-1.03702	-0.05245	1.037022	down	63.83611
down	-1.06644	-0.09281	1.066443	down	1.098313	0.135289	1.098313	up	212.0874
down	-1.13654	-0.18465	1.136543	down	-1.07351	-0.10233	1.073507	down	446.6021
down	-1.16099	-0.21536	1.160993	down	-1.06588	-0.09204	1.065878	down	266.8644
up	-1.27589	-0.3515	1.275886	down	-1.15802	-0.21166	1.158018	down	377.239
up	-1.13365	-0.18097	1.133649	down	-1.01448	-0.02074	1.014478	down	211.1768
up	1.220506	0.287479	1.220506	up	1.155494	0.208509	1.155494	up	165.293
up	-1.07929	-0.11008	1.079287	down	1.006187	0.008898	1.006187	up	54.70745
down	-1.03378	-0.04793	1.033779	down	-1.07961	-0.11051	1.079613	down	429.9292
up	-1.11884	-0.162	1.11884	down	1.062073	0.086883	1.062073	up	562.7954
up	1.296058	0.37413	1.296058	up	1.099544	0.136905	1.099544	up	238.6547
up	1.025352	0.03612	1.025352	up	-1.06993	-0.09752	1.069935	down	229.1301
down	1.232872	0.302023	1.232872	up	1.253674	0.326162	1.253674	up	105.6012
down	-1.07608	-0.10578	1.076076	down	-1.05413	-0.07605	1.05413	down	167.6708
up	1.18329	0.242804	1.18329	up	1.095095	0.131056	1.095095	up	632.3877
down	-1.05552	-0.07795	1.055519	down	-1.14109	-0.19041	1.141086	down	1519.144
up	1.098106	0.135017	1.098106	up	1.145512	0.195992	1.145512	up	37.52343
up	-1.07318	-0.1019	1.073184	down	-1.08303	-0.11508	1.083032	down	166.0807
down	1.134389	0.181915	1.134389	up	1.236022	0.305704	1.236022	up	118.5087
down	-1.02098	-0.02995	1.020977	down	-1.10113	-0.13899	1.101132	down	191.9665
down	1.147311	0.198257	1.147311	up	1.111842	0.152952	1.111842	up	122.2997
up	-1.08206	-0.11378	1.082063	down	-1.07414	-0.10318	1.074135	down	67.97286
up	1.0277	0.03942	1.0277	up	1.122846	0.16716	1.122846	up	1186.225
up	-1.43174	-0.51777	1.431736	down	-1.25056	-0.32258	1.250561	down	200.5396
up	1.054826	0.077004	1.054826	up	1.049036	0.069065	1.049036	up	36.09252
up	1.036014	0.051044	1.036014	up	-1.06329	-0.08854	1.063291	down	353.6825
up	1.223367	0.290858	1.223367	up	1.116711	0.159256	1.116711	up	38.90347
down	1.031866	0.045255	1.031866	up	-1.2465	-0.31789	1.246503	down	100.8607
up	-1.54758	-0.63001	1.547581	down	-1.41458	-0.50037	1.414581	down	205.6668
down	-1.26566	-0.33989	1.265657	down	-1.16986	-0.22634	1.169862	down	58.485
up	1.034771	0.049312	1.034771	up	1.090427	0.124893	1.090427	up	46.70346
down	1.004597	0.006616	1.004597	up	-1.06428	-0.08987	1.064278	down	104.0074

up	-1.21128	-0.27654	1.211284	down	-1.31656	-0.39677	1.316556	down	1454.271
down	1.044127	0.062298	1.044127	up	-1.02746	-0.03908	1.027455	down	34.1996
up	1.056665	0.079518	1.056665	up	1.053728	0.075503	1.053728	up	235.9023
down	1.003711	0.005343	1.003711	up	1.066966	0.093515	1.066966	up	87.31144
up	-1.73761	-0.7971	1.737606	down	-1.45373	-0.53976	1.45373	down	13.7676
up	1.067912	0.094793	1.067912	up	-1.00842	-0.0121	1.008424	down	413.4205
up	1.093008	0.128304	1.093008	up	-1.02765	-0.03935	1.027651	down	218.0823
down	1.319138	0.399595	1.319138	up	-1.09268	-0.12788	1.092684	down	400.517
up	-1.29822	-0.37653	1.29822	down	-1.33722	-0.41924	1.337222	down	39.27813
up	-1.39668	-0.482	1.396678	down	-1.28248	-0.35893	1.282477	down	118.0028
up	-1.15714	-0.21057	1.157145	down	-1.10851	-0.14862	1.108509	down	104.4717
down	1.047999	0.067637	1.047999	up	-1.07091	-0.09884	1.070914	down	81.39311
up	-1.26765	-0.34216	1.267651	down	-1.2828	-0.3593	1.282804	down	123.6569
down	-1.10266	-0.14099	1.102659	down	1.024468	0.034874	1.024468	up	33.97127
down	1.168904	0.225156	1.168904	up	1.154777	0.207614	1.154777	up	61.5473
up	1.188119	0.248679	1.188119	up	-1.01208	-0.01732	1.012076	down	32.35303
down	1.019798	0.028283	1.019798	up	-1.08422	-0.11665	1.084217	down	250.2792
down	1.034063	0.048324	1.034063	up	-1.04989	-0.07024	1.04989	down	66.21139
up	1.1762	0.234134	1.1762	up	1.280721	0.356956	1.280721	up	305.6734
down	-1.11061	-0.15136	1.110613	down	-1.01428	-0.02045	1.014275	down	55.50871
down	1.053932	0.075782	1.053932	up	1.121793	0.165806	1.121793	up	31.38635
down	1.003043	0.004383	1.003043	up	-1.05054	-0.07114	1.050544	down	120.9106
down	1.331705	0.413275	1.331705	up	1.3785	0.463099	1.3785	up	128.2771
down	1.139845	0.188838	1.139845	up	1.102859	0.141249	1.102859	up	37.83038
down	1.157378	0.21086	1.157378	up	1.067051	0.093629	1.067051	up	83.65557
down	1.109013	0.149277	1.109013	up	1.035816	0.050767	1.035816	up	84.0431
up	1.103133	0.141607	1.103133	up	-1.07586	-0.10548	1.075856	down	1106.21
down	-1.06192	-0.08667	1.06192	down	1.000142	2.05E-04	1.000142	up	93.19672
down	1.266875	0.341274	1.266875	up	1.207074	0.271514	1.207074	up	21.18642
down	1.194706	0.256655	1.194706	up	1.253053	0.325447	1.253053	up	23.88281
down	1.067591	0.094359	1.067591	up	1.018566	0.026539	1.018566	up	22.6122
up	-1.10322	-0.14173	1.103223	down	-1.03588	-0.05085	1.035878	down	21.92769
down	1.254091	0.326642	1.254091	up	1.188065	0.248613	1.188065	up	91.98676
down	-1.19118	-0.25239	1.191179	down	-1.32002	-0.40056	1.320018	down	189.4129
down	1.018343	0.026224	1.018343	up	-1.00329	-0.00473	1.003286	down	338.4432
down	-1.08477	-0.11739	1.084774	down	-1.0047	-0.00676	1.0047	down	24.49687
down	-1.24357	-0.31449	1.24357	down	-1.30112	-0.37976	1.301124	down	134.0288
up	-1.21018	-0.27522	1.210178	down	-1.16134	-0.21579	1.161339	down	116.3417
up	-1.40236	-0.48786	1.402364	down	-1.17962	-0.23832	1.179618	down	58.01
up	1.091545	0.126371	1.091545	up	-1.06818	-0.09516	1.068183	down	24.21968
down	1.85673	0.892764	1.85673	up	1.509892	0.594446	1.509892	up	63.30872
up	1.344093	0.426633	1.344093	up	1.049724	0.070009	1.049724	up	37.18936
up	-1.23555	-0.30516	1.235553	down	-1.08915	-0.1232	1.089151	down	24.20976
up	-1.2024	-0.26592	1.202405	down	-1.14378	-0.19381	1.143782	down	85.37199
up	1.228198	0.296543	1.228198	up	1.327542	0.408757	1.327542	up	72.01733
up	-1.11863	-0.16173	1.118631	down	1.094397	0.130136	1.094397	up	152.234
down	1.097744	0.134542	1.097744	up	-1.0251	-0.03576	1.0251	down	101.5176

up	-1.02962	-0.04212	1.029624	down	-1.13872	-0.18741	1.138716	down	132.5682
down	-1.04355	-0.0615	1.043549	down	1.082831	0.114808	1.082831	up	86.89791
up	1.027192	0.038706	1.027192	up	1.06765	0.094439	1.06765	up	20.11215
down	-1.01515	-0.02169	1.01515	down	-1.01512	-0.02165	1.015117	down	23.46712
down	-1.02045	-0.02921	1.020452	down	1.000548	7.90E-04	1.000548	up	21.22483
up	-1.01106	-0.01587	1.011062	down	1.064835	0.090629	1.064835	up	20.49973
down	1.07112	0.09912	1.07112	up	-1.12195	-0.16601	1.121952	down	18.2448
down	1.110385	0.15106	1.110385	up	1.028592	0.04067	1.028592	up	25.82723
up	-1.06251	-0.08747	1.062507	down	-1.17377	-0.23115	1.17377	down	36.83962
up	1.075167	0.10456	1.075167	up	1.044127	0.062297	1.044127	up	68.56687
down	-1.01109	-0.01591	1.01109	down	-1.0053	-0.00762	1.005296	down	39.47626
up	-1.09165	-0.12651	1.091649	down	-1.01549	-0.02218	1.01549	down	188.522
up	-1.17048	-0.2271	1.170482	down	-1.25939	-0.33272	1.259386	down	90.00187
down	1.245239	0.316423	1.245239	up	1.185195	0.245124	1.185195	up	89.90967
down	1.100498	0.138157	1.100498	up	1.036165	0.051253	1.036165	up	37.32589
down	-1.04358	-0.06154	1.043579	down	1.081176	0.112602	1.081176	up	28.78334
down	1.089399	0.123532	1.089399	up	1.01204	0.017266	1.01204	up	30.7318
down	1.115937	0.158255	1.115937	up	-1.33652	-0.41848	1.336516	down	122.2707
up	-1.41573	-0.50154	1.415725	down	-1.41256	-0.49831	1.41256	down	88.79331
up	-1.09838	-0.13538	1.098384	down	1.011234	0.016117	1.011234	up	30.46578
down	-1.01945	-0.02779	1.019449	down	-1.11858	-0.16167	1.11858	down	20.22182
down	-1.20159	-0.26494	1.201586	down	-1.04802	-0.06766	1.048017	down	25.24711
down	1.104318	0.143156	1.104318	up	-1.08542	-0.11826	1.085425	down	40.12429
up	-1.25131	-0.32344	1.251308	down	-1.30198	-0.38071	1.301979	down	16.70673
up	-1.11479	-0.15677	1.114789	down	-1.08337	-0.11552	1.083366	down	104.991
up	-1.15511	-0.20803	1.155107	down	-1.18856	-0.24921	1.18856	down	31.57881
down	-1.2484	-0.32008	1.248396	down	-1.27944	-0.35552	1.279445	down	77.95911
down	-1.12809	-0.17388	1.128088	down	-1.28683	-0.36382	1.286827	down	23.8895
down	1.288093	0.365237	1.288093	up	1.060283	0.084449	1.060283	up	29.10417
up	1.004395	0.006326	1.004395	up	1.055699	0.078199	1.055699	up	134.3012
down	1.143309	0.193216	1.143309	up	-1.07038	-0.09813	1.070384	down	103.878
down	-1.2995	-0.37796	1.299502	down	-1.15566	-0.20872	1.155664	down	15.57038
down	1.03798	0.053778	1.03798	up	1.026621	0.037903	1.026621	up	98.80701
up	1.085886	0.118872	1.085886	up	1.032693	0.046411	1.032693	up	1855.328
down	-1.12956	-0.17576	1.129563	down	-1.0971	-0.13369	1.097097	down	325.259
up	1.067337	0.094016	1.067337	up	-1.27104	-0.34601	1.271039	down	49.60305
up	-1.17811	-0.23647	1.178105	down	-1.13015	-0.17651	1.130148	down	23.89082
down	-1.00025	-3.59E-04	1.000249	down	-1.08282	-0.11479	1.082821	down	70.04926
up	-1.51186	-0.59632	1.511857	down	-1.33551	-0.41739	1.335513	down	52.5565
down	-1.02019	-0.02884	1.02019	down	1.025262	0.035992	1.025262	up	64.48332
up	-1.11353	-0.15515	1.113535	down	-1.13794	-0.18643	1.137944	down	532.7609
down	-1.11132	-0.15227	1.11132	down	-1.0934	-0.12882	1.093396	down	22.71561
down	1.237217	0.307098	1.237217	up	1.009952	0.014287	1.009952	up	1655.232
up	1.073685	0.10257	1.073685	up	-1.04666	-0.0658	1.046662	down	248.577
down	-1.12982	-0.17609	1.129819	down	-1.29388	-0.37171	1.293881	down	124.6845
down	-1.14027	-0.18938	1.140272	down	-1.15389	-0.2065	1.153885	down	483.9817
down	1.071669	0.099859	1.071669	up	1.091851	0.126776	1.091851	up	283.456

up	-1.09547	-0.13155	1.095471	down	-1.00675	-0.00971	1.006751	down	21.99899

[D07-DEX](	[D07-TAA](	[D30-Con](	[D30-DEX](	[D30-TAA](	[D07-Con](	[D07-DEX](	[D07-TAA](	[D30-Con](	[D30-DEX](
872.5004	977.9829	997.1707	919.2115	1032.432	-0.1102	-0.10437	0.060048	0.084875	-0.02943
2859.596	3075.528	2999.391	3272.827	3392.664	-0.1192	-0.09746	0.009485	-0.02687	0.096227
341.7369	360.2467	398.0618	418.1769	372.1173	-0.10009	-0.11325	-0.03683	0.10885	0.174824
227.1109	214.9229	284.4835	252.541	259.6142	-0.16382	-0.05428	-0.13613	0.264736	0.093511
1262.258	1308.568	1259.855	1313.24	1384.463	-0.29843	-0.03842	0.016706	-0.03894	0.019655
614.575	602.7229	436.4609	391.0393	430.1369	0.388609	0.147245	0.133559	-0.35746	-0.51649
5864.014	6252.814	5851.477	5651.58	5840.977	-0.05236	-0.00642	0.086054	-0.00981	-0.06053
26.20618	25.94585	21.89092	21.31137	23.54136	0.038448	0.143243	0.125967	-0.12088	-0.15575
1914.207	2252.275	2577.067	2433.588	2522.079	-0.3863	-0.19219	0.048998	0.230402	0.145682
1028.385	1185.066	759.1442	729.97	799.3911	-0.01011	0.198485	0.405376	-0.25381	-0.30159
137.3655	173.4622	90.28562	124.4615	109.9227	0.333727	0.130095	0.475171	-0.47751	-0.0093
3237.937	3423.337	2363.908	2754.203	2631.327	0.04358	0.177796	0.254234	-0.27914	-0.05216
2266.477	2498.158	2240.487	2186.188	1982.582	-0.09605	0.024992	0.163903	0.009459	-0.02599
1018.463	1105.247	931.9624	987.0856	1004.047	-0.06558	0.025166	0.142705	-0.10465	-0.01927
1913.306	1932.896	2139.239	1929.952	2225.713	-0.09075	-0.0115	0.002898	0.14319	0.001065
649.1071	703.6389	663.924	776.9401	704.007	-0.03694	-0.11094	0.006857	-0.07863	0.147796
25.70481	27.12022	22.59749	19.24632	23.49197	0.300661	0.054219	0.133277	-0.12884	-0.40125
186.9615	134.8341	128.4812	132.4961	135.9747	0.550019	0.430814	-0.04009	-0.11	-0.06504
5103.819	4554.798	4786.945	4394.947	4257.775	-0.00857	0.145063	-0.01732	0.054052	-0.06931
89.9768	84.31054	71.62395	83.25401	75.00314	-0.0279	0.187091	0.093362	-0.14084	0.071016
312.78	314.2325	215.0126	266.9626	219.2294	0.002208	0.234678	0.238581	-0.33693	5.08E-04
1486.302	1282.158	1521.033	1480.674	1475.725	-0.10283	0.027414	-0.18815	0.06064	0.020534
1731.045	1852.585	1833.884	1634.674	1629.073	-0.09681	0.040728	0.136904	0.119372	-0.04125
345.1524	274.837	352.384	310.2297	248.5086	0.173027	0.176601	-0.15475	0.192158	0.014125
2665.992	2828.305	3036.819	2966.586	3122.446	-0.11195	-0.10377	-0.01821	0.083846	0.047293
124.1148	120.3275	101.7806	106.589	93.81995	0.166876	0.118493	0.076353	-0.18095	-0.09929
501.1126	516.3442	516.9641	548.0198	565.1439	-0.15093	-0.0147	0.02831	0.03059	0.107621
308.849	281.8754	162.2634	242.7359	190.2775	0.248577	0.260944	0.129145	-0.72215	-0.08756
250.1101	247.8776	222.9485	243.7254	188.2706	0.119806	0.051294	0.038453	-0.10843	0.025249
386.576	400.1713	835.3039	434.3307	510.2143	-0.17597	-0.12817	-0.07391	0.961905	0.036054
714.2209	739.9172	906.1113	1152.477	955.205	-0.48413	-0.15481	-0.09736	0.147728	0.53668
203.2236	197.2203	164.1587	119.261	132.3142	0.089403	0.239992	0.210818	-0.09382	-0.53234
84.77998	79.22934	54.00747	66.08132	66.87408	0.195065	0.165967	0.072368	-0.52404	-0.2044

1188.517	1222.465	1334.048	1191.577	1326.887	0.010061	-0.07469	-0.03426	0.091165	-0.07131
368.6522	401.4504	332.9652	320.2955	328.2408	0.001064	0.111603	0.235966	-0.03397	-0.08953
598.3586	603.674	609.7919	620.3391	643.1809	-0.24535	-0.02345	-0.01017	0.00403	0.023304
721.6382	761.4788	719.1429	810.5228	749.8718	-0.22891	-0.05153	0.028725	-0.05472	0.120684
1905.345	2117.951	2218.03	2399.358	2167.662	-0.14017	-0.16181	-0.01148	0.057233	0.168113
992.2278	967.9653	936.5752	1095.606	1079.32	-0.1575	-0.00153	-0.03535	-0.0882	0.139926
869.6684	1007.306	628.6149	688.4573	627.5125	0.11692	0.19806	0.413303	-0.27487	-0.13606
5274.445	6003.127	4228.539	4420.594	3888.109	0.097652	0.194778	0.383952	-0.1194	-0.05562
244.0269	192.3434	136.2456	195.8965	167.6103	0.042554	0.372335	0.034096	-0.50426	0.067231
165.1623	174.5816	178.0223	179.9062	201.2287	0.056371	-0.09624	-0.01696	0.012047	0.02751
1523.836	1847.998	1539.627	1337.531	1268.328	0.147234	-0.02465	0.251392	-0.00876	-0.22602
2909.649	2888.877	2959.066	3084.404	3290.178	-0.06836	-0.01366	-0.02367	0.011754	0.070818
959.6872	996.725	869.1812	805.2786	775.5266	0.181059	0.101327	0.154659	-0.03902	-0.16305
70.44769	77.54549	64.99497	58.7282	61.70631	0.133181	0.062133	0.19066	-0.05355	-0.19986
1425.415	1092.182	861.2352	968.3293	995.9261	0.329706	0.433703	0.031419	-0.32803	-0.12126
389.6408	396.707	495.1409	453.5274	425.6296	-0.16025	-0.12774	-0.10252	0.218138	0.091583
295.7498	292.7127	285.0307	284.7972	296.3717	-0.15381	0.042811	0.026327	-0.01097	-0.01125
200.3532	222.1329	202.7624	211.2727	234.3804	-0.21903	-0.04839	0.087145	-0.02869	0.02983
176.2862	179.823	183.218	184.6517	185.5823	-0.15666	-0.046	-0.0162	0.01188	0.023262
181.3344	165.5291	207.4086	226.527	186.4843	-0.21856	-0.05397	-0.19626	0.129948	0.26211
236.8358	239.527	171.3068	218.0072	185.946	0.087379	0.080556	0.099279	-0.39544	-0.0386
154.2322	170.0431	198.12	183.6072	192.6358	-0.26452	-0.13484	0.008663	0.217513	0.112199
38.81617	41.41065	29.44712	30.70689	35.64336	0.127238	0.122197	0.221368	-0.28359	-0.21008
841.0609	576.2532	440.2146	589.0978	508.3862	0.247225	0.481068	-0.10594	-0.489	-0.02375
164.5537	178.6026	201.4163	196.059	190.295	-0.01336	-0.2073	-0.08888	0.085454	0.043725
513.0443	595.5978	346.9877	340.8624	367.8252	0.233217	0.191719	0.406919	-0.37529	-0.41114
48.98711	59.78547	41.56685	41.58479	39.91618	0.164567	0.123793	0.413574	-0.11168	-0.11788
587.5987	598.8477	634.3098	703.6341	619.8745	-0.13108	-0.03664	-0.01161	0.070841	0.222572
170.9008	156.6814	132.3144	128.4361	123.3733	0.074728	0.210332	0.081552	-0.16781	-0.21231
1204.351	985.6934	911.551	1018.368	914.0088	0.315794	0.201624	-0.0831	-0.20202	-0.03646
688.6135	792.8605	624.7455	433.5885	465.6219	0.18515	0.241559	0.454747	0.058685	-0.42742
2815.275	2677.312	3694.569	4122.43	4252.376	-0.17902	-0.24353	-0.3243	0.13424	0.310564
106.3654	153.683	87.62927	98.87946	82.60619	0.065999	0.095445	0.594547	-0.18259	-0.00393
655.7545	772.8699	509.1513	651.5679	470.3223	0.030401	0.082362	0.320133	-0.29966	0.070333
1560.071	1514.555	1294.413	1416.676	1425.416	0.089066	0.083311	0.040209	-0.18679	-0.05529
734.8314	707.5486	535.0603	578.346	478.1666	0.336707	0.20405	0.141426	-0.28906	-0.14445
7906.467	8506.633	7027.389	7611.213	7277.986	0.011977	0.043287	0.150066	-0.12804	-0.01106
362.9237	377.6647	291.3194	289.3092	313.5783	0.244801	0.167561	0.214247	-0.14855	-0.16601
1473.832	1717.598	1235.3	1140.593	1160.582	-0.01455	0.174529	0.391544	-0.07964	-0.2007
555.1846	574.1646	676.1588	624.675	672.6431	-0.02037	-0.08179	-0.03318	0.195669	0.086598
5374.273	5360.663	3593.374	3015.404	3831.693	0.260761	0.299295	0.292646	-0.28571	-0.54453
69.93084	60.61929	42.46956	38.32498	40.68721	0.163899	0.39575	0.230284	-0.30291	-0.44683
281.0812	229.2557	177.3197	208.1624	192.5665	0.062084	0.352991	0.061713	-0.34074	-0.07945
424.6394	461.1011	416.2361	468.4497	494.4688	-0.0168	-0.04499	0.072359	-0.07821	0.094843
3984.531	4264.535	3851.225	3848.854	3876.281	0.135914	-0.01763	0.078805	-0.06796	-0.07131
23.12992	24.37966	20.79853	23.72509	21.62123	0.088662	-2.20E-04	0.077406	-0.1515	0.03776
45.2614	43.69759	50.58641	44.13409	45.90041	-0.17482	0.007238	-0.0434	0.165702	-0.02976

227.474	241.3185	261.1538	262.7604	280.6909	-0.19474	-0.17109	-0.08608	0.033522	0.04321
314.4476	340.9135	269.3249	278.6556	299.9249	-0.00913	0.070242	0.182038	-0.15304	-0.10838
484.3342	534.1989	589.041	562.2767	616.3628	-0.09954	-0.16246	-0.02079	0.11441	0.05319
599.2912	550.1709	373.023	564.7394	449.6046	0.077152	0.252398	0.132349	-0.46518	0.146756
416.2976	394.2162	471.9398	675.4726	701.3038	-0.23545	-0.1647	-0.25231	-0.01348	0.528784
466.4729	412.4587	321.3886	311.5561	363.6916	0.319208	0.232205	0.061309	-0.31317	-0.34344
435.4548	406.1116	411.524	559.9089	493.9504	-0.29101	-0.00413	-0.10403	-0.11042	0.356938
64.83322	54.14601	89.78846	70.00903	68.56921	-0.03591	-0.03579	-0.29358	0.423731	0.065766
119.4502	114.6311	112.6885	111.1601	99.45776	0.383183	0.07058	0.019766	-0.01679	-0.03446
192.009	189.6399	181.1146	198.2544	196.3587	-0.04517	0.023823	0.005843	-0.06023	0.068938
2675.348	2654.932	2357.344	2365.471	2440.564	0.032971	0.049822	0.039314	-0.13268	-0.13138
358.9058	403.3901	448.5873	408.2067	435.1004	-0.0628	-0.19393	-0.02576	0.125489	-0.01188
294.799	342.3141	317.4103	318.2076	335.8342	-0.11689	-0.08776	0.127581	0.018449	0.023218
1841.899	1954.731	1655.267	1559.28	1679.586	0.173325	0.075668	0.16466	-0.07508	-0.16569
676.9004	729.2416	689.9231	712.6567	750.9993	-0.17648	-0.04991	0.057452	-0.02263	0.024279
28.17149	31.11182	28.36379	29.4219	27.20643	0.209691	-0.06901	0.073897	-0.06495	-0.00617
90.5044	122.2505	41.58098	46.75821	50.65198	0.342401	0.241231	0.647594	-0.91392	-0.71205
689.2835	599.4396	755.678	814.0855	771.8763	-0.26617	0.017093	-0.19014	0.14012	0.234807
311.0666	279.0681	155.6952	189.1763	172.3843	0.197568	0.474256	0.320215	-0.5488	-0.23764
434.1686	427.0592	525.1187	514.9304	541.5384	-0.32049	-0.13458	-0.15483	0.141709	0.109939
181.9394	180.387	118.7236	135.9646	137.267	0.202232	0.188583	0.183805	-0.43064	-0.26445
54.78024	57.00463	61.65402	60.61317	64.91377	-0.17668	-0.08392	-0.02668	0.084793	0.062109
1192.506	1265.014	1646.655	1525.112	1508.59	-0.15817	-0.17695	-0.09057	0.287432	0.176606
509.6253	507.9915	643.3741	637.4338	588.6304	-0.23443	-0.10411	-0.10361	0.232191	0.214568
1549.119	1877.25	810.9565	1071.147	994.3539	0.250717	0.432901	0.682461	-0.54467	-0.09933
61.99804	64.17873	50.60786	60.53965	61.64171	0.058243	0.011546	0.060772	-0.28679	-0.02277
675.7781	701.7582	516.1765	597.238	553.046	0.038821	0.138267	0.192989	-0.26313	-0.04031
113.6967	134.6373	89.82964	84.42104	89.01005	0.249953	0.111526	0.337195	-0.24061	-0.33743
2237.521	2007.065	2665.032	3694.989	3402.335	-0.32153	-0.19323	-0.35917	0.012569	0.519476
127.4135	103.8116	94.04926	79.45039	77.54383	0.031913	0.443218	0.185456	0.024426	-0.20044
5030.373	5504.902	5824.943	6597.707	5735.283	-0.00407	-0.14	-0.01075	0.068323	0.252153
10527.47	6769.188	10161.05	9928.332	8765.79	0.089263	0.061711	-0.57875	0.011134	-0.01168
760.3838	669.6211	560.7847	628.4298	622.0857	0.057624	0.182609	-7.73E-04	-0.26826	-0.09285
22.41824	25.55153	26.233	22.12618	20.32968	0.019026	-0.07681	0.109949	0.147941	-0.09651
28.04079	26.2633	25.43215	24.07318	24.26813	0.04128	0.136214	0.041511	-0.00246	-0.08361
142.8996	159.6787	184.8328	149.3393	175.1733	-0.11364	-0.0884	0.068845	0.280315	-0.03597
355.6461	296.4589	310.3025	290.2176	278.0397	0.064522	0.227187	-0.03442	0.029911	-0.06927
345.578	364.3485	405.7017	387.877	419.3612	-0.20479	-0.08818	-0.01083	0.135462	0.077456
228.9577	241.8674	271.7793	265.7643	296.4121	-0.23771	-0.13372	-0.05255	0.115954	0.075623
136.4378	110.201	136.1841	133.0823	132.5574	0.05309	0.00884	-0.29973	0.009445	-0.02489
850.0234	751.1546	1089.216	1078.243	1026.481	-0.10188	-0.11291	-0.29137	0.235617	0.223357
94.80123	171.1738	74.99251	89.19523	87.81155	0.101374	0.001842	0.875205	-0.36406	-0.06297
170.9813	192.5283	86.77016	66.29911	84.19886	0.443945	0.360442	0.541079	-0.66907	-1.05594
109.2508	100.6129	68.67851	81.87672	85.77818	0.093181	0.28442	0.190907	-0.37852	-0.1103
1308.114	1371.333	977.1038	1060.388	973.0524	0.331451	0.098247	0.166268	-0.33755	-0.2289
661.4872	592.3865	323.1535	392.8744	398.2261	0.254901	0.476834	0.294556	-0.58684	-0.28818
1847.502	1773.872	1281.378	1510	1384.302	0.082689	0.243199	0.183158	-0.30551	-0.04639

1654.503	1750.361	1104.575	1223.559	1257.031	0.163888	0.177611	0.221244	-0.46094	-0.25676
4751.456	2336.824	4272.665	4708.485	3539.084	0.028578	0.203697	-0.81475	0.035471	0.162542
380.6544	399.0475	476.7114	488.4506	457.3656	-0.34366	-0.1401	-0.07097	0.184081	0.21546
230.8728	263.9206	217.8206	213.939	214.8486	0.078777	0.045436	0.23845	-0.0373	-0.06272
69.20038	75.73644	68.6038	67.49624	76.87555	-0.18332	-0.00729	0.119999	-0.01927	-0.04301
516.0484	550.9193	525.9639	538.9954	544.0601	-0.12431	-0.02491	0.067176	0.002442	0.037036
303.3335	281.2836	302.354	339.5659	312.8737	-0.25479	0.009867	-0.09972	0.005931	0.163672
903.7491	1030.912	705.3524	757.2076	689.358	0.102337	0.16954	0.344697	-0.18727	-0.08061
347.5153	338.5283	232.3115	267.8964	231.1188	0.060252	0.337209	0.290743	-0.23487	-0.03637
338.2798	300.7588	276.3228	323.2016	229.8933	0.051044	0.134278	-0.03309	-0.16578	0.079602
386.4887	444.0187	453.7549	525.3804	546.8579	-0.14755	-0.136	0.061234	0.082771	0.283169
526.0908	495.4712	731.3782	575.4932	640.9485	-0.21184	0.031391	-0.04601	0.48411	0.151184
1692.771	1666.977	1593.284	1574.343	1743.013	-0.06913	0.066003	0.043667	-0.02084	-0.03918
305.5846	325.1247	353.6297	338.5815	365.5768	-0.1516	-0.11665	-0.02241	0.091939	0.035985
211.569	237.452	155.4739	210.9437	187.0837	-0.09118	0.067446	0.238288	-0.39002	0.065245
30.10796	27.74631	27.68566	31.75008	27.74662	0.066971	0.06298	-0.05342	-0.0571	0.141284
59.42557	64.50854	37.11855	30.54248	32.86828	0.805808	0.451928	0.520014	-0.23973	-0.50083
2133.534	2088.129	2448.634	2419.192	2469.265	-0.17613	-0.05417	-0.08619	0.140864	0.125462
310.296	325.8159	266.032	229.9646	240.0934	0.221215	0.161283	0.230462	-0.08649	-0.26615
65.354	69.3596	99.31409	123.4838	107.2255	-0.06921	-0.2904	-0.19683	0.314792	0.546992
1315.853	1211.494	964.0806	1163.404	956.4199	0.168379	0.132519	0.009246	-0.33043	-0.05474
107.7547	147.5	103.3841	110.1612	114.0216	-0.05876	-0.03394	0.412532	-0.08908	0.005756
1545.102	1669.233	1674.67	1642.789	1847.992	-0.08594	-0.08527	0.026962	0.027783	0.003109
1256.752	1368.924	1315.612	1352.143	1559.707	-0.13799	-0.08449	0.039851	-0.01972	0.019372
43.07605	35.82406	41.5194	39.81976	38.67298	0.208174	0.074734	-0.19014	0.022988	-0.05135
150.8679	142.9741	96.95156	116.0005	87.23782	0.154709	0.396333	0.30384	-0.27072	-0.00985
3411.793	3667.8	3692.016	3933.895	3723.772	-0.08746	-0.07859	0.025761	0.035498	0.122715
301.4387	328.4339	147.6304	211.9507	206.8711	0.021538	0.343879	0.401422	-0.75747	-0.16701
2130.296	2248.798	1480.111	1554.443	1504.976	0.064138	0.351894	0.428647	-0.1661	-0.10054
50.71358	46.29123	37.88101	45.35178	38.45703	0.038182	0.24757	0.125536	-0.16175	0.09978
123.4559	125.8949	160.0837	151.6694	142.203	-0.07876	-0.18394	-0.16281	0.189635	0.105783
2010.298	1684.416	1201.835	1059.921	1218.977	0.325799	0.554607	0.280355	-0.20628	-0.37215
58.79868	51.48974	97.76331	53.40966	72.64077	-0.36597	0.09645	-0.09413	0.749719	-0.05297
456.1694	479.5801	350.2613	515.0258	434.9198	-0.11816	0.022706	0.085456	-0.36349	0.184436
930.4446	1044.711	795.9971	872.129	904.572	0.082753	0.026562	0.186609	-0.20516	-0.06995
230.1853	339.9388	178.817	195.2852	193.8252	0.272287	0.128147	0.660052	-0.26144	-0.11098
399.023	364.5685	313.9034	312.4476	266.9304	0.016474	0.229839	0.099376	-0.13369	-0.12609
196.0796	184.8176	121.8599	125.8728	122.6092	0.217607	0.297264	0.223082	-0.44448	-0.35607
144.3728	133.673	157.8934	202.7282	171.8195	-0.09519	-0.03561	-0.14523	0.067983	0.439278
227.0655	243.0468	274.2816	349.7174	323.1021	-0.06631	-0.22349	-0.12676	0.035301	0.403761
691.959	705.7476	501.6021	521.7559	535.9091	0.203734	0.189333	0.218781	-0.28988	-0.21849
226.0027	128.7089	295.4699	229.8143	198.5588	-0.02865	0.019889	-0.78112	0.429323	0.033976
613.9896	688.8581	664.962	601.4282	598.7633	0.055521	-0.02997	0.135348	0.08464	-0.06024
1228.886	2178.279	772.9816	1009.357	873.8191	0.066705	0.230741	0.939514	-0.4613	-0.05848
83.02538	97.08427	94.11201	100.3206	98.53421	-0.2857	-0.17443	0.051869	0.006015	0.099106
1494.821	1516.335	1369.701	1499.356	1155.719	0.420241	0.02839	0.042036	-0.10194	0.026317
111.9384	124.8519	122.2512	129.1027	130.5666	-0.07633	-0.12166	0.03707	0.005566	0.084551

82.65774	100.9978	107.5708	90.89084	91.49692	-0.09826	-0.17824	0.118895	0.209013	-0.04146
140.3888	119.8455	94.37466	99.76175	118.8952	0.101044	0.206257	-0.02377	-0.41751	-0.29809
2801.274	2905.84	2549.323	2630.751	2399.343	0.045676	0.005567	0.05854	-0.13276	-0.08623
415.2368	483.6248	320.1946	277.0023	238.8585	0.277332	0.229303	0.408747	-0.14431	-0.35192
1316.626	1420.067	1656.224	1552.264	1570.503	-0.15831	-0.10753	0.00337	0.221996	0.121984
2205.195	2815.497	1986.226	1950.11	1817.782	-0.01944	0.094603	0.459576	-0.03776	-0.06667
139.3306	130.5296	113.2921	107.9689	113.841	0.373495	0.204741	0.108205	-0.09104	-0.16281
771.6068	766.1021	745.2468	692.1099	698.9587	0.071356	0.041392	0.031809	-0.00999	-0.11482
608.6063	602.0062	411.5472	480.9492	445.8112	0.085156	0.265359	0.247893	-0.31255	-0.08446
1470.745	658.7136	1237.12	938.5485	953.9846	0.689528	0.4122	-0.83875	0.084255	-0.25856
691.1746	841.0367	651.9326	658.7104	651.5938	0.22971	-0.07454	0.204583	-0.16812	-0.15977
55.3304	58.58614	50.63102	43.29033	50.34723	0.137787	0.022615	0.103462	-0.10733	-0.33125
58.59877	53.98821	94.62827	111.853	98.76814	-0.45389	-0.54898	-0.67762	0.168126	0.389821
53.78832	61.80632	90.0008	109.2846	91.93211	-0.51238	-0.54807	-0.33854	0.207182	0.434773
267.1704	317.1599	211.0317	231.7525	249.3104	0.101912	0.054822	0.294811	-0.3039	-0.15408
522.5707	569.1106	739.5037	682.7647	797.0376	-0.14278	-0.19037	-0.06706	0.305663	0.18809
287.349	297.143	405.4097	371.2336	379.704	-0.2166	-0.18491	-0.1283	0.319306	0.189465
129.6125	106.8562	100.9607	120.4134	122.991	0.067904	0.072028	-0.2062	-0.30933	-0.03506
2676.874	2187.299	861.2708	1217.041	1147.836	0.357022	0.777386	0.475476	-0.89379	-0.35041
262.5061	198.6638	167.3759	217.6997	195.8739	0.180567	0.349926	-0.03471	-0.29218	0.085787
192.2605	174.1485	237.4824	210.9976	224.3256	-0.1253	-0.06695	-0.20836	0.234995	0.068981
141.9838	133.298	96.24613	146.7167	123.4587	0.31678	0.066212	-0.01305	-0.53317	0.10448
19.60295	22.19281	21.88092	19.99318	21.9002	0.168859	-0.17013	0.011247	-0.01414	-0.1438
2491.013	2894.941	2159.087	2469.091	2344.714	-0.04159	0.053223	0.271274	-0.16196	0.040599
39.38107	36.52258	36.80021	40.5126	39.2812	0.213893	-0.00405	-0.1156	-0.1058	0.032471
213.8429	219.8774	359.8175	279.0265	264.6498	-0.12847	-0.09698	-0.0601	0.629934	0.240295
246.2582	261.944	213.8257	229.3191	225.5396	-0.04276	0.083873	0.172418	-0.11864	-0.02069
189.1753	180.8366	154.6342	163.0615	142.0894	0.002708	0.209596	0.148224	-0.07903	-0.01051
420.3439	464.4034	339.7734	411.5694	319.5732	0.158942	0.024618	0.164284	-0.29635	-0.00859
64.87732	85.07199	123.5884	126.2796	123.2693	-0.58921	-0.54647	-0.1421	0.391981	0.375498
201.9768	156.0311	263.8217	265.6903	303.2583	-0.08587	-0.19632	-0.59992	0.18719	0.134662
280.4677	328.7174	330.5182	288.1305	329.0886	-0.03204	-0.12281	0.104209	0.11533	-0.08246
157.7226	143.3198	235.2612	175.0514	189.3914	-0.12265	-0.07109	-0.2321	0.473065	0.068787
341.1784	402.6308	281.393	317.885	308.3829	0.430352	-0.0515	0.183642	-0.33706	-0.16327
66.75407	43.37175	76.4092	58.71986	48.13363	0.164201	0.050285	-0.57088	0.239935	-0.14161
176.9098	200.3554	135.2878	156.0024	134.586	-0.13857	0.193489	0.358188	-0.2027	0.0072
466.907	528.1711	334.6665	388.3905	355.6018	-0.08018	0.221083	0.379309	-0.26785	-0.04547
510.2599	513.1143	503.5183	542.6412	537.3513	-0.1449	-0.01649	-0.0105	-0.03591	0.069757
866.9299	903.233	989.8193	952.168	908.0587	-0.15693	-0.08212	-0.02048	0.111939	0.05616
1505.99	1347.292	937.8074	1096.357	1117.999	0.202162	0.300333	0.131706	-0.37966	-0.15475
74.37748	76.1611	92.41501	89.09034	93.09641	-0.02896	-0.11238	-0.07586	0.190058	0.147684
25.31679	23.70403	24.66951	21.79871	27.87632	0.05598	0.02406	-0.07075	-0.02083	-0.19293
864.0566	1049.724	712.7071	683.5342	735.4829	0.070468	0.135049	0.413745	-0.14655	-0.21075
1350.292	1682.727	1181.933	1178.497	1166.388	0.062105	0.054803	0.371773	-0.14518	-0.1442
38.91867	34.44542	43.40506	35.48661	35.78487	0.245324	0.036482	-0.13827	0.187533	-0.10202
144.1299	242.9853	123.2544	90.02002	93.2736	0.425097	0.048743	0.607478	-0.24073	-0.62203
36.47232	35.63047	30.8649	31.68812	29.7545	0.07005	0.075912	0.042958	-0.17072	-0.12609

79.76066	96.10445	107.3376	98.01708	110.9967	-0.07829	-0.3127	-0.03967	0.115457	-0.00892
119.9361	120.9075	105.5624	101.6543	103.5375	0.180007	0.041169	0.056886	-0.14469	-0.19733
354.264	366.9871	226.6711	313.9265	278.5088	0.13381	0.18289	0.23298	-0.4514	0.00839
309.6288	289.061	366.234	390.1698	375.2595	-0.19825	-0.05722	-0.16278	0.184071	0.252303
584.7615	473.9976	353.1495	467.4571	384.8843	0.063842	0.404971	0.117699	-0.32359	0.084914
222.5023	261.6884	242.5833	248.5238	246.2956	-0.1378	-0.0694	0.165674	0.053437	0.086046
147.0872	159.8908	165.7964	141.4828	157.1318	-0.07682	-0.07439	0.047663	0.099021	-0.1296
341.773	336.9806	255.3328	291.0512	243.8906	-0.03879	0.314691	0.29388	-0.11209	0.051313
305.988	239.1457	132.069	154.5836	141.9533	0.43064	0.709043	0.353593	-0.50504	-0.27334
177.4539	204.1825	215.953	199.5331	214.6389	-0.14016	-0.20327	-7.72E-04	0.080178	-0.03675
401.3437	310.34	504.8877	430.9928	392.9975	-0.01514	0.018686	-0.35035	0.339824	0.122576
71.63683	83.27837	59.06728	59.6559	72.34499	0.25203	-0.02123	0.190276	-0.31837	-0.30573
788.488	582.3905	424.7389	580.4411	548.3977	0.071829	0.410549	-0.01219	-0.53134	-0.02348
437.9681	406.9132	272.9236	360.1603	234.5277	0.255414	0.193848	0.092518	-0.53363	-0.09466
68.65614	66.02053	54.98669	56.42799	54.78553	0.08668	0.064182	0.008232	-0.25764	-0.23418
27.32102	37.72501	23.10946	22.86284	24.3886	0.381603	-0.0635	0.387644	-0.35909	-0.32485
2413.63	2817.045	2543.408	2349.17	2341.417	0.066952	-0.04481	0.178443	0.032511	-0.08577
27.11325	23.76956	22.725	22.59958	22.88568	0.006251	0.201117	0.010875	-0.05307	-0.06761
38.15626	45.52661	44.60457	42.63582	45.39927	0.144664	-0.22721	0.028581	-5.40E-04	-0.06848
127.8675	123.6964	103.7901	131.652	125.8988	-0.00128	0.025436	-0.0167	-0.2704	0.068557
158.5374	174.5834	186.4471	169.9165	190.6231	-0.36626	-0.07308	0.063507	0.144685	0.020404
22.48758	25.26477	24.21401	26.52889	25.2996	0.263099	-0.17043	-0.00636	-0.08015	0.066013
99.69553	90.09943	87.08718	79.99195	84.45586	0.847168	0.097222	-0.012	-0.07299	-0.20302
89.50697	78.37127	64.7411	78.26351	62.9723	0.225186	0.242445	0.054172	-0.21268	0.054404
307.9508	279.7074	217.9285	291.7903	298.3164	0.056576	0.113271	-0.02063	-0.3976	0.022608
221.8431	218.0709	267.1833	261.3464	259.2428	-0.06578	-0.10146	-0.13181	0.167602	0.13418
302.1034	158.2084	268.7715	251.5938	187.3624	0.17391	0.248113	-0.67627	0.060554	-0.00759
69.93444	307.2491	35.42575	75.40517	54.38148	-0.21841	0.090812	2.062346	-0.81941	0.161132
2628.2	2690.357	2856.564	3004.719	3051.356	-0.10102	-0.09657	-0.06015	0.024672	0.098039
27.26502	20.86982	23.39307	26.01633	24.19992	0.022189	0.154425	-0.23004	-0.07119	0.085692
94.2189	93.23517	111.0377	120.4277	121.8497	-0.12026	-0.17628	-0.19488	0.064429	0.179466
2970.131	3425.307	2907.606	3239.767	3140.359	-0.1456	-0.06225	0.148634	-0.08803	0.06798
322.5052	348.6653	238.8308	341.3904	261.2409	0.357618	-0.00266	0.106294	-0.51309	0.074485
141.6853	126.3898	140.928	152.8333	141.1892	-0.2033	0.029292	-0.13975	0.023639	0.138642
449.0605	354.6421	418.1742	497.9449	454.1991	-0.29845	0.09953	-0.24127	-0.009	0.243859
107.9922	27.87907	27.61358	30.43999	24.35459	2.05161	1.654286	-0.11151	-0.0869	0.054104
1008.387	1126.158	1243.567	1031.3	1130.282	-0.059	-0.11163	0.04808	0.183218	-0.07949
19.10224	17.75552	18.63518	16.15834	18.51511	0.192879	0.058261	-0.04206	0.026352	-0.18379
809.7961	691.5487	392.4631	603.0903	581.6998	0.2185	0.345429	0.090769	-0.78155	-0.097
203.0122	210.2359	172.5854	173.0003	163.9714	0.242667	0.056089	0.112575	-0.18152	-0.17559
20.31918	20.12867	16.39446	17.70163	17.07851	0.23847	0.122977	0.106256	-0.18976	-0.07504
95.77057	119.8345	99.61817	87.7966	87.36465	0.062919	-0.02563	0.291399	0.029355	-0.15822
132.501	109.5932	159.5735	132.3093	135.0538	-0.17334	3.56E-04	-0.27678	0.255701	-0.00897
120.2716	134.3706	115.8609	120.1508	120.9669	0.21366	-0.07548	0.085056	-0.14088	-0.07752
482.4289	589.398	413.1362	379.3137	389.6437	0.103605	0.164876	0.452373	-0.0517	-0.1845
915.9057	875.0269	1055.16	1033.806	1014.87	-0.09418	-0.02295	-0.09194	0.173506	0.149673
196.0427	208.056	165.6262	178.0106	157.0345	0.545173	0.056359	0.13317	-0.19289	-0.09162

290.1354	353.6245	164.595	164.3173	173.5533	0.318399	0.388495	0.637759	-0.47604	-0.42738
88.4227	90.1121	135.1974	153.7989	163.9461	-0.4521	-0.44371	-0.43943	0.139799	0.357355
18.16732	18.96491	16.29293	17.97614	16.74669	0.092056	0.014513	0.073655	-0.14565	-0.0023
906.4453	1149.338	420.9677	486.1644	441.7392	0.189495	0.555999	0.809734	-0.55944	-0.34593
93.53753	102.0794	79.81056	96.4401	87.43723	-0.01015	0.076787	0.204777	-0.15346	0.118893
37.18655	46.54811	46.30558	46.28909	46.75435	0.19789	-0.3273	-0.00226	-0.00562	-0.00998
127.4505	128.1284	98.64563	100.9085	100.8616	0.073382	0.163447	0.166637	-0.2103	-0.18629
1260.762	1341.802	1046.544	1110.725	1156.123	0.057556	0.048919	0.135851	-0.22103	-0.14055
481.2976	457.4943	303.5492	329.2719	326.0386	0.020521	0.287815	0.210507	-0.44023	-0.27002
95.64591	104.8768	110.9474	99.72414	114.2696	-0.06204	-0.11079	0.023503	0.1001	-0.05206
371.9162	340.3796	303.1434	270.2611	249.2069	0.106628	0.185755	0.056122	-0.13024	-0.2813
40.00088	41.53031	38.49204	44.60372	34.96566	0.037331	-0.01983	0.03845	-0.0721	0.136353
41.86191	41.99077	36.67331	33.56285	36.68575	0.107953	0.130977	0.135456	-0.05762	-0.18701
788.9976	659.092	862.1577	767.4456	652.6415	0.298298	0.038275	-0.21392	0.171218	0.010349
12.75405	12.76377	39.2201	47.29601	40.52587	-0.49272	-0.7349	-0.75429	0.831724	1.126487
608.493	465.5334	376.2665	575.6926	462.0649	0.046188	0.19623	-0.20686	-0.55804	0.114708
87.98801	83.95109	74.46883	76.95947	81.02828	0.257778	0.088629	0.016881	-0.15214	-0.10685
201.6083	248.9449	211.2015	170.8222	202.2635	0.015208	-0.03473	0.254313	0.032515	-0.28082
258.9049	328.2459	210.195	181.8043	237.8532	0.15691	0.054281	0.387742	-0.24932	-0.45686
540.8054	632.5998	428.6805	382.4287	460.9956	0.18831	0.129172	0.346721	-0.20722	-0.38933
266.7094	238.9932	155.2055	136.6163	142.78	0.342436	0.469651	0.306346	-0.31349	-0.50832
186.3931	178.4321	139.9473	127.1392	111.6414	0.049112	0.430269	0.350674	-7.67E-04	-0.1238
45.33089	47.00493	38.35488	42.54805	41.29981	0.024314	0.061784	0.113234	-0.17976	-0.02873
286.8054	184.0816	211.5347	148.3528	139.017	0.887058	0.627163	-0.02427	0.16214	-0.3168
289.014	293.0149	342.6722	353.3525	335.9405	-0.06445	-0.13511	-0.12076	0.108727	0.150897
465.1024	393.9889	196.3132	313.4891	319.8341	0.280389	0.386382	0.178466	-0.85847	-0.21224
1454.602	1478.374	1811.892	1780.863	1812.502	-0.16076	-0.13868	-0.11231	0.181828	0.15627
196.4709	194.5551	160.3174	160.0387	173.0376	0.332327	0.092492	0.087157	-0.19778	-0.20258
71.18911	69.99146	64.5967	57.13971	65.04856	0.002312	0.077956	0.053975	-0.06322	-0.23796
108.5963	132.9597	128.7877	83.19949	97.88508	0.248093	-0.01377	0.274667	0.219877	-0.43033
106.281	133.8588	106.704	77.84264	80.19752	0.267345	0.022529	0.316895	0.023614	-0.42961
130.623	138.6244	111.5059	131.282	127.6187	-0.00978	-9.55E-04	0.085195	-0.2275	0.003996
27.14808	25.97867	26.08604	25.4376	20.66994	0.122017	0.064981	0.006046	0.006542	-0.03866
155.5198	172.8059	119.0751	123.8626	131.465	0.327917	0.091533	0.239533	-0.29133	-0.26811
15.92566	14.00089	17.12687	21.38352	12.37873	-0.05051	0.034841	-0.15198	0.12762	0.454661
66.26794	55.51497	59.49514	73.6712	55.93773	0.053011	0.092896	-0.16555	-0.06527	0.23854
62.25705	67.05364	63.41507	61.6838	67.95573	0.199593	-0.05798	0.049648	-0.03092	-0.06958
181.5552	174.6323	128.0116	177.7461	149.1846	-0.01477	0.133674	0.092258	-0.37198	0.111273
741.6859	635.6258	534.8447	618.9558	500.3525	0.150565	0.260008	0.034739	-0.22418	-0.00134
163.3734	195.1584	145.2708	137.9619	152.7505	0.029919	0.059634	0.313205	-0.10977	-0.19396
89.09295	78.26696	74.61386	77.86429	81.59329	0.033784	0.179008	-0.00634	-0.07623	-0.01408
50.22916	61.81589	64.66285	59.65873	59.7323	0.144623	-0.28416	0.02641	0.090826	-0.02365
32.91531	38.91751	34.11688	38.14486	32.11021	0.180147	-0.10797	0.137979	-0.04897	0.110807
48.10506	52.39322	38.28147	35.12312	39.53478	0.106435	0.069332	0.192211	-0.275	-0.40496
116.9592	96.07769	122.9212	114.8908	104.348	-0.06687	0.080364	-0.20203	0.150922	0.050465
42.21951	32.68648	46.94788	40.47492	37.60936	-0.07284	0.043826	-0.32686	0.195164	-0.02974
48.82327	49.39727	49.6351	54.3954	55.49392	-0.20287	-0.04141	-0.02457	-0.01715	0.114787

3290.461	3635.683	2935.989	3322.42	2810.859	0.04258	-0.00737	0.13496	-0.17854	0.005928
67.91175	63.78077	67.11257	74.80291	68.53598	0.0711	-0.01699	-0.10884	-0.03415	0.121317
31.23337	32.11208	31.03374	32.5186	34.47495	-0.24059	-0.01394	0.027491	-0.02126	0.04483
15.41075	16.40724	15.62992	15.2197	14.62717	0.260662	-0.05339	0.039704	-0.03022	-0.07326
21.18464	19.09134	16.3565	17.174	22.01069	0.301495	0.147218	0.016624	-0.20759	-0.13782
190.4996	200.2739	158.5519	178.0464	165.3208	0.030622	0.096265	0.163203	-0.17076	-0.00222
45.75109	41.68998	34.89807	36.06163	36.57787	0.101249	0.227575	0.095167	-0.16536	-0.11268
303.2997	326.2058	237.11	208.215	252.5702	0.175685	0.113667	0.20757	-0.27127	-0.43401
91.6289	81.91978	70.82113	67.68269	64.27065	0.230121	0.222252	0.049208	-0.14613	-0.21906
147.7012	146.2648	92.60712	106.2278	97.43121	0.331999	0.400961	0.38747	-0.26837	-0.06722
42.97685	44.78259	34.73589	35.65155	33.91036	0.070825	0.131185	0.188991	-0.17706	-0.14943
71.35003	48.03738	72.93885	69.14025	65.74754	0.421779	0.005466	-0.57313	-3.09E-04	-0.03477
33.13121	34.69537	29.50965	29.70901	32.53397	0.152478	0.030092	0.096356	-0.14192	-0.127
28.17975	30.95184	27.96011	30.93131	32.55396	0.092883	-0.126	0.009876	-0.1376	0.006954
59.12629	52.6514	40.12722	54.01702	47.22939	0.056138	0.14954	-0.02722	-0.40917	0.019845
40.44984	37.85812	34.53961	35.80354	34.56286	0.226763	0.112386	0.020732	-0.1119	-0.06278
43.01184	44.33256	36.44349	31.66331	32.14576	0.282182	0.188492	0.2108	-0.05063	-0.24966
732.2944	855.1359	1216.279	1227.516	1162.428	-0.25548	-0.43395	-0.21604	0.293888	0.29483
43.59594	37.24763	37.54981	39.35393	40.49683	0.299896	0.071145	-0.15342	-0.15924	-0.07862
67.16331	80.31826	61.32921	53.49913	59.75708	0.428511	0.003722	0.26473	-0.11863	-0.34384
80.91618	121.3674	77.75671	91.68838	98.4176	-0.01041	-0.17556	0.390797	-0.24927	-0.00248
3220.542	2891.578	2385.337	2682.806	2660.664	0.077282	0.245087	0.08516	-0.18857	-0.01744
43.47224	30.21491	33.06472	31.46077	27.04813	0.275192	0.405512	-0.08981	0.038116	-0.03466
87.53586	81.62139	78.50268	72.21525	82.30029	0.018666	0.081542	-0.01953	-0.07744	-0.19959
47.77181	46.30237	39.73605	43.15018	44.78235	0.24569	0.082704	0.034747	-0.18377	-0.06348
85.62605	76.82779	37.31054	45.17256	46.16265	0.004631	0.750151	0.722974	-0.29193	-0.01365
23.68866	20.46578	20.69501	17.85611	18.76416	0.068996	0.247926	0.040553	0.052823	-0.15831
37.64051	34.27747	31.78434	37.29749	33.09975	0.121321	0.107596	-0.02379	-0.13452	0.095199
58.76778	55.5003	55.63171	64.35122	59.4469	-0.07444	0.023621	-0.05919	-0.05576	0.153268
2328.911	1736.344	1103.258	1483.138	1217.424	0.183062	0.601281	0.124173	-0.48728	-0.03857
4700.2	4752.187	3202.418	3612.997	3536.285	-0.00559	0.295211	0.313736	-0.25348	-0.09746
3854.023	3751.275	3951.638	3884.197	4037.519	-0.10831	0.013515	-0.02416	0.050782	0.025626
266.9868	243.8831	306.5861	311.3697	278.3866	-0.05294	-0.06766	-0.2022	0.131122	0.155735
1788.838	2121.763	1800.48	1695.43	1759.152	0.112561	-0.02321	0.22201	-0.01789	-0.10258
534.6643	505.8763	449.4262	489.0561	449.6304	0.007272	0.127767	0.048312	-0.12521	-0.0025
881.8416	699.2188	467.646	599.0209	606.6445	0.04509	0.36491	0.00687	-0.60656	-0.20393
410.5365	450.0295	560.8821	820.6605	705.5991	-0.39185	-0.29734	-0.13999	0.150807	0.717844
1481.515	909.393	1433.894	1515.736	1175.29	-0.07561	0.106552	-0.59687	0.040337	0.149305
50.64467	21.5472	11.94383	11.38859	13.20416	0.332212	1.650303	0.313653	-0.29225	-0.31781
215.0812	199.8784	164.6057	174.2849	176.1191	0.094429	0.136238	0.032375	-0.25654	-0.16928
127.1299	105.8814	77.97643	81.69508	77.67521	-0.02514	0.504004	0.256197	-0.17054	-0.09654
5132.692	5186.521	3636.701	3524.791	3753.992	0.09819	0.213639	0.232232	-0.28046	-0.33009
723.2817	790.0164	865.8635	719.6152	704.3967	-0.02521	-0.0483	0.077721	0.211616	-0.06392
245.9858	244.9691	241.2983	207.8974	232.6249	0.196547	0.05025	0.041268	0.022204	-0.1925
669.7321	701.1917	788.7311	717.4898	826.668	-0.18358	-0.09692	-0.03283	0.135379	0.00278
49.74547	37.60356	35.45888	34.50923	40.11349	0.139743	0.31886	-0.08376	-0.18325	-0.22519
361.7177	364.505	372.9099	425.139	389.6785	-0.1596	-0.06481	-0.05255	-0.02182	0.166034

36.24199	36.23213	34.87163	36.25442	38.09418	0.179349	-0.00769	-0.00872	-0.06509	-0.00914
200.6845	189.6161	165.5522	167.8316	165.8375	0.124804	0.173981	0.09141	-0.10542	-0.08465
179.0737	200.1669	156.8515	175.0447	155.1211	-0.03291	0.087982	0.247111	-0.09789	0.058733
182.9666	203.8152	170.332	164.345	154.4325	0.037698	-4.43E-04	0.153799	-0.10553	-0.17622
41.50365	40.03045	38.0137	38.45226	33.67189	0.008292	0.128614	0.07647	0.001421	0.020422
44.46115	41.33461	46.99103	48.54468	51.84111	-0.13701	-0.0385	-0.14265	0.042118	0.090499
386.7323	450.2108	201.8994	190.8348	224.0496	0.531335	0.546492	0.67741	-0.39059	-0.53531
356.734	374.6949	295.1808	300.9988	288.8436	0.041919	0.18664	0.26078	-0.094	-0.05426
107.289	110.8585	108.4521	109.128	102.8476	0.127918	-0.02931	0.018304	-0.01329	-0.00445
2870.176	2895.779	2750.856	2633.488	2625.358	0.02177	0.053227	0.067038	-0.00726	-0.06995
915.5166	1043.984	798.0837	844.4341	794.7849	0.026758	0.05788	0.246921	-0.14365	-0.05803
240.6052	205.9417	240.7398	205.3365	183.2467	0.115486	0.156024	-0.07497	0.149292	-0.07318
918.6597	976.9707	960.9979	1060.115	1101.672	-0.17331	-0.10147	-0.01551	-0.03773	0.1058
641.0904	739.3438	528.4593	618.9548	594.644	0.054379	0.024791	0.228331	-0.26267	-0.0334
1435.851	2226.895	1277.651	1265.388	1387.651	-0.18113	0.090002	0.719386	-0.09412	-0.13203
906.6848	873.5588	639.3992	715.7818	700.5128	0.087701	0.183169	0.133632	-0.32166	-0.16485
45.49054	41.58743	37.76367	37.42	36.62012	0.126196	0.178815	0.049805	-0.08946	-0.10251
401.8152	447.1974	339.8226	322.3603	295.3308	0.028027	0.139362	0.297454	-0.0985	-0.17155
1262.308	1477.203	1141.871	1096.595	1068.209	0.075872	0.018012	0.246762	-0.12359	-0.19407
2268.415	1564.343	996.114	1302.944	1192.211	0.170714	0.715078	0.157322	-0.50687	-0.06956
745.1348	472.7214	401.4846	381.8701	329.6052	0.722345	0.770198	0.111215	-0.14242	-0.18375
552.0417	515.5683	388.0099	480.74	331.4402	0.182499	0.193381	0.106079	-0.36091	0.003487
259.9159	217.9877	221.0936	161.864	164.7097	0.335464	0.295606	0.052968	0.069099	-0.38009
166.6637	190.9047	155.2738	157.0923	147.8892	-0.01391	0.044477	0.238955	-0.05616	-0.03707
379.7735	410.0782	441.4561	416.1907	417.5681	-0.06419	-0.12456	-0.01522	0.09165	0.006765
107.1777	137.7444	79.09457	92.75195	93.61481	0.097069	0.020841	0.374491	-0.4206	-0.20454
252.2731	151.6114	237.0805	228.9605	158.9456	0.153809	0.066016	-0.65699	-0.02397	-0.05781
25.19157	26.73223	25.13944	27.56134	29.58366	0.229958	-0.12699	-0.03909	-0.13236	0.002973
55.05001	46.1471	32.36725	35.62116	34.05476	0.423647	0.39614	0.14926	-0.38356	-0.25353
176.7515	166.4025	124.684	137.1409	122.744	0.213441	0.228444	0.134205	-0.28945	-0.1365
30.67703	30.23805	40.10707	37.81655	32.04358	-0.10879	-0.01293	-0.03313	0.368257	0.268702
1108.71	995.72	715.2347	907.2575	820.5186	0.180957	0.28419	0.125783	-0.3756	-0.00548
2392.601	3056.156	1671.869	1553.48	1472.039	0.167255	0.303023	0.647294	-0.26511	-0.31936
140.6695	122.6079	145.7119	173.164	165.3321	-0.15793	-0.03543	-0.23787	-0.00422	0.26278
270.6182	208.6415	147.0694	172.0047	156.3993	0.06274	0.561818	0.191368	-0.33458	-0.08859
317.0398	297.6438	218.1438	250.2503	221.2197	0.039021	0.331929	0.229515	-0.21494	-0.01143
46.72887	52.78023	38.50463	46.98201	37.79268	0.098056	0.070965	0.259838	-0.19711	0.094032
185.0101	150.6518	204.3391	196.4385	188.1386	-0.10278	0.03419	-0.26231	0.16849	0.116611
283.344	281.6273	285.7531	326.9751	273.632	-0.11441	0.006603	-0.00312	0.019058	0.207862
326.3797	250.5594	176.9702	221.7184	207.2016	0.675891	0.451185	0.046537	-0.44919	-0.11008
691.8937	827.4846	552.4266	504.4579	584.3157	0.103578	0.158074	0.414087	-0.17236	-0.31003
361.3678	360.3717	270.1133	301.0385	277.6712	0.147114	0.162518	0.158746	-0.27246	-0.1065
73.4558	63.61386	81.12473	88.53632	79.5994	-0.1895	-0.0228	-0.23425	0.117167	0.239499
224.0759	253.7794	171.3364	202.8623	198.0383	-0.03477	0.101052	0.281615	-0.29258	-0.04112
1273.199	1208.377	714.6257	943.0788	736.8535	0.195895	0.360889	0.293925	-0.50562	-0.06508
707.8958	877.4185	601.4963	618.0872	599.809	-0.00476	0.093723	0.393381	-0.15066	-0.09787
1459.796	732.8245	620.4693	724.3239	663.8256	0.315369	0.7749	-0.35103	-0.45904	-0.23985

177.4334	149.7283	238.3508	257.8584	248.116	-0.55436	-0.28186	-0.55586	0.147272	0.257263
477.8134	446.1225	425.9594	422.2082	444.0425	-0.0589	0.119941	0.021441	-0.045	-0.05937
351.6886	373.1635	326.8696	376.8657	336.4581	-0.17039	0.005259	0.092173	-0.09912	0.106106
341.7999	172.674	148.3197	216.6158	203.3949	0.058031	0.635275	-0.33179	-0.58035	0.016301
1658.608	1908.564	1537.215	1417.723	1508.589	-0.08051	0.096545	0.302853	-0.00948	-0.13173
837.6137	812.5707	809.5262	1030.157	895.6503	-0.43403	-0.00555	-0.04652	-0.06199	0.284791
1500.341	1493.971	1840.851	1741.156	1872.018	-0.14623	-0.06546	-0.06913	0.222606	0.14276
390.5382	422.0682	124.0072	185.5093	145.6821	0.979919	0.799128	0.767566	-1.22832	-0.27479
341.7872	399.31	430.9859	378.4682	412.5351	-0.12895	-0.18318	0.04224	0.150564	-0.03511
213.8692	219.8307	250.8142	249.6965	237.9101	-0.21379	-0.06053	-0.02182	0.168863	0.16256
670.5337	640.976	762.5361	760.2544	743.3094	-0.1739	-0.02287	-0.09093	0.161769	0.14662
402.6294	393.6805	303.9658	344.769	354.3099	0.056926	0.096781	0.061885	-0.31861	-0.13291
298.4474	305.9068	316.4404	359.2038	341.6591	-0.13728	-0.08458	-0.04975	-0.00139	0.177364
146.0571	167.0084	167.6472	159.6523	159.3076	-0.13933	-0.09645	0.097352	0.099678	0.031708
24.7786	27.63062	25.18257	23.38298	23.93696	0.182197	-0.04198	0.120498	-0.01144	-0.11958
462.0941	403.7529	296.6895	369.8672	313.4739	0.0926	0.343705	0.14545	-0.31896	0.025012
227.4106	232.6564	257.3684	247.5488	278.4915	-0.1539	-0.04379	-0.00901	0.134071	0.070491
2105.939	2660.837	1853.83	1938.562	2075.015	0.0563	-0.01565	0.316885	-0.20192	-0.14045
1201.165	1510.785	1090.935	1059.133	1148.257	0.08587	0.026267	0.351768	-0.11938	-0.15274
1433.641	1425.288	1457.392	1433.701	1422.681	-0.1484	0.001991	-0.00628	0.024647	7.79E-04
1752.729	1441.072	2107.738	2006.366	2116.143	0.0067	-0.09838	-0.37953	0.168796	0.085807
190.5442	168.6069	114.9351	141.2957	142.6367	0.370694	0.277031	0.10651	-0.46968	-0.15098
408.8078	420.3377	425.2006	468.3243	480.8274	-0.06711	-0.05062	-0.01015	0.004514	0.143071
991.9578	988.8474	1266.184	1183.612	1254.322	-0.14228	-0.10356	-0.10599	0.237865	0.152392
3665.733	3949.545	3376.239	3259.708	3464.386	-0.12559	0.100379	0.214545	-0.01266	-0.06376
193.6115	169.1254	186.806	185.3928	181.4076	-0.13592	0.119088	-0.07605	0.067486	0.0563
624.7714	613.2737	496.7378	589.9523	556.8322	0.096182	0.1143	0.092729	-0.21248	0.036174
531.7171	516.2927	555.8962	520.3864	535.8735	-0.15417	0.019322	-0.02413	0.082244	-0.01509
257.0247	293.2785	361.8868	293.8125	334.6013	-0.34791	-0.12669	0.064107	0.336156	0.057136
1236.29	1370.925	1220.513	1130.115	1209.795	-0.08077	0.002245	0.148171	-0.01538	-0.12622
1131.773	775.6474	525.4562	736.0159	713.6108	0.112352	0.496524	-0.08776	-0.64469	-0.12358
1222.164	1272.607	1392.171	1324.292	1462.573	-0.14286	-0.06842	-0.01061	0.120057	0.03996
1281.891	1238.222	937.3901	859.5092	948.2295	0.127817	0.235298	0.185689	-0.2282	-0.34758
2642.177	3008.192	2374.021	2452.31	2225.513	-0.11259	0.07561	0.268626	-0.07381	-0.0263
1072.605	1194.454	923.3393	966.2233	927.667	-0.00527	0.127961	0.28647	-0.08783	-0.0198
72.72767	69.32089	87.78275	87.66794	75.64999	-0.20529	-0.07846	-0.1543	0.188008	0.19296
341.3293	331.7868	450.4808	387.2882	393.2936	-0.14555	-0.11024	-0.15154	0.288717	0.075566
104.4339	107.5464	95.44699	89.37986	98.48841	0.147012	0.060273	0.102581	-0.0705	-0.16515
38.18566	39.01662	41.29174	37.14763	46.04667	-0.1282	-0.0512	-0.01984	0.05908	-0.09591
48.16093	39.37952	34.19608	30.48493	31.2764	0.866051	0.401556	0.091576	-0.0978	-0.26227
115.8489	91.59055	76.22579	76.40051	67.98617	0.875336	0.359408	-0.01401	-0.25719	-0.26741
588.9648	655.5225	687.7813	709.4785	680.1555	-0.14393	-0.14754	0.007327	0.076868	0.121076
131.6723	150.4065	139.6982	146.2831	143.9122	-0.11243	-0.10768	0.083385	-0.02363	0.044735
138.3335	137.8183	143.7476	161.0529	170.4829	-0.09025	-0.04929	-0.05517	0.006214	0.165298
380.5731	318.6004	207.1801	175.2882	199.1909	0.258072	0.31039	0.058058	-0.64412	-0.86441
129.9633	139.0271	176.2096	176.7739	166.3228	-0.1124	-0.33326	-0.22896	0.111282	0.11833
2141.244	2340.533	1900.56	1897.421	1792.349	0.015413	0.105703	0.222647	-0.0659	-0.07449

237.0215	241.6385	307.141	266.3132	292.4287	-0.0212	-0.1129	-0.08242	0.262307	0.053328
375.194	523.2257	332.5415	320.3441	353.7704	-0.07314	0.046186	0.519956	-0.1372	-0.19492
1542.529	1430.817	1209.765	1360.584	1301.535	0.032395	0.129663	0.01803	-0.23144	-0.0549
1231.665	1134.062	1557.031	1245.51	1451.459	-0.20717	-0.00924	-0.13075	0.30883	0.008849
484.8618	482.1969	561.4107	504.8692	517.3808	-0.08298	-0.03834	-0.04637	0.169459	0.017361
152.7129	142.314	189.1243	186.8301	186.2029	-0.03328	-0.13152	-0.23696	0.173503	0.155242
179.2308	180.5401	193.5781	210.1192	196.8899	-0.12485	-0.06266	-0.05498	0.048333	0.164407
446.2169	469.4735	591.8743	515.0064	565.5057	-0.21278	-0.05968	0.01425	0.337111	0.129489
672.1829	625.9128	494.4642	435.4903	532.7681	0.238272	0.197993	0.103064	-0.23705	-0.42005
716.2319	773.4523	546.6929	554.7393	564.8319	0.169041	0.095731	0.204112	-0.3132	-0.29809
387.4944	388.4293	387.8635	404.1475	463.959	0.019686	-0.05139	-0.04858	-0.05003	0.009647
58.27139	67.14323	58.85884	77.20782	69.33437	0.065336	-0.19169	0.018086	-0.17793	0.21087
151.9137	170.6103	151.0983	161.1059	140.098	-0.08995	-0.00936	0.152477	-0.01875	0.075207
119.3344	99.43829	201.7427	134.9955	164.7771	-0.18126	-0.07448	-0.35481	0.616604	0.094179
384.3389	382.4796	194.0256	283.8058	232.1182	0.215348	0.360586	0.331863	-0.6962	-0.07056
91.40839	87.54696	64.24113	80.52006	75.55252	0.079172	0.119959	0.058915	-0.39217	-0.0624
207.9898	239.982	223.1751	215.3049	228.0578	-0.0017	-0.10947	0.095787	-0.00807	-0.0608
207.7472	231.5195	228.7553	254.1271	255.952	-0.36232	-0.15736	0.004143	-0.01661	0.131116
3975.978	4151.232	3831.836	3686.1	3702.892	0.033052	0.028745	0.091068	-0.02401	-0.08195
477.4607	453.7549	445.0886	480.0937	406.6573	0.248088	0.066561	-0.00423	-0.0358	0.072021
63.60476	71.10764	44.0592	50.25426	55.86887	0.461064	0.017328	0.188715	-0.50411	-0.34066
586.1365	625.2773	477.713	508.6696	482.4051	0.214214	-0.01562	0.077748	-0.33224	-0.23981
63.62001	109.6176	54.29356	62.96979	71.16805	0.33669	-0.07767	0.707193	-0.31132	-0.08834
181.7803	205.7393	165.1381	152.5721	151.5291	0.027775	0.039103	0.213535	-0.11652	-0.22216
127.5856	110.2632	142.7126	124.5491	138.6549	-0.1042	0.040853	-0.16669	0.201077	0.008821
563.6826	517.7692	315.743	491.6513	491.1001	-0.06298	0.206954	0.091868	-0.6616	0.00324
305.8168	380.6642	402.2348	388.6435	373.6874	-0.39564	-0.27144	0.059635	0.135981	0.086795
134.2626	114.5997	155.2312	119.4948	113.1669	0.11663	0.112896	-0.11628	0.3243	-0.06719
173.2557	166.1887	145.7869	147.3272	155.6238	0.106105	0.085855	0.025864	-0.16567	-0.15083
584.2504	638.6814	599.015	524.3778	601.482	0.006938	-0.03048	0.096944	0.005691	-0.19029
110.9989	100.1085	112.6704	104.6528	131.2804	0.091257	0.017629	-0.13105	0.037788	-0.06815
2658.431	2683.929	2430.354	2222.56	2299.548	0.26319	0.071192	0.082817	-0.05825	-0.18728
102.1504	104.9178	89.47483	78.4842	77.90958	0.145604	0.219933	0.266888	0.02129	-0.15592
31.61638	32.88097	27.91357	35.73856	31.92221	0.024417	-0.05311	0.002938	-0.23208	0.121593
77.25839	74.35611	93.75091	91.93707	93.45519	0.073237	-0.15976	-0.21313	0.117801	0.088818
109.9723	145.8284	414.3097	401.8742	388.5541	-1.36621	-1.02649	-0.61895	0.880511	0.835797
40.73026	43.79828	32.00271	35.98175	36.74774	0.024517	0.068432	0.174879	-0.29472	-0.11729
141.1529	140.6018	186.636	173.5834	159.7886	-0.17039	-0.13803	-0.1519	0.259385	0.160108
1836.547	1245.667	2309.158	2194.995	1224.097	0.606952	0.010435	-0.5786	0.336517	0.279324
152.6657	173.8672	270.1683	152.6219	175.3773	-0.13208	-0.13543	0.050481	0.640796	-0.15788
320.0652	359.3284	263.2755	270.4596	265.2145	0.071649	0.118539	0.277906	-0.1661	-0.12327
200.4754	216.6003	242.0748	220.4118	245.0457	-0.08387	-0.12202	-0.01118	0.15058	0.011777
94.28971	109.829	76.67193	70.64082	73.76978	0.274334	0.141263	0.356206	-0.13943	-0.2829
52.63714	46.95308	49.35729	55.48539	43.35129	-0.01895	0.085489	-0.0801	-0.00761	0.160341
127.0437	71.58916	118.1512	121.0592	103.0422	0.272904	0.194451	-0.62824	0.048507	0.134508
204.282	288.5711	125.7789	148.2253	144.365	0.156379	0.332462	0.715551	-0.38222	-0.13075
2220.956	2290.112	1728.126	1763.584	1880.293	0.153777	0.075331	0.11891	-0.29162	-0.26832

672.7993	604.5444	564.4894	642.7404	667.9546	0.125651	0.071208	-0.08354	-0.19008	0.002104
100.0812	80.05685	86.7811	80.87891	71.50803	-0.10223	0.256674	-0.06435	0.050813	-0.04852
25.16523	22.59043	20.84095	21.16355	22.54177	0.13774	0.14372	-0.00998	-0.12804	-0.11262
42.46064	43.41856	45.42938	40.17422	40.62266	0.011733	-0.00729	0.025237	0.089017	-0.08718
82.04953	87.63839	55.06366	68.42793	65.40252	0.112522	0.221265	0.308543	-0.35729	-0.03232
67.91446	79.43366	66.58893	67.81387	71.30113	0.220219	-0.05944	0.165782	-0.08852	-0.06064
87.32373	92.90102	73.67859	81.37382	89.56815	0.027195	-0.00192	0.088258	-0.25649	-0.10426
35.65073	95.8157	26.47511	43.70039	33.84781	-0.08891	0.065771	1.322666	-0.36979	0.287234
37.93582	26.83349	29.30525	29.28514	26.25261	0.248474	0.352275	-0.10764	0.012147	0.018414
388.0022	434.3981	303.9395	323.4158	290.1475	0.052813	0.022191	0.175529	-0.35475	-0.25429
770.2382	854.4407	699.8452	682.5645	724.4167	0.007837	0.069517	0.219379	-0.08041	-0.10535
807.5799	814.7039	711.0338	806.8373	773.8513	-0.06739	0.032041	0.044161	-0.15508	0.030611
143.5574	147.6938	112.6369	118.9343	129.6501	0.058313	0.095218	0.134778	-0.26697	-0.18121
5221.297	5039.831	4096.631	4977.232	4426.19	-0.02652	0.091574	0.041502	-0.26832	0.021781
94.85728	96.16714	82.37635	71.75629	67.62415	-0.01774	0.17027	0.186478	-0.03779	-0.23475
18.90725	20.3567	17.83228	20.15669	19.58486	0.240659	-0.08085	0.029385	-0.16457	0.010792
245.6982	205.6317	157.0708	188.8255	197.6604	0.194142	0.242317	-0.0123	-0.41562	-0.13311
78.79417	86.26275	68.22331	71.57795	75.96854	0.274441	0.004485	0.136494	-0.20796	-0.13818
47.26851	46.31377	45.13165	45.86477	47.58815	0.16144	0.029296	-0.00154	-0.03728	-0.01428
19.60278	21.92126	18.34115	23.07771	19.43408	-0.00819	-0.08025	0.081899	-0.1865	0.155197
37.37343	43.19819	32.30467	34.63654	33.44994	0.009603	0.074423	0.264601	-0.13819	-0.03543
1373.891	1329.873	1125.223	1125.849	1113.106	0.014991	0.183571	0.134369	-0.10319	-0.11049
174.8282	166.5553	139.7937	126.8713	137.7858	0.02917	0.1266	0.057587	-0.21915	-0.34984
58.62103	60.73694	50.55992	59.64377	56.89702	0.091567	-0.01755	0.028372	-0.23359	0.005817
94.91995	88.30109	82.96368	77.15617	77.58497	0.169291	0.145816	0.032915	-0.05345	-0.15536
87.26108	98.95011	78.06833	80.27839	73.53533	-0.04099	0.097521	0.278866	-0.05785	-0.01831
75.13294	64.70808	89.15804	102.4048	107.647	-0.54166	-0.17015	-0.38625	0.088555	0.270537
50.90467	55.27959	52.31798	54.36507	58.69394	0.059783	-0.09359	0.025708	-0.0547	0.001984
26.07194	24.72567	23.19869	23.17222	23.74916	0.204441	0.084825	0.008346	-0.0849	-0.08579
23.69582	23.25386	22.65661	21.76976	20.69371	0.211561	0.049244	0.023418	-0.0161	-0.07401
403.7147	412.5717	436.3047	433.6463	451.0602	-0.11947	-0.06837	-0.0372	0.043964	0.034661
304.1294	346.6867	293.1596	277.7051	302.4971	0.061603	5.91E-04	0.189658	-0.05171	-0.13379
1399.995	1512.086	1570.341	1626.059	1829.889	-0.04804	-0.14176	-0.02699	0.024056	0.07582
83.70269	96.35373	75.39991	101.5179	87.80442	0.042811	-0.0875	0.119133	-0.23438	0.188818
24.2459	27.10899	24.9633	22.22412	23.67625	-0.09762	0.05439	0.216066	0.099453	-0.06821
59.37772	49.07992	37.98288	36.89558	43.44264	0.144253	0.355147	0.079468	-0.29121	-0.35049
42.13306	45.96093	55.17442	40.51579	49.1689	0.026703	-0.18605	-0.06252	0.203319	-0.25632
30.09848	27.72018	25.71328	26.56188	25.95992	0.009317	0.134288	0.015964	-0.09581	-0.04634
34.65787	31.25956	26.65613	36.20598	33.48797	-0.18233	0.101289	-0.04755	-0.27977	0.149433
201.2598	207.2441	137.5474	154.5587	147.5795	0.282122	0.212746	0.271389	-0.33216	-0.16402
4021.656	4465.487	3688.898	3646.754	3907.669	0.072785	0.070521	0.222269	-0.05256	-0.069
293.5923	322.3026	345.2842	364.5463	337.3318	-0.18208	-0.16931	-0.03371	0.066043	0.139667
1666.19	1926.757	1406.484	1373.334	1159.154	0.043475	0.043089	0.241937	-0.21755	-0.26212
1539.284	1739.026	1757.748	1760.499	1766.841	-0.06514	-0.15664	0.020749	0.036108	0.037779
851.5174	713.9276	503.4649	729.6799	676.6523	-0.05035	0.26082	0.015301	-0.50838	0.037345
658.3127	754.4591	930.4609	856.7981	826.2446	-0.11547	-0.21983	-0.02464	0.270652	0.159989
251.6203	281.5921	228.4623	215.1655	229.8181	0.079576	0.035885	0.187491	-0.10534	-0.19494

363.404	313.7198	385.1946	354.0155	380.611	-0.1328	0.018852	-0.19506	0.100193	-0.01975
353.4062	357.8788	303.9237	323.6696	289.2738	0.199349	0.067076	0.086386	-0.14887	-0.06054
519.5732	418.3742	430.877	436.6553	429.8514	0.210606	0.199265	-0.10658	-0.06475	-0.04807
386.1542	390.9662	305.7734	308.5252	376.5664	0.121798	0.074562	0.091367	-0.27594	-0.25463
338.9312	357.7474	310.6891	267.0547	284.1888	-0.05028	0.117194	0.186776	-0.01409	-0.23604
430.206	507.9627	590.0879	520.7975	618.945	-0.1541	-0.23893	-7.38E-04	0.199807	0.035042
216.8312	168.549	213.2199	196.7688	195.016	0.102562	0.081595	-0.28173	0.04979	-0.0598
862.5762	1036.693	741.6348	783.6184	767.2111	-0.07255	0.102768	0.367684	-0.11591	-0.03732
380.3937	423.1864	325.2015	303.0086	338.7155	0.02737	0.094851	0.249436	-0.13168	-0.2411
89.95157	76.32134	52.76593	53.00168	46.94267	0.161458	0.310959	0.090412	-0.4832	-0.45332
333.5735	337.3102	375.9739	333.8548	327.0123	0.059319	-0.03944	-0.02392	0.132671	-0.03866
382.9302	294.4319	194.1292	219.6033	234.832	0.242399	0.55827	0.193704	-0.41865	-0.22913
81.786	86.90958	85.74157	77.59284	57.0746	-0.19707	0.041841	0.126322	0.078631	-0.04988
628.4769	646.6965	676.0859	644.7194	718.4432	-0.02435	-0.04668	-0.00547	0.058268	-0.01127
112.7725	97.43552	81.76787	95.88522	67.20983	0.242554	0.247434	0.045399	-0.21445	0.018926
746.3737	698.0615	589.4282	694.4338	712.8431	-0.03938	0.088066	-0.01112	-0.25994	-0.01631
238.9979	262.0021	259.8484	274.5832	283.6969	-0.1313	-0.1067	0.022854	0.013914	0.091739
1210.059	1041.217	839.6431	870.0898	829.9971	0.074303	0.357285	0.146268	-0.17784	-0.11941
799.9609	807.3393	614.8314	644.793	645.8135	0.060022	0.218287	0.233264	-0.15998	-0.10654
1577.1	1541.862	1952.891	1690.815	1691.469	-0.11186	-0.04481	-0.0749	0.264469	0.052629
199.0647	205.9058	147.8655	174.5761	177.7399	0.084614	0.11509	0.159007	-0.31624	-0.08344
2398.319	1386.712	1083.16	1601.953	1305.085	-0.0677	0.690878	-0.14198	-0.51667	0.066897
35.98668	34.91351	44.01406	41.51259	36.24137	0.72569	-0.15506	-0.20921	0.104563	0.043434
82.27895	74.52514	61.08638	62.21389	61.44971	0.165896	0.288344	0.141104	-0.14325	-0.1036
651.6772	667.968	904.4213	721.9414	743.8716	-0.24317	-0.03894	-0.0106	0.410577	0.093845
1228.851	1256.303	1365.476	1399.887	1391.242	-0.08264	-0.04896	-0.01541	0.102413	0.137552
28.31373	29.33749	34.50699	28.78747	25.62867	0.076202	0.012287	0.06673	0.297928	0.036313
1433.109	1623.471	1665.493	1560.248	1548.371	-0.02279	-0.14451	0.035763	0.072065	-0.02229
212.84	203.1108	189.2479	186.2708	188.6074	0.296954	0.102921	0.034334	-0.06654	-0.09532
576.7551	558.8599	614.5655	623.983	551.7165	-0.02946	0.03038	-0.01339	0.121527	0.142612
548.4431	819.3823	602.2398	598.865	607.9606	-0.03038	-0.15978	0.41519	-0.01798	-0.03772
43.12556	47.33477	38.30772	34.32807	37.74175	0.065966	0.092408	0.227078	-0.08097	-0.2418
579.147	583.5327	605.1298	974.9341	844.9905	0.094299	-0.29486	-0.27467	-0.25564	0.473688
910.8294	913.4434	1016.756	978.7471	891.9853	-0.04011	-0.02251	-0.01954	0.135566	0.080495
612.71	598.5052	476.8562	587.5593	531.9471	0.060772	0.078948	0.045576	-0.29241	0.012053
359.013	384.906	475.0686	594.249	538.6475	-0.24087	-0.17231	-0.07823	0.209626	0.545614
204.6693	189.818	149.6875	168.1715	185.5172	0.42631	0.120132	0.003169	-0.32518	-0.19155
562.1243	595.4341	542.3196	633.2501	610.0809	-0.15105	-0.03357	0.050433	-0.08486	0.137148
543.2863	391.9789	402.1574	491.5004	437.6481	0.098302	0.221597	-0.25495	-0.24573	0.077266
225.2369	198.3073	230.7594	245.7356	242.1659	-0.08676	0.028165	-0.15884	0.059	0.153451
31.74974	30.22945	29.55479	37.17923	33.66843	-0.03008	-0.00167	-0.07242	-0.1125	0.222865
152.7206	171.4659	146.4554	121.1675	127.2308	0.046736	0.068385	0.229336	0.005541	-0.26882
363.6469	433.447	371.4052	345.7002	333.9557	0.20743	-0.06577	0.190664	-0.03126	-0.15135
582.2405	597.4488	655.6466	619.0537	641.593	-0.28675	-0.082	-0.04852	0.089741	0.005972
173.2313	207.0293	232.6172	207.9207	219.2279	-0.11905	-0.23605	0.021735	0.185405	0.026871
729.3411	826.0562	839.9917	767.2322	881.874	-0.14466	-0.10497	0.070992	0.098642	-0.03024
82.95741	93.20825	104.6083	94.66862	104.5839	-0.10066	-0.1869	-0.01899	0.136723	0.004054

195.9097	171.9631	159.298	224.9385	150.7332	0.330379	0.02358	-0.15782	-0.26741	0.221522
66.05924	53.17458	76.83219	83.06432	86.23762	-0.05832	-0.16094	-0.48534	0.06142	0.172329
120.1886	108.8926	153.69	134.8993	133.284	-0.19982	0.011186	-0.13225	0.363552	0.165956
288.92	266.5214	243.3205	397.0576	293.272	-0.06714	0.051745	-0.06726	-0.19877	0.481964
84.13811	83.5321	80.2516	98.59715	92.19624	0.036824	-0.08754	-0.09787	-0.15869	0.142361
1253.555	960.7573	651.5534	756.666	714.0217	0.420265	0.556314	0.140183	-0.39895	-0.17248
210.094	191.0231	155.776	186.827	167.3435	0.165531	0.160233	0.024703	-0.27736	-0.00708
1515.376	1349.543	1167.218	1276.079	1253.537	0.021154	0.205112	0.039141	-0.17618	-0.0437
451.342	491.027	478.5878	394.3437	404.5937	0.118824	-0.03188	0.092589	0.052669	-0.23447
151.3662	161.5578	144.169	153.3702	132.3276	-0.06989	0.039351	0.132544	-0.03013	0.058413
222.8503	242.6044	200.9972	201.9362	170.063	0.165586	0.021588	0.132629	-0.13092	-0.14237
86.73047	78.43218	95.62351	112.629	104.4378	-0.17412	-0.1908	-0.33126	-0.04714	0.189112
287.3868	298.2684	341.5864	305.6471	332.3282	-0.09506	-0.08068	-0.02663	0.169017	0.008918
131.1994	141.2782	138.3989	155.9532	169.6606	-0.19175	-0.12911	-0.02048	-0.05233	0.121654
134.5371	111.3316	113.9033	85.14697	106.3975	0.109529	0.248476	-0.02304	0.005786	-0.432
151.6133	136.9493	117.6379	111.8027	123.2723	0.017636	0.260888	0.115503	-0.10591	-0.17538
431.2702	435.4821	367.0992	338.6374	350.45	0.139817	0.096368	0.10685	-0.14894	-0.25706
80.74707	92.47794	61.82489	65.07882	62.79736	0.176063	0.130294	0.332633	-0.27426	-0.17648
34.58889	29.43833	29.1628	36.92035	24.85197	0.067569	0.150396	-0.0676	-0.08712	0.249371
71.05848	93.71555	82.10447	92.05404	89.92596	-0.28202	-0.18926	0.210377	0.016336	0.17989
245.5796	268.7323	310.4916	288.8502	307.6634	-0.00972	-0.1752	-0.04492	0.162885	0.052505
26.94914	22.72779	29.54686	25.2597	25.85043	-0.09683	0.077535	-0.16699	0.21009	-0.01543
31.57384	39.78588	33.19988	32.23461	33.4166	0.057292	-0.07807	0.25183	-0.00353	-0.04576
24.38113	25.22075	24.08039	23.69813	21.09966	0.168282	0.001349	0.052701	-0.01717	-0.03772
46.42763	53.70903	54.34072	46.3116	50.05796	-0.09749	-0.10499	0.102935	0.121922	-0.10854
21.5441	20.31337	20.01131	22.0205	21.48322	0.156626	-0.0074	-0.08472	-0.10718	0.031048
34.86035	39.73416	44.215	42.43398	37.37204	0.055574	-0.26959	-0.07583	0.080093	0.02305
3105.878	3105.408	2708.623	2643.626	2778.625	0.030721	0.094293	0.092688	-0.10598	-0.14144
188.5751	248.1478	348.7802	187.8851	240.5774	0.545258	-0.39024	0.010251	0.417217	-0.45065
18.4766	18.12544	15.03267	14.67166	14.23139	0.056259	0.116954	0.088708	-0.18884	-0.22754
526.5393	455.2757	634.7718	624.1331	612.0819	-0.07158	-0.04159	-0.25292	0.2242	0.201404
62.79348	60.65599	57.34835	55.12549	56.5858	0.185915	0.092529	0.042792	-0.03918	-0.09684
49.98603	48.18726	44.35134	43.77284	49.04845	0.138011	0.043664	-0.00857	-0.13173	-0.15357
648.0544	734.5054	801.1833	666.0683	718.2056	-0.16	-0.09415	0.08577	0.208528	-0.05365
8567.79	6119.154	4757.524	5731.854	4880.223	0.301227	0.486042	-0.06479	-0.35143	-0.08577
744.4539	823.8018	900.9698	833.7974	836.4748	-0.08974	-0.13644	0.010911	0.138198	0.02753
450.4997	459.3968	466.8379	488.4198	502.1443	-0.08153	-0.04796	-0.01965	0.003247	0.06805
308.1203	288.1578	299.5763	294.9683	294.8833	-0.1537	0.060949	-0.03759	0.019385	-0.00209
150.5125	125.132	112.6988	139.1713	114.5953	0.083817	0.187064	-0.0826	-0.2504	0.072541
2478.952	2523.987	2647.748	2450.868	2305.148	0.070851	-0.00647	0.020062	0.0881	-0.02416
1910.024	1994.414	1279.476	1578.856	1499.221	-0.07143	0.229315	0.294213	-0.36416	-0.0541
1532.89	1940.6	1498.961	1460.618	1598.373	0.110016	-0.11078	0.22871	-0.15971	-0.18252
1722.139	1677.819	1723.055	1557.166	1475.436	0.258028	0.074471	0.047162	0.083293	-0.06094
421.6016	482.9196	522.8209	447.809	477.476	-0.12354	-0.14104	0.055678	0.170059	-0.05536
66.97953	70.55396	85.09958	74.0073	76.82288	-0.16881	-0.0806	-0.00527	0.256438	0.049795
494.0245	441.663	383.2862	524.2427	359.6115	0.109335	0.121093	-0.04164	-0.25621	0.185503
50.28834	45.98542	45.91446	48.02319	40.90355	0.195807	0.093011	-0.03455	-0.04106	0.024836

59.80419	70.53547	55.3607	55.67744	52.53836	0.036395	0.078023	0.317476	-0.03123	-0.02519
51.22012	51.21268	51.3385	48.98461	50.03576	0.301298	-0.03889	-0.04052	-0.0382	-0.11471
714.2393	600.1545	517.0556	605.4102	538.7548	0.107896	0.232691	-0.01711	-0.25558	-0.00706
150.8114	149.162	102.0444	157.6023	137.382	-0.03027	0.071331	0.057052	-0.49857	0.110647
312.5028	315.2475	336.599	340.6435	346.3671	-0.07857	-0.05454	-0.04119	0.053388	0.07067
1700.067	1912.982	1366.869	1432.131	1503.777	0.124672	0.058782	0.22562	-0.25677	-0.19448
121.4263	104.869	121.5241	134.8533	122.5637	-0.03042	0.004945	-0.20276	0.004194	0.157115
1074.428	1087.361	1160.724	1132.493	1282.912	-0.12676	-0.06979	-0.05181	0.043662	0.007305
1616.228	1567.233	1587.416	1741.299	1723.564	-0.20383	0.002852	-0.03946	-0.02596	0.113899
12378.36	11460.15	11971.59	12026.8	12555.98	-0.00266	0.02208	-0.08915	-0.02623	-0.02006
85.32872	76.40488	74.93597	92.56089	80.12142	0.111266	0.005605	-0.16139	-0.18193	0.122528
1351.2	1271.147	1383.154	1392.274	1420.469	-0.13472	0.01484	-0.07366	0.045351	0.057016
489.9015	499.3478	539.6963	504.5088	576.7454	-0.0849	-0.05504	-0.03169	0.081783	-0.0123
1494.112	1545.63	1369.481	1268.615	1159.742	0.055531	0.034911	0.089395	-0.08927	-0.20889
161.1169	162.6066	120.907	123.7146	120.9064	0.344975	0.137435	0.157226	-0.27893	-0.24908
89.74817	77.05795	96.21664	106.5338	96.40481	-0.17901	-0.07653	-0.29453	0.029001	0.178295
129.885	116.7008	158.6448	178.8096	144.539	-0.1536	-0.11228	-0.27197	0.176618	0.34662
1003.67	995.2864	900.5851	1043.008	854.5804	0.021824	0.046202	0.036833	-0.10859	0.102302
162.6275	164.3738	173.2013	195.7533	185.7628	-0.07811	-0.0623	-0.04554	0.029555	0.204109
201.6943	176.849	214.6809	188.4818	199.7932	-0.20672	0.073473	-0.11673	0.163723	-0.02526
252.1425	256.4959	320.2805	327.8376	314.9032	-0.11442	-0.13924	-0.10842	0.210671	0.234179
132.0943	141.654	155.1458	143.1863	176.7605	-0.20626	-0.12914	-0.02914	0.10203	-0.01066
549.2928	487.8272	562.9195	554.7128	537.2575	0.016994	0.006375	-0.16116	0.045186	0.024187
517.6549	480.9991	394.6224	370.8215	324.1559	0.218722	0.198987	0.090537	-0.18759	-0.29145
1381.604	920.7576	1002.038	870.3821	872.6856	0.539127	0.345395	-0.26687	-0.12279	-0.3378
22.07334	27.50756	29.15739	30.75437	29.35003	-0.164	-0.30839	0.020701	0.101041	0.177001
32.66988	31.47726	27.6177	37.23791	28.24403	7.62E-04	0.092363	0.030346	-0.15481	0.274748
29.92374	31.71097	33.06528	34.55219	35.82066	0.154318	-0.14903	-0.06588	-0.00711	0.057985
109.4271	124.291	122.2545	139.2919	125.8692	-0.03589	-0.19813	-0.01487	-0.04121	0.150212
740.6008	734.0454	595.8722	654.2917	584.9859	0.20448	0.111681	0.10526	-0.20631	-0.06006
339.0171	417.4921	247.2846	268.8748	225.5417	0.117913	0.269561	0.560155	-0.2022	-0.06297
108.5095	86.40262	136.9706	77.98816	89.3169	0.540916	0.065238	-0.27208	0.386437	-0.4088
632.1578	567.8904	847.2163	649.4974	642.4513	-0.05213	0.033123	-0.11923	0.44434	0.058064
110.1391	113.0396	82.70029	99.37717	100.5978	0.006894	0.071954	0.111387	-0.34427	-0.08016
67.39697	71.97855	54.05896	61.61092	64.14997	0.079198	0.074545	0.167297	-0.24271	-0.04982
65.1254	74.62576	55.20919	53.31118	55.61072	0.118128	0.132281	0.329904	-0.10269	-0.15823
299.1831	316.3257	214.5175	239.0981	224.2104	0.113189	0.103861	0.187126	-0.39039	-0.25887
28.75601	22.10462	17.48878	19.51758	20.6285	0.14093	0.353151	-0.02613	-0.35681	-0.2016
66.26724	71.33125	55.05622	56.87591	53.41343	0.136227	0.07827	0.181	-0.19467	-0.14945
56.49302	68.57693	47.00123	47.05227	50.31135	0.097408	0.148198	0.433306	-0.10378	-0.10661
142.9963	172.1406	127.5633	127.8618	120.99	0.250387	0.049102	0.316066	-0.1185	-0.11918
268.6559	271.5766	298.2298	302.2516	306.086	-0.09222	-0.05913	-0.04436	0.090514	0.108346
19.31943	20.39419	17.55801	19.61847	19.43842	0.435708	-0.02844	0.047604	-0.16871	-0.01144
22.73849	26.11334	26.98691	23.67494	25.64884	-0.15026	-0.12588	0.077024	0.120718	-0.06613
29.69013	37.40243	40.12938	32.10613	42.92552	-0.03796	-0.23479	0.097661	0.17547	-0.12677
76.51258	69.90984	71.143	87.15354	76.19106	-0.19465	0.091347	-0.0375	-0.01567	0.276028
246.0097	239.1829	331.792	284.1193	284.9129	-0.17912	-0.06806	-0.11457	0.346579	0.130077

72.30469	72.82191	44.74321	55.22673	56.27832	-0.0073	0.305613	0.315136	-0.38203	-0.1175
99.888	103.2653	103.0462	107.8627	125.6892	0.013259	-0.06882	-0.02348	-0.02437	0.03885
33.24865	37.99173	29.71187	30.95781	36.14592	0.039523	-0.002	0.182747	-0.16682	-0.1137
240.4063	232.8135	310.764	434.1549	385.8087	-0.13269	-0.14713	-0.17252	0.180395	0.694197
318.4127	383.0773	203.4192	233.3252	230.4734	0.105311	0.183444	0.449732	-0.47354	-0.297
451.2651	458.0284	487.7943	519.2344	521.3487	-0.12608	-0.09991	-0.07474	0.01495	0.103969
19.24571	23.07073	20.81812	22.32745	23.92956	0.051387	-0.18589	0.06951	-0.07379	0.028563
153.5772	165.6101	209.1516	192.3311	185.6301	-0.36592	-0.05133	0.045678	0.369793	0.247996
795.1826	777.576	764.234	842.8767	912.7571	-0.23462	0.002028	-0.02914	-0.05558	0.084685
386.5098	344.4405	301.1674	405.985	340.8303	-0.17388	0.13658	-0.0247	-0.22008	0.205129
5907.986	6520.682	6635.843	6393.202	6444.322	-0.11131	-0.11904	0.021672	0.048078	-0.00598
215.9393	196.25	169.7267	195.4767	216.7265	-0.05309	0.085535	-0.05295	-0.26317	-0.07217
415.1874	561.2515	890.5029	972.1245	757.369	-0.70808	-0.6472	-0.44266	0.450992	0.541292
4510.195	4655.379	5532.666	4554.19	5444	-0.10104	-0.05906	-0.01754	0.231542	-0.04279
158.739	171.859	108.2664	132.2151	122.4499	0.031407	0.216151	0.316888	-0.33661	-0.05765
397.7855	453.4973	362.1726	375.901	412.4752	0.10433	-0.05228	0.136549	-0.19449	-0.13934
143.3093	155.7758	172.209	162.4806	161.9067	-0.20865	-0.13023	-0.00857	0.134805	0.052004
2456.397	2638.181	1903.677	2139.331	1899.394	0.277723	0.071357	0.162462	-0.32804	-0.13865
201.7265	213.5228	227.5708	233.0899	231.361	-0.30583	-0.08919	-0.00376	0.086772	0.116742
315.3845	335.3902	505.8977	484.1011	469.4403	-0.0683	-0.26644	-0.16439	0.403109	0.353381
1326.744	1417.567	1179.636	1141.859	1206.588	0.081257	0.061106	0.152245	-0.1106	-0.15909
970.9328	1012.794	970.2698	983.1299	837.9383	0.14845	-0.03204	0.034279	-0.02833	-0.01367
613.9654	805.717	510.662	601.0142	470.097	0.058947	0.013026	0.403156	-0.27237	-0.01902
672.4032	622.5718	538.3709	622.0517	580.5208	0.037621	0.135739	0.028349	-0.18127	0.025351
723.0048	736.7915	861.3369	848.7551	856.0908	-0.1513	-0.11688	-0.08847	0.1335	0.115016
972.7908	1061.324	1307.851	1248.877	1222.572	-0.22461	-0.145	-0.01878	0.272763	0.199369
742.8493	687.116	332.8365	292.5215	267.3982	0.154796	0.883851	0.691823	-0.27761	-0.46193
242.9031	264.9869	245.2461	259.3957	234.0161	0.069912	-0.07893	0.048816	-0.06317	0.017609
391.1441	345.5079	512.674	498.28	463.8272	-0.10858	-0.11618	-0.29533	0.265982	0.229452
207.3731	195.945	244.9332	251.2975	235.5343	-0.14464	0.019942	-0.0587	0.259879	0.286483
357.0578	357.263	445.3229	552.1497	471.4627	-0.15204	-0.32293	-0.33912	-0.00976	0.308395
390.6001	272.1728	225.4107	271.6759	258.1267	0.227414	0.501716	-0.01733	-0.28793	-0.00625
2634.081	2684.506	3808.481	3488.825	3534.609	-0.29372	-0.09043	-0.06982	0.423544	0.280163
398.7777	445.4487	397.7536	398.0525	414.2146	0.153735	-0.04253	0.118468	-0.04451	-0.0435
884.8355	963.4946	1057.421	959.6431	990.4629	-0.11105	-0.06299	0.059785	0.192855	0.048425
1165.155	1214.529	1392.813	1276.543	1275.506	-0.17882	-0.02888	0.030468	0.222536	0.09096
437.941	445.312	513.843	496.3285	464.5295	-0.08087	-0.03718	-0.01345	0.194271	0.13737
301.9148	284.8539	434.3207	344.7011	348.5731	-0.20904	-0.06669	-0.15395	0.433525	0.096248
272.9417	259.9881	245.1475	192.8319	189.907	0.17955	0.127206	0.059925	-0.05041	-0.37859
536.9315	506.2904	545.3498	577.4597	557.906	-0.18335	0.010171	-0.07581	0.033058	0.113504
20.85151	23.70833	26.65099	24.13471	24.28451	-0.01849	-0.1934	7.26E-04	0.168609	0.026873
965.1743	1009.089	980.7996	1028.322	1104.443	-0.16123	-0.04501	0.01988	-0.02344	0.047808
1087.68	1003.86	805.1362	819.7651	928.7033	0.00568	0.201507	0.088755	-0.23412	-0.20183
234.5997	267.7458	302.1046	290.5938	294.4365	-0.19689	-0.23509	-0.03639	0.136465	0.080721
604.8836	526.3257	506.9168	469.9989	504.9135	0.126666	0.213482	0.012632	-0.04083	-0.15473
472.7465	475.8756	504.4129	520.551	512.597	-0.06354	-0.03837	-0.0292	0.054973	0.09874
62.98839	58.91936	66.55064	52.89766	53.7661	0.022049	0.122538	0.026344	0.203358	-0.12868

180.6362	178.982	203.92	205.122	172.8879	-0.10474	-0.02049	-0.0397	0.155522	0.163893
88.51679	79.18969	93.62449	88.76138	94.15287	-0.14193	-0.01261	-0.17364	0.068603	-0.00757
212.0732	198.3309	170.8765	192.0032	191.0008	0.072708	0.094175	-0.00201	-0.21962	-0.05023
497.5283	448.7982	610.3148	555.8435	551.6675	-0.06457	-0.09265	-0.24155	0.202967	0.0651
526.155	552.348	692.6271	557.833	634.5028	-0.17648	-0.14722	-0.08054	0.244973	-0.0778
35.13823	37.61868	31.20821	30.17421	28.91586	-0.06293	0.100719	0.196436	-0.07451	-0.12101
1427.205	1320	1667.235	1545.695	1607.062	-0.09272	-0.01721	-0.1316	0.203141	0.088034
533.2291	653.2709	497.4642	433.1863	523.1473	0.133387	-0.04626	0.260697	-0.14063	-0.33553
591.5582	624.5161	686.9415	685.0467	695.3941	0.019432	-0.16615	-0.08881	0.04567	0.045254
472.5743	501.4217	686.1213	529.7007	629.9161	-0.19388	-0.12953	-0.04675	0.387864	0.015708
162.7629	167.1825	180.5698	178.5869	186.1434	-0.33684	-0.05539	-0.01668	0.09041	0.073919
233.0957	277.9145	231.238	262.9395	255.5467	-0.0267	-0.11242	0.139819	-0.1263	0.059776
389.5931	321.6952	304.8421	420.5304	270.6937	0.313501	0.12427	-0.14937	-0.25676	0.222098
22.58551	27.37288	27.02757	23.40659	22.04001	0.016966	-0.11546	0.159338	0.141774	-0.06604
83.612	96.34899	102.5121	114.5991	110.9429	-0.09346	-0.2659	-0.05787	0.033386	0.194367
248.8535	225.1307	235.3218	277.462	254.5963	-0.00116	0.051244	-0.09335	-0.02672	0.207904
40.09757	41.66982	44.42482	39.19889	38.51735	0.004705	-0.02791	0.027407	0.119878	-0.06307
91.05589	71.38598	126.796	88.52722	94.74127	-0.10591	0.095679	-0.25865	0.532227	0.038629
402.3807	446.193	547.0026	498.0032	576.691	-0.21224	-0.1059	0.043167	0.316134	0.191974
903.3621	982.3066	1024.416	963.4709	1001.062	0.041894	-0.14101	-0.01996	0.039863	-0.05065
2059.54	2404.094	2562.129	2684.121	2553.751	-0.08766	-0.25983	-0.02742	0.062484	0.124617
554.143	512.8164	492.6834	542.5334	480.6481	0.053232	0.071259	-0.0405	-0.09914	0.040722
1538.889	1118.014	1190.281	1452.269	1370.945	0.051748	0.142822	-0.31761	-0.23924	0.059581
42.857	46.68895	30.72725	39.93918	35.78569	0.119411	0.094062	0.213523	-0.41716	-0.00557
329.6973	323.1262	226.4996	246.6339	233.5883	0.019222	0.286488	0.256577	-0.29844	-0.13066
7395.085	7670.008	6569.254	6928.798	6509.617	0.045142	0.068468	0.119813	-0.10337	-0.02728
54.19228	61.45931	45.55467	54.05481	48.85236	-0.01101	0.03677	0.218917	-0.21214	0.030103
300.1668	339.7012	262.5303	246.5468	322.891	0.048646	0.044259	0.232593	-0.13984	-0.23636
28.69542	34.54018	33.02186	35.68294	34.3623	-0.18626	-0.17457	0.095264	0.029318	0.133996
1063.523	1272.646	856.8923	943.1672	947.0766	0.186454	-0.11237	0.148419	-0.45853	-0.30039
173.5494	172.0276	190.6502	204.34	198.9447	-0.09094	-0.05282	-0.06545	0.080342	0.181233
22.65931	19.89165	17.77317	18.61204	18.90259	0.194713	0.196859	0.009618	-0.15817	-0.09545
28.36814	32.39805	30.90289	30.80709	29.89121	0.155686	-0.14047	0.052336	-0.01621	-0.02024
12817.2	11908.26	12419.51	12579.69	13105.73	0.013712	0.019883	-0.08617	-0.026	-0.00709
5255.51	5073.298	5487.032	5079.807	5780.428	-0.04368	8.37E-04	-0.05027	0.06273	-0.0486
1575.504	1607.988	1963.164	1957.627	1955.178	-0.1409	-0.15977	-0.12693	0.159003	0.15733
31.03048	31.57336	24.42019	27.25495	22.35793	0.175345	0.096126	0.119588	-0.26326	-0.09244
393.1435	431.5664	500.1671	507.3006	480.8222	-0.13845	-0.21843	-0.08522	0.124355	0.150049
197.2019	206.1223	176.4642	187.4878	171.7916	0.181192	0.023681	0.087103	-0.13794	-0.05432
139.9369	153.486	134.6454	133.255	135.9313	0.033909	0.029916	0.162333	-0.02477	-0.03991
3080.817	3425.827	3500.1	3359.881	3433.328	-0.21017	-0.10252	0.049671	0.079038	0.022104
20.54295	21.20514	18.65545	21.67682	20.11937	0.094619	-0.0249	0.019654	-0.16483	0.051069
786.8044	811.8679	742.1907	664.5904	656.9665	0.053449	0.061032	0.105045	-0.02276	-0.19292
32.93321	34.30701	24.57526	36.03492	31.5228	0.195756	-0.02107	0.057796	-0.42485	0.104944
828.289	961.093	690.3273	776.8244	738.1463	0.118827	0.046805	0.257704	-0.21654	-0.04797
10529.5	9855.773	10783.91	11182.7	10802.17	-0.04459	-3.36E-04	-0.09596	0.033215	0.086051
865.4809	1117.497	762.4644	832.9362	847.4973	0.049024	0.015853	0.376332	-0.16905	-0.04908

4371.556	4533.1	4611.067	4161.64	4365.501	-0.1992	-0.00679	0.050934	0.074854	-0.07245
5783.302	5524.904	5040.354	4858.648	5179.62	0.082993	0.081883	0.015219	-0.11875	-0.17364
2192.682	2082.514	1841.546	1856.716	1914.654	0.060699	0.136491	0.059405	-0.12083	-0.10581
24.57595	25.318	24.25269	21.57489	23.37771	0.115908	0.005908	0.050332	-0.01164	-0.18436
316.1031	294.3855	192.837	182.3196	170.2262	0.341173	0.420198	0.31993	-0.28075	-0.35394
68.58025	67.06731	59.86038	55.71773	56.60757	0.108315	0.191171	0.165073	7.97E-04	-0.10235
46.25794	48.32808	29.69877	38.43373	32.83616	0.138985	0.154505	0.211189	-0.53442	-0.11889
941.7838	957.165	913.7463	982.1906	1072.526	-0.04443	-0.03866	-0.01597	-0.0828	0.022148
7597.278	9364.643	6309.703	5774.284	6370.718	0.113843	0.029908	0.327168	-0.28313	-0.36815
6343.451	7909.949	5324.205	5152.766	5480.888	0.099154	0.011819	0.325765	-0.28412	-0.29326
8493.298	7977.44	6704.134	6196.735	6628.639	0.212235	0.165731	0.072247	-0.1915	-0.2916
4424.727	4473.928	4745.596	4077.557	4633.467	0.100258	-0.04324	-0.02578	0.057964	-0.16153
354.0886	417.6995	525.3867	445.7394	493.7301	-0.28684	-0.16921	0.054985	0.380191	0.156521
186.5381	170.7086	137.7566	143.8582	145.9038	0.039995	0.274745	0.146682	-0.16557	-0.10186
708.6099	635.7847	529.6893	636.8836	545.9759	0.303457	0.178931	0.022857	-0.24572	0.014668
2053.014	1393.275	1981.212	1881.467	1654.348	0.093304	0.090733	-0.46626	0.034821	-0.0255
38.51775	32.68675	36.17376	48.38997	35.30195	0.052649	0.031215	-0.20244	-0.06112	0.353985
163.3451	155.3201	201.8792	211.2772	192.6794	-0.22068	-0.21378	-0.29932	0.097371	0.1548
994.4778	1083.342	1006.678	976.5356	981.0041	-0.00807	-0.0059	0.117396	0.011266	-0.03219
116.4027	124.9396	131.9966	129.3354	131.3554	0.089976	-0.12858	-0.02645	0.051699	0.021489
38.79779	39.85158	46.83347	37.569	37.26941	0.05223	-0.02807	0.009393	0.237142	-0.07638
3828.377	3683.316	4010.723	3830.85	4160.333	-0.10392	0.02044	-0.03295	0.09021	0.023251
375.548	362.7505	326.3818	322.5035	307.7158	0.12873	0.109386	0.05849	-0.09042	-0.11158
258.3395	210.6609	246.506	259.6962	221.4142	-0.00484	0.077224	-0.21605	0.011263	0.085007
117.7351	126.0928	125.4642	114.2029	108.1722	0.317844	-0.03826	0.055206	0.04358	-0.08127
117.6248	136.9605	116.4197	116.9707	125.4083	-0.11553	-0.01815	0.202261	-0.03691	-0.02735
4619.73	4651.389	4905.877	4653.514	5238.33	-0.10548	-0.02398	-0.01231	0.065008	-0.01125
395.2228	381.146	151.7284	209.8335	173.8881	1.060139	0.791166	0.646706	-0.67952	-0.1236
54.16037	42.11624	83.70223	54.40892	57.32505	-0.13237	0.088363	-0.27198	0.667812	0.094364
191.8876	164.7848	232.2781	223.9198	242.1525	-0.14792	-0.12873	-0.34737	0.14979	0.095549
1538.317	1459.466	1229.862	1259.788	1133.309	0.189179	0.153278	0.077859	-0.16822	-0.13964
81.6667	75.82658	67.94872	77.98685	79.3068	0.087813	0.065643	-0.0429	-0.19912	-0.00291
30.28478	30.69246	35.19041	30.93761	28.48601	0.219545	-0.04099	-0.02271	0.173605	-0.01152
423.5228	417.1388	442.0258	428.632	402.5956	-0.16761	0.002331	-0.01788	0.065136	0.020675
160.7155	180.427	131.9197	172.6334	172.1906	-0.07519	-0.02203	0.139356	-0.30749	0.068782
24.42839	26.85925	16.12455	15.08048	17.61851	0.342696	0.243945	0.371059	-0.3675	-0.45116
149.1825	146.157	128.5579	138.7263	133.5688	0.141376	0.095118	0.065066	-0.12042	-0.0091
125.4229	112.9184	114.4074	111.6107	120.1611	0.223726	0.072045	-0.07914	-0.06293	-0.09639
101.5851	99.1015	82.51658	79.3855	79.60447	0.186814	0.16437	0.127057	-0.14573	-0.19278
75.32614	60.68121	67.77276	62.12314	62.33359	0.058595	0.186076	-0.12379	0.032965	-0.09703
17.9681	17.46068	17.06229	19.9037	16.08813	0.112729	0.035273	-0.00468	-0.03809	0.183082
577.6617	740.1503	577.9088	583.8983	580.9372	-0.20676	0.024089	0.381292	0.025486	0.040784
32.80473	36.55401	27.17468	30.30274	25.63914	0.008324	0.102602	0.258528	-0.18098	-0.01094
1736.351	1600.898	1194.281	1655.278	1315.837	0.135685	0.176891	0.053524	-0.38629	0.087796
143.5039	141.185	143.9374	177.6765	159.7884	-0.13087	-0.01407	-0.03779	-0.00994	0.279902
3684.77	4027.702	4401.655	4199.51	4437.423	-0.14958	-0.16234	-0.03361	0.094539	0.024055
2457.837	1768.482	1608.629	1161.344	1203.916	0.424573	0.670673	0.150866	-0.00626	-0.40796

297.9235	305.5345	315.6104	281.3562	324.8384	0.097782	-0.04195	-0.00518	0.040365	-0.12879
731.6904	768.6196	880.4062	825.4375	782.1525	-0.09859	-0.08404	-0.01215	0.18147	0.089884
2492.455	2402.384	1338.395	1749.558	1486.93	0.045041	0.340532	0.284183	-0.55461	-0.1954
416.7502	444.4787	286.038	333.1381	312.6114	0.257216	0.180952	0.288379	-0.36177	-0.13595
990.1711	1030.037	1280.406	1289.767	1268.952	-0.20959	-0.15091	-0.0921	0.217983	0.216702
395.4079	390.3178	406.3967	393.8843	376.9284	-0.12162	0.025961	0.008719	0.06677	0.021427
677.2406	686.9841	688.0091	800.4702	794.9387	-0.08887	-0.04886	-0.02759	-0.03247	0.189675
2516.976	2567.2	2834.856	2776.782	2828.833	-0.078	-0.10521	-0.0775	0.066761	0.034777
195.3303	163.7919	170.1704	201.1944	204.7094	0.023392	0.043132	-0.21146	-0.15586	0.076077
1673.247	1729.638	1039.513	1145.948	1123.412	0.024495	0.334114	0.365127	-0.3314	-0.19483
103.8022	107.6406	72.81692	101.5787	88.9162	-0.08431	0.188407	0.232293	-0.34022	0.141366
70.07596	57.92168	52.61309	49.98778	41.59229	0.336446	0.356098	0.083162	-0.10034	-0.12137
1819.577	1985.416	2186.768	2159.439	2147.225	-0.11736	-0.12677	0.00119	0.133617	0.118991
461.3485	439.0207	593.2997	729.312	697.5056	-0.17325	-0.28577	-0.36823	0.050432	0.36359
2388.323	2356.452	1796.953	2074.904	1785.388	0.191134	0.11374	0.092516	-0.30246	-0.09169
521.5633	582.4761	457.5804	502.0068	491.4461	-0.14014	0.054145	0.208533	-0.13611	-5.31E-04
823.4062	784.8687	533.2921	613.1238	600.6308	0.293931	0.285779	0.213624	-0.35549	-0.14999
149.4381	170.4672	107.9351	116.2046	109.6034	0.293846	0.105806	0.281171	-0.38826	-0.26897
1347.462	974.5147	896.9295	1215.677	1055.983	0.035805	0.307331	-0.16187	-0.31612	0.152592
342.8611	277.9123	440.7656	509.4753	454.1816	-0.19732	-0.17277	-0.51298	0.163176	0.381965
85.6265	97.90143	89.0342	83.661	82.56427	-0.10666	6.82E-04	0.192565	0.054772	-0.03367
908.4549	850.0422	630.9598	572.4523	511.6006	0.340656	0.280697	0.169768	-0.25907	-0.41323
625.5587	692.5672	686.7579	638.9644	647.331	-0.11216	-0.05859	0.090663	0.078595	-0.02711
808.2145	823.4646	681.4341	666.8849	656.3283	0.098156	0.096935	0.122246	-0.15781	-0.18221
125.4977	124.7195	143.044	139.9921	144.5905	-0.08984	-0.05855	-0.06811	0.129309	0.099024
1385.913	1496.367	1624.2	1463.063	1583.774	-0.10606	-0.09806	0.012075	0.130449	-0.02203
3415.17	3635.088	3568.928	3172.69	3386.444	-0.08996	0.006651	0.096802	0.0693	-0.10005
405.7505	393.1741	432.6598	465.1816	454.8914	-0.05224	-0.06641	-0.11361	0.026178	0.129822
66.14706	69.13947	52.91803	58.55357	51.29649	0.292825	0.018125	0.080765	-0.33748	-0.16666
183.4339	176.5883	166.9575	199.6286	191.828	-0.15585	0.02876	-0.01696	-0.09784	0.158168
152.0095	146.7955	113.2034	147.3377	151.049	0.059079	0.113509	0.061478	-0.31789	0.060593
565.0605	577.8413	696.4433	635.021	657.785	-0.15945	-0.12228	-0.0956	0.168862	0.046563
192.4798	185.2593	193.8359	212.4698	167.2152	0.005631	-0.02701	-0.08242	-0.01644	0.115244
468.6422	450.9571	429.2675	457.8738	419.9186	0.039794	0.049021	-0.00737	-0.07853	0.013571
209.0544	230.1886	188.4424	144.1983	148.4946	0.469746	0.048551	0.171978	-0.15368	-0.48953
588.4762	697.6726	520.7329	521.2011	522.6201	0.27746	0.017924	0.266929	-0.15541	-0.15936
54.1403	54.99185	43.32742	43.50684	45.40805	0.259888	0.119478	0.150999	-0.19569	-0.18314
71.80929	63.21703	42.5683	44.91552	48.39392	0.196859	0.541604	0.339352	-0.22313	-0.13531
972.8738	814.9862	815.4426	824.8957	758.4117	0.413842	0.152589	-0.11211	-0.11411	-0.08331
520.0925	405.8987	391.5841	528.4119	463.4178	-0.01966	0.114751	-0.26383	-0.29925	0.134789
449.2534	443.2036	467.9092	510.3561	486.1818	-0.14745	-0.05529	-0.0737	0.005236	0.13081
14.83877	15.84686	12.28147	14.32911	13.39571	0.618979	0.01958	0.110546	-0.27875	-0.03355
217.5878	208.9794	189.6688	197.9588	190.0764	0.026274	0.102392	0.04438	-0.09587	-0.03585
30.53042	31.86569	30.83952	31.71512	30.14921	0.228642	-0.00792	0.053578	0.005643	0.044839
3397.803	3282.888	2574.556	2870.512	2774.434	0.06333	0.188121	0.138449	-0.22169	-0.05326
68.07166	62.57211	50.66212	57.12645	51.56568	0.158725	0.183443	0.040885	-0.2455	-0.07256
119.9127	131.751	135.8283	132.6142	115.2105	0.009933	-0.05773	0.077437	0.120409	0.082254

408.6203	543.4156	278.4422	268.0562	315.9054	0.191249	0.060436	0.464363	-0.52053	-0.57489
65.52411	59.07643	73.28074	80.60914	63.60325	-0.02249	-0.04283	-0.19692	0.118536	0.25106
24.68023	27.52721	27.60388	26.17786	26.45583	0.244875	-0.12102	0.040201	0.044144	-0.03063
20.37465	19.71431	17.79017	21.89725	18.40904	0.056302	0.01373	-0.02811	-0.1764	0.123579
98.61822	103.8614	91.35278	80.56406	93.54873	0.163367	0.030982	0.104124	-0.08094	-0.26107
36.23373	33.89107	31.5115	35.12642	34.38766	0.134827	0.078792	-0.01661	-0.1244	0.034225
50.83962	52.95429	72.10171	49.02081	55.31801	-0.11716	-0.05435	-0.00381	0.434231	-0.10797
179.7199	185.0487	194.7853	195.6725	202.2169	-0.14437	-0.08119	-0.03651	0.037097	0.043804
187.9447	176.2526	158.7675	179.9211	174.006	-0.0726	0.101247	0.009524	-0.14038	0.039055
828.2558	983.1663	708.277	656.637	654.1279	0.089616	0.172989	0.424562	-0.05391	-0.16693
333.3611	318.3361	273.9617	302.1133	273.7848	0.041699	0.166532	0.094075	-0.11708	0.023233
21.90898	23.97224	31.49609	27.21566	27.41104	-0.27251	-0.15995	-0.01342	0.359794	0.168451
198.4812	190.7857	191.7045	259.0678	235.7344	-0.28806	-0.03801	-0.08803	-0.08464	0.338405
51.11187	47.42811	37.17847	39.00454	35.83858	0.176848	0.234939	0.141856	-0.21843	-0.14126
44.78488	47.95372	57.74296	54.68996	53.56711	0.031987	-0.21418	-0.11308	0.15155	0.073111
19.82175	20.50514	16.88382	15.79444	16.64006	0.17482	0.053074	0.102166	-0.18283	-0.27848
183.4646	174.1351	123.0945	145.755	148.7667	0.310565	0.143354	0.073338	-0.48747	-0.18943
734.8815	641.8128	433.2871	610.8259	543.556	-0.02046	0.225762	0.014177	-0.57041	-0.0476
1031.201	1086.686	1097.496	1215.199	1194.145	-0.18088	-0.11072	-0.02875	-0.0158	0.128188
433.8545	514.9729	582.4316	521.0024	641.6951	-0.3312	-0.26737	-0.01328	0.167065	5.65E-04
110.7821	140.6023	44.19707	56.83509	43.92012	0.479768	0.392005	0.657925	-0.98675	-0.59944
28.01464	30.45029	24.87824	30.05506	28.70399	0.045238	-0.04281	0.079303	-0.21603	0.059671
166.7121	165.3349	135.6789	149.7583	143.4394	-0.14281	0.179689	0.168515	-0.11837	0.02434
129.8511	117.6305	77.14825	69.19354	73.327	0.401949	0.288221	0.144877	-0.5033	-0.63803
397.446	494.8434	255.2255	256.6215	238.514	-0.02252	0.379358	0.672478	-0.27111	-0.27194
124.8828	79.18765	135.0752	117.8116	98.05701	-0.01272	0.083204	-0.56537	0.195329	0.018028
295.3695	347.953	240.47	239.7713	255.2549	0.118938	0.127748	0.362944	-0.16571	-0.17689
139.7499	163.0454	91.42762	79.51706	79.81918	0.150559	0.235445	0.457003	-0.41015	-0.63411
149.8751	172.0675	126.9871	161.7949	151.8183	-0.10642	0.00249	0.19342	-0.2402	0.106719
39.36429	34.63727	34.00508	31.58211	30.98018	0.10442	0.221334	0.025796	0.006159	-0.09764
149.4414	157.285	117.4534	135.6683	131.4676	0.026302	0.073042	0.145864	-0.27519	-0.06902
173.3424	195.6326	426.8506	361.7948	224.2512	0.943088	-1.25228	-0.68119	0.366038	0.20953
198.9789	210.0792	456.2321	374.6953	259.3928	0.832295	-0.93046	-0.71711	0.382223	0.135034
1187.792	1451.774	1055.429	1101.118	1073.613	0.025496	0.036244	0.324105	-0.13615	-0.07428
2238.829	2311.23	2487.013	2530.289	2590.087	-0.17842	-0.05736	-0.01013	0.094739	0.116015
51.1859	44.2101	69.87894	51.40054	65.02572	-0.15847	0.014498	-0.20435	0.434464	0.010965
72.10665	70.03018	66.04388	53.32073	58.90196	0.112035	0.072003	0.031313	-0.06176	-0.36855
64.22591	70.01913	56.48752	65.3381	54.05549	0.077801	-0.0159	0.104412	-0.21155	0.00485
98.48718	114.851	97.83429	93.68872	94.78528	0.174518	-0.05888	0.162525	-0.07229	-0.13973
162.1084	191.4898	94.37321	135.1956	104.748	0.321653	0.092187	0.329141	-0.73645	-0.24376
8550.655	9068.302	10247.2	9542.357	9749.839	-0.0833	-0.09171	-0.00642	0.166645	0.066698
395.889	316.9869	296.7406	341.2753	287.6728	0.262219	0.225164	-0.09108	-0.20172	0.014626
78.48806	68.08045	64.88751	70.22153	69.17456	0.095856	0.170801	-0.03452	-0.10457	0.008721
27.45369	26.36785	29.48323	32.51668	25.95924	0.011409	-0.01628	-0.07406	0.081696	0.227565
30.92097	29.26917	29.85068	28.65132	30.2251	0.321181	0.017042	-0.0633	-0.04626	-0.0964
24.71952	20.51136	21.17967	21.856	22.10335	0.150074	0.13429	-0.13313	-0.09264	-0.04488
79.36462	93.43738	62.87627	70.49889	56.88932	0.829509	-0.00261	0.212369	-0.38425	-0.15586

133.792	134.0594	296.7905	119.8708	147.1905	-0.15769	-0.07437	-0.036	0.979783	-0.20352
67.55494	67.89861	67.88197	72.63213	62.4874	0.194141	-0.02401	-0.0157	-0.0195	0.081054
26.39957	27.32643	25.51031	23.75199	26.61449	0.201676	-0.01212	0.036852	-0.05885	-0.16143
45.54812	46.27548	45.35611	52.07364	48.49447	0.19474	-0.07169	-0.04277	-0.07138	0.122767
69.04166	59.31287	61.41047	56.20273	63.45852	-0.02248	0.17883	-0.03953	0.014372	-0.11329
1226.817	1236.891	980.246	1000.567	1005.755	0.026788	0.165215	0.173207	-0.17411	-0.13031
58.03422	56.43951	50.63895	48.39833	54.01519	0.05078	0.112676	0.073424	-0.08148	-0.14603
39.84264	36.82384	32.42271	35.0766	33.75121	0.170877	0.156642	0.047072	-0.13852	-0.02222
58.53463	54.92923	50.56376	46.25756	47.46542	0.414464	0.172568	0.073658	-0.03596	-0.16499
57.90066	53.56619	55.25023	53.28488	56.69647	0.213129	0.037127	-0.07707	-0.03025	-0.08432
67.35392	61.31505	62.81018	54.49663	60.28811	-0.2892	0.148738	0.019348	0.053047	-0.15369
52.97887	53.90784	45.86911	46.4268	44.32802	0.1718	0.060419	0.087061	-0.14885	-0.13591
93.84885	84.64695	82.72627	86.60688	67.6366	-0.01337	0.161411	0.013427	-0.02718	0.034835
39.96124	40.7382	30.68952	32.78397	36.75396	0.206671	0.072793	0.097446	-0.30609	-0.22194
448.8293	353.0642	227.7918	287.6378	249.4112	0.183105	0.506141	0.169346	-0.49585	-0.12911
886.2661	788.2958	594.5915	774.4857	773.8608	0.199078	0.139062	-0.03297	-0.44289	-0.07158
1393.109	1477.319	1263.491	1195.294	1142.046	0.010837	0.146895	0.233671	0.006471	-0.07136
1404.76	1189.644	726.9803	885.6643	802.9179	0.417623	0.612786	0.328444	-0.36544	-0.05388
484.6571	113.4278	3120.37	570.1771	494.2218	0.381507	0.095728	-2.22777	2.060975	0.296544
177.2028	174.5687	148.2191	144.4045	146.0556	0.180509	0.176112	0.147345	-0.07841	-0.11943
712.3606	712.3699	664.9106	582.395	687.8195	0.244013	0.055154	0.059362	-0.04471	-0.23204
125.4029	513.5582	91.14691	86.4718	62.92863	-0.14771	0.467449	2.495885	-0.01316	-0.03159
38.04987	35.48825	32.22953	33.07074	36.90155	0.023746	0.103164	0.004501	-0.14194	-0.09755
859.2943	707.9707	654.3911	855.7925	646.5191	0.084852	0.174501	-0.11586	-0.22184	0.162235
105.1324	126.5778	127.364	142.7751	133.6845	-0.14259	-0.23342	0.03121	0.042947	0.206888
4108.635	4751.445	4320.537	3985.695	4115.664	0.016942	-0.04816	0.159818	0.024215	-0.09609
135.4755	143.8903	229.96	177.7489	188.317	-0.47325	-0.16656	-0.071	0.573568	0.179479
1393.379	1632.795	1161.802	1265.825	1232.342	-0.1002	0.117573	0.333956	-0.14702	-0.02043
1054.491	1161.396	863.9647	778.4174	809.1381	0.268524	0.104111	0.249772	-0.20529	-0.33742
1925.958	1930.477	2273.444	2133.558	2157.386	-0.13582	-0.08497	-0.07839	0.154216	0.06688
402.2665	433.287	434.0445	375.8715	371.3695	0.076528	-0.03326	0.072648	0.075787	-0.13357
453.4174	569.3455	394.2111	457.644	422.3762	0.031216	-0.01592	0.310635	-0.2305	-0.00336
2018.584	2924.317	1617.19	1493.704	1495.933	0.045931	0.168573	0.703543	-0.17605	-0.25867
52.89702	54.8211	48.92716	47.42522	44.14789	0.049106	0.057772	0.105086	-0.05931	-0.10031
377.1029	268.4474	320.9104	314.1855	288.4531	0.823349	0.143669	-0.40677	-0.10168	-0.1216
23.92074	22.21163	20.17216	20.82365	20.63849	0.080867	0.149946	0.042037	-0.10107	-0.04946
728.3418	781.3003	798.9133	852.0854	920.8765	-0.05017	-0.13706	-0.03414	-0.00214	0.091203
229.8612	219.2741	189.8521	213.0571	191.3351	0.141108	0.095825	0.02535	-0.18261	-0.01182
609.8735	593.4795	593.8074	588.8832	658.4021	-0.04845	0.019399	-0.02033	-0.01936	-0.03198
40.52431	31.48055	30.60969	26.24338	33.79985	-0.20951	0.355084	-0.01683	-0.05053	-0.28271
1917.48	1834.913	1485.997	1673.08	1535.117	-0.01262	0.241734	0.189593	-0.11405	0.051629
158.0778	157.2098	134.7359	120.9026	133.941	0.219552	0.154315	0.140302	-0.07443	-0.23553
27.50429	22.57904	27.25213	26.42764	27.29266	0.105305	0.035635	-0.25053	0.01878	-0.02501
1173.567	1189.366	1053.345	1054.328	1077.903	0.141793	0.040675	0.059052	-0.11521	-0.11501
724.228	844.1619	871.8723	684.2087	741.2651	0.0608	-0.07803	0.141774	0.185011	-0.16339
203.9045	205.9855	146.0799	170.2883	175.0863	0.078942	0.175244	0.210287	-0.28744	-0.06887
119.5985	126.0029	96.02848	87.19324	92.78489	0.100309	0.151882	0.222568	-0.17568	-0.32054

267.36	308.8282	226.514	209.2154	217.0159	0.234349	0.047953	0.253535	-0.21711	-0.33512
561.7598	450.994	402.917	477.4792	396.1877	0.118569	0.218647	-0.09013	-0.2634	-0.00824
517.0383	528.4819	680.8246	591.6453	649.2447	-0.13545	-0.13398	-0.10534	0.250079	0.059588
256.9419	249.22	186.1779	226.0535	185.2117	-0.02584	0.182902	0.133557	-0.29103	-0.00279
460.251	417.655	402.3149	406.582	389.1145	0.10831	0.140225	0.001129	-0.05669	-0.03806
89.93357	69.63291	134.0309	90.66831	94.00443	0.078838	-0.0612	-0.43613	0.499701	-0.06566
307.6548	362.6328	229.1045	229.5686	246.973	0.350158	0.090361	0.328708	-0.34043	-0.34267
49.38249	56.4821	51.03567	47.50525	50.90755	0.201222	-0.09088	0.102708	-0.04219	-0.15077
563.4569	552.0186	168.3277	245.0592	229.7169	0.403994	0.943648	0.899203	-0.90144	-0.29173
763.1439	953.0969	607.9921	646.5952	634.0823	0.025324	0.217972	0.528298	-0.10039	-0.01675
230.0728	203.91	232.9939	243.6834	179.5779	0.113688	-0.05654	-0.24594	-0.03957	0.021615
78.10696	87.36325	82.48319	75.69054	78.20754	-0.05125	-0.03698	0.12599	0.043029	-0.08168
147.7419	139.7523	133.6226	135.8722	134.7416	0.144992	0.071378	-0.00789	-0.07389	-0.04844
139.6803	142.6145	145.0582	181.1619	162.2627	-0.06657	-0.09355	-0.06219	-0.04419	0.282579
134.7563	118.96	98.64551	118.3621	83.15431	0.239673	0.208176	0.043178	-0.22215	0.051688
676.1424	727.3552	711.3565	721.9128	704.5363	0.127707	-0.09093	0.013892	-0.01791	0.004495
141.2918	133.1492	124.2964	152.2839	117.7675	0.003981	0.074639	-0.01194	-0.11094	0.179712
39.54254	49.76986	44.77657	54.47257	38.42642	-0.02041	-0.14763	0.180611	0.035512	0.310281
48.27748	46.23426	60.61493	45.6604	53.04937	-0.35715	0.009651	-0.04331	0.350928	-0.06289
17.93013	20.27408	16.32214	14.95276	16.39843	0.056262	0.087825	0.264022	-0.04804	-0.17435
297.2828	270.3489	197.9143	206.1958	182.5071	0.133609	0.388223	0.246257	-0.22035	-0.13619
29.56374	33.41876	32.94693	34.27312	26.78437	-0.06049	-0.04373	0.127182	0.1115	0.165695
89.97439	190.6415	92.07746	86.33881	106.5537	0.333211	-0.20778	0.804444	-0.2025	-0.26377
434.6062	470.1478	360.1638	333.7178	379.7805	0.027904	0.115776	0.234964	-0.1649	-0.25898
23.50011	20.7886	23.40146	20.39968	22.02099	0.222781	0.047115	-0.13146	0.036765	-0.1593
2790.722	2570.645	2983.984	4194.478	3569.942	-0.21491	-0.07025	-0.19404	0.016292	0.462798
1007.228	947.6555	931.8825	862.7015	876.6662	0.070001	0.11509	0.030987	0.006137	-0.10544
597.5628	572.3687	401.3695	423.3004	447.6762	0.144886	0.186532	0.116172	-0.39619	-0.35379
595.48	601.7082	630.6762	597.4044	683.3791	-0.14841	-0.02647	-0.01389	0.056365	-0.02681
81.73089	72.76481	68.14774	80.85916	53.24301	0.165746	0.113219	-0.05522	-0.17949	0.076783
3185.214	3019.301	4076.971	4173.359	3958.825	-0.27779	-0.03359	-0.1111	0.304251	0.3259
1414.24	1516.94	1283.844	1169.517	1291.09	0.019933	0.040737	0.142601	-0.09956	-0.24335
777.1716	748.7664	471.8803	625.6316	504.6614	0.323456	0.194694	0.160324	-0.5933	-0.10299
163.2062	165.5434	148.95	163.1376	149.4066	0.022815	0.037587	0.058735	-0.0942	0.037236
393.9873	475.6706	360.7787	339.2438	366.5881	0.035414	0.063256	0.334885	-0.0641	-0.15319
177.571	204.4145	153.9589	163.3339	158.3778	0.102566	0.066129	0.259567	-0.13887	-0.05281
60.1418	51.18448	49.74678	47.19682	48.58934	0.137303	0.220689	-0.01342	-0.05272	-0.12629
178.0175	145.7459	121.169	134.7718	139.4388	0.40118	0.291917	-0.00948	-0.26733	-0.12493
706.2393	813.6831	870.3377	775.1899	829.272	-0.23945	-0.16882	0.033252	0.127484	-0.0352
72.97638	95.165	140.1644	105.5668	113.7607	-0.31445	-0.32928	0.054543	0.583708	0.150276
1052.733	1191.608	1222.006	1257.947	1235.112	-0.22168	-0.16252	0.017935	0.044394	0.096161
214.0499	266.5098	187.2034	175.6575	182.1805	0.177117	0.083953	0.385997	-0.1098	-0.20382
38.18508	41.47405	32.2649	36.41417	34.01224	0.028919	0.056827	0.191201	-0.17161	0.004236
337.4894	241.5779	181.7105	190.5784	130.78	0.885232	0.580245	0.085452	-0.32861	-0.22249
842.8281	789.1539	738.497	711.5095	699.9798	0.570821	0.080068	-0.01499	-0.12707	-0.15652
150.1131	135.7057	115.0119	127.0782	121.0513	0.131329	0.19054	0.043496	-0.19451	-0.05326
505.8549	456.4278	402.2031	344.9398	367.8315	0.240897	0.243712	0.112501	-0.07194	-0.30353

167.6507	127.8778	228.4313	224.879	235.0405	-0.26565	-0.00212	-0.40741	0.429405	0.387228
1540.099	1567.775	1196.689	1364.646	1260.548	0.221765	0.013355	0.040313	-0.37294	-0.1688
227.6497	209.0884	195.9424	191.0135	183.5278	0.107867	0.189258	0.065617	-0.03142	-0.06342
37.92794	36.55404	72.09272	41.48147	50.07034	-0.25421	-0.12216	-0.24176	0.781022	-0.05573
828.2901	1050.788	786.0015	860.292	903.4914	-0.15414	-0.04511	0.302269	-0.11901	0.008062
115.8543	120.0878	96.85333	87.2806	88.68459	0.139383	0.076591	0.127488	-0.18695	-0.34299
92.55536	83.51611	80.5341	103.1823	98.58963	-0.00701	0.023501	-0.13042	-0.17812	0.174754
112.5262	98.01461	95.42687	80.55037	71.71571	0.2504	0.256331	0.059772	0.012659	-0.22428
59.27168	70.8246	69.31259	60.58619	68.30631	-0.03055	-0.18304	0.076083	0.044988	-0.15189
331.3588	285.3188	248.0618	317.1065	282.0803	0.114052	0.199126	-0.01436	-0.21916	0.131776
52.01122	55.07072	70.07363	76.03326	80.26965	-0.23827	-0.20559	-0.1282	0.182241	0.337822
1003.919	953.1045	1021.775	877.8274	917.0198	0.112403	0.045432	-0.02816	0.072184	-0.15212
70.27709	68.25562	49.29046	51.11026	57.39879	0.253187	0.159966	0.121854	-0.3427	-0.33139
154.0984	157.9101	172.5228	183.4019	176.9708	-0.08139	-0.14601	-0.11584	0.017796	0.104475
483.9701	550.6891	515.7474	557.8116	600.4153	-0.21009	-0.09642	0.088139	-0.00495	0.107586
142.2392	127.9958	100.1386	105.261	90.00138	0.234708	0.248294	0.117195	-0.24653	-0.19967
148.4646	120.356	132.1767	162.186	160.0776	-0.14176	0.123406	-0.17755	-0.03841	0.255844
185.4766	178.7518	159.1877	153.213	140.4035	0.026909	0.165197	0.114468	-0.05719	-0.11015
174.2161	159.9573	188.2909	155.6033	185.9335	-0.07468	0.038829	-0.08173	0.149831	-0.12235
451.8897	453.0745	346.7833	367.8375	350.7047	0.208408	0.175279	0.183819	-0.20264	-0.11563
507.4718	543.1036	387.3327	469.6759	437.4634	0.002038	0.096224	0.196156	-0.302	-0.01277
2336.739	2618.316	2382.534	2089.912	2164.792	0.103045	-0.00407	0.158455	0.022197	-0.17054
49.37569	47.16087	40.94905	45.58528	43.17531	0.127272	0.105948	0.04085	-0.16389	-0.00755
808.1721	795.3999	960.5119	872.5623	922.8111	-0.14932	-0.00657	-0.02977	0.239008	0.100038
77.63728	86.24596	98.01069	95.69241	97.16726	-0.13852	-0.19152	-0.0328	0.151589	0.107654
22.33481	44.05693	18.13428	21.57283	21.77529	-0.02382	0.028616	0.986253	-0.26268	-0.02333
601.4038	686.2012	585.716	522.8278	548.2495	0.162154	-0.01418	0.170369	-0.05032	-0.218
521.2014	554.6646	581.3846	588.5689	603.7085	-0.10206	-0.10849	-0.01656	0.049851	0.069003
854.9995	833.5847	992.33	975.8423	942.4849	-0.08489	-0.08049	-0.11601	0.133084	0.110042
1429.671	1283.237	1153.071	1210.966	1263.459	0.010832	0.167124	0.011054	-0.14837	-0.07369
1011.212	979.1074	1215.829	1200.046	1257.879	-0.2384	-0.13737	-0.18798	0.130817	0.111182
2123.321	2165.644	1929.854	1825.007	1931.89	-0.01941	0.089355	0.116676	-0.04782	-0.12727
872.2563	760.3991	915.2285	885.5208	878.7025	-0.1091	-0.00491	-0.20489	0.064523	0.016834
2472.363	2397.332	2439.369	2424.389	2481.997	-0.24477	0.030473	-0.01063	0.014662	0.005608
1881.719	1741.137	820.4841	785.3331	667.9383	0.141869	0.728754	0.543475	-0.50245	-0.54488
49.77182	46.88736	51.17172	45.98586	38.92787	0.03902	0.075112	-0.01162	0.11778	-0.04422
164.8886	163.246	130.4418	132.3423	124.2946	0.246484	0.103643	0.094356	-0.25698	-0.21073
598.8027	686.7358	500.682	452.6138	482.1167	0.044722	0.114031	0.311206	-0.14213	-0.29105
622.8195	571.9747	636.4863	677.7034	616.826	-0.06829	0.009412	-0.11466	0.040233	0.132134
5102.804	5208.719	6057.588	5475.023	5665.163	-0.02571	-0.0382	-0.00852	0.208857	0.058337
1752.885	1876.691	1535.652	1581.295	1412.992	0.011409	0.099199	0.187646	-0.09184	-0.05175
833.8111	821.0562	1143.256	952.2246	969.249	-0.18094	-0.13237	-0.15175	0.315068	0.051528
31.34056	31.89851	45.60965	31.60305	36.45841	-0.19516	-0.05608	-0.02736	0.476168	-0.0422
1030.114	1089.386	1008.31	1046.381	905.1549	0.016391	-0.04064	0.04128	-0.07403	-0.01843
1769.359	1653.063	1566.065	1598.621	1524.53	0.17482	0.088428	-0.01029	-0.09022	-0.05803
104.0022	97.77557	116.4281	116.0299	111.93	-0.0959	-0.07422	-0.16251	0.088974	0.084405
114.9623	120.1099	76.12128	83.36517	89.20318	0.265413	0.154073	0.226111	-0.43864	-0.31981

209.8339	197.286	267.4822	229.361	261.2271	0.05255	-0.10128	-0.18846	0.241006	0.018095
38.58354	39.38842	41.61456	37.25681	36.32717	0.131634	-0.03441	-0.00395	0.075715	-0.08467
289.6905	291.2388	318.6367	292.4232	319.8755	-0.06018	-0.03169	-0.01938	0.109703	-0.0139
5521.402	5513.368	4854.419	4920.482	5313.266	0.003536	0.084332	0.084236	-0.09995	-0.08014
52.07126	49.14623	67.66761	84.86993	72.05116	-0.19277	-0.26041	-0.37187	0.109961	0.418895
687.8754	625.8306	579.5565	626.3067	637.0739	0.305138	0.108954	-0.01812	-0.1301	-0.01799
611.8385	613.9063	565.1709	692.5718	640.2703	-0.24213	-0.0216	-0.01346	-0.13654	0.160126
661.6788	674.6711	481.1268	474.9218	513.4907	0.234882	0.157806	0.191227	-0.30136	-0.32492
2401.853	2280.834	2626.499	2584.013	2749.911	-0.1424	-0.02534	-0.10829	0.103475	0.078876
2999.311	3102.969	3299.429	2794.277	3153.835	0.041171	-0.05686	-0.00671	0.080993	-0.16044
4636.014	4590.351	4936.98	4668.501	5185.913	-0.08201	-0.01244	-0.02514	0.080309	-6.46E-04
212.1808	200.6952	174.5915	171.8355	157.5708	0.092382	0.186696	0.105029	-0.09307	-0.11858
23.29829	15.07713	20.49058	17.57479	17.41264	0.398341	0.279173	-0.35886	0.092178	-0.12667
1026.558	968.2903	1169.581	1381.589	1447.148	-0.10807	-0.22858	-0.28907	-0.02661	0.217588
97.74246	88.98293	90.16632	75.49581	73.9231	-0.02536	0.154754	0.01439	0.037583	-0.22364
1500.455	1689.736	1916.098	1783.764	1939.684	0.030839	-0.26114	-0.08578	0.096215	-0.01326
1881.511	1395.563	1348.376	1665.303	1356.219	0.099824	0.289401	-0.14709	-0.19893	0.11007
189.563	201.8677	182.7155	170.0061	173.4392	0.059583	0.01877	0.106295	-0.03545	-0.1405
172.7997	170.5249	93.00643	107.0996	90.86078	0.295511	0.571803	0.495176	-0.39026	-0.10942
52.73323	56.6207	48.59682	48.36489	49.16265	0.135031	0.042901	0.146859	-0.07809	-0.08116
12998.97	12121.51	12795.44	12976.59	13425.74	-0.00185	0.00306	-0.09779	-0.02028	3.59E-04
33.11263	29.16496	23.47607	23.75343	23.92258	0.462726	0.3046	0.119381	-0.19585	-0.17582
1598.194	1693.463	1449.299	1435.246	1411.282	0.061625	0.038846	0.122858	-0.10154	-0.12179
460.6154	412.2584	348.6363	407.3273	337.1031	0.147105	0.166636	0.005862	-0.24363	-0.01031
271.0429	287.8596	196.345	213.7574	216.947	0.120415	0.083451	0.174971	-0.4118	-0.27102
407.9876	364.1853	341.9435	432.9052	391.178	-0.04108	0.062423	-0.10075	-0.19908	0.149884
989.7528	977.168	819.8552	793.7229	838.9736	0.079471	0.086809	0.067903	-0.18669	-0.24598
86.28339	103.714	104.2119	106.9935	100.4724	-0.1971	-0.15242	0.112847	0.11959	0.155999
20.66669	21.67037	24.08608	22.03099	24.91486	-0.03831	-0.08275	-0.01394	0.136913	0.004665
593.593	566.3024	440.8138	477.6722	466.3091	0.13516	0.180598	0.104147	-0.25983	-0.1362
66.51263	62.97856	68.24574	76.50137	81.24022	0.120199	-0.08597	-0.16317	-0.04862	0.118992
161.7532	155.6249	142.3097	137.2703	155.4055	0.13642	0.057889	9.38E-05	-0.12705	-0.17995
706.4263	697.6499	695.8945	691.8348	612.8298	0.015982	0.039352	0.022558	0.020089	0.011541
163.9333	143.0134	188.1697	169.3458	198.8514	-0.08259	-0.0072	-0.20289	0.191161	0.031316
202.3594	189.433	175.1615	154.1464	172.1855	0.093961	0.142549	0.048041	-0.07829	-0.24935
257.892	257.5021	209.2621	232.9405	229.2046	0.056471	0.105964	0.104722	-0.19593	-0.04085
218.5683	228.1006	271.6913	243.7196	259.289	-0.09158	-0.06208	-3.06E-04	0.245075	0.089213
482.3537	456.722	358.3784	419.3148	396.8527	0.034663	0.201761	0.116531	-0.22713	0.001838
397.6458	495.1978	383.0999	386.9096	360.3154	-0.09761	0.042582	0.354195	-0.01031	-0.01076
761.0452	849.157	940.6829	935.0613	991.2503	-0.17875	-0.21453	-0.05328	0.092667	0.083225
613.9587	598.5559	497.7332	623.0745	567.2933	0.082524	0.050364	0.013643	-0.25515	0.067703
368.0703	377.6717	292.5684	318.8269	303.9729	-0.08861	0.165129	0.203682	-0.16541	-0.04012
596.5098	660.4392	718.5205	655.9482	708.0269	-0.17107	-0.12463	0.019581	0.136124	0.011881
685.9177	779.4564	838.4143	893.0721	916.3308	-0.20351	-0.2575	-0.07412	0.028741	0.115358
1012.15	1335.128	913.3723	917.9266	909.2775	0.027572	0.033922	0.437635	-0.12672	-0.10152
23.11725	17.61131	14.00428	19.04907	17.08492	0.217071	0.310637	-0.09069	-0.4351	0.013487
174.906	180.4562	163.8889	177.4804	182.2555	0.008668	-0.02721	0.018235	-0.12098	-0.0069

34.00852	29.13064	28.19157	28.0379	29.58111	0.186909	0.178643	-0.04147	-0.09402	-0.09636
35.88597	41.54033	41.58307	40.50654	37.91663	-0.26099	-0.1283	0.083628	0.084421	0.047426
255.2919	257.071	199.909	263.4439	200.7739	0.091801	0.074682	0.076288	-0.30211	0.121529
724.6533	796.8926	671.7805	721.5681	803.156	-0.1089	-0.02432	0.113187	-0.13383	-0.0299
362.1864	369.9612	531.2439	417.4518	458.2719	-0.30925	-0.05161	-0.02395	0.469753	0.114728
179.842	174.6292	242.6986	216.1775	234.0671	-0.14114	-0.12669	-0.16959	0.290092	0.139339
450.7102	492.5304	461.3033	429.4834	417.4893	0.093651	0.014172	0.139503	0.048781	-0.05419
142.2931	135.9648	152.5499	144.2085	159.7948	-0.17282	-0.05827	-0.12062	0.045654	-0.03844
80.17781	89.97189	86.79879	87.80335	96.56388	-0.23784	-0.09688	0.069786	0.01532	0.033329
89.35309	91.11941	105.6423	107.6096	111.0786	-0.24981	-0.15452	-0.12575	0.091442	0.11884
98.85741	104.2685	120.291	116.46	115.0481	-0.05098	-0.14649	-0.07165	0.136722	0.089252
1131.762	1268.021	934.6033	962.741	915.0659	0.038719	0.111553	0.276718	-0.16626	-0.12475
140.798	116.9395	103.377	133.3485	144.0922	0.091452	0.109269	-0.1577	-0.34556	0.023295
60.99448	69.43443	50.79842	57.10368	66.34116	-0.34813	0.102011	0.283004	-0.17669	0.007515
201.1244	220.8519	343.0849	227.0347	314.0351	-0.24617	-0.20253	-0.03867	0.563394	1.36E-04
155.5702	141.585	213.4139	130.8385	173.5628	-0.18889	0.086923	-0.05203	0.486922	-0.16376
66.71664	71.38298	75.9903	69.50544	81.86507	-0.04183	-0.0736	0.023022	0.113447	-0.01852
262.7386	225.9796	397.8349	494.3682	344.9554	-0.07807	-0.15455	-0.37408	0.374076	0.738343
370.8987	363.3345	292.0729	279.9616	270.9813	0.315356	0.218062	0.182225	-0.12759	-0.19396
536.3314	615.5537	698.2507	673.1171	656.2955	-0.11581	-0.20324	-0.00761	0.17267	0.118607
327.5434	359.9596	420.6106	419.0494	416.3782	-0.21857	-0.16785	-0.03183	0.188145	0.173091
141.5829	138.1229	155.7792	142.1985	171.6582	-0.22509	-0.05639	-0.09606	0.082979	-0.05125
896.4559	1022.24	617.8684	667.5971	652.6542	0.242699	0.181782	0.371895	-0.37369	-0.24755
2829.618	3359.524	2523.526	2700.01	2579.625	0.050216	0.049784	0.294175	-0.1171	-0.02063
811.0988	801.2923	904.0333	841.7034	958.1403	-0.14228	-0.03932	-0.05949	0.11497	0.011942
307.6427	334.8467	306.1509	359.2666	342.3348	-0.21162	-0.08718	0.03812	-0.09294	0.136191
466.9286	368.6515	474.4866	468.7146	550.6108	-0.08499	0.042776	-0.30562	0.053732	0.047059
1002.399	1160.461	560.3768	572.3894	573.7925	0.419369	0.421981	0.617827	-0.47089	-0.40901
910.1389	859.4981	800.2014	715.8074	708.6511	0.022237	0.149027	0.067438	-0.03606	-0.2126
423.3244	427.944	511.8324	443.2405	455.0139	-0.18565	-0.04232	-0.02784	0.225801	0.01679
561.9879	556.5835	510.4552	552.3117	500.6065	-0.04713	0.095166	0.082314	-0.04257	0.06987
255.8909	244.3154	193.989	182.5532	203.6599	0.299496	0.221832	0.152722	-0.17028	-0.2718
39.81654	38.5308	33.69613	31.63964	39.43437	0.08532	0.086195	0.043228	-0.15403	-0.24296
257.5083	254.7298	285.2037	230.8148	239.5663	0.079367	0.025817	0.011517	0.171171	-0.13106
41.58954	44.64548	31.99954	30.29974	33.01486	0.073242	0.175197	0.287624	-0.19614	-0.28424
114.7458	121.7774	127.8041	117.2413	130.3958	-0.20987	-0.08783	0.004148	0.071852	-0.05219
142.251	127.9471	165.3226	161.7208	165.2539	-0.06252	-0.0354	-0.19104	0.181223	0.147343
143.8044	133.7204	142.9856	172.3203	149.4765	-0.04631	-0.00872	-0.1199	-0.02034	0.252
340.1651	305.6325	348.6155	362.4732	292.5869	0.139963	0.021268	-0.13567	0.053271	0.112308
91.54511	92.47352	74.73377	87.90377	79.62959	-0.0205	0.050301	0.065864	-0.24267	-0.00963
214.1608	207.5089	166.0193	198.8327	192.3966	0.196678	0.044729	-2.16E-04	-0.34119	-0.06825
1084.593	976.149	802.9226	1081.256	582.5242	0.246739	0.158534	0.021191	-0.29965	0.138771
19.62638	21.35505	17.48235	19.09926	19.26554	0.153655	-0.00172	0.125265	-0.16715	-0.03882
37.8291	42.27766	24.53972	33.1307	31.64571	0.120246	0.103617	0.260879	-0.55516	-0.09325
370.3303	416.0099	270.7682	345.6231	330.3752	-0.03665	0.134944	0.29108	-0.34148	0.035041
129.6435	134.3288	161.227	140.1387	157.1565	-0.16638	-0.06712	-0.01504	0.231819	0.044983
73.316	66.18922	68.911	80.15102	74.52361	-0.13178	0.053219	-0.09368	-0.03482	0.182549

198.9616	257.8397	194.153	204.6504	191.2378	0.10113	-0.09451	0.282865	-0.12838	-0.0601
4381.02	4990.473	3284.086	2754.471	2984.35	0.093287	0.278965	0.455942	-0.18139	-0.40903
534.7701	634.5216	431.3732	418.5345	470.458	0.113483	0.107134	0.354852	-0.20066	-0.24369
81.6226	83.88609	68.03117	66.57131	67.51485	0.111798	0.105048	0.148134	-0.15805	-0.18407
394.7286	374.9266	364.8094	317.8756	326.2867	0.17271	0.103595	0.034625	-0.00281	-0.2097
61.33729	64.47822	66.95603	78.75655	71.46655	-0.32029	-0.10368	-0.03124	0.025863	0.242303
1437.4	1452.004	1510.843	1504.34	1489.396	-0.11476	-0.01594	-0.00132	0.05496	0.049784
128.7144	137.0572	129.7371	109.4194	110.1724	0.114301	0.013529	0.102961	0.026449	-0.22393
247.0549	189.5686	201.8463	187.0573	175.1328	0.061372	0.335045	-0.046	0.04692	-0.05654
290.0024	325.056	354.7304	331.4659	307.2683	-0.15125	-0.1188	0.045068	0.171584	0.072189
28.7938	29.36058	28.8269	27.38922	28.21364	0.142589	-0.02843	-1.89E-04	-0.02638	-0.10204
23.549	22.94114	26.4015	20.94602	21.21261	0.006486	-0.00533	-0.04494	0.159338	-0.17434
1008.93	1054.908	961.5187	883.7673	868.9041	0.107353	0.023039	0.085829	-0.04569	-0.166
39.46614	26.88879	25.95085	25.54153	25.38869	0.249485	0.505204	-0.0586	-0.13625	-0.12421
18.70677	18.65891	15.78207	19.19868	19.31582	0.060807	0.01059	0.007995	-0.2345	0.041605
22.99027	18.96798	19.0104	23.21972	22.08938	-0.01971	0.081799	-0.19449	-0.19205	0.099761
32.06998	32.97783	31.8979	29.60178	34.96859	-0.17469	0.023311	0.06759	0.017137	-0.08848
62.2809	60.10633	56.82395	60.08563	56.52514	0.075366	0.048491	-0.00269	-0.08385	-0.0042
33.40339	34.17574	31.00305	26.54173	29.89414	0.232938	0.036827	0.071299	-0.06874	-0.29863
20.75724	20.03678	19.44949	19.24633	21.19762	0.169181	0.015132	-0.03772	-0.07976	-0.09532
17.18783	19.08531	16.28906	17.71148	17.21578	0.133044	-0.05361	0.096535	-0.13622	-0.0107
114.1973	117.927	174.7772	133.7899	143.6679	-0.15057	-0.1568	-0.11476	0.442146	0.046143
118.4733	77.17558	129.8806	151.5345	118.1187	0.031812	-0.00265	-0.62011	0.081344	0.329742
140.8848	155.412	233.4774	194.8898	189.1666	-0.28809	-0.23762	-0.09549	0.463916	0.21802
63.30591	69.82369	63.42667	57.1912	58.80336	-0.12564	0.10027	0.238716	0.100507	-0.0431
638.7565	738.1462	545.6987	567.2617	571.7175	0.007107	0.05968	0.265172	-0.16726	-0.11498
90.06104	85.30195	80.81042	67.6853	73.95991	0.109741	0.143277	0.062517	-0.02362	-0.27906
39.75175	36.77725	37.22308	41.24411	40.85455	0.081656	-0.00733	-0.11935	-0.10285	0.04603
28.22411	28.62268	34.04776	27.6744	29.56981	-0.19763	0.001665	0.017422	0.266878	-0.03605
18.74383	17.04053	15.92885	18.41421	18.69373	-0.04237	0.056055	-0.08462	-0.18514	0.030595
19.39449	20.85675	18.79537	20.23768	19.47022	-0.13455	0.017995	0.123598	-0.02505	0.079577
22.78875	19.20584	18.83692	19.97903	19.69219	0.092835	0.160681	-0.09037	-0.11434	-0.02859
50.33849	55.23818	47.80217	43.25498	51.38748	0.01832	-0.01748	0.121535	-0.08747	-0.23263
23.43766	20.69729	23.06563	21.14493	21.88352	0.097908	0.058533	-0.12183	0.035301	-0.0911
31.87811	28.32949	28.70454	28.38094	26.32641	-0.09874	0.15035	-0.01942	4.45E-06	-0.01882
186.8654	181.1611	145.9834	170.5596	176.7087	0.135569	0.073994	0.032244	-0.28105	-0.059
48.21144	45.43779	42.31706	45.01399	43.75441	-0.13945	0.107853	0.023228	-0.08031	0.010654
47.74509	42.96072	35.95063	35.97986	36.64723	0.198338	0.243219	0.085909	-0.17011	-0.16864
459.4261	469.9149	406.6527	380.0152	396.6064	0.066746	0.11679	0.149035	-0.05945	-0.15644
82.15947	59.01946	68.20644	77.60351	57.30895	0.601931	0.211296	-0.26138	-0.11127	0.143902
75.07092	92.1525	77.11758	85.67179	88.55943	0.079302	-0.19561	0.108981	-0.14783	0.004143
23.7717	24.15944	20.93656	24.04752	23.63312	0.114873	-0.00873	0.021587	-0.18832	0.01406
52.5905	55.33533	53.48912	46.50313	47.37819	0.172258	-0.00185	0.071573	0.017049	-0.17794
164.0613	157.3991	243.4712	188.5277	180.8224	-0.10957	-0.10407	-0.16994	0.430255	0.097061
26.39806	25.42629	26.52159	27.89639	22.82159	0.033648	0.037834	-0.01614	0.040568	0.11392
46.08945	40.65979	41.64656	44.71832	37.58163	-0.0908	0.156558	-0.02511	0.006205	0.109701
19.74821	23.47928	20.77741	21.2493	20.24485	0.17793	-0.12934	0.119382	-0.05813	-0.02866

63.59187	76.5886	58.76485	72.05981	62.66761	-0.12304	-0.01829	0.260457	-0.122	0.172999
72.87579	62.87073	67.14575	64.48729	67.09551	0.11182	0.091979	-0.1215	-0.02804	-0.08778
316.0393	208.4852	355.8457	339.1158	274.6244	0.289982	0.049736	-0.55425	0.196299	0.155103
32.06569	25.52152	26.06334	25.97776	25.62376	0.088228	0.268574	-0.05778	-0.02879	-0.03337
71.49738	85.33749	77.39217	75.97113	75.8169	0.065826	-0.13604	0.119697	-0.02658	-0.05072
21.30649	17.48006	18.46073	19.26799	16.08683	0.015926	0.206978	-0.07595	0.004098	0.064541
45.2365	48.2925	63.96083	59.97465	56.04615	-0.11667	-0.22352	-0.123	0.276613	0.182203
803.9905	942.6249	610.0236	382.6654	415.2687	0.217436	0.070749	0.303637	-0.45614	-1.04766
76.02042	72.2398	54.44519	60.40838	62.03169	0.350211	0.214781	0.142339	-0.25061	-0.10142
99.33887	104.9779	112.5342	109.9195	93.3308	-0.03556	-0.05698	0.022312	0.122197	0.088952
52.89652	62.23262	82.58349	59.79451	75.74098	-0.31312	-0.22815	0.007849	0.393674	-0.07017
318.3108	383.4016	193.0193	202.4163	176.5867	0.212099	0.441437	0.664677	-0.28849	-0.22059
29.06584	27.00937	35.25678	27.33324	26.04963	-0.06549	0.093635	-0.01183	0.369208	0.002175
27.96166	30.95864	28.15803	27.08874	29.75761	0.141082	-0.0601	0.085532	-0.0493	-0.10728
20.35734	21.62273	23.29845	25.22135	25.59783	-0.08368	-0.12829	-0.03752	0.06983	0.184776
111.9003	93.49058	74.16367	105.9336	85.92965	0.095262	0.221434	-0.03867	-0.39398	0.113341
30.80377	32.83056	38.02401	34.58695	35.10515	-0.11855	-0.08361	0.008394	0.214347	0.079847
43.3795	47.42339	31.29955	27.44499	31.9868	0.485679	0.172331	0.30232	-0.31836	-0.50535
84.16988	97.0555	82.46267	95.64056	74.60455	-0.08955	-0.00433	0.196261	-0.03758	0.178269
41.02198	36.83375	34.65403	34.79511	37.54459	0.147082	0.08822	-0.06783	-0.15755	-0.15798
48.32699	43.83614	47.5447	46.53059	42.39035	0.040044	0.04255	-0.09932	0.020136	-0.01236
25.75063	27.82962	33.09579	29.1508	29.02724	0.051758	-0.15095	-0.0397	0.204971	0.027051
26.86929	22.4576	22.28797	26.66594	24.50815	0.138465	0.085186	-0.16941	-0.18178	0.07806
17.63056	16.5733	17.06695	17.98034	18.63073	0.159475	-0.03983	-0.13004	-0.08928	-0.01489
18.42223	18.24857	22.03256	18.24843	19.2371	-0.04659	-0.00957	-0.02315	0.246943	-0.02612
18.91865	17.79783	23.4705	19.68356	19.88598	-0.02902	-0.03917	-0.1239	0.274641	0.018165
39.57864	36.18064	34.35664	41.71351	34.42455	-0.00451	0.100873	-0.02952	-0.10209	0.17391
18.85236	18.73503	19.03023	17.64323	20.45313	0.061999	-0.01216	-0.0206	-1.51E-04	-0.10773
46.29665	68.50005	38.6056	39.93795	47.60829	-0.28522	0.069113	0.570155	-0.19216	-0.15012
48.24962	48.74858	48.74397	48.53479	54.12661	-0.01436	-0.02205	-0.00731	-0.0077	-0.01415
28.7363	31.25721	28.49835	25.83278	25.80699	0.030101	0.013167	0.135521	-0.00347	-0.14066
27.9016	26.59452	22.76784	24.2942	22.5542	0.018787	0.117215	0.047404	-0.18341	-0.09351
115.6465	118.1161	145.9572	138.5233	137.2714	-0.17892	-0.09187	-0.06062	0.244021	0.167705
23.97147	25.98343	23.79574	23.61225	24.93393	0.318318	-0.01924	0.090278	-0.03001	-0.0439
23.84022	29.1013	23.54882	27.876	29.30738	0.18222	-0.22318	0.066859	-0.24677	-0.01077
22.0452	21.57789	21.00644	22.11816	17.91441	0.158638	-0.0117	-0.04675	-0.08595	-0.01698
35.9013	33.52568	34.78091	41.61702	35.69296	0.080431	0.028797	-0.06999	-0.01638	0.234938
35.57068	35.73905	34.97092	31.72982	34.79702	0.224124	-0.00177	0.005962	-0.02795	-0.17409
21.81109	18.82252	17.88243	19.00462	18.81941	0.010787	0.197831	-0.01452	-0.08935	8.42E-04
42.58609	39.83816	35.60268	39.91627	40.10276	0.1234	0.082674	-0.01151	-0.17644	-0.00777
21.96434	21.43377	20.13996	19.08292	20.16318	0.226395	0.077202	0.048952	-0.04048	-0.12246
22.72516	20.43934	22.57462	25.66214	23.28095	0.077697	-0.02681	-0.17769	-0.0338	0.151174
29.69393	24.80606	24.33325	25.70439	27.26257	0.201574	0.139496	-0.12352	-0.14619	-0.07029
17.64367	18.59312	19.71851	16.74892	17.0224	0.041834	-0.05038	0.027735	0.112524	-0.1229
31.36384	28.55667	28.01969	25.93183	27.01218	0.182033	0.130815	-0.00536	-0.03618	-0.14858
26.96406	29.83183	26.34377	25.08233	25.40884	0.238607	0.016678	0.161914	-0.01588	-0.08769
37.23402	37.75555	37.57658	33.69238	37.44696	0.085516	-0.00565	0.011158	0.007317	-0.15173

19.70677	19.36643	17.8162	19.22227	17.16426	0.040768	0.040544	0.01652	-0.10424	0.001681
27.70427	33.60841	33.42865	27.92819	24.91721	0.153556	-0.09179	0.174703	0.171515	-0.08364
27.01933	29.30164	31.12179	32.21634	27.85854	0.050639	-0.09226	0.023827	0.107249	0.160457
45.35735	40.54242	55.45949	55.86681	52.25962	-0.17028	-0.04414	-0.19477	0.254086	0.251467
255.271	238.0628	313.308	279.9514	291.9812	-0.25439	-0.02229	-0.12485	0.265298	0.098044
1125.385	1315.514	1055.18	1065.443	1048.636	0.08956	-0.00481	0.222405	-0.09885	-0.08516
1602.963	1670.815	1275.116	1375.467	1438.867	0.122639	0.051195	0.11443	-0.28466	-0.17263
240.3936	247.3641	280.6577	294.0092	268.9999	-0.24751	-0.11158	-0.07216	0.110174	0.17331
121.1985	112.2612	116.7723	177.1175	160.0685	-0.03516	-0.10383	-0.20902	-0.15997	0.438271
170.9762	142.0214	162.6255	162.1604	143.3378	0.062566	0.112708	-0.15408	0.040837	0.035703
259.2871	261.3387	265.9492	292.1665	286.0547	-0.1995	-0.01592	-0.00537	0.020617	0.155755
201.5292	196.1662	213.9178	226.6946	213.5566	-0.12167	-0.03555	-0.07473	0.048986	0.132904
407.5922	442.9186	640.9844	568.6565	499.8044	-0.19728	-0.10758	0.010946	0.525532	0.332824
759.7791	917.2333	1272.099	971.6313	1086.509	-0.21763	-0.26647	0.005692	0.450918	0.046052
32.76025	33.59135	23.25451	31.3508	31.8984	0.129237	0.074969	0.105925	-0.41678	0.016335
147.7758	151.9706	154.93	179.4705	175.8626	0.033959	-0.10351	-0.06324	-0.03382	0.174772
232.7607	218.6362	182.3098	181.2159	158.4637	0.01611	0.207364	0.115195	-0.15344	-0.15665
496.9099	537.5989	422.588	429.2225	441.2571	0.061406	0.078271	0.192065	-0.15728	-0.13324
783.5026	924.5482	952.6563	816.4394	899.8067	-0.19732	-0.16661	0.074709	0.112374	-0.10589
809.0618	765.764	437.6061	570.7889	538.4988	0.245595	0.340799	0.267889	-0.57561	-0.14924
99.50022	111.6907	68.78702	100.1648	88.00665	-0.09772	0.108844	0.281591	-0.43148	0.096421
153.8062	167.5114	128.6685	121.7861	128.952	0.007918	0.096014	0.22076	-0.16348	-0.26102
1107.01	1037.712	1098.69	1099.281	1194.127	0.006229	0.015006	-0.07811	0.002905	0.005068
223.5943	222.8296	174.3135	200.5379	201.302	0.080273	0.040454	0.034685	-0.31875	-0.12279
88.27691	90.88883	83.82005	77.54524	74.92276	-0.04795	0.075696	0.11823	0.002838	-0.1108
109.1984	105.2255	125.1952	97.84914	118.2359	-0.18191	0.032936	-0.01736	0.227703	-0.12488
309.4829	378.9783	347.8412	319.6958	359.7457	-0.2722	-0.10813	0.189949	0.062429	-0.05273
895.0786	1155.833	1017.254	920.6802	937.1706	-0.00887	-0.1092	0.260959	0.075775	-0.08154
448.5599	486.0531	524.291	401.023	516.1339	-0.18434	-0.08504	0.034979	0.13871	-0.25061
237.6441	243.1492	173.5831	220.0967	172.939	0.103619	0.107251	0.12797	-0.37841	-0.00271
1040.072	1064.73	822.8769	958.1958	775.6118	0.012801	0.077671	0.108834	-0.27334	-0.04507
844.0394	928.9382	668.9488	736.8384	686.5145	0.075941	0.134227	0.275302	-0.21437	-0.0629
350.7044	416.243	323.7507	321.6528	301.1929	0.150045	0.008442	0.230222	-0.10885	-0.12372
790.4421	922.9802	901.6963	828.1186	829.8149	-0.13423	-0.08395	0.138224	0.103634	-0.02239
500.3178	582.3889	368.8686	391.9453	454.3291	0.071607	0.131385	0.324462	-0.31317	-0.2342
40.69238	41.77401	37.27086	43.65135	35.59043	0.157358	-0.00284	0.032689	-0.1446	0.095266
147.8003	127.0849	115.0026	114.5761	116.9964	0.365366	0.252372	0.03071	-0.11597	-0.11597
262.7514	234.1682	208.6067	198.0174	193.2906	0.231993	0.163788	-0.00257	-0.1761	-0.2512
39.30453	44.70973	54.04735	45.63927	46.3899	-0.22879	-0.15422	0.034387	0.307052	0.049894
168.557	194.2844	161.9724	147.3114	145.3466	0.058623	0.024642	0.230758	-0.03204	-0.1792
1184.433	1032.193	749.1135	948.6843	816.5223	-0.05563	0.334174	0.138324	-0.36648	0.016425
258.9035	290.0019	209.9325	223.939	227.6242	0.096858	0.042922	0.202622	-0.26801	-0.16624
511.3911	514.528	569.538	544.2723	562.0277	-0.1724	-0.0692	-0.05845	0.087997	0.01928
513.1769	566.0393	580.9326	587.1186	629.6279	-0.10289	-0.14183	-8.14E-04	0.031818	0.050567
210.5479	206.7674	291.2668	226.9235	294.2358	-0.03645	-0.08761	-0.10977	0.35026	0.019711
74.48452	71.92954	44.0626	43.41723	46.61509	0.033191	0.368228	0.316455	-0.39361	-0.40567
219.8701	245.9085	184.2857	187.551	197.8452	0.106663	0.014998	0.176473	-0.26037	-0.22353

217.174	201.6511	210.4683	234.6462	238.9622	-0.17147	0.025969	-0.08672	-0.02031	0.136112
130.3903	141.3763	168.366	148.2776	170.3056	-0.15592	-0.16645	-0.054	0.196447	0.018826
183.8853	155.4047	171.9832	178.3275	180.8257	-0.12699	0.072306	-0.16931	-0.02794	0.028199
293.6713	267.9043	343.1532	320.0072	306.2558	-0.03486	-0.02219	-0.16106	0.197461	0.098703
23.72047	27.50931	40.27863	41.16855	34.68379	-0.09869	-0.51475	-0.30055	0.260252	0.273672
412.0761	424.6065	418.4823	405.2776	401.7383	0.096396	-0.00404	0.039612	0.018396	-0.02755
1168.968	1121.876	1297.339	1335.068	1242.974	-0.12474	-0.05476	-0.11373	0.097196	0.135599
45.44919	37.95134	46.36063	44.50237	44.10692	-0.02974	0.046303	-0.21308	0.074407	0.014414
418.704	388.6539	336.6917	319.7404	303.4735	0.027598	0.252394	0.142184	-0.06408	-0.13784
618.3351	544.8167	629.2851	568.6829	579.2598	-0.03471	0.052671	-0.13048	0.077996	-0.06936
122.8681	113.4912	137.1952	142.5502	139.4506	-0.0839	-0.03122	-0.14942	0.127982	0.172283
354.6476	371.8283	294.5513	351.3075	285.5424	0.006045	0.065838	0.136405	-0.20354	0.055687
204.321	181.124	119.7474	106.4596	124.8785	0.235525	0.452639	0.265585	-0.33085	-0.49095
310.203	327.2927	468.1191	353.5728	364.2081	-0.20936	-0.15957	-0.07765	0.430427	0.025425
271.4583	286.7031	296.7702	274.3571	248.5327	-0.13925	-0.00488	0.072249	0.122045	0.008383
602.4381	604.1175	664.4841	702.5167	723.8165	-0.09043	-0.08693	-0.08053	0.052557	0.136377
175.5381	127.5979	137.3747	133.7954	126.5073	0.53619	0.309535	-0.1781	-0.06417	-0.09207
589.8137	465.1496	574.316	643.6719	600.503	-0.09285	0.038348	-0.30216	0.004513	0.169911
142.2116	159.4875	183.2037	158.5888	164.2766	-0.12958	-0.15193	0.01243	0.209229	0.004056
585.1714	630.7692	734.7662	685.6296	700.2004	-0.09494	-0.13042	-0.02076	0.191432	0.099513
452.9294	504.2785	331.4186	379.0436	320.1244	0.312502	0.131129	0.285458	-0.33654	-0.11932
137.611	129.5855	274.7477	210.8241	201.026	-0.59501	-0.19054	-0.29093	0.746647	0.360752
347.0533	393.8321	459.8745	370.7601	423.8054	0.013819	-0.18033	3.42E-04	0.218878	-0.09139
75.74491	90.2832	89.70878	107.7903	91.98179	-0.07191	-0.2325	0.028956	0.016205	0.27461
357.8346	477.0291	231.7144	233.9774	266.2962	-0.0487	0.311222	0.619724	-0.31585	-0.31005
328.8204	353.6653	358.2909	400.2581	355.4342	-0.04419	-0.11583	-0.01077	0.006196	0.164924
742.4663	802.3725	876.2581	817.6881	838.9949	-0.10815	-0.12362	-0.01127	0.116638	0.015886
1881.749	1804.662	2182.043	2398.317	2419.868	-0.04473	-0.16078	-0.22334	0.056184	0.189303
158.499	146.4919	203.295	167.4454	195.3729	-0.14127	-0.04427	-0.16003	0.312332	0.035578
246.375	268.8505	227.8271	241.9177	207.1258	0.136564	0.014886	0.140725	-0.10465	-0.0132
70.45064	64.15411	80.71761	67.02274	73.97343	-0.30803	0.046188	-0.08746	0.230458	-0.0282
122.7301	141.4321	161.2697	134.3106	150.8435	-0.0646	-0.19422	0.011063	0.198782	-0.06594
651.9621	573.9139	703.6579	701.6829	668.0461	-0.14351	0.016268	-0.16683	0.123477	0.118479
1041.782	1108.828	974.5249	945.5598	993.832	0.169621	-0.00953	0.082064	-0.10454	-0.15035
286.7205	293.5984	220.3829	220.6427	226.3586	0.105407	0.153573	0.191205	-0.23378	-0.22795
674.212	533.7101	457.6422	476.2648	539.0291	0.252906	0.316429	-0.02387	-0.24982	-0.19445
1488.906	1405.992	1279.205	1191.923	1215.501	0.155307	0.171294	0.100833	-0.03607	-0.13792
40.22831	40.46651	36.64317	35.63483	34.04869	0.532726	0.062592	0.057742	-0.06176	-0.10828
420.3999	452.7161	599.1314	544.504	520.8277	-0.22792	-0.15439	-0.04555	0.349494	0.216832
339.0471	289.2481	187.5026	259.8115	286.57	-0.06382	0.299314	0.077748	-0.58157	-0.07674
38.37413	34.60793	48.12769	41.01544	42.92056	-0.13771	-0.0148	-0.16302	0.307557	0.075964
675.5831	733.2206	1149.632	825.1108	938.5872	-0.20433	-0.25196	-0.13208	0.495328	0.023311
302.5977	303.3388	420.8303	305.0989	341.3653	-0.22916	-0.02701	-0.0247	0.431421	-0.02842
136.8723	149.1768	218.6775	172.2973	189.8821	-0.2993	-0.20413	-0.08237	0.467412	0.119889
170.525	172.7022	235.577	187.5641	227.0192	-0.17603	-0.08292	-0.06687	0.351715	0.052375
227.1904	160.6544	147.6497	193.2728	180.2289	0.402184	0.303417	-0.19041	-0.34992	0.068475
164.9705	165.2287	141.7895	152.7775	154.3247	-0.1122	0.110097	0.112556	-0.10696	1.57E-04

100.656	110.9804	106.9678	105.4466	110.5218	0.162539	-0.12863	0.013267	-0.04366	-0.06196
68.18873	74.9881	78.22263	80.22723	76.53803	-0.08964	-0.147	-0.00978	0.051208	0.084542
63.19375	58.22416	96.63561	87.49176	100.536	-0.0825	-0.39194	-0.51192	0.213948	0.082955
111.2907	113.2231	78.3279	98.84779	102.0848	-0.0501	0.119908	0.148713	-0.38323	-0.04727
341.9803	350.8474	255.9913	320.3263	342.4356	-0.08092	0.074698	0.114834	-0.34573	-0.01672
85.02749	51.83242	42.03977	44.70699	40.30027	0.125377	0.886063	0.183633	-0.1026	-0.0167
52.86408	48.96027	102.5757	67.05728	71.09361	-0.39628	-0.07714	-0.22639	0.828	0.191228
22.37963	21.72809	22.95169	20.28052	25.36902	0.054105	-0.00215	-0.04505	0.032783	-0.14364
229.1666	214.8881	198.2123	215.0055	195.7462	0.195262	0.105761	0.013484	-0.10418	0.005526
240.7832	214.7938	187.7209	221.0529	163.547	0.229011	0.135842	-0.02976	-0.22715	0.016039
252.9179	266.3739	240.8826	226.9861	209.9711	0.153778	0.006821	0.082479	-0.06361	-0.1484
192.932	231.0845	164.3883	190.5954	191.9259	0.105102	0.018453	0.288393	-0.20676	0.005299
92.83772	101.3043	115.6076	106.4804	107.5639	0.037326	-0.1772	-0.05155	0.135163	0.02083
120.3117	121.7572	189.1036	152.882	173.1316	-0.06396	-0.21492	-0.20292	0.411768	0.125096
44.21736	40.71204	58.31029	44.93781	46.95416	-0.06397	-0.00749	-0.12107	0.387394	0.015803
3219.581	3636.389	3249.669	2774.769	3070.466	0.055227	-0.04132	0.132897	-0.03545	-0.26792
140.706	161.8904	152.6037	147.7769	134.6929	0.111862	-0.09204	0.110107	0.025972	-0.02724
117.0821	105.9482	95.29554	108.1745	97.97763	0.006216	0.170569	0.027502	-0.12601	0.054516
127.0901	116.4455	99.52594	115.6426	108.9578	0.155588	0.13567	0.017282	-0.21676	0.005902
260.3503	278.6363	266.1507	298.3097	291.4395	-0.23728	-0.09183	0.011896	-0.05662	0.107943
163.4006	165.6552	233.9856	181.44	181.9593	-0.07726	-0.08443	-0.06145	0.421413	0.048328
17.63307	20.17547	19.54258	16.18499	17.42682	0.086356	-0.11079	0.093624	0.045844	-0.22752
46.98375	47.0252	42.32722	51.07473	47.38016	0.124981	-0.05426	-0.05442	-0.21197	0.067125
266.205	261.8244	360.5113	281.8757	299.1164	-0.06933	-0.02398	-0.0509	0.407262	0.054793
19.43051	17.9146	23.38779	17.54391	17.90334	-0.04351	0.096658	-0.01678	0.367373	-0.04752
111.5437	102.743	127.2689	123.1545	129.5402	-0.13461	-0.07806	-0.20543	0.110092	0.064868
129.902	103.5736	150.3441	138.2721	131.4194	-0.09914	-0.01181	-0.33699	0.198617	0.064564
156.9135	118.705	140.92	121.7586	132.0613	-0.00752	0.233101	-0.1721	0.077222	-0.13448
47.0206	49.30925	37.31577	43.34473	34.42949	-0.07714	0.150018	0.231108	-0.18678	0.042064
212.8596	203.9086	255.7386	204.0607	208.471	-0.22613	0.031973	-0.0297	0.293414	-0.04466
114.8988	113.4561	109.2916	130.9547	96.31295	0.065435	0.001951	-0.02168	-0.06929	0.188408
24.33747	18.32591	29.50062	19.79065	25.63258	-0.15737	0.181372	-0.23278	0.450023	-0.11714
125.0565	133.8613	143.0075	152.6804	159.422	-0.14192	-0.16077	-0.06433	0.028661	0.119887
187.8475	190.9417	188.4879	205.8131	197.0831	-0.10269	-0.04617	-0.0229	-0.0422	0.086619
29.22941	25.33576	23.43978	22.45199	24.10987	0.322914	0.176249	-0.03534	-0.14357	-0.20275
39.90968	29.34963	26.8804	30.36845	26.77965	0.627291	0.339811	-0.11895	-0.23057	-0.06417
671.2372	627.5462	511.741	596.8409	401.098	0.149626	0.167266	0.069657	-0.27235	0.001886
70.85702	47.61609	74.84344	44.85005	62.71306	-0.46461	0.405532	-0.13626	0.489185	-0.23491
257.8536	208.8498	307.1234	269.6994	277.2061	-0.07605	0.039502	-0.26379	0.278227	0.094725
101.6906	110.9441	136.278	117.1866	123.8575	-0.33396	-0.12867	0.003739	0.286598	0.073251
284.5188	211.9939	217.0315	212.483	220.8767	0.129118	0.3271	-0.09344	-0.06191	-0.09318
136.1718	151.4535	120.5213	135.0228	110.8969	0.096786	0.045351	0.188898	-0.13052	0.03215
318.2292	385.7269	265.2529	279.5654	293.1938	0.034819	0.049587	0.329999	-0.22066	-0.13836
66.42893	83.26002	55.84973	54.66439	62.56389	0.311108	-0.05339	0.275408	-0.31635	-0.33147
72.833	86.5202	63.20061	62.05273	69.91203	0.183538	-0.06527	0.193365	-0.27828	-0.29048
138.0267	165.2703	82.62017	107.2189	107.8887	0.103074	0.271462	0.534135	-0.48073	-0.08248
33.1084	33.41941	25.03625	33.89663	28.75624	0.082684	0.076697	0.083376	-0.3268	0.1028

88.73153	78.50733	66.84698	89.10952	61.21687	-0.00664	0.185591	-0.01204	-0.23216	0.191051
302.0378	361.0685	278.8376	288.4217	301.1087	0.043151	-0.04786	0.210162	-0.16783	-0.12024
107.8432	101.0904	131.0453	140.6576	155.6523	-0.00753	-0.1295	-0.22427	0.13823	0.239436
5683.65	6403.023	6386.346	7036.617	6204.71	-0.14231	-0.14465	0.027378	0.021797	0.155893
360.7003	288.8434	170.013	224.337	220.4239	0.257627	0.468191	0.141192	-0.70594	-0.22036
53.12621	49.57319	37.03087	47.90312	50.0886	-0.02583	0.160544	0.053099	-0.37277	0.008464
1589.169	1399.486	1183.269	1209.006	1177.43	0.237504	0.250394	0.074916	-0.1655	-0.15189
324.4385	280.5892	347.923	369.0705	343.7911	-0.07746	0.003529	-0.21503	0.101864	0.181792
46.88742	50.48773	55.76337	57.49696	61.49318	0.077062	-0.20487	-0.09853	0.046583	0.085966
61.04169	57.28098	66.62562	61.43606	59.02329	0.067139	-0.01418	-0.10906	0.111699	-0.00715
83.29015	75.68249	117.8146	100.0006	90.49063	-0.37588	0.027834	-0.1089	0.513271	0.244454
288.6677	227.6832	164.8431	195.2166	184.4644	0.422919	0.393811	0.050818	-0.44128	-0.1865
88.38068	96.0773	123.7472	102.8965	111.5155	-0.18379	-0.11664	0.003982	0.365373	0.103611
36.71285	39.57206	45.26465	78.51492	60.71954	-0.14673	-0.29833	-0.20647	-0.01548	0.776137
142.6942	163.4068	162.2492	170.6077	179.5648	-0.21967	-0.14167	0.050828	0.037512	0.115132
175.5788	184.0046	153.2261	153.2324	172.474	0.076734	0.045543	0.114313	-0.1476	-0.1477
278.9462	270.0371	356.0027	321.6319	317.8367	-0.10922	-0.07829	-0.12421	0.269936	0.110089
83.7781	85.28114	54.11512	62.85555	66.7484	0.047421	0.290764	0.31257	-0.34983	-0.11733
407.2121	404.3702	321.4679	338.2057	298.054	0.106535	0.106127	0.088859	-0.24722	-0.17727
73.93182	86.46869	65.69083	67.85043	74.01708	0.204573	-0.03781	0.188224	-0.20748	-0.17878
57.04053	39.20947	48.40776	53.48282	47.29588	-0.05297	0.166606	-0.38308	-0.06526	0.079826
34.77967	37.49204	44.61522	38.33531	40.09247	0.004322	-0.15607	-0.04704	0.200475	-0.0187
68.13296	79.0342	82.63931	80.69696	78.65635	-0.30801	-0.19077	0.030824	0.091287	0.054893
399.8541	351.511	386.126	412.3811	384.83	-0.04904	0.043227	-0.14139	-0.0065	0.0894
68.41816	65.2751	56.97391	63.0601	58.20514	0.151506	0.080578	0.017022	-0.18509	-0.0373
41.86419	49.05986	48.57146	52.07673	44.62871	-0.07914	-0.16128	0.070289	0.056887	0.153046
47.47419	40.70606	39.87273	39.82576	42.84588	0.03311	0.140719	-0.08167	-0.11306	-0.11866
41.15196	41.38044	48.34883	40.38948	53.44191	-0.02091	-0.06109	-0.05363	0.170721	-0.08897
57.32234	63.24261	67.0758	59.56207	61.69791	-0.25772	-0.03753	0.104831	0.186942	0.014678
132.0248	103.2041	93.63512	72.25791	80.33688	0.028205	0.463623	0.117638	-0.0384	-0.40658
651.126	552.8572	480.0219	577.8214	488.3571	-0.02408	0.229503	0.004271	-0.20987	0.066806
22.8219	21.1857	18.18099	21.35028	20.40261	0.164606	0.108169	0.006449	-0.21479	0.018239
38.33953	47.26324	47.95893	44.04042	41.50995	-0.03021	-0.16337	0.1395	0.158751	0.033325
26.78447	30.40191	35.80388	33.64984	31.64961	-0.13926	-0.25001	-0.06312	0.175341	0.085189
55.65149	68.81242	48.43098	56.65633	53.33906	-0.06105	8.93E-04	0.301476	-0.20046	0.026975
57.24939	63.81444	50.87405	51.80067	51.69445	0.055271	0.042751	0.197916	-0.13051	-0.10686
359.2274	341.1397	179.6286	159.4733	172.2871	0.128655	0.581722	0.556265	-0.51241	-0.58312
63.18172	60.70773	55.0557	57.45292	57.63194	0.168295	0.060026	0.001807	-0.14463	-0.08105
19.95406	25.99125	22.37058	21.38962	23.7231	-0.07132	-0.13404	0.248484	0.031072	-0.02811
318.9564	259.0927	337.5494	403.8432	351.4453	-0.03399	-0.05572	-0.35357	-0.00293	0.287869
76.74027	74.94907	57.79138	63.1144	61.02659	0.230574	0.181445	0.153056	-0.21926	-0.0952
104.0229	96.12214	82.21845	72.67665	81.74896	0.325033	0.203394	0.092547	-0.12624	-0.31838
199.1804	241.5062	244.1498	243.4184	266.8361	-0.32822	-0.28624	-0.00843	0.008022	0.003688
17.61362	16.30226	16.02231	17.84618	16.02984	0.208244	0.072932	-0.03629	-0.06065	0.091497
96.23382	88.0936	116.408	118.1627	106.9883	-0.0916	-0.04926	-0.18153	0.218795	0.242961
37.95202	41.91534	41.87796	39.54402	35.14205	0.125442	-0.07377	0.0679	0.068855	-0.01395
3377.976	2235.762	3541.299	3739.999	2695.737	0.087311	-0.0252	-0.64844	0.025007	0.107614

27.29648	27.54856	49.09708	47.02554	36.60505	-0.31751	-0.16106	-0.14663	0.626063	0.565279
33.99933	34.40461	29.18655	34.01619	37.30577	0.130669	-0.01898	-0.00354	-0.24021	-0.01755
19.59573	22.82538	23.31602	22.5053	24.9574	0.023585	-0.2389	-0.0241	0.011889	-0.04228
420.4572	595.5948	421.6336	398.0603	452.3963	0.046778	0.008276	0.510919	0.013174	-0.07064
150.8114	162.8395	117.2185	122.6602	117.9249	0.066893	0.109334	0.228151	-0.25473	-0.1927
129.5353	119.467	101.6163	89.25832	81.67867	0.185914	0.296019	0.196216	-0.04014	-0.22398
166.9617	144.6558	107.4104	122.3169	126.3075	0.213991	0.316987	0.111049	-0.3317	-0.15397
444.0406	331.2632	449.1348	423.8304	409.2716	-0.07802	0.058881	-0.36159	0.077683	-0.00697
26.8506	28.80597	27.80456	28.78413	31.25198	0.138233	-0.11394	-0.00992	-0.06262	-0.0121
31.42835	26.29448	27.44906	29.92225	28.46534	0.162971	0.122281	-0.13331	-0.07133	0.052748
39.77848	41.73814	41.25363	35.97882	40.88402	0.195014	-0.03345	0.041025	0.024816	-0.17878
35.15675	35.81563	23.14632	29.15553	30.72097	0.115047	0.189975	0.220564	-0.4085	-0.06909
139.5754	127.6433	161.1963	160.3066	162.1731	-0.17822	-0.05751	-0.18598	0.148344	0.13922
41.90927	38.01392	29.03394	41.149	38.8105	0.066717	0.078288	-0.09288	-0.45395	0.040519
40.12693	41.71968	29.61748	28.72357	33.1681	0.324613	0.146071	0.200009	-0.30091	-0.3391
36.49741	37.81206	30.566	32.34251	33.27298	-0.04274	0.088758	0.139728	-0.16934	-0.08517
20.06071	20.4001	17.89336	16.20247	17.19624	-0.01397	0.100263	0.123916	-0.06519	-0.21075
439.0898	353.7344	359.1333	417.4484	490.4392	0.022856	0.125477	-0.18247	-0.16906	0.050669
279.9237	288.3811	195.6347	195.846	208.3521	0.024937	0.315078	0.364109	-0.20566	-0.19519
71.21375	66.48736	60.97873	63.20036	59.9348	0.122483	0.118629	0.022093	-0.10529	-0.05369
121.5433	132.4684	154.1675	141.9373	146.4252	-0.0944	-0.19278	-0.06732	0.151614	0.030366
214.6361	192.6532	143.7022	155.3028	140.461	0.065962	0.248657	0.099545	-0.35947	-0.2199
140.1399	98.04342	79.94064	74.17068	73.84924	0.460152	0.523687	0.017151	-0.27371	-0.3658
147.3438	144.3309	163.4848	159.0884	169.1164	-0.07149	-0.04671	-0.07641	0.104282	0.064264
178.5972	180.6103	255.2618	206.6577	214.1524	-0.06573	-0.16498	-0.14722	0.347531	0.0278
205.858	179.6271	159.7346	150.7924	162.954	0.31933	0.197988	0.015799	-0.15415	-0.25198
171.325	176.4943	204.8191	200.0084	203.0593	-0.1482	-0.11746	-0.07611	0.138258	0.099241
25.69678	26.61176	27.28281	30.92515	25.13816	0.014186	-0.06239	-0.01186	0.022526	0.196938
26.81607	22.88416	24.96524	24.04674	27.43935	0.179252	0.034823	-0.18984	-0.06509	-0.12392
21.96722	23.09271	20.54133	27.49413	20.95548	0.187715	-0.05385	0.017019	-0.15096	0.266739
59.68832	55.16306	53.43129	45.52234	57.63403	0.198589	0.035216	-0.08058	-0.13478	-0.36903
47.74258	46.72337	49.5129	51.45136	46.00824	-0.15605	0.029786	-9.36E-04	0.083365	0.135598
90.47976	80.30481	48.6046	46.43338	49.50989	0.346278	0.595609	0.342018	-0.32387	-0.37317
74.13871	76.76447	95.69758	94.49704	95.89481	-0.22045	-0.22738	-0.17634	0.138657	0.122948
41.21711	36.09802	38.57646	42.52522	40.41018	0.193832	0.001035	-0.19379	-0.10207	0.047722
70.631	54.30972	45.68123	44.62478	44.08321	0.371953	0.366493	0.002554	-0.2684	-0.33559
17.83479	20.65715	25.84633	21.69088	21.96179	-0.2424	-0.23718	-0.03602	0.301297	0.047808
81.96859	69.00481	80.49561	77.48748	62.65181	-0.08312	0.147371	-0.10682	0.114298	0.060853
40.61801	40.54968	35.66944	42.26555	38.78743	0.215881	0.026447	0.02589	-0.16585	0.082699
39.51127	44.11748	40.5849	38.85867	38.89157	0.127721	-0.04469	0.115464	-0.00268	-0.06753
72.05412	70.28444	65.1477	39.43341	52.27827	0.0942	0.195601	0.170575	0.001347	-0.6635
35.89886	22.665	22.65708	24.84699	24.1368	0.107579	0.485586	-0.17274	-0.19627	-0.04491
199.2952	223.9122	174.6169	169.8932	138.4629	-0.05523	0.150941	0.312644	-0.03633	-0.07775
123.0707	118.7994	143.0909	146.3409	146.5462	-0.07528	-0.11813	-0.17129	0.098747	0.133747
35.98629	37.35016	38.08139	33.30321	40.27934	-0.0078	-0.05253	0.002868	0.030691	-0.16517
20.52776	23.34369	27.02165	25.87306	22.18784	-0.05848	-0.14621	0.042363	0.241497	0.190986
20.6761	19.51174	17.92389	18.42898	17.66906	0.176724	0.117148	0.037032	-0.0855	-0.04516

88.67469	61.58644	70.82607	71.18677	73.56791	0.623705	0.273167	-0.26458	-0.04165	-0.04179
38.88255	27.35055	38.05457	34.82649	35.26069	-0.06714	0.16328	-0.3485	0.128627	-0.01627
19.5195	24.51689	24.15119	21.87728	22.59546	0.111805	-0.27612	0.054971	0.031319	-0.11644
20.92915	17.76676	20.27661	18.55989	17.65709	0.06611	0.12463	-0.11213	0.078705	-0.04797
429.0896	496.2901	536.178	529.4704	605.1514	-0.15355	-0.27024	-0.05859	0.054109	0.035397
17.36371	17.36272	16.12467	16.04561	15.20438	0.209601	0.057211	0.054704	-0.05706	-0.05812
28.15422	27.65589	26.16499	32.54804	27.20182	0.100492	0.024684	-0.00211	-0.08088	0.231015
15.68368	18.20923	18.32337	20.49295	16.74556	-0.1033	-0.1588	0.056025	0.066811	0.22949
21.27521	20.24975	22.14018	23.0163	21.53984	-0.12122	0.005054	-0.06677	0.061394	0.113913
48.6687	44.98296	36.95918	50.48257	36.46007	0.066315	0.115643	0.001751	-0.28604	0.164196
38.54114	32.73574	31.63234	38.87584	27.94701	-0.07	0.196864	-0.03263	-0.08557	0.210801
175.418	146.831	203.6753	187.4763	184.8888	-0.16389	0.025818	-0.23451	0.233527	0.106182
234.6368	221.2697	171.6773	158.1006	171.4457	0.28208	0.177758	0.09128	-0.28478	-0.42047
18.97323	18.84922	18.62648	19.74679	21.6288	0.135846	-0.08553	-0.09233	-0.11122	-0.02603
60.27702	63.38841	43.6801	47.64115	47.30553	0.147893	0.14061	0.205814	-0.33609	-0.20435
22.35406	25.43917	27.89685	26.4251	28.0671	0.159053	-0.23696	-0.04444	0.088912	0.004709
51.31081	50.08961	47.44313	44.07936	45.31693	0.116901	0.087156	0.054172	-0.02391	-0.13379
105.2624	112.6706	106.2479	117.7372	109.8679	0.132823	-0.07952	0.017828	-0.06854	0.080894
362.3727	440.9165	237.4607	257.6312	256.3502	0.095339	0.282758	0.547767	-0.33712	-0.19498
175.4236	188.9233	224.3019	192.3353	233.1733	-0.36381	-0.10246	0.001748	0.246775	0.031091
112.2947	97.45936	129.7142	123.0863	127.9885	0.11505	-0.12318	-0.33268	0.084935	0.007146
44.05836	36.53107	36.32531	40.56716	41.2177	0.117612	0.105683	-0.16428	-0.1759	-0.01287
134.841	141.4933	167.5801	125.4695	148.3352	-0.12757	-0.07255	5.41E-04	0.239559	-0.17739
20.12945	20.62608	21.62439	22.36702	20.47198	-0.18586	-0.05226	-0.01527	0.05196	0.101253
60.62991	53.71344	56.17352	73.63964	67.86965	-0.05585	-0.01814	-0.19701	-0.12896	0.25734
32.98517	29.62951	26.52547	30.21917	28.31563	0.198905	0.11028	-0.04364	-0.20882	-0.01878
18.47661	17.03972	15.57111	16.79534	15.72511	0.287026	0.110252	-0.00591	-0.13436	-0.02564
27.94761	28.82838	26.46974	35.89779	28.67725	-0.00844	-0.02999	0.019906	-0.1034	0.332364
41.32013	33.93837	48.58582	33.14114	39.58848	-0.21223	0.133443	-0.14062	0.367039	-0.18987
30.23696	29.97893	24.59123	28.48952	26.53712	0.093146	0.076653	0.066288	-0.22464	-0.00953
42.51042	44.19566	45.39378	39.79909	45.91259	-0.18072	-0.0185	0.039559	0.077728	-0.11179
105.643	89.84583	88.18442	69.15374	69.94291	0.08987	0.460407	0.227064	0.178504	-0.16114
23.21192	21.27804	26.17732	24.62068	25.09486	-0.2501	-0.02215	-0.14985	0.15212	0.054118
27.26415	29.23327	30.86243	29.72125	26.82365	-0.07424	-0.0659	0.034647	0.113124	0.054563
37.37458	41.46347	35.08046	34.385	34.5829	0.25239	-5.88E-05	0.156414	-0.09127	-0.11398
16.65092	17.536	23.01167	18.9578	17.77886	-0.09627	-0.07513	-0.00806	0.387457	0.106447
24.83288	27.44885	27.72326	28.75493	24.86764	-0.13961	-0.09808	0.053763	0.067013	0.120538
29.87872	23.89295	32.24756	27.39475	30.80941	0.152183	-0.06005	-0.39336	0.056489	-0.17999
24.06809	29.44342	28.10587	29.52889	25.78145	0.160849	-0.25961	0.03609	-0.02503	0.046708
87.02997	91.91624	135.5348	71.15775	105.6864	-0.30154	2.70E-04	0.103084	0.595563	-0.29931
2508.282	2110.599	1039.98	1696.099	1571.553	0.375793	0.372984	0.154282	-0.91411	-0.19223
1720.29	1508.667	808.4302	1126.518	1210.434	0.331346	0.265418	0.098214	-0.83172	-0.34714
324.2353	259.1617	233.3815	251.8985	235.8876	0.817707	0.341379	-0.00433	-0.12937	-0.02959
1056.383	920.9312	1199.665	1160.726	1267.165	-0.13071	-0.07595	-0.27187	0.110968	0.063112
179.5667	171.3536	158.427	172.4506	149.4598	0.07655	0.068929	0.002034	-0.11941	0.011273
409.819	467.5533	707.2061	572.3913	517.0091	-0.31068	-0.231	-0.03576	0.534381	0.22587
104.8481	115.9922	134.1558	112.4512	120.0922	-0.34524	-0.11002	0.036306	0.248381	-0.0054

23.26022	25.57307	21.71996	22.68585	24.87898	0.25821	-0.02597	0.107057	-0.12667	-0.06174
50.62815	46.86363	61.89005	54.15667	62.05608	-0.09692	-0.07612	-0.18629	0.207258	0.019643
67.87845	64.22229	60.02163	70.2386	57.67991	0.084368	0.068687	-0.01062	-0.11525	0.111804
30.40793	26.0618	28.8168	26.78189	28.48151	0.328107	0.106916	-0.11618	0.035086	-0.07095
27.60192	24.49544	22.44617	23.11777	23.51641	0.015973	0.197929	0.028723	-0.09711	-0.05334
20.41822	17.22316	17.60741	16.19387	16.35348	0.083297	0.249579	0.007189	0.035876	-0.08696
82.38499	90.84622	78.72379	85.63302	83.9212	0.230359	-0.06354	0.075685	-0.13187	-0.00695
106.6763	109.2419	97.29825	176.5012	99.50244	-0.07433	-0.06426	-0.00652	-0.17557	0.65127
21.66599	26.4114	23.19219	19.96719	21.93557	0.106712	-0.14771	0.145141	-0.0405	-0.25909
137.3472	152.2276	181.9341	171.8682	186.4787	-0.29831	-0.24099	-0.09108	0.166556	0.084206
39.08833	31.85859	39.89384	35.96809	36.75854	-0.13932	0.084262	-0.21909	0.111815	-0.03579
41.40142	52.2753	34.09178	35.02137	37.60242	0.04248	0.041705	0.3647	-0.2711	-0.20662
35.49907	35.34109	46.26364	35.28405	36.53035	-0.19185	-0.02252	-0.03107	0.350527	-0.03126
19.49669	16.17842	22.36354	23.06689	21.94552	-0.16705	0.038661	-0.23133	0.218348	0.259421
39.19663	37.93864	31.15718	44.6662	35.64891	0.104417	0.010921	-0.03614	-0.33359	0.202878
64.63287	53.27553	48.08723	71.10617	63.14452	0.043042	0.17354	-0.09883	-0.24541	0.316607
331.6029	330.256	365.2529	352.4011	388.6414	-0.19092	-0.04402	-0.05276	0.093844	0.039119
22.67535	22.64223	24.01299	21.64694	22.43525	0.146357	-0.02741	-0.02692	0.057805	-0.09405
40.96655	43.63644	69.24867	51.00374	62.24831	-0.05755	-0.2663	-0.17998	0.470392	0.04628
149.5652	94.02427	204.6458	107.7026	120.5687	-0.1195	0.221789	-0.45413	0.647975	-0.31853
33.6241	38.39418	47.30058	46.59288	46.13825	-0.26608	-0.32708	-0.15449	0.169066	0.146938
32.1672	34.52111	29.32626	35.97688	33.68184	-0.04447	-0.04217	0.059462	-0.17583	0.118702
64.62707	60.18875	194.7007	56.539	80.18442	-0.022	0.070184	-0.0044	1.423093	-0.09594
23.17036	21.9157	23.83695	21.91663	21.33373	-0.15816	0.05855	-0.02107	0.101195	-0.01986
510.1559	575.3333	530.0053	546.6981	556.0896	0.151151	-0.12552	0.051716	-0.06737	-0.02377
160.3407	155.8956	178.621	191.0856	161.022	-0.0472	-0.049	-0.08907	0.105956	0.204104
102.7278	109.1909	78.66692	79.44292	83.32965	0.183112	0.103201	0.193104	-0.29484	-0.27039
95.42447	101.0886	78.62829	83.77518	83.27009	0.157112	0.084657	0.161263	-0.19351	-0.10308
110.0665	100.4167	90.38175	89.22698	88.55746	0.258528	0.2074	0.077416	-0.06949	-0.09113
83.1026	52.44405	58.70201	58.24309	46.90412	0.491566	0.466928	-0.18338	-0.00822	-0.0101
22.26094	25.54769	22.37169	22.60469	21.83276	0.168293	-0.05494	0.140083	-0.04673	-0.0341
357.2731	331.128	240.9593	217.3924	220.601	0.21582	0.315859	0.205243	-0.29791	-0.39819
86.11404	82.66269	105.0522	109.9177	103.2892	-0.01913	-0.13454	-0.19744	0.148579	0.214871
68.2365	75.45026	53.52475	47.58475	55.30818	0.611396	0.179938	0.354242	-0.14904	-0.32305
23.84706	22.75354	20.48899	19.67127	20.64529	0.111978	0.097411	0.030886	-0.1227	-0.18399
1978.448	1977.66	1249.816	1564.278	1454.615	0.116815	0.268465	0.272649	-0.41255	-0.06564
42.40776	39.17438	36.62534	38.45571	42.67544	0.165572	0.011418	-0.10334	-0.21207	-0.13141
41.4426	34.48088	38.68286	36.89586	34.52864	-0.14733	0.21384	-0.05007	0.11574	0.042851
672.2861	626.5181	908.6843	753.7681	751.6584	-0.16157	-0.02986	-0.13102	0.400335	0.113325
20.5075	21.35912	18.29786	18.08507	19.32934	0.050834	0.075861	0.136173	-0.09059	-0.10516
29.99646	36.1877	32.60053	32.18626	34.78289	-0.04805	-0.14086	0.131152	-0.01735	-0.03498
115.6181	108.1736	143.5174	125.337	114.949	-0.1694	-0.00171	-0.10067	0.296274	0.111346
128.6408	117.9234	91.72976	115.6847	90.6746	0.042218	0.217563	0.102436	-0.27882	0.059661
140.458	123.9511	149.8216	149.25	149.7597	-0.35753	0.064555	-0.1133	0.143141	0.152839
62.58307	59.44338	64.21848	66.93613	61.31025	-0.18232	0.018906	-0.05483	0.056149	0.11677
17.84234	16.18242	18.54068	20.87045	21.04969	-0.24555	-0.04719	-0.18417	0.011385	0.178913
45.98256	61.4991	52.52564	49.487	51.73382	0.111401	-0.17794	0.240381	0.012638	-0.06864

41.66028	46.04596	39.64001	34.21816	36.61717	0.187195	0.029826	0.170011	-0.05033	-0.26048
33.40079	30.86316	37.09285	31.01026	34.37585	0.128002	0.018967	-0.09386	0.168021	-0.08705
18.59825	16.30689	17.70649	16.53909	13.98123	-0.04456	0.154776	-0.03669	0.076691	-0.01852
44.39145	46.18737	42.20416	49.47328	42.57448	0.167808	-0.06474	-0.00521	-0.13754	0.093174
23.23325	22.50527	23.20225	19.63076	23.14237	0.086581	0.012766	-0.03779	0.009209	-0.23086
44.28545	48.2639	50.91883	50.75248	52.81949	-0.06881	-0.17707	-0.05527	0.024983	0.020472
124.5952	119.8341	129.7532	118.6023	109.6384	-0.25184	0.055106	-0.00479	0.114216	-0.02031
42.43436	43.24771	50.28137	51.90667	50.90738	-0.16604	-0.085	-0.05503	0.159519	0.202046
39.71793	39.89529	44.49991	41.58064	51.58612	-0.01634	-0.02589	-0.0199	0.129156	0.033678
35.85239	36.67756	38.82365	33.71664	34.28621	0.222916	-0.02362	0.008961	0.090269	-0.11391
17.96401	22.13921	22.6897	17.81044	17.61826	0.097455	-0.1478	0.153892	0.181637	-0.16915
98.00238	87.27502	97.95398	101.3353	84.10033	0.189106	0.01211	-0.15949	0.009024	0.059525
43.44812	42.48921	38.25416	44.43409	37.13154	-0.0533	0.105072	0.072116	-0.07706	0.138464
23.06283	23.57937	28.92274	22.24224	23.51908	-0.03505	-0.06577	-0.03378	0.261975	-0.11842
24.73151	24.56399	17.28313	16.93153	14.03564	0.367882	0.312219	0.29518	-0.19033	-0.22913
28.41743	29.82664	28.76752	28.8002	27.60984	0.194516	-0.02067	0.048879	-0.00406	-0.00175
76.97575	74.48727	66.17162	58.48421	66.6625	-0.04933	0.204036	0.158138	-0.007	-0.19057
24.07891	21.27904	25.55635	20.80633	22.69522	-0.00624	0.086359	-0.09159	0.171724	-0.12527
28.8852	29.8518	26.24998	24.42126	27.11937	0.086609	0.057254	0.108127	-0.07659	-0.184
21.55944	18.39358	18.2489	18.6302	16.58267	0.027293	0.15838	-0.07203	-0.08662	-0.05804
22.68286	19.67649	18.63582	19.21764	19.72879	-0.12207	0.211877	0.006162	-0.07183	-0.02896
23.70227	27.39703	21.54717	23.82126	22.5642	0.241447	-0.06131	0.151065	-0.19712	-0.0502
22.1883	23.10045	23.66048	19.47363	21.12793	0.169975	-0.00906	0.049247	0.074526	-0.20168
34.74071	38.3641	40.88258	30.91257	33.90405	-0.0733	0.019751	0.155344	0.25464	-0.15027
69.20634	48.31171	41.24133	46.16281	40.53735	0.070601	0.673573	0.15362	-0.06937	0.057539
91.11957	98.13174	111.0717	112.4145	106.9532	-0.12254	-0.17824	-0.06521	0.112872	0.131287
80.78976	64.36923	172.1536	96.92834	121.6174	-0.42394	-0.06704	-0.40942	0.945837	0.201413
26.34027	34.70978	36.19462	34.97762	30.24774	-0.00664	-0.36707	0.055663	0.118725	0.062542
43.87346	35.70278	29.50694	30.41936	30.84999	0.157554	0.289001	-0.00788	-0.31625	-0.23975
51.61036	42.20335	41.52771	40.40952	40.4144	0.027534	0.284364	-0.00474	-0.03695	-0.06888
29.12892	26.75729	29.17285	28.15558	24.96968	-0.12879	0.093613	-0.02475	0.098628	0.046945
15.53547	16.46159	20.05015	19.09252	16.59963	-0.08446	-0.12348	-0.04653	0.242592	0.173314
76.99119	62.55958	61.13552	51.08435	57.13487	0.162741	0.208227	-0.08538	-0.12514	-0.383
47.7276	43.83992	49.88446	46.64855	51.0267	0.034389	-0.01013	-0.13295	0.052347	-0.04308
146.322	143.3367	109.9855	122.1667	130.2024	0.300797	0.088767	0.059919	-0.33587	-0.18631
49.78459	43.37825	33.27391	39.15928	38.62343	0.463244	0.209153	5.55E-05	-0.40851	-0.15734
104.9671	100.28	90.87152	122.1667	91.7949	-0.0478	0.066611	0.001334	-0.14201	0.275822
23.60342	22.65398	18.81015	22.11029	19.45725	0.18127	0.123163	0.072353	-0.19495	0.037145
33.11776	34.77256	29.79359	30.37102	32.72843	0.150593	2.24E-04	0.069067	-0.16013	-0.12586
83.58523	70.00526	114.5078	104.3956	93.09183	-0.14398	-0.05014	-0.29912	0.399907	0.265448
25.67148	26.7497	23.21487	23.65614	25.81397	0.096641	-0.0209	0.038572	-0.16745	-0.14168
54.5636	44.34926	36.75872	41.55673	39.51285	0.061377	0.297125	-0.00383	-0.27835	-0.09401
60.49974	69.96571	62.12791	76.46409	70.38198	-0.01758	-0.19874	0.013333	-0.16901	0.137702
490.603	369.63	380.0035	373.9646	429.3098	0.405167	0.258418	-0.14188	-0.1112	-0.13727
38.02072	32.60765	34.30688	35.73925	28.56942	0.087539	0.143746	-0.0762	-0.00182	0.053826
16.42919	16.27132	20.62532	16.59887	16.35104	-0.03545	-0.01097	-0.02816	0.318386	0.005574
28.47909	35.08916	27.44836	29.75497	29.2093	-0.02111	-0.03646	0.262515	-0.09239	0.025857

26.38608	19.66747	18.76681	20.8028	20.16001	0.132927	0.34246	-0.07503	-0.14197	0.004556
510.2565	460.642	406.1775	407.0428	433.6027	0.034348	0.188734	0.04304	-0.14365	-0.14258
31.96414	30.48435	19.84945	26.61887	22.31742	0.39055	0.239093	0.202309	-0.44149	0.018823
123.3828	118.9615	87.58384	83.4502	105.0504	0.049973	0.200261	0.156792	-0.3107	-0.35962
33.57419	43.16388	28.34677	30.14726	31.4838	0.093669	-0.00966	0.330423	-0.26904	-0.16499
22.83012	20.74737	22.54887	26.3188	22.26051	-0.03361	0.051084	-0.08863	0.028262	0.254313
31.03043	32.12777	29.0144	27.41271	29.46495	0.149727	0.04427	0.095622	-0.05247	-0.13303
16.7333	20.1912	20.92749	25.42304	20.81238	-0.18103	-0.27007	0.010331	0.0562	0.342442
121.2615	111.2309	187.6481	144.2131	152.0187	0.044003	-0.20419	-0.33468	0.415879	0.035732
37.76687	32.50448	41.0219	43.39505	35.48082	0.159386	-0.05921	-0.27101	0.05611	0.142871
301.1769	389.7797	273.794	286.5407	314.394	0.452731	-0.15517	0.213041	-0.34132	-0.24854
23.90617	20.91811	27.39938	20.91951	24.4735	0.023739	0.051135	-0.14158	0.246694	-0.14608
543.7873	497.6375	452.4776	446.3821	451.85	0.102975	0.17437	0.044834	-0.0948	-0.11456
608.1252	615.9653	737.428	685.427	695.5168	-0.11385	-0.11938	-0.09831	0.161301	0.054839
135.1356	140.478	132.7007	135.3598	159.9987	-0.15323	-0.05254	0.004383	-0.08262	-0.05048
17.491	13.51631	20.48432	16.97581	20.27557	-0.18857	-0.05203	-0.42054	0.168869	-0.10538
88.7127	83.08041	80.81336	93.40852	84.96861	-0.0041	0.049822	-0.0448	-0.08512	0.12444
207.014	225.271	177.9889	192.2105	183.994	0.045697	0.118949	0.23247	-0.10082	0.011256
78.13921	58.58345	55.52603	65.51649	53.10889	0.038158	0.335735	-0.08432	-0.15923	0.081899
1306.772	879.5638	1535.385	1448.544	1140.82	0.355033	-0.00661	-0.59091	0.206405	0.144941
466.6633	480.6761	407.0176	411.8458	350.0699	0.130809	0.086846	0.119304	-0.11688	-0.10256
69.07883	61.81022	54.3846	62.12037	53.12544	0.16529	0.144494	-0.02314	-0.20631	-0.00577
479.1065	540.8394	425.6344	428.1823	397.9919	0.004654	0.101982	0.277541	-0.0677	-0.05782
351.3936	305.5382	371.6289	346.8837	333.2742	0.011271	0.035719	-0.16577	0.111696	0.017865
542.7717	530.2108	421.1342	487.8359	434.4289	0.024268	0.136804	0.103961	-0.23128	-0.01694
1664.818	1586.659	1209.114	1297.632	1305.264	0.176123	0.229376	0.155391	-0.24999	-0.13605
175.8894	169.7259	175.435	177.0309	185.2128	-0.13217	0.042401	-0.00852	0.037307	0.048666
2670.235	2745.365	2346.515	2290.649	2167.79	-0.15623	0.135225	0.173612	-0.0584	-0.08819
498.3285	521.7094	573.5638	542.752	524.7929	-0.19438	-0.05882	0.007784	0.141164	0.062045
38.04787	44.88048	34.60807	39.15841	36.63391	0.090911	-0.02333	0.216449	-0.1602	0.019154
118.6991	119.5919	139.0062	127.1888	121.8725	-0.10371	-0.03212	-0.02141	0.193616	0.065084
1708.23	2016.105	1558.511	1502.004	1503.121	0.16434	0.017078	0.256145	-0.11683	-0.17292
495.0284	499.7406	396.234	384.2441	395.2495	0.285809	0.150256	0.1591	-0.17589	-0.21459
331.7267	390.2313	283.471	329.6558	337.3333	0.018212	-0.004	0.231133	-0.24056	-0.01226
91.55963	122.7986	107.0731	98.6741	96.3193	-0.00404	-0.1917	0.237525	0.038452	-0.07806
23.84466	18.48444	31.0066	18.41288	23.75608	-0.12452	0.17073	-0.14962	0.589984	-0.17476
99.36926	114.6333	89.03194	94.7619	97.28436	0.114795	-9.91E-04	0.213546	-0.15576	-0.06114
43.57407	48.33194	42.86522	41.0734	40.7005	0.210514	-0.00397	0.150354	-0.02247	-0.0847
83.55386	98.816	95.52844	105.9565	103.7405	-0.12414	-0.22796	0.010194	-0.03472	0.113722
22.45642	20.70283	17.57784	16.78866	20.35648	0.059121	0.188529	0.070312	-0.16469	-0.23132
88.17453	73.35522	106.4182	79.27148	81.01037	-0.1186	0.077093	-0.1897	0.3476	-0.0809
63.23511	56.49476	73.8868	72.88982	68.50475	-0.21942	-0.07183	-0.2389	0.157718	0.125981
429.3413	356.5013	384.6178	361.9104	331.7007	0.002057	0.206432	-0.06026	0.050067	-0.03947
17.95146	21.77693	17.01947	20.39961	17.45001	0.0208	-0.01258	0.262225	-0.08926	0.16994
45.97023	44.23563	39.16526	42.85642	34.79989	-0.00455	0.133959	0.086174	-0.09069	0.029492
18.58524	19.41715	19.85397	19.8541	17.49781	-0.21946	-0.0152	0.047659	0.078963	0.079647
340.6905	290.693	261.937	285.1753	256.3672	0.019957	0.322163	0.089836	-0.05732	0.059772

172.8295	169.3951	214.8516	191.0533	193.556	-0.00363	-0.10292	-0.13508	0.212326	0.035493
70.09982	95.09546	114.0282	112.6706	101.1744	-0.22056	-0.46576	-0.08337	0.242314	0.225857
200.0076	198.8076	212.628	301.6399	259.6428	-0.18232	-0.11932	-0.12046	-0.02334	0.474394
67.40306	73.3149	90.05646	78.63326	91.69916	-0.22082	-0.1378	0.003066	0.287456	0.094406
21.91889	22.39063	19.04296	20.82455	23.43524	0.06369	0.009457	0.040769	-0.19254	-0.06572
27.41569	25.11302	29.9715	23.97116	27.38534	-0.1835	0.084137	-0.04092	0.216812	-0.10563
155.8621	160.6097	225.4014	193.0352	216.6965	-0.17266	-0.18139	-0.14184	0.349928	0.125929
21.33554	17.25374	17.57849	16.25704	17.02098	-0.08326	0.296364	-0.00971	0.020572	-0.09452
26.55266	28.35304	24.99403	23.35313	23.37032	0.112177	0.073003	0.170698	-0.0087	-0.1085
37.0104	42.88631	39.16034	36.66031	38.86198	0.046946	-0.08722	0.128127	-0.00226	-0.10038
19.97571	21.06879	18.74539	19.812	17.23141	0.135111	0.029871	0.104134	-0.0624	0.017224
19.86736	23.68005	22.37453	21.87758	23.07258	0.132474	-0.1841	0.068262	-0.01416	-0.04535
82.03473	79.63513	100.5211	81.90116	89.58447	-0.1236	-0.03267	-0.07393	0.262725	-0.03781
143.1512	93.10928	170.8933	176.5865	129.1357	-0.0425	-0.07499	-0.71537	0.130369	0.237805
93.07101	69.9482	56.40882	75.29437	71.13737	0.099232	0.329811	-0.09029	-0.40248	0.03097
18.32009	22.05975	22.72863	19.72478	20.63284	-0.06031	-0.14175	0.125409	0.169954	-0.03232
28.99254	27.1063	18.58301	22.34107	22.96784	0.325237	0.179969	0.095949	-0.44653	-0.18735
18.14591	23.06499	17.1084	16.92148	17.49312	0.098309	0.010657	0.342931	-0.0828	-0.09109
19.97975	18.1793	17.25112	17.6719	19.1506	0.239656	0.094471	-0.0384	-0.11621	-0.08041
24.46898	23.31795	22.24907	19.99653	20.86074	0.044052	0.148273	0.081329	0.013049	-0.1409
154.6211	137.9407	178.0266	166.4523	147.4566	0.011245	-0.04238	-0.21461	0.15836	0.061428
19.53631	17.02954	16.8031	15.73067	16.04735	0.17107	0.149446	-0.0488	-0.07083	-0.16162
66.72669	63.02353	50.75861	58.92649	55.0877	0.124242	0.121809	0.039409	-0.27153	-0.0658
18.91246	22.89884	20.57	20.43043	20.73779	0.063921	-0.15768	0.119527	-0.03568	-0.04492
17.76945	18.27801	19.51512	17.25164	17.49675	0.1924	-0.05482	-0.01389	0.081824	-0.09543
21.75744	23.11862	24.24186	28.72846	24.47296	-0.00688	-0.12638	-0.04126	0.028387	0.274264
38.31809	36.79541	39.45039	37.71965	32.73841	-0.06507	0.044672	-0.01362	0.083446	0.022025
28.33299	30.31355	28.91999	25.54224	25.89713	0.156647	2.64E-04	0.096561	0.029872	-0.15097
49.61381	51.24924	46.35864	40.74516	47.51033	-0.09165	0.086222	0.134322	-0.0098	-0.20391
318.5512	315.2619	383.4801	358.9407	383.2502	-0.15783	-0.06369	-0.07881	0.197296	0.106839
37.90035	32.14821	38.94834	33.46457	34.38001	-0.10411	0.134751	-0.09937	0.173328	-0.04419
697.1346	768.1582	646.1328	644.9937	626.5327	-0.0321	0.083301	0.223307	-0.02535	-0.02849
1220.791	1120.708	1107.582	1020.381	1020.246	0.115408	0.140948	0.016275	1.38E-04	-0.12641
534.7942	501.4375	365.9157	442.6047	397.7959	0.055404	0.259512	0.164039	-0.2924	-0.01969
318.1547	363.6961	185.4211	295.1263	285.2229	-0.01738	0.141181	0.330797	-0.62403	0.064298
1638.287	1375.086	1293.811	1210.032	1438.222	0.162892	0.239581	-0.01239	-0.1016	-0.20408
1228.406	1137.381	1036.466	1181.957	1309.438	-0.09148	0.112611	0.00238	-0.13584	0.05534
99.67529	114.0728	136.8173	127.7657	124.942	-0.19908	-0.25831	-0.06093	0.202644	0.105562
664.0772	609.4615	522.2884	632.003	583.7632	-0.08526	0.15058	0.024776	-0.20119	0.07848
80.5812	64.92646	144.8443	88.73628	96.30669	-0.23497	0.007512	-0.30135	0.776014	0.104347
1512.593	1439.811	1423.795	1205.68	1451.103	0.200825	0.047667	-0.02562	-0.04019	-0.29803
680.335	642.8872	760.8921	739.0422	704.6367	-0.24373	0.036929	-0.04354	0.189343	0.148949
342.5439	353.7268	405.4797	391.3021	424.7937	-0.11214	-0.12835	-0.07439	0.122169	0.070533
70.55017	74.43505	56.98003	62.01189	77.86646	0.11169	0.054406	0.127314	-0.25014	-0.12963
236.0555	156.592	123.0859	116.1479	108.7007	0.753532	0.768265	0.078896	-0.18271	-0.25717
136.7455	114.1462	165.3511	142.1894	148.2698	-0.04404	-9.09E-04	-0.26164	0.270454	0.054304
144.2592	148.465	184.7084	152.5436	182.2739	-0.18497	-0.17627	-0.13375	0.179694	-0.10145

283.7378	275.8689	254.0236	250.1599	297.2534	0.017658	0.036207	-0.00407	-0.12439	-0.14681
159.414	169.1945	138.7164	161.3847	181.9098	-0.17382	-0.02023	0.068749	-0.22155	-0.00595
389.7903	407.2418	506.6914	411.3302	413.0583	-0.16407	-0.0513	0.013454	0.323603	0.018415
119.3576	114.5971	63.68846	92.57804	97.32321	0.113406	0.300694	0.227769	-0.70107	-0.06023
177.3669	202.2225	268.0946	248.265	248.8092	-0.22124	-0.32684	-0.13923	0.268687	0.156475
279.2962	279.1553	317.2304	336.6558	314.6794	-0.1661	-0.03139	-0.03246	0.150605	0.236264
380.5824	424.625	431.2078	422.8972	409.9388	-0.17163	-0.09801	0.059529	0.081773	0.054624
338.9078	224.8116	492.6045	347.1234	332.7566	-0.05255	0.061464	-0.5282	0.593326	0.080795
94.12701	91.86497	153.8881	124.8737	141.9059	-0.13572	-0.18049	-0.21425	0.48787	0.20648
90.97875	91.30048	148.5756	94.14151	116.4378	-0.13026	-0.10736	-0.10459	0.554459	-0.05631
386.856	417.7115	460.1071	461.3971	473.5822	-0.09796	-0.13295	-0.02177	0.107168	0.121747
101.156	115.8893	120.2711	110.7114	121.6229	-0.08524	-0.1732	0.021757	0.07531	-0.0466
63.18225	71.5598	82.83203	72.56551	63.7785	-0.01237	-0.17349	0.004064	0.216639	0.024608
45.04113	34.49861	35.54879	34.71563	38.4206	0.072655	0.257099	-0.12941	-0.08928	-0.12208
48.09494	47.85544	50.4356	60.55689	48.28402	-0.2913	-0.07749	-0.08118	-0.00421	0.253733
57.6201	58.56763	50.6469	46.34386	48.63725	0.246537	0.097065	0.127905	-0.08551	-0.21446
202.7996	204.419	256.6147	241.7658	281.6887	-0.16295	-0.22852	-0.21526	0.117751	0.024944
413.922	426.6154	314.1748	274.6535	289.3029	0.183421	0.07219	0.112964	-0.34798	-0.53263
266.4585	246.1956	287.7819	248.143	270.0232	0.001625	-9.68E-04	-0.11643	0.111985	-0.10337
441.1532	400.1429	514.7766	405.6962	444.3634	-0.17782	0.045556	-0.09297	0.269968	-0.08153
239.3569	225.5779	287.6686	254.5467	282.9703	-0.19637	-0.01569	-0.10117	0.241609	0.060635
189.7356	173.5732	137.3933	168.011	158.9922	-0.02379	0.178868	0.045588	-0.2869	5.80E-04
51.14741	58.87616	74.8336	68.27732	74.28828	-0.17152	-0.26666	-0.07225	0.259233	0.149154
455.9033	423.14	515.9263	499.1981	478.8833	-0.11911	-0.03573	-0.1463	0.142528	0.0946
641.9022	607.6481	696.8877	621.3699	740.3159	-0.15544	0.034628	-0.04269	0.149514	-0.01249
227.3808	258.0266	220.3398	285.437	242.0605	-0.03402	-0.09952	0.084532	-0.14536	0.22877
221.5697	240.5648	264.1603	269.7116	245.7709	-0.09141	-0.14014	-0.02136	0.10761	0.14373
107.4257	88.96679	82.65028	101.4302	102.895	0.08193	0.098171	-0.16692	-0.28216	0.01986
160.3084	141.0675	127.301	118.7553	121.1211	0.336143	0.276793	0.103474	-0.04085	-0.14663
673.0087	717.29	683.5175	809.1335	748.0071	-0.20495	-0.1032	-0.01109	-0.08208	0.160719
1191.281	1412.244	1103.934	1051.614	981.5515	0.356096	-0.00475	0.248842	-0.10205	-0.18001
48.11628	47.35074	45.48719	49.97458	52.25095	-0.35737	0.018606	0.003113	-0.05495	0.080064
164.7923	170.4063	204.4302	189.9501	189.131	-0.13942	-0.15095	-0.0994	0.160548	0.05865
117.8558	107.1222	89.03717	100.8342	109.6311	0.112271	0.102631	-0.03367	-0.30404	-0.12213
158.534	139.3109	108.735	107.0457	98.74706	0.513283	0.24959	0.064879	-0.3022	-0.33215
122.3181	120.8088	170.6314	197.5361	190.2524	-0.3362	-0.33907	-0.3704	0.144255	0.342512
77.24701	69.33698	82.8975	76.95651	77.17039	-0.16707	0.01619	-0.14073	0.119856	0.006072
1291.03	1212.536	1420.435	1458.965	1594.772	-0.18214	-0.063	-0.15101	0.074589	0.114009
176.5981	219.741	217.3296	151.1368	172.862	0.141871	-0.03025	0.285323	0.254464	-0.2633
34.08538	39.25049	33.13925	34.92547	34.74701	0.031174	-0.04813	0.155869	-0.08972	-0.01271
392.9445	419.5336	302.4637	313.448	285.6334	-0.0266	0.135411	0.229226	-0.25583	-0.20478
36.17656	42.18469	30.8909	37.6642	34.42419	0.059699	-0.05235	0.170674	-0.27758	0.013281
113.1848	69.63367	98.47318	100.6631	77.89066	0.21012	0.369373	-0.31484	0.160902	0.206158
321.7663	303.9539	789.2186	589.9023	541.5599	-0.85916	-0.23064	-0.29031	1.010517	0.380503
63.44402	56.99904	77.6331	61.89555	66.46744	-0.14844	-0.03649	-0.18189	0.264333	-0.07555
52.48888	52.76976	41.64449	43.24918	45.55933	-0.04133	0.128397	0.137721	-0.20527	-0.15595
100.2972	98.2875	110.5574	111.2534	103.8448	-0.00581	-0.05802	-0.08599	0.083213	0.089829

1286.977	1540.029	1182.566	980.3528	890.7479	0.164659	-0.00402	0.251647	-0.16485	-0.44139
38.64156	33.28526	32.061	33.40413	31.12526	0.035949	0.208332	-0.00292	-0.06174	5.61E-04
266.0038	268.0326	137.8267	143.1619	142.7428	0.348443	0.52493	0.530724	-0.44587	-0.36635
90.08212	87.32452	67.9697	68.13184	72.52272	0.175356	0.207685	0.173896	-0.18947	-0.18413
12.84149	14.30381	33.5439	19.24043	22.17423	-0.20314	-0.29791	-0.1396	1.03236	0.235259
454.4668	426.322	511.188	543.5288	506.0981	-0.15267	-0.01467	-0.10656	0.150347	0.24514
213.839	229.211	165.9678	180.1074	159.4376	0.160555	0.136811	0.21368	-0.2488	-0.12049
326.208	324.6961	261.4863	332.8677	236.8087	0.304205	0.01102	0.003836	-0.36051	0.039087
42.20942	40.81253	55.07605	43.07539	41.19443	-0.09697	0.011816	-0.03384	0.398945	0.02241
124.3421	123.5667	186.206	135.4603	146.4755	-0.18181	-0.10258	-0.10252	0.490972	0.008973
106.7083	113.3366	82.85622	71.00633	74.10083	0.176151	0.218644	0.296446	-0.16119	-0.37176
80.19331	78.01293	93.34177	98.33743	86.95899	-0.03938	-0.06016	-0.10043	0.155292	0.22293
149.7786	139.8315	199.1084	157.8609	155.884	-0.23638	0.039638	-0.0716	0.446661	0.104503
33.46797	33.05545	37.65776	34.07423	38.51593	-0.00826	-0.03203	-0.04745	0.136625	-0.00436
55.09921	60.02828	72.64866	84.80708	83.7579	-0.16471	-0.31569	-0.19513	0.080352	0.305508
36.63035	34.32294	28.8051	34.24881	28.48248	0.001818	0.176948	0.087383	-0.16543	0.083248
210.6022	246.5459	189.5153	194.8722	173.3184	0.233553	-0.01262	0.211398	-0.1843	-0.15602
77.96232	60.89895	63.90818	65.93094	60.75871	0.018302	0.251238	-0.10637	-0.03815	0.010175
242.0123	320.0398	298.8832	357.6647	383.2521	-0.05452	-0.37838	0.020515	-0.07416	0.159973
46.94951	54.92885	57.44784	51.78639	56.72576	0.019125	-0.22693	0.002015	0.068701	-0.08266
26.62133	26.70395	24.2527	25.4533	27.11215	0.251109	0.008123	0.019753	-0.12501	-0.04922
92.46835	104.2619	83.17902	83.20462	78.26478	0.384474	0.002508	0.169962	-0.16195	-0.15757
124.4351	111.9512	77.39468	100.5336	103.2153	0.265747	0.220024	0.065222	-0.51016	-0.09689
29.99088	33.92415	30.2828	34.72569	33.38721	0.186646	-0.14546	0.032546	-0.13193	0.056908
80.8764	73.79544	65.55544	75.59991	69.72137	0.16173	0.113176	-0.01543	-0.19147	0.019394
72.98477	75.84621	60.87325	67.21345	63.13016	0.233349	0.033511	0.083753	-0.23587	-0.0866
1008.215	1474.718	744.5529	816.6805	724.3437	0.244116	0.134237	0.668352	-0.3169	-0.17529
88.28937	90.37787	84.50069	79.53042	84.45184	0.099947	0.01871	0.055611	-0.04133	-0.12801
20.06816	19.02415	17.37976	22.06751	21.10346	0.071724	-0.00567	-0.08563	-0.21396	0.127312
23.46468	20.78539	19.81683	23.78673	24.84587	0.076547	0.051615	-0.12451	-0.19365	0.063008
18.79233	18.82013	18.84087	20.15076	19.17601	0.228206	-0.03888	-0.03339	-0.03209	0.062264
22.80499	26.42006	22.74243	20.59348	21.95384	-0.01215	0.041686	0.25288	0.038201	-0.10352
94.89071	90.57267	83.72885	105.4624	99.4068	-0.01463	0.03053	-0.0369	-0.15088	0.175762
159.1607	169.1514	137.4877	116.9253	106.4992	0.358796	0.116249	0.204312	-0.09639	-0.34878
336.9497	268.9241	275.0382	280.2504	273.557	0.228113	0.229033	-0.09768	-0.06765	-0.04143
19.74841	22.52903	26.55914	24.43249	26.4044	0.033922	-0.28102	-0.08804	0.145338	0.027943
131.623	111.1854	180.6788	145.4154	140.3968	-0.02505	-0.05175	-0.29199	0.405662	0.091173
114.1695	134.8737	149.2147	123.5923	128.2934	-0.15912	-0.17407	0.066052	0.210845	-0.06437
66.64081	64.22062	105.6618	74.90451	88.36544	-0.26141	-0.07116	-0.11953	0.56651	0.078649
25.52841	28.04187	29.73418	32.34796	27.72952	-0.21189	-0.12644	0.008497	0.088069	0.214441
63.10945	58.25868	37.44997	67.11277	55.09804	0.144794	0.151968	0.035791	-0.66553	0.227236
38.79802	43.67333	34.1102	46.24232	35.81726	-0.05641	0.004875	0.170333	-0.18096	0.245674
25.46512	24.23993	32.25714	25.76966	29.21251	-0.09246	-0.02209	-0.09087	0.301624	-0.00353
81.201	101.31	75.18733	63.27568	65.33317	0.086269	0.009821	0.327552	-0.10676	-0.37268
70.37256	82.14636	53.75563	64.86912	70.44373	0.048632	0.015194	0.226088	-0.40224	-0.1057
165.1028	190.006	103.7895	93.89803	114.2793	0.226377	0.351839	0.556579	-0.31254	-0.47427
93.11626	97.26608	114.6271	125.5382	111.1185	-0.02268	-0.15035	-0.08472	0.142767	0.277309

135.4821	164.961	161.0771	156.162	141.0532	-0.15247	-0.11691	0.168157	0.128638	0.08652
86.09415	81.10464	58.20684	55.61654	62.50813	0.253739	0.247298	0.156376	-0.33767	-0.39916
17.68482	21.04661	20.84924	21.45196	22.26267	-0.03802	-0.22288	0.028414	0.013144	0.05185
25.33261	22.98606	22.85345	22.49935	22.50623	0.017239	0.125857	-0.0135	-0.02221	-0.0439
19.78853	18.70468	17.88584	17.49272	17.85019	0.204751	0.101441	0.02342	-0.04369	-0.0729
21.40314	22.35932	18.73174	18.57469	19.96842	-0.01228	0.051245	0.114013	-0.14187	-0.15774
18.44563	18.02797	20.67032	22.02493	18.3408	-0.03177	-0.01704	-0.04854	0.140701	0.239821
21.74927	24.38296	21.1187	23.40334	21.67332	0.161118	-0.08122	0.083513	-0.12532	0.025744
38.07481	37.96766	58.80924	56.65001	49.83042	-0.3143	-0.24758	-0.26977	0.372379	0.284906
72.98438	74.63044	66.79496	71.84931	69.7562	-0.05043	0.040124	0.072304	-0.08795	0.016609
38.81001	37.95197	33.2772	32.78527	33.04774	0.129362	0.103409	0.072054	-0.12237	-0.13829
201.2238	209.8519	242.5978	221.3536	237.7966	-0.18703	-0.09278	-0.0322	0.170671	0.044162
91.86306	98.61143	122.6647	105.6627	97.16326	-0.11919	-0.08357	0.016914	0.329976	0.102873
93.21323	82.42225	63.82871	79.23943	75.63898	0.166373	0.208416	0.038123	-0.33036	-0.01394
34.20679	33.21004	31.02228	34.17852	32.13485	0.198991	0.071734	0.031615	-0.06645	0.071712
23.68738	27.07048	23.88877	22.86346	25.94383	0.226489	-0.05339	0.140198	-0.04197	-0.10351
28.32501	25.42974	23.87508	26.01913	24.34027	0.225638	0.109806	-0.04583	-0.13876	-0.01523
97.48959	50.58548	101.5769	105.4525	72.16995	0.246875	-0.15747	-1.02634	-0.12261	0.035643
151.1927	117.5977	292.0973	208.1606	195.8044	-0.71693	0.042772	-0.38097	0.879586	0.378045
28.99573	30.65892	27.62466	25.10417	27.88516	0.107612	0.034616	0.114477	-0.03653	-0.17191
18.17791	19.23532	21.79749	21.43625	19.52873	0.016861	-0.1367	-0.05833	0.124767	0.096978
24.03606	23.45773	25.87657	21.52164	24.66825	0.064147	-0.00423	-0.04031	0.100376	-0.16456
47.98927	36.24741	38.58222	42.67989	35.93461	0.003381	0.26536	-0.13969	-0.04916	0.093995
19.38603	16.83153	24.36363	19.49737	18.56845	-0.14763	0.06154	-0.1407	0.384751	0.061314
117.766	112.7056	133.5764	120.2619	123.1107	-0.16564	2.23E-04	-0.06212	0.180711	0.02394
33.83992	31.68161	33.94134	29.31682	28.48488	0.020729	0.121477	0.028186	0.12399	-0.08404
79.87746	77.4923	109.5337	87.80188	85.39597	-0.13021	-0.0741	-0.13539	0.37774	0.057665
24.9268	20.86977	34.30962	29.86594	26.54149	-0.07049	-0.02032	-0.26849	0.423127	0.249247
26.41058	28.85041	24.679	31.99944	26.18366	0.088939	-0.05151	0.072807	-0.1491	0.216139
162.8968	137.9389	108.1077	105.8695	111.3657	0.168467	0.451333	0.199129	-0.17428	-0.16795
113.9843	94.63557	116.7776	133.6149	108.8393	-0.08354	0.046261	-0.22347	0.08521	0.278426
16.29025	15.41619	22.59281	17.37079	19.63583	-0.0727	-0.00805	-0.0855	0.452357	0.074397
76.47125	79.00656	78.7466	81.59382	80.58746	0.312974	-0.06281	-0.00947	-0.02024	0.033537
2018.973	2186.616	2141.724	2329.919	2218.075	-0.16558	-0.04278	0.071291	0.04088	0.159752
287.0959	249.4384	243.6697	215.6028	220.5223	0.323575	0.159457	-0.0405	-0.09299	-0.26875
54.60666	54.15896	63.06012	68.31482	49.43248	-0.10255	0.035689	0.021292	0.238053	0.332069
27.2225	27.43324	30.93981	26.34171	27.45252	-0.20678	-0.01867	-0.00626	0.167455	-0.06901
68.80328	52.31647	65.28458	65.29355	60.38403	0.113693	0.08497	-0.30897	0.010568	0.010209
78.81056	65.00391	143.1149	94.62412	106.5154	-0.56741	0.02228	-0.27571	0.813563	0.217241
62.56435	57.11823	55.83977	54.77174	57.22424	0.161838	0.11559	-0.011	-0.04387	-0.0727
600.0703	617.8785	790.3814	713.4204	692.0192	-0.26008	-0.08829	-0.04699	0.303557	0.148411
20.87128	20.66587	20.88031	18.78807	19.10901	0.147286	0.025123	0.011207	0.026557	-0.12572
1821.978	1651.934	1413.638	1735.02	1416.361	0.031972	0.17	0.024633	-0.20996	0.097135
236.4312	281.0092	201.9293	217.4922	193.2306	0.12578	0.052275	0.283141	-0.1777	-0.07513
145.8735	122.8335	144.94	127.0845	110.9465	-0.00279	0.227031	-0.02416	0.203829	0.027738
481.0835	440.1953	665.5685	583.6762	572.2447	-0.09201	-0.10017	-0.22986	0.358337	0.168959
264.0576	223.1728	198.7872	211.7989	217.0865	0.262859	0.164141	-0.08675	-0.25211	-0.15225

20.9332	22.2859	13.27322	11.89424	13.35687	0.295423	0.216454	0.311313	-0.45032	-0.58187

[D30-TAA](	Gene Symb	Gene Title	Pathway	Gene Onto	Gene Onto	Gene Ontology Molecular Function
0.135517	Rab14	RAB14, member RAS (		0006810 /,	0005622 /,	0000166 // nucleotide binding // inferred fro
0.150408	Ctbp1	C-terminal binding pro		0000122 /,	0005634 /,	0003700 // sequence-specific DNA binding tr
0.009145	Gbf1	golgi-specific brefeldin		0032012 /,	0005622 /,	0005086 // ARF guanyl-nucleotide exchange
0.138667	Zranb1	zinc finger, RAN-bindi		0008152 /,	0005622 /,	0004221 // ubiquitin thiolesterase activity //
0.09539	Ube2v1	ubiquitin-conjugating		0043687 /,	0005634 /,	0005515 // protein binding // inferred from
-0.35562	Ncl	nucleolin	mRNA_pro	0001525 /,	0005634 /,	0000166 // nucleotide binding // inferred fro
-0.01249	Rbbp7	retinoblastoma bindir		0000122 /,	0005634 /,	0016564 // transcription repressor activity /,
-0.01225	Pnliprp1	pancreatic lipase relat		0006629 /,	0005576 /,	0003824 // catalytic activity // inferred from
0.201681	Atp6v1b2	ATPase, H+ transporti		0006200 /,	0005794 /,	0005524 // ATP binding // inferred from elec
-0.17238	Scd2	stearoyl-Co	Fatty_Acid	0006629 /,	0005783 /,	0004768 // stearoyl-CoA 9-desaturase activit
-0.20608	Kitl	kit ligand		0001541 /,	0005576 /,	0005125 // cytokine activity // inferred from
-0.1217	Kitl	kit ligand		0001541 /,	0005576 /,	0005125 // cytokine activity // inferred from
-0.17261	Ghitm	growth hormone inducible transı		0005739 // mitochondrion // inferred from direct assay		
0.004036	Mthfd1	methylenetetrahydro		0000105 /,	0005737 /,	0000166 // nucleotide binding // inferred fro
0.205185	Cstf2t	cleavage st	mRNA_pro	0006378 /,	0005622 /,	0000166 // nucleotide binding // inferred fro
0.008042	Nup62	nucleoporin 62		0006810 /,	0000922 /,	0005515 // protein binding // inferred from
-0.07289	Igf2	insulin-like growth fac		0001503 /,	0005576 /,	0005158 // insulin receptor binding // inferre
-0.06125	Smoc2	SPARC related modula		0007165 /,	0005576 /,	0005509 // calcium ion binding // inferred fr
-0.12014	Cox6c	cytochrome c oxidase		0008152 /,	0005739 /,	0004129 // cytochrome-c oxidase activity //
-0.07467	Carhsp1	calcium regulated hea		0006355 /,	0005737 /,	0003676 // nucleic acid binding // inferred fr
-0.28219	Vcam1	vascular cell adhesion		0001666 /,	0005615 /,	0005178 // integrin binding // not recorded
0.01522	Klhdc3	kelch domain contain		0007126 /,	0000790 /,	0003682 // chromatin binding // inferred fro
-0.04827	Psmc1	protease (prosome, m		0030163 /,	0000502 /,	0000166 // nucleotide binding // inferred fro
-0.30514	Satb1	special AT-rich sequer		0000122 /,	0000785 /,	0003677 // DNA binding // inferred from dir
0.121363	Cct3	chaperonin containing		0006457 /,	0005737 /,	0000166 // nucleotide binding // inferred fro
-0.28658	Tbc1d15	TBC1 domain family, r		0032313 /,	0005576 /,	0005096 // GTPase activator activity // infer
0.159847	Nup85	nucleoporin 85		0000226 /,	0000775 /,	0005515 // protein binding // inferred from
-0.50429	Adm	adrenome	Smooth_m	0001666 /,	0005576 /,	0005179 // hormone activity // inferred fron
-0.34858	Zfand5	zinc finger, AN1-type (		0001701 // in utero e		0003677 // DNA binding // inferred from ele
0.265912	Nsg2	neuron specific gene f		0007212 /,	0005768 /,	0050780 // dopamine receptor binding // int
0.265502	Lox	lysyl oxidase		0001568 /,	0005576 /,	0004720 // protein-lysine 6-oxidase activity ,
-0.37563	Efr3a	EFR3 homolog A (S. ce		0016337 /,	0001533 /,	0005488 // binding // inferred from electror
-0.1735	Nr2f2	nuclear rec	Nuclear_R	0000122 /,	0005634 /,	0001972 // retinoic acid binding // inferred f

0.082262	Rbmxrt	RNA bindir	mRNA_prc	0006397 /,	0005634 /,	0000166 // nucleotide binding // inferred frc
-0.06115	Tmem19	transmembrane protein 19			0016020 // membrane // inferred from electronic anno	
0.082144	Gart	phosphoribosylglycina		0006164 /,	0005737 /,	0000166 // nucleotide binding // inferred frc
0.006426	Ap1m1	adaptor-related prote		0006810 /,	0005794 /,	0005515 // protein binding // inferred from
0.019286	Ugdh	UDP-glucose dehydro,		0001702 /,	0005829 /,	0003824 // catalytic activity // inferred from
0.121466	Abhd4	abhydrolase domain c		0016042 // lipid catab		0016787 // hydrolase activity // inferred fron
-0.29849	Lamp2	lysosomal-associated membrane			0005624 // membrane fraction // not recorded /// 000!	
-0.25006	Lamp2	lysosomal-associated membrane			0005624 // membrane fraction // not recorded /// 000!	
-0.15756	Mcam	melanoma cell adhesi		0007155 /,	0005886 // plasma membrane // not recorded /// 0016	
0.186878	Abcb10	ATP-binding cassette,		0006200 /,	0005739 /,	0000166 // nucleotide binding // inferred frc
-0.30184	Tmem30a	transmeml	Electron_Transport_C		0016020 // membrane // inferred from electronic anno	
0.162217	Rpn1	ribophorin	Proteasom	0006486 /,	0005783 /,	0004579 // dolichyl-diphosphooligosacchari(
-0.20456	Ccng2	cyclin G2	G1_to_S_c	0007049 /,	0005634 // nucleus // inferred from electronic annotati	
-0.12961	Ccne1	cyclin E1	Cell_Cycle_	0001547 /,	0000307 /,	0005515 // protein binding // inferred from
-0.07852	Ppic	peptidylprolyl isomera		0006457 /,	0005737 /,	0003755 // peptidyl-prolyl cis-trans isomeras
-0.00113	Pdpk1	3-phospho	Integrin-m	0006468 /,	0005737 /,	0000166 // nucleotide binding // inferred frc
0.046163	Pnpla6	patatin-like phospholi		0001525 /,	0005624 /,	0004091 // carboxylesterase activity // infer
0.17966	Slc35b4	solute carrier family 3		0006810 /,	0000139 /,	0005462 // UDP-N-acetylglucosamine transn
0.030518	Slc35b4	solute carrier family 3		0006810 /,	0000139 /,	0005462 // UDP-N-acetylglucosamine transn
-0.0141	Acin1	apoptotic (	mRNA_prc	0006915 /,	0005634 /,	0000166 // nucleotide binding // inferred frc
-0.267	Slc1a5	solute carrier family 1		0003333 /,	0016020 /,	0005515 // protein binding // inferred from
0.185455	Coil	coilin			0001674 /,	0005515 // protein binding // inferred from
-4.48E-04	Tcf4	transcription factor 4		0000122 /,	0005634 /,	0003677 // DNA binding // inferred from dir
-0.26329	Col5a1	collagen, type V, alpha		0001568 /,	0005576 /,	0005198 // structural molecule activity // inf
0.00302	Ddx20	DEAD (Asp	mRNA_prc	0000122 /,	0005634 /,	0000166 // nucleotide binding // inferred frc
-0.28829	Wee1	WEE 1 hon	Cell_Cycle_	0006468 /,	0005634 /,	0000166 // nucleotide binding // inferred frc
-0.17063	Wee1	WEE 1 hon	Cell_Cycle_	0006468 /,	0005634 /,	0000166 // nucleotide binding // inferred frc
0.038388	Nxf1	nuclear RN	mRNA_prc	0006405 /,	0005622 /,	0000166 // nucleotide binding // inferred frc
-0.26016	Trpm7	transient receptor pot		0006468 /,	0001726 /,	0000166 // nucleotide binding // inferred frc
-0.19362	Nid1	nidogen 1		0007155 /,	0005576 /,	0005509 // calcium ion binding // inferred fr
-0.33136	Amd1	S-adenosylmethionine		0001701 // in utero e		0004014 // adenosylmethionine decarboxyla
0.349429	Gas1	growth arrest specific		0002053 /,	0005886 /,	0005515 // protein binding // inferred from
-0.27126	Adam8	a disintegrin and meta		0006508 /,	0005886 /,	0004222 // metalloendopeptidase activity //,
-0.43265	Cdk2	cyclin-depe	Cell_Cycle_	0006468 /,	0000307 /,	0000166 // nucleotide binding // inferred frc
-0.04684	Mcl1	myeloid ce	Apoptosis_	0006915 /,	0005634 /,	0005515 // protein binding // inferred from
-0.43471	Mcl1	myeloid ce	Apoptosis_	0006915 /,	0005634 /,	0005515 // protein binding // inferred from
-0.08017	Npc2	Niemann Pick type C2		0030301 /,	0005576 /,	0015485 // cholesterol binding // inferred fr
-0.04088	Kpnb1	karyopherin (importin		0006606 /,	0005634 /,	0005488 // binding // inferred from electron
-0.16966	Trp53inp1	transformation relate		0006915 /,	0005634 // nucleus // inferred from direct assay /// 000	
0.194372	Rbm12	RNA bindir	mRNA_processing_bi		0005634 /,	0000166 // nucleotide binding // inferred frc
-0.19923	Nr1d2	nuclear rec	Circadian_	0006350 /,	0005634 /,	0003677 // DNA binding // inferred from dir
-0.35313	Nr1d2	nuclear rec	Circadian_	0006350 /,	0005634 /,	0003677 // DNA binding // inferred from dir
-0.19146	Mfng	MFNG O-fucosylpepti		0007275 /,	0000139 /,	0016740 // transferase activity // inferred fr
0.173013	Cog4	component of oligom		0006810 /,	0000139 /,	0005515 // protein binding // inferred from
-0.05903	Vps4b	vacuolar protein sorti		0006200 /,	0005634 /,	0000166 // nucleotide binding // inferred frc
-0.09623	Rassf3	Ras association (RalGI		0007165 /,	0005737 /,	0005515 // protein binding // inferred from
0.02741	Slc7a3	solute carrier family 7		0003333 /,	0005886 /,	0000064 // L-ornithine transmembrane tran

0.135621	Ppp1r11	protein phosphatase 1, regulatory (inhibitor			0004864 // phosphoprotein phosphatase inh
0.00232	Armc10	armadillo repeat cont	0040008 //	0005739 //	0005488 // binding // inferred from electron
0.183655	Pafah1b1	platelet-ac Wnt_Signa	0000132 //	0000235 //	0005515 // protein binding // inferred from
-0.1601	Tinagl1	tubulointerstitial neph	0006508 //	0005576 //	0004197 // cysteine-type endopeptidase act
0.553119	Angptl4	angiopoietin-like 4	0001666 //	0005576 //	0004857 // enzyme inhibitor activity // infer
-0.14313	Lpar1	lysophosphatidic acid	0000187 //	0005737 //	0001619 // lysosphingolipid and lysophosph
0.178578	Pdgfrb	platelet derived grow	0001701 //	0005887 //	0000166 // nucleotide binding // inferred fro
0.020391	Mycn	v-myc myelocytomato	0001502 //	0005634 //	0003677 // DNA binding // inferred from ele
-0.19269	Dusp10	dual specificity phosp	0006470 //	0005634 //	0004721 // phosphoprotein phosphatase act
0.055464	Pcsk7	proprotein convertase	0006508 //	0005794 //	0004252 // serine-type endopeptidase activi
-0.08687	Sod2	superoxide dismutase	0000302 //	0005625 //	0003677 // DNA binding // not recorded ///
0.082919	Tmem183a	transmembrane protein 183A		0016020 // membrane // inferred from electronic anno	
0.097048	Tmem183a	transmembrane protein 183A		0016020 // membrane // inferred from electronic anno	
-0.05499	Cetn3	centrin 3	0007049 //	0005737 //	0005509 // calcium ion binding // inferred fr
0.099638	Rlim	ring finger protein, LIM	0006350 //	0005634 //	0004842 // ubiquitin-protein ligase activity /
-0.1222	Cel	carboxyl ester lipase	0006707 //	0005576 //	0004091 // carboxylesterase activity // not r
-0.65837	Ptgs2	prostaglan Eicosanoid	0001516 //	0005634 //	0004601 // peroxidase activity // inferred fro
0.180065	Lynx1	Ly6/neurotoxin 1	0007271 //	0005624 //	0005515 // protein binding // inferred from
-0.36185	Smpdl3b	sphingomyelin phospl	0006685 //	0005576 //	0004767 // sphingomyelin phosphodiesteras
0.179117	Mast2	microtubule associate	0006468 //	0005737 //	0000166 // nucleotide binding // inferred fro
-0.21492	Ppp1r2	protein phosphatase 1	0005975 // carbohydr		0004864 // phosphoprotein phosphatase inh
0.159936	Entpd5	ectonucleoside tripho	0008152 //	0005783 //	0016787 // hydrolase activity // inferred from
0.15645	Npepps	aminopeptidase puro	0006508 //	0005634 //	0004177 // aminopeptidase activity // not re
0.107674	Npepps	aminopeptidase puro	0006508 //	0005634 //	0004177 // aminopeptidase activity // not re
-0.21191	Fmo1	flavin containing mon	0006082 //	0005783 //	0004497 // monooxygenase activity // not re
0.004009	Fam151a	family with sequence simliarity 1		0016020 // membrane // inferred from electronic anno	
-0.15186	Slc12a4	solute carrier family 1	0006810 //	0016020 //	0005215 // transporter activity // inferred fr
-0.24308	Cul4b	cullin 4B	0006281 //	0005634 //	0005515 // protein binding // inferred from
0.41354	Tgfb3	transforming growth f	0000187 //	0005576 //	0005114 // type II transforming growth facto
-0.23401	Cap1	CAP, adenylate cyclase	0000902 //	0005576 //	0003779 // actin binding // inferred from ele
0.04858	2400001E0	RIKEN cDNA 2400001E08 gene		0005886 // plasma membrane // inferred from electron	
-0.20813	Tnnc2	troponin C Striated_m	0003009 //	0005861 //	0003779 // actin binding // inferred from ele
-0.11756	Myh9	myosin, heavy polype	0000212 //	0001725 //	0000146 // microfilament motor activity // r
-0.21823	Fosl1	fos-like ant Wnt_Signa	0006355 //	0005634 //	0003677 // DNA binding // not recorded ///
-0.07189	Spag6	sperm associated anti	0007283 //	0005737 //	0005488 // binding // inferred from electron
0.197385	Atad1	ATPase family, AAA do	0008152 //	0005739 //	0000166 // nucleotide binding // inferred fro
-0.12618	Fyn	Fyn proto- Integrin-m	0001764 //	0005624 //	0000166 // nucleotide binding // inferred fro
0.187333	Ncald	neurocalcin delta			0005509 // calcium ion binding // inferred fr
0.242199	Otub2	OTU domain, ubiquitin	0006519 // cellular an		0004843 // ubiquitin-specific protease activi
-0.0308	Nup210	nucleoporin 210	0006810 //	0005634 //	0005515 // protein binding // inferred from
0.155445	Mtor	mechanistic target of	0001938 //	0000139 //	0000166 // nucleotide binding // inferred fro
-0.13793	Rgs1	regulator c Calcium_re	0007186 //	0005737 //	0004871 // signal transducer activity // infer
-0.67058	Per2	period hon Circadian_	0006350 //	0005634 //	0004871 // signal transducer activity // infer
-0.03857	Itih2	inter-alpha trypsin inh	0010466 //	0005576 //	0004867 // serine-type endopeptidase inhib
-0.32514	Slc12a2	solute carrier family 1	0006810 //	0005624 //	0005215 // transporter activity // inferred fr
-0.25912	Lefty1	left right determinatio	0007275 //	0005576 //	0005125 // cytokine activity // inferred from
-0.17943	Sspn	sarcospan		0005886 // plasma membrane // inferred from electron	

-0.22135	Cdkn1c	cyclin-dep	G1_to_S_c	0000122 //	0005634 //	0004860 // protein kinase inhibitor activity /
-0.23514	Pvalb	parvalbumin			0005737 //	0005509 // calcium ion binding // inferred fr
0.11963	Srrt	serrate RNA effector r		0008283 //	0005634 //	0005515 // protein binding // inferred from
-0.05887	Pter	phosphotriesterase re		0009056 //	0005576 //	0005515 // protein binding // inferred from
0.143503	Rtn4ip1	reticulon 4 interacting		0008152 //	0005739 //	0003824 // catalytic activity // inferred from
0.050532	Diablo	diablo homolog (Dros		0006486 //	0000139 //	0005515 // protein binding // inferred from
0.055933	Drg2	developmentally regulated GTP b			0005622 //	0000166 // nucleotide binding // inferred fro
-0.21998	Soat1	sterol O-ac	Statin_Path	0006629 //	0005783 //	0000062 // acyl-CoA binding // inferred from
-0.25125	Soat1	sterol O-ac	Statin_Path	0006629 //	0005783 //	0000062 // acyl-CoA binding // inferred from
-0.41182	Laptm5	lysosomal-associated		0006810 //	0005764 // lysosome // not recorded /// 0005764 // lys	
0.363263	Ext1	exostoses (multiple) 1		0001503 //	0000139 //	0005515 // protein binding // inferred from
0.323101	Unc13b	unc-13 homolog B (C.		0006887 //	0005737 //	0001566 // non-kinase phorbol ester recepto
0.107491	Cyb5b	cytochrome b5 type B		0006810 //	0005739 //	0005215 // transporter activity // inferred fr
0.145306	Lass4	LAG1 homolog, ceram		0006355 //	0005634 //	0003677 // DNA binding // inferred from ele
-0.11182	Pla1a	phospholipase A1 me		0006629 //	0005576 //	0003824 // catalytic activity // inferred from
-0.0526	Dkkl1	dickkopf-like 1		0045600 //	0005576 //	0005515 // protein binding // inferred from
-0.40308	Ccl11	chemokine (C-C motif		0001938 //	0005576 //	0005125 // cytokine activity // inferred from
0.157368	Kcnb1 /// I	potassium	Calcium_re	0006810 //	0005737 //	0005216 // ion channel activity // inferred fr
-0.20456	Smc1a	structural	mRNA_pro	0000075 //	0000775 //	0000166 // nucleotide binding // inferred fro
0.41856	Spon2	spondin 2, extracellula		0007155 //	0005576 //	0005102 // receptor binding // not recorded
-0.34368	Ctsz	cathepsin Z		0006508 //	0005764 //	0004197 // cysteine-type endopeptidase act
0.054125	Hsd17b7	hydroxyste	Steroid_Bi	0006694 //	0005783 //	0000253 // 3-keto sterol reductase activity /
0.171733	Ube2l3	ubiquitin-conjugating		0000209 //	0005634 //	0000166 // nucleotide binding // inferred fro
0.226842	Ube2l3	ubiquitin-conjugating		0000209 //	0005634 //	0000166 // nucleotide binding // inferred fro
-0.08003	Ccl22	chemokine (C-C motif		0006935 //	0005576 //	0005125 // cytokine activity // inferred from
-0.40561	Cpeb1	cytoplasmi	mRNA_pro	0006397 //	0000932 //	0000166 // nucleotide binding // inferred fro
0.0479	Ap3d1	adaptor-related prote		0006810 //	0000139 //	0005488 // binding // inferred from electron
-0.2195	1190002H	RIKEN cDNA 1190002I		0001934 //	0005634 //	0005515 // protein binding // inferred from
-0.15487	Bhlhe40	basic helix-loop-helix		0006350 //	0005634 //	0003677 // DNA binding // inferred from dir
-0.138	Slc22a1	solute carrier family 2		0006810 //	0005886 //	0005215 // transporter activity // inferred fr
0.019231	Rbm8a	RNA bindir	mRNA_pro	0000184 //	0005634 //	0000166 // nucleotide binding // inferred fro
-0.17238	Dbp	D site albumin promo		0006350 //	0005634 //	0003677 // DNA binding // inferred from ele
0.363819	Ina	internexin neuronal ir		0007275 //	0005882 //	0005198 // structural molecule activity // inf
-0.05167	Plekhg2	pleckstrin homology c		0035023 //	0005622 //	0005085 // guanyl-nucleotide exchange fact
-0.01435	Lims1	LIM and senescent ce		0007160 //	0005886 //	0005515 // protein binding // inferred from
-0.1165	Lims1	LIM and senescent ce		0007160 //	0005886 //	0005515 // protein binding // inferred from
-0.36504	Elf2	E74-like factor 2		0006350 //	0005634 //	0003677 // DNA binding // inferred from ele
-0.38773	Klf6	Kruppel-like factor 6		0006350 //	0005622 //	0003676 // nucleic acid binding // inferred fr
0.218125	Efnb1	ephrin B1		0001755 //	0016020 //	0005515 // protein binding // inferred from
0.287887	Efnb1	ephrin B1		0001755 //	0016020 //	0005515 // protein binding // inferred from
-0.17891	Ifit2	interferon-induced pr		0035457 // cellular re		0005488 // binding // inferred from electron
-0.1614	Rbfox1	RNA bindir	mRNA_pro	0006397 //	0005634 //	0000166 // nucleotide binding // inferred fro
-0.06615	Ufsp2	UFM1-specific peptida		0008152 // metabolic		0008233 // peptidase activity // inferred from
-0.26114	Slc7a5	solute carrier family 7		0003333 //	0005737 //	0015171 // amino acid transmembrane tran
0.071361	Afg3l1	AFG3(ATPa	mRNA_pro	0006508 //	0005739 //	0000166 // nucleotide binding // inferred fro
-0.34589	Gbp3	guanylate binding protein 3			0005737 //	0000166 // nucleotide binding // inferred fro
0.099924	Jrk	jerky		0045449 //	0000775 //	0003676 // nucleic acid binding // inferred fr

-0.02227	Psg23	pregnancy-specific gly		0007565 // female pregnancy // inferred from electronic annotatio		
-0.03375	Bcl6b	B-cell CLL/lymphoma		0000122 /,	0005622 /,	0003676 // nucleic acid binding // inferred fı
-0.21886	Stx7	syntaxin 7		0006886 /,	0005768 /,	0005484 // SNAP receptor activity // inferre
-0.58316	Col8a1	collagen, type VIII, alp		0001525 /,	0005576 // extracellular region // inferred from electro	
0.144557	Xpo1	exportin 1, CRM1 hon		0006611 /,	0000776 /,	0003723 // RNA binding // inferred from ele
-0.1734	Atp1b1	ATPase, Na	Calcium_re	0001666 /,	0005886 /,	0005391 // sodium:potassium-exchanging A
-0.08655	Yes1	Yamaguchi sarcoma v		0006468 /,	0005624 /,	0000166 // nucleotide binding // inferred fro
-0.10056	Vps54	vacuolar protein sorti		0006810 // transport		0005515 // protein binding // inferred from
-0.18736	Snca	synuclein, alpha		0001774 /,	0005624 /,	0000287 // magnesium ion binding // inferre
-0.21731	Dpt	dermatopontin		0007155 /,	0005576 // extracellular region // inferred from electro	
-0.1628	Nudcd2	NudC domain containing 2			0005622 // intracellular // inferred from electronic ann	
-0.12361	Dnaja4	DnaJ (Hsp40) homolog		0006457 /,	0016020 /,	0005524 // ATP binding // inferred from elec
0.233075	Avpr1a	arginine va	GPCRDB_C	0001992 /,	0005886 /,	0004871 // signal transducer activity // infer
0.238978	Avpr1a	arginine va	GPCRDB_C	0001992 /,	0005886 /,	0004871 // signal transducer activity // infer
-0.05589	Spata6	spermatogenesis asso		0007275 /,	0005576 // extracellular region // inferred from electro	
0.415939	Clock	circadian lo	Circadian_	0006350 /,	0005634 /,	0003677 // DNA binding // inferred from ele
0.218178	Clock	circadian lo	Circadian_	0006350 /,	0005634 /,	0003677 // DNA binding // inferred from ele
-0.00532	Hspg2	perlecan (heparan sul		0001525 /,	0005576 /,	0005515 // protein binding // inferred from
-0.43592	Inmt	indolethylamine N-me		0008152 /,	0005737 /,	0008168 // methyltransferase activity // infe
-0.05592	Cdc42ep5	CDC42 effector protei		0007254 /,	0005737 /,	0005515 // protein binding // inferred from
0.151035	Haus8	4HAUS augmin-like cc		0007049 /,	0000922 // spindle pole // inferred from electronic ann	
-0.13459	Twist1	twist homolog 1 (Dros		0000122 /,	0005634 /,	0003677 // DNA binding // inferred from ele
-0.00819	Krt34	keratin 34			0005882 /,	0005198 // structural molecule activity // inf
-0.03528	Cyth3	cytohesin 3		0032012 /,	0001726 /,	0005085 // guanyl-nucleotide exchange fact
-0.0091	Barhl1	BarH-like 1 (Drosophil		0001764 /,	0005634 /,	0003677 // DNA binding // inferred from ele
0.192591	Zfp385a	zinc finger	mRNA_processing_bi		0005622 /,	0003676 // nucleic acid binding // inferred fı
-0.04364	Sfxn4	sideroflexin 4		0006810 /,	0005739 /,	0008324 // cation transmembrane transport
-0.1994	Csnk1d	casein kinase 1, delta		0006468 /,	0005625 /,	0000166 // nucleotide binding // inferred fro
-0.37256	Cebpb	CCAAT/enh	Circadian_	0001892 /,	0005634 /,	0003677 // DNA binding // inferred from dir
0.383945	Nfil3	nuclear factor, interle		0006350 /,	0005634 /,	0003677 // DNA binding // inferred from ele
0.386896	Dio2	deiodinase, iodothyro		0006590 /,	0016020 /,	0004800 // thyroxine 5'-deiodinase activity /
0.107534	Ift74	intraflagellar transport 74 homol			0016023 // cytoplasmic membrane-bounded vesicle //	
0.195364	Camkk1	calcium/calmodulin-d		0006468 /,	0005625 /,	0000166 // nucleotide binding // inferred fro
-0.18376	Ms4a4d	membrane-spanning 4		0007165 /,	0016020 /,	0004872 // receptor activity // inferred from
-0.42813	Arpp21	cyclic AMP-regulated		0034605 /,	0005737 /,	0003676 // nucleic acid binding // inferred fı
-0.20398	Cyp2d22	cytochrome P450, fan		0010033 /,	0005737 /,	0004497 // monooxygenase activity // not re
-0.17958	Cyp2d22	cytochrome P450, fan		0010033 /,	0005737 /,	0004497 // monooxygenase activity // not re
0.058704	Pex14	peroxisomal biogenes		0006810 /,	0005777 /,	0003714 // transcription corepressor activity
-0.01755	Ptpn21	protein tyrosine phos		0006470 /,	0005737 /,	0004721 // phosphoprotein phosphatase ac
-0.13147	Cys1	cystin 1		0006810 /,	0005737 /,	0005215 // transporter activity // inferred fr
0.213405	Nin	ninein			0005737 /,	0000166 // nucleotide binding // inferred fro
0.157798	Scnn1g	sodium channel, nonv		0001666 /,	0005624 /,	0005216 // ion channel activity // inferred fr
-0.09813	Alg14	asparagine-linked glycosylation 1			0005783 // endoplasmic reticulum // inferred from elec	
-0.17053	Pdgfc	platelet-derived grow		0007171 /,	0005576 /,	0005161 // platelet-derived growth factor re
-0.08205	Mfsd6	major facilitator super		0007165 /,	0016020 /,	0004872 // receptor activity // inferred from
-0.59829	Cd163	CD163 antigen		0006953 /,	0005576 /,	0005044 // scavenger receptor activity // no
-0.22002	Gck	glucokinas	Glycolysis_	0001678 /,	0005625 /,	0000166 // nucleotide binding // inferred fro

0.163917	Rec8	REC8 homolog (yeast)	0001556 /,	0000228 /,	0005515 // protein binding // inferred from	
-0.17265	Mapk6	mitogen-a(	Integrin-m	0006468 /,	0005737 /,	0000166 // nucleotide binding // inferred fro
-0.1592	Fhl2	four and a half LIM dc	0001649 /,	0005634 /,	0005515 // protein binding // inferred from	
0.217291	Ccl27a ///	chemokine (C-C motif	0006955 /,	0005576 /,	0005125 // cytokine activity // inferred from	
-0.18739	Spnb2	spectrin beta 2	0007182 /,	0005634 /,	0003779 // actin binding // inferred from ele	
0.072424	Acad8	acyl-Coenzyme A deh\	0008152 /,	0005739 /,	0003995 // acyl-CoA dehydrogenase activity	
0.021725	Dpm1	dolichol-phosphate (b	0006506 /,	0005624 /,	0004169 // dolichyl-phosphate-mannose-pro	
-0.17245	Sorcs2	sortilin-related VPS10	0007165 /,	0016020 /,	0004872 // receptor activity // inferred from	
-0.3931	Fibin	fin bud initiation factor homolog	0005576 // extracellular region // inferred from electro			
0.069757	Med9	mediator of RNA poly	0006350 /,	0005634 /,	0005515 // protein binding // inferred from	
-0.00908	Ank1	ankyrin 1, erythroid	0006779 /,	0005634 /,	0005515 // protein binding // inferred from	
-0.01783	Tbc1d1	TBC1 domain family, r	0032313 /,	0005622 /,	0005096 // GTPase activator activity // infer	
-0.09728	Clec14a	C-type lectin domain family 14, n	0016020 /,	0005488 // binding // inferred from electror		
-0.71775	C3ar1	compleme	GPCRDB_C	0002430 /,	0005886 /,	0001850 // complement component C3a bin
-0.27752	Ripk1	receptor (1	Apoptosis ,	0006468 /,	0005737 /,	0000166 // nucleotide binding // inferred fro
-0.24128	Il12b	interleukin 12b	0002323 /,	0005576 /,	0004896 // cytokine receptor activity // infe	
-0.08714	Atp6v1c1	ATPase, H+ transporti	0006810 /,	0005737 /,	0005515 // protein binding // inferred from	
-0.04443	Mdm4	transformed mouse 3'	0000122 /,	0005622 /,	0005515 // protein binding // inferred from	
0.023135	Fank1	fibronectin type 3 and ankyrin re	0005634 // nucleus // inferred from electronic annotati			
0.009268	Abcd4	ATP-binding cassette,	0006200 /,	0005777 /,	0000166 // nucleotide binding // inferred fro	
0.18982	Zfp292	zinc finger protein 29:	0006350 /,	0005622 /,	0003676 // nucleic acid binding // inferred fr	
2.39E-04	Ctsg	cathepsin G	0006508 /,	0005882 /,	0003824 // catalytic activity // inferred from	
-0.13068	Ifi204	interferon activated g	0006350 /,	0005634 /,	0003712 // transcription cofactor activity //	
-0.25143	Ankrd2	ankyrin repeat domain 2 (stretch	0005634 // nucleus // non-traceable author statement ,			
0.071588	Myo1c	myosin IC	0006200 /,	0005634 /,	0000146 // microfilament motor activity // r	
0.118297	Taf1a	TATA box binding pro	0006350 /,	0000120 /,	0003677 // DNA binding // inferred from ele	
-0.46917	Tpm2	tropomyos	Striated_m	0006936 /,	0005737 /,	0003779 // actin binding // inferred from ele
-0.14983	Chi3l3	chitinase 3-like 3	0005975 /,	0005576 /,	0003824 // catalytic activity // inferred from	
0.119974	Trim25	tripartite motif-contai	0008152 /,	0005634 /,	0004842 // ubiquitin-protein ligase activity /	
-0.01671						
0.194779	Slc35c2	solute carrier family 3	0006810 /,	0016020 // membrane // inferred from electronic anno		
0.023313	Tcta	T-cell leukemia translocation altered gene				
-0.31626	Pax3	paired box gene 3	0000122 /,	0005634 /,	0003677 // DNA binding // inferred from ele	
0.024736	Spen	SPEN hom(	mRNA_prc	0006350 /,	0005634 /,	0000166 // nucleotide binding // inferred fro
0.11971	Ramp3	receptor (c	Smooth_m	0006810 /,	0016020 /,	0004872 // receptor activity // inferred from
-0.27899	LOC10004{	major urinary protein	0006810 /,	0005576 /,	0005215 // transporter activity // inferred fr	
0.053451	Nap1l1	nucleosome assembly	0006334 /,	0005634 /,	0005515 // protein binding // inferred from	
0.018251	Eif2s1	eukaryotic Translatior	0006412 /,	0005634 /,	0003723 // RNA binding // inferred from ele	
-0.13299	Dkk2	dickkopf homolog 2 (X	0007275 /,	0005576 // extracellular region // inferred from sequen		
-0.24923	Ccdc50	coiled-coil domain containing 50	0005737 // cytoplasm // inferred from electronic annot			
-0.1268	Ccdc50	coiled-coil domain containing 50	0005737 // cytoplasm // inferred from electronic annot			
-0.15683	Dcbld2	discoidin, CUB and LC	0007155 /,	0005887 // integral to plasma membrane // inferred fro		
0.020228	Dnajc28	DnaJ (Hsp40) homolog, subfamily C, membe	0031072 // heat shock protein binding // inf			
-0.06627	Pla2g2c	phospholip	Eicosanoid	0006644 /,	0005576 /,	0004623 // phospholipase A2 activity // not
-0.15015	Rnf13	ring finger protein 13	0008152 /,	0005634 /,	0004842 // ubiquitin-protein ligase activity /	
0.12019	Blcap	bladder cancer associ	0006915 /,	0016020 // membrane // inferred from electronic anno		
-0.26869	Ms4a4c	membrane-spanning 4-domains,	0016021 // integral to membrane // inferred from elect			

-0.38114	Sema3c	sema domain, immun	0001755 /	0005576 /	0004872 // receptor activity // inferred from
0.426801	Tex15	testis expressed gene	0007129 // synapsis // inferred from mutant phenotype /// 00071		
-0.10381	Klk4	kallikrein related-pept	0006508 // proteolysi		0003824 // catalytic activity // inferred from
-0.52231	Tsc22d3	TSC22 domain family,	0006355 // regulation		0003700 // sequence-specific DNA binding t
-0.02091	Tas1r3	taste receptor, type 1	0001582 /	0005886 /	0004871 // signal transducer activity // infer
0.006816	Otog	otogelin	0007155 /	0005576 /	0005198 // structural molecule activity // in
-0.17743	Snap23	synaptosomal-associa	0006461 /	0001950 /	0005515 // protein binding // inferred from
-0.08224	Cd2ap	CD2-associated protei	0007049 /	0001726 /	0005172 // vascular endothelial growth fact
-0.28555	Slco2a1	solute carrier organic	0006810 /	0005887 /	0005215 // transporter activity // inferred fr
0.145608	Cpsf2	cleavage a mRNA_prc	0006397 /	0005634 /	0003723 // RNA binding // inferred from ele
-0.39348	Atrx	alpha thalassemia/me	0006281 /	0000228 /	0000166 // nucleotide binding // inferred fro
-0.2103	Fancg	Fanconi anemia, comp	0001541 /	0005634 /	0005488 // binding // inferred from electror
-0.0565	Ubr1	ubiquitin protein ligas	0006511 /	0000151 /	0004842 // ubiquitin-protein ligase activity /
-0.22979	Mef2c	myocyte enhancer fac	0001568 /	0005634 /	0003677 // DNA binding // inferred from dir
0.91559	Npas2	neuronal PAS domain	0006350 /	0005634 /	0003677 // DNA binding // not recorded ///
-0.20798	Mrc2	mannose receptor, C	0006897 /	0009986 /	0004872 // receptor activity // inferred from
-0.03076	Ypel1	yippee-like 1 (Drosophila)		0005634 // nucleus // inferred from electronic annotati	
-0.03321	Mpp5	membrane protein, palmitoylate		0005634 /	0004385 // guanylate kinase activity // infer
-0.08476	Cyp7b1	cytochrome P450, fan	0006629 /	0005783 /	0004497 // monooxygenase activity // infer
-0.10163	Cyp7b1	cytochrome P450, fan	0006629 /	0005783 /	0004497 // monooxygenase activity // infer
-0.43702	Per3	period homolog 3 (Dr	0006350 /	0005634 /	0004871 // signal transducer activity // infer
-0.30924	Bmp2k	BMP2 inducible kinas	0006468 /	0005634 /	0000166 // nucleotide binding // inferred fro
-0.07422	Pga5	pepsinogen 5, group I	0006508 /	0005625 /	0004190 // aspartic-type endopeptidase acti
-0.43746	Epyc	epiphycan		0005576 /	0005515 // protein binding // inferred from
0.080419	Tmem42	transmembrane protein 42		0016020 // membrane // inferred from electronic anno	
-0.13176	Edn3	endothelin 3	0001755 /	0005576 /	0005179 // hormone activity // inferred fror
0.174006	Rpgrip1	retinitis pigmentosa G	0006260 /	0005634 /	0005515 // protein binding // inferred from
-0.08325	Nfia	nuclear factor I/A	0006260 /	0005622 /	0003677 // DNA binding // inferred from dir
-0.05353	Atrn	attractin	0006954 /	0005737 /	0004872 // receptor activity // inferred from
-0.17838	Gabrb3	gamma-aminobutyric	0006810 /	0005886 /	0004872 // receptor activity // inferred from
-0.39019	Itgav	integrin al Integrin-m	0001525 /	0005886 /	0004872 // receptor activity // inferred from
-0.03327	Nudt16	nudix (nucleoside dipl	0008152 /	0005634 /	0003723 // RNA binding // inferred from ele
-0.32464	Ciita	class II transactivator	0006350 /	0005634 /	0000166 // nucleotide binding // inferred fro
-0.15724	Il6st	interleukin 6 signal tra	0005977 /	0005615 /	0004872 // receptor activity // inferred from
-0.33096	Gzmc	granzyme C	0006508 // proteolysi		0003824 // catalytic activity // inferred from
-0.15583	Rbm38	RNA bindir mRNA_prc	0006397 /	0005634 /	0000166 // nucleotide binding // inferred fro
0.068139	Ikzf1	IKAROS family zinc fin	0001779 /	0005622 /	0003676 // nucleic acid binding // inferred fı
-0.14319	Egfl7	EGF-like domain 7	0001525 /	0005576 /	0005509 // calcium ion binding // non-trace
-0.30691	H2-M3	histocompatibility 2, I	0001916 /	0009897 /	0042605 // peptide antigen binding // infer
-0.03824	Abcc1	ATP-bindir Irinotecan_	0006200 /	0005737 /	0000166 // nucleotide binding // inferred fro
0.051459	Egr3	early growth respons	0006350 /	0005622 /	0003676 // nucleic acid binding // inferred fı
-0.0221	Pde4a	phosphodiesterase 4A	0006198 /	0005634 /	0003824 // catalytic activity // inferred from
-0.13577	Snai3	snail homolog 3 (Dros	0006350 /	0005622 /	0003676 // nucleic acid binding // inferred fı
-0.22244	Eya1	eyes absent 1 homolo	0001656 /	0005634 /	0003824 // catalytic activity // inferred from
-0.08422	St8sia5	ST8 alpha-N-acetyl-ne	0006486 /	0000139 /	0003828 // alpha-N-acetylneuraminate alph
-0.13143	Matk	megakaryocyte-associ	0006468 /	0005737 /	0000166 // nucleotide binding // inferred fro
0.140253	Dgcr2	DiGeorge syndrome c	0007155 /	0005792 /	0004872 // receptor activity // inferred from

-0.23804	Msn	moesin	0007159 /,	0001931 /,	0003779 // actin binding // not recorded ///	
-0.0036	Ak4	adenylate kinase 4	0006139 /,	0005737 /,	0000166 // nucleotide binding // inferred fro	
0.128156	Slc22a5	solute carrier family 2	0006810 /,	0005886 /,	0000166 // nucleotide binding // inferred fro	
-0.12641	Pnlip	pancreatic lipase	0006629 /,	0005615 /,	0003824 // catalytic activity // inferred from	
0.204399	Sos1	son of seve	Integrin-m 0007264 /,	0005622 /,	0003677 // DNA binding // inferred from ele	
-0.10787	Tcf12	transcription factor 12	0006350 /,	0005634 /,	0003677 // DNA binding // inferred from dir	
-0.09214	Stag1	stromal antigen 1	0007049 /,	0000785 /,	0005488 // binding // inferred from electror	
-0.1763	Tgfa	transforming growth f	0000187 /,	0005576 /,	0001948 // glycoprotein binding // inferred f	
-0.29323	4833439L1	RIKEN cDNA 4833439L19 gene			0005515 // protein binding // inferred from	
-0.19147	Hivep2	human immunodeficie	0006350 /,	0005622 /,	0003676 // nucleic acid binding // inferred fr	
-0.21671	Spry4	sprouty homolog 4 (D	0007275 /,	0005737 /,	0005515 // protein binding // inferred from	
-0.13387	Pi16	peptidase inhibitor 16	0010466 /,	0005576 /,	0030414 // peptidase inhibitor activity // inf	
0.003967	Parvb	parvin, beta	0007155 /,	0005737 /,	0003779 // actin binding // inferred from ele	
0.082676	Mc1r	melanocor	GPCRDB_C 0007165 /,	0005886 /,	0004871 // signal transducer activity // infer	
-0.17848	Gstm6	glutathione S-transfer	0008152 /,	0005737 /,	0004364 // glutathione transferase activity /	
-0.11358	Cmpk2	cytidine monophosph	0006221 /,	0005739 /,	0000166 // nucleotide binding // inferred fro	
-0.23014	Uhmk1	U2AF hom	mRNA_prc 0006468 /,	0005634 /,	0000166 // nucleotide binding // inferred fro	
0.214577	Hist2h3c2-	histone cluster 2, H3c	0006334 /,	0000786 /,	0003677 // DNA binding // inferred from ele	
-0.03477	Siglec1	sialic acid binding Ig-li	0006897 /,	0005576 /,	0005515 // protein binding // inferred from	
-0.16347	Bdnf	brain derived neurotr	0001657 /,	0005576 /,	0005102 // receptor binding // inferred from	
0.113132	Il13ra2	interleukin 13 recepto	0007165 /,	0005576 /,	0004872 // receptor activity // inferred from	
-0.02998	Cyb5r3	cytochrome b5 reduct	0006694 /,	0005625 /,	0004128 // cytochrome-b5 reductase activit	
-0.27182	Tbx15	T-box 15	0006350 /,	0005634 /,	0003677 // DNA binding // inferred from ele	
-0.01072	Tacr2	tachykinin	Peptide_G 0007165 /,	0005624 /,	0004871 // signal transducer activity // infer	
-0.0153	Fgf7	fibroblast growth fact	0001541 /,	0005576 /,	0005111 // type 2 fibroblast growth factor re	
0.007071	Col10a1	collagen, type X, alpha	0001958 /,	0005576 // extracellular region // inferred from electro		
-0.0871	Mycs	myc-like oncogene, s-	0006355 /,	0005634 /,	0003677 // DNA binding // inferred from ele	
-0.07436	Otp	orthopedia homolog (	0002052 /,	0005634 /,	0003677 // DNA binding // inferred from ele	
0.040979	Sprr3	small proline-rich prot	0031424 /,	0005737 /,	0005515 // protein binding // inferred from	
-0.32719	Col5a2	collagen, type V, alpha	0001501 /,	0005576 /,	0005201 // extracellular matrix structural co	
-0.11704	Ephx1	epoxide hydrolase 1, r	0006725 /,	0005783 /,	0003824 // catalytic activity // inferred from	
0.080784	Snx3	sorting nexin 3	0006810 /,	0005737 /,	0005515 // protein binding // inferred from	
-0.00837	Mogs	mannosyl-oligosaccha	0008152 /,	0005783 /,	0003824 // catalytic activity // inferred from	
-0.05272	Slc30a5	solute carrier family 3	0006810 /,	0005624 /,	0005385 // zinc ion transmembrane transpo	
-0.12408	Xpc	xeroderma pigmentos	0000075 /,	0005634 /,	0003677 // DNA binding // inferred from ele	
-0.18153	Thbs2	thrombospondin 2	0007155 /,	0005576 /,	0005198 // structural molecule activity // inf	
0.50686	Tmem45a	transmembrane protein 45a		0005887 // integral to plasma membrane // inferred fro		
-0.24018	Casq1	calsequest	Calcium_re 0006937 /,	0005737 /,	0005509 // calcium ion binding // inferred fr	
-0.11654	C1qtnf3	C1q and tumor necrosis factor re		0005576 // extracellular region // inferred from direct a		
-0.1541	Etv1	ets variant gene 1	0006350 /,	0005634 /,	0003677 // DNA binding // inferred from ele	
-0.16563	Ctsw	cathepsin W	0006508 // proteolysi		0004197 // cysteine-type endopeptidase act	
-0.239	Rbm3	RNA bindir	mRNA_prc 0006412 /,	0005634 /,	0000166 // nucleotide binding // inferred fro	
-0.08902	Pcmt1	protein-L-isoaspartate	0006464 /,	0005737 /,	0004719 // protein-L-isoaspartate (D-asparta	
-0.02874	Ctr9	Ctr9, Paf1/RNA polym	0010390 /,	0005634 /,	0005488 // binding // inferred from electror	
0.203807	Sub1	SUB1 homolog (S. cere	0006350 /,	0005634 /,	0003677 // DNA binding // inferred from ele	
0.010651	Bbx	bobby sox homolog (D	0006350 /,	0005634 /,	0003677 // DNA binding // inferred from ele	
0.043975	Bat2	HLA-B associated transcript 2		0005634 /,	0005515 // protein binding // inferred from	

0.061684	C9	compleme	Compleme	0006917 /,	0005576 // extracellular region // inferred from electro	
-0.10226	Stard5	StAR-related lipid trar		0006694 // steroid bi		0008289 // lipid binding // inferred from ele
-0.11513	Slc35a2	solute carrier family 3		0006810 /,	0000139 /,	0005338 // nucleotide-sugar transmembrane
-0.247	Trip4	thyroid hormone rece		0006350 /,	0005634 /,	0008270 // zinc ion binding // inferred from
-0.171	Sypl	synaptophysin-like pr		0006810 /,	0008021 /,	0005215 // transporter activity // inferred fr
0.182835	Vamp4	vesicle-associated me		0016192 /,	0005794 // Golgi apparatus // inferred from electronic a	
-0.23078	Fmo2	flavin containing mon		0006082 /,	0005783 /,	0004497 // monooxygenase activity // inferr
-0.11504	Agtrap	angiotensin II, type I r		0001666 /,	0000139 /,	0004872 // receptor activity // inferred from
-0.09123	Kat2a	K(lysine) acetyltransfe		0001701 /,	0000123 /,	0003682 // chromatin binding // inferred fro
-0.07624	Capza2	capping protein (actin		0030036 /,	0008290 /,	0003779 // actin binding // inferred from ele
-0.15266	Ptk2	PTK2 prote	Integrin-m	0000165 /,	0005634 /,	0000166 // nucleotide binding // inferred fro
-0.24524	6720475J1	RIKEN cDNA 6720475J19 gene				
0.160977	Lman2	lectin, mannose-bindi		0006810 /,	0000139 /,	0005529 // sugar binding // inferred from el
-0.08569	Snx9	sorting nexin 9		0006810 /,	0005625 /,	0005515 // protein binding // inferred from
0.022407	Sc4mol	sterol-C4-r	Cholestero	0006633 /,	0005783 /,	0000254 // C-4 methylsterol oxidase activity
-0.18817	Tmod3	tropomodulin 3		0051271 /,	0001726 /,	0003779 // actin binding // inferred from ele
-0.1366	Incenp	inner centromere pro		0000910 /,	0000775 /,	0005515 // protein binding // inferred from
-0.33225	Crbn	cereblon		0006200 /,	0005634 /,	0004176 // ATP-dependent peptidase activit
-0.22915	Crbn	cereblon		0006200 /,	0005634 /,	0004176 // ATP-dependent peptidase activit
-0.20943	Col1a2	collagen, t	Inflammat	0001501 /,	0005576 /,	0005201 // extracellular matrix structural co
-0.45214	Tcap	titin-cap	Striated_m	0001756 /,	0005737 /,	0005515 // protein binding // inferred from
-0.57696	Cd36	CD36 antigen		0001676 /,	0005622 /,	0004872 // receptor activity // not recorded
-0.34862	Itsn2	intersectin 2		0006897 /,	0005622 /,	0005089 // Rho guanyl-nucleotide exchange
-0.1255	Tm9sf4	transmembrane 9 superfamily pr			0016020 // membrane // inferred from electronic anno	
0.011263	Tmem167	transmembrane protein 167			0000139 // Golgi membrane // inferred from electronic	
-0.17846	B4galt6	UDP-Gal:betaGlcNAc l		0005975 /,	0005794 /,	0008378 // galactosyltransferase activity // r
-0.65638	Itgb1bp2	integrin beta 1 bindin		0007229 /,	0030018 /,	0005178 // integrin binding // not recorded
0.10604	Cyp2c68	cytochrome P450, fan		0055114 /,	0005783 /,	0004497 // monooxygenase activity // inferr
-0.28705	Tpbg	trophoblast glycoprotein			0005737 /,	0005515 // protein binding // inferred from
-0.29847	Hhex	hematopoietically exp		0000122 /,	0005634 /,	0003677 // DNA binding // inferred from ele
0.048066	Dnase1l2	deoxyribonuclease 1-l		0006308 /,	0005576 /,	0004518 // nuclease activity // inferred from
-0.14846	Myadm	myeloid-associated differentiatio			0016020 // membrane // inferred from electronic anno	
-0.40437	Slco1c1	solute carrier organic		0006810 /,	0016020 /,	0005215 // transporter activity // inferred fr
0.190957	Cacna1g	calcium channel, volta		0002027 /,	0005886 /,	0005216 // ion channel activity // inferred fr
-0.23454	Fbln2	fibulin 2		0010811 /,	0005576 /,	0005509 // calcium ion binding // inferred fr
-0.19105	Ptgs1	prostaglan	Eicosanoid	0001516 /,	0005634 /,	0004601 // peroxidase activity // inferred fro
-0.22522	Tead1	TEA domain family me		0006350 /,	0005634 /,	0003677 // DNA binding // inferred from dir
0.052022	Bzw2	basic leucine zipper ar		0007275 // multicellu		0005488 // binding // inferred from electror
-0.04348	Gigyf1	GRB10 interacting GYI		0048009 // insulin-like		0005515 // protein binding // inferred from
-0.21735	Steap3	STEAP family member		0006810 /,	0005768 /,	0000293 // ferric-chelate reductase activity ,
-0.09862	Jam3	junction adhesion mo		0007155 /,	0005886 // plasma membrane // inferred from electror	
-0.21792	Swap70	SWA-70 protein		0016444 /,	0005634 /,	0003677 // DNA binding // inferred from dir
0.091171	Prrt1	proline-rich transmen		0009607 /,	0016020 // membrane // inferred from electronic anno	
-0.07723	S1pr1	sphingosin	GPCRDB_C	0001525 /,	0005886 /,	0001619 // lysosphingolipid and lysophosph
-0.47897	Ednrb	endothelin	GPCRDB_C	0001755 /,	0005624 /,	0001600 // endothelin-B receptor activity //
-0.14357	Nek6	NIMA (never in mitosi		0006468 /,	0005634 /,	0000166 // nucleotide binding // inferred fro
-0.33705	Postn	periostin, osteoblast s		0007155 /,	0005576 /,	0008201 // heparin binding // inferred from

0.200651	Alpl	alkaline phosphatase,	0008152 /,	0005615 /,	0003824 // catalytic activity // inferred from	
0.01495	Pcdhga1 //	protocadherin gamma	0007155 /,	0005624 /,	0005509 // calcium ion binding // inferred fr	
-0.05662	Rnf26	ring finger protein 26			0005515 // protein binding // inferred from	
-0.11963	Col1a1	collagen, t	Inflammat	0001501 /,	0005576 /,	0005201 // extracellular matrix structural co
-0.04044	Fads1	fatty acid desaturase :	0006629 /,	0005634 /,	0005506 // iron ion binding // inferred from	
0.088037	Kctd10	potassium channel tet	0006813 /,	0005634 /,	0004842 // ubiquitin-protein ligase activity /	
0.254749	Tmem50b	transmembrane protein 50B		0005783 // endoplasmic reticulum // inferred from elec		
-0.66117	U46068	cDNA sequence U46068		0005576 /,	0008289 // lipid binding // inferred from ele	
0.087646	Oxa1l	oxidase assembly 1-lik	0009060 /,	0005739 // mitochondrion // inferred from direct assay		
0.088005	Rbm10	RNA bindir	mRNA_processing_bi	0005622 /,	0000166 // nucleotide binding // inferred fro	
0.121306	Sf1	splicing factor 1	0006350 /,	0005634 /,	0003676 // nucleic acid binding // inferred fr	
-0.0893	Athl1	ATH1, acid trehalase-l	0005975 // carbohydr		0003824 // catalytic activity // inferred from	
0.110815	Ptcd2	pentatricopeptide rep	0001822 /,	0005739 // mitochondrion // inferred from direct assay		
0.028345	Usp20	ubiquitin specific pept	0006511 /,	0005737 /,	0001664 // G-protein-coupled receptor bind	
-0.08927	Usp20	ubiquitin specific pept	0006511 /,	0005737 /,	0001664 // G-protein-coupled receptor bind	
-0.22077	Dab2	disabled homolog 2 (D	0000904 /,	0005634 /,	0005515 // protein binding // inferred from	
0.246661	Ppme1	protein phosphatase r	0006482 // protein de		0003824 // catalytic activity // inferred from	
-0.05126	Wls	wntless homolog (Dro	0001707 /,	0000139 /,	0005515 // protein binding // inferred from	
-0.04091	Wls	wntless homolog (Dro	0001707 /,	0000139 /,	0005515 // protein binding // inferred from	
-0.00908	2400003C1	RIKEN cDNA 2400003(	0007049 /,	0005793 /,	0005515 // protein binding // inferred from	
0.169265	Acsl1	acyl-CoA sy	Fatty_Acid	0006629 /,	0005625 /,	0000166 // nucleotide binding // inferred fro
-0.13623	Lamc1	laminin, ga	Inflammat	0006461 /,	0005576 /,	0005201 // extracellular matrix structural co
0.184347	Dus1l	dihydrouridine syntha	0008033 // tRNA proc		0003824 // catalytic activity // inferred from	
0.237201	Srsf7	serine/arginine-rich sp	0006397 /,	0005634 /,	0000166 // nucleotide binding // inferred fro	
0.025729	2310044H1	RIKEN cDNA 2310044H10 gene		0005576 // extracellular region // inferred from electro		
0.024197	Kdm4b	lysine (K)-specific dem	0006350 /,	0005634 /,	0003676 // nucleic acid binding // inferred fr	
-0.04567	Fgfr1	fibroblast growth fact	0001525 /,	0016020 /,	0000166 // nucleotide binding // inferred fro	
0.030078	Usp48	ubiquitin specific pept	0006511 /,	0005634 /,	0004221 // ubiquitin thiolesterase activity //	
0.246433	Gdpd1	glycerophosphodieste	0006071 /,	0016020 /,	0008081 // phosphoric diester hydrolase act	
-0.02945	Rtcd1	RNA terminal phospha	0006396 /,	0005634 /,	0000166 // nucleotide binding // inferred fro	
-0.18448	Col6a3	collagen, type VI, alpha 3		0005615 /,	0004867 // serine-type endopeptidase inhib	
0.190249	Ahsa1	AHA1, activator of hea	0006457 /,	0005737 /,	0001671 // ATPase activator activity // not r	
-0.20506	Tef	thyrotroph embryonic	0006350 /,	0005634 /,	0003677 // DNA binding // inferred from ele	
-0.17167	Morf4l1	mortality factor 4 like	0000724 /,	0000785 /,	0003682 // chromatin binding // inferred fro	
-0.08344	Yipf1	Yip1 domain family, member 1		0016020 // membrane // inferred from electronic anno		
-0.02166	Papola	poly (A) po	mRNA_pro	0006350 /,	0005634 /,	0000166 // nucleotide binding // inferred fro
0.085645	1700020C1	RIKEN cDNA 170002O(	0006915 /,	0005739 /,	0004089 // carbonate dehydratase activity /	
-0.02242	Polr3h	polymeras	RNA_trans	0006139 /,	0005634 /,	0003677 // DNA binding // inferred from ele
0.220301	Dyrk3	dual-specificity tyrosir	0006468 /,	0005634 /,	0000166 // nucleotide binding // inferred fro	
-0.2245	Meox2	mesenchyme homeot	0001525 /,	0005634 /,	0003677 // DNA binding // inferred from ele	
-0.45723	Meox2	mesenchyme homeot	0001525 /,	0005634 /,	0003677 // DNA binding // inferred from ele	
0.060147	Fam114a2	family with sequence similarity 114, member A2				
0.019539	1810043G(	RIKEN cDNA 1810043G02 gene				
0.239656	Pomt1	protein-O-mannosyltr	0006493 /,	0001669 /,	0000030 // mannosyltransferase activity // i	
-0.64555	Prkx	protein kinase, X-linke	0006468 // protein pl		0000166 // nucleotide binding // inferred fro	
0.032082	Ppcdc	phosphopantothenoy	0015937 // coenzyme		0003824 // catalytic activity // inferred from	
-0.15081	Armcx3	armadillo repeat containing, X-lir		0016020 /,	0005488 // binding // inferred from electror	

0.184135	Asrgl1	asparaginase like 1	0008152 /,	0005737 /,	0003948 // N4-(beta-N-acetylglucosaminyl)-
-0.0675	Aldh1l1 ///	aldehyde dehydrogen	0006730 /,	0005737 /,	0000036 // acyl carrier activity // inferred fro
-0.11734	Leprot	leptin receptor overla	0007165 /,	0000139 // Golgi membrane // inferred from electronic	
0.219929	Gprasp1	G protein-coupled receptor asso		0005634 /,	0001540 // beta-amyloid binding // inferred
0.055065	Ppa2	pyrophosphatase (ino	0006796 /,	0005737 /,	0000287 // magnesium ion binding // inferre
0.14202	Zfp428	zinc finger protein 428		0005622 /,	0008270 // zinc ion binding // inferred from
0.072716	Tctn3	tectonic family memb	0006915 /,	0016020 // membrane // inferred from electronic anno	
0.277186	Srgap3	SLIT-ROBO Rho GTPas	0007165 /,	0005622 /,	0005096 // GTPase activator activity // infer
-0.13046	Ddx6	DEAD (Asp mRNA_prc	0006200 /,	0000932 /,	0000166 // nucleotide binding // inferred fro
-0.26457	Sumf1	sulfatase n Ribosomal	0055114 /,	0005783 /,	0005488 // binding // inferred from electron
0.203163	Thoc1	THO complex 1	0006350 /,	0000346 /,	0003677 // DNA binding // inferred from ele
0.065097	2010011I2	RIKEN cDNA 2010011I20 gene		0016020 // membrane // inferred from electronic anno	
-0.12582	Rbmx2	RNA bindir mRNA_processing_binding_Reac		0000166 // nucleotide binding // inferred fro	
0.391474	Mapt	microtubule-associate	0000226 /,	0005737 /,	0005515 // protein binding // inferred from
-0.35632	P2ry14	purinergic GPCRDB_C	0006955 /,	0005886 /,	0004871 // signal transducer activity // infer
-0.16043	Wdfy1	WD repeat and FYVE domain con		0005634 /,	0005545 // phosphatidylinositol binding // ir
0.02334	Mios	missing oocyte, meiosis regulator, homolog (Drosophila)			
0.146757	Tmem214	transmembrane protein 214		0016020 /,	0005515 // protein binding // inferred from
-0.07358	Csnk1a1	casein kinase 1, alpha	0000902 /,	0000775 /,	0000166 // nucleotide binding // inferred fro
-0.16452	Trim34	tripartite motif-containing 34		0005622 /,	0005515 // protein binding // inferred from
-0.19052	Spg20	spastic paraplegia 20, spartin (Tro		0005737 // cytoplasm // inferred from electronic annot	
-0.30545	Spg20	spastic paraplegia 20, spartin (Tro		0005737 // cytoplasm // inferred from electronic annot	
0.088264	2310044G	RIKEN cDNA 2310044G17 gene			
-0.2298	Sec63	SEC63-like (S. cerevisi	0006457 /,	0005783 /,	0005515 // protein binding // inferred from
0.162013	2210018M	RIKEN cDNA 2210018I	0006281 /,	0005634 // nucleus // inferred from electronic annotati	
0.010323	Egfr	epidermal growth fac	0000186 /,	0005622 /,	0000166 // nucleotide binding // inferred fro
0.032188	BC022687	cDNA sequence BC022687			
-0.1325	Fbxw7	F-box and WD-40 don	0007062 /,	0005634 /,	0003700 // sequence-specific DNA binding tr
-0.07318	Vrk1	vaccinia related kinas	0006468 /,	0005634 /,	0000166 // nucleotide binding // inferred fro
0.00947	Stx18	syntaxin 18	0006810 /,	0000139 /,	0005484 // SNAP receptor activity // inferre
0.254231	Ephb2	Eph receptor B2	0000902 /,	0005887 /,	0000166 // nucleotide binding // inferred fro
-0.14836	Hmgb1	high mobility group b	0000902 /,	0005615 /,	0000400 // four-way junction DNA binding /
-0.16574	Dnajc18	DnaJ (Hsp40) homolog	0006457 /,	0016020 /,	0031072 // heat shock protein binding // inf
-0.04066	Rarres2	retinoic acid receptor	0006952 /,	0005576 // extracellular region // inferred from electro	
0.112757	BC002059	cDNA sequence BC002059		0005622 /,	0003676 // nucleic acid binding // inferred fr
0.811756	Arntl	aryl hydrocarbon rece	0000060 /,	0005634 /,	0003677 // DNA binding // inferred from phy
-0.08189	Slc44a3	solute carrier family 44, member		0016020 // membrane // inferred from electronic anno	
0.028831	2410075B	RIKEN cDNA 2410075B13 gene			
-0.61066	Myh2	myosin, he Striated_m	0001778 /,	0005730 /,	0000166 // nucleotide binding // inferred fro
0.067816	Rorb	RAR-relate Nuclear_R	0006350 /,	0005634 /,	0003677 // DNA binding // inferred from ele
-0.15193	Sel1l	sel-1 suppressor of lin	0007219 /,	0005783 /,	0005488 // binding // inferred from electron
0.167956	Gatc	glutamyl-tl mRNA_prc	0006450 /,	0005739 // mitochondrion // inferred from direct assay	
-0.20051	Atp6v0a1	ATPase, H+ transporti	0006367 /,	0005634 /,	0003682 // chromatin binding // inferred fro
-0.19471	Col20a1	collagen, type XX, alpl	0007155 /,	0005576 /,	0005198 // structural molecule activity // inf
-0.09291	Asph	aspartate-beta-hydro	0007389 /,	0005783 /,	0004597 // peptide-aspartate beta-dioxyger
-0.1792	Tsc22d3	TSC22 domain family,	0006355 // regulation		0003700 // sequence-specific DNA binding tr
-0.16967	Fam76a	family with sequence similarity 76, member A			

0.05653	Rab43	RAB43, member RAS (	0007264 /,	0005886 /,	0000166 // nucleotide binding // inferred fro
-0.22647	Zfp318	zinc finger protein 318	0006350 /,	0005622 /,	0003676 // nucleic acid binding // inferred fr
-0.01382	BC023105	cDNA sequence BC023105		0016020 /,	0005525 // GTP binding // inferred from ele
-0.0713	Cpsf4l	cleavage and polyadenylation specific factor			0003676 // nucleic acid binding // inferred fr
-0.095	Lats2	large tumor suppresso	0000082 /,	0000922 /,	0000166 // nucleotide binding // inferred fro
0.001375	Msr1	macrophage scavenge	0006897 /,	0005829 /,	0004872 // receptor activity // inferred from
0.036285	Tgfbr2	transformi TGF_Beta_	0001569 /,	0005737 /,	0000166 // nucleotide binding // inferred fro
-0.02495	Chi3l3 /// (	chitinase 3-like 3 /// c	0005975 /,	0005576 /,	0003824 // catalytic activity // inferred from
-0.1403	Grb10	growth factor recepto	0007165 /,	0005737 /,	0001784 // phosphotyrosine binding // trace
-0.40346	Mtmr2	myotubularin related	0016311 /,	0005634 /,	0004437 // inositol or phosphatidylinositol p
-0.02076	Arfrp1	ADP-ribosylation facto	0007264 /,	0005622 /,	0000166 // nucleotide binding // inferred fro
-0.03073	Hip1r	huntingtin interacting	0006897 /,	0005624 /,	0003779 // actin binding // inferred from dir
-0.05394	Tsc22d3	TSC22 domain family,	0006355 // regulation		0003700 // sequence-specific DNA binding t
-0.14807	Anxa5	annexin A5 Prostaglan	0007596 /,	0005622 /,	0005509 // calcium ion binding // inferred fr
-0.31796	Dusp3	dual specificity phosp	0000188 /,	0001772 /,	0004721 // phosphoprotein phosphatase ac
-0.03714	Il13ra1	interleukin 13 recepto	0002639 /,	0016020 /,	0004872 // receptor activity // inferred from
-0.06282	Lima1	LIM domain and actin	0030835 /,	0001725 /,	0003779 // actin binding // inferred from ele
-0.0464	Braf	Braf transf Integrin-m	0000165 /,	0005624 /,	0000166 // nucleotide binding // inferred fro
0.03794	Ccdc77	coiled-coil domain containing 77			
-0.09096	Pld1	phospholipase D1	0006654 /,	0000139 /,	0003824 // catalytic activity // inferred from
-0.08585	Six4	sine oculis-related hoi	0006355 /,	0005634 /,	0003677 // DNA binding // inferred from dir
-0.12189	Tmem167	transmembrane protein 167		0000139 // Golgi membrane // inferred from electronic	
-0.22501	Mbd2	methyl-CpG binding d	0000122 /,	0000118 /,	0003677 // DNA binding // inferred from dir
-0.06345	BC021891	cDNA sequence BC02:	0006468 // protein ph		0000166 // nucleotide binding // inferred fro
-0.14648	Pcsk4	proprotein convertase	0006508 /,	0002080 /,	0004252 // serine-type endopeptidase activi
-0.14994	Rngtt	RNA guany mRNA_prc	0006370 /,	0005634 /,	0000166 // nucleotide binding // inferred fro
0.352739	Caln1	calneuron 1		0005737 /,	0005509 // calcium ion binding // not record
0.112745	Hyal3	hyaluronoglucosamini	0005975 /,	0005576 /,	0003824 // catalytic activity // inferred from
-0.05065	Ly6i	lymphocyte antigen 6 complex, lo		0005886 // plasma membrane // traceable author state	
-0.14621	Mdfi	MyoD family inhibitor	0000122 /,	0005634 /,	0005515 // protein binding // inferred from
0.091691	Hexim1	hexamethylene bis-ac	0000122 /,	0005634 /,	0004861 // cyclin-dependent protein kinase
-0.01095	Cdadc1	cytidine and dCMP de	0008152 // metabolic		0003824 // catalytic activity // inferred from
0.248186	Trim25	tripartite motif-contai	0008152 /,	0005634 /,	0004842 // ubiquitin-protein ligase activity /
-0.0132	Tgm2	transglutaminase 2, C	0001974 /,	0005578 /,	0003810 // protein-glutamine gamma-glutar
0.022532	Rgs9	regulator c Calcium_re	0007186 /,	0005634 /,	0004871 // signal transducer activity // infer
-0.10646	Pde1c	phosphodi G_Protein_	0006198 /,	0043025 /,	0003824 // catalytic activity // inferred from
0.031978	Kcnq2	potassium voltage-gat	0006810 /,	0008076 /,	0005216 // ion channel activity // inferred fr
-0.07835	Cd244 /// I	CD244 natural killer ce	0007165 /,	0009897 /,	0004872 // receptor activity // inferred from
0.052447	Klra18	killer cell lectin-like re	0007155 /,	0005886 /,	0004872 // receptor activity // inferred from
-0.21566	Kitl	kit ligand	0001541 /,	0005576 /,	0005125 // cytokine activity // inferred from
0.028288	Scp2	sterol carri Fatty_Acid	0006637 /,	0005737 /,	0003824 // catalytic activity // inferred from
0.03214	Vps37c	vacuolar protein sortii	0006810 /,	0005768 // endosome // inferred from electronic annot	
-0.48628	Vit	vitrin	0010811 /,	0005576 /,	0005539 // glycosaminoglycan binding // inf
0.042286	Ap1ar	adaptor-related protein complex		0030133 // transport vesicle // inferred from electronic	
-0.06553	Lama2	laminin, alpha 2	0007155 /,	0005576 /,	0005102 // receptor binding // inferred from
0.087038	Zdhhc5	zinc finger, DHHC dom	0008152 /,	0016020 /,	0008270 // zinc ion binding // inferred from
-0.09559	Stt3b	STT3, subunit of the o	0006486 /,	0005783 /,	0004576 // oligosaccharyl transferase activit

0.084786	Taok1	TAO kinase 1		0001934 /,	0005737 /,	0000166 // nucleotide binding // inferred fro
-0.22096	Zc3h11a	zinc finger CCCH type containing 11A				0003676 // nucleic acid binding // inferred fr
-0.08002	Zc3h11a	zinc finger CCCH type containing 11A				0003676 // nucleic acid binding // inferred fr
0.03695	Rin2	Ras and Rab interacto		0006897 /,	0005737 /,	0005096 // GTPase activator activity // infer
-0.14139	Cry2	cryptochrc	Circadian_	0006281 /,	0005634 /,	0000166 // nucleotide binding // inferred fro
0.284318	Pja1	praja1, RING-H2 moti		0008152 /,	0005737 /,	0004842 // ubiquitin-protein ligase activity /
-0.07939	Plcl2	phospholipase C-like 2		0006629 /,	0005737 /,	0004435 // phosphoinositide phospholipase
-0.06968	Pitrm1	pitrilysin metallepetid		0006508 /,	0005739 /,	0003824 // catalytic activity // inferred from
-0.07635	Pias2	protein inhibitor of ac		0006350 /,	0005634 /,	0003676 // nucleic acid binding // inferred fr
-0.62343	Slmap	sarcolemma associated protein			0005737 // cytoplasm // inferred from electronic annot	
-0.06819	Gphn	gephyrin		0006777 /,	0005622 /,	0000166 // nucleotide binding // inferred fro
-0.13904	Nr1d1	nuclear receptor subfa		0006350 /,	0005634 /,	0003677 // DNA binding // inferred from dir
-0.48075	Kdm5c	lysine (K)-specific dem		0006350 /,	0005622 /,	0003677 // DNA binding // inferred from ele
0.144234	Rnf121	ring finger protein 121			0016020 /,	0005515 // protein binding // inferred from
-0.51307	Evi2b	ecotropic viral integration site 2b			0016020 // membrane // inferred from electronic anno	
0.020584	Tagln2	transgelin 2		0007517 /,	0005886 /,	0005515 // protein binding // inferred from
0.140809	Atpbd4	ATP binding domain 4				
-0.18135	Add3	adducin 3 (gamma)		0042493 /,	0000794 /,	0003779 // actin binding // inferred from ele
-0.09314	Sord	sorbitol dehydrogenas		0006060 /,	0005624 /,	0003824 // catalytic activity // inferred from
0.053726	Zmynd8	zinc finger, MYND-typ		0016310 // phosphory		0005515 // protein binding // inferred from
-0.04949	Pus7	pseudouridylate synth		0001522 // pseudouri		0003723 // RNA binding // inferred from ele
-0.18713	Fn1	fibronectir	Inflammat	0001525 /,	0005576 /,	0002020 // protease binding // not recorded
-0.2111	Myh8	myosin, he	Striated_m	0006936 /,	0005737 /,	0000166 // nucleotide binding // inferred fro
-0.12112	Mcm3	minichrom	Cell_Cycle_	0006260 /,	0005634 /,	0000166 // nucleotide binding // inferred fro
0.149644	Cnot6	CCR4-NOT transcriptic		0006350 /,	0005634 /,	0003723 // RNA binding // inferred from ele
0.130012	Hnrnpm	heterogen	mRNA_prc	0000380 /,	0005634 /,	0000166 // nucleotide binding // inferred fro
-0.12788	Des	desmin	Striated_m	0007517 /,	0005622 /,	0005198 // structural molecule activity // inf
-0.034	1810026J2	RIKEN cDNA 1810026J23 gene				
-0.07129	Wasl	Wiskott-Al	G13_Signa	0006350 /,	0000139 /,	0003779 // actin binding // inferred from ele
-0.03237	Kpna6	karyopherin (importin		0006606 /,	0005634 /,	0005488 // binding // inferred from electror
-0.00576	Ahcyl1	S-adenosylhomocyste		0006730 /,	0005783 /,	0003824 // catalytic activity // inferred from
-0.09997	Sec24b	Sec24 related gene fa		0006810 /,	0030127 /,	0005515 // protein binding // inferred from
0.273872	Nov	nephroblastoma over		0001558 /,	0005576 /,	0005520 // insulin-like growth factor binding
-0.05325	Set	SET nuclear oncogene		0006334 /,	0005634 // nucleus // inferred from direct assay /// 000	
-0.12424	Chst14	carbohydrate (N-acety		0005975 /,	0000139 /,	0001537 // N-acetylgalactosamine 4-O-sulfo
0.380836	Dapk1	death associated prot		0006468 /,	0005737 /,	0000166 // nucleotide binding // inferred fro
-0.0169	6330406I1	RIKEN cDNA 6330406I15 gene				
0.084521	Fbxw8	F-box and	Apoptosis_	0006511 /,	0019005 /,	0005515 // protein binding // inferred from
-0.09103	Col6a2	collagen, type VI, alph		0007155 /,	0005576 /,	0005515 // protein binding // inferred from
0.130111	Trp53bp1	transformation relate		0006281 /,	0000775 /,	0003677 // DNA binding // inferred from ele
0.078712	Rps9	ribosomal	mRNA_prc	0006412 /,	0005622 /,	0003723 // RNA binding // inferred from ele
-0.19849	Trim23	tripartite motif-contai		0006471 /,	0000139 /,	0000166 // nucleotide binding // inferred fro
-0.18749	Klhl2 /// LC	kelch-like 2, Mayven (Drosophila)			0001725 /,	0003779 // actin binding // not recorded ///
0.05869	Flywch1	FLYWCH-type zinc finger 1			0005634 /,	0003677 // DNA binding // inferred from ele
0.104274	5430417L2	RIKEN cDNA 5430417L22 gene				
0.170636	Xpr1	xenotropic	Calcium_re	0009615 /,	0005886 /,	0004872 // receptor activity // inferred from
0.146017	Lancl1	LanC (bacterial lantibi		0007186 /,	0005634 /,	0003824 // catalytic activity // inferred from

-0.39099	BC013712	cDNA sequence BC01:	0006954 // inflammatory response // inferred from electronic ann		
0.220234	Adamts15	a disintegrin-like and ı	0006508 /,	0005576 /,	0004222 // metalloendopeptidase activity //
0.154252	Mapre3	microtubule-associate	0007049 /,	0005737 /,	0005515 // protein binding // inferred from
0.075418	Adamts10	a disintegrin-like and ı	0006508 /,	0005576 /,	0004222 // metalloendopeptidase activity //
0.0471	Fchsd1	FCH and double SH3 domains 1			
-0.25531	Col14a1	collagen, type XIV, alp	0007155 /,	0005576 /,	0005198 // structural molecule activity // inf
-0.16986	Pard3b	par-3 partitioning def	0007049 /,	0005923 /,	0005515 // protein binding // inferred from
-0.07354	Npr2	natriuretic peptide re	0001503 /,	0005624 /,	0000166 // nucleotide binding // inferred fro
-0.18657	Zranb1	zinc finger, RAN-bindi	0008152 /,	0005622 /,	0004221 // ubiquitin thiolesterase activity //
-0.15862	AA881470	EST AA881470	0010212 /,	0005634 /,	0005488 // binding // inferred from electron
-0.38367	A230046K0	RIKEN cDNA A230046	0016197 /,	0071203 // WASH complex // inferred from electronic a	
0.079914	Adora1	adenosine GPCRDB_C	0000186 /,	0005783 /,	0001609 // adenosine receptor activity, G-pr
0.127875	Med19	mediator of RNA poly	0006350 /,	0005634 /,	0005515 // protein binding // inferred from
0.242506	Fastkd1	FAST kinase domains :	0006915 // apoptosis		0004672 // protein kinase activity // inferrec
-0.09485	Rasd2	RASD family, member	0001963 /,	0005622 /,	0000166 // nucleotide binding // inferred fro
-0.03453	Myo1b	myosin IB	0008152 /,	0005903 /,	0000166 // nucleotide binding // inferred fro
-0.20656	Wdfy3	WD repeat and FYVE c	0008152 /,	0005635 /,	0003831 // beta-N-acetylglucosaminylglycop
-0.23163	Fbxl3	F-box and leucine-rich	0031146 /,	0005634 /,	0004842 // ubiquitin-protein ligase activity /
-0.32698					
0.133894	Arhgap20	Rho GTPase activating	0007165 /,	0005622 /,	0005096 // GTPase activator activity // infer
0.149396	Papola	poly (A) po mRNA_prc	0006350 /,	0005634 /,	0000166 // nucleotide binding // inferred fro
0.017793	Rian	RNA imprinted and accumulated		0005634 // nucleus // inferred from direct assay	
-5.20E-04	Sema4b	sema domain, immun	0007165 /,	0016020 /,	0004872 // receptor activity // inferred from
-0.2058	Tcrg-V1	T-cell receptor gamma, variable 1			0004872 // receptor activity // inferred from
0.002432	Nek4	NIMA (never in mitosi	0006468 /,	0005634 /,	0000166 // nucleotide binding // inferred fro
-0.00424					
-0.16561	Tcrb-J	T-cell receptor beta, joining regic		0016020 /,	0004872 // receptor activity // inferred from
-0.0665	Tcf12	transcription factor 1:	0006350 /,	0005634 /,	0003677 // DNA binding // inferred from dir
-0.07798	Grik1	glutamate receptor, ic	0006810 /,	0005886 /,	0004872 // receptor activity // inferred from
-0.26077	Lats1	large tumor suppresso	0000086 /,	0000922 /,	0000166 // nucleotide binding // inferred fro
0.156494	Solh	small optic lobes hom	0006508 /,	0005622 /,	0004197 // cysteine-type endopeptidase act
-0.05616	LOC66550(	similar to T-cell receptor beta-2 chain C regi			0004872 // receptor activity // inferred from
0.01749	Igh-VJ558	immunoglobulin heavy chain (J558 family)			
0.054867	Zc3h15	zinc finger CCCH-type	0019221 /,	0005634 /,	0003676 // nucleic acid binding // inferred fr
-0.31639	Col3a1	collagen, t Inflammat	0001501 /,	0005576 /,	0005201 // extracellular matrix structural co
0.031928	Rprd1b	regulation of nuclear pre-mRNA domain containing 1B			
0.108405	Suds3	suppressor of defectiv	0006350 /,	0016580 /,	0005515 // protein binding // inferred from
-0.00234	1110037F(	RIKEN cDNA 1110037I	0006397 /,	0005634 // nucleus // inferred from electronic annotati	
-0.20782	Tmem173	transmembrane prote	0006915 /,	0005739 /,	0005515 // protein binding // inferred from
-0.11066	LOC10004!	transcription elongati	0006350 /,	0005634 /,	0003702 // RNA polymerase II transcription
-0.12108	1200003I1	RIKEN cDNA 1200003I10 gene /// RIKEN cDNA 1200015M12 gene /// RIKEN cDNA A1300			
-0.05598	Lyrm2	LYR motif containing 2		0005739 // mitochondrion // inferred from direct assay	
-0.14426	Acyp2	acylphosphatase 2, m	0008152 /,	0005739 /,	0003998 // acylphosphatase activity // inferı
0.038558	Abhd10	abhydrolase domain c	0006508 /,	0005739 /,	0008233 // peptidase activity // inferred fror
0.115725	Synj2bp	synaptojanin 2 bindin	0006605 /,	0005737 /,	0005515 // protein binding // inferred from
-0.35398	AF251705	cDNA sequence AF25:	0007165 /,	0005886 /,	0004872 // receptor activity // inferred from
-0.20235	Mkl2	MKL/myocardin-like 2	0001701 /,	0005634 /,	0003676 // nucleic acid binding // inferred fı

-0.10925	Zfa /// Zfx	zinc finger protein, au	0001541 /,	0005622 /,	0003676 // nucleic acid binding // inferred fr
-0.07622	Ahnak	AHNAK nucleoprotein (desmoyol		0005911 /,	0005515 // protein binding // inferred from
-0.17856	Slc44a2	solute carrier family 4	0006810 /,	0016020 /,	0004871 // signal transducer activity // infer
-0.06971	Rasl12	RAS-like, family 12	0006184 /,	0016020 /,	0000166 // nucleotide binding // inferred fro
0.093056	Ptcd3	pentatricopeptide repeat domair		0005739 /,	0005515 // protein binding // inferred from
-0.14159	Sh3bgrl	SH3-binding domain glutamic acid-rich prote			0017124 // SH3 domain binding // inferred f
0.021947	Dynlt1a //,	dynein light chain Tctє	0000132 /,	0001917 /,	0003774 // motor activity // inferred from e
0.18231	Atxn7l3b	ataxin 7-like 3B			
0.095556	Tspan9	tetraspanin 9		0016020 // membrane // inferred from electronic anno	
0.042394	Gm16382 ,	ribosomal Ribosomal	0006412 /,	0005622 /,	0003735 // structural constituent of ribosom
-0.09025	Hmha1	histocompatibility (mi	0007165 /,	0005622 /,	0005096 // GTPase activator activity // infer
0.087342	Pdrg1	p53 and DNA damage	0006457 /,	0005737 /,	0051082 // unfolded protein binding // infer
0.181071	Mdp1	magnesium-depender	0008152 // metabolic		0004721 // phosphoprotein phosphatase ac
-0.32594	Arhgap21	Rho GTPase activating	0007165 /,	0000139 /,	0005096 // GTPase activator activity // infer
-0.27118	Ppat	phosphoribosyl pyrop	0006164 /,	0005625 /,	0004044 // amidophosphoribosyltransferase
0.034212	Ppfia4	protein tyrosine phosphatase, receptor type			0005515 // protein binding // inferred from
0.04839	Ppfia4	protein tyrosine phosphatase, receptor type			0005515 // protein binding // inferred from
-0.18418	Blvra	biliverdin reductase A	0008152 /,	0005625 /,	0000166 // nucleotide binding // not record
0.130404	Ddx51	DEAD (Asp-Glu-Ala-As	0006200 /,	0005634 /,	0000166 // nucleotide binding // inferred fro
0.050329	2310003L2	RIKEN cDNA 2310003	0008152 /,	0005739 /,	0008168 // methyltransferase activity // infe
0.187096	2700097O(	RIKEN cDNA 2700097O09 gene			0008757 // S-adenosylmethionine-depender
0.293242	Nolc1	nucleolar and coiled-b	0006970 /,	0005654 /,	0003700 // sequence-specific DNA binding tr
-0.02175	Msl1	male-specific lethal 1	0016568 /,	0005634 // nucleus // inferred from electronic annotati	
-0.47704	Snw1	SNW domain containi	0000398 /,	0005634 /,	0003713 // transcription coactivator activity
-0.34315	Itih5	inter-alpha (globulin)	0010466 /,	0005576 /,	0004867 // serine-type endopeptidase inhib
0.114322	Pus3	pseudouridine syntha	0001522 /,	0005634 /,	0003723 // RNA binding // inferred from ele
-0.11828	Anxa6	annexin A6 Prostaglan	0006816 /,	0005737 /,	0005509 // calcium ion binding // inferred fr
0.103842	Col22a1	collagen, type XXII, alpha 1			
0.004918					
-0.22181	Qk	quaking Circadian_	0001570 /,	0005634 /,	0003723 // RNA binding // traceable author
-0.32104	Acad10	acyl-Coenzyme A deh	0008152 /,	0005739 /,	0003824 // catalytic activity // inferred from
-0.25369	Lyve1	lymphatic vessel endc	0006027 /,	0005886 /,	0004872 // receptor activity // inferred from
0.054857	Styx	serine/threonine/tyrc	0006470 /,	0005737 /,	0004721 // phosphoprotein phosphatase ac
-0.06267	1700017B(	RIKEN cDNA 1700017B05 gene			
0.008791	Ppih	peptidyl prolyl isomer	0006397 /,	0005634 /,	0003755 // peptidyl-prolyl cis-trans isomeras
-0.09082	Cdc37l1	cell division cycle 37 homolog (S.		0005737 // cytoplasm // inferred from direct assay /// (	
-0.31508	Gna13	guanine nu G13_Signa	0001525 /,	0005834 /,	0000166 // nucleotide binding // inferred fro
-0.12666	Steap3	STEAP family member	0006810 /,	0005768 /,	0000293 // ferric-chelate reductase activity ,
-0.23792	0610010K(	RIKEN cDNA 0610010K06 gene		0016020 // membrane // inferred from electronic anno	
-0.0059	Mbd1	methyl-CpG binding d	0006306 /,	0000785 /,	0003677 // DNA binding // inferred from dir
-0.19514	Ccdc50	coiled-coil domain containing 50		0005737 // cytoplasm // inferred from electronic annot	
0.12539	Tars2	threonyl-tRNA synthe	0006412 /,	0005737 /,	0000166 // nucleotide binding // inferred fro
-0.03493	Pmm2	phosphomannomutas	0008152 /,	0005737 /,	0003824 // catalytic activity // inferred from
0.049111					
0.294542	Stx6	syntaxin 6	0006810 /,	0000139 /,	0005484 // SNAP receptor activity // inferre
0.079791	Rgl3	ral guanine nucleotide	0007165 /,	0005622 /,	0005085 // guanyl-nucleotide exchange fact
0.136435	Zfp329	zinc finger protein 32!	0006350 /,	0005622 /,	0003676 // nucleic acid binding // inferred fr

-0.0463	Il34	interleukin 34	0001934 /,	0005576 /,	0005125 // cytokine activity // inferred from
0.256412	Mina	myc induced nuclear a	0008283 /,	0005634 // nucleus // not recorded /// 0005634 // nucl	
0.116013	Prss41	protease, serine, 41	0006508 /,	0005886 /,	0003824 // catalytic activity // inferred from
0.523659	Prrx2	paired related homeo	0002053 /,	0005634 /,	0003677 // DNA binding // inferred from ele
-0.30981	Ak3	adenylate kinase 3	0006139 /,	0005737 /,	0000166 // nucleotide binding // inferred fro
0.111105	Cdc23	CDC23 (cell division cy	0000080 /,	0005622 /,	0005488 // binding // inferred from electron
0.12408	Pnlip	pancreatic lipase	0006629 /,	0005615 /,	0003824 // catalytic activity // inferred from
0.220958	Dos	downstream of Stk11		0016020 // membrane // inferred from electronic anno	
0.201384	Pygb	brain glycogen phospl	0005975 /,	0005625 /,	0004645 // phosphorylase activity // inferre
-0.04058	Marveld1	MARVEL (membrane-associating		0005634 // nucleus // inferred from electronic annotati	
0.006524	Ran	RAN, member RAS on	0006184 /,	0005634 /,	0000166 // nucleotide binding // inferred fro
0.091753	Nlrx1	NLR family member X	0044419 /,	0005739 /,	0000166 // nucleotide binding // inferred fro
0.196128	Ppp1r3c	protein ph Circadian_	0005975 /,	0005625 /,	0004721 // phosphoprotein phosphatase ac
0.214629	Ptplad1	protein tyrosine phos	0006633 /,	0005783 /,	0005515 // protein binding // inferred from
-0.16042	D15Ertd62	DNA segment, Chr 15, ERATO Doi 621, expressed			
-8.43E-04	S100pbp	S100P binding protein		0005634 /,	0048306 // calcium-dependent protein bind
0.045754	Cog1	component of oligom	0006810 /,	0000139 /,	0005515 // protein binding // inferred from
-0.30221	Lrrfip1	leucine rich repeat (in	0006350 /,	0005634 /,	0003677 // DNA binding // inferred from ele
0.105057	Dnajc11	DnaJ (Hsp40) homolog, subfamily		0005739 /,	0031072 // heat shock protein binding // inf
0.321458	Syt17	synaptotagmin XVII	0006810 /,	0005802 /,	0005215 // transporter activity // inferred fr
-0.07927	Vamp3	vesicle-associated me	0001921 /,	0005792 /,	0005515 // protein binding // inferred from
-0.23955	Cdkn1b	cyclin-depe Cell_Cycle_	0001666 /,	0005634 /,	0004860 // protein kinase inhibitor activity /
-0.41588	Lgi4	leucine-rich repeat LG	0008344 /,	0005576 /,	0005515 // protein binding // inferred from
-0.07562	Dcaf8	DDB1 and CUL4 associated facto	0080008 /,	0005515 // protein binding // inferred from	
0.129169	2310061I0	RIKEN cDNA 2310061I04 gene		0005739 // mitochondrion // inferred from direct assay	
0.187411	Polb	polymeras Nucleotide	0006260 /,	0000795 /,	0003677 // DNA binding // not recorded ///
-0.67039	Upk3b	uroplakin 3B	0010629 /,	0005886 // plasma membrane // inferred from electron	
-0.13098	Paxip1	PAX interacting (with	0006974 /,	0005622 /,	0005515 // protein binding // inferred from
0.106892	Solh	small optic lobes hom	0006508 /,	0005622 /,	0004197 // cysteine-type endopeptidase act
0.19327	Solh	small optic lobes hom	0006508 /,	0005622 /,	0004197 // cysteine-type endopeptidase act
0.079043	Samhd1	SAM domain and HD	0008152 /,	0005634 /,	0003824 // catalytic activity // inferred from
-0.08642	Col5a1	collagen, type V, alph	0001568 /,	0005576 /,	0005198 // structural molecule activity // inf
0.315681	Anp32a	acidic (leucine-rich) nu	0006350 /,	0005634 /,	0005515 // protein binding // inferred from
0.014021	Tada2b	transcriptional adapto	0045449 /,	0005634 /,	0003677 // DNA binding // inferred from ele
0.099112	Tjp2	tight junction protein	0010033 /,	0005634 /,	0005515 // protein binding // inferred from
0.096824	Tjp2	tight junction protein	0010033 /,	0005634 /,	0005515 // protein binding // inferred from
0.046966	Vprbp	Vpr (HIV-1) binding protein		0005634 /,	0005488 // binding // inferred from electron
0.141994	Rnf123	ring finger protein 12	0008152 /,	0005737 /,	0005515 // protein binding // inferred from
-0.39265	Tbl1x	transducin (beta)-like	0006350 /,	0005634 /,	0003677 // DNA binding // inferred from dir
0.062999	3110056O	RIKEN cDNA 3110056	0008152 /,	0000139 /,	0004190 // aspartic-type endopeptidase acti
0.03252	Ncan	neurocan	0006887 /,	0005576 /,	0005488 // binding // inferred from electron
0.150164	Drg1	developmentally regulated GTP b		0005622 /,	0000166 // nucleotide binding // inferred fro
-0.02141	Kctd12	potassium channel tet	0006813 /,	0005886 /,	0005249 // voltage-gated potassium channe
0.097077	Opa3	optic atrophy 3 (human)		0005739 // mitochondrion // inferred from direct assay	
-0.04564	Hivep2	human immunodefici	0006350 /,	0005622 /,	0003676 // nucleic acid binding // inferred fr
0.076072	Phf2	PHD finger protein 2	0006350 /,	0005634 /,	0005515 // protein binding // inferred from
-0.10427	Dnajc27	DnaJ (Hsp40) homolog	0007264 /,	0005739 /,	0000166 // nucleotide binding // inferred fro

-0.08527	Amz2	archaelysin family me	0008152 // metabolic	0008233 // peptidase activity // inferred fro
0.075974	Zfr2	zinc finger RNA bindin	0006955 /,	0005622 /,	0003723 // RNA binding // inferred from ele
-0.05709	2010111I0	RIKEN cDNA 2010111I	0006508 /,	0005737 /,	0004177 // aminopeptidase activity // inferr
0.054696	Ahsa2	AHA1, activator of hea	0006950 /,	0005737 /,	0001671 // ATPase activator activity // inferr
0.123697	Tsr2	TSR2, 20S rRNA accun	0006364 // rRNA proc	0005515 // protein binding // inferred from
-0.1811	Odf2	outer dense fiber of s	0007275 /,	0000922 /,	0005200 // structural constituent of cytoske
0.15	Dctn1 /// L	dynactin 1 /// hypoth	0008152 /,	0005737 /,	0003774 // motor activity // inferred from e
-0.05838	Pcmtd1	protein-L-isoaspartate	0006464 /,	0005737 /,	0004719 // protein-L-isoaspartate (D-asparta
0.065513	Abcc5	ATP-binding cassette,	0006200 /,	0005624 /,	0000166 // nucleotide binding // inferred fro
0.268067	Actr10	ARP10 actin-related p	0007018 /,	0005737 /,	0005515 // protein binding // inferred from
0.137333	Zbtb45	zinc finger and BTB domain conta		0005622 /,	0003676 // nucleic acid binding // inferred fr
0.019044	Sfxn2	sideroflexin 2	0006810 /,	0005739 /,	0008324 // cation transmembrane transport
-0.4514	Cd300a	CD300A antigen	0007165 /,	0005886 /,	0004872 // receptor activity // inferred from
-0.15831	Atp1a1	ATPase, Na+/K+ trans	0002026 /,	0005624 /,	0000166 // nucleotide binding // inferred fro
0.143141	Hr	hairless	0006350 /,	0000118 /,	0003677 // DNA binding // inferred from ele
0.083724	Cnot3	CCR4-NOT transcriptio	0006350 /,	0005634 /,	0005515 // protein binding // inferred from
-0.08584	Myo15b	myosin XVB		0005856 /,	0005488 // binding // inferred from electron
0.14324	Tln1	talin 1 Integrin-m	0007016 /,	0001726 /,	0003779 // actin binding // inferred from ele
0.41534	Dnajc5	DnaJ (Hsp40) homolog	0006457 /,	0016020 /,	0031072 // heat shock protein binding // inf
0.007527	Tceb1	transcription elongati	0006350 /,	0005634 /,	0003702 // RNA polymerase II transcription
0.060116	Grina	glutamate receptor, ionotropic, N		0016020 // membrane // inferred from electronic anno	
-0.1364	Gm505	Predicted gene 505		0016607 // nuclear speck // inferred from electronic an	
-0.03655	Nfix	nuclear factor I/X	0006260 /,	0005622 /,	0003677 // DNA binding // inferred from ele
-0.1789	Ptpn14	protein tyrosine phos	0006470 /,	0005737 /,	0004721 // phosphoprotein phosphatase ac
-0.20871	Ptgs1	prostaglan Eicosanoid	0001516 /,	0005634 /,	0004601 // peroxidase activity // inferred fro
-0.1163	Tmed2	transmembrane emp2	0006810 /,	0000139 /,	0005515 // protein binding // inferred from
-0.1276	Slc35a2	solute carrier family 3	0006810 /,	0000139 /,	0005338 // nucleotide-sugar transmembrane
0.146846	1700019G1	RIKEN cDNA 1700019	0008152 // metabolic	0008080 // N-acetyltransferase activity // in
0.08305	1810032O0	RIKEN cDNA 1810032O08 gene			
-0.30266	Snca	synuclein, alpha	0001774 /,	0005624 /,	0000287 // magnesium ion binding // inferre
0.140855	Tars2	threonyl-tRNA synthe	0006412 /,	0005737 /,	0000166 // nucleotide binding // inferred fro
-0.06349	1700010I1	RIKEN cDNA 1700010I14 gene			
-0.06763	Tacc3	transforming, acidic c	0000226 /,	0005737 /,	0019904 // protein domain specific binding /
0.051786	Ero1l /// G	ERO1-like (Ribosomal	0006412 /,	0005622 /,	0003735 // structural constituent of ribosom
0.13739	Clns1a	chloride channel, nucl	0006821 /,	0005634 /,	0005515 // protein binding // inferred from
0.150789	Rbm14	RNA bindir mRNA_pro	0006350 /,	0005634 /,	0000166 // nucleotide binding // inferred fro
-0.38487	Sntb2	syntrophin, basic 2		0005737 /,	0003779 // actin binding // inferred from ele
0.072543	Rbm14	RNA bindir mRNA_pro	0006350 /,	0005634 /,	0000166 // nucleotide binding // inferred fro
-0.17691	Ythdf1	YTH domain family 1			
-0.0106	Snx30	sorting nexin family m	0006810 /,	0005737 /,	0005515 // protein binding // inferred from
0.054979	Xbp1	X-box binding protein	0002070 /,	0005634 /,	0003677 // DNA binding // inferred from ele
-0.05755	Dcun1d5	DCN1, defective in cullin neddylation 1, domain containing 5 (S. cerevisiae)			
-0.20533	Impad1	inositol monophospha	0001501 /,	0005794 /,	0004437 // inositol or phosphatidylinositol p
-0.06707	Bat1a	HLA-B-asso mRNA_pro	0006200 /,	0005634 /,	0000166 // nucleotide binding // inferred fro
-0.12365	Aldh9a1	aldehyde dehydrogen	0001822 /,	0005634 /,	0004029 // aldehyde dehydrogenase (NAD)
0.036234	Rps29	ribosomal protein S29	0006412 /,	0005622 /,	0003735 // structural constituent of ribosom
-0.02783	Wls	wntless homolog (Dro	0001707 /,	0000139 /,	0005515 // protein binding // inferred from

-0.00434	Tmx2	thioredoxin-related tr	0045454 /,	0016020 // membrane // inferred from electronic anno	
-0.07998	Mcl1	myeloid ce Apoptosis_	0006915 /,	0005634 /,	0005515 // protein binding // inferred from
-0.05974	Rhoa	ras homolc G13_Signa	0000902 /,	0005622 /,	0000166 // nucleotide binding // inferred fro
-0.06642	Hbb-bh1	hemoglobin Z, beta-lil	0006810 /,	0005833 /,	0005344 // oxygen transporter activity // inf
-0.45626	Tef	thyrotroph embryonic	0006350 /,	0005634 /,	0003677 // DNA binding // inferred from ele
-0.07963	Phf3	PHD finger protein 3	0006350 /,	0005634 /,	0005515 // protein binding // inferred from
-0.36921	Hhip	Hedgehog-interacting	0007165 /,	0005576 /,	0003824 // catalytic activity // inferred from
0.147351	Dtymk	deoxythymidylate kin	0006233 // dTDP bios		0000166 // nucleotide binding // inferred fro
-0.2418	Slc9a3r1	solute carrier family 9	0016055 /,	0001726 /,	0005515 // protein binding // inferred from
-0.21495	Slc9a3r1	solute carrier family 9	0016055 /,	0001726 /,	0005515 // protein binding // inferred from
-0.2021	Dbp	D site albumin promo	0006350 /,	0005634 /,	0003677 // DNA binding // inferred from ele
0.024414	Sumo1	SMT3 suppressor of m	0006950 /,	0005634 /,	0005515 // protein binding // inferred from
0.30597	Dos	downstream of Stk11		0016020 // membrane // inferred from electronic anno	
-0.07944	Fam120b	family with sequence	0006350 /,	0005634 /,	0005515 // protein binding // inferred from
-0.1905	Tmod3	tropomodulin 3	0051271 /,	0001726 /,	0003779 // actin binding // inferred from ele
-0.21286	Tnni2	troponin I, Striated_m	0003009 /,	0005634 /,	0003779 // actin binding // inferred from ele
-0.09387	Irf3	interferon Apoptosis_	0006350 /,	0005634 /,	0003677 // DNA binding // inferred from ele
0.030606	Thyn1	thymocyte nuclear protein 1		0005634 // nucleus // inferred from direct assay /// 000	
-0.02565	Mtch2	mitochondrial carrier	0006810 /,	0005739 /,	0005488 // binding // inferred from electron
0.045332	Spata5	spermatogenesis asso	0007275 /,	0005737 /,	0000166 // nucleotide binding // inferred fro
-0.0888	Fibp	Fibroblast growth factor (acidic) i		0005634 /,	0005515 // protein binding // inferred from
0.140488	Rpn1	ribophorin Proteasom	0006486 /,	0005783 /,	0004579 // dolichyl-diphosphooligosaccharid
-0.173	Nup54	nucleoporin 54	0006605 /,	0005634 /,	0005487 // nucleocytoplasmic transporter a
-0.14853	Tfpt	TCF3 (E2A) fusion par	0006917 /,	0005634 /,	0005515 // protein binding // inferred from
-0.17657	Ufm1	Ubiquitin-fold modifie	0071569 /,	0005634 // nucleus // inferred from electronic annotati	
0.07071	Haus2	HAUS augmin-like con	0007049 /,	0005737 // cytoplasm // inferred from electronic annot	
0.156793	Rpn1	ribophorin Proteasom	0006486 /,	0005783 /,	0004579 // dolichyl-diphosphooligosaccharid
-0.41906	U46068	cDNA sequence U46068		0005576 /,	0008289 // lipid binding // inferred from ele
0.158211	Fgf11	fibroblast growth factor 11		0005634 /,	0008083 // growth factor activity // inferred
0.207725	Serbp1	serpine1 mRNA bindir	0045767 /,	0005634 /,	0003723 // RNA binding // inferred from ele
-0.28287	Bach1	BTB and CNC homolog	0006350 /,	0005634 /,	0003677 // DNA binding // inferred from dir
0.024421	Cbfa2t3	core-binding factor, ru	0006350 /,	0000139 /,	0003700 // sequence-specific DNA binding t
-0.12834	Erlin1	ER lipid raft associate	0030433 /,	0005783 /,	0005515 // protein binding // inferred from
-0.07017					
0.077094	Gli2	GLI-Kruppel family me	0000122 /,	0005622 /,	0003676 // nucleic acid binding // inferred fr
-0.21803					
-0.06436	Fancc	Fanconi anemia, comp	0002262 /,	0000785 /,	0005515 // protein binding // inferred from
0.008124	Rpl24	ribosomal Ribosomal_	0000027 /,	0005622 /,	0003676 // nucleic acid binding // inferred fr
-0.18708	Nucks1	nuclear casein kinase and cyclin-c		0005634 // nucleus // inferred from direct assay /// 000	
-0.0862	D1Ertd75e	DNA segment, Chr 1, ERATO Doi 75, expressed			
-0.12251	Huwe1	HECT, UBA and WWE	0000209 /,	0005622 /,	0003677 // DNA binding // inferred from ele
0.025554	Fdft1	farnesyl di Cholestero	0006694 /,	0005783 /,	0003824 // catalytic activity // inferred from
-0.26014	Aldh7a1	aldehyde dehydrogen	0008152 /,	0005634 /,	0004029 // aldehyde dehydrogenase (NAD)
-0.22454	Grn	granulin	0001835 /,	0005576 /,	0005125 // cytokine activity // inferred from
0.139661	Top3b	topoisomerase (DNA)	0006259 /,	0000793 /,	0000166 // nucleotide binding // inferred fro
0.104249	Cct3	chaperonin containing	0006457 /,	0005737 /,	0000166 // nucleotide binding // inferred fro
-0.34883	H19	H19 fetal liver mRNA	0008285 /,	0005737 // cytoplasm // not recorded /// 0005844 // po	

0.082206	Taf5l	TAF5-like RNA polyme	0006350 //	0005634 //	0003713 // transcription coactivator activity	
0.014086	Usp10	ubiquitin specific pept	0006281 //	0005634 //	0002039 // p53 binding // inferred from elec	
-0.40204	Tspan4	tetraspanin 4		0016020 //	0005515 // protein binding // inferred from	
-0.21822	Ebf1	early B-cell factor 1	0006350 //	0005634 //	0003677 // DNA binding // inferred from dir	
0.204388	Tnk2	tyrosine ki	G13_Signa 0006468 //	0005886 //	0000166 // nucleotide binding // inferred fro	
-0.04234	Uqcc	ubiquinol-cytochrome c reductas		0005739 // mitochondrion // inferred from direct assay		
0.182475	Tomm34	translocase of outer mitochondri		0005737 //	0005488 // binding // inferred from electron	
0.063326	Ube2d2	ubiquitin-conjugating	0016567 // protein ub		0000166 // nucleotide binding // inferred fro	
0.109824	Snrpg	small nuclear ribonucl	0006397 //	0005634 //	0003723 // RNA binding // inferred from ele	
-0.23002	Sema3b	sema domain, immun	0007165 //	0005576 //	0004872 // receptor activity // inferred from	
-0.04276	Ripk3	receptor-interacting s	0006468 //	0005622 //	0000166 // nucleotide binding // inferred fro	
-0.39427	Top2b	topoisomerase (DNA)	0001764 //	0000792 //	0000166 // nucleotide binding // inferred fro	
0.1142	Amd1	S-adenosylmethionine	0001701 // in utero e		0004014 // adenosylmethionine decarboxyla	
0.304977	Gas1	growth arrest specific	0002053 //	0005886 //	0005515 // protein binding // inferred from	
-0.31098	Mcl1	myeloid ce	Apoptosis_ 0006915 //	0005634 //	0005515 // protein binding // inferred from	
-0.03122	Dpagt1	dolichyl-phosphate (U	0006047 //	0005624 //	0003975 // UDP-N-acetylglucosamine-dolich	
-0.16938	Pla2g4a	phospholip	Prostaglan 0001542 //	0005624 //	0004620 // phospholipase activity // inferre	
-0.35494	Gmfb	glia maturation factor, beta		0005622 //	0003779 // actin binding // inferred from ele	
-0.04875	Col6a1	collagen, type VI, alph	0007155 //	0005576 //	0048407 // platelet-derived growth factor bi	
0.22697	Wisp1	WNT1 inducible signa	0001558 //	0005576 //	0005515 // protein binding // inferred from	
-0.05241	Mmp17	matrix met	Matrix_Me 0006508 //	0005576 //	0004222 // metalloendopeptidase activity //	
-0.55316	Lpar1	lysophosphatidic acid	0000187 //	0005737 //	0001619 // lysosphingolipid and lysophosph	
-0.00629	Prpf31	PRP31 pre-mRNA proc	0000244 //	0005634 //	0003723 // RNA binding // inferred from ele	
-0.20459	Man2a1	mannosidase 2, alpha	0001701 //	0000139 //	0003824 // catalytic activity // inferred from	
0.140075	Ift88	intraflagellar transpor	0001701 //	0005737 //	0005488 // binding // inferred from electron	
0.094311	Dnajb2	DnaJ (Hsp40) homolog	0008285 //	0000502 //	0005515 // protein binding // inferred from	
-0.0073	Ube2e3	ubiquitin-c	Proteasom 0016567 //	0005634 //	0000166 // nucleotide binding // inferred fro	
0.095575	Ubqln4	ubiquilin 4		0005634 //	0005515 // protein binding // inferred from	
-0.37858	Cxcr4	chemokine	GPCRDB_C 0001569 //	0005768 //	0004871 // signal transducer activity // infer	
0.101923	Pml	promyelocytic leukem	0001666 //	0005622 //	0003677 // DNA binding // inferred from ele	
0.098236	Angpt2	angiopoietin 2	0001525 //	0005576 //	0005102 // receptor binding // inferred from	
0.095785	Pigf	phosphatic	Circadian_ 0006506 //	0005783 //	0016780 // phosphotransferase activity, for	
-0.22996	Mafg	v-maf musculoaponeu	0001701 //	0005634 //	0003677 // DNA binding // inferred from dir	
-0.11028	Prkra	protein kinase, interfe	0006468 //	0005622 //	0003723 // RNA binding // inferred from ele	
-0.47993	F13a1	coagulation factor XIII	0006418 //	0005576 //	0003810 // protein-glutamine gamma-glutar	
-0.16161	Usp25	ubiquitin specific pept	0006511 //	0000502 //	0004221 // ubiquitin thiolesterase activity //	
-0.12323	Ino80	INO80 homolog (S. ce	0006338 //	0005634 //	0000166 // nucleotide binding // inferred fro	
-0.02544	Myo1b	myosin IB	0008152 //	0005903 //	0000166 // nucleotide binding // inferred fro	
-0.21702	Pf4	platelet factor 4	0006461 //	0005576 //	0005125 // cytokine activity // inferred from	
-0.05151	Mfap5	microfibrillar associated protein !		0001527 //	0030023 // extracellular matrix constituent c	
0.057026	Nrip1	nuclear rec	Ovarian_In 0000122 //	0000118 //	0003713 // transcription coactivator activity	
-0.12835	Ebf2	early B-cell factor 2	0006350 //	0005634 //	0003677 // DNA binding // inferred from dir	
-0.09506	Gpbp1l1	GC-rich promoter binc	0006350 //	0005634 //	0003677 // DNA binding // inferred from ele	
-0.0255	Rtp4	receptor transporter p	0001580 //	0005737 //	0005515 // protein binding // inferred from	
-0.10482	Cav1	caveolin 1,	Integrin-m 0000165 //	0000139 //	0000149 // SNARE binding // not recorded //	
-0.22064	Traf3	TNF recept	Apoptosis 0001817 // regulation		0004842 // ubiquitin-protein ligase activity /	
-0.11811	Sema4g	sema domain, immun	0007165 //	0016020 //	0004872 // receptor activity // inferred from	

-0.3691	Slco1a5	solute carrier organic	0006810 /,	0005624 /,	0005215 // transporter activity // inferred fr
-0.08467	Itgae	integrin al	Integrin-m	0007155 /,	0008305 /,	0004872 // receptor activity // inferred from
-0.01457	Grin2c	glutamate receptor, io	0001964 /,	0005886 /,	0004872 // receptor activity // inferred from
-0.13459	1700029P1	RIKEN cDNA 1700029P11 gene		0005739 // mitochondrion // inferred from direct assay	
-0.04584	Dcbld1	discoidin, CUB and LCCL domain		0016020 // membrane // inferred from electronic anno	
0.004162	Slc7a13	solute carrier family 7	0003333 /,	0016020 /,	0015171 // amino acid transmembrane tran
0.067623	Dbndd1	dysbindin (dystrobrevin binding	0005737 /,	0005515 // protein binding // inferred from	
0.091249	Zfp235	zinc finger protein 23!	0006355 /,	0005622 /,	0003676 // nucleic acid binding // inferred fr
-0.00921	Riok1	RIO kinase 1 (yeast)	0008152 // metabolic	0000166 // nucleotide binding // inferred fro	
-0.16742	Pdgfc	platelet-derived grow	0007171 /,	0005576 /,	0005161 // platelet-derived growth factor re
-0.11746	Jam2	junction adhesion molecule 2		0005886 // plasma membrane // inferred from electron	
0.173916	Dscam	Down syndrome cell a	0007155 /,	0005886 /,	0005515 // protein binding // inferred from
0.20209	Grk5	G protein-c Calcium_re	0006468 /,	0005886 /,	0000166 // nucleotide binding // inferred fro
-0.26721	Cd93	CD93 antigen	0007155 /,	0005886 /,	0001849 // complement component C1q bir
0.045066	Kcnn1	potassium intermedia	0006810 /,	0016020 /,	0005216 // ion channel activity // inferred fr
-0.20517	Gcnt1	glucosaminyl (N-acety	0008152 /,	0000139 /,	0003829 // beta-1,3-galactosyl-O-glycosyl-gl
-0.15943	Myo1c	myosin IC	0006200 /,	0005634 /,	0000146 // microfilament motor activity // r
-0.21987	Fkbp10	FK506 binding protein	0006457 /,	0005783 /,	0003755 // peptidyl-prolyl cis-trans isomera:
0.108233	Fam73b	family with sequence similarity 7		0016020 // membrane // inferred from electronic anno	
0.299045	Pkp2	plakophilin 2	0007507 /,	0005634 /,	0005488 // binding // inferred from electror
-0.92549	Prg4	proteoglycan 4 (mega	0006955 /,	0005576 /,	0005044 // scavenger receptor activity // inf
-0.00455	Actrt1	actin-related protein T1		0005737 /,	0005515 // protein binding // inferred from
-0.03621	Ephb4	Eph receptor B4	0006468 /,	0005887 /,	0000166 // nucleotide binding // inferred fro
-0.54564	Col4a3bp	collagen, type IV, alph	0000902 /,	0005737 /,	0005515 // protein binding // inferred from
-0.3681	Per1	period hon Circadian_	0006350 /,	0005634 /,	0004871 // signal transducer activity // infer
-0.29739	Hspb3	heat shock protein 3	0006950 /,	0005634 /,	0005515 // protein binding // inferred from
-0.10951	Dynlt3	dynein light chain Tcte	0006810 /,	0000775 /,	0003774 // motor activity // inferred from e
-0.5873	Cpeb4	cytoplasmi mRNA_processing_binding_Reac		0000166 // nucleotide binding // inferred fro	
0.016128	Hyal1 /// N	hyaluronoglucosamini	0005975 /,	0005576 /,	0003824 // catalytic activity // inferred from
-0.12411	Prkce	protein kin Calcium_re	0006468 /,	0000139 /,	0000166 // nucleotide binding // inferred fro
-0.12204	Nat2	N-acetyltransferase 2	0008152 /,	0005737 /,	0004060 // arylamine N-acetyltransferase ac
-0.66817	Tpm3	tropomyos Striated_m	0007420 /,	0001725 /,	0003779 // actin binding // inferred from ele
-0.4306	Tpm3	tropomyos Striated_m	0007420 /,	0001725 /,	0003779 // actin binding // inferred from ele
-0.1208	B3gnt2 ///	UDP-GlcNAc:betaGal	0006486 /,	0000139 /,	0005515 // protein binding // inferred from
0.145279	Add1	adducin 1 (alpha)	0000902 /,	0005737 /,	0003779 // actin binding // inferred from ele
0.338167	Fmn2	formin 2	0007132 /,	0015629 /,	0003779 // actin binding // inferred from ele
-0.22795	Tcerg1	transcription elongati	0006350 /,	0005634 /,	0003700 // sequence-specific DNA binding t
-0.26457	Adam10	a disintegrin and meta	0001701 /,	0005624 /,	0004175 // endopeptidase activity // inferre
-0.11351	Fzd3	frizzled ho Wnt_Signa	0001736 /,	0005886 /,	0004871 // signal transducer activity // infer
-0.64632	Mcoln3	mucolipin 3	0006810 /,	0005737 /,	0005216 // ion channel activity // inferred fr
0.095795	Ppp1cc	protein phosphatase 1	0005975 /,	0000775 /,	0004721 // phosphoprotein phosphatase act
-0.23949	Slfn2	schlafen 2	0008285 // negative r	0005524 // ATP binding // inferred from elec	
-0.01909	Ripk2	receptor (TNFRSF)-int	0001961 /,	0005622 /,	0000166 // nucleotide binding // inferred fro
-0.09866	Ngfr	nerve grov Apoptosis_	0006915 /,	0005634 /,	0004872 // receptor activity // inferred from
-0.0201	Zfp106	zinc finger protein 10	0008286 /,	0005622 /,	0001515 // opioid peptide activity // inferred
-0.02656	Tacr3	tachykinin GPCRDB_C	0007165 /,	0005634 /,	0004871 // signal transducer activity // infer
-0.50422	Ms4a4c	membrane-spanning 4-domains,		0016021 // integral to membrane // inferred from elect	

0.095901	Vax2	ventral anterior home	0000122 /,	0005634 /,	0003677 // DNA binding // inferred from ele
-0.13496	Tas1r2	taste receptor, type 1	0001582 /,	0005886 /,	0004871 // signal transducer activity // infer
0.001758	Zfp354c	zinc finger protein 354	0006350 /,	0005622 /,	0003676 // nucleic acid binding // inferred fı
0.024263	P2rx6	purinergic receptor P2	0006810 /,	0005624 /,	0004872 // receptor activity // inferred from
0.059879	Myoz3	myozenin 3		0005737 /,	0005515 // protein binding // inferred from
-0.12347	Capn6	calpain 6 Integrin-m	0006508 /,	0005622 /,	0004198 // calcium-dependent cysteine-type
0.010729	Hcfc1	host cell factor C1	0007049 /,	0005634 /,	0003713 // transcription coactivator activity
-0.07686	Smad3	MAD hom(Cell_Cycle_	0000122 /,	0005622 /,	0003677 // DNA binding // not recorded ///
-0.12588	Uhmk1	U2AF hom mRNA_prc	0006468 /,	0005634 /,	0000166 // nucleotide binding // inferred fro
0.006772	Itga2	integrin alp Integrin-m	0001666 /,	0005634 /,	0004872 // receptor activity // inferred from
-0.00831	Aen	apoptosis enhancing r	0006915 /,	0005622 /,	0003676 // nucleic acid binding // inferred fı
-0.19484	Musk	muscle, skeletal, recep	0001934 /,	0005886 /,	0000166 // nucleotide binding // inferred fro
-0.31224	H2-K1	histocompatibility 2, K	0001916 /,	0009897 /,	0042605 // peptide antigen binding // inferr
-0.05093	Mid2	midline 2	0008152 /,	0005622 /,	0005515 // protein binding // inferred from
-0.32466	Col5a2	collagen, type V, alpha	0001501 /,	0005576 /,	0005201 // extracellular matrix structural co
-0.06766	Lima1	LIM domain and actin	0030835 /,	0001725 /,	0003779 // actin binding // inferred from ele
-0.14146	Atp6v1a	ATPase, H+ transporti	0006200 /,	0005739 /,	0000166 // nucleotide binding // inferred fro
-0.19248	Ctsk	cathepsin K	0001957 /,	0005615 /,	0004197 // cysteine-type endopeptidase act
0.326255	Cryba1	crystallin, beta A1	0002088 /,	0005625 /,	0005212 // structural constituent of eye lens
-0.10127	Nup50	nucleoporin 50	0001841 /,	0005634 /,	0005487 // nucleocytoplasmic transporter a
0.006431	Phax	phosphorylated adapt	0006408 /,	0005625 /,	0003723 // RNA binding // inferred from ele
-0.48774	Saa3	serum amyloid A 3	0006953 /,	0005576 // extracellular region // inferred from electro	
0.060488	Neurog1	neurogenin 1	0006350 /,	0005634 /,	0003677 // DNA binding // inferred from ele
-0.23872	Serpinh1	serine (or cysteine) pe	0006950 /,	0005737 /,	0004867 // serine-type endopeptidase inhib
0.109786	Pa2g4	proliferation-associat	0006350 /,	0005634 /,	0003677 // DNA binding // not recorded ///
-0.04695	Ube2d3	ubiquitin-conjugating	0000209 /,	0005768 /,	0000166 // nucleotide binding // inferred fro
0.319548	Dclk1	doublecortin-like kina	0001764 // neuron m		0000166 // nucleotide binding // inferred fro
-0.06494	Hgsnat	heparan-alpha-glucos	0008152 /,	0005764 /,	0008415 // acyltransferase activity // inferre
-0.27707	Abi1	abl-interactor 1	0001756 /,	0005622 /,	0005515 // protein binding // inferred from
0.081501	Ap2m1	adaptor protein comp	0006810 /,	0005739 /,	0005515 // protein binding // inferred from
-0.14796	Smek2	SMEK homolog 2, sup	0019216 /,	0005634 /,	0005488 // binding // inferred from electror
-0.12358	Ap3b1	adaptor-related prote	0006622 /,	0005794 /,	0005488 // binding // inferred from electror
-0.25937	Slc9a3r1	solute carrier family 9	0016055 /,	0001726 /,	0005515 // protein binding // inferred from
-0.20253	Cnnm2	cyclin M2	0006810 /,	0005886 // plasma membrane // inferred from electror	
-0.25191	Sfrp4	secreted frizzled-relat	0007275 /,	0005576 /,	0005515 // protein binding // inferred from
-0.06167	1700018B2	enhancer of rudiment	0007049 // cell cycle // inferred from electronic annotation		
0.19481	Sppl3	signal peptide peptida	0008152 /,	0016020 /,	0004190 // aspartic-type endopeptidase acti
-0.17932	2510039O1	RIKEN cDNA 2510039O18 gene		0016020 // membrane // inferred from electronic anno	
0.129236	Cnot8	CCR4-NOT transcriptic	0006350 /,	0005622 /,	0003676 // nucleic acid binding // inferred fı
0.085526	Vash2	vasohibin 2	0001938 /,	0005737 // cytoplasm // not recorded	
-0.0667	Pbxip1	pre-B-cell leukemia transcription		0005634 /,	0005515 // protein binding // inferred from
-0.09039	Trip12	thyroid hormone rece	0006464 /,	0005622 /,	0005488 // binding // inferred from electror
0.022135	Tmem121	transmembrane protein 121		0016020 // membrane // inferred from electronic anno	
-0.08585	Wdr26	WD repeat domain 26		0005737 // cytoplasm // inferred from electronic annot	
-0.04531	Wdr20a	WD repeat domain 20A			
-0.02706	Vcam1	vascular cell adhesion	0001666 /,	0005615 /,	0005178 // integrin binding // not recorded
-0.22252	Mtap	methylthioadenosine	0009116 /,	0005737 /,	0003824 // catalytic activity // inferred from

-0.25341	Mtap	methylthioadenosine	0009116 /,	0005737 /,	0003824 // catalytic activity // inferred from	
-0.27859	Acer2	alkaline ceramidase 2	0001953 /,	0000139 /,	0016787 // hydrolase activity // inferred fro	
0.195672	Lpcat1	lysophosphatidylcholi	0008152 /,	0000139 /,	0005509 // calcium ion binding // inferred fr	
-0.32349	Pnpla7	patatin-like phospholi	0006629 /,	0005634 /,	0004091 // carboxylesterase activity // not r	
-0.10342	Rab22a	RAB22A, member RAS	0006810 /,	0005768 /,	0000166 // nucleotide binding // inferred fro	
-0.003	Spsb4	splA/ryanodine recep	0023034 /,	0005737 /,	0005515 // protein binding // inferred from	
-0.23937	Zdhhc20	zinc finger, DHHC dom	0008152 /,	0016020 /,	0008270 // zinc ion binding // inferred from	
-0.04537	Rgs16	regulator c Calcium_re	0007186 /,	0005624 /,	0004871 // signal transducer activity // infer	
-0.35703	Angptl7	angiopoietin-like 7	0007165 /,	0005576 /,	0005102 // receptor binding // inferred from	
-0.04603	Lyplal1	lysophospholipase-like	0008152 /,	0005737 /,	0016787 // hydrolase activity // inferred fro	
-0.42637	Mef2c	myocyte enhancer fac	0001568 /,	0005634 /,	0003677 // DNA binding // inferred from dir	
-0.03395	Fam116a	family with sequence similarity 116, member A				
-0.06268	Spsb2	splA/ryanodine recep	0023034 /,	0005737 // cytoplasm // inferred from electronic annot		
0.121956	Efnb1	ephrin B1	0001755 /,	0016020 /,	0005515 // protein binding // inferred from	
-0.47353	Mafb	v-maf musculoaponeu	0006350 /,	0005634 /,	0003677 // DNA binding // inferred from dir	
-0.03122	Slu7	SLU7 splicing factor h	0000375 /,	0005634 /,	0000386 // second spliceosomal transesterif	
-0.19978	Ogt	O-linked N-acetylgluc	0006493 /,	0005622 /,	0003824 // catalytic activity // inferred from	
-0.18752	Hmgcr	3-hydroxy- Cholestero	0006694 /,	0005777 /,	0004420 // hydroxymethylglutaryl-CoA redu	
0.151314	Pcdha1 ///	protocadherin alpha 1	0007155 /,	0005624 /,	0004872 // receptor activity // inferred from	
-0.04148	Polr3f	polymerase (RNA) III (	0006350 /,	0005634 /,	0003677 // DNA binding // inferred from ele	
-0.30977	Klhl24	kelch-like 24 (Drosophila)		0005737 /,	0005515 // protein binding // inferred from	
-0.18612	Smg5	Smg-5 homolog, nons	0000184 /,	0005634 /,	0005515 // protein binding // inferred from	
-0.034	Tc2n	tandem C2 domains, nuclear		0005634 // nucleus // inferred from direct assay /// 000		
-0.07677	Cryz	crystallin, zeta	0008152 /,	0005625 /,	0003723 // RNA binding // inferred from ele	
-0.05515	Eda	ectodysplasin-A	0001942 /,	0005576 /,	0005102 // receptor binding // inferred from	
0.274447	Loxl1	lysyl oxidase-like 1	0055114 /,	0005576 /,	0005507 // copper ion binding // inferred fro	
-0.0821	Glyr1	glyoxylate reductase 1	0006098 /,	0005634 /,	0003677 // DNA binding // inferred from ele	
-0.24287	Mgat2	mannoside acetylgluc	0006487 /,	0000139 /,	0005529 // sugar binding // not recorded //,	
0.171672	Zcchc17	zinc finger, mRNA_processing_bi		0005634 /,	0003676 // nucleic acid binding // inferred fr	
-0.50729	Epsti1	epithelial stromal interaction 1 (breast)				
0.267279	Ddx17	DEAD (Asp-Glu-Ala-As	0006200 /,	0005634 /,	0000166 // nucleotide binding // inferred fro	
-0.08904	2310001A2	RIKEN cDNA 2310001,	0009058 /,	0009986 /,	0004064 // arylesterase activity // inferred f	
-0.4056	Ets1	E26 avian leukemia or	0001666 /,	0005634 /,	0003677 // DNA binding // inferred from dir	
-0.09116	Maml1	mastermind like 1 (Dr	0006350 /,	0005634 /,	0003713 // transcription coactivator activity	
-0.04434	Tmem209	transmembrane protein 209		0016020 // membrane // inferred from electronic anno		
-0.09623	Rcc2	regulator of chromoso	0007049 /,	0000775 // chromosome, centromeric region // inferre		
-0.08534	Tbc1d23	TBC1 domain family, r	0032313 /,	0005622 /,	0005097 // Rab GTPase activator activity // i	
-0.05722	6330406I1	RIKEN cDNA 6330406I15 gene				
0.064293	Las1l	LAS1-like (S. cerevisiae)		0005634 /,	0005515 // protein binding // inferred from	
0.286247	Msi1	Musashi h mRNA_pro	0009725 /,	0005634 /,	0000166 // nucleotide binding // inferred fro	
0.068141	Iqsec1	IQ motif and Sec7 dor	0032012 /,	0005622 /,	0005085 // guanyl-nucleotide exchange fact	
-0.15883	Mfsd8	major facilitator supe	0006810 /,	0005764 // lysosome // inferred from electronic annota		
-0.09529	Adamts7	a disintegrin-like and	0006508 /,	0005576 /,	0004222 // metalloendopeptidase activity //,	
-0.86248	Lmod2	leiomodin 2 (cardiac)		0005856 /,	0003779 // actin binding // inferred from ele	
-0.22337	Ifi205 /// N	interferon activated g	0006350 /,	0005634 /,	0005515 // protein binding // inferred from	
-0.12506	Parp4	poly (ADP-ribose) poly	0006471 /,	0005622 /,	0003950 // NAD+ ADP-ribosyltransferase act	
-0.20276	Gpr155	G protein-coupled rec	0007165 /,	0016021 /,	0004872 // receptor activity // inferred from	

0.419087	Gp1bb ///	glycoprotein Ib, beta	0007049 /,	0001725 /,	0000166 // nucleotide binding // inferred fro	
-0.27626	Rell1	RELT-like 1		0016020 // membrane // inferred from electronic anno		
-0.12603	Kdm5a	lysine (K)-specific dem	0006350 /,	0005622 /,	0003677 // DNA binding // inferred from ele	
0.283589	Epha7	Eph receptor A7	0006468 /,	0005887 /,	0000166 // nucleotide binding // inferred fro	
0.082884	Amotl2	angiomotin-like 2		0005923 /,	0005515 // protein binding // inferred from	
-0.32618	Phf3	PHD finger protein 3	0006350 /,	0005634 /,	0005515 // protein binding // inferred from	
0.112673	Atp8b2	ATPase, class I, type 8	0006200 /,	0016020 /,	0000166 // nucleotide binding // inferred fro	
-0.39077	Cep350	centrosomal protein 350				
0.024068	Plcb2	phospholipase C, beta	0001580 /,	0005794 /,	0004435 // phosphoinositide phospholipase	
-0.03617	Kit	kit oncogene	0002318 /,	0001669 /,	0000166 // nucleotide binding // inferred fro	
0.406841	Adamts4	a disintegrin-like and	0006508 /,	0005576 /,	0002020 // protease binding // inferred fron	
-0.08429	Polr2k	polymeras RNA_trans	0006350 /,	0005634 /,	0003677 // DNA binding // inferred from ele	
-0.12507	Taf1d	TATA box binding pro	0006350 /,	0005634 /,	0003677 // DNA binding // inferred from ele	
0.053518	Gtpbp5	GTP binding protein 5		0005622 /,	0000166 // nucleotide binding // inferred fro	
0.205965	Tex261	testis expressed gene	0043065 /,	0016020 // membrane // inferred from electronic anno		
-0.40336	Tnks2	tankyrase, TRF1-inter	0000723 /,	0000242 /,	0003950 // NAD+ ADP-ribosyltransferase act	
0.22955	Cad	carbamoyl-phosphate	0006207 /,	0005625 /,	0002134 // UTP binding // not recorded ///	
-0.24127	Ppat	phosphoribosyl pyrop	0006164 /,	0005625 /,	0004044 // amidophosphoribosyltransferase	
0.134668	Med12l	mediator of RNA poly	0006350 /,	0005634 /,	0016455 // RNA polymerase II transcription	
-0.18244	Gtf2h1	general tra RNA_trans	0006281 /,	0005634 /,	0004672 // protein kinase activity // inferrec	
-0.11637	Plscr1	phospholipid scrambl	0006955 /,	0016020 /,	0017124 // SH3 domain binding // inferred f	
-0.115	2700089E2	RIKEN cDNA 2700089E24 gene				
-0.08747	Pik3cd	phosphatic G13_Signa	0001782 /,	0005942 /,	0000166 // nucleotide binding // inferred fro	
0.176069	Sec14l1	SEC14-like 1 (S. cerevisiae)				
0.133523	Fxc1	fractured callus expre	0006626 /,	0005739 /,	0046872 // metal ion binding // inferred fron	
-2.66E-04	Mbd1	methyl-CpG binding d	0006306 /,	0000785 /,	0003677 // DNA binding // inferred from dir	
-0.15268	4921524J1	RIKEN cDNA 4921524J17 gene				
0.103072	Pi4k2a	phosphatidylinositol 4	0002561 /,	0005737 /,	0000166 // nucleotide binding // inferred fro	
0.060629	Ubap2l	ubiquitin associated protein 2-like			0005515 // protein binding // inferred from	
-0.01123	Rere	arginine glutamic acid	0006338 /,	0000118 /,	0003677 // DNA binding // inferred from ele	
0.174723	Thra	thyroid ho Nuclear_R	0001502 /,	0005634 /,	0002153 // steroid receptor RNA activator R	
-0.04739	Pten	phosphatase and tens	0001525 /,	0005634 /,	0000287 // magnesium ion binding // inferre	
0.004461	Klhdc3	kelch domain contain	0007126 /,	0000790 /,	0003682 // chromatin binding // inferred fro	
0.035158	Dad1	defender against cell	0001824 /,	0016020 /,	0004579 // dolichyl-diphosphooligosaccharic	
-0.86965	Upk3b	uroplakin 3B	0010629 /,	0005886 // plasma membrane // inferred from electror		
-0.2779	Sbno1	sno, strawl mRNA_pro	0008152 // metabolic		0003677 // DNA binding // inferred from ele	
-0.31923	Ptprj	protein tyrosine phos	0001570 /,	0005634 /,	0004721 // phosphoprotein phosphatase ac	
-0.20863	Fbxo3	F-box protein 3		0005634 // nucleus // inferred from electronic annotati		
-0.00495	Usp19	ubiquitin specific pep	0006511 /,	0005783 /,	0004221 // ubiquitin thiolesterase activity //	
0.107803	Btbd1	BTB (POZ) domain containing 1	0000932 /,	0005515 // protein binding // inferred from		
-0.2171	Ube2d3	ubiquitin-conjugating	0000209 /,	0005768 /,	0000166 // nucleotide binding // inferred fro	
0.085954	Hnrnph3	heterogeneous nuclear ribonucleoprotein H			0000166 // nucleotide binding // inferred fro	
0.146447	Diras1	DIRAS family, GTP-bin	0006184 /,	0005622 /,	0000166 // nucleotide binding // inferred fro	
-0.22556	Osbpl11	oxysterol binding prot	0006810 // transport // inferred from electronic annotation /// 00			
-0.1322	Notch2	Notch gene homolog	0001709 /,	0005634 /,	0004872 // receptor activity // inferred from	
0.027695	H13	histocompatibility 13	0008152 /,	0005783 /,	0004190 // aspartic-type endopeptidase act	
-0.20718	Clcnka	chloride channel Ka	0001822 /,	0005783 /,	0005216 // ion channel activity // inferred fr	

0.214545	Taok3	TAO kinase 3		0000165 /,	0005737 /,	0000166 // nucleotide binding // inferred fro	
-0.12009	Prkcc	protein kin	Calcium_re	0006468 /,	0005634 /,	0000166 // nucleotide binding // inferred fro	
0.11588	Usp14	ubiquitin specific pept		0006511 /,	0000502 /,	0004221 // ubiquitin thiolesterase activity //	
0.029059	Snx17	sorting nexin 17		0006810 /,	0005737 /,	0005515 // protein binding // inferred from	
0.203521	Adamts4	a disintegrin-like and		0006508 /,	0005576 /,	0002020 // protease binding // inferred from	
0.00701	Gm13654 /	predicted	mRNA_prc	0000028 /,	0005622 /,	0003735 // structural constituent of ribosom	
0.046115	Slc35a4	solute carrier family 3		0006810 /,	0000139 /,	0005351 // sugar:hydrogen symporter activi	
-0.21257	Secisbp2l	SECIS binding protein 2-like					
0.164886	Calr	calreticulir	Calcium_re	0000122 /,	0001669 /,	0003729 // mRNA binding // inferred from d	
0.016663	Sumo1	SMT3 suppressor of m		0006950 /,	0005634 /,	0005515 // protein binding // inferred from	
0.148418	Rpn1	ribophorin	Proteasom	0006486 /,	0005783 /,	0004579 // dolichyl-diphosphooligosacchari	
-0.24645	Wdr33	WD repeat domain 33			0005634 /,	0005515 // protein binding // inferred from	
-0.15567	Prokr1	prokinetici	GPCRDB_C	0006916 /,	0005886 /,	0004871 // signal transducer activity // infer	
0.28802	Fam101b	family with sequence similarity 101, member B					
-0.26034	Chst11 ///	carbohydrate sulfotra		0002063 /,	0000139 /,	0001537 // N-acetylgalactosamine 4-O-sulfo	
0.10608	Mat2a	methionine adenosylt		0006556 /,	0005737 /,	0000166 // nucleotide binding // inferred fro	
-0.18133	Serpinh1	serine (or cysteine) pe		0006950 /,	0005737 /,	0004867 // serine-type endopeptidase inhib	
-0.10976	Synrg	synergin, gamma		0006810 /,	0005737 /,	0005509 // calcium ion binding // inferred fr	
-0.36771	Enpp1	ectonucleotide pyropl		0006955 /,	0005615 /,	0003676 // nucleic acid binding // inferred fr	
-0.057	Mir425	microRNA 425					
0.049215	Ero1l /// L(	ERO1-like (	Ribosomal	0006412 /,	0005622 /,	0003735 // structural constituent of ribosom	
-0.16073	Pou3f1	POU domain, class 3,		0006350 /,	0005634 /,	0003677 // DNA binding // inferred from dir	
-0.14461	Phyh	phytanoyl-CoA hydro›		0001561 /,	0005739 /,	0003824 // catalytic activity // inferred from	
-0.28398	Tec	tec protein tyrosine ki		0006468 /,	0005737 /,	0000166 // nucleotide binding // inferred fro	
-0.23799	Grasp	GRP1 (general recepto		0006886 /,	0005641 /,	0005515 // protein binding // inferred from	
0.002928	Pkd1	polycystic kidney dise		0001502 /,	0002133 /,	0005261 // cation channel activity // inferre	
-0.16013	Snx2	sorting nexin 2		0006810 /,	0005737 /,	0005515 // protein binding // inferred from	
0.06674	Mrp63	mitochondrial ribosor		0006412 /,	0005739 /,	0003735 // structural constituent of ribosom	
0.184711	Msln	mesothelin		0007155 /,	0005576 // extracellular region // inferred from electro		
-0.16725	Sptlc2	serine palmitoyltransf		0006686 /,	0005739 /,	0003824 // catalytic activity // inferred from	
0.194613	Acsl1	acyl-CoA s	Fatty_Acid	0006629 /,	0005625 /,	0000166 // nucleotide binding // inferred fro	
5.56E-04	Gpr88	G-protein (	GPCRDB_C	0007165 /,	0005886 /,	0004871 // signal transducer activity // infer	
-0.16253	Ythdf2	YTH domain family 2					
0.270834	Top1mt	DNA topoisomerase 1		0006265 /,	0005694 /,	0000166 // nucleotide binding // inferred fro	
-0.08987	Sorl1	sortilin-related recept		0006629 /,	0005771 /,	0004872 // receptor activity // inferred from	
-0.06413	Egfr	epidermal growth fact		0000186 /,	0005622 /,	0000166 // nucleotide binding // inferred fro	
0.182399	Ubp1	upstream binding pro		0001525 /,	0005634 /,	0003677 // DNA binding // inferred from ele	
-0.07768	Paqr7	progestin and adipoQ		0007165 /,	0005886 /,	0004872 // receptor activity // inferred from	
-0.10838	Tm7sf2	transmembrane 7 sup		0006694 /,	0005783 /,	0016491 // oxidoreductase activity // inferre	
0.169237	Unc119b	unc-119 homolog B (C. elegans)					
-0.0631	2010111I0	RIKEN cDNA 2010111I		0006508 /,	0005737 /,	0004177 // aminopeptidase activity // inferr	
-0.11182	BC026585	cDNA sequence BC02(		0008152 /,	0005739 /,	0003824 // catalytic activity // inferred from	
0.121363	Ap1g1	adaptor protein comp		0000226 /,	0005794 /,	0005488 // binding // inferred from electror	
0.15682	Unc119b	unc-119 homolog B (C. elegans)					
-0.12987	Cltb	clathrin, light polypep		0006886 /,	0005905 /,	0005198 // structural molecule activity // inf	
-0.12794	P2rx7	purinergic receptor P2		0000187 /,	0005639 /,	0000287 // magnesium ion binding // not re	
0.031783	Arnt	aryl hydrocarbon rece		0001666 /,	0005634 /,	0003677 // DNA binding // inferred from ger	

-0.0182	Gm9112	predicted gene 9112			
-0.04728	Ly6g6c	Lymphocyte antigen 6 complex, l	0005886 // plasma membrane // inferred from electror		
-0.27072	S1pr2	sphingosin GPCRDB_C	0007165 /,	0005886 /,	0001619 // lysosphingolipid and lysophosph
0.124141	Slc25a1	solute carrier family 2	0006810 /,	0005739 /,	0005215 // transporter activity // inferred fr
0.278348	Adal	adenosine deaminase	0009117 // nucleotide		0004000 // adenosine deaminase activity //
0.251226	5730419I0	RIKEN cDNA 5730419I09 gene			
-0.09586	Cpsf6	cleavage and polyade	0006397 /,	0005634 /,	0000166 // nucleotide binding // inferred fro
0.110302	Ccdc134	coiled-coil domain containing 13		0005576 // extracellular region // inferred from electro	
0.167082	Trub1	TruB pseudouridine (p	0001522 // pseudouri		0003723 // RNA binding // inferred from ele
0.153381	8430427H	RIKEN cDNA 8430427H17 gene			
0.070126	Zswim3	zinc finger, SWIM domain containing 3			0008270 // zinc ion binding // inferred from
-0.19431	Pik3ip1	phosphoinositide-3-kinase intera		0016020 // membrane // inferred from electronic anno	
0.140542	Agpat4	1-acylglycerol-3-phos	0008152 /,	0016020 /,	0003841 // 1-acylglycerol-3-phosphate O-ac
0.208934	Arrdc2	arrestin domain conta	0007165 // signal transduction // inferred from electronic annotati		
0.442231	Acsl3	acyl-CoA s Fatty_Acid	0006629 /,	0005739 /,	0000166 // nucleotide binding // inferred fro
0.22556	Nrn1	neuritin 1	0007399 /,	0005886 // plasma membrane // inferred from electror	
0.221105	Fbrsl1	fibrosin-like 1			
0.213761	Asb2	ankyrin repeat and SC	0023034 // intracellular signaling pathway // inferred from electro		
-0.23537	Coq10b	coenzyme Q10 homolog B (S. cer		0005739 // mitochondrion // inferred from direct assay	
0.087928	Sipa1l3	signal-induced proliferation-associated 1 like			0005515 // protein binding // inferred from
0.177823	Secisbp2	SECIS binding protein 2		0005739 /,	0003723 // RNA binding // not recorded ///
0.216197	2810454L2	RIKEN cDNA 2810454L23 gene			
-0.30882	2210403K0	RIKEN cDNA 2210403K04 gene			
-0.08413	Fmnl2	formin-like 2	0016043 /,	0005737 /,	0003779 // actin binding // inferred from ele
0.194664	Pbx1	pre B-cell leukemia tr	0001655 /,	0005634 /,	0003677 // DNA binding // inferred from dir
0.069815	Fam173b	family with sequence similarity 1		0016020 // membrane // inferred from electronic anno	
0.281201	Prickle2	prickle homolog 2 (Dr	0031175 /,	0005634 /,	0005515 // protein binding // inferred from
-0.4167	Wfikkn2	WAP, follistatin/kazal,	0007179 /,	0005576 /,	0004857 // enzyme inhibitor activity // infer
-0.21455	Tmem57	transmembrane protein 57		0005634 // nucleus // inferred from direct assay /// 001	
0.059796	Rbbp5	retinoblastoma bindir	0006350 /,	0005634 /,	0005515 // protein binding // inferred from
-0.07133	Alkbh3	alkB, alkylation repair	0006281 /,	0005634 /,	0004519 // endonuclease activity // inferred
-0.09978	Paqr8	progestin and adipoQ	0007165 /,	0005886 /,	0004872 // receptor activity // inferred from
0.07196	Enkur	enkurin, TRPC channel interactin		0005929 /,	0005516 // calmodulin binding // inferred fr
-0.07808	Mtf1	metal response eleme	0006350 /,	0005622 /,	0003676 // nucleic acid binding // inferred fr
-0.15142	Dynlrb2	dynein light chain roa	0006810 /,	0005737 /,	0003774 // motor activity // inferred from e
0.102289	Pdp2	pyruvate dehyrogenas	0006470 /,	0005739 /,	0003824 // catalytic activity // inferred from
0.175753	Hexim2	hexamthylene bis-ace	0000122 /,	0005634 /,	0004861 // cyclin-dependent protein kinase
0.045913	1600002K0	RIKEN cDNA 1600002K03 gene		0005737 // cytoplasm // inferred from electronic annot	
-0.19632	Ddi2	DNA-damage inducibl	0006508 // proteolysi		0004190 // aspartic-type endopeptidase acti
-0.15864	Duxbl /// G	double homeobox B-li	0006355 /,	0005634 /,	0003677 // DNA binding // inferred from ele
-0.11149	2010109K1	RIKEN cDNA 2010109K11 gene			
-0.74263	Gvin1	GTPase, very large interferon ind		0005634 /,	0000166 // nucleotide binding // inferred fro
-0.02341	Klk5	kallikrein related-pep	0006508 // proteolysi		0003824 // catalytic activity // inferred from
-0.14774	Ror1	receptor tyrosine kina	0006468 /,	0005887 /,	0000166 // nucleotide binding // inferred fro
-0.02694	Ror1	receptor tyrosine kina	0006468 /,	0005887 /,	0000166 // nucleotide binding // inferred fro
0.20692	Syt11	synaptotagmin XI	0006810 /,	0005887 /,	0005215 // transporter activity // inferred fr
0.074938	Cox10	COX10 homolog, cyto	0000266 /,	0005739 /,	0004129 // cytochrome-c oxidase activity //

-0.15141	Tmem87b	transmembrane protein 87B		0016020 // membrane // inferred from electronic anno	
-0.27416	Sema3c	sema domain, immun	0001755 /,	0005576 /,	0004872 // receptor activity // inferred from
-0.08621	Klhl24	kelch-like 24 (Drosophila)		0005737 /,	0005515 // protein binding // inferred from
-0.16819	Dcaf12	DDB1 and CUL4 associated factor		0005737 // cytoplasm // inferred from electronic annot	
-0.16802	Pik3ca	phosphatic	G13_Signa 0006006 /,	0005942 /,	0000166 // nucleotide binding // inferred fro
0.120503	Rasl11a	RAS-like, family 11, m	0006184 /,	0005634 /,	0000166 // nucleotide binding // inferred fro
0.032334	Gapvd1	GTPase activating pro	0006897 /,	0005622 /,	0005085 // guanyl-nucleotide exchange fact
-0.21243	Prkaa2	protein kinase, AMP-a	0006468 /,	0005634 /,	0000166 // nucleotide binding // inferred fro
-0.15182	Sfrs18	serine/arginine-rich splicing facto		0005634 // nucleus // inferred from electronic annotati	
-0.04047	Bcl2l13	BCL2-like 13 (apoptos	0006915 /,	0005739 // mitochondrion // inferred from direct assay	
-0.06203	Entpd8	ectonucleoside tripho	0008152 /,	0005624 /,	0000166 // nucleotide binding // inferred fro
-0.15848	Ms4a13	membrane-spanning 4	0007165 /,	0016020 /,	0004872 // receptor activity // inferred from
-0.19931	Slc35a3	solute carrier family 3	0006810 /,	0000139 /,	0005338 // nucleotide-sugar transmembrane
-0.1363	Lrrc17	leucine rich repeat co	0001503 /,	0005576 /,	0005515 // protein binding // inferred from
0.056178	2810046L0	RIKEN cDNA 2810046L04 gene			
0.028161	Apool	apolipoprotein O-like		0005576 // extracellular region // inferred from electro	
0.153116	4930553P1	RIKEN cDNA 4930553P18 gene			
-0.09256	Txnrd2	thioredoxin reductase	0000305 /,	0005737 /,	0004791 // thioredoxin-disulfide reductase a
-0.12697	5730455O	RIKEN cDNA 5730455O13 gene		0005634 /,	0005515 // protein binding // inferred from
0.045137	Hyal4	hyaluronoglucosamini	0005975 /,	0016020 /,	0003824 // catalytic activity // inferred from
-0.05102	Wdr20b	WD repeat domain 20b			
0.161569	Cct4	chaperonin containing	0006457 /,	0005737 /,	0000166 // nucleotide binding // inferred fro
-0.00932	2310015B2	RIKEN cDNA 2310015B20 gene			
0.177108	Ttll11	tubulin tyrosine ligase	0006464 /,	0005737 /,	0004835 // tubulin-tyrosine ligase activity //
-0.00179	Phf15	PHD finger protein 15	0043966 // histone H3		0005515 // protein binding // inferred from
-0.11313	Commd8	COMM domain containing 8			
-0.14106	Cstad	CSA-conditional, T cel	0007006 /,	0005739 // mitochondrion // inferred from direct assay	
0.032047	Krt42	keratin 42		0005737 /,	0005198 // structural molecule activity // inf
0.064277	Gpr137c	G protein-coupled rec	0007165 // signal trar		0004872 // receptor activity // inferred from
0.051881	Klf5	Kruppel-like factor 5	0001525 /,	0005622 /,	0003676 // nucleic acid binding // inferred fr
0.023323	9630005C1	RIKEN cDNA 9630005C17 gene			
-0.05464	1810019N2	RIKEN cDNA 1810019N24 gene			
0.017931	Tmem29	transmembrane protein 29		0016021 // integral to membrane // inferred from elect	
-0.04002	4931431B1	RIKEN cDNA 4931431B13 gene			
-0.12616	1700020A2	RIKEN cDNA 1700020A23 gene			
-0.00553	Aff3	AF4/FMR2 family, me	0006350 /,	0005634 /,	0003677 // DNA binding // inferred from ele
-0.03348	Nos1ap	nitric oxide synthase 1 (neuronal) adaptor pr			0005515 // protein binding // inferred from
-0.14246	Cyp20a1	cytochrome P450, fan	0055114 /,	0016020 /,	0004497 // monooxygenase activity // inferr
-0.09563	Aasdhppt	aminoadipate-semialc	0009059 /,	0005737 /,	0000287 // magnesium ion binding // inferre
-0.29667	Rnase6	ribonuclease, RNase A	0008152 // metabolic		0003676 // nucleic acid binding // inferred fr
0.047139	Haus2	HAUS augmin-like con	0007049 /,	0005737 // cytoplasm // inferred from electronic annot	
-0.01	4930524L2	RIKEN cDNA 4930524L23 gene			
-0.15161	Gsdmc2 //,	gasdermin C2 /// gasdermin C4			
0.033953	Scn2b	sodium channel, volta	0006810 /,	0016020 /,	0005216 // ion channel activity // inferred fr
-0.17179	Krtap5-5	keratin associated protein 5-5		0005882 // intermediate filament // inferred from elect	
-0.13811	Myg1	melanocyte proliferating gene 1		0005634 // nucleus // inferred from electronic annotati	
-0.0957	1810063B0	RIKEN cDNA 1810063B07 gene		0016020 // membrane // inferred from electronic anno	

-0.03528	5430427G:	RIKEN cDNA 5430427G11 gene				
-0.02767	Prune2	prune homolog 2 (Drc	0006915 /,	0005737 /,	0016462 // pyrophosphatase activity // infer	
-0.15118	1110002E2	RIKEN cDNA 1110002E22 gene				
-0.05414	Klhl22	kelch-like 22 (Drosophila)			0005515 // protein binding // inferred from	
-0.05142	Pex11c	peroxisomal biogenesis factor 11		0005777 // peroxisome // inferred from electronic anno		
-0.19457	Plcb4	phospholipase C, beta	0006629 /,	0005626 /,	0004435 // phosphoinositide phospholipase	
0.094989	0610038B2	RIKEN cDNA 0610038B21 gene				
-0.88414	Cd47	CD47 antigen (Rh-rela	0007155 /,	0005886 /,	0005515 // protein binding // inferred from	
-0.06572	Chd6	chromodomain helica	0006333 /,	0000785 /,	0003676 // nucleic acid binding // inferred fr	
-0.14863	Ptar1	protein prenyltransferase alpha subunit repeat containing 1				
0.291054	Cpsf2	cleavage a mRNA_prc	0006397 /,	0005634 /,	0003723 // RNA binding // inferred from ele	
-0.41016	1700018L0	RIKEN cDNA 1700018L02 gene				
-0.06169						
0.031089	Dido1	death inducer-oblitera	0006350 /,	0005634 /,	0005515 // protein binding // inferred from	
0.199026	2810417H1	RIKEN cDNA 2810417H13 gene		0005634 // nucleus // inferred from electronic annotati		
-0.16439	4933436C2	RIKEN cDNA 4933436C20 gene				
0.102912	Lztfl1	leucine zipper transcription factor-like 1				
-0.27559	Cml3	camello-like 3	0001702 /,	0005615 /,	0008080 // N-acetyltransferase activity // in	
-0.18404	3110005L2	RIKEN cDNA 3110005L24 gene				
-0.04324	Fyttd1	forty-two-three doma	0006406 /,	0005634 /,	0003723 // RNA binding // inferred from ele	
-0.14713	2310069B0	RIKEN cDNA 2310069B03 gene				
0.017838						
-0.04777	Cdhr4	cadherin-related fami	0007156 /,	0016020 /,	0005509 // calcium ion binding // inferred fr	
0.039851	4930422C2	RIKEN cDNA 4930422C21 gene				
0.051704	4930432N1	RIKEN cDNA 4930432N10 gene				
0.035831	5830453K1	RIKEN cDNA 5830453K13 gene				
-0.10306	4933402J1	RIKEN cDNA 4933402J15 gene				
0.10541	Tmem86b	transmembrane protein 86B		0016020 // membrane // inferred from electronic anno		
0.108645	5031425E2	RIKEN cDNA 5031425E22 gene				
0.143573	Gsdma2	gasdermin A2				
-0.14245	1700028J1	RIKEN cDNA 1700028J19 gene				
-0.18952	6720483E2	RIKEN cDNA 6720483E21 gene				
0.150901	Meaf6	MYST/Esa1-associatec	0006350 /,	0005634 // nucleus // inferred from electronic annotati		
0.034606	4930564B1	RIKEN cDNA 4930564B18 gene				
0.075653	Tmsb15a	thymosin beta 15a	0007010 /,	0005737 /,	0003779 // actin binding // inferred from ele	
-0.31248	Mll5	myeloid/lymphoid or	0002446 /,	0005634 /,	0003713 // transcription coactivator activity	
0.019772	5830415G2	RIKEN cDNA 5830415G21 gene				
-0.03226	6720420G1	RIKEN cDNA 6720420G18 gene				
-0.01363	Galnt11	UDP-N-acetyl-alpha-D	0008152 /,	0000139 /,	0004653 // polypeptide N-acetylgalactosami	
-0.00243	2900057C0	RIKEN cDNA 2900057C01 gene				
-0.03949						
0.010519	3110037C0	RIKEN cDNA 3110037C07 gene				
0.016801	4933428L0	RIKEN cDNA 4933428L01 gene				
-0.1011	6430514K0	RIKEN cDNA 6430514K02 gene				
-0.08565	5033425B0	RIKEN cDNA 5033425B01 gene				
-0.06901	4930563F1	RIKEN cDNA 4930563F15 gene				
0.001778	4930566N2	RIKEN cDNA 4930566N20 gene				

-0.15806	4933417G(	RIKEN cDNA 4933417G07 gene			
-0.24598	4930474A2	RIKEN cDNA 4930474A20 gene			
-0.04771	4921504P1	RIKEN cDNA 4921504P13 gene			
0.168465	Radil	Ras association and D	0007155 // cell adhes		0005515 // protein binding // inferred from
0.16517	Gm15800	predicted gene 15800	0006464 /,	0005622 /,	0016881 // acid-amino acid ligase activity //
-0.10645	Eaf1	ELL associated factor :	0006350 /,	0005634 /,	0005515 // protein binding // inferred from
-0.10498	Acap2	ArfGAP with coiled-co	0030029 /,	0001726 /,	0005096 // GTPase activator activity // infer
0.050483	Adck2	aarF domain containir	0008152 /,	0005739 /,	0000166 // nucleotide binding // inferred frc
0.268957	Fam20a	family with sequence similarity 2		0005576 // extracellular region // inferred from electro	
-0.1438	Fam78a	family with sequence similarity 78, member A			
0.122461	Nat10	N-acetyltransferase 1(	0008152 /,	0005634 /,	0000166 // nucleotide binding // inferred frc
0.048209	Entpd5	ectonucleoside tripho	0008152 /,	0005783 /,	0016787 // hydrolase activity // inferred fron
0.16214	Agpat3	1-acylglycerol-3-phos|	0008152 /,	0005783 /,	0003841 // 1-acylglycerol-3-phosphate O-ac
0.246468	Agpat3	1-acylglycerol-3-phos|	0008152 /,	0005783 /,	0003841 // 1-acylglycerol-3-phosphate O-ac
0.036006	Cdc27	cell division cycle 27 h	0007091 /,	0005634 /,	0005488 // binding // inferred from electror
0.149205	Cc2d1b	coiled-coil and C2 domain containing 1B			
-0.36785	Btbd3	BTB (POZ) domain containing 3			0005515 // protein binding // inferred from
-0.10277	Akap7	A kinase (P G_Protein_	0007178 /,	0005622 /,	0003824 // catalytic activity // inferred from
0.035829	Uspl1	ubiquitin specific pept	0006511 // ubiquitin-(		0004221 // ubiquitin thiolesterase activity //
-0.29463	Slco2b1	solute carrier organic	0006810 /,	0009925 /,	0005215 // transporter activity // inferred fr
-0.06412	Itprip	inositol 1,4,5-triphosphate recep		0005886 // plasma membrane // inferred from electror	
-0.15666	Sephs1	selenophosphate synt	0008152 // metabolic		0000166 // nucleotide binding // inferred frc
0.122919	Foxn3	forkhead box N3	0006350 /,	0005634 /,	0003677 // DNA binding // inferred from ele
-0.11542	Mtmr12	myotubularin related	0016311 /,	0005737 /,	0016791 // phosphatase activity // inferred
-0.16059	Wdr37	WD repeat domain 37			
0.147152	Kctd13	potassium channel tet	0006260 /,	0005634 /,	0004842 // ubiquitin-protein ligase activity /
0.118765	Rab11fip4	RAB11 family interact	0000910 /,	0005768 /,	0005509 // calcium ion binding // inferred fr
-0.044	Lin52	lin-52 hom Smooth_muscle_contraction // GenMAPP /// Striated_muscle_contraction //			
0.119636	Alg11	asparagine-linked glyc	0009058 /,	0016020 /,	0016740 // transferase activity // inferred fr
-0.35078	Mical3	microtubule associate	0008152 /,	0005737 /,	0004497 // monooxygenase activity // inferr
-0.35846	Swap70	SWA-70 protein	0016444 /,	0005634 /,	0003677 // DNA binding // inferred from dir
-0.16081	Frmd4a	FERM domain containing 4A		0005737 /,	0005488 // binding // inferred from electror
-0.21355	Nus1	nuclear undecaprenyl	0001525 /,	0016020 /,	0004872 // receptor activity // inferred from
-0.01459	Gpkow	G patch domain and KOW motifs		0005622 /,	0003676 // nucleic acid binding // inferred fı
-0.01371	Maob	monoamine oxidase E	0009636 /,	0005739 /,	0008131 // primary amine oxidase activity /,
-0.19719	1700008K2	RIKEN cDNA 1700008K24 gene			
-0.08543	Heca	headcase homolog (Drosophila)			
-0.29178	Pdpr	pyruvate dehydrogen	0006546 /,	0005737 /,	0004047 // aminomethyltransferase activity
0.075424	6430548M	RIKEN cDNA 6430548M08 gene		0005634 // nucleus // inferred from electronic annotati	
-0.18781	Eif5a2	eukaryotic translation	0006412 /,	0005634 /,	0003723 // RNA binding // inferred from ele
-0.20501	Tmem204	transmembrane prote	0001945 /,	0005886 /,	0005515 // protein binding // inferred from
-0.1529	Ankrd28	ankyrin repeat domain 28		0005634 /,	0005515 // protein binding // inferred from
0.068993	Ralgapb	Ral GTPase activating	0032859 /,	0005622 /,	0005096 // GTPase activator activity // infer
0.1504	Vhl	von Hippel-Lindau tur	0000122 /,	0005634 /,	0005515 // protein binding // inferred from
0.390126	Fzd3	frizzled ho Wnt_Signa	0001736 /,	0005886 /,	0004871 // signal transducer activity // infer
-0.33572	Rprd2	regulation of nuclear pre-mRNA domain con			0005515 // protein binding // inferred from
-0.14268	Tdrd3	tudor domain contain	0006397 /,	0005634 /,	0003676 // nucleic acid binding // inferred fı

0.15879	Etv6	ets variant Circadian_	0006350 /,	0005634 /,	0003677 // DNA binding // inferred from dir
0.217482	Ncs1	neuronal calcium sens	0010975 /,	0005737 /,	0005509 // calcium ion binding // traceable
0.048645	Zc3h13	zinc finger CCCH type containing 13			0003676 // nucleic acid binding // inferred fr
0.037079	Rnf170	ring finger protein 170		0016020 /,	0005515 // protein binding // inferred from
0.04528	Rnf182	ring finger protein 182	0008152 /,	0005737 /,	0004842 // ubiquitin-protein ligase activity /
-0.04095	Asb7	ankyrin repeat and SC	0023034 // intracellular signaling pathway // inferred from electro		
0.035017	Cisd3	CDGSH iron sulfur domain 3		0005739 /,	0046872 // metal ion binding // inferred fron
-0.00417	4931428F0	RIKEN cDNA 4931428I	0001666 /,	0005622 /,	0005515 // protein binding // inferred from
-0.21557					
-0.04251	Nsd1	nuclear receptor-bind	0000122 /,	0005634 /,	0003682 // chromatin binding // inferred fro
0.145814	0710007G1	RIKEN cDNA 0710007G10 gene /,		0016020 // membrane // inferred from electronic anno	
-0.24204	Sft2d2	SFT2 domain containi	0006810 /,	0016020 // membrane // inferred from electronic anno	
-0.26305	Gm129	predicted gene 129			
0.078381	Gramd1b	GRAM domain containing 1B		0016020 // membrane // inferred from electronic anno	
-0.14023	Ncoa2	nuclear receptor coac	0000122 /,	0005634 /,	0003677 // DNA binding // inferred from ele
0.180322	Rab11b	RAB11B, member RAS	0006810 /,	0005739 /,	0000166 // nucleotide binding // inferred fro
-0.1657	Emilin2	elastin microfibril inte	0007155 /,	0005576 /,	0005515 // protein binding // inferred from
0.062713	AI452195	expressed sequence AI452195			
0.054838	Ip6k2	inositol hexaphosphat	0006817 /,	0005634 /,	0000166 // nucleotide binding // inferred fro
0.126902	Kctd15	potassium channel tet	0006813 /,	0008076 /,	0005216 // ion channel activity // inferred fr
-0.40653	Dock10	dedicator of cytokines	0050790 // regulation		0005085 // guanyl-nucleotide exchange fact
0.350942	D430042O	RIKEN cDNA D430042O09 gene			
0.105219	Ube2n	ubiquitin-conjugating	0000724 /,	0005634 /,	0000166 // nucleotide binding // inferred fro
0.052864	Pisd /// Pis	phosphatidylserine de	0008654 /,	0005739 /,	0004609 // phosphatidylserine decarboxylas
-0.12052	Fmo2	flavin containing mon	0006082 /,	0005783 /,	0004497 // monooxygenase activity // inferr
-0.00342	Kremen1	kringle containing tran	0016055 /,	0016020 // membrane // inferred from sequence or str	
0.053182	Lman2l	lectin, mannose-bindi	0015031 /,	0000139 /,	0005529 // sugar binding // inferred from el
0.196508	Slc5a3	solute carrier family 5	0006020 /,	0005739 /,	0003735 // structural constituent of ribosom
0.244694					
-0.24621	Hk3	hexokinase 3	0005975 /,	0005739 /,	0000166 // nucleotide binding // inferred fro
0.118855	Letm2	leucine zipper-EF-hand containin		0005739 // mitochondrion // inferred from electronic a	
0.102265	AI836003	expressed sequence AI836003		0005737 // cytoplasm // inferred from electronic annot	
0.049054	Ddi2	DNA-damage inducibl	0006508 // proteolysi		0004190 // aspartic-type endopeptidase acti
-0.07781	1810013L2	RIKEN cDNA 1810013L24 gene			
-0.1897	AW413774	expressed sequence AW413774			
-0.00254	Ccdc85b	coiled-coil domain cor	0006350 /,	0005634 /,	0005515 // protein binding // inferred from
-0.10874	Abhd2	abhydrolase domain c	0008152 /,	0016020 /,	0004091 // carboxylesterase activity // infer
-0.16746	Esr1	estrogen re Nuclear_Re	0001547 /,	0005634 /,	0003677 // DNA binding // not recorded ///
0.121593	Clock	circadian lc Circadian_	0006350 /,	0005634 /,	0003677 // DNA binding // inferred from ele
0.041245	Egfr	epidermal growth fact	0000186 /,	0005622 /,	0000166 // nucleotide binding // inferred fro
0.134677	Asxl3	additional sex combs	0006350 /,	0005634 /,	0046872 // metal ion binding // inferred fron
0.229048	Vldlr	very low density lipop	0001666 /,	0005615 /,	0004872 // receptor activity // inferred from
0.126049	6430548M	RIKEN cDNA 6430548M08 gene		0005634 // nucleus // inferred from electronic annotati	
0.271812	Dclk1	doublecortin-like kina	0001764 // neuron m		0000166 // nucleotide binding // inferred fro
0.329417	Nat14	N-acetyltransferase 14	0006350 /,	0016020 /,	0003677 // DNA binding // inferred from ele
-0.03701	Adamts2	a disintegrin-like and	0006508 /,	0005576 /,	0004222 // metalloendopeptidase activity //
0.012985	Zkscan4	zinc finger with KRAB	0006350 /,	0005622 /,	0003676 // nucleic acid binding // inferred fr

0.007017	Tpd52	tumor protein D52	0030183 /,	0005737 /,	0005509 // calcium ion binding // inferred fr	
0.019583	Lrrc16b	leucine rich repeat containing 16B				
0.274441	Gsg1l	GSG1-like		0016021 // integral to membrane // inferred from elect		
-0.00265	Scn7a	sodium channel, volta	0006810 /,	0001518 /,	0005216 // ion channel activity // inferred fr	
0.076322	Scn7a	sodium channel, volta	0006810 /,	0001518 /,	0005216 // ion channel activity // inferred fr	
-0.1641	Lrrc15	leucine rich repeat containing 15		0005622 /,	0005515 // protein binding // inferred from	
0.320422	Sh2d5	SH2 domain containing 5			0005515 // protein binding // inferred from	
0.177365	Tmem212	transmembrane protein 212		0016020 // membrane // inferred from electronic anno		
-0.12183	Vangl2	vang-like 2 (van gogh,	0001736 /,	0005886 /,	0005515 // protein binding // inferred from	
-0.46433	Arhgap30	Rho GTPase activating	0007165 /,	0005622 /,	0005096 // GTPase activator activity // infer	
-0.2561	Arid4a	AT rich interactive doı	0006333 /,	0000785 /,	0003676 // nucleic acid binding // inferred fı	
0.013786	Gk5	glycerol kinase 5 (puta	0005975 // carbohydr		0000166 // nucleotide binding // inferred frc	
0.032321	Grpel2	GrpE-like 2, mitochon	0006457 /,	0005737 /,	0000774 // adenyl-nucleotide exchange fact	
0.300328	Gm10419	predicted gene 10419				
0.077999	Kcnip2	Kv channel-interacting	0006810 /,	0005886 /,	0005216 // ion channel activity // inferred fr	
-0.10992	Ildr2	immunoglobulin-like (	0009749 // response to glucose stimulus // inferred from mutant p			
-0.15656	Scyl2	SCY1-like 2 (S. cerevisi	0006468 /,	0005737 /,	0004672 // protein kinase activity // inferrec	
-0.08691	Dzip1l	DAZ interacting protein 1-like		0005622 /,	0008270 // zinc ion binding // inferred from	
-0.07972	Map3k10	mitogen-activated prc	0006468 // protein pł		0000166 // nucleotide binding // inferred frc	
0.076403	Gpr137	G protein-coupled receptor 137		0016020 // membrane // inferred from electronic anno		
0.070585	Ankdd1b	Ankyrin repeat and de	0007165 // signal trar		0005515 // protein binding // inferred from	
-0.11844	Arhgef33	Rho guanine nucleotic	0035023 /,	0005622 /,	0005085 // guanyl-nucleotide exchange fact	
-0.04132	Gm97	predicted gene 97				
0.134015	Dcaf5	DDB1 and CUL4 associated factoı		0005739 // mitochondrion // inferred from direct assay		
-0.01623	6030405A:	RIKEN cDNA 6030405A18 gene		0016020 // membrane // inferred from electronic anno		
0.136804	Dand5	DAN domain family, n	0007368 /,	0005576 // extracellular region // inferred from electro		
0.007696	LOC10050:	hypothetical protein LOC100503807				
-0.01437	Tanc2	tetratricopeptide repeat, ankyrin repeat anc			0005488 // binding // inferred from electror	
-0.28767	Exoc3l	exocyst complex com	0006887 /,	0000145 /,	0005515 // protein binding // inferred from	
-0.00261	Kif3a	kinesin family membe	0001701 /,	0005874 /,	0000166 // nucleotide binding // inferred frc	
-0.26774	Fcgr1	Fc recepto Inflammat	0001788 /,	0005886 /,	0004872 // receptor activity // inferred from	
0.23594						
0.185703	Grk5	G protein-(Calcium_re	0006468 /,	0005886 /,	0000166 // nucleotide binding // inferred frc	
0.024075	Actr5	ARP5 actin-related pr	0006350 // transcripti		0005515 // protein binding // inferred from	
-0.10509	Pla2g4c	phospholipase A2, grc	0008152 // metabolic		0004620 // phospholipase activity // inferrec	
-0.23945	Itih5	inter-alpha (globulin)	0010466 /,	0005576 /,	0004867 // serine-type endopeptidase inhib	
-0.61448	Cybb	cytochrome b-245, be	0006810 /,	0005622 /,	0005216 // ion channel activity // inferred fr	
0.211054	Pcdh17	protocadherin 17	0007155 /,	0005886 /,	0005509 // calcium ion binding // inferred fr	
0.141396	Ubn2	ubinuclein 2				
0.148475						
-0.04487	Zbtb20	zinc finger and BTB dc	0006350 /,	0005622 /,	0003676 // nucleic acid binding // inferred fı	
-0.24764	A630033E(	RIKEN cDNA A630033	0006355 /,	0005622 /,	0003676 // nucleic acid binding // inferred fı	
-0.0689	Tfdp2	transcripti G1_to_S_c	0006350 /,	0005634 /,	0003677 // DNA binding // inferred from ele	
-0.14813	Rrm2b	ribonucleotide reduct	0001822 /,	0005634 /,	0004748 // ribonucleoside-diphosphate redu	
-0.13118	Rrm2b	ribonucleotide reduct	0001822 /,	0005634 /,	0004748 // ribonucleoside-diphosphate redu	
-0.15142	Gas2l3	growth arrest-specific	0007050 // cell cycle arrest // inferred from electronic annotation			
-0.12726	Susd5	sushi domain containing 5				

-0.36644	Aldh2	aldehyde dehydrogen	0001889 /,	0005739 /,	0004029 // aldehyde dehydrogenase (NAD)
-0.04998	Syde2	synapse defective 1, F	0007165 /,	0005622 // intracellular // inferred from electronic ann	
0.394115	Mmp15	matrix met Matrix_Me	0006508 /,	0016020 /,	0004222 // metalloendopeptidase activity //
-0.01916	Efhd2	EF hand domain containing 2		0016020 /,	0005509 // calcium ion binding // inferred fr
-0.2854	Mkx	mohawk homeobox	0006355 /,	0005634 /,	0003677 // DNA binding // inferred from ele
0.0732	Adamts6	a disintegrin-like and	0006508 /,	0005578 /,	0004222 // metalloendopeptidase activity //
-0.17936	Zbtb20	zinc finger and BTB dc	0006350 /,	0005622 /,	0003676 // nucleic acid binding // inferred fr
0.086828	1110034G:	RIKEN cDNA 1110034G24 gene			
0.178068	Xrcc3	X-ray repair complem	0006200 /,	0005634 /,	0000166 // nucleotide binding // inferred fro
-0.06234	Tgm6	transglutaminase 6	0008152 // metabolic		0003810 // protein-glutamine gamma-glutar
0.148703	Chd7	chromodomain helica	0001501 /,	0000785 /,	0000166 // nucleotide binding // inferred fro
-0.25694	Lrrn4cl	LRRN4 C-terminal like		0016020 // membrane // inferred from electronic anno	
0.192017	Sp6	trans-acting transcript	0006350 /,	0005622 /,	0003676 // nucleic acid binding // inferred fr
0.429508	Foxs1	forkhead box S1	0001503 /,	0005634 /,	0003677 // DNA binding // inferred from dir
0.188986	Dpy19l3	dpy-19-like 3 (C. elegans)		0016020 // membrane // inferred from electronic anno	
0.021691	Alg10b	asparagine-linked glyc	0008152 /,	0005886 /,	0015459 // potassium channel regulator acti
0.111816	Zfp516	zinc finger protein 51(	0006350 /,	0005622 /,	0003676 // nucleic acid binding // inferred fr
-0.02673	Hhip	Hedgehog-interacting	0007165 /,	0005576 /,	0003824 // catalytic activity // inferred from
-0.34873	Lrch1	leucine-rich repeats and calponin homology			0005515 // protein binding // inferred from
-0.03474	Npas3	neuronal PAS domain	0006350 /,	0005634 /,	0003677 // DNA binding // inferred from ele
-0.10726					
0.050407	Senp8	SUMO/sentrin specific	0006508 // proteolysi		0005515 // protein binding // inferred from
0.023131	LOC10050:	hypothetical LOC100503671			
-0.01101	Pggt1b	protein geranylgerany	0008152 /,	0005953 /,	0003824 // catalytic activity // inferred from
-0.14907	AI846148	expressed sequence AI846148			
-0.0658	C130046K2	RIKEN cDNA C130046K22 gene			
-0.00994	Dock2	Dedicator of cyto-kine	0001766 /,	0005737 /,	0005085 // guanyl-nucleotide exchange fact
0.309763					
0.065625	Tbc1d24	TBC1 domain family, r	0032313 /,	0005622 /,	0005096 // GTPase activator activity // infer
-0.24349	Gtf3c1	general transcription	0006350 /,	0000127 /,	0003677 // DNA binding // inferred from ele
-0.18199	Lnpep	leucyl/cystinyl aminop	0006508 /,	0005622 /,	0001595 // angiotensin receptor activity // r
-0.04843	Socs2	suppressor of cytokine	0007595 // lactation /		0005131 // growth hormone receptor bindir
-0.04832	Ado	2-aminoethanethiol (c	0055114 /,	0005739 /,	0016491 // oxidoreductase activity // inferre
-0.0044	Esd	esterase D/formylglut	0008152 /,	0005737 /,	0004091 // carboxylesterase activity // infer
-0.06687	Slc39a14	solute carrier family 3	0006810 /,	0005886 /,	0005385 // zinc ion transmembrane transpo
-0.10471	A530054K:	RIKEN cDNA A530054	0006355 /,	0005622 /,	0003676 // nucleic acid binding // inferred fr
-0.47611	Gkn3	gastrokine 3		0005576 // extracellular region // inferred from electro	
-0.0728	Gcnt2	glucosaminyl (N-acety	0008152 /,	0000139 /,	0008109 // N-acetyllactosaminide beta-1,6-I
0.118933	Ajap1	adherens junction ass	0007155 /,	0005886 // plasma membrane // inferred from electror	
0.082072	Nuak1	NUAK family, SNF1-lik	0006468 // protein pl		0000166 // nucleotide binding // inferred fro
-0.1407	Rassf8	Ras association (RalGI	0007165 // signal transduction // inferred from electronic annotati		
-0.13426	Med13	mediator complex sut	0006350 /,	0005634 /,	0003713 // transcription coactivator activity
0.13522	Nif3l1	Ngg1 interacting facto	0045893 /,	0005737 /,	0005515 // protein binding // inferred from
-0.05993					
0.093578					
-0.18633	Gm10512	predicted gene 10512			
-0.38907	H2-Aa	histocompatibility 2, c	0002504 /,	0005764 /,	0005515 // protein binding // inferred from

0.258131						
0.112928	Zdhhc3	zinc finger, DHHC dom		0006605 /,	0000139 /,	0008270 // zinc ion binding // inferred from
0.109706	Acsbg2	acyl-CoA synthetase b		0006629 /,	0005737 /,	0000166 // nucleotide binding // inferred fro
0.09267	Gclm	glutamate-cysteine lig		0006534 /,	0005625 /,	0004357 // glutamate-cysteine ligase activity
-0.24872	Pik3ip1	phosphoinositide-3-kinase intera			0016020 // membrane // inferred from electronic anno	
-0.35491	Srsf11	serine/argi	mRNA_processing_binding_Reac			0000166 // nucleotide binding // inferred fro
-0.08264	C230081A1	RIKEN cDNA C230081/		0006468 // protein pł		0000166 // nucleotide binding // inferred fro
-0.06627	Mll5	myeloid/lymphoid or		0002446 /,	0005634 /,	0003713 // transcription coactivator activity
0.107089	5830405N1	RIKEN cDNA 5830405N20 gene				
-0.01895	9530010C2	RIKEN cDNA 9530010C24 gene				
0.010076						
0.004677	D630036G	RIKEN cDNA D630036G22 gene				
0.155324	Dot1l	DOT1-like, histone H3		0008152 // metabolic		0003677 // DNA binding // inferred from ele
-0.03243	Atxn7l1	ataxin 7-like 1				
-0.1253	Rlf	rearranged L-myc fusion sequenc			0005622 /,	0003676 // nucleic acid binding // inferred fı
-0.04375						
-0.1274	Ppp4r1l-ps	protein phosphatase 4, regulatory subunit 1-like, pseudogene				
0.286913						
-0.11448	Adc	arginine decarboxylas		0006591 /,	0005739 /,	0003824 // catalytic activity // inferred from
-0.12771	LOC10030	hypothetical protein LOC100302688				
0.07686	4930466K1	RIKEN cDNA 4930466K18 gene				
-0.35666	Ebf1	early B-cell factor 1		0006350 /,	0005634 /,	0003677 // DNA binding // inferred from dir
-0.38875	Adamts12	a disintegrin-like and ı		0006508 /,	0005576 /,	0004222 // metalloendopeptidase activity //
0.150379						
0.096116	9630033F2	RIKEN cDNA 96300331		0008152 /,	0005622 /,	0003824 // catalytic activity // inferred from
-0.12409						
0.125717	Zfp398	zinc finger protein 398		0006355 /,	0005622 /,	0003676 // nucleic acid binding // inferred fı
-0.09336						
0.072439	E330013P(	RIKEN cDNA E330013P04 gene				
-0.12698						
-0.02439	Fam199x	family with sequence similarity 199, X-linked				
-0.02263	Etl4	enhancer trap locus 4		0007275 /,	0005737 // cytoplasm // inferred from direct assay /// (	
-0.30387	Eda2r	ectodysplasin A2 rece		0007165 /,	0005887 /,	0004872 // receptor activity // inferred from
0.144737	Rnf182	ring finger protein 182		0008152 /,	0005737 /,	0004842 // ubiquitin-protein ligase activity /
-0.02714	Sipa1l1	signal-induced prolife		0031532 /,	0005622 /,	0005096 // GTPase activator activity // infer
-0.29873	Mmp16	matrix met	Matrix_Me	0006508 /,	0005886 /,	0004222 // metalloendopeptidase activity //
0.065609	Fam184b	family with sequence similarity 184, member B				
-0.23986	Rbm6	RNA bindir	mRNA_processing_bi		0005622 /,	0000166 // nucleotide binding // inferred fro
-0.03802	Tmem200c	transmembrane protein 200C				
-0.06415	Scyl2	SCY1-like 2 (S. cerevisi		0006468 /,	0005737 /,	0004672 // protein kinase activity // inferrec
-0.25917	Tulp4	tubby like protein 4		0023034 /,	0005737 /,	0005515 // protein binding // inferred from
-0.08512						
-0.38767	Wdr35	WD repeat domain 35				0005488 // binding // inferred from electror
0.133158	Aasdh	aminoadipate-semialc		0006629 // lipid meta		0000036 // acyl carrier activity // inferred fro
0.110031						
-0.0297	Sec11c	SEC11 homolog C (S. c		0006465 /,	0005783 /,	0008233 // peptidase activity // inferred fror
-0.10888	Fam171a2	family with sequence similarity 1		0016020 // membrane // inferred from electronic anno		

-0.00684						
0.013065	Gm2590	predicted gene 2590				
-0.06706						
-0.1195						
0.226688	Cdkn2aipn	CDKN2A interacting protein N-terminal like				
-0.13648	Fam105a	family with sequence similarity 105, member A				
-0.02567	Gle1	GLE1 RNA export mec		0006810 /,	0005634 /,	0005515 // protein binding // inferred from
-0.05966						
0.022479						
-0.32785	LOC10050:	hypothetical LOC100503570				
-0.26161	B230208H:	RIKEN cDNA B230208		0007264 /,	0005622 /,	0000166 // nucleotide binding // inferred fro
0.099047	BC030308	cDNA sequence BC030308				
-0.27659	Nsd1	nuclear receptor-bind		0000122 /,	0005634 /,	0003682 // chromatin binding // inferred fro
0.106503	Kcnh8	potassium voltage-ga		0000160 /,	0016020 /,	0000155 // two-component sensor activity /
-0.23434	LOC10050:	hypothetical LOC100502834				
0.090348	Palm2	paralemmi	G_Protein_	0008360 /,	0005886 // plasma membrane // inferred from electror	
-0.09116	E130309D:	RIKEN cDNA E130309D14 gene				
-0.0175	Arx	Aristaless related hor		0001764 /,	0005634 /,	0003677 // DNA binding // inferred from ele
-0.20768	Spon1	spondin 1, (f-spondin)		0007155 /,	0005576 /,	0005515 // protein binding // inferred from
0.307855	Ssr1	signal sequence receptor, alpha			0005783 // endoplasmic reticulum // inferred from elec	
0.060452	5930412G:	RIKEN cDNA 5930412G12 gene				
4.39E-04	Chd9	chromodomain helica		0006333 /,	0000785 /,	0000166 // nucleotide binding // inferred fro
0.068484	Gabarapl2	gamma-aminobutyric		0006810 /,	0000139 /,	0005515 // protein binding // inferred from
-0.02994	Kif6	kinesin family membe		0007018 /,	0001673 /,	0000166 // nucleotide binding // inferred fro
0.142031	Gpc6	glypican 6			0005576 /,	0043395 // heparan sulfate proteoglycan bir
-0.12219	8030453O:	RIKEN cDNA 8030453O22 gene				
-0.13164	Mmp24	matrix me	Matrix_Me	0006508 /,	0005576 /,	0004222 // metalloendopeptidase activity //
0.001819						
0.081038	Dcc	deleted in colorectal c		0001764 /,	0016020 /,	0003713 // transcription coactivator activity
-0.10988	Spg7	Spastic paraplegia 7 h		0006508 /,	0005739 /,	0000166 // nucleotide binding // inferred fro
0.093935						
-0.13331	AU015603	expressed sequence AU015603				
0.09125	Cbfa2t2	core-binding factor, ru		0006355 /,	0005634 /,	0003700 // sequence-specific DNA binding t
-0.09131	Zcchc8	zinc finger, CCHC dom		0006397 /,	0005681 /,	0003676 // nucleic acid binding // inferred fr
-0.10726	Maml1	Mastermind like 1 (Dr		0006350 /,	0005634 /,	0003713 // transcription coactivator activity
0.009199	Scn1a	sodium channel, volta		0001508 /,	0001518 /,	0005216 // ion channel activity // inferred fr
-0.09096	Rictor	RPTOR independent c		0001938 /,	0031932 /,	0005488 // binding // inferred from electror
-0.01452						
-0.15219	Timm50	translocase of inner m		0001836 /,	0005739 /,	0004721 // phosphoprotein phosphatase ac
0.305455	Snrpn /// S	small nucle	mRNA_processing_bi		0005634 /,	0003676 // nucleic acid binding // inferred fr
-0.27451	Edn3	endothelin 3		0001755 /,	0005576 /,	0005179 // hormone activity // inferred fror
-0.23387	Edn3	endothelin 3		0001755 /,	0005576 /,	0005179 // hormone activity // inferred fror
-0.11239	Itih5	inter-alpha (globulin)		0010466 /,	0005576 /,	0004867 // serine-type endopeptidase inhib
0.17894	Lynx1	Ly6/neurotoxin 1		0007271 /,	0005624 /,	0005515 // protein binding // inferred from
-0.19601	9530082P:	RIKEN cDNA 9530082P21 gene				
0.109617	Hist2h3c1	histone cluster 2, H3c		0006334 /,	0000786 /,	0003677 // DNA binding // inferred from ele
0.086305	AI449212	expressed sequence AI449212				

0.070802	LOC10050:	hypothetical LOC100503166				
0.219519						
-0.16648	BC028454	cDNA sequence BC028454				
0.013476	Panx2	Pannexin 2	0007267 /,	0005886 /,	0005243 // gap junction channel activity // r
-0.02733	Arhgef17	Rho guanine nucleotic	0030036 /,	0005622 /,	0005085 // guanyl-nucleotide exchange fact
-0.06772					
-0.03649					
-0.1387					
-0.1325	Zbtb24	zinc finger and BTB dc	0006350 /,	0005622 /,	0003676 // nucleic acid binding // inferred fr
0.202926	Zfp563	zinc finger protein 56:	0006355 /,	0005622 /,	0003676 // nucleic acid binding // inferred fr
-0.00507	Tmem167	transmembrane protein 167		0000139 // Golgi membrane // inferred from electronic	
-0.0957	Maoa	monoamin Biogenic_A	0006584 /,	0005625 /,	0005515 // protein binding // inferred from
0.012148					
0.199303					
-0.13512	Gm11627	predicted gene 11627			
0.136212					
0.18298	Ccdc66	coiled-coil domain containing 66			
-0.03993					
0.338418					
-0.10103	Grm7	glutamate receptor, n	0001661 /,	0005624 /,	0001640 // adenylate cyclase inhibiting meta
0.10633	Huwe1	HECT, UBA and WWE	0000209 /,	0005622 /,	0003677 // DNA binding // inferred from ele
0.023762	LOC10050!	hypothetical LOC100505042			
0.370284	Vax2os2	Vax2 opposite strand transcript 2			
-0.06017					
0.002552	Bcl10	B-cell leukemia/lympl	0001843 /,	0001772 /,	0003713 // transcription coactivator activity
-0.04625	Zfp882	zinc finger protein 88:	0006355 /,	0005622 /,	0003676 // nucleic acid binding // inferred fr
-0.20142	Rapgef6	Rap guanine nucleotic	0007165 /,	0005622 /,	0005085 // guanyl-nucleotide exchange fact
-0.11985	Chd8	chromodomain helica	0001701 /,	0000785 /,	0000166 // nucleotide binding // inferred fro
-0.102	C2cd2	C2 calcium-dependent domain cc		0005622 /,	0003676 // nucleic acid binding // inferred fr
-0.33644	Cd55	CD55 antig Compleme	0006958 /,	0005886 /,	0004857 // enzyme inhibitor activity // not r
-0.08369	6330408A(	RIKEN cDNA 6330408A02 gene			
-0.38695	Akap13	A kinase (PRKA) anchc	0010611 /,	0005622 /,	0005089 // Rho guanyl-nucleotide exchange
0.123884	Zfp882	zinc finger protein 88:	0006355 /,	0005622 /,	0003676 // nucleic acid binding // inferred fr
-0.10448	Trip11	thyroid hormone rece	0007165 /,	0005634 /,	0004872 // receptor activity // inferred from
-0.11531	A730017C:	RIKEN cDNA A730017C20 gene		0016020 // membrane // inferred from electronic anno	
-0.17275	Spnb2	spectrin beta 2	0007182 /,	0005634 /,	0003779 // actin binding // inferred from ele
0.020252	E330017L1	RIKEN cDNA E330017L17 gene			
-0.05274					
0.128124	Kdm5c	lysine (K)-specific der	0006350 /,	0005622 /,	0003677 // DNA binding // inferred from ele
-0.00771	A530020G	RIKEN cDNA A530020G20 gene			
0.076831	6030446N:	RIKEN cDNA 6030446N20 gene		0016020 // membrane // inferred from electronic anno	
-0.01422	Gm9895 //	predicted gene 9895 /// hypothetical LOC100503337			
-0.27753					
0.159814	1700025G(	RIKEN cDNA 1700025G04 gene			
-0.0115	D630045J1	RIKEN cDNA D630045J12 gene		0016020 // membrane // inferred from electronic anno	
0.194355					
-0.0079	Bri3bp	Bri3 binding protein		0016020 // membrane // inferred from electronic anno	

-0.16796					
0.059393					
-0.25769					
-0.12854	Adam33	a disintegrin and meta	0006508 /,	0016020 /,	0004222 // metalloendopeptidase activity //
0.005932					
0.077329	E230016M	RIKEN cDNA E230016M11 gene			
-0.12845	Pigg	phosphatidylinositol g	0006506 /,	0005783 /,	0003824 // catalytic activity // inferred from
0.172609	BC037032	cDNA Sequence BC037032			
0.351251					
-0.08917	Atxn7l1	ataxin 7-like 1			
-0.17818	Tex9	Testis expressed gene 9			
-0.21145					
-0.12127					
-0.04361	Cdyl2	Chromodomain prote	0006333 /,	0000785 /,	0003682 // chromatin binding // inferred fro
-0.49553	Spg11	spastic paraplegia 11		0005634 // nucleus // inferred from electronic annotati	
-0.06311					
0.002744					
-5.32E-04	Rapgef5	Rap guanine nucleotic	0007264 /,	0005622 /,	0005085 // guanyl-nucleotide exchange fact
-0.02951	Ercc1	Excision repair cross-c	0000720 /,	0000109 /,	0003677 // DNA binding // inferred from ele
-0.2199					
0.006208	Coq9	coenzyme Q9 homolo	0006744 /,	0005739 // mitochondrion // inferred from direct assay	
-0.13366	Ubxn7	UBX domain protein 7			0005515 // protein binding // inferred from
-0.08441					
-0.0159					
-0.09409					
0.053499	Ogdh	oxoglutara Krebs-TCA_	0006091 /,	0005739 /,	0004591 // oxoglutarate dehydrogenase (su
0.444489					
-0.14445					
-0.22695					
-0.06822	AU018466	expressed sequence AU018466			
-0.12803					
-0.02632					
-0.22487	Akap9	A kinase (P G_Protein_Signaling /		0000242 /,	0005515 // protein binding // inferred from
0.087566	9530067D:	Riken cDNA 9530067D14 gene			
-0.07947	Tcf12	Transcription factor 1	0006350 /,	0005634 /,	0003677 // DNA binding // inferred from dir
-0.16961	Ptgfr	prostaglan GPCRDB_C	0007165 /,	0005576 /,	0004871 // signal transducer activity // infer
-0.13266	A130088B(	RIKEN cDNA A130088B03 gene			
-0.15022					
-0.01925	Sorbs2	sorbin and SH3 domain containin		0005622 /,	0008270 // zinc ion binding // inferred from
0.10757	Tuba8	tubulin, alpha 8	0000226 /,	0005874 /,	0000166 // nucleotide binding // inferred fro
-0.0144	E230025N:	Riken cDNA E230025N	0007018 // microtubu		0003777 // microtubule motor activity // inf
-0.19235	Gm5891	Predicted gene 5891	0006468 // protein pl		0000166 // nucleotide binding // inferred fro
0.022158					
0.065148	Dnajc17	DnaJ (Hsp4 mRNA_prc	0006457 // protein fo		0000166 // nucleotide binding // inferred fro
-0.2695					
-0.01625					
9.48E-04					

-0.03676						
-0.04432						
-0.24902						
-0.01915	Myo1b	myosin IB	0008152 /,	0005903 /,	0000166 // nucleotide binding // inferred fro	
-0.11404	Pigt	phosphatidylinositol g	0006506 /,	0005737 /,	0003923 // GPI-anchor transamidase activity	
0.010859						
-0.02848	Wdfy3	WD repeat and FYVE c	0008152 /,	0005635 /,	0003831 // beta-N-acetylglucosaminylglycop	
0.055691	Snhg7	Small nucleolar RNA host gene (non-protein coding) 7				
0.120925	Cux2	cut-like homeobox 2	0006350 /,	0005634 /,	0003677 // DNA binding // inferred from dir	
-0.15202	Itgae	integrin alp Integrin-m	0007155 /,	0008305 /,	0004872 // receptor activity // inferred from	
-0.11301						
0.083298						
-0.09359	Pskh1	protein serine kinase	0006468 /,	0005634 /,	0000166 // nucleotide binding // inferred fro	
0.07615	Opa3	optic atrophy 3 (human)		0005739 // mitochondrion // inferred from direct assay		
0.188763	Drg1	developmentally regulated GTP b		0005622 /,	0000166 // nucleotide binding // inferred fro	
0.163131						
-0.01262	Bat1a	HLA-B-assc mRNA_prc	0006200 /,	0005634 /,	0000166 // nucleotide binding // inferred fro	
-0.05189	Rhebl1	Ras homolog enrichec	0006184 /,	0005622 /,	0000166 // nucleotide binding // inferred fro	
-0.22807	Mapk12	mitogen-ac Integrin-m	0006468 /,	0005625 /,	0000166 // nucleotide binding // inferred fro	
-0.19454	1110002E2	RIKEN cDNA 1110002E22 gene				
-0.32722	Whsc1l1	Wolf-Hirschhorn synd	0006350 /,	0005634 /,	0005515 // protein binding // inferred from	
-0.2305	A430107O	RIKEN cDNA A430107O13 gene		0005783 // endoplasmic reticulum // inferred from elec		
-0.18117	Zdhhc24	zinc finger, DHHC don	0008152 /,	0016020 /,	0008270 // zinc ion binding // inferred from	
-0.04019	R3hdm2	R3H domain containing 2		0005634 /,	0003676 // nucleic acid binding // inferred fr	
-0.18936	Fbxo7	F-box protein 7	0031647 /,	0043234 /,	0005515 // protein binding // inferred from	
-0.12376	Rbms3	RNA bindir mRNA_processing_bi		0005737 /,	0000166 // nucleotide binding // inferred fro	
0.116013	D11Wsu47	DNA segment, Chr 11, Wayne Sta		0016020 // membrane // inferred from electronic anno		
-0.17272	Rapgef2	Rap guanine nucleotic	0007165 /,	0005622 /,	0005085 // guanyl-nucleotide exchange fact	
0.016217	Brms1l	breast cancer metasta	0006350 /,	0005634 // nucleus // inferred from electronic annotati		
-0.07598	Shpk	sedoheptulokinase	0005975 /,	0005737 /,	0000166 // nucleotide binding // inferred fro	
0.006743	1200016B1	RIKEN cDNA 1200016B10 gene				
-0.18019	D19Ertd73	DNA segment, Chr 19, ERATO Doi 737, expressed				
-0.17639	Rnaseh2b	ribonuclease H2, subunit B		0005634 // nucleus // inferred from electronic annotati		
0.019008	3110082I1	RIKEN cDNA 3110082I17 gene			0005515 // protein binding // inferred from	
-0.11846	Wdr37	WD repeat domain 37				
0.202221	Pcdh17	protocadherin 17	0007155 /,	0005886 /,	0005509 // calcium ion binding // inferred fr	
-0.02787	Rasgef1c	RasGEF domain family	0007264 /,	0005622 /,	0005085 // guanyl-nucleotide exchange fact	
-0.10258	Nol9	nucleolar protein 9		0005634 // nucleus // inferred from electronic annotati		
0.082452	Zfp689	zinc finger protein 689	0006350 /,	0005622 /,	0003676 // nucleic acid binding // inferred fr	
0.042449	Sf3b2	splicing fac mRNA_prc	0006397 /,	0005634 /,	0003676 // nucleic acid binding // inferred fr	
-0.04775	Ppm1j	protein phosphatase 1	0006470 // protein de		0003824 // catalytic activity // inferred from	
0.041249	Fndc5	fibronectin type III domain conta		0005777 /,	0005515 // protein binding // inferred from	
-0.1648	Gapvd1	GTPase activating pro	0006897 /,	0005622 /,	0005085 // guanyl-nucleotide exchange fact	
-0.05088	Psmb11	proteasome (prosome	0051603 /,	0000502 /,	0004175 // endopeptidase activity // inferre	
-0.26161	Ube2i	ubiquitin-conjugating	0000122 /,	0001650 /,	0000166 // nucleotide binding // inferred fro	
-0.10296	Zfand5	zinc finger, AN1-type	0001701 // in utero e		0003677 // DNA binding // inferred from ele	
-0.09646	Plxna2	plexin A2	0001756 /,	0005622 /,	0004872 // receptor activity // inferred from	

0.06087	6330531I0	RIKEN cDNA 6330531I01 gene				
0.071985	Ppil6	peptidylpr	S1P_Signal	0006457 // protein fo		0003755 // peptidyl-prolyl cis-trans isomeras
0.25355	C2cd4b	C2 calcium-dependent domain containing 4B				
0.308491	2700008G	RIKEN cDNA 2700008G24 gene				
0.099258	4922501L1	RIKEN cDNA 4922501L14 gene				
0.087039	6430500C	RIKEN cDNA 6430500C12 gene				
0.289974	Srr	serine racemase		0006520 /,	0005625 /,	0003824 // catalytic activity // inferred from
-0.02835	2210408I2	RIKEN cDNA 2210408I21 gene				
-0.10625	C530020B	RIKEN cDNA C530020B09 gene				
-0.01406	Ttc21b	tetratricopeptide repeat domain			0005737 /,	0005488 // binding // inferred from electror
-0.18607	Alkbh8	alkB, alkyla	mRNA_prc	0006974 /,	0005634 /,	0000166 // nucleotide binding // inferred frc
0.030492	Dmrtc1c //	DMRT-like family C1c /// DMRT-like family C1c2				
0.092473	C030039L	RIKEN cDNA C030039		0006355 /,	0005622 /,	0003676 // nucleic acid binding // inferred fı
-0.25029	2310065F	RIKEN cDNA 2310065F04 gene				
-0.05218	Nr3c1	nuclear rec	Apoptosis_	0006111 /,	0005622 /,	0003677 // DNA binding // inferred from dir
0.032607	Mrpl47	mitochondrial ribosor		0006412 /,	0005739 /,	0003735 // structural constituent of ribosor
-0.15456	Col14a1	collagen, type XIV, alp		0007155 /,	0005576 /,	0005198 // structural molecule activity // inf
-0.04242	Pdilt	protein disulfide isom		0007275 /,	0005783 /,	0016853 // isomerase activity // inferred fro
0.036687	4933402D	RIKEN cDNA 4933402D24 gene				
-0.07978	1700040N	RIKEN cDNA 1700040N02 gene				
-0.11509	9530053H	RIKEN cDNA 9530053H05 gene				
-0.14044	2010300F1	RIKEN cDNA 2010300F17 gene				
-0.15536	Ing5	inhibitor of growth fai		0006260 /,	0005634 /,	0005515 // protein binding // inferred from
-0.02499	Exoc6b	exocyst complex com		0006904 /,	0000145 // exocyst // inferred from electronic annotati	
-0.079	Cldnd2	claudin domain containing 2			0016020 // membrane // inferred from electronic anno	
0.042519	4631402F	RIKEN cDNA 4631402F24 gene				
-0.18199	5830420C	RIKEN cDNA 5830420C07 gene				
-0.13167						
0.024962	Fam63a	Family with sequence similarity 63, member				0005515 // protein binding // inferred from
0.194808	Klhl8	kelch-like 8 (Drosophila)				0005515 // protein binding // inferred from
-0.00659	Dgkd	diacylglycerol kinase,		0007205 /,	0005737 /,	0004143 // diacylglycerol kinase activity // n
-0.07525	Ilvbl	ilvB (bacterial acetolac		0008152 /,	0016020 /,	0000287 // magnesium ion binding // inferre
-0.11859	Mib1	mindbomb homolog 1		0001568 /,	0005737 /,	0004842 // ubiquitin-protein ligase activity /
-0.17067	Slco2b1	solute carrier organic		0006810 /,	0009925 /,	0005215 // transporter activity // inferred fr
0.014474	Fbn2	fibrillin 2		0030326 /,	0001527 /,	0005201 // extracellular matrix structural co
0.050981	Nfib	nuclear factor I/B		0006260 /,	0005622 /,	0003677 // DNA binding // inferred from ele
0.205318	Sp1	trans-actin	Smooth_m	0001503 /,	0005622 /,	0003676 // nucleic acid binding // inferred fı
0.068025	Prkcz	protein kin	Calcium_re	0000226 /,	0005625 /,	0000166 // nucleotide binding // inferred frc
-0.0358	Ppm1f	protein phosphatase :		0006470 /,	0008287 /,	0003824 // catalytic activity // inferred from
0.254814	Mapt	microtubule-associate		0000226 /,	0005737 /,	0005515 // protein binding // inferred from
-0.01355	Flrt2	fibronectin leucine rich transmer			0016021 /,	0005515 // protein binding // inferred from
0.079796	Ddx46	DEAD (Asp-Glu-Ala-As		0006200 /,	0005634 /,	0000166 // nucleotide binding // inferred frc
0.182164	Srpk1	serine/argi	mRNA_prc	0006397 /,	0005634 /,	0000166 // nucleotide binding // inferred frc
0.197107	Ttc39a	tetratricopeptide repeat domain 39A				0005488 // binding // inferred from electror
-0.34886	Angptl1	angiopoietin-like 1		0007165 /,	0005576 /,	0005102 // receptor binding // inferred fron
0.106631	Spnb1	spectrin beta 1		0006779 /,	0005737 /,	0003779 // actin binding // inferred from ele
0.163232	Whsc1	Wolf-Hirschhorn synd		0000122 /,	0005634 /,	0003677 // DNA binding // inferred from ele

0.101402	Ccdc127	coiled-coil domain containing 127			
0.173495	Sh3bp4	SH3-domain binding p	0006897 /,	0005634 /,	0005515 // protein binding // inferred from
0.030703	Luc7l	Luc7 homolog (S. cere	0045843 /,	0005634 /,	0005515 // protein binding // inferred from
0.006346	Hhip	Hedgehog-interacting	0007165 /,	0005576 /,	0003824 // catalytic activity // inferred from
0.159271	Leo1	Leo1, Paf1/RNA polyn	0006350 /,	0005634 /,	0005515 // protein binding // inferred from
0.134	Fam193a	family with sequence similarity 193, member A			
0.008543	Dgcr8	DiGeorge syndrome c	0007051 /,	0005622 /,	0003723 // RNA binding // inferred from ele
0.020289	Rbfox1	RNA bindir mRNA_prc	0006397 /,	0005634 /,	0000166 // nucleotide binding // inferred frc
0.397164	Dgkb	diacylglycerol kinase,	0007205 /,	0005624 /,	0000166 // nucleotide binding // inferred frc
0.241329	Rimkla	ribosomal modificatio	0006464 // protein m		0000166 // nucleotide binding // inferred frc
0.15704	Rad23a	RAD23a homolog (S. c	0006281 /,	0005634 /,	0003684 // damaged DNA binding // inferre
0.092308					
-0.16933	Sfmbt1	Scm-like with four mb	0045449 /,	0005634 // nucleus // inferred from electronic annotati	
0.022619	C630043F(	RIKEN cDNA C630043F03 gene			
-0.06993	Bcl9	B-cell CLL/lymphoma	0016055 /,	0005634 /,	0005515 // protein binding // inferred from
-0.13795	Rnf168	ring finger protein 16	0006281 /,	0000151 /,	0003682 // chromatin binding // inferred frc
0.253041	Tomm40l	translocas Nuclear_R	0006810 /,	0005739 /,	0008308 // voltage-gated anion channel acti
-0.45031	Cggbp1	CGG triplet repeat bin	0006350 /,	0005634 /,	0003677 // DNA binding // inferred from ele
0.019943	Tmcc1	transmembrane and coiled coil d	0016020 // membrane // inferred from electronic anno		
0.05831	9630025H	RIKEN cDNA 96300251	0006606 /,	0005634 /,	0005488 // binding // inferred from electror
0.220871	Zfp369	zinc finger protein 36	0006350 /,	0005622 /,	0003676 // nucleic acid binding // inferred fı
-0.07839	Xkr8	X Kell blood group precursor related family member 8 homolog			
0.268131	Gm88	predicted gene 88			
0.032014	Pcif1	PDX1 C-terminal inhibiting factor	0005634 /,	0005515 // protein binding // inferred from	
0.236396	Zfp192	zinc finger protein 19	0006350 /,	0005622 /,	0003676 // nucleic acid binding // inferred fı
-0.00845	Afap1l2	actin filament associated protein	0005737 // cytoplasm // inferred from electronic annot		
0.010088	Dpp9	dipeptidylpeptidase 9	0006508 /,	0005737 /,	0004177 // aminopeptidase activity // inferr
0.044	Tbc1d4	TBC1 domain family, r	0032313 /,	0005622 /,	0005096 // GTPase activator activity // infer
-0.1169	Zbed6	zinc finger, BED doma	0000122 /,	0005634 /,	0003676 // nucleic acid binding // inferred fı
0.048972	Brd9	bromodomain containing 9			
-0.29246	Maf	avian musculoaponeu	0001816 /,	0005634 /,	0003677 // DNA binding // inferred from dir
0.141039	Cep97	centrosomal protein 97		0005737 /,	0005515 // protein binding // inferred from
0.045472	Zmynd8	zinc finger, MYND-typ	0016310 // phosphor		0005515 // protein binding // inferred from
0.001663	Nav3	neuron navigator 3	0008152 /,	0005634 /,	0000166 // nucleotide binding // inferred frc
-0.44119	Lepr	leptin receptor	0001525 /,	0005576 /,	0004872 // receptor activity // inferred from
0.297207	5430407P	RIKEN cDNA 5430407P10 gene			
0.016681	Ubfd1	ubiquitin family domain containing 1			
0.24175	Trim28	tripartite motif-contai	0000122 /,	0000785 /,	0003700 // sequence-specific DNA binding tı
-0.06811	Fam126b	family with sequence similarity 126, member B			
-0.02065	B230217O	RIKEN cDNA B230217O12 gene			
-0.34436	Zeb2	zinc finger TGF_Beta_	0001755 /,	0005622 /,	0003676 // nucleic acid binding // inferred fı
-0.11832					
-0.15698	Myadml2	myeloid-associated differentiatio	0016020 // membrane // inferred from electronic anno		
0.510142	Adcy1	adenylate Calcium_re	0006171 /,	0005634 /,	0000166 // nucleotide binding // inferred frc
0.037994	Rnf130	ring finger protein 13	0006915 /,	0005737 /,	0004842 // ubiquitin-protein ligase activity /
-0.08037	4930422I0	RIKEN cDNA 4930422	0006355 /,	0005622 /,	0003676 // nucleic acid binding // inferred fı
-0.00666	Tmem231	transmembrane protein 231		0016020 // membrane // inferred from electronic anno	

-0.56162	Trpm3	transient receptor pot	0006810 /,	0016020 /,	0004872 // receptor activity // inferred from	
-0.10081	Zfp398	zinc finger protein 398	0006355 /,	0005622 /,	0003676 // nucleic acid binding // inferred fr	
-0.37037	Mthfd1l	methylenetetrahydro	0006730 /,	0005739 /,	0000166 // nucleotide binding // inferred fro	
-0.09596	0610010F0	RIKEN cDNA 0610010F05 gene				
0.4926	Nsg2	neuron specific gene 1	0007212 /,	0005768 /,	0050780 // dopamine receptor binding // inf	
0.138245	Mprip	myosin phosphatase Rho interac		0005737 /,	0003779 // actin binding // inferred from ele	
-0.28815	Dusp3	dual specificity phosp	0000188 /,	0001772 /,	0004721 // phosphoprotein phosphatase act	
-0.48838	Ncf1	neutrophil cytosolic fa	0001878 /,	0005624 /,	0005515 // protein binding // inferred from	
-0.02029	Ints8	integrator complex su	0016180 /,	0005634 /,	0005488 // binding // inferred from electron	
0.132039	Abcd2	ATP-binding cassette,	0006200 /,	0005739 /,	0000166 // nucleotide binding // inferred fro	
-0.30981	Flrt2	fibronectin leucine rich transmer		0016021 /,	0005515 // protein binding // inferred from	
0.05645	D030036P1	RIKEN cDNA D030036P13 gene				
0.08736						
0.1715	Rims4	regulating synaptic me	0006810 /,	0030054 // cell junction // inferred from electronic ann		
0.287966	Cdh26	cadherin-like 26	0007155 /,	0005886 /,	0005509 // calcium ion binding // inferred fr	
-0.18275	Wnt5a	wingless-re	Wnt_Signa	0001667 /,	0005576 /,	0004871 // signal transducer activity // infer
-0.30096	Rhobtb3	Rho-related BTB doma	0006810 /,	0005794 /,	0000166 // nucleotide binding // inferred fro	
-0.10839	Ppp4r1l-ps	protein phosphatase 4, regulatory subunit 1-like, pseudogene				
0.282796						
0.048251	Trpa1	transient receptor pot	0006810 /,	0016020 /,	0005216 // ion channel activity // inferred fr	
0.040799						
-0.23308	Tnrc6a	trinucleotide repeat c	0006417 /,	0000932 /,	0000166 // nucleotide binding // inferred fro	
-0.04706	D130037M	RIKEN cDNA D130037M23 gene				
0.009319						
-0.09784						
-0.18511	Per2	period hon	Circadian_	0006350 /,	0005634 /,	0004871 // signal transducer activity // infer
-0.42238						
-0.04113	Klf14	Kruppel-like factor 14	0006350 /,	0005622 /,	0003676 // nucleic acid binding // inferred fr	
0.057552	Nfkbiz	Nuclear factor of kapp	0006350 /,	0005634 // nucleus // inferred from direct assay /// 000		
0.1318						
-0.00556	Rgs12	regulator of G-protein	0007165 /,	0005737 /,	0004871 // signal transducer activity // infer	
-0.01265	Gpr151	G protein-coupled rec	0007165 /,	0005886 /,	0004871 // signal transducer activity // infer	
0.097733	Nkd2	naked cuticle 2 homol	0006810 /,	0005737 /,	0005509 // calcium ion binding // inferred fr	
-0.49694	Cdkl2	cyclin-dependent kina	0006468 /,	0005634 /,	0000166 // nucleotide binding // inferred fro	
-0.07238	2610316D0	RIKEN cDNA 2610316D01 gene				
0.138573	Osgep	O-sialoglycoprotein en	0006508 // proteolysi		0004175 // endopeptidase activity // inferre	
0.025903	Homer2	homer homolog 2 (Dr	0007216 /,	0005737 /,	0003779 // actin binding // inferred from dir	
-0.00494	Ppara	peroxisom	Nuclear_Re	0001666 /,	0005634 /,	0003677 // DNA binding // inferred from dir
0.32819	Rps15a	ribosomal protein S15	0006412 /,	0005622 /,	0003735 // structural constituent of ribosom	
-0.00709	Arntl	Aryl hydrocarbon rece	0000060 /,	0005634 /,	0003677 // DNA binding // inferred from phy	
-0.07108						
-0.11095	Mfsd11	major facilitator superfamily dom		0016020 // membrane // inferred from electronic anno		
0.17842	Etaa1	Ewing's tumor-associated antiger		0005737 // cytoplasm // inferred from electronic annot		
-0.30057	Ascc3	activating signal coint	0008152 /,	0005622 /,	0003676 // nucleic acid binding // inferred fr	
0.006519	4833442J1	RIKEN cDNA 4833442.	0006479 /,	0005737 /,	0008276 // protein methyltransferase activit	
-0.1824	Trpm3	transient receptor pot	0006810 /,	0016020 /,	0004872 // receptor activity // inferred from	
0.107003	Camta1	calmodulin binding tra	0006350 /,	0005634 /,	0005516 // calmodulin binding // inferred fr	

-0.05877	Rabif	RAB interacting factor	0006810 // transport	0005085 // guanyl-nucleotide exchange fact	
-0.22286	D7Wsu130	DNA segment, Chr 7, Wayne State University 130, expressed			
0.107584					
-0.04385					
-0.0429	Ipo7	importin 7	0006606 //	0005634 //	0005488 // binding // inferred from electron
-0.05124					
-0.02531	Me2	malic enzyme 2, NAD(	0006108 //	0005739 //	0003824 // catalytic activity // inferred from
-0.08465	Flnb	filamin, beta	0007519 //	0001725 //	0003779 // actin binding // inferred from ele
0.141229	AW04620C	expressed sequence AW046200 /// hypothetical LOC100502619			
-0.02565					
-0.12999	Sox4	SRY-box containing ge	0001666 //	0005634 //	0003677 // DNA binding // inferred from ele
0.148494	Med6	mediator of RNA poly	0006350 //	0005634 //	0003677 // DNA binding // inferred from dir
-0.00274	E130006N:	RIKEN cDNA E130006N16 gene			
-0.08524	Clic5	chloride intracellular c	0006810 //	0005626 //	0005216 // ion channel activity // inferred fr
-0.01519	1700027FC	RIKEN cDNA 1700027F06 gene			
0.070636	Kcnq3	potassium voltage-gat	0006810 //	0008076 //	0005216 // ion channel activity // inferred fr
-0.1215	Kdm2a	lysine (K)-specific dem	0006350 //	0005634 //	0003677 // DNA binding // inferred from ele
-0.54109	Abra	actin-binding Rho acti	0000060 //	0005737 //	0003713 // transcription coactivator activity
0.381274	Ntm	neurotrimin	0007155 //	0005615 //	0070492 // oligosaccharide binding // not re
-0.02041	Pde10a	phosphodiesterase 1C	0006198 //	0005624 //	0000166 // nucleotide binding // inferred fro
-0.0369					
0.032713	Gm4793	predicted gene 4793			
-0.16742	8030447M	RIKEN cDNA 8030447M02 gene			
0.004045					
0.065191	Depdc5	DEP domain containin	0023034 // intracellular signaling pathway // inferred from electro		
-0.12523					
0.022222					
0.059309					
-0.06465					
-0.09608	Myo1b	myosin IB	0008152 //	0005903 //	0000166 // nucleotide binding // inferred fro
-0.01292					
0.243634					
0.017662	Gpr107	G protein-coupled rec	0007165 //	0016020 //	0004872 // receptor activity // inferred from
0.087291	Ap1m1	adaptor-related prote	0006810 //	0005794 //	0005515 // protein binding // inferred from
-0.22668	Mia3	melanoma inhibitory a	0002687 //	0005783 //	0005515 // protein binding // inferred from
-0.10796	Phb2	prohibitin 2	0006350 //	0005634 //	0004872 // receptor activity // inferred from
-0.00906					
-0.10423	Ttc3	tetratricopeptide repe	0006511 //	0005634 //	0004842 // ubiquitin-protein ligase activity /
0.396169	Syt7	synaptotagmin VII	0001778 //	0005624 //	0005215 // transporter activity // inferred fr
-0.00787	Hydin	hydrocephalus inducin	0007275 // multicellular organismal development // inferred from		
0.117128	Wrb	tryptophan rich basic protein		0005634 // nucleus // inferred from electronic annotati	
-0.10226	4933407C(	RIKEN cDNA 4933407C09 gene			
-0.19568	Kank2	KN motif and ankyrin	0051497 //	0005737 // cytoplasm // not recorded	
-0.2435	Nus1	nuclear undecaprenyl	0001525 //	0016020 //	0004872 // receptor activity // inferred from
-0.16788	Rfx7	regulatory factor X, 7	0006355 // regulation		0003677 // DNA binding // inferred from ele
0.151837	Ppip5k1	diphosphoinositol per	0006020 //	0005737 //	0000166 // nucleotide binding // inferred fro
-0.12533	Esr1	estrogen r(Nuclear_R(	0001547 //	0005634 //	0003677 // DNA binding // not recorded ///

-0.46003	Hsd17b13	hydroxysteroid (17-be	0008152 /,	0005576 /,	0003824 // catalytic activity // inferred from	

om electronic annotation /// 0001948 // glycoprotein binding // not recorded /// 0001948 // glycoprotein bindi
ranscription factor activity // inferred from direct assay /// 0003824 // catalytic activity // inferred from electro
factor activity // inferred from electronic annotation /// 0005515 // protein binding // inferred from physical in
/ inferred from electronic annotation /// 0004221 // ubiquitin thiolesterase activity // inferred from sequence o
physical interaction /// 0019787 // small conjugating protein ligase activity // inferred from electronic annotati
om electronic annotation /// 0003676 // nucleic acid binding // inferred from electronic annotation /// 000367
/ inferred from direct assay
electronic annotation /// 0004806 // triglyceride lipase activity // inferred from electronic annotation /// 0005
ctronic annotation /// 0016787 // hydrolase activity // inferred from electronic annotation /// 0016820 // hydro
ty // not recorded /// 0004768 // stearoyl-CoA 9-desaturase activity // inferred from electronic annotation /// 0
direct assay /// 0005173 // stem cell factor receptor binding // inferred from direct assay /// 0005173 // stem
direct assay /// 0005173 // stem cell factor receptor binding // inferred from direct assay /// 0005173 // stem
/// 0016020 // membrane // inferred from electronic annotation /// 0016021 // integral to membrane // infer
om electronic annotation /// 0003824 // catalytic activity // inferred from electronic annotation /// 0004329 //
om electronic annotation /// 0003676 // nucleic acid binding // inferred from electronic annotation /// 0003723
electronic annotation /// 0017056 // structural constituent of nuclear pore // inferred from electronic annotati
ed from electronic annotation /// 0005159 // insulin-like growth factor receptor binding // inferred from physic
rom electronic annotation /// 0005539 // glycosaminoglycan binding // inferred from direct assay /// 0008201 /
inferred from electronic annotation
rom electronic annotation /// 0003677 // DNA binding // inferred from electronic annotation /// 0005515 // pro
/// 0005178 // integrin binding // inferred from electronic annotation /// 0050839 // cell adhesion molecule bi
om sequence or structural similarity /// 0005515 // protein binding // inferred from electronic annotation
om electronic annotation /// 0005515 // protein binding // inferred from electronic annotation /// 0005524 // A
ect assay /// 0003677 // DNA binding // inferred from electronic annotation /// 0003700 // sequence-specific D
om electronic annotation /// 0005515 // protein binding // inferred from electronic annotation /// 0005524 // A
red from electronic annotation /// 0005097 // Rab GTPase activator activity // inferred from electronic annotati
electronic annotation /// 0005515 // protein binding // inferred from physical interaction /// 0005516 // calmo
n electronic annotation /// 0031700 // adrenomedullin receptor binding // not recorded
ctronic annotation /// 0008270 // zinc ion binding // inferred from electronic annotation /// 0046872 // metal i
ferred from electronic annotation
// inferred from direct assay /// 0004720 // protein-lysine 6-oxidase activity // not recorded /// 0004720 // prot
ic annotation /// 0042803 // protein homodimerization activity // not recorded
from electronic annotation /// 0001972 // retinoic acid binding // inferred from sequence or structural similarit

om electronic annotation /// 0003676 // nucleic acid binding // inferred from electronic annotation /// 0003723
tation /// 0016021 // integral to membrane // inferred from electronic annotation
om electronic annotation /// 0003824 // catalytic activity // inferred from electronic annotation /// 0004637 //
electronic annotation
electronic annotation /// 0003979 // UDP-glucose 6-dehydrogenase activity // not recorded /// 0003979 // UD
m electronic annotation
5764 // lysosome // inferred from direct assay /// 0005764 // lysosome // not recorded /// 0005764 // lysosome
5764 // lysosome // inferred from direct assay /// 0005764 // lysosome // not recorded /// 0005764 // lysosome
020 // membrane // inferred from electronic annotation /// 0016021 // integral to membrane // inferred from
om electronic annotation /// 0005215 // transporter activity // not recorded /// 0005524 // ATP binding // infer
tation /// 0016021 // integral to membrane // inferred from electronic annotation
de-protein glycotransferase activity // inferred from electronic annotation /// 0005515 // protein binding // infe
on /// 0005737 // cytoplasm // inferred from electronic annotation
physical interaction /// 0005515 // protein binding // inferred from electronic annotation /// 0016301 // kinase
se activity // inferred from electronic annotation /// 0016853 // isomerase activity // inferred from electronic an
om electronic annotation /// 0004672 // protein kinase activity // inferred from electronic annotation /// 00046
red from sequence or structural similarity /// 0004622 // lysophospholipase activity // inferred from electronic
membrane transporter activity // inferred from electronic annotation /// 0005464 // UDP-xylose transmembran
membrane transporter activity // inferred from electronic annotation /// 0005464 // UDP-xylose transmembran
om electronic annotation /// 0003676 // nucleic acid binding // inferred from electronic annotation
electronic annotation /// 0015171 // amino acid transmembrane transporter activity // not recorded /// 00151
physical interaction /// 0005515 // protein binding // inferred from electronic annotation /// 0008022 // protei
ect assay /// 0003677 // DNA binding // not recorded /// 0003677 // DNA binding // inferred from electronic an
ferred from electronic annotation /// 0005201 // extracellular matrix structural constituent // inferred from dire
om electronic annotation /// 0003676 // nucleic acid binding // inferred from electronic annotation /// 0003677
om electronic annotation /// 0000287 // magnesium ion binding // inferred from electronic annotation /// 0004
om electronic annotation /// 0000287 // magnesium ion binding // inferred from electronic annotation /// 0004
om electronic annotation /// 0003723 // RNA binding // inferred from direct assay /// 0003723 // RNA binding /
om electronic annotation /// 0003779 // actin binding // inferred from direct assay /// 0004672 // protein kinas
rom electronic annotation /// 0005515 // protein binding // inferred from physical interaction /// 0005518 // co
ase activity // inferred from direct assay /// 0004014 // adenosylmethionine decarboxylase activity // not recor
physical interaction
/ inferred from electronic annotation /// 0008233 // peptidase activity // inferred from electronic annotation //
om electronic annotation /// 0004672 // protein kinase activity // inferred from electronic annotation /// 00046
physical interaction /// 0005515 // protein binding // inferred from electronic annotation /// 0046982 // protei
physical interaction /// 0005515 // protein binding // inferred from electronic annotation /// 0046982 // protei
om sequence or structural similarity
ic annotation /// 0005515 // protein binding // inferred from physical interaction /// 0005515 // protein bindin
5634 // nucleus // inferred from electronic annotation /// 0005730 // nucleolus // inferred from direct assay //
om electronic annotation /// 0001786 // phosphatidylserine binding // inferred from electronic annotation /// 0
ect assay /// 0003677 // DNA binding // inferred from electronic annotation /// 0003700 // sequence-specific D
ect assay /// 0003677 // DNA binding // inferred from electronic annotation /// 0003700 // sequence-specific D
om electronic annotation /// 0016757 // transferase activity, transferring glycosyl groups // inferred from elect
electronic annotation
om electronic annotation /// 0005515 // protein binding // inferred from physical interaction /// 0005515 // pro
electronic annotation
sporter activity // not recorded /// 0000064 // L-ornithine transmembrane transporter activity // inferred from

ıibitor activity // inferred from direct assay /// 0004864 // phosphoprotein phosphatase inhibitor activity // infe
ıic annotation
physical interaction /// 0005515 // protein binding // inferred from electronic annotation /// 0008017 // microt
:ivity // inferred from electronic annotation /// 0005044 // scavenger receptor activity // inferred from electron
red from direct assay /// 0004857 // enzyme inhibitor activity // inferred from sequence or structural similarity
atidic acid receptor activity // inferred from direct assay /// 0001619 // lysosphingolipid and lysophosphatidic a
om electronic annotation /// 0004672 // protein kinase activity // inferred from electronic annotation /// 00047
:ctronic annotation /// 0003700 // sequence-specific DNA binding transcription factor activity // inferred from e
tivity // inferred from electronic annotation /// 0004725 // protein tyrosine phosphatase activity // inferred fro
ity // inferred from electronic annotation /// 0008233 // peptidase activity // inferred from electronic annotatio
0004784 // superoxide dismutase activity // inferred from direct assay /// 0004784 // superoxide dismutase act
tation /// 0016021 // integral to membrane // inferred from electronic annotation
tation /// 0016021 // integral to membrane // inferred from electronic annotation
rom electronic annotation /// 0031683 // G-protein beta/gamma-subunit binding // inferred from direct assay
/ inferred from direct assay /// 0005515 // protein binding // inferred from physical interaction /// 0005515 //
ecorded /// 0004091 // carboxylesterase activity // inferred from electronic annotation /// 0004622 // lysophos
om electronic annotation /// 0004666 // prostaglandin-endoperoxide synthase activity // inferred from electron
physical interaction /// 0008200 // ion channel inhibitor activity // inferred from direct assay /// 0030550 // ace
se activity // inferred from electronic annotation /// 0016787 // hydrolase activity // inferred from electronic an
om electronic annotation /// 0000287 // magnesium ion binding // inferred from direct assay /// 0000287 // ma
ıibitor activity // inferred from electronic annotation
m electronic annotation /// 0017110 // nucleoside-diphosphatase activity // inferred from electronic annotation
ecorded /// 0004177 // aminopeptidase activity // inferred from electronic annotation /// 0005515 // protein bi
ecorded /// 0004177 // aminopeptidase activity // inferred from electronic annotation /// 0005515 // protein bi
ecorded /// 0004497 // monooxygenase activity // inferred from electronic annotation /// 0004499 // flavin-cor
tation /// 0016021 // integral to membrane // inferred from electronic annotation
rom electronic annotation /// 0015293 // symporter activity // inferred from electronic annotation /// 0015377
electronic annotation /// 0031625 // ubiquitin protein ligase binding // inferred from electronic annotation
or beta receptor binding // inferred from electronic annotation /// 0005160 // transforming growth factor beta
:ctronic annotation /// 0005488 // binding // inferred from electronic annotation
ıic annotation /// 0016020 // membrane // inferred from electronic annotation
:ctronic annotation /// 0005509 // calcium ion binding // inferred from electronic annotation
ıot recorded /// 0000146 // microfilament motor activity // inferred from electronic annotation /// 0000146 //
0003677 // DNA binding // inferred from electronic annotation /// 0003700 // sequence-specific DNA binding t
ıic annotation /// 0005515 // protein binding // inferred from physical interaction
om electronic annotation /// 0005524 // ATP binding // inferred from electronic annotation /// 0017111 // nucl
om electronic annotation /// 0001948 // glycoprotein binding // not recorded /// 0001948 // glycoprotein bindi
rom electronic annotation
ty // inferred from electronic annotation /// 0004843 // ubiquitin-specific protease activity // inferred from seq
electronic annotation /// 0046983 // protein dimerization activity // not recorded /// 0046983 // protein dimer
om electronic annotation /// 0004674 // protein serine/threonine kinase activity // inferred from direct assay //
red from electronic annotation /// 0005096 // GTPase activator activity // inferred from sequence or structural
red from electronic annotation /// 0004872 // receptor activity // inferred from electronic annotation /// 0005
itor activity // inferred from electronic annotation /// 0030414 // peptidase inhibitor activity // inferred from el
rom electronic annotation /// 0005515 // protein binding // inferred from physical interaction /// 0008511 // so
electronic annotation /// 0005160 // transforming growth factor beta receptor binding // inferred from electro
ıic annotation /// 0016020 // membrane // inferred from electronic annotation /// 0016021 // integral to meml

/ inferred from electronic annotation /// 0004861 // cyclin-dependent protein kinase inhibitor activity // inferre
rom electronic annotation /// 0046872 // metal ion binding // inferred from electronic annotation
physical interaction /// 0005515 // protein binding // inferred from electronic annotation
physical interaction /// 0008270 // zinc ion binding // inferred from electronic annotation /// 0016787 // hydrol
electronic annotation /// 0005488 // binding // inferred from electronic annotation /// 0008270 // zinc ion bin
electronic annotation /// 0008378 // galactosyltransferase activity // inferred from electronic annotation /// 00
om electronic annotation /// 0005525 // GTP binding // inferred from electronic annotation
electronic annotation /// 0004607 // phosphatidylcholine-sterol O-acyltransferase activity // inferred from ele
electronic annotation /// 0004607 // phosphatidylcholine-sterol O-acyltransferase activity // inferred from ele
sosome // inferred from electronic annotation /// 0005765 // lysosomal membrane // inferred from electronic a
electronic annotation /// 0008375 // acetylglucosaminyltransferase activity // inferred from electronic annotati
or activity // traceable author statement /// 0046872 // metal ion binding // inferred from electronic annotation
rom electronic annotation /// 0008047 // enzyme activator activity // not recorded /// 0008047 // enzyme activa
ctronic annotation /// 0003700 // sequence-specific DNA binding transcription factor activity // inferred from e
electronic annotation /// 0016787 // hydrolase activity // inferred from electronic annotation
electronic annotation
electronic annotation /// 0008009 // chemokine activity // inferred from electronic annotation
rom electronic annotation /// 0005244 // voltage-gated ion channel activity // inferred from electronic annotati
om electronic annotation /// 0001540 // beta-amyloid binding // inferred from electronic annotation /// 00036
l /// 0005102 // receptor binding // inferred from electronic annotation /// 0046872 // metal ion binding // infe
tivity // inferred from electronic annotation /// 0008233 // peptidase activity // inferred from electronic annotat
/ inferred from direct assay /// 0000253 // 3-keto sterol reductase activity // inferred from electronic annotatio
om electronic annotation /// 0003713 // transcription coactivator activity // inferred from sequence or structura
om electronic annotation /// 0003713 // transcription coactivator activity // inferred from sequence or structura
electronic annotation /// 0008009 // chemokine activity // not recorded /// 0008009 // chemokine activity // i
om electronic annotation /// 0003676 // nucleic acid binding // inferred from electronic annotation /// 0003723
nic annotation /// 0005515 // protein binding // inferred from electronic annotation /// 0008565 // protein tran
physical interaction /// 0030295 // protein kinase activator activity // not recorded
ect assay /// 0003677 // DNA binding // inferred from electronic annotation /// 0005515 // protein binding // ir
rom electronic annotation /// 0005277 // acetylcholine transmembrane transporter activity // not recorded /// 0
om electronic annotation /// 0003676 // nucleic acid binding // inferred from electronic annotation /// 0003723
ctronic annotation /// 0003700 // sequence-specific DNA binding transcription factor activity // inferred from e
ferred from electronic annotation
or activity // inferred from electronic annotation /// 0005089 // Rho guanyl-nucleotide exchange factor activity
physical interaction /// 0008270 // zinc ion binding // inferred from electronic annotation /// 0046872 // metal
physical interaction /// 0008270 // zinc ion binding // inferred from electronic annotation /// 0046872 // metal
ctronic annotation /// 0003700 // sequence-specific DNA binding transcription factor activity // inferred from e
rom electronic annotation /// 0003677 // DNA binding // inferred from sequence or structural similarity /// 000
physical interaction /// 0005515 // protein binding // inferred from electronic annotation /// 0046875 // ephrin
physical interaction /// 0005515 // protein binding // inferred from electronic annotation /// 0046875 // ephrin
nic annotation
om electronic annotation /// 0003676 // nucleic acid binding // inferred from electronic annotation /// 0003723
m electronic annotation /// 0008234 // cysteine-type peptidase activity // inferred from electronic annotation /
sporter activity // inferred from electronic annotation /// 0015179 // L-amino acid transmembrane transporter
om electronic annotation /// 0004222 // metalloendopeptidase activity // not recorded /// 0004222 // metalloe
om electronic annotation /// 0003924 // GTPase activity // inferred from sequence or structural similarity /// 00
rom electronic annotation /// 0003677 // DNA binding // inferred from electronic annotation /// 0003729 // mR

ɔn
rom electronic annotation /// 0003677 // DNA binding // inferred from direct assay /// 0005515 // protein bindi
d from electronic annotation /// 0005515 // protein binding // inferred from physical interaction /// 0005515 //
nic annotation /// 0005578 // proteinaceous extracellular matrix // inferred from electronic annotation /// 000!
ctronic annotation /// 0005488 // binding // inferred from electronic annotation /// 0005515 // protein binding
TPase activity // not recorded /// 0005391 // sodium:potassium-exchanging ATPase activity // inferred from ele
ɔm electronic annotation /// 0004672 // protein kinase activity // inferred from electronic annotation /// 00047
electronic annotation
ed from electronic annotation /// 0005509 // calcium ion binding // inferred from electronic annotation /// 000!
nic annotation /// 0005578 // proteinaceous extracellular matrix // inferred from electronic annotation
otation
ctronic annotation /// 0031072 // heat shock protein binding // inferred from electronic annotation /// 0046872
rred from electronic annotation /// 0004872 // receptor activity // inferred from electronic annotation /// 0004!
rred from electronic annotation /// 0004872 // receptor activity // inferred from electronic annotation /// 0004!
nic annotation
ctronic annotation /// 0003700 // sequence-specific DNA binding transcription factor activity // inferred from d
ctronic annotation /// 0003700 // sequence-specific DNA binding transcription factor activity // inferred from d
physical interaction /// 0005515 // protein binding // inferred from electronic annotation /// 0008022 // protei
erred from electronic annotation /// 0016740 // transferase activity // inferred from electronic annotation /// 0(
physical interaction /// 0017049 // GTP-Rho binding // inferred from electronic annotation
otation /// 0005737 // cytoplasm // inferred from electronic annotation /// 0005815 // microtubule organizing
ctronic annotation /// 0003700 // sequence-specific DNA binding transcription factor activity // inferred from d
ferred from electronic annotation
or activity // inferred from sequence or structural similarity /// 0005085 // guanyl-nucleotide exchange factor a
ctronic annotation /// 0003700 // sequence-specific DNA binding transcription factor activity // inferred from e
rom electronic annotation /// 0005515 // protein binding // inferred from electronic annotation /// 0008270 //
ter activity // inferred from electronic annotation
ɔm electronic annotation /// 0001948 // glycoprotein binding // not recorded /// 0004672 // protein kinase acti
ect assay /// 0003677 // DNA binding // not recorded /// 0003677 // DNA binding // inferred from electronic an
ctronic annotation /// 0003700 // sequence-specific DNA binding transcription factor activity // not recorded //
/ inferred from mutant phenotype /// 0004800 // thyroxine 5'-deiodinase activity // not recorded /// 0004800 /
inferred from electronic annotation /// 0019861 // flagellum // inferred from electronic annotation /// 003141(
ɔm electronic annotation /// 0004672 // protein kinase activity // inferred from electronic annotation /// 00046
ı electronic annotation
rom electronic annotation
ecorded /// 0004497 // monooxygenase activity // inferred from electronic annotation /// 0004509 // steroid 2
ecorded /// 0004497 // monooxygenase activity // inferred from electronic annotation /// 0004509 // steroid 2
/ // inferred from electronic annotation /// 0005515 // protein binding // inferred from electronic annotation //
tivity // inferred from electronic annotation /// 0004725 // protein tyrosine phosphatase activity // not recorde
om electronic annotation
ɔm electronic annotation /// 0005509 // calcium ion binding // inferred from electronic annotation /// 0005515
om electronic annotation /// 0005272 // sodium channel activity // inferred from direct assay /// 0005272 // sc
ctronic annotation /// 0005789 // endoplasmic reticulum membrane // inferred from electronic annotation /// (
eceptor binding // inferred from direct assay /// 0005161 // platelet-derived growth factor receptor binding // ir
ı electronic annotation
t recorded /// 0005044 // scavenger receptor activity // inferred from electronic annotation
ɔm electronic annotation /// 0000287 // magnesium ion binding // not recorded /// 0000287 // magnesium ion

physical interaction /// 0005515 // protein binding // inferred from electronic annotation
om electronic annotation /// 0004672 // protein kinase activity // inferred from electronic annotation /// 00046
physical interaction /// 0005515 // protein binding // inferred from electronic annotation /// 0008270 // zinc io
direct assay /// 0005125 // cytokine activity // inferred from electronic annotation /// 0005515 // protein bind
ctronic annotation /// 0005200 // structural constituent of cytoskeleton // inferred from electronic annotation
// inferred from electronic annotation /// 0016491 // oxidoreductase activity // inferred from electronic annot
otein mannosyltransferase activity // inferred from sequence or structural similarity /// 0004582 // dolichyl-pho
electronic annotation
nic annotation /// 0005794 // Golgi apparatus // inferred from electronic annotation
physical interaction /// 0005515 // protein binding // inferred from electronic annotation /// 0016455 // RNA p
electronic annotation
red from electronic annotation /// 0005097 // Rab GTPase activator activity // inferred from electronic annotati
ic annotation /// 0005529 // sugar binding // inferred from electronic annotation
ding // not recorded /// 0001850 // complement component C3a binding // inferred from electronic annotatio
om electronic annotation /// 0004672 // protein kinase activity // not recorded /// 0004672 // protein kinase ac
rred from electronic annotation /// 0005125 // cytokine activity // inferred from direct assay /// 0005125 // cyt
physical interaction /// 0005515 // protein binding // inferred from electronic annotation /// 0008553 // hydrog
electronic annotation /// 0008270 // zinc ion binding // inferred from electronic annotation /// 0046872 // met
on /// 0005737 // cytoplasm // inferred from electronic annotation
om electronic annotation /// 0005524 // ATP binding // inferred from electronic annotation /// 0016887 // ATPa
rom electronic annotation /// 0003677 // DNA binding // inferred from electronic annotation /// 0008270 // zin
electronic annotation /// 0004252 // serine-type endopeptidase activity // inferred from electronic annotation
inferred from genetic interaction /// 0005515 // protein binding // inferred from physical interaction /// 00055
/// 0030017 // sarcomere // inferred from direct assay /// 0031674 // I band // inferred from direct assay
not recorded /// 0000166 // nucleotide binding // inferred from electronic annotation /// 0003774 // motor acti
ctronic annotation /// 0003701 // RNA polymerase I transcription factor activity // inferred from electronic ann
ctronic annotation /// 0005200 // structural constituent of cytoskeleton // traceable author statement /// 000
electronic annotation /// 0004553 // hydrolase activity, hydrolyzing O-glycosyl compounds // inferred from ele
/ inferred from electronic annotation /// 0005515 // protein binding // inferred from electronic annotation ///

tation /// 0016021 // integral to membrane // inferred from electronic annotation

ctronic annotation /// 0003682 // chromatin binding // inferred from direct assay /// 0003700 // sequence-spe
om electronic annotation /// 0003676 // nucleic acid binding // inferred from electronic annotation /// 0003677
electronic annotation /// 0008565 // protein transporter activity // inferred from electronic annotation /// 001
om electronic annotation /// 0005488 // binding // inferred from electronic annotation /// 0005550 // pheromo
physical interaction /// 0005515 // protein binding // inferred from electronic annotation
ctronic annotation /// 0003743 // translation initiation factor activity // inferred from electronic annotation ///
ce or structural similarity /// 0005576 // extracellular region // inferred from electronic annotation
ation
ation
om electronic annotation /// 0005887 // integral to plasma membrane // inferred from sequence or structural s
erred from electronic annotation
recorded /// 0004623 // phospholipase A2 activity // inferred from electronic annotation /// 0005509 // calciu
/ inferred from direct assay /// 0005515 // protein binding // inferred from electronic annotation /// 0008270 /
tation /// 0016021 // integral to membrane // inferred from electronic annotation /// 0016021 // integral to me
ronic annotation

ı electronic annotation /// 0030215 // semaphorin receptor binding // inferred from physical interaction
30 // synaptonemal complex assembly // inferred from mutant phenotype /// 0007140 // male meiosis // inferr
ı electronic annotation /// 0004252 // serine-type endopeptidase activity // inferred from electronic annotation
ranscription factor activity // inferred from electronic annotation
red from electronic annotation /// 0004872 // receptor activity // inferred from electronic annotation /// 0004
ferred from mutant phenotype /// 0046556 // alpha-N-arabinofuranosidase activity // inferred from electronic a
physical interaction /// 0005515 // protein binding // inferred from electronic annotation /// 0019905 // syntax
or receptor binding // not recorded /// 0005515 // protein binding // inferred from physical interaction /// 0005
om electronic annotation /// 0005515 // protein binding // inferred from electronic annotation /// 0015132 // p
ctronic annotation /// 0005515 // protein binding // inferred from electronic annotation /// 0016787 // hydrola
om electronic annotation /// 0003676 // nucleic acid binding // inferred from electronic annotation /// 0003677
ic annotation /// 0005515 // protein binding // inferred from electronic annotation
/ inferred from direct assay /// 0004842 // ubiquitin-protein ligase activity // inferred from genetic interaction /
ect assay /// 0003677 // DNA binding // not recorded /// 0003677 // DNA binding // inferred from electronic an
0003677 // DNA binding // inferred from electronic annotation /// 0003700 // sequence-specific DNA binding tı
ı electronic annotation /// 0005488 // binding // inferred from electronic annotation /// 0005518 // collagen bir
on
red from sequence or structural similarity /// 0005515 // protein binding // inferred from electronic annotation
red from electronic annotation /// 0005506 // iron ion binding // inferred from electronic annotation /// 00083
red from electronic annotation /// 0005506 // iron ion binding // inferred from electronic annotation /// 00083
red from electronic annotation /// 0005515 // protein binding // inferred from physical interaction
om electronic annotation /// 0004672 // protein kinase activity // inferred from direct assay /// 0004672 // prot
ivity // inferred from direct assay /// 0004190 // aspartic-type endopeptidase activity // inferred from electroni
electronic annotation
tation /// 0016021 // integral to membrane // inferred from electronic annotation
n electronic annotation /// 0031708 // endothelin B receptor binding // inferred from electronic annotation
physical interaction /// 0005525 // GTP binding // inferred from electronic annotation
ect assay /// 0003677 // DNA binding // not recorded /// 0003677 // DNA binding // inferred from electronic an
ı physical interaction /// 0004872 // receptor activity // inferred from electronic annotation /// 0005488 // bind
ı electronic annotation /// 0004890 // GABA-A receptor activity // not recorded /// 0004890 // GABA-A recepto
ı electronic annotation /// 0005515 // protein binding // inferred from electronic annotation /// 0042277 // pep
ctronic annotation /// 0016787 // hydrolase activity // inferred from electronic annotation /// 0046872 // meta
om electronic annotation /// 0005515 // protein binding // inferred from electronic annotation /// 0005524 // A
ı electronic annotation /// 0004896 // cytokine receptor activity // inferred from electronic annotation /// 0004
ı electronic annotation /// 0004252 // serine-type endopeptidase activity // inferred from electronic annotation
om electronic annotation /// 0003676 // nucleic acid binding // inferred from electronic annotation /// 0003723
rom electronic annotation /// 0003677 // DNA binding // inferred from direct assay /// 0003677 // DNA binding
able author statement /// 0005509 // calcium ion binding // inferred from electronic annotation
ed from direct assay /// 0042605 // peptide antigen binding // inferred from mutant phenotype
om electronic annotation /// 0005215 // transporter activity // inferred from electronic annotation /// 0005324
rom electronic annotation /// 0003677 // DNA binding // inferred from electronic annotation /// 0008270 // zin
ı electronic annotation /// 0004114 // 3',5'-cyclic-nucleotide phosphodiesterase activity // inferred from electro
rom electronic annotation /// 0003677 // DNA binding // inferred from electronic annotation /// 0003700 // sec
ı electronic annotation /// 0004721 // phosphoprotein phosphatase activity // inferred from electronic annotati
a-2,8-sialyltransferase activity // inferred from direct assay /// 0008373 // sialyltransferase activity // inferred fr
om electronic annotation /// 0004672 // protein kinase activity // inferred from electronic annotation /// 00047
ı electronic annotation /// 0005488 // binding // inferred from electronic annotation /// 0005529 // sugar bindi

0003779 // actin binding // inferred from electronic annotation /// 0005102 // receptor binding // inferred fror
om electronic annotation /// 0004017 // adenylate kinase activity // inferred from electronic annotation /// 000
om electronic annotation /// 0005215 // transporter activity // inferred from electronic annotation /// 0005524
electronic annotation /// 0004806 // triglyceride lipase activity // not recorded /// 0004806 // triglyceride lipas
ctronic annotation /// 0005085 // guanyl-nucleotide exchange factor activity // inferred from electronic annota
ect assay /// 0003677 // DNA binding // inferred from electronic annotation /// 0005515 // protein binding // ir
ic annotation /// 0005515 // protein binding // inferred from electronic annotation
from direct assay /// 0004708 // MAP kinase kinase activity // inferred from sequence or structural similarity //
electronic annotation
rom electronic annotation /// 0003677 // DNA binding // inferred from electronic annotation /// 0003700 // sec
physical interaction /// 0005515 // protein binding // inferred from electronic annotation
erred from electronic annotation
ctronic annotation
red from electronic annotation /// 0004872 // receptor activity // inferred from electronic annotation /// 0004
/ inferred from electronic annotation /// 0016740 // transferase activity // inferred from electronic annotation
om electronic annotation /// 0004127 // cytidylate kinase activity // not recorded /// 0004127 // cytidylate kina
om electronic annotation /// 0003676 // nucleic acid binding // inferred from electronic annotation /// 0003723
ctronic annotation /// 0005515 // protein binding // inferred from electronic annotation
physical interaction /// 0005529 // sugar binding // inferred from electronic annotation
electronic annotation /// 0005169 // neurotrophin TRKB receptor binding // not recorded /// 0005515 // prote
electronic annotation /// 0004896 // cytokine receptor activity // inferred from electronic annotation
y // not recorded /// 0004128 // cytochrome-b5 reductase activity // inferred from electronic annotation /// 00
ctronic annotation /// 0003700 // sequence-specific DNA binding transcription factor activity // inferred from e
red from electronic annotation /// 0004872 // receptor activity // inferred from electronic annotation /// 0004
eceptor binding // not recorded /// 0005111 // type 2 fibroblast growth factor receptor binding // inferred from
nic annotation /// 0005578 // proteinaceous extracellular matrix // inferred from direct assay /// 0005578 // pr
ctronic annotation /// 0003700 // sequence-specific DNA binding transcription factor activity // inferred from e
ctronic annotation /// 0003700 // sequence-specific DNA binding transcription factor activity // inferred from e
physical interaction /// 0005515 // protein binding // inferred from electronic annotation
nstituent // inferred from electronic annotation /// 0046332 // SMAD binding // inferred from physical interact
electronic annotation /// 0004301 // epoxide hydrolase activity // inferred from electronic annotation /// 0016
electronic annotation /// 0005547 // phosphatidylinositol-3,4,5-trisphosphate binding // inferred from electron
electronic annotation /// 0004573 // mannosyl-oligosaccharide glucosidase activity // inferred from electronic
rter activity // not recorded /// 0005385 // zinc ion transmembrane transporter activity // inferred from electrc
ctronic annotation /// 0003684 // damaged DNA binding // inferred from electronic annotation /// 0005515 //
ferred from electronic annotation /// 0005509 // calcium ion binding // inferred from electronic annotation ///
om sequence or structural similarity /// 0016020 // membrane // inferred from electronic annotation /// 00160
om electronic annotation
ssay /// 0005576 // extracellular region // inferred from electronic annotation
ctronic annotation /// 0003700 // sequence-specific DNA binding transcription factor activity // traceable autho
tivity // inferred from electronic annotation /// 0008233 // peptidase activity // inferred from electronic annota
om electronic annotation /// 0003676 // nucleic acid binding // inferred from electronic annotation /// 0003723
ate) O-methyltransferase activity // inferred from direct assay /// 0004719 // protein-L-isoaspartate (D-aspartat
ic annotation /// 0005515 // protein binding // inferred from physical interaction /// 0042169 // SH2 domain bi
ctronic annotation /// 0003697 // single-stranded DNA binding // inferred from electronic annotation /// 00037
ctronic annotation
electronic annotation

nic annotation /// 0005579 // membrane attack complex // inferred from electronic annotation /// 0005886 //
ctronic annotation /// 0015485 // cholesterol binding // not recorded /// 0015485 // cholesterol binding // infe
e transporter activity // inferred from electronic annotation /// 0005351 // sugar:hydrogen symporter activity /
electronic annotation /// 0016922 // ligand-dependent nuclear receptor binding // inferred from electronic anr
om electronic annotation
annotation /// 0016020 // membrane // inferred from electronic annotation /// 0016021 // integral to membra
ed from direct assay /// 0004497 // monooxygenase activity // inferred from electronic annotation /// 000449
electronic annotation /// 0004945 // angiotensin type II receptor activity // inferred from mutant phenotype /
m direct assay /// 0003713 // transcription coactivator activity // inferred from electronic annotation /// 00044
ctronic annotation /// 0005515 // protein binding // inferred from electronic annotation
m electronic annotation /// 0004672 // protein kinase activity // inferred from electronic annotation /// 00047

ctronic annotation
physical interaction /// 0005515 // protein binding // inferred from electronic annotation /// 0035091 // phosp
// inferred from electronic annotation /// 0005506 // iron ion binding // inferred from electronic annotation //
ctronic annotation /// 0005523 // tropomyosin binding // inferred from electronic annotation /// 0051011 // m
electronic annotation
y // inferred from electronic annotation /// 0005515 // protein binding // inferred from electronic annotation
y // inferred from electronic annotation /// 0005515 // protein binding // inferred from electronic annotation
nstituent // inferred from electronic annotation /// 0005515 // protein binding // inferred from physical interac
electronic annotation /// 0031432 // titin binding // inferred from electronic annotation /// 0070080 // titin Z d
/// 0004872 // receptor activity // inferred from electronic annotation /// 0005041 // low-density lipoprotein r
factor activity // inferred from electronic annotation /// 0005509 // calcium ion binding // inferred from electro
tation /// 0016021 // integral to membrane // inferred from electronic annotation
annotation /// 0005794 // Golgi apparatus // inferred from electronic annotation /// 0016020 // membrane //
not recorded /// 0008489 // UDP-galactose:glucosylceramide beta-1,4-galactosyltransferase activity // not recor
/// 0005509 // calcium ion binding // inferred from direct assay /// 0008270 // zinc ion binding // inferred from
ed from electronic annotation /// 0005506 // iron ion binding // inferred from electronic annotation /// 00090
electronic annotation
ctronic annotation /// 0003682 // chromatin binding // inferred from direct assay /// 0003700 // sequence-spe
electronic annotation /// 0004519 // endonuclease activity // inferred from electronic annotation /// 0004536
tation /// 0016021 // integral to membrane // inferred from electronic annotation
om electronic annotation /// 0015125 // bile acid transmembrane transporter activity // not recorded /// 0015
om electronic annotation /// 0005244 // voltage-gated ion channel activity // inferred from electronic annotati
om electronic annotation /// 0005515 // protein binding // inferred from physical interaction /// 0050840 // ex
m electronic annotation /// 0004666 // prostaglandin-endoperoxide synthase activity // inferred from electror
ect assay /// 0003677 // DNA binding // inferred from electronic annotation /// 0003700 // sequence-specific D
ic annotation /// 0005515 // protein binding // inferred from physical interaction /// 0005515 // protein bindin
physical interaction
// inferred from direct assay /// 0000293 // ferric-chelate reductase activity // inferred from mutant phenotype
ic annotation /// 0009986 // cell surface // not recorded /// 0016020 // membrane // inferred from electronic
ect assay /// 0003677 // DNA binding // inferred from electronic annotation /// 0005515 // protein binding // ir
tation /// 0016021 // integral to membrane // inferred from electronic annotation
atidic acid receptor activity // inferred from electronic annotation /// 0004871 // signal transducer activity // in
inferred from direct assay /// 0001600 // endothelin-B receptor activity // not recorded /// 0001600 // endoth
m electronic annotation /// 0000287 // magnesium ion binding // inferred from electronic annotation /// 0000
direct assay /// 0008201 // heparin binding // inferred from electronic annotation

ı electronic annotation /// 0004035 // alkaline phosphatase activity // inferred from direct assay /// 0004035 //
·om electronic annotation /// 0005515 // protein binding // inferred from physical interaction
electronic annotation /// 0008270 // zinc ion binding // inferred from electronic annotation /// 0046872 // met
ınstituent // inferred from direct assay /// 0005201 // extracellular matrix structural constituent // inferred from
electronic annotation /// 0005515 // protein binding // inferred from electronic annotation /// 0016213 // lino
'/ inferred from sequence or structural similarity /// 0005249 // voltage-gated potassium channel activity // infe
:tronic annotation /// 0005886 // plasma membrane // inferred from electronic annotation /// 0016020 // mem
ctronic annotation
' /// 0005739 // mitochondrion // inferred from electronic annotation /// 0005743 // mitochondrial inner memb
ɔm electronic annotation /// 0003676 // nucleic acid binding // inferred from electronic annotation /// 0003677
rom electronic annotation /// 0003723 // RNA binding // inferred from electronic annotation /// 0008270 // zin
ı electronic annotation /// 0016787 // hydrolase activity // inferred from electronic annotation /// 0016798 // h
' /// 0005739 // mitochondrion // not recorded
ing // inferred from electronic annotation /// 0004197 // cysteine-type endopeptidase activity // inferred from
ing // inferred from electronic annotation /// 0004197 // cysteine-type endopeptidase activity // inferred from
physical interaction /// 0005515 // protein binding // inferred from electronic annotation /// 0008022 // protei
ı electronic annotation /// 0004091 // carboxylesterase activity // inferred from electronic annotation /// 00167
physical interaction /// 0017147 // Wnt-protein binding // inferred from direct assay
physical interaction /// 0017147 // Wnt-protein binding // inferred from direct assay
electronic annotation
ɔm electronic annotation /// 0003824 // catalytic activity // inferred from electronic annotation /// 0004467 // l
ınstituent // inferred from electronic annotation /// 0005201 // extracellular matrix structural constituent // inf
ı electronic annotation /// 0016491 // oxidoreductase activity // inferred from electronic annotation /// 001715
ɔm electronic annotation /// 0003676 // nucleic acid binding // inferred from electronic annotation /// 0003723
nic annotation /// 0016020 // membrane // inferred from electronic annotation /// 0016021 // integral to mem
rom electronic annotation /// 0005515 // protein binding // inferred from electronic annotation /// 0008270 //
ɔm electronic annotation /// 0001948 // glycoprotein binding // not recorded /// 0004672 // protein kinase acti
' inferred from electronic annotation /// 0008233 // peptidase activity // inferred from electronic annotation //
:ivity // inferred from electronic annotation /// 0008889 // glycerophosphodiester phosphodiesterase activity //
ɔm electronic annotation /// 0003824 // catalytic activity // inferred from electronic annotation /// 0003963 // l
itor activity // inferred from electronic annotation
ecorded /// 0001671 // ATPase activator activity // inferred from electronic annotation /// 0001671 // ATPase a
:ctronic annotation /// 0003690 // double-stranded DNA binding // inferred from direct assay /// 0003700 // se
ɔm direct assay /// 0003682 // chromatin binding // inferred from electronic annotation /// 0005515 // protein l
tation /// 0016021 // integral to membrane // inferred from electronic annotation /// 0030133 // transport ves
ɔm electronic annotation /// 0003723 // RNA binding // inferred from electronic annotation /// 0004652 // poly
'/ inferred from electronic annotation /// 0008270 // zinc ion binding // inferred from electronic annotation
:ctronic annotation /// 0003899 // DNA-directed RNA polymerase activity // inferred from electronic annotation
ɔm electronic annotation /// 0000287 // magnesium ion binding // inferred from direct assay /// 0000287 // ma
:ctronic annotation /// 0003700 // sequence-specific DNA binding transcription factor activity // inferred from e
:ctronic annotation /// 0003700 // sequence-specific DNA binding transcription factor activity // inferred from e

nferred from electronic annotation /// 0004169 // dolichyl-phosphate-mannose-protein mannosyltransferase a
ɔm electronic annotation /// 0004672 // protein kinase activity // inferred from electronic annotation /// 00046
ı electronic annotation /// 0004633 // phosphopantothenoylcysteine decarboxylase activity // inferred from ele
ıic annotation

L-asparaginase activity // not recorded /// 0003948 // N4-(beta-N-acetylglucosaminyl)-L-asparaginase activity //
om electronic annotation /// 0003824 // catalytic activity // inferred from electronic annotation /// 0004030 //
: annotation /// 0005768 // endosome // inferred from electronic annotation /// 0005794 // Golgi apparatus //
from direct assay /// 0005488 // binding // inferred from electronic annotation /// 0005515 // protein binding ,
ed from electronic annotation /// 0004427 // inorganic diphosphatase activity // inferred from electronic annota
electronic annotation /// 0046872 // metal ion binding // inferred from electronic annotation
tation /// 0016021 // integral to membrane // inferred from electronic annotation
red from electronic annotation /// 0005515 // protein binding // inferred from physical interaction /// 0005515
om electronic annotation /// 0003676 // nucleic acid binding // inferred from electronic annotation /// 0003723
ic annotation /// 0005515 // protein binding // inferred from physical interaction /// 0016491 // oxidoreductas
ctronic annotation /// 0003711 // transcription elongation regulator activity // inferred from electronic annotat
tation /// 0016021 // integral to membrane // inferred from electronic annotation
om electronic annotation /// 0003676 // nucleic acid binding // inferred from electronic annotation /// 0003723
physical interaction /// 0008017 // microtubule binding // not recorded /// 0008017 // microtubule binding // i
red from electronic annotation /// 0004872 // receptor activity // inferred from electronic annotation /// 0004
nferred from electronic annotation /// 0008270 // zinc ion binding // inferred from electronic annotation /// 00

electronic annotation
om electronic annotation /// 0000287 // magnesium ion binding // not recorded /// 0001948 // glycoprotein bir
electronic annotation /// 0008270 // zinc ion binding // inferred from electronic annotation /// 0046872 // met
ation
ation

electronic annotation /// 0031072 // heat shock protein binding // inferred from electronic annotation /// 0051
on
om electronic annotation /// 0004672 // protein kinase activity // inferred from electronic annotation /// 00047

ranscription factor activity // inferred from direct assay /// 0005515 // protein binding // inferred from physical
om electronic annotation /// 0004672 // protein kinase activity // inferred from electronic annotation /// 00046
d from electronic annotation /// 0005515 // protein binding // inferred from electronic annotation
om electronic annotation /// 0004672 // protein kinase activity // inferred from electronic annotation /// 00047
/ not recorded /// 0000401 // open form four-way junction DNA binding // not recorded /// 0000402 // crossed
erred from electronic annotation /// 0051082 // unfolded protein binding // inferred from electronic annotatio
nic annotation
rom electronic annotation /// 0008270 // zinc ion binding // inferred from electronic annotation
ysical interaction /// 0003677 // DNA binding // not recorded /// 0003677 // DNA binding // inferred from elect
tation /// 0016021 // integral to membrane // inferred from electronic annotation

om electronic annotation /// 0003774 // motor activity // inferred from electronic annotation /// 0003779 // ac
ctronic annotation /// 0003700 // sequence-specific DNA binding transcription factor activity // inferred from d
ic annotation /// 0005515 // protein binding // inferred from electronic annotation

om sequence or structural similarity /// 0003713 // transcription coactivator activity // inferred from sequence c
ferred from electronic annotation
nase activity // inferred from sequence or structural similarity /// 0004597 // peptide-aspartate beta-dioxygenas
ranscription factor activity // inferred from electronic annotation

om electronic annotation /// 0005525 // GTP binding // inferred from electronic annotation
rom electronic annotation /// 0008270 // zinc ion binding // inferred from electronic annotation /// 0046872 //
ctronic annotation /// 0016817 // hydrolase activity, acting on acid anhydrides // inferred from electronic annot
rom electronic annotation /// 0008270 // zinc ion binding // inferred from electronic annotation /// 0046872 //
om electronic annotation /// 0004672 // protein kinase activity // inferred from electronic annotation /// 00046
electronic annotation /// 0005044 // scavenger receptor activity // inferred from electronic annotation /// 000
om electronic annotation /// 0004672 // protein kinase activity // inferred from electronic annotation /// 00046
electronic annotation /// 0004553 // hydrolase activity, hydrolyzing O-glycosyl compounds // inferred from ele
eable author statement /// 0004872 // receptor activity // inferred from electronic annotation /// 0005070 // SI
hosphatase activity // inferred from direct assay /// 0004725 // protein tyrosine phosphatase activity // inferre
om electronic annotation /// 0003924 // GTPase activity // not recorded /// 0005525 // GTP binding // inferred
ect assay /// 0003779 // actin binding // inferred from electronic annotation /// 0005515 // protein binding // i
ranscription factor activity // inferred from electronic annotation
rom electronic annotation /// 0005515 // protein binding // inferred from physical interaction /// 0005515 // pr
tivity // inferred from direct assay /// 0004721 // phosphoprotein phosphatase activity // inferred from electror
electronic annotation /// 0004896 // cytokine receptor activity // inferred from electronic annotation /// 0005
ctronic annotation /// 0003785 // actin monomer binding // inferred from sequence or structural similarity ///
om electronic annotation /// 0004672 // protein kinase activity // inferred from electronic annotation /// 00046

electronic annotation /// 0004630 // phospholipase D activity // not recorded /// 0004630 // phospholipase D
ect assay /// 0003677 // DNA binding // inferred from electronic annotation /// 0003700 // sequence-specific D
annotation /// 0005794 // Golgi apparatus // inferred from electronic annotation /// 0016020 // membrane //
ect assay /// 0003677 // DNA binding // inferred from electronic annotation /// 0003682 // chromatin binding /
om electronic annotation /// 0004672 // protein kinase activity // inferred from electronic annotation /// 00046
ity // inferred from electronic annotation /// 0008233 // peptidase activity // inferred from electronic annotatio
om electronic annotation /// 0003824 // catalytic activity // inferred from electronic annotation /// 0004484 //
led /// 0005509 // calcium ion binding // inferred from electronic annotation
electronic annotation /// 0004415 // hyalurononglucosaminidase activity // inferred from electronic annotatio
ement /// 0005886 // plasma membrane // inferred from electronic annotation /// 0016020 // membrane // inf
physical interaction /// 0005515 // protein binding // inferred from electronic annotation /// 0008134 // transc
inhibitor activity // inferred from electronic annotation /// 0004861 // cyclin-dependent protein kinase inhibito
electronic annotation /// 0008270 // zinc ion binding // inferred from electronic annotation /// 0016787 // hyc
/ inferred from electronic annotation /// 0005515 // protein binding // inferred from electronic annotation ///
nyltransferase activity // inferred from mutant phenotype /// 0003810 // protein-glutamine gamma-glutamyltr
red from electronic annotation /// 0005096 // GTPase activator activity // not recorded /// 0005096 // GTPase
electronic annotation /// 0004114 // 3',5'-cyclic-nucleotide phosphodiesterase activity // inferred from electro
om electronic annotation /// 0005244 // voltage-gated ion channel activity // inferred from electronic annotati
electronic annotation /// 0005515 // protein binding // inferred from physical interaction
electronic annotation /// 0005488 // binding // inferred from electronic annotation /// 0005529 // sugar bindi
direct assay /// 0005173 // stem cell factor receptor binding // inferred from direct assay /// 0005173 // stem
electronic annotation /// 0005515 // protein binding // inferred from physical interaction /// 0005515 // prote
tation /// 0016020 // membrane // inferred from electronic annotation /// 0031902 // late endosome membrar
erred from direct assay
annotation
electronic annotation
electronic annotation /// 0008415 // acyltransferase activity // inferred from electronic annotation /// 001674C
y // inferred from electronic annotation /// 0004579 // dolichyl-diphosphooligosaccharide-protein glycotransfe

om electronic annotation /// 0004672 // protein kinase activity // inferred from electronic annotation /// 00046
rom electronic annotation /// 0005515 // protein binding // inferred from electronic annotation /// 0008270 //
rom electronic annotation /// 0005515 // protein binding // inferred from electronic annotation /// 0008270 //
red from electronic annotation /// 0005515 // protein binding // inferred from electronic annotation
om electronic annotation /// 0003913 // DNA photolyase activity // inferred from electronic annotation /// 000
/ inferred from direct assay /// 0005515 // protein binding // inferred from physical interaction /// 0005515 //
C activity // inferred from electronic annotation /// 0004629 // phospholipase C activity // inferred from electr
electronic annotation /// 0004222 // metalloendopeptidase activity // inferred from electronic annotation ///
rom electronic annotation /// 0003677 // DNA binding // inferred from direct assay /// 0003677 // DNA binding
ation /// 0005783 // endoplasmic reticulum // inferred from electronic annotation /// 0005815 // microtubule
om electronic annotation /// 0003824 // catalytic activity // inferred from electronic annotation /// 0005102 //
ect assay /// 0003677 // DNA binding // inferred from electronic annotation /// 0003700 // sequence-specific D
ctronic annotation /// 0005515 // protein binding // inferred from electronic annotation /// 0008270 // zinc ior
electronic annotation /// 0008270 // zinc ion binding // inferred from electronic annotation /// 0046872 // met
tation /// 0016021 // integral to membrane // inferred from electronic annotation
electronic annotation

ctronic annotation /// 0005080 // protein kinase C binding // not recorded /// 0005080 // protein kinase C binc
electronic annotation /// 0003939 // L-iditol 2-dehydrogenase activity // inferred from direct assay /// 000393
electronic annotation /// 0008270 // zinc ion binding // inferred from electronic annotation /// 0016301 // kina
ctronic annotation /// 0009982 // pseudouridine synthase activity // inferred from electronic annotation
/// 0002020 // protease binding // inferred from electronic annotation /// 0005515 // protein binding // inferr
om electronic annotation /// 0003774 // motor activity // inferred from electronic annotation /// 0003779 // ac
om electronic annotation /// 0003677 // DNA binding // inferred from electronic annotation /// 0005515 // prot
ctronic annotation /// 0004518 // nuclease activity // inferred from electronic annotation /// 0004527 // exonu
om electronic annotation /// 0003676 // nucleic acid binding // inferred from electronic annotation /// 0003723
ferred from electronic annotation /// 0005200 // structural constituent of cytoskeleton // traceable author stat

ctronic annotation /// 0005083 // small GTPase regulator activity // inferred from electronic annotation /// 000
ic annotation /// 0005515 // protein binding // inferred from physical interaction /// 0005515 // protein bindin
electronic annotation /// 0004013 // adenosylhomocysteinase activity // inferred from electronic annotation /
electronic annotation /// 0008270 // zinc ion binding // inferred from electronic annotation
// inferred from electronic annotation /// 0008083 // growth factor activity // inferred from electronic annota
5634 // nucleus // inferred from electronic annotation /// 0005654 // nucleoplasm // inferred from electronic
transferase activity // not recorded /// 0001537 // N-acetylgalactosamine 4-O-sulfotransferase activity // inferr
om electronic annotation /// 0004672 // protein kinase activity // inferred from electronic annotation /// 00046

physical interaction /// 0005515 // protein binding // inferred from electronic annotation
electronic annotation
ctronic annotation /// 0003684 // damaged DNA binding // inferred from direct assay /// 0005515 // protein bi
ctronic annotation /// 0003735 // structural constituent of ribosome // inferred from electronic annotation ///
om electronic annotation /// 0003924 // GTPase activity // inferred from electronic annotation /// 0003924 // G
0003779 // actin binding // inferred from electronic annotation /// 0005515 // protein binding // inferred from
ctronic annotation /// 0046872 // metal ion binding // inferred from electronic annotation

direct assay /// 0004872 // receptor activity // inferred from electronic annotation
electronic annotation /// 0008270 // zinc ion binding // inferred from electronic annotation /// 0008270 // zinc

otation
/ inferred from electronic annotation /// 0008233 // peptidase activity // inferred from electronic annotation //,
physical interaction /// 0008017 // microtubule binding // inferred from direct assay /// 0008017 // microtubul
/ inferred from electronic annotation /// 0008233 // peptidase activity // inferred from electronic annotation //,

ferred from electronic annotation
electronic annotation
om electronic annotation /// 0004383 // guanylate cyclase activity // inferred from direct assay /// 0004383 // g
/ inferred from electronic annotation /// 0004221 // ubiquitin thiolesterase activity // inferred from sequence o
ic annotation /// 0005515 // protein binding // inferred from electronic annotation
annotation /// 0071203 // WASH complex // inferred from sequence or structural similarity
rotein coupled // not recorded /// 0001609 // adenosine receptor activity, G-protein coupled // inferred from el
physical interaction /// 0016455 // RNA polymerase II transcription mediator activity // inferred from electronic
d from electronic annotation /// 0005524 // ATP binding // inferred from electronic annotation
om electronic annotation /// 0003924 // GTPase activity // inferred from electronic annotation /// 0005525 // G
om electronic annotation /// 0003774 // motor activity // inferred from electronic annotation /// 0003779 // ac
peptide beta-1,4-galactosyltransferase activity // inferred from direct assay /// 0005488 // binding // inferred fr
/ inferred from direct assay /// 0005515 // protein binding // inferred from physical interaction

red from electronic annotation
om electronic annotation /// 0003723 // RNA binding // inferred from electronic annotation /// 0004652 // poly

electronic annotation
electronic annotation
om electronic annotation /// 0004672 // protein kinase activity // inferred from electronic annotation /// 00046

electronic annotation
ect assay /// 0003677 // DNA binding // inferred from electronic annotation /// 0005515 // protein binding // in
electronic annotation /// 0004970 // ionotropic glutamate receptor activity // not recorded /// 0004970 // ion
om electronic annotation /// 0000287 // magnesium ion binding // inferred from electronic annotation /// 0000
tivity // inferred from electronic annotation /// 0004198 // calcium-dependent cysteine-type endopeptidase act
electronic annotation

rom electronic annotation /// 0008270 // zinc ion binding // inferred from electronic annotation /// 0046872 //
nstituent // inferred from electronic annotation /// 0005515 // protein binding // inferred from electronic annc

physical interaction /// 0005515 // protein binding // inferred from electronic annotation /// 0042802 // identic
on
electronic annotation
factor activity // not recorded /// 0003702 // RNA polymerase II transcription factor activity // inferred from ele
40M12 gene /// RIKEN cDNA E430024C06 gene

red from electronic annotation /// 0016787 // hydrolase activity // inferred from electronic annotation
m electronic annotation /// 0008236 // serine-type peptidase activity // inferred from electronic annotation ///
electronic annotation /// 0005515 // protein binding // inferred from physical interaction /// 0008022 // protei
electronic annotation /// 0005515 // protein binding // inferred from physical interaction
rom electronic annotation /// 0003713 // transcription coactivator activity // inferred from direct assay /// 000

rom electronic annotation /// 0003677 // DNA binding // inferred from electronic annotation /// 0008270 // zin
electronic annotation
red from electronic annotation
om electronic annotation /// 0003924 // GTPase activity // inferred from electronic annotation /// 0005525 // G
electronic annotation
rom electronic annotation
lectronic annotation /// 0005515 // protein binding // inferred from physical interaction

tation /// 0016021 // integral to membrane // inferred from electronic annotation
ne // inferred from electronic annotation
red from electronic annotation /// 0046872 // metal ion binding // inferred from electronic annotation
red from electronic annotation
tivity // inferred from electronic annotation /// 0004725 // protein tyrosine phosphatase activity // inferred fro
red from electronic annotation /// 0005515 // protein binding // inferred from electronic annotation
e activity // not recorded /// 0004044 // amidophosphoribosyltransferase activity // inferred from electronic an
electronic annotation
electronic annotation
ed /// 0003824 // catalytic activity // inferred from electronic annotation /// 0004074 // biliverdin reductase ac
om electronic annotation /// 0003676 // nucleic acid binding // inferred from electronic annotation /// 0003723
erred from electronic annotation /// 0016740 // transferase activity // inferred from electronic annotation
nt methyltransferase activity // inferred from electronic annotation
ranscription factor activity // not recorded /// 0003700 // sequence-specific DNA binding transcription factor ac
on
// not recorded /// 0005112 // Notch binding // inferred from electronic annotation /// 0005515 // protein bin
itor activity // inferred from electronic annotation /// 0030414 // peptidase inhibitor activity // inferred from el
ctronic annotation /// 0009982 // pseudouridine synthase activity // inferred from direct assay /// 0009982 // p
om electronic annotation /// 0005515 // protein binding // inferred from electronic annotation /// 0005544 //

statement /// 0003723 // RNA binding // inferred from electronic annotation /// 0005515 // protein binding //
electronic annotation /// 0003995 // acyl-CoA dehydrogenase activity // inferred from electronic annotation //
electronic annotation /// 0004888 // transmembrane receptor activity // inferred from direct assay /// 00054
tivity // inferred from electronic annotation /// 0005515 // protein binding // inferred from physical interaction

se activity // inferred from electronic annotation /// 0005515 // protein binding // inferred from electronic ann
005737 // cytoplasm // inferred from electronic annotation
om electronic annotation /// 0001664 // G-protein-coupled receptor binding // not recorded /// 0001664 // G-p
// inferred from direct assay /// 0000293 // ferric-chelate reductase activity // inferred from mutant phenotype
tation
ect assay /// 0003677 // DNA binding // inferred from electronic annotation /// 0008270 // zinc ion binding // ir
ation
om electronic annotation /// 0004812 // aminoacyl-tRNA ligase activity // inferred from electronic annotation //
electronic annotation /// 0004615 // phosphomannomutase activity // inferred from electronic annotation ///

d from electronic annotation /// 0005515 // protein binding // inferred from physical interaction /// 0005515 //
or activity // inferred from electronic annotation /// 0005515 // protein binding // inferred from physical intera
rom electronic annotation /// 0003677 // DNA binding // inferred from electronic annotation /// 0008270 // zin

electronic annotation /// 0005157 // macrophage colony-stimulating factor receptor binding // inferred from s
eus // inferred from electronic annotation /// 0005730 // nucleolus // not recorded /// 0005730 // nucleolus //
electronic annotation /// 0004252 // serine-type endopeptidase activity // inferred from electronic annotation
ctronic annotation /// 0003700 // sequence-specific DNA binding transcription factor activity // inferred from e
om electronic annotation /// 0004017 // adenylate kinase activity // not recorded /// 0004017 // adenylate kina
ic annotation
electronic annotation /// 0004806 // triglyceride lipase activity // not recorded /// 0004806 // triglyceride lipas
tation /// 0016021 // integral to membrane // inferred from electronic annotation
d from electronic annotation /// 0005529 // sugar binding // not recorded /// 0005529 // sugar binding // inferr
on /// 0016020 // membrane // inferred from electronic annotation /// 0016021 // integral to membrane // inf
om electronic annotation /// 0003924 // GTPase activity // inferred from electronic annotation /// 0005515 // p
om electronic annotation /// 0005524 // ATP binding // inferred from electronic annotation
tivity // not recorded /// 0004721 // phosphoprotein phosphatase activity // inferred from electronic annotatio
physical interaction /// 0005515 // protein binding // inferred from electronic annotation /// 0016829 // lyase a

ing // inferred from electronic annotation
electronic annotation
ctronic annotation /// 0005515 // protein binding // inferred from physical interaction /// 0005515 // protein b
erred from electronic annotation
om electronic annotation
physical interaction /// 0005515 // protein binding // inferred from electronic annotation
/ inferred from electronic annotation /// 0004861 // cyclin-dependent protein kinase inhibitor activity // inferre
electronic annotation
electronic annotation

0003677 // DNA binding // inferred from electronic annotation /// 0003684 // damaged DNA binding // not rec
ic annotation /// 0016020 // membrane // inferred from electronic annotation /// 0016021 // integral to meml
physical interaction
tivity // inferred from electronic annotation /// 0004198 // calcium-dependent cysteine-type endopeptidase act
tivity // inferred from electronic annotation /// 0004198 // calcium-dependent cysteine-type endopeptidase act
electronic annotation /// 0016787 // hydrolase activity // inferred from electronic annotation
ferred from electronic annotation /// 0005201 // extracellular matrix structural constituent // inferred from dire
physical interaction /// 0005515 // protein binding // inferred from electronic annotation /// 0008022 // protei
ctronic annotation /// 0005515 // protein binding // inferred from physical interaction /// 0008270 // zinc ion b
physical interaction /// 0005515 // protein binding // inferred from electronic annotation /// 0008022 // protei
physical interaction /// 0005515 // protein binding // inferred from electronic annotation /// 0008022 // protei
ic annotation
electronic annotation /// 0008270 // zinc ion binding // inferred from electronic annotation /// 0016874 // ligas
ect assay /// 0003682 // chromatin binding // inferred from direct assay /// 0003714 // transcription corepress
ivity // inferred from electronic annotation /// 0008233 // peptidase activity // inferred from electronic annotat
ic annotation /// 0005509 // calcium ion binding // inferred from electronic annotation /// 0005515 // protein
om electronic annotation /// 0005515 // protein binding // inferred from electronic annotation /// 0005525 //
activity // inferred from electronic annotation /// 0005515 // protein binding // inferred from electronic annot
/// 0005739 // mitochondrion // inferred from electronic annotation
rom electronic annotation /// 0003677 // DNA binding // inferred from electronic annotation /// 0003700 // sec
electronic annotation /// 0008270 // zinc ion binding // inferred from electronic annotation /// 0008270 // zinc
om electronic annotation /// 0005525 // GTP binding // inferred from electronic annotation /// 0031072 // heat

m electronic annotation /// 0008237 // metallopeptidase activity // inferred from electronic annotation /// 000
ctronic annotation /// 0005524 // ATP binding // inferred from electronic annotation /// 0008270 // zinc ion bir
ed from sequence or structural similarity /// 0004177 // aminopeptidase activity // inferred from electronic anr
red from electronic annotation /// 0051087 // chaperone binding // inferred from electronic annotation
electronic annotation
leton // traceable author statement /// 0005515 // protein binding // inferred from electronic annotation
lectronic annotation /// 0005515 // protein binding // inferred from physical interaction
ate) O-methyltransferase activity // inferred from electronic annotation /// 0008168 // methyltransferase activi
om electronic annotation /// 0005524 // ATP binding // inferred from electronic annotation /// 0016887 // ATPa
physical interaction /// 0005515 // protein binding // inferred from electronic annotation
rom electronic annotation /// 0005515 // protein binding // inferred from electronic annotation /// 0008270 //
ter activity // inferred from electronic annotation
electronic annotation /// 0019902 // phosphatase binding // inferred from physical interaction
om electronic annotation /// 0003824 // catalytic activity // inferred from electronic annotation /// 0003869 //
ctronic annotation /// 0003714 // transcription corepressor activity // inferred from direct assay /// 0003714 //
physical interaction /// 0005515 // protein binding // inferred from electronic annotation /// 0030528 // transc
ic annotation
ctronic annotation /// 0005158 // insulin receptor binding // inferred from electronic annotation /// 0005178 /
erred from electronic annotation /// 0051082 // unfolded protein binding // inferred from electronic annotatio
factor activity // not recorded /// 0003702 // RNA polymerase II transcription factor activity // inferred from ele
tation /// 0016021 // integral to membrane // inferred from electronic annotation
notation
ctronic annotation /// 0003700 // sequence-specific DNA binding transcription factor activity // inferred from e
tivity // inferred from electronic annotation /// 0004725 // protein tyrosine phosphatase activity // inferred fro
om electronic annotation /// 0004666 // prostaglandin-endoperoxide synthase activity // inferred from electror
physical interaction
e transporter activity // inferred from electronic annotation /// 0005351 // sugar:hydrogen symporter activity /
ferred from electronic annotation

ed from electronic annotation /// 0005509 // calcium ion binding // inferred from electronic annotation /// 000
om electronic annotation /// 0004812 // aminoacyl-tRNA ligase activity // inferred from electronic annotation /

// inferred from physical interaction
ne // inferred from electronic annotation /// 0005515 // protein binding // inferred from electronic annotation
electronic annotation
om electronic annotation /// 0003676 // nucleic acid binding // inferred from electronic annotation /// 0003723
ctronic annotation /// 0005515 // protein binding // inferred from electronic annotation /// 0005515 // protei
om electronic annotation /// 0003676 // nucleic acid binding // inferred from electronic annotation /// 0003723

electronic annotation /// 0035091 // phosphoinositide binding // inferred from electronic annotation
ctronic annotation /// 0003700 // sequence-specific DNA binding transcription factor activity // not recorded //

hosphatase activity // inferred from electronic annotation /// 0008254 // 3'-nucleotidase activity // inferred fro
om electronic annotation /// 0003676 // nucleic acid binding // inferred from electronic annotation /// 0003723
activity // not recorded /// 0004029 // aldehyde dehydrogenase (NAD) activity // inferred from electronic anno
ne // not recorded /// 0003735 // structural constituent of ribosome // inferred from electronic annotation ///
physical interaction /// 0017147 // Wnt-protein binding // inferred from direct assay

tation /// 0016021 // integral to membrane // inferred from electronic annotation
physical interaction /// 0005515 // protein binding // inferred from electronic annotation /// 0046982 // protei
om electronic annotation /// 0003924 // GTPase activity // inferred from direct assay /// 0003924 // GTPase act
ferred from electronic annotation /// 0005506 // iron ion binding // inferred from electronic annotation /// 001
ctronic annotation /// 0003690 // double-stranded DNA binding // inferred from direct assay /// 0003700 // se
electronic annotation /// 0008270 // zinc ion binding // inferred from electronic annotation /// 0046872 // met
electronic annotation /// 0005515 // protein binding // inferred from physical interaction /// 0005515 // prote
om electronic annotation /// 0004798 // thymidylate kinase activity // inferred from direct assay /// 0004798 //
electronic annotation /// 0030165 // PDZ domain binding // inferred from electronic annotation /// 0031698 //
electronic annotation /// 0030165 // PDZ domain binding // inferred from electronic annotation /// 0031698 //
ctronic annotation /// 0003700 // sequence-specific DNA binding transcription factor activity // inferred from e
physical interaction /// 0008134 // transcription factor binding // inferred from physical interaction /// 001978
tation /// 0016021 // integral to membrane // inferred from electronic annotation
electronic annotation
ctronic annotation /// 0005523 // tropomyosin binding // inferred from electronic annotation /// 0051011 // m
ctronic annotation /// 0005515 // protein binding // inferred from electronic annotation /// 0031014 // tropon
ctronic annotation /// 0003700 // sequence-specific DNA binding transcription factor activity // inferred from e
)5634 // nucleus // inferred from electronic annotation
nic annotation
om electronic annotation /// 0005488 // binding // inferred from electronic annotation /// 0005524 // ATP bind
electronic annotation /// 0017134 // fibroblast growth factor binding // not recorded /// 0017134 // fibroblast
de-protein glycotransferase activity // inferred from electronic annotation /// 0005515 // protein binding // infe
ctivity // not recorded /// 0005487 // nucleocytoplasmic transporter activity // inferred from electronic annotat
electronic annotation /// 0046982 // protein heterodimerization activity // not recorded
on /// 0005634 // nucleus // inferred from sequence or structural similarity /// 0005737 // cytoplasm // inferre
tation /// 0005813 // centrosome // inferred from sequence or structural similarity /// 0005815 // microtubule
de-protein glycotransferase activity // inferred from electronic annotation /// 0005515 // protein binding // infe
ctronic annotation
from electronic annotation
ctronic annotation /// 0003730 // mRNA 3'-UTR binding // inferred from electronic annotation /// 0003730 // m
ect assay /// 0003677 // DNA binding // inferred from electronic annotation /// 0003700 // sequence-specific D
ranscription factor activity // inferred from electronic annotation /// 0005515 // protein binding // inferred fron
electronic annotation

rom electronic annotation /// 0003677 // DNA binding // inferred from direct assay /// 0003682 // chromatin bi

electronic annotation
rom electronic annotation /// 0003735 // structural constituent of ribosome // not recorded /// 0003735 // stru
)5634 // nucleus // not recorded /// 0005634 // nucleus // inferred from electronic annotation /// 0005737 // c

ctronic annotation /// 0003677 // DNA binding // inferred from sequence or structural similarity /// 0004842 //
electronic annotation /// 0004310 // farnesyl-diphosphate farnesyltransferase activity // not recorded /// 000
activity // inferred from electronic annotation /// 0004043 // L-aminoadipate-semialdehyde dehydrogenase act
electronic annotation
om electronic annotation /// 0003676 // nucleic acid binding // inferred from electronic annotation /// 0003677
om electronic annotation /// 0005515 // protein binding // inferred from electronic annotation /// 0005524 // A
olysome // not recorded

// inferred from electronic annotation /// 0004402 // histone acetyltransferase activity // inferred from electro
ctronic annotation /// 0002039 // p53 binding // inferred from sequence or structural similarity /// 0004221 // u
physical interaction
ect assay /// 0003677 // DNA binding // inferred from electronic annotation /// 0003700 // sequence-specific D
om electronic annotation /// 0000287 // magnesium ion binding // inferred from electronic annotation /// 0004
/ /// 0016023 // cytoplasmic membrane-bounded vesicle // inferred from direct assay /// 0016023 // cytoplasmi
nic annotation
om electronic annotation /// 0004842 // ubiquitin-protein ligase activity // not recorded /// 0004842 // ubiquiti
ctronic annotation
electronic annotation
om electronic annotation /// 0004672 // protein kinase activity // inferred from direct assay /// 0004672 // prot
om electronic annotation /// 0003677 // DNA binding // inferred from electronic annotation /// 0003682 // chro
ase activity // inferred from direct assay /// 0004014 // adenosylmethionine decarboxylase activity // not recor
physical interaction
physical interaction /// 0005515 // protein binding // inferred from electronic annotation /// 0046982 // protei
yl-phosphate N-acetylglucosaminephosphotransferase activity // inferred from direct assay /// 0003975 // UDP
d from electronic annotation /// 0004622 // lysophospholipase activity // inferred from electronic annotation //
ectronic annotation /// 0008083 // growth factor activity // inferred from electronic annotation
inding // not recorded /// 0048407 // platelet-derived growth factor binding // inferred from electronic annotat
electronic annotation /// 0005520 // insulin-like growth factor binding // inferred from electronic annotation
/ inferred from electronic annotation /// 0005509 // calcium ion binding // inferred from electronic annotation /
atidic acid receptor activity // inferred from direct assay /// 0001619 // lysosphingolipid and lysophosphatidic a
ctronic annotation /// 0043021 // ribonucleoprotein binding // not recorded /// 0043021 // ribonucleoprotein b
electronic annotation /// 0004553 // hydrolase activity, hydrolyzing O-glycosyl compounds // inferred from ele
nic annotation /// 0005515 // protein binding // inferred from physical interaction /// 0005515 // protein bindin
electronic annotation /// 0030544 // Hsp70 protein binding // inferred from electronic annotation /// 0031072
om electronic annotation /// 0004842 // ubiquitin-protein ligase activity // inferred from electronic annotation /
electronic annotation /// 0042802 // identical protein binding // inferred from electronic annotation
red from electronic annotation /// 0004872 // receptor activity // inferred from electronic annotation /// 0004
ctronic annotation /// 0003713 // transcription coactivator activity // traceable author statement /// 0003713 /
electronic annotation /// 0005172 // vascular endothelial growth factor receptor binding // traceable author s
other substituted phosphate groups // traceable author statement
ect assay /// 0003677 // DNA binding // inferred from physical interaction /// 0003677 // DNA binding // inferre
ctronic annotation /// 0003725 // double-stranded RNA binding // inferred from electronic annotation /// 0005
myltransferase activity // inferred from mutant phenotype /// 0003810 // protein-glutamine gamma-glutamyltr
/ inferred from electronic annotation /// 0004843 // ubiquitin-specific protease activity // inferred from sequen
om electronic annotation /// 0003676 // nucleic acid binding // inferred from electronic annotation /// 0003677
om electronic annotation /// 0003774 // motor activity // inferred from electronic annotation /// 0003779 // ac
electronic annotation /// 0008009 // chemokine activity // inferred from electronic annotation /// 0008201 //
conferring elasticity // traceable author statement
// inferred from electronic annotation /// 0003714 // transcription corepressor activity // inferred from direct a
ect assay /// 0003677 // DNA binding // inferred from electronic annotation /// 0005515 // protein binding // in
ctronic annotation
electronic annotation
// 0000149 // SNARE binding // inferred from electronic annotation /// 0005198 // structural molecule activity /
/ inferred from electronic annotation /// 0004872 // receptor activity // inferred from electronic annotation ///
electronic annotation

rom electronic annotation /// 0008028 // monocarboxylic acid transmembrane transporter activity // not record
n electronic annotation
n electronic annotation /// 0004970 // ionotropic glutamate receptor activity // not recorded /// 0004970 // ion
'
tation /// 0016021 // integral to membrane // inferred from electronic annotation
sporter activity // inferred from electronic annotation
electronic annotation
rom electronic annotation /// 0008270 // zinc ion binding // inferred from electronic annotation /// 0046872 //
om electronic annotation /// 0003824 // catalytic activity // inferred from electronic annotation /// 0004674 //
eceptor binding // inferred from direct assay /// 0005161 // platelet-derived growth factor receptor binding // ir
nic annotation /// 0005923 // tight junction // inferred from electronic annotation /// 0016020 // membrane //
physical interaction /// 0005515 // protein binding // inferred from electronic annotation
om electronic annotation /// 0004672 // protein kinase activity // inferred from electronic annotation /// 00046
nding // inferred from electronic annotation /// 0004872 // receptor activity // inferred from electronic annotati
rom electronic annotation /// 0005515 // protein binding // inferred from electronic annotation /// 0005516 //
ycoprotein beta-1,6-N-acetylglucosaminyltransferase activity // not recorded /// 0003829 // beta-1,3-galactosy
not recorded /// 0000166 // nucleotide binding // inferred from electronic annotation /// 0003774 // motor acti
se activity // inferred from electronic annotation /// 0005509 // calcium ion binding // inferred from electronic
tation /// 0016021 // integral to membrane // inferred from electronic annotation
nic annotation
ferred from electronic annotation /// 0030247 // polysaccharide binding // inferred from electronic annotation
electronic annotation
om electronic annotation /// 0004672 // protein kinase activity // inferred from electronic annotation /// 00047
electronic annotation /// 0016301 // kinase activity // inferred from direct assay
rred from electronic annotation /// 0005515 // protein binding // inferred from physical interaction
electronic annotation
lectronic annotation /// 0005515 // protein binding // inferred from electronic annotation /// 0042802 // identi
om electronic annotation /// 0003676 // nucleic acid binding // inferred from electronic annotation /// 0003723
electronic annotation /// 0004415 // hyaluronoglucosaminidase activity // not recorded /// 0004415 // hyalu
om electronic annotation /// 0004672 // protein kinase activity // inferred from electronic annotation /// 00046
ctivity // inferred from direct assay /// 0004060 // arylamine N-acetyltransferase activity // inferred from mutan
ectronic annotation /// 0005515 // protein binding // inferred from electronic annotation
ectronic annotation /// 0005515 // protein binding // inferred from electronic annotation
electronic annotation /// 0008378 // galactosyltransferase activity // inferred from electronic annotation /// 00
ectronic annotation /// 0005198 // structural molecule activity // inferred from direct assay /// 0005516 // calm
ectronic annotation
ranscription factor activity // inferred from sequence or structural similarity /// 0005515 // protein binding // in
d from direct assay /// 0004222 // metalloendopeptidase activity // not recorded /// 0004222 // metalloendope
rred from electronic annotation /// 0004872 // receptor activity // inferred from electronic annotation /// 0004
rom electronic annotation
tivity // inferred from electronic annotation /// 0004722 // protein serine/threonine phosphatase activity // infe
ctronic annotation
om electronic annotation /// 0004672 // protein kinase activity // inferred from electronic annotation /// 00046
n electronic annotation /// 0005030 // neurotrophin receptor activity // inferred from electronic annotation ///
d from physical interaction /// 0008270 // zinc ion binding // inferred from electronic annotation /// 0017124 //
rred from electronic annotation /// 0004872 // receptor activity // inferred from electronic annotation /// 0004
tronic annotation

ctronic annotation /// 0003700 // sequence-specific DNA binding transcription factor activity // inferred from e
rred from electronic annotation /// 0004872 // receptor activity // inferred from electronic annotation /// 0004
rom electronic annotation /// 0003677 // DNA binding // inferred from electronic annotation /// 0008270 // zin
electronic annotation /// 0004931 // extracellular ATP-gated cation channel activity // not recorded /// 00049
physical interaction /// 0005515 // protein binding // inferred from electronic annotation
e endopeptidase activity // inferred from electronic annotation
// traceable author statement /// 0005515 // protein binding // inferred from electronic annotation
0003677 // DNA binding // inferred from electronic annotation /// 0003690 // double-stranded DNA binding //
om electronic annotation /// 0003676 // nucleic acid binding // inferred from electronic annotation /// 0003723
electronic annotation /// 0005178 // integrin binding // not recorded /// 0005178 // integrin binding // inferre
rom electronic annotation /// 0004518 // nuclease activity // inferred from electronic annotation /// 0004527 /
om electronic annotation /// 0004672 // protein kinase activity // inferred from electronic annotation /// 00047
ed from direct assay
electronic annotation /// 0008270 // zinc ion binding // inferred from electronic annotation /// 0016874 // ligas
nstituent // inferred from electronic annotation /// 0046332 // SMAD binding // inferred from physical interact
ctronic annotation /// 0003785 // actin monomer binding // inferred from sequence or structural similarity ///
om electronic annotation /// 0005524 // ATP binding // inferred from electronic annotation /// 0016787 // hydr
tivity // inferred from electronic annotation /// 0005515 // protein binding // inferred from electronic annotatio
s // traceable author statement /// 0005212 // structural constituent of eye lens // inferred from electronic ann
ctivity // traceable author statement /// 0005515 // protein binding // inferred from physical interaction /// 00
ctronic annotation /// 0005515 // protein binding // inferred from electronic annotation /// 0015643 // toxin bi
nic annotation /// 0034364 // high-density lipoprotein particle // inferred from electronic annotation
ctronic annotation /// 0030528 // transcription regulator activity // inferred from electronic annotation
itor activity // inferred from electronic annotation /// 0005515 // protein binding // inferred from physical inter
0003700 // sequence-specific DNA binding transcription factor activity // not recorded /// 0003723 // RNA bind
om electronic annotation /// 0004842 // ubiquitin-protein ligase activity // not recorded /// 0004842 // ubiquiti
om electronic annotation /// 0004672 // protein kinase activity // not recorded /// 0004672 // protein kinase ac
ed from electronic annotation /// 0015019 // heparan-alpha-glucosaminide N-acetyltransferase activity // inferr
physical interaction /// 0030296 // protein tyrosine kinase activator activity // inferred from direct assay
electronic annotation /// 0008289 // lipid binding // inferred from electronic annotation
nic annotation /// 0005515 // protein binding // inferred from electronic annotation
nic annotation /// 0005515 // protein binding // inferred from electronic annotation /// 0008565 // protein tran
electronic annotation /// 0030165 // PDZ domain binding // inferred from electronic annotation /// 0031698 //
nic annotation /// 0016020 // membrane // inferred from electronic annotation /// 0016021 // integral to meml
electronic annotation /// 0017147 // Wnt-protein binding // inferred from physical interaction /// 0017147 // W

ivity // inferred from electronic annotation /// 0008233 // peptidase activity // inferred from electronic annotat
tation /// 0016021 // integral to membrane // inferred from electronic annotation
rom electronic annotation /// 0005515 // protein binding // inferred from electronic annotation

electronic annotation
nic annotation /// 0016881 // acid-amino acid ligase activity // inferred from electronic annotation
tation /// 0016021 // integral to membrane // inferred from electronic annotation
ation

/// 0005178 // integrin binding // inferred from electronic annotation /// 0050839 // cell adhesion molecule bir
electronic annotation /// 0016740 // transferase activity // inferred from electronic annotation /// 0016757 //

ı electronic annotation /// 0016740 // transferase activity // inferred from electronic annotation /// 0016757 //
m electronic annotation /// 0016811 // hydrolase activity, acting on carbon-nitrogen (but not peptide) bonds, ir
rom electronic annotation /// 0008415 // acyltransferase activity // inferred from electronic annotation /// 0016
ecorded /// 0016787 // hydrolase activity // inferred from electronic annotation
om electronic annotation /// 0003924 // GTPase activity // inferred from direct assay /// 0005515 // protein bin
electronic annotation
electronic annotation /// 0008415 // acyltransferase activity // inferred from electronic annotation /// 001674(
red from electronic annotation /// 0005096 // GTPase activator activity // inferred from sequence or structural
ı electronic annotation
m electronic annotation
ect assay /// 0003677 // DNA binding // not recorded /// 0003677 // DNA binding // inferred from electronic an

ation
physical interaction /// 0005515 // protein binding // inferred from electronic annotation /// 0046875 // ephrin
ect assay /// 0003677 // DNA binding // inferred from electronic annotation /// 0003700 // sequence-specific D
fication activity // inferred from electronic annotation /// 0000386 // second spliceosomal transesterification ac
ı sequence or structural similarity /// 0005488 // binding // inferred from electronic annotation /// 0005515 // p
ctase (NADPH) activity // inferred from direct assay /// 0004420 // hydroxymethylglutaryl-CoA reductase (NADI
ı electronic annotation /// 0005509 // calcium ion binding // inferred from electronic annotation /// 0005515 //
ctronic annotation /// 0003899 // DNA-directed RNA polymerase activity // inferred from electronic annotation
electronic annotation
electronic annotation /// 0051721 // protein phosphatase 2A binding // inferred from electronic annotation ///
)5634 // nucleus // inferred from electronic annotation
ctronic annotation /// 0003730 // mRNA 3'-UTR binding // not recorded /// 0003730 // mRNA 3'-UTR binding //
ı electronic annotation /// 0005164 // tumor necrosis factor receptor binding // inferred from electronic annota
om electronic annotation /// 0005515 // protein binding // inferred from physical interaction /// 0016491 // oxi
ctronic annotation /// 0003824 // catalytic activity // inferred from electronic annotation /// 0004616 // phospl
/ 0005529 // sugar binding // inferred from electronic annotation /// 0008455 // alpha-1,6-mannosylglycoprote
rom electronic annotation /// 0003723 // RNA binding // traceable author statement /// 0003723 // RNA bindin

om electronic annotation /// 0003676 // nucleic acid binding // inferred from electronic annotation /// 0003723
rom electronic annotation /// 0016844 // strictosidine synthase activity // inferred from electronic annotation
ect assay /// 0003677 // DNA binding // inferred from electronic annotation /// 0003700 // sequence-specific D
// inferred from direct assay /// 0003713 // transcription coactivator activity // not recorded /// 0003713 // tra
tation /// 0016021 // integral to membrane // inferred from electronic annotation
d from electronic annotation /// 0005634 // nucleus // inferred from electronic annotation /// 0005730 // nucle
nferred from electronic annotation

electronic annotation
om electronic annotation /// 0003676 // nucleic acid binding // inferred from electronic annotation /// 0003723
or activity // inferred from electronic annotation /// 0005086 // ARF guanyl-nucleotide exchange factor activity
ation /// 0005765 // lysosomal membrane // inferred from electronic annotation /// 0016020 // membrane // ir
/ inferred from electronic annotation /// 0008233 // peptidase activity // inferred from electronic annotation //
ctronic annotation /// 0005523 // tropomyosin binding // inferred from electronic annotation
electronic annotation
tivity // inferred from electronic annotation /// 0019899 // enzyme binding // not recorded /// 0019899 // enzy
ı electronic annotation

ɔm electronic annotation /// 0005515 // protein binding // inferred from physical interaction /// 0005515 // prc
tation /// 0016021 // integral to membrane // inferred from electronic annotation
ctronic annotation /// 0003682 // chromatin binding // inferred from direct assay /// 0005515 // protein bindin
ɔm electronic annotation /// 0004672 // protein kinase activity // inferred from electronic annotation /// 00047
physical interaction /// 0042802 // identical protein binding // inferred from direct assay
electronic annotation /// 0008270 // zinc ion binding // inferred from electronic annotation /// 0046872 // met
ɔm electronic annotation /// 0000287 // magnesium ion binding // inferred from electronic annotation /// 0004

C activity // inferred from electronic annotation /// 0004629 // phospholipase C activity // not recorded /// 000
ɔm electronic annotation /// 0004672 // protein kinase activity // inferred from electronic annotation /// 00047
n electronic annotation /// 0004222 // metalloendopeptidase activity // inferred from electronic annotation ///
ctronic annotation /// 0003899 // DNA-directed RNA polymerase activity // inferred from electronic annotation
ctronic annotation /// 0005515 // protein binding // inferred from electronic annotation
ɔm electronic annotation /// 0003924 // GTPase activity // inferred from electronic annotation /// 0005525 // G
tation /// 0016021 // integral to membrane // inferred from electronic annotation
tivity // inferred from electronic annotation
0003824 // catalytic activity // inferred from electronic annotation /// 0004070 // aspartate carbamoyltransfera
e activity // not recorded /// 0004044 // amidophosphoribosyltransferase activity // inferred from electronic an
mediator activity // inferred from electronic annotation
d from electronic annotation /// 0004672 // protein kinase activity // inferred from sequence or structural simila
from electronic annotation

ɔm electronic annotation /// 0004428 // inositol or phosphatidylinositol kinase activity // inferred from electron

m electronic annotation
ect assay /// 0003677 // DNA binding // inferred from electronic annotation /// 0008270 // zinc ion binding // ir

ɔm electronic annotation /// 0004430 // 1-phosphatidylinositol 4-kinase activity // not recorded /// 0004430 //
electronic annotation
ctronic annotation /// 0003700 // sequence-specific DNA binding transcription factor activity // inferred from e
NA binding // inferred from direct assay /// 0003677 // DNA binding // inferred from direct assay /// 0003677 /
ed from electronic annotation /// 0004437 // inositol or phosphatidylinositol phosphatase activity // not record
ɔm sequence or structural similarity /// 0005515 // protein binding // inferred from electronic annotation
de-protein glycotransferase activity // inferred from electronic annotation /// 0016740 // transferase activity //
nic annotation /// 0016020 // membrane // inferred from electronic annotation /// 0016021 // integral to meml
ctronic annotation /// 0005524 // ATP binding // inferred from electronic annotation /// 0016787 // hydrolase a
tivity // inferred from electronic annotation /// 0004725 // protein tyrosine phosphatase activity // inferred fro
on
/ inferred from electronic annotation /// 0008233 // peptidase activity // inferred from electronic annotation //
electronic annotation
ɔm electronic annotation /// 0004842 // ubiquitin-protein ligase activity // not recorded /// 0004842 // ubiquiti
ɔm electronic annotation /// 0003676 // nucleic acid binding // inferred from electronic annotation /// 0005515
ɔm electronic annotation /// 0003924 // GTPase activity // inferred from electronic annotation /// 0005525 // G
06869 // lipid transport // inferred from electronic annotation /// 0008202 // steroid metabolic process // infer
n electronic annotation /// 0005509 // calcium ion binding // inferred from electronic annotation /// 0005515 //
ivity // inferred from electronic annotation /// 0008233 // peptidase activity // inferred from electronic annotat
rom electronic annotation /// 0005244 // voltage-gated ion channel activity // not recorded /// 0005244 // volta

om electronic annotation /// 0004672 // protein kinase activity // inferred from electronic annotation /// 00046
om electronic annotation /// 0004672 // protein kinase activity // inferred from electronic annotation /// 00046
/ inferred from electronic annotation /// 0008233 // peptidase activity // inferred from electronic annotation //
physical interaction /// 0005515 // protein binding // inferred from electronic annotation /// 0008022 // protei
n electronic annotation /// 0004222 // metalloendopeptidase activity // inferred from electronic annotation ///
ne // not recorded /// 0003735 // structural constituent of ribosome // inferred from electronic annotation
ty // inferred from electronic annotation

lirect assay /// 0003729 // mRNA binding // not recorded /// 0003729 // mRNA binding // inferred from electro
physical interaction /// 0008134 // transcription factor binding // inferred from physical interaction /// 001978
de-protein glycotransferase activity // inferred from electronic annotation /// 0005515 // protein binding // infe
electronic annotation
red from electronic annotation /// 0004872 // receptor activity // inferred from electronic annotation /// 0004

transferase activity // inferred from electronic annotation /// 0003779 // actin binding // inferred from electror
om electronic annotation /// 0000287 // magnesium ion binding // inferred from electronic annotation /// 0004
itor activity // inferred from electronic annotation /// 0005515 // protein binding // inferred from physical inter
om electronic annotation
rom electronic annotation /// 0003824 // catalytic activity // inferred from electronic annotation /// 0004528 //

ne // inferred from electronic annotation /// 0005515 // protein binding // inferred from electronic annotation /
ect assay /// 0003677 // DNA binding // inferred from electronic annotation /// 0003700 // sequence-specific D
direct assay /// 0005515 // protein binding // inferred from physical interaction /// 0016491 // oxidoreductase
om electronic annotation /// 0004672 // protein kinase activity // inferred from electronic annotation /// 00047
physical interaction /// 0005515 // protein binding // inferred from electronic annotation /// 0030165 // PDZ do
d from physical interaction /// 0005262 // calcium channel activity // traceable author statement /// 0005488 /
electronic annotation /// 0008565 // protein transporter activity // inferred from electronic annotation /// 003
ne // inferred from direct assay
nic annotation /// 0005615 // extracellular space // not recorded /// 0005794 // Golgi apparatus // inferred fro
electronic annotation /// 0004758 // serine C-palmitoyltransferase activity // inferred from direct assay /// 000
om electronic annotation /// 0003824 // catalytic activity // inferred from electronic annotation /// 0004467 //
red from electronic annotation /// 0004872 // receptor activity // inferred from electronic annotation /// 0004

om electronic annotation /// 0003677 // DNA binding // inferred from electronic annotation /// 0003916 // DN
electronic annotation /// 0030169 // low-density lipoprotein binding // inferred from electronic annotation
om electronic annotation /// 0004672 // protein kinase activity // inferred from electronic annotation /// 00047
ctronic annotation
electronic annotation /// 0005496 // steroid binding // inferred from electronic annotation /// 0008289 // lipic
ed from electronic annotation /// 0050613 // delta14-sterol reductase activity // inferred from electronic annot

ed from sequence or structural similarity /// 0004177 // aminopeptidase activity // inferred from electronic anr
electronic annotation /// 0005488 // binding // inferred from electronic annotation /// 0008270 // zinc ion bin
ic annotation /// 0005515 // protein binding // inferred from physical interaction /// 0005515 // protein bindin

ferred from electronic annotation /// 0005515 // protein binding // inferred from electronic annotation /// 004
corded /// 0000287 // magnesium ion binding // inferred from electronic annotation /// 0001530 // lipopolysac
netic interaction /// 0003677 // DNA binding // inferred from electronic annotation /// 0003700 // sequence-sp

ıic annotation /// 0016020 // membrane // inferred from electronic annotation /// 0031225 // anchored to mer
atidic acid receptor activity // inferred from electronic annotation /// 0004871 // signal transducer activity // in
rom electronic annotation /// 0005488 // binding // inferred from electronic annotation /// 0005515 // protein l
inferred from electronic annotation /// 0016787 // hydrolase activity // inferred from electronic annotation ///

om electronic annotation /// 0003676 // nucleic acid binding // inferred from electronic annotation /// 0003723
nic annotation
ctronic annotation /// 0009982 // pseudouridine synthase activity // inferred from electronic annotation /// 00

electronic annotation /// 0046872 // metal ion binding // inferred from electronic annotation
tation /// 0016021 // integral to membrane // inferred from electronic annotation
yltransferase activity // inferred from electronic annotation /// 0005515 // protein binding // inferred from elec
ion /// 0007600 // sensory perception // inferred from electronic annotation
om electronic annotation /// 0003824 // catalytic activity // inferred from electronic annotation /// 0004467 // l
ıic annotation /// 0016020 // membrane // inferred from electronic annotation /// 0031225 // anchored to mer

nic annotation
/// 0005739 // mitochondrion // inferred from electronic annotation /// 0005743 // mitochondrial inner memk
electronic annotation
0003723 // RNA binding // inferred from electronic annotation /// 0003730 // mRNA 3'-UTR binding // not reco

ectronic annotation /// 0005488 // binding // inferred from electronic annotation /// 0017048 // Rho GTPase bi
ect assay /// 0003677 // DNA binding // inferred from electronic annotation /// 0003700 // sequence-specific D
tation /// 0016021 // integral to membrane // inferred from electronic annotation
physical interaction /// 0008270 // zinc ion binding // inferred from electronic annotation /// 0046872 // metal
red from electronic annotation /// 0004867 // serine-type endopeptidase inhibitor activity // inferred from elec
16020 // membrane // inferred from electronic annotation /// 0016021 // integral to membrane // inferred fro
physical interaction /// 0005515 // protein binding // inferred from electronic annotation /// 0010843 // promc
l from direct assay /// 0004527 // exonuclease activity // inferred from direct assay /// 0008198 // ferrous iron l
electronic annotation /// 0005496 // steroid binding // inferred from electronic annotation /// 0008289 // lipic
om electronic annotation /// 0017124 // SH3 domain binding // inferred from electronic annotation
rom electronic annotation /// 0003677 // DNA binding // inferred from direct assay /// 0003677 // DNA binding
lectronic annotation /// 0005515 // protein binding // inferred from physical interaction
electronic annotation /// 0004721 // phosphoprotein phosphatase activity // inferred from electronic annotati
inhibitor activity // inferred from electronic annotation /// 0005515 // protein binding // inferred from electror
tation /// 0016020 // membrane // inferred from electronic annotation /// 0016021 // integral to membrane //
ivity // inferred from electronic annotation
ctronic annotation /// 0003700 // sequence-specific DNA binding transcription factor activity // inferred from e

om electronic annotation /// 0005525 // GTP binding // inferred from electronic annotation
electronic annotation /// 0004252 // serine-type endopeptidase activity // inferred from electronic annotation
om electronic annotation /// 0004672 // protein kinase activity // inferred from electronic annotation /// 00047
om electronic annotation /// 0004672 // protein kinase activity // inferred from electronic annotation /// 00047
rom electronic annotation /// 0005509 // calcium ion binding // not recorded /// 0005509 // calcium ion binding
inferred from electronic annotation /// 0004311 // farnesyltranstransferase activity // not recorded /// 000465

tation /// 0016021 // integral to membrane // inferred from electronic annotation
ı electronic annotation /// 0030215 // semaphorin receptor binding // inferred from physical interaction
electronic annotation
:ation /// 0005813 // centrosome // inferred from electronic annotation /// 0005815 // microtubule organizing
om electronic annotation /// 0004428 // inositol or phosphatidylinositol kinase activity // inferred from electron
om electronic annotation /// 0003924 // GTPase activity // inferred from electronic annotation /// 0005515 // p
or activity // inferred from mutant phenotype /// 0005085 // guanyl-nucleotide exchange factor activity // infer
om electronic annotation /// 0004672 // protein kinase activity // not recorded /// 0004672 // protein kinase ac
on /// 0016607 // nuclear speck // inferred from electronic annotation
/// 0005739 // mitochondrion // inferred from electronic annotation /// 0016020 // membrane // inferred fror
om electronic annotation /// 0005524 // ATP binding // inferred from electronic annotation /// 0016787 // hydr
ı electronic annotation
e transporter activity // inferred from electronic annotation /// 0005351 // sugar:hydrogen symporter activity /
electronic annotation

nic annotation /// 0005739 // mitochondrion // inferred from direct assay /// 0005743 // mitochondrial inner m

activity // not recorded /// 0004791 // thioredoxin-disulfide reductase activity // inferred from electronic annot
electronic annotation
electronic annotation /// 0004415 // hyalurononglucosaminidase activity // inferred from electronic annotatio

om electronic annotation /// 0005515 // protein binding // inferred from electronic annotation /// 0005524 // A

inferred from electronic annotation /// 0016874 // ligase activity // inferred from electronic annotation
electronic annotation /// 0008270 // zinc ion binding // inferred from electronic annotation /// 0046872 // met

/// 0005741 // mitochondrial outer membrane // inferred from direct assay
ferred from electronic annotation
ı electronic annotation
rom electronic annotation /// 0003677 // DNA binding // inferred from direct assay /// 0003677 // DNA binding

:ronic annotation

ctronic annotation /// 0003700 // sequence-specific DNA binding transcription factor activity // traceable autho
electronic annotation /// 0030165 // PDZ domain binding // not recorded /// 0030165 // PDZ domain binding /
red from electronic annotation /// 0005506 // iron ion binding // inferred from electronic annotation /// 00090
ed from electronic annotation /// 0000287 // magnesium ion binding // inferred from sequence or structural sin
rom electronic annotation /// 0004518 // nuclease activity // inferred from electronic annotation /// 0004519 /
:ation /// 0005813 // centrosome // inferred from sequence or structural similarity /// 0005815 // microtubule

om electronic annotation /// 0005244 // voltage-gated ion channel activity // inferred from electronic annotati
:ronic annotation /// 0045095 // keratin filament // inferred from electronic annotation
on /// 0005739 // mitochondrion // inferred from electronic annotation
tation /// 0016021 // integral to membrane // inferred from electronic annotation

rred from electronic annotation

electronic annotation
otation /// 0005778 // peroxisomal membrane // inferred from electronic annotation /// 0016020 // membrane
C activity // inferred from electronic annotation /// 0004629 // phospholipase C activity // inferred from electr

physical interaction /// 0005515 // protein binding // inferred from electronic annotation /// 0070053 // throm
rom electronic annotation /// 0003677 // DNA binding // inferred from electronic annotation /// 0003682 // chr

ctronic annotation /// 0005515 // protein binding // inferred from electronic annotation /// 0016787 // hydrola

electronic annotation /// 0008270 // zinc ion binding // inferred from electronic annotation /// 0046872 // met
on /// 0005739 // mitochondrion // inferred from electronic annotation

ferred from electronic annotation /// 0008080 // N-acetyltransferase activity // non-traceable author statemen

ctronic annotation /// 0003729 // mRNA binding // inferred from electronic annotation /// 0003729 // mRNA b

rom electronic annotation

tation /// 0016021 // integral to membrane // inferred from electronic annotation

on /// 0005730 // nucleolus // inferred from electronic annotation /// 0005730 // nucleolus // inferred from se

ctronic annotation
// inferred from electronic annotation /// 0003713 // transcription coactivator activity // inferred from sequen

inyltransferase activity // inferred from electronic annotation /// 0005529 // sugar binding // inferred from elec

electronic annotation
inferred from electronic annotation
electronic annotation /// 0008134 // transcription factor binding // not recorded /// 0016563 // transcription a
red from electronic annotation /// 0008060 // ARF GTPase activator activity // not recorded /// 0008060 // ARF
om electronic annotation /// 0004674 // protein serine/threonine kinase activity // inferred from electronic ann
nic annotation

om electronic annotation /// 0005515 // protein binding // inferred from electronic annotation /// 0005524 // A
m electronic annotation /// 0017110 // nucleoside-diphosphatase activity // inferred from electronic annotation
yltransferase activity // inferred from electronic annotation /// 0008415 // acyltransferase activity // inferred fr
yltransferase activity // inferred from electronic annotation /// 0008415 // acyltransferase activity // inferred fr
nic annotation

electronic annotation
electronic annotation /// 0005515 // protein binding // inferred from physical interaction /// 0008022 // prote
/ inferred from electronic annotation
om electronic annotation /// 0008514 // organic anion transmembrane transporter activity // not recorded ///
nic annotation /// 0016020 // membrane // inferred from electronic annotation
om electronic annotation /// 0003824 // catalytic activity // inferred from electronic annotation /// 0004756 // s
ctronic annotation /// 0003690 // double-stranded DNA binding // inferred from sequence or structural similari
from electronic annotation

/ inferred from sequence or structural similarity /// 0005249 // voltage-gated potassium channel activity // infe
om electronic annotation /// 0005515 // protein binding // inferred from electronic annotation /// 0017137 // I
GenMAPP
om electronic annotation /// 0016757 // transferase activity, transferring glycosyl groups // inferred from electr
red from electronic annotation /// 0008270 // zinc ion binding // inferred from electronic annotation /// 00164[illegible]
ect assay /// 0003677 // DNA binding // inferred from electronic annotation /// 0005515 // protein binding // ir
nic annotation /// 0008092 // cytoskeletal protein binding // inferred from electronic annotation
electronic annotation /// 0016765 // transferase activity, transferring alkyl or aryl (other than methyl) groups /
rom electronic annotation
/ not recorded /// 0008131 // primary amine oxidase activity // inferred from electronic annotation /// 0016491

// inferred from electronic annotation /// 0016491 // oxidoreductase activity // inferred from electronic annota
on /// 0005737 // cytoplasm // inferred from electronic annotation
ctronic annotation /// 0003743 // translation initiation factor activity // inferred from electronic annotation ///
physical interaction
electronic annotation
red from electronic annotation /// 0005515 // protein binding // inferred from electronic annotation /// 00171[illegible]
physical interaction /// 0005515 // protein binding // inferred from electronic annotation
red from electronic annotation /// 0004872 // receptor activity // inferred from electronic annotation /// 0004[illegible]
electronic annotation
rom electronic annotation /// 0003723 // RNA binding // inferred from electronic annotation

ect assay /// 0003677 // DNA binding // inferred from electronic annotation /// 0003700 // sequence-specific D
author statement /// 0005509 // calcium ion binding // inferred from electronic annotation /// 0005515 // prot
rom electronic annotation /// 0008270 // zinc ion binding // inferred from electronic annotation /// 0046872 //
electronic annotation /// 0008270 // zinc ion binding // inferred from electronic annotation /// 0046872 // met
/ inferred from sequence or structural similarity /// 0005515 // protein binding // inferred from electronic annc
nic annotation
m electronic annotation /// 0051536 // iron-sulfur cluster binding // inferred from electronic annotation /// 005
electronic annotation /// 0005515 // protein binding // inferred from physical interaction

om direct assay /// 0003712 // transcription cofactor activity // inferred from direct assay /// 0003712 // transcr
tation /// 0016021 // integral to membrane // inferred from electronic annotation
tation /// 0016021 // integral to membrane // inferred from electronic annotation

tation /// 0016021 // integral to membrane // inferred from electronic annotation
ctronic annotation /// 0003682 // chromatin binding // inferred from direct assay /// 0003713 // transcription c
om electronic annotation /// 0003924 // GTPase activity // inferred from electronic annotation /// 0003924 // G
electronic annotation

om electronic annotation /// 0000832 // inositol hexakisphosphate 5-kinase activity // inferred from electronic a
rom electronic annotation /// 0005249 // voltage-gated potassium channel activity // inferred from electronic ar
or activity // inferred from electronic annotation /// 0005089 // Rho guanyl-nucleotide exchange factor activity

om electronic annotation /// 0004842 // ubiquitin-protein ligase activity // inferred from direct assay /// 00048[illegible]
se activity // inferred from electronic annotation /// 0016829 // lyase activity // inferred from electronic annota
red from direct assay /// 0004497 // monooxygenase activity // inferred from electronic annotation /// 0004499
uctural similarity /// 0016020 // membrane // inferred from electronic annotation /// 0016021 // integral to me
ectronic annotation
ne // inferred from electronic annotation /// 0005215 // transporter activity // inferred from electronic annotati

om electronic annotation /// 0004396 // hexokinase activity // not recorded /// 0004396 // hexokinase activity /
nnotation /// 0005743 // mitochondrial inner membrane // inferred from electronic annotation /// 0016020 //
ation
ivity // inferred from electronic annotation

physical interaction
red from electronic annotation /// 0016787 // hydrolase activity // inferred from electronic annotation
0003677 // DNA binding // inferred from electronic annotation /// 0003700 // sequence-specific DNA binding tr
ctronic annotation /// 0003700 // sequence-specific DNA binding transcription factor activity // inferred from d
om electronic annotation /// 0004672 // protein kinase activity // inferred from electronic annotation /// 00047
m electronic annotation
electronic annotation /// 0005509 // calcium ion binding // inferred from electronic annotation /// 0005515 //
on /// 0005737 // cytoplasm // inferred from electronic annotation
om electronic annotation /// 0004672 // protein kinase activity // not recorded /// 0004672 // protein kinase ac
ctronic annotation /// 0008080 // N-acetyltransferase activity // inferred from electronic annotation /// 00084[illegible]
/ inferred from electronic annotation /// 0008233 // peptidase activity // inferred from direct assay /// 0008233
rom electronic annotation /// 0003700 // sequence-specific DNA binding transcription factor activity // inferred

rom electronic annotation /// 0005509 // calcium ion binding // inferred from sequence or structural similarity /

ctronic annotation
rom electronic annotation /// 0005244 // voltage-gated ion channel activity // inferred from electronic annotati
rom electronic annotation /// 0005244 // voltage-gated ion channel activity // inferred from electronic annotati
electronic annotation
electronic annotation
tation /// 0016021 // integral to membrane // inferred from electronic annotation
physical interaction
red from electronic annotation
rom electronic annotation /// 0003677 // DNA binding // inferred from electronic annotation /// 0003682 // chr
om electronic annotation /// 0004370 // glycerol kinase activity // inferred from electronic annotation /// 0005
or activity // inferred from electronic annotation /// 0005515 // protein binding // inferred from electronic ann

rom electronic annotation /// 0005244 // voltage-gated ion channel activity // inferred from electronic annotati
phenotype /// 0030073 // insulin secretion // inferred from mutant phenotype /// 0030154 // cell differentiatio
d from electronic annotation /// 0005488 // binding // inferred from electronic annotation /// 0005524 // ATP b
electronic annotation /// 0046872 // metal ion binding // inferred from electronic annotation
om electronic annotation /// 0003714 // transcription corepressor activity // inferred from direct assay /// 0004
tation /// 0016021 // integral to membrane // inferred from electronic annotation
electronic annotation
or activity // inferred from electronic annotation /// 0005089 // Rho guanyl-nucleotide exchange factor activity

/// 0080008 // CUL4 RING ubiquitin ligase complex // inferred from electronic annotation /// 0080008 // CUL4
tation /// 0016021 // integral to membrane // inferred from electronic annotation
nic annotation

nic annotation
physical interaction
om electronic annotation /// 0003774 // motor activity // inferred from electronic annotation /// 0003777 // mi
electronic annotation /// 0019770 // IgG receptor activity // inferred from mutant phenotype /// 0019864 // Ig

om electronic annotation /// 0004672 // protein kinase activity // inferred from electronic annotation /// 00046
electronic annotation /// 0005524 // ATP binding // inferred from electronic annotation
d from electronic annotation /// 0047499 // calcium-independent phospholipase A2 activity // inferred from ele
itor activity // inferred from electronic annotation /// 0030414 // peptidase inhibitor activity // inferred from el
rom electronic annotation /// 0005244 // voltage-gated ion channel activity // inferred from electronic annotati
rom electronic annotation

rom electronic annotation /// 0003677 // DNA binding // inferred from direct assay /// 0003677 // DNA binding
rom electronic annotation /// 0008270 // zinc ion binding // inferred from electronic annotation /// 0046872 //
ctronic annotation /// 0003700 // sequence-specific DNA binding transcription factor activity // inferred from d
uctase activity // inferred from direct assay /// 0004748 // ribonucleoside-diphosphate reductase activity // infe
uctase activity // inferred from direct assay /// 0004748 // ribonucleoside-diphosphate reductase activity // infe

activity // not recorded /// 0004029 // aldehyde dehydrogenase (NAD) activity // inferred from electronic anno
otation
/ inferred from sequence or structural similarity /// 0004222 // metalloendopeptidase activity // inferred from e
rom direct assay /// 0005509 // calcium ion binding // inferred from electronic annotation /// 0005515 // protei
ctronic annotation /// 0003700 // sequence-specific DNA binding transcription factor activity // inferred from e
/ inferred from electronic annotation /// 0008233 // peptidase activity // inferred from electronic annotation //
rom electronic annotation /// 0003677 // DNA binding // inferred from direct assay /// 0003677 // DNA binding

om electronic annotation /// 0003677 // DNA binding // inferred from electronic annotation /// 0005524 // ATP
nyltransferase activity // inferred from electronic annotation /// 0008415 // acyltransferase activity // inferred
om electronic annotation /// 0003676 // nucleic acid binding // inferred from electronic annotation /// 0003677
tation /// 0016021 // integral to membrane // inferred from electronic annotation
rom electronic annotation /// 0003677 // DNA binding // inferred from sequence or structural similarity /// 000
ect assay /// 0003677 // DNA binding // inferred from electronic annotation /// 0003690 // double-stranded DN
tation /// 0016021 // integral to membrane // inferred from electronic annotation
ivity // not recorded /// 0015459 // potassium channel regulator activity // inferred from electronic annotation
rom electronic annotation /// 0003677 // DNA binding // inferred from electronic annotation /// 0008270 // zin
electronic annotation /// 0005515 // protein binding // inferred from physical interaction /// 0005515 // prote
electronic annotation
ctronic annotation /// 0004871 // signal transducer activity // inferred from electronic annotation /// 0030528

electronic annotation /// 0008233 // peptidase activity // inferred from electronic annotation /// 0008234 // cy

electronic annotation /// 0004659 // prenyltransferase activity // inferred from electronic annotation /// 0004

or activity // inferred from electronic annotation /// 0005515 // protein binding // inferred from electronic ann

red from electronic annotation /// 0005097 // Rab GTPase activator activity // inferred from electronic annotati
ctronic annotation /// 0003709 // RNA polymerase III transcription factor activity // not recorded /// 0003709 /
not recorded /// 0004177 // aminopeptidase activity // inferred from mutant phenotype /// 0004177 // aminop
ng // inferred from physical interaction /// 0005159 // insulin-like growth factor receptor binding // inferred fro
ed from electronic annotation /// 0016702 // oxidoreductase activity, acting on single donors with incorporatio
red from electronic annotation /// 0016787 // hydrolase activity // inferred from electronic annotation /// 001
rter activity // inferred from direct assay /// 0015093 // ferrous iron transmembrane transporter activity // infe
rom electronic annotation /// 0005515 // protein binding // inferred from electronic annotation /// 0008270 //
nic annotation
N-acetylglucosaminyltransferase activity // inferred from electronic annotation /// 0008375 // acetylglucosamin
nic annotation /// 0005912 // adherens junction // inferred from electronic annotation /// 0016020 // membrar
om electronic annotation /// 0004672 // protein kinase activity // inferred from electronic annotation /// 00046
ion
// not recorded /// 0016455 // RNA polymerase II transcription mediator activity // inferred from electronic an
electronic annotation /// 0008134 // transcription factor binding // inferred from electronic annotation

physical interaction /// 0042605 // peptide antigen binding // inferred from direct assay

electronic annotation /// 0008415 // acyltransferase activity // inferred from electronic annotation /// 0016409
om electronic annotation /// 0003824 // catalytic activity // inferred from electronic annotation /// 0004467 // l
y // not recorded /// 0004357 // glutamate-cysteine ligase activity // inferred from electronic annotation /// 000
tation /// 0016021 // integral to membrane // inferred from electronic annotation
om electronic annotation /// 0003676 // nucleic acid binding // inferred from electronic annotation
om electronic annotation /// 0004672 // protein kinase activity // inferred from electronic annotation /// 00047
// inferred from electronic annotation /// 0003713 // transcription coactivator activity // inferred from sequen

ctronic annotation /// 0005515 // protein binding // inferred from electronic annotation /// 0008168 // methyl

rom electronic annotation /// 0005515 // protein binding // inferred from electronic annotation /// 0008270 //

electronic annotation /// 0004586 // ornithine decarboxylase activity // inferred from direct assay /// 0005515

ect assay /// 0003677 // DNA binding // inferred from electronic annotation /// 0003700 // sequence-specific D
/ inferred from electronic annotation /// 0008233 // peptidase activity // inferred from electronic annotation //

electronic annotation /// 0004331 // fructose-2,6-bisphosphate 2-phosphatase activity // inferred from electro

rom electronic annotation /// 0008270 // zinc ion binding // inferred from electronic annotation /// 0046872 //

0005737 // cytoplasm // inferred from electronic annotation
electronic annotation /// 0004872 // receptor activity // inferred from sequence or structural similarity /// 000
/ inferred from sequence or structural similarity /// 0005515 // protein binding // inferred from electronic anno
red from electronic annotation /// 0005515 // protein binding // inferred from electronic annotation /// 00510
/ inferred from electronic annotation /// 0005509 // calcium ion binding // inferred from electronic annotation /

om electronic annotation /// 0003676 // nucleic acid binding // inferred from electronic annotation /// 0008270

d from electronic annotation /// 0005488 // binding // inferred from electronic annotation /// 0005524 // ATP b
electronic annotation

nic annotation
om electronic annotation /// 0000166 // nucleotide binding // inferred from electronic annotation /// 0003824

m electronic annotation /// 0008236 // serine-type peptidase activity // inferred from electronic annotation ///
tation /// 0016021 // integral to membrane // inferred from electronic annotation

electronic annotation

om electronic annotation /// 0005525 // GTP binding // inferred from electronic annotation

om direct assay /// 0003712 // transcription cofactor activity // inferred from direct assay /// 0003712 // transcr
/ inferred from electronic annotation /// 0003705 // sequence-specific enhancer binding RNA polymerase II tra

nic annotation /// 0016020 // membrane // inferred from electronic annotation

ctronic annotation /// 0003700 // sequence-specific DNA binding transcription factor activity // inferred from e
electronic annotation
ctronic annotation /// 0005789 // endoplasmic reticulum membrane // inferred from electronic annotation /// 0

om electronic annotation /// 0003676 // nucleic acid binding // inferred from electronic annotation /// 0003677
physical interaction /// 0005515 // protein binding // inferred from electronic annotation
om electronic annotation /// 0003774 // motor activity // inferred from electronic annotation /// 0003777 // mi
nding // inferred from electronic annotation

/ inferred from electronic annotation /// 0005509 // calcium ion binding // inferred from electronic annotation /

// not recorded /// 0003713 // transcription coactivator activity // inferred from electronic annotation /// 0004
om electronic annotation /// 0004222 // metalloendopeptidase activity // inferred from electronic annotation /

ranscription factor activity // inferred from electronic annotation /// 0003714 // transcription corepressor activi
rom electronic annotation /// 0005515 // protein binding // inferred from electronic annotation /// 0008270 //
// inferred from direct assay /// 0003713 // transcription coactivator activity // not recorded /// 0003713 // tra
rom electronic annotation /// 0005244 // voltage-gated ion channel activity // inferred from electronic annotati
nic annotation

tivity // not recorded /// 0004721 // phosphoprotein phosphatase activity // inferred from electronic annotatio
rom electronic annotation /// 0003723 // RNA binding // inferred from electronic annotation
n electronic annotation /// 0031708 // endothelin B receptor binding // inferred from electronic annotation
n electronic annotation /// 0031708 // endothelin B receptor binding // inferred from electronic annotation
itor activity // inferred from electronic annotation /// 0030414 // peptidase inhibitor activity // inferred from el
physical interaction /// 0008200 // ion channel inhibitor activity // inferred from direct assay /// 0030550 // ace

ctronic annotation /// 0003677 // DNA binding // non-traceable author statement /// 0005515 // protein bindi

ıot recorded /// 0046982 // protein heterodimerization activity // not recorded
or activity // not recorded /// 0005085 // guanyl-nucleotide exchange factor activity // inferred from electronic

rom electronic annotation /// 0003677 // DNA binding // inferred from electronic annotation /// 0003700 // sec
rom electronic annotation /// 0008270 // zinc ion binding // inferred from electronic annotation /// 0046872 //
annotation /// 0005794 // Golgi apparatus // inferred from electronic annotation /// 0016020 // membrane //
electronic annotation /// 0008131 // primary amine oxidase activity // not recorded /// 0008131 // primary am

abotropic glutamate receptor activity // not recorded /// 0001642 // group III metabotropic glutamate receptor
ctronic annotation /// 0003677 // DNA binding // inferred from sequence or structural similarity /// 0004842 //

// inferred from electronic annotation /// 0003713 // transcription coactivator activity // inferred from sequen
rom electronic annotation /// 0008270 // zinc ion binding // inferred from electronic annotation /// 0046872 //
or activity // inferred from electronic annotation /// 0005515 // protein binding // inferred from electronic ann
om electronic annotation /// 0002039 // p53 binding // inferred from physical interaction /// 0003676 // nucleic
rom electronic annotation /// 0005515 // protein binding // inferred from electronic annotation /// 0008270 //
ecorded

factor activity // inferred from electronic annotation /// 0005515 // protein binding // inferred from physical in
rom electronic annotation /// 0008270 // zinc ion binding // inferred from electronic annotation /// 0046872 //
electronic annotation /// 0005515 // protein binding // inferred from electronic annotation
tation /// 0016021 // integral to membrane // inferred from electronic annotation
ctronic annotation /// 0005200 // structural constituent of cytoskeleton // inferred from electronic annotation

ctronic annotation /// 0005515 // protein binding // inferred from electronic annotation /// 0008270 // zinc ior

tation /// 0016021 // integral to membrane // inferred from electronic annotation

tation /// 0016021 // integral to membrane // inferred from electronic annotation

tation /// 0016021 // integral to membrane // inferred from electronic annotation

/ non-traceable author statement /// 0004222 // metalloendopeptidase activity // inferred from electronic annc

ı electronic annotation /// 0051267 // CP2 mannose-ethanolamine phosphotransferase activity // not recorded

›m electronic annotation /// 0003824 // catalytic activity // inferred from electronic annotation /// 0005515 // p
on /// 0005737 // cytoplasm // inferred from electronic annotation /// 0005829 // cytosol // inferred from elec

or activity // inferred from electronic annotation /// 0017034 // Rap guanyl-nucleotide exchange factor activity
ctronic annotation /// 0003684 // damaged DNA binding // inferred from electronic annotation /// 0004518 //

/ /// 0005739 // mitochondrion // inferred from electronic annotation
electronic annotation

ccinyl-transferring) activity // not recorded /// 0004591 // oxoglutarate dehydrogenase (succinyl-transferring) a

physical interaction /// 0005515 // protein binding // inferred from electronic annotation

ect assay /// 0003677 // DNA binding // inferred from electronic annotation /// 0005515 // protein binding // ir
rred from electronic annotation /// 0004872 // receptor activity // inferred from electronic annotation /// 0004[illegible]

electronic annotation
›m electronic annotation /// 0003924 // GTPase activity // inferred from electronic annotation /// 0005198 // s[illegible]
ferred from electronic annotation /// 0005524 // ATP binding // inferred from electronic annotation
›m electronic annotation /// 0004672 // protein kinase activity // inferred from electronic annotation /// 00046

›m electronic annotation /// 0003676 // nucleic acid binding // inferred from electronic annotation /// 0003723

om electronic annotation /// 0003774 // motor activity // inferred from electronic annotation /// 0003779 // ac
/ // inferred from mutant phenotype /// 0005515 // protein binding // inferred from electronic annotation

peptide beta-1,4-galactosyltransferase activity // inferred from direct assay /// 0005488 // binding // inferred fr

ect assay /// 0003677 // DNA binding // inferred from electronic annotation /// 0003700 // sequence-specific D
electronic annotation

om electronic annotation /// 0004672 // protein kinase activity // inferred from direct assay /// 0004672 // prot
/// 0005739 // mitochondrion // inferred from electronic annotation
om electronic annotation /// 0005515 // protein binding // inferred from electronic annotation /// 0005525 // G

om electronic annotation /// 0003676 // nucleic acid binding // inferred from electronic annotation /// 0003723
om electronic annotation /// 0003924 // GTPase activity // inferred from electronic annotation /// 0005515 // p
om electronic annotation /// 0000287 // magnesium ion binding // inferred from electronic annotation /// 0004

electronic annotation /// 0008168 // methyltransferase activity // inferred from electronic annotation /// 0008
ctronic annotation
electronic annotation /// 0008415 // acyltransferase activity // inferred from electronic annotation /// 00167
rom electronic annotation
electronic annotation
om electronic annotation /// 0003676 // nucleic acid binding // inferred from electronic annotation /// 0003723
tation /// 0016021 // integral to membrane // inferred from electronic annotation
or activity // inferred from electronic annotation /// 0005515 // protein binding // inferred from electronic ann
on
om electronic annotation /// 0005524 // ATP binding // inferred from electronic annotation /// 0016301 // kinas

on
electronic annotation

rom electronic annotation
or activity // inferred from electronic annotation
on /// 0005730 // nucleolus // inferred from electronic annotation
rom electronic annotation /// 0003677 // DNA binding // inferred from electronic annotation /// 0008270 // zin
rom electronic annotation /// 0005515 // protein binding // inferred from physical interaction /// 0005515 // pr
electronic annotation /// 0004721 // phosphoprotein phosphatase activity // inferred from electronic annotati
physical interaction
or activity // inferred from mutant phenotype /// 0005085 // guanyl-nucleotide exchange factor activity // infer
d from electronic annotation /// 0004298 // threonine-type endopeptidase activity // inferred from electronic a
om electronic annotation /// 0004842 // ubiquitin-protein ligase activity // not recorded /// 0004842 // ubiquiti
ctronic annotation /// 0008270 // zinc ion binding // inferred from electronic annotation /// 0046872 // metal i
electronic annotation /// 0017154 // semaphorin receptor activity // inferred from genetic interaction /// 001

se activity // inferred from electronic annotation /// 0016853 // isomerase activity // inferred from electronic ar

ı electronic annotation /// 0003941 // L-serine ammonia-lyase activity // inferred from electronic annotation ///

ıic annotation
om electronic annotation /// 0003676 // nucleic acid binding // inferred from electronic annotation /// 0003723

rom electronic annotation /// 0008270 // zinc ion binding // inferred from electronic annotation /// 0046872 //

ect assay /// 0003677 // DNA binding // not recorded /// 0003677 // DNA binding // inferred from electronic an
ne // inferred from electronic annotation
ferred from electronic annotation
m electronic annotation

electronic annotation /// 0008270 // zinc ion binding // inferred from electronic annotation /// 0046872 // met
on
tation /// 0016021 // integral to membrane // inferred from electronic annotation

electronic annotation
electronic annotation
ot recorded /// 0004143 // diacylglycerol kinase activity // inferred from electronic annotation /// 0042803 // p
ed from electronic annotation /// 0003824 // catalytic activity // inferred from electronic annotation /// 001674
/ inferred from electronic annotation /// 0005515 // protein binding // inferred from physical interaction /// 00
om electronic annotation /// 0008514 // organic anion transmembrane transporter activity // not recorded ///
nstituent // inferred from electronic annotation /// 0005488 // binding // inferred from electronic annotation /
ctronic annotation /// 0003690 // double-stranded DNA binding // not recorded /// 0003690 // double-strande
rom electronic annotation /// 0003677 // DNA binding // inferred from direct assay /// 0003677 // DNA binding
om electronic annotation /// 0004672 // protein kinase activity // inferred from direct assay /// 0004672 // prot
ı electronic annotation /// 0004721 // phosphoprotein phosphatase activity // inferred from electronic annotati
physical interaction /// 0008017 // microtubule binding // not recorded /// 0008017 // microtubule binding // i
electronic annotation
om electronic annotation /// 0003676 // nucleic acid binding // inferred from electronic annotation /// 0003723
om electronic annotation /// 0000287 // magnesium ion binding // inferred from direct assay /// 0004672 // prc
ıic annotation
ı physical interaction /// 0005102 // receptor binding // inferred from electronic annotation
ctronic annotation /// 0005200 // structural constituent of cytoskeleton // inferred from electronic annotation
ctronic annotation /// 0003682 // chromatin binding // inferred from direct assay /// 0005515 // protein bindin

electronic annotation
physical interaction /// 0005515 // protein binding // inferred from electronic annotation /// 0046872 // metal
ı electronic annotation /// 0005515 // protein binding // inferred from physical interaction /// 0005515 // prote
electronic annotation

ctronic annotation /// 0003725 // double-stranded RNA binding // inferred from electronic annotation /// 0005
om electronic annotation /// 0003676 // nucleic acid binding // inferred from electronic annotation /// 0003723
om electronic annotation /// 0004143 // diacylglycerol kinase activity // inferred from direct assay /// 0004143 /
om electronic annotation /// 0003824 // catalytic activity // inferred from electronic annotation /// 0005524 // /
d from electronic annotation /// 0005515 // protein binding // inferred from electronic annotation

on

electronic annotation
om electronic annotation /// 0003682 // chromatin binding // inferred from sequence or structural similarity ///
ivity // inferred from electronic annotation /// 0015288 // porin activity // inferred from electronic annotation
ctronic annotation /// 0003690 // double-stranded DNA binding // not recorded
tation /// 0016021 // integral to membrane // inferred from electronic annotation
ic annotation /// 0005515 // protein binding // inferred from physical interaction /// 0005515 // protein bindin
rom electronic annotation /// 0003700 // sequence-specific DNA binding transcription factor activity // inferred

electronic annotation
rom electronic annotation /// 0003700 // sequence-specific DNA binding transcription factor activity // inferred
tation
ed from electronic annotation /// 0008233 // peptidase activity // inferred from electronic annotation /// 0008
red from electronic annotation /// 0005097 // Rab GTPase activator activity // inferred from electronic annotati
rom electronic annotation /// 0003677 // DNA binding // inferred from electronic annotation /// 0046872 // me

ect assay /// 0003677 // DNA binding // inferred from electronic annotation /// 0003690 // double-stranded DN
electronic annotation
electronic annotation /// 0008270 // zinc ion binding // inferred from electronic annotation /// 0016301 // kina
om electronic annotation /// 0005524 // ATP binding // inferred from electronic annotation /// 0017111 // nucl
ı electronic annotation /// 0004888 // transmembrane receptor activity // traceable author statement /// 0004

ranscription factor activity // inferred from direct assay /// 0003713 // transcription coactivator activity // infer

rom electronic annotation /// 0003677 // DNA binding // inferred from electronic annotation /// 0003700 // sec

tation /// 0016021 // integral to membrane // inferred from electronic annotation
om electronic annotation /// 0004016 // adenylate cyclase activity // inferred from genetic interaction /// 0004
/ inferred from sequence or structural similarity /// 0005515 // protein binding // inferred from electronic anno
rom electronic annotation /// 0008270 // zinc ion binding // inferred from electronic annotation /// 0046872 //
tation /// 0016021 // integral to membrane // inferred from electronic annotation

ı electronic annotation /// 0005216 // ion channel activity // inferred from electronic annotation /// 0005261 //
rom electronic annotation /// 0008270 // zinc ion binding // inferred from electronic annotation /// 0046872 //
om electronic annotation /// 0003824 // catalytic activity // inferred from electronic annotation /// 0004329 // [illegible]

ferred from electronic annotation
ectronic annotation
tivity // inferred from direct assay /// 0004721 // phosphoprotein phosphatase activity // inferred from electror
physical interaction /// 0005515 // protein binding // inferred from electronic annotation /// 0016175 // superc
ic annotation /// 0005515 // protein binding // inferred from electronic annotation
om electronic annotation /// 0005515 // protein binding // inferred from physical interaction /// 0005515 // pro
electronic annotation

otation /// 0045202 // synapse // inferred from electronic annotation
om electronic annotation
red from electronic annotation /// 0005102 // receptor binding // inferred from physical interaction /// 000510
om electronic annotation /// 0005515 // protein binding // inferred from electronic annotation /// 0005524 // A

om direct assay /// 0005216 // ion channel activity // inferred from mutant phenotype /// 0005216 // ion chanı

om electronic annotation /// 0003676 // nucleic acid binding // inferred from electronic annotation /// 0003723

red from electronic annotation /// 0004872 // receptor activity // inferred from electronic annotation /// 0005[illegible]

rom electronic annotation /// 0003677 // DNA binding // inferred from sequence or structural similarity /// 000
)5634 // nucleus // inferred from electronic annotation

red from electronic annotation /// 0005057 // receptor signaling protein activity // inferred from electronic anr
red from electronic annotation /// 0004872 // receptor activity // inferred from electronic annotation /// 0004[illegible]
om electronic annotation /// 0005515 // protein binding // inferred from electronic annotation
om electronic annotation /// 0004672 // protein kinase activity // inferred from electronic annotation /// 00046

d from electronic annotation /// 0004222 // metalloendopeptidase activity // inferred from electronic annotati
ect assay /// 0003824 // catalytic activity // inferred from electronic annotation /// 0004601 // peroxidase activ
ect assay /// 0003677 // DNA binding // inferred from electronic annotation /// 0003700 // sequence-specific D
ne // inferred from electronic annotation /// 0005515 // protein binding // inferred from electronic annotation
ysical interaction /// 0003677 // DNA binding // not recorded /// 0003677 // DNA binding // inferred from elect

tation /// 0016021 // integral to membrane // inferred from electronic annotation
tation
rom electronic annotation /// 0004386 // helicase activity // inferred from electronic annotation /// 0005524 //
ty // inferred from electronic annotation
ı electronic annotation /// 0005216 // ion channel activity // inferred from electronic annotation /// 0005261 //
om electronic annotation /// 0030528 // transcription regulator activity // inferred from electronic annotation

or activity // inferred from electronic annotation /// 0008270 // zinc ion binding // inferred from electronic ann

ic annotation /// 0005515 // protein binding // inferred from physical interaction /// 0005515 // protein bindin

electronic annotation /// 0004470 // malic enzyme activity // inferred from electronic annotation /// 0005488
ctronic annotation /// 0005515 // protein binding // inferred from physical interaction

ctronic annotation /// 0003700 // sequence-specific DNA binding transcription factor activity // inferred from e
ect assay /// 0003713 // transcription coactivator activity // not recorded /// 0005515 // protein binding // infe

om electronic annotation /// 0005244 // voltage-gated ion channel activity // inferred from electronic annotati

om electronic annotation /// 0005244 // voltage-gated ion channel activity // inferred from electronic annotati
ctronic annotation /// 0005515 // protein binding // inferred from electronic annotation /// 0008270 // zinc ior
// inferred from direct assay /// 0003779 // actin binding // inferred from direct assay /// 0003779 // actin binc
corded /// 0070492 // oligosaccharide binding // inferred from electronic annotation
om electronic annotation /// 0003824 // catalytic activity // inferred from electronic annotation /// 0004112 //

nic annotation

om electronic annotation /// 0003774 // motor activity // inferred from electronic annotation /// 0003779 // ac

electronic annotation
electronic annotation
electronic annotation
electronic annotation /// 0005515 // protein binding // inferred from physical interaction /// 0005515 // prote

/ inferred from electronic annotation /// 0004842 // ubiquitin-protein ligase activity // inferred from sequence
om electronic annotation /// 0005509 // calcium ion binding // not recorded /// 0005509 // calcium ion binding
mutant phenotype
on /// 0005730 // nucleolus // inferred from electronic annotation /// 0016020 // membrane // inferred from e

electronic annotation /// 0016765 // transferase activity, transferring alkyl or aryl (other than methyl) groups /
ctronic annotation
om electronic annotation /// 0000827 // inositol 1,3,4,5,6-pentakisphosphate kinase activity // inferred from ele
0003677 // DNA binding // inferred from electronic annotation /// 0003700 // sequence-specific DNA binding tr

electronic annotation /// 0005488 // binding // inferred from electronic annotation /// 0016491 // oxidoreduct

ng // inferred from electronic annotation /// 0003924 // GTPase activity // inferred from sequence or structural
nic annotation /// 0005488 // binding // inferred from electronic annotation /// 0005515 // protein binding // ir
nteraction /// 0005515 // protein binding // inferred from electronic annotation
r structural similarity /// 0005515 // protein binding // inferred from electronic annotation /// 0008233 // pepti
on
' // DNA binding // inferred from electronic annotation /// 0003723 // RNA binding // inferred from direct assay

509 // calcium ion binding // inferred from sequence or structural similarity /// 0005515 // protein binding // in
olase activity, acting on acid anhydrides, catalyzing transmembrane movement of substances // inferred from ele
0005506 // iron ion binding // inferred from electronic annotation /// 0016491 // oxidoreductase activity // infer
cell factor receptor binding // inferred from electronic annotation /// 0005515 // protein binding // inferred fro
cell factor receptor binding // inferred from electronic annotation /// 0005515 // protein binding // inferred fro
red from electronic annotation
formate-tetrahydrofolate ligase activity // inferred from electronic annotation /// 0004477 // methenyltetrahyd
; // RNA binding // inferred from direct assay /// 0003723 // RNA binding // inferred from electronic annotation
on /// 0030159 // receptor signaling complex scaffold activity // inferred from electronic annotation /// 003015
al interaction /// 0005179 // hormone activity // inferred from electronic annotation /// 0005515 // protein bin
/ heparin binding // inferred from direct assay

otein binding // inferred from physical interaction
nding // inferred from electronic annotation

ATP binding // inferred from electronic annotation /// 0016787 // hydrolase activity // inferred from electronic a
NA binding transcription factor activity // inferred from direct assay /// 0003700 // sequence-specific DNA bind
ATP binding // inferred from electronic annotation /// 0051082 // unfolded protein binding // inferred from elec
ion /// 0005097 // Rab GTPase activator activity // inferred from direct assay
dulin binding // inferred from electronic annotation /// 0031727 // CCR2 chemokine receptor binding // not rec

on binding // inferred from electronic annotation

tein-lysine 6-oxidase activity // inferred from electronic annotation /// 0005507 // copper ion binding // not rec

/ /// 0003677 // DNA binding // inferred from direct assay /// 0003677 // DNA binding // inferred from electron

; // RNA binding // inferred from electronic annotation

phosphoribosylamine-glycine ligase activity // inferred from electronic annotation /// 0004641 // phosphoribos

P-glucose 6-dehydrogenase activity // inferred from electronic annotation /// 0005488 // binding // inferred fro

e // inferred from electronic annotation /// 0005765 // lysosomal membrane // not recorded /// 0005765 // lys
e // inferred from electronic annotation /// 0005765 // lysosomal membrane // not recorded /// 0005765 // lys
electronic annotation
red from electronic annotation /// 0015421 // oligopeptide-transporting ATPase activity // inferred from seque

erred from electronic annotation /// 0016740 // transferase activity // inferred from electronic annotation

activity // inferred from direct assay /// 0016538 // cyclin-dependent protein kinase regulator activity // inferre
nnotation /// 0042277 // peptide binding // inferred from electronic annotation
74 // protein serine/threonine kinase activity // inferred from electronic annotation /// 0004676 // 3-phosphoi
annotation /// 0016787 // hydrolase activity // inferred from electronic annotation
e transporter activity // inferred from electronic annotation
e transporter activity // inferred from electronic annotation

75 // neutral amino acid transmembrane transporter activity // inferred from direct assay /// 0015194 // L-serir
n C-terminus binding // not recorded /// 0008022 // protein C-terminus binding // inferred from electronic ann
notation /// 0003690 // double-stranded DNA binding // not recorded /// 0003714 // transcription corepressor
ect assay /// 0005201 // extracellular matrix structural constituent // inferred from electronic annotation /// 00
' // DNA binding // inferred from electronic annotation /// 0004386 // helicase activity // inferred from electron
672 // protein kinase activity // inferred from electronic annotation /// 0004674 // protein serine/threonine kir
672 // protein kinase activity // inferred from electronic annotation /// 0004674 // protein serine/threonine kir
/ inferred from electronic annotation /// 0003729 // mRNA binding // inferred from direct assay /// 0005487 //
e activity // not recorded /// 0004674 // protein serine/threonine kinase activity // inferred from electronic ann
llagen binding // inferred from direct assay /// 0043237 // laminin-1 binding // inferred from direct assay /// 00
ded /// 0004014 // adenosylmethionine decarboxylase activity // inferred from electronic annotation /// 00055

/ 0008237 // metallopeptidase activity // inferred from direct assay /// 0008237 // metallopeptidase activity //
74 // protein serine/threonine kinase activity // inferred from electronic annotation /// 0004693 // cyclin-deper
n heterodimerization activity // not recorded /// 0046982 // protein heterodimerization activity // inferred from
n heterodimerization activity // not recorded /// 0046982 // protein heterodimerization activity // inferred from

g // inferred from electronic annotation /// 0008565 // protein transporter activity // inferred from direct assay
/ 0005737 // cytoplasm // inferred from electronic annotation
003676 // nucleic acid binding // inferred from electronic annotation /// 0003723 // RNA binding // inferred fro
NA binding transcription factor activity // inferred from direct assay /// 0003700 // sequence-specific DNA bind
NA binding transcription factor activity // inferred from direct assay /// 0003700 // sequence-specific DNA bind
ronic annotation /// 0033829 // O-fucosylpeptide 3-beta-N-acetylglucosaminyltransferase activity // inferred fro

otein binding // inferred from electronic annotation /// 0005524 // ATP binding // inferred from electronic anno

electronic annotation /// 0015171 // amino acid transmembrane transporter activity // inferred from electroni

erred from electronic annotation /// 0005515 // protein binding // inferred from electronic annotation

:ubule binding // inferred from direct assay /// 0008017 // microtubule binding // not recorded /// 0016787 // h
ic annotation /// 0008234 // cysteine-type peptidase activity // inferred from electronic annotation /// 0030247
/// 0005102 // receptor binding // inferred from electronic annotation
cid receptor activity // not recorded /// 0001619 // lysosphingolipid and lysophosphatidic acid receptor activity
'13 // protein tyrosine kinase activity // inferred from electronic annotation /// 0004714 // transmembrane rece
lectronic annotation /// 0005515 // protein binding // inferred from physical interaction /// 0030528 // transcri
m electronic annotation /// 0008138 // protein tyrosine/serine/threonine phosphatase activity // inferred from
n /// 0008236 // serine-type peptidase activity // inferred from electronic annotation /// 0016787 // hydrolase
tivity // inferred from mutant phenotype /// 0004784 // superoxide dismutase activity // not recorded /// 00047

protein binding // inferred from electronic annotation /// 0008270 // zinc ion binding // inferred from electroni
spholipase activity // not recorded /// 0004622 // lysophospholipase activity // inferred from electronic annotat
nic annotation /// 0005515 // protein binding // inferred from electronic annotation /// 0008289 // lipid binding
etylcholine receptor inhibitor activity // inferred from direct assay
notation /// 0016798 // hydrolase activity, acting on glycosyl bonds // inferred from electronic annotation
gnesium ion binding // inferred from electronic annotation /// 0004672 // protein kinase activity // inferred fro

n
nding // inferred from electronic annotation /// 0008233 // peptidase activity // inferred from electronic annot
nding // inferred from electronic annotation /// 0008233 // peptidase activity // inferred from electronic annot
ntaining monooxygenase activity // not recorded /// 0004499 // flavin-containing monooxygenase activity // inf

// cation:chloride symporter activity // inferred from electronic annotation

receptor binding // inferred from electronic annotation /// 0005515 // protein binding // inferred from electror

microfilament motor activity // inferred from sequence or structural similarity /// 0000166 // nucleotide binding
ranscription factor activity // inferred from electronic annotation /// 0005515 // protein binding // inferred from

eoside-triphosphatase activity // inferred from electronic annotation
ng // inferred from electronic annotation /// 0004672 // protein kinase activity // inferred from direct assay ///

uence or structural similarity /// 0005515 // protein binding // inferred from electronic annotation /// 0008233
ization activity // inferred from electronic annotation
/ 0004674 // protein serine/threonine kinase activity // not recorded /// 0004674 // protein serine/threonine k
similarity /// 0005096 // GTPase activator activity // traceable author statement
515 // protein binding // inferred from physical interaction /// 0005515 // protein binding // inferred from elect
ectronic annotation
dium:potassium:chloride symporter activity // not recorded /// 0008511 // sodium:potassium:chloride symport
onic annotation /// 0008083 // growth factor activity // inferred from electronic annotation
brane // inferred from electronic annotation /// 0030054 // cell junction // inferred from electronic annotation

ed from electronic annotation /// 0005515 // protein binding // inferred from physical interaction /// 0005515 /

lase activity // inferred from electronic annotation /// 0016788 // hydrolase activity, acting on ester bonds // in
ding // inferred from electronic annotation /// 0016491 // oxidoreductase activity // inferred from electronic ar
16740 // transferase activity // inferred from electronic annotation /// 0016757 // transferase activity, transfer

ctronic annotation /// 0004772 // sterol O-acyltransferase activity // inferred from direct assay /// 0004772 // s
ctronic annotation /// 0004772 // sterol O-acyltransferase activity // inferred from direct assay /// 0004772 // s
nnotation /// 0005887 // integral to plasma membrane // inferred from electronic annotation /// 0016020 // m
on /// 0015020 // glucuronosyltransferase activity // inferred from electronic annotation /// 0016740 // transfe
n
ator activity // inferred from electronic annotation /// 0020037 // heme binding // inferred from electronic ann
lectronic annotation /// 0043565 // sequence-specific DNA binding // inferred from electronic annotation /// 0(

on /// 0005249 // voltage-gated potassium channel activity // not recorded /// 0005249 // voltage-gated potass
32 // chromatin binding // inferred from electronic annotation /// 0003682 // chromatin binding // inferred fron
rred from electronic annotation
tion /// 0008234 // cysteine-type peptidase activity // inferred from electronic annotation /// 0016787 // hydro
n /// 0003824 // catalytic activity // inferred from electronic annotation /// 0004303 // estradiol 17-beta-dehyc
al similarity /// 0004842 // ubiquitin-protein ligase activity // inferred from electronic annotation /// 0004842 //
al similarity /// 0004842 // ubiquitin-protein ligase activity // inferred from electronic annotation /// 0004842 //
nferred from electronic annotation
: // RNA binding // inferred from electronic annotation /// 0003729 // mRNA binding // inferred from direct assa
sporter activity // inferred from electronic annotation

nferred from physical interaction /// 0005515 // protein binding // inferred from electronic annotation /// 0016
0005329 // dopamine transmembrane transporter activity // not recorded /// 0005333 // norepinephrine trans
: // RNA binding // inferred from electronic annotation /// 0003729 // mRNA binding // inferred from direct assa
lectronic annotation /// 0043565 // sequence-specific DNA binding // inferred from electronic annotation /// 0(

// inferred from electronic annotation
ion binding // inferred from electronic annotation
ion binding // inferred from electronic annotation
lectronic annotation /// 0005515 // protein binding // inferred from physical interaction /// 0005515 // protein
3677 // DNA binding // inferred from electronic annotation /// 0003690 // double-stranded DNA binding // not
receptor binding // inferred from physical interaction
receptor binding // inferred from physical interaction

: // RNA binding // inferred from electronic annotation
// 0016787 // hydrolase activity // inferred from electronic annotation /// 0016790 // thiolester hydrolase activ
activity // inferred from direct assay
endopeptidase activity // inferred from electronic annotation /// 0005524 // ATP binding // inferred from electro
03924 // GTPase activity // inferred from electronic annotation /// 0005525 // GTP binding // inferred from ele
RNA binding // inferred from direct assay

ing // inferred from physical interaction /// 0005515 // protein binding // inferred from electronic annotation //
' protein binding // inferred from electronic annotation /// 0032403 // protein complex binding // not recorded
5604 // basement membrane // inferred from electronic annotation
; // inferred from physical interaction /// 0005515 // protein binding // inferred from electronic annotation /// 0
ctronic annotation /// 0005515 // protein binding // inferred from electronic annotation /// 0005515 // protein
'13 // protein tyrosine kinase activity // not recorded /// 0004713 // protein tyrosine kinase activity // inferred f

5515 // protein binding // inferred from electronic annotation /// 0005543 // phospholipid binding // not record

2 // metal ion binding // inferred from electronic annotation /// 0051082 // unfolded protein binding // inferred
930 // G-protein coupled receptor activity // inferred from electronic annotation /// 0005000 // vasopressin rec
930 // G-protein coupled receptor activity // inferred from electronic annotation /// 0005000 // vasopressin rec

irect assay /// 0003700 // sequence-specific DNA binding transcription factor activity // not recorded /// 00037
irect assay /// 0003700 // sequence-specific DNA binding transcription factor activity // not recorded /// 00037
n C-terminus binding // inferred from electronic annotation
030748 // amine N-methyltransferase activity // inferred from electronic annotation

center // inferred from electronic annotation /// 0005819 // spindle // inferred from electronic annotation /// 0
irect assay /// 0005515 // protein binding // inferred from electronic annotation /// 0008134 // transcription fa

ctivity // inferred from electronic annotation /// 0005086 // ARF guanyl-nucleotide exchange factor activity // ir
lectronic annotation /// 0005515 // protein binding // inferred from electronic annotation /// 0030528 // transc
zinc ion binding // inferred from electronic annotation /// 0046872 // metal ion binding // inferred from electro

vity // inferred from direct assay /// 0004672 // protein kinase activity // inferred from electronic annotation //
notation /// 0003700 // sequence-specific DNA binding transcription factor activity // inferred from electronic a
'/ 0003700 // sequence-specific DNA binding transcription factor activity // inferred from electronic annotation
// thyroxine 5'-deiodinase activity // inferred from electronic annotation /// 0016491 // oxidoreductase activity
) // cytoplasmic vesicle // inferred from electronic annotation
74 // protein serine/threonine kinase activity // inferred from electronic annotation /// 0004683 // calmodulin-

1-monooxygenase activity // not recorded /// 0004509 // steroid 21-monooxygenase activity // inferred from el
1-monooxygenase activity // not recorded /// 0004509 // steroid 21-monooxygenase activity // inferred from el
'/ 0047485 // protein N-terminus binding // inferred from electronic annotation
d /// 0004725 // protein tyrosine phosphatase activity // inferred from electronic annotation /// 0005488 // bin

// protein binding // inferred from physical interaction /// 0005525 // GTP binding // inferred from electronic a
dium channel activity // inferred from electronic annotation /// 0005515 // protein binding // inferred from ele
0016020 // membrane // inferred from electronic annotation /// 0016021 // integral to membrane // inferred fr
nferred from electronic annotation /// 0008083 // growth factor activity // inferred from electronic annotation /

binding // inferred from electronic annotation /// 0004340 // glucokinase activity // inferred from direct assay /

74 // protein serine/threonine kinase activity // inferred from electronic annotation /// 0004707 // MAP kinase
n binding // inferred from electronic annotation /// 0016566 // specific transcriptional repressor activity // infer
ing // inferred from physical interaction /// 0008009 // chemokine activity // inferred from electronic annotatio
/// 0005515 // protein binding // inferred from physical interaction /// 0005515 // protein binding // inferred fr
ation /// 0016627 // oxidoreductase activity, acting on the CH-CH group of donors // inferred from electronic ar
sphate beta-D-mannosyltransferase activity // inferred from mutant phenotype /// 0004582 // dolichyl-phosph

olymerase II transcription mediator activity // inferred from electronic annotation

ion

n /// 0004871 // signal transducer activity // inferred from electronic annotation /// 0004872 // receptor activit
tivity // inferred from electronic annotation /// 0004674 // protein serine/threonine kinase activity // inferred f
okine activity // inferred from electronic annotation /// 0005515 // protein binding // inferred from electronic a
gen-exporting ATPase activity, phosphorylative mechanism // inferred from direct assay /// 0016820 // hydrolas
al ion binding // inferred from electronic annotation

ase activity // inferred from electronic annotation /// 0017111 // nucleoside-triphosphatase activity // inferred f
c ion binding // inferred from electronic annotation /// 0016563 // transcription activator activity // not recorde
/// 0008201 // heparin binding // not recorded /// 0008201 // heparin binding // inferred from electronic annc
15 // protein binding // inferred from electronic annotation

ivity // inferred from electronic annotation /// 0003779 // actin binding // non-traceable author statement /// 0
otation /// 0005515 // protein binding // inferred from physical interaction /// 0005515 // protein binding // inf
5515 // protein binding // inferred from physical interaction
ctronic annotation /// 0004563 // beta-N-acetylhexosaminidase activity // inferred from direct assay /// 00045
0008270 // zinc ion binding // inferred from electronic annotation /// 0016874 // ligase activity // inferred from

cific DNA binding transcription factor activity // traceable author statement /// 0003700 // sequence-specific DI
/ // DNA binding // inferred from direct assay /// 0003677 // DNA binding // inferred from electronic annotation
15026 // coreceptor activity // inferred from direct assay /// 0015026 // coreceptor activity // not recorded
one binding // inferred from electronic annotation

0003743 // translation initiation factor activity // inferred from sequence or structural similarity /// 0005515 //

imilarity /// 0009986 // cell surface // inferred from electronic annotation /// 0009986 // cell surface // inferrec

n ion binding // inferred from electronic annotation /// 0016787 // hydrolase activity // inferred from electronic
/ zinc ion binding // inferred from electronic annotation /// 0016874 // ligase activity // inferred from electroni
embrane // non-traceable author statement

:ed from mutant phenotype /// 0007283 // spermatogenesis // inferred from mutant phenotype /// 0009566 //
/// 0008233 // peptidase activity // inferred from electronic annotation /// 0008236 // serine-type peptidase a

930 // G-protein coupled receptor activity // traceable author statement /// 0004930 // G-protein coupled recep
annotation
:in binding // not recorded
5515 // protein binding // inferred from electronic annotation /// 0008013 // beta-catenin binding // not record
prostaglandin transmembrane transporter activity // inferred from direct assay
ise activity // inferred from electronic annotation
' // DNA binding // inferred from electronic annotation /// 0003682 // chromatin binding // inferred from direct

/// 0004842 // ubiquitin-protein ligase activity // inferred from electronic annotation /// 0005515 // protein bin
notation /// 0003700 // sequence-specific DNA binding transcription factor activity // inferred from direct assay
ranscription factor activity // inferred from electronic annotation /// 0004871 // signal transducer activity // infe
nding // not recorded /// 0005529 // sugar binding // not recorded /// 0005529 // sugar binding // inferred fron

96 // oxysterol 7-alpha-hydroxylase activity // inferred from direct assay /// 0009055 // electron carrier activity ,
96 // oxysterol 7-alpha-hydroxylase activity // inferred from direct assay /// 0009055 // electron carrier activity ,

:ein kinase activity // inferred from electronic annotation /// 0004674 // protein serine/threonine kinase activity
: annotation /// 0008233 // peptidase activity // inferred from electronic annotation /// 0016787 // hydrolase a

notation /// 0003700 // sequence-specific DNA binding transcription factor activity // inferred from electronic a
ling // inferred from electronic annotation /// 0005515 // protein binding // inferred from physical interaction /,
r activity // inferred from electronic annotation /// 0005216 // ion channel activity // inferred from electronic a
otide binding // not recorded
l ion binding // inferred from electronic annotation
ATP binding // inferred from electronic annotation /// 0008022 // protein C-terminus binding // inferred from el
897 // ciliary neurotrophic factor receptor activity // inferred from electronic annotation /// 0004915 // interleu
/// 0008233 // peptidase activity // inferred from electronic annotation /// 0008236 // serine-type peptidase a
: // RNA binding // inferred from electronic annotation /// 0003729 // mRNA binding // inferred from electronic
// inferred from electronic annotation /// 0003700 // sequence-specific DNA binding transcription factor activit

// long-chain fatty acid transporter activity // not recorded /// 0005524 // ATP binding // inferred from electron
c ion binding // inferred from electronic annotation /// 0046872 // metal ion binding // inferred from electronic
nic annotation /// 0004115 // 3',5'-cyclic-AMP phosphodiesterase activity // not recorded /// 0004115 // 3',5'-c
quence-specific DNA binding transcription factor activity // inferred from direct assay /// 0008270 // zinc ion bin
on /// 0004725 // protein tyrosine phosphatase activity // inferred from direct assay /// 0004725 // protein tyro
rom electronic annotation /// 0016740 // transferase activity // inferred from electronic annotation /// 001675
13 // protein tyrosine kinase activity // inferred from electronic annotation /// 0004715 // non-membrane spar
ng // inferred from electronic annotation

n electronic annotation /// 0005488 // binding // inferred from electronic annotation /// 0005515 // protein bi
)5524 // ATP binding // inferred from electronic annotation /// 0005525 // GTP binding // inferred from electror
// ATP binding // inferred from electronic annotation /// 0015075 // ion transmembrane transporter activity //
se activity // inferred from electronic annotation /// 0016298 // lipase activity // not recorded /// 0016298 // lip
tion /// 0005088 // Ras guanyl-nucleotide exchange factor activity // inferred from mutant phenotype /// 0005
nferred from physical interaction /// 0016563 // transcription activator activity // inferred from direct assay ///

/ 0005154 // epidermal growth factor receptor binding // not recorded /// 0005154 // epidermal growth factor

quence-specific DNA binding transcription factor activity // traceable author statement /// 0008270 // zinc ion b

930 // G-protein coupled receptor activity // inferred from electronic annotation /// 0004977 // melanocortin re

se activity // inferred from electronic annotation /// 0004798 // thymidylate kinase activity // inferred from ele
// RNA binding // inferred from electronic annotation /// 0004672 // protein kinase activity // traceable autho

ein binding // inferred from physical interaction /// 0005515 // protein binding // inferred from electronic anno

16208 // AMP binding // not recorded /// 0016491 // oxidoreductase activity // inferred from electronic annota
lectronic annotation
930 // G-protein coupled receptor activity // inferred from electronic annotation /// 0004995 // tachykinin rece
electronic annotation /// 0005515 // protein binding // inferred from physical interaction /// 0008083 // grow
oteinaceous extracellular matrix // not recorded /// 0005578 // proteinaceous extracellular matrix // inferred fr
lectronic annotation /// 0030528 // transcription regulator activity // inferred from electronic annotation
lectronic annotation /// 0030528 // transcription regulator activity // inferred from electronic annotation /// 0C

ion
787 // hydrolase activity // inferred from electronic annotation /// 0019899 // enzyme binding // not recorded
ic annotation /// 0005547 // phosphatidylinositol-3,4,5-trisphosphate binding // inferred from sequence or stru
annotation /// 0016787 // hydrolase activity // inferred from electronic annotation /// 0016798 // hydrolase ac
nic annotation /// 0008324 // cation transmembrane transporter activity // inferred from electronic annotation
protein binding // inferred from electronic annotation
0005515 // protein binding // inferred from electronic annotation /// 0008201 // heparin binding // inferred fro
21 // integral to membrane // inferred from electronic annotation

r statement /// 0003700 // sequence-specific DNA binding transcription factor activity // inferred from electror
tion /// 0008234 // cysteine-type peptidase activity // inferred from electronic annotation /// 0016787 // hydro
// RNA binding // inferred from electronic annotation /// 0005515 // protein binding // inferred from electroni
e) O-methyltransferase activity // not recorded /// 0004719 // protein-L-isoaspartate (D-aspartate) O-methyltra
inding // inferred from direct assay
713 // transcription coactivator activity // inferred from electronic annotation /// 0005488 // binding // inferred

plasma membrane // inferred from electronic annotation /// 0016020 // membrane // inferred from electronic
rred from electronic annotation /// 0017127 // cholesterol transporter activity // inferred from electronic annot
/ inferred from electronic annotation /// 0005459 // UDP-galactose transmembrane transporter activity // infer
notation /// 0030528 // transcription regulator activity // inferred from electronic annotation /// 0046872 // me

ne // inferred from electronic annotation
) // flavin-containing monooxygenase activity // inferred from electronic annotation /// 0016491 // oxidoreduct
// 0005515 // protein binding // inferred from physical interaction
402 // histone acetyltransferase activity // inferred from direct assay /// 0004402 // histone acetyltransferase ac

'13 // protein tyrosine kinase activity // not recorded /// 0004713 // protein tyrosine kinase activity // inferred f

hoinositide binding // inferred from direct assay /// 0035091 // phosphoinositide binding // inferred from electr
/ 0016491 // oxidoreductase activity // inferred from electronic annotation
nicrotubule minus-end binding // not recorded

ction /// 0005515 // protein binding // inferred from electronic annotation /// 0030674 // protein binding, bridg
omain binding // inferred from electronic annotation
eceptor activity // inferred from mutant phenotype /// 0008035 // high-density lipoprotein binding // inferred f
onic annotation /// 0005515 // protein binding // inferred from physical interaction /// 0005515 // protein bind

inferred from electronic annotation /// 0016021 // integral to membrane // inferred from electronic annotatior
rded /// 0008489 // UDP-galactose:glucosylceramide beta-1,4-galactosyltransferase activity // inferred from ele
direct assay /// 0017124 // SH3 domain binding // inferred from electronic annotation /// 0046872 // metal ior
55 // electron carrier activity // inferred from electronic annotation /// 0016491 // oxidoreductase activity // inf

cific DNA binding transcription factor activity // inferred from direct assay /// 0003700 // sequence-specific DN/
// deoxyribonuclease activity // inferred from electronic annotation /// 0005515 // protein binding // inferred

125 // bile acid transmembrane transporter activity // inferred from electronic annotation
on /// 0005245 // voltage-gated calcium channel activity // inferred from direct assay /// 0005245 // voltage-ga
tracellular matrix binding // inferred from direct assay
ic annotation /// 0008289 // lipid binding // not recorded /// 0008289 // lipid binding // inferred from electron
NA binding transcription factor activity // inferred from direct assay /// 0003700 // sequence-specific DNA bind
g // inferred from electronic annotation

/// 0003824 // catalytic activity // inferred from electronic annotation /// 0005488 // binding // inferred from e
annotation /// 0016021 // integral to membrane // inferred from electronic annotation
nferred from physical interaction /// 0005524 // ATP binding // inferred from direct assay

ferred from electronic annotation /// 0004872 // receptor activity // inferred from electronic annotation /// 000
elin-B receptor activity // inferred from electronic annotation /// 0004871 // signal transducer activity // inferre
)287 // magnesium ion binding // inferred from sequence or structural similarity /// 0004672 // protein kinase a

alkaline phosphatase activity // not recorded /// 0004035 // alkaline phosphatase activity // inferred from elect

al ion binding // inferred from electronic annotation
ı electronic annotation /// 0005515 // protein binding // inferred from physical interaction /// 0005515 // prote
leoyl-CoA desaturase activity // not recorded /// 0016491 // oxidoreductase activity // inferred from electronic
rred from electronic annotation /// 0005515 // protein binding // inferred from electronic annotation
ıbrane // inferred from electronic annotation /// 0016021 // integral to membrane // inferred from electronic a

orane // inferred from direct assay /// 0005743 // mitochondrial inner membrane // inferred from electronic an
' // DNA binding // inferred from electronic annotation /// 0003697 // single-stranded DNA binding // inferred f
c ion binding // inferred from electronic annotation /// 0046872 // metal ion binding // inferred from electronic
ydrolase activity, acting on glycosyl bonds // inferred from electronic annotation

electronic annotation /// 0004197 // cysteine-type endopeptidase activity // inferred from sequence or structu
electronic annotation /// 0004197 // cysteine-type endopeptidase activity // inferred from sequence or structu
n C-terminus binding // inferred from electronic annotation
'87 // hydrolase activity // inferred from electronic annotation /// 0019903 // protein phosphatase binding // in

long-chain fatty acid-CoA ligase activity // not recorded /// 0004467 // long-chain fatty acid-CoA ligase activity /
erred from sequence or structural similarity /// 0005515 // protein binding // inferred from physical interaction
0 // tRNA dihydrouridine synthase activity // inferred from electronic annotation /// 0050660 // FAD or FADH2
; // RNA binding // inferred from electronic annotation /// 0005515 // protein binding // inferred from physical i
ıbrane // inferred from electronic annotation
zinc ion binding // inferred from electronic annotation /// 0016491 // oxidoreductase activity // inferred from e
vity // inferred from electronic annotation /// 0004713 // protein tyrosine kinase activity // inferred from electr
/ 0008234 // cysteine-type peptidase activity // inferred from electronic annotation /// 0016787 // hydrolase ac
' inferred from electronic annotation /// 0016787 // hydrolase activity // inferred from electronic annotation //
RNA-3'-phosphate cyclase activity // inferred from electronic annotation /// 0005524 // ATP binding // inferred

ictivator activity // inferred from sequence or structural similarity /// 0005515 // protein binding // inferred fror
quence-specific DNA binding transcription factor activity // inferred from electronic annotation /// 0005515 // p
binding // inferred from physical interaction /// 0005515 // protein binding // inferred from electronic annotati
icle // inferred from electronic annotation
ynucleotide adenylyltransferase activity // inferred from electronic annotation /// 0005524 // ATP binding // infe

ı /// 0003899 // DNA-directed RNA polymerase activity // inferred from sequence or structural similarity
ıgnesium ion binding // inferred from electronic annotation /// 0004672 // protein kinase activity // inferred fro
lectronic annotation /// 0005515 // protein binding // inferred from electronic annotation /// 0030528 // transc
lectronic annotation /// 0005515 // protein binding // inferred from electronic annotation /// 0030528 // transc

ctivity // inferred from electronic annotation /// 0016740 // transferase activity // inferred from electronic anno
i74 // protein serine/threonine kinase activity // inferred from sequence or structural similarity /// 0004674 // p
ctronic annotation /// 0016829 // lyase activity // inferred from electronic annotation /// 0016831 // carboxy-ly

/ inferred from sequence or structural similarity /// 0004067 // asparaginase activity // not recorded /// 000406
aldehyde dehydrogenase [NAD(P)+] activity // not recorded /// 0008168 // methyltransferase activity // inferre
inferred from electronic annotation /// 0010008 // endosome membrane // inferred from electronic annotatior
// inferred from electronic annotation
ation /// 0016787 // hydrolase activity // inferred from electronic annotation /// 0046872 // metal ion binding /

// protein binding // inferred from electronic annotation
3 // RNA binding // inferred from electronic annotation /// 0004386 // helicase activity // inferred from electron
e activity // inferred from electronic annotation /// 0042803 // protein homodimerization activity // inferred fro
tion /// 0003723 // RNA binding // inferred from electronic annotation /// 0004221 // ubiquitin thiolesterase ac

3 // RNA binding // inferred from electronic annotation
nferred from sequence or structural similarity /// 0019899 // enzyme binding // not recorded /// 0019901 // pr
930 // G-protein coupled receptor activity // inferred from electronic annotation /// 0045028 // purinergic nucle
46872 // metal ion binding // inferred from electronic annotation

nding // not recorded /// 0004672 // protein kinase activity // inferred from electronic annotation /// 0004674 /
al ion binding // inferred from electronic annotation

.082 // unfolded protein binding // inferred from electronic annotation

'13 // protein tyrosine kinase activity // inferred from electronic annotation /// 0004713 // protein tyrosine kina

interaction /// 0005515 // protein binding // inferred from electronic annotation
74 // protein serine/threonine kinase activity // inferred from direct assay /// 0004674 // protein serine/threon

'13 // protein tyrosine kinase activity // inferred from direct assay /// 0004713 // protein tyrosine kinase activity
l form four-way junction DNA binding // not recorded /// 0003677 // DNA binding // inferred from electronic an
n

ronic annotation /// 0003700 // sequence-specific DNA binding transcription factor activity // inferred from dire

tin binding // inferred from electronic annotation /// 0005515 // protein binding // inferred from electronic ann
irect assay /// 0003700 // sequence-specific DNA binding transcription factor activity // not recorded /// 00037

or structural similarity /// 0005515 // protein binding // inferred from physical interaction /// 0015078 // hydrog

se activity // inferred from electronic annotation /// 0005488 // binding // inferred from electronic annotation /

metal ion binding // inferred from electronic annotation
:ation
metal ion binding // inferred from electronic annotation
'74 // protein serine/threonine kinase activity // inferred from electronic annotation /// 0004674 // protein seri
)5515 // protein binding // inferred from physical interaction /// 0005515 // protein binding // inferred from ele
'74 // protein serine/threonine kinase activity // inferred from electronic annotation /// 0004702 // receptor sig
ctronic annotation /// 0004563 // beta-N-acetylhexosaminidase activity // inferred from direct assay /// 00045
H3/SH2 adaptor activity // inferred from physical interaction /// 0005158 // insulin receptor binding // inferred
d from electronic annotation /// 0005515 // protein binding // inferred from electronic annotation /// 0016787
from electronic annotation
nferred from electronic annotation /// 0005543 // phospholipid binding // inferred from electronic annotation

otein binding // inferred from electronic annotation /// 0005544 // calcium-dependent phospholipid binding //
ic annotation /// 0004725 // protein tyrosine phosphatase activity // inferred from electronic annotation /// 0
515 // protein binding // inferred from electronic annotation /// 0016515 // interleukin-13 receptor activity // r
0008270 // zinc ion binding // inferred from electronic annotation /// 0046872 // metal ion binding // inferred
'74 // protein serine/threonine kinase activity // not recorded /// 0004674 // protein serine/threonine kinase ac

activity // inferred from electronic annotation /// 0005515 // protein binding // inferred from electronic annota
NA binding transcription factor activity // inferred from direct assay /// 0003700 // sequence-specific DNA bind
inferred from electronic annotation /// 0016021 // integral to membrane // inferred from electronic annotatio
/ inferred from direct assay /// 0003729 // mRNA binding // inferred from direct assay /// 0005515 // protein b
'74 // protein serine/threonine kinase activity // inferred from electronic annotation /// 0004709 // MAP kinase
n /// 0008236 // serine-type peptidase activity // inferred from electronic annotation /// 0016787 // hydrolase
mRNA guanylyltransferase activity // inferred from electronic annotation /// 0004651 // polynucleotide 5'-phos

n /// 0016787 // hydrolase activity // inferred from electronic annotation /// 0016798 // hydrolase activity, acti
erred from electronic annotation /// 0031225 // anchored to membrane // inferred from electronic annotation
ription factor binding // inferred from physical interaction
r activity // inferred from sequence or structural similarity /// 0005515 // protein binding // inferred from elect
lrolase activity // inferred from electronic annotation /// 0046872 // metal ion binding // inferred from electron
)008270 // zinc ion binding // inferred from electronic annotation /// 0016874 // ligase activity // inferred from
ansferase activity // not recorded /// 0003810 // protein-glutamine gamma-glutamyltransferase activity // infer
activator activity // inferred from sequence or structural similarity /// 0005096 // GTPase activator activity // tr
nic annotation /// 0004115 // 3',5'-cyclic-AMP phosphodiesterase activity // not recorded /// 0004115 // 3',5'-c
on /// 0005249 // voltage-gated potassium channel activity // inferred from electronic annotation /// 0005267

ng // inferred from electronic annotation
cell factor receptor binding // inferred from electronic annotation /// 0005515 // protein binding // inferred fro
in binding // inferred from electronic annotation /// 0008289 // lipid binding // inferred from electronic annota
ne // inferred from electronic annotation

) // transferase activity // inferred from electronic annotation /// 0046872 // metal ion binding // inferred from
rase activity // inferred from electronic annotation /// 0005515 // protein binding // inferred from electronic ar

;74 // protein serine/threonine kinase activity // not recorded /// 0004674 // protein serine/threonine kinase ac
zinc ion binding // inferred from electronic annotation /// 0046872 // metal ion binding // inferred from electro
zinc ion binding // inferred from electronic annotation /// 0046872 // metal ion binding // inferred from electro

4872 // receptor activity // inferred from electronic annotation /// 0005515 // protein binding // inferred from p
protein binding // inferred from electronic annotation /// 0008270 // zinc ion binding // inferred from electronic
onic annotation /// 0004871 // signal transducer activity // inferred from electronic annotation /// 0005509 // c
0004222 // metalloendopeptidase activity // inferred from sequence or structural similarity /// 0008233 // pept
// inferred from electronic annotation /// 0003702 // RNA polymerase II transcription factor activity // inferred
organizing center // inferred from electronic annotation /// 0005856 // cytoskeleton // inferred from electronic
receptor binding // not recorded /// 0005524 // ATP binding // inferred from electronic annotation /// 0008092
NA binding transcription factor activity // inferred from direct assay /// 0003700 // sequence-specific DNA bind
binding // inferred from electronic annotation /// 0016491 // oxidoreductase activity // inferred from electron
al ion binding // inferred from electronic annotation

ding // inferred from electronic annotation /// 0005198 // structural molecule activity // inferred from direct ass
9 // L-iditol 2-dehydrogenase activity // inferred from electronic annotation /// 0005488 // binding // inferred fr
se activity // inferred from electronic annotation /// 0046872 // metal ion binding // inferred from electronic ar

red from physical interaction /// 0008201 // heparin binding // inferred from electronic annotation /// 0016504
tin binding // inferred from electronic annotation /// 0005515 // protein binding // inferred from electronic ann
tein binding // inferred from electronic annotation /// 0005524 // ATP binding // inferred from electronic annot
clease activity // inferred from electronic annotation /// 0005515 // protein binding // inferred from electronic
3 // RNA binding // inferred from electronic annotation /// 0005515 // protein binding // inferred from electroni
ement /// 0005515 // protein binding // inferred from physical interaction /// 0005515 // protein binding // infe

)5515 // protein binding // inferred from physical interaction /// 0005515 // protein binding // inferred from ele
g // inferred from electronic annotation /// 0008565 // protein transporter activity // inferred from direct assay
// 0005488 // binding // inferred from electronic annotation /// 0016787 // hydrolase activity // inferred from e

tion
annotation /// 0005737 // cytoplasm // inferred from electronic annotation /// 0005783 // endoplasmic reticulu
ed from electronic annotation /// 0001537 // N-acetylgalactosamine 4-O-sulfotransferase activity // inferred fro
;74 // protein serine/threonine kinase activity // inferred from electronic annotation /// 0005515 // protein binc

nding // inferred from physical interaction /// 0005515 // protein binding // inferred from electronic annotatior
0005515 // protein binding // inferred from electronic annotation /// 0019843 // rRNA binding // inferred from
iTPase activity // inferred from sequence or structural similarity /// 0004842 // ubiquitin-protein ligase activity /
electronic annotation /// 0042803 // protein homodimerization activity // not recorded

c ion binding // inferred from sequence or structural similarity /// 0017124 // SH3 domain binding // inferred fro

/ 0008237 // metallopeptidase activity // inferred from electronic annotation /// 0008270 // zinc ion binding //
e binding // inferred from electronic annotation
/ 0008237 // metallopeptidase activity // inferred from electronic annotation /// 0008270 // zinc ion binding //

guanylate cyclase activity // not recorded /// 0004383 // guanylate cyclase activity // inferred from electronic an
r structural similarity /// 0005515 // protein binding // inferred from electronic annotation /// 0008233 // pepti

lectronic annotation /// 0001883 // purine nucleoside binding // not recorded /// 0001883 // purine nucleoside
: annotation

GTP binding // not recorded /// 0005525 // GTP binding // inferred from electronic annotation /// 0031624 // ub
tin binding // inferred from electronic annotation /// 0005515 // protein binding // inferred from electronic ann
om electronic annotation /// 0005545 // phosphatidylinositol binding // not recorded /// 0008270 // zinc ion bi

nucleotide adenylyltransferase activity // inferred from electronic annotation /// 0005524 // ATP binding // infe

74 // protein serine/threonine kinase activity // inferred from electronic annotation /// 0005524 // ATP binding

nferred from physical interaction /// 0016563 // transcription activator activity // inferred from direct assay /// (
otropic glutamate receptor activity // inferred from electronic annotation /// 0005216 // ion channel activity //
287 // magnesium ion binding // inferred from sequence or structural similarity /// 0004672 // protein kinase a
ivity // inferred from electronic annotation /// 0008233 // peptidase activity // inferred from electronic annotat

metal ion binding // inferred from electronic annotation
otation /// 0046332 // SMAD binding // inferred from physical interaction /// 0048407 // platelet-derived growt

cal protein binding // inferred from physical interaction /// 0042826 // histone deacetylase binding // inferred fr

ctronic annotation /// 0005515 // protein binding // inferred from physical interaction /// 0005515 // protein b

0016787 // hydrolase activity // inferred from electronic annotation
n C-terminus binding // not recorded

3713 // transcription coactivator activity // inferred from electronic annotation /// 0003779 // actin binding // ir

c ion binding // inferred from electronic annotation /// 0030528 // transcription regulator activity // inferred fr

iTP binding // inferred from electronic annotation

m electronic annotation /// 0016787 // hydrolase activity // inferred from electronic annotation /// 0016791 //

notation /// 0016740 // transferase activity // inferred from electronic annotation /// 0016757 // transferase ac

tivity // inferred from direct assay /// 0004074 // biliverdin reductase activity // not recorded /// 0004074 // bil
: // RNA binding // inferred from electronic annotation /// 0004386 // helicase activity // inferred from electron

tivity // inferred from electronic annotation /// 0005515 // protein binding // inferred from physical interaction

ding // inferred from physical interaction /// 0005515 // protein binding // inferred from electronic annotation ,
ectronic annotation
pseudouridine synthase activity // inferred from electronic annotation /// 0016853 // isomerase activity // inferi
calcium-dependent phospholipid binding // not recorded /// 0005544 // calcium-dependent phospholipid bindi

inferred from electronic annotation /// 0017124 // SH3 domain binding // inferred from electronic annotation
'/ 0016491 // oxidoreductase activity // inferred from electronic annotation /// 0016627 // oxidoreductase activ
38 // binding // inferred from electronic annotation /// 0005540 // hyaluronic acid binding // inferred from direc
/// 0008138 // protein tyrosine/serine/threonine phosphatase activity // inferred from electronic annotation //

otation /// 0016853 // isomerase activity // inferred from electronic annotation /// 0043021 // ribonucleoprote

rotein-coupled receptor binding // inferred from electronic annotation /// 0003924 // GTPase activity // not re
/// 0003824 // catalytic activity // inferred from electronic annotation /// 0005488 // binding // inferred from e

nferred from electronic annotation /// 0046872 // metal ion binding // inferred from electronic annotation

'/ 0004829 // threonine-tRNA ligase activity // inferred from electronic annotation /// 0005524 // ATP binding /
0016853 // isomerase activity // inferred from electronic annotation

' protein binding // inferred from electronic annotation
ction /// 0008321 // Ral guanyl-nucleotide exchange factor activity // inferred from direct assay /// 0017016 //
c ion binding // inferred from electronic annotation /// 0046872 // metal ion binding // inferred from electronic

;equence or structural similarity /// 0005157 // macrophage colony-stimulating factor receptor binding // inferr
inferred from electronic annotation /// 0005737 // cytoplasm // inferred from electronic annotation
/// 0008233 // peptidase activity // inferred from electronic annotation /// 0008236 // serine-type peptidase a
lectronic annotation /// 0030528 // transcription regulator activity // inferred from electronic annotation /// 0C
ise activity // traceable author statement /// 0004017 // adenylate kinase activity // inferred from electronic an

;e activity // inferred from electronic annotation /// 0016298 // lipase activity // not recorded /// 0016298 // lip

red from electronic annotation /// 0008144 // drug binding // not recorded /// 0008144 // drug binding // infer
ferred from electronic annotation
rotein binding // inferred from physical interaction /// 0005515 // protein binding // inferred from electronic ar

n /// 0005515 // protein binding // inferred from electronic annotation /// 0019899 // enzyme binding // not re
ictivity // inferred from electronic annotation

inding // inferred from electronic annotation

ed from direct assay /// 0004861 // cyclin-dependent protein kinase inhibitor activity // inferred from electronic

orded /// 0003684 // damaged DNA binding // inferred from electronic annotation /// 0003824 // catalytic activ
brane // inferred from electronic annotation

ivity // inferred from electronic annotation /// 0008233 // peptidase activity // inferred from electronic annotat
ivity // inferred from electronic annotation /// 0008233 // peptidase activity // inferred from electronic annotat

ct assay /// 0005201 // extracellular matrix structural constituent // inferred from electronic annotation /// 00(
n C-terminus binding // not recorded /// 0050839 // cell adhesion molecule binding // not recorded
binding // inferred from electronic annotation
n C-terminus binding // not recorded /// 0008022 // protein C-terminus binding // inferred from electronic ann(
n C-terminus binding // not recorded /// 0008022 // protein C-terminus binding // inferred from electronic ann(

;e activity // inferred from electronic annotation /// 0046872 // metal ion binding // inferred from electronic an
or activity // inferred from mutant phenotype /// 0005515 // protein binding // inferred from physical interactic
tion /// 0016787 // hydrolase activity // inferred from electronic annotation
binding // inferred from electronic annotation /// 0005529 // sugar binding // inferred from electronic annotati
GTP binding // inferred from electronic annotation
tation

quence-specific DNA binding transcription factor activity // traceable author statement /// 0008270 // zinc ion b
ion binding // inferred from sequence or structural similarity /// 0032454 // histone demethylase activity (H3-K
shock protein binding // inferred from electronic annotation

8270 // zinc ion binding // inferred from electronic annotation /// 0016787 // hydrolase activity // inferred from
nding // inferred from electronic annotation /// 0016740 // transferase activity // inferred from electronic annot
notation /// 0005488 // binding // inferred from electronic annotation /// 0008233 // peptidase activity // inferr

ty // inferred from electronic annotation /// 0016740 // transferase activity // inferred from electronic annotati
ase activity // inferred from electronic annotation /// 0017111 // nucleoside-triphosphatase activity // inferred f

zinc ion binding // inferred from electronic annotation /// 0046872 // metal ion binding // inferred from electro

4-nitrophenylphosphatase activity // inferred from mutant phenotype /// 0005391 // sodium:potassium-exchar
' transcription corepressor activity // not recorded /// 0003714 // transcription corepressor activity // inferred f
ription regulator activity // inferred from electronic annotation

'/ integrin binding // not recorded /// 0005198 // structural molecule activity // inferred from electronic annota
n
ctronic annotation /// 0005515 // protein binding // inferred from physical interaction /// 0005515 // protein b

lectronic annotation /// 0005515 // protein binding // inferred from physical interaction /// 0016563 // transcri
m electronic annotation /// 0005488 // binding // inferred from electronic annotation /// 0016787 // hydrolase
nic annotation /// 0008289 // lipid binding // not recorded /// 0008289 // lipid binding // inferred from electron

/ inferred from electronic annotation /// 0005459 // UDP-galactose transmembrane transporter activity // infer

5515 // protein binding // inferred from electronic annotation /// 0005543 // phospholipid binding // not recorc
'/ 0004829 // threonine-tRNA ligase activity // inferred from electronic annotation /// 0005524 // ATP binding /

'// 0016491 // oxidoreductase activity // inferred from direct assay /// 0016491 // oxidoreductase activity // inf

: // RNA binding // inferred from electronic annotation /// 0005515 // protein binding // inferred from electroni
n binding // inferred from physical interaction /// 0005516 // calmodulin binding // inferred from electronic ann
: // RNA binding // inferred from electronic annotation /// 0005515 // protein binding // inferred from electroni

'/ 0003700 // sequence-specific DNA binding transcription factor activity // inferred from electronic annotation

m direct assay /// 0008934 // inositol-1(or 4)-monophosphatase activity // inferred from electronic annotation
: // RNA binding // inferred from electronic annotation /// 0004386 // helicase activity // inferred from electron
tation /// 0016491 // oxidoreductase activity // inferred from electronic annotation /// 0016620 // oxidoreduct
)008270 // zinc ion binding // not recorded /// 0046872 // metal ion binding // inferred from electronic annotat

n heterodimerization activity // not recorded /// 0046982 // protein heterodimerization activity // inferred fron
ivity // traceable author statement /// 0005515 // protein binding // inferred from physical interaction /// 0005
9825 // oxygen binding // inferred from electronic annotation /// 0020037 // heme binding // not recorded /// (
quence-specific DNA binding transcription factor activity // inferred from electronic annotation /// 0005515 // p
al ion binding // inferred from electronic annotation
in binding // inferred from electronic annotation /// 0008270 // zinc ion binding // inferred from electronic ann
thymidylate kinase activity // not recorded /// 0004798 // thymidylate kinase activity // inferred from electroni
beta-2 adrenergic receptor binding // inferred from electronic annotation
beta-2 adrenergic receptor binding // inferred from electronic annotation
lectronic annotation /// 0043565 // sequence-specific DNA binding // inferred from electronic annotation /// 0(
) // SUMO ligase activity // inferred from direct assay /// 0031625 // ubiquitin protein ligase binding // inferred

ıicrotubule minus-end binding // not recorded
in T binding // inferred from electronic annotation
lectronic annotation /// 0010843 // promoter binding // inferred from direct assay /// 0042803 // protein homo

ing // inferred from electronic annotation /// 0017111 // nucleoside-triphosphatase activity // inferred from ele
growth factor binding // inferred from electronic annotation
erred from electronic annotation /// 0016740 // transferase activity // inferred from electronic annotation
tion /// 0005515 // protein binding // inferred from electronic annotation

d from electronic annotation /// 0005737 // cytoplasm // inferred from sequence or structural similarity
organizing center // inferred from electronic annotation /// 0005819 // spindle // inferred from electronic anno
erred from electronic annotation /// 0016740 // transferase activity // inferred from electronic annotation

nRNA 3'-UTR binding // inferred from sequence or structural similarity /// 0005515 // protein binding // inferrec
NA binding transcription factor activity // inferred from direct assay /// 0003700 // sequence-specific DNA bind
n electronic annotation /// 0008270 // zinc ion binding // inferred from electronic annotation /// 0046872 // me

nding // inferred from direct assay /// 0003700 // sequence-specific DNA binding transcription factor activity //

ıctural constituent of ribosome // inferred from electronic annotation /// 0005515 // protein binding // inferred
ytoplasm // not recorded

' ubiquitin-protein ligase activity // inferred from sequence or structural similarity /// 0005488 // binding // infe
4310 // farnesyl-diphosphate farnesyltransferase activity // inferred from electronic annotation /// 0005515 // p
ivity // inferred from electronic annotation /// 0008802 // betaine-aldehyde dehydrogenase activity // inferred

' // DNA binding // inferred from electronic annotation /// 0003916 // DNA topoisomerase activity // inferred fr
ATP binding // inferred from electronic annotation /// 0051082 // unfolded protein binding // inferred from elec

ɪnic annotation /// 0030528 // transcription regulator activity // inferred from electronic annotation
ubiquitin thiolesterase activity // inferred from electronic annotation /// 0004221 // ubiquitin thiolesterase activ

NA binding transcription factor activity // inferred from direct assay /// 0005515 // protein binding // inferred f
672 // protein kinase activity // inferred from electronic annotation /// 0004713 // protein tyrosine kinase activ
ic membrane-bounded vesicle // inferred from electronic annotation /// 0031410 // cytoplasmic vesicle // infer

n-protein ligase activity // inferred from electronic annotation /// 0004842 // ubiquitin-protein ligase activity //

ein kinase activity // inferred from electronic annotation /// 0004674 // protein serine/threonine kinase activity
omatin binding // inferred from electronic annotation /// 0003700 // sequence-specific DNA binding transcriptic
ded /// 0004014 // adenosylmethionine decarboxylase activity // inferred from electronic annotation /// 00055

n heterodimerization activity // not recorded /// 0046982 // protein heterodimerization activity // inferred from
-N-acetylglucosamine-dolichyl-phosphate N-acetylglucosaminephosphotransferase activity // not recorded ///
/ 0004623 // phospholipase A2 activity // inferred from electronic annotation /// 0004623 // phospholipase A2

tion

/// 0008233 // peptidase activity // inferred from electronic annotation /// 0008237 // metallopeptidase activity
cid receptor activity // not recorded /// 0001619 // lysosphingolipid and lysophosphatidic acid receptor activity
binding // inferred from electronic annotation /// 0070990 // snRNP binding // inferred from electronic annotat
ctronic annotation /// 0004559 // alpha-mannosidase activity // inferred from electronic annotation /// 00045
g // inferred from electronic annotation
// heat shock protein binding // inferred from electronic annotation /// 0031593 // polyubiquitin binding // infe
/// 0004842 // ubiquitin-protein ligase activity // inferred from sequence or structural similarity /// 0005515 //

930 // G-protein coupled receptor activity // inferred from electronic annotation /// 0005515 // protein binding
/ transcription coactivator activity // inferred from electronic annotation /// 0005515 // protein binding // infer
tatement /// 0005515 // protein binding // inferred from physical interaction /// 0030971 // receptor tyrosine k

ed from electronic annotation /// 0003700 // sequence-specific DNA binding transcription factor activity // infer
515 // protein binding // inferred from physical interaction /// 0005515 // protein binding // inferred from elec
ansferase activity // inferred from electronic annotation /// 0004812 // aminoacyl-tRNA ligase activity // inferre
ce or structural similarity /// 0008233 // peptidase activity // inferred from electronic annotation /// 0008234 //
// DNA binding // inferred from electronic annotation /// 0003677 // DNA binding // inferred from sequence o
tin binding // inferred from electronic annotation /// 0005515 // protein binding // inferred from electronic ann
heparin binding // inferred from electronic annotation /// 0008201 // heparin binding // inferred from sequenc

assay /// 0003714 // transcription corepressor activity // inferred from electronic annotation /// 0004872 // rec
nferred from physical interaction /// 0030528 // transcription regulator activity // inferred from electronic anno

// inferred from electronic annotation /// 0005515 // protein binding // inferred from physical interaction /// 00
0005515 // protein binding // inferred from physical interaction /// 0005515 // protein binding // inferred from

ded /// 0008028 // monocarboxylic acid transmembrane transporter activity // inferred from electronic annotat

otropic glutamate receptor activity // inferred from electronic annotation /// 0004972 // N-methyl-D-aspartate

metal ion binding // inferred from electronic annotation
protein serine/threonine kinase activity // inferred from electronic annotation /// 0005524 // ATP binding // inf
nferred from electronic annotation /// 0008083 // growth factor activity // inferred from electronic annotation /
inferred from electronic annotation /// 0016021 // integral to membrane // inferred from electronic annotation

74 // protein serine/threonine kinase activity // inferred from electronic annotation /// 0004703 // G-protein co
ion /// 0005488 // binding // inferred from electronic annotation /// 0005509 // calcium ion binding // inferred
calmodulin binding // inferred from direct assay /// 0005516 // calmodulin binding // inferred from electronic a
l-O-glycosyl-glycoprotein beta-1,6-N-acetylglucosaminyltransferase activity // inferred from electronic annotatio
ivity // inferred from electronic annotation /// 0003779 // actin binding // non-traceable author statement /// 0
annotation /// 0005515 // protein binding // inferred from physical interaction /// 0016853 // isomerase activit

13 // protein tyrosine kinase activity // inferred from electronic annotation /// 0004714 // transmembrane rece

cal protein binding // not recorded
// RNA binding // inferred from electronic annotation
rononglucosaminidase activity // inferred from electronic annotation /// 0008080 // N-acetyltransferase activity
74 // protein serine/threonine kinase activity // inferred from electronic annotation /// 0004697 // protein kina
it phenotype /// 0004060 // arylamine N-acetyltransferase activity // not recorded /// 0004060 // arylamine N-a

16740 // transferase activity // inferred from electronic annotation /// 0016757 // transferase activity, transferr
odulin binding // inferred from electronic annotation /// 0042608 // T cell receptor binding // not recorded ///

ferred from electronic annotation
eptidase activity // inferred from electronic annotation /// 0005515 // protein binding // inferred from physical
388 // transmembrane receptor activity // inferred from electronic annotation /// 0004930 // G-protein couplec

erred from direct assay /// 0004722 // protein serine/threonine phosphatase activity // inferred from mutant ph

74 // protein serine/threonine kinase activity // inferred from electronic annotation /// 0004872 // receptor act
0005035 // death receptor activity // inferred from direct assay /// 0005515 // protein binding // inferred from
SH3 domain binding // inferred from direct assay /// 0046872 // metal ion binding // inferred from electronic a
930 // G-protein coupled receptor activity // inferred from electronic annotation /// 0004995 // tachykinin recep

lectronic annotation /// 0030528 // transcription regulator activity // inferred from electronic annotation /// 0C
930 // G-protein coupled receptor activity // inferred from electronic annotation /// 0008527 // taste receptor a
c ion binding // inferred from electronic annotation /// 0046872 // metal ion binding // inferred from electronic
31 // extracellular ATP-gated cation channel activity // traceable author statement /// 0004931 // extracellular A

inferred from direct assay /// 0003700 // sequence-specific DNA binding transcription factor activity // inferred
3 // RNA binding // inferred from electronic annotation /// 0004672 // protein kinase activity // traceable author
ed from electronic annotation /// 0005515 // protein binding // inferred from electronic annotation /// 0032403
/ exonuclease activity // inferred from electronic annotation /// 0004527 // exonuclease activity // inferred from
'13 // protein tyrosine kinase activity // not recorded /// 0004713 // protein tyrosine kinase activity // inferred f

se activity // inferred from electronic annotation /// 0046872 // metal ion binding // inferred from electronic an
ion
0008270 // zinc ion binding // inferred from electronic annotation /// 0046872 // metal ion binding // inferred
rolase activity // inferred from electronic annotation /// 0016820 // hydrolase activity, acting on acid anhydrides
n /// 0008233 // peptidase activity // inferred from electronic annotation /// 0008234 // cysteine-type peptidas
otation /// 0042803 // protein homodimerization activity // inferred from physical interaction
15288 // porin activity // inferred from electronic annotation
inding // not recorded

raction /// 0051082 // unfolded protein binding // inferred from direct assay
ling // inferred from electronic annotation
n-protein ligase activity // inferred from electronic annotation /// 0004842 // ubiquitin-protein ligase activity //
tivity // inferred from electronic annotation /// 0004674 // protein serine/threonine kinase activity // inferred f
ed from electronic annotation /// 0016740 // transferase activity // inferred from electronic annotation

sporter activity // inferred from electronic annotation /// 0019903 // protein phosphatase binding // inferred fr
beta-2 adrenergic receptor binding // inferred from electronic annotation
brane // inferred from electronic annotation
Vnt-protein binding // not recorded

tion /// 0016787 // hydrolase activity // inferred from electronic annotation

nding // inferred from electronic annotation
transferase activity, transferring glycosyl groups // inferred from electronic annotation /// 0016763 // transfera

transferase activity, transferring glycosyl groups // inferred from electronic annotation /// 0016763 // transfera
ı linear amides // inferred from electronic annotation /// 0017040 // ceramidase activity // inferred from electro
5740 // transferase activity // inferred from electronic annotation /// 0047184 // 1-acylglycerophosphocholine (

ding // inferred from physical interaction /// 0005515 // protein binding // inferred from electronic annotation

) // transferase activity // inferred from electronic annotation /// 0046872 // metal ion binding // inferred from
similarity /// 0005096 // GTPase activator activity // traceable author statement

ınotation /// 0003700 // sequence-specific DNA binding transcription factor activity // inferred from direct assay

ı receptor binding // inferred from physical interaction
NA binding transcription factor activity // inferred from electronic annotation /// 0005515 // protein binding //
tivity // inferred from sequence or structural similarity /// 0003676 // nucleic acid binding // inferred from elect
protein binding // inferred from physical interaction /// 0005515 // protein binding // inferred from electronic a
PH) activity // inferred from electronic annotation /// 0016491 // oxidoreductase activity // inferred from electr
' protein binding // inferred from physical interaction
ı /// 0005515 // protein binding // inferred from electronic annotation

0051721 // protein phosphatase 2A binding // inferred from sequence or structural similarity

' inferred from electronic annotation /// 0003730 // mRNA 3'-UTR binding // inferred from sequence or structur
ation /// 0005515 // protein binding // inferred from electronic annotation
doreductase activity // inferred from electronic annotation /// 0016641 // oxidoreductase activity, acting on the
hogluconate dehydrogenase (decarboxylating) activity // inferred from electronic annotation /// 0005488 // bin
in 2-beta-N-acetylglucosaminyltransferase activity // inferred from mutant phenotype /// 0008455 // alpha-1,6-
ıg // inferred from electronic annotation /// 0008270 // zinc ion binding // inferred from electronic annotation /

; // RNA binding // inferred from electronic annotation /// 0004386 // helicase activity // inferred from electron

NA binding transcription factor activity // inferred from direct assay /// 0003700 // sequence-specific DNA bind
ınscription coactivator activity // inferred from electronic annotation /// 0005515 // protein binding // inferred

eolus // inferred from electronic annotation /// 0005737 // cytoplasm // inferred from electronic annotation ///

; // RNA binding // inferred from electronic annotation /// 0003727 // single-stranded RNA binding // inferred fr
// inferred from electronic annotation
ıferred from electronic annotation /// 0016021 // integral to membrane // inferred from electronic annotation
/ 0008237 // metallopeptidase activity // inferred from direct assay /// 0008237 // metallopeptidase activity //

me binding // inferred from electronic annotation

otein binding // inferred from electronic annotation /// 0005525 // GTP binding // not recorded /// 0005525 // G

ıg // inferred from electronic annotation /// 0005515 // protein binding // inferred from physical interaction ///
'13 // protein tyrosine kinase activity // not recorded /// 0004713 // protein tyrosine kinase activity // inferred f

al ion binding // inferred from electronic annotation
.012 // phospholipid-translocating ATPase activity // inferred from electronic annotation /// 0005515 // protein

)4629 // phospholipase C activity // inferred from electronic annotation /// 0004871 // signal transducer activity
'13 // protein tyrosine kinase activity // not recorded /// 0004713 // protein tyrosine kinase activity // inferred f
0005515 // protein binding // inferred from electronic annotation /// 0008233 // peptidase activity // inferred
ı /// 0046872 // metal ion binding // inferred from electronic annotation

iTP binding // inferred from electronic annotation

ıse activity // not recorded /// 0004070 // aspartate carbamoyltransferase activity // inferred from electronic ar
notation /// 0016740 // transferase activity // inferred from electronic annotation /// 0016757 // transferase ac

arity /// 0005515 // protein binding // inferred from electronic annotation /// 0008094 // DNA-dependent ATPa

ıic annotation /// 0005488 // binding // inferred from electronic annotation /// 0005515 // protein binding // in

ıferred from electronic annotation /// 0046872 // metal ion binding // inferred from electronic annotation

1-phosphatidylinositol 4-kinase activity // inferred from electronic annotation /// 0005524 // ATP binding // inf

lectronic annotation /// 0005515 // protein binding // inferred from physical interaction /// 0008270 // zinc ion
/ DNA binding // inferred from electronic annotation /// 0003700 // sequence-specific DNA binding transcriptio
ed /// 0004437 // inositol or phosphatidylinositol phosphatase activity // inferred from electronic annotation //

inferred from electronic annotation
brane // inferred from electronic annotation
activity // inferred from electronic annotation
m electronic annotation /// 0004872 // receptor activity // inferred from electronic annotation /// 0005515 // p

/ 0008234 // cysteine-type peptidase activity // inferred from electronic annotation /// 0008270 // zinc ion bind

n-protein ligase activity // inferred from electronic annotation /// 0004842 // ubiquitin-protein ligase activity //
; // protein binding // inferred from electronic annotation
iTP binding // inferred from electronic annotation
red from electronic annotation
' protein binding // inferred from physical interaction /// 0005515 // protein binding // inferred from electronic
tion /// 0008233 // peptidase activity // inferred from sequence or structural similarity /// 0016787 // hydrolase
ige-gated ion channel activity // inferred from electronic annotation /// 0005247 // voltage-gated chloride chan

74 // protein serine/threonine kinase activity // inferred from electronic annotation /// 0005524 // ATP binding
74 // protein serine/threonine kinase activity // inferred from electronic annotation /// 0004697 // protein kina
/ 0008234 // cysteine-type peptidase activity // inferred from electronic annotation /// 0016787 // hydrolase ac
n C-terminus binding // inferred from electronic annotation /// 0035091 // phosphoinositide binding // inferred
0005515 // protein binding // inferred from electronic annotation /// 0008233 // peptidase activity // inferred

nic annotation /// 0005178 // integrin binding // inferred from electronic annotation /// 0005506 // iron ion bin
) // SUMO ligase activity // inferred from direct assay /// 0031625 // ubiquitin protein ligase binding // inferred
rred from electronic annotation /// 0016740 // transferase activity // inferred from electronic annotation

930 // G-protein coupled receptor activity // not recorded /// 0004930 // G-protein coupled receptor activity //

nic annotation /// 0004864 // phosphoprotein phosphatase inhibitor activity // inferred from electronic annotat
478 // methionine adenosyltransferase activity // inferred from direct assay /// 0004478 // methionine adenos
action /// 0051082 // unfolded protein binding // inferred from direct assay

phosphodiesterase I activity // inferred from electronic annotation /// 0004551 // nucleotide diphosphatase ac

// 0016491 // oxidoreductase activity // inferred from direct assay /// 0016491 // oxidoreductase activity // inf
NA binding transcription factor activity // not recorded /// 0003700 // sequence-specific DNA binding transcript
activity // inferred from electronic annotation /// 0016702 // oxidoreductase activity, acting on single donors w
13 // protein tyrosine kinase activity // inferred from electronic annotation /// 0004715 // non-membrane span
omain binding // not recorded /// 0030165 // PDZ domain binding // inferred from electronic annotation /// 003
binding // inferred from electronic annotation /// 0005515 // protein binding // inferred from physical interac
5091 // phosphoinositide binding // inferred from electronic annotation

m electronic annotation /// 0005886 // plasma membrane // inferred from electronic annotation /// 0009986 /
04758 // serine C-palmitoyltransferase activity // inferred from electronic annotation /// 0008415 // acyltransfe
long-chain fatty acid-CoA ligase activity // not recorded /// 0004467 // long-chain fatty acid-CoA ligase activity /
388 // transmembrane receptor activity // inferred from sequence or structural similarity /// 0004930 // G-prot

topoisomerase activity // inferred from electronic annotation /// 0003917 // DNA topoisomerase type I activit

13 // protein tyrosine kinase activity // inferred from electronic annotation /// 0004713 // protein tyrosine kina

d binding // inferred from electronic annotation
ation

notation /// 0005488 // binding // inferred from electronic annotation /// 0008233 // peptidase activity // infer
ding // inferred from electronic annotation /// 0016491 // oxidoreductase activity // inferred from electronic ar
g // inferred from electronic annotation /// 0005518 // collagen binding // not recorded /// 0008022 // protein

2277 // peptide binding // not recorded /// 0042277 // peptide binding // inferred from electronic annotation
charide binding // inferred from direct assay /// 0001614 // purinergic nucleotide receptor activity // not recor
ecific DNA binding transcription factor activity // traceable author statement /// 0003700 // sequence-specific D

mbrane // inferred from electronic annotation
ferred from electronic annotation /// 0004872 // receptor activity // inferred from electronic annotation /// 00(
binding // inferred from electronic annotation
0019239 // deaminase activity // inferred from electronic annotation /// 0046872 // metal ion binding // inferr

// RNA binding // inferred from electronic annotation

16853 // isomerase activity // inferred from electronic annotation

tronic annotation /// 0008415 // acyltransferase activity // inferred from electronic annotation /// 0016740 // t

long-chain fatty acid-CoA ligase activity // not recorded /// 0004467 // long-chain fatty acid-CoA ligase activity /
mbrane // inferred from electronic annotation

brane // inferred from electronic annotation /// 0016020 // membrane // inferred from electronic annotation

rded /// 0003730 // mRNA 3'-UTR binding // inferred from electronic annotation

nding // inferred from electronic annotation
NA binding transcription factor activity // inferred from electronic annotation /// 0005515 // protein binding //

ion binding // inferred from electronic annotation
tronic annotation /// 0005515 // protein binding // inferred from physical interaction /// 0005515 // protein bi
n electronic annotation
oter binding // inferred from electronic annotation /// 0042800 // histone methyltransferase activity (H3-K4 spe
binding // inferred from electronic annotation /// 0008198 // ferrous iron binding // inferred from sequence or
d binding // inferred from electronic annotation

// inferred from electronic annotation /// 0003700 // sequence-specific DNA binding transcription factor activit

on /// 0004722 // protein serine/threonine phosphatase activity // inferred from electronic annotation /// 0004
ic annotation /// 0016564 // transcription repressor activity // inferred from electronic annotation /// 0017069
inferred from electronic annotation /// 0044421 // extracellular region part // inferred from electronic annotati

lectronic annotation /// 0043565 // sequence-specific DNA binding // inferred from electronic annotation

/// 0005515 // protein binding // inferred from electronic annotation /// 0008233 // peptidase activity // inferr
13 // protein tyrosine kinase activity // inferred from electronic annotation /// 0004714 // transmembrane rece
13 // protein tyrosine kinase activity // inferred from electronic annotation /// 0004714 // transmembrane rece
// inferred from electronic annotation /// 0005515 // protein binding // inferred from electronic annotation //
9 // prenyltransferase activity // inferred from electronic annotation /// 0008495 // protoheme IX farnesyltrans

center // inferred from electronic annotation /// 0005856 // cytoskeleton // inferred from electronic annotatior
ic annotation /// 0005488 // binding // inferred from electronic annotation /// 0005515 // protein binding // in
rotein binding // inferred from physical interaction /// 0005525 // GTP binding // inferred from electronic anno
red from electronic annotation /// 0005096 // GTPase activator activity // inferred from electronic annotation /
tivity // inferred from electronic annotation /// 0004674 // protein serine/threonine kinase activity // not recor

n electronic annotation /// 0016021 // integral to membrane // inferred from sequence or structural similarity /
olase activity // inferred from electronic annotation /// 0017110 // nucleoside-diphosphatase activity // inferre

/ inferred from electronic annotation

nembrane // inferred from direct assay

ation /// 0004791 // thioredoxin-disulfide reductase activity // inferred from sequence or structural similarity //

n /// 0016787 // hydrolase activity // inferred from electronic annotation /// 0016798 // hydrolase activity, acti

TP binding // inferred from electronic annotation /// 0051082 // unfolded protein binding // inferred from elec

al ion binding // inferred from electronic annotation

// inferred from sequence or structural similarity /// 0003677 // DNA binding // inferred from electronic annot

or statement
/ inferred from electronic annotation /// 0050998 // nitric-oxide synthase binding // not recorded /// 0050998 /
55 // electron carrier activity // inferred from electronic annotation /// 0016491 // oxidoreductase activity // inf
nilarity /// 0005515 // protein binding // inferred from electronic annotation /// 0008897 // holo-[acyl-carrier-p
/ endonuclease activity // inferred from electronic annotation /// 0004522 // pancreatic ribonuclease activity //
organizing center // inferred from electronic annotation /// 0005819 // spindle // inferred from electronic anno

on /// 0005248 // voltage-gated sodium channel activity // not recorded /// 0005248 // voltage-gated sodium c

e // inferred from electronic annotation /// 0016021 // integral to membrane // inferred from electronic annota
onic annotation /// 0004871 // signal transducer activity // inferred from electronic annotation /// 0005509 // c

bospondin receptor activity // inferred from electronic annotation
romatin binding // inferred from electronic annotation /// 0004386 // helicase activity // inferred from electron

se activity // inferred from electronic annotation

al ion binding // inferred from electronic annotation

t /// 0008415 // acyltransferase activity // inferred from electronic annotation /// 0016740 // transferase activit

inding // inferred from sequence or structural similarity /// 0005515 // protein binding // inferred from electron

quence or structural similarity /// 0035267 // NuA4 histone acetyltransferase complex // inferred from sequenc

ce or structural similarity /// 0005515 // protein binding // inferred from electronic annotation /// 0008168 // n

tronic annotation /// 0016740 // transferase activity // inferred from electronic annotation /// 0016757 // trans

ctivator activity // not recorded
GTPase activator activity // inferred from electronic annotation /// 0008270 // zinc ion binding // inferred from
otation /// 0005524 // ATP binding // inferred from electronic annotation /// 0016301 // kinase activity // infer

\TP binding // inferred from electronic annotation /// 0008080 // N-acetyltransferase activity // inferred from el
1
om electronic annotation /// 0016740 // transferase activity // inferred from electronic annotation
om electronic annotation /// 0016740 // transferase activity // inferred from electronic annotation

in C-terminus binding // not recorded /// 0008022 // protein C-terminus binding // inferred from electronic ann

0008514 // organic anion transmembrane transporter activity // inferred from electronic annotation /// 00153[illegible]

selenide, water dikinase activity // inferred from electronic annotation /// 0005524 // ATP binding // inferred fr
ity /// 0003700 // sequence-specific DNA binding transcription factor activity // inferred from sequence or struc

rred from electronic annotation /// 0005515 // protein binding // inferred from electronic annotation /// 0017[illegible]
Rab GTPase binding // inferred from electronic annotation /// 0017137 // Rab GTPase binding // inferred from s

ronic annotation
)1 // oxidoreductase activity // inferred from electronic annotation /// 0046872 // metal ion binding // inferred
nferred from physical interaction /// 0005524 // ATP binding // inferred from direct assay

'/ inferred from electronic annotation

L // oxidoreductase activity // inferred from electronic annotation /// 0042803 // protein homodimerization acti

ation

0003746 // translation elongation factor activity // inferred from electronic annotation /// 0005515 // protein b

23 // Ral GTPase activator activity // inferred from sequence or structural similarity /// 0046982 // protein heter

388 // transmembrane receptor activity // inferred from electronic annotation /// 0004930 // G-protein couplec

NA binding transcription factor activity // inferred from direct assay /// 0003700 // sequence-specific DNA bind
ein binding // inferred from electronic annotation
metal ion binding // inferred from electronic annotation
al ion binding // inferred from electronic annotation
otation /// 0008270 // zinc ion binding // inferred from electronic annotation /// 0016874 // ligase activity // inf

1537 // 2 iron, 2 sulfur cluster binding // inferred from electronic annotation

iption cofactor activity // inferred from sequence or structural similarity /// 0003712 // transcription cofactor a

coactivator activity // inferred from direct assay /// 0003713 // transcription coactivator activity // inferred from
TPase activity // inferred from sequence or structural similarity /// 0005525 // GTP binding // inferred from ele

annotation /// 0005524 // ATP binding // inferred from electronic annotation /// 0008440 // inositol trisphosph
nnotation /// 0005515 // protein binding // inferred from electronic annotation
// inferred from direct assay /// 0005488 // binding // inferred from electronic annotation /// 0005525 // GTP b

42 // ubiquitin-protein ligase activity // not recorded /// 0004842 // ubiquitin-protein ligase activity // inferred f
tion /// 0016831 // carboxy-lyase activity // inferred from electronic annotation
// flavin-containing monooxygenase activity // inferred from electronic annotation /// 0016491 // oxidoreduct
embrane // non-traceable author statement /// 0016021 // integral to membrane // inferred from electronic an

ion /// 0015293 // symporter activity // inferred from electronic annotation /// 0019843 // rRNA binding // infe

// inferred from electronic annotation /// 0005515 // protein binding // inferred from electronic annotation ///
membrane // inferred from electronic annotation /// 0016021 // integral to membrane // inferred from electro

ranscription factor activity // inferred from electronic annotation /// 0003707 // steroid hormone receptor activ
irect assay /// 0003700 // sequence-specific DNA binding transcription factor activity // not recorded /// 00037
13 // protein tyrosine kinase activity // inferred from electronic annotation /// 0004713 // protein tyrosine kina

protein binding // inferred from physical interaction /// 0005515 // protein binding // inferred from electronic

tivity // inferred from electronic annotation /// 0004674 // protein serine/threonine kinase activity // inferred f
15 // acyltransferase activity // inferred from electronic annotation /// 0016740 // transferase activity // inferre
// peptidase activity // inferred from electronic annotation /// 0008237 // metallopeptidase activity // inferred
from electronic annotation /// 0008270 // zinc ion binding // inferred from electronic annotation /// 0046872 /

'// 0005515 // protein binding // inferred from physical interaction /// 0005515 // protein binding // inferred fro

on /// 0005248 // voltage-gated sodium channel activity // inferred from electronic annotation /// 0005272 // s
on /// 0005248 // voltage-gated sodium channel activity // inferred from electronic annotation /// 0005272 // s

romatin binding // inferred from electronic annotation /// 0016564 // transcription repressor activity // inferrec
524 // ATP binding // inferred from electronic annotation /// 0016301 // kinase activity // inferred from electror
otation /// 0042803 // protein homodimerization activity // inferred from electronic annotation /// 0051082 //

on /// 0005267 // potassium channel activity // inferred from electronic annotation /// 0005509 // calcium ion l
n // inferred from mutant phenotype /// 0031016 // pancreas development // inferred from mutant phenotype
binding // inferred from electronic annotation

672 // protein kinase activity // inferred from direct assay /// 0004672 // protein kinase activity // inferred fron

// inferred from electronic annotation

RING ubiquitin ligase complex // inferred from sequence or structural similarity

icrotubule motor activity // inferred from electronic annotation /// 0005515 // protein binding // inferred from
gG binding // inferred from direct assay /// 0019864 // IgG binding // inferred from mutant phenotype /// 0019

74 // protein serine/threonine kinase activity // inferred from electronic annotation /// 0004703 // G-protein co

ctronic annotation
ectronic annotation
on /// 0005506 // iron ion binding // inferred from electronic annotation /// 0005515 // protein binding // infer

// inferred from electronic annotation /// 0005515 // protein binding // inferred from electronic annotation ///
metal ion binding // inferred from electronic annotation
irect assay /// 0003700 // sequence-specific DNA binding transcription factor activity // inferred from electronic
rred from electronic annotation /// 0016491 // oxidoreductase activity // inferred from electronic annotation /
rred from electronic annotation /// 0016491 // oxidoreductase activity // inferred from electronic annotation /

tation /// 0016491 // oxidoreductase activity // inferred from electronic annotation /// 0042802 // identical pro

electronic annotation /// 0005509 // calcium ion binding // inferred from electronic annotation /// 0005515 // p
n binding // inferred from physical interaction
lectronic annotation /// 0030528 // transcription regulator activity // inferred from electronic annotation /// 00
/ 0008237 // metallopeptidase activity // inferred from electronic annotation /// 0008270 // zinc ion binding //
// inferred from electronic annotation /// 0005515 // protein binding // inferred from electronic annotation ///

binding // inferred from electronic annotation /// 0008094 // DNA-dependent ATPase activity // inferred from
from electronic annotation /// 0016740 // transferase activity // inferred from electronic annotation
' // DNA binding // inferred from electronic annotation /// 0003682 // chromatin binding // inferred from electr

3677 // DNA binding // inferred from electronic annotation /// 0003702 // RNA polymerase II transcription facto
IA binding // inferred from sequence or structural similarity /// 0003700 // sequence-specific DNA binding trans

/// 0016740 // transferase activity // inferred from electronic annotation /// 0016757 // transferase activity, tra
c ion binding // inferred from electronic annotation /// 0046872 // metal ion binding // inferred from electronic
in binding // inferred from electronic annotation /// 0008270 // zinc ion binding // inferred from electronic ann

// transcription regulator activity // inferred from electronic annotation

steine-type peptidase activity // not recorded /// 0008234 // cysteine-type peptidase activity // inferred from e

662 // CAAX-protein geranylgeranyltransferase activity // not recorded /// 0004662 // CAAX-protein geranylgera

otation /// 0005525 // GTP binding // inferred from electronic annotation /// 0030675 // Rac GTPase activator a

ion
'/ RNA polymerase III transcription factor activity // inferred from electronic annotation /// 0005515 // protein b
eptidase activity // not recorded /// 0004177 // aminopeptidase activity // inferred from electronic annotation ,
m sequence or structural similarity /// 0005515 // protein binding // inferred from electronic annotation /// 000
ı of molecular oxygen, incorporation of two atoms of oxygen // inferred from electronic annotation /// 0046872
5788 // hydrolase activity, acting on ester bonds // inferred from direct assay /// 0018738 // S-formylglutathione
rred from direct assay /// 0046873 // metal ion transmembrane transporter activity // inferred from electronic
zinc ion binding // inferred from electronic annotation /// 0046872 // metal ion binding // inferred from electro

ıyltransferase activity // inferred from direct assay /// 0008375 // acetylglucosaminyltransferase activity // infer
ıe // inferred from electronic annotation /// 0016021 // integral to membrane // inferred from electronic annot
574 // protein serine/threonine kinase activity // inferred from electronic annotation /// 0005515 // protein binc

notation

) // palmitoyltransferase activity // inferred from direct assay /// 0016740 // transferase activity // inferred from
long-chain fatty acid-CoA ligase activity // inferred from direct assay /// 0004467 // long-chain fatty acid-CoA lig
)4357 // glutamate-cysteine ligase activity // inferred from sequence or structural similarity /// 0005515 // prot

'13 // protein tyrosine kinase activity // inferred from electronic annotation /// 0004715 // non-membrane spar
ce or structural similarity /// 0005515 // protein binding // inferred from electronic annotation /// 0008168 // n

transferase activity // inferred from electronic annotation /// 0016740 // transferase activity // inferred from el

zinc ion binding // inferred from electronic annotation

// protein binding // inferred from physical interaction /// 0008792 // arginine decarboxylase activity // inferre

NA binding transcription factor activity // inferred from direct assay /// 0005515 // protein binding // inferred f
/ 0008237 // metallopeptidase activity // inferred from electronic annotation /// 0008270 // zinc ion binding //

onic annotation /// 0016787 // hydrolase activity // inferred from electronic annotation

metal ion binding // inferred from electronic annotation

)5515 // protein binding // inferred from physical interaction
otation /// 0008270 // zinc ion binding // inferred from electronic annotation /// 0016874 // ligase activity // inf
15 // actin filament binding // not recorded
'/// 0008233 // peptidase activity // inferred from electronic annotation /// 0008237 // metallopeptidase activity

) // zinc ion binding // inferred from electronic annotation

inding // inferred from electronic annotation

// catalytic activity // inferred from electronic annotation /// 0004043 // L-aminoadipate-semialdehyde dehydr

0016787 // hydrolase activity // inferred from electronic annotation

ription cofactor activity // inferred from sequence or structural similarity /// 0003712 // transcription cofactor a
nscription factor activity // not recorded /// 0003705 // sequence-specific enhancer binding RNA polymerase II

lectronic annotation /// 0030528 // transcription regulator activity // inferred from electronic annotation /// 00

0016020 // membrane // inferred from electronic annotation /// 0016021 // integral to membrane // inferred fr

' // DNA binding // inferred from electronic annotation /// 0003682 // chromatin binding // inferred from electr

icrotubule motor activity // inferred from electronic annotation /// 0005515 // protein binding // inferred from

'// 0008233 // peptidase activity // inferred from electronic annotation /// 0008237 // metallopeptidase activity

4872 // receptor activity // inferred from electronic annotation /// 0005042 // netrin receptor activity // not rec
// 0005524 // ATP binding // inferred from electronic annotation /// 0008233 // peptidase activity // inferred fr

ity // inferred from direct assay /// 0005515 // protein binding // inferred from physical interaction /// 0008270
zinc ion binding // inferred from electronic annotation /// 0046872 // metal ion binding // inferred from electro
nscription coactivator activity // inferred from electronic annotation /// 0005515 // protein binding // inferred
on /// 0005248 // voltage-gated sodium channel activity // inferred from mutant phenotype /// 0005248 // volt

n /// 0004722 // protein serine/threonine phosphatase activity // inferred from electronic annotation /// 00047

ectronic annotation
etylcholine receptor inhibitor activity // inferred from direct assay

ng // inferred from electronic annotation

annotation /// 0005089 // Rho guanyl-nucleotide exchange factor activity // inferred from electronic annotatio

quence-specific DNA binding transcription factor activity // non-traceable author statement /// 0005515 // prot
metal ion binding // inferred from electronic annotation
inferred from electronic annotation /// 0016021 // integral to membrane // inferred from electronic annotation
ine oxidase activity // inferred from electronic annotation /// 0016491 // oxidoreductase activity // inferred fro

activity // not recorded /// 0001642 // group III metabotropic glutamate receptor activity // traceable author s
ubiquitin-protein ligase activity // inferred from sequence or structural similarity /// 0005488 // binding // infe

ce or structural similarity /// 0005515 // protein binding // inferred from physical interaction /// 0005515 // pro
metal ion binding // inferred from electronic annotation
otation
acid binding // inferred from electronic annotation /// 0003677 // DNA binding // inferred from direct assay //
zinc ion binding // inferred from electronic annotation /// 0046872 // metal ion binding // inferred from electro

teraction /// 0016301 // kinase activity // inferred from electronic annotation /// 0017048 // Rho GTPase bindi
metal ion binding // inferred from electronic annotation

/// 0005515 // protein binding // inferred from physical interaction /// 0005515 // protein binding // inferred fr

binding // inferred from electronic annotation /// 0016491 // oxidoreductase activity // inferred from electron

otation /// 0008233 // peptidase activity // inferred from electronic annotation /// 0008237 // metallopeptidase

/// 0051267 // CP2 mannose-ethanolamine phosphotransferase activity // inferred from electronic annotation

protein binding // inferred from electronic annotation
tronic annotation /// 0016020 // membrane // inferred from electronic annotation /// 0016021 // integral to m

// inferred from sequence or structural similarity /// 0030742 // GTP-dependent protein binding // inferred fro
nuclease activity // inferred from electronic annotation /// 0004519 // endonuclease activity // inferred from el

ctivity // inferred from electronic annotation /// 0004591 // oxoglutarate dehydrogenase (succinyl-transferring)

nferred from physical interaction /// 0016563 // transcription activator activity // inferred from direct assay /// (
930 // G-protein coupled receptor activity // inferred from electronic annotation /// 0004955 // prostaglandin r

tructural molecule activity // inferred from electronic annotation /// 0005200 // structural constituent of cytosk

74 // protein serine/threonine kinase activity // inferred from electronic annotation /// 0005524 // ATP binding

// RNA binding // inferred from electronic annotation /// 0031072 // heat shock protein binding // inferred fro

tin binding // inferred from electronic annotation /// 0005515 // protein binding // inferred from electronic ann

om electronic annotation /// 0005545 // phosphatidylinositol binding // not recorded /// 0008270 // zinc ion bi

NA binding transcription factor activity // inferred from electronic annotation /// 0016564 // transcription repr

ein kinase activity // inferred from electronic annotation /// 0004674 // protein serine/threonine kinase activit

TP binding // inferred from electronic annotation

// RNA binding // inferred from electronic annotation /// 0004386 // helicase activity // inferred from electron
rotein binding // inferred from electronic annotation /// 0005525 // GTP binding // inferred from electronic ann
672 // protein kinase activity // inferred from electronic annotation /// 0004674 // protein serine/threonine ki

270 // zinc ion binding // inferred from electronic annotation /// 0016740 // transferase activity // inferred fron

// transferase activity // inferred from electronic annotation /// 0046872 // metal ion binding // inferred from

// RNA binding // inferred from electronic annotation

otation /// 0017034 // Rap guanyl-nucleotide exchange factor activity // inferred from electronic annotation ///

se activity // inferred from electronic annotation /// 0016740 // transferase activity // inferred from electronic a

c ion binding // inferred from electronic annotation /// 0046872 // metal ion binding // inferred from electronic
otein binding // inferred from electronic annotation
on /// 0004722 // protein serine/threonine phosphatase activity // inferred from direct assay /// 0005515 // pr

red from electronic annotation /// 0005096 // GTPase activator activity // inferred from electronic annotation /
annotation /// 0008233 // peptidase activity // inferred from electronic annotation /// 0016787 // hydrolase act
n-protein ligase activity // inferred from electronic annotation /// 0005515 // protein binding // inferred from p
on binding // inferred from electronic annotation
7154 // semaphorin receptor activity // inferred from physical interaction

nnotation

/ 0005509 // calcium ion binding // inferred from direct assay /// 0005515 // protein binding // inferred from ph

3 // RNA binding // inferred from electronic annotation /// 0003824 // catalytic activity // inferred from electron

metal ion binding // inferred from electronic annotation

nnotation /// 0003682 // chromatin binding // not recorded /// 0003682 // chromatin binding // inferred from e

al ion binding // inferred from electronic annotation

protein homodimerization activity // not recorded /// 0042803 // protein homodimerization activity // inferred f
0 // transferase activity // inferred from electronic annotation /// 0030976 // thiamin pyrophosphate binding //
005515 // protein binding // inferred from electronic annotation /// 0008270 // zinc ion binding // inferred from
0008514 // organic anion transmembrane transporter activity // inferred from electronic annotation /// 00153
// 0005509 // calcium ion binding // traceable author statement /// 0005509 // calcium ion binding // inferred f
d DNA binding // inferred from electronic annotation /// 0003700 // sequence-specific DNA binding transcriptic
// inferred from sequence or structural similarity /// 0003677 // DNA binding // inferred from electronic annot
tein kinase activity // inferred from electronic annotation /// 0004674 // protein serine/threonine kinase activity
on /// 0004722 // protein serine/threonine phosphatase activity // inferred from electronic annotation /// 001
nferred from sequence or structural similarity /// 0019899 // enzyme binding // not recorded /// 0019901 // pr

3 // RNA binding // inferred from electronic annotation /// 0004386 // helicase activity // inferred from electron
otein kinase activity // inferred from electronic annotation /// 0004674 // protein serine/threonine kinase activi

g // inferred from electronic annotation /// 0008168 // methyltransferase activity // inferred from electronic ar

ion binding // inferred from electronic annotation /// 0050733 // RS domain binding // inferred from physical ir
in binding // inferred from electronic annotation /// 0008270 // zinc ion binding // inferred from electronic ann

096 // GTPase activator activity // inferred from electronic annotation /// 0005515 // protein binding // inferre
// RNA binding // inferred from electronic annotation
// diacylglycerol kinase activity // not recorded /// 0004143 // diacylglycerol kinase activity // inferred from elec
ATP binding // inferred from electronic annotation /// 0046872 // metal ion binding // inferred from electronic

0004842 // ubiquitin-protein ligase activity // inferred from electronic annotation /// 0004842 // ubiquitin-prot

g // inferred from electronic annotation /// 0008565 // protein transporter activity // inferred from direct assay
from electronic annotation /// 0004872 // receptor activity // inferred from electronic annotation /// 0005515

from electronic annotation /// 0005515 // protein binding // inferred from electronic annotation /// 0008270 /

236 // serine-type peptidase activity // inferred from electronic annotation /// 0016787 // hydrolase activity // i
ion
tal ion binding // inferred from electronic annotation /// 0046983 // protein dimerization activity // inferred fro

IA binding // not recorded /// 0003690 // double-stranded DNA binding // inferred from electronic annotation /

se activity // inferred from electronic annotation /// 0046872 // metal ion binding // inferred from electronic ar
eoside-triphosphatase activity // inferred from electronic annotation
896 // cytokine receptor activity // inferred from electronic annotation /// 0005515 // protein binding // inferre

red from direct assay /// 0004672 // protein kinase activity // inferred from direct assay /// 0005515 // protein l

quence-specific DNA binding transcription factor activity // inferred from electronic annotation /// 0008270 // zi

016 // adenylate cyclase activity // traceable author statement /// 0004016 // adenylate cyclase activity // infer
tation /// 0008270 // zinc ion binding // inferred from electronic annotation /// 0016874 // ligase activity // inf
metal ion binding // inferred from electronic annotation

' cation channel activity // not recorded /// 0005261 // cation channel activity // inferred from electronic annota
metal ion binding // inferred from electronic annotation
formate-tetrahydrofolate ligase activity // not recorded /// 0004329 // formate-tetrahydrofolate ligase activity ,

nic annotation /// 0004725 // protein tyrosine phosphatase activity // inferred from electronic annotation /// 00
oxide-generating NADPH oxidase activity // inferred from direct assay /// 0016175 // superoxide-generating NAI

otein binding // inferred from electronic annotation /// 0005524 // ATP binding // inferred from electronic anno

)2 // receptor binding // traceable author statement /// 0005109 // frizzled binding // inferred from physical int
ATP binding // inferred from electronic annotation /// 0005524 // ATP binding // inferred from sequence or stru

nel activity // inferred from electronic annotation /// 0005262 // calcium channel activity // inferred from direct

; // RNA binding // inferred from electronic annotation /// 0032947 // protein complex scaffold // inferred from

515 // protein binding // inferred from physical interaction /// 0005515 // protein binding // inferred from elect

3677 // DNA binding // inferred from electronic annotation /// 0008270 // zinc ion binding // inferred from elec

notation /// 0005096 // GTPase activator activity // not recorded /// 0005096 // GTPase activator activity // infe
930 // G-protein coupled receptor activity // inferred from electronic annotation

674 // protein serine/threonine kinase activity // inferred from electronic annotation /// 0004693 // cyclin-deper

on /// 0005515 // protein binding // inferred from electronic annotation /// 0008233 // peptidase activity // inf
vity // inferred from electronic annotation /// 0005515 // protein binding // inferred from physical interaction //
NA binding transcription factor activity // inferred from mutant phenotype /// 0003700 // sequence-specific DN

ronic annotation /// 0003700 // sequence-specific DNA binding transcription factor activity // inferred from dire

ATP binding // inferred from electronic annotation /// 0008026 // ATP-dependent helicase activity // inferred f

' cation channel activity // not recorded /// 0005261 // cation channel activity // inferred from electronic annota

otation /// 0008270 // zinc ion binding // inferred from sequence or structural similarity /// 0046872 // metal ic

g // inferred from electronic annotation /// 0008565 // protein transporter activity // inferred from electronic a

// binding // inferred from electronic annotation /// 0016491 // oxidoreductase activity // inferred from electro

lectronic annotation /// 0005515 // protein binding // inferred from electronic annotation /// 0016563 // transc
rred from physical interaction /// 0016455 // RNA polymerase II transcription mediator activity // inferred from

on /// 0005247 // voltage-gated chloride channel activity // inferred from electronic annotation /// 0005254 //

on /// 0005249 // voltage-gated potassium channel activity // inferred from electronic annotation /// 0005267 /
ı binding // inferred from electronic annotation /// 0016491 // oxidoreductase activity // inferred from electron
ding // inferred from electronic annotation

cyclic-nucleotide phosphodiesterase activity // not recorded /// 0004114 // 3',5'-cyclic-nucleotide phosphodiest

tin binding // inferred from electronic annotation /// 0005515 // protein binding // inferred from electronic ann

ein binding // inferred from electronic annotation /// 0016566 // specific transcriptional repressor activity // infe

or structural similarity /// 0005488 // binding // inferred from electronic annotation /// 0005515 // protein binc
; // inferred from electronic annotation /// 0046872 // metal ion binding // inferred from electronic annotation

electronic annotation /// 0016021 // integral to membrane // inferred from electronic annotation

'/ inferred from electronic annotation

ctronic annotation /// 0000827 // inositol 1,3,4,5,6-pentakisphosphate kinase activity // inferred from sequenc
ranscription factor activity // inferred from electronic annotation /// 0003707 // steroid hormone receptor activ

tase activity // inferred from electronic annotation

similarity /// 0005515 // protein binding // inferred from electronic annotation /// 0005525 // GTP binding // ir
nferred from physical interaction /// 0005515 // protein binding // inferred from electronic annotation /// 0008

dase activity // inferred from electronic annotation /// 0008234 // cysteine-type peptidase activity // inferred fr

/// 0003723 // RNA binding // inferred from electronic annotation /// 0003723 // RNA binding // inferred from

ferred from electronic annotation /// 0016787 // hydrolase activity // inferred from electronic annotation /// 0(
ectronic annotation /// 0046933 // hydrogen ion transporting ATP synthase activity, rotational mechanism // inf
rred from electronic annotation /// 0016717 // oxidoreductase activity, acting on paired donors, with oxidation
m physical interaction /// 0005515 // protein binding // inferred from electronic annotation /// 0008083 // grov
m physical interaction /// 0005515 // protein binding // inferred from electronic annotation /// 0008083 // grov

lrofolate cyclohydrolase activity // inferred from electronic annotation /// 0004488 // methylenetetrahydrofola

9 // receptor signaling complex scaffold activity // inferred from sequence or structural similarity /// 0030528 //
ding // inferred from physical interaction /// 0005515 // protein binding // inferred from electronic annotation

nnotation /// 0017111 // nucleoside-triphosphatase activity // inferred from electronic annotation
ing transcription factor activity // inferred from electronic annotation /// 0016564 // transcription repressor act
tronic annotation

orded

orded /// 0005507 // copper ion binding // inferred from electronic annotation /// 0005515 // protein binding /

ic annotation /// 0003700 // sequence-specific DNA binding transcription factor activity // inferred from electro

ylformylglycinamidine cyclo-ligase activity // inferred from electronic annotation /// 0004644 // phosphoribosyl

om electronic annotation /// 0016491 // oxidoreductase activity // inferred from electronic annotation /// 0016[illegible]

osomal membrane // inferred from electronic annotation /// 0005768 // endosome // not recorded /// 00057[illegible]
osomal membrane // inferred from electronic annotation /// 0005768 // endosome // not recorded /// 00057[illegible]

nce or structural similarity /// 0016887 // ATPase activity // inferred from electronic annotation /// 0017111 //

ed from direct assay /// 0032403 // protein complex binding // not recorded /// 0032403 // protein complex bir

nositide-dependent protein kinase activity // inferred from direct assay /// 0004676 // 3-phosphoinositide-depe

ne transmembrane transporter activity // not recorded /// 0015293 // symporter activity // inferred from electr
otation /// 0015036 // disulfide oxidoreductase activity // inferred from direct assay
activity // inferred from direct assay /// 0005515 // protein binding // inferred from physical interaction /// 00[illegible]
05515 // protein binding // inferred from physical interaction /// 0008201 // heparin binding // not recorded //
ic annotation /// 0005515 // protein binding // inferred from physical interaction /// 0005515 // protein binding
nase activity // inferred from electronic annotation /// 0004713 // protein tyrosine kinase activity // inferred fro
nase activity // inferred from electronic annotation /// 0004713 // protein tyrosine kinase activity // inferred fro
nucleocytoplasmic transporter activity // inferred from direct assay /// 0005515 // protein binding // inferred f
notation /// 0004872 // receptor activity // inferred from electronic annotation /// 0005216 // ion channel activi
50840 // extracellular matrix binding // inferred from direct assay
15 // protein binding // inferred from physical interaction /// 0016829 // lyase activity // inferred from electroni

inferred from electronic annotation /// 0008270 // zinc ion binding // inferred from electronic annotation /// 00
ndent protein kinase activity // inferred from direct assay /// 0004693 // cyclin-dependent protein kinase activit
n electronic annotation /// 0046983 // protein dimerization activity // not recorded /// 0051400 // BH domain b
n electronic annotation /// 0046983 // protein dimerization activity // not recorded /// 0051400 // BH domain b

/// 0008565 // protein transporter activity // inferred from electronic annotation /// 0019904 // protein doma

om electronic annotation /// 0005515 // protein binding // inferred from electronic annotation
ing transcription factor activity // inferred from electronic annotation /// 0003707 // steroid hormone receptor
ing transcription factor activity // inferred from electronic annotation /// 0003707 // steroid hormone receptor
om electronic annotation

tation /// 0008022 // protein C-terminus binding // inferred from electronic annotation /// 0016887 // ATPase a

c annotation /// 0015181 // arginine transmembrane transporter activity // inferred from direct assay /// 00151

ıydrolase activity // inferred from electronic annotation /// 0032403 // protein complex binding // not recordec
' // polysaccharide binding // inferred from electronic annotation /// 0043236 // laminin binding // inferred fror

// inferred from electronic annotation /// 0001965 // G-protein alpha-subunit binding // not recorded /// 0001!
ɛptor protein tyrosine kinase activity // inferred from electronic annotation /// 0004871 // signal transducer act
ption regulator activity // inferred from electronic annotation
electronic annotation /// 0016787 // hydrolase activity // inferred from electronic annotation /// 0016791 // pl
activity // inferred from electronic annotation
784 // superoxide dismutase activity // inferred from electronic annotation /// 0004784 // superoxide dismutas

c annotation /// 0016874 // ligase activity // inferred from electronic annotation /// 0046872 // metal ion bindi
:ion /// 0004771 // sterol esterase activity // not recorded /// 0004771 // sterol esterase activity // inferred fror
; // not recorded /// 0008289 // lipid binding // inferred from electronic annotation /// 0016491 // oxidoreducta

ım electronic annotation /// 0004674 // protein serine/threonine kinase activity // inferred from direct assay //

ation /// 0008237 // metallopeptidase activity // inferred from electronic annotation /// 0008270 // zinc ion bir
ation /// 0008237 // metallopeptidase activity // inferred from electronic annotation /// 0008270 // zinc ion bir
erred from electronic annotation /// 0016491 // oxidoreductase activity // inferred from electronic annotation /

ıic annotation /// 0008083 // growth factor activity // inferred from electronic annotation /// 0034713 // type I

g // inferred from electronic annotation /// 0003774 // motor activity // inferred from electronic annotation ///
n electronic annotation /// 0016563 // transcription activator activity // not recorded /// 0016563 // transcripti

0004672 // protein kinase activity // not recorded /// 0004672 // protein kinase activity // inferred from electrc

// peptidase activity // inferred from electronic annotation /// 0008234 // cysteine-type peptidase activity // in

inase activity // inferred from electronic annotation /// 0005488 // binding // inferred from electronic annotatic

ronic annotation

:er activity // inferred from electronic annotation /// 0015293 // symporter activity // inferred from electronic a

/// 0030133 // transport vesicle // inferred from electronic annotation /// 0042383 // sarcolemma // inferred fr

/ protein binding // inferred from electronic annotation /// 0016301 // kinase activity // inferred from electroni

ferred from electronic annotation /// 0046872 // metal ion binding // inferred from electronic annotation
notation
ring glycosyl groups // inferred from electronic annotation

sterol O-acyltransferase activity // not recorded /// 0004772 // sterol O-acyltransferase activity // inferred from
sterol O-acyltransferase activity // not recorded /// 0004772 // sterol O-acyltransferase activity // inferred from
nembrane // inferred from electronic annotation /// 0016021 // integral to membrane // inferred from electron
erase activity // inferred from electronic annotation /// 0016757 // transferase activity, transferring glycosyl gro

otation /// 0046872 // metal ion binding // inferred from electronic annotation
050291 // sphingosine N-acyltransferase activity // inferred from direct assay

sium channel activity // inferred from electronic annotation /// 0005251 // delayed rectifier potassium channel
n sequence or structural similarity /// 0003824 // catalytic activity // inferred from electronic annotation /// 00(

lase activity // inferred from electronic annotation
drogenase activity // inferred from electronic annotation /// 0005148 // prolactin receptor binding // not record
/ ubiquitin-protein ligase activity // inferred from sequence or structural similarity /// 0005524 // ATP binding //
/ ubiquitin-protein ligase activity // inferred from sequence or structural similarity /// 0005524 // ATP binding //

ay /// 0005515 // protein binding // inferred from physical interaction

564 // transcription repressor activity // inferred from direct assay /// 0016564 // transcription repressor activit
membrane transporter activity // not recorded /// 0005515 // protein binding // inferred from electronic annot
ay
046983 // protein dimerization activity // inferred from electronic annotation

binding // inferred from electronic annotation /// 0016563 // transcription activator activity // inferred from el
recorded /// 0003690 // double-stranded DNA binding // inferred from electronic annotation /// 0003700 // se

ity // inferred from direct assay /// 0019783 // small conjugating protein-specific protease activity // inferred fr

onic annotation /// 0008233 // peptidase activity // inferred from electronic annotation /// 0008237 // metallo
ctronic annotation

/ 0008270 // zinc ion binding // inferred from electronic annotation /// 0016564 // transcription repressor activ

008565 // protein transporter activity // inferred from direct assay /// 0008565 // protein transporter activity /
binding // inferred from physical interaction
rom electronic annotation /// 0004715 // non-membrane spanning protein tyrosine kinase activity // inferred fr

ded /// 0008017 // microtubule binding // not recorded /// 0008198 // ferrous iron binding // inferred from ele

from electronic annotation
eptor activity // not recorded /// 0005000 // vasopressin receptor activity // inferred from electronic annotatio
eptor activity // not recorded /// 0005000 // vasopressin receptor activity // inferred from electronic annotatio

00 // sequence-specific DNA binding transcription factor activity // inferred from electronic annotation /// 0004
00 // sequence-specific DNA binding transcription factor activity // inferred from electronic annotation /// 0004

005856 // cytoskeleton // inferred from electronic annotation /// 0005874 // microtubule // inferred from elec
ctor binding // inferred from electronic annotation /// 0030528 // transcription regulator activity // inferred fro

nferred from electronic annotation /// 0005515 // protein binding // inferred from physical interaction /// 0005
cription regulator activity // inferred from electronic annotation /// 0043565 // sequence-specific DNA binding /
nic annotation

/ 0004672 // protein kinase activity // inferred from sequence or structural similarity /// 0004674 // protein ser
annotation /// 0003705 // sequence-specific enhancer binding RNA polymerase II transcription factor activity //
/// 0030528 // transcription regulator activity // inferred from electronic annotation /// 0043565 // sequence-s
// inferred from electronic annotation

dependent protein kinase activity // not recorded /// 0004683 // calmodulin-dependent protein kinase activity

ectronic annotation /// 0005506 // iron ion binding // inferred from electronic annotation /// 0008391 // arach
ectronic annotation /// 0005506 // iron ion binding // inferred from electronic annotation /// 0008391 // arach

nding // inferred from electronic annotation /// 0016787 // hydrolase activity // inferred from electronic annota

nnotation
ctronic annotation /// 0015280 // ligand-gated sodium channel activity // inferred from direct assay /// 001528
om electronic annotation
/// 0042803 // protein homodimerization activity // inferred from electronic annotation /// 0043498 // cell surf

/// 0004340 // glucokinase activity // inferred from mutant phenotype /// 0004340 // glucokinase activity // no

activity // inferred from electronic annotation /// 0005515 // protein binding // inferred from physical interacti
rred from direct assay /// 0042802 // identical protein binding // inferred from electronic annotation /// 00468
on
rom electronic annotation /// 0005516 // calmodulin binding // inferred from electronic annotation /// 003240
nnotation /// 0050660 // FAD or FADH2 binding // inferred from electronic annotation
nate beta-D-mannosyltransferase activity // not recorded /// 0004582 // dolichyl-phosphate beta-D-mannosyltra

y // inferred from electronic annotation /// 0004930 // G-protein coupled receptor activity // inferred from elec
from electronic annotation /// 0005123 // death receptor binding // inferred from electronic annotation /// 000
nnotation /// 0005515 // protein binding // inferred from physical interaction /// 0008083 // growth factor acti
se activity, acting on acid anhydrides, catalyzing transmembrane movement of substances // inferred from elect

from electronic annotation /// 0042626 // ATPase activity, coupled to transmembrane movement of substances
ed /// 0046872 // metal ion binding // inferred from electronic annotation
otation /// 0008233 // peptidase activity // not recorded /// 0008233 // peptidase activity // inferred from elect

003779 // actin binding // inferred from electronic annotation /// 0005515 // protein binding // inferred from p
ferred from electronic annotation /// 0030528 // transcription regulator activity // traceable author statement

68 // chitinase activity // inferred from direct assay /// 0004568 // chitinase activity // inferred from electronic a
electronic annotation /// 0016881 // acid-amino acid ligase activity // inferred from electronic annotation /// 0

NA binding transcription factor activity // inferred from electronic annotation /// 0003705 // sequence-specific
/// 0003697 // single-stranded DNA binding // inferred from direct assay /// 0003700 // sequence-specific DN

protein binding // inferred from physical interaction /// 0005515 // protein binding // inferred from electronic

d from sequence or structural similarity /// 0016020 // membrane // inferred from electronic annotation /// 001

c annotation /// 0046872 // metal ion binding // inferred from electronic annotation /// 0047498 // calcium-dep
c annotation /// 0046872 // metal ion binding // inferred from electronic annotation

fertilization // inferred from mutant phenotype /// 0010569 // regulation of double-strand break repair via hor
ctivity // inferred from electronic annotation /// 0016787 // hydrolase activity // inferred from electronic annot

ptor activity // inferred from electronic annotation /// 0008527 // taste receptor activity // not recorded /// 00(

ed /// 0008013 // beta-catenin binding // inferred from electronic annotation /// 0008022 // protein C-terminu

assay /// 0004386 // helicase activity // inferred from electronic annotation /// 0005515 // protein binding // ir

ding // inferred from physical interaction /// 0008270 // zinc ion binding // inferred from electronic annotation
y /// 0003700 // sequence-specific DNA binding transcription factor activity // inferred from electronic annotati
erred from electronic annotation /// 0030528 // transcription regulator activity // inferred from electronic anno
n electronic annotation

// inferred from electronic annotation /// 0016491 // oxidoreductase activity // inferred from electronic annota
// inferred from electronic annotation /// 0016491 // oxidoreductase activity // inferred from electronic annota

/ // inferred from electronic annotation /// 0005524 // ATP binding // inferred from electronic annotation /// 0(
ctivity // inferred from electronic annotation

annotation /// 0005515 // protein binding // inferred from electronic annotation /// 0016563 // transcription ac
// 0005529 // sugar binding // inferred from electronic annotation
nnotation /// 0005230 // extracellular ligand-gated ion channel activity // inferred from electronic annotation /

ectronic annotation /// 0010843 // promoter binding // inferred from electronic annotation /// 0016563 // tran
ukin-6 receptor activity // inferred from electronic annotation /// 0004921 // interleukin-11 receptor activity //
ctivity // inferred from electronic annotation /// 0016787 // hydrolase activity // inferred from electronic annot
annotation /// 0003730 // mRNA 3'-UTR binding // inferred from electronic annotation
ty // inferred from direct assay /// 0005515 // protein binding // inferred from physical interaction /// 0008270

nic annotation /// 0008559 // xenobiotic-transporting ATPase activity // not recorded /// 0015238 // drug trans
annotation
yclic-AMP phosphodiesterase activity // inferred from sequence or structural similarity /// 0004115 // 3',5'-cycli
nding // inferred from electronic annotation /// 0046872 // metal ion binding // inferred from electronic annota
osine phosphatase activity // inferred from electronic annotation /// 0005515 // protein binding // inferred fron
7 // transferase activity, transferring glycosyl groups // inferred from electronic annotation
nning protein tyrosine kinase activity // inferred from electronic annotation /// 0005515 // protein binding // inf

nding // inferred from electronic annotation /// 0008092 // cytoskeletal protein binding // inferred from electro
nic annotation /// 0016301 // kinase activity // inferred from electronic annotation /// 0016740 // transferase a
inferred from electronic annotation /// 0015226 // carnitine transporter activity // inferred from direct assay //
ase activity // inferred from electronic annotation /// 0016787 // hydrolase activity // inferred from electronic a
089 // Rho guanyl-nucleotide exchange factor activity // inferred from electronic annotation /// 0005515 // pro
0016563 // transcription activator activity // not recorded /// 0016563 // transcription activator activity // inferr

receptor binding // inferred from direct assay /// 0005154 // epidermal growth factor receptor binding // inferr

binding // inferred from electronic annotation /// 0046872 // metal ion binding // traceable author statement //

eceptor activity // inferred from direct assay /// 0004977 // melanocortin receptor activity // inferred from elec

ctronic annotation /// 0005524 // ATP binding // inferred from electronic annotation /// 0016301 // kinase activ
r statement /// 0004672 // protein kinase activity // inferred from electronic annotation /// 0004674 // protein

tation /// 0008083 // growth factor activity // inferred from electronic annotation

ation /// 0043531 // ADP binding // not recorded /// 0050660 // FAD or FADH2 binding // not recorded /// 0051

ptor activity // inferred from electronic annotation /// 0016497 // substance K receptor activity // not recorded
th factor activity // not recorded /// 0008083 // growth factor activity // inferred from electronic annotation ///
rom electronic annotation /// 0005938 // cell cortex // inferred from direct assay

043565 // sequence-specific DNA binding // inferred from electronic annotation

/// 0033961 // cis-stilbene-oxide hydrolase activity // inferred from electronic annotation
uctural similarity /// 0019903 // protein phosphatase binding // inferred from electronic annotation /// 0035091
tivity, acting on glycosyl bonds // inferred from electronic annotation
n

om electronic annotation

nic annotation /// 0016563 // transcription activator activity // inferred from direct assay /// 0043565 // sequen
lase activity // inferred from electronic annotation
c annotation /// 0043023 // ribosomal large subunit binding // inferred from physical interaction
ansferase activity // inferred from electronic annotation /// 0005515 // protein binding // inferred from physical

from electronic annotation /// 0005515 // protein binding // inferred from electronic annotation

annotation /// 0016021 // integral to membrane // inferred from electronic annotation
tation
rred from direct assay /// 0005459 // UDP-galactose transmembrane transporter activity // inferred from mutar
etal ion binding // inferred from electronic annotation

ase activity // inferred from electronic annotation /// 0050660 // FAD or FADH2 binding // inferred from electrc

tivity // inferred from electronic annotation /// 0005515 // protein binding // inferred from physical interactior

rom electronic annotation /// 0004715 // non-membrane spanning protein tyrosine kinase activity // inferred fi

ronic annotation

ing // inferred from electronic annotation /// 0042802 // identical protein binding // inferred from electronic ar

rom direct assay /// 0008289 // lipid binding // inferred from electronic annotation /// 0008289 // lipid binding
ing // inferred from electronic annotation

n
ctronic annotation /// 0016740 // transferase activity // inferred from electronic annotation /// 0016757 // trar
binding // inferred from electronic annotation
erred from electronic annotation /// 0020037 // heme binding // inferred from electronic annotation /// 00468

binding transcription factor activity // inferred from electronic annotation /// 0005515 // protein binding // in
from electronic annotation /// 0016787 // hydrolase activity // inferred from electronic annotation

ted calcium channel activity // inferred from electronic annotation /// 0005262 // calcium channel activity // in

ic annotation /// 0016491 // oxidoreductase activity // inferred from electronic annotation /// 0016702 // oxidc
ing transcription factor activity // inferred from electronic annotation /// 0005515 // protein binding // inferred

electronic annotation /// 0005506 // iron ion binding // inferred from electronic annotation /// 0009055 // elect

04930 // G-protein coupled receptor activity // not recorded /// 0004930 // G-protein coupled receptor activity
d from electronic annotation /// 0004872 // receptor activity // inferred from electronic annotation /// 000493
ctivity // inferred from electronic annotation /// 0004674 // protein serine/threonine kinase activity // inferred

:ronic annotation /// 0016787 // hydrolase activity // inferred from electronic annotation /// 0016791 // phosph

ein binding // inferred from electronic annotation /// 0042802 // identical protein binding // inferred from elect
annotation /// 0016717 // oxidoreductase activity, acting on paired donors, with oxidation of a pair of donors re

nnotation

notation /// 0016020 // membrane // inferred from electronic annotation /// 0016021 // integral to membrane
rom electronic annotation /// 0003723 // RNA binding // inferred from electronic annotation /// 0008270 // zin
: annotation

ral similarity /// 0004221 // ubiquitin thiolesterase activity // inferred from electronic annotation /// 0004221 /,
ral similarity /// 0004221 // ubiquitin thiolesterase activity // inferred from electronic annotation /// 0004221 /,

ferred from electronic annotation /// 0019903 // protein phosphatase binding // inferred from sequence or stru

/ inferred from electronic annotation /// 0005524 // ATP binding // inferred from electronic annotation /// 001(
/// 0043208 // glycosphingolipid binding // inferred from direct assay
binding // inferred from electronic annotation
interaction /// 0005515 // protein binding // inferred from electronic annotation /// 0008270 // zinc ion binding

lectronic annotation /// 0016702 // oxidoreductase activity, acting on single donors with incorporation of mole
onic annotation /// 0004714 // transmembrane receptor protein tyrosine kinase activity // inferred from electr
:tivity // inferred from electronic annotation
/ 0046872 // metal ion binding // inferred from electronic annotation
from electronic annotation /// 0016874 // ligase activity // inferred from electronic annotation /// 0016886 // l

m electronic annotation /// 0051087 // chaperone binding // not recorded /// 0051087 // chaperone binding //
protein binding // inferred from physical interaction /// 0016563 // transcription activator activity // inferred fro
on /// 0047485 // protein N-terminus binding // inferred from electronic annotation

erred from electronic annotation /// 0016740 // transferase activity // inferred from electronic annotation /// 0

m direct assay /// 0004672 // protein kinase activity // inferred from electronic annotation /// 0004674 // prote
cription regulator activity // inferred from electronic annotation /// 0043565 // sequence-specific DNA binding /
cription regulator activity // inferred from electronic annotation /// 0043565 // sequence-specific DNA binding /

otation /// 0016757 // transferase activity, transferring glycosyl groups // inferred from electronic annotation //
protein serine/threonine kinase activity // inferred from electronic annotation /// 0005524 // ATP binding // infe
ase activity // inferred from electronic annotation

57 // asparaginase activity // inferred from electronic annotation /// 0016787 // hydrolase activity // inferred fro
d from electronic annotation /// 0016155 // formyltetrahydrofolate dehydrogenase activity // inferred from ele
n /// 0016020 // membrane // inferred from electronic annotation /// 0016021 // integral to membrane // infer

/ inferred from electronic annotation

ic annotation /// 0005515 // protein binding // inferred from electronic annotation /// 0005524 // ATP binding ,
om physical interaction /// 0046872 // metal ion binding // inferred from electronic annotation
tivity // inferred from electronic annotation /// 0005515 // protein binding // inferred from electronic annotatio

otein kinase binding // not recorded /// 0019904 // protein domain specific binding // not recorded
eotide receptor activity, G-protein coupled // inferred from electronic annotation

/ protein serine/threonine kinase activity // not recorded /// 0004674 // protein serine/threonine kinase activit

ise activity // inferred from sequence or structural similarity /// 0004714 // transmembrane receptor protein tyr

nine kinase activity // inferred from electronic annotation /// 0005515 // protein binding // inferred from electro

/ // inferred from electronic annotation /// 0004714 // transmembrane receptor protein tyrosine kinase activity
nnotation /// 0003681 // bent DNA binding // not recorded /// 0003690 // double-stranded DNA binding // not r

ect assay /// 0003700 // sequence-specific DNA binding transcription factor activity // not recorded /// 000370C

notation /// 0005524 // ATP binding // inferred from electronic annotation /// 0051015 // actin filament binding
00 // sequence-specific DNA binding transcription factor activity // inferred from electronic annotation /// 0003

gen ion transmembrane transporter activity // inferred from electronic annotation /// 0016563 // transcription a

// 0016491 // oxidoreductase activity // inferred from electronic annotation /// 0016702 // oxidoreductase acti

ne/threonine kinase activity // inferred from sequence or structural similarity /// 0005515 // protein binding //
ctronic annotation /// 0030169 // low-density lipoprotein binding // inferred from mutant phenotype
naling protein serine/threonine kinase activity // inferred from electronic annotation /// 0004872 // receptor a
58 // chitinase activity // inferred from direct assay /// 0004568 // chitinase activity // inferred from electronic a
from physical interaction /// 0005515 // protein binding // inferred from physical interaction /// 0005515 // pro
// hydrolase activity // inferred from electronic annotation /// 0016791 // phosphatase activity // inferred from

/// 0035091 // phosphoinositide binding // inferred from electronic annotation /// 0035091 // phosphoinositide

not recorded /// 0005544 // calcium-dependent phospholipid binding // inferred from electronic annotation //
008138 // protein tyrosine/serine/threonine phosphatase activity // inferred from direct assay /// 0008138 // pr
not recorded /// 0016515 // interleukin-13 receptor activity // inferred from electronic annotation
from electronic annotation /// 0051015 // actin filament binding // inferred from sequence or structural similar
tivity // inferred from electronic annotation /// 0004709 // MAP kinase kinase kinase activity // not recorded //

ation /// 0016787 // hydrolase activity // inferred from electronic annotation /// 0035091 // phosphoinositide b
ing transcription factor activity // inferred from electronic annotation /// 0030528 // transcription regulator act
n
inding // inferred from electronic annotation /// 0008327 // methyl-CpG binding // inferred from direct assay //
kinase kinase activity // inferred from electronic annotation /// 0005524 // ATP binding // inferred from electro
activity // inferred from electronic annotation
phatase activity // inferred from electronic annotation /// 0004725 // protein tyrosine phosphatase activity // in

ng on glycosyl bonds // inferred from electronic annotation

ronic annotation /// 0016564 // transcription repressor activity // inferred from electronic annotation /// 0016
ic annotation
electronic annotation /// 0016881 // acid-amino acid ligase activity // inferred from electronic annotation /// 0
red from electronic annotation /// 0005509 // calcium ion binding // non-traceable author statement /// 0005
aceable author statement /// 0005096 // GTPase activator activity // inferred from electronic annotation /// 00
yclic-AMP phosphodiesterase activity // inferred from electronic annotation /// 0004117 // calmodulin-depend
// potassium channel activity // inferred from electronic annotation /// 0047485 // protein N-terminus binding

m physical interaction /// 0005515 // protein binding // inferred from electronic annotation /// 0008083 // grov
tion /// 0016491 // oxidoreductase activity // inferred from electronic annotation /// 0016740 // transferase ac

electronic annotation
notation /// 0016740 // transferase activity // inferred from electronic annotation

:tivity // inferred from electronic annotation /// 0005524 // ATP binding // inferred from electronic annotation /
›nic annotation
›nic annotation

›hysical interaction /// 0005515 // protein binding // inferred from electronic annotation /// 0009881 // photor
c annotation /// 0016874 // ligase activity // inferred from electronic annotation /// 0046872 // metal ion bindi
:alcium ion binding // inferred from electronic annotation /// 0008081 // phosphoric diester hydrolase activity /
:idase activity // inferred from electronic annotation /// 0008237 // metallopeptidase activity // inferred from e
l from direct assay /// 0005515 // protein binding // inferred from physical interaction /// 0005515 // protein bi
annotation /// 0005886 // plasma membrane // inferred from electronic annotation /// 0016020 // membrane
. // cytoskeletal protein binding // traceable author statement /// 0016740 // transferase activity // inferred fro
ing transcription factor activity // inferred from electronic annotation /// 0003707 // steroid hormone receptor
ic annotation /// 0016702 // oxidoreductase activity, acting on single donors with incorporation of molecular o

say /// 0005516 // calmodulin binding // inferred from electronic annotation /// 0046872 // metal ion binding /
rom electronic annotation /// 0008270 // zinc ion binding // not recorded /// 0008270 // zinc ion binding // infe
ınotation

// peptidase activator activity // inferred from direct assay /// 0045340 // mercury ion binding // not recorded
ıotation /// 0005524 // ATP binding // inferred from electronic annotation /// 0051015 // actin filament binding
ation /// 0017111 // nucleoside-triphosphatase activity // inferred from electronic annotation
annotation /// 0016787 // hydrolase activity // inferred from electronic annotation /// 0046872 // metal ion bi
c annotation /// 0019904 // protein domain specific binding // inferred from electronic annotation
:rred from electronic annotation

:ctronic annotation
/ /// 0008565 // protein transporter activity // inferred from electronic annotation
:lectronic annotation

ım // inferred from electronic annotation /// 0005829 // cytosol // inferred from electronic annotation
›m sequence or structural similarity /// 0005515 // protein binding // inferred from physical interaction /// 000
ling // inferred from physical interaction /// 0005515 // protein binding // inferred from electronic annotation /

ı /// 0008134 // transcription factor binding // traceable author statement /// 0042162 // telomeric DNA bindin
electronic annotation /// 0045182 // translation regulator activity // inferred from electronic annotation
/ inferred from electronic annotation /// 0004842 // ubiquitin-protein ligase activity // inferred from sequence

›m electronic annotation /// 0043295 // glutathione binding // inferred from electronic annotation /// 0043295

inferred from electronic annotation /// 0016787 // hydrolase activity // inferred from electronic annotation ///

inferred from electronic annotation /// 0016787 // hydrolase activity // inferred from electronic annotation ///

ınotation /// 0004672 // protein kinase activity // inferred from electronic annotation /// 0004872 // receptor a
dase activity // inferred from electronic annotation /// 0008234 // cysteine-type peptidase activity // inferred fı

binding // inferred from electronic annotation /// 0004629 // phospholipase C activity // not recorded /// 0004

ɔiquitin conjugating enzyme binding // not recorded /// 0043548 // phosphoinositide 3-kinase binding // not rec
ıotation /// 0005516 // calmodulin binding // inferred from electronic annotation /// 0005524 // ATP binding //
nding // inferred from electronic annotation /// 0016740 // transferase activity // inferred from electronic anno

ɛrred from electronic annotation /// 0016740 // transferase activity // inferred from electronic annotation /// 0

; // inferred from electronic annotation /// 0016301 // kinase activity // inferred from electronic annotation ///

)016563 // transcription activator activity // not recorded /// 0016563 // transcription activator activity // inferı
inferred from electronic annotation /// 0005234 // extracellular-glutamate-gated ion channel activity // not rec
ctivity // inferred from electronic annotation /// 0004674 // protein serine/threonine kinase activity // inferred
:ion /// 0008234 // cysteine-type peptidase activity // inferred from electronic annotation /// 0008270 // zinc io

h factor binding // not recorded /// 0048407 // platelet-derived growth factor binding // inferred from electron

rom direct assay

ɔinding // inferred from electronic annotation

ıferred from direct assay /// 0005515 // protein binding // inferred from physical interaction /// 0005515 // pro

om electronic annotation /// 0046872 // metal ion binding // inferred from electronic annotation

phosphatase activity // not recorded /// 0046872 // metal ion binding // inferred from electronic annotation

tivity, transferring glycosyl groups // inferred from electronic annotation /// 0046872 // metal ion binding // inf

iverdin reductase activity // inferred from electronic annotation /// 0005488 // binding // inferred from electron
ic annotation /// 0005524 // ATP binding // inferred from electronic annotation /// 0008026 // ATP-dependent

/// 0005515 // protein binding // inferred from electronic annotation /// 0008139 // nuclear localization seque

/// 0016564 // transcription repressor activity // not recorded /// 0016564 // transcription repressor activity //

red from electronic annotation
ng // inferred from electronic annotation

ity, acting on the CH-CH group of donors // inferred from electronic annotation /// 0016787 // hydrolase activit
t assay /// 0005540 // hyaluronic acid binding // inferred from electronic annotation
/ 0016787 // hydrolase activity // inferred from electronic annotation /// 0016791 // phosphatase activity // in

in binding // inferred from electronic annotation

corded /// 0003924 // GTPase activity // traceable author statement /// 0004871 // signal transducer activity //
electronic annotation /// 0005506 // iron ion binding // inferred from electronic annotation /// 0009055 // elect

/ inferred from electronic annotation /// 0016874 // ligase activity // inferred from electronic annotation /// 00

Ras GTPase binding // inferred from direct assay
annotation

ed from electronic annotation /// 0008083 // growth factor activity // inferred from electronic annotation

ctivity // inferred from electronic annotation /// 0016787 // hydrolase activity // inferred from electronic annot
)43565 // sequence-specific DNA binding // inferred from electronic annotation
notation /// 0005524 // ATP binding // inferred from electronic annotation /// 0005525 // GTP binding // inferr

ase activity // inferred from electronic annotation /// 0016787 // hydrolase activity // inferred from electronic a

red from electronic annotation /// 0008184 // glycogen phosphorylase activity // not recorded /// 0016740 // tr

notation /// 0005525 // GTP binding // inferred from electronic annotation

corded /// 0019899 // enzyme binding // inferred from electronic annotation

annotation /// 0005515 // protein binding // inferred from physical interaction /// 0005515 // protein binding

vity // inferred from electronic annotation /// 0003887 // DNA-directed DNA polymerase activity // traceable au

tion /// 0008234 // cysteine-type peptidase activity // inferred from electronic annotation /// 0008270 // zinc io
tion /// 0008234 // cysteine-type peptidase activity // inferred from electronic annotation /// 0008270 // zinc io

05515 // protein binding // inferred from physical interaction /// 0008201 // heparin binding // not recorded //

otation
otation

notation
on /// 0010843 // promoter binding // inferred from direct assay

on /// 0005540 // hyaluronic acid binding // inferred from electronic annotation

inding // inferred from electronic annotation /// 0046872 // metal ion binding // traceable author statement //
9 specific) // traceable author statement /// 0035064 // methylated histone residue binding // inferred from se

ı electronic annotation /// 0046872 // metal ion binding // inferred from electronic annotation
tation
·ed from electronic annotation /// 0008237 // metallopeptidase activity // inferred from sequence or structural

on
from electronic annotation /// 0042626 // ATPase activity, coupled to transmembrane movement of substances

ınic annotation

ıging ATPase activity // inferred from mutant phenotype /// 0005391 // sodium:potassium-exchanging ATPase a
rom electronic annotation /// 0005515 // protein binding // inferred from physical interaction /// 0042809 // vi

tion /// 0005200 // structural constituent of cytoskeleton // inferred from electronic annotation /// 0005488 //

ɔinding // inferred from electronic annotation

ption activator activity // inferred from direct assay
activity // inferred from electronic annotation /// 0016791 // phosphatase activity // inferred from electronic a
ic annotation /// 0016491 // oxidoreductase activity // inferred from electronic annotation /// 0016702 // oxido

·red from direct assay /// 0005459 // UDP-galactose transmembrane transporter activity // inferred from mutar

ded /// 0008017 // microtubule binding // not recorded /// 0008198 // ferrous iron binding // inferred from ele
/ inferred from electronic annotation /// 0016874 // ligase activity // inferred from electronic annotation /// 00

erred from electronic annotation /// 0016671 // oxidoreductase activity, acting on a sulfur group of donors, dis

c annotation /// 0030374 // ligand-dependent nuclear receptor transcription coactivator activity // inferred fro
otation
c annotation /// 0030374 // ligand-dependent nuclear receptor transcription coactivator activity // inferred fro

/// 0043565 // sequence-specific DNA binding // not recorded /// 0043565 // sequence-specific DNA binding //

/// 0016787 // hydrolase activity // inferred from electronic annotation /// 0046872 // metal ion binding // infe
ic annotation /// 0005524 // ATP binding // inferred from electronic annotation /// 0008026 // ATP-dependent
ase activity, acting on the aldehyde or oxo group of donors, NAD or NADP as acceptor // inferred from direct as
:ion

ı electronic annotation /// 0046983 // protein dimerization activity // not recorded /// 0051400 // BH domain b
515 // protein binding // inferred from electronic annotation /// 0005525 // GTP binding // inferred from electr
0020037 // heme binding // inferred from electronic annotation /// 0031721 // hemoglobin alpha binding // no
protein binding // inferred from physical interaction /// 0016563 // transcription activator activity // inferred fro

otation /// 0008270 // zinc ion binding // inferred from sequence or structural similarity /// 0046872 // metal ic
ic annotation /// 0005524 // ATP binding // inferred from electronic annotation /// 0016301 // kinase activity //

046983 // protein dimerization activity // inferred from electronic annotation
from sequence or structural similarity

odimerization activity // inferred from direct assay

ectronic annotation

tation /// 0005856 // cytoskeleton // inferred from electronic annotation /// 0005874 // microtubule // inferred

d from electronic annotation
ing transcription factor activity // inferred from electronic annotation /// 0005515 // protein binding // inferred
etal ion binding // inferred from electronic annotation

inferred from direct assay /// 0003700 // sequence-specific DNA binding transcription factor activity // inferred

l from electronic annotation /// 0008270 // zinc ion binding // inferred from electronic annotation

rred from electronic annotation /// 0016874 // ligase activity // inferred from electronic annotation /// 001688
protein binding // inferred from electronic annotation /// 0016491 // oxidoreductase activity // inferred from el
from electronic annotation /// 0016491 // oxidoreductase activity // inferred from electronic annotation

om electronic annotation /// 0003917 // DNA topoisomerase type I activity // inferred from electronic annotati
tronic annotation

vity // inferred from sequence or structural similarity /// 0004843 // ubiquitin-specific protease activity // inferr

rom physical interaction /// 0030528 // transcription regulator activity // inferred from electronic annotation //
vity // not recorded /// 0004713 // protein tyrosine kinase activity // inferred from electronic annotation /// 000
red from electronic annotation

inferred from sequence or structural similarity /// 0005515 // protein binding // inferred from physical interact

/ // inferred from electronic annotation /// 0004704 // NF-kappaB-inducing kinase activity // inferred from direc
on factor activity // inferred from electronic annotation /// 0003916 // DNA topoisomerase activity // inferred fr
15 // protein binding // inferred from physical interaction /// 0016829 // lyase activity // inferred from electroni

n electronic annotation /// 0046983 // protein dimerization activity // not recorded /// 0051400 // BH domain b
0003975 // UDP-N-acetylglucosamine-dolichyl-phosphate N-acetylglucosaminephosphotransferase activity // in
activity // inferred from sequence or structural similarity /// 0005509 // calcium ion binding // inferred from se

y // inferred from electronic annotation /// 0008270 // zinc ion binding // inferred from electronic annotation //
// inferred from electronic annotation /// 0001965 // G-protein alpha-subunit binding // not recorded /// 0001
ion
72 // mannosyl-oligosaccharide 1,3-1,6-alpha-mannosidase activity // inferred from electronic annotation /// 00

erred from electronic annotation /// 0070628 // proteasome binding // inferred from electronic annotation
protein binding // inferred from physical interaction /// 0005524 // ATP binding // inferred from electronic anno

// inferred from physical interaction /// 0016493 // C-C chemokine receptor activity // inferred from electronic
rred from physical interaction /// 0005515 // protein binding // inferred from electronic annotation /// 000827
kinase binding // inferred from electronic annotation

rred from electronic annotation /// 0043565 // sequence-specific DNA binding // inferred from electronic annot
tronic annotation /// 0016301 // kinase activity // inferred from electronic annotation
ed from electronic annotation /// 0005524 // ATP binding // inferred from electronic annotation /// 0008415 //
/ cysteine-type peptidase activity // inferred from electronic annotation /// 0016787 // hydrolase activity // infe
r structural similarity /// 0003678 // DNA helicase activity // inferred from electronic annotation /// 0003678 //
notation /// 0005516 // calmodulin binding // inferred from electronic annotation /// 0005524 // ATP binding //
e or structural similarity

eptor activity // inferred from electronic annotation /// 0005102 // receptor binding // inferred from physical ir
tation /// 0046872 // metal ion binding // inferred from electronic annotation

005515 // protein binding // inferred from electronic annotation /// 0008022 // protein C-terminus binding // in
n electronic annotation /// 0008270 // zinc ion binding // inferred from electronic annotation /// 0019901 // pro

ion /// 0008514 // organic anion transmembrane transporter activity // not recorded /// 0015125 // bile acid tr

selective glutamate receptor activity // inferred from genetic interaction /// 0004972 // N-methyl-D-aspartate s

erred from electronic annotation /// 0016301 // kinase activity // inferred from electronic annotation /// 00167
/// 0042803 // protein homodimerization activity // inferred from electronic annotation /// 0043498 // cell surfa
n /// 0030054 // cell junction // inferred from electronic annotation

oupled receptor kinase activity // inferred from electronic annotation /// 0004871 // signal transducer activity /
from electronic annotation /// 0005515 // protein binding // inferred from electronic annotation /// 0005529 /
nnotation /// 0015269 // calcium-activated potassium channel activity // inferred from electronic annotation //
on /// 0008375 // acetylglucosaminyltransferase activity // inferred from electronic annotation /// 0016740 // t
003779 // actin binding // inferred from electronic annotation /// 0005515 // protein binding // inferred from p
y // inferred from electronic annotation

eptor protein tyrosine kinase activity // inferred from electronic annotation /// 0004872 // receptor activity // ir

y // inferred from electronic annotation /// 0008415 // acyltransferase activity // inferred from electronic annot
ase C activity // inferred from electronic annotation /// 0004699 // calcium-independent protein kinase C activit
acetyltransferase activity // inferred from electronic annotation /// 0008415 // acyltransferase activity // inferre

ring glycosyl groups // inferred from electronic annotation /// 0019871 // sodium channel inhibitor activity // nc
0042608 // T cell receptor binding // inferred from electronic annotation /// 0046872 // metal ion binding // inf

interaction /// 0005515 // protein binding // inferred from electronic annotation /// 0008233 // peptidase activ
d receptor activity // inferred from electronic annotation /// 0005515 // protein binding // inferred from physica

henotype /// 0005515 // protein binding // inferred from physical interaction /// 0016787 // hydrolase activity /

tivity // inferred from electronic annotation /// 0005515 // protein binding // inferred from electronic annotatic
physical interaction /// 0005515 // protein binding // inferred from electronic annotation /// 0043121 // neuro
annotation
ptor activity // not recorded /// 0004995 // tachykinin receptor activity // inferred from electronic annotation

)31490 // chromatin DNA binding // inferred from direct assay /// 0043565 // sequence-specific DNA binding //
ıctivity // inferred from electronic annotation /// 0033041 // sweet taste receptor activity // inferred from elect
: annotation
ATP-gated cation channel activity // inferred from electronic annotation /// 0005216 // ion channel activity // in

l from direct assay /// 0003700 // sequence-specific DNA binding transcription factor activity // not recorded ///
r statement /// 0004672 // protein kinase activity // inferred from electronic annotation /// 0004674 // protein
; // protein complex binding // not recorded /// 0032403 // protein complex binding // inferred from electronic
n sequence or structural similarity /// 0016787 // hydrolase activity // inferred from electronic annotation
rom electronic annotation /// 0004714 // transmembrane receptor protein tyrosine kinase activity // traceable

notation

from electronic annotation /// 0051015 // actin filament binding // inferred from sequence or structural similar
;, catalyzing transmembrane movement of substances // inferred from electronic annotation /// 0046933 // hyc
se activity // inferred from electronic annotation /// 0016787 // hydrolase activity // inferred from electronic an

inferred from sequence or structural similarity /// 0005524 // ATP binding // inferred from electronic annotatic
rom electronic annotation /// 0005524 // ATP binding // inferred from electronic annotation /// 0016301 // kin

om electronic annotation

ıse activity, transferring pentosyl groups // inferred from electronic annotation /// 0017061 // S-methyl-5-thioa

ıse activity, transferring pentosyl groups // inferred from electronic annotation /// 0017061 // S-methyl-5-thioa
onic annotation
O-acyltransferase activity // inferred from direct assay /// 0047184 // 1-acylglycerophosphocholine O-acyltransf

/// 0005525 // GTP binding // inferred from direct assay /// 0005525 // GTP binding // inferred from electronic

electronic annotation

y /// 0003700 // sequence-specific DNA binding transcription factor activity // inferred from electronic annotati

inferred from physical interaction /// 0005515 // protein binding // inferred from electronic annotation /// 000
tronic annotation /// 0008270 // zinc ion binding // inferred from electronic annotation /// 0008270 // zinc ion
nnotation /// 0008080 // N-acetyltransferase activity // traceable author statement /// 0008375 // acetylglucos
onic annotation /// 0016616 // oxidoreductase activity, acting on the CH-OH group of donors, NAD or NADP as

al similarity /// 0003824 // catalytic activity // inferred from electronic annotation /// 0003960 // NADPH:quinc

e CH-NH2 group of donors, oxygen as acceptor // inferred from direct assay /// 0016641 // oxidoreductase activ
ding // inferred from electronic annotation /// 0016491 // oxidoreductase activity // inferred from electronic a
-mannosylglycoprotein 2-beta-N-acetylglucosaminyltransferase activity // not recorded /// 0008455 // alpha-1,
// 0046872 // metal ion binding // inferred from electronic annotation

ic annotation /// 0005524 // ATP binding // inferred from electronic annotation /// 0008026 // ATP-dependent

ing transcription factor activity // inferred from electronic annotation /// 0003700 // sequence-specific DNA bin
from electronic annotation /// 0019901 // protein kinase binding // inferred from electronic annotation /// 004

0005819 // spindle // inferred from electronic annotation /// 0005856 // cytoskeleton // inferred from electror

rom direct assay /// 0008266 // poly(U) RNA binding // inferred from direct assay

inferred from electronic annotation /// 0008270 // zinc ion binding // inferred from electronic annotation /// 00

GTP binding // inferred from electronic annotation /// 0019003 // GDP binding // not recorded /// 0019905 // sy

0008270 // zinc ion binding // inferred from electronic annotation /// 0016491 // oxidoreductase activity // infe
from electronic annotation /// 0004713 // protein tyrosine kinase activity // inferred from sequence or structura

binding // inferred from physical interaction /// 0005524 // ATP binding // inferred from electronic annotation

y // not recorded /// 0004871 // signal transducer activity // inferred from electronic annotation /// 0005509 //
from electronic annotation /// 0004714 // transmembrane receptor protein tyrosine kinase activity // not record
from electronic annotation /// 0008237 // metallopeptidase activity // inferred from direct assay /// 0008237 //

notation /// 0004086 // carbamoyl-phosphate synthase activity // inferred from electronic annotation /// 0004
tivity, transferring glycosyl groups // inferred from electronic annotation /// 0046872 // metal ion binding // inf

se activity // inferred from electronic annotation /// 0008094 // DNA-dependent ATPase activity // inferred from

ferred from physical interaction /// 0005524 // ATP binding // inferred from electronic annotation /// 0016301

erred from electronic annotation /// 0016301 // kinase activity // inferred from electronic annotation /// 00167

binding // inferred from electronic annotation /// 0043565 // sequence-specific DNA binding // inferred from e
n factor activity // inferred from electronic annotation /// 0003707 // steroid hormone receptor activity // infer
/ 0004438 // phosphatidylinositol-3-phosphatase activity // inferred from electronic annotation /// 0004438 //

rotein binding // inferred from electronic annotation /// 0016787 // hydrolase activity // inferred from electron

ling // inferred from electronic annotation /// 0016787 // hydrolase activity // inferred from electronic annotati

inferred from sequence or structural similarity /// 0005524 // ATP binding // inferred from electronic annotatic

annotation
activity // inferred from electronic annotation
nel activity // inferred from electronic annotation /// 0005254 // chloride channel activity // not recorded /// 0

; // inferred from electronic annotation /// 0016301 // kinase activity // inferred from electronic annotation ///
ise C activity // not recorded /// 0004697 // protein kinase C activity // inferred from electronic annotation /// C
:tivity // inferred from electronic annotation
l from electronic annotation /// 0050750 // low-density lipoprotein receptor binding // inferred from electronic
from electronic annotation /// 0008237 // metallopeptidase activity // inferred from direct assay /// 0008237 /,

ıding // not recorded /// 0005509 // calcium ion binding // inferred from direct assay /// 0005509 // calcium ior
from sequence or structural similarity

traceable author statement /// 0004930 // G-protein coupled receptor activity // inferred from electronic anno

:ion /// 0008146 // sulfotransferase activity // inferred from direct assay /// 0008146 // sulfotransferase activity
yltransferase activity // inferred from electronic annotation /// 0005515 // protein binding // inferred from phys

:tivity // inferred from electronic annotation /// 0005044 // scavenger receptor activity // inferred from electror

ferred from electronic annotation /// 0016671 // oxidoreductase activity, acting on a sulfur group of donors, disu
tion factor activity // inferred from electronic annotation /// 0003705 // sequence-specific enhancer binding RN
vith incorporation of molecular oxygen, incorporation of two atoms of oxygen // inferred from electronic annota
ıning protein tyrosine kinase activity // inferred from electronic annotation /// 0005515 // protein binding // inf
30306 // ADP-ribosylation factor binding // not recorded /// 0030306 // ADP-ribosylation factor binding // inferr
tion /// 0005515 // protein binding // inferred from electronic annotation /// 0005529 // sugar binding // inferr

/ cell surface // not recorded /// 0016020 // membrane // inferred from electronic annotation /// 0031225 // ar
rase activity // inferred from electronic annotation /// 0016740 // transferase activity // inferred from electroni
/ inferred from electronic annotation /// 0005524 // ATP binding // inferred from electronic annotation /// 001
ein coupled receptor activity // inferred from sequence or structural similarity /// 0004930 // G-protein coupled

y // inferred from electronic annotation /// 0003918 // DNA topoisomerase (ATP-hydrolyzing) activity // inferre

ıse activity // inferred from sequence or structural similarity /// 0004714 // transmembrane receptor protein tyr

red from electronic annotation /// 0008237 // metallopeptidase activity // inferred from sequence or structural
ınotation
C-terminus binding // not recorded /// 0008565 // protein transporter activity // inferred from electronic annot

ded /// 0001614 // purinergic nucleotide receptor activity // inferred from electronic annotation /// 0004872 //
DNA binding transcription factor activity // inferred from electronic annotation /// 0003705 // sequence-specific

)4930 // G-protein coupled receptor activity // not recorded /// 0004930 // G-protein coupled receptor activity

ed from electronic annotation

transferase activity // inferred from electronic annotation

/ inferred from electronic annotation /// 0005515 // protein binding // inferred from electronic annotation /// (

inferred from physical interaction /// 0005515 // protein binding // inferred from electronic annotation /// 000

nding // inferred from electronic annotation /// 0008191 // metalloendopeptidase inhibitor activity // inferred f

cific) // inferred from electronic annotation
structural similarity /// 0010302 // 2-oxoglutarate-dependent dioxygenase activity // inferred from direct assay

ty // inferred from direct assay /// 0005515 // protein binding // inferred from electronic annotation /// 000827

4724 // magnesium-dependent protein serine/threonine phosphatase activity // not recorded /// 0004724 // ma
) // snRNA binding // inferred from electronic annotation
ion

red from electronic annotation /// 0008236 // serine-type peptidase activity // inferred from electronic annotat
eptor protein tyrosine kinase activity // traceable author statement /// 0004714 // transmembrane receptor pro
eptor protein tyrosine kinase activity // traceable author statement /// 0004714 // transmembrane receptor pro
/ 0005544 // calcium-dependent phospholipid binding // not recorded /// 0005544 // calcium-dependent phosp
sferase activity // inferred from electronic annotation /// 0016740 // transferase activity // inferred from electro

ı /// 0080008 // CUL4 RING ubiquitin ligase complex // inferred from electronic annotation /// 0080008 // CUL4
ferred from physical interaction /// 0005515 // protein binding // inferred from electronic annotation /// 00055
tation
'// 0005515 // protein binding // inferred from physical interaction /// 0032794 // GTPase activating protein bin
ded /// 0004674 // protein serine/threonine kinase activity // inferred from electronic annotation /// 0004679 ,

/// 0016021 // integral to membrane // inferred from electronic annotation /// 0031966 // mitochondrial meml
d from direct assay /// 0017111 // nucleoside-triphosphatase activity // inferred from direct assay

'/ 0005515 // protein binding // inferred from physical interaction /// 0009055 // electron carrier activity // infe

ng on glycosyl bonds // inferred from electronic annotation

tronic annotation

ation /// 0003700 // sequence-specific DNA binding transcription factor activity // inferred from direct assay ///

'/ nitric-oxide synthase binding // inferred from electronic annotation
erred from electronic annotation /// 0020037 // heme binding // inferred from electronic annotation /// 00468
rotein] synthase activity // inferred from electronic annotation /// 0008897 // holo-[acyl-carrier-protein] syntha
inferred from electronic annotation /// 0016787 // hydrolase activity // inferred from electronic annotation
tation /// 0005856 // cytoskeleton // inferred from electronic annotation /// 0005874 // microtubule // inferrec

hannel activity // inferred from electronic annotation /// 0005272 // sodium channel activity // inferred from el

tion
:alcium ion binding // inferred from electronic annotation /// 0005515 // protein binding // inferred from physic

ic annotation /// 0005524 // ATP binding // inferred from electronic annotation /// 0008026 // ATP-dependent

ty // inferred from electronic annotation

nic annotation

:e or structural similarity /// 0070776 // MOZ/MORF histone acetyltransferase complex // inferred from sequen

nethyltransferase activity // inferred from electronic annotation /// 0008270 // zinc ion binding // inferred from

sferase activity, transferring glycosyl groups // inferred from electronic annotation

ı electronic annotation /// 0046872 // metal ion binding // inferred from electronic annotation
red from electronic annotation /// 0016740 // transferase activity // inferred from electronic annotation

lectronic annotation /// 0008415 // acyltransferase activity // inferred from electronic annotation /// 0016740 /

ıotation /// 0016208 // AMP binding // not recorded /// 0016208 // AMP binding // inferred from electronic anı

ŀ7 // sodium-independent organic anion transmembrane transporter activity // inferred from electronic annota

om electronic annotation /// 0016301 // kinase activity // inferred from electronic annotation /// 0016740 // tr
:tural similarity /// 0003704 // specific RNA polymerase II transcription factor activity // inferred from sequence

)49 // GTP-Rho binding // inferred from sequence or structural similarity /// 0042802 // identical protein bindin
sequence or structural similarity /// 0030306 // ADP-ribosylation factor binding // inferred from electronic anno

from electronic annotation

ivity // not recorded /// 0042803 // protein homodimerization activity // inferred from electronic annotation //

ɔinding // inferred from electronic annotation /// 0043022 // ribosome binding // inferred from electronic anno

rodimerization activity // inferred from electronic annotation /// 0046982 // protein heterodimerization activity

d receptor activity // inferred from electronic annotation /// 0005515 // protein binding // inferred from physica

ing transcription factor activity // inferred from electronic annotation /// 0005515 // protein binding // inferred

erred from electronic annotation /// 0046872 // metal ion binding // inferred from electronic annotation

ctivity // inferred from electronic annotation /// 0003714 // transcription corepressor activity // inferred from c

ı genetic interaction /// 0003713 // transcription coactivator activity // inferred from electronic annotation /// (
ctronic annotation /// 0005525 // GTP binding // inferred from sequence or structural similarity /// 0019003 //

ate 3-kinase activity // inferred from electronic annotation /// 0016301 // kinase activity // inferred from electr

›inding // inferred from electronic annotation /// 0017048 // Rho GTPase binding // inferred from physical inter

rom electronic annotation /// 0004842 // ubiquitin-protein ligase activity // inferred from sequence or structur

ase activity // inferred from electronic annotation /// 0050660 // FAD or FADH2 binding // inferred from electrc
notation

rred from electronic annotation

0005524 // ATP binding // inferred from electronic annotation /// 0016301 // kinase activity // inferred from el
›nic annotation

vity // inferred from electronic annotation /// 0004872 // receptor activity // inferred from electronic annotatioı
00 // sequence-specific DNA binding transcription factor activity // inferred from electronic annotation /// 0004
ıse activity // inferred from sequence or structural similarity /// 0004714 // transmembrane receptor protein tyı

annotation /// 0030229 // very-low-density lipoprotein receptor activity // inferred from electronic annotation

rom electronic annotation /// 0005524 // ATP binding // inferred from electronic annotation /// 0016301 // kin
d from electronic annotation
l from electronic annotation /// 0008270 // zinc ion binding // inferred from electronic annotation /// 0016787 ,
'/ metal ion binding // inferred from electronic annotation

ɔm electronic annotation /// 0042802 // identical protein binding // inferred from physical interaction /// 0042[illegible]

odium channel activity // inferred from electronic annotation
odium channel activity // inferred from electronic annotation

l from electronic annotation
nic annotation /// 0016740 // transferase activity // inferred from electronic annotation /// 0016773 // phospho
unfolded protein binding // inferred from direct assay /// 0051087 // chaperone binding // inferred from electr

binding // not recorded /// 0005509 // calcium ion binding // inferred from electronic annotation
/// 0048873 // homeostasis of number of cells within a tissue // inferred from mutant phenotype

ı electronic annotation /// 0004674 // protein serine/threonine kinase activity // inferred from electronic annot

physical interaction /// 0005515 // protein binding // inferred from electronic annotation /// 0005524 // ATP bi
864 // IgG binding // inferred from electronic annotation

oupled receptor kinase activity // inferred from electronic annotation /// 0004871 // signal transducer activity /

red from electronic annotation /// 0009055 // electron carrier activity // inferred from electronic annotation //

/ 0008270 // zinc ion binding // inferred from electronic annotation /// 0046872 // metal ion binding // inferred

c annotation /// 0008134 // transcription factor binding // inferred from electronic annotation /// 0019904 // p
// 0046872 // metal ion binding // inferred from electronic annotation /// 0046914 // transition metal ion bindi
// 0046872 // metal ion binding // inferred from electronic annotation /// 0046914 // transition metal ion bindi

›tein binding // not recorded /// 0042802 // identical protein binding // inferred from electronic annotation

›rotein binding // inferred from electronic annotation /// 0008233 // peptidase activity // inferred from electror

›43565 // sequence-specific DNA binding // inferred from electronic annotation
inferred from electronic annotation /// 0016787 // hydrolase activity // inferred from electronic annotation ///
' 0008270 // zinc ion binding // inferred from electronic annotation /// 0046872 // metal ion binding // inferred

electronic annotation

:onic annotation /// 0004386 // helicase activity // inferred from electronic annotation /// 0005524 // ATP bindi

or activity // inferred from sequence or structural similarity /// 0008270 // zinc ion binding // inferred from elec
scription factor activity // inferred from sequence or structural similarity /// 0003700 // sequence-specific DNA l

ansferring glycosyl groups // inferred from electronic annotation /// 0016758 // transferase activity, transferring
: annotation
otation /// 0008270 // zinc ion binding // inferred from sequence or structural similarity /// 0046872 // metal ic

lectronic annotation /// 0016787 // hydrolase activity // inferred from electronic annotation /// 0019784 // NEI

anyltransferase activity // inferred from electronic annotation /// 0005515 // protein binding // inferred from e

ıctivity // inferred from mutant phenotype /// 0030676 // Rac guanyl-nucleotide exchange factor activity // infe

›inding // inferred from electronic annotation
/// 0005515 // protein binding // inferred from physical interaction /// 0008233 // peptidase activity // inferred
)5515 // protein binding // inferred from physical interaction
! // metal ion binding // inferred from electronic annotation /// 0047800 // cysteamine dioxygenase activity // i
e hydrolase activity // inferred from electronic annotation
annotation
›nic annotation

rred from electronic annotation /// 0016740 // transferase activity // inferred from electronic annotation /// 00
ation /// 0016323 // basolateral plasma membrane // inferred from electronic annotation /// 0016324 // apical
ding // inferred from electronic annotation /// 0005524 // ATP binding // inferred from electronic annotation //

n electronic annotation /// 0046872 // metal ion binding // inferred from electronic annotation
;ase activity // not recorded /// 0004467 // long-chain fatty acid-CoA ligase activity // inferred from electronic ar
:ein binding // inferred from electronic annotation /// 0016491 // oxidoreductase activity // inferred from electr

nning protein tyrosine kinase activity // inferred from electronic annotation /// 0005524 // ATP binding // inferre
nethyltransferase activity // inferred from electronic annotation /// 0008270 // zinc ion binding // inferred from

ectronic annotation /// 0018024 // histone-lysine N-methyltransferase activity // inferred from electronic anno

:d from direct assay /// 0042978 // ornithine decarboxylase activator activity // inferred from genetic interactior

rom physical interaction /// 0030528 // transcription regulator activity // inferred from electronic annotation //
inferred from electronic annotation /// 0016787 // hydrolase activity // inferred from electronic annotation ///

erred from electronic annotation /// 0046872 // metal ion binding // inferred from electronic annotation

y // inferred from electronic annotation /// 0008270 // zinc ion binding // inferred from electronic annotation //

ogenase activity // inferred from electronic annotation /// 0005524 // ATP binding // inferred from electronic ar

ctivity // inferred from electronic annotation /// 0003714 // transcription corepressor activity // inferred from c
transcription factor activity // inferred from electronic annotation /// 0004871 // signal transducer activity // in

043565 // sequence-specific DNA binding // inferred from electronic annotation

rom electronic annotation

ronic annotation /// 0004386 // helicase activity // inferred from electronic annotation /// 0005515 // protein bi

electronic annotation /// 0005524 // ATP binding // inferred from electronic annotation

y // inferred from electronic annotation /// 0008270 // zinc ion binding // inferred from electronic annotation //

orded /// 0005042 // netrin receptor activity // inferred from electronic annotation /// 0005515 // protein bind
om electronic annotation /// 0008237 // metallopeptidase activity // inferred from electronic annotation /// 00

) // zinc ion binding // inferred from electronic annotation /// 0046872 // metal ion binding // inferred from elec
onic annotation
from electronic annotation /// 0019901 // protein kinase binding // inferred from electronic annotation /// 004
tage-gated sodium channel activity // not recorded /// 0005248 // voltage-gated sodium channel activity // infer

722 // protein serine/threonine phosphatase activity // inferred from sequence or structural similarity /// 00047

n

ein binding // inferred from electronic annotation /// 0008270 // zinc ion binding // inferred from electronic anr

n
m electronic annotation /// 0050660 // FAD or FADH2 binding // not recorded /// 0051378 // serotonin binding

tatement /// 0004871 // signal transducer activity // inferred from electronic annotation /// 0004872 // recepto
rred from electronic annotation /// 0016874 // ligase activity // inferred from electronic annotation /// 001688

otein binding // inferred from electronic annotation /// 0008022 // protein C-terminus binding // inferred from e

/ 0003677 // DNA binding // inferred from electronic annotation /// 0003677 // DNA binding // inferred from se
onic annotation

ng // not recorded /// 0032947 // protein complex scaffold // not recorded /// 0046872 // metal ion binding //

rom electronic annotation /// 0005516 // calmodulin binding // inferred from electronic annotation /// 003240

ic annotation /// 0016702 // oxidoreductase activity, acting on single donors with incorporation of molecular ox

: activity // inferred from electronic annotation /// 0008270 // zinc ion binding // non-traceable author stateme

embrane // inferred from electronic annotation

m sequence or structural similarity
ectronic annotation /// 0016787 // hydrolase activity // inferred from electronic annotation

) activity // inferred from sequence or structural similarity /// 0016491 // oxidoreductase activity // inferred fro

)016563 // transcription activator activity // not recorded /// 0016563 // transcription activator activity // infer
eceptor activity // inferred from electronic annotation /// 0004958 // prostaglandin F receptor activity // inferre

keleton // not recorded /// 0005525 // GTP binding // inferred from electronic annotation

; // inferred from electronic annotation /// 0016772 // transferase activity, transferring phosphorus-containing

m electronic annotation /// 0051082 // unfolded protein binding // inferred from electronic annotation

ıotation /// 0005516 // calmodulin binding // inferred from electronic annotation /// 0005524 // ATP binding //

nding // inferred from electronic annotation /// 0016740 // transferase activity // inferred from electronic anno

essor activity // inferred from direct assay /// 0030528 // transcription regulator activity // inferred from electro

/ // inferred from electronic annotation /// 0005524 // ATP binding // inferred from electronic annotation /// 00

ic annotation /// 0005524 // ATP binding // inferred from electronic annotation /// 0008026 // ATP-dependent
ıotation
ıase activity // not recorded /// 0004674 // protein serine/threonine kinase activity // inferred from electronic a

ı electronic annotation /// 0018024 // histone-lysine N-methyltransferase activity // inferred from electronic an

electronic annotation

' 0017034 // Rap guanyl-nucleotide exchange factor activity // inferred from sequence or structural similarity //

annotation /// 0016773 // phosphotransferase activity, alcohol group as acceptor // inferred from electronic an

: annotation

otein binding // inferred from physical interaction /// 0016787 // hydrolase activity // inferred from electronic a

'// 0005515 // protein binding // inferred from physical interaction /// 0032794 // GTPase activating protein bin
:ivity // inferred from electronic annotation
hysical interaction /// 0005515 // protein binding // inferred from electronic annotation /// 0005524 // ATP bin

ıysical interaction /// 0005524 // ATP binding // inferred from direct assay /// 0016594 // glycine binding // infe

ıic annotation /// 0005515 // protein binding // inferred from electronic annotation /// 0008168 // methyltransf

ılectronic annotation /// 0003690 // double-stranded DNA binding // not recorded /// 0003690 // double-stranc

from electronic annotation /// 0046982 // protein heterodimerization activity // not recorded /// 0046982 // pr
/ inferred from electronic annotation /// 0046872 // metal ion binding // inferred from electronic annotation
electronic annotation /// 0016874 // ligase activity // inferred from electronic annotation /// 0046872 // metal
47 // sodium-independent organic anion transmembrane transporter activity // inferred from electronic annota
from electronic annotation
ın factor activity // inferred from electronic annotation /// 0016563 // transcription activator activity // inferred
ation /// 0003690 // double-stranded DNA binding // inferred from direct assay /// 0003690 // double-stranded
/ // not recorded /// 0004674 // protein serine/threonine kinase activity // inferred from electronic annotation
5787 // hydrolase activity // inferred from electronic annotation /// 0046872 // metal ion binding // inferred fro
otein kinase binding // not recorded /// 0019904 // protein domain specific binding // not recorded

ic annotation /// 0005524 // ATP binding // inferred from electronic annotation /// 0008026 // ATP-dependent
ty // inferred from direct assay /// 0004674 // protein serine/threonine kinase activity // inferred from electron

ınotation /// 0008270 // zinc ion binding // inferred from electronic annotation /// 0016740 // transferase activ

nteraction
otation /// 0008270 // zinc ion binding // inferred from sequence or structural similarity /// 0046872 // metal ic

d from electronic annotation /// 0046872 // metal ion binding // inferred from electronic annotation

tronic annotation /// 0005509 // calcium ion binding // inferred from electronic annotation /// 0005515 // prot
annotation

tein ligase activity // inferred from sequence or structural similarity /// 0005515 // protein binding // inferred fr

/// 0008565 // protein transporter activity // inferred from electronic annotation
// protein binding // inferred from physical interaction /// 0008270 // zinc ion binding // inferred from electron

/ zinc ion binding // inferred from electronic annotation /// 0046872 // metal ion binding // inferred from electr

inferred from electronic annotation

om electronic annotation

// 0003700 // sequence-specific DNA binding transcription factor activity // inferred from direct assay /// 0003

notation

d from physical interaction /// 0005515 // protein binding // inferred from electronic annotation /// 0016500 //

binding // inferred from physical interaction /// 0005515 // protein binding // inferred from electronic annotatic

inc ion binding // inferred from electronic annotation /// 0016564 // transcription repressor activity // non-trac

red from electronic annotation /// 0005516 // calmodulin binding // inferred from electronic annotation /// 000
erred from electronic annotation /// 0046872 // metal ion binding // inferred from electronic annotation

ation

// inferred from electronic annotation /// 0005488 // binding // inferred from electronic annotation /// 00055[illegible]

)08138 // protein tyrosine/serine/threonine phosphatase activity // inferred from direct assay /// 0008138 // pr
DPH oxidase activity // inferred from mutant phenotype /// 0016175 // superoxide-generating NADPH oxidase a

tation /// 0016887 // ATPase activity // inferred from electronic annotation /// 0017111 // nucleoside-triphospl

eraction /// 0005109 // frizzled binding // inferred from electronic annotation /// 0005110 // frizzled-2 binding ,
ctural similarity /// 0005525 // GTP binding // inferred from electronic annotation /// 0016787 // hydrolase acti

t assay /// 0005262 // calcium channel activity // inferred from mutant phenotype /// 0015267 // channel activi

sequence or structural similarity

ronic annotation

:tronic annotation /// 0046872 // metal ion binding // inferred from electronic annotation

rred from sequence or structural similarity /// 0005096 // GTPase activator activity // inferred from electronic a

ndent protein kinase activity // inferred from electronic annotation /// 0005524 // ATP binding // inferred from

erred from electronic annotation /// 0008237 // metallopeptidase activity // inferred from electronic annotatio
'/ 0005515 // protein binding // inferred from electronic annotation /// 0016209 // antioxidant activity // inferr
JA binding transcription factor activity // inferred from electronic annotation /// 0003706 // ligand-regulated tra

ect assay /// 0003700 // sequence-specific DNA binding transcription factor activity // not recorded /// 000370C

rom electronic annotation /// 0016787 // hydrolase activity // inferred from electronic annotation

ation

ɔn binding // inferred from electronic annotation

nnotation /// 0042393 // histone binding // inferred from direct assay

ɔnic annotation /// 0016616 // oxidoreductase activity, acting on the CH-OH group of donors, NAD or NADP as a

cription activator activity // not recorded
electronic annotation

chloride channel activity // not recorded /// 0005254 // chloride channel activity // inferred from electronic ann

// potassium channel activity // inferred from electronic annotation
ic annotation /// 0016702 // oxidoreductase activity, acting on single donors with incorporation of molecular ox

terase activity // inferred from direct assay /// 0004114 // 3',5'-cyclic-nucleotide phosphodiesterase activity // in

notation /// 0005516 // calmodulin binding // inferred from electronic annotation /// 0005524 // ATP binding //

erred from direct assay /// 0016566 // specific transcriptional repressor activity // inferred from electronic anno

ding // inferred from physical interaction /// 0005515 // protein binding // inferred from electronic annotation /

e or structural similarity /// 0000832 // inositol hexakisphosphate 5-kinase activity // inferred from electronic a
vity // inferred from electronic annotation /// 0004872 // receptor activity // inferred from electronic annotatio

nferred from electronic annotation /// 0005525 // GTP binding // inferred from sequence or structural similarity
134 // transcription factor binding // inferred from physical interaction /// 0008134 // transcription factor bindi

rom electronic annotation /// 0008270 // zinc ion binding // inferred from electronic annotation /// 0016787 //

sequence or structural similarity /// 0005515 // protein binding // inferred from physical interaction /// 00055[illegible]

046872 // metal ion binding // inferred from electronic annotation
ferred from electronic annotation /// 0046961 // proton-transporting ATPase activity, rotational mechanism // i
of a pair of donors resulting in the reduction of molecular oxygen to two molecules of water // inferred from ele
wth factor activity // inferred from electronic annotation /// 0042802 // identical protein binding // inferred fro
wth factor activity // inferred from electronic annotation /// 0042802 // identical protein binding // inferred fro

te dehydrogenase (NADP+) activity // inferred from electronic annotation /// 0005488 // binding // inferred fro

/ transcription regulator activity // inferred from electronic annotation /// 0030528 // transcription regulator ac
/// 0008083 // growth factor activity // inferred from electronic annotation

tivity // inferred from direct assay /// 0043565 // sequence-specific DNA binding // inferred from electronic ann

/ inferred from physical interaction /// 0016491 // oxidoreductase activity // inferred from electronic annotatic

onic annotation /// 0003706 // ligand-regulated transcription factor activity // inferred from electronic annotatic

glycinamide formyltransferase activity // not recorded /// 0004644 // phosphoribosylglycinamide formyltransfe

616 // oxidoreductase activity, acting on the CH-OH group of donors, NAD or NADP as acceptor // inferred from

8 // endosome // inferred from electronic annotation /// 0005770 // late endosome // inferred from direct assa
8 // endosome // inferred from electronic annotation /// 0005770 // late endosome // inferred from direct assa

nucleoside-triphosphatase activity // inferred from electronic annotation /// 0042626 // ATPase activity, couple

nding // inferred from electronic annotation

endent protein kinase activity // not recorded /// 0004676 // 3-phosphoinositide-dependent protein kinase activ

onic annotation /// 0017153 // sodium:dicarboxylate symporter activity // inferred from electronic annotation

30528 // transcription regulator activity // inferred from electronic annotation
/ 0008201 // heparin binding // inferred from electronic annotation /// 0043394 // proteoglycan binding // infer
g // inferred from electronic annotation /// 0005524 // ATP binding // inferred from electronic annotation /// 0
m electronic annotation /// 0004715 // non-membrane spanning protein tyrosine kinase activity // inferred fro
m electronic annotation /// 0004715 // non-membrane spanning protein tyrosine kinase activity // inferred fro
rom physical interaction /// 0005515 // protein binding // inferred from electronic annotation
ty // inferred from electronic annotation /// 0005261 // cation channel activity // not recorded /// 0005262 // c

ic annotation /// 0016831 // carboxy-lyase activity // inferred from electronic annotation

016787 // hydrolase activity // inferred from electronic annotation /// 0046872 // metal ion binding // inferred f
y // not recorded /// 0004693 // cyclin-dependent protein kinase activity // inferred from electronic annotation
binding // not recorded
binding // not recorded

in specific binding // inferred from electronic annotation

activity // inferred from electronic annotation /// 0004872 // receptor activity // inferred from electronic anno
activity // inferred from electronic annotation /// 0004872 // receptor activity // inferred from electronic anno

activity // not recorded /// 0016887 // ATPase activity // inferred from electronic annotation /// 0017111 // nuc

81 // arginine transmembrane transporter activity // not recorded /// 0015181 // arginine transmembrane tran

| /// 0042803 // protein homodimerization activity // inferred from physical interaction /// 0045502 // dynein b
n physical interaction

965 // G-protein alpha-subunit binding // inferred from electronic annotation /// 0004871 // signal transducer a
ivity // inferred from direct assay /// 0004872 // receptor activity // inferred from electronic annotation /// 000

nosphatase activity // inferred from electronic annotation /// 0017017 // MAP kinase tyrosine/serine/threonine

e activity // inferred from sequence or structural similarity /// 0005515 // protein binding // inferred from elect

ng // inferred from electronic annotation
n electronic annotation /// 0004806 // triglyceride lipase activity // not recorded /// 0004806 // triglyceride lipa
ase activity // inferred from electronic annotation /// 0016702 // oxidoreductase activity, acting on single donor

/ 0004674 // protein serine/threonine kinase activity // inferred from electronic annotation /// 0005515 // prot

nding // inferred from electronic annotation /// 0016787 // hydrolase activity // inferred from electronic annota
nding // inferred from electronic annotation /// 0016787 // hydrolase activity // inferred from electronic annota
/// 0050660 // FAD or FADH2 binding // inferred from electronic annotation /// 0050661 // NADP or NADPH bin

transforming growth factor beta receptor binding // inferred from electronic annotation /// 0042802 // identica

0003779 // actin binding // not recorded /// 0003779 // actin binding // inferred from electronic annotation //
on activator activity // inferred from electronic annotation /// 0043565 // sequence-specific DNA binding // infe

onic annotation /// 0004713 // protein tyrosine kinase activity // inferred from direct assay /// 0004713 // prote

ferred from electronic annotation /// 0008242 // omega peptidase activity // inferred from electronic annotatio

on /// 0005515 // protein binding // inferred from physical interaction /// 0005515 // protein binding // inferred

nnotation /// 0015377 // cation:chloride symporter activity // inferred from electronic annotation /// 0051739

rom direct assay /// 0042383 // sarcolemma // inferred from electronic annotation /// 0045202 // synapse // in

ic annotation /// 0016563 // transcription activator activity // inferred from electronic annotation /// 0016564 /

electronic annotation /// 0008415 // acyltransferase activity // inferred from electronic annotation /// 0015485
electronic annotation /// 0008415 // acyltransferase activity // inferred from electronic annotation /// 0015485
ic annotation
ups // inferred from electronic annotation /// 0016758 // transferase activity, transferring hexosyl groups // infe

activity // not recorded /// 0005267 // potassium channel activity // inferred from electronic annotation /// 000
03857 // 3-hydroxyacyl-CoA dehydrogenase activity // inferred from electronic annotation /// 0004303 // estrad

led /// 0005148 // prolactin receptor binding // inferred from electronic annotation /// 0005488 // binding // in
/ inferred from electronic annotation /// 0016874 // ligase activity // inferred from electronic annotation /// 001
/ inferred from electronic annotation /// 0016874 // ligase activity // inferred from electronic annotation /// 001

y // not recorded /// 0016564 // transcription repressor activity // inferred from electronic annotation /// 0030
ation /// 0008504 // monoamine transmembrane transporter activity // not recorded /// 0008513 // secondary

ectronic annotation /// 0016563 // transcription activator activity // inferred from sequence or structural simila
quence-specific DNA binding transcription factor activity // not recorded /// 0003700 // sequence-specific DNA

om direct assay

peptidase activity // inferred from electronic annotation /// 0008270 // zinc ion binding // inferred from electro

vity // inferred from direct assay /// 0046872 // metal ion binding // inferred from electronic annotation

/ inferred from electronic annotation

rom electronic annotation /// 0005515 // protein binding // inferred from physical interaction /// 0005515 // pr

ctronic annotation /// 0008270 // zinc ion binding // inferred from electronic annotation /// 0019894 // kinesin

n /// 0017046 // peptide hormone binding // not recorded /// 0017046 // peptide hormone binding // inferred
n /// 0017046 // peptide hormone binding // not recorded /// 0017046 // peptide hormone binding // inferred

402 // histone acetyltransferase activity // inferred from electronic annotation /// 0004871 // signal transducer
402 // histone acetyltransferase activity // inferred from electronic annotation /// 0004871 // signal transducer

tronic annotation /// 0070652 // HAUS complex // inferred from sequence or structural similarity
m electronic annotation /// 0042803 // protein homodimerization activity // inferred from direct assay /// 004

547 // phosphatidylinositol-3,4,5-trisphosphate binding // inferred from direct assay
/ inferred from electronic annotation

ine/threonine kinase activity // not recorded /// 0004674 // protein serine/threonine kinase activity // inferred
inferred from direct assay /// 0005515 // protein binding // inferred from physical interaction /// 0005515 // pr
pecific DNA binding // inferred from electronic annotation /// 0046983 // protein dimerization activity // inferre

// inferred from sequence or structural similarity /// 0004683 // calmodulin-dependent protein kinase activity /

idonic acid monooxygenase activity // not recorded /// 0008391 // arachidonic acid monooxygenase activity //
idonic acid monooxygenase activity // not recorded /// 0008391 // arachidonic acid monooxygenase activity //

tion /// 0016791 // phosphatase activity // inferred from electronic annotation

0 // ligand-gated sodium channel activity // inferred from genetic interaction /// 0015280 // ligand-gated sodiu

ace binding // inferred from electronic annotation

t recorded /// 0004340 // glucokinase activity // inferred from electronic annotation /// 0004340 // glucokinase

ion /// 0005524 // ATP binding // inferred from electronic annotation /// 0016301 // kinase activity // inferred f
72 // metal ion binding // inferred from electronic annotation

3 // protein complex binding // not recorded /// 0032403 // protein complex binding // inferred from electronic

ansferase activity // inferred from electronic annotation /// 0004582 // dolichyl-phosphate beta-D-mannosyltra

ctronic annotation /// 0004942 // anaphylatoxin receptor activity // inferred from electronic annotation /// 000
05515 // protein binding // inferred from physical interaction /// 0005515 // protein binding // inferred from ele
ivity // inferred from electronic annotation /// 0042164 // interleukin-12 alpha subunit binding // inferred from
ronic annotation

; // inferred from electronic annotation

ronic annotation /// 0008236 // serine-type peptidase activity // inferred from electronic annotation /// 001678

physical interaction /// 0005515 // protein binding // inferred from electronic annotation /// 0005516 // calmod

annotation /// 0005529 // sugar binding // traceable author statement /// 0005529 // sugar binding // inferred
0046872 // metal ion binding // inferred from electronic annotation

enhancer binding RNA polymerase II transcription factor activity // inferred from direct assay /// 0003705 // sec
A binding transcription factor activity // inferred from direct assay /// 0003714 // transcription corepressor activ

annotation

16021 // integral to membrane // inferred from electronic annotation

pendent phospholipase A2 activity // not recorded /// 0047498 // calcium-dependent phospholipase A2 activity

nologous recombination // inferred from mutant phenotype /// 0030539 // male genitalia development // infer
ation

)8527 // taste receptor activity // inferred from electronic annotation /// 0033041 // sweet taste receptor activi

s binding // not recorded /// 0017124 // SH3 domain binding // inferred from electronic annotation /// 001990

nferred from physical interaction /// 0005515 // protein binding // inferred from electronic annotation /// 0005

/// 0016874 // ligase activity // inferred from electronic annotation /// 0046872 // metal ion binding // inferred
on /// 0005515 // protein binding // inferred from physical interaction /// 0016563 // transcription activator act
tation /// 0051879 // Hsp90 protein binding // inferred from electronic annotation

tion /// 0020037 // heme binding // inferred from electronic annotation /// 0033783 // 25-hydroxycholesterol
tion /// 0020037 // heme binding // inferred from electronic annotation /// 0033783 // 25-hydroxycholesterol

)16301 // kinase activity // inferred from electronic annotation /// 0016740 // transferase activity // inferred fro

tivator activity // inferred from direct assay

// 0005254 // chloride channel activity // not recorded /// 0005254 // chloride channel activity // inferred from

scription activator activity // not recorded /// 0016563 // transcription activator activity // inferred from electro
inferred from direct assay /// 0004923 // leukemia inhibitory factor receptor activity // inferred from electronic
ation

// zinc ion binding // inferred from electronic annotation /// 0010843 // promoter binding // inferred from dire

membrane transporter activity // not recorded /// 0015431 // glutathione S-conjugate-exporting ATPase activit

ic-AMP phosphodiesterase activity // inferred from electronic annotation /// 0005515 // protein binding // infer
tion
n physical interaction /// 0016787 // hydrolase activity // inferred from electronic annotation /// 0046872 // me

ferred from electronic annotation /// 0005524 // ATP binding // inferred from electronic annotation /// 001630

ɔnic annotation /// 0050839 // cell adhesion molecule binding // inferred from electronic annotation
ctivity // inferred from electronic annotation /// 0016776 // phosphotransferase activity, phosphate group as ac
// 0015226 // carnitine transporter activity // inferred from mutant phenotype /// 0015226 // carnitine transpo
annotation
tein binding // inferred from physical interaction /// 0005515 // protein binding // inferred from electronic annc
red from electronic annotation /// 0030528 // transcription regulator activity // inferred from electronic annota

red from sequence or structural similarity /// 0008083 // growth factor activity // inferred from electronic annot

/ 0046872 // metal ion binding // inferred from electronic annotation

tronic annotation /// 0004980 // melanocyte-stimulating hormone receptor activity // inferred from direct assa

vity // inferred from electronic annotation /// 0016740 // transferase activity // inferred from electronic annota
serine/threonine kinase activity // not recorded /// 0004674 // protein serine/threonine kinase activity // inferr

.287 // NAD or NADH binding // not recorded

0008201 // heparin binding // not recorded /// 0008201 // heparin binding // inferred from electronic annotat

. // phosphoinositide binding // inferred from electronic annotation

nce-specific DNA binding // inferred from electronic annotation

l interaction /// 0008168 // methyltransferase activity // inferred from electronic annotation /// 0008757 // S-a

ıt phenotype

ɔnic annotation /// 0050661 // NADP or NADPH binding // inferred from electronic annotation

ı /// 0005515 // protein binding // inferred from electronic annotation /// 0008080 // N-acetyltransferase activi

rom electronic annotation /// 0004871 // signal transducer activity // inferred from electronic annotation /// 0C

ınotation /// 0046332 // SMAD binding // inferred from physical interaction /// 0048407 // platelet-derived grc

// inferred from sequence or structural similarity /// 0030169 // low-density lipoprotein binding // inferred fror

ısferase activity, transferring glycosyl groups // inferred from electronic annotation /// 0046872 // metal ion bir

72 // metal ion binding // inferred from electronic annotation /// 0070330 // aromatase activity // inferred fror

ferred from electronic annotation /// 0008134 // transcription factor binding // inferred from electronic annota

ferred from electronic annotation /// 0008332 // low voltage-gated calcium channel activity // inferred from dir

ɔreductase activity, acting on single donors with incorporation of molecular oxygen, incorporation of two atoms
l from physical interaction /// 0005515 // protein binding // inferred from electronic annotation /// 0016563 //

tron carrier activity // inferred from electronic annotation /// 0016491 // oxidoreductase activity // inferred fror

// inferred from electronic annotation /// 0046625 // sphingolipid binding // not recorded /// 0046625 // sphin
0 // G-protein coupled receptor activity // inferred from electronic annotation /// 0004962 // endothelin recept
from electronic annotation /// 0004674 // protein serine/threonine kinase activity // inferred from sequence o

ıatase activity // inferred from electronic annotation /// 0046872 // metal ion binding // inferred from electron

ronic annotation /// 0048407 // platelet-derived growth factor binding // not recorded /// 0048407 // platelet-ɑ
esulting in the reduction of molecular oxygen to two molecules of water // not recorded /// 0016717 // oxidore

: // inferred from electronic annotation /// 0032592 // integral to mitochondrial membrane // inferred from ele
c ion binding // inferred from electronic annotation /// 0030528 // transcription regulator activity // inferred fr

/ ubiquitin thiolesterase activity // inferred from sequence or structural similarity /// 0008233 // peptidase activ
/ ubiquitin thiolesterase activity // inferred from sequence or structural similarity /// 0008233 // peptidase activ

ıctural similarity /// 0051721 // protein phosphatase 2A binding // inferred from electronic annotation /// 0051

6874 // ligase activity // inferred from electronic annotation /// 0043758 // acetate-CoA ligase (ADP-forming) ac

; // inferred from electronic annotation /// 0046872 // metal ion binding // inferred from electronic annotation

cular oxygen, incorporation of two atoms of oxygen // inferred from electronic annotation /// 0046872 // metal
onic annotation /// 0004872 // receptor activity // inferred from electronic annotation /// 0005007 // fibroblast

igase activity, forming phosphoric ester bonds // inferred from electronic annotation

inferred from electronic annotation
om direct assay /// 0043565 // sequence-specific DNA binding // inferred from electronic annotation /// 004698

016779 // nucleotidyltransferase activity // inferred from electronic annotation

ein serine/threonine kinase activity // inferred from electronic annotation /// 0004713 // protein tyrosine kinase
'/ not recorded /// 0043565 // sequence-specific DNA binding // inferred from electronic annotation
'/ not recorded /// 0043565 // sequence-specific DNA binding // inferred from electronic annotation

'/ 0046872 // metal ion binding // inferred from electronic annotation
erred from electronic annotation /// 0016301 // kinase activity // inferred from electronic annotation /// 00167

m electronic annotation
ctronic annotation /// 0016491 // oxidoreductase activity // inferred from electronic annotation /// 0016742 //
red from electronic annotation

// inferred from electronic annotation /// 0008026 // ATP-dependent helicase activity // inferred from electroni

n /// 0008233 // peptidase activity // inferred from electronic annotation /// 0008234 // cysteine-type peptida

y // inferred from electronic annotation /// 0005524 // ATP binding // not recorded /// 0005524 // ATP binding

rosine kinase activity // inferred from electronic annotation /// 0004716 // receptor signaling protein tyrosine k

nic annotation /// 0005524 // ATP binding // inferred from electronic annotation /// 0016301 // kinase activity

// inferred from electronic annotation /// 0004872 // receptor activity // inferred from direct assay /// 000487
recorded /// 0003697 // single-stranded DNA binding // not recorded /// 0005125 // cytokine activity // inferre

// sequence-specific DNA binding transcription factor activity // inferred from electronic annotation /// 00048

// inferred from electronic annotation
707 // steroid hormone receptor activity // inferred from electronic annotation /// 0004872 // receptor activity

activator activity // inferred from sequence or structural similarity /// 0046966 // thyroid hormone receptor bin

vity, acting on single donors with incorporation of molecular oxygen, incorporation of two atoms of oxygen // ir

inferred from electronic annotation /// 0005524 // ATP binding // inferred from electronic annotation /// 0005[illegible]

ctivity // inferred from electronic annotation /// 0005026 // transforming growth factor beta receptor activity, t
annotation /// 0005529 // sugar binding // traceable author statement /// 0005529 // sugar binding // inferred
otein binding // inferred from electronic annotation
electronic annotation /// 0042803 // protein homodimerization activity // inferred from physical interaction

binding // inferred from sequence or structural similarity

/ 0030971 // receptor tyrosine kinase binding // not recorded /// 0030971 // receptor tyrosine kinase binding //
rotein tyrosine/serine/threonine phosphatase activity // inferred from electronic annotation /// 0016787 // hyd

ity
/ 0004709 // MAP kinase kinase kinase activity // inferred from electronic annotation /// 0005057 // receptor s

inding // inferred from electronic annotation /// 0070290 // NAPE-specific phospholipase D activity // inferred f
ivity // inferred from electronic annotation /// 0043565 // sequence-specific DNA binding // inferred from direc

/ 0019904 // protein domain specific binding // inferred from electronic annotation /// 0035197 // siRNA bindi
onic annotation /// 0016301 // kinase activity // inferred from electronic annotation /// 0016740 // transferase

nferred from electronic annotation /// 0005525 // GTP binding // inferred from electronic annotation /// 00081

564 // transcription repressor activity // inferred from sequence or structural similarity /// 0016566 // specific tr

0046872 // metal ion binding // inferred from electronic annotation
515 // protein binding // inferred from electronic annotation /// 0005525 // GTP binding // not recorded /// 000
05515 // protein binding // inferred from physical interaction /// 0032403 // protein complex binding // not rec
ent cyclic-nucleotide phosphodiesterase activity // not recorded /// 0004117 // calmodulin-dependent cyclic-nu
/ not recorded

wth factor activity // inferred from electronic annotation /// 0042802 // identical protein binding // inferred fro
tivity // inferred from electronic annotation /// 0016747 // transferase activity, transferring acyl groups other th

'// 0016301 // kinase activity // inferred from electronic annotation /// 0016740 // transferase activity // inferre

eceptor activity // inferred from electronic annotation /// 0016829 // lyase activity // inferred from electronic a
ng // inferred from electronic annotation
/ inferred from electronic annotation
lectronic annotation /// 0008270 // zinc ion binding // inferred from electronic annotation /// 0016787 // hydrc
nding // inferred from electronic annotation /// 0008270 // zinc ion binding // inferred from electronic annotati
// inferred from electronic annotation /// 0016021 // integral to membrane // inferred from direct assay /// 0C
m electronic annotation /// 0016779 // nucleotidyltransferase activity // inferred from electronic annotation //
activity // inferred from electronic annotation /// 0004872 // receptor activity // inferred from electronic annot
xygen, incorporation of two atoms of oxygen // inferred from electronic annotation /// 0046872 // metal ion bir

/ inferred from electronic annotation
rred from electronic annotation /// 0016491 // oxidoreductase activity // inferred from electronic annotation /

/// 0045340 // mercury ion binding // inferred from electronic annotation
; // inferred from electronic annotation

nding // inferred from electronic annotation

3146 // sulfotransferase activity // inferred from electronic annotation /// 0016740 // transferase activity // infe
'// 0005516 // calmodulin binding // inferred from direct assay /// 0005516 // calmodulin binding // inferred frc

ng // inferred from direct assay

or structural similarity /// 0005515 // protein binding // inferred from electronic annotation /// 0005525 // GTF

// glutathione binding // inferred from sequence or structural similarity /// 0046872 // metal ion binding // infe

0046872 // metal ion binding // inferred from electronic annotation

0046872 // metal ion binding // inferred from electronic annotation

ctivity // inferred from electronic annotation /// 0005524 // ATP binding // not recorded /// 0005524 // ATP bir
rom electronic annotation /// 0008270 // zinc ion binding // inferred from electronic annotation /// 0016787 //

4871 // signal transducer activity // inferred from electronic annotation /// 0004872 // receptor activity // inferr

corded
inferred from electronic annotation
tation /// 0016757 // transferase activity, transferring glycosyl groups // inferred from electronic annotation //

016779 // nucleotidyltransferase activity // inferred from electronic annotation

0016740 // transferase activity // inferred from electronic annotation /// 0046872 // metal ion binding // inferr

red from electronic annotation /// 0030528 // transcription regulator activity // inferred from electronic annota
corded /// 0005234 // extracellular-glutamate-gated ion channel activity // inferred from electronic annotation
from electronic annotation /// 0004674 // protein serine/threonine kinase activity // inferred from sequence o
n binding // inferred from electronic annotation /// 0016787 // hydrolase activity // inferred from electronic an

ic annotation

tein binding // inferred from electronic annotation /// 0042802 // identical protein binding // inferred from ele

ferred from electronic annotation

nic annotation /// 0008270 // zinc ion binding // inferred from electronic annotation /// 0016491 // oxidoreduc
helicase activity // inferred from electronic annotation /// 0016787 // hydrolase activity // inferred from electro

ence binding // not recorded /// 0008139 // nuclear localization sequence binding // inferred from electronic an

inferred from electronic annotation /// 0042809 // vitamin D receptor binding // not recorded

ty // inferred from electronic annotation /// 0050660 // FAD or FADH2 binding // inferred from electronic annot

ferred from electronic annotation

inferred from electronic annotation /// 0005515 // protein binding // inferred from electronic annotation /// 0
tron carrier activity // inferred from electronic annotation /// 0016491 // oxidoreductase activity // inferred fro

16876 // ligase activity, forming aminoacyl-tRNA and related compounds // inferred from electronic annotation

ation

ed from electronic annotation /// 0016301 // kinase activity // inferred from electronic annotation /// 0016740

annotation

ansferase activity // inferred from electronic annotation /// 0016757 // transferase activity, transferring glycosy

// not recorded /// 0005515 // protein binding // inferred from electronic annotation /// 0030544 // Hsp70 pro

uthor statement /// 0003887 // DNA-directed DNA polymerase activity // inferred from electronic annotation //

n binding // inferred from electronic annotation /// 0016787 // hydrolase activity // inferred from electronic an
n binding // inferred from electronic annotation /// 0016787 // hydrolase activity // inferred from electronic an

/ 0008201 // heparin binding // inferred from electronic annotation /// 0043394 // proteoglycan binding // infer

/ 0046872 // metal ion binding // inferred from electronic annotation
quence or structural similarity /// 0046872 // metal ion binding // inferred from electronic annotation

similarity /// 0008237 // metallopeptidase activity // inferred from electronic annotation /// 0008270 // zinc ion

; // inferred from electronic annotation

activity // not recorded /// 0005391 // sodium:potassium-exchanging ATPase activity // inferred from electronic
tamin D receptor binding // not recorded /// 0042809 // vitamin D receptor binding // inferred from electronic

binding // inferred from electronic annotation /// 0005515 // protein binding // inferred from physical interacti

nnotation
reductase activity, acting on single donors with incorporation of molecular oxygen, incorporation of two atoms

nt phenotype

ctronic annotation /// 0008270 // zinc ion binding // inferred from electronic annotation /// 0019894 // kinesin
16876 // ligase activity, forming aminoacyl-tRNA and related compounds // inferred from electronic annotation

ulfide as acceptor // inferred from electronic annotation /// 0050660 // FAD or FADH2 binding // inferred from

m sequence or structural similarity /// 0030374 // ligand-dependent nuclear receptor transcription coactivator

m sequence or structural similarity /// 0030374 // ligand-dependent nuclear receptor transcription coactivator

inferred from electronic annotation /// 0046983 // protein dimerization activity // inferred from electronic ann

erred from electronic annotation
helicase activity // inferred from electronic annotation /// 0016787 // hydrolase activity // inferred from electro
say /// 0019145 // aminobutyraldehyde dehydrogenase activity // not recorded /// 0019145 // aminobutyraldel

›inding // not recorded
›onic annotation /// 0017022 // myosin binding // inferred from electronic annotation /// 0019904 // protein do
t recorded /// 0031721 // hemoglobin alpha binding // inferred from electronic annotation /// 0046872 // meta
›m direct assay /// 0043565 // sequence-specific DNA binding // inferred from electronic annotation /// 004698

›n binding // inferred from electronic annotation
' inferred from electronic annotation /// 0016740 // transferase activity // inferred from electronic annotation

d from electronic annotation /// 0070652 // HAUS complex // inferred from sequence or structural similarity

l from physical interaction /// 0005515 // protein binding // inferred from electronic annotation /// 0043565 //

d from electronic annotation /// 0005515 // protein binding // inferred from electronic annotation /// 0005515 ,

1 // acid-amino acid ligase activity // inferred from electronic annotation
ectronic annotation /// 0016740 // transferase activity // inferred from electronic annotation /// 0051996 // sq

on /// 0005515 // protein binding // inferred from electronic annotation /// 0005524 // ATP binding // inferred

ed from electronic annotation /// 0004843 // ubiquitin-specific protease activity // inferred from sequence or st

/ 0046872 // metal ion binding // inferred from electronic annotation /// 0070742 // C2H2 zinc finger domain b
)4715 // non-membrane spanning protein tyrosine kinase activity // inferred from electronic annotation /// 000

ion /// 0005515 // protein binding // inferred from electronic annotation /// 0005524 // ATP binding // inferred

ct assay /// 0004872 // receptor activity // inferred from electronic annotation /// 0005515 // protein binding //
rom electronic annotation /// 0003918 // DNA topoisomerase (ATP-hydrolyzing) activity // inferred from electro
ic annotation /// 0016831 // carboxy-lyase activity // inferred from electronic annotation

pinding // not recorded
nferred from electronic annotation /// 0003976 // UDP-N-acetylglucosamine-lysosomal-enzyme N-acetylglucosa
quence or structural similarity /// 0005544 // calcium-dependent phospholipid binding // inferred from sequen

/ 0016787 // hydrolase activity // inferred from electronic annotation /// 0046872 // metal ion binding // inferr
965 // G-protein alpha-subunit binding // inferred from electronic annotation /// 0004871 // signal transducer a

08270 // zinc ion binding // inferred from electronic annotation /// 0015923 // mannosidase activity // inferred

otation /// 0016874 // ligase activity // inferred from electronic annotation /// 0019787 // small conjugating pro

annotation /// 0016494 // C-X-C chemokine receptor activity // inferred from electronic annotation
) // zinc ion binding // inferred from electronic annotation /// 0016564 // transcription repressor activity // infe

ation

acyltransferase activity // inferred from electronic annotation /// 0016740 // transferase activity // inferred fron
erred from electronic annotation
DNA helicase activity // inferred from sequence or structural similarity /// 0004386 // helicase activity // inferre
inferred from electronic annotation

nteraction /// 0005515 // protein binding // inferred from physical interaction /// 0005515 // protein binding //

ferred from electronic annotation /// 0016504 // peptidase activator activity // inferred from mutant phenotyp
otein kinase binding // inferred from physical interaction /// 0046872 // metal ion binding // inferred from elect

ansmembrane transporter activity // not recorded /// 0015125 // bile acid transmembrane transporter activity

selective glutamate receptor activity // inferred from mutant phenotype /// 0004972 // N-methyl-D-aspartate s

'40 // transferase activity // inferred from electronic annotation
ace binding // inferred from electronic annotation

/ inferred from electronic annotation /// 0005515 // protein binding // inferred from electronic annotation /// (
/ sugar binding // inferred from electronic annotation
'/ 0016286 // small conductance calcium-activated potassium channel activity // not recorded /// 0016286 // sm
ransferase activity // inferred from electronic annotation /// 0016757 // transferase activity, transferring glycos
physical interaction /// 0005515 // protein binding // inferred from electronic annotation /// 0005516 // calmod

nferred from electronic annotation /// 0005003 // ephrin receptor activity // inferred from electronic annotatior

tation /// 0016740 // transferase activity // inferred from electronic annotation /// 0016787 // hydrolase activity
y // inferred from direct assay /// 0005515 // protein binding // inferred from electronic annotation /// 00055
d from electronic annotation /// 0016407 // acetyltransferase activity // inferred from electronic annotation //

ot recorded /// 0042802 // identical protein binding // inferred from electronic annotation
erred from electronic annotation

vity // inferred from electronic annotation /// 0008237 // metallopeptidase activity // inferred from electronic a
al interaction /// 0017147 // Wnt-protein binding // inferred from electronic annotation

'/ inferred from electronic annotation /// 0042802 // identical protein binding // inferred from physical interacti

on /// 0005524 // ATP binding // inferred from electronic annotation /// 0016301 // kinase activity // inferred fr
trophin binding // inferred from electronic annotation /// 0048406 // nerve growth factor binding // inferred fr

inferred from electronic annotation
ronic annotation

ferred from electronic annotation /// 0005524 // ATP binding // inferred from electronic annotation /// 00428[illegible]

/ 0003700 // sequence-specific DNA binding transcription factor activity // inferred from electronic annotation /
serine/threonine kinase activity // not recorded /// 0004674 // protein serine/threonine kinase activity // infer
annotation /// 0042802 // identical protein binding // inferred from electronic annotation /// 0043236 // lamin

author statement /// 0004714 // transmembrane receptor protein tyrosine kinase activity // inferred from elec

ity
drogen ion transporting ATP synthase activity, rotational mechanism // inferred from electronic annotation /// [illegible]
notation

on /// 0016874 // ligase activity // inferred from electronic annotation /// 0019787 // small conjugating protein
ase activity // inferred from electronic annotation /// 0016740 // transferase activity // inferred from electronic

denosine phosphorylase activity // inferred from electronic annotation

denosine phosphorylase activity // inferred from electronic annotation

erase activity // inferred from electronic annotation /// 0047192 // 1-alkylglycerophosphocholine O-acetyltrans

annotation

on /// 0005515 // protein binding // inferred from physical interaction /// 0016563 // transcription activator act

8134 // transcription factor binding // inferred from electronic annotation /// 0043565 // sequence-specific DN
binding // inferred from sequence or structural similarity /// 0030628 // pre-mRNA 3'-splice site binding // infer
aminyltransferase activity // inferred from electronic annotation /// 0016262 // protein N-acetylglucosaminyltr
acceptor // inferred from electronic annotation /// 0042282 // hydroxymethylglutaryl-CoA reductase activity //

one reductase activity // not recorded /// 0003960 // NADPH:quinone reductase activity // inferred from electro

vity, acting on the CH-NH2 group of donors, oxygen as acceptor // inferred from electronic annotation /// 00468
nnotation /// 0050662 // coenzyme binding // inferred from electronic annotation
6-mannosylglycoprotein 2-beta-N-acetylglucosaminyltransferase activity // inferred from electronic annotation ,

helicase activity // inferred from electronic annotation /// 0016787 // hydrolase activity // inferred from electro

nding transcription factor activity // inferred from sequence or structural similarity /// 0005515 // protein bindin
2605 // peptide antigen binding // inferred from electronic annotation

nic annotation /// 0005874 // microtubule // inferred from electronic annotation

016787 // hydrolase activity // inferred from electronic annotation /// 0046872 // metal ion binding // inferred f

ɣntaxin binding // not recorded /// 0035091 // phosphoinositide binding // not recorded /// 0042803 // protein

erred from electronic annotation /// 0016563 // transcription activator activity // inferred from electronic anno
al similarity /// 0004714 // transmembrane receptor protein tyrosine kinase activity // inferred from electronic a

/// 0015662 // ATPase activity, coupled to transmembrane movement of ions, phosphorylative mechanism // ir

calcium ion binding // inferred from electronic annotation /// 0005515 // protein binding // inferred from elect
ded /// 0004714 // transmembrane receptor protein tyrosine kinase activity // inferred from electronic annotat
/ metallopeptidase activity // not recorded /// 0008237 // metallopeptidase activity // inferred from electronic

088 // carbamoyl-phosphate synthase (glutamine-hydrolyzing) activity // not recorded /// 0004151 // dihydroo
ferred from electronic annotation

n sequence or structural similarity /// 0008353 // RNA polymerase II carboxy-terminal domain kinase activity //

// kinase activity // inferred from electronic annotation /// 0016303 // 1-phosphatidylinositol-3-kinase activity /

40 // transferase activity // inferred from electronic annotation /// 0016773 // phosphotransferase activity, alc

electronic annotation /// 0046872 // metal ion binding // inferred from electronic annotation
red from electronic annotation /// 0003727 // single-stranded RNA binding // inferred from direct assay /// 000
phosphatidylinositol-3-phosphatase activity // inferred from sequence or structural similarity /// 0004721 // ph

ic annotation /// 0016791 // phosphatase activity // inferred from electronic annotation

on /// 0046872 // metal ion binding // inferred from electronic annotation

on /// 0016874 // ligase activity // inferred from electronic annotation /// 0019787 // small conjugating protein

005254 // chloride channel activity // inferred from electronic annotation /// 0005515 // protein binding // infe

0016740 // transferase activity // inferred from electronic annotation
)005524 // ATP binding // inferred from electronic annotation /// 0008270 // zinc ion binding // inferred from e

annotation
/ metallopeptidase activity // not recorded /// 0008237 // metallopeptidase activity // inferred from electronic a

ı binding // not recorded /// 0005509 // calcium ion binding // inferred from electronic annotation /// 0005515

tation /// 0004983 // neuropeptide Y receptor activity // inferred from electronic annotation

/ // inferred from electronic annotation /// 0008157 // protein phosphatase 1 binding // not recorded /// 00167
sical interaction /// 0005524 // ATP binding // inferred from electronic annotation /// 0016740 // transferase ac

nic annotation /// 0016787 // hydrolase activity // inferred from electronic annotation /// 0030247 // polysacch

ulfide as acceptor // inferred from electronic annotation /// 0050660 // FAD or FADH2 binding // inferred from e
IA polymerase II transcription factor activity // not recorded /// 0016563 // transcription activator activity // infe
ation /// 0031418 // L-ascorbic acid binding // inferred from electronic annotation /// 0046872 // metal ion binc
ferred from physical interaction /// 0005515 // protein binding // inferred from electronic annotation /// 00055
red from electronic annotation
ed from electronic annotation

nchored to membrane // inferred from electronic annotation
c annotation /// 0016769 // transferase activity, transferring nitrogenous groups // inferred from electronic anr
6874 // ligase activity // inferred from electronic annotation /// 0043758 // acetate-CoA ligase (ADP-forming) ac
l receptor activity // inferred from electronic annotation

d from electronic annotation /// 0005524 // ATP binding // inferred from electronic annotation /// 0016853 // i

rosine kinase activity // inferred from electronic annotation /// 0004716 // receptor signaling protein tyrosine k

similarity /// 0008237 // metallopeptidase activity // inferred from electronic annotation /// 0008270 // zinc ior

tation

receptor activity // inferred from mutant phenotype /// 0004872 // receptor activity // inferred from electronic
: enhancer binding RNA polymerase II transcription factor activity // inferred from electronic annotation /// 000

// inferred from electronic annotation

)005524 // ATP binding // inferred from electronic annotation /// 0016874 // ligase activity // inferred from ele

8134 // transcription factor binding // inferred from electronic annotation /// 0030528 // transcription regulatc

rom electronic annotation /// 0030414 // peptidase inhibitor activity // inferred from electronic annotation

/// 0016491 // oxidoreductase activity // inferred from electronic annotation /// 0016702 // oxidoreductase ac

'0 // zinc ion binding // inferred from electronic annotation /// 0042156 // zinc-mediated transcriptional activat

agnesium-dependent protein serine/threonine phosphatase activity // inferred from electronic annotation /// C

ion /// 0016787 // hydrolase activity // inferred from electronic annotation
otein tyrosine kinase activity // inferred from electronic annotation /// 0004872 // receptor activity // inferred fr
otein tyrosine kinase activity // inferred from electronic annotation /// 0004872 // receptor activity // inferred fr
pholipid binding // inferred from electronic annotation /// 0046872 // metal ion binding // inferred from electrc
onic annotation

ɨ RING ubiquitin ligase complex // inferred from sequence or structural similarity
ɨ24 // ATP binding // inferred from electronic annotation /// 0016301 // kinase activity // inferred from electror

ɩding // inferred from mutant phenotype
// AMP-activated protein kinase activity // not recorded /// 0004679 // AMP-activated protein kinase activity //

ɔrane // inferred from electronic annotation

rred from electronic annotation /// 0016491 // oxidoreductase activity // inferred from electronic annotation /,

' 0003700 // sequence-specific DNA binding transcription factor activity // inferred from mutant phenotype /// (

72 // metal ion binding // inferred from electronic annotation
ɩse activity // inferred from sequence or structural similarity /// 0016740 // transferase activity // inferred from

ɟ from electronic annotation /// 0070652 // HAUS complex // inferred from sequence or structural similarity

ectronic annotation

:al interaction /// 0008081 // phosphoric diester hydrolase activity // inferred from electronic annotation /// 00

helicase activity // inferred from electronic annotation

ce or structural similarity

ı electronic annotation /// 0016740 // transferase activity // inferred from electronic annotation /// 0018024 //

'/ transferase activity // inferred from electronic annotation

ıotation /// 0016301 // kinase activity // inferred from electronic annotation /// 0019901 // protein kinase bind

tion

ansferase activity // inferred from electronic annotation
or structural similarity /// 0003705 // sequence-specific enhancer binding RNA polymerase II transcription facto

g // inferred from physical interaction
tation /// 0030306 // ADP-ribosylation factor binding // inferred from sequence or structural similarity /// 0042

/ 0050660 // FAD or FADH2 binding // not recorded /// 0050660 // FAD or FADH2 binding // inferred from elect

tation

' // inferred from sequence or structural similarity

ıl interaction /// 0017147 // Wnt-protein binding // inferred from electronic annotation

l from physical interaction /// 0005515 // protein binding // inferred from electronic annotation /// 0019904 //

direct assay /// 0004872 // receptor activity // inferred from electronic annotation /// 0005515 // protein bindir

0004402 // histone acetyltransferase activity // inferred from electronic annotation /// 0004871 // signal transd
GDP binding // inferred from electronic annotation /// 0019003 // GDP binding // inferred from sequence or str

onic annotation /// 0016740 // transferase activity // inferred from electronic annotation

action /// 0051020 // GTPase binding // inferred from electronic annotation

al similarity /// 0005515 // protein binding // inferred from physical interaction /// 0005515 // protein binding /

onic annotation /// 0050661 // NADP or NADPH binding // inferred from electronic annotation

ectronic annotation /// 0016740 // transferase activity // inferred from electronic annotation /// 0016773 // ph

n /// 0004879 // ligand-dependent nuclear receptor activity // inferred from direct assay /// 0004879 // ligand-
402 // histone acetyltransferase activity // inferred from electronic annotation /// 0004871 // signal transducer
rosine kinase activity // inferred from electronic annotation /// 0004716 // receptor signaling protein tyrosine k

/// 0030229 // very-low-density lipoprotein receptor activity // inferred from sequence or structural similarity

ase activity // inferred from electronic annotation /// 0016740 // transferase activity // inferred from electronic

// hydrolase activity // inferred from electronic annotation /// 0046872 // metal ion binding // inferred from ele

302 // identical protein binding // inferred from electronic annotation /// 0042803 // protein homodimerization

otransferase activity, alcohol group as acceptor // inferred from electronic annotation
onic annotation

tation /// 0004709 // MAP kinase kinase kinase activity // inferred from electronic annotation /// 0005515 // pr

nding // inferred from electronic annotation

/ inferred from electronic annotation /// 0005515 // protein binding // inferred from electronic annotation /// (

/ 0016175 // superoxide-generating NADPH oxidase activity // inferred from electronic annotation /// 0016491

from electronic annotation

rotein domain specific binding // inferred from electronic annotation
ng // inferred from electronic annotation
ng // inferred from electronic annotation

ıic annotation /// 0008237 // metallopeptidase activity // inferred from electronic annotation /// 0008270 // zir

0046872 // metal ion binding // inferred from electronic annotation
from electronic annotation

ng // inferred from electronic annotation /// 0016787 // hydrolase activity // inferred from electronic annotatic

:tronic annotation /// 0046872 // metal ion binding // inferred from electronic annotation
›inding transcription factor activity // inferred from electronic annotation /// 0003704 // specific RNA polymera

; hexosyl groups // inferred from electronic annotation

›n binding // inferred from electronic annotation

DD8-specific protease activity // not recorded

lectronic annotation /// 0008144 // drug binding // not recorded /// 0008144 // drug binding // inferred from e

rred from electronic annotation /// 0030676 // Rac guanyl-nucleotide exchange factor activity // inferred from :

| from electronic annotation /// 0008237 // metallopeptidase activity // inferred from electronic annotation ///

nferred from electronic annotation

16757 // transferase activity, transferring glycosyl groups // inferred from electronic annotation
| plasma membrane // inferred from electronic annotation /// 0030054 // cell junction // inferred from electron
/ 0016301 // kinase activity // inferred from electronic annotation /// 0016740 // transferase activity // inferred

nnotation /// 0005524 // ATP binding // inferred from electronic annotation /// 0016291 // acyl-CoA thioesteras
ronic annotation /// 0035226 // glutamate-cysteine ligase catalytic subunit binding // inferred from direct assay

ed from electronic annotation /// 0016301 // kinase activity // inferred from electronic annotation /// 0016740
electronic annotation /// 0016740 // transferase activity // inferred from electronic annotation /// 0018024 //

tation /// 0042054 // histone methyltransferase activity // inferred from direct assay

n

/ 0046872 // metal ion binding // inferred from electronic annotation /// 0070742 // C2H2 zinc finger domain b
0046872 // metal ion binding // inferred from electronic annotation

/ 0016787 // hydrolase activity // inferred from electronic annotation /// 0046872 // metal ion binding // inferr

nnotation /// 0016491 // oxidoreductase activity // inferred from electronic annotation /// 0016874 // ligase act

direct assay /// 0004872 // receptor activity // inferred from electronic annotation /// 0005515 // protein bindir
ferred from electronic annotation /// 0005216 // ion channel activity // inferred from electronic annotation ///

inding // inferred from electronic annotation /// 0005524 // ATP binding // inferred from electronic annotation

/ 0016787 // hydrolase activity // inferred from electronic annotation /// 0046872 // metal ion binding // inferr

ing // inferred from physical interaction /// 0005515 // protein binding // inferred from electronic annotation /
08270 // zinc ion binding // inferred from electronic annotation /// 0016787 // hydrolase activity // inferred fro

ctronic annotation

2605 // peptide antigen binding // inferred from electronic annotation
rred from electronic annotation /// 0005249 // voltage-gated potassium channel activity // inferred from electro

'25 // protein tyrosine phosphatase activity // inferred from electronic annotation /// 0004725 // protein tyrosi

ıotation /// 0046872 // metal ion binding // inferred from electronic annotation

// not recorded

ɔr activity // inferred from electronic annotation /// 0004930 // G-protein coupled receptor activity // inferred f
1 // acid-amino acid ligase activity // inferred from electronic annotation

ɛlectronic annotation /// 0008022 // protein C-terminus binding // inferred from sequence or structural similari

ɛquence or structural similarity /// 0003678 // DNA helicase activity // inferred from electronic annotation /// 0

inferred from electronic annotation /// 0051018 // protein kinase A binding // not recorded

3 // protein complex binding // not recorded /// 0032403 // protein complex binding // inferred from electronic

xygen, incorporation of two atoms of oxygen // inferred from electronic annotation /// 0046872 // metal ion bir

ınt /// 0008270 // zinc ion binding // inferred from electronic annotation /// 0016787 // hydrolase activity // inf

m electronic annotation /// 0016624 // oxidoreductase activity, acting on the aldehyde or oxo group of donors,

red from electronic annotation /// 0030528 // transcription regulator activity // inferred from electronic annota
ed from electronic annotation

groups // inferred from electronic annotation

inferred from electronic annotation

tation /// 0016757 // transferase activity, transferring glycosyl groups // inferred from electronic annotation ///

onic annotation /// 0043565 // sequence-specific DNA binding // inferred from electronic annotation

016301 // kinase activity // inferred from electronic annotation /// 0016740 // transferase activity // inferred fro

helicase activity // inferred from electronic annotation /// 0016787 // hydrolase activity // inferred from electro

annotation /// 0004707 // MAP kinase activity // not recorded /// 0004707 // MAP kinase activity // inferred fro

notation /// 0046872 // metal ion binding // inferred from electronic annotation

/ 0046582 // Rap GTPase activator activity // inferred from electronic annotation

notation /// 0050277 // sedoheptulokinase activity // inferred from electronic annotation

annotation

nding // inferred from mutant phenotype

ding // inferred from electronic annotation /// 0008022 // protein C-terminus binding // not recorded /// 0008

rred from direct assay /// 0016853 // isomerase activity // inferred from electronic annotation /// 0018114 // tl

ferase activity // inferred from electronic annotation /// 0016300 // tRNA (uracil) methyltransferase activity // ir

ded DNA binding // inferred from electronic annotation /// 0003700 // sequence-specific DNA binding transcript

otein heterodimerization activity // inferred from electronic annotation

ion binding // inferred from electronic annotation
tion

l from direct assay
l DNA binding // not recorded /// 0003690 // double-stranded DNA binding // inferred from electronic annotati
/// 0004697 // protein kinase C activity // inferred from electronic annotation /// 0005515 // protein binding //
m electronic annotation

helicase activity // inferred from electronic annotation /// 0016787 // hydrolase activity // inferred from electro
ic annotation /// 0005515 // protein binding // inferred from physical interaction /// 0005524 // ATP binding //

ity // inferred from electronic annotation /// 0018024 // histone-lysine N-methyltransferase activity // inferred

›n binding // inferred from electronic annotation

:ein binding // inferred from electronic annotation /// 0005524 // ATP binding // inferred from electronic annota

om electronic annotation /// 0008270 // zinc ion binding // inferred from electronic annotation /// 0016874 // l

ic annotation /// 0046872 // metal ion binding // inferred from electronic annotation

ronic annotation

700 // sequence-specific DNA binding transcription factor activity // inferred from electronic annotation /// 000

/ protein-hormone receptor activity // not recorded /// 0016500 // protein-hormone receptor activity // inferre

›n /// 0008270 // zinc ion binding // inferred from electronic annotation /// 0016564 // transcription repressor

eable author statement /// 0043565 // sequence-specific DNA binding // inferred from electronic annotation //

)5524 // ATP binding // inferred from electronic annotation /// 0008294 // calcium- and calmodulin-responsive

5 // protein binding // inferred from electronic annotation /// 0005524 // ATP binding // inferred from electron

rotein tyrosine/serine/threonine phosphatase activity // inferred from electronic annotation /// 0016787 // hyd
activity // not recorded /// 0035091 // phosphoinositide binding // inferred from electronic annotation /// 0035

hatase activity // inferred from electronic annotation

// inferred from direct assay /// 0005115 // receptor tyrosine kinase-like orphan receptor binding // inferred fro
vity // inferred from electronic annotation /// 0016887 // ATPase activity // inferred from electronic annotation

ty // inferred from direct assay

annotation /// 0005515 // protein binding // inferred from electronic annotation /// 0030695 // GTPase regulato

electronic annotation /// 0016301 // kinase activity // inferred from electronic annotation /// 0016740 // trans

n /// 0008270 // zinc ion binding // inferred from electronic annotation /// 0016787 // hydrolase activity // infe
ed from electronic annotation /// 0016491 // oxidoreductase activity // inferred from electronic annotation ///
anscription factor activity // not recorded /// 0003706 // ligand-regulated transcription factor activity // inferred

) // sequence-specific DNA binding transcription factor activity // inferred from electronic annotation /// 00048

cceptor // inferred from electronic annotation /// 0016619 // malate dehydrogenase (oxaloacetate-decarboxyla

notation /// 0005515 // protein binding // inferred from electronic annotation

xygen, incorporation of two atoms of oxygen // inferred from electronic annotation /// 0046872 // metal ion bir

nferred from electronic annotation /// 0008144 // drug binding // not recorded /// 0016787 // hydrolase activit

inferred from electronic annotation

tation

'// 0008270 // zinc ion binding // inferred from electronic annotation /// 0016874 // ligase activity // inferred fr

nnotation /// 0000832 // inositol hexakisphosphate 5-kinase activity // inferred from sequence or structural sim
n /// 0004879 // ligand-dependent nuclear receptor activity // inferred from direct assay /// 0004879 // ligand-

/// 0019003 // GDP binding // inferred from electronic annotation /// 0019003 // GDP binding // inferred from
ng // inferred from electronic annotation /// 0016491 // oxidoreductase activity // inferred from electronic ann

hydrolase activity // inferred from electronic annotation /// 0046872 // metal ion binding // inferred from elect

15 // protein binding // inferred from electronic annotation /// 0008022 // protein C-terminus binding // inferre

inferred from electronic annotation
ectronic annotation
m electronic annotation
m electronic annotation

m electronic annotation /// 0005515 // protein binding // inferred from electronic annotation /// 0005524 // A

tivity // inferred from sequence or structural similarity /// 0042169 // SH2 domain binding // inferred from sequ

otation

on /// 0016641 // oxidoreductase activity, acting on the CH-NH2 group of donors, oxygen as acceptor // inferred

on /// 0003706 // ligand-regulated transcription factor activity // inferred from sequence or structural similarity

ːrase activity // inferred from electronic annotation /// 0005515 // protein binding // inferred from electronic a

electronic annotation /// 0051287 // NAD or NADH binding // inferred from electronic annotation

ıy /// 0005770 // late endosome // not recorded /// 0005886 // plasma membrane // inferred from electronic a
ıy /// 0005770 // late endosome // not recorded /// 0005886 // plasma membrane // inferred from electronic a

ːd to transmembrane movement of substances // inferred from electronic annotation /// 0042803 // protein ho

ɿity // inferred from electronic annotation /// 0005158 // insulin receptor binding // not recorded /// 0005158 /

rred from electronic annotation /// 0048407 // platelet-derived growth factor binding // not recorded /// 0048[illegible]
008026 // ATP-dependent helicase activity // inferred from electronic annotation /// 0016564 // transcription r
m electronic annotation /// 0005515 // protein binding // inferred from electronic annotation /// 0005524 // AT
m electronic annotation /// 0005515 // protein binding // inferred from electronic annotation /// 0005524 // AT

calcium channel activity // inferred from direct assay /// 0005262 // calcium channel activity // not recorded ///

from electronic annotation
ı /// 0005515 // protein binding // inferred from physical interaction /// 0005515 // protein binding // inferred f

tation /// 0004879 // ligand-dependent nuclear receptor activity // inferred from electronic annotation /// 0004
tation /// 0004879 // ligand-dependent nuclear receptor activity // inferred from electronic annotation /// 0004

cleoside-triphosphatase activity // inferred from electronic annotation

ısporter activity // inferred from electronic annotation /// 0015189 // L-lysine transmembrane transporter activ

inding // inferred from electronic annotation /// 0045505 // dynein intermediate chain binding // not recorded

ictivity // inferred from electronic annotation /// 0004872 // receptor activity // inferred from electronic annota
5017 // platelet-derived growth factor receptor activity // not recorded /// 0005019 // platelet-derived growth

phosphatase activity // inferred from electronic annotation

ronic annotation /// 0016491 // oxidoreductase activity // inferred from electronic annotation /// 0019825 // o

ise activity // inferred from electronic annotation /// 0016787 // hydrolase activity // inferred from electronic a
s with incorporation of molecular oxygen, incorporation of two atoms of oxygen // not recorded /// 0016702 //

ein binding // inferred from physical interaction /// 0005515 // protein binding // inferred from electronic anno

tion /// 0046872 // metal ion binding // inferred from electronic annotation
tion /// 0046872 // metal ion binding // inferred from electronic annotation
iding // inferred from electronic annotation

il protein binding // inferred from electronic annotation /// 0046982 // protein heterodimerization activity // no

/ 0005515 // protein binding // inferred from physical interaction /// 0005515 // protein binding // inferred fror
rred from electronic annotation /// 0046983 // protein dimerization activity // inferred from electronic annotat

ein tyrosine kinase activity // not recorded /// 0004713 // protein tyrosine kinase activity // inferred from electr

on /// 0016787 // hydrolase activity // inferred from electronic annotation

d from electronic annotation /// 0005524 // ATP binding // inferred from electronic annotation /// 0008144 // d

// ammonia transmembrane transporter activity // inferred from electronic annotation

ferred from electronic annotation /// 0045211 // postsynaptic membrane // inferred from electronic annotatior

/ transcription repressor activity // inferred from electronic annotation

5 // cholesterol binding // inferred from electronic annotation /// 0016740 // transferase activity // inferred from
5 // cholesterol binding // inferred from electronic annotation /// 0016740 // transferase activity // inferred from

erred from electronic annotation /// 0042803 // protein homodimerization activity // inferred from electronic a

05515 // protein binding // inferred from electronic annotation /// 0005515 // protein binding // inferred from p
diol 17-beta-dehydrogenase activity // inferred from electronic annotation /// 0005488 // binding // inferred fro

ferred from electronic annotation /// 0005515 // protein binding // inferred from physical interaction /// 00164
19787 // small conjugating protein ligase activity // inferred from electronic annotation
19787 // small conjugating protein ligase activity // inferred from electronic annotation

528 // transcription regulator activity // inferred from electronic annotation
/ active organic cation transmembrane transporter activity // not recorded /// 0015075 // ion transmembrane tr

rity /// 0016564 // transcription repressor activity // inferred from electronic annotation /// 0043565 // sequen
binding transcription factor activity // inferred from electronic annotation /// 0008270 // zinc ion binding // inf

nic annotation /// 0016787 // hydrolase activity // inferred from electronic annotation /// 0017111 // nucleosid

otein binding // inferred from electronic annotation /// 0005524 // ATP binding // inferred from electronic ann

binding // inferred from electronic annotation /// 0019904 // protein domain specific binding // not recorded /

from electronic annotation /// 0031894 // V1A vasopressin receptor binding // not recorded /// 0031894 // V1

from electronic annotation /// 0031894 // V1A vasopressin receptor binding // not recorded /// 0031894 // V1

activity // inferred from electronic annotation /// 0005515 // protein binding // inferred from physical interact

activity // inferred from electronic annotation /// 0005515 // protein binding // inferred from physical interact

5982 // protein heterodimerization activity // inferred from direct assay /// 0070888 // E-box binding // inferrec

from electronic annotation /// 0005515 // protein binding // inferred from physical interaction /// 0005515 //

rotein binding // inferred from electronic annotation /// 0008134 // transcription factor binding // not recorded

ed from electronic annotation

/ inferred from electronic annotation /// 0005516 // calmodulin binding // inferred from electronic annotation

inferred from electronic annotation /// 0009055 // electron carrier activity // inferred from electronic annotatic

inferred from electronic annotation /// 0009055 // electron carrier activity // inferred from electronic annotatic

m channel activity // not recorded /// 0015280 // ligand-gated sodium channel activity // inferred from electror

activity // inferred from sequence or structural similarity /// 0004396 // hexokinase activity // inferred from ele

from electronic annotation /// 0016740 // transferase activity // inferred from electronic annotation

: annotation

nsferase activity // inferred from sequence or structural similarity /// 0005537 // mannose binding // not record

4943 // C3a anaphylatoxin receptor activity // not recorded /// 0004943 // C3a anaphylatoxin receptor activity /
ctronic annotation /// 0005524 // ATP binding // inferred from electronic annotation /// 0016301 // kinase acti
physical interaction /// 0042164 // interleukin-12 alpha subunit binding // not recorded /// 0042164 // interleu

37 // hydrolase activity // inferred from electronic annotation

lulin binding // non-traceable author statement /// 0005516 // calmodulin binding // inferred from electronic ar

from electronic annotation /// 0008061 // chitin binding // inferred from electronic annotation /// 0043169 // c

quence-specific enhancer binding RNA polymerase II transcription factor activity // inferred from genetic interac
vity // inferred from direct assay /// 0003723 // RNA binding // inferred from electronic annotation /// 0005488

// inferred from electronic annotation

rred from mutant phenotype /// 0032880 // regulation of protein localization // inferred from mutant phenotyp

ity // inferred from electronic annotation

4 // protein domain specific binding // inferred from electronic annotation /// 0032403 // protein complex bind

524 // ATP binding // inferred from electronic annotation /// 0008270 // zinc ion binding // inferred from electr

l from electronic annotation
tivity // inferred from direct assay /// 0042826 // histone deacetylase binding // inferred from physical interacti

7alpha-hydroxylase activity // inferred from electronic annotation /// 0046872 // metal ion binding // inferred f
7alpha-hydroxylase activity // inferred from electronic annotation /// 0046872 // metal ion binding // inferred f

om electronic annotation /// 0019208 // phosphatase regulator activity // inferred from direct assay

electronic annotation /// 0005515 // protein binding // inferred from electronic annotation

onic annotation /// 0016564 // transcription repressor activity // inferred from mutant phenotype /// 0016564 /
annotation /// 0004924 // oncostatin-M receptor activity // inferred from electronic annotation /// 0005127 //

ct assay /// 0016564 // transcription repressor activity // inferred from direct assay /// 0016566 // specific trans

y // not recorded /// 0015562 // efflux transmembrane transporter activity // not recorded /// 0016787 // hydr

rred from electronic annotation /// 0016787 // hydrolase activity // inferred from electronic annotation /// 004[illegible]

etal ion binding // inferred from electronic annotation

1 // kinase activity // inferred from electronic annotation /// 0016740 // transferase activity // inferred from ele

:ceptor // inferred from electronic annotation /// 0019201 // nucleotide kinase activity // inferred from electro
rter activity // not recorded /// 0015293 // symporter activity // inferred from electronic annotation /// 001565

otation /// 0017124 // SH3 domain binding // not recorded
ition

:ation /// 0008083 // growth factor activity // inferred from sequence or structural similarity

y /// 0004980 // melanocyte-stimulating hormone receptor activity // inferred from electronic annotation /// 0(

tion /// 0033862 // UMP kinase activity // not recorded
red from electronic annotation /// 0004674 // protein serine/threonine kinase activity // inferred from sequenc

ion /// 0042056 // chemoattractant activity // inferred from electronic annotation

denosylmethionine-dependent methyltransferase activity // not recorded /// 0016740 // transferase activity //

ty // inferred from electronic annotation /// 0010484 // H3 histone acetyltransferase activity // inferred from d

)05178 // integrin binding // not recorded /// 0005488 // binding // inferred from electronic annotation /// 000!

owth factor binding // not recorded /// 0048407 // platelet-derived growth factor binding // inferred from electr

m electronic annotation /// 0030169 // low-density lipoprotein binding // inferred from mutant phenotype /// (

nding // inferred from electronic annotation

n electronic annotation

tion /// 0008190 // eukaryotic initiation factor 4E binding // inferred from electronic annotation /// 0008301 //

ect assay /// 0008332 // low voltage-gated calcium channel activity // not recorded /// 0008332 // low voltage-

of oxygen // inferred from electronic annotation /// 0020037 // heme binding // inferred from electronic anno
transcription activator activity // inferred from genetic interaction

m electronic annotation /// 0046872 // metal ion binding // inferred from electronic annotation /// 0050660 //

golipid binding // inferred from electronic annotation
tor activity // inferred from mutant phenotype /// 0004962 // endothelin receptor activity // inferred from elect
r structural similarity /// 0004871 // signal transducer activity // inferred from electronic annotation /// 000551

ic annotation

derived growth factor binding // inferred from electronic annotation
ductase activity, acting on paired donors, with oxidation of a pair of donors resulting in the reduction of molecu

ctronic annotation /// 0043234 // protein complex // inferred from electronic annotation
om electronic annotation /// 0046872 // metal ion binding // inferred from electronic annotation

vity // inferred from electronic annotation /// 0008234 // cysteine-type peptidase activity // inferred from electr
vity // inferred from electronic annotation /// 0008234 // cysteine-type peptidase activity // inferred from electr

.721 // protein phosphatase 2A binding // inferred from sequence or structural similarity /// 0051722 // protein

ctivity // inferred from electronic annotation

l ion binding // inferred from electronic annotation
t growth factor receptor activity // inferred from direct assay /// 0005007 // fibroblast growth factor receptor a

3 // protein dimerization activity // inferred from electronic annotation

e activity // not recorded /// 0004713 // protein tyrosine kinase activity // inferred from electronic annotation /

40 // transferase activity // inferred from electronic annotation

' hydroxymethyl-, formyl- and related transferase activity // inferred from electronic annotation /// 0032403 // [illegible]

ic annotation /// 0016787 // hydrolase activity // inferred from electronic annotation

ıse activity // inferred from electronic annotation /// 0016787 // hydrolase activity // inferred from electronic ar

; // inferred from electronic annotation /// 0016301 // kinase activity // inferred from electronic annotation ///

inase activity // inferred from electronic annotation /// 0004871 // signal transducer activity // inferred from di

// inferred from electronic annotation /// 0016740 // transferase activity // inferred from electronic annotatior

2 // receptor activity // inferred from electronic annotation /// 0005003 // ephrin receptor activity // inferred fr
d from direct assay /// 0005125 // cytokine activity // not recorded /// 0005488 // binding // inferred from elec

71 // signal transducer activity // inferred from electronic annotation /// 0004872 // receptor activity // inferrec

' // inferred from electronic annotation /// 0004879 // ligand-dependent nuclear receptor activity // inferred frc

ıding // inferred from sequence or structural similarity

ıferred from electronic annotation

524 // ATP binding // inferred from sequence or structural similarity /// 0016301 // kinase activity // inferred fro

:ype II // not recorded /// 0005026 // transforming growth factor beta receptor activity, type II // inferred from
from electronic annotation /// 0008061 // chitin binding // inferred from electronic annotation /// 0043169 // c

/ inferred from electronic annotation /// 0043499 // eukaryotic cell surface binding // inferred from direct assay
lrolase activity // inferred from electronic annotation /// 0016791 // phosphatase activity // inferred from electr

ignaling protein activity // inferred from electronic annotation /// 0005515 // protein binding // inferred from p

from electronic annotation
:t assay /// 0043565 // sequence-specific DNA binding // inferred from electronic annotation

ng // inferred from direct assay /// 0070742 // C2H2 zinc finger domain binding // inferred from electronic annc
activity // inferred from electronic annotation

38 // protein tyrosine/serine/threonine phosphatase activity // inferred from electronic annotation /// 001674C

anscriptional repressor activity // inferred from direct assay /// 0017069 // snRNA binding // inferred from elec

)5525 // GTP binding // non-traceable author statement /// 0005525 // GTP binding // inferred from electronic a
orded /// 0032403 // protein complex binding // inferred from electronic annotation
ıcleotide phosphodiesterase activity // inferred from electronic annotation /// 0005516 // calmodulin binding //

m electronic annotation
ıan amino-acyl groups // inferred from electronic annotation /// 0032934 // sterol binding // inferred from elec

ed from electronic annotation /// 0019901 // protein kinase binding // not recorded /// 0019901 // protein kina

nnotation

plase activity // inferred from electronic annotation /// 0046872 // metal ion binding // inferred from electronic
ion /// 0016563 // transcription activator activity // inferred from direct assay /// 0019789 // SUMO ligase activi
016021 // integral to membrane // inferred from electronic annotation /// 0030018 // Z disc // inferred from ele
/ 0032947 // protein complex scaffold // not recorded /// 0042803 // protein homodimerization activity // not r
tation /// 0004879 // ligand-dependent nuclear receptor activity // inferred from electronic annotation /// 0008
nding // inferred from electronic annotation

// 0042802 // identical protein binding // not recorded /// 0046872 // metal ion binding // inferred from electro

erred from electronic annotation
om electronic annotation /// 0005524 // ATP binding // inferred from direct assay /// 0005524 // ATP binding //

binding // not recorded /// 0005525 // GTP binding // inferred from electronic annotation /// 0005525 // GTP

erred from electronic annotation /// 0050750 // low-density lipoprotein receptor binding // not recorded /// 00

nding // inferred from electronic annotation /// 0005525 // GTP binding // not recorded /// 0005525 // GTP binc
hydrolase activity // inferred from electronic annotation /// 0046872 // metal ion binding // inferred from elect

red from electronic annotation /// 0004930 // G-protein coupled receptor activity // inferred from electronic an

/ 0046872 // metal ion binding // inferred from electronic annotation

ed from electronic annotation

ation
/// 0005515 // protein binding // inferred from physical interaction /// 0008144 // drug binding // not recorded
r structural similarity /// 0005515 // protein binding // inferred from electronic annotation /// 0005524 // ATP b
notation /// 0046872 // metal ion binding // inferred from electronic annotation

ctronic annotation

tase activity // inferred from electronic annotation /// 0046872 // metal ion binding // inferred from electronic
onic annotation

notation /// 0034512 // box C/D snoRNA binding // not recorded /// 0034512 // box C/D snoRNA binding // infe

tation

005525 // GTP binding // inferred from electronic annotation /// 0019001 // guanyl nucleotide binding // inferr
m electronic annotation /// 0046872 // metal ion binding // inferred from electronic annotation /// 0050660 //

l

// transferase activity // inferred from electronic annotation /// 0016776 // phosphotransferase activity, phosp

yl groups // inferred from electronic annotation /// 0030170 // pyridoxal phosphate binding // inferred from ele

tein binding // not recorded /// 0032403 // protein complex binding // not recorded /// 0032403 // protein com

/ 0003887 // DNA-directed DNA polymerase activity // inferred from sequence or structural similarity /// 00055

notation /// 0046872 // metal ion binding // inferred from electronic annotation
notation /// 0046872 // metal ion binding // inferred from electronic annotation

rred from electronic annotation /// 0048407 // platelet-derived growth factor binding // not recorded /// 0048

n binding // inferred from electronic annotation /// 0016787 // hydrolase activity // inferred from electronic an

: annotation /// 0005515 // protein binding // inferred from physical interaction /// 0005515 // protein binding ,
annotation /// 0042826 // histone deacetylase binding // not recorded /// 0042826 // histone deacetylase bind

ion /// 0005515 // protein binding // inferred from electronic annotation /// 0017166 // vinculin binding // infer

of oxygen // inferred from electronic annotation /// 0020037 // heme binding // inferred from electronic anno

binding // inferred from electronic annotation /// 0019904 // protein domain specific binding // not recorded /
n

electronic annotation

activity // inferred from electronic annotation

activity // inferred from electronic annotation

otation

onic annotation
hyde dehydrogenase activity // inferred from electronic annotation /// 0042803 // protein homodimerization ac

omain specific binding // not recorded
al ion binding // inferred from electronic annotation
3 // protein dimerization activity // inferred from electronic annotation

sequence-specific DNA binding // inferred from electronic annotation /// 0046983 // protein dimerization activi

// protein binding // inferred from physical interaction /// 0008270 // zinc ion binding // inferred from electroni

ualene synthase activity // inferred from electronic annotation

from electronic annotation /// 0016853 // isomerase activity // inferred from electronic annotation

tructural similarity /// 0005515 // protein binding // inferred from electronic annotation /// 0008233 // peptida:

inding // not recorded
4872 // receptor activity // inferred from electronic annotation /// 0005515 // protein binding // inferred from

l from electronic annotation /// 0016874 // ligase activity // inferred from electronic annotation /// 0019787 //

' inferred from physical interaction /// 0005515 // protein binding // inferred from electronic annotation /// 000
nic annotation /// 0005080 // protein kinase C binding // inferred from electronic annotation /// 0005524 // AT

minephosphotransferase activity // inferred from sequence or structural similarity /// 0008963 // phospho-N-a
ce or structural similarity /// 0016787 // hydrolase activity // inferred from electronic annotation /// 0047498 /

red from electronic annotation
activity // inferred from electronic annotation /// 0004872 // receptor activity // inferred from electronic annota

from direct assay /// 0015923 // mannosidase activity // inferred from electronic annotation /// 0016787 // hy

tein ligase activity // inferred from electronic annotation

rred from electronic annotation /// 0030528 // transcription regulator activity // inferred from direct assay /// (

n electronic annotation /// 0046872 // metal ion binding // inferred from electronic annotation

ed from electronic annotation /// 0005524 // ATP binding // inferred from electronic annotation /// 0016787 //

inferred from electronic annotation /// 0030331 // estrogen receptor binding // inferred from electronic annota

e /// 0019900 // kinase binding // not recorded /// 0019900 // kinase binding // inferred from electronic annota
ronic annotation

// inferred from electronic annotation /// 0015349 // thyroid hormone transmembrane transporter activity // r

elective glutamate receptor activity // not recorded /// 0004972 // N-methyl-D-aspartate selective glutamate re

)005524 // ATP binding // inferred from electronic annotation /// 0016301 // kinase activity // inferred from ele

nall conductance calcium-activated potassium channel activity // inferred from electronic annotation
yl groups // inferred from electronic annotation
lulin binding // non-traceable author statement /// 0005516 // calmodulin binding // inferred from electronic ar

ı /// 0005515 // protein binding // inferred from physical interaction /// 0005515 // protein binding // inferred

y // inferred from electronic annotation /// 0016798 // hydrolase activity, acting on glycosyl bonds // inferred fr
4 // ATP binding // inferred from electronic annotation /// 0016301 // kinase activity // inferred from electronic
/ 0016740 // transferase activity // inferred from electronic annotation

nnotation /// 0008237 // metallopeptidase activity // inferred from mutant phenotype /// 0008270 // zinc ion b

ion /// 0046872 // metal ion binding // inferred from electronic annotation

om electronic annotation /// 0016740 // transferase activity // inferred from electronic annotation /// 0030274
om direct assay

)2 // identical protein binding // inferred from electronic annotation

'// 0003706 // ligand-regulated transcription factor activity // not recorded /// 0003706 // ligand-regulated tran
:ed from electronic annotation /// 0004674 // protein serine/threonine kinase activity // inferred from sequenc
in binding // not recorded /// 0043236 // laminin binding // inferred from electronic annotation /// 0046982 //

:tronic annotation /// 0004872 // receptor activity // inferred from electronic annotation /// 0005515 // protein

)046961 // proton-transporting ATPase activity, rotational mechanism // inferred from electronic annotation

ligase activity // inferred from electronic annotation
: annotation

ferase activity // inferred from electronic annotation

tivity // inferred from direct assay /// 0042826 // histone deacetylase binding // inferred from physical interacti

A binding // inferred from electronic annotation
red from electronic annotation /// 0030628 // pre-mRNA 3'-splice site binding // inferred from sequence or stru
ansferase activity // not recorded /// 0016262 // protein N-acetylglucosaminyltransferase activity // inferred fro
not recorded /// 0042282 // hydroxymethylglutaryl-CoA reductase activity // inferred from electronic annotatic

onic annotation /// 0003960 // NADPH:quinone reductase activity // inferred from sequence or structural simila

72 // metal ion binding // inferred from electronic annotation

/// 0016740 // transferase activity // inferred from electronic annotation /// 0016757 // transferase activity, tra

onic annotation

g // inferred from physical interaction /// 0005515 // protein binding // inferred from electronic annotation ///

from electronic annotation

homodimerization activity // not recorded

tation /// 0016702 // oxidoreductase activity, acting on single donors with incorporation of molecular oxygen, ir
annotation /// 0004872 // receptor activity // inferred from electronic annotation /// 0005003 // ephrin recepto

nferred from electronic annotation /// 0016787 // hydrolase activity // inferred from electronic annotation /// 0

tronic annotation /// 0005543 // phospholipid binding // not recorded /// 0005543 // phospholipid binding // in
ion /// 0004872 // receptor activity // inferred from electronic annotation /// 0005020 // stem cell factor recep
annotation /// 0008270 // zinc ion binding // inferred from electronic annotation /// 0016787 // hydrolase activ

rotase activity // not recorded /// 0004672 // protein kinase activity // not recorded /// 0005524 // ATP binding

/ inferred from electronic annotation /// 0008353 // RNA polymerase II carboxy-terminal domain kinase activity

// inferred from electronic annotation /// 0016740 // transferase activity // inferred from electronic annotation

ohol group as acceptor // inferred from electronic annotation /// 0032403 // protein complex binding // not rec

03727 // single-stranded RNA binding // not recorded /// 0003727 // single-stranded RNA binding // inferred fro
osphoprotein phosphatase activity // inferred from electronic annotation /// 0004722 // protein serine/threoni

ligase activity // inferred from electronic annotation

rred from physical interaction

lectronic annotation /// 0016301 // kinase activity // inferred from electronic annotation /// 0016740 // transfe

annotation /// 0008270 // zinc ion binding // inferred from electronic annotation /// 0016787 // hydrolase activ

// protein binding // inferred from electronic annotation /// 0005529 // sugar binding // inferred from electron

40 // transferase activity // inferred from electronic annotation /// 0047756 // chondroitin 4-sulfotransferase a
:tivity // inferred from electronic annotation /// 0046872 // metal ion binding // inferred from electronic annota

aride binding // inferred from electronic annotation /// 0046872 // metal ion binding // inferred from electroni

electronic annotation
erred from direct assay /// 0030528 // transcription regulator activity // inferred from electronic annotation ///
ding // inferred from electronic annotation /// 0048244 // phytanoyl-CoA dioxygenase activity // not recorded /
24 // ATP binding // inferred from electronic annotation /// 0016301 // kinase activity // inferred from electroni

notation /// 0030170 // pyridoxal phosphate binding // inferred from electronic annotation
:tivity // inferred from electronic annotation

somerase activity // inferred from electronic annotation

inase activity // inferred from electronic annotation /// 0004871 // signal transducer activity // inferred from di

n binding // inferred from electronic annotation /// 0016787 // hydrolase activity // inferred from electronic an

: annotation /// 0004931 // extracellular ATP-gated cation channel activity // inferred from mutant phenotype /
3713 // transcription coactivator activity // inferred from direct assay /// 0004871 // signal transducer activity /

ctronic annotation

r activity // inferred from electronic annotation /// 0043565 // sequence-specific DNA binding // inferred from

tivity, acting on single donors with incorporation of molecular oxygen, incorporation of two atoms of oxygen //

or activity // inferred from direct assay /// 0046872 // metal ion binding // inferred from electronic annotation

)004741 // [pyruvate dehydrogenase (lipoamide)] phosphatase activity // not recorded /// 0004741 // [pyruvate

rom electronic annotation /// 0005515 // protein binding // inferred from electronic annotation /// 0005524 //
rom electronic annotation /// 0005515 // protein binding // inferred from electronic annotation /// 0005524 //
onic annotation

ıic annotation /// 0016303 // 1-phosphatidylinositol-3-kinase activity // inferred from direct assay /// 0016303 /

traceable author statement /// 0004679 // AMP-activated protein kinase activity // inferred from electronic an

'/ 0016668 // oxidoreductase activity, acting on a sulfur group of donors, NAD or NADP as acceptor // inferred f

)005515 // protein binding // inferred from physical interaction /// 0008270 // zinc ion binding // inferred from

electronic annotation /// 0046872 // metal ion binding // inferred from electronic annotation

51019 // mitogen-activated protein kinase binding // not recorded

histone-lysine N-methyltransferase activity // inferred from electronic annotation /// 0019899 // enzyme bindi

ling // inferred from direct assay /// 0019904 // protein domain specific binding // not recorded /// 0019904 //

or activity // inferred from sequence or structural similarity /// 0008134 // transcription factor binding // inferre

803 // protein homodimerization activity // inferred from electronic annotation /// 0042803 // protein homodi

ronic annotation

protein domain specific binding // inferred from electronic annotation /// 0043565 // sequence-specific DNA bi

ng // inferred from physical interaction /// 0005515 // protein binding // inferred from electronic annotation ///

lucer activity // inferred from electronic annotation /// 0004872 // receptor activity // inferred from electronic a
uctural similarity

/ inferred from electronic annotation /// 0005524 // ATP binding // inferred from electronic annotation /// 001

osphotransferase activity, alcohol group as acceptor // inferred from electronic annotation /// 0019899 // enzy

dependent nuclear receptor activity // inferred from electronic annotation /// 0005496 // steroid binding // infe
activity // inferred from electronic annotation /// 0005515 // protein binding // inferred from physical interact
inase activity // inferred from electronic annotation /// 0004871 // signal transducer activity // inferred from di

annotation

ctronic annotation

activity // inferred from direct assay /// 0042803 // protein homodimerization activity // inferred from electror

otein binding // inferred from physical interaction /// 0005524 // ATP binding // inferred from electronic annota

)005524 // ATP binding // inferred from electronic annotation /// 0016301 // kinase activity // inferred from ele

// oxidoreductase activity // inferred from electronic annotation /// 0020037 // heme binding // inferred from e

ıc ion binding // inferred from electronic annotation /// 0016787 // hydrolase activity // inferred from electroni

ɔn

ıse II transcription factor activity // inferred from sequence or structural similarity /// 0003705 // sequence-spec

lectronic annotation /// 0008270 // zinc ion binding // not recorded /// 0008270 // zinc ion binding // inferred f

sequence or structural similarity /// 0042608 // T cell receptor binding // inferred from electronic annotation //

0008270 // zinc ion binding // inferred from electronic annotation /// 0016787 // hydrolase activity // inferred f

ic annotation
l from electronic annotation

se activity // not recorded /// 0016291 // acyl-CoA thioesterase activity // inferred from electronic annotation /
/// 0035226 // glutamate-cysteine ligase catalytic subunit binding // not recorded /// 0035226 // glutamate-cys

// transferase activity // inferred from electronic annotation
histone-lysine N-methyltransferase activity // inferred from electronic annotation /// 0019899 // enzyme bindi

inding // not recorded

red from electronic annotation

tivity // inferred from electronic annotation /// 0016878 // acid-thiol ligase activity // inferred from sequence o

ıg // inferred from physical interaction /// 0005515 // protein binding // inferred from electronic annotation ///
0005244 // voltage-gated ion channel activity // inferred from electronic annotation /// 0005249 // voltage-gat

/// 0016787 // hydrolase activity // inferred from electronic annotation

red from electronic annotation

// 0042802 // identical protein binding // inferred from electronic annotation
m electronic annotation /// 0017111 // nucleoside-triphosphatase activity // inferred from electronic annotatio

onic annotation /// 0005267 // potassium channel activity // inferred from electronic annotation /// 0005272 //

ne phosphatase activity // inferred from sequence or structural similarity /// 0005134 // interleukin-2 receptor l

rom electronic annotation /// 0005245 // voltage-gated calcium channel activity // inferred from direct assay //

ty /// 0008134 // transcription factor binding // inferred from electronic annotation /// 0008134 // transcriptior

003678 // DNA helicase activity // inferred from sequence or structural similarity /// 0003682 // chromatin binc

: annotation

nding // inferred from electronic annotation

erred from electronic annotation /// 0046872 // metal ion binding // inferred from electronic annotation

disulfide as acceptor // inferred from electronic annotation /// 0030976 // thiamin pyrophosphate binding // in

ıtion

/ 0046872 // metal ion binding // inferred from electronic annotation

om electronic annotation

onic annotation

m electronic annotation /// 0005515 // protein binding // inferred from physical interaction /// 0005515 // pro

022 // protein C-terminus binding // inferred from electronic annotation /// 0016874 // ligase activity // inferrec

hreonine racemase activity // inferred from direct assay /// 0030165 // PDZ domain binding // inferred from ele

nferred from electronic annotation /// 0016300 // tRNA (uracil) methyltransferase activity // inferred from sequ

tion factor activity // not recorded /// 0003700 // sequence-specific DNA binding transcription factor activity //

on /// 0003700 // sequence-specific DNA binding transcription factor activity // inferred from direct assay /// 00
inferred from physical interaction /// 0005515 // protein binding // inferred from electronic annotation /// 000

onic annotation
inferred from direct assay /// 0005524 // ATP binding // inferred from electronic annotation /// 0016301 // kin

from direct assay /// 0018024 // histone-lysine N-methyltransferase activity // inferred from electronic annotat

ation /// 0016301 // kinase activity // inferred from electronic annotation /// 0016740 // transferase activity //

ligase activity // inferred from electronic annotation /// 0042393 // histone binding // inferred from electronic a

5515 // protein binding // inferred from physical interaction /// 0016563 // transcription activator activity // no

d from electronic annotation /// 0017046 // peptide hormone binding // not recorded /// 0017046 // peptide h

activity // inferred from direct assay /// 0043565 // sequence-specific DNA binding // inferred from direct assay

/ 0046872 // metal ion binding // inferred from electronic annotation

adenylate cyclase activity // not recorded /// 0016829 // lyase activity // inferred from electronic annotation //

ic annotation /// 0016874 // ligase activity // inferred from electronic annotation /// 0042803 // protein homod

lrolase activity // inferred from electronic annotation /// 0016791 // phosphatase activity // inferred from electr
091 // phosphoinositide binding // inferred from sequence or structural similarity

om electronic annotation /// 0005125 // cytokine activity // inferred from direct assay /// 0005515 // protein bir
ı /// 0016887 // ATPase activity // inferred from sequence or structural similarity /// 0017137 // Rab GTPase bir

or activity // inferred from electronic annotation

ferase activity // inferred from electronic annotation

rred from electronic annotation /// 0046872 // metal ion binding // inferred from electronic annotation
0016787 // hydrolase activity // inferred from electronic annotation /// 0030160 // GKAP/Homer scaffold activi
d from electronic annotation /// 0003707 // steroid hormone receptor activity // traceable author statement ///

71 // signal transducer activity // inferred from electronic annotation /// 0004872 // receptor activity // inferrec

ating) activity // inferred from electronic annotation /// 0046872 // metal ion binding // inferred from electronic

nding // inferred from electronic annotation /// 0051864 // histone demethylase activity (H3-K36 specific) // inf

y // inferred from electronic annotation /// 0030552 // cAMP binding // not recorded /// 0030552 // cAMP binc

om electronic annotation /// 0046872 // metal ion binding // inferred from electronic annotation

nilarity /// 0003993 // acid phosphatase activity // inferred from electronic annotation /// 0005524 // ATP bindi
dependent nuclear receptor activity // inferred from electronic annotation /// 0005496 // steroid binding // infe

ı sequence or structural similarity
otation /// 0016564 // transcription repressor activity // inferred from direct assay /// 0016616 // oxidoreducta

:ronic annotation

ed from electronic annotation /// 0042162 // telomeric DNA binding // inferred from electronic annotation /// 0

TP binding // inferred from electronic annotation /// 0016155 // formyltetrahydrofolate dehydrogenase activity

ence or structural similarity /// 0043130 // ubiquitin binding // inferred from electronic annotation /// 004313

from electronic annotation /// 0046872 // metal ion binding // inferred from electronic annotation

/// 0003707 // steroid hormone receptor activity // inferred from electronic annotation /// 0004872 // recepto

nnotation /// 0005524 // ATP binding // inferred from electronic annotation /// 0008168 // methyltransferase a

nnotation /// 0010008 // endosome membrane // inferred from electronic annotation /// 0016020 // membrar
nnotation /// 0010008 // endosome membrane // inferred from electronic annotation /// 0016020 // membrar

modimerization activity // inferred from physical interaction

/ insulin receptor binding // inferred from electronic annotation /// 0005524 // ATP binding // inferred from ele

407 // platelet-derived growth factor binding // inferred from electronic annotation
epressor activity // inferred from direct assay /// 0016564 // transcription repressor activity // not recorded ///
P binding // inferred from electronic annotation /// 0016301 // kinase activity // inferred from electronic annot
P binding // inferred from electronic annotation /// 0016301 // kinase activity // inferred from electronic annot

0005262 // calcium channel activity // inferred from electronic annotation /// 0005524 // ATP binding // inferre

from electronic annotation /// 0005524 // ATP binding // inferred from electronic annotation /// 0016301 // kir

887 // thyroid hormone receptor activity // inferred from electronic annotation /// 0008270 // zinc ion binding
887 // thyroid hormone receptor activity // inferred from electronic annotation /// 0008270 // zinc ion binding

ity // inferred from direct assay /// 0015189 // L-lysine transmembrane transporter activity // not recorded ///

/// 0045505 // dynein intermediate chain binding // inferred from electronic annotation /// 0051219 // phosph

ation /// 0004930 // G-protein coupled receptor activity // inferred from electronic annotation /// 0005543 // p
factor beta-receptor activity // inferred from electronic annotation /// 0005021 // vascular endothelial growth

xygen binding // not recorded /// 0019825 // oxygen binding // inferred from electronic annotation /// 003014

nnotation /// 0032403 // protein complex binding // not recorded /// 0032403 // protein complex binding // inf
' oxidoreductase activity, acting on single donors with incorporation of molecular oxygen, incorporation of two a

tation /// 0005524 // ATP binding // inferred from direct assay /// 0005524 // ATP binding // inferred from elec

ot recorded /// 0046982 // protein heterodimerization activity // inferred from electronic annotation /// 00504

n electronic annotation /// 0005516 // calmodulin binding // inferred from electronic annotation /// 0005524 /
tion

onic annotation /// 0004715 // non-membrane spanning protein tyrosine kinase activity // inferred from electro

lrug binding // inferred from electronic annotation /// 0016301 // kinase activity // not recorded /// 0016301 //

n

n electronic annotation /// 0034736 // cholesterol O-acyltransferase activity // inferred from electronic annota
n electronic annotation /// 0034736 // cholesterol O-acyltransferase activity // inferred from electronic annota

nnotation /// 0046982 // protein heterodimerization activity // inferred from electronic annotation /// 005050

physical interaction /// 0008092 // cytoskeletal protein binding // inferred from direct assay /// 0008270 // zinc
om electronic annotation /// 0005496 // steroid binding // inferred from electronic annotation /// 0005515 // p

491 // oxidoreductase activity // inferred from electronic annotation

ransporter activity // inferred from electronic annotation /// 0015101 // organic cation transmembrane transpo

nce-specific DNA binding // inferred from electronic annotation
erred from electronic annotation /// 0046872 // metal ion binding // inferred from electronic annotation

le-triphosphatase activity // inferred from electronic annotation /// 0046872 // metal ion binding // inferred fro

otation /// 0016301 // kinase activity // inferred from electronic annotation /// 0016740 // transferase activity /

// 0030544 // Hsp70 protein binding // inferred from electronic annotation /// 0042393 // histone binding // inf

A vasopressin receptor binding // inferred from electronic annotation
A vasopressin receptor binding // inferred from electronic annotation

ion /// 0008134 // transcription factor binding // not recorded /// 0008415 // acyltransferase activity // inferred
ion /// 0008134 // transcription factor binding // not recorded /// 0008415 // acyltransferase activity // inferred

d from direct assay

protein binding // inferred from electronic annotation /// 0005524 // ATP binding // not recorded /// 0005524 /
l /// 0008134 // transcription factor binding // inferred from electronic annotation /// 0016563 // transcription

/// 0005524 // ATP binding // inferred from electronic annotation /// 0016301 // kinase activity // inferred from

on /// 0016491 // oxidoreductase activity // inferred from electronic annotation /// 0016712 // oxidoreductase a
on /// 0016491 // oxidoreductase activity // inferred from electronic annotation /// 0016712 // oxidoreductase a

nic annotation /// 0050699 // WW domain binding // inferred from physical interaction /// 0050699 // WW dom

ectronic annotation /// 0005515 // protein binding // inferred from physical interaction /// 0005515 // protein b

ded /// 0016740 // transferase activity // inferred from electronic annotation /// 0016757 // transferase activity

// inferred from electronic annotation
vity // inferred from electronic annotation /// 0016740 // transferase activity // inferred from electronic annota
kin-12 alpha subunit binding // inferred from electronic annotation /// 0042803 // protein homodimerization a

nnotation /// 0005524 // ATP binding // non-traceable author statement /// 0005524 // ATP binding // inferred

cation binding // inferred from electronic annotation

ction /// 0005515 // protein binding // inferred from physical interaction /// 0016563 // transcription activator a
// binding // inferred from electronic annotation /// 0005515 // protein binding // inferred from physical intera

e /// 0034502 // protein localization to chromosome // inferred from mutant phenotype /// 0048873 // homec

ing // not recorded /// 0032403 // protein complex binding // inferred from electronic annotation /// 0045296 /

ronic annotation /// 0016787 // hydrolase activity // inferred from electronic annotation /// 0046872 // metal ic

on /// 0043565 // sequence-specific DNA binding // inferred from direct assay /// 0043565 // sequence-specific

rom electronic annotation
rom electronic annotation

// transcription repressor activity // not recorded /// 0016564 // transcription repressor activity // inferred from
' ciliary neurotrophic factor receptor binding // inferred from electronic annotation /// 0005138 // interleukin-6

scriptional repressor activity // inferred from direct assay /// 0043565 // sequence-specific DNA binding // infer

rolase activity // inferred from electronic annotation /// 0016887 // ATPase activity // inferred from electronic a

6872 // metal ion binding // inferred from electronic annotation

ctronic annotation

nic annotation /// 0019205 // nucleobase, nucleoside, nucleotide kinase activity // inferred from electronic ann
51 // quaternary ammonium group transmembrane transporter activity // inferred from direct assay /// 001565

005515 // protein binding // inferred from physical interaction

e or structural similarity /// 0005515 // protein binding // inferred from physical interaction /// 0005515 // prot

inferred from electronic annotation

irect assay /// 0016740 // transferase activity // inferred from electronic annotation /// 0042826 // histone dea

5515 // protein binding // inferred from physical interaction /// 0005515 // protein binding // inferred from elec

ronic annotation

0050431 // transforming growth factor beta binding // not recorded /// 0051636 // Gram-negative bacterial cell

DNA bending activity // inferred from electronic annotation /// 0016564 // transcription repressor activity // in

gated calcium channel activity // inferred from electronic annotation

tation /// 0046872 // metal ion binding // inferred from electronic annotation

FAD or FADH2 binding // inferred from electronic annotation

tronic annotation /// 0005515 // protein binding // inferred from physical interaction /// 0031702 // type 1 angi
5 // protein binding // inferred from electronic annotation /// 0005524 // ATP binding // inferred from electron

lar oxygen to two molecules of water // inferred from electronic annotation /// 0020037 // heme binding // infe

ronic annotation /// 0008270 // zinc ion binding // inferred from electronic annotation /// 0016787 // hydrolase
ronic annotation /// 0008270 // zinc ion binding // inferred from electronic annotation /// 0016787 // hydrolase

C-terminal methylesterase activity // inferred from electronic annotation /// 0051722 // protein C-terminal me

ctivity // not recorded /// 0005007 // fibroblast growth factor receptor activity // inferred from electronic anno

'// 0005515 // protein binding // inferred from electronic annotation /// 0005524 // ATP binding // inferred from

protein complex binding // not recorded /// 0048037 // cofactor binding // inferred from electronic annotation

nnotation

0016740 // transferase activity // inferred from electronic annotation /// 0042277 // peptide binding // not rec

rect assay /// 0004872 // receptor activity // inferred from electronic annotation /// 0004888 // transmembran

n

rom direct assay /// 0005003 // ephrin receptor activity // non-traceable author statement /// 0005003 // ephri
tronic annotation /// 0008097 // 5S rRNA binding // not recorded /// 0008201 // heparin binding // not recorde

d from electronic annotation /// 0005515 // protein binding // inferred from physical interaction /// 0008134 //

om electronic annotation /// 0005515 // protein binding // inferred from physical interaction /// 0008270 // zin

ɔm electronic annotation /// 0016740 // transferase activity // inferred from electronic annotation /// 0046872

electronic annotation /// 0005515 // protein binding // inferred from physical interaction /// 0005524 // ATP bi
:ation binding // inferred from electronic annotation

'

ronic annotation /// 0033549 // MAP kinase phosphatase activity // inferred from electronic annotation

ɔhysical interaction /// 0005515 // protein binding // inferred from electronic annotation /// 0005524 // ATP bin

ɔtation

) // transferase activity // inferred from electronic annotation /// 0016779 // nucleotidyltransferase activity // ir

:tronic annotation /// 0017069 // snRNA binding // inferred from sequence or structural similarity

annotation /// 0008415 // acyltransferase activity // inferred from electronic annotation /// 0016740 // transfer

' inferred from electronic annotation /// 0016787 // hydrolase activity // inferred from electronic annotation //,

tronic annotation /// 0033814 // propanoyl-CoA C-acyltransferase activity // inferred from electronic annotatio

se binding // inferred from electronic annotation /// 0030295 // protein kinase activator activity // not recordec

annotation
ity // inferred from direct assay /// 0019789 // SUMO ligase activity // inferred from electronic annotation /// 0
ctronic annotation /// 0031430 // M band // inferred from electronic annotation /// 0042383 // sarcolemma //
ecorded /// 0046872 // metal ion binding // inferred from electronic annotation
3270 // zinc ion binding // inferred from electronic annotation /// 0043565 // sequence-specific DNA binding // i

onic annotation /// 0051287 // NAD or NADH binding // inferred from electronic annotation

inferred from electronic annotation /// 0016301 // kinase activity // inferred from electronic annotation /// 00

binding // inferred from sequence or structural similarity /// 0008270 // zinc ion binding // inferred from electro

50750 // low-density lipoprotein receptor binding // inferred from electronic annotation

ding // inferred from electronic annotation /// 0008528 // peptide receptor activity, G-protein coupled // inferre
tronic annotation

notation /// 0031683 // G-protein beta/gamma-subunit binding // not recorded /// 0031683 // G-protein beta/

/// 0008144 // drug binding // inferred from electronic annotation /// 0015277 // kainate selective glutamate r
binding // inferred from electronic annotation /// 0005524 // ATP binding // inferred from sequence or structura

annotation

erred from electronic annotation /// 0034513 // box H/ACA snoRNA binding // not recorded /// 0034513 // box

ed from electronic annotation /// 0031702 // type 1 angiotensin receptor binding // not recorded /// 0031702 /
FAD or FADH2 binding // inferred from electronic annotation

hate group as acceptor // inferred from electronic annotation /// 0019201 // nucleotide kinase activity // inferr

ectronic annotation /// 0042803 // protein homodimerization activity // not recorded /// 0042803 // protein hor

nplex binding // inferred from electronic annotation /// 0051087 // chaperone binding // not recorded

515 // protein binding // inferred from electronic annotation /// 0008017 // microtubule binding // not recordec

407 // platelet-derived growth factor binding // inferred from electronic annotation

notation /// 0046872 // metal ion binding // inferred from electronic annotation

// inferred from electronic annotation /// 0005524 // ATP binding // not recorded /// 0005524 // ATP binding //
ing // inferred from electronic annotation /// 0046872 // metal ion binding // inferred from electronic annotati

rred from electronic annotation /// 0030274 // LIM domain binding // inferred from electronic annotation /// 00

tation /// 0046872 // metal ion binding // inferred from electronic annotation

// 0030544 // Hsp70 protein binding // inferred from electronic annotation /// 0042393 // histone binding // inf

ctivity // not recorded /// 0042803 // protein homodimerization activity // inferred from electronic annotation /

ity // inferred from electronic annotation

ic annotation /// 0010843 // promoter binding // inferred from direct assay /// 0016563 // transcription activat

se activity // inferred from electronic annotation /// 0008234 // cysteine-type peptidase activity // inferred fron

physical interaction /// 0005515 // protein binding // inferred from electronic annotation /// 0005524 // ATP bi

small conjugating protein ligase activity // inferred from electronic annotation

)5524 // ATP binding // inferred from electronic annotation /// 0016301 // kinase activity // inferred from electr
'P binding // inferred from electronic annotation /// 0008022 // protein C-terminus binding // inferred from ele

cetylmuramoyl-pentapeptide-transferase activity // inferred from electronic annotation /// 0016740 // transfer
/ calcium-dependent phospholipase A2 activity // not recorded /// 0047498 // calcium-dependent phospholipas

ation /// 0004930 // G-protein coupled receptor activity // inferred from electronic annotation /// 0005543 // p

drolase activity // inferred from electronic annotation /// 0016798 // hydrolase activity, acting on glycosyl bond

)031625 // ubiquitin protein ligase binding // inferred from electronic annotation /// 0042803 // protein homod

hydrolase activity // inferred from electronic annotation /// 0016887 // ATPase activity // inferred from electro

ation /// 0035257 // nuclear hormone receptor binding // inferred from electronic annotation /// 0035259 // gl

ation /// 0019905 // syntaxin binding // not recorded /// 0019905 // syntaxin binding // inferred from electroni

ıot recorded /// 0015349 // thyroid hormone transmembrane transporter activity // inferred from electronic an

ɛceptor activity // inferred from electronic annotation /// 0005215 // transporter activity // inferred from electr

ɛctronic annotation /// 0016740 // transferase activity // inferred from electronic annotation

ınotation /// 0005524 // ATP binding // non-traceable author statement /// 0005524 // ATP binding // inferred

from electronic annotation /// 0005524 // ATP binding // inferred from electronic annotation /// 0016301 // kir

rom electronic annotation
annotation /// 0016740 // transferase activity // inferred from electronic annotation /// 0017124 // SH3 domai

binding // inferred from electronic annotation /// 0016787 // hydrolase activity // inferred from electronic anno

// LIM domain binding // inferred from electronic annotation /// 0050700 // CARD domain binding // not recor

ıscription factor activity // inferred from electronic annotation /// 0005160 // transforming growth factor beta r
e or structural similarity /// 0005515 // protein binding // inferred from physical interaction /// 0005515 // prot
protein heterodimerization activity // not recorded /// 0046982 // protein heterodimerization activity // inferre

binding // inferred from physical interaction /// 0005515 // protein binding // inferred from electronic annotat

on /// 0043565 // sequence-specific DNA binding // inferred from direct assay /// 0043565 // sequence-specific

uctural similarity /// 0046872 // metal ion binding // inferred from electronic annotation
om electronic annotation /// 0016740 // transferase activity // inferred from electronic annotation /// 0016757
on /// 0042803 // protein homodimerization activity // not recorded /// 0042803 // protein homodimerization a

rity /// 0005488 // binding // inferred from electronic annotation /// 0008270 // zinc ion binding // inferred fron

ansferring glycosyl groups // inferred from electronic annotation

0016563 // transcription activator activity // not recorded /// 0016563 // transcription activator activity // infe

ncorporation of two atoms of oxygen // inferred from electronic annotation /// 0046872 // metal ion binding //
or activity // inferred from electronic annotation /// 0005515 // protein binding // inferred from electronic anno

016820 // hydrolase activity, acting on acid anhydrides, catalyzing transmembrane movement of substances //

nferred from electronic annotation /// 0008081 // phosphoric diester hydrolase activity // inferred from electror
tor activity // inferred from direct assay /// 0005515 // protein binding // inferred from physical interaction ///
vity // inferred from electronic annotation /// 0046872 // metal ion binding // inferred from electronic annotatic

g // not recorded /// 0005524 // ATP binding // inferred from electronic annotation /// 0016597 // amino acid b

// inferred from sequence or structural similarity

/// 0016773 // phosphotransferase activity, alcohol group as acceptor // inferred from electronic annotation //

corded /// 0032403 // protein complex binding // inferred from electronic annotation

om electronic annotation /// 0004872 // receptor activity // inferred from electronic annotation /// 0004879 // l
ne phosphatase activity // inferred from electronic annotation /// 0004722 // protein serine/threonine phospha

erase activity // inferred from electronic annotation /// 0046872 // metal ion binding // inferred from electronic

vity // inferred from electronic annotation /// 0046872 // metal ion binding // inferred from electronic annotatic

ic annotation /// 0016564 // transcription repressor activity // inferred from electronic annotation /// 0042277

ctivity // inferred from electronic annotation
ation

c annotation

0043565 // sequence-specific DNA binding // not recorded /// 0043565 // sequence-specific DNA binding // inf
// 0048244 // phytanoyl-CoA dioxygenase activity // inferred from electronic annotation
ic annotation /// 0016740 // transferase activity // inferred from electronic annotation /// 0046872 // metal ion

rect assay /// 0004872 // receptor activity // inferred from electronic annotation /// 0004888 // transmembrane

notation /// 0046872 // metal ion binding // inferred from electronic annotation

/// 0004931 // extracellular ATP-gated cation channel activity // not recorded /// 0004931 // extracellular ATP-g
/ inferred from electronic annotation /// 0004872 // receptor activity // inferred from electronic annotation ///

direct assay /// 0043565 // sequence-specific DNA binding // inferred from genetic interaction /// 0043565 // s[illegible]

inferred from electronic annotation /// 0043734 // DNA-N1-methyladenine dioxygenase activity // inferred fro[illegible]

[illegible] dehydrogenase (lipoamide)] phosphatase activity // inferred from electronic annotation /// 0016787 // hydrol[illegible]

ATP binding // inferred from electronic annotation /// 0016301 // kinase activity // inferred from electronic an[illegible]
ATP binding // inferred from electronic annotation /// 0016301 // kinase activity // inferred from electronic an[illegible]

'/ 1-phosphatidylinositol-3-kinase activity // inferred from electronic annotation /// 0016740 // transferase activ

notation /// 0005515 // protein binding // inferred from electronic annotation /// 0005524 // ATP binding // no

rom electronic annotation /// 0042803 // protein homodimerization activity // not recorded /// 0042803 // pro

electronic annotation /// 0046872 // metal ion binding // inferred from electronic annotation

ng // inferred from electronic annotation /// 0042800 // histone methyltransferase activity (H3-K4 specific) // in

protein domain specific binding // inferred from electronic annotation /// 0032947 // protein complex scaffold

ed from sequence or structural similarity /// 0008301 // DNA bending activity // inferred from sequence or struc

merization activity // inferred from sequence or structural similarity

nding // inferred from electronic annotation

0008168 // methyltransferase activity // inferred from electronic annotation /// 0008270 // zinc ion binding //

annotation /// 0005102 // receptor binding // inferred from direct assay /// 0005515 // protein binding // inferr

6874 // ligase activity // inferred from electronic annotation /// 0019787 // small conjugating protein ligase acti

me binding // not recorded /// 0019899 // enzyme binding // inferred from electronic annotation /// 0042562 /

erred from electronic annotation /// 0005515 // protein binding // inferred from physical interaction /// 000551
ion /// 0008134 // transcription factor binding // not recorded /// 0008415 // acyltransferase activity // inferre
rect assay /// 0004872 // receptor activity // inferred from electronic annotation /// 0004888 // transmembran

ıic annotation /// 0046982 // protein heterodimerization activity // inferred from direct assay /// 0046982 // pr

ation /// 0016301 // kinase activity // inferred from electronic annotation /// 0016740 // transferase activity // i

ctronic annotation /// 0016740 // transferase activity // inferred from electronic annotation

electronic annotation /// 0046872 // metal ion binding // inferred from electronic annotation /// 0046982 // pro

c annotation /// 0046872 // metal ion binding // inferred from electronic annotation

cific enhancer binding RNA polymerase II transcription factor activity // inferred from sequence or structural sim

from electronic annotation /// 0016740 // transferase activity // inferred from electronic annotation /// 001984

/ 0042608 // T cell receptor binding // inferred from sequence or structural similarity /// 0051020 // GTPase bir

from electronic annotation /// 0017046 // peptide hormone binding // not recorded /// 0046872 // metal ion bi

// 0016874 // ligase activity // inferred from electronic annotation
steine ligase catalytic subunit binding // inferred from electronic annotation /// 0035226 // glutamate-cysteine

ng // inferred from electronic annotation /// 0042800 // histone methyltransferase activity (H3-K4 specific) // in

r structural similarity /// 0031177 // phosphopantetheine binding // inferred from electronic annotation /// 004

' 0008168 // methyltransferase activity // inferred from electronic annotation /// 0008270 // zinc ion binding //
ed potassium channel activity // not recorded /// 0005249 // voltage-gated potassium channel activity // inferr

on /// 0046872 // metal ion binding // inferred from electronic annotation

' sodium channel activity // inferred from electronic annotation /// 0031402 // sodium ion binding // not record

binding // not recorded /// 0005134 // interleukin-2 receptor binding // inferred from electronic annotation ///

'/ 0005246 // calcium channel regulator activity // inferred from direct assay /// 0005516 // calmodulin binding

ı factor binding // inferred from sequence or structural similarity /// 0008656 // caspase activator activity // no

ding // inferred from electronic annotation /// 0004386 // helicase activity // inferred from electronic annotatio

ıferred from electronic annotation /// 0031072 // heat shock protein binding // not recorded /// 0034602 // oxc

tein binding // inferred from electronic annotation /// 0005524 // ATP binding // not recorded /// 0005524 // A[illegible]

[illegible] from electronic annotation /// 0019787 // small conjugating protein ligase activity // inferred from electronic [illegible]

ctronic annotation /// 0030170 // pyridoxal phosphate binding // inferred from mutant phenotype /// 0030170

ience or structural similarity /// 0016491 // oxidoreductase activity // inferred from electronic annotation /// 00

inferred from electronic annotation /// 0003707 // steroid hormone receptor activity // inferred from electroni

003700 // sequence-specific DNA binding transcription factor activity // inferred from mutant phenotype /// 000
5524 // ATP binding // inferred from electronic annotation /// 0008270 // zinc ion binding // inferred from elec

ase activity // inferred from electronic annotation /// 0016740 // transferase activity // inferred from electronic

tion /// 0046872 // metal ion binding // inferred from electronic annotation

inferred from electronic annotation /// 0046872 // metal ion binding // inferred from electronic annotation

annotation /// 0042393 // histone binding // inferred from sequence or structural similarity /// 0043130 // ubiq

ot recorded /// 0016563 // transcription activator activity // inferred from electronic annotation /// 0042803 // p

hormone binding // inferred from electronic annotation

/ /// 0046872 // metal ion binding // inferred from electronic annotation /// 0070087 // chromo shadow domain

/ 0016849 // phosphorus-oxygen lyase activity // inferred from electronic annotation /// 0046872 // metal ion b

limerization activity // inferred from electronic annotation

ronic annotation /// 0033549 // MAP kinase phosphatase activity // inferred from electronic annotation

nding // inferred from physical interaction
nding // inferred from electronic annotation

ity // inferred from direct assay /// 0035256 // metabotropic glutamate receptor binding // not recorded /// 003
/ 0003707 // steroid hormone receptor activity // inferred from electronic annotation /// 0004872 // receptor a

d from electronic annotation /// 0005515 // protein binding // inferred from physical interaction /// 0008134 //

: annotation /// 0051287 // NAD or NADH binding // inferred from electronic annotation

erred from electronic annotation

ding // inferred from electronic annotation /// 0030553 // cGMP binding // not recorded /// 0030553 // cGMP b

ng // inferred from electronic annotation /// 0016301 // kinase activity // inferred from electronic annotation //
erred from electronic annotation /// 0005515 // protein binding // inferred from physical interaction /// 000551

ıse activity, acting on the CH-OH group of donors, NAD or NADP as acceptor // inferred from electronic annotati

)042162 // telomeric DNA binding // inferred from sequence or structural similarity

// inferred from mutant phenotype /// 0016491 // oxidoreductase activity // inferred from electronic annotatio

0 // ubiquitin binding // inferred from sequence or structural similarity /// 0046966 // thyroid hormone recepto

ɔr activity // inferred from electronic annotation /// 0004879 // ligand-dependent nuclear receptor activity // in

ıctivity // inferred from electronic annotation /// 0016740 // transferase activity // inferred from electronic ann

ıe // inferred from electronic annotation /// 0016021 // integral to membrane // inferred from electronic annot
ıe // inferred from electronic annotation /// 0016021 // integral to membrane // inferred from electronic annot

ɛctronic annotation /// 0016301 // kinase activity // inferred from electronic annotation /// 0016740 // transfer

0016564 // transcription repressor activity // inferred from electronic annotation /// 0016787 // hydrolase activ
tation /// 0016740 // transferase activity // inferred from electronic annotation /// 0046872 // metal ion binding
tation /// 0016740 // transferase activity // inferred from electronic annotation /// 0046872 // metal ion binding

ɛd from electronic annotation /// 0016301 // kinase activity // inferred from direct assay /// 0016301 // kinase a

ıase activity // inferred from direct assay /// 0016301 // kinase activity // inferred from electronic annotation //

// inferred from electronic annotation /// 0043565 // sequence-specific DNA binding // inferred from electroni
// inferred from electronic annotation /// 0043565 // sequence-specific DNA binding // inferred from electroni

0015189 // L-lysine transmembrane transporter activity // inferred from electronic annotation /// 0015326 // c

ıoprotein binding // inferred from physical interaction

hospholipid binding // not recorded /// 0005543 // phospholipid binding // inferred from electronic annotation
factor receptor activity // inferred from electronic annotation /// 0005161 // platelet-derived growth factor rec

5 // manganese ion binding // not recorded /// 0030145 // manganese ion binding // inferred from electronic ar

ferred from electronic annotation /// 0043208 // glycosphingolipid binding // not recorded /// 0043208 // glyco
ıtoms of oxygen // inferred from electronic annotation /// 0020037 // heme binding // inferred from electronic

tronic annotation /// 0008017 // microtubule binding // inferred from direct assay /// 0016301 // kinase activity

31 // transforming growth factor beta binding // inferred from electronic annotation

/ ATP binding // not recorded /// 0005524 // ATP binding // inferred from electronic annotation /// 0016887 //

ɔnic annotation /// 0005102 // receptor binding // not recorded /// 0005515 // protein binding // inferred from

' kinase activity // inferred from electronic annotation /// 0016740 // transferase activity // inferred from electr

tion /// 0034737 // ergosterol O-acyltransferase activity // inferred from electronic annotation /// 0034738 // la
tion /// 0034737 // ergosterol O-acyltransferase activity // inferred from electronic annotation /// 0034738 // la

3 // glucuronosyl-N-acetylglucosaminyl-proteoglycan 4-alpha-N-acetylglucosaminyltransferase activity // inferre

ion binding // inferred from electronic annotation /// 0015271 // outward rectifier potassium channel activity /
rotein binding // inferred from electronic annotation /// 0005524 // ATP binding // inferred from electronic ann

rter activity // inferred from direct assay /// 0015101 // organic cation transmembrane transporter activity // n

m electronic annotation

// inferred from electronic annotation

ferred from direct assay /// 0042393 // histone binding // inferred from electronic annotation /// 0042802 // id

d from electronic annotation /// 0016740 // transferase activity // inferred from electronic annotation /// 0030
d from electronic annotation /// 0016740 // transferase activity // inferred from electronic annotation /// 0030

/ ATP binding // inferred from electronic annotation /// 0016301 // kinase activity // inferred from electronic a
activator activity // inferred from direct assay /// 0042803 // protein homodimerization activity // inferred from

n electronic annotation /// 0016740 // transferase activity // inferred from electronic annotation

activity, acting on paired donors, with incorporation or reduction of molecular oxygen, reduced flavin or flavopr
activity, acting on paired donors, with incorporation or reduction of molecular oxygen, reduced flavin or flavopr

nain binding // not recorded /// 0050699 // WW domain binding // inferred from electronic annotation

binding // inferred from electronic annotation /// 0005524 // ATP binding // not recorded /// 0005524 // ATP bi

, transferring glycosyl groups // inferred from electronic annotation /// 0043178 // alcohol binding // not record

ation /// 0070513 // death domain binding // inferred from electronic annotation
ctivity // inferred from direct assay /// 0046982 // protein heterodimerization activity // not recorded /// 0046

from electronic annotation /// 0005543 // phospholipid binding // traceable author statement /// 0008022 // p

ctivity // inferred from direct assay /// 0016566 // specific transcriptional repressor activity // inferred from dir
ction /// 0005515 // protein binding // inferred from electronic annotation /// 0016563 // transcription activat

›stasis of number of cells within a tissue // inferred from mutant phenotype

// cadherin binding // not recorded /// 0045296 // cadherin binding // inferred from electronic annotation

›n binding // inferred from electronic annotation /// 0070087 // chromo shadow domain binding // inferred fro

DNA binding // inferred from electronic annotation

› electronic annotation /// 0030528 // transcription regulator activity // inferred from electronic annotation ///
receptor binding // inferred from electronic annotation /// 0005515 // protein binding // inferred from physica

red from mutant phenotype /// 0046872 // metal ion binding // inferred from electronic annotation /// 004698

nnotation /// 0017111 // nucleoside-triphosphatase activity // inferred from electronic annotation /// 0034040

otation
1 // quaternary ammonium group transmembrane transporter activity // not recorded /// 0042895 // antibiotic

tein binding // inferred from electronic annotation /// 0005524 // ATP binding // inferred from electronic annot

cetylase binding // inferred from electronic annotation /// 0043997 // histone acetyltransferase activity (H4-K1[illegible]

ctronic annotation /// 0005524 // ATP binding // inferred from electronic annotation /// 0016301 // kinase activ

surface binding // inferred from direct assay /// 0051637 // Gram-positive bacterial cell surface binding // infer

ferred from direct assay /// 0016564 // transcription repressor activity // inferred from electronic annotation //

iotensin receptor binding // not recorded
ic annotation /// 0005524 // ATP binding // inferred from sequence or structural similarity /// 0016301 // kinas

erred from electronic annotation

e activity // inferred from electronic annotation /// 0046872 // metal ion binding // inferred from electronic ann
e activity // inferred from electronic annotation /// 0046872 // metal ion binding // inferred from electronic ann

ethylesterase activity // inferred from sequence or structural similarity

tation /// 0005515 // protein binding // inferred from physical interaction /// 0005515 // protein binding // infe

n direct assay /// 0005524 // ATP binding // inferred from electronic annotation /// 0016301 // kinase activity /,

orded /// 0051219 // phosphoprotein binding // not recorded

e receptor activity // not recorded /// 0004888 // transmembrane receptor activity // inferred from electronic a

n receptor activity // inferred from electronic annotation /// 0005005 // transmembrane-ephrin receptor activit
:d /// 0008201 // heparin binding // inferred from electronic annotation /// 0008301 // DNA bending activity //

transcription factor binding // not recorded /// 0017162 // aryl hydrocarbon receptor binding // inferred from e

c ion binding // inferred from electronic annotation /// 0008502 // melatonin receptor activity // not recorded /

// metal ion binding // inferred from electronic annotation

nding // inferred from electronic annotation /// 0016301 // kinase activity // inferred from electronic annotatio

nding // inferred from electronic annotation /// 0016301 // kinase activity // inferred from electronic annotation

nferred from electronic annotation /// 0016787 // hydrolase activity // inferred from electronic annotation /// 0

rase activity // inferred from electronic annotation /// 0019904 // protein domain specific binding // not record

/ 0030552 // cAMP binding // not recorded /// 0030552 // cAMP binding // inferred from electronic annotation

n /// 0050632 // propionyl-CoA C2-trimethyltridecanoyltransferase activity // traceable author statement

d /// 0030295 // protein kinase activator activity // inferred from electronic annotation

030331 // estrogen receptor binding // not recorded /// 0035259 // glucocorticoid receptor binding // not recor
' inferred from electronic annotation

inferred from electronic annotation /// 0046872 // metal ion binding // inferred from electronic annotation

16740 // transferase activity // inferred from electronic annotation

onic annotation /// 0016874 // ligase activity // inferred from electronic annotation /// 0019003 // GDP binding

ed from electronic annotation /// 0016829 // lyase activity // inferred from electronic annotation /// 0016849 //

gamma-subunit binding // inferred from electronic annotation /// 0032795 // heterotrimeric G-protein binding

receptor activity // inferred from direct assay /// 0015277 // kainate selective glutamate receptor activity // infe
al similarity /// 0016301 // kinase activity // inferred from electronic annotation /// 0016740 // transferase activ

H/ACA snoRNA binding // inferred from electronic annotation

'/ type 1 angiotensin receptor binding // inferred from electronic annotation /// 0031752 // D5 dopamine recep

ed from electronic annotation /// 0019205 // nucleobase, nucleoside, nucleotide kinase activity // inferred fron

modimerization activity // inferred from electronic annotation

d /// 0008017 // microtubule binding // inferred from electronic annotation /// 0016740 // transferase activity /

/ inferred from electronic annotation /// 0015077 // monovalent inorganic cation transmembrane transporter a
on /// 0046966 // thyroid hormone receptor binding // not recorded /// 0046966 // thyroid hormone receptor b

)32403 // protein complex binding // not recorded

ferred from direct assay /// 0042393 // histone binding // inferred from electronic annotation /// 0042802 // id

/// 0043176 // amine binding // not recorded /// 0043176 // amine binding // inferred from electronic annotati

or activity // inferred from electronic annotation /// 0043565 // sequence-specific DNA binding // inferred from

n electronic annotation /// 0016787 // hydrolase activity // inferred from electronic annotation /// 0042980 // c

nding // inferred from electronic annotation /// 0016301 // kinase activity // inferred from electronic annotatio

ronic annotation /// 0016740 // transferase activity // inferred from electronic annotation
ctronic annotation /// 0016853 // isomerase activity // inferred from electronic annotation /// 0042826 // histo

ase activity // inferred from electronic annotation /// 0016757 // transferase activity, transferring glycosyl grou
se A2 activity // inferred from electronic annotation

hospholipid binding // not recorded /// 0005543 // phospholipid binding // inferred from electronic annotation

ls // inferred from electronic annotation /// 0016799 // hydrolase activity, hydrolyzing N-glycosyl compounds //

limerization activity // inferred from electronic annotation /// 0046332 // SMAD binding // inferred from physic

nic annotation /// 0016887 // ATPase activity // inferred from sequence or structural similarity

ucocorticoid receptor binding // inferred from electronic annotation /// 0042826 // histone deacetylase binding

c annotation /// 0042802 // identical protein binding // not recorded /// 0042802 // identical protein binding //

notation

onic annotation /// 0005216 // ion channel activity // inferred from electronic annotation /// 0005234 // extrac

from electronic annotation /// 0005543 // phospholipid binding // traceable author statement /// 0008022 // p

nase activity // inferred from electronic annotation /// 0016740 // transferase activity // inferred from electroni

in binding // not recorded /// 0019899 // enzyme binding // inferred from electronic annotation /// 0019901 //

tation /// 0017124 // SH3 domain binding // inferred from electronic annotation /// 0019901 // protein kinase

rded /// 0050700 // CARD domain binding // inferred from electronic annotation

receptor binding // inferred from electronic annotation /// 0005515 // protein binding // inferred from physical
tein binding // inferred from electronic annotation /// 0005524 // ATP binding // inferred from electronic annota
ed from electronic annotation

tion /// 0005524 // ATP binding // inferred from electronic annotation /// 0016301 // kinase activity // inferred f

DNA binding // inferred from electronic annotation

// transferase activity, transferring glycosyl groups // inferred from electronic annotation /// 0042277 // peptid
ıctivity // inferred from electronic annotation /// 0050661 // NADP or NADPH binding // inferred from electroni

n electronic annotation /// 0016491 // oxidoreductase activity // inferred from electronic annotation /// 00506

rred from electronic annotation /// 0043565 // sequence-specific DNA binding // inferred from direct assay /// [illegible]

inferred from electronic annotation
ɔtation /// 0005524 // ATP binding // inferred from electronic annotation /// 0016301 // kinase activity // inferre

inferred from electronic annotation /// 0046872 // metal ion binding // inferred from electronic annotation

ıic annotation /// 0016787 // hydrolase activity // inferred from electronic annotation /// 0046872 // metal ion
0005515 // protein binding // inferred from electronic annotation /// 0005524 // ATP binding // inferred from e
ɔn

inding // inferred from electronic annotation /// 0016740 // transferase activity // inferred from electronic ann

/ 0046934 // phosphatidylinositol-4,5-bisphosphate 3-kinase activity // inferred from electronic annotation

igand-dependent nuclear receptor activity // inferred from direct assay /// 0004879 // ligand-dependent nuclea
atase activity // inferred from sequence or structural similarity /// 0004725 // protein tyrosine phosphatase acti

annotation

ɔn

// peptide binding // not recorded /// 0042562 // hormone binding // not recorded /// 0046872 // metal ion bi

ferred from electronic annotation

ı binding // inferred from electronic annotation

e receptor activity // not recorded /// 0004888 // transmembrane receptor activity // inferred from electronic a

gated cation channel activity // inferred from electronic annotation /// 0005102 // receptor binding // not recor
/ 0005515 // protein binding // inferred from physical interaction /// 0005515 // protein binding // inferred from

equence-specific DNA binding // inferred from electronic annotation /// 0046982 // protein heterodimerization

n electronic annotation /// 0043734 // DNA-N1-methyladenine dioxygenase activity // inferred from sequence

ase activity // inferred from electronic annotation /// 0046872 // metal ion binding // inferred from electronic a

notation /// 0016740 // transferase activity // inferred from electronic annotation /// 0017147 // Wnt-protein b
notation /// 0016740 // transferase activity // inferred from electronic annotation /// 0017147 // Wnt-protein b

vity // inferred from electronic annotation /// 0016773 // phosphotransferase activity, alcohol group as accepto

ot recorded /// 0005524 // ATP binding // inferred from electronic annotation /// 0016301 // kinase activity // ir

tein homodimerization activity // inferred from electronic annotation /// 0050660 // FAD or FADH2 binding // ir

ıferred from electronic annotation /// 0042800 // histone methyltransferase activity (H3-K4 specific) // inferred

// not recorded /// 0032947 // protein complex scaffold // inferred from electronic annotation /// 0034237 // p

:tural similarity /// 0010843 // promoter binding // inferred from sequence or structural similarity /// 0016563 /

inferred from electronic annotation /// 0016740 // transferase activity // inferred from electronic annotation //

ed from physical interaction /// 0016564 // transcription repressor activity // inferred from direct assay /// 003

ivity // inferred from electronic annotation /// 0043130 // ubiquitin binding // inferred from electronic annotati

/ hormone binding // not recorded /// 0042562 // hormone binding // inferred from electronic annotation

5 // protein binding // inferred from electronic annotation /// 0008134 // transcription factor binding // inferre
d from electronic annotation /// 0016740 // transferase activity // inferred from electronic annotation /// 0030
e receptor activity // not recorded /// 0004888 // transmembrane receptor activity // inferred from electronic a

otein heterodimerization activity // inferred from electronic annotation

inferred from electronic annotation

otein heterodimerization activity // inferred from electronic annotation /// 0050660 // FAD or FADH2 binding //

ıilarity /// 0008134 // transcription factor binding // inferred from sequence or structural similarity /// 0008301

0 // isoprenoid binding // not recorded /// 0019840 // isoprenoid binding // inferred from electronic annotation

ıding // inferred from electronic annotation

inding // inferred from electronic annotation

ligase catalytic subunit binding // inferred from sequence or structural similarity /// 0046982 // protein heterod

ıferred from electronic annotation /// 0042800 // histone methyltransferase activity (H3-K4 specific) // inferred

ı8037 // cofactor binding // inferred from electronic annotation

inferred from electronic annotation /// 0016740 // transferase activity // inferred from electronic annotation //
ed from electronic annotation /// 0005267 // potassium channel activity // inferred from electronic annotation

led /// 0031402 // sodium ion binding // inferred from electronic annotation

0005515 // protein binding // inferred from electronic annotation /// 0043021 // ribonucleoprotein binding // i

// not recorded /// 0016595 // glutamate binding // not recorded /// 0030165 // PDZ domain binding // not rec

t recorded /// 0019209 // kinase activator activity // inferred from electronic annotation /// 0019209 // kinase a

n /// 0005515 // protein binding // inferred from physical interaction /// 0005515 // protein binding // inferred

‹oglutarate dehydrogenase (NAD+) activity // inferred from direct assay /// 0051087 // chaperone binding // not

TP binding // inferred from electronic annotation /// 0016301 // kinase activity // inferred from electronic anno

annotation /// 0019789 // SUMO ligase activity // not recorded /// 0019789 // SUMO ligase activity // inferred f

) // pyridoxal phosphate binding // not recorded /// 0030170 // pyridoxal phosphate binding // inferred from ele

)16740 // transferase activity // inferred from electronic annotation /// 0046872 // metal ion binding // inferred

c annotation /// 0004872 // receptor activity // inferred from electronic annotation /// 0004879 // ligand-deper

)3700 // sequence-specific DNA binding transcription factor activity // inferred from electronic annotation /// 0
tronic annotation /// 0015459 // potassium channel regulator activity // not recorded /// 0015459 // potassium

: annotation

uitin binding // inferred from electronic annotation /// 0043130 // ubiquitin binding // inferred from sequence [illegible]

protein homodimerization activity // not recorded /// 0042803 // protein homodimerization activity // inferred

[illegible] binding // inferred from electronic annotation

[illegible]inding // inferred from electronic annotation

35256 // metabotropic glutamate receptor binding // inferred from electronic annotation /// 0051920 // peroxi
ctivity // inferred from direct assay /// 0004872 // receptor activity // inferred from electronic annotation /// 00

transcription factor binding // not recorded /// 0017162 // aryl hydrocarbon receptor binding // inferred from e

›inding // inferred from electronic annotation /// 0046872 // metal ion binding // inferred from electronic annot

'/ 0016740 // transferase activity // inferred from electronic annotation /// 0033857 // diphosphoinositol-penta
.5 // protein binding // inferred from electronic annotation /// 0008134 // transcription factor binding // inferre

on /// 0019904 // protein domain specific binding // inferred from electronic annotation /// 0042803 // protein

on /// 0016787 // hydrolase activity // inferred from electronic annotation /// 0016874 // ligase activity // inferr

r binding // inferred from electronic annotation /// 0046966 // thyroid hormone receptor binding // inferred fro

ferred from electronic annotation /// 0005515 // protein binding // inferred from electronic annotation /// 000

otation /// 0016742 // hydroxymethyl-, formyl- and related transferase activity // inferred from electronic anno

tation /// 0030670 // phagocytic vesicle membrane // inferred from mutant phenotype /// 0031088 // platelet c
tation /// 0030670 // phagocytic vesicle membrane // inferred from mutant phenotype /// 0031088 // platelet c

ase activity // inferred from electronic annotation /// 0019901 // protein kinase binding // not recorded /// 001

vity // inferred from electronic annotation /// 0019904 // protein domain specific binding // not recorded /// 00
g // inferred from electronic annotation
g // inferred from electronic annotation

activity // inferred from electronic annotation /// 0016740 // transferase activity // inferred from electronic anr

/ 0016740 // transferase activity // inferred from electronic annotation /// 0032403 // protein complex binding

c annotation /// 0046872 // metal ion binding // inferred from electronic annotation
c annotation /// 0046872 // metal ion binding // inferred from electronic annotation

ationic amino acid transmembrane transporter activity // inferred from direct assay /// 0015326 // cationic ami

/// 0030165 // PDZ domain binding // inferred from physical interaction
eptor binding // inferred from electronic annotation /// 0005515 // protein binding // inferred from electronic a

nnotation /// 0030145 // manganese ion binding // inferred from sequence or structural similarity /// 0042802 /

sphingolipid binding // inferred from electronic annotation /// 0050253 // retinyl-palmitate esterase activity //
annotation /// 0046872 // metal ion binding // inferred from electronic annotation

y // inferred from electronic annotation /// 0016740 // transferase activity // inferred from electronic annotatio

ATPase activity // inferred from electronic annotation /// 0016887 // ATPase activity // inferred from sequence

physical interaction /// 0005515 // protein binding // inferred from electronic annotation /// 0005524 // ATP b

onic annotation /// 0016772 // transferase activity, transferring phosphorus-containing groups // inferred from

ınosterol O-acyltransferase activity // inferred from electronic annotation
ınosterol O-acyltransferase activity // inferred from electronic annotation

ːd from direct assay /// 0050508 // glucuronosyl-N-acetylglucosaminyl-proteoglycan 4-alpha-N-acetylglucosamiı

/ not recorded /// 0016301 // kinase activity // inferred from electronic annotation /// 0046872 // metal ion biı
ıotation /// 0016491 // oxidoreductase activity // inferred from electronic annotation /// 0018454 // acetoacety

ot recorded /// 0015651 // quaternary ammonium group transmembrane transporter activity // not recorded

entical protein binding // inferred from direct assay /// 0043014 // alpha-tubulin binding // inferred from electr

528 // transcription regulator activity // inferred from electronic annotation
528 // transcription regulator activity // inferred from electronic annotation

nnotation /// 0016740 // transferase activity // inferred from electronic annotation /// 0042277 // peptide bind
n direct assay /// 0042803 // protein homodimerization activity // not recorded /// 0042803 // protein homodin

rotein as one donor, and incorporation of one atom of oxygen // inferred from electronic annotation /// 002003
rotein as one donor, and incorporation of one atom of oxygen // inferred from electronic annotation /// 002003

nding // inferred from electronic annotation /// 0005524 // ATP binding // inferred from sequence or structural

ded

)82 // protein heterodimerization activity // inferred from electronic annotation

protein C-terminus binding // inferred from physical interaction /// 0030898 // actin-dependent ATPase activity ,

ect assay /// 0030528 // transcription regulator activity // inferred from electronic annotation /// 0043565 // se
or activity // inferred from direct assay /// 0016564 // transcription repressor activity // inferred from direct ass

m electronic annotation

0032403 // protein complex binding // inferred from electronic annotation /// 0032403 // protein complex binc
l interaction /// 0005515 // protein binding // inferred from electronic annotation /// 0019838 // growth factor

2 // protein heterodimerization activity // inferred from physical interaction

// lipid-transporting ATPase activity // not recorded /// 0034634 // glutathione transmembrane transporter act

: transporter activity // not recorded

ation /// 0016301 // kinase activity // inferred from electronic annotation /// 0016740 // transferase activity //

2 specific) // inferred from direct assay

vity // inferred from electronic annotation /// 0016740 // transferase activity // inferred from electronic annota

rred from direct assay /// 0070053 // thrombospondin receptor activity // not recorded /// 0070892 // lipoteich

'/ 0016565 // general transcriptional repressor activity // not recorded /// 0016565 // general transcriptional re

e activity // inferred from electronic annotation /// 0016740 // transferase activity // inferred from electronic a

ıotation
ıotation

ːrred from electronic annotation /// 0005524 // ATP binding // inferred from electronic annotation /// 0008201

/ inferred from electronic annotation /// 0016740 // transferase activity // inferred from electronic annotation /

ınnotation /// 0005006 // epidermal growth factor receptor activity // inferred from direct assay /// 0005006 //

ty // traceable author statement /// 0005102 // receptor binding // inferred from physical interaction /// 00055
not recorded /// 0010858 // calcium-dependent protein kinase regulator activity // inferred from direct assay /

electronic annotation /// 0030528 // transcription regulator activity // inferred from electronic annotation /// 0[illegible]

'// 0008502 // melatonin receptor activity // inferred from electronic annotation /// 0043565 // sequence-speci

n /// 0016740 // transferase activity // inferred from electronic annotation /// 0031435 // mitogen-activated pr

ı /// 0016740 // transferase activity // inferred from electronic annotation /// 0031434 // mitogen-activated prc

)016791 // phosphatase activity // inferred from electronic annotation

ed /// 0019904 // protein domain specific binding // inferred from electronic annotation /// 0046872 // metal ic

/// 0046872 // metal ion binding // inferred from electronic annotation /// 0048101 // calcium- and calmodulir

rded /// 0046872 // metal ion binding // inferred from electronic annotation /// 0050681 // androgen receptor

[illegible] // inferred from electronic annotation /// 0019003 // GDP binding // inferred from sequence or structural simil

/ phosphorus-oxygen lyase activity // inferred from electronic annotation /// 0016941 // natriuretic peptide rec

// not recorded /// 0032795 // heterotrimeric G-protein binding // inferred from electronic annotation /// 004[illegible]

erred from genetic interaction /// 0015277 // kainate selective glutamate receptor activity // inferred from muta
vity // inferred from electronic annotation /// 0019901 // protein kinase binding // inferred from electronic ann

tor binding // not recorded /// 0031752 // D5 dopamine receptor binding // inferred from electronic annotatio

ı electronic annotation /// 0042802 // identical protein binding // not recorded /// 0042802 // identical protein

'/ inferred from electronic annotation /// 0016779 // nucleotidyltransferase activity // inferred from electronic a

ıctivity // inferred from electronic annotation /// 0015662 // ATPase activity, coupled to transmembrane moven
binding // inferred from electronic annotation

entical protein binding // inferred from direct assay /// 0043014 // alpha-tubulin binding // inferred from electr

on /// 0047105 // 4-trimethylammoniobutyraldehyde dehydrogenase activity // not recorded /// 0047105 // 4-t

direct assay /// 0043565 // sequence-specific DNA binding // inferred from electronic annotation

cystic fibrosis transmembrane conductance regulator binding // inferred from electronic annotation /// 004298(

n /// 0016740 // transferase activity // inferred from electronic annotation /// 0046872 // metal ion binding // i

ne deacetylase binding // inferred from electronic annotation /// 0046982 // protein heterodimerization activit

ps // inferred from electronic annotation

/// 0030165 // PDZ domain binding // inferred from physical interaction

' inferred from direct assay /// 0030246 // carbohydrate binding // inferred from electronic annotation /// 0043

al interaction /// 0046872 // metal ion binding // inferred from electronic annotation /// 0046982 // protein he

; // inferred from physical interaction /// 0042974 // retinoic acid receptor binding // inferred from physical inte

' inferred from electronic annotation /// 0050998 // nitric-oxide synthase binding // not recorded /// 0050998 /,

:ellular-glutamate-gated ion channel activity // inferred from electronic annotation /// 0005261 // cation chann[illegible]

[illegible]rotein C-terminus binding // inferred from physical interaction /// 0030898 // actin-dependent ATPase activity [illegible]

c annotation

protein kinase binding // not recorded /// 0046872 // metal ion binding // inferred from electronic annotation

binding // inferred from direct assay /// 0042169 // SH2 domain binding // non-traceable author statement /// [illegible]

interaction /// 0005515 // protein binding // inferred from electronic annotation /// 0005518 // collagen bindin
ation /// 0016301 // kinase activity // inferred from electronic annotation /// 0016740 // transferase activity //

from electronic annotation /// 0016740 // transferase activity // inferred from electronic annotation /// 00199C

e binding // not recorded /// 0042277 // peptide binding // inferred from electronic annotation /// 0048029 //
c annotation /// 0050662 // coenzyme binding // inferred from electronic annotation

61 // NADP or NADPH binding // not recorded /// 0070402 // NADPH binding // inferred from electronic annota

0043565 // sequence-specific DNA binding // not recorded /// 0043565 // sequence-specific DNA binding // infe

ed from electronic annotation /// 0016740 // transferase activity // inferred from electronic annotation /// 004[illegible]

binding // inferred from electronic annotation
electronic annotation /// 0016301 // kinase activity // inferred from electronic annotation /// 0016740 // transfe[illegible]

otation /// 0016743 // carboxyl- or carbamoyltransferase activity // inferred from electronic annotation /// 001

ar receptor activity // inferred from electronic annotation /// 0004887 // thyroid hormone receptor activity // n[illegible]
vity // inferred from electronic annotation /// 0004725 // protein tyrosine phosphatase activity // inferred from

nding // inferred from electronic annotation /// 0050681 // androgen receptor binding // inferred from electron

nnotation /// 0005006 // epidermal growth factor receptor activity // inferred from direct assay /// 0005006 //

ded /// 0005102 // receptor binding // inferred from electronic annotation /// 0005216 // ion channel activity /
n electronic annotation /// 0008134 // transcription factor binding // inferred from electronic annotation /// 00

activity // inferred from physical interaction

or structural similarity /// 0046872 // metal ion binding // inferred from electronic annotation /// 0051747 // cy

ınnotation

inding // inferred from electronic annotation
inding // inferred from electronic annotation

ɔr // inferred from electronic annotation /// 0043560 // insulin receptor substrate binding // inferred from physi

ıferred from electronic annotation /// 0016740 // transferase activity // inferred from electronic annotation ///

ıferred from electronic annotation /// 0050661 // NADP or NADPH binding // inferred from electronic annotatio

from sequence or structural similarity /// 0046872 // metal ion binding // inferred from electronic annotation

rotein kinase A regulatory subunit binding // not recorded /// 0034237 // protein kinase A regulatory subunit b

/ transcription activator activity // inferred from sequence or structural similarity /// 0016566 // specific transc

// 0016922 // ligand-dependent nuclear receptor binding // inferred from physical interaction /// 0018024 // his

0165 // PDZ domain binding // not recorded /// 0030374 // ligand-dependent nuclear receptor transcription co

on /// 0043130 // ubiquitin binding // inferred from sequence or structural similarity

d from physical interaction /// 0008270 // zinc ion binding // inferred from electronic annotation /// 0008289 //
528 // transcription regulator activity // inferred from electronic annotation
annotation /// 0005006 // epidermal growth factor receptor activity // inferred from direct assay /// 0005006 //

' inferred from electronic annotation

// DNA bending activity // inferred from sequence or structural similarity /// 0010843 // promoter binding // in

n /// 0042277 // peptide binding // not recorded /// 0042277 // peptide binding // inferred from electronic ann

limerization activity // inferred from physical interaction /// 0046982 // protein heterodimerization activity // n[illegible]

from sequence or structural similarity /// 0046872 // metal ion binding // inferred from electronic annotation

// 0016922 // ligand-dependent nuclear receptor binding // inferred from physical interaction /// 0018024 // his

nferred from electronic annotation

:orded /// 0030165 // PDZ domain binding // traceable author statement

activator activity // inferred from sequence or structural similarity /// 0019899 // enzyme binding // inferred frc

from electronic annotation /// 0005524 // ATP binding // inferred from electronic annotation /// 0005524 // AT

recorded

ɔtation /// 0016740 // transferase activity // inferred from electronic annotation /// 0046872 // metal ion bindir

from electronic annotation /// 0043398 // HLH domain binding // inferred from physical interaction /// 004342!

ectronic annotation /// 0030378 // serine racemase activity // inferred from direct assay /// 0030378 // serine r

d from electronic annotation

ndent nuclear receptor activity // inferred from electronic annotation /// 0004883 // glucocorticoid receptor act

005515 // protein binding // inferred from physical interaction /// 0005515 // protein binding // inferred from e
channel regulator activity // inferred from electronic annotation /// 0016301 // kinase activity // inferred from

or structural similarity /// 0046872 // metal ion binding // inferred from electronic annotation

from electronic annotation /// 0043565 // sequence-specific DNA binding // inferred from direct assay /// 0043

redoxin activity // inferred from electronic annotation
004879 // ligand-dependent nuclear receptor activity // not recorded /// 0004879 // ligand-dependent nuclear r

electronic annotation /// 0030528 // transcription regulator activity // inferred from electronic annotation /// 0

tation /// 0047555 // 3',5'-cyclic-GMP phosphodiesterase activity // inferred from electronic annotation

akisphosphate kinase activity // inferred from electronic annotation /// 0033857 // diphosphoinositol-pentakisp
d from physical interaction /// 0008270 // zinc ion binding // inferred from electronic annotation /// 0008289 /

ı homodimerization activity // not recorded /// 0048037 // cofactor binding // in

red from electronic annotation

om sequence or structural similarity /// 0051425 // PTB domain binding // infer

8270 // zinc ion binding // inferred from electronic annotation /// 0010843 //

tation /// 0016874 // ligase activity // inferred from electronic annotation /// 004687

dense granule membrane // not recorded
dense granule membrane // not recorded

9901 // protein kinase binding // inferred from electronic annotation

019904 // protein domain specific binding // inferred from electronic annotation

notation /// 0017022 // myosin binding // inferred from direct assay /// 004

// not recorded /// 0035173 // histone kinase activity // inferred from electro

no acid transmembrane transporter activity // not recorded /// 0015326 // cationic amino acid tran

annotation /// 0005515 // protein binding // inferred from physical interaction

// identical protein binding // not recorded /// 0046872 // metal ion binding // inferred from ele

not recorded /// 0050253 // retinyl-palmitate esterase activity // inferred f

n /// 0019902 // phosphatase binding // inferred from electronic annotation /// 004

or structural similarity /// 0030898 // actin-dependent ATPase activity // not recorded

inding // inferred from electronic annotation /// 0015631 // tubulin binding // i

electronic annotation /// 0016773 // phosphotransferase activity, alcohol group

ıyltransferase activity // inferred from electronic annotation /// 0050509 // N-acetylgl

ıding // inferred from electronic annotation /// 0047485 // protein N-terminus bind
ʼl-CoA reductase activity // inferred from electronic annotation /// 0042802 // identical p

onic annotation /// 0043027 // caspase inhibitor activity // inferred from electronic an

ing // not recorded /// 0051219 // phosphoprotein binding // not recorded
nerization activity // inferred from electronic annotation /// 0043565 // sequence-spec

7 // heme binding // inferred from electronic annotation /// 0046872 // metal ion binding // infer
7 // heme binding // inferred from electronic annotation /// 0046872 // metal ion binding // infer

l similarity /// 0005536 // glucose binding // not recorded /// 0005536 // glucose bindin

// not recorded

ːquence-specific DNA binding // inferred from electronic annotation
ːay

ding // inferred from sequence or structural similarity /// 0033613 // transcription
binding // inferred from electronic annotation /// 0019970 // interleukin-1

tivity // not recorded /// 0042626 // ATPase activity, coupled to transmembrane movement of

inferred from electronic annotation /// 0043021 // ribonucleoprotein binding //

tion /// 0032403 // protein complex binding // not recorded /// 0042169 // SH2 do

ıoic acid receptor activity // inferred from mutant phenotype

pressor activity // inferred from electronic annotation /// 0030528 // transcr

nnotation /// 0019901 // protein kinase binding // inferred from sequence or stru

// heparin binding // inferred from electronic annotation /// 0016301 // kinase

/// 0046872 // metal ion binding // inferred from electronic annotation

' epidermal growth factor receptor activity // not recorded /// 0005006 // epiderm

515 // protein binding // inferred from physical interaction /// 0005515 // protei
// 0030295 // protein kinase activator activity // inferred from direct assay /// 0046983 // protein d

051879 // Hsp90 protein binding // inferred from electronic annotation

ific DNA binding // inferred from direct assay /// 0043565 // sequence-specific DNA bi

rotein kinase kinase kinase binding // not recorded /// 0046332 // SMAD binding

otein kinase kinase binding // not recorded /// 0031434 // mitogen-activated prote

on binding // inferred from electronic annotation

n-regulated 3',5'-cyclic-GMP phosphodiesterase activity // not recorded /// 0048101 // calcium

binding // not recorded

larity /// 0046872 // metal ion binding // inferred from electronic annotation

eptor activity // inferred from direct assay /// 0016941 // natriuretic peptide rec

;982 // protein heterodimerization activity // not recorded /// 0046982 // protein heterodimeriz

ant phenotype /// 0015277 // kainate selective glutamate receptor activity // not
otation /// 0046872 // metal ion binding // inferred from electronic annotation

n

binding // inferred from electronic annotation /// 0046899 // nucleoside triphospha

annotation /// 0016829 // lyase activity // inferred from electronic annotation /// 0016829 // lyase

nent of ions, phosphorylative mechanism // inferred from electronic annotation /// 0016787 // hydrolase act

onic annotation /// 0043027 // caspase inhibitor activity // inferred from electronic an

trimethylammoniobutyraldehyde dehydrogenase activity // inferred from electronic annotation /// 0051287 //

) // cystic fibrosis transmembrane conductance regulator binding // inferred

inferred from electronic annotation

y // inferred from electronic annotation

169 // cation binding // inferred from electronic annotation /// 0046872 // metal ion bi

terodimerization activity // inferred from electronic annotation /// 0050897 //

eraction /// 0046965 // retinoid X receptor binding // inferred from physical in

/ nitric-oxide synthase binding // inferred from electronic annotation

el activity // inferred from mutant phenotype /// 0005261 // cation channel activity // n

// not recorded

0042803 // protein homodimerization activity // inferred from physical interaction /// 0046

ıg // inferred from physical interaction /// 0008134 // transcription factor binding /
inferred from electronic annotation /// 0043021 // ribonucleoprotein binding //

)1 // protein kinase binding // not recorded /// 0030165 // PDZ domain binding // in

monosaccharide binding // not recorded /// 0048029 // monosaccharide binding //

ation /// 0070402 // NADPH binding // inferred from sequence or structural similarity /// 0070404 // N

erred from electronic annotation

5499 // chemorepellent activity // inferred from direct assay /// 0046875 // e

erase activity // inferred from electronic annotation

6787 // hydrolase activity // inferred from electronic annotation /// 0016810 // hydrolase

ot recorded /// 0004887 // thyroid hormone receptor activity // traceable author statement
ı sequence or structural similarity /// 0005161 // platelet-derived growth factor recepto

nic annotation /// 0051082 // unfolded protein binding // inferred from electronic annotation

' epidermal growth factor receptor activity // not recorded /// 0005006 // epiderm

/ inferred from electronic annotation /// 0005507 // copper ion binding // not recorded /
16563 // transcription activator activity // not recorded /// 0017162 // aryl hyd

ytosine C-5 DNA demethylase activity // inferred from direct assay

ical interaction /// 0046934 // phosphatidylinositol-4,5-bisphosphate 3-kinase

0030674 // protein binding, bridging // not recorded /// 0030674 // protein binding

on

inding // inferred from electronic annotation /// 0051018 // protein kinase A binding //

riptional repressor activity // inferred from sequence or structural similar

stone-lysine N-methyltransferase activity // inferred from electronic anno

activator activity // inferred from direct assay /// 0030375 // thyroid hormo

/ lipid binding // inferred from electronic annotation /// 0030284 // estrogen recept

' epidermal growth factor receptor activity // not recorded /// 0005006 // epiderm

ıferred from sequence or structural similarity /// 0016563 // transcription activ

otation /// 0046872 // metal ion binding // inferred from electronic annotation

ot recorded /// 0046982 // protein heterodimerization activity // inferred f

stone-lysine N-methyltransferase activity // inferred from electronic anno

›m electronic annotation /// 0019900 // kinase binding // inferred from e

ΓP binding // inferred from sequence or structural similarity /// 0008013 // beta-cat

ıg // inferred from electronic annotation

5 // bHLH transcription factor binding // not recorded /// 0043425 // bHLH transcri

acemase activity // not recorded /// 0030378 // serine racemase activity // inferred fr

tivity // inferred from direct assay /// 0004883 // glucocorticoid receptor activity // not recor

electronic annotation /// 0008022 // protein C-terminus binding // inferred from electronic a
electronic annotation /// 0016740 // transferase activity // inferred from ele

565 // sequence-specific DNA binding // inferred from electronic annotation /// 0046982 /

receptor activity // inferred from electronic annotation /// 0005515 // protein binding

051879 // Hsp90 protein binding // inferred from electronic annotation

hosphate kinase activity // inferred from sequence or structural similarity
/ lipid binding // inferred from electronic annotation /// 0030284 // estrogen recept

NAD

NAD
